# The Marine Cembranoid Sarcophine Suppressed the Progression and Recurrence of the Metastatic Castration-Resistant Prostate Cancer via Downregulating EZH2-β-Catenin-Centered Oncogenic Network

**DOI:** 10.3390/md24070223

**Published:** 2026-06-23

**Authors:** Abdullah T. Alhowiriny, Hassan Y. Ebrahim, Ethar A. Mudhish, Dalal Dawud, Khalid A. El Sayed

**Affiliations:** 1 School of Basic Pharmaceutical and Toxicological Sciences, College of Pharmacy, University of Louisiana at Monroe, 1800 Bienville Drive, Monroe, LA 71201, USA; alhowirinya@warhawks.ulm.edu (A.T.A.); hebrahim@vcom.edu (H.Y.E.); etharmudhish@gmail.com (E.A.M.); dawuddr@warhawks.ulm.edu (D.D.); 2 Department of Biomedical Sciences, Discipline of Pharmacology, Edward Via College of Osteopathic Medicine, Monroe, LA 71201, USA

**Keywords:** AKT, ASCL1, β-catenin, BRN2, EZH2, marine natural products, metastatic castration-resistant prostate cancer progression, recurrence, sarcophine

## Abstract

Prostate cancer (PCa) is among the highest incidence malignancies in men, with high rates of inevitable resistance development, relapse, and mortality. Castration-resistant prostate cancer (CRPC) continued to pose substantial therapeutic challenges, highlighting the urgent need for effective treatment options. This study assessed the marine cembranoid sarcophine activity against the progression and recurrence of the metastatic CRPC (mCRPC) in mouse xenograft models. Protein and phosphorylation levels were assessed by immunoblotting and mRNA expression by qPCR and RNA sequencing. The in vivo efficacy was evaluated through tumor progression over 3 weeks followed by primary tumor excision and recurrence monitoring over an 8-week course. Sarcophine significantly reduced the mCRPC CWR-R1ca tumor volume by 74.1% and suppressed the epigenetic regulators EZH2 and SMYD2; lineage plasticity factors ASCL1 and BRN2; Wnt/stemness signaling markers β-catenin and LGR6; AKT total expression and activation; and invasion-associated proteins TRPC4 and MMP2 in primary tumors. Sarcophine effectively prevented the mCRPC locoregional recurrence, as well as lung and spleen distant recurrences, and effectively reduced recurrence in other organs. Transcriptomics-RNA-Seq analysis of primary tumors identified 2697 downregulated and 3534 upregulated genes, indicating broad transcriptional reprogramming following sarcophine treatments. These findings demonstrate coordinated suppression of multi-oncogenic pathways and validate the therapeutic potential of sarcophine to control mCRPC.

## 1. Introduction

Prostate cancer (PCa) remains a significant worldwide health concern, ranking as the second most diagnosed cancer and the fifth leading cause of cancer-related death in men [1]. Recent surveillance data from the United States confirms that after years of decline, PCa incidence is escalating again with an annual growth rate of 2.9% from 2014 through 2022 [2]. The incidence rates of distant-stage disease (metastatic) have been rising, and diagnoses of regional-stage disease have been rising by 4.6% per year since 2013 [2]. While localized PCa has a 5-year survival rate of nearly 100% and is considered curable, metastatic PCa necessitates the use of systemic therapies, which remain largely ineffective [2]. Therefore, there is a critical need for the discovery of novel therapeutics to combat this growing public health crisis.

The current treatment options for metastatic disease are limited to androgen deprivation therapy (ADT); however, tumors invariably progress to metastatic castration-resistant prostate cancer (mCRPC), an aggressive and lethal form of PCa [3,4]. While mCRPC remains dependent on androgen receptor (AR) signaling, patients will ultimately become resistant to second-generation androgen pathway inhibitors (APIs) such as abiraterone and enzalutamide [2,4]. Moreover, an estimated 20–40% of patients are primary de novo therapy resistant, and nearly all responding patients eventually develop secondary (acquired) resistance [4,5]. Resistance to therapy is mediated by a complex heterogeneous molecular mechanism, including AR gene amplification, expression of constitutively active AR splice variants such as AR-V7, and activation of alternative oncogenic signaling pathways [4,5,6]. One of the most aggressive mechanisms of resistance is the lineage plasticity-driven transdifferentiation of primary adenocarcinoma into neuroendocrine prostate cancer (NEPC), an AR-independent tumor subtype with extremely poor prognosis [7]. As such, there remains an urgent need to identify novel therapeutic agents to overcome various mechanisms underlying therapeutic resistance.

Marine organisms have unique ecological environments that force them to biosynthesize powerful bioactive secondary metabolites. Marine invertebrates and microbes exclusively produce unique structurally diverse metabolites with potent biological activities. Numerous new bioactive marine natural products (MNPs) have already been reported [8,9,10]. Natural products represent the backbone of oncologic therapeutics, with roughly 80% of the FDA-approved chemotherapeutic drugs deriving from natural products or their synthetic analogues [8]. Several MNPs are in clinical use, including Cytarabine (Ara-C) for leukemia and non-Hodgkin’s lymphoma; Trabectedin (Yondelis) for soft tissue sarcoma; and Eribulin mesylate (Halaven) for metastatic breast cancer, highlighting the therapeutic value of marine biodiscovery [8,9]. In PCa particularly, several MNPs are under active examination and have shown promising activity in preclinical studies [9].

The development of drug-resistant and metastatic forms of PCa involves the activation of key oncogenic signaling pathways and transcriptional regulators, which represent ideal molecular therapeutic targets. Among these, one of the most consistently dysregulated pro-survival pathways in advanced PCa is AKT signaling, which is overactivated due to loss-of-function mutations in the tumor suppressor PTEN in up to 100% of mCRPC cases [11,12]. Activation of this pathway promotes cell growth, proliferation, and survival and has been linked to therapeutic resistance [11,12,13]. Hence, therapeutic inhibitors of AKT represent promising agents for improving patient outcomes.

The Wnt/β-catenin signaling pathway also plays an essential role in PCa tumorigenesis, proliferation, and development of drug resistance [14]. Loss of Wnt/β-catenin regulation led to nuclear β-catenin accumulation, acting as a transcriptional co-activator of genes involved in cellular proliferation and invasion [15]. Activation of Wnt/β-catenin is prevalent in drug-resistant PCa. Dysregulated Wnt/β-catenin crosstalk with the AR signaling axis, where β-catenin can directly bind and co-activate AR, promoting tumor growth even in the absence of androgens [14,16]. Furthermore, amplification of the gene encoding for leucine-rich repeat-containing G-protein-coupled receptor 6 (LGR6), a Wnt co-receptor, has also been associated with metastatic PCa, which activates Wnt signaling, promoting tumor progression [17].

The transdifferentiation to the aggressive NEPC phenotype is controlled by the lineage plasticity process, which is driven by master transcriptional regulators. Enhancer of Zeste Homolog 2 (EZH2) is the catalytic subunit of the Polycomb Repressive Complex 2 (PRC2), which catalyzes the methylation of histone H3 on lysine 27 (H3K27me3) to silence gene expression. Moreover, EZH2 is recognized as a key epigenetic modulator that is commonly overexpressed in solid tumors and NEPC [18,19]. EZH2 exerts its tumor-promoting functions primarily through its canonical role as a histone lysine methyltransferase, epigenetically silencing tumor suppressor genes. However, EZH2 has also non-canonical roles, including the lysine methylation of non-histone proteins as well as transcriptional co-factor [18,20]. The enzymatic inhibitors of EZH2 have had limited clinical success in controlling PCa, suggesting that non-canonical EZH2 functions may emerge as a viable PCa therapeutic target [18]. Moreover, EZH2 activity is regulated by other enzymes, such as SET and MYND Domain Containing 2 (SMYD2), an upstream lysine methyltransferase, which methylates and activates EZH2 to promote tumorigenesis [21,22]. There are also several well-established transcription factors that regulate neuroendocrine differentiation (NED), namely the core set of transcription factors Achaete-Scute Family BHLH Transcription Factor 1 (ASCL1) and POU Class 3 Homeobox 2 (POU3F2, BRN2) among others, such as ONECUT2 and FOXA2 [7,23,24,25,26]. BRN2 is repressed by AR and becomes upregulated following AR inhibition, which drives NEPC development and aggressive tumor growth [7]. Likewise, ASCL1 is another driver for lineage plasticity and essential for NED and sustained growth of NEPC tumors [23,24]. Aberrant expression of these transcription factors upon long-term use of APIs validates the need for novel interventions that can simultaneously co-target multiple resistance factors.

Ion channels and intracellular signaling are another important class of proteins dysregulated in PCa. Calcium (Ca^2+^) signaling contributes to multiple hallmarks of cancer, including cell proliferation, apoptosis, and migration [27]. The Transient Receptor Potential (TRP) family of ion channel are key regulators of intracellular Ca^2+^ homeostasis. Notably, TRP channels expression has been associated with PCa prognosis, with reports indicating opposing outcomes. Transient Receptor Potential Cation Channel Subfamily C member 4 (TRPC4) proved overexpressed in patients who had a lower risk of systemic recurrence following radical prostatectomy, regardless of Gleason score or tumor stage [28]. Thus, Ca^2+^ signaling has broad regulatory functions in PCa progression. The TRP ion channels represent potential PCa therapeutic targets and prognostic biomarkers as important regulators of Ca^2+^ signaling.

Another critical component of diving tumor progression is the breakdown of the extracellular matrix (ECM), a process largely mediated by enzymes such as Matrix Metalloproteinase-2 (MMP-2). MMP-2, a zinc-dependent endopeptidase, is involved in tissue remodeling, tumor invasion, and metastasis [29]. Elevated MMP-2 expression has been widely reported in PCa and is strongly correlated with larger tumor size, higher Gleason score, and more advanced pathological stages [29]. Importantly, MMP-2 overexpression is an independent predictor of decreased disease-free survival and associated with metastasis, making it a crucial therapeutic target for limiting metastasis [29].

The marine cembranoid diterpenes emerged as a promising MNPs bioactive secondary metabolite class with distinct pharmacological activities. Sarcophine, a furanocembranoid diterpene first identified in 1974, from the Red Sea soft coral *Sarcophyton glaucum* [30]. Structurally, it is characterized by a 14-membered carbocyclic ring containing α,β-unsaturated γ-lactone and epoxide functionality. Sarcophine and its derivatives have been studied for diverse biological activities such as anti-inflammatory, antimicrobial, and anticancer effects [31,32]. Sarcophine and its semisynthetic derivatives have shown cancer chemopreventive and antitumor activity against different cancer types. Preliminary studies exhibited its ability to inhibit tumorigenesis by blocking TPA-induced cell transformation [33]. Sarcophine-diol, a semisynthetic derivative, inhibited melanoma cell proliferation by promoting cell cycle arrest and inducing apoptosis through both intrinsic and extrinsic pathways [34]. Sarcophine and its carbamate analogues inhibited the migration of the mCRPC PC-3 cells [35].

Considering the unique chemistry and promising biological activities, this study systematically validates sarcophine as a prospective lead for the control of PCa progression and recurrence. Sarcophine’s molecular effects on mCRPC proved to target a critical EZH2-β-catenin-centered oncogenic network, validating its potential as a novel lead for the control of mCRPC progression and recurrence.

## 2. Results

### 2.1. Identification of Sarcophine as a Potential EZH2 Inhibitor

The concept of targeting EZH2 emerged via participation in the Eli Lilly and Company Open Innovation Drug Discovery (OIDD) Program. Interestingly, sarcophine (OIDD ID 107845410) was identified as a promising EZH2 hit inhibitor in the OIDD hEZH2_5-mer scintillation proximity assay developed to measure the inhibition of human EZH2 by testing compounds ability to reduce H3K27 methylation. The “5-mer” indicates the five-amino acid peptide substrate obtained from Histone H3 and used as a target substrate for methylation by the EZH2 enzyme in the presence of radioactive methyl donors. Sarcophine at a single 100 µM concentration inhibited 39.2% of the 5-mer methylation by EZH2 catalytic activity within the human PRC2, the heteropentameric protein complex. This finding motivated further investigation of sarcophine molecular effects in advanced PCa phenotypes, particularly its impact on the EZH2-driven transcriptional and epigenetic network that contributes to PCa pathogenesis and NED.

### 2.2. Comparative Expression Analysis of Targeted Oncogenic Proteins in Diverse Prostate Cancer Cell Lines

**Figure 1:**
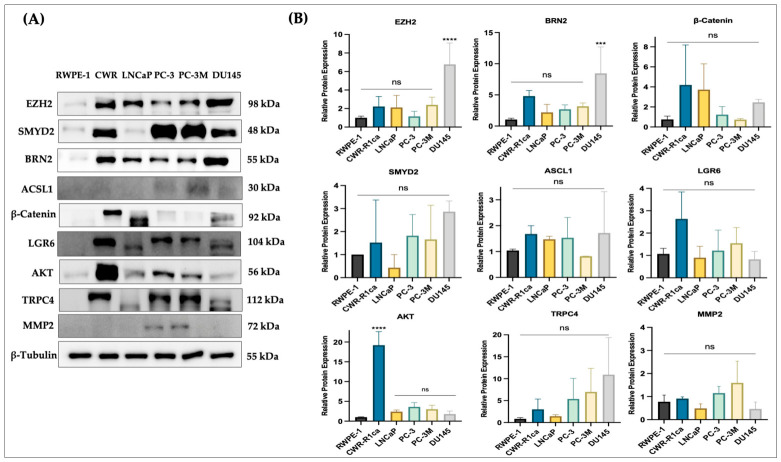
Comparative expression of targeted oncogenic proteins across the PCa and non-tumorigenic prostate epithelial cell lines. (**A**) Immunoblots and (**B**) Densitometric values of targeted oncogenic drivers EZH2, SMYD2, BRN2, ASCL1, β-Catenin, LGR6, AKT, TRPC4, and MMP2 were normalized to β-tubulin and expressed relative to RWPE-1 cells. Data represent mean ± SD of three independent experiments. Statistical analysis was performed using one-way ANOVA **** *p* < 0.0001, *** *p* < 0.001), “ns” indicates statistical non-significance at *p* < 0.05.

To identify the appropriate model for investigating the molecular effects of sarcophine in prostate cancer, an initial screening was conducted across the non-tumorigenic epithelial RWPE-1 cells and a diverse panel of human PCa cell lines, including PC-3, PC-3M, LNCaP, DU145, and CWR-R1ca. Each cell line represents distinct molecular and phenotypic characteristics ranging from androgen receptor (AR)-positive to AR-negative and highly metastatic castration-resistant variants. Among the tested cell line, the mCRPC CWR-R1ca cells demonstrated the most aggressive phenotype and exhibited excessive expression of key oncogenic proteins central to this study, including EZH2, SMYD2, BRN2, ASCL1, β-catenin, LGR6, AKT, TRPC4, and MMP2 (Figure 1). Based on this comprehensive evaluation, CWR-R1ca was selected as the primary model for subsequent mechanistic and functional assays to determine the inhibitory effects of sarcophine on the EZH2-associated signaling network in PCa.

### 2.3. Assessment of In Vitro Sarcophine’s Effects on the Viability of Diverse PCa and Non-Tumorigenic Epithelial Prostate Cell Lines

**Figure 2:**
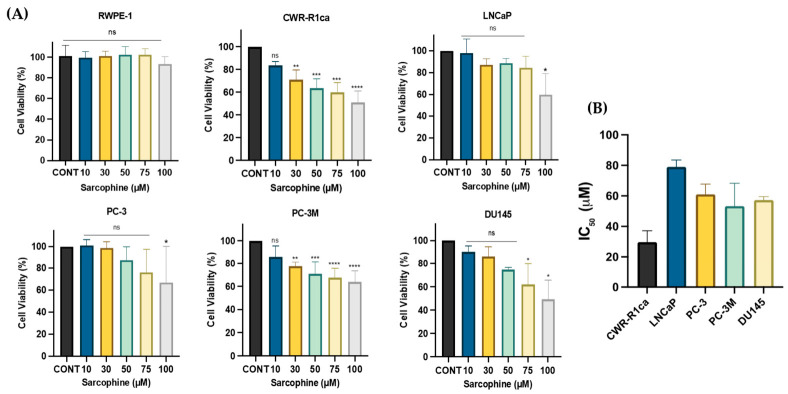
The effects of sarcophine on the viability of the human non-tumorigenic prostate epithelial cells RWPE-1 and human PCa cell lines panel. (**A**) Bar graphs represent the cells viability percentage in vehicle, 10, 30, 50, 75, and 100 µM of sarcophine-treated cell lines. Figures represent treatments to RWPE-1, CWR-R1ca, LNCaP, PC-3, PC-3M, and DU145 cells, respectively. (**B**) IC_50_ Values for sarcophine against all tested PCa cell lines. “ns” indicates statistical non-significance at *p* < 0.05. *, **, ***, **** indicate statistical significance relative to control cells at *p* < 0.05, *p* < 0.01, *p* < 0.001, and *p* < 0.0001, respectively.

The cellular viability effects of sarcophine treatments were assessed using the MTT assay against a panel of PCa cell lines representing distinct disease phenotypes, including the mCRPC CWR-R1ca cells, androgen-dependent LNCaP cells, and androgen-independent PC-3, PC-3M, and DU145 cells, in addition to the immortalized non-tumorigenic RWPE-1 prostate epithelial cells. Cells were treated with a wide range, 10–100 μM, of sarcophine concentration. Sarcophine revealed modest cytotoxic activity across PCa cell lines (Table 1). The mCRPC CWR-R1ca cells showed the highest sensitivity (IC_50_ = 29.7 ± 7.5 μM). Higher IC_50_ values were observed against LNCaP (76.7 ± 2.9 μM), PC-3 (61.1 ± 6.7 μM), PC-3M (53.3 ± 14.9 μM), and DU145 (57.3 ± 2.2 μM). On the other hand, the RWPE-1 cells showed no statistically significant reduction in viability across the same concentration range, indicating potential selective cytotoxicity of sarcophine toward PCa cells (Figure 2). To validate the in vitro biological assays, the sarcophine antiproliferative activity was compared with the standard positive drug control commonly used in clinical practice, docetaxel, a standard chemotherapy, and the second-generation androgen pathway inhibitor enzalutamide, representing the targeted therapies (Table 1).

### 2.4. Sarcophine Effectively Suppressed Clonogenicity of Different PCa Cells

**Figure 3:**
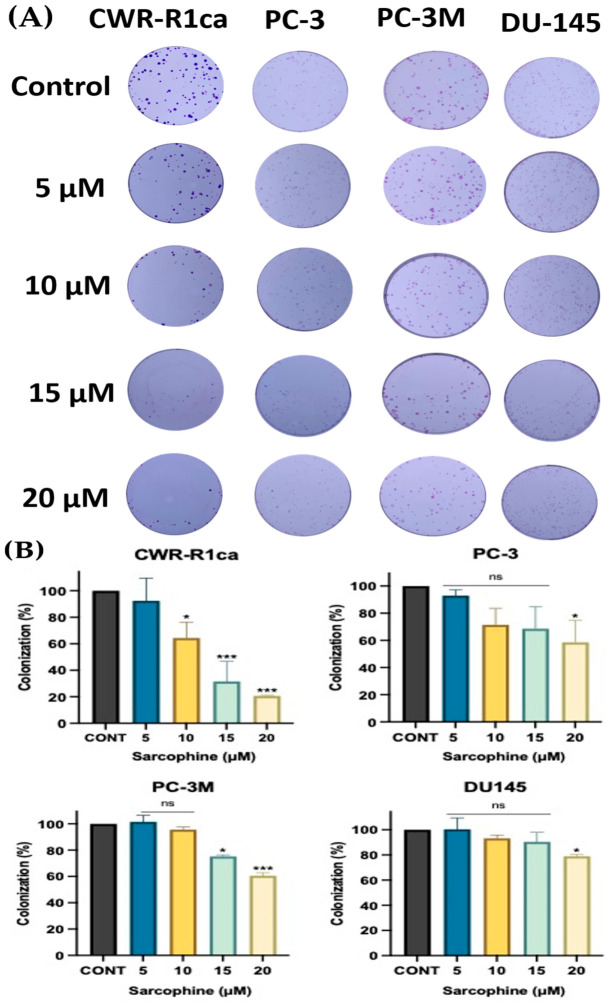
Effects of sarcophine treatments on the clonogenicity of CWR-R1ca, PC-3, PC-3M, and DU145 PCa cells. (**A**) Representative images of CWR-R1ca, PC-3, PC-3M, and DU145 colonies in control cells and cells treated with sarcophine for 14 days and stained with Giemsa at the end of the experiment. (**B**) Quantification analysis of different sarcophine concentrations (5, 10, 15, and 20 μM) and colonization percentage. Bar graphs represent the mean percent colony formation (±SD) at indicated concentrations (µM) relative to vehicle-treated control cells (CONT, 100% clonogenicity). *, *** indicate statistical significance relative to control cells at *p* < 0.05 and *p* < 0.001, respectively. “ns” indicates statistical non-significance at *p* < 0.05.

To evaluate the long-term effects of sarcophine treatments on clonogenicity, colony formation assays were performed against CWR-R1ca, PC-3, PC-3M, and DU145 PCa cell lines following different sarcophine treatments in the range of 5–20 μM. Sarcophine induced a dose-dependent suppression of colony formation in CWR-R1ca cells, with an approximately 36%, 69%, and 80% observed inhibition at 10, 15, and 20 μM, respectively, compared with vehicle-treated controls. In contrast, PC-3 cells exhibited only a modest response, showing a 42% reduction in colony formation at 20 μM, while PC-3M cells displayed moderate sensitivity, with colony numbers reduced by 25% at 15 μM and 40% at 20 μM. However, DU145 cells were the least responsive to sarcophine, demonstrating only a 21% reduction in colony formation at the highest concentration tested. These findings indicate the mCRPC CWR-R1ca cells were the most sensitive to the clonogenicity inhibitory effect of sarcophine treatments (Figure 3).

### 2.5. Molecular Effects of Sarcophine Treatments on Oncogenic Signaling in mCRPC CWR-R1ca Cells

#### 2.5.1. Sarcophine Downregulates the Epigenetic Regulators EZH2 and SMYD2 in CWR-R1ca Cells

**Figure 4:**
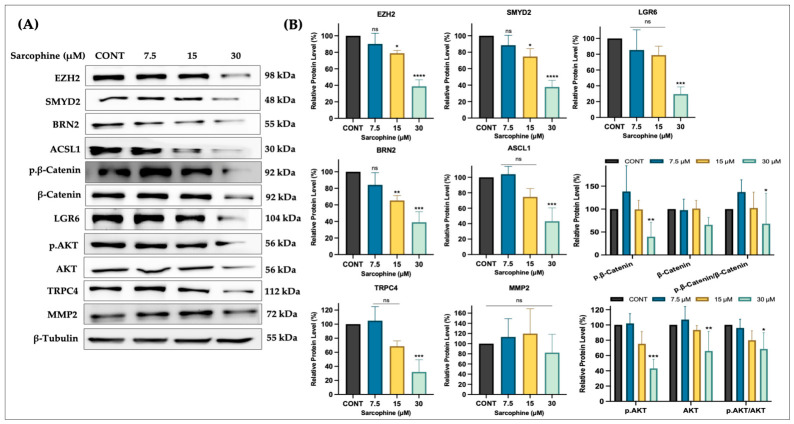
Sarcophine treatment effects on the expression levels of SMYD2, EZH2, BRN2, ASCL1, p-AKT, AKT, p-β-Catenin, β-Catenin, LGR6, TRPC4, and MMP2 in CWR-R1ca mCRPC cell line at the indicated concentrations for 48 h. (**A**) Immunoblots of the targeted oncogenic drivers in cells treated with the vehicle control and 7.5, 15, and 30 μM sarcophine over 48 h. (**B**) Densitometric analysis of the targeted oncogenic drivers’ expression in CWR-R1ca cells. Scanning densitometry was obtained for all blots, carried out in triplicate, and the integrated optical density of each band normalized to β-tubulin. Bar graphs represent mean relative protein expression percent (±SD). “ns” indicates statistical non-significance at *p* < 0.05. *, **, ***, **** indicate statistical significance relative to control cells at *p* < 0.05, *p* < 0.01, *p* < 0.001, and *p* < 0.0001 respectively.

To investigate whether sarcophine modulates epigenetic regulatory mechanisms in CWR-R1ca cells, we first examined the expression of EZH2 and SMYD2 following 7.5, 15, and 30 μM treatments over 48 h. Western blot analysis revealed a significant downregulation of EZH2 and SMYD2 levels compared to vehicle control-treated cells (Figure 4). Densitometric analysis indicated a reduction of EZH2 and SMYD2 expressions by approximately 61% and 63%, respectively, at the highest sarcophine concentration examined.

#### 2.5.2. Suppression of Lineage Plasticity–Associated Transcription Factors ASCL1 and BRN2

Epigenetic reprogramming is closely linked to lineage plasticity, NED, and transcriptional remodeling in advanced PCa phenotypes. The effects of sarcophine on the expression of lineage plasticity-associated transcription factors ASCL1 and BRN2 were assessed. Treatment with sarcophine resulted in a substantial reduction in ASCL1 and BRN2 expression levels by 57% and 61%, respectively (Figure 4). Together, these results indicate that sarcophine downregulates the upstream epigenetic regulators and is accompanied by the suppression of lineage plasticity-associated transcriptional programs in the mCRPC CWR-R1ca cells.

#### 2.5.3. Modulation of Wnt/Stemness Signaling via β-Catenin and LGR6

Activation of Wnt/β-catenin signaling and stemness-related markers has been linked to tumor maintenance and metastatic potential. To determine the effect of sarcophine on this pathway, total β-catenin, phosphorylated β-catenin (p-β-catenin), and LGR6 protein expressions were evaluated in CWR-R1ca cells. Protein analysis demonstrated that the total β-catenin level was not significantly altered following the sarcophine treatment. However, the phosphorylated β-catenin level was drastically reduced in sarcophine-treated cells compared to the vehicle-treated (VC) control (~60%). Densitometric quantification confirmed a statistically significant decrease (~32%) in the p-β-catenin/β-tubulin ratio, indicating suppression of β-catenin activation rather than changes in total protein abundance (Figure 4). In addition, relative LGR6 abundance was notably downregulated (~71%) in treated CWR-R1ca cells relative to VC-treated cells, further validating sarcophine as a Wnt/stemness-associated signaling modulator (Figure 4).

#### 2.5.4. AKT Expression and Activation Reduction

The AKT pathway is a central regulator of survival signaling in prostate cancer. To determine whether sarcophine influences this signaling pathway, total AKT and phosphorylated AKT (p-AKT) expressions were evaluated in CWR-R1ca cells. Immunoblot results showed that sarcophine significantly reduced the total AKT expression level (~34%) compared to VC-treated cells. In parallel, p-AKT expression was markedly decreased (~57%). Densitometric quantification confirmed a statistically significant reduction in both total AKT and p-AKT levels. Furthermore, the p-AKT/AKT ratio was significantly diminished in treated cells (~32%), indicating the attenuation of both total and activated AKT expression (Figure 4) and evidencing the suppression of survival-associated AKT signaling components (Figure 4).

#### 2.5.5. Sarcophine Downregulated the Invasion-Associated Proteins TRPC4 and MMP2 in CWR-R1ca Cells

Ca^++^-dependent signaling and extracellular matrix remodeling contribute to tumor invasion and metastatic progression. To evaluate the sarcophine effects on invasion-associated pathways, TRPC4 and MMP2 protein expressions were examined in CWR-R1ca cells following sarcophine treatments. Immunoblot analysis showed that TRPC4 expression level was significantly reduced in treated cells compared to VC (~68%). In contrast, MMP2 expression exhibited a modest reduction of nearly 18%; however, this decrease did not reach statistical significance. Densitometric quantification confirmed the significant downregulation of TRPC4 level, unlike MMP2 level, which remained statistically comparable in both treatment and VC groups (Figure 4).

### 2.6. In Vivo Effects of Sarcophine on the mCRPC CWR-R1ca-Luc Cells Primary Tumor Progression in a Nude Mouse Xenograft Model

**Figure 5:**
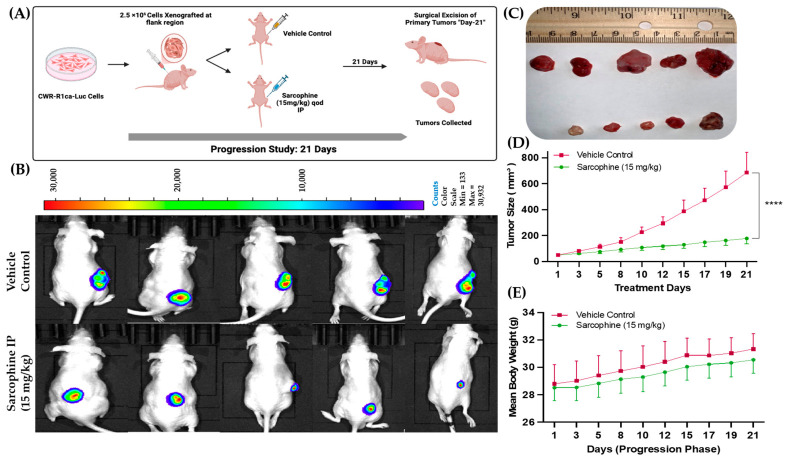
In vivo mCRPC CWR-R1ca-Luc progression-suppressive effects of sarcophine in a nude mouse xenograft model. (**A**) Overview experimental design of the xenograft and PCa progression model. (**B**) Bioluminescence intensity comparison of CWR-R1ca-Luc cell tumors in intact live animals for VC versus sarcophine 15 mg/kg, ip, 3x/week treatment groups at the end of experiment. (**C**) Photographic comparison of the surgically excised primary tumors for VC (top) versus sarcophine-treated (bottom) groups. (**D**) Comparative monitoring of tumor volume in sarcophine versus VC-treated groups over the progression phase experiment. **** indicates statistical significance relative to VC tumors at *p* < 0.0001. (**E**) Comparative monitoring of the effects of sarcophine vs. VC groups on the mean mice total body weight over the progression phase course. Data points represent mean body weight per group in g ± SD. The mean body weights comparison was statistically non-significant, *p* > 0.05.

To evaluate the lead candidacy and therapeutic efficacy of sarcophine under physiologically relevant conditions, sarcophine anti-mCRPC activity was investigated in an in vivo male athymic nude mouse xenograft model that closely reflects tumor growth dynamics and microenvironmental interactions. About 2.5 × 10^6^ CWR-R1ca-luciferase-labeled cells (CWR-R1ca-Luc) were subcutaneously xenografted into the right suprascapular region male athymic nude mice (*n* = 10). Once tumors became palpable, reaching 50 mm^3^, mice were randomized into VC and sarcophine-treated groups, *n* = 5, each. Sarcophine intraperitoneal (ip) maximum tolerated dose (MTD) was determined, using the up-and-down methodology, to be 45 mg/kg in male nude mice [36]. Thus, a 15 mg/kg therapeutic dose was selected for this study, representing 1/3 of its MTD. Sarcophine was administered ip at 15 mg/kg, three times per week for 21 days (Figure 5A). Tumor growth was monitored throughout the study period (Figure 5D). On day-21 of dosing, primary tumors were surgically excised from both VC and sarcophine-treated animal groups. The average tumor volume in the VC-treated group was 686 mm^3^, whereas the sarcophine-treated group exhibited a markedly reduced average tumor volume of 177.5 mm^3^, corresponding to a 74.1% tumor reduction compared to VC (Figure 5C,D). Bioluminescent imaging was performed using the IVIS imaging system prior and after tumor excision surgery to monitor tumor burden in all mice and confirm effective tumor resection, respectively (Figure 5B).

### 2.7. In Vivo Validation of Sarcophine Molecular Targets in Collected Primary CWR-R1ca Tumors

**Figure 6:**
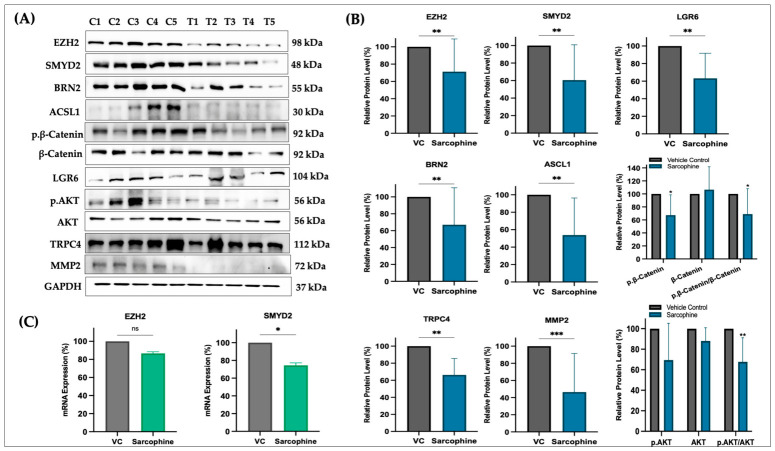
The effects of sarcophine on the targeted oncogenic drivers in CWR-R1ca cell primary tumor tissues. (**A**) Immunoblots and (**B**) Densitometric analysis of the oncogenic targeted proteins expression levels in VC and sarcophine-treated primary tumors. (**C**) Quantitative real-time PCR analysis of EZH2 and SMYD2 mRNA expression in primary tumor tissues from VC and sarcophine-treated groups. Transcript levels were normalized to β-actin and expressed relative to the VC. Data are presented as mean ± SD. “ns” indicates statistical non-significance; * *p* < 0.05, ** *p* < 0.01, and *** *p* < 0.001 indicate statistical significance relative to the vehicle control.

To validate whether the molecular alterations observed and evaluated in vitro earlier in CWR-R1ca cell culture (Figure 4) were recapitulated in vivo, protein expression of the targeted signaling axes was evaluated in collected CWR-R1ca primary tumor lysates derived from VC and sarcophine-treated animal groups. Consistent with the cellular findings, tumors from sarcophine-treated animals exhibited reduced expression of EZH2 (29%), SMYD2 (39.5%), ASCL1 (46%), BRN2 (33%), LGR6 (37%), TRPC4 (34%), total AKT (12%), and p-AKT (32%). A significant decrease in the p-β-catenin/β-catenin (31%) and p-AKT/AKT (32%) ratios also observed, indicating attenuation of these pathways’ activation in vivo (Figure 6A,B). Notably, MMP2 expression was significantly downregulated in tumor lysates (54%), whereas only a modest, non-significant reduction was observed in vitro (Figure 4). Moreover, quantitative real-time PCR (qPCR) analysis was performed to assess SMYD2 and EZH2 mRNA expression in primary tumor tissues. SMYD2 transcript levels were significantly reduced in treated tumors compared to VC, whereas EZH2 mRNA exhibited a reduction that did not reach the statistical significance level (Figure 6C). These findings provide additional validation for the sarcophine’s lead candidacy and potential to suppress mCRPC pathogenesis.

### 2.8. Effects of Sarcophine on the mCRPC CWR-R1ca-Luc Cells Locoregional and Distant Tumor Recurrences in a Nude Mouse Xenograft Model

**Figure 7:**
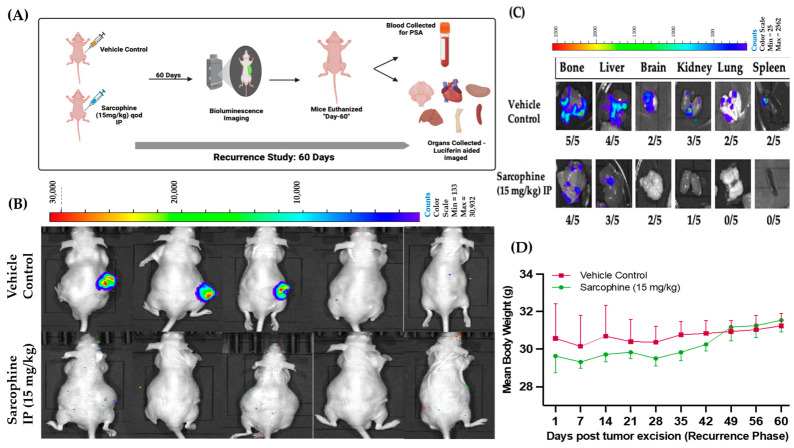
Comparison of the effects of sarcophine on the mCRPC CWR-R1ca cells locoregional and distant recurrences in a male nude mouse xenograft model after surgical excision of primary tumors. (**A**) Overview of the recurrence study experimental design. (**B**) Bioluminescence intensity monitoring of CWR-R1ca-Luc cells in intact animals showing the recurrence-suppressive effects of sarcophine 15 mg/kg, ip, 3x/week versus VC treatment groups at the end of study course (60 days). (**C**) Bioluminescence comparison of the sarcophine versus VC treatments on bone and major animal organs collected at the study end, showing representative organs. (**D**) Comparative monitoring of the effects of sarcophine versus VC treatments on the mean mice total body weight over the recurrence phase study. Data points represent mean body weight per group in g ± SD. The mean body weights comparison was statistically non-significant, *p* > 0.05.

Following the surgical excision of the primary tumors, the same cohort of mice continued on the dosing regimen previously used in the progression experiment (15 mg/kg, ip, 3x per week) to evaluate the sustained effects of sarcophine treatments on the mCRPC locoregional and distant recurrences over the 60-day recurrence study course (Figure 7A). In the VC group, 3 out of 5 mice developed locoregional tumor recurrence. These animals were sacrificed when the tumor volume reached the predefined cutoff endpoint of 1500 mm^3^. One mouse was euthanized during the third week of the recurrence phase, while the remaining two mice were sacrificed during the fourth week. The remaining two VC mice didn’t show locoregional recurrence over the study course. In contrast, none of the sarcophine-treated group mice developed a locoregional tumor recurrence (Figure 7B). To evaluate distant recurrence and dissemination, bone, and major organs, including the liver, brain, kidneys, lungs, and spleen, were collected at the study end after the animals sacrifice and analyzed for bioluminescent tumor colonies using luciferin-aided bioluminescent imaging. These findings indicate an organ-specific effect for sarcophine on distant recurrences, with an absolute prevention of distant recurrence in the lungs and spleens and marked distant recurrence reduction in the kidney, liver, and bone (based on tumor bioluminescence intensity), whereas the incidence of distant recurrence in the brain remained largely comparable with the VC-treated group despite notably reduced tumor foci size in treated animal brains (Figure 7C, Supplementary Figure S1). Interestingly, only a single mouse in the sarcophine-treated group showed neither detectable locoregional nor distant recurrences over the 60-day recurrence study course.

### 2.9. Transcriptomics Profiling Reveals Sarcophine Treatment Modulated Distinct Differentially Expressed Genes in Primary mCRPC Tumors Oncogenic Signaling

**Figure 8:**
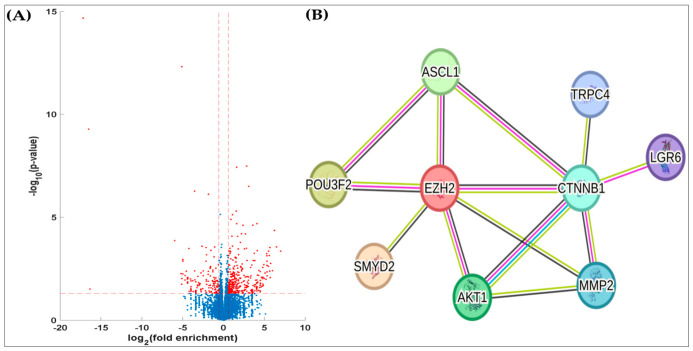
RNA sequencing analysis of differentially expressed genes in primary tumors following sarcophine treatment. (**A**) Volcano plot displaying differentially expressed genes identified by RNA sequencing using a fold-change threshold of ±1.5 with FDR < 0.1 and *p* < 0.05. Red dots represent significantly upregulated gene, blue dots represent significantly downregulated genes. (**B**) Protein–protein interaction (PPI) network generated using the STRING database illustrating the functional connectivity between the targeted proteins evaluated in this study. The network was constructed using an intermediate confidence interaction score threshold (confidence score > 0.4). Nodes represent proteins, and edges represent predicted or experimentally validated interactions.

To complement protein-level analyses, RNA sequencing (RNA-seq) was performed comparing primary tumor tissues to assess global gene expression changes induced by sarcophine treatment versus VC. Differential expression analysis identified a total of 3534 upregulated and 2697 downregulated genes in sarcophine-treated tumors compared to VC, using a fold-change cutoff of ±1.5 (Figure 8A). Among the significantly downregulated transcripts, LGR6 and TRPC4 exhibited marked reductions, consistent with the protein-level suppression observed in both cell cultures and tumor lysates. MMP2 also demonstrated notable downregulation at the transcript level. However, EZH2 showed a modest reduction in mRNA expression, although this change did not reach statistical significance (Table 2). Collectively, these transcriptomics findings indicate broad gene expression reprogramming following the sarcophine treatment and further support modulation of stemness- and invasion-associated oncogenic pathways.

### 2.10. Protein-Protein Interaction Network Analysis Reveals Functional Connectivity Among Targeted Oncogenic Proteins

To explore the functional relationships among the proteins modulated by sarcophine treatment, a protein-protein interaction (PPI) network was constructed using the STRING database (Search Tool for the Retrieval of Interacting Genes/Proteins). Network analysis demonstrated a high degree of connectivity between the oncogenic targeted proteins, including SMYD2, EZH2, BRN2 (POU3F2), ASCL1, β-catenin (CTNNB1), LGR6, TRPC4, AKT, and MMP2. The interaction map revealed clustering of epigenetic regulators, stemness-associated factors, and survival and invasion-related proteins within an interconnected signaling network. Notably, EZH2 and β-catenin were positioned as central nodes within the interaction network, connecting various functional pathways (Figure 8B). These findings illustrate the integrated nature of the mCRPC oncogenic signaling axes modulated by the sarcophine treatment and provide an overview of the unique molecular interactions among the evaluated targets.

### 2.11. Total Serum PSA Levels Reduction Following Sarcophine Treatments

**Figure 9:**
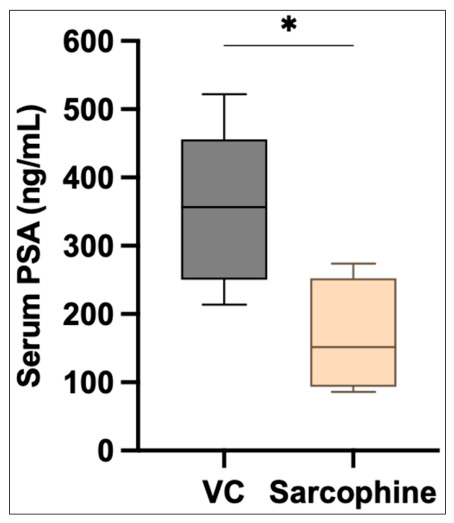
Effect of sarcophine treatments on PSA serum level in male nude mice xenografted with the mCRPC CWR-R1ca-Luc cells at the study end. Data represent median, minimum, and maximum of VC and sarcophine-treated groups. * indicates statistical significance at *p* < 0.05.

Study animals’ prostate-specific antigen (PSA) systemic levels were measured in serum samples collected at the recurrence study end. PSA serum level analysis exhibited a substantial reduction in sarcophine-treated mice compared to VC-treated animals (151.8 ± 64.93 versus 356.4 ± 94.62 ng/mL, Figure 9). These results provide an additional validation for the sarcophine potential to limit mCRPC biochemical recurrence.

## 3. Discussion

To date, there is no curative treatment for advanced PCa phenotypes. Primary hormone-sensitive PCa inevitably progresses to incurable mCRPC, which causes the majority of PCa deaths, accounting for the disease’s increasing mortality rate [2]. Moreover, there are no effective therapies available to prevent or treat biochemical recurrence in PCa patients [3]. Thus, there remains a significant unmet clinical need for the discovery of new PCa therapeutic interventions and approaches that can delay resistance and prolong progression-free survival. This study validates sarcophine as a potential anti-mCRPC lead with significant potency through concurrent downregulation of multiple interconnected EZH2-β-catenin-centered oncogenic pathways required for PCa aggressive pathogenesis.

A central theme emerging from this study’s results is that sarcophine appears to be acting as a powerful anti-mCRPC multi-targeted lead entity. Downregulation of the AKT signaling represents one of our most significant findings. As one of the most important pro-survival pathways in cancer, hyperactivation of PI3K/AKT signaling promotes proliferation and survival in advanced PCa and drives resistance to AR-targeted therapies [11,12]. The failure of many PI3K/AKT-targeted single agents in the clinic can be attributed to robust feedback activation of parallel pathways [37,38]. Sarcophine downregulated the AKT expression, which indicates it suppresses this survival signaling axis in mCRPC.

Concomitant with sarcophine’s ability to downregulate AKT expression, sarcophine also downregulated several other important drivers of mCRPC. The progression of the AR-independence stage is not simply the result of sustained activation of alternative growth pathways but rather a complex process of cellular reprogramming that relies on extensive signaling crosstalk between PI3K/AKT and Wnt/β-catenin as well as epigenetic regulators of lineage plasticity [4,19,37,38,39,40,41,42,43,44,45]. Study results validated the sarcophine’s anti-mCRPC efficacy via inducing overwhelming stress on malignant cells by suppressing the critical survival pathway AKT, deconstructing the central mediator of survival-therapeutic resistance β-catenin, and suppressing master NED regulators BRN2, ASCL1, and EZH2. The ability to target multiple aspects of tumor cell physiology is a highly desirable feature for a lead entity, as it should theoretically present a much greater barrier to cancer cell resistance development compared to individual targeted therapies. Previous studies have shown that sarcophine and its derivatives possess anticancer activity against diverse cancer types, providing proof for its potential as a multifunctional anticancer entity [31,32,33,34,35]. Sarcophine-diol was shown to inhibit proliferation and induce apoptosis in melanoma cells [38]. Sarcophine bioconversion products were found to effectively inhibit skin cancer transformation [39].

The downregulation of β-catenin in mCRPC by sarcophine is also a noteworthy molecular direction because β-catenin is a central mediator of therapeutic resistance in mCRPC [40,41,42,43,44]. In primary hormone-dependent PCa, genomic alterations in Wnt/β-catenin are exceedingly rare; however, they become significantly more prevalent upon development of the metastatic and castration-resistant disease phenotype, where they strongly correlate to AR-targeted therapies resistance [16,40,41,42]. Once activated, β-catenin can directly bind to and co-activate AR, driving tumor growth even with low levels of androgens [16,40]. Moreover, the amplification of the Wnt co-receptor LGR6, which is observed in metastatic disease, further emphasizes the contribution of this pathway to promote tumor progression [17]. Thus, sarcophine suppressing the core driver of AR-targeted therapy resistance mechanism, β-catenin, would hold promise not only as a monotherapy but also in combination with AR-targeted drugs like enzalutamide to prevent or delay the outgrowth of Wnt-driven resistant cells. Combining Wnt inhibitors with APIs is currently in clinical assessment [42,43].

In parallel to sarcophine effects on Wnt signaling, its treatments downregulated the lysine methyltransferase epigenetic regulators EZH2 and SMYD2 as well as the lineage-defining transcription factors BRN2 and ASCL1 that serve as key regulators of NED, which leads the mCRPC differentiation to the deadly NEPC phenotype with extended API therapy [44,45]. EZH2 is one of the most highly upregulated genes in NEPC and functions as a master epigenetic regulator of lineage plasticity that silences tumor suppressor genes and enforces transcriptional programs that promote NED [18,19,20]. Sarcophine downregulated EZH2, as well as its upstream regulator SMYD2 [21,22], providing more comprehensive epigenetic machinery suppression than what can be achieved by enzymatic EZH2 inhibitors alone [18,19,20,21,22,44,45]. Furthermore, by downregulating both BRN2 and ASCL1, the major transcription factors responsible for driving NEPC transcriptional programs [7,23,24,45], sarcophine thereby disrupts the regulatory hierarchy that drives this lethal phenotype. Worth noting, the CWR-R1ca cells represent a highly advanced form of mCRPC that combine the features of primary hormone-sensitive PCa and CRPC markers expression of wild and mutant types of AR, in addition to a full panel of NE markers distinct for the NEPC phenotype [46].

Lastly, TRPC4 and MMP-2 introduce another dimension of sarcophine molecular therapeutic interest. Calcium signaling in cancer has been recognized as highly context-dependent [27]. Thus, this study’s findings revealed that sarcophine downregulated the expression of the calcium channel TRPC4, which is therapeutically important in light of reports linking the overexpressed TRPC4 involvement in tumor angiogenesis and vasculogenesis [28]. While MMP-2 expression downregulation was not evident in the in vitro experiments, potent downregulation was observed in the in vivo collected tumor lysates. The possible explanation for this observation is that sarcophine does not directly regulate MMP-2 expression but rather employs an indirect effect through possible metabolic activation or direct modulation at the tumor microenvironment level, a process that cannot be fully mimicked in a simple in vitro culture system. Results underscore the importance of in vivo models for fully elucidating the mechanisms of novel anticancer agents.

A limitation for this study’s RNA-seq results is the low number of sequenced samples, *n* = 2 per group, which may adversely influence the dispersion estimation and the interpretation of the differentially expressed gene set. The slight rise in the sarcophine-treated tumors RNA-Seq mRNA levels of POU3F2 (+2.11) and ASCL1 (+1.22, Table 2) is discordant with both proteins’ reduced expression level in Western blotting, which showed statistically significant reductions (** *p* < 0.01, Figure 6B). The complex regulatory mechanisms controlling the patterns of protein abundance and post-translational modification in tumors are yet to be fully understood. This discordance between mRNA and transcript protein expression might also be a compensatory mechanism exerted by the tumors in response to sarcophine treatment-induced reduction of POU3F2 and ASCL1 levels. It can also be a plausible resistance development, especially if the mRNA increase was associated with mutation(s). This discordance might warrant future mechanistic studies, which are outside this study scope.

Sarcophine not only showed effectiveness in vivo in mCRPC progression suppression but also absolutely prevented its locoregional recurrence and distant recurrence in the lung and spleen. Sarcophine effectively suppressed distant recurrence in other organs. This result was consistent with the high sarcophine activity in in vitro clonogenicity assays, which mimic the in vivo distant recurrence model since circulating tumor cells will have to form colonies at the distant organs before further progression to metastatic foci. The course of the 60-day recurrence study in the mouse model translates to 23.1 years of human life since each 2.6 days of a mouse life is equivalent to one year in humans [47]. The effective recurrence prevention/suppression of the aggressive mCRPC CWR-R1ca cells was also associated with a significant reduction of the AR-expressing PCa biochemical recurrence marker PSA, validating sarcophine as an effective recurrence suppression lead.

## 4. Materials and Methods

### 4.1. Sarcophine Isolation and Purification

The sarcophine-source soft coral, *Sarcophyton glaucum,* was collected in December 2003 by SCUBA diving at a depth of—5m from the Red Sea near Hurghada, Egypt. A voucher sample (03RS24) was deposited at the School of Basic Pharmaceutical & Toxicological Sciences, College of Pharmacy, University of Louisiana at Monroe, Louisiana. The freeze-dried coral material was exhaustively extracted with methanol (MeOH) at room temperature. The crude methanolic extract was concentrated under reduced pressure and partitioned between hexane and ethyl acetate (EtOAc, 7:3) ratio. The organic phase was evaporated to dryness and subjected to successive chromatographic separations, including silica gel column chromatography, Sephadex LH-20 (Sigma-Aldrich Corporation, St. Louis, MO, USA), and reversed-phase (RP-18) chromatography [39]. Final purification of the sarcophine was achieved by crystallization from ethanol-toluene (9:1), reaching a purity of >99%, based on q^1^H NMR and HPLC analyses. The structure of the purified sarcophine was confirmed by spectroscopic techniques; ^1^H NMR and ^13^C NMR were recorded at 400 and 100 MHz, respectively, in CDCl_3_, using the residual solvent peak at δ_H_ 7.26 and δ_C_ 77.1 as an internal reference, on a JNM-ECZL400S FT-NMR spectrometer (JEOL Inc., Peabody, MA, USA) (Supplementary Figure S2).

### 4.2. Cell Lines and Culture Conditions

Human PCa cell lines, including LNCaP, PC-3, PC-3M, and DU145, were purchased from the American Type Culture Collection (ATCC, Rockville, MD, USA). The CWR-R1ca cell line was purchased from Millipore/Sigma (Burlington, MA, USA). Cells were maintained in RPMI-1640 medium supplemented with 10% fetal bovine serum (FBS) (Avantor^®^, Radnor, PA, USA), amphotericin B (2.5 µg/mL), streptomycin (100 µg/mL), and penicillin G (100 IU/mL) (Quality Biological, Inc., Gaithersburg, MD, USA). Cultures were incubated at 37 °C in a humidified incubator containing 5% CO_2_. For routine passaging, cells at 70–80% confluency were washed with phosphate-buffered saline (PBS) (SeraCare Life Sciences, Milford, MA, USA) free of Ca^2+^ and Mg^2+^, followed by detachment using 0.05% trypsin-EDTA (0.02% EDTA in PBS) for up to 5 min at 37 °C. Trypsinization was neutralized by adding complete growth medium, and cells were collected by centrifugation before being reseeded into fresh culture plates.

### 4.3. Sarcophine Preparation and Stock Solution

Sarcophine was dissolved in dimethyl sulfoxide (DMSO) (VWR International, LLC, Radnor, PA, USA) to prepare a 10 mM stock solution, which was aliquoted and stored at −20 °C until use. For cell treatment, cells were treated with appropriate concentrations and diluted culture medium. The final concentration of DMSO in all treatment and VC groups was adjusted to be equal and preserved at a maximum of 0.5%, which showed no observable cytotoxicity on the tested cells.

### 4.4. Cell Viability Assay

Cells were seeded in 96-well plates at a density of 1 × 10^4^ cells per well (triple replicates per cell line) in RPMI-1640 medium supplemented with 10% fetal bovine serum (FBS) and incubated overnight. The following day, cells were treated with different concentrations of 10 to 100 µM of each of sarcophine and enzalutamide (Ambeed, Buffalo Grove, IL, USA) and 0.5–10 nM docetaxel (TCI America, Portland, OR, USA) for 48 h. Cell viability was then assessed using 3-(4,5-dimethylthiazolyl-2)-2,5-diphenyltetrazolium bromide (MTT) colorimetric assay. MTT solution was added to each well at a final concentration of 1.0 mg/mL and incubated at 37 °C for 4 h. The medium was aspirated, and formazan crystals were solubilized in 100 µL of DMSO. Absorbance measured at 570 nm using a microplate reader (BioTek Instruments, Winooski, VT, USA). IC_50_ values were analyzed using GraphPad Prism software, version 10.6.1 (La Jolla, CA, USA).

### 4.5. Colony Formation Assay

Colony formation assays were carried out using 12-well plates, with 1 × 10^3^ cells seeded per well and cultured for 14 days. Cells were treated with varying concentrations of sarcophine ranging from 1 to 20 μM, with culture medium refreshed every other day during the experiment. On day 14, the medium was aspirated, and the cells were gently washed with cold phosphate-buffered saline (PBS), followed by fixation with cold methanol and staining using Giemsa blue solution (MERCK KGAA, DARMSTADT, GERMANY). Images were captured using a digital camera, and colonies were quantified using ImageJ software (version 1, National Institutes of Health, Bethesda, MD, USA). Images were converted to 8-bit grayscale, thresholded, and analyzed using the “Analyze Particles” function to determine the colonies number and area. All experiments were conducted in triplicate to ensure reproducibility and statistical robustness.

### 4.6. Western Blot Assays

PCa cell lines were seeded at a density of 1 × 10^6^ cells per 100 mm culture dish in RPMI-1640 medium supplemented with 10% fetal bovine serum (FBS) and incubated overnight to allow cell adherence. The next day, cells were washed with phosphate-buffered saline (PBS) and then treated with control or treatment media containing different subtoxic concentrations of sarcophine for 48 h. After treatment, cells were harvested and washed twice with cold PBS and lysed in radioimmunoprecipitation assay (RIPA) buffer (Qiagen Sciences Inc., Valencia, CA, USA) at 4 °C for 30 min. The lysate samples were centrifuged at 16,000× *g* for 10 min, and the supernatants were collected and stored at −80 °C. For protein extraction from animal tumor tissues, Dulbecco’s phosphate-buffered saline (DPBS) was prepared with the addition of 10 μL protease inhibitor (G-Biosciences, Saint Louis, MO, USA) per 1 mL DPBS. Tumor samples were weighed and homogenized in a volume of DPBS corresponding to 1 mg tissue per 5 μL DPBS using an ultrasonic homogenizer (Qsonica Sonicator, Newtown, CT, USA). Tissue homogenates were lysed in RIPA buffer at 4 °C for 30 min, followed by centrifugation at 16,000× *g* for 10 min. The supernatants were collected and stored at −80 °C. Protein concentrations were determined using the Pierce BCA Protein Assay Kit (Bio-Rad, Hercules, CA, USA). For Western blot analysis, 20 μg of cellular lysate and 17 μg of tumor lysate were loaded per lane on 10% SDS-PAGE gels, then transferred on polyvinylidene difluoride (PVDF) membranes. After transfer, membranes were blocked with EveryBlot™ Blocking Buffer (Bio-Rad Laboratories, Hercules, CA, USA) for 5 min at room temperature as per the manufacturer’s instructions and incubated overnight at 4 °C with primary antibodies. After three washes with TBST buffer, membranes were incubated for 1 h at room temperature with species-appropriate horseradish peroxidase (HRP)-conjugated secondary antibodies, followed by additional TBST washes. Protein detection was carried out using the ChemiDoc XRS chemiluminescence imaging system and analyzed with Image Lab software (Bio-Rad, Hercules, CA, USA). The primary antibodies were purchased from Cell Signaling Technology (Danvers, MA, USA) and ProteinTech (Rosemont, IL, USA) and used at a 1:1000 dilution unless otherwise specified (Supplementary Figure S3). MMP2 (Cell Signaling Technology; Cat. No. 87809), phospho-β-catenin (Ser33/37/Thr41) (Cell Signaling Technology; Cat. No. 9561), β-catenin (Cell Signaling Technology; Cat. No. 8480), SMYD2 (Cell Signaling Technology; Cat. No. 9734), EZH2 (Cell Signaling Technology; Cat. No. 5246), ASCL1 (Cell Signaling Technology; Cat. No. 10585), phospho-AKT (Ser473) (Cell Signaling Technology; Cat. No. 4060), AKT (Cell Signaling Technology; Cat. No. 9272), TRPC4 (Proteintech; Cat. No. 85334-2-RR), LGR6 (Proteintech; Cat. No. 17658-1-AP), and BRN2 (Proteintech; Cat. No. 14596-1-AP). Anti-GAPDH mouse monoclonal antibody (Proteintech; Cat. No. 60004-1-Ig) and anti-β-tubulin rabbit monoclonal antibody (Cell Signaling Technology; Cat. No. 2146S) were used as loading controls to ensure equal protein loading in all samples. Horseradish peroxidase (HRP)-conjugated secondary antibodies were obtained from Cell Signaling Technology (Danvers, MA, USA) and used according to the manufacturer’s instructions. All experiments were repeated at least three times, and representative blots are shown in the presented figures. Detailed Research Resource Identifier (RRID) information for antibodies used in the study is included in Supplementary Table S1.

### 4.7. In Vivo Studies

#### 4.7.1. Animal Model and Treatment Mode

CWR-R1ca were bioluminescently tagged and transduced using lentiviral vectors and the firefly luciferase (Luc) reporter following manufacturer protocol (Kerafast, Boston, MA, USA). Cells were selected under antibiotic pressure at a concentration of 0.5 µg/mL puromycin. The pure clones were examined for fluorescence using an IVIS imager, and proven cells were kept frozen in liquid nitrogen until use. Athymic nude mice (Foxn^1nu^/Foxn^1+^, aged 4–5 weeks) were purchased from Envigo (Indianapolis, IN, USA). Upon arrival, animals were acclimated at the University of Louisiana at Monroe (ULM) animal facility and maintained under sterile conditions in individually ventilated cages with Alpha-Dri bedding. Housing conditions were tightly controlled with high-efficiency particulate air (HEPA)-filtered ventilation, a temperature of 25 °C, relative humidity of 55–65%, and a 12-h light/dark cycle. Mice were provided ad libitum access to standard rodent chow (No. 7012, Harlan/Teklad, Madison, WI, USA). All animal procedures were performed according to NIH guidelines and were approved by the ULM Institutional Animal Care and Use Committee (IACUC), protocol #21-DEC-KES-01. Luciferase-labeled CWR-R1ca (CWR-Luc) mCRPC cells were harvested, pelleted, and resuspended in cold Matrigel (Corning Life Sciences, Tewksbury, MA, USA). About 2.5 × 10^6^ cells in 100 μL of Matrigel were subcutaneously inoculated into the right flank of each mouse. Tumor volume at the injection site was monitored over 2–3 weeks. Once the average tumor volume reached approximately 50 mm^3^, around day 21 post-implantation, mice were intraperitoneally injected with (+)-luciferin (150 mg/kg; PerkinElmer, Waltham, MA, USA), anesthetized using isoflurane, USP (Piramal Critical Care, Bethlehem, PA, USA), and imaged on the IVIS Lumina Series III imaging system (PerkinElmer, Waltham, MA, USA) to confirm tumor establishment via bioluminescence. Following imaging, animals were randomized into two groups (*n* = 5 per group): vehicle control and sarcophine-treated groups. Treatments were administered intraperitoneally using U-100 insulin syringes with fixed needles. The VC-treated animals received sterile DMSO dissolved in corn oil, and the treatment group received sarcophine at a dosage of 15 mg/kg, 3 times per week for three and eight-and-half consecutive weeks over the tumor progression and recurrence study experiments, respectively.

#### 4.7.2. Progression Study

Once the tumor volume became palpable, reaching an approximate volume of 50 mm^3^, the VC mice received sterile DMSO in corn oil, and the treatment group mice were intraperitoneally injected with sarcophine, 15 mg/kg, 3 times per week, for 3 weeks. Tumor volume (V) in each mouse was calculated using the formula V = (L × W2)/2, where L is the tumor length and W is the width. On dosing day 21, mice were anesthetized by isoflurane, and tumors were surgically resected and stored at −80 ◦C for further Western blot, qPCR, and RNA-sequencing experiments.

#### 4.7.3. Recurrence Study

After primary tumor excision, the mice of both VC and sarcophine-treated groups continued the same dosage regimen for eight-and-half consecutive weeks. At the study’s end, animals were isoflurane-anesthetized and subjected to entire-body bioluminescent imaging to monitor tumor recurrence and metastasis. Following the imaging, mice were euthanized, and bones and major organs, including the brain, heart, lungs, liver, kidneys, and spleen, were collected and exposed to luciferin-aided bioluminescence imaging to test the presence and evaluate the extent of distant recurrences spread. Quantification of luciferase activity, represented as photons emitted per second, was performed using Living Image software (PerkinElmer Waltham, MA, USA). Color-scaled images indicating relative light intensity “ranging from blue for the lowest to red for the highest” were generated.

### 4.8. Assessment of Animals Systemic PSA Level Using a Human PSA-Total ELISA Kit

Fresh blood samples were collected immediately post-mortem, and serum samples were separated and stored at −80 °C. Total plasma prostate-specific antigen (PSA) levels were subsequently measured using the RayBiotech ELISA kit (RayBiotech, Peachtree Corners, GA, USA) according to the manufacturer’s protocol [46].

### 4.9. qPCR

Complementary DNA (cDNA) was generated using the Bio-Rad iScript cDNA synthesis kit (Bio-Rad Laboratories, Hercules, CA, USA) (Cat. No. 1708891) following the manufacturer’s instructions. Quantitative real-time PCR (qPCR) was performed on a Bio-Rad CFX96 system utilizing the iTaq Universal SYBR Green Supermix (Bio-Rad Laboratories, Hercules, CA, USA) (Cat. No. 1725121). Gene-specific primers were designed and synthesized via the PrimeQuest tool (v.15.26.03, Integrated DNA Technologies, Iowa). The primer sequences were as follows: EZH2 forward: 5′-GAC CTC TGT CTT ACT TGT GGA GC-3′, reverse: 5′-CGT CAG ATG GTG CCA GCA ATA G-3′; SMYD2 forward: 5′-AAG GCA GAA GCC ATC CGA GAC A-3′, reverse: 5′-TCA TCT TCT CCT GGC TGA GCT C-3′; β-actin forward: 5′-GCA CCA CAC CTT CTA CAA TGA-3′, reverse: 5′-GTC ATC TTC TCG CGG TTG GC-3′, with β-actin serving as the internal reference gene. Relative gene expression levels were normalized to the untreated control group, and fold changes were calculated using the −ΔΔCT method. All reactions were performed in duplicate and repeated independently three times to ensure reproducibility.

### 4.10. RNA Extraction

Approximately 50 mg of each excised tumor preserved in RNAlater™ solution (Invitrogen, Thermo Fisher Scientific, Waltham, MA, USA) was transferred into RNase/DNase-free Eppendorf tubes containing 1 mL of TRIzol reagent (Invitrogen, Thermo Fisher Scientific, Waltham, MA, USA). Samples were homogenized using a MISONIX sonicator (QSonica LLC, Newtown, CT, USA) and incubated on ice for 4 h. Subsequently, 200 µL of molecular-grade chloroform (CHCl_3_) (Thermo Fisher Scientific, Waltham, MA, USA) was added, followed by a 3-min incubation at room temperature. Samples were vortexed for 30 s and kept on ice for 15 min to allow phase separation. The interphase was discarded, and the aqueous layer was collected and centrifuged at 12,000× *g* for 15 min at 4 °C. The resulting supernatant was transferred to clean microtubes, and 500 µL of molecular-grade isopropanol (Thermo Fisher Scientific, Waltham, MA, USA) was added to precipitate RNA, followed by incubation on ice for 30 min. Samples were again centrifuged at 12,000× *g* for 10 min at 4 °C, and the supernatant was removed. RNA pellets were washed twice with 70% molecular-grade ethanol, centrifuged at 7500× *g* for 5 min at 4 °C, and air-dried for 2–3 min. The dried RNA pellets were resuspended in 30–50 µL of nuclease-free water (VWR International, Radnor, PA, USA). RNA concentration and purity were determined using a NanoDrop One microvolume spectrophotometer (Thermo Fisher Scientific, Waltham, MA, USA). RNA samples are stored at −80 °C for subsequent analyses.

### 4.11. RNA-Seq Data Processing

RNA sequencing was conducted on two control and two treated tumor samples using a strand-specific 100-cycle paired-end approach on an Illumina NovaSeq 6000 platform (Illumina, San Diego, CA, USA). Treated and control tumor samples were multiplexed across two lanes of a flow cell, yielding between 40.04 and 48.71 million reads per sample. Read quality was evaluated using FastQC software [48,49]. All samples exhibited a mean Phred quality score above 30, indicating high sequencing accuracy [50]. As expected for high-quality RNA-seq data, no adapter contamination requiring trimming was detected. Sequencing reads were aligned to a combined human (GRCh38) and mouse (GRCm39) reference genome using the STAR aligner, version 2.7.11b [50,51], achieving an average mapping rate of 91%, corresponding to 36.0–42.9 million mapped reads per sample, with approximately 80.4% uniquely mapped reads. Transcript abundance was quantified using featureCounts [51], and differential expression analysis was performed with DESeq2 [48] to identify significantly altered genes. *p*-values were adjusted for multiple hypothesis testing using the Benjamini–Hochberg correction method [52], and a false discovery rate (FDR) was calculated for each gene to ensure statistical rigor (Supplementary Tables S2 and S3).

### 4.12. Statistical Analysis

GraphPad Prism software version 10.6.1 (Boston, MA, USA) was used to perform all statistical analyses. Data are presented as mean ± standard deviation (SD). Unpaired *t*-test, one-way ordinary ANOVA, and two-way ANOVA were used to determine significance between treatment and control groups. *p*-values indicating statistical significance are as follows: * *p* < 0.05, ** *p* < 0.01, *** *p* < 0.001, and **** *p* < 0.0001. The minimum animal number required for statistical significance of the sarcophine treatment effect on tumor sizes, *n* = 5, was the practical cohort size consistent with field practice for xenograft progression and recurrence studies and within the ARRIVE 2.0 reporting expectations [53,54].

## 5. Conclusions

This study established the marine-derived cembranoid diterpene sarcophine as a preclinical multi-targeted mCRPC progression inhibitory lead that merits further development. Sarcophine showed instrumental mCRPC recurrence suppression, highlighting potential for extending progression-free survival to mCRPC patients and survivors. Sarcophine exerted broad antitumor activity by modulating several unique oncogenic signaling networks that are associated with PCa pathogenesis and aggression. Sarcophine suppressed AR-independent PCa growth-survival signaling by downregulating Wnt/β-catenin signaling and targeted the epigenetic transcriptional machinery that drives NED of primary PCa and CRPC to the lethal NEPC phenotype by downregulating EZH2, SMYD2, BRN2 and ASCL1. Additionally, sarcophine demonstrated key pro-survival AKT pathway signaling and invasion-associated protein inhibition. Many cancer types, including PCa, have robust compensatory feedback loops between Wnt/β-catenin-AKT signaling, which has been a major obstacle in developing entities that exclusively target either pathway. Thus, this study’s results have uncovered sarcophine as a potent marine-derived chemical probe with multi-targeted activity against interconnected oncogenic pathways relevant to PCa pathogenesis that could be used as a scaffold for future drug development efforts to address the urgent clinical need for an effective advanced PCa control.

## Figures and Tables

**Table 1:** Calculated sarcophine IC_50_ values (µM ± SD) versus the standard positive control drugs docetaxel and enzalutamide in MTT assay against diverse human PCa cells.

Cell Line	Sarcophine IC_50_ µM	Docetaxel IC_50_ nM	Enzalutamide IC_50_ µM
CWR-R1ca	29.7 ± 7.5	0.9 ± 1.3	33.4 ± 12.9
LNCaP	76.7 ± 2.9	-	-
PC-3	61.1 ± 6.7	2.3 ± 0.4	20.3 ± 12.4
PC-3M	53.3 ± 14.9	2.1 ± 0.5	36.6 ± 14.5
DU145	57.3 ± 2.2	2.3 ± 1.2	37.4 ± 14.5

**Table 2:** Comparison of the Log^2^ FPKM (Fragments/Kilobase Million) fold-change expression of targeted genes in sarcophine-treated versus VC mCRPC primary tumors.

Gene Symbol	Fold-Change Expression in Primary Tumors
SMYD2	+1.55
EZH2	−1.62
POU3F2 (BRN2)	+2.11
ASCL1	+1.22
AKT	−1.02
β-catenin	−1.09
LGR6	−25.56 *
TRPC4	−27.10 *
MMP2	−4.96 *

* Significantly downregulated.

## Data Availability

All data used to support the findings of this study including RNA-Seq data made available in this publication as figures, tables, or Supplementary Materials.

## Supplementary Materials

The following supporting information can be downloaded at: https://www.mdpi.com/article/10.3390/md24070223/s1, Figure S1: Distant recurrence comparison of vehicle control versus sarcophine-treated collected organs; Figure S2: Proton and carbon NMR spectra of sarcophine used in anti-mCRPC testing; Figure S3: The original Western blot gels for the study targeted oncogenic proteins. Supplementary Table S1: Detailed RRID information for antibodies used in the study. Supplementary Table S2: Differential RNA sequencing expression data. Supplementary Table S3: RNA-Seq Raw counts for the two control and two sarcophine treated tumor samples.
