# Supplementary material for: The Marine Cembranoid Sarcophine Suppressed the Progression and Recurrence of the Metastatic Castration-Resistant Prostate Cancer via Downregulating EZH2-β-Catenin-Centered Oncogenic Network
Source: Mar Drugs. 2026 Jun 23;24(7):223. doi: 10.3390/md24070223 (PMC13412892; doi:10.3390/md24070223)
Supplement: Supplementary file 1 [file marinedrugs-4367293-supplementary.pdf › marinedrugs-4367293-supplementary.pdf]

marine drugs

Article Supplementary Materials

The Marine Cembranoid Sarcophine Suppressed the Progression and Recurrence of the Metastatic Castration-Resistant Prostate Cancer via Downregulating EZH2-β-Catenin-Centered Oncogenic Network

Abdullah T. Alhowiriny, [1] Hassan Y. Ebrahim, [1,2] Ethar A. Mudhish, [1,†] Dalal Dawud, [1,2] and Khalid A. El Sayed [1,*]

[1] School of Basic Pharmaceutical and Toxicological Sciences, College of Pharmacy, University of Louisiana at Monroe, 1800 Bienville Drive, Monroe, LA 71201, USA; alhowirinya@warhawks.ulm.edu (A.T.A.); hebrahim@vcom.edu (H.Y.E.); etharmudhish@gmail.com (E.A.M.); dawuddr@warhawks.ulm.edu (D.D)

[2] Department of Biomedical Sciences, Discipline of Pharmacology, Edward Via College of Osteopathic Medicine, Monroe, LA 71201, USA.

* Correspondence: elsayed@ulm.edu; Tel.: +1-318-342-1725.

† Current Address: Department of Pharmacognosy, College of Pharmacy, King Khalid University, Abha, Al-Faraa, Saudi Arabia.

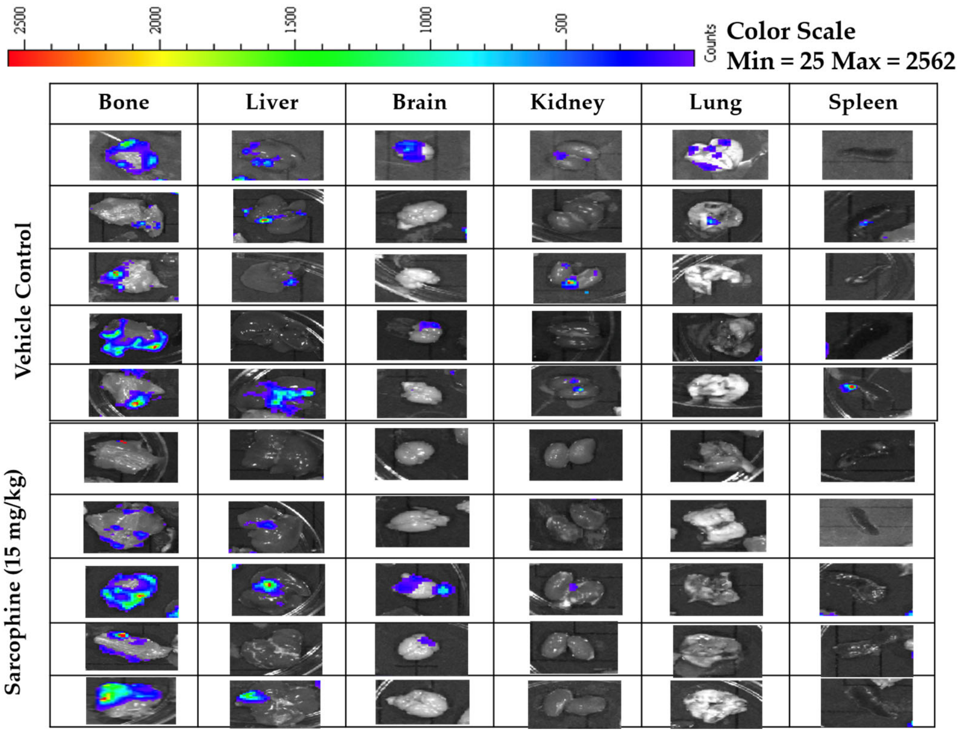

Supplementary Figure S1: Distant recurrence comparison of vehicle control versus sarcophine-treated collected organs. The top five rows show collected organs of VC-treated photographs. The bottom five rows show collected organs of sarcophine-treated photographs.

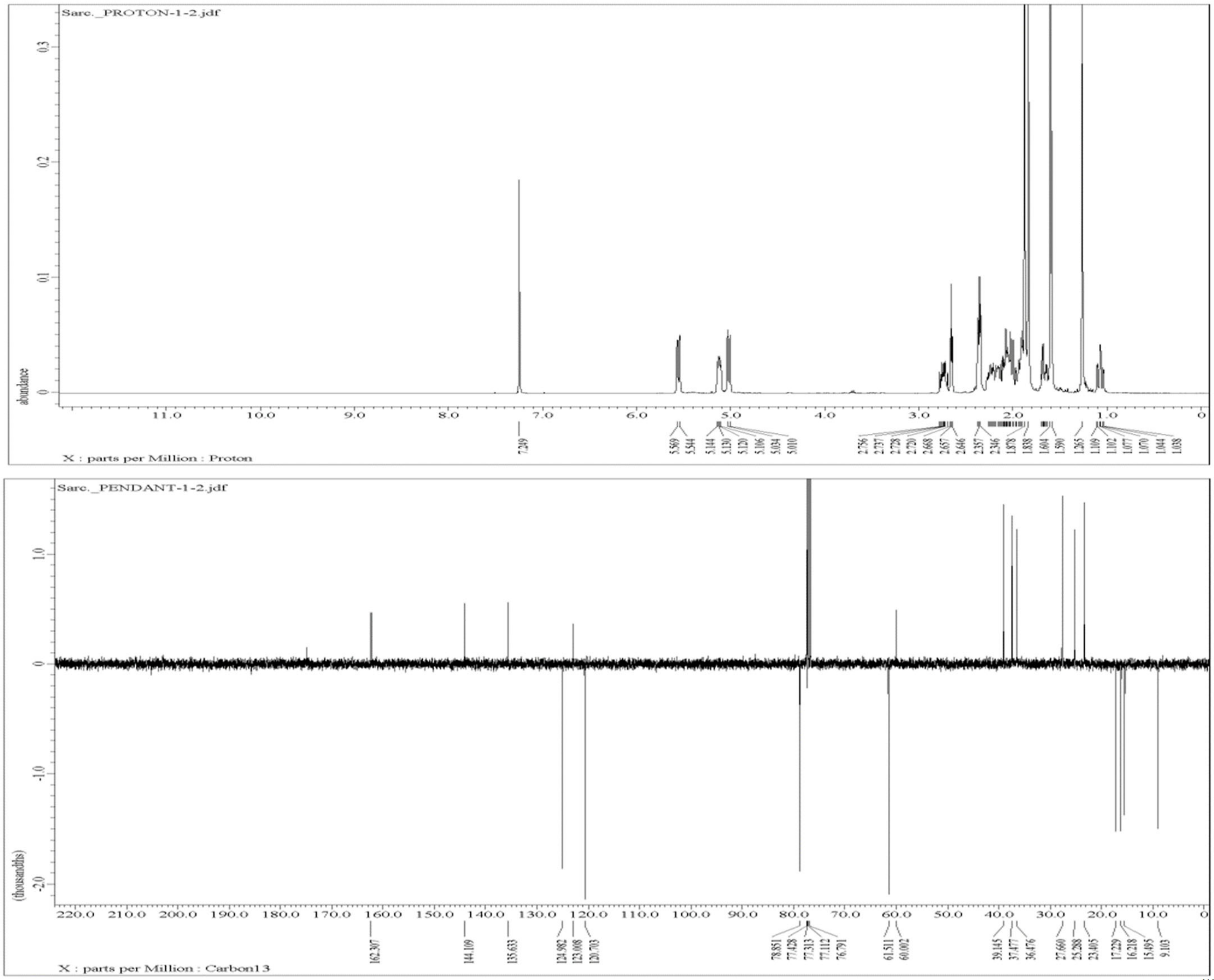

Supplementary Figure S2: Proton and carbon NMR spectra of sarcophine used in anti-mCRPC testing. Top spectrum: ^{1}H NMR spectrum of sarcophine. Bottom spectrum: ^{13}C-PENDANT NMR spectrum of sarcophine. Proton and carbon spectra were recorded at a resonance frequency of 400 and 100 MHz, respectively, in $CDCl_3$, using a JNM-ECZL400S FT-NMR spectrometer.

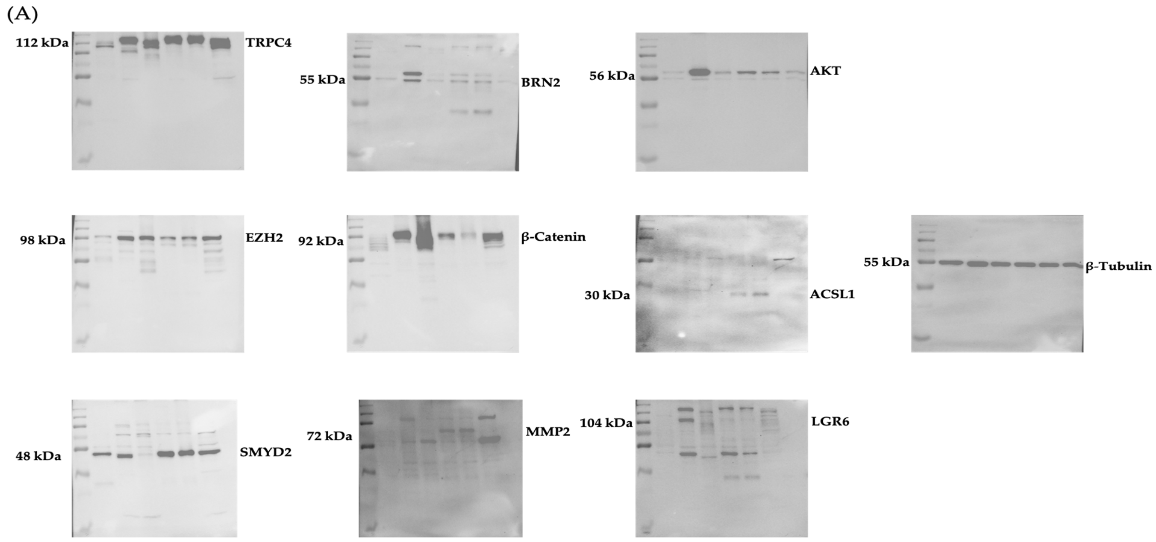

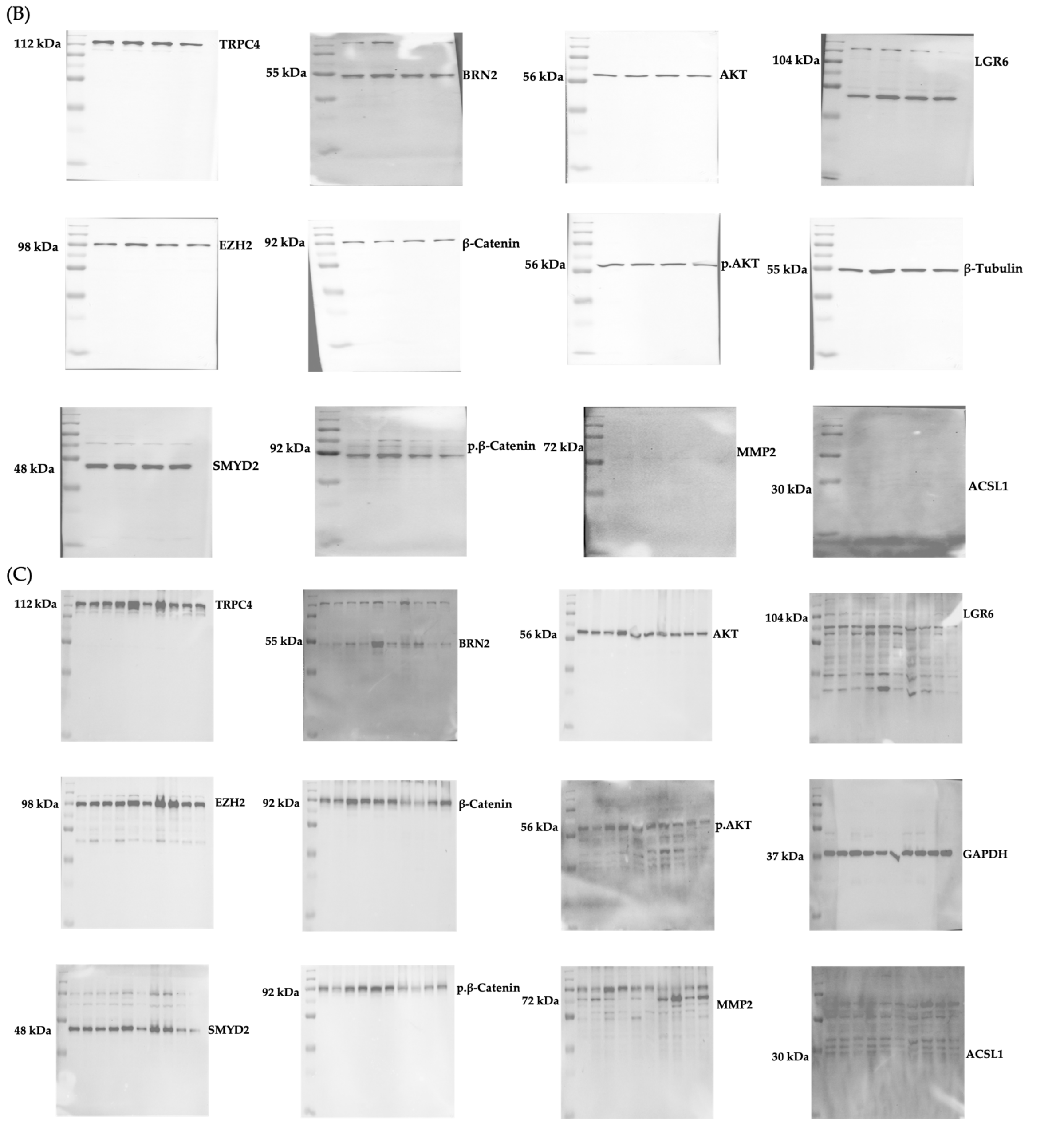

Supplementary Figure S3: The original Western blot gels for study targeted oncogenic proteins: ASCL1, AKT, BRN2, β-catenin, MMP2, LGR6, SMYD2, EZH2, TRPC4. (A) Immunoblots of the *in vitro* expression of targeted oncogenic proteins across the PCa and non-tumorigenic prostate epithelial cell lines. (B) Immunoblots of the targeted oncogenic drivers in CWR-R1ca cells treated with the vehicle control versus 7.5, 15, and 30 μM sarcophine over 48 h. (C) Immunoblots of the *in vivo* oncogenic targeted protein expression levels in VC and sarcophine treated primary tumors.

Supplementary Table S1. Detailed Research Resource Identifier (RRID) information for antibodies used in the study.

Antibodies (Cat. No.)	Source	RRID
MMP-2 (D2O4T) Rabbit mAb (#87809)	Cell Signaling Technology	AB_2800107
ASCL1 Rabbit mAb (#10585)	Cell Signaling Technology	AB_3698070
SMYD2 (D14H7) Rabbit mAb (#9734)	Cell Signaling Technology	AB_10889559
EZH2 (D2C9) Rabbit mAb (#5246)	Cell Signaling Technology	AB_10694683
phospho-AKT (Ser473) (D9E) Rabbit mAb (#4060)	Cell Signaling Technology	AB_2315049
AKT Antibody (#9272)	Cell Signaling Technology	AB_329827
p-β-Catenin (Ser33/37/Thr41) Antibody (#9561)	Cell Signaling Technology	AB_331729
β-Catenin (D10A8) Rabbit mAb (#8480)	Cell Signaling Technology	AB_11127855
TRPC4 Recombinant mAb (#85334-2-RR)	Proteintech	N/A
LGR6 Polyclonal antibody (#14596-1-AP)	Proteintech	AB_2135163
BRN2 Polyclonal antibody (#14596-1-AP)	Proteintech	AB_2167371
GAPDH mouse mAb (Clone. #1E6D9, #60004-1-Ig)	Proteintech	AB_2107436
β-tubulin rabbit mAb (#2146S)	Cell Signaling Technology	N/A

Supplementary Table S2: Differential RNA sequencing expression data.

															8									
															A_Treatment vs. A_Control									
#	gene_id	gene_nam	gene_biotype	seqnar	start_pos	end_pos	stran	genome	havana_gene	human_syml	Entrez Gene Name	Location	Type(s)	Drug(s)	FC	|FC| ≥ 1.5	PValue	Pvalue ≤ 0.05	FDR	FDR ≤ 0.1	Nude_Mouse A_Control Sample_AC1	Nude_Mouse A_Control Sample_AC2	Nude_Mouse A_Treatment Sample_AT1	Nude_Mouse A_Treatment Sample_AT2
1	**ENSG0000000000**	TSPAN6	protein_coding	chrX	100627108	100639991	-	Human GRCh	OTTHUMG000000	TSPAN6	**tetraspanin 6**	Plasma Mem	other		-1.229411495	0	0.45495097	0	1	0	9.60015285	9.95837064	9.77303568	9.30367406
3	**ENSG0000000041**	DPM1	protein_coding	chr20	50934867	50959140	-	Human GRCh	OTTHUMG000000	DPM1	**dolichyl-phosphate mannosyltransferase subunit 1, catalytic**	Cytoplasm	enzyme		1.062079925	0	0.74821653	0	1	0	10.1354966	10.2650462	10.3616286	10.1667977
4	**ENSG0000000045**	SCYL3	protein_coding	chr1	169849631	169894267	-	Human GRCh	OTTHUMG000000	SCYL3	**SCY1 like pseudokinase 3**	Cytoplasm	kinase		-1.215490011	0	0.42612562	0	1	0	8.859812	9.23933939	9.03189993	8.64360811
5	**ENSG0000000046**	C1orf112	protein_coding	chr1	169662007	169854080	+	Human GRCh	OTTHUMG000000	FIRRM	**FIGNL1 interacting regulator of recombination and mitosis**	Nucleus	other		-1.063840066	0	0.78827414	0	1	0	9.9284867	10.1079116	10.1521158	9.71569285
7	**ENSG0000000097**	CFH	protein_coding	chr1	196651754	196752476	+	Human GRCh	OTTHUMG000000	CFH	**complement factor H**	Extracellular	other	GT103	-1.627978146	-1	0.55162562	0	1	0	3.57193651	3.13151215	3.19463932	2.64583903
8	**ENSG0000000103**	FUCA2	protein_coding	chr6	143494812	143511720	-	Human GRCh	OTTHUMG000000	FUCA2	**alpha-L-fucosidase 2**	Extracellular	enzyme		1.226116153	0	0.33833385	0	1	0	9.08078709	9.27338132	9.50437063	9.28213987
9	**ENSG0000000108**	GCLC	protein_coding	chr6	53497341	53616970	-	Human GRCh	OTTHUMG000001	GCLC	**glutamate-cysteine ligase catalytic subunit**	Cytoplasm	enzyme		-1.070846373	0	0.7441244	0	1	0	10.3630302	10.6491255	10.5039363	10.3725772
10	**ENSG0000000116**	NFYA	protein_coding	chr6	41072974	41102403	+	Human GRCh	OTTHUMG000000	NFYA	**nuclear transcription factor Y subunit alpha**	Nucleus	transcription		1.011912988	0	0.95604917	0	1	0	10.7555937	11.0029561	11.0650244	10.7055698
11	**ENSG0000000146**	STPG1	protein_coding	chr1	24356999	24416934	-	Human GRCh	OTTHUMG000000	STPG1	**sperm tail PG-rich repeat containing 1**	Cytoplasm	other		-1.031834531	0	0.89372337	0	1	0	8.45395095	8.78927582	8.6141483	8.58438931
12	**ENSG0000000146**	NIPAL3	protein_coding	chr1	24415802	24472976	+	Human GRCh	OTTHUMG000000	NIPAL3	**NIPA like domain containing 3**	Other	other		-1.124144842	0	0.57058267	0	1	0	10.7605043	11.1633227	10.9103153	10.7930671
13	**ENSG0000000149**	LAS1L	protein_coding	chrX	65438549	65534810	-	Human GRCh	OTTHUMG000000	LAS1L	**LAS1 like ribosome biogenesis factor**	Nucleus	other		-1.01597473	0	0.93949623	0	1	0	10.7806982	10.9468869	10.932811	10.7590691
14	**ENSG0000000156**	ENPP4	protein_coding	chr6	46129989	46146688	+	Human GRCh	OTTHUMG000000	ENPP4	**ectonucleotide pyrophosphatase/phosphodiesterase 4**	Cytoplasm	enzyme		1.131992446	0	0.57285564	0	1	0	8.97857767	9.10719935	9.33249735	9.00044399
15	**ENSG0000000161**	SEMA3F	protein_coding	chr3	50155045	50189075	+	Human GRCh	OTTHUMG000001	SEMA3F	**semaphorin 3F**	Extracellular	other		1.178145545	0	0.53363128	0	1	0	7.02502468	7.03049154	7.10060402	7.27671059
16	**ENSG0000000162**	CFTR	protein_coding	chr7	117287120	117715971	+	Human GRCh	OTTHUMG000000	CFTR	**CF transmembrane conductance regulator**	Plasma Mem	ion channel	ivacaftor/t	5.831768294	1	0.38410531	0	NA	0	0.3678557	0.81779365	1.18576421	1.34456179
17	**ENSG0000000162**	ANKIB1	protein_coding	chr7	92245974	92401383	+	Human GRCh	OTTHUMG000001	ANKIB1	**ankyrin repeat and IBR domain containing 1**	Nucleus	transcription		-1.088295026	0	0.68815709	0	1	0	10.5861571	10.758136	10.7861203	10.3409901
18	**ENSG0000000163**	CYP51A1	protein_coding	chr7	92084987	92134803	-	Human GRCh	OTTHUMG000001	CYP51A1	**cytochrome P450 family 51 subfamily A member 1**	Cytoplasm	enzyme	voriconazc	-1.163316548	0	0.4895281	0	1	0	12.1207879	12.415092	12.2809367	11.8940718
19	**ENSG0000000163**	KRIT1	protein_coding	chr7	92197498	92246166	-	Human GRCh	OTTHUMG000001	KRIT1	**KRIT1 ankyrin repeat containing**	Plasma Mem	other		-1.106465482	0	0.64523353	0	1	0	11.1405395	11.3885768	11.2851713	11.0248281
20	**ENSG0000000201**	RAD52	protein_coding	chr12	911736	990053	-	Human GRCh	OTTHUMG000000	RAD52	**RAD52 homolog, DNA repair protein**	Nucleus	other		-1.040246777	0	0.87948482	0	1	0	8.87833021	9.18159668	9.20223487	8.74834174
21	**ENSG0000000233**	BAD	protein_coding	chr11	64269830	64284704	-	Human GRCh	OTTHUMG000001	BAD	**BCL2 associated agonist of cell death**	Cytoplasm	other		1.082261078	0	0.78102839	0	1	0	9.66810771	10.0133118	10.0049872	9.87536679
22	**ENSG0000000254**	LAP3	protein_coding	chr4	17577198	17607972	+	Human GRCh	OTTHUMG000000	LAP3	**leucine aminopeptidase 3**	Cytoplasm	peptidase		-1.045670698	0	0.83232336	0	1	0	10.1699691	10.5952468	10.4572773	10.2333331
23	**ENSG0000000258**	CD99	protein_coding	chrX	2691187	2741309	+	Human GRCh	OTTHUMG000000	CD99	**CD99 molecule (Xg blood group)**	Plasma Mem	other		-1.108804924	0	0.69771254	0	1	0	9.78885996	9.68127289	9.78544207	9.42776581
24	**ENSG0000000258**	HS3ST1	protein_coding	chr4	11393150	11429564	-	Human GRCh	OTTHUMG000000	HS3ST1	**heparan sulfate-glucosamine 3-sulfotransferase 1**	Cytoplasm	enzyme		1.037928369	0	0.95789843	0	1	0	7.89411121	8.33141482	8.67714803	7.2722459
25	**ENSG0000000272**	AOC1	protein_coding	chr7	150824627	150861504	+	Human GRCh	OTTHUMG000001	AOC1	**amine oxidase copper containing 1**	Extracellular	enzyme		-5.434101933	-1	0.17520264	0	1	0	2.85916484	2.4109823	2.39307376	2.306009
26	**ENSG0000000274**	WNT16	protein_coding	chr7	121325367	121341104	+	Human GRCh	OTTHUMG000001	WNT16	**Wnt family member 16**	Extracellular	other		5.786319401	1	0.54782751	0	NA	0	0.54154609	0.56467464	1.13041455	0.54291096
27	**ENSG0000000274**	HECW1	protein_coding	chr7	43112629	43566001	+	Human GRCh	OTTHUMG000001	HECW1	**HECT, C2 and WW domain containing E3 ubiquitin protein liga**	Cytoplasm	enzyme		1.563706418	1	0.71724301	0	NA	0	2.04751519	1.3535817	1.82673084	2.05410256
28	**ENSG0000000282**	MAD1L1	protein_coding	chr7	1815793	2233243	-	Human GRCh	OTTHUMG000001	MAD1L1	**mitotic arrest deficient 1 like 1**	Nucleus	other		1.076229395	0	0.73323655	0	1	0	10.0336427	10.3397169	10.3595296	10.1864535
29	**ENSG0000000283**	LASP1	protein_coding	chr17	38869859	38921770	+	Human GRCh	OTTHUMG000001	LASP1	**LIM and SH3 protein 1**	Cytoplasm	transporter		1.121607131	0	0.54272524	0	1	0	10.6184942	10.8522806	10.6433858	11.0472207
30	**ENSG0000000291**	SNX11	protein_coding	chr17	48103357	48123601	+	Human GRCh	OTTHUMG000001	SNX11	**sorting nexin 11**	Cytoplasm	transporter		-1.112972751	0	0.66003225	0	1	0	8.56021704	8.62765134	8.65509985	8.27777109
31	**ENSG0000000293**	TMEM176	protein_coding	chr7	150800403	150805118	+	Human GRCh	OTTHUMG000001	TMEM176A	**transmembrane protein 176A**	Other	other		1	0	1	0	1	0	0.81254126	0.91753069	0.9008793	0.81900129
32	**ENSG0000000305**	M6PR	protein_coding	chr12	8940361	8949761	-	Human GRCh	OTTHUMG000001	M6PR	**mannose-6-phosphate receptor, cation dependent**	Cytoplasm	transporter	alglucosida	-1.150400403	0	0.50995467	0	1	0	9.96770484	10.2505366	10.0828703	9.82782478
33	**ENSG0000000309**	KLHL13	protein_coding	chrX	117897813	118117340	-	Human GRCh	OTTHUMG000000	KLHL13	**kelch like family member 13**	Cytoplasm	enzyme		1.444720839	0	0.41066052	0	1	0	5.23092958	5.55406155	5.4730458	5.97283837
34	**ENSG0000000313**	CYP26B1	protein_coding	chr2	72129238	72147862	-	Human GRCh	OTTHUMG000001	CYP26B1	**cytochrome P450 family 26 subfamily B member 1**	Cytoplasm	enzyme		-2.380367317	-1	0.03974948	1	1	0	4.32521562	5.06927218	3.8033483	4.11770397
35	**ENSG0000000314**	ICA1	protein_coding	chr7	8113184	8262687	-	Human GRCh	OTTHUMG000001	ICA1	**islet cell autoantigen 1**	Cytoplasm	other		-1.129838123	0	0.57864648	0	1	0	9.79192738	10.0715417	9.9720246	9.61704462
36	**ENSG0000000324**	DBNDD1	protein_coding	chr16	90004871	90019890	-	Human GRCh	OTTHUMG000001	DBNDD1	**dysbindin domain containing 1**	Other	other		1.03192047	0	0.93220559	0	1	0	9.74244385	9.9196392	9.92382881	9.81294489
37	**ENSG0000000339**	ALS2	protein_coding	chr2	201700267	201782112	-	Human GRCh	OTTHUMG000001	ALS2	**alsin Rho guanine nucleotide exchange factor ALS2**	Cytoplasm	other		-1.048281107	0	0.81456675	0	1	0	10.3935359	10.5935001	10.5480999	10.3364581
38	**ENSG0000000340**	CASP10	protein_coding	chr2	201182872	201229428	+	Human GRCh	OTTHUMG000001	CASP10	**caspase 10**	Cytoplasm	peptidase	caspase 1C	-1.588880318	-1	0.37049729	0	1	0	4.20086682	4.5418353	4.16280101	3.80492519
39	**ENSG0000000340**	CFLAR	protein_coding	chr2	201116154	201176687	+	Human GRCh	OTTHUMG000001	CFLAR	**CASP8 and FADD like apoptosis regulator**	Cytoplasm	other		-1.263404623	0	0.32372103	0	1	0	11.6139302	11.9563678	11.8191675	11.1767461
40	**ENSG0000000343**	TFPI	protein_coding	chr2	187464230	187565760	-	Human GRCh	OTTHUMG000001	TFPI	**tissue factor pathway inhibitor**	Extracellular	other	dalteparin,	-3.878502129	-1	0.33413426	0	1	0	3.10261011	1.42683764	1.40808115	2.07218342
41	**ENSG0000000350**	NDUFAF7	protein_coding	chr2	37231631	37253403	+	Human GRCh	OTTHUMG000001	NDUFAF7	**NADH:ubiquinone oxidoreductase complex assembly factor 7**	Cytoplasm	enzyme		1.064665761	0	0.78376719	0	1	0	8.66174283	9.02394616	9.0359559	8.80217167
42	**ENSG0000000375**	RBM5	protein_coding	chr3	50088919	50119021	+	Human GRCh	OTTHUMG000001	RBM5	**RNA binding motif protein 5**	Nucleus	other		-1.056048297	0	0.79354683	0	1	0	11.7165343	11.9753783	11.9538997	11.6087112
43	**ENSG0000000398**	MTMR7	protein_coding	chr8	17296794	17413528	-	Human GRCh	OTTHUMG000001	MTMR7	**myotubularin related protein 7**	Cytoplasm	phosphatase		1	0	1	0	1	0	1.462678	1.58459669	1.56524551	1.47017456
44	**ENSG0000000398**	SLC7A2	protein_coding	chr8	17497088	17570573	+	Human GRCh	OTTHUMG000001	SLC7A2	**solute carrier family 7 member 2**	Plasma Mem	transporter		1.118961779	0	0.85548888	0	1	0	7.44182803	7.89933745	7.92618012	7.68214266
45	**ENSG0000000405**	ARF5	protein_coding	chr7	127588386	127591700	+	Human GRCh	OTTHUMG000000	ARF5	**ADP ribosylation factor 5**	Cytoplasm	enzyme		-1.218299125	0	0.36007556	0	1	0	11.0111083	11.2995258	10.9901422	10.8932156
46	**ENSG0000000413**	SARM1	protein_coding	chr17	28364356	28404049	+	Human GRCh	OTTHUMG000001	SARM1	**sterile alpha and TIR motif containing 1**	Plasma Mem	transmembra		-1.110912366	0	0.66170173	0	1	0	10.8307975	11.0727896	11.0200546	10.6317623
47	**ENSG0000000414**	POLDIP2	protein_coding	chr17	28346633	28357527	-	Human GRCh	OTTHUMG000001	POLDIP2	**DNA polymerase delta interacting protein 2**	Cytoplasm	other		-1.221106996	0	0.3568587	0	1	0	11.5585745	11.9822786	11.6173758	11.5076262
48	**ENSG0000000439**	PLXND1	protein_coding	chr3	129555214	129606676	-	Human GRCh	OTTHUMG000001	PLXND1	**plexin D1**	Plasma Mem	transmembra		-1.151120292	0	0.71370677	0	1	0	6.61023397	6.69372444	6.24218844	6.76493734
49	**ENSG0000000445**	AK2	protein_coding	chr1	33007986	33080996	-	Human GRCh	OTTHUMG000000	AK2	**adenylate kinase 2**	Cytoplasm	kinase		-1.14589906	0	0.49804457	0	1	0	12.7130352	13.0536041	12.8860599	12.5804102
50	**ENSG0000000446**	CD38	protein_coding	chr4	15778275	15853232	+	Human GRCh	OTTHUMG000000	CD38	**CD38 molecule**	Plasma Mem	enzyme	XmAb1896	10.76106399	1	0.06551909	0	1	0	0.74602353	0.85192299	1.75174963	1.62672749
51	**ENSG0000000447**	FKBP4	protein_coding	chr12	2794970	2805423	+	Human GRCh	OTTHUMG000000	FKBP4	**FKBP prolyl isomerase 4**	Nucleus	enzyme		-1.053682439	0	0.79547831	0	1	0	12.2497968	12.4215797	12.3926777	12.1533082
52	**ENSG0000000448**	KDM1A	protein_coding	chr1	23019443	23083689	+	Human GRCh	OTTHUMG000000	KDM1A	**lysine demethylase 1A**	Nucleus	enzyme	SYHA1807,	-1.077741022	0	0.69711452	0	1	0	12.0675598	12.305588	12.2804326	11.9090452
53	**ENSG0000000453**	RBM6	protein_coding	chr3	49940007	50100045	+	Human GRCh	OTTHUMG000001	RBM6	**RNA binding motif protein 6**	Nucleus	other		-1.028861153	0	0.89115497	0	1	0	10.7712594	10.9428608	11.0257592	10.5936829
54	**ENSG0000000466**	CAMKK1	protein_coding	chr17	3860315	3894891	-	Human GRCh	OTTHUMG000000	CAMKK1	**calcium/calmodulin dependent protein kinase kinase 1**	Cytoplasm	kinase		1.355959669	0	0.14249487	0	1	0	8.32517418	8.67770576	8.77378335	8.91146515
55	**ENSG0000000470**	RECQL	protein_coding	chr12	21468910	21501669	-	Human GRCh	OTTHUMG000001	RECQL	**RecQ like helicase**	Nucleus	enzyme		-1.062004328	0	0.79014292	0	1	0	9.55383676	10.0659939	9.80834406	9.73306491
56	**ENSG0000000476**	VPS50	protein_coding	chr7	93232340	93361123	+	Human GRCh	OTTHUMG000001	VPS50	**VPS50 subunit of EARP/GARPII complex**	Cytoplasm	other		-1.169185924	0	0.48570588	0	1	0	9.77596129	9.98046341	9.91402612	9.4682
57	**ENSG0000000477**	HSPB6	protein_coding	chr19	35754566	35758079	-	Human GRCh	OTTHUMG000000	HSPB6	**heat shock protein family B (small) member 6**	Cytoplasm	other		1.366281574	0	0.24706601	0	1	0	9.66222617	10.0051433	10.0692476	10.3491454
58	**ENSG0000000477**	ARHGAP33	protein_coding	chr19	35774532	35788822	+	Human GRCh	OTTHUMG000001	ARHGAP33	**Rho GTPase activating protein 33**	Plasma Mem	transporter		1.038137043	0	0.8665911	0	1	0	11.1691472	11.5146776	11.4541483	11.3337286
59	**ENSG0000000477**	NDUFAB1	protein_coding	chr16	23581014	23596316	-	Human GRCh	OTTHUMG000000	NDUFAB1	**NADH:ubiquinone oxidoreductase subunit AB1**	Cytoplasm	enzyme		1.048541822	0	0.82624356	0	1	0	10.0371568	10.344646	10.357657	10.1403219
60	**ENSG0000000479**	PDK4	protein_coding	chr7	95583499	95596516	-	Human GRCh	OTTHUMG000001	PDK4	**pyruvate dehydrogenase kinase 4**	Cytoplasm	kinase		1.005207876	0	0.98736496	0	1	0	9.57570863	9.46034114	9.27498837	9.73175845
62	**ENSG0000000483**	ZMYND10	protein_coding	chr3	50341110	50345732	-	Human GRCh	OTTHUMG000001	ZMYND10	**zinc finger MYND-type containing 10**	Cytoplasm	other		1.003785228	0	0.99001922	0	1	0	8.31629173	8.74806079	8.73078807	8.35039427
63	**ENSG0000000484**	ABCB5	protein_coding	chr7	20615667	20777038	+	Human GRCh	OTTHUMG000000	ABCB5	**ATP binding cassette subfamily B member 5**	Plasma Mem	transporter		2.88636818	1	0.2745137	0	1	0	2.47262347	3.33586026	3.37142123	4.26113245
64	**ENSG0000000484**	ARX	protein_coding	chrX	25003694	25016420	-	Human GRCh	OTTHUMG000000	ARX	**aristaless related homeobox**	Nucleus	transcription		1.146683047	0	0.88335806	0	NA	0	2.96699017	3.3427126	2.96138343	3.51702955
65	**ENSG0000000486**	SLC25A13	protein_coding	chr7	96120220	96322147	-	Human GRCh	OTTHUMG000000	SLC25A13	**solute carrier family 25 member 13**	Cytoplasm	transporter		-1.102731164	0	0.67812776	0	1	0	10.8765303	11.3163811	11.1765894	10.8105219
66	**ENSG0000000486**	ST7	protein_coding	chr7	116953238	117230176	+	Human GRCh	OTTHUMG000000	ST7	**suppression of tumorigenicity 7**	Other	other		-1.234998483	0	0.33818143	0	1	0	9.96297662	10.2009606	9.8969562	9.8086982
67	**ENSG0000000489**	CDC27	protein_coding	chr17	47117703	47189422	-	Human GRCh	OTTHUMG000001	CDC27	**cell division cycle 27**	Nucleus	other		-1.068947989	0	0.75434136	0	1	0	11.3002015	11.6315224	11.4912908	11.3078706
69	**ENSG0000000494**	CALCR	protein_coding	chr7	93424486	93574730	-	Human GRCh	OTTHUMG000000	CALCR	**calcitonin receptor**	Plasma Mem	G-protein co	pramlintid	-5.137613377	-1	0.36972086	0	NA	0	1.31644253	0.97208768	0.97011678	0.96099819
70	**ENSG0000000496**	HCCS	protein_coding	chrX	11111301	11123086	+	Human GRCh	OTTHUMG000000	HCCS	**holocytochrome c synthase**	Cytoplasm	enzyme		1.035925052	0	0.89254448	0	1	0	8.59781887	8.64764424	8.75383083	8.56191981
71	**ENSG0000000497**	DVL2	protein_coding	chr17	7225342	7234517	-	Human GRCh	OTTHUMG000001	DVL2	**dishevelled segment polarity protein 2**	Cytoplasm	other		1.044172248	0	0.83332164	0	1	0	11.6729299	12.0377713	12.0481204	11.771238
72	**ENSG0000000500**	PRSS22	protein_coding	chr16	2852730	2858170	-	Human GRCh	OTTHUMG000001	PRSS22	**serine protease 22**	Extracellular	peptidase		2.735730632	1	0.56116523	0	1	0	1.64163145	1.67143991	1.93597209	1.64337599
73	**ENSG0000000500**	UPF1	protein_coding	chr19	18831959	18868230	+	Human GRCh	OTTHUMG000001	UPF1	**UPF1 RNA helicase and ATPase**	Nucleus	enzyme		1.002828361	0	0.98768952	0	1	0	12.0072701	12.3204479	12.3016021	12.0384045
74	**ENSG0000000502**	SKAP2	protein_coding	chr7	26667068	26995239	-	Human GRCh	OTTHUMG000000	SKAP2	**src kinase associated phosphoprotein 2**	Cytoplasm	other		1.183251449	0	0.55408607	0	1	0	8.95703787	9.32156769	9.29916954	9.35797401
75	**ENSG0000000502**	SLC25A5	protein_coding	chrX	119468422	119471396	+	Human GRCh	OTTHUMG000000	SLC25A5	**solute carrier family 25 member 5**	Cytoplasm	transporter	clodronic a	-1.149240427	0	0.54169522	0	1	0	12.1902905	12.6305142	12.35968	12.1830133
76	**ENSG0000000505**	MCUB	protein_coding	chr4	109560209	109688719	+	Human GRCh	OTTHUMG000001	MCUB	**mitochondrial calcium uniporter dominant negative subunit b**	Cytoplasm	other		1.302256328	0	0.35944922	0	1	0	8.7383434	9.19536889	9.37031215	9.18575458
77	**ENSG0000000507**	HOXA11	protein_coding	chr7	27181157	27185232	-	Human GRCh	OTTHUMG000000	HOXA11	**homeobox A11**	Nucleus	transcription		21.56729048	1	0.0274355	1	1	0	2.50080263	2.58640267	3.44873239	3.33109393
78	**ENSG0000000507**	POLR2J	protein_coding	chr7	102473128	102478922	-	Human GRCh	OTTHUMG000001	POLR2J	**RNA polymerase II subunit J**	Nucleus	enzyme		1.150504178	0	0.55983823	0	1	0	8.19528804	8.7388511	8.65250504	8.64864081
79	**ENSG0000000510**	DHX33	protein_coding	chr17	5440917	5468982	-	Human GRCh	OTTHUMG000001	DHX33	**DEAH-box helicase 33**	Nucleus	enzyme		-1.128714243	0	0.53515441	0	1	0	11.267225	11.6568558	11.5130677	11.1405307
81	**ENSG0000000510**	THSD7A	protein_coding	chr7	11370365	11832198	-	Human GRCh	OTTHUMG000001	THSD7A	**thrombospondin type 1 domain containing 7A**	Cytoplasm	other		-1.188809277	0	0.624801	0	1	0	8.87418947	9.10848302	9.10517624	8.39362276
82	**ENSG0000000515**	LIG3	protein_coding	chr17	34980512	35009743	+	Human GRCh	OTTHUMG000001	LIG3	**DNA ligase 3**	Nucleus	enzyme		-1.166675245	0	0.46878545	0	1	0	11.9452112	12.2006703	12.0794804	11.7066459
83	**ENSG0000000517**	RPAP3	protein_coding	chr12	47661249	47706030	-	Human GRCh	OTTHUMG000001	RPAP3	**RNA polymerase II associated protein 3**	Cytoplasm	enzyme		1.034754465	0	0.86701418	0	1	0	9.35016815	9.60338766	9.65104125	9.37281416

84	**ENSG0000000518**	ACSM3	protein_coding	chr16	20610243	20797581	+	Human GRCh	OTTHUMG000001	ACSM3	**acyl-CoA synthetase medium chain family member 3**	Cytoplasm	enzyme		-1.615457036	-1	0.2911165	0	1	0	6.42706647	6.76915497	6.45958688	5.59672696
85	**ENSG0000000518**	REXO5	protein_coding	chr16	20806429	20849665	+	Human GRCh	OTTHUMG000001	REXO5	**RNA exonuclease 5**	Nucleus	enzyme		-1.026584177	0	0.91767851	0	1	0	10.0192328	10.3854985	10.4428809	9.86105901
86	**ENSG0000000519**	CIAPIN1	protein_coding	chr16	57428187	57447420	-	Human GRCh	OTTHUMG000001	CIAPIN1	**cytokine induced apoptosis inhibitor 1**	Cytoplasm	other		-1.088134806	0	0.68350399	0	1	0	10.2580461	10.6240505	10.5114721	10.1856311
87	**ENSG0000000520**	SPPL2B	protein_coding	chr19	2328615	2355095	+	Human GRCh	OTTHUMG000001	SPPL2B	**signal peptide peptidase like 2B**	Plasma Mem	peptidase		-1.004386454	0	0.98479309	0	1	0	11.912982	12.0989885	12.1333505	11.8605409
88	**ENSG0000000523**	ATOSB	protein_coding	chr9	35104112	35116341	-	Human GRCh	OTTHUMG000000	ATOSB	**atos homolog B**	Nucleus	other		-1.148497395	0	0.64513386	0	1	0	8.24922026	8.79924944	8.32970645	8.46697007
90	**ENSG0000000524**	PRKAR2B	protein_coding	chr7	107044705	107161811	+	Human GRCh	OTTHUMG000001	PRKAR2B	**protein kinase cAMP-dependent type II regulatory subunit bet**	Cytoplasm	kinase		-1.0479343	0	0.87079365	0	1	0	8.70613963	9.21851103	9.07175346	8.78650892
91	**ENSG0000000530**	MSL3	protein_coding	chrX	11758159	11775772	+	Human GRCh	OTTHUMG000000	MSL3	**MSL complex subunit 3**	Nucleus	transcription		-1.191765375	0	0.43338001	0	1	0	9.75552411	10.1617181	9.92338607	9.62218913
92	**ENSG0000000533**	CREBBP	protein_coding	chr16	3725054	3880713	-	Human GRCh	OTTHUMG000001	CREBBP	**CREB binding protein**	Nucleus	transcription	FT-7051, P	-1.107809911	0	0.6071455	0	1	0	12.4570799	12.7322114	12.5768577	12.3969302
93	**ENSG0000000537**	TSPOAP1	protein_coding	chr17	58301228	58328795	-	Human GRCh	OTTHUMG000001	TSPOAP1	**TSPO associated protein 1**	Plasma Mem	other	zaleplon	1.190915285	0	0.63036789	0	1	0	8.65954713	8.66552556	9.25503754	8.19601419
95	**ENSG0000000542**	PON1	protein_coding	chr7	95297676	95324532	-	Human GRCh	OTTHUMG000001	PON1	**paraoxonase 1**	Extracellular	phosphatase		2.079531387	1	0.63957127	0	1	0	1.74447751	1.5461597	2.34235275	1.7533479
96	**ENSG0000000543**	GCFC2	protein_coding	chr2	75652000	75710985	-	Human GRCh	OTTHUMG000001	GCFC2	**GC-rich sequence DNA-binding factor 2**	Nucleus	transcription		-1.15718363	0	0.52571848	0	1	0	9.11228898	9.23772392	9.09859524	8.93756157
97	**ENSG0000000544**	WDR54	protein_coding	chr2	74421678	74425755	+	Human GRCh	OTTHUMG000001	WDR54	**WD repeat domain 54**	Other	other		1.28551815	0	0.31665459	0	1	0	8.8859387	9.04993855	9.32862606	9.15187069
98	**ENSG0000000546**	CROT	protein_coding	chr7	87345664	87399794	+	Human GRCh	OTTHUMG000000	CROT	**carnitine O-octanoyltransferase**	Cytoplasm	enzyme		1.016798442	0	0.96000996	0	1	0	7.97651237	8.51588837	8.61851698	7.84791917
99	**ENSG0000000547**	ABCB4	protein_coding	chr7	87401696	87480435	-	Human GRCh	OTTHUMG000000	ABCB4	**ATP binding cassette subfamily B member 4**	Plasma Mem	transporter		28.09025796	1	0.0221992	1	NA	0	1.49249986	1.51396134	2.6534724	1.89157575
100	**ENSG0000000548**	KMT2E	protein_coding	chr7	104940943	105115019	+	Human GRCh	OTTHUMG000001	KMT2E	**lysine methyltransferase 2E (inactive)**	Nucleus	enzyme		-1.004397835	0	0.98255692	0	1	0	11.865009	12.1284656	12.063392	11.9313508
101	**ENSG0000000548**	RHBDD2	protein_coding	chr7	75842602	75888926	+	Human GRCh	OTTHUMG000001	RHBDD2	**rhomboid domain containing 2**	Plasma Mem	other		-1.148429537	0	0.46953102	0	1	0	10.510111	10.6991378	10.5341771	10.3762404
102	**ENSG0000000551**	SOX8	protein_coding	chr16	981770	986979	+	Human GRCh	OTTHUMG000001	SOX8	**SRY-box transcription factor 8**	Nucleus	transcription		-1.063456914	0	0.92223542	0	1	0	5.05031025	4.94025871	5.09648793	4.74915072
103	**ENSG0000000570**	IBTK	protein_coding	chr6	82169986	82247754	-	Human GRCh	OTTHUMG000000	IBTK	**inhibitor of Bruton tyrosine kinase**	Cytoplasm	other		-1.019263745	0	0.92201684	0	1	0	11.0244666	11.3171823	11.3246498	10.9656569
104	**ENSG0000000580**	ZNF195	protein_coding	chr11	3339261	3379222	-	Human GRCh	OTTHUMG000000	ZNF195	**zinc finger protein 195**	Nucleus	transcription		1.013851611	0	0.94988913	0	1	0	10.9328578	11.0346584	11.180381	10.7863725
105	**ENSG0000000581**	MYCBP2	protein_coding	chr13	77042474	77327094	-	Human GRCh	OTTHUMG000000	MYCBP2	**MYC binding protein 2**	Nucleus	enzyme		-1.130298012	0	0.55666308	0	1	0	12.5775936	12.9165456	12.7658857	12.4567802
106	**ENSG0000000581**	FBXL3	protein_coding	chr13	76992598	77027195	-	Human GRCh	OTTHUMG000000	FBXL3	**F-box and leucine rich repeat protein 3**	Nucleus	enzyme		-1.114898928	0	0.61793808	0	1	0	10.0629027	10.2855821	10.1818621	9.92467914
108	**ENSG0000000588**	PDK2	protein_coding	chr17	50094737	50112152	+	Human GRCh	OTTHUMG000001	PDK2	**pyruvate dehydrogenase kinase 2**	Cytoplasm	kinase	AZD7545	-1.119547327	0	0.65516065	0	1	0	9.93139277	10.2721205	9.92169779	10.0455263
109	**ENSG0000000588**	ITGA3	protein_coding	chr17	50055968	50090481	+	Human GRCh	OTTHUMG000001	ITGA3	**integrin subunit alpha 3**	Plasma Mem	other		-1.060799344	0	0.7963358	0	1	0	8.84757032	8.64421521	8.85929418	8.48818934
110	**ENSG0000000588**	ZFX	protein_coding	chrX	24149173	24216255	+	Human GRCh	OTTHUMG000000	ZFX	**zinc finger protein X-linked**	Nucleus	transcription		-1.065770922	0	0.75857881	0	1	0	9.97555142	10.2057571	10.1184071	9.9319733
111	**ENSG0000000589**	LAMP2	protein_coding	chrX	120426148	120469365	-	Human GRCh	OTTHUMG000000	LAMP2	**lysosomal associated membrane protein 2**	Plasma Mem	other		-1.089232031	0	0.7031161	0	1	0	10.9245703	11.1882649	11.1641901	10.7379245
112	**ENSG0000000596**	ITGA2B	protein_coding	chr17	44372180	44389649	-	Human GRCh	OTTHUMG000001	ITGA2B	**integrin subunit alpha 2b**	Plasma Mem	transmembra	abciximab,	-1.124983972	0	0.7405686	0	1	0	5.62778579	5.43080987	5.48697213	5.35702936
114	**ENSG0000000600**	GDE1	protein_coding	chr16	19501693	19522123	-	Human GRCh	OTTHUMG000001	GDE1	**glycerophosphodiester phosphodiesterase 1**	Plasma Mem	enzyme		-1.160923095	0	0.58255255	0	1	0	11.2304535	11.5723347	11.4385848	11.007207
115	**ENSG0000000601**	REX1BD	protein_coding	chr19	18588685	18592337	+	Human GRCh	OTTHUMG000001	REX1BD	**required for excision 1-B domain containing**	Other	other		1.126608775	0	0.50305683	0	1	0	10.0118369	10.1712218	10.3086679	10.1393289
116	**ENSG0000000601**	CRLF1	protein_coding	chr19	18572220	18607741	-	Human GRCh	OTTHUMG000001	CRLF1	**cytokine receptor like factor 1**	Extracellular	cytokine		-1.240547375	0	0.56761628	0	1	0	6.49113085	6.25848025	6.05363509	6.25876696
117	**ENSG0000000602**	OSBPL7	protein_coding	chr17	47807372	47821803	-	Human GRCh	OTTHUMG000001	OSBPL7	**oxysterol binding protein like 7**	Cytoplasm	other		5.417409121	1	0.01116223	1	1	0	3.48804195	2.79377427	3.61218846	4.59234028
118	**ENSG0000000604**	TMEM98	protein_coding	chr17	32927910	32945106	+	Human GRCh	OTTHUMG000001	TMEM98	**transmembrane protein 98**	Cytoplasm	other		-1.176506175	0	0.53055872	0	1	0	8.94149296	9.23595575	8.9564687	8.88013111
119	**ENSG0000000604**	YBX2	protein_coding	chr17	7288263	7294639	-	Human GRCh	OTTHUMG000001	YBX2	**Y-box binding protein 2**	Cytoplasm	translation re		-1.196682215	0	0.50866319	0	1	0	8.98088824	9.66161418	9.51904551	8.65486491
121	**ENSG0000000606**	MAP3K14	protein_coding	chr17	45263119	45317029	-	Human GRCh	OTTHUMG000001	MAP3K14	**mitogen-activated protein kinase kinase kinase 14**	Cytoplasm	kinase	CW15337	1.10153474	0	0.63842812	0	1	0	9.35438694	9.54750912	9.65276908	9.45783712
122	**ENSG0000000607**	ABCC8	protein_coding	chr11	17392498	17476894	-	Human GRCh	OTTHUMG000001	ABCC8	**ATP binding cassette subfamily C member 8**	Plasma Mem	transporter	nateglinide	-1.501092513	-1	0.54778451	0	1	0	5.08331151	5.67502061	4.8466705	5.31921714
124	**ENSG0000000611**	TMEM132	protein_coding	chr11	60924460	60937159	+	Human GRCh	OTTHUMG000001	TMEM132A	**transmembrane protein 132A**	Cytoplasm	other		1.217341394	0	0.49538441	0	1	0	12.0266695	12.2747514	12.5225276	12.2172269
125	**ENSG0000000612**	AP2B1	protein_coding	chr17	35578046	35726413	+	Human GRCh	OTTHUMG000001	AP2B1	**adaptor related protein complex 2 subunit beta 1**	Plasma Mem	transporter		-1.243973902	0	0.34915191	0	1	0	13.2110957	13.6135003	13.4077871	12.9035376
127	**ENSG0000000619**	ZNF263	protein_coding	chr16	3283283	3301401	+	Human GRCh	OTTHUMG000001	ZNF263	**zinc finger protein 263**	Nucleus	transcription		-1.118952936	0	0.60550459	0	1	0	9.37772327	9.40069726	9.43319717	9.07077953
128	**ENSG0000000621**	CX3CL1	protein_coding	chr16	57372477	57385044	+	Human GRCh	OTTHUMG000001	CX3CL1	**C-X3-C motif chemokine ligand 1**	Extracellular	cytokine		-1.105364688	0	0.69168202	0	1	0	11.2512047	11.4121373	11.4055593	10.9928648
129	**ENSG0000000628**	SPATA20	protein_coding	chr17	50543058	50555852	+	Human GRCh	OTTHUMG000001	SPATA20	**spermatogenesis associated 20**	Other	other		-1.058034012	0	0.77224427	0	1	0	10.4350038	10.6067791	10.6020456	10.3027787
130	**ENSG0000000628**	CACNA1G	protein_coding	chr17	50560715	50627474	+	Human GRCh	OTTHUMG000001	CACNA1G	**calcium voltage-gated channel subunit alpha1 G**	Plasma Mem	ion channel	cinnarizine	3.158946746	1	0.01311085	1	1	0	3.53892892	4.20221646	4.9154358	4.68259597
131	**ENSG0000000632**	TNFRSF12	protein_coding	chr16	3018445	3022383	+	Human GRCh	OTTHUMG000001	TNFRSF12A	**TNF receptor superfamily member 12A**	Plasma Mem	transmembra	enavatuzu	1.79900365	1	0.03377826	1	1	0	7.22487734	7.15911407	7.79977548	7.87770189
132	**ENSG0000000637**	DLX6	protein_coding	chr7	97005553	97011040	+	Human GRCh	OTTHUMG000001	DLX6	**distal-less homeobox 6**	Nucleus	transcription		-1.124234836	0	0.63286139	0	1	0	7.72658015	8.13242068	8.00936764	7.59770968
133	**ENSG0000000643**	MAP3K9	protein_coding	chr14	70722526	70809534	-	Human GRCh	OTTHUMG000001	MAP3K9	**mitogen-activated protein kinase kinase kinase 9**	Cytoplasm	kinase		-1.160681954	0	0.52015927	0	1	0	10.8545232	11.1539965	11.0430703	10.6204532
134	**ENSG0000000645**	RALA	protein_coding	chr7	39623565	39708120	+	Human GRCh	OTTHUMG000001	RALA	**RAS like proto-oncogene A**	Cytoplasm	enzyme		1.011412146	0	0.95659783	0	1	0	9.96971767	10.3607779	10.3969756	9.94748423
135	**ENSG0000000645**	BAIAP2L1	protein_coding	chr7	98291650	98401090	-	Human GRCh	OTTHUMG000001	BAIAP2L1	**BAR/IMD domain containing adaptor protein 2 like 1**	Cytoplasm	other		-1.252506397	0	0.23671822	0	1	0	10.366542	10.5967311	10.3076597	10.1610826
136	**ENSG0000000645**	KDM7A	protein_coding	chr7	140084746	140176983	-	Human GRCh	OTTHUMG000001	KDM7A	**lysine demethylase 7A**	Nucleus	enzyme		-1.1333277	0	0.59308448	0	1	0	11.5866843	11.8851062	11.7504892	11.4386608
137	**ENSG0000000646**	ETV1	protein_coding	chr7	13891229	13991425	-	Human GRCh	OTTHUMG000001	ETV1	**ETS variant transcription factor 1**	Nucleus	transcription		-1.788095932	-1	0.07467687	0	1	0	8.39538015	8.81013802	7.9370593	8.04534376
138	**ENSG0000000653**	AGK	protein_coding	chr7	141551278	141655244	+	Human GRCh	OTTHUMG000001	AGK	**acylglycerol kinase**	Cytoplasm	kinase	SM1-71	-1.025370863	0	0.91190397	0	1	0	9.78090214	10.068801	10.1157908	9.65086169
139	**ENSG0000000653**	ALDH3B1	protein_coding	chr11	68008578	68029282	+	Human GRCh	OTTHUMG000001	ALDH3B1	**aldehyde dehydrogenase 3 family member B1**	Cytoplasm	enzyme		5.060917782	1	0.01297867	1	1	0	4.0131927	2.86491359	3.57639689	5.32181483
140	**ENSG0000000655**	TTC22	protein_coding	chr1	54779712	54801323	-	Human GRCh	OTTHUMG000000	TTC22	**tetratricopeptide repeat domain 22**	Other	other		10.33004524	1	0.07877218	0	1	0	2.06988271	2.12991776	2.12010893	2.90003341
141	**ENSG0000000657**	PHTF2	protein_coding	chr7	77798773	77957503	+	Human GRCh	OTTHUMG000001	PHTF2	**putative homeodomain transcription factor 2**	Cytoplasm	other		-1.055353777	0	0.8167271	0	1	0	10.9572169	11.258865	11.2750628	10.7927434
143	**ENSG0000000660**	FARP2	protein_coding	chr2	241356285	241494841	+	Human GRCh	OTTHUMG000001	FARP2	**FERM, ARH/RhoGEF and pleckstrin domain protein 2**	Cytoplasm	other		-1.049959035	0	0.80982081	0	1	0	10.8904961	11.3618135	11.2515424	10.9160904
144	**ENSG0000000661**	USH1C	protein_coding	chr11	17493895	17544416	-	Human GRCh	OTTHUMG000001	USH1C	**USH1 protein network component harmonin**	Plasma Mem	other		-7.903282364	-1	0.01628522	1	1	0	3.51173651	4.351615	2.8267389	3.21075975
145	**ENSG0000000662**	GGCT	protein_coding	chr7	30496621	30504841	-	Human GRCh	OTTHUMG000001	GGCT	**gamma-glutamylcyclotransferase**	Cytoplasm	enzyme		1.031523622	0	0.89911826	0	1	0	9.81995157	10.1629754	10.0631136	10.0112101
146	**ENSG0000000663**	DBF4	protein_coding	chr7	87876216	87909553	+	Human GRCh	OTTHUMG000001	DBF4	**DBF4 zinc finger**	Nucleus	kinase		-1.109916838	0	0.61783531	0	1	0	10.2020609	10.5875317	10.4293695	10.1487737
147	**ENSG0000000663**	TBXA2R	protein_coding	chr19	3594507	3606875	-	Human GRCh	OTTHUMG000001	TBXA2R	**thromboxane A2 receptor**	Plasma Mem	G-protein co	S-18886, if	-1.120236953	0	0.80066673	0	1	0	6.44358542	6.61344069	6.21503372	6.5748484
148	**ENSG0000000665**	IFRD1	protein_coding	chr7	112422887	112481017	+	Human GRCh	OTTHUMG000001	IFRD1	**interferon related developmental regulator 1**	Nucleus	other		1.090723385	0	0.69258876	0	1	0	10.2741645	10.2781771	10.6046381	10.0828081
150	**ENSG0000000669**	COX10	protein_coding	chr17	14069490	14231736	+	Human GRCh	OTTHUMG000000	COX10	**cytochrome c oxidase assembly factor heme A:farnesyltransfe**	Cytoplasm	enzyme		-1.024317541	0	0.91173427	0	1	0	9.60630829	9.97921635	9.85172715	9.70406883
151	**ENSG0000000670**	GTF2IRD1	protein_coding	chr7	74453790	74602605	+	Human GRCh	OTTHUMG000000	GTF2IRD1	**GTF2I repeat domain containing 1**	Nucleus	transcription		-1.186171	0	0.4501466	0	1	0	10.4804168	10.560021	10.4425369	10.226069
152	**ENSG0000000671**	PAF1	protein_coding	chr19	39385629	39391154	-	Human GRCh	OTTHUMG000001	PAF1	**PAF1 homolog, Paf1/RNA polymerase II complex component**	Nucleus	other		-1.071175613	0	0.74726693	0	1	0	10.4941413	10.8671627	10.6209096	10.6137545
153	**ENSG0000000671**	VPS41	protein_coding	chr7	38722974	38932394	-	Human GRCh	OTTHUMG000000	VPS41	**VPS41 subunit of HOPS complex**	Cytoplasm	transporter		-1.116352792	0	0.57901122	0	1	0	10.0810646	10.3469144	10.2399938	9.9468551
154	**ENSG0000000674**	ARHGAP4	protein_coding	chr17	12789498	12991643	+	Human GRCh	OTTHUMG000000	ARHGAP44	**Rho GTPase activating protein 44**	Cytoplasm	other		-1.23711288	0	0.37197326	0	1	0	8.62318603	8.69740622	8.46235307	8.40423329
155	**ENSG0000000674**	ELAC2	protein_coding	chr17	12991612	13018065	-	Human GRCh	OTTHUMG000000	ELAC2	**elaC ribonuclease Z 2**	Nucleus	enzyme		-1.092814583	0	0.6490776	0	1	0	11.4286798	11.8335646	11.6324765	11.4550955
156	**ENSG0000000674**	SCIN	protein_coding	chr7	12570577	12660182	+	Human GRCh	OTTHUMG000001	SCIN	**scinderin**	Cytoplasm	other		1.987646561	1	0.12943084	0	1	0	4.01524655	4.56432839	5.06060563	4.41401776
157	**ENSG0000000675**	ARSD	protein_coding	chrX	2903972	2929349	-	Human GRCh	OTTHUMG000000	ARSD	**arylsulfatase D**	Cytoplasm	enzyme		-1.171121908	0	0.54200439	0	1	0	9.25063406	9.37768959	9.32764668	8.92363074
158	**ENSG0000000675**	PNPLA4	protein_coding	chrX	7898247	7927739	-	Human GRCh	OTTHUMG000000	PNPLA4	**patatin like phospholipase domain containing 4**	Cytoplasm	enzyme		1.114811108	0	0.72591241	0	1	0	7.39846424	8.02964228	7.84579831	7.90118218
160	**ENSG0000000683**	ADIPOR2	protein_coding	chr12	1688574	1788674	+	Human GRCh	OTTHUMG000001	ADIPOR2	**adiponectin receptor 2**	Plasma Mem	transmembra		-1.073198493	0	0.74079794	0	1	0	10.5276576	10.9241009	10.8189791	10.4861996
161	**ENSG0000000683**	CDKL3	protein_coding	chr5	134286350	134371047	-	Human GRCh	OTTHUMG000001	CDKL3	**cyclin dependent kinase like 3**	Cytoplasm	kinase		-1.186392177	0	0.58741091	0	1	0	6.31863778	6.89260479	6.63772539	6.26182968
163	**ENSG0000000703**	PRSS21	protein_coding	chr16	2817180	2826304	+	Human GRCh	OTTHUMG000001	PRSS21	**serine protease 21**	Cytoplasm	peptidase		-1.134317467	0	0.62742605	0	1	0	9.30225339	9.51521553	9.52859941	8.91908443
164	**ENSG0000000704**	MARK4	protein_coding	chr19	45079288	45305284	+	Human GRCh	OTTHUMG000001	MARK4	**microtubule affinity regulating kinase 4**	Cytoplasm	kinase		1.194347025	0	0.41405694	0	1	0	10.1109399	10.2290848	10.4481956	10.2870561
166	**ENSG0000000708**	CCDC124	protein_coding	chr19	17933015	17943991	+	Human GRCh	OTTHUMG000001	CCDC124	**coiled-coil domain containing 124**	Other	other		1.248915399	0	0.28648465	0	1	0	10.4052148	10.66156	10.7906348	10.789387
168	**ENSG0000000716**	PAFAH1B1	protein_coding	chr17	2593210	2685615	+	Human GRCh	OTTHUMG000001	PAFAH1B1	**platelet activating factor acetylhydrolase 1b regulatory subuni**	Cytoplasm	enzyme		1.024947027	0	0.90193508	0	1	0	12.1431137	12.3542471	12.4382822	12.0981259
169	**ENSG0000000717**	NOS2	protein_coding	chr17	27756766	27800529	-	Human GRCh	OTTHUMG000001	NOS2	**nitric oxide synthase 2**	Cytoplasm	enzyme	ASP9853,	-10.7676705	-1	0.04038164	1	1	0	2.64665612	1.60208017	1.583062	1.48969923
171	**ENSG0000000720**	BLTP2	protein_coding	chr17	28614446	28645454	-	Human GRCh	OTTHUMG000001	BLTP2	**bridge-like lipid transfer protein family member 2**	Extracellular	other		-1.163393425	0	0.43124571	0	1	0	13.1877312	13.5121782	13.2813149	13.094325
172	**ENSG0000000721**	SLC13A2	protein_coding	chr17	28473293	28497781	+	Human GRCh	OTTHUMG000001	SLC13A2	**solute carrier family 13 member 2**	Plasma Mem	transporter		-2.759678648	-1	0.49761734	0	1	0	1.63702423	2.07188762	1.6942379	1.64112427
173	**ENSG0000000723**	GAS7	protein_coding	chr17	9910606	10198606	-	Human GRCh	OTTHUMG000001	GAS7	**growth arrest specific 7**	Cytoplasm	other		-1.233098876	0	0.78722736	0	1	0	4.43023042	4.69058589	4.36809111	4.32942113
174	**ENSG0000000725**	TRAPPC6A	protein_coding	chr19	45162928	45178237	-	Human GRCh	OTTHUMG000001	TRAPPC6A	**trafficking protein particle complex subunit 6A**	Cytoplasm	other		1.127774659	0	0.66148676	0	1	0	8.66138018	9.07667048	9.24488091	8.7470339
175	**ENSG0000000726**	MATK	protein_coding	chr19	3777970	3802129	-	Human GRCh	OTTHUMG000001	MATK	**megakaryocyte-associated tyrosine kinase**	Cytoplasm	kinase		1.046528815	0	0.88545042	0	1	0	9.46614167	9.47846612	9.68457758	9.34658978
176	**ENSG0000000730**	CEACAM7	protein_coding	chr19	41673303	41706976	-	Human GRCh	OTTHUMG000001	CEACAM7	**CEA cell adhesion molecule 7**	Plasma Mem	other		1	0	1	0	1	0	1.32943274	1.3630037	1.35747298	1.33140944
178	**ENSG0000000731**	SCN4A	protein_coding	chr17	63938554	63972918	-	Human GRCh	OTTHUMG000001	SCN4A	**sodium voltage-gated channel alpha subunit 4**	Plasma Mem	ion channel	diclofenac,	-2.225102496	-1	0.32912886	0	NA	0	3.5532627	3.15691448	3.01024837	2.61367348
179	**ENSG0000000734**	ST7L	protein_coding	chr1	112523514	112620825	-	Human GRCh	OTTHUMG000000	ST7L	**suppression of tumorigenicity 7 like**	Other	other		-1.016768558	0	0.94111604	0	1	0	9.49511243	9.78455157	9.81942455	9.40515118
180	**ENSG0000000735**	TKTL1	protein_coding	chrX	154295795	154330350	+	Human GRCh	OTTHUMG000000	TKTL1	**transketolase like 1**	Cytoplasm	enzyme		-1.199266555	0	0.85848978	0	1	0	4.93266624	5.27144282	3.75447798	5.4875064
181	**ENSG0000000737**	PAX6	protein_coding	chr11	31784779	31817961	-	Human GRCh	OTTHUMG000000	PAX6	**paired box 6**	Nucleus	transcription		-1.299628607	0	0.34179507	0	1	0	8.85627097	9.1229687	8.65176638	8.75571881

182	**ENSG0000000737**	RPUSD1	protein_coding	chr16	784974	788397	-	Human GRCh	OTTHUMG000000	RPUSD1	**RNA pseudouridine synthase domain containing 1**	Other	enzyme		1.131104467	0	0.59425168	0	1	0	10.4161037	10.567807	10.7879718	10.4572955
183	**ENSG0000000738**	RHBDF1	protein_coding	chr16	58059	76355	-	Human GRCh	OTTHUMG000000	RHBDF1	**rhomboid 5 homolog 1**	Cytoplasm	other		1.018573675	0	0.94911452	0	1	0	9.91004743	10.0947421	10.0190851	10.0309357
184	**ENSG0000000739**	LUC7L	protein_coding	chr16	188969	229463	-	Human GRCh	OTTHUMG000000	LUC7L	**LUC7 like**	Nucleus	other		1.036358453	0	0.86659195	0	1	0	11.1641656	11.2126294	11.4276766	10.9866068
185	**ENSG0000000740**	CACNA2D2	protein_coding	chr3	50362613	50504244	-	Human GRCh	OTTHUMG000001	CACNA2D2	**calcium voltage-gated channel auxiliary subunit alpha2delta 2**	Plasma Mem	ion channel	gabapentir	1.136884836	0	0.82782394	0	1	0	8.17113064	8.10245792	8.18737316	8.32125429
186	**ENSG0000000751**	BAIAP3	protein_coding	chr16	1333638	1349439	+	Human GRCh	OTTHUMG000000	BAIAP3	**BAI1 associated protein 3**	Extracellular	other		1.104562515	0	0.75479572	0	1	0	9.76454838	10.2041242	10.2006623	10.0222844
187	**ENSG0000000752**	TSR3	protein_coding	chr16	1349240	1351878	-	Human GRCh	OTTHUMG000000	TSR3	**TSR3 ribosome maturation factor**	Cytoplasm	enzyme		1.188828482	0	0.47976343	0	1	0	8.31953802	8.6000343	8.67118987	8.64340586
188	**ENSG0000000754**	PIGQ	protein_coding	chr16	566995	584109	+	Human GRCh	OTTHUMG000001	PIGQ	**phosphatidylinositol glycan anchor biosynthesis class Q**	Cytoplasm	enzyme		-1.074567644	0	0.74564318	0	1	0	11.4679022	11.6814536	11.6033525	11.3832664
189	**ENSG0000000754**	CRAMP1	protein_coding	chr16	1612337	1677908	+	Human GRCh	OTTHUMG000000	CRAMP1	**cramped chromatin regulator homolog 1**	Other	other		-1.145955837	0	0.49319486	0	1	0	11.452426	11.7384963	11.5680484	11.321939
190	**ENSG0000000786**	TEAD3	protein_coding	chr6	35473597	35497079	-	Human GRCh	OTTHUMG000000	TEAD3	**TEA domain transcription factor 3**	Nucleus	transcription		-1.012400515	0	0.96040579	0	1	0	9.14009702	9.48577779	9.41538569	9.19874693
192	**ENSG0000000792**	DNAJC11	protein_coding	chr1	6634168	6701924	-	Human GRCh	OTTHUMG000000	DNAJC11	**DnaJ heat shock protein family (Hsp40) member C11**	Cytoplasm	other		-1.152802422	0	0.4681459	0	1	0	11.7049631	12.027938	11.8748257	11.5399635
193	**ENSG0000000793**	FMO3	protein_coding	chr1	171090901	171117819	+	Human GRCh	OTTHUMG000000	FMO3	**flavin containing dimethylaniline monoxygenase 3**	Cytoplasm	enzyme		1	0	1	0	NA	0	0.67196816	0.69389881	0.69028071	0.67325721
194	**ENSG0000000794**	MYLIP	protein_coding	chr6	16129086	16148248	+	Human GRCh	OTTHUMG000000	MYLIP	**myosin regulatory light chain interacting protein**	Cytoplasm	enzyme		-1.155375033	0	0.50785216	0	1	0	8.27292646	8.42560101	8.28673942	8.10205089
196	**ENSG0000000796**	E2F2	protein_coding	chr1	23506438	23531233	-	Human GRCh	OTTHUMG000000	E2F2	**E2F transcription factor 2**	Nucleus	transcription		-1.00806477	0	0.970544	0	1	0	10.3362586	10.7952402	10.5086719	10.6451953
197	**ENSG0000000801**	PSMB1	protein_coding	chr6	170535120	170553307	-	Human GRCh	OTTHUMG000000	PSMB1	**proteasome 20S subunit beta 1**	Cytoplasm	peptidase	bortezomi	-1.035958392	0	0.85962853	0	1	0	10.8346034	11.1925441	11.0296297	10.9442817
198	**ENSG0000000805**	SYN1	protein_coding	chrX	47571901	47619857	-	Human GRCh	OTTHUMG000000	SYN1	**synapsin I**	Plasma Mem	other		-1.208587031	0	0.69106231	0	1	0	4.20379604	3.64643451	4.01454843	3.60059705
199	**ENSG0000000808**	JARID2	protein_coding	chr6	15246069	15522042	+	Human GRCh	OTTHUMG000000	JARID2	**jumonji and AT-rich interaction domain containing 2**	Nucleus	transcription		-1.19765933	0	0.37721605	0	1	0	10.5356268	10.893214	10.5236263	10.5264704
200	**ENSG0000000808**	CDKL5	protein_coding	chrX	18425583	18653629	+	Human GRCh	OTTHUMG000000	CDKL5	**cyclin dependent kinase like 5**	Nucleus	kinase		1.170973281	0	0.49181702	0	1	0	8.54858764	8.81898293	8.90348371	8.81703734
202	**ENSG0000000812**	CDK11A	protein_coding	chr1	1702379	1724357	-	Human GRCh	OTTHUMG000000	CDK11A	**cyclin dependent kinase 11A**	Nucleus	kinase		1.15386251	0	0.56470944	0	1	0	7.96560357	8.35435718	8.47700089	8.15379493
203	**ENSG0000000813**	NADK	protein_coding	chr1	1751232	1780457	-	Human GRCh	OTTHUMG000000	NADK	**NAD kinase**	Cytoplasm	kinase		-1.07466969	0	0.71823698	0	1	0	11.4230344	11.7469696	11.6535842	11.3587377
204	**ENSG0000000819**	TFAP2B	protein_coding	chr6	50818723	50847619	+	Human GRCh	OTTHUMG000000	TFAP2B	**transcription factor AP-2 beta**	Nucleus	transcription		-1.397501905	0	0.61777149	0	1	0	2.78326492	3.08972162	2.76181746	2.63858737
205	**ENSG0000000819**	TFAP2D	protein_coding	chr6	50713526	50773033	+	Human GRCh	OTTHUMG000000	TFAP2D	**transcription factor AP-2 delta**	Nucleus	transcription		1	0	1	0	NA	0	0.61499915	0.64772953	0.64238631	0.61694646
206	**ENSG0000000822**	DLEC1	protein_coding	chr3	38039205	38124025	+	Human GRCh	OTTHUMG000001	DLEC1	**DLEC1 cilia and flagella associated protein**	Cytoplasm	other		1.215568568	0	0.66274048	0	1	0	4.21377204	3.5946503	4.37698622	3.82448003
207	**ENSG0000000825**	CYTH3	protein_coding	chr7	6161779	6272644	-	Human GRCh	OTTHUMG000000	CYTH3	**cytohesin 3**	Cytoplasm	other		-1.212670883	0	0.43669694	0	1	0	10.7560173	11.1242121	10.9279659	10.507871
208	**ENSG0000000827**	ADAM22	protein_coding	chr7	87934143	88202889	+	Human GRCh	OTTHUMG000001	ADAM22	**ADAM metallopeptidase domain 22**	Plasma Mem	peptidase		-1.05633373	0	0.84933368	0	1	0	8.74243414	8.69590526	8.85831982	8.43202414
209	**ENSG0000000828**	SYPL1	protein_coding	chr7	106090505	106112589	-	Human GRCh	OTTHUMG000001	SYPL1	**synaptophysin like 1**	Plasma Mem	transporter		1.120982426	0	0.61928116	0	1	0	10.2194022	10.5736084	10.7187756	10.3237238
210	**ENSG0000000828**	CYB561	protein_coding	chr17	63432304	63446354	-	Human GRCh	OTTHUMG000001	CYB561	**cytochrome b561**	Cytoplasm	enzyme		-1.1228106	0	0.54413671	0	1	0	11.8268934	12.0557583	11.9834913	11.6202034
211	**ENSG0000000829**	SPAG9	protein_coding	chr17	50962174	51120868	-	Human GRCh	OTTHUMG000001	SPAG9	**sperm associated antigen 9**	Cytoplasm	other		-1.133141709	0	0.51760353	0	1	0	11.8036512	12.1380592	11.9990557	11.6640822
212	**ENSG0000000830**	CELSR3	protein_coding	chr3	48636463	48662886	-	Human GRCh	OTTHUMG000001	CELSR3	**cadherin EGF LAG seven-pass G-type receptor 3**	Plasma Mem	G-protein cou		1.006713981	0	0.97618155	0	1	0	11.1821402	11.5453717	11.5248475	11.2264135
213	**ENSG0000000831**	AASS	protein_coding	chr7	122064583	122144308	-	Human GRCh	OTTHUMG000001	AASS	**aminoadipate-semialdehyde synthase**	Cytoplasm	enzyme		-1.04021185	0	0.9067953	0	1	0	8.97580296	9.01258663	9.09771478	8.78337643
214	**ENSG0000000832**	PLEKHG6	protein_coding	chr12	6310436	6328506	+	Human GRCh	OTTHUMG000001	PLEKHG6	**pleckstrin homology and RhoGEF domain containing G6**	Cytoplasm	other		1.067016952	0	0.84878178	0	1	0	6.58316012	6.43635067	6.08267907	6.91274637
215	**ENSG0000000832**	SS18L2	protein_coding	chr3	42581840	42596934	+	Human GRCh	OTTHUMG000001	SS18L2	**SS18 like 2**	Other	other		-1.143260238	0	0.64208439	0	1	0	7.97547934	8.11757484	7.99656591	7.80323594
216	**ENSG0000000838**	MPND	protein_coding	chr19	4343527	4360086	+	Human GRCh	OTTHUMG000001	MPND	**MPN domain containing**	Other	other		1.022907352	0	0.91870562	0	1	0	8.83570264	9.26760777	9.20901048	8.97245475
217	**ENSG0000000839**	MGST1	protein_coding	chr12	16347142	16609259	+	Human GRCh	OTTHUMG000001	MGST1	**microsomal glutathione S-transferase 1**	Cytoplasm	enzyme		-1.237251504	0	0.52915656	0	1	0	8.92308405	9.22104869	8.77868546	8.9411222
218	**ENSG0000000840**	CRY1	protein_coding	chr12	106991364	107093549	-	Human GRCh	OTTHUMG000001	CRY1	**cryptochrome circadian regulator 1**	Nucleus	enzyme		1.017891663	0	0.92487047	0	1	0	10.1103807	10.379986	10.3410128	10.1986512
220	**ENSG0000000844**	NFIX	protein_coding	chr19	12995475	13098796	+	Human GRCh	OTTHUMG000001	NFIX	**nuclear factor I X**	Nucleus	transcription		1.063830147	0	0.77416972	0	1	0	14.2272799	14.479515	14.5414202	14.3089158
221	**ENSG0000000851**	ST3GAL1	protein_coding	chr8	133454848	133571926	-	Human GRCh	OTTHUMG000001	ST3GAL1	**ST3 beta-galactoside alpha-2,3-sialyltransferase 1**	Cytoplasm	enzyme		1.228178528	0	0.57127297	0	1	0	10.2345876	10.1956318	10.6745112	10.2062015
223	**ENSG0000000851**	IL32	protein_coding	chr16	3065297	3082192	+	Human GRCh	OTTHUMG000001	IL32	**interleukin 32**	Extracellular	cytokine		-5.191053484	-1	0.31539106	0	NA	0	1.63548951	1.28195997	1.27937412	1.26739446
224	**ENSG0000000871**	PKD1	protein_coding	chr16	2088708	2135898	-	Human GRCh	OTTHUMG000001	PKD1	**polycystin 1, transient receptor potential channel interacting**	Plasma Mem	ion channel		1.074582173	0	0.76447118	0	1	0	12.3638649	12.5817662	12.660404	12.441242
225	**ENSG0000000873**	MAPK8IP2	protein_coding	chr22	50600793	50613981	+	Human GRCh	OTTHUMG000001	MAPK8IP2	**mitogen-activated protein kinase 8 interacting protein 2**	Cytoplasm	other		1.1038187	0	0.61878863	0	1	0	11.1151181	11.3565049	11.4236451	11.2773521
226	**ENSG0000000883**	MED24	protein_coding	chr17	40019097	40061215	-	Human GRCh	OTTHUMG000001	MED24	**mediator complex subunit 24**	Nucleus	transcription		-1.145340533	0	0.48494553	0	1	0	11.905279	12.2676347	12.073195	11.8065546
227	**ENSG0000000885**	RHOBTB2	protein_coding	chr8	22987417	23020509	+	Human GRCh	OTTHUMG000000	RHOBTB2	**Rho related BTB domain containing 2**	Cytoplasm	enzyme		1.119725889	0	0.59986827	0	1	0	9.77632353	9.77412427	10.0496892	9.7305742
228	**ENSG0000000886**	HEATR5B	protein_coding	chr2	36968383	37084372	-	Human GRCh	OTTHUMG000001	HEATR5B	**HEAT repeat containing 5B**	Cytoplasm	other		-1.132553985	0	0.56137792	0	1	0	10.8501382	11.2036062	11.0037682	10.7884526
229	**ENSG0000000895**	SEC62	protein_coding	chr3	169966635	169998373	+	Human GRCh	OTTHUMG000001	SEC62	**SEC62 homolog, preprotein translocation factor**	Cytoplasm	transporter		1.011700419	0	0.95272023	0	1	0	10.5781951	10.7059783	10.7438122	10.5622725
230	**ENSG0000000898**	RPS20	protein_coding	chr8	56067254	56074510	-	Human GRCh	OTTHUMG000001	RPS20	**ribosomal protein S20**	Cytoplasm	other		-1.15525236	0	0.49602867	0	1	0	12.9919928	13.3228327	13.1095839	12.9019762
231	**ENSG0000000930**	CSDE1	protein_coding	chr1	114716913	114758676	-	Human GRCh	OTTHUMG000000	CSDE1	**cold shock domain containing E1**	Cytoplasm	enzyme		-1.066898692	0	0.75272577	0	1	0	13.367459	13.6139964	13.5436787	13.293193
232	**ENSG0000000933**	UBE3C	protein_coding	chr7	157138926	157269370	+	Human GRCh	OTTHUMG000001	UBE3C	**ubiquitin protein ligase E3C**	Nucleus	enzyme		-1.099713084	0	0.61412379	0	1	0	12.4708104	12.7350974	12.6374534	12.3503076
233	**ENSG0000000941**	REV3L	protein_coding	chr6	111299028	111483715	-	Human GRCh	OTTHUMG000000	REV3L	**REV3 like, DNA directed polymerase zeta catalytic subunit**	Nucleus	enzyme		-1.132455312	0	0.53093618	0	1	0	11.9048813	12.1881454	12.0330568	11.7965757
234	**ENSG0000000969**	TENM1	protein_coding	chrX	124375903	125204312	-	Human GRCh	OTTHUMG000000	TENM1	**teneurin transmembrane protein 1**	Plasma Mem	transmembra		-1.0922015	0	0.88681594	0	1	0	6.86370722	6.92866777	6.80475897	6.80157863
235	**ENSG0000000970**	PAX7	protein_coding	chr1	18630846	18748866	+	Human GRCh	OTTHUMG000000	PAX7	**paired box 7**	Nucleus	transcription		1.714497985	1	0.76458835	0	NA	0	0.49718249	0.96517655	0.50696725	1.16459543
236	**ENSG0000000972**	MASP2	protein_coding	chr1	11022009	11047239	-	Human GRCh	OTTHUMG000000	MASP2	**MBL associated serine protease 2**	Extracellular	peptidase		1.587828578	1	0.35808518	0	1	0	4.35812428	3.72880793	4.38478861	4.58238579
237	**ENSG0000000976**	IYD	protein_coding	chr6	150368892	150405969	+	Human GRCh	OTTHUMG000000	IYD	**iodotyrosine deiodinase**	Plasma Mem	enzyme		-2.078015795	-1	0.59433605	0	NA	0	2.83281111	2.20082748	1.20045661	2.5355284
238	**ENSG0000000978**	FAM76A	protein_coding	chr1	27725961	27763116	+	Human GRCh	OTTHUMG000000	FAM76A	**family with sequence similarity 76 member A**	Nucleus	other		-1.027931568	0	0.89171476	0	1	0	8.71989882	9.13840198	9.06747261	8.74549891
240	**ENSG0000000983**	POMT2	protein_coding	chr14	77274956	77320883	-	Human GRCh	OTTHUMG000001	POMT2	**protein O-mannosyltransferase 2**	Cytoplasm	enzyme		-1.245593492	0	0.27993901	0	1	0	10.430726	10.5833436	10.4168231	10.1079992
241	**ENSG0000000984**	VTA1	protein_coding	chr6	142147162	142224685	+	Human GRCh	OTTHUMG000000	VTA1	**vesicle trafficking 1**	Cytoplasm	other		-1.087820034	0	0.70020299	0	1	0	10.3378147	10.7199865	10.6096379	10.2677545
242	**ENSG0000000995**	MLXIPL	protein_coding	chr7	73593194	73624543	-	Human GRCh	OTTHUMG000001	MLXIPL	**MLX interacting protein like**	Nucleus	transcription		1.043857749	0	0.88774074	0	1	0	9.22313404	9.87484419	9.64533	9.63757918
243	**ENSG0000000995**	BAZ1B	protein_coding	chr7	73440406	73522293	-	Human GRCh	OTTHUMG000000	BAZ1B	**bromodomain adjacent to zinc finger domain 1B**	Nucleus	transcription		-1.040744626	0	0.83478953	0	1	0	12.4302485	12.7256578	12.6378186	12.4406786
244	**ENSG0000001001**	RANBP9	protein_coding	chr6	13621498	13711835	-	Human GRCh	OTTHUMG000000	RANBP9	**RAN binding protein 9**	Nucleus	other		1.004885182	0	0.98139791	0	1	0	10.5626964	10.8651366	10.8129701	10.6383685
245	**ENSG0000001003**	ETV7	protein_coding	chr6	36354091	36387800	-	Human GRCh	OTTHUMG000000	ETV7	**ETS variant transcription factor 7**	Nucleus	transcription		2.648742546	1	0.11315446	0	1	0	2.79363033	3.47322521	3.81214159	3.82335716
246	**ENSG0000001007**	SPRTN	protein_coding	chr1	231337104	231355023	+	Human GRCh	OTTHUMG000000	SPRTN	**SprT-like N-terminal domain**	Nucleus	peptidase		1.033160009	0	0.87500392	0	1	0	9.85955622	10.1460891	10.1859131	9.89154768
247	**ENSG0000001016**	METTL13	protein_coding	chr1	171781660	171814023	+	Human GRCh	OTTHUMG000000	METTL13	**methyltransferase 13, eEF1A N-terminus and K55**	Cytoplasm	enzyme		1.064329386	0	0.74472965	0	1	0	10.0084393	10.2226075	10.4399901	9.87127239
248	**ENSG0000001021**	DYRK4	protein_coding	chr12	4562208	4615302	+	Human GRCh	OTTHUMG000001	DYRK4	**dual specificity tyrosine phosphorylation regulated kinase 4**	Nucleus	kinase		-1.135321942	0	0.68104845	0	1	0	7.49753164	8.0293055	7.87169408	7.38634728
249	**ENSG0000001024**	ZNF207	protein_coding	chr17	32350132	32381885	+	Human GRCh	OTTHUMG000001	ZNF207	**zinc finger protein 207**	Nucleus	transcription		-1.070697184	0	0.73405697	0	1	0	12.8987526	13.1673209	13.0879729	12.8282516
250	**ENSG0000001025**	UQCRC1	protein_coding	chr3	48599002	48610976	-	Human GRCh	OTTHUMG000001	UQCRC1	**ubiquinol-cytochrome c reductase core protein 1**	Cytoplasm	enzyme		-1.014501491	0	0.94928928	0	1	0	12.0356494	12.4585521	12.3862656	12.0949639
251	**ENSG0000001027**	STARD3NL	protein_coding	chr7	38178245	38230670	+	Human GRCh	OTTHUMG000000	STARD3NL	**STARD3 N-terminal like**	Cytoplasm	other		-1.241922368	0	0.30949441	0	1	0	8.73276714	8.84985627	8.59190323	8.53204964
252	**ENSG0000001027**	CD9	protein_coding	chr12	6199715	6238271	+	Human GRCh	OTTHUMG000000	CD9	**CD9 molecule**	Plasma Mem	other	KBA1412	1.043275713	0	0.89405193	0	1	0	9.73336242	9.96422157	9.88305922	9.91214149
253	**ENSG0000001028**	HHATL	protein_coding	chr3	42692663	42702824	-	Human GRCh	OTTHUMG000001	HHATL	**hedgehog acyltransferase like**	Cytoplasm	enzyme		-1.355965172	0	0.62518676	0	1	0	2.99140861	3.62570629	3.38371488	2.84136505
254	**ENSG0000001029**	NCAPD2	protein_coding	chr12	6493356	6531955	+	Human GRCh	OTTHUMG000001	NCAPD2	**non-SMC condensin I complex subunit D2**	Nucleus	other		-1.187649759	0	0.41800767	0	1	0	12.3907406	12.8035275	12.5166045	12.3219684
255	**ENSG0000001029**	IFFO1	protein_coding	chr12	6538961	6556073	-	Human GRCh	OTTHUMG000001	IFFO1	**intermediate filament family orphan 1**	Nucleus	other		1.436166399	0	0.54956754	0	1	0	5.14770939	5.12312579	5.393893	5.57646152
256	**ENSG0000001031**	GIPR	protein_coding	chr19	45668221	45683722	+	Human GRCh	OTTHUMG000001	GIPR	**gastric inhibitory polypeptide receptor**	Plasma Mem	G-protein cou	VK2735, LY	1.433556061	0	0.53502005	0	1	0	5.77622724	5.69315372	5.89630954	6.27755442
257	**ENSG0000001031**	PHF7	protein_coding	chr3	52410660	52423641	+	Human GRCh	OTTHUMG000001	PHF7	**PHD finger protein 7**	Nucleus	other		-1.261700795	0	0.32943481	0	1	0	8.91292125	9.04222589	8.85676816	8.57076894
258	**ENSG0000001031**	SEMA3G	protein_coding	chr3	52433035	52445103	-	Human GRCh	OTTHUMG000001	SEMA3G	**semaphorin 3G**	Cytoplasm	other		1.207876005	0	0.56765013	0	1	0	8.25228727	8.38650006	8.7024977	8.35214986
259	**ENSG0000001032**	NISCH	protein_coding	chr3	52455118	52493068	+	Human GRCh	OTTHUMG000001	NISCH	**nischarin**	Plasma Mem	other	moxonidin	-1.082003089	0	0.7105158	0	1	0	11.7898083	11.9709126	11.9499286	11.6212086
260	**ENSG0000001032**	STAB1	protein_coding	chr3	52495338	52524495	+	Human GRCh	OTTHUMG000001	STAB1	**stabilin 1**	Plasma Mem	transporter	FP-1305	2.301442802	1	0.02432991	1	1	0	5.06896623	5.17822192	5.66783185	6.08954447
261	**ENSG0000001036**	FUZ	protein_coding	chr19	49806866	49817376	-	Human GRCh	OTTHUMG000001	FUZ	**fuzzy planar cell polarity protein**	Plasma Mem	other		1.141031546	0	0.57075224	0	1	0	8.68901853	8.84407999	9.11938343	8.66357971
262	**ENSG0000001037**	SLC6A13	protein_coding	chr12	220621	262873	-	Human GRCh	OTTHUMG000001	SLC6A13	**solute carrier family 6 member 13**	Plasma Mem	transporter	tiagabine	-2.054954923	-1	0.47615403	0	NA	0	3.70285772	3.28051804	3.06523455	2.84384663
263	**ENSG0000001040**	IDS	protein_coding	chrX	149476988	149521096	-	Human GRCh	OTTHUMG000000	IDS	**iduronate 2-sulfatase**	Cytoplasm	enzyme		-1.026242737	0	0.90518674	0	1	0	10.0559828	10.1441279	10.1016739	10.0429343
264	**ENSG0000001043**	PRSS3	protein_coding	chr9	33750679	33799231	+	Human GRCh	OTTHUMG000000	PRSS3	**serine protease 3**	Extracellular	peptidase		-5.192149076	-1	0.31441182	0	NA	0	1.44283138	1.03291723	1.02980496	1.01530344
265	**ENSG0000001053**	ZNF200	protein_coding	chr16	3222325	3236221	-	Human GRCh	OTTHUMG000001	ZNF200	**zinc finger protein 200**	Nucleus	transcription		-1.077863373	0	0.76234686	0	1	0	8.70563985	9.03763474	8.9072343	8.68268122
266	**ENSG0000001061**	CD4	protein_coding	chr12	6786858	6820799	+	Human GRCh	OTTHUMG000001	CD4	**CD4 molecule**	Plasma Mem	transmembra	zanolimun	-1.904341183	-1	0.47705852	0	1	0	4.174784	4.12477153	3.83801496	3.15151086
267	**ENSG0000001062**	LRRC23	protein_coding	chr12	6873569	6914241	+	Human GRCh	OTTHUMG000001	LRRC23	**leucine rich repeat containing 23**	Plasma Mem	other		-1.059648265	0	0.84345093	0	1	0	7.54975542	7.98341614	7.95969601	7.42839671
270	**ENSG0000001080**	SCMH1	protein_coding	chr1	41027202	41242306	-	Human GRCh	OTTHUMG000000	SCMH1	**Scm polycomb group protein homolog 1**	Nucleus	transcription		-1.184672581	0	0.40827472	0	1	0	10.5675583	10.8167318	10.6615484	10.3428856
271	**ENSG0000001081**	FYN	protein_coding	chr6	111660332	111873452	-	Human GRCh	OTTHUMG000000	FYN	**FYN proto-oncogene, Src family tyrosine kinase**	Plasma Mem	kinase	PD162531,	-1.497556737	0	0.25716503	0	1	0	7.77327039	7.53942618	7.36028852	7.19899396
272	**ENSG0000001081**	HIVEP2	protein_coding	chr6	142751469	142956698	-	Human GRCh	OTTHUMG000000	HIVEP2	**HIVEP zinc finger 2**	Nucleus	transcription		-1.133192559	0	0.56166865	0	1	0	11.043813	11.3713406	11.1508185	11.0030496

274	**ENSG0000001100**	ELOA	protein_coding	chr1	23743448	23762059	+	Human GRCh	OTTHUMG000000	ELOA	**elongin A**	Nucleus	transcription		-1.054751592	0	0.78623392	0	1	0	11.2109922	11.5186845	11.4117544	11.2083095
275	**ENSG0000001100**	LYPLA2	protein_coding	chr1	23791145	23795539	+	Human GRCh	OTTHUMG000000	LYPLA2	**lysophospholipase 2**	Cytoplasm	enzyme		1.085977291	0	0.69411547	0	1	0	11.1868713	11.3588638	11.4855354	11.2436593
276	**ENSG0000001102**	CLCN6	protein_coding	chr1	11806096	11848079	+	Human GRCh	OTTHUMG000000	CLCN6	**chloride voltage-gated channel 6**	Plasma Mem	ion channel		-1.145359509	0	0.54841991	0	1	0	10.3704049	10.7822532	10.5323084	10.3502869
277	**ENSG0000001102**	MRC2	protein_coding	chr17	62627670	62693597	+	Human GRCh	OTTHUMG000001	MRC2	**mannose receptor C type 2**	Plasma Mem	transmembra		3.342306171	1	0.00211858	1	0.96	0	6.37438254	5.40759618	6.61782939	7.59250288
279	**ENSG0000001110**	TSPAN9	protein_coding	chr12	3077355	3286564	+	Human GRCh	OTTHUMG000001	TSPAN9	**tetraspanin 9**	Plasma Mem	other		1.287923273	0	0.53514994	0	1	0	6.00068004	6.20924714	6.20881534	6.49641751
280	**ENSG0000001111**	BTBD7	protein_coding	chr14	93237550	93333092	-	Human GRCh	OTTHUMG000001	BTBD7	**BTB domain containing 7**	Nucleus	other		-1.038196044	0	0.85815881	0	1	0	10.8382837	11.0803866	11.1372492	10.6657314
281	**ENSG0000001113**	APBA3	protein_coding	chr19	3750772	3761692	-	Human GRCh	OTTHUMG000001	APBA3	**amyloid beta precursor protein binding family A member 3**	Cytoplasm	transporter		-1.015834857	0	0.94450973	0	1	0	9.43292254	9.72344075	9.7221444	9.3947538
282	**ENSG0000001114**	MKS1	protein_coding	chr17	58205441	58219605	-	Human GRCh	OTTHUMG000001	MKS1	**MKS transition zone complex subunit 1**	Cytoplasm	other		-1.122513887	0	0.61914151	0	1	0	9.34261261	9.71670418	9.56058096	9.2529573
283	**ENSG0000001119**	ABHD5	protein_coding	chr3	43690108	43734371	+	Human GRCh	OTTHUMG000001	ABHD5	**abhydrolase domain containing 5, lysophosphatidic acid acyltr**	Cytoplasm	enzyme		1.053506024	0	0.81614779	0	1	0	9.32659468	9.76853999	9.79094524	9.43309599
284	**ENSG0000001120**	ANOS1	protein_coding	chrX	8528874	8732137	-	Human GRCh	OTTHUMG000000	ANOS1	**anosmin 1**	Extracellular	other		6.581468049	1	1.79E-07	1	0	1	7.78520737	7.90511544	9.50851636	10.1554783
285	**ENSG0000001124**	AKAP8L	protein_coding	chr19	15380050	15419141	-	Human GRCh	OTTHUMG000001	AKAP8L	**A-kinase anchoring protein 8 like**	Nucleus	other		1.076087281	0	0.73478286	0	1	0	11.535161	11.8216305	11.8548303	11.6746392
286	**ENSG0000001125**	MBTD1	protein_coding	chr17	51177425	51260163	-	Human GRCh	OTTHUMG000001	MBTD1	**mbt domain containing 1**	Nucleus	other		-1.21260688	0	0.3594217	0	1	0	10.6107764	10.8032001	10.7010456	10.2510789
287	**ENSG0000001126**	UTP18	protein_coding	chr17	51260546	51297936	+	Human GRCh	OTTHUMG000001	UTP18	**UTP18 small subunit processome component**	Nucleus	other		1.034927396	0	0.8634202	0	1	0	9.68118207	9.95843979	10.107939	9.56056918
288	**ENSG0000001127**	RNF216	protein_coding	chr7	5620047	5781696	-	Human GRCh	OTTHUMG000001	RNF216	**ring finger protein 216**	Cytoplasm	enzyme		-1.033569282	0	0.87762691	0	1	0	10.6413253	10.9219478	10.9361526	10.5374345
289	**ENSG0000001129**	TTC19	protein_coding	chr17	15999784	16045015	+	Human GRCh	OTTHUMG000000	TTC19	**tetratricopeptide repeat domain 19**	Cytoplasm	other		-1.049638668	0	0.80623786	0	1	0	10.7026273	11.0005418	10.9285481	10.6706292
290	**ENSG0000001130**	PTBP1	protein_coding	chr19	797075	812327	+	Human GRCh	OTTHUMG000001	PTBP1	**polypyrimidine tract binding protein 1**	Nucleus	enzyme		-1.085845158	0	0.68468917	0	1	0	13.5041281	13.8517235	13.7002637	13.4916743
291	**ENSG0000001133**	DPF1	protein_coding	chr19	38211006	38229714	-	Human GRCh	OTTHUMG000001	DPF1	**double PHD fingers 1**	Nucleus	other		1.283985829	0	0.4229274	0	1	0	6.68887286	6.96335389	7.28195741	6.85840673
292	**ENSG0000001134**	SYT7	protein_coding	chr11	61513714	61581148	-	Human GRCh	OTTHUMG000001	SYT7	**synaptotagmin 7**	Cytoplasm	transporter		1.009304625	0	0.96878066	0	1	0	9.16085537	9.40930591	9.25260968	9.34605875
293	**ENSG0000001137**	LARS2	protein_coding	chr3	45388561	45554726	+	Human GRCh	OTTHUMG000001	LARS2	**leucyl-tRNA synthetase 2, mitochondrial**	Cytoplasm	enzyme		-1.0841856	0	0.68860226	0	1	0	10.899636	11.2458024	11.1255598	10.8471088
294	**ENSG0000001140**	PIK3C2A	protein_coding	chr11	17077730	17207986	-	Human GRCh	OTTHUMG000001	PIK3C2A	**phosphatidylinositol-4-phosphate 3-kinase catalytic subunit ty**	Cytoplasm	kinase		-1.018590151	0	0.93530648	0	1	0	11.7150182	12.0009761	11.9733282	11.7038511
295	**ENSG0000001142**	PLAUR	protein_coding	chr19	43646095	43670547	-	Human GRCh	OTTHUMG000001	PLAUR	**plasminogen activator, urokinase receptor**	Plasma Mem	transmembra		-1.422028247	0	0.25830665	0	1	0	6.43389836	6.21809116	6.24703929	5.66174449
296	**ENSG0000001142**	ANLN	protein_coding	chr7	36389821	36453791	+	Human GRCh	OTTHUMG000000	ANLN	**anillin, actin binding protein**	Cytoplasm	other		-1.156055757	0	0.50418804	0	1	0	11.4368979	11.8283431	11.6172574	11.3489449
297	**ENSG0000001145**	WIZ	protein_coding	chr19	15419978	15449956	-	Human GRCh	OTTHUMG000001	WIZ	**WIZ zinc finger**	Nucleus	transcription		-1.040213771	0	0.84028145	0	1	0	11.6545824	12.0119402	11.8118129	11.792812
298	**ENSG0000001145**	RABGAP1	protein_coding	chr9	122940833	123104866	+	Human GRCh	OTTHUMG000000	RABGAP1	**RAB GTPase activating protein 1**	Cytoplasm	other		-1.160257789	0	0.44297075	0	1	0	11.1560284	11.4241148	11.2499051	11.0108453
299	**ENSG0000001146**	DCN	protein_coding	chr12	91140484	91183217	-	Human GRCh	OTTHUMG000001	DCN	**decorin**	Extracellular	other		10.79841993	1	0.61289175	0	1	0	0.24395188	0.3073946	1.2184888	1.11148854
300	**ENSG0000001147**	QPCTL	protein_coding	chr19	45692403	45703989	+	Human GRCh	OTTHUMG000001	QPCTL	**glutaminyl-peptide cyclotransferase like**	Cytoplasm	enzyme		-1.113926554	0	0.6829291	0	1	0	9.38745505	9.75803092	9.62718482	9.27222288
301	**ENSG0000001148**	PPP5C	protein_coding	chr19	46347087	46392981	+	Human GRCh	OTTHUMG000001	PPP5C	**protein phosphatase 5 catalytic subunit**	Nucleus	phosphatase		-1.13722519	0	0.50722336	0	1	0	11.7424992	12.0652861	11.8729253	11.658133
302	**ENSG0000001152**	CEP68	protein_coding	chr2	65056354	65087004	+	Human GRCh	OTTHUMG000001	CEP68	**centrosomal protein 68**	Cytoplasm	other		1.011480192	0	0.95705889	0	1	0	9.61213935	9.80304237	9.7552639	9.68999284
303	**ENSG0000001156**	MAP4K3	protein_coding	chr2	39249266	39437301	-	Human GRCh	OTTHUMG000001	MAP4K3	**mitogen-activated protein kinase kinase kinase kinase 3**	Plasma Mem	kinase		1.106837796	0	0.63194834	0	1	0	10.1383608	10.5387025	10.6833234	10.2000204
304	**ENSG0000001159**	ZBTB32	protein_coding	chr19	35704558	35717038	+	Human GRCh	OTTHUMG000000	ZBTB32	**zinc finger and BTB domain containing 32**	Nucleus	transcription		10.78000958	1	0.13298986	0	NA	0	1.12740563	1.13650396	1.48564817	1.43937259
306	**ENSG0000001163**	LDAF1	protein_coding	chr16	21158377	21180616	+	Human GRCh	OTTHUMG000001	LDAF1	**lipid droplet assembly factor 1**	Other	other		1.078952891	0	0.81788774	0	1	0	6.15659299	6.39000127	6.40228626	6.29844725
307	**ENSG0000001167**	GABRA3	protein_coding	chrX	152166234	152451315	-	Human GRCh	OTTHUMG000000	GABRA3	**gamma-aminobutyric acid type A receptor subunit alpha3**	Plasma Mem	ion channel	flunitrazep	-1.270855581	0	0.3561579	0	1	0	7.99895626	8.30805875	7.88295465	7.94073268
308	**ENSG0000001204**	BRCA1	protein_coding	chr17	43044295	43170245	-	Human GRCh	OTTHUMG000001	BRCA1	**BRCA1 DNA repair associated**	Nucleus	transcription		-1.147380317	0	0.49681052	0	1	0	11.5785417	11.7607121	11.6668159	11.3555098
309	**ENSG0000001206**	ERCC1	protein_coding	chr19	45407334	45478828	-	Human GRCh	OTTHUMG000001	ERCC1	**ERCC excision repair 1, endonuclease non-catalytic subunit**	Nucleus	enzyme		-1.004558372	0	0.98541324	0	1	0	10.1852802	10.5121756	10.5124944	10.1729713
311	**ENSG0000001217**	SEMA3B	protein_coding	chr3	50267558	50277546	+	Human GRCh	OTTHUMG000001	SEMA3B	**semaphorin 3B**	Extracellular	other		-1.129568737	0	0.6818337	0	1	0	8.59164647	8.86154671	8.64885529	8.55436402
312	**ENSG0000001217**	MBTPS2	protein_coding	chrX	21839617	21885423	+	Human GRCh	OTTHUMG000000	MBTPS2	**membrane bound transcription factor peptidase, site 2**	Cytoplasm	peptidase		-1.241283414	0	0.36336413	0	1	0	9.8975885	10.1958514	9.97832029	9.63022673
313	**ENSG0000001221**	PRICKLE3	protein_coding	chrX	49174802	49186528	-	Human GRCh	OTTHUMG000000	PRICKLE3	**prickle planar cell polarity protein 3**	Cytoplasm	other		1.024550082	0	0.91231517	0	1	0	8.47333005	8.95418894	8.73282571	8.79437875
314	**ENSG0000001222**	LTF	protein_coding	chr3	46435645	46485234	-	Human GRCh	OTTHUMG000001	LTF	**lactotransferrin**	Extracellular	other		5.411634092	1	0.18270981	0	1	0	1.60136698	1.70358467	2.39451382	1.60760201
315	**ENSG0000001223**	EXTL3	protein_coding	chr8	28600469	28756561	+	Human GRCh	OTTHUMG000001	EXTL3	**exostosin like glycosyltransferase 3**	Cytoplasm	enzyme		-1.132322224	0	0.56899749	0	1	0	10.2457658	10.1471043	10.2145268	9.88606287
317	**ENSG0000001266**	ELOVL5	protein_coding	chr6	53267398	53349179	-	Human GRCh	OTTHUMG000000	ELOVL5	**ELOVL fatty acid elongase 5**	Cytoplasm	enzyme		-1.044804721	0	0.83736042	0	1	0	11.8196398	12.1333508	12.0573002	11.8056553
318	**ENSG0000001277**	ALOX5	protein_coding	chr10	45374176	45446119	+	Human GRCh	OTTHUMG000000	ALOX5	**arachidonate 5-lipoxygenase**	Cytoplasm	enzyme	diclofenac,	1.257024296	0	0.79857817	0	1	0	3.38338131	3.84628272	3.49485252	3.94386598
319	**ENSG0000001281**	KDM5D	protein_coding	chrY	19703865	19744939	-	Human GRCh	OTTHUMG000000	KDM5D	**lysine demethylase 5D**	Nucleus	enzyme		-1.111081983	0	0.66608817	0	1	0	9.96558009	10.148723	10.1179635	9.71527285
320	**ENSG0000001282**	CALCOCO1	protein_coding	chr12	53708517	53727745	-	Human GRCh	OTTHUMG000001	CALCOCO1	**calcium binding and coiled-coil domain 1**	Nucleus	transcription		-1.296792106	0	0.25443299	0	1	0	10.9338883	11.2239576	10.8684698	10.728508
321	**ENSG0000001296**	UBR7	protein_coding	chr14	93207241	93229215	+	Human GRCh	OTTHUMG000001	UBR7	**ubiquitin protein ligase E3 component n-recognin 7**	Other	enzyme		-1.129811266	0	0.56416615	0	1	0	10.8850214	11.2340974	11.0509216	10.8151429
322	**ENSG0000001298**	MAP4K5	protein_coding	chr14	50418501	50561126	-	Human GRCh	OTTHUMG000001	MAP4K5	**mitogen-activated protein kinase kinase kinase kinase 5**	Cytoplasm	kinase	binimetinil	1.101360506	0	0.62545744	0	1	0	10.9384191	11.1642414	11.3420199	10.9493872
323	**ENSG0000001301**	EHD3	protein_coding	chr2	31234152	31269451	+	Human GRCh	OTTHUMG000000	EHD3	**EH domain containing 3**	Cytoplasm	other		1.107650252	0	0.75828589	0	1	0	6.79833392	7.51695509	7.44008771	7.18571706
324	**ENSG0000001327**	PSMC4	protein_coding	chr19	39971165	39981764	+	Human GRCh	OTTHUMG000001	PSMC4	**proteasome 26S subunit, ATPase 4**	Nucleus	peptidase		-1.123017943	0	0.59763591	0	1	0	11.1040325	11.5322054	11.2655881	11.1429733
325	**ENSG0000001328**	MAN2B2	protein_coding	chr4	6575189	6623362	+	Human GRCh	OTTHUMG000001	MAN2B2	**mannosidase alpha class 2B member 2**	Cytoplasm	enzyme		-1.179172586	0	0.46964312	0	1	0	8.93483795	9.13706738	8.91958229	8.80118249
326	**ENSG0000001329**	SLC7A14	protein_coding	chr3	170459548	170586075	-	Human GRCh	OTTHUMG000001	SLC7A14	**solute carrier family 7 member 14**	Cytoplasm	ion channel		-5.05217155	-1	0.49619963	0	1	0	2.32205617	1.7400589	1.71926062	1.61705958
327	**ENSG0000001329**	CLDN11	protein_coding	chr3	170418868	170454733	+	Human GRCh	OTTHUMG000001	CLDN11	**claudin 11**	Plasma Mem	other		-1.358935777	0	0.57814373	0	1	0	3.92099712	3.14753901	3.48932971	3.36367092
328	**ENSG0000001330**	SLC25A39	protein_coding	chr17	44319625	44324870	-	Human GRCh	OTTHUMG000001	SLC25A39	**solute carrier family 25 member 39**	Cytoplasm	other		-1.000199955	0	0.99921099	0	1	0	12.9642213	13.2499121	13.1991442	13.0252871
329	**ENSG0000001336**	MVP	protein_coding	chr16	29820394	29848039	+	Human GRCh	OTTHUMG000000	MVP	**major vault protein**	Nucleus	other		1.154136355	0	0.70295063	0	1	0	8.02645152	8.22512359	7.95513533	8.5284731
330	**ENSG0000001337**	NUB1	protein_coding	chr7	151341772	151378449	+	Human GRCh	OTTHUMG000001	NUB1	**negative regulator of ubiquitin like proteins 1**	Nucleus	other		-1.275500765	0	0.23808512	0	1	0	10.3834022	10.6247363	10.3673598	10.0958226
331	**ENSG0000001337**	PGM3	protein_coding	chr6	83147324	83193936	-	Human GRCh	OTTHUMG000000	PGM3	**phosphoglucomutase 3**	Cytoplasm	enzyme		-1.115358722	0	0.63626277	0	1	0	11.3538084	11.5573771	11.5113885	11.134311
332	**ENSG0000001339**	RWDD2A	protein_coding	chr6	83193357	83198935	+	Human GRCh	OTTHUMG000000	RWDD2A	**RWD domain containing 2A**	Other	other		-1.003126637	0	0.99148414	0	1	0	8.10157478	8.03146731	8.2656736	7.82102541
333	**ENSG0000001344**	CLK1	protein_coding	chr2	200853009	200864691	-	Human GRCh	OTTHUMG000001	CLK1	**CDC like kinase 1**	Nucleus	kinase	KH-CB19	-1.002359676	0	0.9914459	0	1	0	11.3853518	11.5710982	11.6941193	11.215992
334	**ENSG0000001350**	POLR3B	protein_coding	chr12	106357748	106510198	+	Human GRCh	OTTHUMG000001	POLR3B	**RNA polymerase III subunit B**	Nucleus	enzyme		-1.152322675	0	0.49348539	0	1	0	9.49373903	9.75292153	9.58203154	9.35568658
335	**ENSG0000001352**	ANGEL1	protein_coding	chr14	76786009	76826246	-	Human GRCh	OTTHUMG000001	ANGEL1	**angel homolog 1**	Extracellular	other		-1.08653987	0	0.66047342	0	1	0	10.0870846	10.1347713	10.0924866	9.9474294
336	**ENSG0000001356**	RNF14	protein_coding	chr5	141958328	141990292	+	Human GRCh	OTTHUMG000001	RNF14	**ring finger protein 14**	Cytoplasm	transcription		-1.283277697	0	0.21711183	0	1	0	10.3324464	10.6287559	10.3019016	10.1120815
337	**ENSG0000001356**	DNASE1L1	protein_coding	chrX	154401236	154412112	-	Human GRCh	OTTHUMG000000	DNASE1L1	**deoxyribonuclease 1 like 1**	Cytoplasm	enzyme		-1.288416197	0	0.29214755	0	1	0	10.0359993	10.2528722	10.1177925	9.5521802
338	**ENSG0000001357**	DDX11	protein_coding	chr12	31073860	31104799	+	Human GRCh	OTTHUMG000001	DDX11	**DEAD/H-box helicase 11**	Cytoplasm	enzyme		-1.145943458	0	0.53429968	0	1	0	12.914586	13.1962635	13.0999176	12.6871968
339	**ENSG0000001358**	HEBP1	protein_coding	chr12	12974870	13000265	-	Human GRCh	OTTHUMG000001	HEBP1	**heme binding protein 1**	Cytoplasm	other		1.0122619	0	0.96072576	0	1	0	8.1215456	8.19959431	8.38839596	7.91327486
340	**ENSG0000001358**	GPRC5A	protein_coding	chr12	12891559	12917937	+	Human GRCh	OTTHUMG000001	GPRC5A	**G protein-coupled receptor class C group 5 member A**	Plasma Mem	G-protein cou		1.077890431	0	0.89341711	0	1	0	7.27482151	7.56359664	7.48578205	7.53383401
341	**ENSG0000001361**	MAMLD1	protein_coding	chrX	150361422	150514178	+	Human GRCh	OTTHUMG000000	MAMLD1	**mastermind like domain containing 1**	Nucleus	other		1.180422122	0	0.63596256	0	1	0	5.80673703	5.77608428	5.48115368	6.27087527
342	**ENSG0000001372**	CD6	protein_coding	chr11	60971680	61020377	+	Human GRCh	OTTHUMG000001	CD6	**CD6 molecule**	Plasma Mem	transmembra		1.171849273	0	0.7098121	0	1	0	5.91867286	6.08900812	6.12808272	6.20942879
343	**ENSG0000001381**	TACC3	protein_coding	chr4	1712858	1745171	+	Human GRCh	OTTHUMG000000	TACC3	**transforming acidic coiled-coil containing protein 3**	Nucleus	other		-1.103779312	0	0.6148697	0	1	0	12.1027749	12.3960352	12.2633964	12.0214026
344	**ENSG0000001412**	UFL1	protein_coding	chr6	96521595	96555276	+	Human GRCh	OTTHUMG000000	UFL1	**UFM1 specific ligase 1**	Cytoplasm	enzyme		-1.051178042	0	0.82581673	0	1	0	10.2441499	10.5284963	10.4234263	10.2491235
345	**ENSG0000001413**	POLA2	protein_coding	chr11	65261913	65305959	+	Human GRCh	OTTHUMG000001	POLA2	**DNA polymerase alpha 2, accessory subunit**	Nucleus	enzyme		-1.112507219	0	0.6050422	0	1	0	11.3405828	11.6240298	11.5385664	11.1829303
346	**ENSG0000001416**	ZC3H3	protein_coding	chr8	143437659	143541447	-	Human GRCh	OTTHUMG000001	ZC3H3	**zinc finger CCCH-type containing 3**	Nucleus	other		1.030589549	0	0.88388713	0	1	0	10.3157134	10.6668476	10.6130668	10.4562415
347	**ENSG0000001421**	CAPN1	protein_coding	chr11	65180566	65212006	+	Human GRCh	OTTHUMG000001	CAPN1	**calpain 1**	Cytoplasm	peptidase	alicapistat,	-1.017472505	0	0.93183783	0	1	0	10.4026839	10.6787027	10.6344685	10.415174
348	**ENSG0000001425**	ACP3	protein_coding	chr3	132317369	132368302	+	Human GRCh	OTTHUMG000001	ACP3	**acid phosphatase 3**	Extracellular	phosphatase		-1.75903469	-1	0.40446574	0	1	0	3.67453515	3.94580544	3.52083422	3.33192196
349	**ENSG0000001464**	MDH1	protein_coding	chr2	63588609	63607197	+	Human GRCh	OTTHUMG000001	MDH1	**malate dehydrogenase 1**	Cytoplasm	enzyme		-1.066163095	0	0.79056995	0	1	0	11.4557064	11.8332162	11.6934827	11.470297
350	**ENSG0000001482**	SLC30A9	protein_coding	chr4	41990502	42090461	+	Human GRCh	OTTHUMG000000	SLC30A9	**solute carrier family 30 member 9**	Nucleus	transporter		-1.041627426	0	0.84651312	0	1	0	11.4103165	11.7638059	11.7142245	11.3701996
351	**ENSG0000001491**	MTMR11	protein_coding	chr1	149928651	149936879	-	Human GRCh	OTTHUMG000000	MTMR11	**myotubularin related protein 11**	Cytoplasm	other		-1.103917741	0	0.74520493	0	1	0	8.20968589	8.08788795	8.09508255	7.99267465
352	**ENSG0000001491**	COX15	protein_coding	chr10	99710868	99732127	-	Human GRCh	OTTHUMG000000	COX15	**cytochrome c oxidase assembly homolog COX15**	Cytoplasm	enzyme		-1.065799683	0	0.76483956	0	1	0	10.4824213	10.6834387	10.6699299	10.3382652
353	**ENSG0000001513**	CCDC88C	protein_coding	chr14	91271323	91417844	-	Human GRCh	OTTHUMG000001	CCDC88C	**coiled-coil domain containing 88C**	Cytoplasm	other		1.001275554	0	0.99536207	0	1	0	10.7371413	11.0920878	11.0370236	10.8108534
354	**ENSG0000001515**	YAF2	protein_coding	chr12	42157104	42238349	-	Human GRCh	OTTHUMG000001	YAF2	**YY1 associated factor 2**	Nucleus	transcription		-1.202688058	0	0.43283611	0	1	0	9.75592923	10.0438823	9.7774266	9.63047123
355	**ENSG0000001517**	ZMYND11	protein_coding	chr10	130088	254637	+	Human GRCh	OTTHUMG000000	ZMYND11	**zinc finger MYND-type containing 11**	Nucleus	transcription		-1.08377738	0	0.68487569	0	1	0	10.8718165	11.0508595	11.047206	10.6773275
356	**ENSG0000001528**	WAS	protein_coding	chrX	48676596	48691431	+	Human GRCh	OTTHUMG000000	WAS	**WASP actin nucleation promoting factor**	Cytoplasm	other		-1.33767072	0	0.57432932	0	1	0	5.20385733	5.17236021	4.82000739	5.01449444
357	**ENSG0000001541**	DPEP1	protein_coding	chr16	89613308	89638456	+	Human GRCh	OTTHUMG000001	DPEP1	**dipeptidase 1**	Cytoplasm	peptidase	cilastatin	1.887966567	1	0.71916004	0	1	0	0.50538094	1.93423364	0.5652768	2.46049503
358	**ENSG0000001547**	BID	protein_coding	chr22	17734138	17774770	-	Human GRCh	OTTHUMG000001	BID	**BH3 interacting domain death agonist**	Cytoplasm	other		-1.348418183	0	0.4309318	0	1	0	6.34721979	6.89944181	6.51483775	6.1607236
359	**ENSG0000001547**	MATR3	protein_coding	chr5	139293674	139331677	+	Human GRCh	OTTHUMG000001	MATR3	**matrin 3**	Nucleus	other		-1.008332766	0	0.96738654	0	1	0	12.9847619	13.1426997	13.2678134	12.8003958
360	**ENSG0000001552**	NPC1L1	protein_coding	chr7	44512535	44541330	-	Human GRCh	OTTHUMG000000	NPC1L1	**NPC1 like intracellular cholesterol transporter 1**	Cytoplasm	transporter	ezetimibe/	1.088032493	0	0.83154964	0	1	0	4.98793002	4.87461701	5.05538123	4.95595944

361	**ENSG0000001553**	XYLT2	protein_coding	chr17	50346126	50363138	+	Human GRCh	OTTHUMG000001	XYLT2	**xylosyltransferase 2**	Cytoplasm	enzyme		1.096396983	0	0.69451556	0	1	0	9.24404094	9.39223841	9.56976694	9.25195377
362	**ENSG0000001556**	RGPD5	protein_coding	chr2	109792758	109857705	-	Human GRCh	OTTHUMG000001	RGPD4 (inclu	**RANBP2 like and GRIP domain containing 3**	Nucleus	enzyme		1.259731345	0	0.68565397	0	1	0	4.33037361	4.75034021	4.68954769	4.8235314
364	**ENSG0000001567**	NUDCD3	protein_coding	chr7	44379119	44490658	-	Human GRCh	OTTHUMG000001	NUDCD3	**NudC domain containing 3**	Cytoplasm	other		-1.243808787	0	0.30306746	0	1	0	11.3647981	11.5441789	11.4522011	10.9205784
365	**ENSG0000001608**	ISL1	protein_coding	chr5	51383448	51394730	+	Human GRCh	OTTHUMG000001	ISL1	**ISL LIM homeobox 1**	Nucleus	transcription		-1.398274961	0	0.48959463	0	1	0	5.63760939	6.1021364	5.63540177	5.41697628
366	**ENSG0000001639**	CHDH	protein_coding	chr3	53812335	53846419	-	Human GRCh	OTTHUMG000001	CHDH	**choline dehydrogenase**	Cytoplasm	enzyme		-1.052939828	0	0.86091051	0	1	0	11.5171348	11.8381486	11.8254639	11.3958969
367	**ENSG0000001640**	IL20RA	protein_coding	chr6	136999971	137045180	-	Human GRCh	OTTHUMG000000	IL20RA	**interleukin 20 receptor subunit alpha**	Plasma Mem	transmembra		-1.333938819	0	0.51455833	0	1	0	8.06226543	8.10527986	7.95283329	7.49824975
370	**ENSG0000001686**	GLT8D1	protein_coding	chr3	52694486	52706032	-	Human GRCh	OTTHUMG000001	GLT8D1	**glycosyltransferase 8 domain containing 1**	Cytoplasm	enzyme		1.005459386	0	0.97882621	0	1	0	9.52363692	9.63403104	9.7875122	9.34337259
371	**ENSG0000001726**	ATP2C1	protein_coding	chr3	130850595	131016712	+	Human GRCh	OTTHUMG000001	ATP2C1	**ATPase secretory pathway Ca2+ transporting 1**	Cytoplasm	transporter	sevofluran	-1.128186537	0	0.57437512	0	1	0	11.1164333	11.3236641	11.3221677	10.8251234
372	**ENSG0000001742**	IGF1	protein_coding	chr12	102395874	102481744	-	Human GRCh	OTTHUMG000001	IGF1	**insulin like growth factor 1**	Extracellular	growth facto	MEDI-573,	1.162511426	0	0.81795378	0	1	0	6.95565966	7.17690563	7.45660655	6.9767132
373	**ENSG0000001748**	SLC38A5	protein_coding	chrX	48458537	48470260	-	Human GRCh	OTTHUMG000000	SLC38A5	**solute carrier family 38 member 5**	Plasma Mem	transporter		1.51401579	1	0.57503904	0	1	0	3.25178259	3.46093725	3.61208354	3.58022491
374	**ENSG0000001779**	RALBP1	protein_coding	chr18	9475009	9538114	+	Human GRCh	OTTHUMG000001	RALBP1	**ralA binding protein 1**	Cytoplasm	enzyme		-1.163518088	0	0.44507613	0	1	0	10.93995	11.184432	10.9986558	10.7914363
375	**ENSG0000001818**	RUFY3	protein_coding	chr4	70704204	70808619	+	Human GRCh	OTTHUMG000001	RUFY3	**RUN and FYVE domain containing 3**	Plasma Mem	other		1.027211665	0	0.90219627	0	1	0	10.4727795	10.6575495	10.8805895	10.2268633
376	**ENSG0000001823**	CNTN1	protein_coding	chr12	40692439	41072415	+	Human GRCh	OTTHUMG000001	CNTN1	**contactin 1**	Plasma Mem	enzyme		-1.555252604	-1	0.53829883	0	1	0	2.89423836	4.43645228	4.00323901	2.90183837
377	**ENSG0000001828**	SLC11A1	protein_coding	chr2	218382029	218396894	+	Human GRCh	OTTHUMG000000	SLC11A1	**solute carrier family 11 member 1**	Plasma Mem	transporter		1.074645321	0	0.90438641	0	1	0	4.66933588	4.89017015	4.42373141	5.15777428
378	**ENSG0000001840**	WWTR1	protein_coding	chr3	149517235	149736714	-	Human GRCh	OTTHUMG000001	WWTR1	**WW domain containing transcription regulator 1**	Nucleus	transcription		1.651173253	1	0.0492089	1	1	0	9.73947935	9.92591137	10.7846073	9.89524364
379	**ENSG0000001851**	AGPS	protein_coding	chr2	177392746	177567024	+	Human GRCh	OTTHUMG000001	AGPS	**alkylglycerone phosphate synthase**	Cytoplasm	enzyme		1.364883796	0	0.25682605	0	1	0	8.59685573	8.80070398	9.08587784	8.94013905
380	**ENSG0000001861**	STEEP1	protein_coding	chrX	119538149	119565409	-	Human GRCh	OTTHUMG000000	STEEP1	**STING1 ER exit protein 1**	Extracellular	other		-1.115779674	0	0.62098173	0	1	0	9.06456346	9.4373448	9.18447882	9.10532557
381	**ENSG0000001862**	ATP1A2	protein_coding	chr1	160115759	160143591	+	Human GRCh	OTTHUMG000000	ATP1A2	**ATPase Na+/K+ transporting subunit alpha 2**	Plasma Mem	transporter	digoxin, et	-1.452481716	0	0.60072296	0	1	0	12.7624324	13.0563915	12.9498149	11.62157
382	**ENSG0000001869**	TTC27	protein_coding	chr2	32628032	32821051	+	Human GRCh	OTTHUMG000001	TTC27	**tetratricopeptide repeat domain 27**	Other	other		1.028154013	0	0.90680928	0	1	0	9.25805632	9.5780759	9.65520035	9.22537153
383	**ENSG0000001886**	ZNF582	protein_coding	chr19	56375846	56393538	-	Human GRCh	OTTHUMG000001	ZNF582	**zinc finger protein 582**	Other	other		1.360338615	0	0.59065214	0	1	0	5.1346274	5.38185827	5.93793059	5.044177
384	**ENSG0000001910**	VSIG2	protein_coding	chr11	124747474	124752255	-	Human GRCh	OTTHUMG000001	VSIG2	**V-set and immunoglobulin domain containing 2**	Plasma Mem	other		4.180713019	1	0.11381787	0	1	0	2.75893519	1.80323535	3.61195689	2.86549201
385	**ENSG0000001914**	PHLDB1	protein_coding	chr11	118606440	118658031	+	Human GRCh	OTTHUMG000001	PHLDB1	**pleckstrin homology like domain family B member 1**	Cytoplasm	other		-1.174820157	0	0.44427094	0	1	0	10.4209896	10.5876915	10.4399494	10.231421
388	**ENSG0000001948**	PRDM11	protein_coding	chr11	45095806	45235110	+	Human GRCh	OTTHUMG000001	PRDM11	**PR/SET domain 11**	Other	other		-2.174252392	-1	0.00104607	1	0.63	0	7.73843663	8.04777415	7.37708023	6.84723124
389	**ENSG0000001950**	SYT13	protein_coding	chr11	45240302	45286341	-	Human GRCh	OTTHUMG000001	SYT13	**synaptotagmin 13**	Plasma Mem	transporter		1.932135432	1	0.46114158	0	1	0	3.72695844	3.8504323	3.83051788	4.23339005
390	**ENSG0000001954**	SNAI2	protein_coding	chr8	48917598	48921740	-	Human GRCh	OTTHUMG000001	SNAI2	**snail family transcriptional repressor 2**	Nucleus	transcription		1.005689896	0	0.99612888	0	1	0	4.51404661	4.00343568	3.7449733	4.66395802
391	**ENSG0000001958**	CD74	protein_coding	chr5	150401637	150412969	-	Human GRCh	OTTHUMG000001	CD74	**CD74 molecule**	Plasma Mem	transmembra	milatuzum	-1.962208094	-1	0.18899261	0	1	0	3.83410369	3.60842948	3.80598684	2.23966176
393	**ENSG0000001999**	ZRANB1	protein_coding	chr10	124942123	124988189	+	Human GRCh	OTTHUMG000000	ZRANB1	**zinc finger RANBP2-type containing 1**	Nucleus	peptidase		-1.103110141	0	0.63053147	0	1	0	10.5612072	10.858178	10.7257572	10.482248
394	**ENSG0000002012**	NCDN	protein_coding	chr1	35557473	35567274	+	Human GRCh	OTTHUMG000000	NCDN	**neurochondrin**	Cytoplasm	other		1.114710089	0	0.54196728	0	1	0	9.9726087	10.0760644	10.2174259	10.0711938
395	**ENSG0000002018**	ADGRA2	protein_coding	chr8	37784191	37844896	+	Human GRCh	OTTHUMG000001	ADGRA2	**adhesion G protein-coupled receptor A2**	Plasma Mem	G-protein cou		5.489590522	1	0.11303309	0	1	0	2.17457158	2.6435556	2.62973715	3.36581894
396	**ENSG0000002025**	ZFP64	protein_coding	chr20	52051663	52204308	-	Human GRCh	OTTHUMG000000	ZFP64	**ZFP64 zinc finger protein**	Nucleus	transcription		-1.22376464	0	0.3413378	0	1	0	9.41566557	9.6646908	9.41843308	9.22536614
397	**ENSG0000002042**	MNAT1	protein_coding	chr14	60734742	60969965	+	Human GRCh	OTTHUMG000001	MNAT1	**MNAT1 component of CDK activating kinase**	Nucleus	other		-1.220017988	0	0.36371304	0	1	0	9.38478347	9.81323827	9.50708069	9.29699119
398	**ENSG0000002057**	SAMD4A	protein_coding	chr14	54566117	54793315	+	Human GRCh	OTTHUMG000001	SAMD4A	**sterile alpha motif domain containing 4A**	Cytoplasm	translation re		-1.418419828	0	0.44452277	0	1	0	5.4360764	5.18066699	4.79034978	5.12933983
399	**ENSG0000002063**	RUNX3	protein_coding	chr1	24899511	24965121	-	Human GRCh	OTTHUMG000000	RUNX3	**RUNX family transcription factor 3**	Nucleus	transcription		-5.312741119	-1	0.22974923	0	1	0	1.68736265	1.04021651	1.0286112	0.97231665
400	**ENSG0000002092**	MRE11	protein_coding	chr11	94415570	94493885	-	Human GRCh	OTTHUMG000001	MRE11	**MRE11 homolog, double strand break repair nuclease**	Nucleus	enzyme		-1.127621437	0	0.57424428	0	1	0	10.715079	11.0213039	10.8827354	10.5929241
401	**ENSG0000002130**	PLEKHB1	protein_coding	chr11	73646178	73662819	+	Human GRCh	OTTHUMG000001	PLEKHB1	**pleckstrin homology domain containing B1**	Cytoplasm	other		1.017832509	0	0.95318706	0	1	0	8.40415312	8.38282694	8.62136762	8.15625398
402	**ENSG0000002135**	SERPINB1	protein_coding	chr6	2832332	2841959	-	Human GRCh	OTTHUMG000000	SERPINB1	**serpin family B member 1**	Cytoplasm	other		-1.091219069	0	0.75851428	0	1	0	7.29849245	7.40647047	7.29347598	7.23417257
404	**ENSG0000002148**	SLC7A9	protein_coding	chr19	32830509	32869767	-	Human GRCh	OTTHUMG000001	SLC7A9	**solute carrier family 7 member 9**	Plasma Mem	transporter		1.166396211	0	0.7207125	0	1	0	4.80937532	4.75280803	5.08390497	4.72603426
405	**ENSG0000002157**	SPAST	protein_coding	chr2	32063556	32157637	+	Human GRCh	OTTHUMG000001	SPAST	**spastin**	Nucleus	enzyme		1.022543352	0	0.91871831	0	1	0	10.0634837	10.1996281	10.3813994	9.88225881
406	**ENSG0000002164**	NRXN3	protein_coding	chr14	78170373	79868291	+	Human GRCh	OTTHUMG000001	NRXN3	**neurexin 3**	Plasma Mem	transmembra		1.296273102	0	0.37876576	0	1	0	9.00860373	9.5496783	9.5101607	9.6315448
407	**ENSG0000002176**	OSBPL5	protein_coding	chr11	3087107	3166739	-	Human GRCh	OTTHUMG000000	OSBPL5	**oxysterol binding protein like 5**	Cytoplasm	transporter		-1.092321406	0	0.73450578	0	1	0	8.68598746	8.82356133	8.79476458	8.51963149
408	**ENSG0000002177**	AQR	protein_coding	chr15	34851782	34969742	-	Human GRCh	OTTHUMG000001	AQR	**aquarius intron-binding spliceosomal factor**	Nucleus	enzyme		-1.088356691	0	0.71451764	0	1	0	11.4997665	11.8235601	11.7698571	11.3490509
409	**ENSG0000002182**	CPS1	protein_coding	chr2	210477682	210679107	+	Human GRCh	OTTHUMG000001	CPS1	**carbamoyl-phosphate synthase 1**	Cytoplasm	enzyme	N-carbamy	1.888120655	1	0.42289575	0	NA	0	3.33866818	3.53486807	4.06082686	3.7893672
411	**ENSG0000002226**	FHL1	protein_coding	chrX	136146702	136211359	+	Human GRCh	OTTHUMG000000	FHL1	**four and a half LIM domains 1**	Cytoplasm	other		-1.801709576	-1	0.3283095	0	1	0	5.29332518	5.00133355	4.641035	4.57482416
412	**ENSG0000002227**	RTF2	protein_coding	chr20	56468585	56519449	+	Human GRCh	OTTHUMG000000	RTF2	**replication termination factor 2**	Nucleus	other		1.015786655	0	0.94180063	0	1	0	9.26740448	9.76073581	9.68991625	9.40635199
414	**ENSG0000002255**	NLRP2	protein_coding	chr19	54953130	55001142	+	Human GRCh	OTTHUMG000000	NLRP2	**NLR family pyrin domain containing 2**	Nucleus	other		1.199450056	0	0.85944318	0	1	0	9.69082149	10.1218797	9.96854044	10.3052723
415	**ENSG0000002256**	SLC45A4	protein_coding	chr8	141207166	141308305	-	Human GRCh	OTTHUMG000001	SLC45A4	**solute carrier family 45 member 4**	Other	transporter		-1.0105734	0	0.9763619	0	1	0	9.78852273	10.0125367	9.95379652	9.83365737
416	**ENSG0000002284**	RNF10	protein_coding	chr12	120533480	120577588	+	Human GRCh	OTTHUMG000001	RNF10	**ring finger protein 10**	Cytoplasm	enzyme		-1.141441965	0	0.51525788	0	1	0	11.6384435	12.0040461	11.7800914	11.5869068
417	**ENSG0000002297**	ZNF839	protein_coding	chr14	102317377	102342702	+	Human GRCh	OTTHUMG000001	ZNF839	**zinc finger protein 839**	Other	other		-1.112981167	0	0.63058427	0	1	0	9.73982837	10.1267261	9.99807958	9.63627031
418	**ENSG0000002304**	ZDHHC6	protein_coding	chr10	112424428	112447572	-	Human GRCh	OTTHUMG000000	ZDHHC6	**zinc finger DHHC-type palmitoyltransferase 6**	Nucleus	enzyme		-1.176289996	0	0.42946328	0	1	0	10.1318097	10.3794469	10.1909559	9.96460462
419	**ENSG0000002317**	GRAMD1B	protein_coding	chr11	123358428	123627774	+	Human GRCh	OTTHUMG000001	GRAMD1B	**GRAM domain containing 1B**	Cytoplasm	transporter		-2.12556097	-1	0.16170661	0	1	0	3.58221967	4.35621283	3.83915177	2.84139998
420	**ENSG0000002319**	RNH1	protein_coding	chr11	494512	507300	-	Human GRCh	OTTHUMG000001	RNH1	**ribonuclease/angiogenin inhibitor 1**	Cytoplasm	other		1.159482034	0	0.49074157	0	1	0	10.5899386	10.861063	10.9935438	10.7974782
421	**ENSG0000002322**	NDUFS1	protein_coding	chr2	206114817	206159509	-	Human GRCh	OTTHUMG000001	NDUFS1	**NADH:ubiquinone oxidoreductase core subunit S1**	Cytoplasm	enzyme		-1.09738856	0	0.67264029	0	1	0	12.570184	12.9892164	12.8709862	12.4855581
422	**ENSG0000002328**	RB1CC1	protein_coding	chr8	52622458	52745843	-	Human GRCh	OTTHUMG000001	RB1CC1	**RB1 inducible coiled-coil 1**	Nucleus	other		-1.204432786	0	0.31968003	0	1	0	11.758919	12.0939264	11.8424877	11.618028
423	**ENSG0000002331**	ERP44	protein_coding	chr9	99979185	100099052	-	Human GRCh	OTTHUMG000000	ERP44	**endoplasmic reticulum protein 44**	Cytoplasm	enzyme		-1.118840109	0	0.59369798	0	1	0	10.6593117	10.9054178	10.8339167	10.4654081
424	**ENSG0000002333**	ALAS1	protein_coding	chr3	52198086	52214327	+	Human GRCh	OTTHUMG000001	ALAS1	**5'-aminolevulinate synthase 1**	Cytoplasm	enzyme	givosiran	-1.154945319	0	0.53664367	0	1	0	10.3330828	10.8555418	10.5892405	10.3180241
425	**ENSG0000002344**	BIRC3	protein_coding	chr11	102317484	102339403	+	Human GRCh	OTTHUMG000001	BIRC3	**baculoviral IAP repeat containing 3**	Cytoplasm	enzyme	TQB3728,	1.234988524	0	0.58677233	0	1	0	5.06877086	5.2621289	4.99164767	5.63343727
426	**ENSG0000002351**	AKAP11	protein_coding	chr13	42272152	42323261	+	Human GRCh	OTTHUMG000000	AKAP11	**A-kinase anchoring protein 11**	Cytoplasm	other		-1.057057283	0	0.79547197	0	1	0	11.5691947	11.7342962	11.7703868	11.3848487
427	**ENSG0000002357**	GLRX2	protein_coding	chr1	193090866	193106114	-	Human GRCh	OTTHUMG000000	GLRX2	**glutaredoxin 2**	Cytoplasm	enzyme		1.254535904	0	0.4038512	0	1	0	9.52493005	9.73369606	10.1473401	9.5609909
428	**ENSG0000002360**	SNAPC1	protein_coding	chr14	61762420	61796428	+	Human GRCh	OTTHUMG000001	SNAPC1	**small nuclear RNA activating complex polypeptide 1**	Nucleus	transcription		1.089391744	0	0.7544425	0	1	0	8.31066517	8.73137103	8.81511863	8.41628643
429	**ENSG0000002369**	DERA	protein_coding	chr12	15911302	16037381	+	Human GRCh	OTTHUMG000001	DERA	**deoxyribose-phosphate aldolase**	Cytoplasm	enzyme		-1.1536738	0	0.59194238	0	1	0	8.45950714	8.73466037	8.50580038	8.40328195
430	**ENSG0000002373**	STRAP	protein_coding	chr12	15882387	15903478	+	Human GRCh	OTTHUMG000001	STRAP	**serine/threonine kinase receptor associated protein**	Plasma Mem	other		-1.087985115	0	0.65143873	0	1	0	11.3835952	11.6923157	11.5637658	11.3314158
431	**ENSG0000002383**	ABCC2	protein_coding	chr10	99782640	99852594	+	Human GRCh	OTTHUMG000000	ABCC2	**ATP binding cassette subfamily C member 2**	Plasma Mem	transporter	sulfinpyraz	-1.391104817	0	0.3725952	0	1	0	6.0133457	6.11748354	5.63316904	5.83184763
432	**ENSG0000002389**	DEF6	protein_coding	chr6	35297818	35321771	+	Human GRCh	OTTHUMG000000	DEF6	**DEF6 guanine nucleotide exchange factor**	Extracellular	other		-1.009635358	0	0.9687881	0	1	0	8.74978443	9.10432937	8.88219946	8.97433304
433	**ENSG0000002390**	PLEKHO1	protein_coding	chr1	150149183	150164720	+	Human GRCh	OTTHUMG000000	PLEKHO1	**pleckstrin homology domain containing O1**	Plasma Mem	other		-1.139492554	0	0.60068555	0	1	0	9.4838084	9.72593917	9.62569692	9.28489521
434	**ENSG0000002390**	GCLM	protein_coding	chr1	93885199	93909456	-	Human GRCh	OTTHUMG000000	GCLM	**glutamate-cysteine ligase modifier subunit**	Cytoplasm	enzyme		-1.021513228	0	0.92385437	0	1	0	8.15903129	8.41425185	8.42363302	8.0951483
435	**ENSG0000002404**	UBR2	protein_coding	chr6	42564029	42693505	+	Human GRCh	OTTHUMG000000	UBR2	**ubiquitin protein ligase E3 component n-recognin 2**	Nucleus	enzyme		-1.148131319	0	0.47797116	0	1	0	11.314725	11.5914091	11.4330921	11.1710905
436	**ENSG0000002442**	EHD2	protein_coding	chr19	47713422	47743134	+	Human GRCh	OTTHUMG000001	EHD2	**EH domain containing 2**	Nucleus	other		-2.904541354	-1	0.31370588	0	NA	0	3.17794521	3.12174213	1.79261156	2.73884943
437	**ENSG0000002452**	DEPDC1	protein_coding	chr1	68474152	68497221	-	Human GRCh	OTTHUMG000000	DEPDC1	**DEP domain containing 1**	Nucleus	transcription		-1.113889441	0	0.63589178	0	1	0	11.13128	11.2810246	11.2616294	10.890409
438	**ENSG0000002486**	CCDC28A	protein_coding	chr6	138773769	138793319	+	Human GRCh	OTTHUMG000000	CCDC28A	**coiled-coil domain containing 28A**	Other	other		-1.219861597	0	0.43219165	0	1	0	7.22599518	7.2949245	7.10652593	7.00762782
439	**ENSG0000002503**	RRAGD	protein_coding	chr6	89364616	89412273	-	Human GRCh	OTTHUMG000000	RRAGD	**Ras related GTP binding D**	Cytoplasm	enzyme		1.099045139	0	0.68480889	0	1	0	10.5665939	10.8072951	10.9693329	10.5971666
440	**ENSG0000002515**	HSF2	protein_coding	chr6	122399551	122433119	+	Human GRCh	OTTHUMG000000	HSF2	**heat shock transcription factor 2**	Nucleus	transcription		-1.053475349	0	0.82011521	0	1	0	8.99157282	9.30074874	9.29402109	8.86772018
441	**ENSG0000002529**	PHF20	protein_coding	chr20	35771974	35950370	+	Human GRCh	OTTHUMG000000	PHF20	**PHD finger protein 20**	Nucleus	transcription		-1.11915707	0	0.56774299	0	1	0	10.7741157	11.0789158	10.9881947	10.6046428
442	**ENSG0000002542**	HSD17B6	protein_coding	chr12	56752161	56787790	+	Human GRCh	OTTHUMG000001	HSD17B6	**hydroxysteroid 17-beta dehydrogenase 6**	Other	enzyme	3-hydroxys	-1.376332762	0	0.38861858	0	1	0	5.40347594	5.24696828	5.37250439	4.61960724
443	**ENSG0000002543**	NR1H3	protein_coding	chr11	47248300	47269033	+	Human GRCh	OTTHUMG000001	NR1H3	**nuclear receptor subfamily 1 group H member 3**	Nucleus	ligand-depen		1.162057334	0	0.52472649	0	1	0	8.56786944	8.65823934	8.94297357	8.58444771
444	**ENSG0000002570**	TYMP	protein_coding	chr22	50525752	50530032	-	Human GRCh	OTTHUMG000001	TYMP	**thymidine phosphorylase**	Extracellular	growth facto	tipiracil	-1.076550821	0	0.88538564	0	1	0	5.85033349	6.25740315	5.8982096	6.08143899
445	**ENSG0000002577**	NCAPH2	protein_coding	chr22	50508224	50524780	+	Human GRCh	OTTHUMG000001	NCAPH2	**non-SMC condensin II complex subunit H2**	Nucleus	other		-1.07589775	0	0.72457758	0	1	0	10.5168933	10.9003352	10.7499	10.5253055
446	**ENSG0000002577**	TOMM34	protein_coding	chr20	44942130	44960397	-	Human GRCh	OTTHUMG000000	TOMM34	**translocase of outer mitochondrial membrane 34**	Cytoplasm	other		-1.132877644	0	0.57766708	0	1	0	10.0383775	10.3802117	10.2716195	9.85053736
447	**ENSG0000002579**	SEC63	protein_coding	chr6	107867756	107958208	-	Human GRCh	OTTHUMG000000	SEC63	**SEC63 homolog, protein translocation regulator**	Cytoplasm	transporter		-1.046297691	0	0.81945161	0	1	0	11.5931084	11.8292072	11.7509874	11.5771429
448	**ENSG0000002580**	KPNA6	protein_coding	chr1	32108056	32176563	+	Human GRCh	OTTHUMG000000	KPNA6	**karyopherin subunit alpha 6**	Nucleus	other		-1.132491679	0	0.53072514	0	1	0	11.6910476	12.0701381	11.8903853	11.6069896
449	**ENSG0000002602**	VIM	protein_coding	chr10	17228241	17237593	+	Human GRCh	OTTHUMG000000	VIM	**vimentin**	Cytoplasm	other	pritumum	1.087182329	0	0.85301435	0	1	0	4.05917737	4.65402921	4.62278371	4.28838618
450	**ENSG0000002610**	FAS	protein_coding	chr10	88953813	89029605	+	Human GRCh		FAS	**Fas cell surface death receptor**	Plasma Mem	transmembra	APO010, n	-1.554146985	-1	0.39794635	0	1	0	5.5134989	5.7079765	4.98102087	5.42010586
451	**ENSG0000002629**	RNASET2	protein_coding	chr6	166922113	166957191	-	Human GRCh	OTTHUMG000000	RNASET2	**ribonuclease T2**	Cytoplasm	enzyme		1.12656927	0	0.61438542	0	1	0	9.45798087	9.8737953	9.94779891	9.66971983
452	**ENSG0000002650**	CD44	protein_coding	chr11	35138882	35232402	+	Human GRCh	OTTHUMG000000	CD44	**CD44 molecule (Indian blood group)**	Plasma Mem	other	SPL-108, A	1.498486966	0	0.17281479	0	1	0	7.47568567	7.52732171	7.8906369	7.95419679

453	**ENSG0000002655**	KCNG1	protein_coding	chr20	51003656	51023107	-	Human GRCh	OTTHUMG000000	KCNG1	**potassium voltage-gated channel modifier subfamily G membe**	Plasma Mem	ion channel		1.297913467	0	0.77223888	0	1	0	4.43112822	3.31495652	3.13770569	4.80734735
454	**ENSG0000002665**	AGPAT4	protein_coding	chr6	161129967	161274061	-	Human GRCh	OTTHUMG000000	AGPAT4	**1-acylglycerol-3-phosphate O-acyltransferase 4**	Cytoplasm	enzyme		-2.056589188	-1	0.01639459	1	1	0	7.84039612	8.33551384	7.43741706	7.23217037
456	**ENSG0000002695**	BTN3A1	protein_coding	chr6	26402237	26415208	+	Human GRCh	OTTHUMG000000	BTN3A1	**butyrophilin subfamily 3 member A1**	Extracellular	other		-1.166830553	0	0.58677136	0	1	0	6.81702843	7.13357271	6.89973784	6.75852849
457	**ENSG0000002700**	MIPEP	protein_coding	chr13	23730189	23889400	-	Human GRCh	OTTHUMG000000	MIPEP	**mitochondrial intermediate peptidase**	Cytoplasm	peptidase		-1.129457293	0	0.60954375	0	1	0	8.92205527	9.23816513	9.08393193	8.80974475
458	**ENSG0000002707**	PRKCH	protein_coding	chr14	61187559	61550976	+	Human GRCh	OTTHUMG000001	PRKCH	**protein kinase C eta**	Cytoplasm	kinase	ingenol me	1.386995026	0	0.33736649	0	1	0	7.49773942	8.0082572	7.71497332	8.41731791
459	**ENSG0000002764**	INSRR	protein_coding	chr1	156840063	156859117	-	Human GRCh	OTTHUMG000000	INSRR	**insulin receptor related receptor**	Plasma Mem	kinase		13.23734294	1	0.02278421	1	1	0	2.45966216	2.56125358	2.54489523	3.478642
460	**ENSG0000002769**	IFNGR1	protein_coding	chr6	137197483	137219449	-	Human GRCh	OTTHUMG000000	IFNGR1	**interferon gamma receptor 1**	Plasma Mem	transmembra	interferon	-1.286096027	0	0.26388264	0	1	0	9.36702317	9.48067791	9.18896683	9.09865554
461	**ENSG0000002784**	B4GALT7	protein_coding	chr5	177600132	177610330	+	Human GRCh	OTTHUMG000001	B4GALT7	**beta-1,4-galactosyltransferase 7**	Cytoplasm	enzyme		1.031481996	0	0.89125131	0	1	0	9.25231785	9.51732272	9.42327257	9.42889091
462	**ENSG0000002786**	SH2D2A	protein_coding	chr1	156806243	156816848	-	Human GRCh	OTTHUMG000000	SH2D2A	**SH2 domain containing 2A**	Cytoplasm	other		1.197979608	0	0.54881541	0	1	0	5.59684742	5.47942946	5.33618418	5.96631095
463	**ENSG0000002811**	VRK2	protein_coding	chr2	57907629	58159920	+	Human GRCh	OTTHUMG000001	VRK2	**VRK serine/threonine kinase 2**	Nucleus	kinase		-1.215459686	0	0.37790885	0	1	0	9.01632282	9.23404072	8.9743019	8.86015161
464	**ENSG0000002813**	TNFRSF1B	protein_coding	chr1	12166991	12209228	+	Human GRCh	OTTHUMG000000	TNFRSF1B	**TNF receptor superfamily member 1B**	Plasma Mem	transmembra	BI-1808, H	16.45273556	1	0.03611782	1	1	0	2.34981369	2.43988267	3.34401695	2.87807049
465	**ENSG0000002820**	VEZT	protein_coding	chr12	95217746	95302799	+	Human GRCh	OTTHUMG000001	VEZT	**vezatin, adherens junctions transmembrane protein**	Plasma Mem	other		-1.153508458	0	0.46331514	0	1	0	11.1313019	11.2780854	11.1741555	10.9276306
466	**ENSG0000002827**	POU2F2	protein_coding	chr19	42086110	42196585	-	Human GRCh	OTTHUMG000001	POU2F2	**POU class 2 homeobox 2**	Nucleus	transcription		-1.209495183	0	0.54753439	0	1	0	6.83051198	7.47676526	7.01231314	6.98518399
467	**ENSG0000002831**	BRD9	protein_coding	chr5	850291	892801	-	Human GRCh	OTTHUMG000001	BRD9	**bromodomain containing 9**	Nucleus	other	FHD-609, (	-1.136224991	0	0.51164038	0	1	0	11.2808514	11.5754836	11.4251142	11.1547552
468	**ENSG0000002852**	SNX1	protein_coding	chr15	64094123	64146090	+	Human GRCh	OTTHUMG000001	SNX1	**sorting nexin 1**	Cytoplasm	transporter		-1.05921736	0	0.77305796	0	1	0	11.2983576	11.6337969	11.5258758	11.2902817
469	**ENSG0000002883**	TBPL1	protein_coding	chr6	133952170	133990432	+	Human GRCh	OTTHUMG000000	TBPL1	**TATA-box binding protein like 1**	Nucleus	transcription		-1.107984583	0	0.66412834	0	1	0	11.1816943	11.4050708	11.4010322	10.9107745
470	**ENSG0000002915**	BMAL2	protein_coding	chr12	27332836	27425289	+	Human GRCh	OTTHUMG000001	BMAL2	**basic helix-loop-helix ARNT like 2**	Nucleus	transcription		-1.206058088	0	0.46033427	0	1	0	10.0917163	10.4034531	10.2766869	9.76468762
471	**ENSG0000002936**	BCLAF1	protein_coding	chr6	136256627	136289851	-	Human GRCh	OTTHUMG000000	BCLAF1	**BCL2 associated transcription factor 1**	Nucleus	transcription		-1.213525624	0	0.33088212	0	1	0	12.2786123	12.6021741	12.3627535	12.1009602
472	**ENSG0000002936**	SLC39A9	protein_coding	chr14	69398015	69462390	+	Human GRCh	OTTHUMG000001	SLC39A9	**solute carrier family 39 member 9**	Cytoplasm	transporter		-1.122550925	0	0.59841349	0	1	0	11.7949453	12.1656995	11.9947971	11.7217514
473	**ENSG0000002953**	ANK1	protein_coding	chr8	41653220	41896741	-	Human GRCh	OTTHUMG000001	ANK1	**ankyrin 1**	Plasma Mem	other		-1.320840528	0	0.53059417	0	1	0	4.59675832	4.47064594	4.60523319	4.03001632
474	**ENSG0000002955**	IBSP	protein_coding	chr4	87799554	87812435	+	Human GRCh	OTTHUMG000001	IBSP	**integrin binding sialoprotein**	Extracellular	other		-10.21043898	-1	0.1386331	0	NA	0	2.14110068	1.438369	1.43405934	1.41389873
475	**ENSG0000002963**	TFB1M	protein_coding	chr6	155256134	155314493	-	Human GRCh	OTTHUMG000000	TFB1M	**transcription factor B1, mitochondrial**	Cytoplasm	transcription		-1.315235401	0	0.20988172	0	1	0	8.62376652	9.10973996	8.82497693	8.30876908
476	**ENSG0000002972**	RABEP1	protein_coding	chr17	5282265	5386340	+	Human GRCh	OTTHUMG000001	RABEP1	**rabaptin, RAB GTPase binding effector protein 1**	Cytoplasm	transporter		-1.028830999	0	0.87854247	0	1	0	10.9340612	11.2449585	11.1372451	10.9944814
477	**ENSG0000002999**	HMGB3	protein_coding	chrX	150980509	150990771	+	Human GRCh	OTTHUMG000000	HMGB3	**high mobility group box 3**	Nucleus	other		-1.160606915	0	0.44531802	0	1	0	11.37817	11.6494683	11.4755264	11.2329789
478	**ENSG0000003006**	NUP160	protein_coding	chr11	47778087	47848555	-	Human GRCh	OTTHUMG000001	NUP160	**nucleoporin 160**	Nucleus	other		-1.143121391	0	0.51316724	0	1	0	11.6590075	11.9985939	11.8854527	11.4684035
479	**ENSG0000003011**	BAK1	protein_coding	chr6	33572547	33580293	-	Human GRCh	OTTHUMG000000	BAK1	**BCL2 antagonist/killer 1**	Cytoplasm	transporter		1.188240728	0	0.39675022	0	1	0	8.94111833	9.18836166	9.37619467	9.13752949
481	**ENSG0000003041**	IKZF2	protein_coding	chr2	212999691	213152427	-	Human GRCh	OTTHUMG000001	IKZF2	**IKAROS family zinc finger 2**	Nucleus	transcription	DKY709	-1.194627319	0	0.54429195	0	1	0	10.6665103	10.7031357	10.6430235	10.2975355
482	**ENSG0000003058**	GRN	protein_coding	chr17	44345246	44353106	+	Human GRCh	OTTHUMG000001	GRN	**granulin precursor**	Extracellular	growth facto	AG01	1.04109031	0	0.85725033	0	1	0	11.3697077	11.6655371	11.6502793	11.4869737
483	**ENSG0000003100**	FAM13B	protein_coding	chr5	137937960	138051961	-	Human GRCh	OTTHUMG000001	FAM13B	**family with sequence similarity 13 member B**	Cytoplasm	other		-1.090901069	0	0.71037828	0	1	0	10.6438594	10.9437549	10.9253487	10.441129
484	**ENSG0000003108**	ARHGAP31	protein_coding	chr3	119294383	119420714	+	Human GRCh	OTTHUMG000001	ARHGAP31	**Rho GTPase activating protein 31**	Cytoplasm	other		-2.16514734	-1	0.16804988	0	1	0	4.90091373	4.43249707	4.13968812	3.80556378
485	**ENSG0000003169**	CENPQ	protein_coding	chr6	49463370	49493107	+	Human GRCh	OTTHUMG000000	CENPQ	**centromere protein Q**	Nucleus	other		-1.523154141	-1	0.08672443	0	1	0	7.82441853	8.23172818	7.76391511	7.40158078
486	**ENSG0000003169**	SARS1	protein_coding	chr1	109213918	109238182	+	Human GRCh	OTTHUMG000000	SARS1	**seryl-tRNA synthetase 1**	Cytoplasm	enzyme		-1.195787862	0	0.32292327	0	1	0	11.3425311	11.3939314	11.3103012	11.0073355
487	**ENSG0000003182**	RANBP3	protein_coding	chr19	5916139	5978142	-	Human GRCh	OTTHUMG000001	RANBP3	**RAN binding protein 3**	Nucleus	other		-1.117449066	0	0.55983431	0	1	0	11.0647888	11.3423073	11.20952	10.9573098
488	**ENSG0000003221**	ARID4A	protein_coding	chr14	58298504	58373887	+	Human GRCh	OTTHUMG000001	ARID4A	**AT-rich interaction domain 4A**	Nucleus	transcription		1.034926507	0	0.8549585	0	1	0	9.84958505	10.0745268	10.0781078	9.92592681
489	**ENSG0000003238**	EIPR1	protein_coding	chr2	3188925	3377830	-	Human GRCh	OTTHUMG000000	EIPR1	**EARP complex and GARP complex interacting protein 1**	Cytoplasm	other		-1.087987391	0	0.65421816	0	1	0	9.52829857	9.84942991	9.73004672	9.46804752
490	**ENSG0000003244**	PNPLA6	protein_coding	chr19	7534004	7561764	+	Human GRCh	OTTHUMG000001	PNPLA6	**patatin like phospholipase domain containing 6**	Cytoplasm	enzyme		-1.004977848	0	0.97935709	0	1	0	10.9953602	11.2704859	11.1982178	11.0688394
491	**ENSG0000003274**	IFT88	protein_coding	chr13	20567138	20691444	+	Human GRCh	OTTHUMG000000	IFT88	**intraflagellar transport 88**	Cytoplasm	other		-1.019648392	0	0.93698649	0	1	0	9.58712902	9.89067937	9.98310293	9.39926684
492	**ENSG0000003301**	ALG1	protein_coding	chr16	5033702	5087379	+	Human GRCh	OTTHUMG000001	ALG1	**ALG1 chitobiosyldiphosphodolichol beta-mannosyltransferase**	Cytoplasm	enzyme		-1.099125748	0	0.65327453	0	1	0	10.0427299	10.1596492	10.1279307	9.84914099
493	**ENSG0000003303**	ZCCHC8	protein_coding	chr12	122471600	122500932	-	Human GRCh	OTTHUMG000001	ZCCHC8	**zinc finger CCHC-type containing 8**	Nucleus	other		-1.013498277	0	0.94729158	0	1	0	10.1937761	10.2798209	10.4190026	9.98757321
494	**ENSG0000003305**	ABCF2	protein_coding	chr7	151211484	151227205	-	Human GRCh	OTTHUMG000001	ABCF2	**ATP binding cassette subfamily F member 2**	Cytoplasm	transporter		-1.069315508	0	0.74018006	0	1	0	11.5236593	11.8586193	11.7309306	11.5105185
495	**ENSG0000003310**	CHPF2	protein_coding	chr7	151232483	151240979	+	Human GRCh	OTTHUMG000001	CHPF2	**chondroitin polymerizing factor 2**	Cytoplasm	enzyme		-1.06876271	0	0.76679096	0	1	0	11.236882	11.3907081	11.371748	11.0936627
496	**ENSG0000003312**	LRRC7	protein_coding	chr1	69567922	70151945	+	Human GRCh	OTTHUMG000000	LRRC7	**leucine rich repeat containing 7**	Plasma Mem	other		-1.024532571	0	0.96700345	0	1	0	6.72722152	6.91921577	6.85202589	6.75089643
497	**ENSG0000003317**	FUT8	protein_coding	chr14	65410592	65744121	+	Human GRCh	OTTHUMG000001	FUT8	**fucosyltransferase 8**	Cytoplasm	enzyme		1.339828676	0	0.25224537	0	1	0	7.80185077	7.67156461	7.72713288	8.26214503
498	**ENSG0000003317**	UBA6	protein_coding	chr4	67612652	67701155	-	Human GRCh	OTTHUMG000001	UBA6	**ubiquitin like modifier activating enzyme 6**	Cytoplasm	enzyme		-1.031748747	0	0.88535733	0	1	0	11.3893084	11.6539205	11.6775777	11.2771753
499	**ENSG0000003332**	GAB2	protein_coding	chr11	78215293	78418348	-	Human GRCh	OTTHUMG000001	GAB2	**GRB2 associated binding protein 2**	Cytoplasm	other		-1.106574572	0	0.67970052	0	1	0	9.49869722	9.80973725	9.70441704	9.3853159
500	**ENSG0000003362**	ATP6V0A1	protein_coding	chr17	42458844	42522582	+	Human GRCh	OTTHUMG000001	ATP6V0A1	**ATPase H+ transporting V0 subunit a1**	Cytoplasm	transporter		-1.040253335	0	0.84995706	0	1	0	11.5377043	11.7842709	11.7273252	11.5096341
501	**ENSG0000003380**	PIAS1	protein_coding	chr15	68054309	68198603	+	Human GRCh	OTTHUMG000001	PIAS1	**protein inhibitor of activated STAT 1**	Nucleus	transcription		-1.579090436	-1	0.08335732	0	1	0	12.8477806	13.0621691	12.5818793	12.2486354
502	**ENSG0000003386**	SLC4A7	protein_coding	chr3	27372721	27484420	-	Human GRCh	OTTHUMG000001	SLC4A7	**solute carrier family 4 member 7**	Plasma Mem	transporter		-1.096395766	0	0.69049892	0	1	0	10.9100856	11.2133127	11.1346533	10.7760963
503	**ENSG0000003405**	APBA2	protein_coding	chr15	28884483	29118315	+	Human GRCh	OTTHUMG000001	APBA2	**amyloid beta precursor protein binding family A member 2**	Cytoplasm	transporter		-1.033643594	0	0.89237251	0	1	0	9.89909594	10.0233484	10.0713043	9.76093907
504	**ENSG0000003415**	MAP2K3	protein_coding	chr17	21284672	21315232	+	Human GRCh	OTTHUMG000001	MAP2K3	**mitogen-activated protein kinase kinase 3**	Cytoplasm	kinase		1.222195122	0	0.32481187	0	1	0	9.52732421	9.92579684	9.86657744	10.0606359
505	**ENSG0000003423**	CLXN	protein_coding	chr8	48710789	48735311	-	Human GRCh	OTTHUMG000001	CLXN	**calaxin**	Cytoplasm	other		1.778012391	1	0.5310831	0	1	0	4.51128842	4.72366446	5.26642497	5.16929396
506	**ENSG0000003451**	TMSB10	protein_coding	chr2	84905656	84906671	+	Human GRCh	OTTHUMG000001	TMSB10/TMS	**thymosin beta 4 X-linked**	Cytoplasm	other		-1.060110431	0	0.77172352	0	1	0	14.3981328	14.7166054	14.5992355	14.3966892
507	**ENSG0000003453**	ASTE1	protein_coding	chr3	131013875	131027649	-	Human GRCh	OTTHUMG000001	ASTE1	**asteroid homolog 1**	Other	other		1.25777123	0	0.42721753	0	1	0	6.86605789	7.13263738	7.54085217	6.84384273
508	**ENSG0000003467**	RNF19A	protein_coding	chr8	100257060	100336218	-	Human GRCh	OTTHUMG000001	RNF19A	**ring finger protein 19A, RBR E3 ubiquitin protein ligase**	Nucleus	enzyme		-1.06212159	0	0.76574086	0	1	0	9.79067459	10.041447	9.95442251	9.75477215
509	**ENSG0000003469**	PEX3	protein_coding	chr6	143450805	143490616	+	Human GRCh	OTTHUMG000000	PEX3	**peroxisomal biogenesis factor 3**	Cytoplasm	other		1.117691162	0	0.64940586	0	1	0	8.9444088	9.27866598	9.42037249	9.04056873
510	**ENSG0000003471**	GABARAPL	protein_coding	chr16	75566375	75577881	+	Human GRCh	OTTHUMG000001	GABARAPL2	**GABA type A receptor associated protein like 2**	Cytoplasm	other		-1.101820373	0	0.67646488	0	1	0	9.94606751	10.2317099	10.0935101	9.87367304
512	**ENSG0000003511**	SH3YL1	protein_coding	chr2	217730	266398	-	Human GRCh	OTTHUMG000001	SH3YL1	**SH3 and SYLF domain containing 1**	Plasma Mem	other		1.0487681	0	0.84488708	0	1	0	9.53888347	9.66495524	9.90031863	9.34623407
513	**ENSG0000003514**	FAM136A	protein_coding	chr2	70295976	70302067	-	Human GRCh	OTTHUMG000001	FAM136A	**family with sequence similarity 136 member A**	Cytoplasm	other		-1.050031737	0	0.80815396	0	1	0	10.7098505	10.9645896	10.9338711	10.6249244
514	**ENSG0000003540**	VCL	protein_coding	chr10	73995193	74121363	+	Human GRCh	OTTHUMG000000	VCL	**vinculin**	Plasma Mem	other		-1.024295366	0	0.90307387	0	1	0	12.0496353	12.3307901	12.2189568	12.1221309
515	**ENSG0000003549**	DEPDC1B	protein_coding	chr5	60596912	60700190	-	Human GRCh	OTTHUMG000000	DEPDC1B	**DEP domain containing 1B**	Cytoplasm	other		-1.235176157	0	0.34550749	0	1	0	9.76984529	10.0896112	9.79666004	9.62035195
516	**ENSG0000003566**	DAPK2	protein_coding	chr15	63907036	64072033	-	Human GRCh	OTTHUMG000001	DAPK2	**death associated protein kinase 2**	Cytoplasm	kinase		1.400541934	0	0.20305001	0	1	0	6.47096721	6.3680133	6.57948907	6.87874013
517	**ENSG0000003568**	NSMAF	protein_coding	chr8	58583508	58659853	-	Human GRCh	OTTHUMG000001	NSMAF	**neutral sphingomyelinase activation associated factor**	Cytoplasm	other		-1.070592154	0	0.74957142	0	1	0	9.70766529	9.99118558	9.91123348	9.64447025
518	**ENSG0000003568**	ADSS2	protein_coding	chr1	244408494	244451909	-	Human GRCh	OTTHUMG000000	ADSS2	**adenylosuccinate synthase 2**	Cytoplasm	enzyme	alanosine,	1.0093917	0	0.96303665	0	1	0	10.0618491	10.3787426	10.4101589	10.0440089
519	**ENSG0000003572**	STAP1	protein_coding	chr4	67558727	67607337	+	Human GRCh	OTTHUMG000001	STAP1	**signal transducing adaptor family member 1**	Cytoplasm	other		-1.170684925	0	0.86511438	0	1	0	3.08102219	2.22374695	2.46474377	2.87240303
520	**ENSG0000003586**	TIMP2	protein_coding	chr17	78852977	78925387	-	Human GRCh	OTTHUMG000001	TIMP2	**TIMP metallopeptidase inhibitor 2**	Extracellular	other		1.525808226	1	0.30938931	0	1	0	5.88145299	5.41980739	5.91321466	6.16502822
521	**ENSG0000003592**	RFC1	protein_coding	chr4	39287456	39366375	-	Human GRCh	OTTHUMG000000	RFC1	**replication factor C subunit 1**	Nucleus	transcription		-1.096739358	0	0.67656873	0	1	0	11.5590153	11.9625719	11.8038574	11.5295225
522	**ENSG0000003605**	TBC1D23	protein_coding	chr3	100260992	100325251	+	Human GRCh	OTTHUMG000001	TBC1D23	**TBC1 domain family member 23**	Cytoplasm	other		1.037768173	0	0.86661527	0	1	0	9.18311326	9.35644242	9.47435558	9.12589538
523	**ENSG0000003625**	CUL3	protein_coding	chr2	224470150	224585397	-	Human GRCh	OTTHUMG000001	CUL3	**cullin 3**	Nucleus	enzyme		-1.010722699	0	0.95890954	0	1	0	11.1715622	11.5402967	11.5025253	11.1942079
526	**ENSG0000003653**	CYP46A1	protein_coding	chr14	99684298	99727301	+	Human GRCh	OTTHUMG000001	CYP46A1	**cytochrome P450 family 46 subfamily A member 1**	Cytoplasm	enzyme		1.052428777	0	0.91474659	0	1	0	6.58060601	7.0466057	6.78773721	6.98765163
527	**ENSG0000003654**	ZZZ3	protein_coding	chr1	77562416	77683419	-	Human GRCh	OTTHUMG000000	ZZZ3	**zinc finger ZZ-type containing 3**	Nucleus	transcription		-1.082720731	0	0.70215635	0	1	0	11.0729328	11.3506122	11.3458949	10.8711533
528	**ENSG0000003656**	SLC18A1	protein_coding	chr8	20144855	20183206	-	Human GRCh	OTTHUMG000000	SLC18A1	**solute carrier family 18 member A1**	Plasma Mem	transporter	reserpine,	1	0	1	0	NA	0	0.92503486	0.96254703	0.95639382	0.92725465
529	**ENSG0000003667**	USP2	protein_coding	chr11	119355215	119381711	-	Human GRCh	OTTHUMG000001	USP2	**ubiquitin specific peptidase 2**	Cytoplasm	peptidase		1.561459888	1	0.22555286	0	1	0	8.25745239	8.75035523	8.75736334	9.23180195
530	**ENSG0000003682**	CASR	protein_coding	chr3	122183668	122291629	+	Human GRCh	OTTHUMG000001	CASR	**calcium sensing receptor**	Plasma Mem	G-protein cou	velcalcetid	20.00696262	1	0.10408227	0	NA	0	0.99900114	1.01235045	1.01011637	2.01367406
531	**ENSG0000003704**	TUBG2	protein_coding	chr17	42659284	42667006	+	Human GRCh	OTTHUMG000001	TUBG2	**tubulin gamma 2**	Cytoplasm	other	docetaxel/	1.096494448	0	0.72261279	0	1	0	8.66897057	8.9038435	8.91656738	8.86957263
532	**ENSG0000003724**	RPL26L1	protein_coding	chr5	172958729	172969771	+	Human GRCh	OTTHUMG000001	RPL26L1	**ribosomal protein L26 like 1**	Cytoplasm	other		-1.037597573	0	0.89122702	0	1	0	8.29362891	8.84401134	8.50002807	8.6145447
533	**ENSG0000003728**	FLT4	protein_coding	chr5	180601506	180649624	-	Human GRCh	OTTHUMG000001	FLT4	**fms related receptor tyrosine kinase 4**	Plasma Mem	transmembra	docetaxel/	1.810995977	1	0.12975091	0	1	0	4.4171914	5.40694709	5.15073586	5.78760907
534	**ENSG0000003747**	NSUN2	protein_coding	chr5	6599239	6633291	-	Human GRCh	OTTHUMG000000	NSUN2	**NOP2/Sun RNA methyltransferase 2**	Nucleus	enzyme		-1.095228962	0	0.6302746	0	1	0	11.4966381	11.8328753	11.7159012	11.4140398
535	**ENSG0000003763**	FBXO42	protein_coding	chr1	16246840	16352480	-	Human GRCh	OTTHUMG000000	FBXO42	**F-box protein 42**	Other	other		-1.045442878	0	0.84188278	0	1	0	10.8405528	11.120293	11.0537362	10.8126714
536	**ENSG0000003774**	MFAP3	protein_coding	chr5	154038959	154220478	+	Human GRCh	OTTHUMG000001	MFAP3	**microfibril associated protein 3**	Extracellular	other		-1.150391761	0	0.4947434	0	1	0	10.1437431	10.3377879	10.2457322	9.90973522
537	**ENSG0000003775**	MRI1	protein_coding	chr19	13764522	13774282	+	Human GRCh	OTTHUMG000001	MRI1	**methylthioribose-1-phosphate isomerase 1**	Cytoplasm	translation re		-1.070913701	0	0.74219759	0	1	0	10.4891532	10.805776	10.6598111	10.4967441
538	**ENSG0000003789**	METTL1	protein_coding	chr12	57768471	57772119	-	Human GRCh	OTTHUMG000001	METTL1	**methyltransferase 1, tRNA methylguanosine**	Nucleus	enzyme		-1.003856507	0	0.98755343	0	1	0	9.05087938	9.26031327	9.34063883	8.93709244
539	**ENSG0000003796**	HOXC8	protein_coding	chr12	54008985	54012769	+	Human GRCh	OTTHUMG000001	HOXC8	**homeobox C8**	Nucleus	transcription		-1.119630259	0	0.82062598	0	1	0	6.5579303	6.57820107	6.72135185	6.07517337
540	**ENSG0000003800**	AGA	protein_coding	chr4	177430774	177442437	-	Human GRCh	OTTHUMG000001	AGA	**aspartylglucosaminidase**	Cytoplasm	enzyme		-1.072310617	0	0.82005218	0	1	0	6.90366223	7.51761211	7.35585161	6.96600749

541	**ENSG0000003821**	PI4K2B	protein_coding	chr4	25160663	25279204	+	Human GRCh	OTTHUMG000001	PI4K2B	**phosphatidylinositol 4-kinase type 2 beta**	Cytoplasm	kinase		-1.086756082	0	0.73269871	0	1	0	9.87645771	10.0633505	10.1470899	9.54359569
542	**ENSG0000003821**	BOD1L1	protein_coding	chr4	13568738	13627725	-	Human GRCh	OTTHUMG000000	BOD1L1	**biorientation of chromosomes in cell division 1 like 1**	Nucleus	other		-1.082180464	0	0.70172552	0	1	0	11.0985399	11.3701098	11.1839577	11.1266942
543	**ENSG0000003827**	MAT2B	protein_coding	chr5	163503114	163519558	+	Human GRCh	OTTHUMG000001	MAT2B	**methionine adenosyltransferase 2 non-catalytic beta subunit**	Cytoplasm	enzyme		1.040813961	0	0.85104499	0	1	0	9.50943485	9.74390317	9.81116421	9.52355251
544	**ENSG0000003829**	TLL1	protein_coding	chr4	165873237	166104457	+	Human GRCh	OTTHUMG000001	TLL1	**tolloid like 1**	Extracellular	peptidase		1.053326146	0	0.9264135	0	1	0	4.39138188	5.45598169	5.2765982	4.88302819
545	**ENSG0000003835**	EDC4	protein_coding	chr16	67873052	67884499	+	Human GRCh	OTTHUMG000001	EDC4	**enhancer of mRNA decapping 4**	Cytoplasm	other		-1.096082255	0	0.65640663	0	1	0	11.9285423	12.2789887	12.1306391	11.8816565
546	**ENSG0000003838**	TRIO	protein_coding	chr5	14143342	14532128	+	Human GRCh	OTTHUMG000001	TRIO	**trio Rho guanine nucleotide exchange factor**	Cytoplasm	kinase		-1.248370409	0	0.29328597	0	1	0	12.6144028	12.8481755	12.6104891	12.3671982
547	**ENSG0000003842**	VCAN	protein_coding	chr5	83471618	83582303	+	Human GRCh	OTTHUMG000001	VCAN	**versican**	Extracellular	other		-1.076079846	0	0.91282284	0	1	0	7.1572741	7.32039573	6.98688123	7.3448414
548	**ENSG0000003853**	CLEC16A	protein_coding	chr16	10944539	11182186	+	Human GRCh	OTTHUMG000001	CLEC16A	**C-type lectin domain containing 16A**	Cytoplasm	other		1.10304532	0	0.64941648	0	1	0	10.7767116	11.0751695	11.0593654	11.0290443
549	**ENSG0000003894**	MSR1	protein_coding	chr8	16107878	16567490	-	Human GRCh	OTTHUMG000000	MSR1	**macrophage scavenger receptor 1**	Plasma Mem	transmembra		1	0	1	0	NA	0	0.9999089	1.02014486	1.01678282	1.00108885
550	**ENSG0000003906**	CDH1	protein_coding	chr16	68737292	68835537	+	Human GRCh	OTTHUMG000001	CDH1	**cadherin 1**	Plasma Mem	other		-1.176921859	0	0.47872594	0	1	0	12.3901029	12.7960778	12.5254796	12.3199563
551	**ENSG0000003912**	MTREX	protein_coding	chr5	55307989	55425579	+	Human GRCh	OTTHUMG000000	MTREX	**Mtr4 exosome RNA helicase**	Nucleus	enzyme		-1.109039238	0	0.5983429	0	1	0	11.118088	11.4569624	11.3310754	11.0191565
552	**ENSG0000003913**	DNAH5	protein_coding	chr5	13690328	14011818	-	Human GRCh	OTTHUMG000000	DNAH5	**dynein axonemal heavy chain 5**	Cytoplasm	enzyme		-1.855469343	-1	0.04678757	1	1	0	8.30470612	8.66967649	7.99813011	7.65039603
553	**ENSG0000003931**	ZFYVE16	protein_coding	chr5	80408013	80483379	+	Human GRCh	OTTHUMG000001	ZFYVE16	**zinc finger FYVE-type containing 16**	Nucleus	other		-1.061263278	0	0.81407751	0	1	0	12.0642625	12.432636	12.3586032	12.0052556
554	**ENSG0000003952**	RIPOR1	protein_coding	chr16	67518418	67546788	+	Human GRCh	OTTHUMG000001	RIPOR1	**RHO family interacting cell polarization regulator 1**	Cytoplasm	other		1.066712943	0	0.78665114	0	1	0	9.9383421	10.1335332	10.1431799	10.0829359
555	**ENSG0000003953**	C6	protein_coding	chr5	41142116	41261438	-	Human GRCh	OTTHUMG000000	C6	**complement C6**	Extracellular	other		5.106753269	1	0.37399862	0	NA	0	1.06271527	1.08411875	1.08056513	1.51335994
556	**ENSG0000003956**	RAI14	protein_coding	chr5	34656328	34832612	+	Human GRCh	OTTHUMG000001	RAI14	**retinoic acid induced 14**	Nucleus	transcription		-1.15219557	0	0.65300082	0	1	0	8.67820381	8.88859397	8.64742607	8.62889037
558	**ENSG0000003965**	PNKP	protein_coding	chr19	49859882	49878351	-	Human GRCh	OTTHUMG000001	PNKP	**polynucleotide kinase 3'-phosphatase**	Nucleus	kinase		1.05796176	0	0.78364653	0	1	0	11.4276063	11.6685858	11.7322532	11.4904831
559	**ENSG0000003998**	BEST2	protein_coding	chr19	12751702	12758458	+	Human GRCh	OTTHUMG000001	BEST2	**bestrophin 2**	Plasma Mem	ion channel		1.401835975	0	0.75714741	0	NA	0	1.9561782	2.01933418	1.65484023	2.37212714
560	**ENSG0000004019**	PHLPP2	protein_coding	chr16	71637835	71724701	-	Human GRCh	OTTHUMG000001	PHLPP2	**PH domain and leucine rich repeat protein phosphatase 2**	Cytoplasm	enzyme		1.033644327	0	0.8803744	0	1	0	9.61376708	9.96789881	10.0051356	9.64951678
561	**ENSG0000004027**	SPDL1	protein_coding	chr5	169583636	169604778	+	Human GRCh	OTTHUMG000001	SPDL1	**spindle apparatus coiled-coil protein 1**	Nucleus	other		-1.120408074	0	0.57294201	0	1	0	10.0892295	10.3153382	10.1954123	9.95825277
562	**ENSG0000004034**	STAU2	protein_coding	chr8	73420369	73747708	-	Human GRCh	OTTHUMG000001	STAU2	**staufen double-stranded RNA binding protein 2**	Cytoplasm	other		-1.080510325	0	0.72799625	0	1	0	10.5662995	10.7926241	10.723261	10.4672162
563	**ENSG0000004048**	SLC66A1	protein_coding	chr1	19312326	19329300	+	Human GRCh	OTTHUMG000000	SLC66A1	**solute carrier family 66 member 1**	Cytoplasm	transporter		-1.09758293	0	0.68673589	0	1	0	9.1494479	9.17787958	9.18806987	8.9215714
564	**ENSG0000004053**	CTNS	protein_coding	chr17	3636459	3663103	+	Human GRCh	OTTHUMG000000	CTNS	**cystinosin, lysosomal cystine transporter**	Cytoplasm	transporter		1.016794096	0	0.94014614	0	1	0	9.12133884	9.52849698	9.51402207	9.18494143
565	**ENSG0000004060**	RTN4R	protein_coding	chr22	20241415	20283246	-	Human GRCh	OTTHUMG000001	RTN4R	**reticulon 4 receptor**	Plasma Mem	transmembra		1.251107443	0	0.42614306	0	1	0	6.63492725	7.11136171	7.01647332	7.22475514
566	**ENSG0000004063**	PHF23	protein_coding	chr17	7235029	7239722	-	Human GRCh	OTTHUMG000001	PHF23	**PHD finger protein 23**	Nucleus	other		-1.035795993	0	0.86641586	0	1	0	10.3567066	10.687276	10.6524413	10.3109554
568	**ENSG0000004093**	INPP4A	protein_coding	chr2	98444854	98594392	+	Human GRCh	OTTHUMG000001	INPP4A	**inositol polyphosphate-4-phosphatase type I A**	Cytoplasm	phosphatase		-1.094252947	0	0.65344895	0	1	0	10.303421	10.4295602	10.4020529	10.1252922
569	**ENSG0000004135**	RAB27B	protein_coding	chr18	54717860	54895516	+	Human GRCh	OTTHUMG000001	RAB27B	**RAB27B, member RAS oncogene family**	Cytoplasm	enzyme		-1.4127429	0	0.73365886	0	1	0	2.76933198	3.26541023	2.64021553	2.96335935
570	**ENSG0000004135**	PSMA4	protein_coding	chr15	78540405	78552417	+	Human GRCh	OTTHUMG000001	PSMA4	**proteasome 20S subunit alpha 4**	Cytoplasm	peptidase		-1.11312718	0	0.59955905	0	1	0	11.06253	11.4303613	11.2070552	11.0704138
571	**ENSG0000004151**	MYO16	protein_coding	chr13	108596152	109208005	+	Human GRCh	OTTHUMG000000	MYO16	**myosin XVI**	Nucleus	other		-1.428974232	0	0.479602	0	1	0	7.06943055	7.35923809	6.87403187	6.74758287
572	**ENSG0000004180**	LSG1	protein_coding	chr3	194640791	194672463	-	Human GRCh	OTTHUMG000001	LSG1	**large 60S subunit nuclear export GTPase 1**	Cytoplasm	enzyme		-1.024784484	0	0.89719477	0	1	0	10.5679413	10.947063	10.8861117	10.5866591
573	**ENSG0000004188**	PARP3	protein_coding	chr3	51942345	51948847	+	Human GRCh	OTTHUMG000001	PARP3	**poly(ADP-ribose) polymerase family member 3**	Nucleus	enzyme	rucaparib,	-1.241261894	0	0.353302	0	1	0	7.93524793	8.42356585	8.08496247	7.85282623
574	**ENSG0000004198**	TNC	protein_coding	chr9	115019575	115118207	-	Human GRCh	OTTHUMG000000	TNC	**tenascin C**	Extracellular	other	F16-IL2 fus	-3.473202713	-1	0.18516109	0	1	0	6.98594632	6.70990744	5.57749986	6.11373566
575	**ENSG0000004198**	THAP3	protein_coding	chr1	6624868	6635586	+	Human GRCh	OTTHUMG000000	THAP3	**THAP domain containing 3**	Nucleus	transcription		-1.007920459	0	0.97086221	0	1	0	8.58725827	8.83619203	8.85838682	8.53927838
576	**ENSG0000004206**	RIPOR3	protein_coding	chr20	50586108	50691542	-	Human GRCh	OTTHUMG000000	RIPOR3	**RIPOR family member 3**	Other	other		-1.031881789	0	0.96994636	0	1	0	2.41965389	2.55060965	1.90065807	2.80368195
577	**ENSG0000004208**	TDP1	protein_coding	chr14	89954939	90044764	+	Human GRCh	OTTHUMG000001	TDP1	**tyrosyl-DNA phosphodiesterase 1**	Nucleus	enzyme		-1.107834606	0	0.70012208	0	1	0	10.2528939	10.638309	10.4497177	10.2402379
578	**ENSG0000004228**	AIFM2	protein_coding	chr10	70098223	70132934	-	Human GRCh	OTTHUMG000000	AIFM2	**apoptosis inducing factor mitochondria associated 2**	Cytoplasm	enzyme		-1.322664453	0	0.39089649	0	1	0	5.77881605	6.34095537	5.92339121	5.71914213
579	**ENSG0000004231**	SPATA7	protein_coding	chr14	88384924	88470350	+	Human GRCh	OTTHUMG000001	SPATA7	**spermatogenesis associated 7**	Cytoplasm	other		1.16546392	0	0.53697167	0	1	0	7.71305359	8.00904225	8.06382628	7.99645484
580	**ENSG0000004242**	MED17	protein_coding	chr11	93784227	93814963	+	Human GRCh	OTTHUMG000001	MED17	**mediator complex subunit 17**	Nucleus	transcription		-1.159932553	0	0.50863775	0	1	0	10.4668861	10.7468162	10.6408035	10.2242137
581	**ENSG0000004244**	RETSAT	protein_coding	chr2	85341955	85354531	-	Human GRCh	OTTHUMG000001	RETSAT	**retinol saturase**	Cytoplasm	enzyme		-1.16739819	0	0.4589441	0	1	0	10.0617059	10.4428979	10.2187986	9.95043513
582	**ENSG0000004249**	CAPG	protein_coding	chr2	85394753	85418432	-	Human GRCh	OTTHUMG000001	CAPG	**capping actin protein, gelsolin like**	Nucleus	other		-1.057190084	0	0.88756946	0	1	0	7.05958955	7.61845286	7.51808216	7.05648983
583	**ENSG0000004275**	AP2S1	protein_coding	chr19	46838136	46850846	-	Human GRCh	OTTHUMG000001	AP2S1	**adaptor related protein complex 2 subunit sigma 1**	Cytoplasm	transporter		1.047755055	0	0.81458524	0	1	0	10.391822	10.7023704	10.6902358	10.5230437
584	**ENSG0000004278**	USH2A	protein_coding	chr1	215622891	216423448	-	Human GRCh	OTTHUMG000000	USH2A	**usherin**	Extracellular	other		-2.258134355	-1	0.20171725	0	1	0	3.69136073	4.54178775	3.58229679	3.59385348
586	**ENSG0000004283**	TG	protein_coding	chr8	132866958	133134903	+	Human GRCh	OTTHUMG000001	TG	**thyroglobulin**	Extracellular	other		1.322732545	0	0.75766506	0	1	0	2.85841403	3.38159795	3.10113794	3.62633488
587	**ENSG0000004298**	ADAM28	protein_coding	chr8	24294069	24359014	+	Human GRCh	OTTHUMG000001	ADAM28	**ADAM metallopeptidase domain 28**	Plasma Mem	peptidase		1.136043296	0	0.9026718	0	1	0	3.45965942	3.46403216	3.52936174	3.62169296
588	**ENSG0000004303**	BARX2	protein_coding	chr11	129375848	129452279	+	Human GRCh	OTTHUMG000001	BARX2	**BARX homeobox 2**	Nucleus	transcription		1.041523833	0	0.94133405	0	1	0	4.0731954	3.70359844	4.36976735	3.28011143
589	**ENSG0000004309**	DCUN1D1	protein_coding	chr3	182938074	182985953	-	Human GRCh	OTTHUMG000001	DCUN1D1	**defective in cullin neddylation 1 domain containing 1**	Nucleus	other		1.010306546	0	0.96188545	0	1	0	10.3555505	10.5522081	10.6446449	10.2639403
590	**ENSG0000004314**	JADE2	protein_coding	chr5	134524312	134583230	+	Human GRCh	OTTHUMG000001	JADE2	**jade family PHD finger 2**	Nucleus	enzyme		-1.069241383	0	0.77709822	0	1	0	10.1984729	10.6937244	10.4827013	10.3062615
591	**ENSG0000004335**	ZIC2	protein_coding	chr13	99981784	99986765	+	Human GRCh	OTTHUMG000000	ZIC2	**Zic family member 2**	Nucleus	transcription		1.060561097	0	0.85044446	0	1	0	9.62292476	9.72283133	9.77006954	9.70319439
593	**ENSG0000004351**	TRIT1	protein_coding	chr1	39838110	39883511	-	Human GRCh	OTTHUMG000000	TRIT1	**tRNA isopentenyltransferase 1**	Cytoplasm	enzyme		-1.066648218	0	0.74873562	0	1	0	9.98554526	10.3453421	10.222521	9.98412611
594	**ENSG0000004359**	ADRB1	protein_coding	chr10	114043866	114046904	+	Human GRCh	OTTHUMG000000	ADRB1	**adrenoceptor beta 1**	Plasma Mem	G-protein co	KUL 7211,	2.544757505	1	0.29963487	0	1	0	5.06283286	5.38178081	6.1440494	6.36516755
596	**ENSG0000004409**	CUL7	protein_coding	chr6	43037617	43053943	-	Human GRCh	OTTHUMG000000	CUL7	**cullin 7**	Cytoplasm	enzyme		-1.191032475	0	0.45743929	0	1	0	11.3012438	11.4121286	11.3648775	10.9144254
597	**ENSG0000004411**	CTNNA1	protein_coding	chr5	138610967	138935034	+	Human GRCh	OTTHUMG000001	CTNNA1	**catenin alpha 1**	Plasma Mem	other		-1.170220327	0	0.47272722	0	1	0	13.0915988	13.4132688	13.228401	12.9286465
598	**ENSG0000004444**	PHKA2	protein_coding	chrX	18892298	18984114	-	Human GRCh	OTTHUMG000000	PHKA2	**phosphorylase kinase regulatory subunit alpha 2**	Cytoplasm	kinase	K-252	-1.169216001	0	0.54425938	0	1	0	10.0144451	10.4616348	10.2082399	9.95871383
599	**ENSG0000004445**	CNTLN	protein_coding	chr9	17134982	17503923	+	Human GRCh	OTTHUMG000000	CNTLN	**centlein**	Cytoplasm	other		1.127707177	0	0.67463951	0	1	0	9.0269213	9.39430828	9.42334841	9.27254697
600	**ENSG0000004452**	EPHA3	protein_coding	chr3	89107621	89482134	+	Human GRCh	OTTHUMG000001	EPHA3	**EPH receptor A3**	Plasma Mem	kinase	anti-EphA3	1.056904065	0	0.95750298	0	1	0	5.74272721	6.24457052	5.84290253	6.28325995
601	**ENSG0000004457**	HSPA5	protein_coding	chr9	125234853	125241382	-	Human GRCh	OTTHUMG000000	HSPA5	**heat shock protein family A (Hsp70) member 5**	Cytoplasm	enzyme	SHetA2, PA	-1.180355535	0	0.46089525	0	1	0	13.9350341	14.2073487	13.9446065	13.8342665
602	**ENSG0000004660**	DSG2	protein_coding	chr18	31498177	31549008	+	Human GRCh	OTTHUMG000001	DSG2	**desmoglein 2**	Plasma Mem	other		-1.180552168	0	0.41291904	0	1	0	12.2069091	12.4173933	12.2630449	11.9848342
603	**ENSG0000004664**	GEMIN8	protein_coding	chrX	14006726	14029893	-	Human GRCh	OTTHUMG000000	GEMIN8	**gem nuclear organelle associated protein 8**	Cytoplasm	other		1.002720696	0	0.99052985	0	1	0	8.9249587	9.17564211	9.18552552	8.91909702
604	**ENSG0000004665**	OFD1	protein_coding	chrX	13734743	13777955	+	Human GRCh	OTTHUMG000000	OFD1	**OFD1 centriole and centriolar satellite protein**	Plasma Mem	other		-1.12388328	0	0.5710242	0	1	0	10.496762	10.8083553	10.6828209	10.3720916
605	**ENSG0000004665**	GPM6B	protein_coding	chrX	13770939	13938638	-	Human GRCh	OTTHUMG000000	GPM6B	**glycoprotein M6B**	Plasma Mem	other		-1.128307226	0	0.7252864	0	1	0	6.87658596	7.37641591	7.14128215	6.89827163
606	**ENSG0000004677**	MAGEC2	protein_coding	chrX	142202342	142205290	-	Human GRCh	OTTHUMG000000	MAGEC2	**MAGE family member C2**	Nucleus	other		2.044433713	1	0.50827097	0	1	0	2.18265527	1.62853179	2.2945242	2.19076106
607	**ENSG0000004688**	PREX2	protein_coding	chr8	67952046	68237032	+	Human GRCh	OTTHUMG000001	PREX2	**phosphatidylinositol-3,4,5-trisphosphate dependent Rac excha**	Cytoplasm	other		-2.71747181	-1	0.41347091	0	1	0	4.40078612	5.11887894	3.62119724	3.91616482
608	**ENSG0000004705**	WDR37	protein_coding	chr10	1049538	1160991	+	Human GRCh	OTTHUMG000000	WDR37	**WD repeat domain 37**	Other	other		-1.135164173	0	0.5859431	0	1	0	10.279734	10.6765167	10.4272306	10.2728686
609	**ENSG0000004718**	YTHDC2	protein_coding	chr5	113513694	113595285	+	Human GRCh	OTTHUMG000001	YTHDC2	**YTH N6-methyladenosine RNA binding protein C2**	Cytoplasm	enzyme		-1.021665704	0	0.91918214	0	1	0	10.6303949	10.9293493	10.9691814	10.5183564
610	**ENSG0000004723**	CTPS2	protein_coding	chrX	16587999	16712936	-	Human GRCh	OTTHUMG000000	CTPS2	**CTP synthase 2**	Cytoplasm	enzyme		-1.107286259	0	0.66303302	0	1	0	9.68075752	10.0659447	9.97372224	9.54458374
611	**ENSG0000004724**	ATP6V1H	protein_coding	chr8	53715543	53843558	-	Human GRCh	OTTHUMG000001	ATP6V1H	**ATPase H+ transporting V1 subunit H**	Cytoplasm	transporter		-1.209700935	0	0.38042078	0	1	0	10.2079033	10.5076151	10.3357818	9.95399532
612	**ENSG0000004731**	POLR2B	protein_coding	chr4	56977722	57031158	+	Human GRCh	OTTHUMG000001	POLR2B	**RNA polymerase II subunit B**	Nucleus	enzyme		-1.182410151	0	0.4254787	0	1	0	11.8443773	12.2841666	12.0273513	11.7498233
613	**ENSG0000004734**	ATOSA	protein_coding	chr15	52581317	52709817	-	Human GRCh	OTTHUMG000001	ATOSA	**atos homolog A**	Other	other		-1.283357173	0	0.21607836	0	1	0	10.8368249	10.9640352	10.6233985	10.612294
614	**ENSG0000004736**	ARAP2	protein_coding	chr4	35948221	36244514	-	Human GRCh	OTTHUMG000000	ARAP2	**ArfGAP with RhoGAP domain, ankyrin repeat and PH domain 2**	Cytoplasm	other		-1.067506429	0	0.78608226	0	1	0	8.889155	9.32644978	9.03130131	9.0792979
615	**ENSG0000004741**	TPR	protein_coding	chr1	186311652	186375693	-	Human GRCh	OTTHUMG000000	TPR	**translocated promoter region, nuclear basket protein**	Nucleus	other		-1.141000353	0	0.46566669	0	1	0	13.1496498	13.4279046	13.2349598	13.0590241
616	**ENSG0000004745**	CP	protein_coding	chr3	149162410	149221829	-	Human GRCh	OTTHUMG000001	CP	**ceruloplasmin**	Extracellular	enzyme	copper	-1.926027435	-1	0.73945447	0	NA	0	1.1859447	0.80293532	0.7917989	0.73877074
617	**ENSG0000004757**	KATNIP	protein_coding	chr16	27550133	27780344	+	Human GRCh	OTTHUMG000001	KATNIP	**katanin interacting protein**	Extracellular	other		-1.096773556	0	0.69179838	0	1	0	10.6057786	10.7161602	10.775581	10.2957706
618	**ENSG0000004757**	DTNBP1	protein_coding	chr6	15522807	15663058	-	Human GRCh	OTTHUMG000000	DTNBP1	**dystrobrevin binding protein 1**	Plasma Mem	other		-1.096263811	0	0.68652115	0	1	0	8.37080113	8.73982486	8.60497182	8.31816289
619	**ENSG0000004759**	XK	protein_coding	chrX	37685791	37732130	+	Human GRCh	OTTHUMG000000	XK	**X-linked Kx blood group antigen, Kell and VPS13A binding prot**	Plasma Mem	other	zanamivir	-1.297028843	0	0.53151959	0	1	0	6.60937729	7.00938349	6.36876214	6.72843089
620	**ENSG0000004761**	ANO2	protein_coding	chr12	5531869	5946232	-	Human GRCh	OTTHUMG000001	ANO2	**anoctamin 2**	Plasma Mem	ion channel		-1.007074553	0	0.98644821	0	1	0	6.15751729	5.97138572	6.23953076	5.85241158
621	**ENSG0000004762**	C12orf4	protein_coding	chr12	4487735	4538508	-	Human GRCh	OTTHUMG000001	C12orf4	**chromosome 12 open reading frame 4**	Cytoplasm	other		1.031419827	0	0.89272526	0	1	0	9.30951935	9.45134759	9.62321501	9.16617191
622	**ENSG0000004763**	SCML1	protein_coding	chrX	17737449	17754988	+	Human GRCh	OTTHUMG000000	SCML1	**Scm polycomb group protein like 1**	Nucleus	transcription		1.014049134	0	0.95493457	0	1	0	10.2989747	10.6096176	10.5626428	10.3914112
623	**ENSG0000004764**	WWC3	protein_coding	chrX	10015254	10144474	+	Human GRCh	OTTHUMG000000	WWC3	**WWC family member 3**	Cytoplasm	other		1.053727585	0	0.81272313	0	1	0	9.23526448	9.44299391	9.47339914	9.31963705
624	**ENSG0000004764**	ARHGAP6	protein_coding	chrX	11117651	11665920	-	Human GRCh	OTTHUMG000000	ARHGAP6	**Rho GTPase activating protein 6**	Cytoplasm	other		-1.034828235	0	0.9599939	0	1	0	8.01352563	8.25408374	8.31324086	7.84111633
625	**ENSG0000004766**	FAM184B	protein_coding	chr4	17629306	17781621	-	Human GRCh	OTTHUMG000001	FAM184B	**family with sequence similarity 184 member B**	Other	other		1.096180778	0	0.90420976	0	1	0	3.48953875	3.04030904	2.21692833	3.86421932
626	**ENSG0000004784**	MAP4	protein_coding	chr3	47850690	48089272	-	Human GRCh	OTTHUMG000001	MAP4	**microtubule associated protein 4**	Cytoplasm	other		-1.110856322	0	0.54725329	0	1	0	13.2010946	13.4905787	13.303188	13.1642282
627	**ENSG0000004793**	GOPC	protein_coding	chr6	117560269	117602542	-	Human GRCh	OTTHUMG000000	GOPC	**golgi associated PDZ and coiled-coil motif containing**	Cytoplasm	transporter		1.065599303	0	0.76925315	0	1	0	10.0187705	10.2701608	10.41471	9.98817695
629	**ENSG0000004802**	USP28	protein_coding	chr11	113797874	113875572	-	Human GRCh	OTTHUMG000001	USP28	**ubiquitin specific peptidase 28**	Nucleus	peptidase		-1.12777168	0	0.61172276	0	1	0	9.80367243	10.3095813	10.0585008	9.834276

630	**ENSG0000004805**	HDAC9	protein_coding	chr7	18086949	19002416	+	Human GRCh	OTTHUMG000001	HDAC9	**histone deacetylase 9**	Nucleus	transcription	tributyrin,	2.043373184	1	0.2147378	0	1	0	5.01029865	5.17572884	5.09132502	6.02479231
631	**ENSG0000004814**	TSPAN17	protein_coding	chr5	176647387	176659054	+	Human GRCh	OTTHUMG000001	TSPAN17	**tetraspanin 17**	Other	enzyme		-1.046659731	0	0.85809014	0	1	0	9.94137098	10.1550715	10.1667828	9.81162401
632	**ENSG0000004816**	NOP16	protein_coding	chr5	176383946	176388927	-	Human GRCh	OTTHUMG000001	NOP16	**NOP16 nucleolar protein**	Nucleus	other		1.106493006	0	0.62847127	0	1	0	9.29255157	9.43860096	9.64671486	9.29232384
633	**ENSG0000004834**	CC2D2A	protein_coding	chr4	15469865	15601552	+	Human GRCh	OTTHUMG000001	CC2D2A	**coiled-coil and C2 domain containing 2A**	Cytoplasm	other		1.153047902	0	0.59021862	0	1	0	7.13967162	7.27548948	7.49008714	7.208472
634	**ENSG0000004839**	RRM2B	protein_coding	chr8	102204502	102238961	-	Human GRCh	OTTHUMG000001	RRM2B	**ribonucleotide reductase regulatory TP53 inducible subunit M**	Nucleus	enzyme	cladribine/	1.111658243	0	0.63753429	0	1	0	9.38672659	9.75279641	9.8978243	9.45881493
635	**ENSG0000004840**	ZNF800	protein_coding	chr7	127346790	127431924	-	Human GRCh	OTTHUMG000000	ZNF800	**zinc finger protein 800**	Other	other		1.027785127	0	0.90326595	0	1	0	10.1358262	10.4585106	10.5212318	10.1229236
637	**ENSG0000004847**	SNX29	protein_coding	chr16	11976734	12574287	+	Human GRCh	OTTHUMG000001	SNX29	**sorting nexin 29**	Cytoplasm	other		-1.093210549	0	0.70866818	0	1	0	9.34370928	9.60978848	9.5388309	9.21392518
638	**ENSG0000004854**	LMO3	protein_coding	chr12	16548372	16610594	-	Human GRCh	OTTHUMG000001	LMO3	**LIM domain only 3**	Nucleus	other		1.308434161	0	0.62409652	0	1	0	5.45017969	5.69552643	5.88992052	5.65433447
639	**ENSG0000004854**	MRPS10	protein_coding	chr6	42206807	42217861	-	Human GRCh	OTTHUMG000000	MRPS10	**mitochondrial ribosomal protein S10**	Cytoplasm	other		1.015442804	0	0.942186	0	1	0	9.8341895	10.1149176	10.0794453	9.9139286
641	**ENSG0000004864**	RSF1	protein_coding	chr11	77660009	77820869	-	Human GRCh	OTTHUMG000001	RSF1	**remodeling and spacing factor 1**	Nucleus	transcription		-1.08786092	0	0.68477484	0	1	0	11.7582115	12.1118956	12.0039481	11.6844298
642	**ENSG0000004870**	VPS13D	protein_coding	chr1	12230030	12512047	+	Human GRCh	OTTHUMG000000	VPS13D	**vacuolar protein sorting 13 homolog D**	Cytoplasm	other		-1.223580915	0	0.33661161	0	1	0	12.1170803	12.5399742	12.2375948	11.9996131
643	**ENSG0000004874**	CELF2	protein_coding	chr10	10798397	11336675	+	Human GRCh	OTTHUMG000000	CELF2	**CUGBP Elav-like family member 2**	Nucleus	other		5.925103738	1	0.00103509	1	0.63	0	5.48970117	4.87928171	6.24520318	7.74174287
644	**ENSG0000004882**	FAM120A	protein_coding	chr9	93451685	93566112	+	Human GRCh	OTTHUMG000000	FAM120A	**family with sequence similarity 120 member A**	Cytoplasm	other		-1.044893024	0	0.8134324	0	1	0	12.7952913	13.0104508	13.0113888	12.6831369
645	**ENSG0000004899**	R3HDM1	protein_coding	chr2	135531455	135725270	+	Human GRCh	OTTHUMG000001	R3HDM1	**R3H domain containing 1**	Other	other		-1.017965756	0	0.92795054	0	1	0	10.2085949	10.5540212	10.4387173	10.3057645
646	**ENSG0000004908**	COL9A2	protein_coding	chr1	40300489	40317813	-	Human GRCh	OTTHUMG000000	COL9A2	**collagen type IX alpha 2 chain**	Extracellular	other	collagenas	-1.284469731	0	0.75844768	0	1	0	3.32716053	2.11881939	2.64629587	2.88981492
647	**ENSG0000004913**	KITLG	protein_coding	chr12	88492793	88580851	-	Human GRCh	OTTHUMG000001	KITLG	**KIT ligand**	Extracellular	growth facto		-1.126962204	0	0.66468633	0	1	0	9.97744774	10.078619	9.88995056	9.90909029
648	**ENSG0000004916**	ERCC8	protein_coding	chr5	60866454	60945073	-	Human GRCh	OTTHUMG000000	ERCC8	**ERCC excision repair 8, CSA ubiquitin ligase complex subunit**	Nucleus	transcription		-1.059486789	0	0.79560582	0	1	0	10.592567	10.7960379	10.8496088	10.3721279
649	**ENSG0000004919**	ADAMTS6	protein_coding	chr5	65148738	65481920	-	Human GRCh	OTTHUMG000000	ADAMTS6	**ADAM metallopeptidase with thrombospondin type 1 motif 6**	Extracellular	peptidase		-1.138992124	0	0.80573897	0	1	0	5.21461725	5.74966472	5.37635338	5.40635997
650	**ENSG0000004923**	H6PD	protein_coding	chr1	9234774	9271337	+	Human GRCh	OTTHUMG000000	H6PD	**hexose-6-phosphate dehydrogenase/glucose 1-dehydrogenase**	Cytoplasm	enzyme		-1.367709708	0	0.1356623	0	1	0	10.2919435	10.5065209	10.1492979	9.97617505
651	**ENSG0000004924**	VAMP3	protein_coding	chr1	7771296	7781432	+	Human GRCh	OTTHUMG000000	VAMP3	**vesicle associated membrane protein 3**	Plasma Mem	other		-1.043361981	0	0.85040717	0	1	0	10.4281172	10.7553357	10.7649497	10.3013457
652	**ENSG0000004924**	PER3	protein_coding	chr1	7784291	7845177	+	Human GRCh	OTTHUMG000000	PER3	**period circadian regulator 3**	Nucleus	other		1.286913649	0	0.46575414	0	1	0	7.81296508	8.18865255	8.67871638	7.65332335
653	**ENSG0000004924**	UTS2	protein_coding	chr1	7843083	7853512	-	Human GRCh	OTTHUMG000000	UTS2	**urotensin 2**	Extracellular	other		1	0	1	0	NA	0	-0.3107019	-0.3044221	-0.3054643	-0.3103353
654	**ENSG0000004924**	TNFRSF9	protein_coding	chr1	7915871	7943165	-	Human GRCh	OTTHUMG000000	TNFRSF9	**TNF receptor superfamily member 9**	Plasma Mem	transmembra	PRS-344/S	1.369837123	0	0.80805324	0	NA	0	1.81457432	1.18916733	1.48971874	1.82089893
655	**ENSG0000004928**	EPN3	protein_coding	chr17	50532682	50543750	+	Human GRCh	OTTHUMG000001	EPN3	**epsin 3**	Cytoplasm	other		1.086029664	0	0.78808121	0	1	0	8.1525443	8.42928398	8.31916182	8.46023642
656	**ENSG0000004932**	LTBP1	protein_coding	chr2	32946953	33399509	+	Human GRCh	OTTHUMG000001	LTBP1	**latent transforming growth factor beta binding protein 1**	Extracellular	other		-1.065338364	0	0.80214837	0	1	0	9.77464576	9.95350608	9.91388666	9.66013258
657	**ENSG0000004944**	RCN1	protein_coding	chr11	32091074	32105722	+	Human GRCh	OTTHUMG000001	RCN1	**reticulocalbin 1**	Cytoplasm	other		-1.374565508	0	0.12478322	0	1	0	10.5600379	10.580233	10.4637039	9.91875588
658	**ENSG0000004954**	ELN	protein_coding	chr7	74027789	74069907	+	Human GRCh	OTTHUMG000001	ELN	**elastin**	Extracellular	other		-2.755615175	-1	0.492555	0	1	0	1.5058193	1.99398884	1.57622654	1.51090342
659	**ENSG0000004954**	RFC2	protein_coding	chr7	74231499	74254458	-	Human GRCh	OTTHUMG000000	RFC2	**replication factor C subunit 2**	Nucleus	enzyme		-1.110602156	0	0.64026952	0	1	0	10.6306167	10.9415921	10.7440956	10.6161191
660	**ENSG0000004961**	ARID1B	protein_coding	chr6	156776020	157210779	+	Human GRCh	OTTHUMG000000	ARID1B	**AT-rich interaction domain 1B**	Nucleus	transcription		-1.094039803	0	0.6585391	0	1	0	12.7029072	12.8804923	12.8452306	12.5303999
661	**ENSG0000004965**	CLPTM1L	protein_coding	chr5	1317752	1345099	-	Human GRCh	OTTHUMG000001	CLPTM1L	**CLPTM1 like**	Cytoplasm	transporter		-1.132224674	0	0.53373687	0	1	0	11.7042437	11.9197031	11.7984815	11.5465476
662	**ENSG0000004975**	NEDD4L	protein_coding	chr18	58044226	58401540	+	Human GRCh	OTTHUMG000001	NEDD4L	**NEDD4 like E3 ubiquitin protein ligase**	Cytoplasm	enzyme		1.089137326	0	0.75737443	0	1	0	12.0742607	12.2143063	12.4820255	11.9461776
663	**ENSG0000004976**	FOXP3	protein_coding	chrX	49250438	49264800	-	Human GRCh	OTTHUMG000000	FOXP3	**forkhead box P3**	Nucleus	transcription	AZD8701	1.508450322	1	0.6216039	0	1	0	2.6992445	3.18152731	3.16054186	3.2603419
664	**ENSG0000004976**	PPP1R3F	protein_coding	chrX	49269793	49301461	+	Human GRCh	OTTHUMG000000	PPP1R3F	**protein phosphatase 1 regulatory subunit 3F**	Other	other		1.117480499	0	0.72425496	0	1	0	6.9848327	6.88796424	7.30334146	6.73141618
665	**ENSG0000004986**	HEXB	protein_coding	chr5	74640023	74722647	+	Human GRCh	OTTHUMG000001	HEXB	**hexosaminidase subunit beta**	Cytoplasm	enzyme		1.006706932	0	0.97974292	0	1	0	9.31408053	9.48804155	9.53778534	9.27010882
666	**ENSG0000004988**	PTCD2	protein_coding	chr5	72320367	72368395	+	Human GRCh	OTTHUMG000001	PTCD2	**pentatricopeptide repeat domain 2**	Cytoplasm	other		1.043804359	0	0.84963387	0	1	0	9.98576121	10.2756129	10.3998103	9.92952147
667	**ENSG0000005003**	NEXMIF	protein_coding	chrX	74732856	74925472	-	Human GRCh	OTTHUMG000000	NEXMIF	**neurite extension and migration factor**	Nucleus	enzyme		-1.100919917	0	0.80154301	0	1	0	7.58114253	8.19455657	7.93831452	7.70572234
668	**ENSG0000005013**	JKAMP	protein_coding	chr14	59484443	59505410	+	Human GRCh	OTTHUMG000001	JKAMP	**JNK1/MAPK8 associated membrane protein**	Cytoplasm	other		-1.021754139	0	0.92042188	0	1	0	9.29170397	9.438557	9.53962229	9.10942955
669	**ENSG0000005016**	DKK3	protein_coding	chr11	11956207	12009769	-	Human GRCh	OTTHUMG000001	DKK3	**dickkopf WNT signaling pathway inhibitor 3**	Extracellular	cytokine		2.933691281	1	0.29791282	0	1	0	2.33814209	1.86164299	1.85166135	2.95591543
670	**ENSG0000005032**	ARHGEF5	protein_coding	chr7	144355288	144380632	+	Human GRCh	OTTHUMG000001	ARHGEF5	**Rho guanine nucleotide exchange factor 5**	Cytoplasm	other		-1.235697317	0	0.32939822	0	1	0	10.3979169	10.6830834	10.3791121	10.218481
671	**ENSG0000005034**	NFE2L3	protein_coding	chr7	26152198	26187137	+	Human GRCh	OTTHUMG000000	NFE2L3	**NFE2 like bZIP transcription factor 3**	Nucleus	transcription		1.366826456	0	0.26595784	0	1	0	7.37877944	7.37831392	7.811884	7.58236694
672	**ENSG0000005039**	MCUR1	protein_coding	chr6	13786557	13814568	-	Human GRCh	OTTHUMG000000	MCUR1	**mitochondrial calcium uniporter regulator 1**	Cytoplasm	other		-1.190824466	0	0.3963097	0	1	0	10.2371526	10.5686451	10.3283803	10.1073277
673	**ENSG0000005040**	LIMA1	protein_coding	chr12	50175788	50283520	-	Human GRCh	OTTHUMG000001	LIMA1	**LIM domain and actin binding 1**	Cytoplasm	other		-1.239854866	0	0.35538838	0	1	0	9.36310331	9.77955169	9.5612554	9.10232159
674	**ENSG0000005042**	LETMD1	protein_coding	chr12	51047962	51060424	+	Human GRCh	OTTHUMG000001	LETMD1	**LETM1 domain containing 1**	Plasma Mem	other		-1.116415359	0	0.63357594	0	1	0	11.1156241	11.4054874	11.3180403	10.9440951
675	**ENSG0000005043**	SLC4A8	protein_coding	chr12	51391317	51515763	+	Human GRCh	OTTHUMG000001	SLC4A8	**solute carrier family 4 member 8**	Plasma Mem	transporter		-1.313242647	0	0.45996224	0	1	0	6.88265553	6.97252947	7.01013809	6.19142415
676	**ENSG0000005055**	LAMC3	protein_coding	chr9	131009174	131094473	+	Human GRCh	OTTHUMG000000	LAMC3	**laminin subunit gamma 3**	Extracellular	other		1.339081335	0	0.483601	0	1	0	6.23240316	6.45123339	6.78377503	6.54194606
677	**ENSG0000005062**	PTGER3	protein_coding	chr1	70852353	71047816	-	Human GRCh	OTTHUMG000000	PTGER3	**prostaglandin E receptor 3**	Plasma Mem	G-protein co	misoprost	1.890868397	1	0.44009978	0	1	0	3.07435334	3.13265271	3.38442601	3.6837023
679	**ENSG0000005074**	MAPK9	protein_coding	chr5	180233143	180292099	-	Human GRCh	OTTHUMG000001	MAPK9	**mitogen-activated protein kinase 9**	Cytoplasm	kinase	SP600125,	-1.122313073	0	0.59021583	0	1	0	10.6534166	10.9483182	10.7728798	10.5846984
680	**ENSG0000005076**	COL23A1	protein_coding	chr5	178237476	178590393	-	Human GRCh	OTTHUMG000001	COL23A1	**collagen type XXIII alpha 1 chain**	Plasma Mem	other	collagenas	-1.285691992	0	0.6911481	0	1	0	4.02000642	4.44072697	4.12315434	3.93901475
681	**ENSG0000005082**	BCAR1	protein_coding	chr16	75228181	75268053	-	Human GRCh	OTTHUMG000001	BCAR1	**BCAR1 scaffold protein, Cas family member**	Cytoplasm	other		1.185041411	0	0.38213417	0	1	0	10.8441081	11.0282945	11.1964028	11.0589603
682	**ENSG0000005100**	FHIP1B	protein_coding	chr11	6211345	6234711	-	Human GRCh	OTTHUMG000001	FHIP1B	**FHF complex subunit HOOK interacting protein 1B**	Cytoplasm	other		-1.008862638	0	0.96949014	0	1	0	9.95125776	10.2407712	10.3003473	9.84613903
683	**ENSG0000005110**	HERPUD1	protein_coding	chr16	56932142	56944864	+	Human GRCh	OTTHUMG000001	HERPUD1	**homocysteine inducible ER protein with ubiquitin like domain**	Cytoplasm	other		-1.060558519	0	0.79328789	0	1	0	11.8942563	12.0013778	11.963002	11.7939715
684	**ENSG0000005112**	HOMER3	protein_coding	chr19	18929201	18941261	-	Human GRCh	OTTHUMG000001	HOMER3	**homer scaffold protein 3**	Plasma Mem	other		-1.078137705	0	0.80763336	0	1	0	8.63026507	8.80053676	8.77838257	8.47367534
685	**ENSG0000005118**	RAD51	protein_coding	chr15	40694774	40732340	+	Human GRCh	OTTHUMG000001	RAD51	**RAD51 recombinase**	Nucleus	enzyme	CYT-0851	1.030574376	0	0.89834503	0	1	0	8.7703016	8.87402997	8.98787288	8.71013252
686	**ENSG0000005134**	POLQ	protein_coding	chr3	121431431	121545988	-	Human GRCh	OTTHUMG000001	POLQ	**DNA polymerase theta**	Nucleus	enzyme	ART4215	-1.148541758	0	0.50589169	0	1	0	11.1055241	11.5469648	11.3307846	11.0370098
687	**ENSG0000005138**	PIK3CB	protein_coding	chr3	138652698	138834928	-	Human GRCh	OTTHUMG000001	PIK3CB	**phosphatidylinositol-4,5-bisphosphate 3-kinase catalytic subu**	Cytoplasm	kinase	TGX-221, /	1.034659113	0	0.86702196	0	1	0	10.1461664	10.45612	10.4807147	10.1994493
688	**ENSG0000005152**	CYBA	protein_coding	chr16	88643275	88651083	-	Human GRCh	OTTHUMG000001	CYBA	**cytochrome b-245 alpha chain**	Cytoplasm	enzyme		-1.348650211	0	0.63768009	0	1	0	7.16432923	7.68658761	7.27452884	6.93613084
689	**ENSG0000005159**	THOC3	protein_coding	chr5	175917873	176034680	-	Human GRCh	OTTHUMG000001	THOC3	**THO complex subunit 3**	Nucleus	other		1.124372919	0	0.57762202	0	1	0	9.39235153	9.6553305	9.84323929	9.44707875
690	**ENSG0000005163**	HEBP2	protein_coding	chr6	138403531	138422197	+	Human GRCh	OTTHUMG000000	HEBP2	**heme binding protein 2**	Cytoplasm	other		-1.224663781	0	0.40903591	0	1	0	11.2974668	11.5387593	11.2651737	11.1263515
691	**ENSG0000005182**	MPHOSPH	protein_coding	chr12	123152320	123244014	-	Human GRCh	OTTHUMG000001	MPHOSPH9	**M-phase phosphoprotein 9**	Cytoplasm	other		-1.157094582	0	0.50323091	0	1	0	11.9361356	12.2795737	12.1258713	11.7677958
692	**ENSG0000005212**	PLEKHA5	protein_coding	chr12	19129669	19376400	+	Human GRCh	OTTHUMG000001	PLEKHA5	**pleckstrin homology domain containing A5**	Cytoplasm	other		-1.271900718	0	0.26291338	0	1	0	11.181999	11.473941	11.1944717	10.9497152
693	**ENSG0000005234**	PRSS8	protein_coding	chr16	31131433	31135727	-	Human GRCh	OTTHUMG000001	PRSS8	**serine protease 8**	Extracellular	peptidase		-1.009358991	0	0.97194038	0	1	0	11.0463477	11.211226	11.1815615	11.0573411
694	**ENSG0000005272**	SIKE1	protein_coding	chr1	114769479	114780685	-	Human GRCh	OTTHUMG000000	SIKE1	**suppressor of IKBKE 1**	Cytoplasm	other		1.147476526	0	0.53481156	0	1	0	10.3395077	10.596087	10.8386473	10.3665539
695	**ENSG0000005274**	RRP12	protein_coding	chr10	97356358	97426076	-	Human GRCh	OTTHUMG000000	RRP12	**ribosomal RNA processing 12 homolog**	Nucleus	other		-1.007535202	0	0.96960057	0	1	0	11.4598003	11.6933521	11.6892016	11.4457061
696	**ENSG0000005279**	FNIP2	protein_coding	chr4	158769026	158908050	+	Human GRCh	OTTHUMG000001	FNIP2	**folliculin interacting protein 2**	Cytoplasm	other		1.05596437	0	0.80935035	0	1	0	10.3802803	10.5579261	10.5760606	10.4885515
697	**ENSG0000005280**	MSMO1	protein_coding	chr4	165327667	165343164	+	Human GRCh	OTTHUMG000001	MSMO1	**methylsterol monooxygenase 1**	Cytoplasm	enzyme		1.031866748	0	0.90212027	0	1	0	10.6338865	11.0767699	11.0475931	10.7582397
698	**ENSG0000005284**	TTC17	protein_coding	chr11	43358920	43494933	+	Human GRCh	OTTHUMG000001	TTC17	**tetratricopeptide repeat domain 17**	Cytoplasm	other		-1.092781391	0	0.63004485	0	1	0	11.5305758	11.7449924	11.6721707	11.4040526
699	**ENSG0000005285**	ALX4	protein_coding	chr11	44260440	44310139	-	Human GRCh	OTTHUMG000001	ALX4	**ALX homeobox 4**	Nucleus	transcription		-1.248896446	0	0.38488225	0	1	0	11.6406144	11.91662	11.8335511	11.1208586
700	**ENSG0000005310**	FSTL4	protein_coding	chr5	133196445	133612541	-	Human GRCh	OTTHUMG000001	FSTL4	**follistatin like 4**	Cytoplasm	other		-8.245169575	-1	0.07885665	0	1	0	3.02842003	3.37248614	2.84781939	2.77172473
701	**ENSG0000005325**	FOXN3	protein_coding	chr14	89124871	89619149	-	Human GRCh	OTTHUMG000001	FOXN3	**forkhead box N3**	Nucleus	transcription		-1.198920062	0	0.51988319	0	1	0	11.6725235	12.1460554	11.8733426	11.5699919
702	**ENSG0000005332**	METTL24	protein_coding	chr6	110243940	110358349	-	Human GRCh	OTTHUMG000000	METTL24	**methyltransferase like 24**	Other	other		-5.261974539	-1	0.2605388	0	1	0	2.19768574	1.81549844	1.81058335	1.7875867
703	**ENSG0000005337**	AKR7A2	protein_coding	chr1	19303965	19312144	-	Human GRCh	OTTHUMG000000	AKR7A2	**aldo-keto reductase family 7 member A2**	Cytoplasm	enzyme		-1.044649232	0	0.84925206	0	1	0	10.1098606	10.2883743	10.2720383	10.0231986
704	**ENSG0000005337**	MRTO4	protein_coding	chr1	19251805	19260128	+	Human GRCh	OTTHUMG000000	MRTO4	**MRT4 homolog, ribosome maturation factor**	Cytoplasm	other		-1.038413049	0	0.84498154	0	1	0	10.7241664	10.9390212	10.9540028	10.6094699
705	**ENSG0000005343**	NNAT	protein_coding	chr20	37521206	37523690	+	Human GRCh	OTTHUMG000000	NNAT	**neuronatin**	Plasma Mem	transporter		-5.878820065	-1	0.32533151	0	NA	0	0.3418641	1.00787684	0.36419047	0.34345073
706	**ENSG0000005350**	USE1	protein_coding	chr19	17215346	17219829	+	Human GRCh	OTTHUMG000001	USE1	**unconventional SNARE in the ER 1**	Cytoplasm	other		-1.149438465	0	0.59619459	0	1	0	8.17925509	8.64135704	8.31949635	8.23972913
707	**ENSG0000005352**	MCF2L2	protein_coding	chr3	183178041	183428778	-	Human GRCh	OTTHUMG000001	MCF2L2	**MCF.2 cell line derived transforming sequence-like 2**	Other	other		1.314940963	0	0.41347991	0	1	0	6.41096628	6.70641657	6.9074204	6.77683785
708	**ENSG0000005370**	NRIP2	protein_coding	chr12	2825348	2835544	-	Human GRCh	OTTHUMG000001	NRIP2	**nuclear receptor interacting protein 2**	Nucleus	transcription		1	0	1	0	NA	0	-0.006778	0.02041285	0.01601517	-0.0051434
709	**ENSG0000005374**	LAMA3	protein_coding	chr18	23689453	23956222	+	Human GRCh	OTTHUMG000001	LAMA3	**laminin subunit alpha 3**	Extracellular	other		1.394117972	0	0.3835915	0	1	0	6.66980201	6.50993124	6.71757264	7.06896396
710	**ENSG0000005377**	AP5M1	protein_coding	chr14	57268924	57298742	+	Human GRCh	OTTHUMG000001	AP5M1	**adaptor related protein complex 5 subunit mu 1**	Cytoplasm	other		-1.076573929	0	0.74271468	0	1	0	10.9842513	11.1888814	11.2095546	10.7685465
711	**ENSG0000005390**	ANAPC4	protein_coding	chr4	25377263	25418498	+	Human GRCh	OTTHUMG000000	ANAPC4	**anaphase promoting complex subunit 4**	Nucleus	enzyme		1.117334684	0	0.61630438	0	1	0	9.21759253	9.4715433	9.58506595	9.34348978
712	**ENSG0000005391**	KCNQ1	protein_coding	chr11	2444654	2849105	+	Human GRCh	OTTHUMG000000	KCNQ1	**potassium voltage-gated channel subfamily Q member 1**	Plasma Mem	ion channel	dextromet	-1.188998479	0	0.68682369	0	1	0	5.05861011	4.69005933	4.7497608	4.72063542
713	**ENSG0000005411**	TRAPPC3	protein_coding	chr1	36136570	36156053	-	Human GRCh	OTTHUMG000000	TRAPPC3	**trafficking protein particle complex subunit 3**	Cytoplasm	other	birabresib	-1.130072405	0	0.58502982	0	1	0	10.4115461	10.7961923	10.5875808	10.36871
714	**ENSG0000005411**	THRAP3	protein_coding	chr1	36224432	36305357	+	Human GRCh	OTTHUMG000000	THRAP3	**thyroid hormone receptor associated protein 3**	Nucleus	transcription		-1.232610893	0	0.35040091	0	1	0	13.4959128	13.6489516	13.4500707	13.2127181
715	**ENSG0000005414**	PHPT1	protein_coding	chr9	136848724	136851027	+	Human GRCh	OTTHUMG000000	PHPT1	**phosphohistidine phosphatase 1**	Cytoplasm	phosphatase		1.135568893	0	0.53951155	0	1	0	10.423153	10.7176842	10.7458863	10.6947548

716	**ENSG0000005417**	ENTPD2	protein_coding	chr9	137048107	137054061	-	Human GRCh	OTTHUMG000000	ENTPD2	**ectonucleoside triphosphate diphosphohydrolase 2**	Cytoplasm	enzyme		-1.017305808	0	0.95619448	0	1	0	6.00447996	6.42228834	6.45304037	5.91473911
717	**ENSG0000005421**	LY75	protein_coding	chr2	159803355	159904756	-	Human GRCh	OTTHUMG000001	LY75	**lymphocyte antigen 75**	Plasma Mem	transmembra	CDX 1401,	1	0	1	0	1	0	1.00121176	1.09401304	1.07922584	1.00689117
718	**ENSG0000005426**	ARID4B	protein_coding	chr1	235131634	235328219	-	Human GRCh	OTTHUMG000000	ARID4B	**AT-rich interaction domain 4B**	Nucleus	transcription		-1.020981533	0	0.91495425	0	1	0	11.312104	11.6154681	11.5581449	11.3321191
719	**ENSG0000005427**	OPN3	protein_coding	chr1	241590102	241677376	-	Human GRCh	OTTHUMG000000	OPN3	**opsin 3**	Plasma Mem	G-protein cou		-1.048001125	0	0.88614313	0	1	0	5.2658005	5.55622349	5.46366519	5.27792083
720	**ENSG0000005428**	SDCCAG8	protein_coding	chr1	243256034	243500091	+	Human GRCh	OTTHUMG000000	SDCCAG8	**SHH signaling and ciliogenesis regulator SDCCAG8**	Cytoplasm	other		-1.214602847	0	0.36173563	0	1	0	9.69017673	9.9664997	9.78195588	9.43701031
722	**ENSG0000005439**	HHAT	protein_coding	chr1	210328252	210676296	+	Human GRCh	OTTHUMG000000	HHAT	**hedgehog acyltransferase**	Cytoplasm	enzyme		-1.331574851	0	0.46164701	0	1	0	5.18316508	5.3306872	5.3413041	4.58361005
723	**ENSG0000005452**	KIF1B	protein_coding	chr1	10210570	10381603	+	Human GRCh	OTTHUMG000000	KIF1B	**kinesin family member 1B**	Cytoplasm	transporter		-1.060590738	0	0.76653421	0	1	0	12.2922802	12.4632395	12.453394	12.1599055
724	**ENSG0000005459**	FOXC1	protein_coding	chr6	1609915	1613897	+	Human GRCh	OTTHUMG000000	FOXC1	**forkhead box C1**	Nucleus	transcription		1.611877907	1	0.24576827	0	1	0	7.2185217	7.30298483	7.6569606	7.8437039
725	**ENSG0000005461**	TBC1D22A	protein_coding	chr22	46762617	47175699	+	Human GRCh	OTTHUMG000001	TBC1D22A	**TBC1 domain family member 22A**	Other	other		-1.091243028	0	0.67131397	0	1	0	10.0970295	10.3871887	10.2823074	10.012434
726	**ENSG0000005465**	SYNE2	protein_coding	chr14	63761899	64226433	+	Human GRCh	OTTHUMG000001	SYNE2	**spectrin repeat containing nuclear envelope protein 2**	Nucleus	other		-1.082882576	0	0.73006637	0	1	0	13.248799	13.4806181	13.4273777	13.1268065
727	**ENSG0000005469**	PLEKHH1	protein_coding	chr14	67533290	67589612	+	Human GRCh	OTTHUMG000001	PLEKHH1	**pleckstrin homology, MyTH4 and FERM domain containing H1**	Cytoplasm	other		-1.136517644	0	0.52554263	0	1	0	10.8567772	11.0880817	10.9654712	10.6855728
728	**ENSG0000005479**	ATP9A	protein_coding	chr20	51596514	51768390	-	Human GRCh	OTTHUMG000000	ATP9A	**ATPase phospholipid transporting 9A (putative)**	Cytoplasm	transporter		-1.18249185	0	0.51654193	0	1	0	10.8483324	11.1240425	10.9845356	10.5924688
731	**ENSG0000005493**	CHRDL2	protein_coding	chr11	74696429	74731426	-	Human GRCh	OTTHUMG000001	CHRDL2	**chordin like 2**	Cytoplasm	other		1	0	1	0	NA	0	-0.1764404	-0.1721189	-0.1728439	-0.1761912
732	**ENSG0000005496**	FAM168A	protein_coding	chr11	73400487	73598189	-	Human GRCh	OTTHUMG000001	FAM168A	**family with sequence similarity 168 member A**	Other	other		-1.061704249	0	0.7855702	0	1	0	10.9999531	11.2656999	11.2137497	10.9190531
733	**ENSG0000005496**	RELT	protein_coding	chr11	73376399	73397474	+	Human GRCh	OTTHUMG000001	RELT	**RELT TNF receptor**	Plasma Mem	transmembra		1.147863115	0	0.55150105	0	1	0	9.33014488	9.60189719	9.72514625	9.50702975
734	**ENSG0000005498**	GALC	protein_coding	chr14	87837820	87993665	-	Human GRCh	OTTHUMG000000	GALC	**galactosylceramidase**	Cytoplasm	enzyme		-1.046223446	0	0.8829074	0	1	0	8.25642854	8.55825987	8.47416325	8.25326097
735	**ENSG0000005504**	NOP58	protein_coding	chr2	202265736	202303661	+	Human GRCh	OTTHUMG000001	NOP58	**NOP58 ribonucleoprotein**	Nucleus	enzyme		-1.017669251	0	0.93124817	0	1	0	11.1984932	11.5413009	11.5055074	11.2001572
736	**ENSG0000005507**	SZRD1	protein_coding	chr1	16352575	16398145	+	Human GRCh	OTTHUMG000000	SZRD1	**SUZ RNA binding domain containing 1**	Other	other		-1.025328046	0	0.89430665	0	1	0	11.8049563	12.0610038	12.0279531	11.7836112
737	**ENSG0000005511**	KCNH2	protein_coding	chr7	150944961	150978321	-	Human GRCh	OTTHUMG000001	KCNH2	**potassium voltage-gated channel subfamily H member 2**	Plasma Mem	ion channel	dofetilide,	1.217644768	0	0.60318743	0	1	0	9.13425647	9.77505286	9.48266291	9.91655042
738	**ENSG0000005513**	CUL1	protein_coding	chr7	148697914	148801110	+	Human GRCh	OTTHUMG000001	CUL1	**cullin 1**	Nucleus	enzyme		-1.096005497	0	0.66351289	0	1	0	10.9810951	11.3704754	11.2189284	10.9401669
739	**ENSG0000005514**	FAM114A2	protein_coding	chr5	153990148	154038936	-	Human GRCh	OTTHUMG000001	FAM114A2	**family with sequence similarity 114 member A2**	Other	other		1.045126627	0	0.85633928	0	1	0	8.76522664	9.07965167	9.12612308	8.81584934
740	**ENSG0000005516**	CYFIP2	protein_coding	chr5	157266079	157395595	+	Human GRCh	OTTHUMG000001	CYFIP2	**cytoplasmic FMR1 interacting protein 2**	Cytoplasm	other		-1.127314601	0	0.67266147	0	1	0	9.3851197	9.5801644	9.43111323	9.27653822
741	**ENSG0000005520**	TAB2	protein_coding	chr6	149218641	149411613	+	Human GRCh	OTTHUMG000000	TAB2	**TGF-beta activated kinase 1 (MAP3K7) binding protein 2**	Cytoplasm	other		-1.196142125	0	0.36052773	0	1	0	11.1108236	11.4214373	11.1301064	11.0316145
742	**ENSG0000005521**	GINM1	protein_coding	chr6	149566294	149591748	+	Human GRCh	OTTHUMG000000	GINM1	**glycosylated integral membrane protein 1**	Cytoplasm	other		-1.22772778	0	0.34855223	0	1	0	9.29032016	9.58688018	9.34598806	9.09567632
743	**ENSG0000005533**	EIF2AK2	protein_coding	chr2	37099210	37157522	-	Human GRCh	OTTHUMG000001	EIF2AK2	**eukaryotic translation initiation factor 2 alpha kinase 2**	Cytoplasm	kinase	etavopivat	-1.191947916	0	0.35787463	0	1	0	11.2948652	11.5337014	11.38454	11.0532691
744	**ENSG0000005548**	USP36	protein_coding	chr17	78787381	78841441	-	Human GRCh	OTTHUMG000001	USP36	**ubiquitin specific peptidase 36**	Nucleus	peptidase		-1.013277918	0	0.95125669	0	1	0	11.4202854	11.7538387	11.7367996	11.4083534
745	**ENSG0000005560**	KMT2C	protein_coding	chr7	152134922	152436644	-	Human GRCh	OTTHUMG000001	KMT2C	**lysine methyltransferase 2C**	Nucleus	transcription		-1.082050233	0	0.71220491	0	1	0	14.1630765	14.393977	14.3107349	14.0686766
746	**ENSG0000005573**	MCOLN3	protein_coding	chr1	85018082	85048500	-	Human GRCh	OTTHUMG000000	MCOLN3	**mucolipin TRP cation channel 3**	Plasma Mem	ion channel		-1.080790478	0	0.87480258	0	1	0	8.17361302	8.48797451	8.12739743	8.38444092
747	**ENSG0000005581**	CCDC85A	protein_coding	chr2	56183990	56386172	+	Human GRCh	OTTHUMG000001	CCDC85A	**coiled-coil domain containing 85A**	Other	other		2.77177999	1	0.31512262	0	1	0	1.96399276	2.55863239	2.00859812	3.02835418
748	**ENSG0000005591**	PUM2	protein_coding	chr2	20248691	20352234	-	Human GRCh	OTTHUMG000001	PUM2	**pumilio RNA binding family member 2**	Cytoplasm	other		-1.062949079	0	0.76774708	0	1	0	12.4167252	12.6801094	12.6648223	12.2789066
749	**ENSG0000005595**	MRPL43	protein_coding	chr10	100969458	100987515	-	Human GRCh	OTTHUMG000000	MRPL43	**mitochondrial ribosomal protein L43**	Cytoplasm	translation re		-1.084697213	0	0.71176595	0	1	0	9.99325882	10.4868743	10.2215	10.1234133
750	**ENSG0000005595**	ITIH4	protein_coding	chr3	52812962	52830688	-	Human GRCh	OTTHUMG000001	ITIH4	**inter-alpha-trypsin inhibitor heavy chain 4**	Extracellular	other		-1.108513881	0	0.71595033	0	1	0	7.17729356	7.31637943	7.27426753	6.99095673
752	**ENSG0000005605**	HPF1	protein_coding	chr4	169729470	169757944	-	Human GRCh	OTTHUMG000001	HPF1	**histone PARylation factor 1**	Nucleus	other		-1.079098617	0	0.7882511	0	1	0	7.02234416	7.46380852	7.35678457	6.9812022
753	**ENSG0000005609**	ZFR	protein_coding	chr5	32354350	32444740	-	Human GRCh	OTTHUMG000001	ZFR	**zinc finger RNA binding protein**	Nucleus	other		-1.041521731	0	0.83489015	0	1	0	11.4281411	11.7235835	11.6437622	11.4278676
754	**ENSG0000005627**	ZNF280C	protein_coding	chrX	130202707	130268899	-	Human GRCh	OTTHUMG000000	ZNF280C	**zinc finger protein 280C**	Nucleus	transcription		-1.104025364	0	0.66578646	0	1	0	8.90109618	8.97744361	8.92262209	8.73772479
755	**ENSG0000005629**	NPFFR2	protein_coding	chr4	72031902	72148305	+	Human GRCh	OTTHUMG000001	NPFFR2	**neuropeptide FF receptor 2**	Plasma Mem	G-protein cou		10.19398882	1	0.12467426	0	1	0	0.94014795	0.98224894	0.97537903	1.91675418
756	**ENSG0000005648**	PHF21B	protein_coding	chr22	44881162	45010005	-	Human GRCh	OTTHUMG000001	PHF21B	**PHD finger protein 21B**	Nucleus	other		-1.156672609	0	0.61732768	0	1	0	8.70742685	9.0375779	8.86695608	8.57393326
757	**ENSG0000005655**	TRAF1	protein_coding	chr9	120902393	120929173	-	Human GRCh	OTTHUMG000000	TRAF1	**TNF receptor associated factor 1**	Cytoplasm	other		-1.161731401	0	0.65559609	0	1	0	7.18194516	7.48795984	7.23873445	7.13840211
758	**ENSG0000005658**	RC3H2	protein_coding	chr9	122844556	122905359	-	Human GRCh	OTTHUMG000000	RC3H2	**ring finger and CCCH-type domains 2**	Plasma Mem	enzyme		-1.056014223	0	0.78760045	0	1	0	11.9962449	12.1888608	12.182583	11.8691839
759	**ENSG0000005673**	IL17RB	protein_coding	chr3	53846568	53865794	+	Human GRCh	OTTHUMG000001	IL17RB	**interleukin 17 receptor B**	Plasma Mem	transmembra		1.135640245	0	0.67459933	0	1	0	9.76246625	10.2725401	10.3622961	9.97248532
760	**ENSG0000005697**	TRAF3IP2	protein_coding	chr6	111555381	111606906	-	Human GRCh	OTTHUMG000000	TRAF3IP2	**TRAF3 interacting protein 2**	Cytoplasm	enzyme		-1.186366182	0	0.43655182	0	1	0	9.10656087	9.29787404	8.95254612	9.09168104
761	**ENSG0000005699**	GYG2	protein_coding	chrX	2828822	2882818	+	Human GRCh	OTTHUMG000000	GYG2	**glycogenin 2**	Cytoplasm	enzyme		1	0	1	0	NA	0	0.07861698	0.09916381	0.09580694	0.0798381
762	**ENSG0000005701**	DCBLD2	protein_coding	chr3	98795941	98901695	-	Human GRCh	OTTHUMG000001	DCBLD2	**discoidin, CUB and LCCL domain containing 2**	Plasma Mem	other		1.010854433	0	0.96410947	0	1	0	9.87894718	10.1597435	10.2003949	9.85279133
764	**ENSG0000005725**	SOAT1	protein_coding	chr1	179293714	179358680	+	Human GRCh	OTTHUMG000000	SOAT1	**sterol O-acyltransferase 1**	Cytoplasm	enzyme	pactimibe,	-1.086206524	0	0.70493125	0	1	0	9.72695688	10.0640271	10.027488	9.56047924
765	**ENSG0000005729**	PKP2	protein_coding	chr12	32790745	32896798	-	Human GRCh	OTTHUMG000001	PKP2	**plakophilin 2**	Plasma Mem	other		-1.233164099	0	0.44217271	0	1	0	11.2405148	11.4613566	11.2727276	10.9314545
766	**ENSG0000005746**	MSH4	protein_coding	chr1	75796882	75913242	+	Human GRCh	OTTHUMG000000	MSH4	**mutS homolog 4**	Nucleus	enzyme		-1.020054822	0	0.96663587	0	1	0	5.54268796	4.74377744	5.42908717	4.91507889
767	**ENSG0000005759**	F7	protein_coding	chr13	113105788	113120685	+	Human GRCh	OTTHUMG000000	F7	**coagulation factor VII**	Plasma Mem	peptidase	nematode	-1.378134813	0	0.69477798	0	1	0	2.49149525	3.6057148	3.34342513	2.49582907
768	**ENSG0000005760**	GDI2	protein_coding	chr10	5765223	5842132	-	Human GRCh	OTTHUMG000000	GDI2	**GDP dissociation inhibitor 2**	Cytoplasm	other		-1.176942825	0	0.43016194	0	1	0	12.5633396	12.9414138	12.7420091	12.4046535
769	**ENSG0000005765**	PRDM1	protein_coding	chr6	105993463	106109939	+	Human GRCh	OTTHUMG000000	PRDM1	**PR/SET domain 1**	Nucleus	transcription		2.683371993	1	0.24343537	0	1	0	3.16865354	2.60920461	3.56771276	3.56076337
770	**ENSG0000005766**	ATG5	protein_coding	chr6	106045423	106325791	-	Human GRCh	OTTHUMG000000	ATG5	**autophagy related 5**	Cytoplasm	other		-1.064851079	0	0.76792558	0	1	0	9.16121788	9.40943075	9.36470082	9.06403432
771	**ENSG0000005770**	TMCC3	protein_coding	chr12	94567122	94650557	-	Human GRCh	OTTHUMG000001	TMCC3	**transmembrane and coiled-coil domain family 3**	Cytoplasm	other		-1.16605507	0	0.63347056	0	1	0	8.10797179	8.62038747	8.16335204	8.27438394
772	**ENSG0000005775**	PITHD1	protein_coding	chr1	23778418	23788232	+	Human GRCh	OTTHUMG000000	PITHD1	**PITH domain containing 1**	Nucleus	other		1.045144639	0	0.83617525	0	1	0	9.88592642	10.0971361	10.0979551	9.98998634
773	**ENSG0000005793**	MTA3	protein_coding	chr2	42494569	42756947	+	Human GRCh	OTTHUMG000001	MTA3	**metastasis associated 1 family member 3**	Nucleus	transcription		-1.24136359	0	0.37253779	0	1	0	8.86982931	9.11364668	8.85792374	8.68961865
774	**ENSG0000005805**	USP13	protein_coding	chr3	179653032	179804366	+	Human GRCh	OTTHUMG000001	USP13	**ubiquitin specific peptidase 13**	Cytoplasm	peptidase		-1.031373446	0	0.88970801	0	1	0	10.4734562	10.8211966	10.7969546	10.4297863
775	**ENSG0000005806**	ATP11B	protein_coding	chr3	182793503	182921629	+	Human GRCh	OTTHUMG000001	ATP11B	**ATPase phospholipid transporting 11B (putative)**	Plasma Mem	transporter		-1.031719434	0	0.89154016	0	1	0	10.0457588	10.4644026	10.2836142	10.1944108
776	**ENSG0000005808**	LAMC2	protein_coding	chr1	183186238	183245127	+	Human GRCh	OTTHUMG000000	LAMC2	**laminin subunit gamma 2**	Extracellular	other		-1.482287167	0	0.70201422	0	1	0	2.68354948	3.1327839	3.11144104	2.21453415
777	**ENSG0000005809**	CDK14	protein_coding	chr7	90466424	91210590	+	Human GRCh	OTTHUMG000000	CDK14	**cyclin dependent kinase 14**	Nucleus	kinase		-1.355659323	0	0.19338679	0	1	0	10.6413347	10.8389002	10.4965207	10.3033216
778	**ENSG0000005826**	SEC61A1	protein_coding	chr3	128051641	128071705	+	Human GRCh	OTTHUMG000001	SEC61A1	**SEC61 translocon subunit alpha 1**	Cytoplasm	transporter	KZR-261	-1.194781345	0	0.36285862	0	1	0	12.9478655	13.2244911	13.026868	12.7413901
779	**ENSG0000005827**	PPP1R12A	protein_coding	chr12	79773563	79935460	-	Human GRCh	OTTHUMG000001	PPP1R12A	**protein phosphatase 1 regulatory subunit 12A**	Cytoplasm	phosphatase		-1.015698045	0	0.93744341	0	1	0	11.0981802	11.3175018	11.2903305	11.0936686
780	**ENSG0000005833**	RASGRF1	protein_coding	chr15	78959906	79090780	-	Human GRCh	OTTHUMG000001	RASGRF1	**Ras protein specific guanine nucleotide releasing factor 1**	Cytoplasm	other		1.089455712	0	0.91887755	0	1	0	3.77249247	3.34105358	2.82504831	4.0660067
781	**ENSG0000005840**	CAMK2B	protein_coding	chr7	44210019	44334577	-	Human GRCh	OTTHUMG000000	CAMK2B	**calcium/calmodulin dependent protein kinase II beta**	Cytoplasm	kinase		-1.002808144	0	0.99086733	0	1	0	8.61838316	8.72235209	8.96058181	8.2785834
782	**ENSG0000005845**	CROCC	protein_coding	chr1	16740273	16972964	+	Human GRCh	OTTHUMG000000	CROCC	**ciliary rootlet coiled-coil, rootletin**	Plasma Mem	other		1.02516189	0	0.92464856	0	1	0	9.35574674	9.52252736	9.59352383	9.3285137
783	**ENSG0000005860**	POLR3E	protein_coding	chr16	22297375	22335101	+	Human GRCh	OTTHUMG000000	POLR3E	**RNA polymerase III subunit E**	Nucleus	transcription		1.014858677	0	0.94022471	0	1	0	11.1054168	11.4299169	11.3192685	11.27068
784	**ENSG0000005866**	ATP2B4	protein_coding	chr1	203626832	203744081	+	Human GRCh	OTTHUMG000000	ATP2B4	**ATPase plasma membrane Ca2+ transporting 4**	Plasma Mem	transporter		-1.226264531	0	0.42152948	0	1	0	8.97408578	8.94398279	8.58528305	8.88774369
785	**ENSG0000005867**	ZC3H11A	protein_coding	chr1	203795623	203854999	+	Human GRCh	OTTHUMG000000	ZC3H11A	**zinc finger CCCH-type containing 11A**	Nucleus	other		1.021510072	0	0.94556221	0	1	0	6.60500994	6.85845131	6.93842313	6.55024062
786	**ENSG0000005872**	RIOK2	protein_coding	chr5	97160867	97183247	-	Human GRCh	OTTHUMG000001	RIOK2	**RIO kinase 2**	Cytoplasm	kinase		-1.176682336	0	0.48198382	0	1	0	9.32322387	9.84702581	9.54722265	9.29638312
787	**ENSG0000005879**	YIPF1	protein_coding	chr1	53851719	53889798	-	Human GRCh	OTTHUMG000000	YIPF1	**Yip1 domain family member 1**	Cytoplasm	other		-1.065349106	0	0.7853157	0	1	0	8.35457467	8.20822938	8.2305123	8.20157038
788	**ENSG0000005880**	NDC1	protein_coding	chr1	53765478	53838463	-	Human GRCh	OTTHUMG000000	NDC1	**NDC1 transmembrane nucleoporin**	Nucleus	other		-1.123652262	0	0.58644944	0	1	0	11.3808541	11.6640816	11.5518217	11.2302172
789	**ENSG0000005886**	DGKG	protein_coding	chr3	186105668	186362234	-	Human GRCh	OTTHUMG000001	DGKG	**diacylglycerol kinase gamma**	Cytoplasm	kinase		-5.203714937	-1	0.3043573	0	1	0	2.67382806	2.22930321	2.21719352	2.15913702
790	**ENSG0000005912**	FLYWCH1	protein_coding	chr16	2911931	2951208	+	Human GRCh	OTTHUMG000001	FLYWCH1	**FLYWCH-type zinc finger 1**	Nucleus	transcription		1.224657434	0	0.34618231	0	1	0	9.6234617	9.80107228	10.0312863	9.83751339
791	**ENSG0000005914**	UNKL	protein_coding	chr16	1363205	1414751	-	Human GRCh	OTTHUMG000001	UNKL	**unk like zinc finger**	Cytoplasm	enzyme		-1.185644623	0	0.43067034	0	1	0	10.095735	10.3073359	10.0539209	9.98694272
792	**ENSG0000005937**	TBXAS1	protein_coding	chr7	139777051	140020325	+	Human GRCh	OTTHUMG000001	TBXAS1	**thromboxane A synthase 1**	Plasma Mem	enzyme	ridogrel, d	2.229949495	1	0.06707396	0	1	0	4.66722845	4.06913603	4.85688679	5.2579181
793	**ENSG0000005937**	PARP12	protein_coding	chr7	140023749	140062951	-	Human GRCh	OTTHUMG000001	PARP12	**poly(ADP-ribose) polymerase family member 12**	Nucleus	enzyme		1.493737153	0	0.13236028	0	1	0	8.43821532	8.68976044	9.02925155	8.96774601
794	**ENSG0000005957**	ALDH18A1	protein_coding	chr10	95605941	95656711	-	Human GRCh	OTTHUMG000000	ALDH18A1	**aldehyde dehydrogenase 18 family member A1**	Cytoplasm	kinase		-1.146806707	0	0.49574074	0	1	0	11.5505472	11.9287442	11.7250443	11.4539764
795	**ENSG0000005958**	TARBP1	protein_coding	chr1	234391313	234479179	-	Human GRCh	OTTHUMG000000	TARBP1	**TAR (HIV-1) RNA binding protein 1**	Nucleus	transcription		1.403045061	0	0.27800084	0	1	0	9.18175211	9.41257567	9.77932042	9.51345788
796	**ENSG0000005969**	GATB	protein_coding	chr4	151670504	151761007	-	Human GRCh	OTTHUMG000001	GATB	**glutamyl-tRNA amidotransferase subunit B**	Cytoplasm	enzyme		-1.14536531	0	0.55398454	0	1	0	9.16916434	9.51308631	9.35435139	9.02673098
797	**ENSG0000005972**	MXD1	protein_coding	chr2	69897688	69942945	+	Human GRCh	OTTHUMG000001	MXD1	**MAX dimerization protein 1**	Nucleus	transcription		-1.158571056	0	0.5193592	0	1	0	9.63517455	10.0373883	9.71187597	9.66300365
798	**ENSG0000005975**	CDK17	protein_coding	chr12	96278261	96400480	-	Human GRCh	OTTHUMG000001	CDK17	**cyclin dependent kinase 17**	Cytoplasm	kinase	SM1-71	1.10535467	0	0.65639316	0	1	0	9.91446549	10.023102	10.2208306	9.91431356
799	**ENSG0000005976**	DNAJC25	protein_coding	chr9	111631334	111654351	+	Human GRCh	OTTHUMG000000	DNAJC25	**DnaJ heat shock protein family (Hsp40) member C25**	Other	other		1.104326759	0	0.71703185	0	1	0	8.43164612	9.07027673	9.13185363	8.61988889
800	**ENSG0000005980**	SLC2A3	protein_coding	chr12	7919230	8019007	-	Human GRCh	OTTHUMG000001	SLC2A3	**solute carrier family 2 member 3**	Plasma Mem	transporter		1.282078693	0	0.76536875	0	1	0	8.10113563	7.86638971	7.52315011	8.74622427
801	**ENSG0000005991**	PSD	protein_coding	chr10	102402617	102421539	-	Human GRCh	OTTHUMG000000	PSD	**pleckstrin and Sec7 domain containing**	Cytoplasm	transporter		-1.10831813	0	0.70135005	0	1	0	7.47369928	7.61670482	7.60789586	7.24766358
802	**ENSG0000006006**	CTDP1	protein_coding	chr18	79679803	79754503	+	Human GRCh	OTTHUMG000001	CTDP1	**CTD phosphatase subunit 1**	Nucleus	phosphatase		1.023239163	0	0.91298173	0	1	0	9.8381655	10.1653093	10.0954791	9.97827722
803	**ENSG0000006013**	YBX3	protein_coding	chr12	10699089	10723323	-	Human GRCh	OTTHUMG000001	YBX3	**Y-box binding protein 3**	Nucleus	transcription		-1.079691193	0	0.70687036	0	1	0	11.887226	12.1961374	11.9471492	11.9782882

804	ENSG0000006014	STYK1	protein_coding	chr12	10618923	10674318	-	Human GRCh	OTTHUMG000001	STYK1	serine/threonine/tyrosine kinase 1	Cytoplasm	kinase		-1.194829046	0	0.67918121	0	1	0	6.32739929	6.77269866	6.21086437	6.53557461
805	ENSG0000006023	WNK1	protein_coding	chr12	752579	911452	+	Human GRCh	OTTHUMG000000	WNK1	WNK lysine deficient protein kinase 1	Cytoplasm	kinase	WNK463	-1.095637687	0	0.62987976	0	1	0	13.0726747	13.3357745	13.1848584	13.0324364
806	ENSG0000006033	CCAR1	protein_coding	chr10	68721012	68792377	+	Human GRCh	OTTHUMG000000	CCAR1	cell division cycle and apoptosis regulator 1	Nucleus	transcription		-1.171749728	0	0.40310516	0	1	0	12.1171875	12.4833443	12.247703	12.0185225
807	ENSG0000006049	OGFR	protein_coding	chr20	62804835	62814000	+	Human GRCh	OTTHUMG000000	OGFR	opioid growth factor receptor	Plasma Mem	other	enkephalir	1.11898847	0	0.54382845	0	1	0	10.0490844	10.2580961	10.4217261	10.123287
810	ENSG0000006064	PIGV	protein_coding	chr1	26787054	26800659	+	Human GRCh	OTTHUMG000000	PIGV	phosphatidylinositol glycan anchor biosynthesis class V	Cytoplasm	enzyme		-1.190797994	0	0.46471545	0	1	0	8.97225502	9.17231724	8.8929438	8.88057466
811	ENSG0000006065	PTPRU	protein_coding	chr1	29236516	29326813	+	Human GRCh	OTTHUMG000000	PTPRU	protein tyrosine phosphatase receptor type U	Plasma Mem	phosphatase		1.016116313	0	0.95610631	0	1	0	8.51122397	8.46835556	8.54462936	8.4669394
812	ENSG0000006068	SNRNP40	protein_coding	chr1	31259568	31296788	-	Human GRCh	OTTHUMG000000	SNRNP40	small nuclear ribonucleoprotein U5 subunit 40	Nucleus	other		-1.171822999	0	0.41418963	0	1	0	10.6122432	11.0268123	10.7814041	10.5301255
814	ENSG0000006071	COL11A1	protein_coding	chr1	102876467	103108872	-	Human GRCh	OTTHUMG000000	COL11A1	collagen type XI alpha 1 chain	Extracellular	other	collagenas	-5.797943875	-1	0.26978566	0	NA	0	1.43776319	1.88073083	1.4524801	1.43878687
815	ENSG0000006074	QSER1	protein_coding	chr11	32892811	32993316	+	Human GRCh	OTTHUMG000001	QSER1	glutamine and serine rich 1	Other	other		-1.118686511	0	0.60900851	0	1	0	12.1096069	12.4453522	12.237444	12.0912206
816	ENSG0000006076	MPC1	protein_coding	chr6	166364919	166383013	-	Human GRCh	OTTHUMG000000	MPC1	mitochondrial pyruvate carrier 1	Cytoplasm	transporter		1.007021229	0	0.97699176	0	1	0	9.35083001	9.61149937	9.62916893	9.34619209
817	ENSG0000006097	ACAA1	protein_coding	chr3	38103129	38137242	-	Human GRCh	OTTHUMG000001	ACAA1	acetyl-CoA acyltransferase 1	Cytoplasm	enzyme	trimetazidi	1.131158944	0	0.54242349	0	1	0	9.99740152	10.182929	10.3833595	10.0574571
818	ENSG0000006098	BCAT1	protein_coding	chr12	24810024	24949101	-	Human GRCh	OTTHUMG000001	BCAT1	branched chain amino acid transaminase 1	Cytoplasm	enzyme		-1.318578492	0	0.56784458	0	1	0	5.79522745	5.5636727	5.44128529	5.36377496
819	ENSG0000006127	HDAC7	protein_coding	chr12	47782722	47833132	-	Human GRCh	OTTHUMG000001	HDAC7	histone deacetylase 7	Nucleus	transcription	tributyrin,	-1.1363594	0	0.58309534	0	1	0	10.5514703	10.5419659	10.5580064	10.2277912
820	ENSG0000006133	LZTS1	protein_coding	chr8	20246165	20303963	-	Human GRCh	OTTHUMG000000	LZTS1	leucine zipper tumor suppressor 1	Nucleus	other		1	0	1	0	1	0	2.40696485	2.51347466	2.49636669	2.41342454
821	ENSG0000006145	PRDM6	protein_coding	chr5	123089241	123194266	+	Human GRCh	OTTHUMG000001	PRDM6	PR/SET domain 6	Nucleus	transcription		-1.514917658	-1	0.39386061	0	1	0	4.75218115	4.32175144	3.81336769	4.46936436
823	ENSG0000006165	SPAG4	protein_coding	chr20	35615829	35621094	+	Human GRCh	OTTHUMG000000	SPAG4	sperm associated antigen 4	Cytoplasm	other		-1.222213878	0	0.60892217	0	1	0	7.7284296	8.22284811	7.78212513	7.77209526
824	ENSG0000006167	NCKAP1	protein_coding	chr2	182909115	183038858	-	Human GRCh	OTTHUMG000001	NCKAP1	NCK associated protein 1	Plasma Mem	other		-1.118984489	0	0.56977823	0	1	0	12.356074	12.6505339	12.5342322	12.2192536
825	ENSG0000006179	MRPS35	protein_coding	chr12	27710822	27756295	+	Human GRCh	OTTHUMG000001	MRPS35	mitochondrial ribosomal protein S35	Cytoplasm	other		-1.099575101	0	0.67003761	0	1	0	11.0387772	11.3514288	11.2257979	10.9528771
826	ENSG0000006191	GUCY1B1	protein_coding	chr4	155758992	155807811	+	Human GRCh	OTTHUMG000001	GUCY1B1	guanylate cyclase 1 soluble subunit beta 1	Cytoplasm	enzyme	isosorbide-	1.519833573	1	0.29436485	0	1	0	7.92022325	8.47700627	8.69781413	8.73113583
827	ENSG0000006193	SFSWAP	protein_coding	chr12	131711081	131799738	+	Human GRCh	OTTHUMG000001	SFSWAP	splicing factor SWAP	Nucleus	other		1.033733047	0	0.86273259	0	1	0	11.0190577	11.2213805	11.2963943	11.0074583
828	ENSG0000006193	TNK2	protein_coding	chr3	195863364	195911945	-	Human GRCh	OTTHUMG000001	TNK2	tyrosine kinase non receptor 2	Cytoplasm	kinase	osimertinil	-1.063399993	0	0.8030403	0	1	0	12.5041532	12.6443458	12.600251	12.4034941
829	ENSG0000006198	MON2	protein_coding	chr12	62466817	62600476	+	Human GRCh	OTTHUMG000001	MON2	MON2 homolog, regulator of endosome-to-Golgi trafficking	Cytoplasm	other		-1.002734032	0	0.99043215	0	1	0	11.3785768	11.6493805	11.6757816	11.3374598
830	ENSG0000006203	CDH3	protein_coding	chr16	68636189	68727468	+	Human GRCh	OTTHUMG000001	CDH3	cadherin 3	Plasma Mem	other	PF-373201	-1.062425893	0	0.85853205	0	1	0	6.18095297	6.06696713	6.09870033	6.04019614
832	ENSG0000006219	GPBP1	protein_coding	chr5	57173948	57264679	+	Human GRCh	OTTHUMG000001	GPBP1	GC-rich promoter binding protein 1	Nucleus	transcription		-1.039140176	0	0.84447428	0	1	0	11.3434536	11.5469501	11.56792	11.2215577
833	ENSG0000006228	DGAT2	protein_coding	chr11	75759512	75801535	+	Human GRCh	OTTHUMG000001	DGAT2	diacylglycerol O-acyltransferase 2	Cytoplasm	enzyme		1.197004827	0	0.48570196	0	1	0	7.50509114	7.56438068	7.96095887	7.42770885
834	ENSG0000006237	ZNF112	protein_coding	chr19	44326555	44367217	-	Human GRCh	OTTHUMG000001	ZNF112	zinc finger protein 112	Nucleus	other		1.207087912	0	0.61049787	0	1	0	6.79068039	6.97416392	7.32868049	6.77009858
835	ENSG0000006248	CS	protein_coding	chr12	56271699	56300391	-	Human GRCh	OTTHUMG000001	CS	citrate synthase	Cytoplasm	enzyme		-1.132078368	0	0.53376314	0	1	0	13.2715714	13.6710374	13.4217097	13.2718204
836	ENSG0000006252	LTK	protein_coding	chr15	41503637	41513887	-	Human GRCh	OTTHUMG000001	LTK	leukocyte receptor tyrosine kinase	Plasma Mem	kinase	lorlatinib	-1.348385705	0	0.76430758	0	NA	0	3.0015483	3.41765993	2.91001445	3.12321518
837	ENSG0000006258	MRPS24	protein_coding	chr7	43866558	43869893	-	Human GRCh	OTTHUMG000000	MRPS24	mitochondrial ribosomal protein S24	Cytoplasm	other		-1.05394837	0	0.80946525	0	1	0	10.1475375	10.5049304	10.2569389	10.3040564
838	ENSG0000006259	ELMO2	protein_coding	chr20	46366050	46432985	-	Human GRCh	OTTHUMG000000	ELMO2	engulfment and cell motility 2	Cytoplasm	other		-1.119308186	0	0.54652324	0	1	0	9.45804608	9.6391317	9.43714664	9.42214374
839	ENSG0000006265	WAPL	protein_coding	chr10	86435256	86521792	-	Human GRCh	OTTHUMG000000	WAPL	WAPL cohesin release factor	Nucleus	other		-1.008314104	0	0.9647061	0	1	0	11.1972731	11.525916	11.4975132	11.2131467
840	ENSG0000006271	VMP1	protein_coding	chr17	59707192	59842255	+	Human GRCh	OTTHUMG000001	VMP1	vacuole membrane protein 1	Cytoplasm	transporter		1.063155274	0	0.79325463	0	1	0	10.6027134	10.8412786	10.9305098	10.6403833
841	ENSG0000006272	APPBP2	protein_coding	chr17	60443158	60526242	-	Human GRCh	OTTHUMG000001	APPBP2	amyloid beta precursor protein binding protein 2	Cytoplasm	other		-1.112379186	0	0.6358692	0	1	0	10.4154945	10.5907204	10.5792177	10.1612637
842	ENSG0000006282	POLD1	protein_coding	chr19	50384204	50418018	+	Human GRCh	OTTHUMG000001	POLD1	DNA polymerase delta 1, catalytic subunit	Nucleus	enzyme	entecavir,	-1.044699833	0	0.82569643	0	1	0	11.5093943	11.771005	11.7001535	11.4867936
843	ENSG0000006301	SEZ6	protein_coding	chr17	28954901	29006440	-	Human GRCh	OTTHUMG000001	SEZ6	seizure related 6 homolog	Extracellular	other	ABBV-706	16.88080303	1	0.0232093	1	1	0	3.51104517	3.59654966	4.474911	3.51615093
844	ENSG0000006304	EIF4B	protein_coding	chr12	53006282	53042215	+	Human GRCh	OTTHUMG000001	EIF4B	eukaryotic translation initiation factor 4B	Cytoplasm	translation re		-1.048185306	0	0.83118281	0	1	0	14.1392521	14.3821006	14.3795799	14.0214776
845	ENSG0000006312	SLC6A16	protein_coding	chr19	49289638	49325215	-	Human GRCh	OTTHUMG000001	SLC6A16	solute carrier family 6 member 16	Plasma Mem	transporter		-1.709572848	-1	0.23767187	0	1	0	6.85575566	7.36386842	6.58029037	6.46090264
846	ENSG0000006316	BICRA	protein_coding	chr19	47608196	47703277	+	Human GRCh	OTTHUMG000001	BICRA	BRD4 interacting chromatin remodeling complex associated pr	Nucleus	transcription		1.020114507	0	0.9278533	0	1	0	10.511301	10.7902798	10.6607844	10.7006938
847	ENSG0000006317	SPHK2	protein_coding	chr19	48619291	48630717	+	Human GRCh	OTTHUMG000001	SPHK2	sphingosine kinase 2	Cytoplasm	kinase	(3S,5R)-5-(	1.015781387	0	0.94293017	0	1	0	10.1800245	10.4283369	10.4544938	10.187099
848	ENSG0000006317	RPL18	protein_coding	chr19	48615328	48619184	-	Human GRCh	OTTHUMG000001	RPL18	ribosomal protein L18	Cytoplasm	other		1.016207216	0	0.93838854	0	1	0	13.3019811	13.5667857	13.5517647	13.3604972
849	ENSG0000006318	CA11	protein_coding	chr19	48637946	48646187	-	Human GRCh	OTTHUMG000001	CA11	carbonic anhydrase 11	Extracellular	enzyme		-1.013706902	0	0.95892201	0	1	0	8.3091534	8.80970604	8.57755685	8.56223295
850	ENSG0000006324	ISOC2	protein_coding	chr19	55452985	55462343	-	Human GRCh	OTTHUMG000001	ISOC2	isochorismatase domain containing 2	Cytoplasm	enzyme		-1.088420488	0	0.71948895	0	1	0	10.7886656	11.1501237	11.1014962	10.6229513
851	ENSG0000006324	U2AF2	protein_coding	chr19	55654146	55674716	+	Human GRCh	OTTHUMG000001	U2AF2	U2 small nuclear RNA auxiliary factor 2	Nucleus	other		-1.003369794	0	0.98528025	0	1	0	12.6237938	12.9179374	12.9013686	12.6361773
852	ENSG0000006324	EPN1	protein_coding	chr19	55675226	55709858	+	Human GRCh	OTTHUMG000001	EPN1	epsin 1	Plasma Mem	other		-1.060243768	0	0.77287621	0	1	0	12.7321501	13.0581231	12.9301084	12.7423949
853	ENSG0000006332	MED29	protein_coding	chr19	39391303	39400641	+	Human GRCh	OTTHUMG000001	MED29	mediator complex subunit 29	Nucleus	other		-1.034095017	0	0.87439744	0	1	0	10.6010365	11.0520702	10.917537	10.6920681
854	ENSG0000006343	AHRR	protein_coding	chr5	321714	438291	+	Human GRCh	OTTHUMG000001	AHRR	aryl hydrocarbon receptor repressor	Nucleus	transcription		1.120297479	0	0.69471976	0	1	0	7.72348355	8.14367299	8.1728102	7.96609646
856	ENSG0000006358	ZNF275	protein_coding	chrX	153334147	153360110	+	Human GRCh	OTTHUMG000000	ZNF275	zinc finger protein 275	Nucleus	transcription		-1.087754079	0	0.70330931	0	1	0	10.5082263	10.7574371	10.7435627	10.3117538
857	ENSG0000006360	MTMR1	protein_coding	chrX	150692971	150765108	+	Human GRCh	OTTHUMG000000	MTMR1	myotubularin related protein 1	Cytoplasm	phosphatase		-1.057378832	0	0.79232068	0	1	0	10.2958938	10.5362242	10.4810071	10.2265122
858	ENSG0000006366	GPC1	protein_coding	chr2	240435663	240468076	+	Human GRCh	OTTHUMG000001	GPC1	glypican 1	Plasma Mem	transmembra		1.207651453	0	0.57088523	0	1	0	10.4398771	10.7973375	11.1897845	10.3588769
859	ENSG0000006376	ADCK1	protein_coding	chr14	77800109	77935014	+	Human GRCh	OTTHUMG000001	ADCK1	aarF domain containing kinase 1	Cytoplasm	kinase		-1.18737376	0	0.4599809	0	1	0	7.93052726	8.26687259	8.0944474	7.73342402
860	ENSG0000006385	HAGH	protein_coding	chr16	1795620	1827157	-	Human GRCh	OTTHUMG000001	HAGH	hydroxyacylglutathione hydrolase	Cytoplasm	enzyme		-1.004649604	0	0.98465187	0	1	0	9.85956126	10.3118375	10.1421712	10.0598187
861	ENSG0000006397	RNF4	protein_coding	chr4	2462220	2515857	+	Human GRCh	OTTHUMG000001	RNF4	ring finger protein 4	Nucleus	transcription		-1.090862894	0	0.692604	0	1	0	11.3282356	11.6417745	11.5364239	11.2409498
862	ENSG0000006401	CASP8	protein_coding	chr2	201233443	201361836	+	Human GRCh	OTTHUMG000001	CASP8	caspase 8	Nucleus	peptidase		1.301338348	0	0.52792248	0	1	0	6.74469816	7.0574086	7.64612681	6.36526699
863	ENSG0000006404	LIMCH1	protein_coding	chr4	41359607	41700044	+	Human GRCh	OTTHUMG000001	LIMCH1	LIM and calponin homology domains 1	Cytoplasm	other		-1.036486509	0	0.95163314	0	1	0	8.41824269	8.09362159	7.57800339	8.6401742
864	ENSG0000006410	INTS13	protein_coding	chr12	26905181	26938326	-	Human GRCh	OTTHUMG000001	INTS13	integrator complex subunit 13	Cytoplasm	other		-1.045902789	0	0.83659148	0	1	0	10.027606	10.5233291	10.396005	10.0833078
865	ENSG0000006411	TM7SF3	protein_coding	chr12	26971579	27014434	-	Human GRCh	OTTHUMG000001	TM7SF3	transmembrane 7 superfamily member 3	Plasma Mem	other		1.060225051	0	0.79163914	0	1	0	10.4293753	10.6821779	10.8754378	10.3106484
866	ENSG0000006419	DLX3	protein_coding	chr17	49990005	49995224	-	Human GRCh	OTTHUMG000001	DLX3	distal-less homeobox 3	Nucleus	transcription		2.261217678	1	0.29050586	0	1	0	2.18914135	3.4833724	3.01968045	3.86401462
867	ENSG0000006419	SPA17	protein_coding	chr11	124673844	124697518	+	Human GRCh	OTTHUMG000001	SPA17	sperm autoantigenic protein 17	Plasma Mem	other		1.067712398	0	0.81749884	0	1	0	8.96204524	9.27014591	9.44114953	8.89185436
869	ENSG0000006420	CCN5	protein_coding	chr20	44714844	44728509	+	Human GRCh	OTTHUMG000000	CCN5	cellular communication network factor 5	Extracellular	growth facto		2.585365024	1	0.67545379	0	NA	0	1.60157371	0.38107936	1.06291904	2.19106045
870	ENSG0000006421	DMRT3	protein_coding	chr9	976655	991732	+	Human GRCh	OTTHUMG000000	DMRT3	doublesex and mab-3 related transcription factor 3	Nucleus	transcription		1.006379565	0	0.99151706	0	1	0	4.27770244	4.30183577	4.30208244	4.29036158
871	ENSG0000006422	ST3GAL6	protein_coding	chr3	98732236	98821201	+	Human GRCh	OTTHUMG000001	ST3GAL6	ST3 beta-galactoside alpha-2,3-sialyltransferase 6	Cytoplasm	enzyme		-1.077049781	0	0.80506153	0	1	0	7.76033609	8.1090035	7.90839535	7.81319975
872	ENSG0000006427	ATP2C2	protein_coding	chr16	84368527	84464187	+	Human GRCh	OTTHUMG000001	ATP2C2	ATPase secretory pathway Ca2+ transporting 2	Cytoplasm	enzyme		1.52865151	1	0.20480139	0	1	0	5.39934081	5.46113086	5.7216635	5.8917056
873	ENSG0000006430	NGFR	protein_coding	chr17	49495293	49515008	+	Human GRCh	OTTHUMG000001	NGFR	nerve growth factor receptor	Plasma Mem	transmembra	LM11A-31	1.171480441	0	0.73243995	0	1	0	4.90681999	4.93450738	5.08735508	5.07788721
874	ENSG0000006430	CDON	protein_coding	chr11	125955796	126063335	-	Human GRCh	OTTHUMG000001	CDON	cell adhesion associated, oncogene regulated	Plasma Mem	other		-1.219291881	0	0.45517906	0	1	0	8.82716817	9.00648348	8.98398492	8.38152014
875	ENSG0000006431	TAF2	protein_coding	chr8	119730774	119832863	-	Human GRCh	OTTHUMG000001	TAF2	TATA-box binding protein associated factor 2	Nucleus	transcription		-1.102907926	0	0.62201681	0	1	0	11.1570935	11.3315178	11.2670426	11.0046168
876	ENSG0000006439	HIPK2	protein_coding	chr7	139561570	139777998	-	Human GRCh	OTTHUMG000001	HIPK2	homeodomain interacting protein kinase 2	Nucleus	kinase		-1.002802709	0	0.98879015	0	1	0	12.9801041	13.1200293	12.9863143	13.1086235
877	ENSG0000006441	TNPO3	protein_coding	chr7	128954180	129055173	-	Human GRCh	OTTHUMG000001	TNPO3	transportin 3	Cytoplasm	other		-1.141813229	0	0.49452084	0	1	0	11.2362394	11.5258418	11.3364784	11.1362272
878	ENSG0000006449	RFXANK	protein_coding	chr19	19192229	19201869	+	Human GRCh	OTTHUMG000001	RFXANK	regulatory factor X associated ankyrin containing protein	Nucleus	transcription		-1.135476533	0	0.59884041	0	1	0	9.50131832	9.90224852	9.59547852	9.55682602
879	ENSG0000006454	TMEM161	protein_coding	chr19	19119169	19138513	-	Human GRCh	OTTHUMG000001	TMEM161A	transmembrane protein 161A	Other	other		1.054047061	0	0.79442542	0	1	0	9.78945225	10.0635446	10.095357	9.88134474
880	ENSG0000006454	LPAR2	protein_coding	chr19	19623655	19628930	-	Human GRCh	OTTHUMG000001	LPAR2	lysophosphatidic acid receptor 2	Plasma Mem	G-protein cou		-1.098256307	0	0.75007541	0	1	0	7.86996697	8.64441091	8.31891563	8.10744641
881	ENSG0000006460	CTSA	protein_coding	chr20	45890144	45898949	+	Human GRCh	OTTHUMG000000	CTSA	cathepsin A	Cytoplasm	peptidase		-1.102843429	0	0.64339705	0	1	0	10.9096819	11.3030689	11.1193344	10.8954267
882	ENSG0000006460	SUGP2	protein_coding	chr19	18990888	19034023	-	Human GRCh	OTTHUMG000001	SUGP2	SURP and G-patch domain containing 2	Nucleus	other		-1.126757669	0	0.56007831	0	1	0	12.4545123	12.7927184	12.6515731	12.3333186
883	ENSG0000006465	SLC12A2	protein_coding	chr5	128083766	128189677	+	Human GRCh	OTTHUMG000001	SLC12A2	solute carrier family 12 member 2	Plasma Mem	transporter	bumetanic	1.095366925	0	0.65862395	0	1	0	11.0323399	11.4125182	11.4550837	11.2154943
884	ENSG0000006465	SNX24	protein_coding	chr5	122843439	123029354	+	Human GRCh	OTTHUMG000001	SNX24	sorting nexin 24	Other	transporter		-1.414837313	0	0.21574784	0	1	0	7.33579865	7.76301908	7.11519651	7.28746922
885	ENSG0000006465	EYA2	protein_coding	chr20	46894624	47188844	+	Human GRCh	OTTHUMG000000	EYA2	EYA transcriptional coactivator and phosphatase 2	Nucleus	phosphatase		-2.039436979	-1	0.22689626	0	1	0	3.88806518	4.80846506	3.20601538	4.11108288
886	ENSG0000006466	CNN2	protein_coding	chr19	1026586	1039068	+	Human GRCh	OTTHUMG000001	CNN2	calponin 2	Cytoplasm	other		1.278027959	0	0.37802506	0	1	0	11.3157831	11.78756	11.6968698	12.0069725
887	ENSG0000006468	ABCA7	protein_coding	chr19	1039997	1065572	+	Human GRCh	OTTHUMG000001	ABCA7	ATP binding cassette subfamily A member 7	Plasma Mem	transporter		1.352618431	0	0.22995213	0	1	0	11.8113422	12.1577216	12.2543928	12.4249858
888	ENSG0000006469	SNCAIP	protein_coding	chr5	122311354	122464219	+	Human GRCh	OTTHUMG000001	SNCAIP	synuclein alpha interacting protein	Cytoplasm	transcription		-1.217527398	0	0.744849	0	1	0	3.57618274	4.38938116	3.41411145	4.21714059
889	ENSG0000006470	DDX20	protein_coding	chr1	111754832	111775602	+	Human GRCh	OTTHUMG000000	DDX20	DEAD-box helicase 20	Nucleus	transcription		-1.025829666	0	0.9045246	0	1	0	9.74173774	10.1209329	10.033416	9.78964395
890	ENSG0000006472	BTBD1	protein_coding	chr15	83016423	83067252	-	Human GRCh	OTTHUMG000001	BTBD1	BTB domain containing 1	Cytoplasm	other		1.01703013	0	0.94159419	0	1	0	11.4475893	11.8273957	11.8310625	11.4875403
891	ENSG0000006476	FAR2	protein_coding	chr12	29149016	29341121	+	Human GRCh	OTTHUMG000001	FAR2	fatty acyl-CoA reductase 2	Cytoplasm	enzyme		-1.137292553	0	0.60712287	0	1	0	9.9202436	10.3146609	10.1508736	9.80299648
892	ENSG0000006478	BCAS1	protein_coding	chr20	53936777	54070594	-	Human GRCh	OTTHUMG000000	BCAS1	brain enriched myelin associated protein 1	Plasma Mem	other		-1.018840357	0	0.98782033	0	1	0	5.34226219	5.74039979	5.94036347	4.91700095
895	ENSG0000006493	SBNO2	protein_coding	chr19	1107637	1174268	-	Human GRCh	OTTHUMG000001	SBNO2	strawberry notch homolog 2	Other	transcription		-1.012422017	0	0.95521678	0	1	0	10.8573576	11.1921668	11.0356291	11.0102013

896	**ENSG0000006493**	PMS1	protein_coding	chr2	189784085	189877629	+	Human GRCh	OTTHUMG000001	PMS1	**PMS1 homolog 1, mismatch repair system component**	Nucleus	enzyme		1.120059393	0	0.61166537	0	1	0	9.83986921	9.99052747	10.3041625	9.70773682
897	**ENSG0000006496**	HMG20B	protein_coding	chr19	3572777	3579088	+	Human GRCh	OTTHUMG000001	HMG20B	**high mobility group 20B**	Nucleus	transcription		1.032425679	0	0.87907021	0	1	0	12.2035706	12.5640853	12.4930514	12.3718653
898	**ENSG0000006498**	CALCRL	protein_coding	chr2	187341964	187448460	-	Human GRCh	OTTHUMG000001	CALCRL	**calcitonin receptor like receptor**	Plasma Mem	G-protein co	zavegepan	-1.028059537	0	0.98407514	0	NA	0	2.97292834	3.10682994	3.50851786	2.05551091
899	**ENSG0000006499**	TAF11	protein_coding	chr6	34877462	34889192	-	Human GRCh	OTTHUMG000000	TAF11	**TATA-box binding protein associated factor 11**	Nucleus	transcription		-1.000442398	0	0.99828971	0	1	0	9.83822852	10.1413617	10.1763973	9.78999865
900	**ENSG0000006499**	ANKS1A	protein_coding	chr6	34889255	35091406	+	Human GRCh	OTTHUMG000000	ANKS1A	**ankyrin repeat and sterile alpha motif domain containing 1A**	Cytoplasm	other		-1.053291674	0	0.78829045	0	1	0	11.0769688	11.288832	11.2599084	10.9839653
901	**ENSG0000006500**	AP3D1	protein_coding	chr19	2100988	2164468	-	Human GRCh	OTTHUMG000001	AP3D1	**adaptor related protein complex 3 subunit delta 1**	Cytoplasm	other		-1.086927825	0	0.676632	0	1	0	13.0423878	13.3669007	13.1622439	13.0796636
902	**ENSG0000006502**	ZNF76	protein_coding	chr6	35258909	35295985	+	Human GRCh	OTTHUMG000000	ZNF76	**zinc finger protein 76**	Nucleus	transcription		-1.153326226	0	0.4844302	0	1	0	10.9360012	11.2218568	11.0826215	10.7537392
903	**ENSG0000006505**	NHERF2	protein_coding	chr16	2025356	2039026	+	Human GRCh	OTTHUMG000001	NHERF2	**NHERF family PDZ scaffold protein 2**	Plasma Mem	transporter		1.252904451	0	0.4066521	0	1	0	9.61117487	9.89623271	9.98930901	10.0296536
904	**ENSG0000006505**	NTHL1	protein_coding	chr16	2039815	2047866	-	Human GRCh	OTTHUMG000001	NTHL1	**nth like DNA glycosylase 1**	Nucleus	enzyme		1.191594738	0	0.39150152	0	1	0	8.86201414	9.33469818	9.3600701	9.26346456
905	**ENSG0000006506**	BLTP3A	protein_coding	chr6	34792083	34877514	+	Human GRCh	OTTHUMG000000	BLTP3A	**bridge-like lipid transfer protein family member 3A**	Cytoplasm	other		-1.162197464	0	0.44513789	0	1	0	11.7690398	11.9118313	11.7920521	11.5502148
906	**ENSG0000006513**	GNAI3	protein_coding	chr1	109548615	109600195	+	Human GRCh	OTTHUMG000001	GNAI3	**G protein subunit alpha i3**	Cytoplasm	enzyme		-1.052293924	0	0.80750858	0	1	0	11.09118	11.1768627	11.2399201	10.8906954
907	**ENSG0000006515**	IPO5	protein_coding	chr13	97953658	98024296	+	Human GRCh	OTTHUMG000000	IPO5	**importin 5**	Nucleus	transporter		-1.165143373	0	0.43659927	0	1	0	12.8632557	13.2058952	13.0108603	12.726016
908	**ENSG0000006515**	OAT	protein_coding	chr10	124397303	124418976	-	Human GRCh	OTTHUMG000000	OAT	**ornithine aminotransferase**	Cytoplasm	enzyme		1.015429728	0	0.95270492	0	1	0	9.532469	9.81723649	9.95476819	9.37675548
909	**ENSG0000006518**	WDR3	protein_coding	chr1	117929720	117966543	+	Human GRCh	OTTHUMG000000	WDR3	**WD repeat domain 3**	Cytoplasm	other		1.060540857	0	0.7811304	0	1	0	10.3235015	10.7675026	10.766652	10.4803406
910	**ENSG0000006524**	PKN2	protein_coding	chr1	88684222	88836255	+	Human GRCh	OTTHUMG000000	PKN2	**protein kinase N2**	Cytoplasm	kinase	fasudil	-1.102500437	0	0.62595656	0	1	0	11.5747	11.8444526	11.7444363	11.4587214
911	**ENSG0000006526**	WDR18	protein_coding	chr19	984332	998438	+	Human GRCh	OTTHUMG000001	WDR18	**WD repeat domain 18**	Nucleus	other		1.048656986	0	0.83319996	0	1	0	10.4898911	10.7861043	10.8285313	10.5567793
912	**ENSG0000006530**	TRAM2	protein_coding	chr6	52497408	52577060	-	Human GRCh	OTTHUMG000000	TRAM2	**translocation associated membrane protein 2**	Other	other		-1.001276383	0	0.99516184	0	1	0	8.99733497	9.25469948	9.24390396	9.00726083
913	**ENSG0000006532**	NTN1	protein_coding	chr17	9021510	9244000	+	Human GRCh	OTTHUMG000001	NTN1	**netrin 1**	Extracellular	growth facto	NP137	1.027677543	0	0.96370646	0	1	0	9.48467173	9.75437505	9.98872698	9.19573913
915	**ENSG0000006532**	MCM10	protein_coding	chr10	13161558	13211110	+	Human GRCh	OTTHUMG000000	MCM10	**minichromosome maintenance 10 replication initiation factor**	Nucleus	other		-1.089913453	0	0.65793953	0	1	0	10.4290223	10.6203081	10.5424765	10.3135519
916	**ENSG0000006535**	DGKA	protein_coding	chr12	55927319	55954027	+	Human GRCh	OTTHUMG000001	DGKA	**diacylglycerol kinase alpha**	Cytoplasm	kinase		-1.022264527	0	0.92538178	0	1	0	7.67490821	7.84736122	8.01704442	7.38044791
917	**ENSG0000006536**	ERBB3	protein_coding	chr12	56076799	56103505	+	Human GRCh	OTTHUMG000001	ERBB3	**erb-b2 receptor tyrosine kinase 3**	Plasma Mem	kinase	afatinib, SI	-1.179292582	0	0.49216616	0	1	0	11.7049098	12.0534392	11.8191101	11.5922835
919	**ENSG0000006541**	ANKRD44	protein_coding	chr2	196967017	197311173	-	Human GRCh	OTTHUMG000001	ANKRD44	**ankyrin repeat domain 44**	Other	other		-1.063430924	0	0.8249162	0	1	0	8.69509068	9.00196759	8.90815127	8.66532338
920	**ENSG0000006542**	KARS1	protein_coding	chr16	75627474	75648643	-	Human GRCh	OTTHUMG000001	KARS1	**lysyl-tRNA synthetase 1**	Cytoplasm	enzyme		-1.047613672	0	0.82930905	0	1	0	11.5827524	12.0212934	11.8769887	11.6496882
921	**ENSG0000006545**	ADAT1	protein_coding	chr16	75596868	75623300	-	Human GRCh	OTTHUMG000001	ADAT1	**adenosine deaminase tRNA specific 1**	Other	enzyme		-1.062171597	0	0.76774258	0	1	0	10.3983423	10.7044775	10.6831706	10.269253
922	**ENSG0000006548**	PDIA5	protein_coding	chr3	123067025	123225227	+	Human GRCh	OTTHUMG000001	PDIA5	**protein disulfide isomerase family A member 5**	Cytoplasm	enzyme		-1.112582142	0	0.63305217	0	1	0	10.4184891	10.5674911	10.5180418	10.2161263
923	**ENSG0000006549**	TBC1D22B	protein_coding	chr6	37257772	37332970	+	Human GRCh	OTTHUMG000000	TBC1D22B	**TBC1 domain family member 22B**	Other	other		-1.011318465	0	0.95727102	0	1	0	9.38015618	9.51789527	9.47068495	9.40577878
924	**ENSG0000006551**	NDUFB4	protein_coding	chr3	120596328	120602507	+	Human GRCh	OTTHUMG000001	NDUFB4	**NADH:ubiquinone oxidoreductase subunit B4**	Cytoplasm	transporter		-1.083204343	0	0.72694954	0	1	0	10.7070827	11.1071036	10.8651863	10.8037277
925	**ENSG0000006552**	SPEN	protein_coding	chr1	15836095	15940456	+	Human GRCh	OTTHUMG000000	SPEN	**spen family transcriptional repressor**	Nucleus	transcription		-1.10300605	0	0.63450584	0	1	0	13.3711279	13.7237334	13.5314479	13.3669348
926	**ENSG0000006553**	MYLK	protein_coding	chr3	123610049	123884332	-	Human GRCh	OTTHUMG000001	MYLK	**myosin light chain kinase**	Cytoplasm	kinase	K-252	1.521877254	1	0.27575724	0	1	0	6.02561018	5.96036713	6.07630594	6.56747346
927	**ENSG0000006554**	ZC3H15	protein_coding	chr2	186486253	186509361	+	Human GRCh	OTTHUMG000001	ZC3H15	**zinc finger CCCH-type containing 15**	Cytoplasm	translation re		-1.048477584	0	0.82016645	0	1	0	10.497424	10.8900522	10.7693374	10.5318635
928	**ENSG0000006555**	MAP2K4	protein_coding	chr17	12020829	12143830	+	Human GRCh	OTTHUMG000001	MAP2K4	**mitogen-activated protein kinase kinase 4**	Cytoplasm	kinase	cetuximab,	-1.092184943	0	0.68516665	0	1	0	9.96250189	10.2582383	10.2052268	9.80227035
929	**ENSG0000006560**	PACC1	protein_coding	chr1	212363928	212414901	-	Human GRCh	OTTHUMG000000	PACC1	**proton activated chloride channel 1**	Plasma Mem	ion channel		-1.030179633	0	0.88292219	0	1	0	8.84718441	9.02894268	9.02429512	8.78129393
930	**ENSG0000006560**	SNAP91	protein_coding	chr6	83552880	83709691	-	Human GRCh	OTTHUMG000000	SNAP91	**synaptosome associated protein 91**	Plasma Mem	other		-1.252216285	0	0.4817422	0	1	0	8.17073166	8.2188996	7.91929862	7.96424615
931	**ENSG0000006561**	SLK	protein_coding	chr10	103967140	104029233	+	Human GRCh	OTTHUMG000000	SLK	**STE20 like kinase**	Nucleus	kinase		-1.088504479	0	0.6632358	0	1	0	11.0648544	11.3996007	11.2642369	11.0269105
932	**ENSG0000006561**	CYB5R4	protein_coding	chr6	83859656	83967423	+	Human GRCh	OTTHUMG000000	CYB5R4	**cytochrome b5 reductase 4**	Cytoplasm	enzyme		1.227141391	0	0.4304396	0	1	0	7.53800334	7.80538575	7.81534936	7.94827938
934	**ENSG0000006562**	GSTO2	protein_coding	chr10	104268873	104304950	+	Human GRCh	OTTHUMG000000	GSTO2	**glutathione S-transferase omega 2**	Cytoplasm	enzyme		-1.075664969	0	0.8822281	0	1	0	9.91380538	10.2337111	10.1289304	9.84963404
935	**ENSG0000006566**	SEC61A2	protein_coding	chr10	12129637	12169961	+	Human GRCh	OTTHUMG000000	SEC61A2	**SEC61 translocon subunit alpha 2**	Cytoplasm	transporter		-1.135707141	0	0.60750494	0	1	0	8.82062893	8.93854257	8.90477494	8.55609961
936	**ENSG0000006567**	PRKCQ	protein_coding	chr10	6427143	6580301	-	Human GRCh	OTTHUMG000000	PRKCQ	**protein kinase C theta**	Cytoplasm	kinase	sotrastauri	1	0	1	0	NA	0	0.45019658	0.48065103	0.47568569	0.45201103
937	**ENSG0000006571**	TLE2	protein_coding	chr19	2997639	3047635	-	Human GRCh	OTTHUMG000001	TLE2	**TLE family member 2, transcriptional corepressor**	Nucleus	transcription		-1.257549755	0	0.28023658	0	1	0	9.80483286	9.86014004	9.7630158	9.36858395
938	**ENSG0000006580**	ASB1	protein_coding	chr2	238426742	238452250	+	Human GRCh	OTTHUMG000001	ASB1	**ankyrin repeat and SOCS box containing 1**	Nucleus	transcription		-1.040135527	0	0.86654107	0	1	0	10.1031888	10.3621996	10.3019083	10.0807503
939	**ENSG0000006580**	FAM107B	protein_coding	chr10	14518557	14774897	-	Human GRCh	OTTHUMG000000	FAM107B	**family with sequence similarity 107 member B**	Other	other		-1.099850837	0	0.73005973	0	1	0	10.2405819	10.5076309	10.5103481	9.98159726
940	**ENSG0000006583**	ME1	protein_coding	chr6	83210402	83431051	-	Human GRCh	OTTHUMG000000	ME1	**malic enzyme 1**	Cytoplasm	enzyme		3.07861063	1	0.24369409	0	1	0	2.37606415	1.84616993	2.90352041	2.38454806
941	**ENSG0000006588**	TBC1D1	protein_coding	chr4	37891084	38139175	+	Human GRCh	OTTHUMG000001	TBC1D1	**TBC1 domain family member 1**	Nucleus	other		1.105893071	0	0.69815321	0	1	0	10.9608587	10.6996248	10.6003333	11.2132562
942	**ENSG0000006588**	CDK13	protein_coding	chr7	39950121	40099580	+	Human GRCh	OTTHUMG000000	CDK13	**cyclin dependent kinase 13**	Nucleus	kinase	SR4835, TI	1.044437382	0	0.83245262	0	1	0	11.8653128	12.0559064	12.1913345	11.8009663
943	**ENSG0000006591**	MTHFD2	protein_coding	chr2	74186172	74217565	+	Human GRCh	OTTHUMG000001	MTHFD2	**methylenetetrahydrofolate dehydrogenase (NADP+ dependen**	Cytoplasm	enzyme		-1.105746087	0	0.63518449	0	1	0	12.1894748	12.1965776	12.2320743	11.9074884
944	**ENSG0000006592**	SLC9A7	protein_coding	chrX	46599251	46759118	-	Human GRCh	OTTHUMG000000	SLC9A7	**solute carrier family 9 member A7**	Cytoplasm	transporter		1.180949722	0	0.42089353	0	1	0	9.08505312	9.36953179	9.53232769	9.27150398
945	**ENSG0000006597**	FOXJ2	protein_coding	chr12	8032716	8055517	+	Human GRCh	OTTHUMG000001	FOXJ2	**forkhead box J2**	Nucleus	transcription		-1.15285793	0	0.47223188	0	1	0	10.5914547	10.9063443	10.6752707	10.5180086
946	**ENSG0000006597**	YBX1	protein_coding	chr1	42682418	42703805	+	Human GRCh	OTTHUMG000000	YBX1	**Y-box binding protein 1**	Nucleus	transcription		1.0672458	0	0.75777768	0	1	0	12.9557032	13.3346056	13.2646036	13.2019324
947	**ENSG0000006598**	PDE4A	protein_coding	chr19	10416773	10469630	+	Human GRCh	OTTHUMG000001	PDE4A	**phosphodiesterase 4A**	Cytoplasm	enzyme	dyphylline,	-1.028943477	0	0.90949892	0	1	0	10.3267573	10.7408627	10.6329178	10.3894608
948	**ENSG0000006602**	PPP2R5A	protein_coding	chr1	212285410	212361853	+	Human GRCh	OTTHUMG000000	PPP2R5A	**protein phosphatase 2 regulatory subunit B'alpha**	Cytoplasm	phosphatase		1.162566183	0	0.48139771	0	1	0	10.3185232	10.4828096	10.7186194	10.4032077
949	**ENSG0000006603**	CTNNA2	protein_coding	chr2	79185231	80648861	+	Human GRCh	OTTHUMG000001	CTNNA2	**catenin alpha 2**	Plasma Mem	other		1.118951175	0	0.75119767	0	1	0	6.5141855	7.02170562	6.60491734	7.14157856
950	**ENSG0000006604**	ELAVL1	protein_coding	chr19	7958573	8005659	-	Human GRCh	OTTHUMG000001	ELAVL1	**ELAV like RNA binding protein 1**	Cytoplasm	other		-1.05680824	0	0.78608973	0	1	0	11.8331548	12.1768365	12.0676958	11.8319311
952	**ENSG0000006608**	DIP2B	protein_coding	chr12	50504985	50748657	+	Human GRCh	OTTHUMG000001	DIP2B	**disco interacting protein 2 homolog B**	Cytoplasm	other		-1.018932272	0	0.92449163	0	1	0	11.8959448	12.0181113	12.0418988	11.8230196
953	**ENSG0000006611**	SMARCD1	protein_coding	chr12	50085200	50100707	+	Human GRCh	OTTHUMG000001	SMARCD1	**SWI/SNF related, matrix associated, actin dependent regulato**	Nucleus	transcription		-1.180914032	0	0.41872605	0	1	0	11.228483	11.4809562	11.2860365	11.0586146
954	**ENSG0000006613**	KDM4A	protein_coding	chr1	43650149	43705518	+	Human GRCh	OTTHUMG000000	KDM4A	**lysine demethylase 4A**	Nucleus	enzyme		-1.079080206	0	0.69189282	0	1	0	10.7409286	10.8085937	10.8807746	10.467337
955	**ENSG0000006613**	NFYC	protein_coding	chr1	40691648	40771603	+	Human GRCh	OTTHUMG000000	NFYC	**nuclear transcription factor Y subunit gamma**	Nucleus	transcription		-1.105134494	0	0.60277288	0	1	0	10.2918982	10.5244616	10.4219043	10.172652
956	**ENSG0000006618**	ZMYND12	protein_coding	chr1	42430329	42456253	-	Human GRCh	OTTHUMG000000	ZMYND12	**zinc finger MYND-type containing 12**	Other	other		1.260592304	0	0.61103118	0	1	0	5.21310775	5.84691223	6.10176401	5.38088261
957	**ENSG0000006623**	SLC9A3	protein_coding	chr5	470456	524449	-	Human GRCh	OTTHUMG000000	SLC9A3	**solute carrier family 9 member A3**	Plasma Mem	ion channel	AVE0657, t	1.492745777	0	0.32188541	0	1	0	4.76012471	4.32249404	5.2597365	4.4596153
958	**ENSG0000006624**	NGEF	protein_coding	chr2	232878701	233013256	-	Human GRCh	OTTHUMG000001	NGEF	**neuronal guanine nucleotide exchange factor**	Cytoplasm	other		1.3701043	0	0.2597932	0	1	0	6.71285443	6.84417259	7.06472349	7.12487898
959	**ENSG0000006627**	ASPM	protein_coding	chr1	197084121	197146694	-	Human GRCh	OTTHUMG000000	ASPM	**assembly factor for spindle microtubules**	Nucleus	other		-1.256326006	0	0.30951223	0	1	0	14.1866637	14.4687857	14.244671	13.8915131
961	**ENSG0000006632**	ELOVL1	protein_coding	chr1	43363398	43368074	-	Human GRCh	OTTHUMG000000	ELOVL1	**ELOVL fatty acid elongase 1**	Cytoplasm	enzyme		-1.086409345	0	0.6935119	0	1	0	10.7718629	10.9882564	10.9596111	10.5930504
963	**ENSG0000006637**	POLR1H	protein_coding	chr6	30058899	30064909	+	Human GRCh	OTTHUMG000000	POLR1H	**RNA polymerase I subunit H**	Nucleus	transcription		1.062123263	0	0.80903909	0	1	0	7.74857618	8.01734733	8.01059838	7.89701762
964	**ENSG0000006638**	MPPED2	protein_coding	chr11	30384493	30586872	-	Human GRCh	OTTHUMG000001	MPPED2	**metallophosphoesterase domain containing 2**	Other	enzyme		1.572511889	1	0.61782191	0	1	0	6.37824598	7.24388166	7.6648415	7.08369963
966	**ENSG0000006642**	ZBTB11	protein_coding	chr3	101648889	101677135	-	Human GRCh	OTTHUMG000001	ZBTB11	**zinc finger and BTB domain containing 11**	Nucleus	transcription		1.179140423	0	0.41064233	0	1	0	10.4628447	10.8931687	11.0667285	10.6555887
967	**ENSG0000006642**	ATXN3	protein_coding	chr14	92044496	92106621	-	Human GRCh	OTTHUMG000001	ATXN3	**ataxin 3**	Nucleus	peptidase		-1.200300406	0	0.42004526	0	1	0	9.9770251	10.3131374	10.1152647	9.77965082
968	**ENSG0000006645**	GOLGA5	protein_coding	chr14	92794305	92839947	+	Human GRCh	OTTHUMG000001	GOLGA5	**golgin A5**	Cytoplasm	kinase		-1.118095529	0	0.61357375	0	1	0	10.1202102	10.5074793	10.3336952	10.0644548
969	**ENSG0000006646**	FGFR2	protein_coding	chr10	121478332	121598458	-	Human GRCh	OTTHUMG000000	FGFR2	**fibroblast growth factor receptor 2**	Plasma Mem	kinase	selpercatir	1.04723148	0	0.89754801	0	1	0	6.36274874	6.37050851	6.47392736	6.34537739
970	**ENSG0000006655**	LRRC40	protein_coding	chr1	70144805	70205579	-	Human GRCh	OTTHUMG000000	LRRC40	**leucine rich repeat containing 40**	Nucleus	other		1.010778648	0	0.96311283	0	1	0	9.33486218	9.59922713	9.64886344	9.29749968
971	**ENSG0000006658**	ISOC1	protein_coding	chr5	129094749	129114028	+	Human GRCh	OTTHUMG000001	ISOC1	**isochorismatase domain containing 1**	Cytoplasm	enzyme		-1.053341299	0	0.82016375	0	1	0	9.90633335	10.302699	10.1256268	9.99503295
972	**ENSG0000006662**	EML1	protein_coding	chr14	99737693	99942060	+	Human GRCh	OTTHUMG000001	EML1	**EMAP like 1**	Cytoplasm	other		-1.49801471	0	0.18584875	0	1	0	10.3263729	10.3934812	9.93483739	9.84454386
973	**ENSG0000006665**	TRMT11	protein_coding	chr6	125986479	126203817	+	Human GRCh	OTTHUMG000000	TRMT11	**tRNA methyltransferase 11 homolog**	Other	enzyme		1.025869609	0	0.92333775	0	1	0	9.15200455	9.44475177	9.56739036	9.04342374
974	**ENSG0000006665**	THUMPD1	protein_coding	chr16	20702816	20742084	-	Human GRCh	OTTHUMG000001	THUMPD1	**THUMP domain containing 1**	Nucleus	other		1.030821562	0	0.88147269	0	1	0	10.3853614	10.6869422	10.7312879	10.4046771
975	**ENSG0000006669**	MSANTD3	protein_coding	chr9	100427143	100451734	+	Human GRCh	OTTHUMG000000	MSANTD3	**Myb/SANT DNA binding domain containing 3**	Other	other		-1.001277969	0	0.99551237	0	1	0	8.02568513	8.22866076	8.33152945	7.88737913
976	**ENSG0000006673**	KIF26A	protein_coding	chr14	104136454	104180894	+	Human GRCh	OTTHUMG000001	KIF26A	**kinesin family member 26A**	Cytoplasm	other		1.961660649	1	0.26129599	0	1	0	4.9850409	5.52622188	5.8142699	5.29331385
977	**ENSG0000006673**	ATG2B	protein_coding	chr14	96279195	96363341	-	Human GRCh	OTTHUMG000001	ATG2B	**autophagy related 2B**	Cytoplasm	transporter		-1.109363088	0	0.64631648	0	1	0	11.0936172	11.3035211	11.2389159	10.92199
978	**ENSG0000006677**	ARFGEF1	protein_coding	chr8	67173511	67343781	-	Human GRCh	OTTHUMG000001	ARFGEF1	**ADP ribosylation factor guanine nucleotide exchange factor 1**	Cytoplasm	other		-1.082588715	0	0.71468184	0	1	0	11.5956497	11.9612706	11.8527846	11.5412634
980	**ENSG0000006682**	ZFAT	protein_coding	chr8	134477788	134713049	-	Human GRCh	OTTHUMG000001	ZFAT	**zinc finger and AT-hook domain containing**	Nucleus	transcription		1.042350871	0	0.85458141	0	1	0	9.15715506	9.34840703	9.51531903	9.03804886
981	**ENSG0000006685**	MTFR1	protein_coding	chr8	65644734	65771261	+	Human GRCh	OTTHUMG000001	MTFR1	**mitochondrial fission regulator 1**	Cytoplasm	other		1.006817293	0	0.9736339	0	1	0	10.4428844	10.6743385	10.6971024	10.4315064
982	**ENSG0000006692**	STAG3	protein_coding	chr7	100177563	100214387	+	Human GRCh	OTTHUMG000001	STAG3	**STAG3 cohesin complex component**	Nucleus	other		-1.242419996	0	0.72081354	0	1	0	4.30234709	4.80838231	4.32085507	4.45646573
983	**ENSG0000006692**	FECH	protein_coding	chr18	57544377	57586732	-	Human GRCh	OTTHUMG000001	FECH	**ferrochelatase**	Cytoplasm	enzyme		-1.028278039	0	0.90747594	0	1	0	9.92609801	10.254728	10.256403	9.85076258
984	**ENSG0000006693**	MYO9A	protein_coding	chr15	71822291	72118577	-	Human GRCh	OTTHUMG000001	MYO9A	**myosin IXA**	Cytoplasm	enzyme		-1.060801914	0	0.78385602	0	1	0	11.0860058	11.2913543	11.3210053	10.8982328
985	**ENSG0000006704**	DDX3Y	protein_coding	chrY	12904108	12920478	+	Human GRCh	OTTHUMG000000	DDX3Y	**DEAD-box helicase 3 Y-linked**	Cytoplasm	enzyme		-1.009506656	0	0.96537575	0	1	0	10.6198378	10.8860811	10.9575688	10.4973764
986	**ENSG0000006705**	PFKP	protein_coding	chr10	3066333	3137718	+	Human GRCh	OTTHUMG000000	PFKP	**phosphofructokinase, platelet**	Cytoplasm	kinase		1.188486713	0	0.60385986	0	1	0	8.8142546	9.52230859	8.90152279	9.74129682

987	**ENSG0000006706**	IDI1	protein_coding	chr10	1039152	1049119	-	Human GRCh	OTTHUMG000000	IDI1	**isopentenyl-diphosphate delta isomerase 1**	Cytoplasm	enzyme		-1.054867701	0	0.82973826	0	1	0	10.9085164	11.4093644	11.2676237	10.9610858
988	**ENSG0000006706**	SP100	protein_coding	chr2	230415942	230545606	+	Human GRCh	OTTHUMG000001	SP100	**SP100 nuclear antigen**	Nucleus	transcription		1.826087398	1	0.1739037	0	1	0	3.51272828	4.38704143	3.89848252	4.85726133
989	**ENSG0000006708**	KLF6	protein_coding	chr10	3775996	3785281	-	Human GRCh	OTTHUMG000000	KLF6	**KLF transcription factor 6**	Nucleus	transcription		-1.140779939	0	0.54858684	0	1	0	10.5508429	10.6316964	10.3333724	10.5521081
990	**ENSG0000006711**	PLPP1	protein_coding	chr5	55424854	55534969	-	Human GRCh	OTTHUMG000001	PLPP1	**phospholipid phosphatase 1**	Plasma Mem	phosphatase		-1.195810669	0	0.56813736	0	1	0	8.36962713	8.40696219	8.21361958	8.17285161
991	**ENSG0000006714**	NEO1	protein_coding	chr15	73051710	73305205	+	Human GRCh	OTTHUMG000001	NEO1	**neogenin 1**	Plasma Mem	transmembra		-1.239887175	0	0.39118004	0	1	0	10.3101591	10.5893778	10.3502956	10.0887859
992	**ENSG0000006716**	TRAM1	protein_coding	chr8	70573218	70608416	-	Human GRCh	OTTHUMG000001	TRAM1	**translocation associated membrane protein 1**	Cytoplasm	other		-1.023301154	0	0.91790289	0	1	0	11.7281468	11.9076191	12.0007477	11.5475717
993	**ENSG0000006717**	PHKA1	protein_coding	chrX	72578814	72714319	-	Human GRCh	OTTHUMG000000	PHKA1	**phosphorylase kinase regulatory subunit alpha 1**	Cytoplasm	kinase		-1.247470296	0	0.30486284	0	1	0	10.2016311	10.5921071	10.323556	9.97119648
994	**ENSG0000006718**	TNFRSF1A	protein_coding	chr12	6328757	6342114	-	Human GRCh	OTTHUMG000001	TNFRSF1A	**TNF receptor superfamily member 1A**	Plasma Mem	transmembra	GSK28622	-1.233046972	0	0.40183025	0	1	0	9.80575169	10.0970457	9.70864348	9.7433457
995	**ENSG0000006719**	CACNB1	protein_coding	chr17	39173453	39197702	-	Human GRCh	OTTHUMG000001	CACNB1	**calcium voltage-gated channel auxiliary subunit beta 1**	Plasma Mem	ion channel	verapamil,	1.158806052	0	0.68287113	0	1	0	8.12797811	8.27708267	8.37986194	8.34455737
996	**ENSG0000006720**	EVI5	protein_coding	chr1	92508696	92792404	-	Human GRCh	OTTHUMG000000	EVI5	**ecotropic viral integration site 5**	Cytoplasm	other		1.057596414	0	0.76678611	0	1	0	9.95865866	10.1607244	10.2721265	9.95824541
997	**ENSG0000006722**	STOML1	protein_coding	chr15	73978926	73994622	-	Human GRCh	OTTHUMG000001	STOML1	**stomatin like 1**	Other	other		1.276900425	0	0.33966035	0	1	0	7.69137365	7.73501094	8.1220524	7.81406424
998	**ENSG0000006722**	PKM	protein_coding	chr15	72199029	72231819	-	Human GRCh	OTTHUMG000001	PKM	**pyruvate kinase M1/2**	Cytoplasm	kinase	TP-1454, C	-1.134959012	0	0.56642832	0	1	0	14.1735088	14.6175795	14.3448874	14.1958873
999	**ENSG0000006724**	DHX29	protein_coding	chr5	55256055	55307694	-	Human GRCh	OTTHUMG000001	DHX29	**DExH-box helicase 29**	Cytoplasm	enzyme		-1.29008469	0	0.21078564	0	1	0	10.325304	10.7053269	10.3442161	10.1390068
1000	**ENSG0000006733**	DNTTIP2	protein_coding	chr1	93866284	93879918	-	Human GRCh	OTTHUMG000000	DNTTIP2	**deoxynucleotidyltransferase terminal interacting protein 2**	Nucleus	other		1.002618486	0	0.98901984	0	1	0	10.1012539	10.4093673	10.4164671	10.0985502
1001	**ENSG0000006736**	METTL22	protein_coding	chr16	8621683	8649654	+	Human GRCh	OTTHUMG000001	METTL22	**methyltransferase 22, Kin17 lysine**	Nucleus	enzyme		-1.242430962	0	0.36155809	0	1	0	10.1455352	10.3871128	10.1755203	9.87502377
1002	**ENSG0000006736**	TP53BP1	protein_coding	chr15	43403061	43510728	-	Human GRCh	OTTHUMG000000	TP53BP1	**tumor protein p53 binding protein 1**	Nucleus	transcription		-1.119410635	0	0.55720461	0	1	0	11.5078237	11.650901	11.6159336	11.2685992
1003	**ENSG0000006744**	TRO	protein_coding	chrX	54920462	54931431	+	Human GRCh	OTTHUMG000000	TRO	**trophinin**	Plasma Mem	other		1	0	1	0	NA	0	0.46655447	0.48097545	0.47858269	0.46739657
1004	**ENSG0000006753**	RRP15	protein_coding	chr1	218285293	218337983	+	Human GRCh	OTTHUMG000000	RRP15	**ribosomal RNA processing 15 homolog**	Nucleus	other		1.158695654	0	0.53128397	0	1	0	10.0113591	10.3168421	10.5568403	10.0633102
1005	**ENSG0000006756**	RHOA	protein_coding	chr3	49359139	49412998	-	Human GRCh	OTTHUMG000001	RHOA	**ras homolog family member A**	Cytoplasm	enzyme		-1.107265027	0	0.60380515	0	1	0	12.7868051	13.1746284	12.9987428	12.756664
1006	**ENSG0000006759**	DHX8	protein_coding	chr17	43483865	43610338	+	Human GRCh	OTTHUMG000001	DHX8	**DEAH-box helicase 8**	Nucleus	enzyme		-1.318097416	0	0.16187915	0	1	0	10.995306	11.3163665	10.9554505	10.7570156
1007	**ENSG0000006760**	PRKCZ	protein_coding	chr1	2050411	2185395	+	Human GRCh	OTTHUMG000000	PRKCZ	**protein kinase C zeta**	Cytoplasm	kinase	pkc-zeta in	-1.064201419	0	0.83682467	0	1	0	11.5194818	11.7316519	11.6990308	11.400904
1008	**ENSG0000006764**	ZFY	protein_coding	chrY	2935281	2982506	+	Human GRCh	OTTHUMG000000	ZFY	**zinc finger protein Y-linked**	Nucleus	transcription		-1.161898394	0	0.57830105	0	1	0	7.86992302	8.30555749	8.02580355	7.82339776
1009	**ENSG0000006770**	IARS2	protein_coding	chr1	220094132	220148041	+	Human GRCh	OTTHUMG000000	IARS2	**isoleucyl-tRNA synthetase 2, mitochondrial**	Cytoplasm	enzyme		-1.084737157	0	0.73020175	0	1	0	12.3340352	12.7934953	12.6805643	12.2695802
1010	**ENSG0000006771**	SYT1	protein_coding	chr12	78863993	79452008	+	Human GRCh	OTTHUMG000001	SYT1	**synaptotagmin 1**	Cytoplasm	transporter		1.932979857	1	0.19553303	0	1	0	5.54105226	6.21293509	6.7366357	6.43687559
1011	**ENSG0000006779**	NAV3	protein_coding	chr12	77324641	78213010	+	Human GRCh	OTTHUMG000001	NAV3	**neuron navigator 3**	Nucleus	other		-1.422054755	0	0.53291378	0	1	0	5.7535092	5.92672936	5.89229686	4.83835625
1012	**ENSG0000006782**	IDH3G	protein_coding	chrX	153785766	153794512	-	Human GRCh	OTTHUMG000000	IDH3G	**isocitrate dehydrogenase (NAD(+)) 3 non-catalytic subunit gan**	Cytoplasm	enzyme		-1.056639841	0	0.81179657	0	1	0	10.3468478	10.6490148	10.4583542	10.4333889
1013	**ENSG0000006783**	ROGDI	protein_coding	chr16	4796968	4802880	-	Human GRCh	OTTHUMG000001	ROGDI	**rogdi atypical leucine zipper**	Nucleus	other		1.242059201	0	0.43430306	0	1	0	8.69668524	8.82015819	8.94604183	9.04685577
1014	**ENSG0000006784**	PDZD4	protein_coding	chrX	153802166	153830565	-	Human GRCh	OTTHUMG000000	PDZD4	**PDZ domain containing 4**	Cytoplasm	other		-1.251164436	0	0.49557262	0	1	0	5.35698458	5.41788246	4.99988938	5.3373017
1015	**ENSG0000006784**	ATP2B3	protein_coding	chrX	153517642	153582939	+	Human GRCh	OTTHUMG000000	ATP2B3	**ATPase plasma membrane Ca2+ transporting 3**	Plasma Mem	transporter		-1.139997184	0	0.7993845	0	1	0	6.82585109	6.14887188	6.39859459	6.41568445
1016	**ENSG0000006790**	ROCK1	protein_coding	chr18	20944612	21111813	-	Human GRCh	OTTHUMG000001	ROCK1	**Rho associated coiled-coil containing protein kinase 1**	Cytoplasm	kinase	ripasudil, (	-1.007009415	0	0.96899469	0	1	0	11.7055055	11.9287612	11.9710697	11.6343606
1017	**ENSG0000006795**	CBFB	protein_coding	chr16	67028984	67101058	+	Human GRCh	OTTHUMG000001	CBFB	**core-binding factor subunit beta**	Nucleus	transcription		1.143248081	0	0.55122787	0	1	0	10.030156	10.3810628	10.5881341	10.0922794
1018	**ENSG0000006799**	PDK3	protein_coding	chrX	24465244	24550466	+	Human GRCh	OTTHUMG000000	PDK3	**pyruvate dehydrogenase kinase 3**	Cytoplasm	kinase		1.087369533	0	0.72220742	0	1	0	10.772328	11.2026955	11.1698869	11.0219258
1019	**ENSG0000006800**	HYAL2	protein_coding	chr3	50317790	50322782	-	Human GRCh	OTTHUMG000001	HYAL2	**hyaluronidase 2**	Cytoplasm	enzyme	hyaluronic	1.003917461	0	0.98588099	0	1	0	9.18178412	9.36435517	9.31588458	9.24446328
1020	**ENSG0000006802**	HDAC4	protein_coding	chr2	239048168	239401654	-	Human GRCh	OTTHUMG000001	HDAC4	**histone deacetylase 4**	Nucleus	transcription	tributyrin,	-1.202350491	0	0.42689358	0	1	0	9.98007368	10.1825575	9.99978433	9.75642715
1021	**ENSG0000006802**	RASSF1	protein_coding	chr3	50329782	50340980	-	Human GRCh	OTTHUMG000001	RASSF1	**Ras association domain family member 1**	Nucleus	other		1.029626075	0	0.8968435	0	1	0	9.2679055	9.50658737	9.63335055	9.17308079
1022	**ENSG0000006807**	FGFR3	protein_coding	chr4	1793293	1808872	+	Human GRCh	OTTHUMG000001	FGFR3	**fibroblast growth factor receptor 3**	Plasma Mem	kinase	docetaxel/	1.143146662	0	0.68541224	0	1	0	5.73741692	5.68109677	5.76882638	5.88481022
1023	**ENSG0000006807**	IFI35	protein_coding	chr17	43006740	43014456	+	Human GRCh	OTTHUMG000001	IFI35	**interferon induced protein 35**	Nucleus	other		1.630086401	1	0.11751785	0	1	0	5.42315549	5.88133069	6.15552375	6.13071472
1024	**ENSG0000006809**	HEATR6	protein_coding	chr17	60041008	60078922	-	Human GRCh	OTTHUMG000001	HEATR6	**HEAT repeat containing 6**	Other	other		-1.094709535	0	0.65221347	0	1	0	9.37204323	9.39149934	9.42541743	9.12296859
1025	**ENSG0000006812**	COASY	protein_coding	chr17	42561467	42566277	+	Human GRCh	OTTHUMG000001	COASY	**Coenzyme A synthase**	Cytoplasm	kinase		-1.07455703	0	0.71510442	0	1	0	11.173843	11.3929762	11.3432631	11.0533062
1026	**ENSG0000006813**	PLEKHH3	protein_coding	chr17	42667914	42676994	-	Human GRCh	OTTHUMG000001	PLEKHH3	**pleckstrin homology, MyTH4 and FERM domain containing H3**	Extracellular	other		1.124399329	0	0.5214464	0	1	0	10.8674853	11.0608561	11.2173119	10.9700241
1027	**ENSG0000006830**	MEF2A	protein_coding	chr15	99565417	99716488	+	Human GRCh	OTTHUMG000001	MEF2A	**myocyte enhancer factor 2A**	Nucleus	transcription		-1.045284938	0	0.84080089	0	1	0	10.74849	11.0648296	10.9364114	10.7964132
1028	**ENSG0000006830**	OTUD5	protein_coding	chrX	48922024	48958386	-	Human GRCh	OTTHUMG000000	OTUD5	**OTU deubiquitinase 5**	Cytoplasm	enzyme		-1.098688008	0	0.62977844	0	1	0	10.7473279	11.0268045	10.8500396	10.7264542
1029	**ENSG0000006832**	TFE3	protein_coding	chrX	49028726	49043410	-	Human GRCh	OTTHUMG000000	TFE3	**transcription factor binding to IGHM enhancer 3**	Nucleus	transcription		-1.026871123	0	0.8991853	0	1	0	9.79623779	10.0114661	10.0241371	9.7147118
1030	**ENSG0000006835**	TBC1D25	protein_coding	chrX	48539714	48562609	+	Human GRCh	OTTHUMG000000	TBC1D25	**TBC1 domain family member 25**	Cytoplasm	other		-1.246767027	0	0.33742151	0	1	0	9.22709304	9.49913778	9.17284281	9.07974356
1031	**ENSG0000006836**	ACSL4	protein_coding	chrX	109624244	109733403	-	Human GRCh	OTTHUMG000000	ACSL4	**acyl-CoA synthetase long chain family member 4**	Cytoplasm	enzyme		-1.059658954	0	0.8518063	0	1	0	8.18046744	8.50055752	8.24247732	8.3362259
1032	**ENSG0000006838**	INPP5A	protein_coding	chr10	132537787	132783480	+	Human GRCh	OTTHUMG000000	INPP5A	**inositol polyphosphate-5-phosphatase A**	Plasma Mem	phosphatase		-1.122784517	0	0.63163549	0	1	0	8.40687841	8.60681996	8.4620983	8.30618917
1033	**ENSG0000006839**	GPKOW	protein_coding	chrX	49113407	49123735	-	Human GRCh	OTTHUMG000000	GPKOW	**G-patch domain and KOW motifs**	Nucleus	other		-1.095234984	0	0.68632993	0	1	0	9.0799601	9.49770326	9.30882295	9.09424662
1034	**ENSG0000006840**	GRIPAP1	protein_coding	chrX	48973720	49002265	-	Human GRCh	OTTHUMG000000	GRIPAP1	**GRIP1 associated protein 1**	Plasma Mem	other		-1.09232507	0	0.68405034	0	1	0	10.7175587	11.0594068	10.9362066	10.6580033
1035	**ENSG0000006843**	FTSJ1	protein_coding	chrX	48476021	48486364	+	Human GRCh	OTTHUMG000000	FTSJ1	**FtsJ RNA 2'-O-methyltransferase 1**	Cytoplasm	enzyme		-1.2253975	0	0.34699555	0	1	0	10.2934125	10.6472275	10.3955315	10.0975151
1036	**ENSG0000006848**	PRR11	protein_coding	chr17	59155732	59206709	+	Human GRCh	OTTHUMG000001	PRR11	**proline rich 11**	Cytoplasm	other		-1.255374839	0	0.26771112	0	1	0	10.4739906	10.8919903	10.4731164	10.423997
1037	**ENSG0000006861**	REEP1	protein_coding	chr2	86213993	86338083	-	Human GRCh	OTTHUMG000001	REEP1	**receptor accessory protein 1**	Cytoplasm	other		-1.03821062	0	0.89092963	0	1	0	7.29190445	7.5358128	7.5207281	7.22307468
1038	**ENSG0000006865**	ATP11A	protein_coding	chr13	112690038	112887168	+	Human GRCh	OTTHUMG000000	ATP11A	**ATPase phospholipid transporting 11A**	Plasma Mem	transporter		-1.111247421	0	0.59369784	0	1	0	11.133114	11.3490322	11.2786442	10.9585842
1039	**ENSG0000006865**	POLR1A	protein_coding	chr2	86020216	86106155	-	Human GRCh	OTTHUMG000001	POLR1A	**RNA polymerase I subunit A**	Nucleus	enzyme		-1.138919155	0	0.56024216	0	1	0	12.6193448	13.0130773	12.7927177	12.5717079
1040	**ENSG0000006869**	LAPTM4A	protein_coding	chr2	20032650	20051628	-	Human GRCh	OTTHUMG000000	LAPTM4A	**lysosomal protein transmembrane 4 alpha**	Cytoplasm	other		-1.232353043	0	0.34069871	0	1	0	10.6016801	10.8834824	10.645056	10.3737704
1041	**ENSG0000006872**	TTC7A	protein_coding	chr2	46915869	47076137	+	Human GRCh	OTTHUMG000001	TTC7A	**tetratricopeptide repeat domain 7A**	Plasma Mem	other		-1.0619529	0	0.83563045	0	1	0	8.78243309	9.01344692	8.87850822	8.80089566
1042	**ENSG0000006874**	IP6K2	protein_coding	chr3	48688003	48740353	-	Human GRCh	OTTHUMG000001	IP6K2	**inositol hexakisphosphate kinase 2**	Cytoplasm	kinase		-1.10208438	0	0.62489667	0	1	0	11.1216175	11.268726	11.1874582	10.9852937
1043	**ENSG0000006878**	SRBD1	protein_coding	chr2	45388680	45612165	-	Human GRCh	OTTHUMG000001	SRBD1	**S1 RNA binding domain 1**	Extracellular	other		-1.063398169	0	0.77050593	0	1	0	8.65470018	8.78444361	8.77743792	8.52203598
1044	**ENSG0000006879**	KIF2A	protein_coding	chr5	62306162	62391025	+	Human GRCh	OTTHUMG000000	KIF2A	**kinesin family member 2A**	Cytoplasm	other		-1.025973699	0	0.90698197	0	1	0	11.037653	11.359782	11.3902839	10.9277033
1045	**ENSG0000006883**	RASGRP2	protein_coding	chr11	64726911	64745456	-	Human GRCh	OTTHUMG000000	RASGRP2	**RAS guanyl releasing protein 2**	Cytoplasm	other		-1.51779739	-1	0.50474028	0	1	0	4.03165542	4.10913117	3.54486364	3.89340626
1046	**ENSG0000006887**	PSME4	protein_coding	chr2	53864069	53970993	-	Human GRCh	OTTHUMG000001	PSME4	**proteasome activator subunit 4**	Cytoplasm	other		-1.046681097	0	0.82561745	0	1	0	12.1736808	12.4818348	12.4217495	12.1349158
1047	**ENSG0000006888**	IFT80	protein_coding	chr3	160256986	160399880	-	Human GRCh	OTTHUMG000001	IFT80	**intraflagellar transport 80**	Extracellular	other		1.007792704	0	0.97366689	0	1	0	9.93095903	10.0292731	10.2066228	9.72223939
1048	**ENSG0000006890**	SIRT2	protein_coding	chr19	38878555	38899862	-	Human GRCh	OTTHUMG000001	SIRT2	**sirtuin 2**	Nucleus	transcription		1.040509502	0	0.84996362	0	1	0	11.6112522	12.01461	12.0163308	11.7152055
1049	**ENSG0000006891**	ERLEC1	protein_coding	chr2	53787009	53833038	+	Human GRCh	OTTHUMG000001	ERLEC1	**endoplasmic reticulum lectin 1**	Cytoplasm	other		-1.01907473	0	0.92801799	0	1	0	9.79568792	9.99300821	10.0599812	9.66209711
1050	**ENSG0000006897**	PPP2R5B	protein_coding	chr11	64917553	64934475	+	Human GRCh	OTTHUMG000001	PPP2R5B	**protein phosphatase 2 regulatory subunit B'beta**	Cytoplasm	phosphatase		1.01635226	0	0.96376308	0	1	0	8.77898249	9.24382389	9.07894388	9.02463889
1051	**ENSG0000006897**	PYGM	protein_coding	chr11	64746389	64759974	-	Human GRCh	OTTHUMG000000	PYGM	**glycogen phosphorylase, muscle associated**	Cytoplasm	enzyme		-1.611791132	-1	0.15468095	0	1	0	6.18532115	6.2477735	5.66231206	5.83113163
1052	**ENSG0000006898**	PAGE1	protein_coding	chrX	49687447	49695984	-	Human GRCh	OTTHUMG000000	PAGE1	**PAGE family member 1**	Other	other		1.590794001	1	0.59200699	0	1	0	3.18448904	3.52564571	4.40391785	2.60170879
1053	**ENSG0000006901**	PITX1	protein_coding	chr5	135027734	135034813	-	Human GRCh	OTTHUMG000001	PITX1	**paired like homeodomain 1**	Nucleus	transcription		1.281473547	0	0.24191722	0	1	0	9.04271498	9.21509759	9.16527321	9.60109292
1055	**ENSG0000006902**	MAST4	protein_coding	chr5	66596380	67169593	+	Human GRCh	OTTHUMG000001	MAST4	**microtubule associated serine/threonine kinase family membe**	Other	kinase		-1.236059419	0	0.44813269	0	1	0	10.2785153	10.7588482	10.5504527	10.0097471
1056	**ENSG0000006912**	ADGRF5	protein_coding	chr6	46852522	46954943	-	Human GRCh	OTTHUMG000000	ADGRF5	**adhesion G protein-coupled receptor F5**	Plasma Mem	G-protein co		-1.084453656	0	0.92807303	0	1	0	1.27397486	2.5704851	1.34881077	2.4166623
1057	**ENSG0000006918**	SDK2	protein_coding	chr17	73334384	73644445	-	Human GRCh	OTTHUMG000001	SDK2	**sidekick cell adhesion molecule 2**	Plasma Mem	other		-1.559197688	-1	0.50829511	0	1	0	10.082007	10.7813017	10.2771475	9.6125874
1058	**ENSG0000006920**	ADAM7	protein_coding	chr8	24440930	24526970	+	Human GRCh	OTTHUMG000000	ADAM7	**ADAM metallopeptidase domain 7**	Plasma Mem	peptidase		5.24319142	1	0.71884804	0	NA	0	0.28949998	1.26049366	1.74863563	2.24086841
1059	**ENSG0000006924**	NUP133	protein_coding	chr1	229440259	229508341	-	Human GRCh	OTTHUMG000000	NUP133	**nucleoporin 133**	Nucleus	other		-1.132905351	0	0.54634443	0	1	0	11.6741558	11.9958478	11.8379997	11.5580022
1060	**ENSG0000006927**	NUCKS1	protein_coding	chr1	205712822	205750182	-	Human GRCh	OTTHUMG000000	NUCKS1	**nuclear casein kinase and cyclin dependent kinase substrate 1**	Nucleus	kinase		-1.127322917	0	0.60618163	0	1	0	13.8418532	14.1258867	14.0514087	13.6363657
1061	**ENSG0000006932**	VPS35	protein_coding	chr16	46656132	46689518	-	Human GRCh	OTTHUMG000001	VPS35	**VPS35 retromer complex component**	Cytoplasm	transporter		-1.201716953	0	0.40356054	0	1	0	12.088911	12.4511988	12.2261322	11.9041932
1062	**ENSG0000006934**	DNAJA2	protein_coding	chr16	46955362	46973674	-	Human GRCh	OTTHUMG000001	DNAJA2	**DnaJ heat shock protein family (Hsp40) member A2**	Nucleus	enzyme		-1.035296285	0	0.88284721	0	1	0	10.398756	10.7559619	10.7135162	10.363966
1063	**ENSG0000006939**	BCL3	protein_coding	chr19	44747705	44760044	+	Human GRCh	OTTHUMG000001	BCL3	**BCL3 transcription coactivator**	Nucleus	transcription		-1.086791401	0	0.79669763	0	1	0	6.31472608	6.78335453	6.58865321	6.36805999
1064	**ENSG0000006942**	KCNAB2	protein_coding	chr1	5990927	6101193	+	Human GRCh	OTTHUMG000000	KCNAB2	**potassium voltage-gated channel subfamily A regulatory beta**	Plasma Mem	ion channel		1.159022739	0	0.48604415	0	1	0	8.78231012	9.03974603	9.11001385	9.03123592
1065	**ENSG0000006943**	ABCC9	protein_coding	chr12	21797389	21942543	-	Human GRCh	OTTHUMG000001	ABCC9	**ATP binding cassette subfamily C member 9**	Plasma Mem	ion channel		-1.703009306	-1	0.67957913	0	NA	0	3.36100459	3.35247452	2.94494307	2.86500112
1066	**ENSG0000006948**	GAL	protein_coding	chr11	68683779	68691175	+	Human GRCh	OTTHUMG000001	GAL	**galanin and GMAP prepropeptide**	Extracellular	other		-1.234008839	0	0.52192716	0	1	0	8.03188661	8.17773315	7.72319256	7.99469098
1067	**ENSG0000006949**	CLEC2D	protein_coding	chr12	9664969	9699553	+	Human GRCh	OTTHUMG000001	CLEC2D	**C-type lectin domain family 2 member D**	Plasma Mem	transmembra		-1.234626876	0	0.57783445	0	1	0	7.67860419	7.99156882	7.85350075	7.34282546
1068	**ENSG0000006950**	FUNDC1	protein_coding	chrX	44523639	44542859	-	Human GRCh	OTTHUMG000000	FUNDC1	**FUN14 domain containing 1**	Cytoplasm	other		-1.057618999	0	0.84673675	0	1	0	7.85254606	7.97670493	8.08276508	7.57404883
1070	**ENSG0000006966**	RORA	protein_coding	chr15	60488284	61229302	-	Human GRCh	OTTHUMG000001	RORA	**RAR related orphan receptor A**	Nucleus	ligand-depen		-1.038828273	0	0.91019148	0	1	0	9.31183802	9.61245805	9.36509477	9.49260238
1071	**ENSG0000006969**	DRD4	protein_coding	chr11	637269	640706	+	Human GRCh	OTTHUMG000001	DRD4	**dopamine receptor D4**	Plasma Mem	G-protein co	ziprasidon	1.00600048	0	0.98483972	0	1	0	8.08612568	8.73366044	8.38311279	8.53136659

1072	**ENSG0000006970**	TGFBR3	protein_coding	chr1	91680343	91906335	-	Human GRCh	OTTHUMG000000	TGFBR3	**transforming growth factor beta receptor 3**	Plasma Mem	kinase		-1.410701571	0	0.33938407	0	1	0	11.0508546	11.3367398	11.1319879	10.3323463
1073	**ENSG0000006976**	PLA2G10	protein_coding	chr16	14672548	14694308	-	Human GRCh	OTTHUMG000000	PLA2G10	**phospholipase A2 group X**	Cytoplasm	enzyme	varespladil	1.09052281	0	0.89669955	0	1	0	4.79103012	5.01387026	5.00107386	4.98464841
1074	**ENSG0000006981**	HES2	protein_coding	chr1	6412418	6424670	-	Human GRCh	OTTHUMG000000	HES2	**hes family bHLH transcription factor 2**	Nucleus	transcription		1.324839948	0	0.60500204	0	1	0	3.61618574	5.36091277	5.14211916	4.91438955
1075	**ENSG0000006984**	ATP1B3	protein_coding	chr3	141876124	141926549	+	Human GRCh	OTTHUMG000001	ATP1B3	**ATPase Na+/K+ transporting subunit beta 3**	Plasma Mem	transporter		-1.039023037	0	0.86622099	0	1	0	9.94624345	10.2651746	10.3063034	9.78411111
1076	**ENSG0000006986**	NEDD4	protein_coding	chr15	55826922	55993660	-	Human GRCh	OTTHUMG000001	NEDD4	**NEDD4 E3 ubiquitin protein ligase**	Cytoplasm	enzyme		1.082382265	0	0.79191042	0	1	0	7.87149538	7.80115639	7.73378757	8.08377221
1077	**ENSG0000006994**	PIGB	protein_coding	chr15	55318960	55355648	+	Human GRCh	OTTHUMG000001	PIGB	**phosphatidylinositol glycan anchor biosynthesis class B**	Cytoplasm	enzyme		-1.377218668	0	0.22474945	0	1	0	8.51192535	8.73540775	8.4462345	8.07900716
1078	**ENSG0000006995**	MAPK6	protein_coding	chr15	51952106	52067375	+	Human GRCh	OTTHUMG000001	MAPK6	**mitogen-activated protein kinase 6**	Cytoplasm	kinase		-1.439025858	0	0.13856392	0	1	0	11.825335	12.0792973	11.6778508	11.391379
1079	**ENSG0000006996**	GNB5	protein_coding	chr15	52115100	52191392	-	Human GRCh	OTTHUMG000001	GNB5	**G protein subunit beta 5**	Plasma Mem	other		1.073125817	0	0.77591473	0	1	0	7.95739272	8.40794737	8.34280255	8.20603419
1080	**ENSG0000006997**	RAB27A	protein_coding	chr15	55202966	55319113	-	Human GRCh	OTTHUMG000001	RAB27A	**RAB27A, member RAS oncogene family**	Cytoplasm	enzyme		-1.004082865	0	0.98736321	0	1	0	9.27439825	9.59083392	9.77836281	8.95958047
1081	**ENSG0000006999**	HDHD5	protein_coding	chr22	17137511	17165287	-	Human GRCh	OTTHUMG000001	HDHD5	**haloacid dehalogenase like hydrolase domain containing 5**	Cytoplasm	other		1.016139105	0	0.94431269	0	1	0	10.3412346	10.5677376	10.6947569	10.2122299
1082	**ENSG0000007001**	UFD1	protein_coding	chr22	19449911	19479202	-	Human GRCh	OTTHUMG000001	UFD1	**ubiquitin recognition factor in ER associated degradation 1**	Cytoplasm	peptidase		-1.244426302	0	0.28041332	0	1	0	10.988399	11.445496	11.0395015	10.9423482
1083	**ENSG0000007001**	LRP6	protein_coding	chr12	12116025	12267044	-	Human GRCh	OTTHUMG000001	LRP6	**LDL receptor related protein 6**	Plasma Mem	transmembra	BI-905677	-1.026574242	0	0.90277722	0	1	0	10.8274057	11.076221	11.0686715	10.7730011
1086	**ENSG0000007004**	PHRF1	protein_coding	chr11	576470	612222	+	Human GRCh	OTTHUMG000001	PHRF1	**PHD and ring finger domains 1**	Nucleus	other		1.074007281	0	0.73117521	0	1	0	12.1083364	12.4211745	12.4608408	12.2392856
1087	**ENSG0000007006**	ELP1	protein_coding	chr9	108866898	108934328	-	Human GRCh	OTTHUMG000000	ELP1	**elongator acetyltransferase complex subunit 1**	Cytoplasm	other		-1.013094682	0	0.95073426	0	1	0	11.2172122	11.4634492	11.498578	11.1390901
1088	**ENSG0000007008**	NUCB2	protein_coding	chr11	17208153	17349980	+	Human GRCh	OTTHUMG000001	NUCB2	**nucleobindin 2**	Nucleus	other		-1.27032846	0	0.33626112	0	1	0	11.3457482	11.6629033	11.2911361	11.19717
1089	**ENSG0000007008**	PFN2	protein_coding	chr3	149964904	150050788	-	Human GRCh	OTTHUMG000001	PFN2	**profilin 2**	Cytoplasm	enzyme		1.050388614	0	0.8454405	0	1	0	9.29817995	9.43303278	9.54368151	9.27085223
1090	**ENSG0000007015**	PTPN3	protein_coding	chr9	109375466	109498313	-	Human GRCh	OTTHUMG000000	PTPN3	**protein tyrosine phosphatase non-receptor type 3**	Cytoplasm	phosphatase		-1.061878519	0	0.79904037	0	1	0	9.92898148	10.2052605	10.1290705	9.87956081
1091	**ENSG0000007018**	SPTB	protein_coding	chr14	64746283	64879907	-	Human GRCh	OTTHUMG000001	SPTB	**spectrin beta, erythrocytic**	Plasma Mem	other		-1.041982376	0	0.87870018	0	1	0	6.76527126	6.74943717	6.54946674	6.86234544
1092	**ENSG0000007019**	DAPP1	protein_coding	chr4	99816827	99870190	+	Human GRCh	OTTHUMG000001	DAPP1	**dual adaptor of phosphotyrosine and 3-phosphoinositides 1**	Cytoplasm	other		1	0	1	0	NA	0	0.37576877	0.38567738	0.38402485	0.37634397
1094	**ENSG0000007021**	SLC44A1	protein_coding	chr9	105244622	105439171	+	Human GRCh	OTTHUMG000000	SLC44A1	**solute carrier family 44 member 1**	Plasma Mem	transporter		-1.071081832	0	0.75789464	0	1	0	10.6007816	10.9483672	10.8447132	10.568359
1095	**ENSG0000007026**	TMEM260	protein_coding	chr14	56488354	56650606	+	Human GRCh	OTTHUMG000001	TMEM260	**transmembrane protein 260**	Cytoplasm	enzyme		1.092983352	0	0.72663162	0	1	0	9.39491311	9.44815422	9.75797921	9.21021249
1096	**ENSG0000007036**	SMG6	protein_coding	chr17	2059839	2303785	-	Human GRCh	OTTHUMG000001	SMG6	**SMG6 nonsense mediated mRNA decay factor**	Nucleus	enzyme		-1.173998093	0	0.45668054	0	1	0	10.7215308	11.0035504	10.7846051	10.5946356
1097	**ENSG0000007036**	EXOC5	protein_coding	chr14	57200507	57268905	-	Human GRCh	OTTHUMG000001	EXOC5	**exocyst complex component 5**	Cytoplasm	other		-1.007979142	0	0.96913797	0	1	0	11.4614139	11.7349488	11.7721226	11.3921014
1098	**ENSG0000007037**	CLTCL1	protein_coding	chr22	19179473	19291719	-	Human GRCh	OTTHUMG000001	CLTCL1	**clathrin heavy chain like 1**	Plasma Mem	other		-1.052872337	0	0.84751806	0	1	0	8.68469949	8.87733769	8.8729892	8.56757217
1099	**ENSG0000007038**	FGF22	protein_coding	chr19	639879	644373	+	Human GRCh	OTTHUMG000001	FGF22	**fibroblast growth factor 22**	Extracellular	growth facto		2.174106355	1	0.2851534	0	1	0	4.04494402	4.86609131	5.45174881	5.15142205
1100	**ENSG0000007040**	FSTL3	protein_coding	chr19	676392	683392	+	Human GRCh	OTTHUMG000001	FSTL3	**follistatin like 3**	Extracellular	other		1.163932196	0	0.67569196	0	1	0	6.51534717	6.85117585	6.76434966	6.94031026
1101	**ENSG0000007041**	DGCR2	protein_coding	chr22	19036282	19122454	-	Human GRCh	OTTHUMG000001	DGCR2	**DiGeorge syndrome critical region gene 2**	Plasma Mem	other		-1.050073117	0	0.80631411	0	1	0	11.2475699	11.5789624	11.4829599	11.2480633
1102	**ENSG0000007042**	RNF126	protein_coding	chr19	647526	663233	-	Human GRCh	OTTHUMG000001	RNF126	**ring finger protein 126**	Other	enzyme		1.074469514	0	0.72833394	0	1	0	11.233369	11.4347873	11.5225483	11.3064038
1103	**ENSG0000007044**	MNT	protein_coding	chr17	2384073	2401104	-	Human GRCh	OTTHUMG000000	MNT	**MAX network transcriptional repressor**	Nucleus	transcription		-1.129499773	0	0.5916833	0	1	0	12.3637347	12.7265664	12.4011848	12.4356054
1104	**ENSG0000007047**	ZXDC	protein_coding	chr3	126437601	126475891	-	Human GRCh	OTTHUMG000001	ZXDC	**ZXD family zinc finger C**	Other	transcription		-1.103892913	0	0.62836081	0	1	0	10.7603087	11.1095268	10.9641402	10.7010234
1105	**ENSG0000007049**	JMJD6	protein_coding	chr17	76712832	76726799	-	Human GRCh	OTTHUMG000001	JMJD6	**jumonji domain containing 6, arginine demethylase and lysine**	Nucleus	enzyme		1.009372438	0	0.96785015	0	1	0	10.0840914	10.6069157	10.5295038	10.2191695
1106	**ENSG0000007050**	POLB	protein_coding	chr8	42338454	42371808	+	Human GRCh	OTTHUMG000001	POLB	**DNA polymerase beta**	Nucleus	enzyme	lamivudine	-1.080437295	0	0.73137326	0	1	0	9.22365793	9.62236825	9.50305811	9.18290975
1107	**ENSG0000007052**	ST6GALNA	protein_coding	chr17	76624761	76643786	-	Human GRCh	OTTHUMG000001	ST6GALNAC1	**ST6 N-acetylgalactosaminide alpha-2,6-sialyltransferase 1**	Cytoplasm	enzyme		1.122707929	0	0.8788647	0	1	0	11.3700259	11.8967252	11.90293	11.6981242
1108	**ENSG0000007054**	WIPI1	protein_coding	chr17	68420948	68457513	-	Human GRCh	OTTHUMG000001	WIPI1	**WD repeat domain, phosphoinositide interacting 1**	Cytoplasm	other		-2.112432141	-1	0.01737257	1	1	0	10.5062431	10.8662711	9.98616768	9.60182432
1109	**ENSG0000007060**	FRMPD1	protein_coding	chr9	37650954	37746904	+	Human GRCh	OTTHUMG000000	FRMPD1	**FERM and PDZ domain containing 1**	Cytoplasm	other		-1.329474511	0	0.78137547	0	NA	0	2.23478214	2.82608749	2.48000483	2.41952796
1110	**ENSG0000007061**	GBA2	protein_coding	chr9	35736866	35749228	-	Human GRCh	OTTHUMG000000	GBA2	**glucosylceramidase beta 2**	Cytoplasm	enzyme	sinbaglust	-1.154633867	0	0.45028837	0	1	0	11.6219306	11.7955194	11.7198229	11.3565304
1111	**ENSG0000007061**	NDST1	protein_coding	chr5	150485818	150558211	+	Human GRCh	OTTHUMG000001	NDST1	**N-deacetylase and N-sulfotransferase 1**	Cytoplasm	enzyme		-1.172931596	0	0.44566157	0	1	0	11.3458554	11.5597439	11.4644175	11.0608572
1112	**ENSG0000007066**	ASNS	protein_coding	chr7	97851677	97872542	-	Human GRCh	OTTHUMG000000	ASNS	**asparagine synthetase (glutamine-hydrolyzing)**	Cytoplasm	enzyme		1.181412237	0	0.58472271	0	1	0	7.66741168	7.29443589	8.02028764	7.18005421
1113	**ENSG0000007071**	AP3M2	protein_coding	chr8	42152946	42171673	+	Human GRCh	OTTHUMG000001	AP3M2	**adaptor related protein complex 3 subunit mu 2**	Cytoplasm	transporter		-1.00346165	0	0.98710841	0	1	0	9.68493389	9.75380814	9.87855265	9.52782909
1114	**ENSG0000007072**	CNGB1	protein_coding	chr16	57882340	57971128	-	Human GRCh	OTTHUMG000001	CNGB1	**cyclic nucleotide gated channel subunit beta 1**	Plasma Mem	ion channel		1	0	1	0	1	0	1.9582065	2.06990212	2.05204861	1.965019
1115	**ENSG0000007073**	ST6GALNA	protein_coding	chr17	76565377	76586956	-	Human GRCh	OTTHUMG000001	ST6GALNAC2	**ST6 N-acetylgalactosaminide alpha-2,6-sialyltransferase 2**	Cytoplasm	enzyme		1.198652438	0	0.74509147	0	1	0	4.36329529	5.2591431	5.06843251	5.06286402
1117	**ENSG0000007075**	PABPC1	protein_coding	chr8	100685816	100722809	-	Human GRCh	OTTHUMG000001	PABPC1	**poly(A) binding protein cytoplasmic 1**	Cytoplasm	translation re		-1.060004301	0	0.78031389	0	1	0	14.8493164	15.179879	15.0645095	14.8484901
1118	**ENSG0000007075**	TESK2	protein_coding	chr1	45343883	45491163	-	Human GRCh	OTTHUMG000000	TESK2	**testis associated actin remodelling kinase 2**	Nucleus	kinase		-1.296187619	0	0.29089567	0	1	0	8.55268584	8.98413051	8.69614592	8.27779523
1119	**ENSG0000007076**	CFAP20	protein_coding	chr16	58113592	58129381	-	Human GRCh	OTTHUMG000001	CFAP20	**cilia and flagella associated protein 20**	Cytoplasm	other		-1.147905583	0	0.56452985	0	1	0	9.63411373	10.0252047	9.75434522	9.62471552
1120	**ENSG0000007077**	CSNK2A2	protein_coding	chr16	58157907	58198106	-	Human GRCh	OTTHUMG000001	CSNK2A2	**casein kinase 2 alpha 2**	Cytoplasm	kinase		-1.185410493	0	0.40544567	0	1	0	11.3117832	11.5452991	11.3912406	11.0762679
1121	**ENSG0000007077**	PTPN21	protein_coding	chr14	88465778	88555007	-	Human GRCh	OTTHUMG000001	PTPN21	**protein tyrosine phosphatase non-receptor type 21**	Cytoplasm	phosphatase		1.390907875	0	0.35771184	0	1	0	6.734808	7.02461997	7.41591017	7.00291331
1122	**ENSG0000007078**	EIF2B3	protein_coding	chr1	44850522	44986722	-	Human GRCh	OTTHUMG000000	EIF2B3	**eukaryotic translation initiation factor 2B subunit gamma**	Cytoplasm	translation re		-1.016397305	0	0.93558512	0	1	0	9.41919465	9.61027869	9.65283217	9.3255284
1124	**ENSG0000007081**	TCOF1	protein_coding	chr5	150357629	150400308	+	Human GRCh	OTTHUMG000001	TCOF1	**treacle ribosome biogenesis factor 1**	Nucleus	transcription		-1.001954039	0	0.99180269	0	1	0	11.6921134	12.0247753	11.9814066	11.7422754
1125	**ENSG0000007083**	CDC42	protein_coding	chr1	22052627	22101360	+	Human GRCh	OTTHUMG000000	CDC42	**cell division cycle 42**	Cytoplasm	enzyme		-1.061702214	0	0.78348326	0	1	0	12.6308878	12.9069186	12.8752683	12.5178955
1126	**ENSG0000007088**	OSBPL3	protein_coding	chr7	24796540	24981634	-	Human GRCh	OTTHUMG000000	OSBPL3	**oxysterol binding protein like 3**	Plasma Mem	transporter		1.164038999	0	0.58220305	0	1	0	9.20038034	9.54416838	9.64180459	9.42625902
1127	**ENSG0000007088**	EPHA8	protein_coding	chr1	22563489	22603595	+	Human GRCh	OTTHUMG000000	EPHA8	**EPH receptor A8**	Plasma Mem	kinase		-1.929968881	-1	0.10354089	0	1	0	6.08781439	6.03951571	4.75830069	5.84653004
1129	**ENSG0000007095**	RAD18	protein_coding	chr3	8775402	8963773	-	Human GRCh	OTTHUMG000000	RAD18	**RAD18 E3 ubiquitin protein ligase**	Nucleus	other		-1.061168588	0	0.77487482	0	1	0	10.5366774	10.9281025	10.8147262	10.5336365
1130	**ENSG0000007096**	ATP2B1	protein_coding	chr12	89588049	89709366	-	Human GRCh	OTTHUMG000001	ATP2B1	**ATPase plasma membrane Ca2+ transporting 1**	Plasma Mem	transporter		1.026306479	0	0.90576206	0	1	0	11.3962862	11.6374037	11.7626326	11.2950842
1131	**ENSG0000007098**	TRPM5	protein_coding	chr11	2404515	2444514	-	Human GRCh	OTTHUMG000000	TRPM5	**transient receptor potential cation channel subfamily M meml**	Plasma Mem	ion channel		8.015636698	1	0.0966877	0	1	0	2.21796306	2.04351822	2.72457962	2.64408392
1132	**ENSG0000007105**	NCK2	protein_coding	chr2	105744912	105894274	+	Human GRCh	OTTHUMG000001	NCK2	**NCK adaptor protein 2**	Cytoplasm	kinase		-1.022024898	0	0.91511979	0	1	0	9.47486603	9.62733366	9.66817679	9.37381824
1133	**ENSG0000007105**	MAP4K4	protein_coding	chr2	101696850	101894690	+	Human GRCh	OTTHUMG000001	MAP4K4	**mitogen-activated protein kinase kinase kinase kinase 4**	Cytoplasm	kinase	PF-062609	-1.251015162	0	0.25684262	0	1	0	11.9808595	12.2878119	12.0220045	11.7599109
1134	**ENSG0000007107**	MGAT4A	protein_coding	chr2	98619106	98731132	-	Human GRCh	OTTHUMG000001	MGAT4A	**alpha-1,3-mannosyl-glycoprotein 4-beta-N-acetylglucosaminyl**	Cytoplasm	enzyme		1.362752469	0	0.18300703	0	1	0	8.77891051	8.89766015	9.35655533	8.93143519
1135	**ENSG0000007108**	RPL31	protein_coding	chr2	101002229	101024032	+	Human GRCh	OTTHUMG000001	RPL31	**ribosomal protein L31**	Cytoplasm	other		1.080489568	0	0.6875628	0	1	0	11.7859308	12.0422239	12.1736433	11.8189944
1136	**ENSG0000007112**	WDR1	protein_coding	chr4	10068089	10116972	-	Human GRCh	OTTHUMG000001	WDR1	**WD repeat domain 1**	Extracellular	other		-1.130777918	0	0.53655152	0	1	0	12.6953423	12.9677799	12.8564784	12.5211155
1137	**ENSG0000007118**	SNX13	protein_coding	chr7	17790761	17940501	-	Human GRCh	OTTHUMG000001	SNX13	**sorting nexin 13**	Cytoplasm	transporter		1.044234979	0	0.84378708	0	1	0	10.9877669	11.233674	11.3028783	11.006252
1138	**ENSG0000007120**	MS4A12	protein_coding	chr11	60492778	60507430	+	Human GRCh	OTTHUMG000001	MS4A12	**membrane spanning 4-domains A12**	Plasma Mem	other		1	0	1	0	NA	0	-0.164076	-0.1562458	-0.1575438	-0.1636183
1139	**ENSG0000007120**	ARHGAP1(	protein_coding	chr4	147732063	148072776	+	Human GRCh	OTTHUMG000001	ARHGAP10	**Rho GTPase activating protein 10**	Cytoplasm	other		1.457529329	0	0.35124419	0	1	0	6.81546798	6.84129543	6.71543019	7.53031016
1140	**ENSG0000007124**	RPS6KA2	protein_coding	chr6	166409364	166906451	-	Human GRCh	OTTHUMG000000	RPS6KA2	**ribosomal protein S6 kinase A2**	Nucleus	kinase	BI-D1870,	-1.287381566	0	0.28969224	0	1	0	9.47785033	9.38644973	9.24154072	9.0708599
1141	**ENSG0000007124**	ING3	protein_coding	chr7	120950763	120977216	+	Human GRCh	OTTHUMG000001	ING3	**inhibitor of growth family member 3**	Nucleus	transcription		-1.097793145	0	0.67685599	0	1	0	8.61079609	8.98838765	8.82410569	8.59241612
1142	**ENSG0000007124**	VASH1	protein_coding	chr14	76761468	76783015	+	Human GRCh	OTTHUMG000001	VASH1	**vasohibin 1**	Extracellular	peptidase		1.149490995	0	0.62444181	0	1	0	7.00239892	6.45832899	7.19002949	6.52658222
1143	**ENSG0000007128**	LMCD1	protein_coding	chr3	8501807	8574668	+	Human GRCh	OTTHUMG000001	LMCD1	**LIM and cysteine rich domains 1**	Cytoplasm	transcription		-12.20310861	-1	2.41E-07	1	0	1	7.04775529	6.91149351	4.43353975	4.66589608
1144	**ENSG0000007146**	BUD23	protein_coding	chr7	73683597	73698212	+	Human GRCh	OTTHUMG000000	BUD23	**BUD23 rRNA methyltransferase and ribosome maturation fact**	Nucleus	enzyme		1.097614569	0	0.61446861	0	1	0	10.2107671	10.5829647	10.5759335	10.449223
1145	**ENSG0000007153**	SEL1L	protein_coding	chr14	81471547	81533853	-	Human GRCh	OTTHUMG000001	SEL1L	**SEL1L adaptor subunit of SYVN1 ubiquitin ligase**	Cytoplasm	other		-1.213590465	0	0.37951705	0	1	0	11.6886186	12.0887142	11.7547682	11.6211961
1146	**ENSG0000007153**	TRIP13	protein_coding	chr5	892884	919357	+	Human GRCh	OTTHUMG000000	TRIP13	**thyroid hormone receptor interactor 13**	Cytoplasm	transcription		-1.073726431	0	0.75693602	0	1	0	10.4509043	10.7430656	10.7127558	10.30533
1147	**ENSG0000007155**	ATP6AP1	protein_coding	chrX	154428633	154436516	+	Human GRCh	OTTHUMG000000	ATP6AP1	**ATPase H+ transporting accessory protein 1**	Cytoplasm	transporter		-1.157684794	0	0.53059238	0	1	0	11.6684913	12.0153779	11.7720224	11.6022068
1148	**ENSG0000007156**	TCF3	protein_coding	chr19	1609291	1652615	-	Human GRCh	OTTHUMG000001	TCF3	**transcription factor 3**	Nucleus	transcription		1.071515187	0	0.69969395	0	1	0	11.7883596	12.0413691	12.0906381	11.8980452
1149	**ENSG0000007157**	TRIB2	protein_coding	chr2	12716910	12742734	+	Human GRCh	OTTHUMG000000	TRIB2	**tribbles pseudokinase 2**	Plasma Mem	kinase		-1.352134096	0	0.29912813	0	1	0	9.18258674	9.44286556	9.18337311	8.80047932
1150	**ENSG0000007162**	DAZAP1	protein_coding	chr19	1407569	1435687	+	Human GRCh	OTTHUMG000001	DAZAP1	**DAZ associated protein 1**	Other	other		1.039949979	0	0.84209095	0	1	0	12.5127344	12.77614	12.8203917	12.5558557
1151	**ENSG0000007165**	MBD3	protein_coding	chr19	1573596	1592865	-	Human GRCh	OTTHUMG000001	MBD3	**methyl-CpG binding domain protein 3**	Nucleus	other		-1.07574332	0	0.71623534	0	1	0	11.9482926	12.2514494	12.1611682	11.8809957
1153	**ENSG0000007179**	HLTF	protein_coding	chr3	149030127	149086554	-	Human GRCh	OTTHUMG000001	HLTF	**helicase like transcription factor**	Nucleus	transcription		-1.00815478	0	0.9719695	0	1	0	11.3420779	11.6195975	11.6711985	11.2515407
1154	**ENSG0000007185**	FAM50A	protein_coding	chrX	154444141	154450654	+	Human GRCh	OTTHUMG000000	FAM50A	**family with sequence similarity 50 member A**	Nucleus	other		1.056506501	0	0.80311113	0	1	0	9.87000618	10.0763722	10.1346822	9.9337201
1155	**ENSG0000007188**	FAM3A	protein_coding	chrX	154506159	154516242	-	Human GRCh	OTTHUMG000000	FAM3A	**FAM3 metabolism regulating signaling molecule A**	Extracellular	other		-1.02320575	0	0.91779992	0	1	0	9.94109082	10.341016	10.1681444	10.0948653
1156	**ENSG0000007189**	CPSF1	protein_coding	chr8	144393229	144409335	-	Human GRCh	OTTHUMG000001	CPSF1	**cleavage and polyadenylation specific factor 1**	Nucleus	other		-1.014248969	0	0.94272152	0	1	0	12.2877134	12.6098606	12.6056644	12.2570338
1157	**ENSG0000007190**	MYO3B	protein_coding	chr2	170178145	170655171	+	Human GRCh	OTTHUMG000001	MYO3B	**myosin IIIB**	Plasma Mem	kinase		-1.101958358	0	0.90202491	0	1	0	1.42749235	3.01189421	1.50575207	2.84521889
1158	**ENSG0000007196**	CYBRD1	protein_coding	chr2	171522247	171558129	+	Human GRCh	OTTHUMG000001	CYBRD1	**cytochrome b reductase 1**	Cytoplasm	enzyme		-1.100717494	0	0.94110523	0	NA	0	2.19387027	1.52920224	2.17268342	1.51521131
1160	**ENSG0000007199**	PDCD2	protein_coding	chr6	170575295	170584692	-	Human GRCh	OTTHUMG000000	PDCD2	**programmed cell death 2**	Nucleus	other		-1.045325574	0	0.82307239	0	1	0	10.3944344	10.7000833	10.5810598	10.4295145
1161	**ENSG0000007204**	SLC6A15	protein_coding	chr12	84859491	84913629	-	Human GRCh	OTTHUMG000001	SLC6A15	**solute carrier family 6 member 15**	Plasma Mem	transporter		1.110923563	0	0.70310794	0	1	0	8.67765202	8.84696394	9.11182783	8.60441651
1162	**ENSG0000007204**	RDH11	protein_coding	chr14	67676800	67695793	-	Human GRCh	OTTHUMG000001	RDH11	**retinol dehydrogenase 11**	Cytoplasm	enzyme		-1.205883997	0	0.43009486	0	1	0	10.9363721	11.225305	10.9745849	10.7724794

1163	**ENSG0000007206**	PRKACA	protein_coding	chr19	14091688	14118084	-	Human GRCh	OTTHUMG000001	PRKACA	**protein kinase cAMP-activated catalytic subunit alpha**	Cytoplasm	kinase		1.089555837	0	0.66946428	0	1	0	11.0905586	11.4973032	11.4412089	11.3720099
1164	**ENSG0000007207**	ADGRL1	protein_coding	chr19	14147743	14206187	-	Human GRCh	OTTHUMG000001	ADGRL1	**adhesion G protein-coupled receptor L1**	Plasma Mem	G-protein cou		1.052362367	0	0.8109626	0	1	0	13.0530903	13.2845576	13.3149468	13.1436586
1166	**ENSG0000007211**	ACTN1	protein_coding	chr14	68874128	68979440	-	Human GRCh	OTTHUMG000001	ACTN1	**actinin alpha 1**	Nucleus	transcription		-1.20020188	0	0.33907987	0	1	0	11.6958747	11.9338122	11.6447144	11.5847573
1167	**ENSG0000007212**	ZFYVE26	protein_coding	chr14	67727374	67816590	-	Human GRCh	OTTHUMG000001	ZFYVE26	**zinc finger FYVE-type containing 26**	Cytoplasm	other		-1.255154817	0	0.26489183	0	1	0	11.1897213	11.532614	11.2477385	10.9757588
1168	**ENSG0000007213**	RPS6KA6	protein_coding	chrX	84058346	84206356	-	Human GRCh	OTTHUMG000000	RPS6KA6	**ribosomal protein S6 kinase A6**	Cytoplasm	kinase	BI-D1870,	-1.057378381	0	0.81379078	0	1	0	9.96711307	10.3258278	10.2579008	9.9144125
1169	**ENSG0000007213**	EPN2	protein_coding	chr17	19215615	19336715	+	Human GRCh	OTTHUMG000001	EPN2	**epsin 2**	Cytoplasm	other		-1.026911176	0	0.9001966	0	1	0	11.2471344	11.4220724	11.4131766	11.1925336
1170	**ENSG0000007213**	PTPN18	protein_coding	chr2	130356045	130375405	+	Human GRCh	OTTHUMG000001	PTPN18	**protein tyrosine phosphatase non-receptor type 18**	Nucleus	phosphatase		-1.11122139	0	0.62961961	0	1	0	10.2269608	10.4232111	10.390146	10.0065373
1171	**ENSG0000007216**	LIMS2	protein_coding	chr2	127638381	127681786	-	Human GRCh	OTTHUMG000001	LIMS2	**LIM zinc finger domain containing 2**	Cytoplasm	other		1	0	1	0	1	0	1.29496811	1.35413451	1.34454219	1.29851727
1172	**ENSG0000007218**	ASIC4	protein_coding	chr2	219514170	219538772	+	Human GRCh	OTTHUMG000000	ASIC4	**acid sensing ion channel subunit family member 4**	Plasma Mem	ion channel	amiloride,	-1.44976158	0	0.64813289	0	1	0	2.87054276	3.24945845	2.72386898	2.88011308
1173	**ENSG0000007219**	SPEG	protein_coding	chr2	219434843	219493629	+	Human GRCh	OTTHUMG000001	SPEG	**striated muscle enriched protein kinase**	Nucleus	kinase		-1.054734448	0	0.91208735	0	1	0	4.20319278	5.10413591	4.58811142	4.7809898
1174	**ENSG0000007220**	LNX1	protein_coding	chr4	53459301	53701405	-	Human GRCh	OTTHUMG000001	LNX1	**ligand of numb-protein X 1**	Cytoplasm	enzyme		-1.450190297	0	0.26015888	0	1	0	9.02883099	9.60139628	9.06407347	8.76440269
1175	**ENSG0000007221**	ALDH3A2	protein_coding	chr17	19648136	19685760	+	Human GRCh	OTTHUMG000000	ALDH3A2	**aldehyde dehydrogenase 3 family member A2**	Cytoplasm	enzyme		1.12451882	0	0.55679857	0	1	0	10.1937115	10.3134337	10.5126125	10.2317438
1176	**ENSG0000007227**	TFRC	protein_coding	chr3	196012511	196082153	-	Human GRCh	OTTHUMG000001	TFRC	**transferrin receptor**	Plasma Mem	transporter	CALAA-01,	-1.060987287	0	0.82650653	0	1	0	12.9741869	13.3936377	13.3502884	12.8725985
1177	**ENSG0000007231**	SREBF1	protein_coding	chr17	17810399	17837002	-	Human GRCh	OTTHUMG000000	SREBF1	**sterol regulatory element binding transcription factor 1**	Nucleus	transcription		-1.030514487	0	0.92315514	0	1	0	12.0538711	12.6060256	12.2693092	12.3793749
1179	**ENSG0000007236**	AFF4	protein_coding	chr5	132875395	132963634	-	Human GRCh	OTTHUMG000000	AFF4	**ALF transcription elongation factor 4**	Nucleus	transcription		-1.074289937	0	0.72338968	0	1	0	12.8835147	13.0872584	13.077956	12.712755
1180	**ENSG0000007240**	UBE2D1	protein_coding	chr10	58334979	58370751	+	Human GRCh	OTTHUMG000000	UBE2D1	**ubiquitin conjugating enzyme E2 D1**	Cytoplasm	enzyme		1.085822142	0	0.70302318	0	1	0	8.55012917	8.54107549	8.75549928	8.49792401
1181	**ENSG0000007241**	PALS1	protein_coding	chr14	67240713	67336061	+	Human GRCh	OTTHUMG000001	PALS1	**protein associated with LIN7 1, MAGUK p55 family member**	Plasma Mem	kinase		-1.016191421	0	0.94001371	0	1	0	10.3349056	10.451426	10.5495598	10.1747858
1182	**ENSG0000007242**	RHOBTB1	protein_coding	chr10	60869438	61001440	-	Human GRCh	OTTHUMG000000	RHOBTB1	**Rho related BTB domain containing 1**	Cytoplasm	enzyme		-1.485478953	0	0.28595999	0	1	0	7.06527989	7.40191632	6.75828753	6.95542576
1183	**ENSG0000007250**	SMC1A	protein_coding	chrX	53374149	53422728	-	Human GRCh	OTTHUMG000000	SMC1A	**structural maintenance of chromosomes 1A**	Nucleus	enzyme		-1.105874009	0	0.60740099	0	1	0	12.3793297	12.5957047	12.4657199	12.2936739
1184	**ENSG0000007250**	HSD17B10	protein_coding	chrX	53431258	53434370	-	Human GRCh	OTTHUMG000000	HSD17B10	**hydroxysteroid 17-beta dehydrogenase 10**	Cytoplasm	enzyme		-1.052268077	0	0.82802377	0	1	0	10.5008348	10.8965018	10.6949174	10.6188358
1185	**ENSG0000007251**	MARK2	protein_coding	chr11	63838928	63911020	+	Human GRCh	OTTHUMG000001	MARK2	**microtubule affinity regulating kinase 2**	Cytoplasm	kinase	SM1-71	1.045398347	0	0.8095628	0	1	0	11.3535417	11.6356793	11.5939522	11.5081705
1186	**ENSG0000007257**	HMMR	protein_coding	chr5	163460203	163491941	+	Human GRCh	OTTHUMG000001	HMMR	**hyaluronan mediated motility receptor**	Plasma Mem	transmembra		-1.132606355	0	0.61538322	0	1	0	10.7211092	10.9874288	10.8254357	10.6107392
1187	**ENSG0000007260**	CHFR	protein_coding	chr12	132822187	132956304	-	Human GRCh	OTTHUMG000001	CHFR	**checkpoint with forkhead and ring finger domains**	Nucleus	enzyme		-1.090950686	0	0.71411789	0	1	0	10.4591978	10.8052534	10.7549564	10.2888364
1188	**ENSG0000007265**	TRHDE	protein_coding	chr12	72087266	72670758	+	Human GRCh	OTTHUMG000001	TRHDE	**thyrotropin releasing hormone degrading enzyme**	Plasma Mem	peptidase		-1.470209028	0	0.56966027	0	1	0	3.66462958	3.60018747	3.68873766	3.0714214
1189	**ENSG0000007268**	P4HA2	protein_coding	chr5	132190147	132295315	-	Human GRCh	OTTHUMG000000	P4HA2	**prolyl 4-hydroxylase subunit alpha 2**	Cytoplasm	transporter		-1.085830026	0	0.77243494	0	1	0	8.24227639	8.76701132	8.47055708	8.41799823
1191	**ENSG0000007273**	NFATC3	protein_coding	chr16	68084751	68229259	+	Human GRCh	OTTHUMG000001	NFATC3	**nuclear factor of activated T cells 3**	Nucleus	transcription		-1.047212413	0	0.82729942	0	1	0	10.8783182	11.3302925	11.1806136	10.9576504
1192	**ENSG0000007275**	TRNT1	protein_coding	chr3	3126933	3153435	+	Human GRCh	OTTHUMG000000	TRNT1	**tRNA nucleotidyl transferase 1**	Cytoplasm	enzyme		1.012942275	0	0.95344644	0	1	0	10.1882652	10.3089996	10.4710699	10.0129685
1193	**ENSG0000007277**	ACADVL	protein_coding	chr17	7217125	7225266	+	Human GRCh	OTTHUMG000001	ACADVL	**acyl-CoA dehydrogenase very long chain**	Cytoplasm	enzyme		-1.092662562	0	0.62513183	0	1	0	13.0450638	13.2978495	13.1796605	12.9677433
1194	**ENSG0000007278**	STK10	protein_coding	chr5	172042079	172188224	-	Human GRCh	OTTHUMG000001	STK10	**serine/threonine kinase 10**	Cytoplasm	kinase		-1.121092151	0	0.68085399	0	1	0	8.43211962	8.65445573	8.60014528	8.20517708
1195	**ENSG0000007280**	FBXW11	protein_coding	chr5	171861549	172006873	-	Human GRCh	OTTHUMG000001	FBXW11	**F-box and WD repeat domain containing 11**	Cytoplasm	enzyme		-1.022550707	0	0.91624583	0	1	0	10.948945	11.1582292	11.2230197	10.8072887
1196	**ENSG0000007281**	ACAP1	protein_coding	chr17	7336529	7351477	+	Human GRCh	OTTHUMG000001	ACAP1	**ArfGAP with coiled-coil, ankyrin repeat and PH domains 1**	Plasma Mem	other		1.110708704	0	0.91205322	0	NA	0	2.24514204	3.11795672	2.53733771	3.07344068
1197	**ENSG0000007283**	CRMP1	protein_coding	chr4	5748084	5893086	-	Human GRCh	OTTHUMG000001	CRMP1	**collapsin response mediator protein 1**	Cytoplasm	enzyme		1	0	1	0	1	0	2.83085109	2.93451891	2.91781561	2.83711626
1198	**ENSG0000007284**	EVC	protein_coding	chr4	5711201	5814305	+	Human GRCh	OTTHUMG000000	EVC	**EvC ciliary complex subunit 1**	Cytoplasm	other		-1.263288696	0	0.33712348	0	1	0	8.81183052	8.55493748	8.47513838	8.38217462
1199	**ENSG0000007284**	DERL2	protein_coding	chr17	5471254	5486811	-	Human GRCh	OTTHUMG000001	DERL2	**derlin 2**	Cytoplasm	other		-1.125128987	0	0.57804705	0	1	0	9.93422862	10.2046308	10.0401957	9.84662254
1200	**ENSG0000007285**	SIDT1	protein_coding	chr3	113532555	113629575	+	Human GRCh	OTTHUMG000001	SIDT1	**SID1 transmembrane family member 1**	Cytoplasm	other		-1.61553577	-1	0.66701173	0	1	0	1.59719143	2.41252059	1.63607552	2.11917692
1201	**ENSG0000007286**	NDE1	protein_coding	chr16	15643267	15734691	+	Human GRCh	OTTHUMG000001	NDE1	**nudE neurodevelopment protein 1**	Nucleus	other		-1.144727125	0	0.52860876	0	1	0	9.99177878	10.3526923	10.1395951	9.92875932
1203	**ENSG0000007295**	TMEM38A	protein_coding	chr19	16661139	16690023	+	Human GRCh	OTTHUMG000001	TMEM38A	**transmembrane protein 38A**	Cytoplasm	ion channel		1.191629456	0	0.61339716	0	1	0	6.62074066	7.39944144	7.36455613	7.13466672
1204	**ENSG0000007295**	AP1M1	protein_coding	chr19	16197854	16245906	+	Human GRCh	OTTHUMG000001	AP1M1	**adaptor related protein complex 1 subunit mu 1**	Cytoplasm	transporter		1.048855751	0	0.82019848	0	1	0	9.49785295	9.83128856	9.80509835	9.64593325
1205	**ENSG0000007300**	PVR	protein_coding	chr19	44643798	44666162	+	Human GRCh	OTTHUMG000001	PVR	**PVR cell adhesion molecule**	Plasma Mem	other	NTX-1088	-1.140578541	0	0.48463453	0	1	0	11.430727	11.5279037	11.5031471	11.129848
1206	**ENSG0000007305**	XRCC1	protein_coding	chr19	43543311	43580473	-	Human GRCh	OTTHUMG000001	XRCC1	**X-ray repair cross complementing 1**	Nucleus	other		-1.007740983	0	0.96970436	0	1	0	10.1681209	10.4743205	10.3841549	10.2567619
1207	**ENSG0000007306**	SCARB1	protein_coding	chr12	124776856	124882668	-	Human GRCh	OTTHUMG000001	SCARB1	**scavenger receptor class B member 1**	Plasma Mem	transporter	zeaxanthin	-1.000491037	0	0.9984033	0	1	0	10.7393227	11.1406766	11.0006324	10.909734
1208	**ENSG0000007306**	CYP2W1	protein_coding	chr7	983181	989640	+	Human GRCh	OTTHUMG000000	CYP2W1	**cytochrome P450 family 2 subfamily W member 1**	Cytoplasm	enzyme		-5.23696464	-1	0.27807535	0	NA	0	1.72979343	1.25832027	1.25286508	1.22713956
1209	**ENSG0000007311**	MCM2	protein_coding	chr3	127598410	127622436	+	Human GRCh	OTTHUMG000001	MCM2	**minichromosome maintenance complex component 2**	Nucleus	enzyme		-1.144163267	0	0.5541829	0	1	0	12.301422	12.6745274	12.4556874	12.2452178
1210	**ENSG0000007314**	MOV10L1	protein_coding	chr22	50090006	50161690	+	Human GRCh	OTTHUMG000000	MOV10L1	**Mov10 like RISC complex RNA helicase 1**	Nucleus	enzyme		1.559433115	1	0.38340592	0	1	0	6.01953679	6.24883614	6.61544199	6.65072897
1211	**ENSG0000007315**	PANX2	protein_coding	chr22	50170731	50180295	+	Human GRCh	OTTHUMG000000	PANX2	**pannexin 2**	Plasma Mem	transporter		1.318274839	0	0.57474699	0	1	0	9.56519856	9.55605498	9.68450741	10.0382005
1212	**ENSG0000007316**	SELENOO	protein_coding	chr22	50201011	50217616	+	Human GRCh	OTTHUMG000000	SELENOO	**selenoprotein O**	Extracellular	enzyme		-1.017299037	0	0.94289539	0	1	0	10.3130031	10.6282809	10.4398013	10.4825311
1213	**ENSG0000007328**	TP63	protein_coding	chr3	189631389	189897276	+	Human GRCh	OTTHUMG000001	TP63	**tumor protein p63**	Nucleus	transcription		-1.027771337	0	0.98857574	0	NA	0	0.83737093	1.21823917	1.20825676	0.83783691
1214	**ENSG0000007333**	ALPK1	protein_coding	chr4	112285509	112442621	+	Human GRCh	OTTHUMG000001	ALPK1	**alpha kinase 1**	Cytoplasm	kinase		-1.160570375	0	0.72878259	0	1	0	6.25084574	6.44703434	6.46732737	5.86718321
1215	**ENSG0000007335**	LLGL2	protein_coding	chr17	75525080	75575209	+	Human GRCh	OTTHUMG000001	LLGL2	**LLGL scribble cell polarity complex component 2**	Cytoplasm	other		-1.069086664	0	0.76484538	0	1	0	11.8094959	12.1566732	12.0534484	11.7689518
1216	**ENSG0000007341**	PDE8A	protein_coding	chr15	84980440	85139145	+	Human GRCh	OTTHUMG000001	PDE8A	**phosphodiesterase 8A**	Cytoplasm	enzyme	pentoxifyll	1.020902539	0	0.91878029	0	1	0	9.92701723	10.0734894	10.1519549	9.88320567
1217	**ENSG0000007346**	CLCN4	protein_coding	chrX	10156975	10237660	+	Human GRCh	OTTHUMG000000	CLCN4	**chloride voltage-gated channel 4**	Plasma Mem	ion channel		-1.279625829	0	0.4285272	0	1	0	7.05090792	7.251943	6.87192854	6.93517658
1218	**ENSG0000007353**	NLE1	protein_coding	chr17	35128730	35142304	-	Human GRCh	OTTHUMG000001	NLE1	**notchless homolog 1**	Nucleus	enzyme		1.111370351	0	0.64015234	0	1	0	10.1276877	10.4563903	10.5740355	10.246164
1219	**ENSG0000007357**	SDHA	protein_coding	chr5	218303	257082	+	Human GRCh	OTTHUMG000000	SDHA	**succinate dehydrogenase complex flavoprotein subunit A**	Cytoplasm	enzyme		-1.033671407	0	0.88263817	0	1	0	12.150171	12.4930852	12.4420685	12.1315033
1220	**ENSG0000007358**	SMARCE1	protein_coding	chr17	40624962	40648654	-	Human GRCh	OTTHUMG000001	SMARCE1	**SWI/SNF related, matrix associated, actin dependent regulato**	Nucleus	transcription		-1.061366212	0	0.76933438	0	1	0	11.5259653	11.737201	11.7196094	11.4015606
1222	**ENSG0000007360**	GSDMB	protein_coding	chr17	39904595	39919854	-	Human GRCh	OTTHUMG000001	GSDMB	**gasdermin B**	Cytoplasm	other		-1.165949137	0	0.56221535	0	1	0	9.26200464	9.41880802	9.3420917	8.98641539
1223	**ENSG0000007361**	KDM5A	protein_coding	chr12	280057	389320	-	Human GRCh	OTTHUMG000001	KDM5A	**lysine demethylase 5A**	Nucleus	enzyme	CPI-455	-1.186183998	0	0.40149836	0	1	0	11.6637432	11.9644955	11.7876173	11.4580061
1224	**ENSG0000007367**	ADAM11	protein_coding	chr17	44758988	44781846	+	Human GRCh	OTTHUMG000001	ADAM11	**ADAM metallopeptidase domain 11**	Plasma Mem	peptidase		1.202204993	0	0.49770546	0	1	0	8.27845251	8.53003138	8.70406277	8.50698717
1225	**ENSG0000007371**	PPP2R3A	protein_coding	chr3	135965728	136147894	+	Human GRCh	OTTHUMG000001	PPP2R3A	**protein phosphatase 2 regulatory subunit B''alpha**	Nucleus	phosphatase		-1.345692951	0	0.28854395	0	1	0	8.272214	8.54922647	8.26082587	7.91812051
1226	**ENSG0000007371**	FERMT2	protein_coding	chr14	52857268	52952435	-	Human GRCh	OTTHUMG000001	FERMT2	**FERM domain containing kindlin 2**	Cytoplasm	other		-1.023423882	0	0.91322736	0	1	0	10.2859217	10.4739284	10.5172198	10.1739346
1230	**ENSG0000007375**	PTGS2	protein_coding	chr1	186671791	186680922	-	Human GRCh	OTTHUMG000000	PTGS2	**prostaglandin-endoperoxide synthase 2**	Cytoplasm	enzyme	aspirin/riva	1	0	1	0	NA	0	0.56333906	0.57454056	0.57267167	0.56398904
1231	**ENSG0000007379**	IGF2BP2	protein_coding	chr3	185643130	185825042	-	Human GRCh	OTTHUMG000000	IGF2BP2	**insulin like growth factor 2 mRNA binding protein 2**	Cytoplasm	translation re		1.20058736	0	0.60769552	0	1	0	8.96932549	8.85986141	8.8312229	9.35997099
1232	**ENSG0000007380**	MAP3K13	protein_coding	chr3	185282941	185489094	+	Human GRCh	OTTHUMG000001	MAP3K13	**mitogen-activated protein kinase kinase kinase 13**	Cytoplasm	kinase		1.016325042	0	0.94779453	0	1	0	9.512714	9.83601687	9.74607429	9.65789921
1233	**ENSG0000007384**	ST6GAL1	protein_coding	chr3	186930325	187078553	+	Human GRCh	OTTHUMG000001	ST6GAL1	**ST6 beta-galactoside alpha-2,6-sialyltransferase 1**	Cytoplasm	enzyme		-1.134372907	0	0.61585946	0	1	0	10.0801184	10.3452591	10.2903343	9.80517899
1235	**ENSG0000007391**	FRY	protein_coding	chr13	31846713	32299125	+	Human GRCh	OTTHUMG000000	FRY	**FRY microtubule binding protein**	Extracellular	other		-1.002001137	0	0.99554393	0	1	0	8.79595478	9.13419586	8.78955909	9.13436608
1236	**ENSG0000007392**	PICALM	protein_coding	chr11	85957175	86069882	-	Human GRCh	OTTHUMG000001	PICALM	**phosphatidylinositol binding clathrin assembly protein**	Cytoplasm	other		-1.068836644	0	0.79977262	0	1	0	12.8466967	13.1362325	13.2021921	12.5617082
1237	**ENSG0000007396**	NSF	protein_coding	chr17	46590669	46757679	+	Human GRCh	OTTHUMG000001	NSF	**N-ethylmaleimide sensitive factor, vesicle fusing ATPase**	Cytoplasm	transporter		-1.064070075	0	0.78399369	0	1	0	10.1592969	10.7062333	10.4794901	10.3058763
1238	**ENSG0000007404**	GLI2	protein_coding	chr2	120735623	120992653	+	Human GRCh	OTTHUMG000001	GLI2	**GLI family zinc finger 2**	Nucleus	transcription		-1.035046349	0	0.97193344	0	1	0	3.94544421	4.2170783	3.147608	4.55086865
1239	**ENSG0000007405**	CLASP1	protein_coding	chr2	121337776	121649476	-	Human GRCh	OTTHUMG000001	CLASP1	**cytoplasmic linker associated protein 1**	Cytoplasm	other		-1.164377525	0	0.44869811	0	1	0	11.3326097	11.6098903	11.4333043	11.1790558
1240	**ENSG0000007407**	MRPS34	protein_coding	chr16	1771890	1773150	-	Human GRCh	OTTHUMG000001	MRPS34	**mitochondrial ribosomal protein S34**	Cytoplasm	other		1.075038135	0	0.73236955	0	1	0	10.4038238	10.6455041	10.637295	10.5876465
1241	**ENSG0000007418**	NOTCH3	protein_coding	chr19	15159038	15200995	-	Human GRCh	OTTHUMG000001	NOTCH3	**notch receptor 3**	Plasma Mem	transcription	PF-066508	-1.157484087	0	0.58483781	0	1	0	9.84948103	9.88511872	9.70111579	9.70713241
1242	**ENSG0000007420**	CLNS1A	protein_coding	chr11	77514936	77637794	-	Human GRCh	OTTHUMG000001	CLNS1A	**chloride nucleotide-sensitive channel 1A**	Plasma Mem	ion channel		-1.090451703	0	0.65920942	0	1	0	10.863263	11.1759141	11.0711829	10.7782888
1243	**ENSG0000007421**	PPP2R2C	protein_coding	chr4	6320578	6563600	-	Human GRCh	OTTHUMG000000	PPP2R2C	**protein phosphatase 2 regulatory subunit Bgamma**	Nucleus	phosphatase		1.138574599	0	0.78904591	0	1	0	4.83891452	3.44746797	3.75264859	4.89288665
1244	**ENSG0000007421**	TEAD2	protein_coding	chr19	49340595	49362457	-	Human GRCh	OTTHUMG000001	TEAD2	**TEA domain transcription factor 2**	Nucleus	transcription	MGH-CP1	-1.424511168	0	0.1695125	0	1	0	10.2605363	10.5854932	10.113461	9.8977242
1245	**ENSG0000007426**	EED	protein_coding	chr11	86201212	86278813	+	Human GRCh	OTTHUMG000001	EED	**embryonic ectoderm development**	Nucleus	transcription	APG-5918,	1.049672406	0	0.8317072	0	1	0	8.79326421	9.12208556	9.24302918	8.74542441
1246	**ENSG0000007427**	CDHR2	protein_coding	chr5	176542511	176595974	+	Human GRCh	OTTHUMG000001	CDHR2	**cadherin related family member 2**	Plasma Mem	other		-1.41930706	0	0.63159539	0	1	0	3.50535657	4.04838671	3.7429628	3.32502455
1247	**ENSG0000007431**	SNCB	protein_coding	chr5	176620082	176630556	-	Human GRCh	OTTHUMG000001	SNCB	**synuclein beta**	Cytoplasm	other		-3.477622201	-1	0.17652968	0	NA	0	3.44079683	3.25695257	3.01012864	2.0982956
1248	**ENSG0000007431**	TSG101	protein_coding	chr11	18468336	18526951	-	Human GRCh	OTTHUMG000001	TSG101	**tumor susceptibility 101**	Cytoplasm	transcription		-1.148453387	0	0.575067	0	1	0	10.2197451	10.4127274	10.2982822	10.0203846
1249	**ENSG0000007435**	NCBP3	protein_coding	chr17	3802158	3846246	-	Human GRCh	OTTHUMG000001	NCBP3	**nuclear cap binding subunit 3**	Nucleus	other		-1.060617393	0	0.76495404	0	1	0	11.2836808	11.5375441	11.4169674	11.2834561
1250	**ENSG0000007437**	ATP2A3	protein_coding	chr17	3923870	3964464	-	Human GRCh	OTTHUMG000001	ATP2A3	**ATPase sarcoplasmic/endoplasmic reticulum Ca2+ transportin**	Cytoplasm	transporter		-1.465539059	0	0.57543164	0	1	0	6.62691371	7.00811228	6.36372516	6.56958672
1251	**ENSG0000007441**	CA12	protein_coding	chr15	63321378	63381846	-	Human GRCh	OTTHUMG000001	CA12	**carbonic anhydrase 12**	Plasma Mem	enzyme	hydrochlor	1.384562703	0	0.23317932	0	1	0	11.3397017	11.6724697	12.1105826	11.6795843
1252	**ENSG0000007441**	MGLL	protein_coding	chr3	127689062	128052190	-	Human GRCh	OTTHUMG000001	MGLL	**monoglyceride lipase**	Plasma Mem	enzyme	elcubragist	-1.148594157	0	0.76989899	0	1	0	6.48704865	6.78201965	5.89578249	6.89646901
1253	**ENSG0000007452**	NTN4	protein_coding	chr12	95657807	95791189	-	Human GRCh	OTTHUMG000001	NTN4	**netrin 4**	Extracellular	other		-1.168358561	0	0.7685625	0	1	0	4.58980803	4.04803672	4.02223553	4.39998211
1254	**ENSG0000007458**	BCS1L	protein_coding	chr2	218658764	218663443	+	Human GRCh	OTTHUMG000001	BCS1L	**BCS1 homolog, ubiquinol-cytochrome c reductase complex cha**	Cytoplasm	enzyme		-1.041798024	0	0.85245575	0	1	0	9.43320847	9.82557199	9.60718552	9.59150302

1255	**ENSG0000007459**	NUAK1	protein_coding	chr12	106063345	106138954	-	Human GRCh	OTTHUMG000001	NUAK1	**NUAK family kinase 1**	Nucleus	kinase	narazacicli	-1.3933498	0	0.21208163	0	1	0	10.0545559	10.3532417	9.9505773	9.7358747
1256	**ENSG0000007460**	DPP8	protein_coding	chr15	65442463	65517704	-	Human GRCh	OTTHUMG000001	DPP8	**dipeptidyl peptidase 8**	Cytoplasm	peptidase		-1.070907499	0	0.74870885	0	1	0	10.5782884	11.0028067	10.860642	10.5907559
1257	**ENSG0000007462**	SLC24A1	protein_coding	chr15	65611366	65660995	+	Human GRCh	OTTHUMG000001	SLC24A1	**solute carrier family 24 member 1**	Plasma Mem	transporter		1.021299892	0	0.92642053	0	1	0	7.57640714	7.72171616	7.72172752	7.62191834
1258	**ENSG0000007465**	ZNF532	protein_coding	chr18	58862600	58986480	+	Human GRCh	OTTHUMG000001	ZNF532	**zinc finger protein 532**	Nucleus	transcription		-1.170978846	0	0.48581585	0	1	0	10.4209688	10.5420546	10.3143649	10.3157257
1259	**ENSG0000007466**	SCARF1	protein_coding	chr17	1633858	1645744	-	Human GRCh	OTTHUMG000000	SCARF1	**scavenger receptor class F member 1**	Plasma Mem	transmembra		1.102061107	0	0.82021335	0	1	0	5.53492425	6.12910955	6.03566484	5.89556185
1260	**ENSG0000007469**	LMAN1	protein_coding	chr18	59327823	59359265	-	Human GRCh	OTTHUMG000001	LMAN1	**lectin, mannose binding 1**	Cytoplasm	other		-1.059435434	0	0.76918277	0	1	0	11.5945153	11.8357762	11.8244686	11.4641129
1261	**ENSG0000007469**	HACD3	protein_coding	chr15	65530418	65578349	+	Human GRCh	OTTHUMG000001	HACD3	**3-hydroxyacyl-CoA dehydratase 3**	Cytoplasm	enzyme		1.034543269	0	0.86352945	0	1	0	11.4640458	11.7586003	11.8341834	11.4511541
1262	**ENSG0000007470**	IPCEF1	protein_coding	chr6	154154496	154356803	-	Human GRCh	OTTHUMG000000	IPCEF1	**interaction protein for cytohesin exchange factors 1**	Cytoplasm	enzyme		1.824564729	1	0.09149547	0	1	0	5.60543744	5.45057829	6.02813026	6.19723893
1263	**ENSG0000007475**	ZZEF1	protein_coding	chr17	4004445	4143030	-	Human GRCh	OTTHUMG000000	ZZEF1	**zinc finger ZZ-type and EF-hand domain containing 1**	Nucleus	transcription		-1.198368783	0	0.38252702	0	1	0	11.845289	12.1088096	11.8904138	11.6661702
1265	**ENSG0000007480**	ENO1	protein_coding	chr1	8861000	8879190	-	Human GRCh	OTTHUMG000000	ENO1	**enolase 1**	Cytoplasm	enzyme		-1.070586943	0	0.77298338	0	1	0	14.8900361	15.3365105	15.1424096	14.9643243
1266	**ENSG0000007480**	SLC12A1	protein_coding	chr15	48178438	48304078	+	Human GRCh	OTTHUMG000001	SLC12A1	**solute carrier family 12 member 1**	Plasma Mem	transporter	deserpidin	1	0	1	0	NA	0	0.99253729	1.00796532	1.00538953	0.99343186
1267	**ENSG0000007484**	MYDGF	protein_coding	chr19	4641374	4670362	-	Human GRCh	OTTHUMG000001	MYDGF	**myeloid derived growth factor**	Extracellular	cytokine		1.006891552	0	0.9760122	0	1	0	10.2334856	10.5834805	10.5150803	10.3361311
1268	**ENSG0000007485**	ANO8	protein_coding	chr19	17323223	17334855	-	Human GRCh	OTTHUMG000001	ANO8	**anoctamin 8**	Plasma Mem	ion channel		1.139751684	0	0.65757732	0	1	0	10.2623899	10.4677456	10.6234093	10.3988698
1269	**ENSG0000007493**	TUBE1	protein_coding	chr6	112070663	112087529	-	Human GRCh	OTTHUMG000000	TUBE1	**tubulin epsilon 1**	Cytoplasm	other	docetaxel/	1.034673952	0	0.89655622	0	1	0	9.65821199	9.1337364	9.71892261	9.13471489
1270	**ENSG0000007496**	ARHGEF10	protein_coding	chr1	17539698	17697874	+	Human GRCh	OTTHUMG000000	ARHGEF10L	**Rho guanine nucleotide exchange factor 10 like**	Cytoplasm	enzyme		1.345147557	0	0.21922574	0	1	0	7.45335379	7.74059658	7.87503036	7.92172187
1271	**ENSG0000007496**	TXK	protein_coding	chr4	48066393	48134250	-	Human GRCh	OTTHUMG000001	TXK	**TXK tyrosine kinase**	Cytoplasm	kinase	PRN694	-1.305341051	0	0.71725516	0	1	0	3.61487385	3.99426421	3.20393023	3.92020604
1273	**ENSG0000007504**	KCNQ2	protein_coding	chr20	63400208	63472677	-	Human GRCh	OTTHUMG000000	KCNQ2	**potassium voltage-gated channel subfamily Q member 2**	Plasma Mem	ion channel		1.029889978	0	0.9414476	0	1	0	4.42220266	5.09318922	4.73036031	4.90950335
1274	**ENSG0000007507**	TACR2	protein_coding	chr10	69403903	69416918	-	Human GRCh	OTTHUMG000000	TACR2	**tachykinin receptor 2**	Plasma Mem	G-protein cou	SR 48968	1.384857834	0	0.71603706	0	1	0	2.84446285	4.03455137	3.56846389	4.11719357
1275	**ENSG0000007508**	ACTR6	protein_coding	chr12	100199122	100241865	+	Human GRCh	OTTHUMG000001	ACTR6	**actin related protein 6**	Nucleus	transporter		-1.101321995	0	0.65756088	0	1	0	8.65388929	8.82852599	8.90434002	8.29767807
1276	**ENSG0000007513**	TIPIN	protein_coding	chr15	66336191	66386746	-	Human GRCh	OTTHUMG000001	TIPIN	**TIMELESS interacting protein**	Nucleus	other		-1.029867226	0	0.88966903	0	1	0	8.37760911	8.5795878	8.50178866	8.39732186
1277	**ENSG0000007514**	SRI	protein_coding	chr7	88205115	88226993	-	Human GRCh	OTTHUMG000001	SRI	**sorcin**	Cytoplasm	transporter		-1.0635822	0	0.79274192	0	1	0	9.12996703	9.51795006	9.43974047	9.07906922
1278	**ENSG0000007515**	EIF4G3	protein_coding	chr1	20806292	21177285	-	Human GRCh	OTTHUMG000000	EIF4G3	**eukaryotic translation initiation factor 4 gamma 3**	Cytoplasm	translation re		-1.109453758	0	0.57623078	0	1	0	11.441148	11.4620775	11.3744387	11.3029323
1279	**ENSG0000007518**	NUP37	protein_coding	chr12	102073103	102120120	-	Human GRCh	OTTHUMG000001	NUP37	**nucleoporin 37**	Nucleus	transporter		-1.101698358	0	0.67028262	0	1	0	9.21793787	9.38877501	9.41388413	8.93762416
1280	**ENSG0000007521**	SEMA3A	protein_coding	chr7	83955777	84492724	-	Human GRCh	OTTHUMG000000	SEMA3A	**semaphorin 3A**	Extracellular	other		-2.534604826	-1	0.2911069	0	1	0	2.34799639	3.17404408	2.739121	2.00831892
1281	**ENSG0000007521**	GTSE1	protein_coding	chr22	46296870	46330810	+	Human GRCh	OTTHUMG000001	GTSE1	**G2 and S-phase expressed 1**	Cytoplasm	other		-1.098295758	0	0.68229123	0	1	0	10.5577201	10.9216581	10.7672896	10.5225035
1282	**ENSG0000007522**	SEMA3C	protein_coding	chr7	80742538	80922359	-	Human GRCh	OTTHUMG000000	SEMA3C	**semaphorin 3C**	Extracellular	other		1.868129809	1	0.1652289	0	1	0	7.52971485	8.03699751	8.68091266	8.32223455
1283	**ENSG0000007523**	TTC38	protein_coding	chr22	46267961	46294008	+	Human GRCh	OTTHUMG000001	TTC38	**tetratricopeptide repeat domain 38**	Cytoplasm	other		-1.099065164	0	0.62764411	0	1	0	9.63421984	9.82708345	9.72024916	9.53020566
1284	**ENSG0000007523**	ACAT1	protein_coding	chr11	108116695	108147603	+	Human GRCh	OTTHUMG000001	ACAT1	**acetyl-CoA acetyltransferase 1**	Cytoplasm	enzyme		-1.090306743	0	0.74116738	0	1	0	10.6563426	10.9922745	10.8640985	10.5959964
1285	**ENSG0000007524**	GRAMD4	protein_coding	chr22	46576012	46679790	+	Human GRCh	OTTHUMG000001	GRAMD4	**GRAM domain containing 4**	Cytoplasm	other		-1.002832626	0	0.98868974	0	1	0	9.91320689	10.0827246	10.1734706	9.79218143
1286	**ENSG0000007527**	CELSR1	protein_coding	chr22	46360834	46537620	-	Human GRCh	OTTHUMG000001	CELSR1	**cadherin EGF LAG seven-pass G-type receptor 1**	Plasma Mem	G-protein cou		-1.193222157	0	0.42234607	0	1	0	9.55339143	9.43356143	9.26973053	9.35029909
1287	**ENSG0000007529**	WNT8B	protein_coding	chr10	100463009	100483744	+	Human GRCh	OTTHUMG000000	WNT8B	**Wnt family member 8B**	Extracellular	other		-1.290150959	0	0.51307133	0	1	0	6.21467132	6.53564007	5.99732925	6.21849739
1288	**ENSG0000007529**	ZNF638	protein_coding	chr2	71276561	71435069	+	Human GRCh	OTTHUMG000001	ZNF638	**zinc finger protein 638**	Nucleus	other		-1.054396	0	0.79297175	0	1	0	12.5341668	12.7278009	12.7416784	12.3853087
1289	**ENSG0000007530**	SLC25A40	protein_coding	chr7	87833568	87876360	-	Human GRCh	OTTHUMG000001	SLC25A40	**solute carrier family 25 member 40**	Cytoplasm	other		-1.006120866	0	0.98133216	0	1	0	9.83934766	10.2048626	10.2348344	9.7805682
1290	**ENSG0000007533**	TIMM21	protein_coding	chr18	74148523	74160531	+	Human GRCh	OTTHUMG000001	TIMM21	**translocase of inner mitochondrial membrane 21**	Cytoplasm	other		1.107306617	0	0.66274278	0	1	0	9.21852008	9.51914163	9.70602249	9.23142944
1291	**ENSG0000007534**	ADD2	protein_coding	chr2	70607618	70768225	-	Human GRCh	OTTHUMG000001	ADD2	**adducin 2**	Cytoplasm	other		2.652313644	1	0.59880351	0	1	0	2.82399989	2.92730718	2.91065903	3.09367519
1293	**ENSG0000007539**	RASAL2	protein_coding	chr1	178094104	178484147	+	Human GRCh	OTTHUMG000000	RASAL2	**RAS protein activator like 2**	Extracellular	other		1.130907014	0	0.57989031	0	1	0	10.0765857	10.3791236	10.5311771	10.1807451
1294	**ENSG0000007539**	VPS9D1	protein_coding	chr16	89707134	89720898	-	Human GRCh	OTTHUMG000001	VPS9D1	**VPS9 domain containing 1**	Other	other		1.014316902	0	0.94836893	0	1	0	8.30005187	8.48944616	8.40840961	8.41830225
1295	**ENSG0000007540**	ZNF37A	protein_coding	chr10	38094334	38150293	+	Human GRCh	OTTHUMG000001	ZNF37A	**zinc finger protein 37A**	Nucleus	transcription		-1.003298322	0	0.98631001	0	1	0	11.2953874	11.5552715	11.5511741	11.2921817
1296	**ENSG0000007541**	MARK3	protein_coding	chr14	103385377	103503831	+	Human GRCh	OTTHUMG000001	MARK3	**microtubule affinity regulating kinase 3**	Cytoplasm	kinase	cdc25C ph	-1.024366955	0	0.90781729	0	1	0	11.9642271	12.1127482	12.2017673	11.7951127
1297	**ENSG0000007541**	SLC25A3	protein_coding	chr12	98593591	98606379	+	Human GRCh	OTTHUMG000001	SLC25A3	**solute carrier family 25 member 3**	Cytoplasm	transporter		-1.089728811	0	0.67425985	0	1	0	12.707398	13.0722472	12.9663474	12.6239616
1298	**ENSG0000007542**	FNDC3B	protein_coding	chr3	172039578	172401669	+	Human GRCh	OTTHUMG000001	FNDC3B	**fibronectin type III domain containing 3B**	Cytoplasm	other		1.023285487	0	0.91691876	0	1	0	11.9297412	12.0988691	12.1315975	11.9465357
1299	**ENSG0000007542**	FOSL2	protein_coding	chr2	28392448	28417317	+	Human GRCh	OTTHUMG000000	FOSL2	**FOS like 2, AP-1 transcription factor subunit**	Nucleus	transcription		-1.07797288	0	0.71900956	0	1	0	11.7396502	12.0220571	11.9203877	11.6720265
1300	**ENSG0000007542**	CACNG5	protein_coding	chr17	66835117	66894751	+	Human GRCh	OTTHUMG000001	CACNG5	**calcium voltage-gated channel auxiliary subunit gamma 5**	Plasma Mem	ion channel		1	0	1	0	1	0	2.07631754	2.19264147	2.17404536	2.08341088
1301	**ENSG0000007546**	CACNG4	protein_coding	chr17	66964707	67033398	+	Human GRCh	OTTHUMG000001	CACNG4	**calcium voltage-gated channel auxiliary subunit gamma 4**	Plasma Mem	ion channel		1.511576337	1	0.38957004	0	1	0	4.32884287	3.86094184	4.42039951	4.50412319
1302	**ENSG0000007553**	FRYL	protein_coding	chr4	48497357	48780322	-	Human GRCh	OTTHUMG000001	FRYL	**FRY like transcription coactivator**	Other	other		-1.13271107	0	0.55622627	0	1	0	12.0650751	12.2861718	12.1948571	11.8687728
1303	**ENSG0000007556**	TMEM131	protein_coding	chr2	97756333	97995948	-	Human GRCh	OTTHUMG000001	TMEM131	**transmembrane protein 131**	Extracellular	other		-1.158189925	0	0.49283746	0	1	0	11.8816895	12.2827398	12.1038668	11.7413101
1304	**ENSG0000007561**	FSCN1	protein_coding	chr7	5592816	5606655	+	Human GRCh	OTTHUMG000000	FSCN1	**fascin actin-bundling protein 1**	Cytoplasm	other		-1.103234828	0	0.74023774	0	1	0	8.73098	8.85803072	8.65955404	8.73056096
1305	**ENSG0000007562**	ACTB	protein_coding	chr7	5526409	5563902	-	Human GRCh	OTTHUMG000000	ACTB	**actin beta**	Cytoplasm	other		-1.118401322	0	0.56443096	0	1	0	15.6893764	15.9776728	15.7757935	15.6555618
1306	**ENSG0000007564**	MOCOS	protein_coding	chr18	36187497	36272157	+	Human GRCh	OTTHUMG000001	MOCOS	**molybdenum cofactor sulfurase**	Cytoplasm	enzyme		1.020762727	0	0.93698612	0	1	0	9.05292907	9.17449405	9.14090878	9.1335975
1307	**ENSG0000007565**	PLD1	protein_coding	chr3	171600404	171810950	-	Human GRCh	OTTHUMG000001	PLD1	**phospholipase D1**	Cytoplasm	enzyme		-1.643285771	-1	0.39454709	0	1	0	4.6868468	4.1875533	3.91293815	4.23052488
1309	**ENSG0000007570**	WDR62	protein_coding	chr19	36054649	36105108	+	Human GRCh	OTTHUMG000000	WDR62	**WD repeat domain 62**	Nucleus	other		-1.217536648	0	0.34089137	0	1	0	11.6392857	12.0505337	11.7631079	11.5173766
1310	**ENSG0000007571**	DLG1	protein_coding	chr3	197042560	197299330	-	Human GRCh	OTTHUMG000000	DLG1	**discs large MAGUK scaffold protein 1**	Cytoplasm	other		-1.132515717	0	0.5276163	0	1	0	12.0155624	12.2915297	12.1546507	11.8870207
1311	**ENSG0000007578**	RAB7A	protein_coding	chr3	128693669	128825942	+	Human GRCh	OTTHUMG000001	RAB7A	**RAB7A, member RAS oncogene family**	Cytoplasm	enzyme		-1.021896644	0	0.90558994	0	1	0	12.094358	12.400274	12.2964692	12.1657671
1312	**ENSG0000007579**	BCAP29	protein_coding	chr7	107580246	107629170	+	Human GRCh	OTTHUMG000001	BCAP29	**B cell receptor associated protein 29**	Cytoplasm	transporter		-1.217203222	0	0.45240736	0	1	0	10.3246029	10.6008195	10.6262321	9.69276743
1313	**ENSG0000007582**	SEC31B	protein_coding	chr10	100486646	100519864	-	Human GRCh	OTTHUMG000000	SEC31B	**SEC31 homolog B, COPII coat complex component**	Cytoplasm	other		-1.108936571	0	0.68614826	0	1	0	9.37612007	9.60873559	9.54932491	9.19148151
1314	**ENSG0000007585**	SART3	protein_coding	chr12	108522214	108561400	-	Human GRCh	OTTHUMG000001	SART3	**spliceosome associated factor 3, U4/U6 recycling protein**	Nucleus	other		1.004935048	0	0.98112072	0	1	0	10.8837062	11.1685866	11.1722786	10.8912821
1315	**ENSG0000007588**	ARHGAP15	protein_coding	chr2	143091362	143768352	+	Human GRCh	OTTHUMG000001	ARHGAP15	**Rho GTPase activating protein 15**	Cytoplasm	other		1.219541727	0	0.65543648	0	1	0	5.46656761	5.47155112	5.65633422	5.71076742
1316	**ENSG0000007588**	TUBA3D	protein_coding	chr2	131476119	131482934	+	Human GRCh	OTTHUMG000001	TUBA3C/TUB	**tubulin alpha 3c**	Nucleus	other	docetaxel/	1.260524262	0	0.85264538	0	NA	0	3.0370495	2.91360501	3.13863263	3.20123326
1317	**ENSG0000007589**	PAX2	protein_coding	chr10	100735396	100829944	+	Human GRCh	OTTHUMG000000	PAX2	**paired box 2**	Nucleus	transcription		-5.345874216	-1	0.14764247	0	NA	0	2.56147613	3.26780972	1.7867379	2.01540358
1318	**ENSG0000007591**	EXOSC7	protein_coding	chr3	44975241	45036066	+	Human GRCh	OTTHUMG000001	EXOSC7	**exosome component 7**	Nucleus	enzyme		-1.076187173	0	0.74138726	0	1	0	9.09620319	9.50412709	9.42260629	9.01460613
1319	**ENSG0000007594**	KIFAP3	protein_coding	chr1	169921326	170085208	-	Human GRCh	OTTHUMG000000	KIFAP3	**kinesin associated protein 3**	Cytoplasm	other		1.015572162	0	0.93894291	0	1	0	10.1673527	10.297648	10.383815	10.1022782
1320	**ENSG0000007597**	MKRN2	protein_coding	chr3	12557057	12586208	+	Human GRCh	OTTHUMG000001	MKRN2	**makorin ring finger protein 2**	Nucleus	enzyme		-1.132441368	0	0.49719129	0	1	0	9.90815186	10.0985981	10.000038	9.72407013
1321	**ENSG0000007600**	MCM6	protein_coding	chr2	135839626	135876443	-	Human GRCh	OTTHUMG000001	MCM6	**minichromosome maintenance complex component 6**	Nucleus	enzyme		-1.07199757	0	0.77303928	0	1	0	10.4562601	10.9379956	10.7773376	10.499879
1322	**ENSG0000007604**	REXO2	protein_coding	chr11	114439435	114450279	+	Human GRCh	OTTHUMG000001	REXO2	**RNA exonuclease 2**	Cytoplasm	enzyme		-1.079860628	0	0.71822306	0	1	0	9.63634563	9.98331023	9.89664936	9.55334885
1323	**ENSG0000007605**	RBM7	protein_coding	chr11	114400030	114414203	+	Human GRCh	OTTHUMG000001	RBM7	**RNA binding motif protein 7**	Nucleus	other		1.004957483	0	0.98340887	0	1	0	9.14796176	9.23409325	9.36805864	8.99741934
1324	**ENSG0000007606**	RBMS2	protein_coding	chr12	56521820	56596193	+	Human GRCh	OTTHUMG000001	RBMS2	**RNA binding motif single stranded interacting protein 2**	Nucleus	other		-1.027559185	0	0.92474657	0	1	0	9.26473922	9.4134267	9.32102227	9.30318639
1325	**ENSG0000007610**	BAZ2A	protein_coding	chr12	56595596	56636816	-	Human GRCh	OTTHUMG000001	BAZ2A	**bromodomain adjacent to zinc finger domain 2A**	Nucleus	transcription		-1.145496476	0	0.49525802	0	1	0	13.2189425	13.5833566	13.3209192	13.2036346
1326	**ENSG0000007620**	PTPN23	protein_coding	chr3	47381011	47413435	+	Human GRCh	OTTHUMG000001	PTPN23	**protein tyrosine phosphatase non-receptor type 23**	Cytoplasm	phosphatase		1.059075863	0	0.75206236	0	1	0	11.4696427	11.7241365	11.7267689	11.6041864
1327	**ENSG0000007624**	MLH1	protein_coding	chr3	36993226	37050896	+	Human GRCh	OTTHUMG000001	MLH1	**mutL homolog 1**	Nucleus	enzyme		-1.089269829	0	0.65599327	0	1	0	10.4889509	10.8023106	10.7388183	10.3524425
1328	**ENSG0000007624**	UNG	protein_coding	chr12	109097597	109126725	+	Human GRCh	OTTHUMG000001	UNG	**uracil DNA glycosylase**	Nucleus	enzyme		-1.093019557	0	0.66844609	0	1	0	11.153328	11.4993556	11.3726452	11.0897878
1329	**ENSG0000007625**	FMO4	protein_coding	chr1	171314183	171342084	+	Human GRCh	OTTHUMG000000	FMO4	**flavin containing dimethylaniline monoxygenase 4**	Cytoplasm	enzyme		-1.072108294	0	0.87947337	0	1	0	5.01971671	5.2388143	4.92792517	5.19183626
1330	**ENSG0000007632**	KLHL20	protein_coding	chr1	173714941	173786692	+	Human GRCh	OTTHUMG000000	KLHL20	**kelch like family member 20**	Plasma Mem	enzyme		-1.002484636	0	0.99195311	0	1	0	8.72860801	9.09092294	9.02715994	8.80379973
1331	**ENSG0000007634**	RGS11	protein_coding	chr16	268301	275980	-	Human GRCh	OTTHUMG000000	RGS11	**regulator of G protein signaling 11**	Plasma Mem	enzyme		1.247311155	0	0.54939542	0	1	0	6.22402812	7.06543978	7.06922448	6.8175132
1332	**ENSG0000007635**	SLC46A1	protein_coding	chr17	28394642	28407197	-	Human GRCh	OTTHUMG000001	SLC46A1	**solute carrier family 46 member 1**	Plasma Mem	transporter		-1.125681954	0	0.5744346	0	1	0	10.5510475	10.6894137	10.7011156	10.2395595
1333	**ENSG0000007635**	PLXNA2	protein_coding	chr1	208022242	208244384	-	Human GRCh	OTTHUMG000000	PLXNA2	**plexin A2**	Plasma Mem	transmembra		1.392916829	0	0.49547328	0	1	0	9.51495426	9.47937857	10.0703784	9.48716762
1334	**ENSG0000007638**	SPAG5	protein_coding	chr17	28577565	28599025	-	Human GRCh	OTTHUMG000001	SPAG5	**sperm associated antigen 5**	Nucleus	peptidase		-1.225187787	0	0.32639134	0	1	0	11.9390463	12.3861958	12.0142289	11.906078
1335	**ENSG0000007651**	ANKRD13A	protein_coding	chr12	109999186	110039763	+	Human GRCh	OTTHUMG000001	ANKRD13A	**ankyrin repeat domain 13A**	Plasma Mem	other		-1.189683699	0	0.45885101	0	1	0	10.7561236	11.101734	10.9144121	10.5559413
1336	**ENSG0000007655**	TPD52	protein_coding	chr8	80034745	80231232	-	Human GRCh	OTTHUMG000001	TPD52	**tumor protein D52**	Cytoplasm	other		-1.048704162	0	0.85209521	0	1	0	12.0861084	12.4008608	12.3255168	12.0678199
1337	**ENSG0000007655**	ACACB	protein_coding	chr12	109116587	109268226	+	Human GRCh	OTTHUMG000001	ACACB	**acetyl-CoA carboxylase beta**	Cytoplasm	enzyme		-1.14456553	0	0.56574139	0	1	0	9.09859444	9.34421084	9.06171217	9.1016741
1338	**ENSG0000007660**	TRAF4	protein_coding	chr17	28744011	28750956	+	Human GRCh	OTTHUMG000001	TRAF4	**TNF receptor associated factor 4**	Cytoplasm	other		-1.17871852	0	0.44827104	0	1	0	11.7659704	12.0634059	11.8393942	11.6278376
1339	**ENSG0000007664**	PAG1	protein_coding	chr8	80967810	81112068	-	Human GRCh	OTTHUMG000001	PAG1	**phosphoprotein membrane anchor with glycosphingolipid mic**	Plasma Mem	other		1.267330795	0	0.38607317	0	1	0	8.2126106	8.29536128	8.63744059	8.39116213
1340	**ENSG0000007665**	GPATCH1	protein_coding	chr19	33080899	33130542	+	Human GRCh	OTTHUMG000001	GPATCH1	**G-patch domain containing 1**	Extracellular	other		-1.200131761	0	0.43425989	0	1	0	8.87828808	9.32474004	9.0876907	8.73068698
1341	**ENSG0000007666**	ICAM3	protein_coding	chr19	10333776	10339661	-	Human GRCh	OTTHUMG000001	ICAM3	**intercellular adhesion molecule 3**	Plasma Mem	transmembra		-1.059328686	0	0.84831903	0	1	0	7.83275882	8.31458223	8.02267678	8.04049258

1342	**ENSG0000007668**	NT5C2	protein_coding	chr10	103087185	103277605	-	Human GRCh	OTTHUMG000000	NT5C2	**5'-nucleotidase, cytosolic II**	Cytoplasm	phosphatase		-1.147878895	0	0.55979137	0	1	0	10.8028014	11.1348342	11.0072068	10.6149211
1343	**ENSG0000007670**	MCAM	protein_coding	chr11	119308529	119321521	-	Human GRCh	OTTHUMG000001	MCAM	**melanoma cell adhesion molecule**	Plasma Mem	other		2.314552089	1	0.01657636	1	1	0	6.67348617	6.4073554	6.74147791	7.73870127
1344	**ENSG0000007671**	GPC4	protein_coding	chrX	133300103	133415489	-	Human GRCh	OTTHUMG000000	GPC4	**glypican 4**	Plasma Mem	transmembra		-1.087536609	0	0.73683379	0	1	0	9.13735218	9.34716166	9.21894082	9.08399879
1345	**ENSG0000007677**	MBNL3	protein_coding	chrX	132369320	132489968	-	Human GRCh	OTTHUMG000000	MBNL3	**muscleblind like splicing regulator 3**	Nucleus	other		-1.125474525	0	0.70489305	0	1	0	10.7890102	11.0576872	10.9608687	10.6140932
1346	**ENSG0000007682**	CAMSAP3	protein_coding	chr19	7595863	7618304	+	Human GRCh	OTTHUMG000001	CAMSAP3	**calmodulin regulated spectrin associated protein family memb**	Cytoplasm	other		-1.011075968	0	0.95732389	0	1	0	9.62284317	9.87555858	9.70976523	9.77677452
1347	**ENSG0000007686**	RAP1GAP	protein_coding	chr1	21596221	21669357	-	Human GRCh	OTTHUMG000000	RAP1GAP	**RAP1 GTPase activating protein**	Cytoplasm	other		1.089547269	0	0.77436622	0	1	0	6.85212071	6.94873812	6.93914479	7.02834883
1348	**ENSG0000007692**	XAB2	protein_coding	chr19	7619525	7629545	-	Human GRCh	OTTHUMG000001	XAB2	**XPA binding protein 2**	Nucleus	other		-1.109237375	0	0.60688709	0	1	0	10.888108	11.1949217	10.9734997	10.8975183
1349	**ENSG0000007692**	ARHGEF1	protein_coding	chr19	41883173	41930150	+	Human GRCh	OTTHUMG000001	ARHGEF1	**Rho guanine nucleotide exchange factor 1**	Cytoplasm	other		1.046347084	0	0.83354284	0	1	0	10.2995367	10.6877554	10.6935541	10.407706
1350	**ENSG0000007694**	STXBP2	protein_coding	chr19	7636772	7647873	+	Human GRCh	OTTHUMG000001	STXBP2	**syntaxin binding protein 2**	Plasma Mem	transporter		-1.128417064	0	0.56108933	0	1	0	10.7727466	11.0829729	10.792553	10.8147541
1351	**ENSG0000007698**	MAP2K7	protein_coding	chr19	7903843	7914478	+	Human GRCh	OTTHUMG000001	MAP2K7	**mitogen-activated protein kinase kinase 7**	Cytoplasm	kinase		1.08690257	0	0.68746162	0	1	0	10.9855435	11.2650563	11.332275	11.1086534
1353	**ENSG0000007704**	DGKD	protein_coding	chr2	233354494	233472104	+	Human GRCh	OTTHUMG000001	DGKD	**diacylglycerol kinase delta**	Cytoplasm	kinase		-1.022911537	0	0.90493841	0	1	0	10.1751927	10.3634108	10.3628853	10.1212418
1354	**ENSG0000007706**	CTTNBP2	protein_coding	chr7	117710651	117874139	-	Human GRCh	OTTHUMG000000	CTTNBP2	**cortactin binding protein 2**	Cytoplasm	other		-1.56589379	-1	0.16651401	0	1	0	6.97372182	6.95255939	6.58051813	6.49078827
1355	**ENSG0000007708**	ACTL6B	protein_coding	chr7	100643097	100656448	-	Human GRCh	OTTHUMG000001	ACTL6B	**actin like 6B**	Nucleus	other		2.683258916	1	0.48881026	0	1	0	2.01693746	2.12858315	2.11073165	2.36973472
1356	**ENSG0000007709**	RARB	protein_coding	chr3	24687887	25597932	+	Human GRCh	OTTHUMG000001	RARB	**retinoic acid receptor beta**	Nucleus	ligand-depen	daunorubi	-1.081096346	0	0.81577285	0	1	0	9.74010806	9.9529931	9.95487424	9.52806269
1357	**ENSG0000007709**	TOP2B	protein_coding	chr3	25597978	25664909	-	Human GRCh	OTTHUMG000001	TOP2B	**DNA topoisomerase II beta**	Nucleus	enzyme	daunorubi	-1.100582955	0	0.63031652	0	1	0	12.8899259	13.1793204	13.1094089	12.734343
1358	**ENSG0000007714**	TM9SF3	protein_coding	chr10	96518110	96587452	-	Human GRCh	OTTHUMG000000	TM9SF3	**transmembrane 9 superfamily member 3**	Cytoplasm	transporter		1.049133464	0	0.80516584	0	1	0	12.1277102	12.2966758	12.4440679	12.0624563
1359	**ENSG0000007715**	NFKB2	protein_coding	chr10	102394110	102402524	+	Human GRCh	OTTHUMG000000	NFKB2	**nuclear factor kappa B subunit 2**	Nucleus	transcription		1.119981061	0	0.6250676	0	1	0	10.0326218	10.3481705	10.41929	10.2341267
1360	**ENSG0000007715**	UBE2T	protein_coding	chr1	202331544	202341984	-	Human GRCh	OTTHUMG000000	UBE2T	**ubiquitin conjugating enzyme E2 T**	Nucleus	enzyme		-1.085355243	0	0.74759151	0	1	0	10.17671	10.5193674	10.3360969	10.1974855
1361	**ENSG0000007715**	PPP1R12B	protein_coding	chr1	202348699	202592706	+	Human GRCh	OTTHUMG000000	PPP1R12B	**protein phosphatase 1 regulatory subunit 12B**	Cytoplasm	phosphatase		-1.193142619	0	0.40867813	0	1	0	11.8426192	12.1780883	11.8919094	11.7596949
1362	**ENSG0000007723**	DNAJC10	protein_coding	chr2	182716255	182794464	+	Human GRCh	OTTHUMG000001	DNAJC10	**DnaJ heat shock protein family (Hsp40) member C10**	Cytoplasm	enzyme		-1.154005241	0	0.49810261	0	1	0	12.0435216	12.2400921	12.0968018	11.8676416
1363	**ENSG0000007723**	GTF3C1	protein_coding	chr16	27459555	27549913	-	Human GRCh	OTTHUMG000001	GTF3C1	**general transcription factor IIIC subunit 1**	Nucleus	transcription		-1.05015335	0	0.82318222	0	1	0	12.48212	12.7501699	12.7398419	12.3683346
1364	**ENSG0000007723**	IL4R	protein_coding	chr16	27313668	27364778	+	Human GRCh	OTTHUMG000000	IL4R	**interleukin 4 receptor**	Plasma Mem	transmembra	dupilumab	7.866153369	1	0.00017643	1	0.24	0	4.41723124	4.30240532	7.1134747	4.7474944
1365	**ENSG0000007725**	USP33	protein_coding	chr1	77695987	77759852	-	Human GRCh	OTTHUMG000000	USP33	**ubiquitin specific peptidase 33**	Cytoplasm	peptidase		-1.121224297	0	0.58511613	0	1	0	11.1879286	11.3231222	11.2537339	10.9943651
1366	**ENSG0000007726**	PAK3	protein_coding	chrX	110944285	111227361	+	Human GRCh	OTTHUMG000000	PAK3	**p21 (RAC1) activated kinase 3**	Cytoplasm	kinase	FRAX-597,	1.10483498	0	0.91159896	0	1	0	3.15614173	2.97846959	3.05041846	3.23311753
1368	**ENSG0000007727**	DCX	protein_coding	chrX	111293779	111412429	-	Human GRCh	OTTHUMG000000	DCX	**doublecortin**	Cytoplasm	other		1	0	1	0	NA	0	0.22457461	0.26054991	0.25468437	0.2267181
1369	**ENSG0000007731**	SNRPA	protein_coding	chr19	40750637	40765389	+	Human GRCh	OTTHUMG000001	SNRPA	**small nuclear ribonucleoprotein polypeptide A**	Nucleus	other		-1.054989918	0	0.79499545	0	1	0	11.1609808	11.4765937	11.2816729	11.256093
1370	**ENSG0000007732**	SPAG6	protein_coding	chr10	22345445	22454224	+	Human GRCh	OTTHUMG000000	SPAG6	**sperm associated antigen 6**	Cytoplasm	other		-2.408989187	-1	0.21790119	0	1	0	3.1569606	2.32046387	2.7457858	1.47662052
1371	**ENSG0000007734**	EXOSC5	protein_coding	chr19	41386371	41397362	-	Human GRCh	OTTHUMG000001	EXOSC5	**exosome component 5**	Nucleus	enzyme		-1.105549411	0	0.67759599	0	1	0	9.66474859	9.99415643	9.82859386	9.61752855
1372	**ENSG0000007738**	DYNC1I2	protein_coding	chr2	171687409	171750158	+	Human GRCh	OTTHUMG000001	DYNC1I2	**dynein cytoplasmic 1 intermediate chain 2**	Cytoplasm	other		-1.039566971	0	0.85249015	0	1	0	9.57856239	9.81837668	9.7796473	9.53305934
1374	**ENSG0000007745**	LRCH4	protein_coding	chr7	100574011	100586129	-	Human GRCh	OTTHUMG000001	LRCH4	**leucine rich repeats and calponin homology domain containing**	Cytoplasm	transcription		1.133361092	0	0.59121207	0	1	0	11.4041372	11.665393	11.7423907	11.6201539
1375	**ENSG0000007745**	FAM76B	protein_coding	chr11	95768953	95790409	-	Human GRCh	OTTHUMG000001	FAM76B	**family with sequence similarity 76 member B**	Nucleus	other		-1.069219132	0	0.77295626	0	1	0	9.41725518	9.71400079	9.69205336	9.27679417
1376	**ENSG0000007746**	SIRT6	protein_coding	chr19	4174109	4182566	-	Human GRCh	OTTHUMG000001	SIRT6	**sirtuin 6**	Nucleus	enzyme		1.070512428	0	0.73396176	0	1	0	9.23852109	9.5340759	9.56337927	9.37022245
1378	**ENSG0000007751**	POLD3	protein_coding	chr11	74493851	74669117	+	Human GRCh	OTTHUMG000001	POLD3	**DNA polymerase delta 3, accessory subunit**	Nucleus	transcription		-1.144762429	0	0.51170096	0	1	0	10.3112417	10.6213446	10.4497167	10.1973085
1379	**ENSG0000007752**	ACTN2	protein_coding	chr1	236664141	236764631	+	Human GRCh	OTTHUMG000000	ACTN2	**actinin alpha 2**	Nucleus	transcription		-1.006726064	0	0.99073872	0	1	0	4.99487661	5.14790491	5.39311717	4.61066335
1380	**ENSG0000007754**	CAPZB	protein_coding	chr1	19338775	19485539	-	Human GRCh	OTTHUMG000000	CAPZB	**capping actin protein of muscle Z-line subunit beta**	Cytoplasm	other	vacuolin-1	-1.154005659	0	0.61387538	0	1	0	12.9331325	13.1671944	13.1342469	12.599602
1381	**ENSG0000007758**	GPR137B	protein_coding	chr1	236142505	236221865	+	Human GRCh	OTTHUMG000000	GPR137B	**G protein-coupled receptor 137B**	Plasma Mem	other		-1.117835887	0	0.60729334	0	1	0	8.55418972	8.81585686	8.71394047	8.40800275
1383	**ENSG0000007768**	JADE1	protein_coding	chr4	128809700	128875224	+	Human GRCh	OTTHUMG000001	JADE1	**jade family PHD finger 1**	Nucleus	transcription		1.056172363	0	0.81730082	0	1	0	11.3294346	11.6535576	11.720672	11.38392
1384	**ENSG0000007771**	SLC25A43	protein_coding	chrX	119399336	119454478	+	Human GRCh	OTTHUMG000000	SLC25A43	**solute carrier family 25 member 43**	Cytoplasm	transporter		-1.047199843	0	0.90443682	0	1	0	6.09451551	6.67528292	6.31859846	6.42481
1385	**ENSG0000007772**	UBE2A	protein_coding	chrX	119474802	119591083	+	Human GRCh	OTTHUMG000000	UBE2A	**ubiquitin conjugating enzyme E2 A**	Cytoplasm	enzyme		-1.151976661	0	0.47863483	0	1	0	9.98790247	10.2406134	10.073671	9.84558973
1386	**ENSG0000007778**	FGFR1	protein_coding	chr8	38400215	38468834	-	Human GRCh	OTTHUMG000001	FGFR1	**fibroblast growth factor receptor 1**	Plasma Mem	kinase	tasurgratir	1.174598026	0	0.57784987	0	1	0	11.0270689	11.3459799	11.4891664	11.2663391
1388	**ENSG0000007793**	SMC1B	protein_coding	chr22	45344063	45413619	-	Human GRCh	OTTHUMG000001	SMC1B	**structural maintenance of chromosomes 1B**	Nucleus	enzyme		1.080990676	0	0.79504761	0	1	0	6.51521004	7.0966852	7.07020489	6.75228686
1389	**ENSG0000007794**	FBLN1	protein_coding	chr22	45502238	45601135	+	Human GRCh	OTTHUMG000001	FBLN1	**fibulin 1**	Extracellular	other		-2.077507924	-1	0.02774568	1	1	0	7.32804797	7.75211579	7.00973689	6.7991347
1390	**ENSG0000007794**	ITGA8	protein_coding	chr10	15513954	15719922	-	Human GRCh	OTTHUMG000000	ITGA8	**integrin subunit alpha 8**	Plasma Mem	other		1.401751884	0	0.87137002	0	1	0	2.3647497	0.88024942	1.70068252	2.37576245
1392	**ENSG0000007801**	MAP2	protein_coding	chr2	209424047	209734147	+	Human GRCh	OTTHUMG000001	MAP2	**microtubule associated protein 2**	Plasma Mem	other	estramusti	-2.043948148	-1	0.24861657	0	1	0	6.89553284	7.24907827	6.51856231	6.53092699
1393	**ENSG0000007804**	PIAS2	protein_coding	chr18	46803218	46920160	-	Human GRCh	OTTHUMG000001	PIAS2	**protein inhibitor of activated STAT 2**	Nucleus	transcription		1.007270638	0	0.97698417	0	1	0	11.2599266	11.5535444	11.6403825	11.1580281
1394	**ENSG0000007805**	AMPH	protein_coding	chr7	38383704	38631420	-	Human GRCh	OTTHUMG000000	AMPH	**amphiphysin**	Plasma Mem	other		1.233328794	0	0.77032036	0	1	0	4.94916192	4.93592616	3.68854258	5.71325595
1395	**ENSG0000007806**	ARAF	protein_coding	chrX	47561205	47571908	+	Human GRCh	OTTHUMG000000	ARAF	**A-Raf proto-oncogene, serine/threonine kinase**	Cytoplasm	kinase	cetuximab	-1.102902229	0	0.62605021	0	1	0	10.4642136	10.7398454	10.6037574	10.3926296
1396	**ENSG0000007807**	MCCC1	protein_coding	chr3	183015218	183116075	-	Human GRCh	OTTHUMG000001	MCCC1	**methylcrotonyl-CoA carboxylase subunit 1**	Cytoplasm	enzyme		-1.099298373	0	0.68657796	0	1	0	10.6438247	10.9864457	10.875189	10.544824
1397	**ENSG0000007808**	LAMP3	protein_coding	chr3	183122215	183163839	-	Human GRCh	OTTHUMG000001	LAMP3	**lysosomal associated membrane protein 3**	Plasma Mem	other		-1.241646866	0	0.55538851	0	1	0	5.07052459	5.10139564	4.76258789	5.00447358
1399	**ENSG0000007811**	NEBL	protein_coding	chr10	20779973	21293011	-	Human GRCh	OTTHUMG000000	NEBL	**nebulette**	Plasma Mem	other		-1.027492928	0	0.92610071	0	1	0	10.5856934	10.83166	10.6153796	10.751668
1400	**ENSG0000007812**	ACER3	protein_coding	chr11	76860859	77026797	+	Human GRCh	OTTHUMG000001	ACER3	**alkaline ceramidase 3**	Cytoplasm	enzyme		1.042678705	0	0.88370176	0	1	0	9.15289329	9.42871471	9.39718693	9.28597911
1401	**ENSG0000007814**	UBE2K	protein_coding	chr4	39698109	39782792	+	Human GRCh	OTTHUMG000001	UBE2K	**ubiquitin conjugating enzyme E2 K**	Cytoplasm	transcription		1.08956055	0	0.69711011	0	1	0	11.6316853	11.9928377	12.1529556	11.6315278
1402	**ENSG0000007814**	PIK3C3	protein_coding	chr18	41955234	42087830	+	Human GRCh	OTTHUMG000001	PIK3C3	**phosphatidylinositol 3-kinase catalytic subunit type 3**	Cytoplasm	kinase	PIK-III, VPS	-1.095626088	0	0.6862313	0	1	0	10.5649454	10.9127189	10.8091531	10.4668552
1403	**ENSG0000007817**	N4BP2	protein_coding	chr4	40056797	40158252	+	Human GRCh	OTTHUMG000001	N4BP2	**NEDD4 binding protein 2**	Cytoplasm	enzyme		-1.102768443	0	0.65350265	0	1	0	11.0963972	11.2929632	11.3283867	10.7952448
1404	**ENSG0000007823**	TIGAR	protein_coding	chr12	4307763	4360028	+	Human GRCh	OTTHUMG000001	TIGAR	**TP53 induced glycolysis regulatory phosphatase**	Cytoplasm	enzyme		1.010613045	0	0.96963347	0	1	0	8.06009303	8.22077153	8.30215982	7.98674145
1405	**ENSG0000007824**	TULP3	protein_coding	chr12	2877223	2941138	+	Human GRCh	OTTHUMG000001	TULP3	**TUB like protein 3**	Extracellular	other		-1.221007793	0	0.37571829	0	1	0	10.56567	10.9926765	10.6300992	10.5071383
1406	**ENSG0000007826**	SYNJ2	protein_coding	chr6	157981863	158099176	+	Human GRCh	OTTHUMG000000	SYNJ2	**synaptojanin 2**	Cytoplasm	phosphatase		-1.00970633	0	0.96711859	0	1	0	9.29739384	9.6448118	9.4516296	9.49529596
1407	**ENSG0000007829**	ADCY2	protein_coding	chr5	7396138	7830081	+	Human GRCh	OTTHUMG000000	ADCY2	**adenylate cyclase 2**	Plasma Mem	enzyme		1.968300908	1	0.58874528	0	1	0	2.60533578	2.41475902	2.39931401	2.84520275
1408	**ENSG0000007830**	PPP2R5C	protein_coding	chr14	101761709	101927989	+	Human GRCh	OTTHUMG000001	PPP2R5C	**protein phosphatase 2 regulatory subunit B'gamma**	Nucleus	other		1.022832261	0	0.91533206	0	1	0	11.9799525	12.258916	12.1900431	12.1142721
1409	**ENSG0000007832**	RBFOX1	protein_coding	chr16	5239802	7713340	+	Human GRCh	OTTHUMG000001	RBFOX1	**RNA binding fox-1 homolog 1**	Cytoplasm	other		10.13085861	1	0.03923429	1	1	0	1.72494847	2.42361868	1.7841493	4.08461192
1410	**ENSG0000007836**	GNB1	protein_coding	chr1	1785285	1892292	-	Human GRCh	OTTHUMG000000	GNB1	**G protein subunit beta 1**	Plasma Mem	other		-1.16991234	0	0.44291236	0	1	0	13.2322365	13.5858174	13.3495121	13.1388515
1411	**ENSG0000007839**	HOXA9	protein_coding	chr7	27162438	27175180	-	Human GRCh	OTTHUMG000000	HOXA9	**homeobox A9**	Nucleus	transcription		1.251628938	0	0.69449035	0	1	0	7.9678166	8.35993664	8.4237498	8.37881801
1412	**ENSG0000007840**	EDN1	protein_coding	chr6	12290361	12297194	+	Human GRCh	OTTHUMG000000	EDN1	**endothelin 1**	Extracellular	cytokine		1.074498625	0	0.92210553	0	1	0	4.87319571	4.39747729	4.12765684	5.1187358
1413	**ENSG0000007840**	MLLT10	protein_coding	chr10	21524646	21743630	+	Human GRCh	OTTHUMG000000	MLLT10	**MLLT10 histone lysine methyltransferase DOT1L cofactor**	Nucleus	transcription		-1.048078591	0	0.81413354	0	1	0	11.203418	11.4361096	11.4608495	11.0513825
1414	**ENSG0000007848**	ZCWPW1	protein_coding	chr7	100400826	100428992	-	Human GRCh	OTTHUMG000001	ZCWPW1	**zinc finger CW-type and PWWP domain containing 1**	Nucleus	other		1.272732887	0	0.52120963	0	1	0	4.8641679	5.50220614	5.63943543	5.23188353
1415	**ENSG0000007854**	ADCYAP1R	protein_coding	chr7	31052308	31111474	+	Human GRCh	OTTHUMG000000	ADCYAP1R1	**ADCYAP receptor type I**	Plasma Mem	G-protein cou		-7.899422464	-1	0.13941413	0	NA	0	2.8608248	1.76976387	1.20479493	1.68278888
1419	**ENSG0000007861**	NRDC	protein_coding	chr1	51789191	51878805	-	Human GRCh	OTTHUMG000000	NRDC	**nardilysin convertase**	Cytoplasm	peptidase		-1.105567384	0	0.63882834	0	1	0	12.0170284	12.4775913	12.3267311	11.9610432
1420	**ENSG0000007866**	VDAC3	protein_coding	chr8	42391624	42405937	+	Human GRCh	OTTHUMG000001	VDAC3	**voltage dependent anion channel 3**	Cytoplasm	ion channel		-1.105693684	0	0.65916074	0	1	0	10.6470372	11.1117422	10.9834346	10.5554741
1421	**ENSG0000007867**	PCM1	protein_coding	chr8	17922842	18029948	+	Human GRCh	OTTHUMG000001	PCM1	**pericentriolar material 1**	Cytoplasm	other		-1.171537184	0	0.44490981	0	1	0	12.8275131	13.1655814	13.0081684	12.6260612
1422	**ENSG0000007868**	TNRC6C	protein_coding	chr17	77958703	78108822	+	Human GRCh	OTTHUMG000001	TNRC6C	**trinucleotide repeat containing adaptor 6C**	Cytoplasm	other		-1.227290564	0	0.37398612	0	1	0	10.9623356	11.2870943	11.1183623	10.6582208
1423	**ENSG0000007869**	CBFA2T2	protein_coding	chr20	33490075	33650036	+	Human GRCh	OTTHUMG000000	CBFA2T2	**CBFA2/RUNX1 partner transcriptional co-repressor 2**	Nucleus	transcription		-1.09399094	0	0.65802995	0	1	0	11.2354653	11.5163157	11.4144008	11.1480743
1424	**ENSG0000007872**	BRINP1	protein_coding	chr9	119153458	119369435	-	Human GRCh	OTTHUMG000000	BRINP1	**BMP/retinoic acid inducible neural specific 1**	Nucleus	peptidase		5.762184138	1	0.47961306	0	1	0	0.73329254	0.80085343	1.61208735	0.7373784
1425	**ENSG0000007874**	ITCH	protein_coding	chr20	34363235	34511773	+	Human GRCh	OTTHUMG000000	ITCH	**itchy E3 ubiquitin protein ligase**	Nucleus	enzyme		-1.091709236	0	0.66083077	0	1	0	11.4754177	11.7831164	11.7355265	11.310596
1427	**ENSG0000007880**	TP53INP2	protein_coding	chr20	34704339	34713439	+	Human GRCh	OTTHUMG000000	TP53INP2	**tumor protein p53 inducible nuclear protein 2**	Nucleus	other		-1.148110693	0	0.63533933	0	1	0	9.09096052	9.78518511	9.25255986	9.41572516
1428	**ENSG0000007880**	SDF4	protein_coding	chr1	1216931	1232031	-	Human GRCh	OTTHUMG000000	SDF4	**stromal cell derived factor 4**	Cytoplasm	other		-1.043929825	0	0.82452771	0	1	0	11.4932946	11.7207904	11.6694458	11.4496092
1429	**ENSG0000007881**	MYH7B	protein_coding	chr20	34955810	35002437	+	Human GRCh	OTTHUMG000000	MYH7B	**myosin heavy chain 7B**	Cytoplasm	other		-1.000202955	0	0.99958867	0	1	0	7.0659277	7.64453831	7.49814486	7.27123523
1431	**ENSG0000007890**	TP73	protein_coding	chr1	3652516	3736201	+	Human GRCh	OTTHUMG000000	TP73	**tumor protein p73**	Nucleus	transcription		-1.260742557	0	0.69396731	0	1	0	7.48885157	7.93791969	7.94763967	6.74649397
1432	**ENSG0000007890**	TOLLIP	protein_coding	chr11	1274371	1309654	-	Human GRCh	OTTHUMG000001	TOLLIP	**toll interacting protein**	Cytoplasm	other		-1.001754544	0	0.99383725	0	1	0	9.89582731	10.2216692	10.2079442	9.90910316
1433	**ENSG0000007896**	UBE2D4	protein_coding	chr7	43926436	43956136	+	Human GRCh	OTTHUMG000001	UBE2D4	**ubiquitin conjugating enzyme E2 D4 (putative)**	Other	enzyme		-1.059428213	0	0.78999216	0	1	0	9.09509395	9.43325468	9.34680229	9.06072808
1434	**ENSG0000007910**	CLUL1	protein_coding	chr18	596988	650334	+	Human GRCh	OTTHUMG000001	CLUL1	**clusterin like 1**	Extracellular	other		3.367085365	1	0.20998396	0	1	0	1.9020794	2.76786345	3.35106084	2.65451471
1435	**ENSG0000007910**	RUNX1T1	protein_coding	chr8	91954972	92103286	-	Human GRCh	OTTHUMG000001	RUNX1T1	**RUNX1 partner transcriptional co-repressor 1**	Nucleus	transcription		-1.479105187	0	0.19455166	0	1	0	7.48963295	7.67824649	7.44847093	6.87483258
1436	**ENSG0000007911**	CDH17	protein_coding	chr8	94127162	94217303	-	Human GRCh	OTTHUMG000001	CDH17	**cadherin 17**	Plasma Mem	transporter	BI 905711	1	0	1	0	NA	0	-0.037722	-0.0302888	-0.0315266	-0.0372897
1437	**ENSG0000007913**	THOC1	protein_coding	chr18	214520	268050	-	Human GRCh	OTTHUMG000001	THOC1	**THO complex subunit 1**	Nucleus	transcription		-1.031879754	0	0.87818759	0	1	0	10.4648703	10.7342557	10.7284162	10.3942554

1438	**ENSG0000007915**	FKBP7	protein_coding	chr2	178463664	178478600	-	Human GRCh	OTTHUMG000001	FKBP7	**FKBP prolyl isomerase 7**	Cytoplasm	enzyme		-1.085043023	0	0.81722734	0	1	0	6.15209719	6.19351845	6.19311155	5.98601971
1439	**ENSG0000007915**	OSBPL6	protein_coding	chr2	178194481	178402893	+	Human GRCh	OTTHUMG000001	OSBPL6	**oxysterol binding protein like 6**	Cytoplasm	transporter		-1.826001766	-1	0.10091411	0	1	0	8.01878638	7.43447045	7.01800989	7.24498352
1440	**ENSG0000007921**	SLC1A3	protein_coding	chr5	36596588	36688334	+	Human GRCh	OTTHUMG000000	SLC1A3	**solute carrier family 1 member 3**	Plasma Mem	transporter	riluzole	-1.104157777	0	0.71935582	0	1	0	7.75880768	8.21341759	7.8343639	7.9415308
1441	**ENSG0000007924**	XRCC5	protein_coding	chr2	216107464	216206303	+	Human GRCh	OTTHUMG000001	XRCC5	**X-ray repair cross complementing 5**	Nucleus	enzyme		-1.173022555	0	0.46949084	0	1	0	13.2273023	13.588909	13.3741962	13.098634
1443	**ENSG0000007926**	SP140	protein_coding	chr2	230203110	230313215	+	Human GRCh	OTTHUMG000001	SP140	**SP140 nuclear body protein**	Nucleus	transcription		1.012599437	0	0.98836436	0	1	0	3.48434567	3.12024801	3.66199496	2.83004098
1444	**ENSG0000007927**	MKNK1	protein_coding	chr1	46557407	46616843	-	Human GRCh	OTTHUMG000000	MKNK1	**MAPK interacting serine/threonine kinase 1**	Cytoplasm	kinase	SEL201, da	1.089028318	0	0.73844553	0	1	0	8.84603235	9.2586571	9.3765858	8.90099787
1445	**ENSG0000007930**	TNS1	protein_coding	chr2	217799588	218033982	-	Human GRCh	OTTHUMG000001	TNS1	**tensin 1**	Plasma Mem	other		-1.320616785	0	0.41727913	0	1	0	9.5720117	9.89863074	9.39298255	9.54250291
1446	**ENSG0000007931**	REXO1	protein_coding	chr19	1815248	1848483	-	Human GRCh	OTTHUMG000001	REXO1	**RNA exonuclease 1 homolog**	Nucleus	transcription		1.035815399	0	0.84628273	0	1	0	11.221633	11.4274682	11.4540388	11.2754539
1447	**ENSG0000007933**	SAR1A	protein_coding	chr10	70147289	70170523	-	Human GRCh	OTTHUMG000000	SAR1A	**secretion associated Ras related GTPase 1A**	Cytoplasm	enzyme		1.00071647	0	0.99718542	0	1	0	11.0829934	11.3750627	11.4166595	11.0292077
1448	**ENSG0000007933**	CDC14A	protein_coding	chr1	100345001	100520277	+	Human GRCh	OTTHUMG000000	CDC14A	**cell division cycle 14A**	Nucleus	phosphatase		1.085271265	0	0.74570023	0	1	0	9.08313634	9.14081603	9.35349381	9.02458562
1449	**ENSG0000007933**	RAPGEF3	protein_coding	chr12	47734363	47771040	-	Human GRCh	OTTHUMG000001	RAPGEF3	**Rap guanine nucleotide exchange factor 3**	Nucleus	other		-1.381344362	0	0.48171429	0	1	0	5.94628617	5.85577396	5.62487762	5.55122014
1450	**ENSG0000007938**	CEACAM1	protein_coding	chr19	42507304	42561234	-	Human GRCh	OTTHUMG000001	CEACAM1	**CEA cell adhesion molecule 1**	Plasma Mem	transporter	anti-CEAC/	1	0	1	0	NA	0	1.36322703	1.39392654	1.38885669	1.36502965
1451	**ENSG0000007938**	SENP1	protein_coding	chr12	48039784	48106079	-	Human GRCh	OTTHUMG000001	SENP1	**SUMO specific peptidase 1**	Nucleus	peptidase		-1.155264043	0	0.5252423	0	1	0	10.2887308	10.6806635	10.4157743	10.2657652
1452	**ENSG0000007939**	DUSP13	protein_coding	chr10	75094432	75109221	-	Human GRCh	OTTHUMG000000	DUSP13B	**dual specificity phosphatase 13B**	Cytoplasm	phosphatase		1.404408997	0	0.7488429	0	1	0	2.15630193	2.2650983	1.66335274	2.76647822
1453	**ENSG0000007943**	CIC	protein_coding	chr19	42268537	42295797	+	Human GRCh	OTTHUMG000001	CIC	**capicua transcriptional repressor**	Nucleus	transcription		1.054230494	0	0.79779483	0	1	0	12.1071889	12.4505538	12.3382444	12.362102
1454	**ENSG0000007943**	LIPE	protein_coding	chr19	42401514	42427388	-	Human GRCh	OTTHUMG000001	LIPE	**lipase E, hormone sensitive type**	Cytoplasm	enzyme		1.07485088	0	0.8053606	0	1	0	7.80939012	8.11140944	8.14699943	7.94640319
1455	**ENSG0000007945**	FDFT1	protein_coding	chr8	11795573	11839395	+	Human GRCh	OTTHUMG000000	FDFT1	**farnesyl-diphosphate farnesyltransferase 1**	Cytoplasm	enzyme	lapaquista	-1.209154912	0	0.49433385	0	1	0	12.4969434	13.1039646	12.6658963	12.5656239
1456	**ENSG0000007946**	PAFAH1B3	protein_coding	chr19	42297033	42303546	-	Human GRCh	OTTHUMG000001	PAFAH1B3	**platelet activating factor acetylhydrolase 1b catalytic subunit 3**	Cytoplasm	enzyme		-1.019578816	0	0.93401229	0	1	0	10.8290126	11.2636635	11.1817634	10.8887574
1457	**ENSG0000007948**	OPHN1	protein_coding	chrX	67949349	68433913	-	Human GRCh	OTTHUMG000000	OPHN1	**oligophrenin 1**	Cytoplasm	other		1.031780802	0	0.93790517	0	1	0	6.63885769	6.89180053	6.74426473	6.86380354
1459	**ENSG0000007961**	KIF22	protein_coding	chr16	29790727	29805385	+	Human GRCh	OTTHUMG000000	KIF22	**kinesin family member 22**	Nucleus	other		-1.15397663	0	0.4476958	0	1	0	11.7623945	12.0263406	11.8710388	11.6097041
1461	**ENSG0000007969**	CARMIL1	protein_coding	chr6	25279078	25620530	+	Human GRCh	OTTHUMG000000	CARMIL1	**capping protein regulator and myosin 1 linker 1**	Cytoplasm	enzyme		-1.24675376	0	0.37910429	0	1	0	11.1293328	11.3563382	11.0528999	10.9451645
1462	**ENSG0000007973**	PGM1	protein_coding	chr1	63593411	63660245	+	Human GRCh	OTTHUMG000000	PGM1	**phosphoglucomutase 1**	Cytoplasm	enzyme		-1.110795114	0	0.66149388	0	1	0	10.0557135	10.2159137	10.1984702	9.81255254
1463	**ENSG0000007978**	DDX1	protein_coding	chr2	15591178	15634346	+	Human GRCh	OTTHUMG000000	DDX1	**DEAD-box helicase 1**	Nucleus	enzyme		-1.136959274	0	0.53358727	0	1	0	11.4741939	11.809866	11.689404	11.2989156
1464	**ENSG0000007980**	DNM2	protein_coding	chr19	10718055	10833488	+	Human GRCh	OTTHUMG000001	DNM2	**dynamin 2**	Plasma Mem	enzyme		-1.039618064	0	0.84778778	0	1	0	12.7173982	13.021555	12.9278105	12.7359145
1465	**ENSG0000007981**	EPB41L2	protein_coding	chr6	130839347	131063322	-	Human GRCh	OTTHUMG000000	EPB41L2	**erythrocyte membrane protein band 4.1 like 2**	Plasma Mem	other		1.008755317	0	0.97585653	0	1	0	9.6463696	10.1223102	9.98885325	9.84171869
1466	**ENSG0000007984**	RIMS1	protein_coding	chr6	71886550	72403150	+	Human GRCh	OTTHUMG000000	RIMS1	**regulating synaptic membrane exocytosis 1**	Plasma Mem	other		1.137804163	0	0.73834498	0	1	0	7.56560365	8.18387193	8.23236915	7.84337334
1467	**ENSG0000007993**	MOXD1	protein_coding	chr6	132296055	132401475	-	Human GRCh	OTTHUMG000000	MOXD1	**monooxygenase DBH like 1**	Cytoplasm	enzyme		-1.009223456	0	0.98900979	0	1	0	4.57323428	4.76760953	4.35268012	4.90614547
1468	**ENSG0000007995**	STX7	protein_coding	chr6	132445867	132513198	-	Human GRCh	OTTHUMG000000	STX7	**syntaxin 7**	Plasma Mem	transporter		-1.007776626	0	0.9742773	0	1	0	9.64105371	9.82781741	9.83799296	9.61019652
1469	**ENSG0000007997**	RABL2B	protein_coding	chr22	50767501	50783667	-	Human GRCh	OTTHUMG000001	RABL2B	**RAB, member of RAS oncogene family like 2B**	Cytoplasm	other		-1.164935678	0	0.47771814	0	1	0	9.41033653	9.70569964	9.57675175	9.19067991
1470	**ENSG0000007999**	KEAP1	protein_coding	chr19	10486125	10503558	-	Human GRCh	OTTHUMG000001	KEAP1	**kelch like ECH associated protein 1**	Cytoplasm	other	dimethyl fı	-1.038503343	0	0.86217683	0	1	0	10.3838294	10.6729985	10.6513911	10.3158947
1472	**ENSG0000008003**	PTPRH	protein_coding	chr19	55181247	55209506	-	Human GRCh	OTTHUMG000001	PTPRH	**protein tyrosine phosphatase receptor type H**	Plasma Mem	phosphatase		1.221501427	0	0.58918487	0	1	0	6.0154449	5.86299443	5.97085098	6.28413081
1473	**ENSG0000008016**	DCT	protein_coding	chr13	94436811	94479682	-	Human GRCh	OTTHUMG000000	DCT	**dopachrome tautomerase**	Cytoplasm	enzyme		1.075356674	0	0.93881689	0	1	0	2.67066493	1.69403684	2.53745248	2.13082676
1474	**ENSG0000008018**	SLC35C2	protein_coding	chr20	46345980	46364458	-	Human GRCh	OTTHUMG000000	SLC35C2	**solute carrier family 35 member C2**	Cytoplasm	transporter		-1.143887621	0	0.50707642	0	1	0	10.6533336	10.8458667	10.7089057	10.4906814
1475	**ENSG0000008020**	CRYBG3	protein_coding	chr3	97822011	97944984	+	Human GRCh	OTTHUMG000001	CRYBG3	**crystallin beta-gamma domain containing 3**	Other	other		1.086682342	0	0.78609807	0	1	0	9.74759394	9.68657852	9.8464743	9.75174092
1476	**ENSG0000008022**	EPHA6	protein_coding	chr3	96814581	97761532	+	Human GRCh	OTTHUMG000001	EPHA6	**EPH receptor A6**	Plasma Mem	kinase	dacomitini	1.013390643	0	0.98108399	0	1	0	6.82265429	6.70727379	6.15327578	7.17619331
1478	**ENSG0000008029**	RFX3	protein_coding	chr9	3218297	3526004	-	Human GRCh	OTTHUMG000000	RFX3	**regulatory factor X3**	Nucleus	transcription		1.034764767	0	0.88912083	0	1	0	9.83833115	9.99110171	10.1179428	9.76372732
1479	**ENSG0000008034**	RIF1	protein_coding	chr2	151409883	151508013	+	Human GRCh	OTTHUMG000001	RIF1	**replication timing regulatory factor 1**	Nucleus	other		-1.06100062	0	0.77473758	0	1	0	11.8684671	12.1615084	12.1119777	11.7888528
1480	**ENSG0000008037**	RAB21	protein_coding	chr12	71754863	71800286	+	Human GRCh	OTTHUMG000001	RAB21	**RAB21, member RAS oncogene family**	Cytoplasm	enzyme		1.052310138	0	0.82193693	0	1	0	11.3156176	11.5047515	11.7274433	11.1409846
1481	**ENSG0000008049**	SLC4A4	protein_coding	chr4	71062667	71572087	+	Human GRCh	OTTHUMG000001	SLC4A4	**solute carrier family 4 member 4**	Plasma Mem	transporter		2.1929612	1	0.0686959	0	1	0	4.27792863	4.51086957	5.27377579	4.73799464
1482	**ENSG0000008050**	SMARCA2	protein_coding	chr9	1980290	2193624	+	Human GRCh	OTTHUMG000000	SMARCA2	**SWI/SNF related, matrix associated, actin dependent regulato**	Nucleus	transcription	FHD-286	-1.194173081	0	0.48634679	0	1	0	11.954816	12.2513127	12.0235331	11.7807597
1484	**ENSG0000008054**	SESN1	protein_coding	chr6	108984309	109094846	-	Human GRCh	OTTHUMG000000	SESN1	**sestrin 1**	Nucleus	other		1.085491208	0	0.73678133	0	1	0	8.33734881	8.46501969	8.6369537	8.32266115
1485	**ENSG0000008056**	MID2	protein_coding	chrX	107825755	107931637	+	Human GRCh	OTTHUMG000000	MID2	**midline 2**	Cytoplasm	enzyme		-1.025738669	0	0.91723413	0	1	0	8.16478226	8.75133193	8.45535304	8.47401122
1488	**ENSG0000008060**	SRCAP	protein_coding	chr16	30698209	30741409	+	Human GRCh	OTTHUMG000001	SRCAP	**Snf2 related CREBBP activator protein**	Cytoplasm	transcription		-1.172415891	0	0.3970215	0	1	0	13.6064949	13.9203117	13.7049845	13.4829111
1489	**ENSG0000008060**	PUM3	protein_coding	chr9	2720469	2844095	-	Human GRCh	OTTHUMG000000	PUM3	**pumilio RNA binding family member 3**	Nucleus	other		1.031303947	0	0.87187587	0	1	0	10.3543966	10.6807703	10.6852825	10.4264071
1491	**ENSG0000008064**	CHRNA3	protein_coding	chr15	78593052	78621295	-	Human GRCh	OTTHUMG000001	CHRNA3	**cholinergic receptor nicotinic alpha 3 subunit**	Plasma Mem	transmembra	succinylch	1.229678942	0	0.73814053	0	1	0	3.20190152	3.36520266	3.33913517	3.4847344
1492	**ENSG0000008070**	KCNN2	protein_coding	chr5	114055926	114496500	+	Human GRCh	OTTHUMG000001	KCNN2	**potassium calcium-activated channel subfamily N member 2**	Plasma Mem	ion channel		-1.087634674	0	0.78472442	0	1	0	8.34965646	8.43560727	8.31597909	8.27361357
1493	**ENSG0000008080**	CNOT4	protein_coding	chr7	135361795	135510145	-	Human GRCh	OTTHUMG000001	CNOT4	**CCR4-NOT transcription complex subunit 4**	Cytoplasm	enzyme		-1.121717741	0	0.59080962	0	1	0	10.0437507	10.226171	10.1074798	9.90679115
1494	**ENSG0000008081**	PSEN1	protein_coding	chr14	73136418	73223691	+	Human GRCh	OTTHUMG000001	PSEN1	**presenilin 1**	Plasma Mem	peptidase	tarenflurbi	-1.111593802	0	0.6145492	0	1	0	11.4752001	11.7153246	11.6228659	11.3299549
1495	**ENSG0000008081**	CPOX	protein_coding	chr3	98579446	98593648	-	Human GRCh	OTTHUMG000001	CPOX	**coproporphyrinogen oxidase**	Cytoplasm	enzyme		1.154654748	0	0.47520293	0	1	0	9.35171042	9.39321462	9.74127186	9.28006036
1496	**ENSG0000008082**	CLDND1	protein_coding	chr3	98497912	98523066	-	Human GRCh	OTTHUMG000001	CLDND1	**claudin domain containing 1**	Plasma Mem	other		-1.178315257	0	0.43081975	0	1	0	10.3049067	10.4190414	10.3047264	10.0473986
1497	**ENSG0000008082**	MOK	protein_coding	chr14	102224500	102305190	-	Human GRCh	OTTHUMG000001	MOK	**MOK protein kinase**	Plasma Mem	kinase		1.055781122	0	0.81021355	0	1	0	9.70623827	9.95914694	10.0431048	9.73438966
1498	**ENSG0000008082**	HSP90AA1	protein_coding	chr14	102080742	102139699	-	Human GRCh	OTTHUMG000001	HSP90AA1	**heat shock protein 90 alpha family class A member 1**	Cytoplasm	enzyme	alvespimy	-1.047484312	0	0.80332539	0	1	0	14.6012904	14.7676061	14.7105793	14.55593
1499	**ENSG0000008083**	RBL1	protein_coding	chr20	36996349	37095997	-	Human GRCh	OTTHUMG000000	RBL1	**RB transcriptional corepressor like 1**	Nucleus	transcription		-1.081970637	0	0.70233477	0	1	0	10.1999885	10.3637399	10.2298196	10.1679959
1500	**ENSG0000008084**	DLGAP4	protein_coding	chr20	36306336	36528637	+	Human GRCh	OTTHUMG000000	DLGAP4	**DLG associated protein 4**	Plasma Mem	other		-1.130002293	0	0.56740999	0	1	0	10.5756253	10.7397462	10.6667798	10.3718602
1501	**ENSG0000008085**	IGSF9B	protein_coding	chr11	133896438	133956968	-	Human GRCh	OTTHUMG000001	IGSF9B	**immunoglobulin superfamily member 9B**	Plasma Mem	other		1.575416508	1	0.04096826	1	1	0	10.3629105	10.2873993	10.9626558	10.6549274
1503	**ENSG0000008098**	NDC80	protein_coding	chr18	2571557	2616635	+	Human GRCh	OTTHUMG000001	NDC80	**NDC80 kinetochore complex component**	Nucleus	other	T 1101	-1.139130301	0	0.58640218	0	1	0	10.0572233	10.4699206	10.2765307	9.98509653
1504	**ENSG0000008101**	AP4E1	protein_coding	chr15	50908672	51005895	+	Human GRCh	OTTHUMG000001	AP4E1	**adaptor related protein complex 4 subunit epsilon 1**	Cytoplasm	other		1.090595339	0	0.70262234	0	1	0	9.78066609	10.2100827	10.2723075	9.92581502
1505	**ENSG0000008101**	RSBN1	protein_coding	chr1	113761832	113812476	-	Human GRCh	OTTHUMG000000	RSBN1	**round spermatid basic protein 1**	Nucleus	enzyme		1.023612053	0	0.91776914	0	1	0	10.3318102	10.5929065	10.7135886	10.2273863
1506	**ENSG0000008102**	MAGI3	protein_coding	chr1	113390515	113685923	+	Human GRCh	OTTHUMG000000	MAGI3	**membrane associated guanylate kinase, WW and PDZ domain**	Cytoplasm	kinase		-1.023870123	0	0.92071891	0	1	0	11.3067643	11.7469759	11.7074579	11.3000288
1507	**ENSG0000008104**	CXCL2	protein_coding	chr4	74097040	74099196	-	Human GRCh	OTTHUMG000001	CXCL2	**C-X-C motif chemokine ligand 2**	Extracellular	cytokine		-1.260973225	0	0.80952081	0	NA	0	3.44130164	4.19540387	3.57834855	3.84266915
1509	**ENSG0000008105**	COL4A4	protein_coding	chr2	227002714	227164453	-	Human GRCh	OTTHUMG000001	COL4A4	**collagen type IV alpha 4 chain**	Extracellular	other	collagenas	1.69896974	1	0.34768096	0	1	0	5.10319769	5.69548202	5.27321317	6.39956429
1510	**ENSG0000008105**	TCF7	protein_coding	chr5	134114681	134151865	+	Human GRCh	OTTHUMG000001	TCF7	**transcription factor 7**	Nucleus	transcription		1.024665468	0	0.93341354	0	1	0	6.73185073	7.2790874	7.11581276	7.00382543
1511	**ENSG0000008108**	OSTM1	protein_coding	chr6	108029245	108165854	-	Human GRCh	OTTHUMG000000	OSTM1	**osteoclastogenesis associated transmembrane protein 1**	Cytoplasm	other		1.359874479	0	0.16402542	0	1	0	8.19486983	8.24069884	8.68232757	8.3745729
1512	**ENSG0000008113**	CDH7	protein_coding	chr18	65750252	65890337	+	Human GRCh	OTTHUMG000001	CDH7	**cadherin 7**	Plasma Mem	other		-1.159036969	0	0.76283089	0	1	0	5.48820721	5.35658094	5.26999466	5.27540928
1513	**ENSG0000008114**	IMPG2	protein_coding	chr3	101222546	101320575	-	Human GRCh	OTTHUMG000001	IMPG2	**interphotoreceptor matrix proteoglycan 2**	Extracellular	other		-1.250860564	0	0.63498719	0	1	0	5.8064937	6.58801518	6.15909044	5.86656952
1514	**ENSG0000008115**	PCNP	protein_coding	chr3	101574180	101594465	+	Human GRCh	OTTHUMG000001	PCNP	**PEST proteolytic signal containing nuclear protein**	Nucleus	other		1.199121956	0	0.32660449	0	1	0	10.7049658	10.9219759	11.176312	10.8290786
1515	**ENSG0000008117**	EXD2	protein_coding	chr14	69191498	69244020	+	Human GRCh	OTTHUMG000001	EXD2	**exonuclease 3'-5' domain containing 2**	Cytoplasm	enzyme		-1.07770011	0	0.71760599	0	1	0	9.42963737	9.61503578	9.60995257	9.25091772
1516	**ENSG0000008118**	ARG2	protein_coding	chr14	67619920	67651708	+	Human GRCh	OTTHUMG000001	ARG2	**arginase 2**	Cytoplasm	enzyme		-1.023955483	0	0.93980002	0	1	0	7.89282909	7.86654305	7.88550665	7.8223251
1517	**ENSG0000008118**	MEF2C	protein_coding	chr5	88717117	88904257	-	Human GRCh	OTTHUMG000001	MEF2C	**myocyte enhancer factor 2C**	Nucleus	transcription		1.088293194	0	0.85996507	0	1	0	5.54721439	5.63251891	5.54793237	5.78314073
1520	**ENSG0000008127**	PKP1	protein_coding	chr1	201283452	201332993	+	Human GRCh	OTTHUMG000000	PKP1	**plakophilin 1**	Plasma Mem	other		1.26392967	0	0.76710043	0	1	0	5.01145204	3.45606459	4.46891425	4.90190543
1521	**ENSG0000008130**	UBA5	protein_coding	chr3	132654446	132679794	+	Human GRCh	OTTHUMG000001	UBA5	**ubiquitin like modifier activating enzyme 5**	Cytoplasm	enzyme		-1.017601361	0	0.93601033	0	1	0	10.5813011	10.9217042	10.9868913	10.4383798
1522	**ENSG0000008132**	STK17B	protein_coding	chr2	196133583	196176503	-	Human GRCh	OTTHUMG000001	STK17B	**serine/threonine kinase 17b**	Nucleus	kinase		-1.060574766	0	0.82980115	0	1	0	10.3290155	10.6309074	10.6149937	10.1938128
1523	**ENSG0000008137**	CDC14B	protein_coding	chr9	96490241	96619843	-	Human GRCh	OTTHUMG000000	CDC14B	**cell division cycle 14B**	Nucleus	phosphatase		-1.130804194	0	0.5823668	0	1	0	9.63957679	9.71852225	9.72070285	9.33372351
1524	**ENSG0000008138**	ZNF510	protein_coding	chr9	96754553	96778129	-	Human GRCh	OTTHUMG000000	ZNF510	**zinc finger protein 510**	Nucleus	transcription		-1.119044148	0	0.60236311	0	1	0	9.98883848	10.306348	10.1557592	9.8979997
1525	**ENSG0000008147**	LRP2	protein_coding	chr2	169127109	169362534	-	Human GRCh	OTTHUMG000001	LRP2	**LDL receptor related protein 2**	Plasma Mem	transporter		-1.758248226	-1	0.14003444	0	1	0	5.50748221	5.97614648	5.09348134	5.36099609
1526	**ENSG0000008166**	ZNF506	protein_coding	chr19	19785839	19821751	-	Human GRCh	OTTHUMG000001	ZNF506	**zinc finger protein 506**	Nucleus	transcription		1.299224737	0	0.40892943	0	1	0	9.20374992	9.61618396	9.99779064	9.32144046
1527	**ENSG0000008169**	JMJD4	protein_coding	chr1	227730425	227735411	-	Human GRCh	OTTHUMG000000	JMJD4	**jumonji domain containing 4**	Cytoplasm	enzyme		-1.090029464	0	0.65700415	0	1	0	9.96843257	10.0684965	10.082372	9.74052433
1528	**ENSG0000008172**	DUSP12	protein_coding	chr1	161749758	161757238	+	Human GRCh	OTTHUMG000000	DUSP12	**dual specificity phosphatase 12**	Nucleus	phosphatase		-1.004737023	0	0.98278342	0	1	0	8.88799453	9.13532713	9.1766801	8.82274521
1529	**ENSG0000008176**	AACS	protein_coding	chr12	125065434	125143333	+	Human GRCh	OTTHUMG000001	AACS	**acetoacetyl-CoA synthetase**	Cytoplasm	enzyme		-1.086868073	0	0.74358118	0	1	0	11.0530171	11.3436943	11.2562399	10.9555495
1530	**ENSG0000008179**	DELE1	protein_coding	chr5	141923855	141942047	+	Human GRCh	OTTHUMG000001	DELE1	**DAP3 binding cell death enhancer 1**	Cytoplasm	other		-1.226998007	0	0.3539443	0	1	0	10.5788107	10.9945583	10.7257851	10.4065633
1532	**ENSG0000008180**	CADPS2	protein_coding	chr7	122318411	122886759	-	Human GRCh	OTTHUMG000001	CADPS2	**calcium dependent secretion activator 2**	Plasma Mem	other		-1.204358432	0	0.52053391	0	1	0	11.1276516	11.413257	11.371165	10.6675089
1533	**ENSG0000008181**	PCDHB4	protein_coding	chr5	141121818	141125623	+	Human GRCh	OTTHUMG000001	PCDHB4	**protocadherin beta 4**	Plasma Mem	other		1.034965968	0	0.91280835	0	1	0	6.87357053	7.12369482	7.18325567	6.87936764
1534	**ENSG0000008184**	PCDHA6	protein_coding	chr5	140827958	141012347	+	Human GRCh	OTTHUMG000001	PCDHA6	**protocadherin alpha 6**	Plasma Mem	other		1.023983702	0	0.94926193	0	1	0	6.57611718	6.79792778	6.88481738	6.51789854

1535	**ENSG0000008185**	PCDHGA2	protein_coding	chr5	141338760	141512975	+	Human GRCh	OTTHUMG000001	PCDHGA2	**protocadherin gamma subfamily A, 2**	Other	other		1.633538443	1	0.1390099	0	1	0	6.0741809	5.80515461	6.63332138	6.22687778
1536	**ENSG0000008187**	IFT25	protein_coding	chr1	53916574	53945699	-	Human GRCh	OTTHUMG000000	IFT25	**intraflagellar transport 25**	Extracellular	other		1.004508378	0	0.98512399	0	1	0	8.76199424	9.07848761	9.09422096	8.75208459
1537	**ENSG0000008191**	PHLPP1	protein_coding	chr18	62715541	62980433	+	Human GRCh	OTTHUMG000001	PHLPP1	**PH domain and leucine rich repeat protein phosphatase 1**	Cytoplasm	enzyme		1.016905795	0	0.94156053	0	1	0	10.2096948	10.5823758	10.4852297	10.3665717
1538	**ENSG0000008192**	ATP8B1	protein_coding	chr18	57646426	57803315	-	Human GRCh	OTTHUMG000001	ATP8B1	**ATPase phospholipid transporting 8B1**	Plasma Mem	transporter		-1.162048036	0	0.47104411	0	1	0	9.47059051	9.63424504	9.38939798	9.39472328
1539	**ENSG0000008198**	IL12RB2	protein_coding	chr1	67307364	67398724	+	Human GRCh	OTTHUMG000000	IL12RB2	**interleukin 12 receptor subunit beta 2**	Plasma Mem	transmembra		1	0	1	0	1	0	1.3757114	1.43346468	1.42408734	1.37916979
1540	**ENSG0000008201**	SMARCD3	protein_coding	chr7	151238764	151277896	-	Human GRCh	OTTHUMG000001	SMARCD3	**SWI/SNF related, matrix associated, actin dependent regulato**	Nucleus	transcription		1.027352695	0	0.9334108	0	1	0	8.8171939	9.20150368	9.17409188	8.92411277
1541	**ENSG0000008206**	WDR70	protein_coding	chr5	37379285	37753435	+	Human GRCh	OTTHUMG000001	WDR70	**WD repeat domain 70**	Nucleus	other		-1.21631122	0	0.34080653	0	1	0	9.81610814	10.0180545	9.82520723	9.58141814
1544	**ENSG0000008214**	STRADB	protein_coding	chr2	201387858	201480846	+	Human GRCh	OTTHUMG000001	STRADB	**STE20 related adaptor beta**	Cytoplasm	kinase		-1.106185343	0	0.6801032	0	1	0	9.17704405	9.37403854	9.31287569	9.00131141
1545	**ENSG0000008215**	BZW1	protein_coding	chr2	200810594	200827338	+	Human GRCh	OTTHUMG000001	BZW1	**basic leucine zipper and W2 domains 1**	Cytoplasm	translation re		-1.024640633	0	0.91127943	0	1	0	11.666336	12.0548816	11.9857663	11.6948294
1547	**ENSG0000008219**	C1QTNF3	protein_coding	chr5	34017858	34043213	-	Human GRCh	OTTHUMG000000	C1QTNF3	**C1q and TNF related 3**	Extracellular	other		-1.015683745	0	0.96325567	0	1	0	8.19373309	8.55264751	8.6766123	7.95274799
1548	**ENSG0000008221**	ME2	protein_coding	chr18	50879080	50954257	+	Human GRCh	OTTHUMG000001	ME2	**malic enzyme 2**	Cytoplasm	enzyme		1.053093025	0	0.81910098	0	1	0	10.7739004	10.9771548	11.0787472	10.7770609
1549	**ENSG0000008221**	C5orf22	protein_coding	chr5	31532287	31555053	+	Human GRCh	OTTHUMG000001	C5orf22	**chromosome 5 open reading frame 22**	Other	other		-1.004853676	0	0.98140216	0	1	0	10.4593443	10.7820276	10.7174791	10.5272379
1550	**ENSG0000008225**	CCNT2	protein_coding	chr2	134918235	134959342	+	Human GRCh	OTTHUMG000001	CCNT2	**cyclin T2**	Nucleus	transcription		-1.046196715	0	0.8354556	0	1	0	10.2586352	10.5081322	10.5280672	10.119246
1551	**ENSG0000008226**	FAM135A	protein_coding	chr6	70412941	70561174	+	Human GRCh	OTTHUMG000000	FAM135A	**family with sequence similarity 135 member A**	Other	enzyme		1.000405767	0	0.99861331	0	1	0	10.1518213	10.2637459	10.4222073	9.94955818
1552	**ENSG0000008229**	COL19A1	protein_coding	chr6	69866556	70212468	+	Human GRCh	OTTHUMG000000	COL19A1	**collagen type XIX alpha 1 chain**	Extracellular	other	collagenas	-3.026375184	-1	0.26317067	0	1	0	3.4458743	3.90222736	3.55639515	3.0167832
1553	**ENSG0000008239**	EPB41L3	protein_coding	chr18	5392381	5630700	-	Human GRCh	OTTHUMG000001	EPB41L3	**erythrocyte membrane protein band 4.1 like 3**	Plasma Mem	other		-1.288605933	0	0.74453684	0	1	0	3.59092054	2.38212267	2.9063864	3.14629414
1554	**ENSG0000008243**	COBLL1	protein_coding	chr2	164653624	164843679	-	Human GRCh	OTTHUMG000000	COBLL1	**cordon-bleu WH2 repeat protein like 1**	Extracellular	other		-1.075995898	0	0.74779448	0	1	0	10.7572448	10.9229725	10.8962367	10.6067461
1555	**ENSG0000008245**	DLG3	protein_coding	chrX	70444835	70505490	+	Human GRCh	OTTHUMG000000	DLG3	**discs large MAGUK scaffold protein 3**	Plasma Mem	kinase		-1.177724074	0	0.4927313	0	1	0	10.5178316	10.8459815	10.6495429	10.3692437
1556	**ENSG0000008248**	KCNK2	protein_coding	chr1	215005775	215237090	+	Human GRCh	OTTHUMG000000	KCNK2	**potassium two pore domain channel subfamily K member 2**	Plasma Mem	ion channel		1.244192003	0	0.6072489	0	1	0	6.54896452	7.31675538	7.12066097	7.32777805
1557	**ENSG0000008249**	SERTAD4	protein_coding	chr1	210232796	210246631	+	Human GRCh	OTTHUMG000000	SERTAD4	**SERTA domain containing 4**	Other	other		-3.717662599	-1	0.0295944	1	1	0	5.64669297	6.17583564	4.73827811	4.99211504
1558	**ENSG0000008251**	TRAF5	protein_coding	chr1	211326615	211374946	+	Human GRCh	OTTHUMG000000	TRAF5	**TNF receptor associated factor 5**	Cytoplasm	transporter		1.128297274	0	0.64465411	0	1	0	8.02738158	8.14408447	8.3947703	7.99958319
1559	**ENSG0000008251**	MRPL22	protein_coding	chr5	154941073	154969411	+	Human GRCh	OTTHUMG000001	MRPL22	**mitochondrial ribosomal protein L22**	Cytoplasm	other		-1.074809819	0	0.76287906	0	1	0	9.04844646	9.49583902	9.35853605	9.04705567
1560	**ENSG0000008251**	GEMIN5	protein_coding	chr5	154887411	154938211	-	Human GRCh	OTTHUMG000001	GEMIN5	**gem nuclear organelle associated protein 5**	Nucleus	other		-1.020245485	0	0.92467498	0	1	0	10.9077395	11.3686769	11.2647699	10.9944425
1561	**ENSG0000008255**	OPRK1	protein_coding	chr8	53225724	53251637	-	Human GRCh	OTTHUMG000001	OPRK1	**opioid receptor kappa 1**	Plasma Mem	G-protein co	aspirin/caf	1	0	1	0	1	0	3.31730034	3.40792158	3.39320403	3.32272649
1562	**ENSG0000008264**	NFE2L1	protein_coding	chr17	48048329	48061545	+	Human GRCh	OTTHUMG000001	NFE2L1	**NFE2 like bZIP transcription factor 1**	Nucleus	transcription		-1.119687171	0	0.54301931	0	1	0	12.2891751	12.5130849	12.3314385	12.2250301
1563	**ENSG0000008268**	SEMA5B	protein_coding	chr3	122909082	123028605	-	Human GRCh	OTTHUMG000001	SEMA5B	**semaphorin 5B**	Plasma Mem	other		2.446420809	1	0.31929827	0	1	0	2.70232538	3.3252386	3.30312211	3.37464727
1564	**ENSG0000008270**	GSK3B	protein_coding	chr3	119821321	120094994	-	Human GRCh	OTTHUMG000001	GSK3B	**glycogen synthase kinase 3 beta**	Nucleus	kinase	aloisine A,	-1.093113523	0	0.65403914	0	1	0	12.4106611	12.7090664	12.5687512	12.3656663
1565	**ENSG0000008278**	ITGB5	protein_coding	chr3	124761948	124901418	-	Human GRCh	OTTHUMG000001	ITGB5	**integrin subunit beta 5**	Plasma Mem	other	cilengitide	-1.086755012	0	0.71644964	0	1	0	12.0312522	12.2025083	12.2250924	11.7793295
1566	**ENSG0000008280**	ERC1	protein_coding	chr12	990509	1495933	+	Human GRCh	OTTHUMG000001	ERC1	**ELKS/RAB6-interacting/CAST family member 1**	Cytoplasm	other		-1.106601883	0	0.66786225	0	1	0	10.7155108	11.0581563	10.945037	10.606599
1567	**ENSG0000008289**	XPO1	protein_coding	chr2	61476032	61538741	-	Human GRCh	OTTHUMG000001	XPO1	**exportin 1**	Nucleus	transporter	bortezomi	1.025090679	0	0.89929369	0	1	0	13.5069848	13.7814644	13.8232256	13.5159375
1569	**ENSG0000008299**	RNF13	protein_coding	chr3	149812770	149962139	+	Human GRCh	OTTHUMG000001	RNF13	**ring finger protein 13**	Cytoplasm	enzyme		-1.153680865	0	0.50905593	0	1	0	9.72519027	10.1310848	9.90259033	9.6674112
1570	**ENSG0000008306**	TRPM3	protein_coding	chr9	70529060	71446904	-	Human GRCh	OTTHUMG000000	TRPM3	**transient receptor potential cation channel subfamily M meml**	Plasma Mem	ion channel	TRPM3-inh	19.02204989	1	0.03008129	1	1	0	1.36127652	1.4260492	2.48888334	2.19980493
1571	**ENSG0000008309**	PALB2	protein_coding	chr16	23603160	23641321	-	Human GRCh	OTTHUMG000001	PALB2	**partner and localizer of BRCA2**	Nucleus	other		-1.088963155	0	0.70802409	0	1	0	9.50782939	9.82847098	9.69821872	9.46151803
1572	**ENSG0000008309**	DOP1A	protein_coding	chr6	83067666	83171350	+	Human GRCh	OTTHUMG000000	DOP1A	**DOP1 leucine zipper like protein A**	Cytoplasm	other		-1.148756269	0	0.52504801	0	1	0	10.4387298	10.7645298	10.5430092	10.3725199
1573	**ENSG0000008309**	LYRM2	protein_coding	chr6	89568144	89638749	-	Human GRCh	OTTHUMG000000	LYRM2	**LYR motif containing 2**	Cytoplasm	other		-1.006119094	0	0.97514111	0	1	0	9.71525689	9.93986454	9.98917953	9.6370418
1574	**ENSG0000008312**	BCKDHB	protein_coding	chr6	80106647	80346270	+	Human GRCh	OTTHUMG000000	BCKDHB	**branched chain keto acid dehydrogenase E1 subunit beta**	Cytoplasm	enzyme		1.006135616	0	0.98194031	0	1	0	9.26924197	9.51460957	9.61182982	9.15401855
1575	**ENSG0000008316**	KAT6A	protein_coding	chr8	41929479	42051994	-	Human GRCh	OTTHUMG000001	KAT6A	**lysine acetyltransferase 6A**	Nucleus	enzyme	PF-072481	-1.031997163	0	0.87471006	0	1	0	12.2567785	12.5543834	12.4863182	12.2632051
1576	**ENSG0000008322**	TUT7	protein_coding	chr9	86287733	86354454	-	Human GRCh	OTTHUMG000000	TUT7	**terminal uridylyl transferase 7**	Cytoplasm	enzyme		-1.135119452	0	0.62525106	0	1	0	10.2386904	10.5061935	10.293933	10.1839742
1577	**ENSG0000008329**	ULK2	protein_coding	chr17	19770829	19867936	-	Human GRCh	OTTHUMG000000	ULK2	**unc-51 like autophagy activating kinase 2**	Cytoplasm	kinase	DCC-3116,	-1.169811319	0	0.5180293	0	1	0	9.63377661	9.94733757	9.74098059	9.50570056
1578	**ENSG0000008330**	GRHL2	protein_coding	chr8	101492439	101669726	+	Human GRCh	OTTHUMG000001	GRHL2	**grainyhead like transcription factor 2**	Nucleus	transcription		-1.229208783	0	0.3302926	0	1	0	13.5138333	13.8054797	13.5906347	13.2508653
1579	**ENSG0000008331**	TNPO1	protein_coding	chr5	72816312	72916733	+	Human GRCh	OTTHUMG000001	TNPO1	**transportin 1**	Nucleus	transporter		-1.025666684	0	0.90555541	0	1	0	12.7491823	13.1396195	13.1165402	12.7128395
1580	**ENSG0000008344**	PLOD1	protein_coding	chr1	11934205	11975538	+	Human GRCh	OTTHUMG000000	PLOD1	**procollagen-lysine,2-oxoglutarate 5-dioxygenase 1**	Cytoplasm	enzyme		-1.106134863	0	0.6219392	0	1	0	11.3989777	11.5570134	11.5364801	11.1693732
1581	**ENSG0000008345**	P2RX5	protein_coding	chr17	3672199	3696240	-	Human GRCh	OTTHUMG000000	P2RX5	**purinergic receptor P2X 5**	Plasma Mem	ion channel		-1.380688518	0	0.3431374	0	1	0	6.57312892	7.17471796	6.69105923	6.40556124
1582	**ENSG0000008345**	ITGAE	protein_coding	chr17	3714628	3801188	-	Human GRCh	OTTHUMG000001	ITGAE	**integrin subunit alpha E**	Plasma Mem	other		-1.152819865	0	0.54457989	0	1	0	7.72701886	8.20117959	7.92207348	7.73672543
1583	**ENSG0000008352**	DIS3	protein_coding	chr13	72752169	72782096	-	Human GRCh	OTTHUMG000000	DIS3	**DIS3 homolog, exosome endoribonuclease and 3'-5' exoribonu**	Nucleus	enzyme		-1.103493867	0	0.6251428	0	1	0	10.9776833	11.2623425	11.1405434	10.8829004
1584	**ENSG0000008353**	PIBF1	protein_coding	chr13	72782133	73016461	+	Human GRCh	OTTHUMG000000	PIBF1	**progesterone immunomodulatory binding factor 1**	Nucleus	other		-1.011378543	0	0.95834278	0	1	0	9.32270056	9.57785633	9.59975105	9.2664874
1585	**ENSG0000008354**	TDRD3	protein_coding	chr13	60396457	60573878	+	Human GRCh	OTTHUMG000000	TDRD3	**tudor domain containing 3**	Nucleus	transcription		-1.148455061	0	0.50033644	0	1	0	9.7664613	9.99526727	9.91323824	9.51801821
1587	**ENSG0000008363**	NUFIP1	protein_coding	chr13	44939249	44989471	-	Human GRCh	OTTHUMG000000	NUFIP1	**nuclear FMR1 interacting protein 1**	Nucleus	other		1.141065587	0	0.58091025	0	1	0	8.83667016	9.06868749	9.29864189	8.87267579
1588	**ENSG0000008364**	PDS5B	protein_coding	chr13	32586452	32778019	+	Human GRCh	OTTHUMG000000	PDS5B	**PDS5 cohesin associated factor B**	Nucleus	other		1.021491772	0	0.92668435	0	1	0	10.8736617	11.0953693	11.2242589	10.754653
1589	**ENSG0000008372**	OXCT1	protein_coding	chr5	41730065	41870425	-	Human GRCh	OTTHUMG000000	OXCT1	**3-oxoacid CoA-transferase 1**	Cytoplasm	enzyme		-1.026095842	0	0.91535955	0	1	0	10.9404584	11.4368728	11.3337208	11.0169506
1590	**ENSG0000008375**	RRAGB	protein_coding	chrX	55717749	55758774	+	Human GRCh	OTTHUMG000000	RRAGB	**Ras related GTP binding B**	Cytoplasm	enzyme		-1.060215615	0	0.81135657	0	1	0	8.25146308	8.56190505	8.54739858	8.12327285
1592	**ENSG0000008379**	CYLD	protein_coding	chr16	50742050	50801935	+	Human GRCh	OTTHUMG000001	CYLD	**CYLD lysine 63 deubiquitinase**	Nucleus	transcription		-1.113450904	0	0.64184694	0	1	0	9.60143148	10.0207373	9.87605987	9.51496223
1593	**ENSG0000008380**	SLC27A5	protein_coding	chr19	58479512	58512413	-	Human GRCh	OTTHUMG000001	SLC27A5	**solute carrier family 27 member 5**	Cytoplasm	transporter		1.098912474	0	0.73194082	0	1	0	9.01835003	9.52183672	9.66150106	9.07148848
1594	**ENSG0000008381**	ZNF324	protein_coding	chr19	58467056	58475436	+	Human GRCh	OTTHUMG000001	ZNF324	**zinc finger protein 324**	Nucleus	transcription		1.133778596	0	0.59929254	0	1	0	9.35878454	9.69903608	9.90589102	9.39904766
1595	**ENSG0000008381**	ZNF671	protein_coding	chr19	57719751	57727624	-	Human GRCh	OTTHUMG000001	ZNF671	**zinc finger protein 671**	Nucleus	transcription		-1.677019989	-1	0.64943749	0	1	0	3.75930609	3.47047661	3.22467223	3.21115672
1596	**ENSG0000008381**	ZNF416	protein_coding	chr19	57571566	57578911	-	Human GRCh	OTTHUMG000001	ZNF416	**zinc finger protein 416**	Nucleus	transcription		1.135768342	0	0.60228495	0	1	0	7.30583013	7.63922856	7.65969213	7.57402243
1597	**ENSG0000008382**	ZNF586	protein_coding	chr19	57769655	57819939	+	Human GRCh	OTTHUMG000001	ZNF586	**zinc finger protein 586**	Other	other		-1.095981104	0	0.69276587	0	1	0	10.1776774	10.3583421	10.2959799	10.0244893
1598	**ENSG0000008383**	ZNF446	protein_coding	chr19	58474017	58481230	+	Human GRCh	OTTHUMG000001	ZNF446	**zinc finger protein 446**	Nucleus	transcription		-1.085248402	0	0.7153436	0	1	0	8.53068509	8.62921784	8.32991251	8.63390999
1599	**ENSG0000008384**	ZNF264	protein_coding	chr19	57191500	57222846	+	Human GRCh	OTTHUMG000001	ZNF264	**zinc finger protein 264**	Nucleus	transcription		-1.013801471	0	0.95341914	0	1	0	10.9397613	11.2581229	11.3333046	10.7942599
1600	**ENSG0000008384**	RPS5	protein_coding	chr19	58386400	58394806	+	Human GRCh	OTTHUMG000001	RPS5	**ribosomal protein S5**	Cytoplasm	other		-1.02798055	0	0.89293556	0	1	0	13.2343594	13.4904264	13.4238506	13.2451777
1601	**ENSG0000008385**	FAT1	protein_coding	chr4	186587794	186726722	-	Human GRCh	OTTHUMG000001	FAT1	**FAT atypical cadherin 1**	Plasma Mem	other		1.189362548	0	0.55865671	0	1	0	12.3915907	12.5564191	12.7937318	12.5609606
1602	**ENSG0000008389**	YTHDC1	protein_coding	chr4	68310387	68350090	-	Human GRCh	OTTHUMG000001	YTHDC1	**YTH N6-methyladenosine RNA binding protein C1**	Cytoplasm	other		-1.217879735	0	0.27581173	0	1	0	11.4344402	11.7229948	11.4466233	11.2822496
1603	**ENSG0000008393**	CHMP2B	protein_coding	chr3	87227271	87255556	+	Human GRCh	OTTHUMG000001	CHMP2B	**charged multivesicular body protein 2B**	Cytoplasm	other		1.148706393	0	0.50290553	0	1	0	10.073752	10.2855161	10.4296522	10.2381291
1604	**ENSG0000008407**	SMAP2	protein_coding	chr1	40344850	40423326	+	Human GRCh	OTTHUMG000000	SMAP2	**small ArfGAP2**	Cytoplasm	other		-1.002855733	0	0.99043443	0	1	0	9.72443218	10.0177376	9.95154831	9.79715087
1605	**ENSG0000008407**	PPIE	protein_coding	chr1	39692182	39763914	+	Human GRCh	OTTHUMG000000	PPIE	**peptidylprolyl isomerase E**	Nucleus	enzyme		-1.027899874	0	0.89059429	0	1	0	9.88160069	10.1039725	10.1199899	9.79378663
1606	**ENSG0000008407**	ZMPSTE24	protein_coding	chr1	40258041	40294180	+	Human GRCh	OTTHUMG000000	ZMPSTE24	**zinc metallopeptidase STE24**	Nucleus	peptidase		1.056764536	0	0.80965518	0	1	0	10.8902594	11.1377006	11.2698693	10.8590774
1607	**ENSG0000008409**	STARD7	protein_coding	chr2	96184859	96208827	-	Human GRCh	OTTHUMG000001	STARD7	**StAR related lipid transfer domain containing 7**	Cytoplasm	other		-1.091514742	0	0.66755773	0	1	0	12.085732	12.4596247	12.3209007	12.0427127
1608	**ENSG0000008409**	NOA1	protein_coding	chr4	56963350	56977606	-	Human GRCh	OTTHUMG000001	NOA1	**nitric oxide associated 1**	Cytoplasm	other		-1.048109069	0	0.84363301	0	1	0	10.3414348	10.738103	10.6423906	10.3450818
1609	**ENSG0000008409**	REST	protein_coding	chr4	56907876	56966808	+	Human GRCh	OTTHUMG000001	REST	**RE1 silencing transcription factor**	Nucleus	transcription		-1.089963122	0	0.67600537	0	1	0	11.3599116	11.6176874	11.5757928	11.1874988
1610	**ENSG0000008411**	HAL	protein_coding	chr12	95972662	95996365	-	Human GRCh	OTTHUMG000001	HAL	**histidine ammonia-lyase**	Cytoplasm	enzyme		1.998393056	1	0.26161274	0	1	0	2.90450117	3.89838999	4.09898132	3.89497286
1611	**ENSG0000008411**	SSH1	protein_coding	chr12	108778191	108857590	-	Human GRCh	OTTHUMG000001	SSH1	**slingshot protein phosphatase 1**	Cytoplasm	phosphatase		-1.175115527	0	0.38590389	0	1	0	10.4574061	10.8034251	10.6064779	10.3009801
1612	**ENSG0000008420**	GSTP1	protein_coding	chr11	67583742	67586656	+	Human GRCh	OTTHUMG000001	GSTP1	**glutathione S-transferase pi 1**	Cytoplasm	enzyme	zeaxanthir	1	0	1	0	1	0	1.45868161	1.59075811	1.56982772	1.4668179
1613	**ENSG0000008423**	APLP2	protein_coding	chr11	130068147	130144811	+	Human GRCh	OTTHUMG000001	APLP2	**amyloid beta precursor like protein 2**	Cytoplasm	other		-1.042130348	0	0.87894512	0	1	0	14.3858999	14.6683255	14.6936844	14.239971
1614	**ENSG0000008444**	FAM234B	protein_coding	chr12	13044381	13142521	+	Human GRCh	OTTHUMG000001	FAM234B	**family with sequence similarity 234 member B**	Other	other		-1.144218319	0	0.5816265	0	1	0	8.95674198	9.26737406	9.10769272	8.82640735
1615	**ENSG0000008445**	SLCO1A2	protein_coding	chr12	21264600	21419594	-	Human GRCh	OTTHUMG000001	SLCO1A2	**solute carrier organic anion transporter family member 1A2**	Plasma Mem	transporter		-1.094794321	0	0.92920219	0	1	0	3.6590322	4.08136133	3.33849367	4.1691061
1616	**ENSG0000008446**	WBP11	protein_coding	chr12	14784582	14803486	-	Human GRCh	OTTHUMG000001	WBP11	**WW domain binding protein 11**	Nucleus	phosphatase		-1.150242726	0	0.43426013	0	1	0	11.4161268	11.7639923	11.5110732	11.3755393
1617	**ENSG0000008462**	EIF3I	protein_coding	chr1	32221077	32245397	+	Human GRCh	OTTHUMG000000	EIF3I	**eukaryotic translation initiation factor 3 subunit I**	Cytoplasm	translation re		-1.11890516	0	0.5670533	0	1	0	11.7606247	12.0568036	11.8850771	11.6876996
1618	**ENSG0000008462**	NKAIN1	protein_coding	chr1	31179745	31239887	-	Human GRCh	OTTHUMG000000	NKAIN1	**sodium/potassium transporting ATPase interacting 1**	Plasma Mem	other		-2.131094378	-1	0.13490224	0	1	0	4.8271405	5.05527106	3.90748588	4.59099828
1619	**ENSG0000008463**	COL16A1	protein_coding	chr1	31652263	31704319	-	Human GRCh	OTTHUMG000000	COL16A1	**collagen type XVI alpha 1 chain**	Extracellular	other	collagenas	-1.047410182	0	0.95375939	0	1	0	3.33950777	3.03834042	2.16881835	3.65162172
1620	**ENSG0000008465**	TXLNA	protein_coding	chr1	32179675	32198285	+	Human GRCh	OTTHUMG000000	TXLNA	**taxilin alpha**	Extracellular	cytokine		1.039273337	0	0.84766485	0	1	0	11.6128336	11.8833103	11.9897189	11.5705143
1622	**ENSG0000008467**	NCOA1	protein_coding	chr2	24491254	24770702	+	Human GRCh	OTTHUMG000001	NCOA1	**nuclear receptor coactivator 1**	Nucleus	transcription		-1.038195009	0	0.86054534	0	1	0	10.5406052	10.8155876	10.6247486	10.6648301
1623	**ENSG0000008469**	AGBL5	protein_coding	chr2	27042364	27070622	+	Human GRCh	OTTHUMG000001	AGBL5	**AGBL carboxypeptidase 5**	Cytoplasm	peptidase		-1.042147362	0	0.8520676	0	1	0	10.6300064	10.9065784	10.8411277	10.6080528
1624	**ENSG0000008471**	EFR3B	protein_coding	chr2	25042076	25159135	+	Human GRCh	OTTHUMG000001	EFR3B	**EFR3 homolog B**	Plasma Mem	other		-1.384998493	0	0.36460418	0	1	0	5.88006251	6.50198123	6.22483509	5.54874336

1625	**ENSG0000008473**	KIF3C	protein_coding	chr2	25926598	25982749	-	Human GRCh	OTTHUMG000000	KIF3C	**kinesin family member 3C**	Cytoplasm	other		-1.170340774	0	0.51145972	0	1	0	8.54038674	8.6721476	8.61660372	8.22688182
1626	**ENSG0000008473**	RAB10	protein_coding	chr2	26034084	26137454	+	Human GRCh	OTTHUMG000000	RAB10	**RAB10, member RAS oncogene family**	Cytoplasm	enzyme		1.014224373	0	0.94482628	0	1	0	11.7801461	12.038491	12.0901375	11.7498688
1628	**ENSG0000008475**	HADHA	protein_coding	chr2	26190635	26244672	-	Human GRCh	OTTHUMG000000	HADHA	**hydroxyacyl-CoA dehydrogenase trifunctional multienzyme co**	Cytoplasm	enzyme		-1.134455444	0	0.56543903	0	1	0	11.9685967	12.3792402	12.1836944	11.8939793
1629	**ENSG0000008476**	MAPRE3	protein_coding	chr2	26970637	27027219	+	Human GRCh	OTTHUMG000000	MAPRE3	**microtubule associated protein RP/EB family member 3**	Cytoplasm	enzyme		1.331873443	0	0.22733857	0	1	0	7.71607372	7.43265972	7.85577957	7.93634618
1630	**ENSG0000008477**	CAD	protein_coding	chr2	27217369	27243943	+	Human GRCh	OTTHUMG000000	CAD	**carbamoyl-phosphate synthetase 2, aspartate transcarbamyla**	Cytoplasm	enzyme		-1.074440183	0	0.7224562	0	1	0	12.4879277	12.8087105	12.7816241	12.331643
1631	**ENSG0000008506**	CD59	protein_coding	chr11	33703010	33736479	-	Human GRCh	OTTHUMG000001	CD59	**CD59 molecule (CD59 blood group)**	Plasma Mem	other		1.026091948	0	0.91006517	0	1	0	10.1769374	10.3020289	10.4322916	10.080219
1632	**ENSG0000008511**	CD82	protein_coding	chr11	44564427	44620358	+	Human GRCh	OTTHUMG000001	CD82	**CD82 molecule**	Plasma Mem	other		1.245030327	0	0.38363883	0	1	0	7.36036058	7.41521646	7.66460121	7.5356587
1633	**ENSG0000008518**	BCORL1	protein_coding	chrX	129981107	130058071	+	Human GRCh	OTTHUMG000000	BCORL1	**BCL6 corepressor like 1**	Nucleus	transcription		1.064672908	0	0.7831702	0	1	0	10.087943	10.4582252	10.4569436	10.2445695
1634	**ENSG0000008522**	ATRX	protein_coding	chrX	77504880	77786233	-	Human GRCh	OTTHUMG000000	ATRX	**ATRX chromatin remodeler**	Nucleus	transcription		-1.075640557	0	0.7234858	0	1	0	12.1361759	12.393794	12.3779394	11.9781534
1635	**ENSG0000008523**	AK6	protein_coding	chr5	69350984	69370013	-	Human GRCh	OTTHUMG000001	AK6	**adenylate kinase 6**	Nucleus	kinase		1.049306252	0	0.83859456	0	1	0	7.51721816	7.71400811	7.82759651	7.49207066
1637	**ENSG0000008527**	MYNN	protein_coding	chr3	169773396	169789716	+	Human GRCh	OTTHUMG000001	MYNN	**myoneurin**	Nucleus	transcription		1.056015053	0	0.81925844	0	1	0	9.15225503	9.43797649	9.61893121	9.03425225
1638	**ENSG0000008527**	MECOM	protein_coding	chr3	169083499	169663775	-	Human GRCh	OTTHUMG000001	MECOM	**MDS1 and EVI1 complex locus**	Nucleus	transcription		-1.432445484	0	0.29809399	0	1	0	6.83349426	7.0035193	6.5189513	6.61940007
1639	**ENSG0000008536**	SCAMP1	protein_coding	chr5	78360611	78480739	+	Human GRCh	OTTHUMG000001	SCAMP1	**secretory carrier membrane protein 1**	Cytoplasm	transporter		1.122189923	0	0.61497297	0	1	0	10.8520706	10.9534591	11.2254455	10.7974762
1640	**ENSG0000008537**	PREP	protein_coding	chr6	105273218	105454062	-	Human GRCh	OTTHUMG000000	PREP	**prolyl endopeptidase**	Cytoplasm	peptidase	Z 321	-1.072110043	0	0.74986735	0	1	0	10.6619362	10.9926517	10.7800845	10.7396353
1641	**ENSG0000008538**	HACE1	protein_coding	chr6	104728094	104859919	-	Human GRCh	OTTHUMG000000	HACE1	**HECT domain and ankyrin repeat containing E3 ubiquitin prote**	Cytoplasm	enzyme		-1.036565599	0	0.87619226	0	1	0	9.77678508	9.93547691	10.0041319	9.6025295
1642	**ENSG0000008541**	SEH1L	protein_coding	chr18	12947133	12987536	+	Human GRCh	OTTHUMG000001	SEH1L	**SEH1 like nucleoporin**	Cytoplasm	transporter		1.092197791	0	0.65730099	0	1	0	10.6995099	10.9613081	11.0755656	10.7728206
1643	**ENSG0000008543**	WDR47	protein_coding	chr1	108970214	109042113	-	Human GRCh	OTTHUMG000000	WDR47	**WD repeat domain 47**	Cytoplasm	other		-1.072670747	0	0.76129068	0	1	0	8.68759668	9.06525221	8.98695428	8.61297386
1644	**ENSG0000008544**	WDFY1	protein_coding	chr2	223855716	223945357	-	Human GRCh	OTTHUMG000001	WDFY1	**WD repeat and FYVE domain containing 1**	Cytoplasm	other		-1.082268086	0	0.70972212	0	1	0	10.1047343	10.3473596	10.3182984	9.94174306
1645	**ENSG0000008546**	OVGP1	protein_coding	chr1	111414319	111427735	-	Human GRCh	OTTHUMG000000	OVGP1	**oviductal glycoprotein 1**	Extracellular	other		-1.3751581	0	0.37925144	0	1	0	7.01861271	7.288513	7.14608776	6.37695479
1646	**ENSG0000008549**	SLC25A24	protein_coding	chr1	108134043	108200849	-	Human GRCh	OTTHUMG000000	SLC25A24	**solute carrier family 25 member 24**	Cytoplasm	transporter		-1.392723137	0	0.09058572	0	1	0	9.34758435	9.47815917	9.18064849	8.93645433
1647	**ENSG0000008551**	MAP3K4	protein_coding	chr6	160991727	161117385	+	Human GRCh	OTTHUMG000000	MAP3K4	**mitogen-activated protein kinase kinase kinase 4**	Cytoplasm	kinase		-1.078854601	0	0.69850282	0	1	0	10.8202146	11.0985488	10.9441488	10.8204536
1648	**ENSG0000008551**	PILRA	protein_coding	chr7	100367530	100400096	+	Human GRCh	OTTHUMG000001	PILRA	**paired immunoglobin like type 2 receptor alpha**	Plasma Mem	other		-1.573459051	-1	0.13694289	0	1	0	6.89405396	7.3632633	6.70501684	6.63042574
1649	**ENSG0000008555**	IGSF9	protein_coding	chr1	159927039	159945613	-	Human GRCh	OTTHUMG000000	IGSF9	**immunoglobulin superfamily member 9**	Plasma Mem	other		-1.074522663	0	0.79715749	0	1	0	9.83719686	9.85108402	10.0170138	9.44787358
1651	**ENSG0000008564**	ZNF213	protein_coding	chr16	3129777	3142804	+	Human GRCh	OTTHUMG000001	ZNF213	**zinc finger protein 213**	Nucleus	transcription		1.099028061	0	0.63083591	0	1	0	9.32322536	9.49890854	9.5558709	9.47728342
1652	**ENSG0000008566**	AKR1B1	protein_coding	chr7	134442356	134459284	-	Human GRCh	OTTHUMG000001	AKR1B1	**aldo-keto reductase family 1 member B**	Cytoplasm	enzyme	sorbinil	1.119947224	0	0.90711253	0	1	0	5.73390092	5.77585445	5.92009202	5.84595515
1653	**ENSG0000008571**	CPNE3	protein_coding	chr8	86514435	86561498	+	Human GRCh	OTTHUMG000001	CPNE3	**copine 3**	Cytoplasm	kinase		-1.060411349	0	0.84143266	0	1	0	12.6280159	12.9879888	12.9966906	12.4511116
1654	**ENSG0000008572**	RRN3	protein_coding	chr16	15060033	15094311	-	Human GRCh	OTTHUMG000001	RRN3	**RRN3 homolog, RNA polymerase I transcription factor**	Nucleus	transcription		1.050455123	0	0.81704362	0	1	0	10.4978189	10.8659767	10.9076086	10.5698462
1655	**ENSG0000008573**	CTTN	protein_coding	chr11	70398404	70436584	+	Human GRCh	OTTHUMG000001	CTTN	**cortactin**	Plasma Mem	other		1.043674026	0	0.84202182	0	1	0	12.129134	12.4992695	12.4962838	12.2432157
1656	**ENSG0000008574**	WNT11	protein_coding	chr11	76186325	76210736	-	Human GRCh	OTTHUMG000001	WNT11	**Wnt family member 11**	Extracellular	other		-22.49244608	-1	0.00702657	1	1	0	2.72506749	3.49234692	2.26588533	2.20246644
1657	**ENSG0000008576**	MTIF2	protein_coding	chr2	55236595	55269347	-	Human GRCh	OTTHUMG000001	MTIF2	**mitochondrial translational initiation factor 2**	Cytoplasm	translation re		-1.029734472	0	0.89865422	0	1	0	9.50890643	9.94100236	9.88244069	9.51360416
1658	**ENSG0000008578**	DDHD2	protein_coding	chr8	38225218	38275558	+	Human GRCh	OTTHUMG000001	DDHD2	**DDHD domain containing 2**	Cytoplasm	enzyme		-1.036205672	0	0.87429115	0	1	0	10.9389675	11.3542649	11.2501349	10.9865886
1659	**ENSG0000008583**	TTC39A	protein_coding	chr1	51287258	51345116	-	Human GRCh	OTTHUMG000000	TTC39A	**tetratricopeptide repeat domain 39A**	Cytoplasm	other		1.059400611	0	0.80174048	0	1	0	9.11787508	9.46802239	9.37393169	9.35991988
1660	**ENSG0000008583**	EPS15	protein_coding	chr1	51354263	51519328	-	Human GRCh	OTTHUMG000000	EPS15	**epidermal growth factor receptor pathway substrate 15**	Cytoplasm	other		-1.145013609	0	0.50899234	0	1	0	11.2800781	11.5441432	11.4286694	11.0855201
1661	**ENSG0000008584**	ORC1	protein_coding	chr1	52372829	52404423	-	Human GRCh	OTTHUMG000000	ORC1	**origin recognition complex subunit 1**	Nucleus	other		-1.090627435	0	0.70297373	0	1	0	9.75476418	10.2680288	10.0282318	9.85033559
1662	**ENSG0000008587**	MGST2	protein_coding	chr4	139665768	139740745	+	Human GRCh	OTTHUMG000001	MGST2	**microsomal glutathione S-transferase 2**	Cytoplasm	enzyme		-1.423569409	0	0.25467036	0	1	0	7.91801335	8.0326588	7.61577056	7.57714549
1663	**ENSG0000008587**	CHERP	protein_coding	chr19	16517894	16542437	-	Human GRCh	OTTHUMG000001	CHERP	**calcium homeostasis endoplasmic reticulum protein**	Cytoplasm	other		-1.024120472	0	0.90125433	0	1	0	11.796568	12.1963275	12.0711556	11.8951053
1664	**ENSG0000008597**	ATG16L1	protein_coding	chr2	233210051	233295674	+	Human GRCh	OTTHUMG000001	ATG16L1	**autophagy related 16 like 1**	Cytoplasm	enzyme		-1.021212779	0	0.91976958	0	1	0	9.53895035	9.91151282	9.84202532	9.57603123
1665	**ENSG0000008598**	USP40	protein_coding	chr2	233475526	233566782	-	Human GRCh	OTTHUMG000001	USP40	**ubiquitin specific peptidase 40**	Other	peptidase		-1.143049615	0	0.50558606	0	1	0	10.4383379	10.5402643	10.4745681	10.1968967
1666	**ENSG0000008599**	POMGNT1	protein_coding	chr1	46188683	46220305	-	Human GRCh	OTTHUMG000000	POMGNT1	**protein O-linked mannose N-acetylglucosaminyltransferase 1 (**	Cytoplasm	enzyme		-1.074547333	0	0.71633399	0	1	0	10.667199	10.9305481	10.8722608	10.5599642
1667	**ENSG0000008599**	RAD54L	protein_coding	chr1	46246461	46278480	+	Human GRCh	OTTHUMG000000	RAD54L	**RAD54 like**	Nucleus	enzyme		-1.167161282	0	0.46549006	0	1	0	10.5037806	10.8215177	10.6302925	10.3654235
1668	**ENSG0000008601**	MAST2	protein_coding	chr1	45786987	46036122	+	Human GRCh	OTTHUMG000000	MAST2	**microtubule associated serine/threonine kinase 2**	Cytoplasm	kinase		-1.059985964	0	0.77434886	0	1	0	10.9579515	11.1116384	11.080508	10.8588197
1669	**ENSG0000008606**	DNAJA1	protein_coding	chr9	33025273	33039907	+	Human GRCh	OTTHUMG000000	DNAJA1	**DnaJ heat shock protein family (Hsp40) member A1**	Nucleus	other		1.234000619	0	0.29109145	0	1	0	11.7721828	12.1218119	12.3698788	11.9882361
1670	**ENSG0000008606**	B4GALT1	protein_coding	chr9	33104082	33167356	-	Human GRCh	OTTHUMG000000	B4GALT1	**beta-1,4-galactosyltransferase 1**	Cytoplasm	enzyme		1.078905223	0	0.72823694	0	1	0	11.0444938	11.3752121	11.1572141	11.4369009
1671	**ENSG0000008606**	CHMP5	protein_coding	chr9	33264879	33282070	+	Human GRCh	OTTHUMG000000	CHMP5	**charged multivesicular body protein 5**	Cytoplasm	other		-1.190569347	0	0.43198226	0	1	0	10.2215975	10.5376414	10.3972906	9.95638351
1672	**ENSG0000008610**	NFX1	protein_coding	chr9	33290512	33371157	+	Human GRCh	OTTHUMG000000	NFX1	**nuclear transcription factor, X-box binding 1**	Nucleus	transcription		-1.09604964	0	0.64262846	0	1	0	11.8120957	12.0779237	11.9570491	11.731428
1674	**ENSG0000008618**	DIMT1	protein_coding	chr5	62347284	62403943	-	Human GRCh	OTTHUMG000001	DIMT1	**DIM1 rRNA methyltransferase and ribosome maturation facto**	Cytoplasm	enzyme		-1.021027984	0	0.92967254	0	1	0	9.2167706	9.45125231	9.50088579	9.09802292
1675	**ENSG0000008620**	IPO11	protein_coding	chr5	62403972	62628582	+	Human GRCh	OTTHUMG000001	IPO11	**importin 11**	Nucleus	other		1.007990804	0	0.9709743	0	1	0	10.0468695	10.2735219	10.4433552	9.82495299
1676	**ENSG0000008620**	FOLH1	protein_coding	chr11	49145092	49208638	-	Human GRCh	OTTHUMG000001	FOLH1	**folate hydrolase 1**	Plasma Mem	peptidase	lutetium Lu	-1.471276202	0	0.26268081	0	1	0	8.8380001	9.22856821	8.70182261	8.49081971
1677	**ENSG0000008623**	EIF2AK1	protein_coding	chr7	6022247	6059175	-	Human GRCh	OTTHUMG000000	EIF2AK1	**eukaryotic translation initiation factor 2 alpha kinase 1**	Cytoplasm	kinase		-1.244227266	0	0.28276501	0	1	0	12.2131711	12.4756933	12.241979	11.9617712
1678	**ENSG0000008628**	NME8	protein_coding	chr7	37848597	37900397	+	Human GRCh	OTTHUMG000000	NME8	**NME/NM23 family member 8**	Cytoplasm	kinase		1	0	1	0	NA	0	0.09008601	0.09920801	0.09769021	0.09061695
1679	**ENSG0000008628**	EPDR1	protein_coding	chr7	37920640	37951936	+	Human GRCh	OTTHUMG000001	EPDR1	**ependymin related 1**	Nucleus	other		-1.31509465	0	0.64994764	0	1	0	2.78940707	3.75552998	2.89364641	3.39191887
1680	**ENSG0000008630**	SNX10	protein_coding	chr7	26291862	26374383	+	Human GRCh	OTTHUMG000000	SNX10	**sorting nexin 10**	Cytoplasm	transporter		-1.152339789	0	0.79866729	0	1	0	4.95885676	4.89100178	4.07708074	5.23260672
1681	**ENSG0000008647**	SEPHS1	protein_coding	chr10	13317428	13348298	-	Human GRCh	OTTHUMG000000	SEPHS1	**selenophosphate synthetase 1**	Other	enzyme		-1.105700781	0	0.64371874	0	1	0	10.7822019	11.0469977	10.9778854	10.6278114
1682	**ENSG0000008650**	MRPL28	protein_coding	chr16	366969	371289	-	Human GRCh	OTTHUMG000000	MRPL28	**mitochondrial ribosomal protein L28**	Cytoplasm	other		1.048475813	0	0.85461178	0	1	0	11.0230741	11.2309924	11.3858204	10.9463028
1683	**ENSG0000008650**	HBQ1	protein_coding	chr16	180459	181179	+	Human GRCh	OTTHUMG000000	HBQ1	**hemoglobin subunit theta 1**	Other	other		1.527699625	1	0.44542237	0	1	0	5.5842284	5.50449186	5.79777428	6.14500308
1684	**ENSG0000008654**	ITPKC	protein_coding	chr19	40717112	40740860	+	Human GRCh	OTTHUMG000001	ITPKC	**inositol-trisphosphate 3-kinase C**	Cytoplasm	kinase	GNF362	-1.107280572	0	0.63017157	0	1	0	9.59763031	9.91729257	9.77577226	9.51608815
1685	**ENSG0000008654**	CEACAM6	protein_coding	chr19	41750977	41772211	+	Human GRCh	OTTHUMG000001	CEACAM6	**CEA cell adhesion molecule 6**	Plasma Mem	other	L-DOS47	36.23375812	1	0.00224749	1	0.96	0	4.16728561	4.23214611	4.52005912	5.21517225
1686	**ENSG0000008657**	FAT2	protein_coding	chr5	151504092	151591331	-	Human GRCh	OTTHUMG000001	FAT2	**FAT atypical cadherin 2**	Plasma Mem	other		-5.356115832	-1	0.20762751	0	1	0	2.04609715	1.48605937	1.47455297	1.4190532
1687	**ENSG0000008658**	RBM22	protein_coding	chr5	150690792	150701077	-	Human GRCh	OTTHUMG000001	RBM22	**RNA binding motif protein 22**	Nucleus	other		-1.219603104	0	0.3437319	0	1	0	10.5368303	10.920506	10.6676699	10.3599863
1688	**ENSG0000008659**	TMED2	protein_coding	chr12	123584533	123598582	+	Human GRCh	OTTHUMG000001	TMED2	**transmembrane p24 trafficking protein 2**	Cytoplasm	transporter		-1.027496889	0	0.89454584	0	1	0	12.5362512	12.8308089	12.8400547	12.4528182
1689	**ENSG0000008661**	ERO1B	protein_coding	chr1	236214681	236282019	-	Human GRCh	OTTHUMG000000	ERO1B	**endoplasmic reticulum oxidoreductase 1 beta**	Cytoplasm	enzyme		1.121451312	0	0.678376	0	1	0	10.9235147	11.3088001	11.4135291	11.0757238
1690	**ENSG0000008666**	ZFAND6	protein_coding	chr15	80059568	80138393	+	Human GRCh	OTTHUMG000001	ZFAND6	**zinc finger AN1-type containing 6**	Cytoplasm	other		1.025661997	0	0.90414574	0	1	0	9.89319664	10.0255857	10.0891415	9.87950299
1692	**ENSG0000008671**	TXLNG	protein_coding	chrX	16786432	16844519	+	Human GRCh	OTTHUMG000000	TXLNG	**taxilin gamma**	Nucleus	other		1.02615876	0	0.906566	0	1	0	10.0427766	10.1786799	10.2687099	9.99897725
1694	**ENSG0000008673**	LAT2	protein_coding	chr7	74199652	74229834	+	Human GRCh	OTTHUMG000001	LAT2	**linker for activation of T cells family member 2**	Plasma Mem	other		-1.133638267	0	0.79608142	0	1	0	4.85528931	4.8603939	4.6058156	4.86701839
1695	**ENSG0000008675**	HUWE1	protein_coding	chrX	53532096	53686752	-	Human GRCh	OTTHUMG000000	HUWE1	**HECT, UBA and WWE domain containing E3 ubiquitin protein li**	Nucleus	transcription		-1.206420802	0	0.29603924	0	1	0	14.2822276	14.5510666	14.2915817	14.1389559
1696	**ENSG0000008682**	ZW10	protein_coding	chr11	113733187	113773735	-	Human GRCh	OTTHUMG000001	ZW10	**zw10 kinetochore protein**	Nucleus	other		-1.230355329	0	0.36468428	0	1	0	9.94268228	10.3626371	10.0256568	9.85177355
1697	**ENSG0000008684**	ALG9	protein_coding	chr11	111782195	111871581	-	Human GRCh	OTTHUMG000001	ALG9	**ALG9 alpha-1,2-mannosyltransferase**	Cytoplasm	enzyme		-1.090507289	0	0.70102714	0	1	0	10.087304	10.3894248	10.2548133	10.0381952
1698	**ENSG0000008696**	MYBPC2	protein_coding	chr19	50432892	50466321	+	Human GRCh	OTTHUMG000001	MYBPC2	**myosin binding protein C2**	Cytoplasm	other		-1.143714734	0	0.91939335	0	NA	0	2.13000934	1.89541341	1.72886261	2.13702461
1700	**ENSG0000008700**	ACOX3	protein_coding	chr4	8366282	8440723	-	Human GRCh	OTTHUMG000000	ACOX3	**acyl-CoA oxidase 3, pristanoyl**	Cytoplasm	enzyme		1.015139278	0	0.95720068	0	1	0	8.45461631	8.78870135	8.80676371	8.46877887
1701	**ENSG0000008705**	MTMR2	protein_coding	chr11	95821766	95925315	-	Human GRCh	OTTHUMG000001	MTMR2	**myotubularin related protein 2**	Cytoplasm	phosphatase		-1.19576469	0	0.41464599	0	1	0	10.8996374	11.2047758	11.0768556	10.6020156
1702	**ENSG0000008707**	PPP1R15A	protein_coding	chr19	48872212	48876059	+	Human GRCh	OTTHUMG000001	PPP1R15A	**protein phosphatase 1 regulatory subunit 15A**	Cytoplasm	other	icerguastat	-1.047386995	0	0.79016053	0	1	0	10.7032373	10.8050273	10.7042341	10.7015469
1703	**ENSG0000008707**	HSD17B14	protein_coding	chr19	48813018	48836510	-	Human GRCh	OTTHUMG000001	HSD17B14	**hydroxysteroid 17-beta dehydrogenase 14**	Cytoplasm	enzyme		1.332641032	0	0.42229437	0	1	0	7.10006317	7.46060073	7.64552208	7.55697141
1704	**ENSG0000008707**	TRIP6	protein_coding	chr7	100867387	100873454	+	Human GRCh	OTTHUMG000001	TRIP6	**thyroid hormone receptor interactor 6**	Extracellular	cytokine		1.074189907	0	0.82292416	0	1	0	7.49661107	7.5519995	7.82139561	7.33680786
1705	**ENSG0000008708**	ACHE	protein_coding	chr7	100889994	100896974	-	Human GRCh	OTTHUMG000001	ACHE	**acetylcholinesterase (Cartwright blood group)**	Plasma Mem	enzyme	huperzine	1.434776506	0	0.53023058	0	1	0	6.26626712	5.7457539	6.49020834	6.34133136
1706	**ENSG0000008708**	FTL	protein_coding	chr19	48965309	48966879	+	Human GRCh	OTTHUMG000001	FTL	**ferritin light chain**	Cytoplasm	enzyme		1.128742215	0	0.63660193	0	1	0	12.9013179	13.2505827	13.2288341	13.2235363
1707	**ENSG0000008708**	SRRT	protein_coding	chr7	100875103	100888664	+	Human GRCh	OTTHUMG000001	SRRT	**serrate, RNA effector molecule**	Nucleus	other		-1.068684325	0	0.71764135	0	1	0	12.2166642	12.5778283	12.5016459	12.1447998
1708	**ENSG0000008708**	BAX	protein_coding	chr19	48954815	48961798	+	Human GRCh	OTTHUMG000001	BAX	**BCL2 associated X, apoptosis regulator**	Cytoplasm	transporter		1.163004684	0	0.55041214	0	1	0	8.90596346	9.30613318	9.36550558	9.20190571
1709	**ENSG0000008709**	NLK	protein_coding	chr17	28041737	28196381	+	Human GRCh	OTTHUMG000001	NLK	**nemo like kinase**	Nucleus	kinase		-1.027237138	0	0.89619817	0	1	0	9.59922043	9.82253706	9.80998005	9.55051734
1710	**ENSG0000008711**	PIGS	protein_coding	chr17	28553383	28571794	-	Human GRCh	OTTHUMG000001	PIGS	**phosphatidylinositol glycan anchor biosynthesis class S**	Cytoplasm	enzyme		-1.191624335	0	0.3864144	0	1	0	11.6757411	11.9361395	11.6654301	11.5623168
1711	**ENSG0000008711**	ADAMTS2	protein_coding	chr5	179110853	179345461	-	Human GRCh	OTTHUMG000001	ADAMTS2	**ADAM metallopeptidase with thrombospondin type 1 motif 2**	Extracellular	peptidase		3.075897719	1	0.19991955	0	1	0	3.24636787	2.95687312	3.39741351	4.57876277
1712	**ENSG0000008712**	TMPRSS11	protein_coding	chr4	68447463	68497604	+	Human GRCh	OTTHUMG000001	TMPRSS11E	**transmembrane serine protease 11E**	Extracellular	peptidase		1.295333497	0	0.83780336	0	NA	0	2.93221222	2.55169548	2.35811617	3.11689415
1713	**ENSG0000008715**	ATXN7L3	protein_coding	chr17	44191805	44200113	-	Human GRCh	OTTHUMG000001	ATXN7L3	**ataxin 7 like 3**	Nucleus	transcription		-1.070350071	0	0.73019882	0	1	0	10.9508861	11.1507799	11.1153194	10.82633
1714	**ENSG0000008715**	PGS1	protein_coding	chr17	78378649	78425114	+	Human GRCh	OTTHUMG000001	PGS1	**phosphatidylglycerophosphate synthase 1**	Cytoplasm	enzyme		-1.117497616	0	0.62040334	0	1	0	9.45090302	9.90114891	9.73607891	9.39497784

1715	**ENSG0000008719**	PSMC5	protein_coding	chr17	63827152	63832026	+	Human GRCh	OTTHUMG000001	PSMC5	**proteasome 26S subunit, ATPase 5**	Nucleus	transcription		-1.055864022	0	0.75959522	0	1	0	11.0635507	11.3698225	11.2174558	11.1105937
1716	**ENSG0000008720**	UIMC1	protein_coding	chr5	176905005	177022633	-	Human GRCh	OTTHUMG000001	UIMC1	**ubiquitin interaction motif containing 1**	Nucleus	other		-1.1457679	0	0.52941366	0	1	0	10.0838942	10.2401186	10.1563717	9.85458177
1718	**ENSG0000008724**	MMP2	protein_coding	chr16	55389700	55506691	+	Human GRCh	OTTHUMG000001	MMP2	**matrix metallopeptidase 2**	Extracellular	peptidase	MMP2 MN	-4.964745293	-1	0.76016999	0	NA	0	0.59619848	0.0692571	0.06578745	0.04923669
1719	**ENSG0000008725**	MT3	protein_coding	chr16	56589074	56591088	+	Human GRCh	OTTHUMG000001	MT3	**metallothionein 3**	Cytoplasm	other		-1.459174065	0	0.59315718	0	1	0	4.05636444	4.13085445	3.59240718	3.97384772
1720	**ENSG0000008725**	LPCAT2	protein_coding	chr16	55509072	55586666	+	Human GRCh	OTTHUMG000001	LPCAT2	**lysophosphatidylcholine acyltransferase 2**	Cytoplasm	enzyme		1.290301131	0	0.55199008	0	1	0	5.10036293	5.59795188	5.81408212	5.45256942
1721	**ENSG0000008725**	GNAO1	protein_coding	chr16	56191390	56357444	+	Human GRCh	OTTHUMG000001	GNAO1	**G protein subunit alpha o1**	Plasma Mem	enzyme		1.495566762	0	0.36982057	0	1	0	4.40911285	5.08710995	4.52139268	5.39291666
1722	**ENSG0000008726**	OGFOD1	protein_coding	chr16	56451521	56479104	+	Human GRCh	OTTHUMG000001	OGFOD1	**2-oxoglutarate and iron dependent oxygenase domain contain**	Cytoplasm	enzyme		-1.087998106	0	0.65225803	0	1	0	10.5657934	10.7653933	10.6606558	10.4808511
1723	**ENSG0000008726**	SH3BP2	protein_coding	chr4	2793071	2841098	+	Human GRCh	OTTHUMG000001	SH3BP2	**SH3 domain binding protein 2**	Cytoplasm	other		-1.171238399	0	0.53115922	0	1	0	12.1677085	12.5051906	12.5099478	11.6973519
1724	**ENSG0000008726**	NOP14	protein_coding	chr4	2937933	2963406	-	Human GRCh	OTTHUMG000001	NOP14	**NOP14 nucleolar protein**	Nucleus	other		-1.065683489	0	0.76110303	0	1	0	11.0845133	11.4222937	11.325699	11.0445034
1725	**ENSG0000008727**	ADD1	protein_coding	chr4	2843844	2930076	+	Human GRCh	OTTHUMG000001	ADD1	**adducin 1**	Cytoplasm	other		-1.324624369	0	0.18375066	0	1	0	12.3563435	12.6688565	12.4405702	11.9219433
1726	**ENSG0000008729**	L2HGDH	protein_coding	chr14	50237563	50312229	-	Human GRCh	OTTHUMG000001	L2HGDH	**L-2-hydroxyglutarate dehydrogenase**	Cytoplasm	enzyme		-1.003127133	0	0.98892884	0	1	0	9.71298587	10.0355502	10.090013	9.62865978
1727	**ENSG0000008730**	TXNDC16	protein_coding	chr14	52430596	52552522	-	Human GRCh	OTTHUMG000001	TXNDC16	**thioredoxin domain containing 16**	Extracellular	enzyme		-1.081549199	0	0.79689687	0	1	0	9.98213311	10.3726059	10.2121443	9.99798064
1728	**ENSG0000008730**	RTRAF	protein_coding	chr14	51989514	52010694	+	Human GRCh	OTTHUMG000001	RTRAF	**RNA transcription, translation and transport factor**	Nucleus	other		-1.200237666	0	0.34989824	0	1	0	10.9552618	11.308125	11.0590228	10.811663
1729	**ENSG0000008730**	NID2	protein_coding	chr14	52004809	52069059	-	Human GRCh	OTTHUMG000001	NID2	**nidogen 2**	Extracellular	other		-1.21314449	0	0.7364194	0	1	0	4.98583098	5.06871899	5.23893947	4.17205019
1730	**ENSG0000008733**	GMCL1	protein_coding	chr2	69829660	69881384	+	Human GRCh	OTTHUMG000001	GMCL1	**germ cell-less 1, spermatogenesis associated**	Nucleus	other		1.064073619	0	0.80344323	0	1	0	10.4145616	10.5926928	10.8257467	10.2583892
1731	**ENSG0000008736**	SF3B2	protein_coding	chr11	66050729	66069308	+	Human GRCh	OTTHUMG000001	SF3B2	**splicing factor 3b subunit 2**	Nucleus	other		-1.063041504	0	0.77605355	0	1	0	12.3602138	12.6678542	12.5827535	12.313338
1732	**ENSG0000008744**	KLHL42	protein_coding	chr12	27780048	27803040	+	Human GRCh	OTTHUMG000001	KLHL42	**kelch like family member 42**	Nucleus	enzyme		1.040662133	0	0.84638421	0	1	0	10.399646	10.6963882	10.7313856	10.4542815
1733	**ENSG0000008746**	GNAS	protein_coding	chr20	58839718	58911192	+	Human GRCh	OTTHUMG000000	GNAS	**GNAS complex locus**	Plasma Mem	enzyme		-1.135038295	0	0.53310502	0	1	0	13.7256184	14.0347179	13.8666192	13.6246503
1734	**ENSG0000008747**	DNM1L	protein_coding	chr12	32679200	32745650	+	Human GRCh	OTTHUMG000001	DNM1L	**dynamin 1 like**	Cytoplasm	enzyme		-1.259799629	0	0.25744476	0	1	0	12.4322956	12.6534896	12.5196314	11.9904258
1735	**ENSG0000008749**	PTHLH	protein_coding	chr12	27958084	27972733	-	Human GRCh	OTTHUMG000001	PTHLH	**parathyroid hormone like hormone**	Extracellular	other	monoclona	2.616521229	1	0.10161428	0	1	0	3.8251852	3.14815933	4.30209523	4.16657804
1736	**ENSG0000008749**	PHACTR3	protein_coding	chr20	59577509	59847711	+	Human GRCh	OTTHUMG000000	PHACTR3	**phosphatase and actin regulator 3**	Nucleus	other		44.91072981	1	0.01580495	1	1	0	2.38436704	2.44875886	2.43824321	4.23561707
1737	**ENSG0000008750**	ERGIC2	protein_coding	chr12	29337352	29381189	-	Human GRCh	OTTHUMG000001	ERGIC2	**ERGIC and golgi 2**	Cytoplasm	other		-1.114549595	0	0.59238071	0	1	0	10.4044348	10.6329402	10.5048612	10.29527
1738	**ENSG0000008751**	TFAP2C	protein_coding	chr20	56629306	56639283	+	Human GRCh	OTTHUMG000000	TFAP2C	**transcription factor AP-2 gamma**	Nucleus	transcription		-1.005088506	0	0.98818147	0	1	0	7.85085503	8.32019159	8.04684906	8.15665357
1739	**ENSG0000008758**	AURKA	protein_coding	chr20	56369389	56392337	-	Human GRCh	OTTHUMG000000	AURKA	**aurora kinase A**	Nucleus	kinase	aurora A ir	-1.131491502	0	0.630338	0	1	0	10.5046877	10.8372553	10.7130182	10.3508709
1740	**ENSG0000008758**	CASS4	protein_coding	chr20	56412112	56460387	+	Human GRCh	OTTHUMG000000	CASS4	**Cas scaffold protein family member 4**	Plasma Mem	other		-2.1123846	-1	0.46365103	0	NA	0	3.18218239	2.56602242	2.54965439	2.47166303
1741	**ENSG0000008784**	PIR	protein_coding	chrX	15384799	15493564	-	Human GRCh	OTTHUMG000000	PIR	**pirin**	Nucleus	transcription		-1.238382901	0	0.43779462	0	1	0	6.29658332	6.50603502	5.83323312	6.45860006
1742	**ENSG0000008788**	AAMDC	protein_coding	chr11	77821109	77918432	+	Human GRCh	OTTHUMG000001	AAMDC	**adipogenesis associated Mth938 domain containing**	Cytoplasm	other		1.163967668	0	0.62527372	0	1	0	7.1094147	7.59905253	7.64320522	7.43216363
1743	**ENSG0000008790**	RFX2	protein_coding	chr19	5993164	6199572	-	Human GRCh	OTTHUMG000001	RFX2	**regulatory factor X2**	Nucleus	transcription		1.108807388	0	0.66252143	0	1	0	9.03392823	9.31707533	9.43026499	9.14218067
1744	**ENSG0000008799**	METTL2A	protein_coding	chr17	62423875	62453385	+	Human GRCh	OTTHUMG000001	METTL2A	**methyltransferase 2A, tRNA N3-cytidine**	Cytoplasm	enzyme		-1.126444274	0	0.55796695	0	1	0	9.85704184	10.0944589	10.0454484	9.61377522
1745	**ENSG0000008800**	SULT2B1	protein_coding	chr19	48552172	48599425	+	Human GRCh	OTTHUMG000001	SULT2B1	**sulfotransferase family 2B member 1**	Cytoplasm	enzyme		1.00329158	0	0.9948631	0	1	0	4.70629866	4.9984626	4.93377975	4.78902789
1746	**ENSG0000008803**	ALG6	protein_coding	chr1	63367575	63438553	+	Human GRCh	OTTHUMG000000	ALG6	**ALG6 alpha-1,3-glucosyltransferase**	Cytoplasm	enzyme		1.158517549	0	0.55912462	0	1	0	8.72844328	8.80009443	9.08229477	8.73214785
1747	**ENSG0000008803**	CNOT3	protein_coding	chr19	54137749	54155681	+	Human GRCh	OTTHUMG000000	CNOT3	**CCR4-NOT transcription complex subunit 3**	Cytoplasm	other		-1.049857179	0	0.78357638	0	1	0	11.7032545	11.981675	11.8418788	11.7479722
1748	**ENSG0000008805**	GP6	protein_coding	chr19	55013705	55038264	-	Human GRCh	OTTHUMG000001	GP6	**glycoprotein VI platelet**	Plasma Mem	transmembra		-1.096579357	0	0.88721714	0	1	0	5.09139208	4.81724876	4.76378578	4.9664952
1749	**ENSG0000008817**	PTPN4	protein_coding	chr2	119759922	119984899	+	Human GRCh	OTTHUMG000001	PTPN4	**protein tyrosine phosphatase non-receptor type 4**	Cytoplasm	phosphatase		-1.041083847	0	0.87736108	0	1	0	9.86737603	10.3114283	10.4142526	9.56908964
1750	**ENSG0000008820**	DDX18	protein_coding	chr2	117814691	117832377	+	Human GRCh	OTTHUMG000000	DDX18	**DEAD-box helicase 18**	Nucleus	enzyme		-1.100167895	0	0.64902092	0	1	0	10.3359366	10.7607445	10.5815178	10.3295251
1751	**ENSG0000008824**	KHSRP	protein_coding	chr19	6413102	6424811	-	Human GRCh	OTTHUMG000001	KHSRP	**KH-type splicing regulatory protein**	Nucleus	enzyme		1.015851907	0	0.93734289	0	1	0	13.6290155	13.8573264	13.8525392	13.6732644
1752	**ENSG0000008825**	GNA11	protein_coding	chr19	3094362	3123999	+	Human GRCh	OTTHUMG000001	GNA11	**G protein subunit alpha 11**	Plasma Mem	enzyme		1.017483403	0	0.93136392	0	1	0	11.4700095	11.7193313	11.6958934	11.5401133
1753	**ENSG0000008828**	ASAP3	protein_coding	chr1	23428563	23484568	-	Human GRCh	OTTHUMG000000	ASAP3	**ArfGAP with SH3 domain, ankyrin repeat and PH domain 3**	Plasma Mem	other		1.002449983	0	0.99390087	0	1	0	7.46186066	7.50388739	7.4837122	7.48715656
1754	**ENSG0000008829**	EDEM2	protein_coding	chr20	35115364	35147336	-	Human GRCh	OTTHUMG000000	EDEM2	**ER degradation enhancing alpha-mannosidase like protein 2**	Cytoplasm	enzyme		-1.059169003	0	0.79191816	0	1	0	8.77221452	8.99860754	8.94039215	8.70314257
1755	**ENSG0000008830**	DNMT3B	protein_coding	chr20	32762385	32809359	+	Human GRCh	OTTHUMG000000	DNMT3B	**DNA methyltransferase 3 beta**	Nucleus	enzyme		-1.184493482	0	0.49431016	0	1	0	9.02711972	9.39838304	9.05521126	9.03883878
1757	**ENSG0000008832**	TPX2	protein_coding	chr20	31739271	31801805	+	Human GRCh	OTTHUMG000000	TPX2	**TPX2 microtubule nucleation factor**	Nucleus	other		-1.21377514	0	0.38184312	0	1	0	12.4868892	12.8936265	12.5939777	12.3782413
1758	**ENSG0000008835**	PDRG1	protein_coding	chr20	31944337	31952046	-	Human GRCh	OTTHUMG000000	PDRG1	**p53 and DNA damage regulated 1**	Cytoplasm	other		-1.004505287	0	0.98346877	0	1	0	8.35760119	8.63078703	8.5832689	8.40379699
1759	**ENSG0000008836**	EPB41L1	protein_coding	chr20	36091504	36232799	+	Human GRCh	OTTHUMG000000	EPB41L1	**erythrocyte membrane protein band 4.1 like 1**	Plasma Mem	other		1.088193168	0	0.66505814	0	1	0	9.65555125	9.84800912	9.78094581	9.91037043
1760	**ENSG0000008838**	SLC15A1	protein_coding	chr13	98683801	98752672	-	Human GRCh	OTTHUMG000000	SLC15A1	**solute carrier family 15 member 1**	Plasma Mem	transporter		1.610351264	1	0.34979788	0	1	0	4.43539182	4.82374369	4.74544402	5.29724636
1761	**ENSG0000008838**	DOCK9	protein_coding	chr13	98793429	99086625	-	Human GRCh	OTTHUMG000000	DOCK9	**dedicator of cytokinesis 9**	Cytoplasm	other		-1.036414673	0	0.86794478	0	1	0	10.267886	10.3970375	10.368849	10.2197205
1762	**ENSG0000008844**	ANKRD10	protein_coding	chr13	110878540	110915069	-	Human GRCh	OTTHUMG000000	ANKRD10	**ankyrin repeat domain 10**	Nucleus	transcription		-1.020719096	0	0.92746954	0	1	0	11.3537044	11.5057397	11.5833217	11.2060599
1763	**ENSG0000008845**	TGDS	protein_coding	chr13	94574054	94596242	-	Human GRCh	OTTHUMG000000	TGDS	**TDP-glucose 4,6-dehydratase**	Extracellular	enzyme		1.065172682	0	0.78261313	0	1	0	8.42574165	8.61621277	8.7114934	8.46176349
1764	**ENSG0000008853**	DOCK3	protein_coding	chr3	50674927	51384198	+	Human GRCh	OTTHUMG000001	DOCK3	**dedicator of cytokinesis 3**	Cytoplasm	other		-1.17811535	0	0.47654295	0	1	0	10.7261894	11.0878454	10.900419	10.5489559
1765	**ENSG0000008854**	C3orf18	protein_coding	chr3	50558025	50571027	-	Human GRCh	OTTHUMG000001	C3orf18	**chromosome 3 open reading frame 18**	Other	other		1.430589203	0	0.23147124	0	1	0	6.99277485	7.23227787	7.64383137	7.31884602
1766	**ENSG0000008868**	COQ9	protein_coding	chr16	57447425	57461270	+	Human GRCh	OTTHUMG000001	COQ9	**coenzyme Q9**	Cytoplasm	other		-1.190542116	0	0.3805799	0	1	0	10.9792372	11.2106943	11.0535702	10.7292917
1768	**ENSG0000008872**	KIF9	protein_coding	chr3	47227998	47283451	-	Human GRCh	OTTHUMG000001	KIF9	**kinesin family member 9**	Cytoplasm	enzyme		-1.027004821	0	0.904638	0	1	0	8.33712011	8.58174199	8.52265963	8.34294563
1769	**ENSG0000008875**	ARHGAP28	protein_coding	chr18	6729716	6915716	+	Human GRCh	OTTHUMG000001	ARHGAP28	**Rho GTPase activating protein 28**	Cytoplasm	other		1.451937822	0	0.1765761	0	1	0	7.65717636	7.7544097	8.12606681	8.04032894
1770	**ENSG0000008876**	CRLS1	protein_coding	chr20	6006093	6040053	+	Human GRCh	OTTHUMG000000	CRLS1	**cardiolipin synthase 1**	Cytoplasm	enzyme		1.011195982	0	0.96098528	0	1	0	9.62565869	9.82340393	9.8214694	9.65432696
1772	**ENSG0000008880**	PPP1R13B	protein_coding	chr14	103733195	103847575	-	Human GRCh	OTTHUMG000001	PPP1R13B	**protein phosphatase 1 regulatory subunit 13B**	Cytoplasm	phosphatase		-1.179983991	0	0.42330431	0	1	0	10.220087	10.455113	10.2157155	10.1161583
1773	**ENSG0000008881**	ATRN	protein_coding	chr20	3471018	3651118	+	Human GRCh	OTTHUMG000000	ATRN	**attractin**	Extracellular	other		-1.035320202	0	0.87956299	0	1	0	11.3228721	11.7367752	11.6520773	11.3464986
1774	**ENSG0000008882**	SMOX	protein_coding	chr20	4120980	4187747	+	Human GRCh	OTTHUMG000000	SMOX	**spermine oxidase**	Cytoplasm	enzyme		-1.317216977	0	0.56373912	0	1	0	6.30148175	6.73950028	6.01496258	6.44931294
1775	**ENSG0000008882**	SIGLEC1	protein_coding	chr20	3686970	3712600	-	Human GRCh	OTTHUMG000000	SIGLEC1	**sialic acid binding Ig like lectin 1**	Plasma Mem	other		-2.037698165	-1	0.39594127	0	1	0	2.97039907	3.9805958	2.90169361	3.19650391
1776	**ENSG0000008883**	FKBP1A	protein_coding	chr20	1368977	1393164	-	Human GRCh	OTTHUMG000000	FKBP1A	**FKBP prolyl isomerase 1A**	Cytoplasm	enzyme	cyclospori	-1.070730687	0	0.71629962	0	1	0	12.0368581	12.347745	12.2079098	12.0414682
1777	**ENSG0000008883**	NSFL1C	protein_coding	chr20	1442162	1473842	-	Human GRCh	OTTHUMG000000	NSFL1C	**NSFL1 cofactor**	Cytoplasm	other		-1.147582416	0	0.48462427	0	1	0	10.711298	11.0175492	10.8006743	10.637091
1778	**ENSG0000008883**	SLC4A11	protein_coding	chr20	3227417	3239559	-	Human GRCh	OTTHUMG000000	SLC4A11	**solute carrier family 4 member 11**	Plasma Mem	transporter		1.208351653	0	0.58481371	0	1	0	8.9541035	8.95837336	9.27051747	9.04790455
1779	**ENSG0000008885**	DNAAF9	protein_coding	chr20	3249306	3407669	-	Human GRCh	OTTHUMG000000	DNAAF9	**dynein axonemal assembly factor 9**	Nucleus	other		1.062905541	0	0.90841465	0	1	0	7.25286002	7.53012568	7.57325004	7.34891032
1780	**ENSG0000008887**	ZNF343	protein_coding	chr20	2481817	2524702	-	Human GRCh	OTTHUMG000000	ZNF343	**zinc finger protein 343**	Other	other		1.085497253	0	0.71900989	0	1	0	8.71844667	8.69384274	8.93261935	8.64272899
1781	**ENSG0000008888**	EBF4	protein_coding	chr20	2692780	2760108	+	Human GRCh	OTTHUMG000000	EBF4	**EBF family member 4**	Nucleus	transcription		-1.686642289	-1	0.432828	0	1	0	3.85962622	5.28804362	4.18820714	4.48509334
1782	**ENSG0000008888**	CPXM1	protein_coding	chr20	2794074	2800627	-	Human GRCh	OTTHUMG000000	CPXM1	**carboxypeptidase X, M14 family member 1**	Extracellular	peptidase		1	0	1	0	1	0	1.30127074	1.36659994	1.35604035	1.30520333
1783	**ENSG0000008888**	MAVS	protein_coding	chr20	3846799	3876123	+	Human GRCh	OTTHUMG000000	MAVS	**mitochondrial antiviral signaling protein**	Cytoplasm	other		-1.111266038	0	0.60355477	0	1	0	12.5906513	12.8571578	12.7163298	12.4990405
1784	**ENSG0000008889**	LZTS3	protein_coding	chr20	3162617	3173549	-	Human GRCh	OTTHUMG000000	LZTS3	**leucine zipper tumor suppressor family member 3**	Cytoplasm	other		1.216736471	0	0.47786362	0	1	0	8.14646996	8.50489482	8.68195747	8.37135575
1786	**ENSG0000008893**	XRN2	protein_coding	chr20	21303331	21389825	+	Human GRCh	OTTHUMG000000	XRN2	**5'-3' exoribonuclease 2**	Nucleus	enzyme		-1.117275293	0	0.61555999	0	1	0	11.6893799	12.0977988	11.9337571	11.6200503
1787	**ENSG0000008897**	KIZ	protein_coding	chr20	21125983	21246622	+	Human GRCh	OTTHUMG000000	KIZ	**kizuna centrosomal protein**	Cytoplasm	other		-1.462981724	0	0.13044436	0	1	0	9.03954766	9.17670659	8.8560412	8.50357585
1788	**ENSG0000008898**	DYNLL1	protein_coding	chr12	120469850	120498493	+	Human GRCh	OTTHUMG000001	DYNLL1	**dynein light chain LC8-type 1**	Cytoplasm	other		1.215004108	0	0.44452751	0	1	0	10.9713312	11.287272	11.514152	11.1764868
1789	**ENSG0000008899**	TESC	protein_coding	chr12	117038923	117099479	-	Human GRCh	OTTHUMG000001	TESC	**tescalcin**	Cytoplasm	other		-1.599697653	-1	0.43183262	0	1	0	4.36862916	4.05253021	4.11303856	3.60144094
1790	**ENSG0000008900**	SNX5	protein_coding	chr20	17941597	17968980	-	Human GRCh	OTTHUMG000000	SNX5	**sorting nexin 5**	Cytoplasm	transporter		-1.099236256	0	0.64017754	0	1	0	12.0899668	12.2527172	12.2334227	11.874733
1791	**ENSG0000008900**	RPL6	protein_coding	chr12	112405189	112418838	-	Human GRCh	OTTHUMG000001	RPL6	**ribosomal protein L6**	Nucleus	other		-1.028085118	0	0.89501761	0	1	0	13.1311502	13.4217509	13.3590575	13.1381666
1793	**ENSG0000008902**	MAPKAPK5	protein_coding	chr12	111842013	111902222	+	Human GRCh	OTTHUMG000001	MAPKAPK5	**MAPK activated protein kinase 5**	Cytoplasm	kinase	GLPG0259	-1.063894778	0	0.78109923	0	1	0	11.1639845	11.545081	11.499707	11.064194
1794	**ENSG0000008904**	P2RX7	protein_coding	chr12	121132819	121188032	+	Human GRCh	OTTHUMG000001	P2RX7	**purinergic receptor P2X 7**	Plasma Mem	ion channel	coomassie	-2.42573932	-1	0.23762599	0	1	0	3.08162495	4.45121885	2.87281811	3.65262286
1795	**ENSG0000008904**	ESF1	protein_coding	chr20	13714322	13784919	-	Human GRCh	OTTHUMG000000	ESF1	**ESF1 nucleolar pre-rRNA processing protein homolog**	Nucleus	other		1.331851667	0	0.22840536	0	1	0	8.52295516	9.00986283	9.26467433	8.91553131
1796	**ENSG0000008905**	RBBP9	protein_coding	chr20	18486540	18497225	-	Human GRCh	OTTHUMG000000	RBBP9	**RB binding protein 9, serine hydrolase**	Nucleus	other		1.010389512	0	0.96595176	0	1	0	9.71143382	9.95825714	9.89975585	9.80337038
1797	**ENSG0000008905**	ANAPC5	protein_coding	chr12	121308245	121399896	-	Human GRCh	OTTHUMG000001	ANAPC5	**anaphase promoting complex subunit 5**	Nucleus	other		-1.146973166	0	0.52376155	0	1	0	12.4186489	12.762838	12.6296947	12.2393911
1798	**ENSG0000008905**	SLC23A2	protein_coding	chr20	4852356	5010293	-	Human GRCh	OTTHUMG000000	SLC23A2	**solute carrier family 23 member 2**	Plasma Mem	transporter		-1.016283641	0	0.9559475	0	1	0	9.09208272	9.22711843	9.26108714	9.0143791
1799	**ENSG0000008906**	SLC8B1	protein_coding	chr12	113298759	113359493	-	Human GRCh	OTTHUMG000001	SLC8B1	**solute carrier family 8 member B1**	Plasma Mem	transporter		1.652578967	1	0.07653303	0	1	0	7.33916743	7.26326155	7.90699199	7.73343019
1800	**ENSG0000008906**	TMEM230	protein_coding	chr20	5068232	5113103	-	Human GRCh	OTTHUMG000000	TMEM230	**transmembrane protein 230**	Cytoplasm	other		-1.160583929	0	0.46639916	0	1	0	10.6360385	10.8868517	10.6744344	10.5306931
1801	**ENSG0000008909**	DZANK1	protein_coding	chr20	18383367	18467281	-	Human GRCh	OTTHUMG000000	DZANK1	**double zinc ribbon and ankyrin repeat domains 1**	Cytoplasm	other		1.037493101	0	0.90045614	0	1	0	7.79439787	7.68788863	8.03047344	7.46555182
1802	**ENSG0000008909**	KDM2B	protein_coding	chr12	121429096	121581023	-	Human GRCh	OTTHUMG000001	KDM2B	**lysine demethylase 2B**	Nucleus	enzyme		-1.047227751	0	0.81537491	0	1	0	10.7473891	11.1257871	11.0388444	10.7456327
1803	**ENSG0000008910**	CFAP61	protein_coding	chr20	20052514	20360703	+	Human GRCh	OTTHUMG000000	CFAP61	**cilia and flagella associated protein 61**	Cytoplasm	other		1.106087841	0	0.81684224	0	1	0	4.52031325	4.64386026	4.9932267	4.20648913

1804	**ENSG0000008911**	LHX5	protein_coding	chr12	113462033	113472280	-	Human GRCh	OTTHUMG000001	LHX5	**LIM homeobox 5**	Nucleus	transcription		1.028130812	0	0.97501812	0	1	0	2.79613558	2.02399657	2.55836545	2.47351547
1805	**ENSG0000008912**	TASP1	protein_coding	chr20	13389392	13638492	-	Human GRCh	OTTHUMG000000	TASP1	**taspase 1**	Nucleus	peptidase		1.036356289	0	0.86873489	0	1	0	9.18437864	9.49486642	9.5501941	9.20183594
1806	**ENSG0000008912**	OAS1	protein_coding	chr12	112905856	112933219	+	Human GRCh	OTTHUMG000001	OAS1	**2'-5'-oligoadenylate synthetase 1**	Cytoplasm	enzyme		-1.005034383	0	0.99633872	0	1	0	2.18984214	1.60449118	1.58826245	2.19890835
1807	**ENSG0000008915**	GCN1	protein_coding	chr12	120127202	120194715	-	Human GRCh	OTTHUMG000001	GCN1	**GCN1 activator of EIF2AK4**	Cytoplasm	translation re		-1.056666522	0	0.76501306	0	1	0	12.9155681	13.2931453	13.2099533	12.8825085
1808	**ENSG0000008915**	RPLP0	protein_coding	chr12	120196699	120201235	-	Human GRCh	OTTHUMG000001	RPLP0	**ribosomal protein lateral stalk subunit P0**	Cytoplasm	other		-1.094860849	0	0.68286286	0	1	0	14.7753733	15.0352781	14.9767202	14.6154231
1809	**ENSG0000008915**	PXN	protein_coding	chr12	120210439	120265771	-	Human GRCh	OTTHUMG000001	PXN	**paxillin**	Cytoplasm	other		1.154187435	0	0.54122236	0	1	0	10.8582948	11.0682284	11.0711842	11.1774165
1810	**ENSG0000008916**	SIRT4	protein_coding	chr12	120302316	120313249	+	Human GRCh	OTTHUMG000001	SIRT4	**sirtuin 4**	Cytoplasm	enzyme		-1.578031861	-1	0.64750795	0	NA	0	2.76426711	3.15055175	2.78872468	2.61766809
1811	**ENSG0000008916**	RPH3A	protein_coding	chr12	112570380	112898881	+	Human GRCh	OTTHUMG000001	RPH3A	**rabphilin 3A**	Plasma Mem	transporter		2.235153444	1	0.51370126	0	1	0	1.18889533	1.84624375	1.2234479	2.23958722
1812	**ENSG0000008917**	KIF16B	protein_coding	chr20	16272104	16573448	-	Human GRCh	OTTHUMG000000	KIF16B	**kinesin family member 16B**	Cytoplasm	enzyme		1.04619684	0	0.83602128	0	1	0	9.14856974	9.43165238	9.48261838	9.19522722
1813	**ENSG0000008919**	TRMT6	protein_coding	chr20	5937228	5950558	-	Human GRCh	OTTHUMG000000	TRMT6	**tRNA methyltransferase 6 non-catalytic subunit**	Nucleus	other		1.02551678	0	0.89167533	0	1	0	9.536664	9.83198221	9.82975208	9.60174497
1814	**ENSG0000008919**	CHGB	protein_coding	chr20	5911510	5925353	+	Human GRCh	OTTHUMG000000	CHGB	**chromogranin B**	Extracellular	other		5.328134786	1	0.18631378	0	1	0	1.90944175	2.01795279	2.0006004	2.54065415
1815	**ENSG0000008922**	PEBP1	protein_coding	chr12	118136124	118145584	+	Human GRCh	OTTHUMG000001	PEBP1	**phosphatidylethanolamine binding protein 1**	Cytoplasm	other		-1.054670019	0	0.81235407	0	1	0	12.3160262	12.7960483	12.6656401	12.3547755
1816	**ENSG0000008922**	TBX5	protein_coding	chr12	114353911	114408442	-	Human GRCh	OTTHUMG000001	TBX5	**T-box transcription factor 5**	Nucleus	transcription		1	0	1	0	1	0	1.86930184	1.96331723	1.94820974	1.87500028
1817	**ENSG0000008923**	BRAP	protein_coding	chr12	111642146	111685956	-	Human GRCh	OTTHUMG000001	BRAP	**BRCA1 associated protein**	Cytoplasm	enzyme		-1.122290805	0	0.58493595	0	1	0	9.6755445	9.94148162	9.79423645	9.57420959
1818	**ENSG0000008924**	ERP29	protein_coding	chr12	112013348	112023449	+	Human GRCh	OTTHUMG000001	ERP29	**endoplasmic reticulum protein 29**	Cytoplasm	transporter		-1.039514184	0	0.82676347	0	1	0	10.5991463	10.7722376	10.7334349	10.5512942
1819	**ENSG0000008925**	NOS1	protein_coding	chr12	117208142	117452170	-	Human GRCh	OTTHUMG000001	NOS1	**nitric oxide synthase 1**	Cytoplasm	enzyme	N(G)-monc	1.370389347	0	0.58716846	0	1	0	7.68638002	7.66416389	8.43634891	7.34208788
1820	**ENSG0000008928**	FUS	protein_coding	chr16	31180138	31191605	+	Human GRCh	OTTHUMG000001	FUS	**FUS RNA binding protein**	Nucleus	transcription		-1.02073931	0	0.91829726	0	1	0	13.0861925	13.3918648	13.3601353	13.0757463
1821	**ENSG0000008928**	IGBP1	protein_coding	chrX	70133447	70166324	+	Human GRCh	OTTHUMG000000	IGBP1	**immunoglobulin binding protein 1**	Cytoplasm	phosphatase		-1.006625224	0	0.97895063	0	1	0	9.82256935	9.93936858	10.0817163	9.62469067
1822	**ENSG0000008932**	FXYD5	protein_coding	chr19	35154730	35169881	+	Human GRCh	OTTHUMG000000	FXYD5	**FXYD domain containing ion transport regulator 5**	Plasma Mem	ion channel		-2.595514702	-1	0.17467453	0	1	0	2.87588606	3.32158352	1.99023192	2.88539866
1823	**ENSG0000008933**	ZNF302	protein_coding	chr19	34677639	34686397	+	Human GRCh	OTTHUMG000001	ZNF302	**zinc finger protein 302**	Nucleus	transcription		1.119293409	0	0.59804846	0	1	0	9.35210495	9.48524692	9.72895965	9.31298096
1824	**ENSG0000008935**	GRAMD1A	protein_coding	chr19	34994784	35026471	+	Human GRCh	OTTHUMG000001	GRAMD1A	**GRAM domain containing 1A**	Cytoplasm	transporter		-1.29703315	0	0.31604487	0	1	0	10.7873762	11.0312657	10.8855721	10.3085433
1825	**ENSG0000008935**	FXYD3	protein_coding	chr19	35115823	35124324	+	Human GRCh	OTTHUMG000001	FXYD3	**FXYD domain containing ion transport regulator 3**	Plasma Mem	ion channel		1.099530607	0	0.87696998	0	1	0	6.45908449	6.69139884	6.53513221	6.7973454
1827	**ENSG0000008948**	CDIP1	protein_coding	chr16	4510669	4538828	-	Human GRCh	OTTHUMG000001	CDIP1	**cell death inducing p53 target 1**	Nucleus	other		-1.02769957	0	0.90212338	0	1	0	9.96839859	10.2060228	10.2220113	9.87833198
1828	**ENSG0000008950**	CMTM1	protein_coding	chr16	66566393	66579137	+	Human GRCh	OTTHUMG000001	CMTM1	**CKLF like MARVEL transmembrane domain containing 1**	Other	other		1.05004417	0	0.87258262	0	1	0	7.07478112	7.03615516	7.20037556	7.00672615
1829	**ENSG0000008955**	KCNH4	protein_coding	chr17	42156891	42181142	-	Human GRCh	OTTHUMG000001	KCNH4	**potassium voltage-gated channel subfamily H member 4**	Plasma Mem	ion channel		3.257435892	1	0.13526494	0	1	0	2.39043908	2.75493331	3.10808813	3.25670153
1830	**ENSG0000008959**	GANAB	protein_coding	chr11	62624826	62646726	-	Human GRCh	OTTHUMG000001	GANAB	**glucosidase II alpha subunit**	Cytoplasm	enzyme	miglitol	-1.12713427	0	0.5827785	0	1	0	13.6559623	13.9040695	13.7866197	13.5026057
1831	**ENSG0000008963**	GMIP	protein_coding	chr19	19629476	19643657	-	Human GRCh	OTTHUMG000001	GMIP	**GEM interacting protein**	Cytoplasm	enzyme		-1.059455685	0	0.82059193	0	1	0	9.533793	10.065167	9.77935549	9.74758516
1832	**ENSG0000008968**	RBM41	protein_coding	chrX	107061885	107118823	-	Human GRCh	OTTHUMG000000	RBM41	**RNA binding motif protein 41**	Other	other		-1.078979308	0	0.73635527	0	1	0	10.1613482	10.5751861	10.4525891	10.1335659
1833	**ENSG0000008968**	BIRC5	protein_coding	chr17	78214186	78225636	+	Human GRCh	OTTHUMG000001	BIRC5	**baculoviral IAP repeat containing 5**	Cytoplasm	other	gataparser	-1.105123334	0	0.61800374	0	1	0	11.5290479	11.8940951	11.7106068	11.5077365
1834	**ENSG0000008969**	LAG3	protein_coding	chr12	6772512	6778455	+	Human GRCh	OTTHUMG000001	LAG3	**lymphocyte activating 3**	Plasma Mem	transmembra	EMB-02, LI	-1.588263269	-1	0.50802483	0	1	0	5.31598097	5.62956552	4.99914668	5.15787234
1835	**ENSG0000008969**	MLF2	protein_coding	chr12	6747996	6767475	-	Human GRCh	OTTHUMG000001	MLF2	**myeloid leukemia factor 2**	Nucleus	other		-1.007233091	0	0.97218977	0	1	0	12.2176825	12.5008512	12.3887083	12.3296801
1836	**ENSG0000008972**	OTUB2	protein_coding	chr14	94026340	94048930	+	Human GRCh	OTTHUMG000001	OTUB2	**OTU deubiquitinase, ubiquitin aldehyde binding 2**	Nucleus	enzyme		1.323297289	0	0.39163511	0	1	0	5.84424437	6.44991542	6.35203853	6.57488569
1837	**ENSG0000008973**	DDX24	protein_coding	chr14	94048287	94081202	-	Human GRCh	OTTHUMG000001	DDX24	**DEAD-box helicase 24**	Nucleus	enzyme		-1.090237287	0	0.67975253	0	1	0	11.9247954	12.3300642	12.1373161	11.9512202
1838	**ENSG0000008977**	ZBTB25	protein_coding	chr14	64449106	64505213	-	Human GRCh	OTTHUMG000001	ZBTB25	**zinc finger and BTB domain containing 25**	Nucleus	transcription		-1.042413817	0	0.87172076	0	1	0	10.2142774	10.540853	10.5188719	10.1367805
1839	**ENSG0000008981**	NECAP1	protein_coding	chr12	8076939	8097881	+	Human GRCh	OTTHUMG000001	NECAP1	**NECAP endocytosis associated 1**	Plasma Mem	other		-1.133298626	0	0.58544926	0	1	0	10.5275316	10.8390358	10.6703338	10.4206791
1840	**ENSG0000008982**	ARHGAP4	protein_coding	chrX	153907367	153934999	-	Human GRCh	OTTHUMG000000	ARHGAP4	**Rho GTPase activating protein 4**	Cytoplasm	other		2.394512619	1	0.02720844	1	1	0	6.7392406	7.17767095	7.66298977	8.05976841
1841	**ENSG0000008984**	ANKRD24	protein_coding	chr19	4182689	4224814	+	Human GRCh	OTTHUMG000001	ANKRD24	**ankyrin repeat domain 24**	Plasma Mem	other		1.234938368	0	0.46784693	0	1	0	8.65659137	8.98293037	9.13063359	9.01073524
1842	**ENSG0000008987**	DHX32	protein_coding	chr10	125836337	125896436	-	Human GRCh	OTTHUMG000000	DHX32	**DEAH-box helicase 32 (putative)**	Other	enzyme		1.507093204	1	0.11470816	0	1	0	8.1246304	8.52395325	8.7060884	8.84698455
1843	**ENSG0000008990**	RCOR1	protein_coding	chr14	102592646	102730561	+	Human GRCh	OTTHUMG000001	RCOR1	**REST corepressor 1**	Nucleus	transcription		-1.070645525	0	0.74682853	0	1	0	10.8560165	11.188442	11.0429158	10.8662966
1844	**ENSG0000008991**	GPATCH2L	protein_coding	chr14	76151916	76254342	+	Human GRCh	OTTHUMG000001	GPATCH2L	**G-patch domain containing 2 like**	Other	other		-1.110422075	0	0.62130897	0	1	0	11.934172	12.1018568	12.0840688	11.6939068
1845	**ENSG0000009000**	LTBP4	protein_coding	chr19	40592883	40629818	+	Human GRCh	OTTHUMG000001	LTBP4	**latent transforming growth factor beta binding protein 4**	Extracellular	growth facto		-1.14715075	0	0.55897477	0	1	0	10.5288125	10.9959855	10.6563529	10.5993715
1846	**ENSG0000009001**	BLVRB	protein_coding	chr19	40447765	40465764	-	Human GRCh	OTTHUMG000001	BLVRB	**biliverdin reductase B**	Cytoplasm	enzyme		1.081186475	0	0.7976503	0	1	0	7.51830404	7.95667667	7.87221183	7.81365617
1847	**ENSG0000009002**	SLC9A1	protein_coding	chr1	27098809	27166981	-	Human GRCh	OTTHUMG000000	SLC9A1	**solute carrier family 9 member A1**	Plasma Mem	ion channel	amiloride,	-1.247634571	0	0.54761399	0	1	0	9.93618977	10.2177127	9.77270317	9.9116763
1848	**ENSG0000009005**	SPTLC1	protein_coding	chr9	92000087	92115413	-	Human GRCh	OTTHUMG000000	SPTLC1	**serine palmitoyltransferase long chain base subunit 1**	Cytoplasm	enzyme		-1.026926789	0	0.89487886	0	1	0	10.1694972	10.437556	10.4094493	10.1391015
1849	**ENSG0000009006**	PAPOLA	protein_coding	chr14	96501433	96567116	+	Human GRCh	OTTHUMG000001	PAPOLA	**poly(A) polymerase alpha**	Nucleus	enzyme		-1.10556245	0	0.59986974	0	1	0	12.6585699	12.9477317	12.7973773	12.5998656
1850	**ENSG0000009006**	CCNK	protein_coding	chr14	99481169	99535044	+	Human GRCh	OTTHUMG000001	CCNK	**cyclin K**	Nucleus	kinase		1.000347414	0	0.9985612	0	1	0	10.7258473	10.9956792	10.9341785	10.7996729
1851	**ENSG0000009009**	PCBP4	protein_coding	chr3	51957454	51974016	-	Human GRCh	OTTHUMG000001	PCBP4	**poly(rC) binding protein 4**	Nucleus	other		1.213688907	0	0.50179944	0	1	0	9.68500671	9.96641775	10.0445764	10.0587317
1853	**ENSG0000009023**	YPEL3	protein_coding	chr16	30092314	30096915	-	Human GRCh	OTTHUMG000001	YPEL3	**yippee like 3**	Other	other		-1.091288699	0	0.70216793	0	1	0	8.61582369	8.94009314	8.6258037	8.75909191
1854	**ENSG0000009026**	MRPS33	protein_coding	chr7	141002610	141015228	-	Human GRCh	OTTHUMG000001	MRPS33	**mitochondrial ribosomal protein S33**	Cytoplasm	other		-1.084569906	0	0.74043493	0	1	0	9.37437593	9.70026854	9.65024004	9.22945752
1855	**ENSG0000009026**	NDUFB2	protein_coding	chr7	140690777	140722790	+	Human GRCh	OTTHUMG000001	NDUFB2	**NADH:ubiquinone oxidoreductase subunit B2**	Cytoplasm	enzyme		1.093200799	0	0.67171938	0	1	0	9.16299986	9.50154869	9.58085652	9.28648344
1856	**ENSG0000009027**	NUDC	protein_coding	chr1	26900238	26946871	+	Human GRCh	OTTHUMG000000	NUDC	**nuclear distribution C, dynein complex regulator**	Cytoplasm	other		1.275628127	0	0.23765843	0	1	0	10.7942622	11.1500491	11.2969849	11.2140956
1857	**ENSG0000009031**	MAEA	protein_coding	chr4	1289887	1340147	+	Human GRCh	OTTHUMG000001	MAEA	**macrophage erythroblast attacher, E3 ubiquitin ligase**	Plasma Mem	other		-1.118723208	0	0.55911228	0	1	0	11.2718196	11.5792773	11.402759	11.2073503
1858	**ENSG0000009033**	ICAM1	protein_coding	chr19	10271093	10286615	+	Human GRCh	OTTHUMG000001	ICAM1	**intercellular adhesion molecule 1**	Plasma Mem	transmembra	tyroserleut	2.387185182	1	0.14754807	0	1	0	3.78247098	4.06273809	4.9543787	4.12907306
1859	**ENSG0000009037**	STRN4	protein_coding	chr19	46719511	46746994	-	Human GRCh	OTTHUMG000001	STRN4	**striatin 4**	Cytoplasm	other		-1.039285363	0	0.83059092	0	1	0	11.9350463	12.1722954	12.119337	11.903468
1863	**ENSG0000009043**	MUL1	protein_coding	chr1	20499448	20508151	-	Human GRCh	OTTHUMG000000	MUL1	**mitochondrial E3 ubiquitin protein ligase 1**	Cytoplasm	enzyme		-1.096832551	0	0.66450912	0	1	0	9.20966501	9.39469953	9.31732845	9.07734102
1864	**ENSG0000009044**	TFAP4	protein_coding	chr16	4257186	4273075	-	Human GRCh	OTTHUMG000001	TFAP4	**transcription factor AP-4**	Nucleus	transcription		-1.112105514	0	0.57582236	0	1	0	10.5631889	10.8980196	10.6858851	10.5593124
1865	**ENSG0000009047**	PDCD7	protein_coding	chr15	65117379	65133808	-	Human GRCh	OTTHUMG000001	PDCD7	**programmed cell death 7**	Nucleus	other		-1.003670347	0	0.98606268	0	1	0	9.29108072	9.60695563	9.62265985	9.26049628
1866	**ENSG0000009048**	SPG21	protein_coding	chr15	64963022	64990310	-	Human GRCh	OTTHUMG000001	SPG21	**SPG21 abhydrolase domain containing, maspardin**	Plasma Mem	enzyme		-1.066004835	0	0.78796912	0	1	0	10.6973071	11.0606638	10.9544793	10.6710991
1868	**ENSG0000009052**	DNAJB11	protein_coding	chr3	186567403	186585800	+	Human GRCh	OTTHUMG000001	DNAJB11	**DnaJ heat shock protein family (Hsp40) member B11**	Cytoplasm	other		-1.055452458	0	0.79420042	0	1	0	10.4564972	10.6945063	10.6412228	10.3888646
1869	**ENSG0000009053**	P3H2	protein_coding	chr3	189956728	190122437	-	Human GRCh	OTTHUMG000001	P3H2	**prolyl 3-hydroxylase 2**	Cytoplasm	enzyme		1.022594672	0	0.97709542	0	1	0	3.68668925	4.08983641	3.9768476	3.87329562
1870	**ENSG0000009053**	THPO	protein_coding	chr3	184371935	184381968	-	Human GRCh	OTTHUMG000001	THPO	**thrombopoietin**	Extracellular	cytokine		2.01715069	1	0.43801491	0	1	0	2.82005002	3.3291585	2.44127418	4.07285083
1871	**ENSG0000009053**	CHRD	protein_coding	chr3	184380054	184390736	+	Human GRCh	OTTHUMG000001	CHRD	**chordin**	Extracellular	other		2.163370795	1	0.10861412	0	1	0	4.25491239	4.28494871	4.57986479	5.15893239
1872	**ENSG0000009055**	FLT3LG	protein_coding	chr19	49474207	49486231	+	Human GRCh	OTTHUMG000001	FLT3LG	**fms related receptor tyrosine kinase 3 ligand**	Extracellular	cytokine		1.276341627	0	0.57630211	0	1	0	5.32153605	5.75997434	6.12575042	5.33365064
1873	**ENSG0000009056**	RAB11FIP3	protein_coding	chr16	425649	523011	+	Human GRCh	OTTHUMG000000	RAB11FIP3	**RAB11 family interacting protein 3**	Cytoplasm	other		1.109968643	0	0.62876841	0	1	0	11.0489455	11.2926203	11.4408752	11.1221809
1874	**ENSG0000009058**	GNPTG	protein_coding	chr16	1351931	1365737	+	Human GRCh	OTTHUMG000000	GNPTG	**N-acetylglucosamine-1-phosphate transferase subunit gamma**	Cytoplasm	enzyme		-1.005914793	0	0.98009624	0	1	0	9.84828759	10.1399106	10.123186	9.85452158
1875	**ENSG0000009061**	ZNF268	protein_coding	chr12	133181409	133214832	+	Human GRCh	OTTHUMG000001	ZNF268	**zinc finger protein 268**	Nucleus	transcription		-1.046649795	0	0.86039133	0	1	0	9.40375064	9.57800847	9.65284168	9.18554051
1876	**ENSG0000009061**	GOLGA3	protein_coding	chr12	132768902	132829143	-	Human GRCh	OTTHUMG000001	GOLGA3	**golgin A3**	Cytoplasm	transporter		-1.096243434	0	0.65885122	0	1	0	12.5632611	12.8993767	12.776545	12.4802106
1877	**ENSG0000009062**	PABPC4	protein_coding	chr1	39560709	39576790	-	Human GRCh	OTTHUMG000000	PABPC4	**poly(A) binding protein cytoplasmic 4**	Cytoplasm	translation re		-1.013997695	0	0.94082247	0	1	0	12.1807368	12.3830707	12.3863014	12.142569
1879	**ENSG0000009066**	CERS4	protein_coding	chr19	8206736	8262421	+	Human GRCh	OTTHUMG000001	CERS4	**ceramide synthase 4**	Cytoplasm	transcription		-1.391402883	0	0.34026639	0	1	0	11.2442792	11.3627608	10.9508996	10.9453429
1880	**ENSG0000009067**	MCOLN1	protein_coding	chr19	7522624	7534010	+	Human GRCh	OTTHUMG000001	MCOLN1	**mucolipin TRP cation channel 1**	Cytoplasm	ion channel		-1.044193974	0	0.86142714	0	1	0	9.57936133	9.89459887	9.78510195	9.60527774
1881	**ENSG0000009068**	USP48	protein_coding	chr1	21678298	21783606	-	Human GRCh	OTTHUMG000000	USP48	**ubiquitin specific peptidase 48**	Plasma Mem	peptidase		-1.064981237	0	0.75013456	0	1	0	11.054747	11.3437575	11.3155289	10.9299032
1882	**ENSG0000009077**	EFNB1	protein_coding	chrX	68829021	68842160	+	Human GRCh	OTTHUMG000000	EFNB1	**ephrin B1**	Plasma Mem	other		1.027925782	0	0.88763178	0	1	0	7.63704347	7.77729544	7.61777622	7.84150781
1883	**ENSG0000009085**	PDPR	protein_coding	chr16	70114332	70162537	+	Human GRCh	OTTHUMG000001	PDPR	**pyruvate dehydrogenase phosphatase regulatory subunit**	Cytoplasm	enzyme		-1.297995463	0	0.22010935	0	1	0	12.1778609	12.5056057	12.1208279	11.9861703
1884	**ENSG0000009086**	AARS1	protein_coding	chr16	70251983	70289707	-	Human GRCh	OTTHUMG000001	AARS1	**alanyl-tRNA synthetase 1**	Cytoplasm	enzyme		-1.071097847	0	0.70991717	0	1	0	12.5367883	12.60022	12.6537334	12.3045529
1885	**ENSG0000009086**	GLG1	protein_coding	chr16	74447427	74607144	-	Human GRCh	OTTHUMG000001	GLG1	**golgi glycoprotein 1**	Cytoplasm	other		-1.178833087	0	0.37436438	0	1	0	12.2462057	12.478867	12.3137856	12.0386395
1886	**ENSG0000009088**	KIF4A	protein_coding	chrX	70290104	70420886	+	Human GRCh	OTTHUMG000000	KIF4A	**kinesin family member 4A**	Nucleus	other		-1.192495119	0	0.46041792	0	1	0	11.0187613	11.4170395	11.24687	10.7921892
1887	**ENSG0000009090**	TNRC6A	protein_coding	chr16	24610209	24827632	+	Human GRCh	OTTHUMG000000	TNRC6A	**trinucleotide repeat containing adaptor 6A**	Nucleus	other		-1.071855604	0	0.71566965	0	1	0	11.8784953	12.1277652	12.0635509	11.7860772
1888	**ENSG0000009092**	PLEKHG2	protein_coding	chr19	39412669	39428415	+	Human GRCh	OTTHUMG000001	PLEKHG2	**pleckstrin homology and RhoGEF domain containing G2**	Cytoplasm	other		-1.146044758	0	0.53294039	0	1	0	9.69446668	9.77942754	9.59271562	9.58479914
1889	**ENSG0000009093**	DLL3	protein_coding	chr19	39498895	39508481	+	Human GRCh	OTTHUMG000001	DLL3	**delta like canonical Notch ligand 3**	Extracellular	other	HPN328, B	1.437360533	0	0.22859236	0	1	0	5.85291115	5.82196256	6.26036989	6.06537267
1890	**ENSG0000009097**	NAT14	protein_coding	chr19	55485188	55487568	+	Human GRCh	OTTHUMG000001	NAT14	**N-acetyltransferase 14 (putative)**	Extracellular	other		1.450039376	0	0.09668411	0	1	0	8.38965575	8.52718401	9.08776943	8.57424231
1891	**ENSG0000009097**	PITPNM2	protein_coding	chr12	122983480	123151090	-	Human GRCh	OTTHUMG000001	PITPNM2	**phosphatidylinositol transfer protein membrane associated 2**	Cytoplasm	enzyme		1.236047013	0	0.36257624	0	1	0	9.793136	9.95963778	10.2354411	9.92689736
1892	**ENSG0000009098**	EXOC1	protein_coding	chr4	55853648	55905086	+	Human GRCh	OTTHUMG000001	EXOC1	**exocyst complex component 1**	Cytoplasm	transporter		-1.100540478	0	0.66427186	0	1	0	9.45195824	9.5982205	9.5260063	9.3127371
1893	**ENSG0000009100**	RBM27	protein_coding	chr5	146203605	146289223	+	Human GRCh	OTTHUMG000001	RBM27	**RNA binding motif protein 27**	Nucleus	other		-1.124170818	0	0.55615144	0	1	0	11.2389605	11.620939	11.4143649	11.2066311

1894	**ENSG0000009101**	POU4F3	protein_coding	chr5	146338839	146341728	+	Human GRCh	OTTHUMG000001	POU4F3	**POU class 4 homeobox 3**	Nucleus	transcription		2.24365857	1	0.41293067	0	NA	0	2.11420073	2.32336827	2.17038432	2.83691457
1895	**ENSG0000009103**	OSBPL8	protein_coding	chr12	76351797	76559809	-	Human GRCh	OTTHUMG000001	OSBPL8	**oxysterol binding protein like 8**	Plasma Mem	transporter		1.094083348	0	0.68602181	0	1	0	11.3406513	11.6452008	11.7848372	11.384175
1896	**ENSG0000009107**	DTX2	protein_coding	chr7	76461676	76505995	+	Human GRCh	OTTHUMG000001	DTX2	**deltex E3 ubiquitin ligase 2**	Nucleus	other		-1.087969094	0	0.75353754	0	1	0	8.84277288	9.29836411	8.8659343	9.12075191
1898	**ENSG0000009112**	PUS7	protein_coding	chr7	105439661	105522271	-	Human GRCh	OTTHUMG000001	PUS7	**pseudouridine synthase 7**	Nucleus	enzyme		-1.060044994	0	0.77791488	0	1	0	10.5588013	10.964826	10.8787438	10.5209039
1900	**ENSG0000009112**	NRCAM	protein_coding	chr7	108147623	108456717	-	Human GRCh	OTTHUMG000001	NRCAM	**neuronal cell adhesion molecule**	Plasma Mem	other		-1.887374289	-1	0.29658426	0	1	0	6.27890371	6.56293502	5.98463926	5.82180911
1901	**ENSG0000009113**	LAMB1	protein_coding	chr7	107923799	108003213	-	Human GRCh	OTTHUMG000001	LAMB1	**laminin subunit beta 1**	Extracellular	other		-1.141600921	0	0.7157132	0	1	0	7.87017324	7.92994942	7.92904034	7.55411788
1902	**ENSG0000009113**	SLC26A4	protein_coding	chr7	107660828	107717809	+	Human GRCh	OTTHUMG000001	SLC26A4	**solute carrier family 26 member 4**	Plasma Mem	transporter		-1.291578705	0	0.59277684	0	1	0	5.65572844	5.47165379	5.49019215	5.07309836
1903	**ENSG0000009113**	SLC26A3	protein_coding	chr7	107765467	107803225	-	Human GRCh	OTTHUMG000001	SLC26A3	**solute carrier family 26 member 3**	Plasma Mem	transporter		1.252288643	0	0.78351949	0	1	0	3.38786489	3.45845686	3.77483945	3.4487596
1904	**ENSG0000009114**	DLD	protein_coding	chr7	107891162	107931730	+	Human GRCh	OTTHUMG000001	DLD	**dihydrolipoamide dehydrogenase**	Cytoplasm	enzyme	hexachlorc	-1.047231234	0	0.83374039	0	1	0	10.4833484	10.844328	10.7836653	10.443007
1905	**ENSG0000009115**	WDR7	protein_coding	chr18	56651343	57029811	+	Human GRCh	OTTHUMG000001	WDR7	**WD repeat domain 7**	Cytoplasm	other		-1.152932841	0	0.51151203	0	1	0	10.1031095	10.3545289	10.1847578	9.97045373
1906	**ENSG0000009116**	TXNL1	protein_coding	chr18	56597209	56651600	-	Human GRCh	OTTHUMG000001	TXNL1	**thioredoxin like 1**	Cytoplasm	enzyme		-1.017394185	0	0.92946818	0	1	0	10.5615082	10.8852376	10.827238	10.5917451
1907	**ENSG0000009118**	IL5RA	protein_coding	chr3	3066324	3126613	-	Human GRCh	OTTHUMG000000	IL5RA	**interleukin 5 receptor subunit alpha**	Plasma Mem	transmembra	benralizum	-1.049285718	0	0.97210719	0	NA	0	1.86839318	1.91711227	1.69289096	2.02779247
1908	**ENSG0000009126**	ABCC6	protein_coding	chr16	16149565	16223522	-	Human GRCh	OTTHUMG000001	ABCC6	**ATP binding cassette subfamily C member 6**	Plasma Mem	transporter		2.058743593	1	0.18238413	0	1	0	3.63894089	2.94311232	4.32636071	3.05208074
1909	**ENSG0000009131**	CMTM6	protein_coding	chr3	32481312	32502852	-	Human GRCh	OTTHUMG000001	CMTM6	**CKLF like MARVEL transmembrane domain containing 6**	Extracellular	cytokine		-1.061188649	0	0.76470207	0	1	0	10.1690934	10.262704	10.294118	9.99221246
1910	**ENSG0000009140**	ITGA6	protein_coding	chr2	172427354	172506459	+	Human GRCh	OTTHUMG000001	ITGA6	**integrin subunit alpha 6**	Plasma Mem	transmembra		-1.102623079	0	0.70243633	0	1	0	7.64418724	7.71088169	7.66382824	7.49920077
1911	**ENSG0000009142**	RAPGEF4	protein_coding	chr2	172735274	173052893	+	Human GRCh	OTTHUMG000001	RAPGEF4	**Rap guanine nucleotide exchange factor 4**	Cytoplasm	other		-1.029425228	0	0.95263843	0	1	0	5.25037629	5.14082585	5.0102804	5.30872564
1912	**ENSG0000009143**	MAP3K20	protein_coding	chr2	173075435	173268015	+	Human GRCh	OTTHUMG000001	MAP3K20	**mitogen-activated protein kinase kinase kinase 20**	Cytoplasm	kinase		1.142536453	0	0.65371019	0	1	0	8.74533501	8.86132007	9.16144837	8.68538385
1914	**ENSG0000009148**	FH	protein_coding	chr1	241497511	241519799	-	Human GRCh	OTTHUMG000000	FH	**fumarate hydratase**	Cytoplasm	enzyme		-1.05841105	0	0.80543177	0	1	0	11.5350019	11.8460217	11.7318207	11.5324892
1915	**ENSG0000009149**	SEL1L3	protein_coding	chr4	25747433	25863760	-	Human GRCh	OTTHUMG000001	SEL1L3	**SEL1L family member 3**	Nucleus	other		1.210602263	0	0.61791642	0	1	0	7.75204465	7.4376401	7.84120309	7.73397964
1916	**ENSG0000009151**	TF	protein_coding	chr3	133746040	133796641	+	Human GRCh	OTTHUMG000001	TF	**transferrin**	Extracellular	transporter	TLD-1433,	-2.799293081	-1	0.16791352	0	1	0	3.16530995	3.70677564	3.12176321	2.50806426
1917	**ENSG0000009152**	CDV3	protein_coding	chr3	133573686	133590261	+	Human GRCh	OTTHUMG000001	CDV3	**CDV3 homolog**	Cytoplasm	other		-1.003226369	0	0.98514127	0	1	0	11.8944181	12.1882003	12.1391004	11.9466946
1918	**ENSG0000009153**	MYO15A	protein_coding	chr17	18108756	18179802	+	Human GRCh	OTTHUMG000000	MYO15A	**myosin XVA**	Cytoplasm	other		-1.466536401	0	0.31395119	0	1	0	6.39277483	6.61976417	6.20687573	6.029267
1919	**ENSG0000009154**	ALKBH5	protein_coding	chr17	18183078	18209954	+	Human GRCh	OTTHUMG000000	ALKBH5	**alkB homolog 5, RNA demethylase**	Nucleus	enzyme		1.076704924	0	0.7272243	0	1	0	11.6391856	12.0772198	12.0746881	11.8373583
1921	**ENSG0000009159**	NLRP1	protein_coding	chr17	5499415	5619424	-	Human GRCh	OTTHUMG000001	NLRP1	**NLR family pyrin domain containing 1**	Cytoplasm	enzyme		4.50936296	1	0.00613628	1	1	0	5.55143606	6.38424024	7.22875457	7.20828126
1922	**ENSG0000009162**	PITPNM3	protein_coding	chr17	6451263	6556555	-	Human GRCh	OTTHUMG000001	PITPNM3	**PITPNM family member 3**	Cytoplasm	enzyme		1.036554109	0	0.90087192	0	1	0	8.75566501	9.00307869	8.81838658	9.02126712
1923	**ENSG0000009164**	SPAG7	protein_coding	chr17	4959226	4967817	-	Human GRCh	OTTHUMG000001	SPAG7	**sperm associated antigen 7**	Nucleus	other		-1.116197161	0	0.58145369	0	1	0	9.22735252	9.26889977	9.2769736	8.9572552
1924	**ENSG0000009165**	ORC6	protein_coding	chr16	46689643	46698394	+	Human GRCh	OTTHUMG000001	ORC6	**origin recognition complex subunit 6**	Nucleus	other		-1.292224657	0	0.21094285	0	1	0	10.5584562	10.7666525	10.5404494	10.1916165
1925	**ENSG0000009165**	ZFHX4	protein_coding	chr8	76681239	76867281	+	Human GRCh	OTTHUMG000001	ZFHX4	**zinc finger homeobox 4**	Nucleus	transcription		1.188786998	0	0.60667122	0	1	0	9.67530883	9.83999766	9.86766672	10.0336601
1926	**ENSG0000009166**	SLC17A6	protein_coding	chr11	22338381	22379503	+	Human GRCh	OTTHUMG000001	SLC17A6	**solute carrier family 17 member 6**	Plasma Mem	transporter		1	0	1	0	1	0	0.37797799	0.4915338	0.47364051	0.38501661
1927	**ENSG0000009170**	CPA1	protein_coding	chr7	130380339	130388114	+	Human GRCh	OTTHUMG000001	CPA1	**carboxypeptidase A1**	Extracellular	peptidase		1	0	1	0	1	0	1.42250822	1.48474406	1.47464838	1.42623886
1928	**ENSG0000009173**	ZC3HC1	protein_coding	chr7	130018287	130051451	-	Human GRCh	OTTHUMG000001	ZC3HC1	**zinc finger C3HC-type containing 1**	Nucleus	other		1.063984769	0	0.79277732	0	1	0	8.24190632	8.89608946	8.86845052	8.45876266
1929	**ENSG0000009183**	ESR1	protein_coding	chr6	151656691	152129619	+	Human GRCh	OTTHUMG000000	ESR1	**estrogen receptor 1**	Nucleus	ligand-depen	4-hydroxy-	-5.529001288	-1	0.17817103	0	1	0	1.93629923	2.69225433	2.02340132	1.94260815
1930	**ENSG0000009184**	RGS17	protein_coding	chr6	153004459	153131282	-	Human GRCh	OTTHUMG000000	RGS17	**regulator of G protein signaling 17**	Cytoplasm	enzyme		-1.534086288	-1	0.3266841	0	1	0	3.90136169	4.8939385	4.48900729	3.79208872
1931	**ENSG0000009187**	ANGPT2	protein_coding	chr8	6499632	6563409	-	Human GRCh	OTTHUMG000000	ANGPT2	**angiopoietin 2**	Extracellular	growth facto	AMG 780,	-1.893210282	-1	0.452278	0	1	0	3.07498206	3.86759857	3.374297	2.43305858
1932	**ENSG0000009194**	TMEM101	protein_coding	chr17	44011188	44023946	-	Human GRCh	OTTHUMG000001	TMEM101	**transmembrane protein 101**	Extracellular	other		1.223909714	0	0.36913361	0	1	0	8.71829794	8.9487085	9.24328193	8.84780948
1933	**ENSG0000009197**	CD200	protein_coding	chr3	112332347	112362812	+	Human GRCh	OTTHUMG000001	CD200	**CD200 molecule**	Plasma Mem	other	samalizum	-4.880446235	-1	0.22505905	0	NA	0	2.4324351	2.32040632	1.38329145	1.43990212
1934	**ENSG0000009198**	CCDC80	protein_coding	chr3	112596797	112649530	-	Human GRCh	OTTHUMG000001	CCDC80	**coiled-coil domain containing 80**	Nucleus	other		-1.55132078	-1	0.52669496	0	1	0	4.38302604	5.80127405	5.34529961	4.00303434
1936	**ENSG0000009201**	PSME1	protein_coding	chr14	24136163	24138967	+	Human GRCh	OTTHUMG000000	PSME1	**proteasome activator subunit 1**	Cytoplasm	other		-1.044116117	0	0.86913397	0	1	0	9.95432906	10.6073139	10.4099478	10.128933
1937	**ENSG0000009202**	PPP2R3C	protein_coding	chr14	35085467	35122517	-	Human GRCh	OTTHUMG000001	PPP2R3C	**protein phosphatase 2 regulatory subunit B''gamma**	Cytoplasm	other		-1.306570772	0	0.25018498	0	1	0	8.61025634	9.12439119	8.81855835	8.34738631
1938	**ENSG0000009203**	HAUS4	protein_coding	chr14	22946228	22957161	-	Human GRCh	OTTHUMG000000	HAUS4	**HAUS augmin like complex subunit 4**	Cytoplasm	other		-1.057456808	0	0.81877847	0	1	0	9.17543839	9.46061508	9.35453669	9.1703677
1939	**ENSG0000009205**	JPH4	protein_coding	chr14	23568038	23578790	-	Human GRCh	OTTHUMG000000	JPH4	**junctophilin 4**	Cytoplasm	other		1.375207602	0	0.70517703	0	1	0	3.22658699	2.61757655	2.85056318	3.46994052
1942	**ENSG0000009206**	SLC7A8	protein_coding	chr14	23125295	23183674	-	Human GRCh	OTTHUMG000000	SLC7A8	**solute carrier family 7 member 8**	Plasma Mem	transporter		1.000959566	0	0.9980605	0	1	0	5.58732441	5.44453901	5.08165581	5.82660446
1943	**ENSG0000009209**	OSGEP	protein_coding	chr14	20446401	20455089	-	Human GRCh	OTTHUMG000000	OSGEP	**O-sialoglycoprotein endopeptidase**	Nucleus	peptidase		-1.097137562	0	0.67007453	0	1	0	10.3539306	10.4526508	10.4842989	10.0889715
1944	**ENSG0000009209**	SLC22A17	protein_coding	chr14	23346314	23352912	-	Human GRCh	OTTHUMG000000	SLC22A17	**solute carrier family 22 member 17**	Plasma Mem	transporter		1.263193535	0	0.6553186	0	1	0	5.51835748	3.7037371	5.44166059	4.83903617
1945	**ENSG0000009209**	RNF31	protein_coding	chr14	24146683	24160660	+	Human GRCh	OTTHUMG000000	RNF31	**ring finger protein 31**	Cytoplasm	enzyme		-1.13145349	0	0.5662911	0	1	0	11.3039423	11.5491632	11.4320098	11.1395011
1946	**ENSG0000009210**	SCFD1	protein_coding	chr14	30622291	30737694	+	Human GRCh	OTTHUMG000000	SCFD1	**sec1 family domain containing 1**	Cytoplasm	transporter		-1.001106694	0	0.99587346	0	1	0	11.0834505	11.359412	11.35378	11.0877501
1947	**ENSG0000009214**	G2E3	protein_coding	chr14	30559158	30620064	+	Human GRCh	OTTHUMG000001	G2E3	**G2/M-phase specific E3 ubiquitin protein ligase**	Cytoplasm	enzyme		1.115044097	0	0.62858873	0	1	0	10.2322372	10.4406071	10.6598562	10.2132002
1948	**ENSG0000009214**	HECTD1	protein_coding	chr14	31100117	31207804	-	Human GRCh	OTTHUMG000001	HECTD1	**HECT domain E3 ubiquitin protein ligase 1**	Other	enzyme		-1.114199115	0	0.5846524	0	1	0	12.7573476	12.9841055	12.9234609	12.559341
1949	**ENSG0000009219**	HNRNPC	protein_coding	chr14	21209136	21269494	-	Human GRCh	OTTHUMG000001	HNRNPC	**heterogeneous nuclear ribonucleoprotein C**	Nucleus	other		-1.124662353	0	0.55883005	0	1	0	14.0796807	14.3619003	14.2223581	13.9572576
1950	**ENSG0000009220**	RPGRIP1	protein_coding	chr14	21280083	21351301	+	Human GRCh	OTTHUMG000001	RPGRIP1	**RPGR interacting protein 1**	Extracellular	other		-2.341050257	-1	0.3551738	0	NA	0	2.7179938	3.4822094	2.26064262	2.97671387
1951	**ENSG0000009220**	SUPT16H	protein_coding	chr14	21351476	21384019	-	Human GRCh	OTTHUMG000000	SUPT16H	**SPT16 homolog, facilitates chromatin remodeling subunit**	Nucleus	transcription		-1.110564722	0	0.61061421	0	1	0	12.5347228	12.8408684	12.7091879	12.4404719
1952	**ENSG0000009220**	TOX4	protein_coding	chr14	21476597	21499175	+	Human GRCh	OTTHUMG000001	TOX4	**TOX high mobility group box family member 4**	Nucleus	other		-1.17926972	0	0.4383875	0	1	0	11.4687259	11.8895534	11.5975794	11.4210243
1953	**ENSG0000009220**	GEMIN2	protein_coding	chr14	39114285	39136973	+	Human GRCh	OTTHUMG000000	GEMIN2	**gem nuclear organelle associated protein 2**	Nucleus	other		1.11985912	0	0.66684709	0	1	0	8.21062134	8.54561934	8.75975917	8.20214161
1954	**ENSG0000009229**	TGM1	protein_coding	chr14	24249114	24264432	-	Human GRCh	OTTHUMG000000	TGM1	**transglutaminase 1**	Plasma Mem	enzyme		-1.559730083	-1	0.20227029	0	1	0	6.91932287	7.14613092	6.79289863	6.32849523
1955	**ENSG0000009233**	TINF2	protein_coding	chr14	24238286	24242663	-	Human GRCh	OTTHUMG000001	TINF2	**TERF1 interacting nuclear factor 2**	Nucleus	other		-1.008025969	0	0.97100892	0	1	0	9.64877299	9.89789998	9.89122351	9.63747686
1957	**ENSG0000009237**	TBL1Y	protein_coding	chrY	6910686	7101970	+	Human GRCh	OTTHUMG000000	TBL1Y	**transducin beta like 1 Y-linked**	Nucleus	other		-1.143188081	0	0.74508021	0	1	0	5.90756519	5.77753255	5.89806879	5.45442182
1958	**ENSG0000009242**	SEMA6A	protein_coding	chr5	116443555	116574823	-	Human GRCh	OTTHUMG000001	SEMA6A	**semaphorin 6A**	Plasma Mem	transmembra		1.089471344	0	0.76669274	0	1	0	9.57281934	9.85193908	9.76021687	9.87157465
1959	**ENSG0000009243**	TRPM7	protein_coding	chr15	50552473	50686797	-	Human GRCh	OTTHUMG000001	TRPM7	**transient receptor potential cation channel subfamily M meml**	Plasma Mem	kinase		-1.069020107	0	0.75407459	0	1	0	11.6788484	11.9352002	11.9412675	11.4951942
1960	**ENSG0000009244**	TYRO3	protein_coding	chr15	41557675	41583589	+	Human GRCh	OTTHUMG000001	TYRO3	**TYRO3 protein tyrosine kinase**	Plasma Mem	kinase	cabozantir	-1.172217577	0	0.46240259	0	1	0	9.337753	9.54171317	9.3002917	9.22573836
1961	**ENSG0000009247**	WDR76	protein_coding	chr15	43826980	43868412	+	Human GRCh	OTTHUMG000000	WDR76	**WD repeat domain 76**	Nucleus	other		-1.155099015	0	0.53776702	0	1	0	10.0733876	10.6176071	10.2464758	10.1868079
1962	**ENSG0000009252**	CAPN3	protein_coding	chr15	42359498	42412949	+	Human GRCh	OTTHUMG000001	CAPN3	**calpain 3**	Cytoplasm	peptidase		1.083403363	0	0.8555957	0	1	0	5.89310535	6.32742482	6.31921996	6.09041756
1963	**ENSG0000009253**	SNAP23	protein_coding	chr15	42491233	42545356	+	Human GRCh	OTTHUMG000001	SNAP23	**synaptosome associated protein 23**	Plasma Mem	transporter		1.003048142	0	0.98915907	0	1	0	9.33387774	9.66051805	9.62263696	9.38928428
1964	**ENSG0000009260**	TBX15	protein_coding	chr1	118883046	118989556	-	Human GRCh	OTTHUMG000000	TBX15	**T-box transcription factor 15**	Nucleus	transcription		-1.548509279	-1	0.35537773	0	1	0	4.40337904	5.01741664	4.43028807	4.28963034
1965	**ENSG0000009262**	PHGDH	protein_coding	chr1	119648411	119744218	+	Human GRCh	OTTHUMG000000	PHGDH	**phosphoglycerate dehydrogenase**	Cytoplasm	enzyme		-1.389680739	0	0.21735255	0	1	0	11.1934704	10.8376123	10.7867063	10.5580629
1966	**ENSG0000009275**	COL9A3	protein_coding	chr20	62816244	62841159	+	Human GRCh	OTTHUMG000000	COL9A3	**collagen type IX alpha 3 chain**	Extracellular	other	collagenas	1.134151176	0	0.68596998	0	1	0	7.52715016	7.51449074	7.60496536	7.71392508
1967	**ENSG0000009282**	EZR	protein_coding	chr6	158765741	158819368	-	Human GRCh	OTTHUMG000000	EZR	**ezrin**	Plasma Mem	other		-1.02332322	0	0.93152853	0	1	0	11.9831335	12.3112516	12.2367194	12.02133
1968	**ENSG0000009284**	MYL6	protein_coding	chr12	56158346	56163496	+	Human GRCh	OTTHUMG000001	MYL6	**myosin light chain 6**	Cytoplasm	enzyme		1.046241696	0	0.80376967	0	1	0	11.7823962	12.1164251	12.1148372	11.8966215
1969	**ENSG0000009284**	AGO1	protein_coding	chr1	35869808	35930532	+	Human GRCh	OTTHUMG000000	AGO1	**argonaute RISC component 1**	Cytoplasm	translation re		-1.041890643	0	0.86089586	0	1	0	12.5086943	12.7943389	12.7444138	12.4670597
1970	**ENSG0000009285**	TEKT2	protein_coding	chr1	36084094	36088275	+	Human GRCh	OTTHUMG000000	TEKT2	**tektin 2**	Cytoplasm	other		1.166909045	0	0.71473024	0	1	0	4.06585193	4.90766251	4.78215994	4.58417769
1971	**ENSG0000009285**	CLSPN	protein_coding	chr1	35720218	35769978	-	Human GRCh	OTTHUMG000000	CLSPN	**claspin**	Nucleus	other		-1.087220713	0	0.6574591	0	1	0	10.3538114	10.658602	10.4557994	10.3976303
1972	**ENSG0000009287**	RFFL	protein_coding	chr17	35005990	35089319	-	Human GRCh	OTTHUMG000001	RFFL	**ring finger and FYVE like domain containing E3 ubiquitin prote**	Cytoplasm	enzyme		1.082814198	0	0.71352975	0	1	0	10.0145779	10.1606053	10.4159642	9.87223082
1973	**ENSG0000009292**	UNC13D	protein_coding	chr17	75827225	75844785	-	Human GRCh	OTTHUMG000001	UNC13D	**unc-13 homolog D**	Cytoplasm	other		-1.753014357	-1	0.19757289	0	1	0	5.88941571	5.55936256	5.39656679	5.00115126
1974	**ENSG0000009293**	MFSD11	protein_coding	chr17	76735865	76781449	+	Human GRCh	OTTHUMG000001	MFSD11	**major facilitator superfamily domain containing 11**	Other	other		-1.209485432	0	0.37053222	0	1	0	9.30776795	9.69088993	9.52192191	9.04860402
1975	**ENSG0000009296**	DPYSL2	protein_coding	chr8	26514031	26658178	+	Human GRCh	OTTHUMG000000	DPYSL2	**dihydropyrimidinase like 2**	Cytoplasm	enzyme		-1.323743826	0	0.25872445	0	1	0	11.6185093	11.9849971	11.4618833	11.5552236
1976	**ENSG0000009296**	TGFB2	protein_coding	chr1	218345336	218444619	+	Human GRCh	OTTHUMG000000	TGFB2	**transforming growth factor beta 2**	Extracellular	growth facto	TASO-001,	-1.229916978	0	0.69495262	0	1	0	5.40424588	5.27140914	4.92589801	5.32535022
1977	**ENSG0000009297**	GPATCH2	protein_coding	chr1	217426992	217631090	-	Human GRCh	OTTHUMG000000	GPATCH2	**G-patch domain containing 2**	Nucleus	other		-1.123926953	0	0.56588078	0	1	0	11.0838426	11.4250967	11.2814358	10.9673072
1978	**ENSG0000009300**	NUP50	protein_coding	chr22	45163925	45188017	+	Human GRCh	OTTHUMG000001	NUP50	**nucleoporin 50**	Nucleus	transporter		1.023586862	0	0.89847014	0	1	0	11.7418711	11.8970919	11.9572812	11.7273336
1979	**ENSG0000009300**	CDC45	protein_coding	chr22	19479457	19520612	+	Human GRCh	OTTHUMG000001	CDC45	**cell division cycle 45**	Nucleus	other		-1.255422648	0	0.29333093	0	1	0	10.5505101	10.7796361	10.4902963	10.3305912
1980	**ENSG0000009301**	COMT	protein_coding	chr22	19941371	19969975	+	Human GRCh	OTTHUMG000001	COMT	**catechol-O-methyltransferase**	Cytoplasm	enzyme	carbidopa/	-1.005724001	0	0.97866741	0	1	0	9.89911552	10.3106923	10.1340703	10.0974157
1981	**ENSG0000009307**	ADA2	protein_coding	chr22	17178790	17258235	-	Human GRCh	OTTHUMG000000	ADA2	**adenosine deaminase 2**	Extracellular	enzyme		3.430453395	1	0.17232136	0	1	0	2.59043456	2.88452668	2.65905109	3.66207663
1982	**ENSG0000009314**	ECHDC1	protein_coding	chr6	127288712	127343609	-	Human GRCh	OTTHUMG000000	ECHDC1	**ethylmalonyl-CoA decarboxylase 1**	Cytoplasm	enzyme		1	0	1	0	1	0	2.84192212	2.93951745	2.9237547	2.84780361
1983	**ENSG0000009316**	LRRFIP2	protein_coding	chr3	37052626	37183689	-	Human GRCh	OTTHUMG000001	LRRFIP2	**LRR binding FLII interacting protein 2**	Cytoplasm	other		-1.086818204	0	0.70629367	0	1	0	11.1831445	11.5258708	11.5301291	10.9453779
1984	**ENSG0000009318**	SEC22C	protein_coding	chr3	42547969	42601080	-	Human GRCh	OTTHUMG000001	SEC22C	**SEC22 homolog C, vesicle trafficking protein**	Cytoplasm	other		-1.1236165	0	0.60787422	0	1	0	10.8927416	11.0298502	10.9500729	10.7030757

1985	**ENSG0000009321**	XYLB	protein_coding	chr3	38346760	38421348	+	Human GRCh	OTTHUMG000001	XYLB	**xylulokinase**	Cytoplasm	kinase		-1.038446067	0	0.8591229	0	1	0	10.6122	10.9073626	10.884567	10.5438509
1986	**ENSG0000009463**	HDAC6	protein_coding	chrX	48801377	48824982	+	Human GRCh	OTTHUMG000000	HDAC6	**histone deacetylase 6**	Nucleus	transcription	trametinib	-1.087495036	0	0.68698736	0	1	0	10.4005164	10.6295018	10.6487785	10.1597126
1990	**ENSG0000009480**	CDC6	protein_coding	chr17	40287879	40304657	+	Human GRCh	OTTHUMG000001	CDC6	**cell division cycle 6**	Nucleus	other		-1.159019768	0	0.47620129	0	1	0	11.160604	11.527273	11.3185459	11.0484918
1991	**ENSG0000009484**	UPRT	protein_coding	chrX	75156388	75304885	+	Human GRCh	OTTHUMG000000	UPRT	**uracil phosphoribosyltransferase homolog**	Nucleus	enzyme		1.155536143	0	0.5832557	0	1	0	8.01059832	8.26242434	8.47354432	8.09557056
1992	**ENSG0000009488**	CDC23	protein_coding	chr5	138187650	138213343	-	Human GRCh	OTTHUMG000001	CDC23	**cell division cycle 23**	Nucleus	enzyme		-1.056461915	0	0.81463754	0	1	0	10.3210053	10.7790321	10.5764922	10.4402044
1993	**ENSG0000009491**	AAAS	protein_coding	chr12	53307456	53324864	-	Human GRCh	OTTHUMG000001	AAAS	**aladin WD repeat nucleoporin**	Nucleus	other		-1.119384747	0	0.59560816	0	1	0	10.5197242	10.7538386	10.5650299	10.4703585
1994	**ENSG0000009491**	CBX5	protein_coding	chr12	54230942	54280133	-	Human GRCh	OTTHUMG000001	CBX5	**chromobox 5**	Nucleus	transcription		-1.175451628	0	0.50305956	0	1	0	13.0440901	13.1744955	13.1800138	12.6384064
1996	**ENSG0000009497**	SUCO	protein_coding	chr1	172532349	172611833	+	Human GRCh	OTTHUMG000000	SUCO	**SUN domain containing ossification factor**	Cytoplasm	other		1.006941263	0	0.9767844	0	1	0	11.6006872	11.8607794	11.9025423	11.5643827
1997	**ENSG0000009500**	MSH2	protein_coding	chr2	47403067	47663146	+	Human GRCh	OTTHUMG000001	MSH2	**mutS homolog 2**	Nucleus	enzyme		-1.0609216	0	0.80162334	0	1	0	10.435862	10.8439758	10.7372355	10.4333108
1998	**ENSG0000009501**	MAP3K1	protein_coding	chr5	56815549	56896152	+	Human GRCh	OTTHUMG000000	MAP3K1	**mitogen-activated protein kinase kinase kinase 1**	Cytoplasm	kinase	IKK-beta a	-1.002683621	0	0.99028768	0	1	0	9.71178774	10.1300122	9.92006651	9.95240047
1999	**ENSG0000009505**	DHPS	protein_coding	chr19	12675717	12681880	-	Human GRCh	OTTHUMG000001	DHPS	**deoxyhypusine synthase**	Cytoplasm	enzyme	dapsone	-1.003545843	0	0.98560117	0	1	0	10.3057679	10.4887322	10.5229517	10.2557124
2000	**ENSG0000009506**	HOOK2	protein_coding	chr19	12763003	12872740	-	Human GRCh	OTTHUMG000001	HOOK2	**hook microtubule tethering protein 2**	Cytoplasm	other		-1.044616231	0	0.84692751	0	1	0	10.6132091	10.7822044	10.7856708	10.5020745
2002	**ENSG0000009513**	ARCN1	protein_coding	chr11	118572390	118603033	+	Human GRCh	OTTHUMG000001	ARCN1	**archain 1**	Cytoplasm	other		-1.162019374	0	0.50677294	0	1	0	12.1307629	12.5115457	12.277722	12.0461156
2003	**ENSG0000009520**	EPB41L4B	protein_coding	chr9	109171974	109321059	-	Human GRCh	OTTHUMG000000	EPB41L4B	**erythrocyte membrane protein band 4.1 like 4B**	Cytoplasm	transporter		-1.140572923	0	0.55165816	0	1	0	10.7188064	11.1411363	10.8795759	10.7225839
2004	**ENSG0000009520**	TMEM38B	protein_coding	chr9	105694541	105776629	+	Human GRCh	OTTHUMG000000	TMEM38B	**transmembrane protein 38B**	Nucleus	ion channel		1.132515203	0	0.60801093	0	1	0	9.22017512	9.35731479	9.58734044	9.24384756
2005	**ENSG0000009526**	PSMD5	protein_coding	chr9	120815496	120842951	-	Human GRCh	OTTHUMG000000	PSMD5	**proteasome 26S subunit, non-ATPase 5**	Cytoplasm	other		-1.068202618	0	0.7617059	0	1	0	10.6965682	10.9166267	10.8574147	10.6063618
2006	**ENSG0000009530**	PTGS1	protein_coding	chr9	122370530	122395703	+	Human GRCh	OTTHUMG000000	PTGS1	**prostaglandin-endoperoxide synthase 1**	Cytoplasm	enzyme	sulindac/ta	1.019718787	0	0.97131885	0	1	0	4.7018131	4.74375682	4.4049634	4.99288823
2007	**ENSG0000009531**	NUP188	protein_coding	chr9	128947699	129007096	+	Human GRCh	OTTHUMG000000	NUP188	**nucleoporin 188**	Nucleus	other		-1.148851053	0	0.50182996	0	1	0	13.013313	13.4128938	13.1670054	12.9713337
2008	**ENSG0000009532**	CRAT	protein_coding	chr9	129094142	129111189	-	Human GRCh	OTTHUMG000001	CRAT	**carnitine O-acetyltransferase**	Cytoplasm	enzyme		-1.04138694	0	0.8881739	0	1	0	10.3730676	10.7377775	10.611247	10.4294857
2009	**ENSG0000009537**	SH2D3C	protein_coding	chr9	127738317	127778710	-	Human GRCh	OTTHUMG000000	SH2D3C	**SH2 domain containing 3C**	Cytoplasm	other		1.470826393	0	0.53630383	0	1	0	4.14063214	4.11370345	4.28588239	4.58659188
2010	**ENSG0000009538**	NANS	protein_coding	chr9	98056732	98083077	+	Human GRCh	OTTHUMG000000	NANS	**N-acetylneuraminate synthase**	Cytoplasm	enzyme		-1.117958395	0	0.65458622	0	1	0	10.2393761	10.5709208	10.3483409	10.2273787
2011	**ENSG0000009538**	TBC1D2	protein_coding	chr9	98199011	98255649	-	Human GRCh	OTTHUMG000000	TBC1D2	**TBC1 domain family member 2**	Cytoplasm	other		1.178713349	0	0.5643534	0	1	0	6.91776472	6.83954385	7.19543497	6.88063226
2012	**ENSG0000009539**	WHRN	protein_coding	chr9	114402080	114505473	-	Human GRCh	OTTHUMG000000	WHRN	**whirlin**	Plasma Mem	other		1.085613841	0	0.74069854	0	1	0	8.37925861	8.71556507	8.72795507	8.56266074
2014	**ENSG0000009548**	CWF19L1	protein_coding	chr10	100232298	100267680	-	Human GRCh	OTTHUMG000000	CWF19L1	**CWF19 like cell cycle control factor 1**	Other	other		-1.119656183	0	0.55991744	0	1	0	9.47845817	9.65405073	9.51504347	9.37061028
2015	**ENSG0000009553**	SEMA4G	protein_coding	chr10	100969504	100985871	+	Human GRCh	OTTHUMG000000	SEMA4G	**semaphorin 4G**	Plasma Mem	other		1.730855649	1	0.08060872	0	1	0	7.38723835	7.60173585	8.04863604	8.16613934
2016	**ENSG0000009556**	BTAF1	protein_coding	chr10	91923770	92031437	+	Human GRCh	OTTHUMG000000	BTAF1	**B-TFIID TATA-box binding protein associated factor 1**	Nucleus	transcription		-1.026698855	0	0.90125084	0	1	0	11.6146128	11.8967024	11.8481691	11.6092053
2017	**ENSG0000009557**	IKZF5	protein_coding	chr10	122990807	123008812	-	Human GRCh	OTTHUMG000000	IKZF5	**IKAROS family zinc finger 5**	Nucleus	transcription		-1.008304833	0	0.96904509	0	1	0	9.60060495	9.81256425	9.80488444	9.58955825
2018	**ENSG0000009558**	BLNK	protein_coding	chr10	96189171	96271587	-	Human GRCh	OTTHUMG000000	BLNK	**B cell linker**	Cytoplasm	other		-1.144865743	0	0.84741917	0	1	0	4.68531548	4.68696579	4.78678491	4.28956478
2019	**ENSG0000009558**	TLL2	protein_coding	chr10	96364608	96513926	-	Human GRCh	OTTHUMG000000	TLL2	**tolloid like 2**	Extracellular	peptidase		-1.475585523	0	0.45876691	0	1	0	4.55936758	4.41908306	4.56767105	3.57354867
2020	**ENSG0000009559**	CYP26A1	protein_coding	chr10	93073475	93077885	+	Human GRCh	OTTHUMG000000	CYP26A1	**cytochrome P450 family 26 subfamily A member 1**	Cytoplasm	enzyme		2.90863673	1	0.2665542	0	NA	0	2.56198096	2.45987818	3.52843349	2.73958569
2021	**ENSG0000009562**	TDRD1	protein_coding	chr10	114179270	114232666	+	Human GRCh	OTTHUMG000000	TDRD1	**tudor domain containing 1**	Cytoplasm	other		5.972772275	1	0.00942293	1	1	0	5.73289181	4.64491822	5.19254839	7.45765116
2022	**ENSG0000009563**	SORBS1	protein_coding	chr10	95311771	95561414	-	Human GRCh	OTTHUMG000000	SORBS1	**sorbin and SH3 domain containing 1**	Plasma Mem	other		-1.016293792	0	0.94701196	0	1	0	7.27110761	7.13444871	7.23922942	7.13979277
2023	**ENSG0000009571**	CRTAC1	protein_coding	chr10	97865000	98030828	-	Human GRCh	OTTHUMG000000	CRTAC1	**cartilage acidic protein 1**	Extracellular	other		1	0	1	0	1	0	2.12001158	2.22458661	2.20781101	2.12636311
2024	**ENSG0000009573**	BAMBI	protein_coding	chr10	28677510	28682932	+	Human GRCh	OTTHUMG000000	BAMBI	**BMP and activin membrane bound inhibitor**	Plasma Mem	other		1.245996576	0	0.6480529	0	1	0	5.26007576	4.68841278	5.52837504	4.76949423
2025	**ENSG0000009575**	IL11	protein_coding	chr19	55364382	55370463	-	Human GRCh	OTTHUMG000001	IL11	**interleukin 11**	Extracellular	cytokine		-1.675613699	-1	0.46484134	0	1	0	3.82716225	4.06558207	3.79142403	3.29152161
2027	**ENSG0000009578**	WAC	protein_coding	chr10	28532493	28623112	+	Human GRCh	OTTHUMG000000	WAC	**WW domain containing adaptor with coiled-coil**	Nucleus	other		-1.124050212	0	0.55540642	0	1	0	12.3478823	12.6032132	12.4414023	12.2592575
2028	**ENSG0000009579**	CREM	protein_coding	chr10	35126791	35212958	+	Human GRCh	OTTHUMG000000	CREM	**cAMP responsive element modulator**	Nucleus	transcription		1.049629834	0	0.85952256	0	1	0	7.65775022	7.87184294	8.06455741	7.51130138
2029	**ENSG0000009590**	NUBP2	protein_coding	chr16	1782932	1789186	+	Human GRCh	OTTHUMG000001	NUBP2	**NUBP iron-sulfur cluster assembly factor 2, cytosolic**	Nucleus	other		1.008406661	0	0.96823886	0	1	0	10.679319	10.9216316	10.9419508	10.6749877
2030	**ENSG0000009591**	TPSD1	protein_coding	chr16	1256059	1259008	+	Human GRCh	OTTHUMG000001	TPSD1	**tryptase delta 1**	Extracellular	peptidase		1	0	1	0	NA	0	0.76736559	0.78638477	0.78323159	0.76847727
2031	**ENSG0000009593**	SMIM24	protein_coding	chr19	3473986	3480525	-	Human GRCh	OTTHUMG000001	SMIM24	**small integral membrane protein 24**	Cytoplasm	other		2.899497066	1	0.00039451	1	0.38	0	5.47823811	5.16764059	6.03299851	6.63411104
2032	**ENSG0000009595**	HIVEP1	protein_coding	chr6	12008762	12164999	+	Human GRCh	OTTHUMG000000	HIVEP1	**HIVEP zinc finger 1**	Nucleus	transcription		-1.080750657	0	0.70516698	0	1	0	11.0643849	11.3599394	11.2228752	11.0399626
2034	**ENSG0000009598**	KCNK16	protein_coding	chr6	39314698	39322968	-	Human GRCh	OTTHUMG000000	KCNK16	**potassium two pore domain channel subfamily K member 16**	Plasma Mem	ion channel		1	0	1	0	1	0	1.2169385	1.30756949	1.29305208	1.2224507
2035	**ENSG0000009600**	CRISP3	protein_coding	chr6	49727376	49744437	-	Human GRCh	OTTHUMG000000	CRISP3	**cysteine rich secretory protein 3**	Extracellular	other		-5.968641622	-1	0.42631923	0	NA	0	0.52249275	0.97124307	0.53119351	0.52309778
2036	**ENSG0000009606**	FKBP5	protein_coding	chr6	35573585	35728583	-	Human GRCh	OTTHUMG000000	FKBP5	**FKBP prolyl isomerase 5**	Nucleus	enzyme		-1.190287942	0	0.45339684	0	1	0	14.7243498	15.0391239	14.8584789	14.4822971
2037	**ENSG0000009606**	SRPK1	protein_coding	chr6	35832966	35921342	-	Human GRCh	OTTHUMG000000	SRPK1	**SRSF protein kinase 1**	Nucleus	kinase	sphinx31,	-1.131715559	0	0.52842209	0	1	0	12.0910844	12.5054276	12.3120475	12.0267653
2038	**ENSG0000009607**	BRPF3	protein_coding	chr6	36196744	36232790	+	Human GRCh	OTTHUMG000000	BRPF3	**bromodomain and PHD finger containing 3**	Nucleus	other		-1.168373923	0	0.47708593	0	1	0	10.7703952	10.923176	10.7654568	10.5940164
2039	**ENSG0000009608**	MRPS18A	protein_coding	chr6	43671202	43687791	-	Human GRCh	OTTHUMG000000	MRPS18A	**mitochondrial ribosomal protein S18A**	Cytoplasm	other		-1.096536572	0	0.6539593	0	1	0	9.70847971	10.0633306	9.89005171	9.69434084
2040	**ENSG0000009608**	PGC	protein_coding	chr6	41736711	41754109	-	Human GRCh	OTTHUMG000000	PGC	**progastricsin**	Extracellular	peptidase		1.500131474	1	0.47596378	0	1	0	4.79267037	4.84878137	4.69229706	5.62488559
2041	**ENSG0000009609**	TMEM14A	protein_coding	chr6	52671113	52686588	+	Human GRCh	OTTHUMG000000	TMEM14A	**transmembrane protein 14A**	Cytoplasm	other		1.408749159	0	0.22910654	0	1	0	6.73382536	6.92910536	7.42421373	6.86572868
2042	**ENSG0000009609**	EFHC1	protein_coding	chr6	52362123	52529886	+	Human GRCh	OTTHUMG000000	EFHC1	**EF-hand domain containing 1**	Cytoplasm	other		-1.002684084	0	0.99251391	0	1	0	8.98810926	9.12235473	9.26089287	8.80294271
2044	**ENSG0000009638**	HSP90AB1	protein_coding	chr6	44246166	44253888	+	Human GRCh	OTTHUMG000000	HSP90AB1	**heat shock protein 90 alpha family class B member 1**	Cytoplasm	enzyme	alvespimyc	-1.145321035	0	0.48900931	0	1	0	14.6515734	14.9735534	14.8080689	14.5137079
2046	**ENSG0000009640**	CDC5L	protein_coding	chr6	44387706	44450425	+	Human GRCh	OTTHUMG000000	CDC5L	**cell division cycle 5 like**	Nucleus	transcription		-1.0686491	0	0.73749705	0	1	0	10.6275429	10.9454337	10.8487438	10.5859502
2047	**ENSG0000009643**	ITPR3	protein_coding	chr6	33620365	33696574	+	Human GRCh	OTTHUMG000000	ITPR3	**inositol 1,4,5-trisphosphate receptor type 3**	Cytoplasm	ion channel		1.112576208	0	0.75169174	0	1	0	11.0366831	11.4874178	10.8428138	11.7616207
2048	**ENSG0000009665**	ZNF184	protein_coding	chr6	27450743	27473118	-	Human GRCh	OTTHUMG000000	ZNF184	**zinc finger protein 184**	Nucleus	transcription		-1.04256918	0	0.84676857	0	1	0	8.65324307	8.8355109	8.81274403	8.58247015
2049	**ENSG0000009669**	DSP	protein_coding	chr6	7541338	7586714	+	Human GRCh	OTTHUMG000000	DSP	**desmoplakin**	Plasma Mem	other		1.299268067	0	0.44662362	0	1	0	12.6875117	12.6959369	12.887805	13.0309619
2050	**ENSG0000009671**	SIRT1	protein_coding	chr10	67884656	67918390	+	Human GRCh	OTTHUMG000000	SIRT1	**sirtuin 1**	Nucleus	transcription	SIRT1 inhib	-1.177407322	0	0.42495529	0	1	0	9.54474152	9.63512899	9.55912944	9.25266355
2051	**ENSG0000009674**	HNRNPH3	protein_coding	chr10	68331174	68343193	+	Human GRCh	OTTHUMG000000	HNRNPH3	**heterogeneous nuclear ribonucleoprotein H3**	Nucleus	other		1.258501267	0	0.25662075	0	1	0	10.9968519	11.0419779	11.4388832	11.0632672
2052	**ENSG0000009687**	IFT74	protein_coding	chr9	26947039	27066134	+	Human GRCh	OTTHUMG000000	IFT74	**intraflagellar transport 74**	Cytoplasm	other		1.049761901	0	0.82166135	0	1	0	9.37594441	9.51998149	9.66752257	9.31150221
2053	**ENSG0000009696**	JAK2	protein_coding	chr9	4984390	5129948	+	Human GRCh	OTTHUMG000000	JAK2	**Janus kinase 2**	Cytoplasm	kinase	EXEL-8232	1.04983503	0	0.83604081	0	1	0	8.93129603	9.24680066	9.27760888	9.01349224
2055	**ENSG0000009700**	ABL1	protein_coding	chr9	130713043	130887675	+	Human GRCh	OTTHUMG000000	ABL1	**ABL proto-oncogene 1, non-receptor tyrosine kinase**	Nucleus	kinase	olveremba	-1.070644274	0	0.7321801	0	1	0	11.6153776	11.8782898	11.8451621	11.4874481
2056	**ENSG0000009702**	ACOT7	protein_coding	chr1	6264269	6393767	-	Human GRCh	OTTHUMG000000	ACOT7	**acyl-CoA thioesterase 7**	Cytoplasm	enzyme		-1.167491458	0	0.58138545	0	1	0	9.93658467	10.3309815	9.86561045	10.0853075
2057	**ENSG0000009703**	SH3GLB1	protein_coding	chr1	86704570	86748184	+	Human GRCh	OTTHUMG000000	SH3GLB1	**SH3 domain containing GRB2 like, endophilin B1**	Cytoplasm	enzyme		-1.153861685	0	0.49964952	0	1	0	10.7563758	11.0713867	10.9230293	10.5890734
2058	**ENSG0000009704**	CDC7	protein_coding	chr1	91500851	91525764	+	Human GRCh	OTTHUMG000000	CDC7	**cell division cycle 7**	Nucleus	kinase	TQB3824,	-1.360530235	0	0.19578094	0	1	0	9.86025258	10.1426619	9.85614075	9.47356113
2059	**ENSG0000009709**	SYDE2	protein_coding	chr1	85156889	85201724	-	Human GRCh	OTTHUMG000000	SYDE2	**synapse defective Rho GTPase homolog 2**	Cytoplasm	other		1.187621681	0	0.50514772	0	1	0	8.80643726	9.09567904	9.12593632	9.14964859
2060	**ENSG0000009913**	PCSK5	protein_coding	chr9	75890644	76362975	+	Human GRCh	OTTHUMG000000	PCSK5	**proprotein convertase subtilisin/kexin type 5**	Extracellular	peptidase		1.114669721	0	0.76230649	0	1	0	8.33741759	8.81478513	8.86252276	8.56252799
2061	**ENSG0000009919**	SCD	protein_coding	chr10	100347233	100364826	+	Human GRCh	OTTHUMG000000	SCD	**stearoyl-CoA desaturase**	Cytoplasm	enzyme		-1.16548296	0	0.59351164	0	1	0	14.2456438	14.7014922	14.3817941	14.2469179
2062	**ENSG0000009920**	TMED1	protein_coding	chr19	10832067	10836318	-	Human GRCh	OTTHUMG000001	TMED1	**transmembrane p24 trafficking protein 1**	Extracellular	transporter		-1.210757666	0	0.41730973	0	1	0	8.63488992	8.83550833	8.58106667	8.47710939
2063	**ENSG0000009920**	ABLIM1	protein_coding	chr10	114431112	114768061	-	Human GRCh	OTTHUMG000000	ABLIM1	**actin binding LIM protein 1**	Cytoplasm	other		1.043672311	0	0.85036511	0	1	0	11.0393337	11.1325803	11.0659325	11.2021219
2064	**ENSG0000009921**	ERMP1	protein_coding	chr9	5749832	5879537	-	Human GRCh	OTTHUMG000000	ERMP1	**endoplasmic reticulum metallopeptidase 1**	Cytoplasm	other		-1.208537316	0	0.40709812	0	1	0	10.4872303	10.9113684	10.669445	10.3167394
2065	**ENSG0000009924**	RAB18	protein_coding	chr10	27504174	27543207	+	Human GRCh	OTTHUMG000000	RAB18	**RAB18, member RAS oncogene family**	Cytoplasm	enzyme		1.106314465	0	0.62571934	0	1	0	10.7325755	10.8659254	11.1115405	10.6711002
2066	**ENSG0000009925**	NRP1	protein_coding	chr10	33177492	33336262	-	Human GRCh	OTTHUMG000000	NRP1	**neuropilin 1**	Plasma Mem	transmembra	vesencuma	1.407100669	0	0.28781785	0	1	0	8.97979231	9.4692519	9.77433569	9.46684574
2067	**ENSG0000009925**	PRTFDC1	protein_coding	chr10	24848614	24952606	-	Human GRCh	OTTHUMG000000	PRTFDC1	**phosphoribosyl transferase domain containing 1**	Cytoplasm	enzyme		-5.686572114	-1	0.00464886	1	1	0	4.69891439	4.53660987	4.0027509	3.20744312
2068	**ENSG0000009926**	PALMD	protein_coding	chr1	99646113	99694541	+	Human GRCh	OTTHUMG000000	PALMD	**palmdelphin**	Cytoplasm	other		-1.281715031	0	0.75970036	0	1	0	6.8706354	7.43817906	6.37073291	7.26365797
2069	**ENSG0000009928**	TSPAN15	protein_coding	chr10	69451465	69507666	+	Human GRCh	OTTHUMG000000	TSPAN15	**tetraspanin 15**	Plasma Mem	other		1.504520865	1	0.12917859	0	1	0	6.69960926	6.21517942	7.06795383	6.64781176
2070	**ENSG0000009928**	MACROH2	protein_coding	chr10	70052544	70112282	+	Human GRCh	OTTHUMG000000	MACROH2A2	**macroH2A.2 histone**	Nucleus	other		-1.015307466	0	0.94511973	0	1	0	10.407764	10.6125199	10.6688621	10.2995589
2071	**ENSG0000009929**	WASHC2A	protein_coding	chr10	50067888	50133509	+	Human GRCh	OTTHUMG000000	WASHC2A/W	**WASH complex subunit 2A**	Cytoplasm	other		-1.164761462	0	0.66199397	0	1	0	9.98298455	10.3196328	10.1630191	9.79877207
2072	**ENSG0000009930**	MAST3	protein_coding	chr19	18097778	18151692	+	Human GRCh	OTTHUMG000001	MAST3	**microtubule associated serine/threonine kinase 3**	Cytoplasm	kinase		1.181092661	0	0.52790007	0	1	0	8.9291075	9.26304255	9.31119185	9.26182261
2073	**ENSG0000009932**	MZF1	protein_coding	chr19	58561931	58573575	-	Human GRCh	OTTHUMG000001	MZF1	**myeloid zinc finger 1**	Nucleus	transcription		-1.077285603	0	0.72865484	0	1	0	10.6253001	10.8102383	10.7290937	10.5426308
2074	**ENSG0000009933**	OCEL1	protein_coding	chr19	17226213	17229219	+	Human GRCh	OTTHUMG000001	OCEL1	**occludin/ELL domain containing 1**	Other	other		1.132296314	0	0.70420478	0	1	0	7.88435878	8.2180074	8.23550386	8.16147754
2075	**ENSG0000009933**	MYO9B	protein_coding	chr19	17075777	17214537	+	Human GRCh	OTTHUMG000001	MYO9B	**myosin IXB**	Cytoplasm	enzyme		-1.081107342	0	0.72411005	0	1	0	11.600963	11.886348	11.8017232	11.513122
2076	**ENSG0000009933**	KCNK6	protein_coding	chr19	38319845	38332076	+	Human GRCh	OTTHUMG000001	KCNK6	**potassium two pore domain channel subfamily K member 6**	Plasma Mem	ion channel		2.004214634	1	0.18929556	0	1	0	4.50217537	4.82865508	5.62928378	4.92977622
2077	**ENSG0000009933**	CATSPERG	protein_coding	chr19	38335775	38370943	+	Human GRCh	OTTHUMG000001	CATSPERG	**cation channel sperm associated auxiliary subunit gamma**	Extracellular	other		-1.191340477	0	0.5730149	0	1	0	7.78127227	8.00394181	7.77307227	7.65062379
2078	**ENSG0000009934**	PSMD8	protein_coding	chr19	38374563	38383850	+	Human GRCh	OTTHUMG000001	PSMD8	**proteasome 26S subunit, non-ATPase 8**	Cytoplasm	other		-1.117236147	0	0.60728778	0	1	0	11.7181248	12.096435	11.7961202	11.7984783

2079	**ENSG0000009936**	FBXL19	protein_coding	chr16	30923055	30948783	+	Human GRCh	OTTHUMG000001	FBXL19	**F-box and leucine rich repeat protein 19**	Cytoplasm	enzyme		1.179692013	0	0.40674984	0	1	0	10.1226756	10.2265716	10.4731328	10.2314938
2080	**ENSG0000009936**	STX1B	protein_coding	chr16	30989256	31010638	-	Human GRCh	OTTHUMG000001	STX1B	**syntaxin 1B**	Plasma Mem	other		1.417990653	0	0.65609044	0	1	0	2.85747208	3.93160296	3.94861879	3.59241019
2081	**ENSG0000009937**	HSD3B7	protein_coding	chr16	30985207	30989147	+	Human GRCh	OTTHUMG000001	HSD3B7	**hydroxy-delta-5-steroid dehydrogenase, 3 beta- and steroid de**	Cytoplasm	enzyme		-1.063055083	0	0.85948322	0	1	0	10.0079908	10.2265502	10.1273188	9.97234622
2082	**ENSG0000009938**	SETD1A	protein_coding	chr16	30957294	30984664	+	Human GRCh	OTTHUMG000001	SETD1A	**SET domain containing 1A, histone lysine methyltransferase**	Nucleus	ion channel		-1.139088106	0	0.47801381	0	1	0	11.5033439	11.7768916	11.5293529	11.4813472
2083	**ENSG0000009938**	BCL7C	protein_coding	chr16	30833626	30894302	-	Human GRCh	OTTHUMG000001	BCL7C	**BAF chromatin remodeling complex subunit BCL7C**	Nucleus	other		1.219092614	0	0.33132786	0	1	0	9.33583345	9.74540806	9.84081353	9.70759157
2084	**ENSG0000009939**	MAGEB2	protein_coding	chrX	30215563	30220089	+	Human GRCh	OTTHUMG000000	MAGEB2	**MAGE family member B2**	Cytoplasm	other		-1.514226273	-1	0.79688623	0	1	0	1.04956055	3.12304053	1.98522467	2.33649709
2085	**ENSG0000009961**	EFNA2	protein_coding	chr19	1285873	1301431	+	Human GRCh	OTTHUMG000001	EFNA2	**ephrin A2**	Plasma Mem	kinase		-1.436244809	0	0.37038061	0	1	0	5.10472513	5.00287699	4.74871249	4.70365896
2086	**ENSG0000009962**	CIRBP	protein_coding	chr19	1259384	1274880	+	Human GRCh	OTTHUMG000001	CIRBP	**cold inducible RNA binding protein**	Nucleus	translation re		1.08917494	0	0.69370366	0	1	0	12.137353	12.3135753	12.4398673	12.1931469
2087	**ENSG0000009962**	ATP5F1D	protein_coding	chr19	1241746	1244825	+	Human GRCh	OTTHUMG000001	ATP5F1D	**ATP synthase F1 subunit delta**	Cytoplasm	transporter		1.092475244	0	0.69148975	0	1	0	11.5008848	11.8746882	11.7669617	11.8394814
2088	**ENSG0000009962**	CBARP	protein_coding	chr19	1228287	1238027	-	Human GRCh	OTTHUMG000001	CBARP	**CACN subunit beta associated regulatory protein**	Plasma Mem	other		1.409988344	0	0.26782713	0	1	0	8.05998487	8.64988573	8.75405813	8.80242343
2089	**ENSG0000009971**	PCDH11Y	protein_coding	chrY	5000226	5742224	+	Human GRCh	OTTHUMG000000	PCDH11Y	**protocadherin 11 Y-linked**	Other	other		1.288518974	0	0.86158906	0	NA	0	2.15683179	2.42891314	2.80513376	2.02150977
2091	**ENSG0000009976**	IGFALS	protein_coding	chr16	1790413	1794971	-	Human GRCh	OTTHUMG000001	IGFALS	**insulin like growth factor binding protein acid labile subunit**	Extracellular	other		-2.740412201	-1	0.1509948	0	1	0	4.57630431	4.37590914	3.85569207	3.45501203
2092	**ENSG0000009978**	HNRNPM	protein_coding	chr19	8444767	8489114	+	Human GRCh	OTTHUMG000001	HNRNPM	**heterogeneous nuclear ribonucleoprotein M**	Nucleus	other		1.053787906	0	0.78998015	0	1	0	12.3981297	12.6828137	12.725804	12.4754046
2093	**ENSG0000009978**	MARCHF2	protein_coding	chr19	8413270	8439017	+	Human GRCh	OTTHUMG000001	MARCHF2	**membrane associated ring-CH-type finger 2**	Cytoplasm	enzyme		-1.111175096	0	0.75037802	0	1	0	8.05325258	8.37688309	8.15546502	8.06407176
2094	**ENSG0000009979**	NDUFB7	protein_coding	chr19	14566078	14572066	-	Human GRCh	OTTHUMG000001	NDUFB7	**NADH:ubiquinone oxidoreductase subunit B7**	Cytoplasm	enzyme		1.037182417	0	0.85424045	0	1	0	9.89203473	10.1056058	10.0524532	10.0352398
2095	**ENSG0000009979**	TECR	protein_coding	chr19	14517085	14565980	+	Human GRCh	OTTHUMG000001	TECR	**trans-2,3-enoyl-CoA reductase**	Plasma Mem	enzyme		-1.141785962	0	0.52227275	0	1	0	11.5807128	11.8273273	11.6399147	11.4770201
2096	**ENSG0000009980**	TIMM13	protein_coding	chr19	2425625	2427586	-	Human GRCh	OTTHUMG000001	TIMM13	**translocase of inner mitochondrial membrane 13**	Cytoplasm	transporter		1.182762357	0	0.42085811	0	1	0	10.660869	10.9259496	11.0164822	10.9622121
2097	**ENSG0000009980**	CDC34	protein_coding	chr19	531760	542092	+	Human GRCh	OTTHUMG000001	CDC34	**cell division cycle 34, ubiqiutin conjugating enzyme**	Nucleus	enzyme		1.051911007	0	0.81843884	0	1	0	10.2952969	10.6270832	10.5114686	10.5464435
2098	**ENSG0000009981**	MTAP	protein_coding	chr9	21802636	21937651	+	Human GRCh	OTTHUMG000000	MTAP	**methylthioadenosine phosphorylase**	Nucleus	enzyme		1.055134417	0	0.81094159	0	1	0	10.9808037	11.0478601	11.2763542	10.8279384
2099	**ENSG0000009981**	MISP	protein_coding	chr19	751112	764318	+	Human GRCh	OTTHUMG000001	MISP	**mitotic spindle positioning**	Plasma Mem	other		-1.005093151	0	0.98945553	0	1	0	6.13761413	5.42432293	5.94615601	5.72389891
2100	**ENSG0000009981**	CEP170B	protein_coding	chr14	104865268	104896747	+	Human GRCh	OTTHUMG000001	CEP170B	**centrosomal protein 170B**	Other	other		-1.042448047	0	0.83753992	0	1	0	12.8650953	13.1474277	13.0809593	12.8432623
2101	**ENSG0000009981**	POLR2E	protein_coding	chr19	1086574	1095380	-	Human GRCh	OTTHUMG000001	POLR2E	**RNA polymerase II, I and III subunit E**	Nucleus	enzyme		1.019142125	0	0.92690328	0	1	0	11.5618329	11.8987705	11.7944991	11.7317236
2102	**ENSG0000009982**	POLRMT	protein_coding	chr19	617221	633537	-	Human GRCh	OTTHUMG000001	POLRMT	**RNA polymerase mitochondrial**	Cytoplasm	enzyme		1.082221427	0	0.69943376	0	1	0	11.5468003	11.8936721	11.903	11.7351543
2103	**ENSG0000009982**	HCN2	protein_coding	chr19	589881	617159	+	Human GRCh	OTTHUMG000001	HCN2	**hyperpolarization activated cyclic nucleotide gated potassium**	Plasma Mem	ion channel		1.074730781	0	0.80210706	0	1	0	6.28393893	6.2190786	6.4157889	6.22457962
2104	**ENSG0000009983**	CDHR5	protein_coding	chr11	616577	626078	-	Human GRCh	OTTHUMG000001	CDHR5	**cadherin related family member 5**	Plasma Mem	other		-4.955126715	-1	0.06115074	0	1	0	2.81230065	3.69890015	2.22777065	2.81850143
2105	**ENSG0000009984**	IZUMO4	protein_coding	chr19	2096429	2099593	+	Human GRCh	OTTHUMG000001	IZUMO4	**IZUMO family member 4**	Nucleus	other		2.080316717	1	0.08751034	0	1	0	7.05347011	7.60353387	8.17381804	8.19878632
2106	**ENSG0000009984**	RASSF7	protein_coding	chr11	560404	564025	+	Human GRCh	OTTHUMG000001	RASSF7	**Ras association domain family member 7**	Cytoplasm	other		1.271317908	0	0.35837365	0	1	0	9.33785831	9.48136075	9.78194603	9.57191768
2107	**ENSG0000009986**	GADD45B	protein_coding	chr19	2476122	2478259	+	Human GRCh	OTTHUMG000001	GADD45B	**growth arrest and DNA damage inducible beta**	Cytoplasm	other		-1.095002803	0	0.76380959	0	1	0	8.92009612	8.86998967	8.784848	8.79502455
2108	**ENSG0000009986**	PALM	protein_coding	chr19	708935	748329	+	Human GRCh	OTTHUMG000001	PALM	**paralemmin**	Plasma Mem	other		-1.186483759	0	0.67409502	0	1	0	7.20925519	7.65574622	7.64264954	6.70581452
2109	**ENSG0000009986**	MADCAM1	protein_coding	chr19	489176	505343	+	Human GRCh	OTTHUMG000001	MADCAM1	**mucosal vascular addressin cell adhesion molecule 1**	Plasma Mem	other	SHP647	-1.058546798	0	0.89552303	0	1	0	5.33050441	5.07469641	5.30852361	4.99139451
2111	**ENSG0000009987**	MKNK2	protein_coding	chr19	2037465	2051244	-	Human GRCh	OTTHUMG000001	MKNK2	**MAPK interacting serine/threonine kinase 2**	Cytoplasm	kinase	SEL201, to	1.208416225	0	0.40619777	0	1	0	11.9159665	12.24209	12.2340227	12.3663
2112	**ENSG0000009988**	ARVCF	protein_coding	chr22	19969896	20016823	-	Human GRCh	OTTHUMG000000	ARVCF	**ARVCF delta catenin family member**	Plasma Mem	other		1.051445493	0	0.81776519	0	1	0	10.7189961	10.8617908	10.9490041	10.7394316
2113	**ENSG0000009989**	TRMT2A	protein_coding	chr22	20111875	20117392	-	Human GRCh	OTTHUMG000001	TRMT2A	**tRNA methyltransferase 2 homolog A**	Cytoplasm	kinase		1.109752452	0	0.59500106	0	1	0	10.1520972	10.462697	10.5719181	10.2790194
2114	**ENSG0000009990**	RANBP1	protein_coding	chr22	20115938	20127355	+	Human GRCh	OTTHUMG000001	RANBP1	**RAN binding protein 1**	Nucleus	other		-1.060674922	0	0.76678817	0	1	0	11.4536152	11.8835352	11.7114638	11.5247932
2115	**ENSG0000009990**	ZDHHC8	protein_coding	chr22	20129456	20148007	+	Human GRCh	OTTHUMG000001	ZDHHC8	**zinc finger DHHC-type palmitoyltransferase 8**	Cytoplasm	enzyme		1.032747575	0	0.88268463	0	1	0	10.8246787	11.1971856	11.1913004	10.9169139
2116	**ENSG0000009991**	KLHL22	protein_coding	chr22	20441519	20495844	-	Human GRCh	OTTHUMG000001	KLHL22	**kelch like family member 22**	Cytoplasm	other		1.062459452	0	0.77838278	0	1	0	10.1484831	10.4269526	10.5441478	10.1504991
2117	**ENSG0000009991**	MED15	protein_coding	chr22	20495913	20587632	+	Human GRCh	OTTHUMG000001	MED15	**mediator complex subunit 15**	Nucleus	transcription		1.056036303	0	0.78872137	0	1	0	11.4169114	11.8232408	11.7947151	11.5951025
2118	**ENSG0000009993**	SERPIND1	protein_coding	chr22	20774113	20787720	+	Human GRCh	OTTHUMG000001	SERPIND1	**serpin family D member 1**	Extracellular	other	ardeparin,	1.129192909	0	0.94748685	0	NA	0	1.02375918	0.68154464	1.07774693	0.67305925
2119	**ENSG0000009994**	SNAP29	protein_coding	chr22	20859007	20891214	+	Human GRCh	OTTHUMG000001	SNAP29	**synaptosome associated protein 29**	Cytoplasm	other		-1.095376471	0	0.68151241	0	1	0	10.0589797	10.4488055	10.2359822	10.0943432
2120	**ENSG0000009994**	CRKL	protein_coding	chr22	20917407	20953747	+	Human GRCh	OTTHUMG000001	CRKL	**CRK like proto-oncogene, adaptor protein**	Cytoplasm	kinase		-1.072851848	0	0.69419765	0	1	0	11.8168067	12.095207	11.9694588	11.7946373
2121	**ENSG0000009994**	LZTR1	protein_coding	chr22	20982269	20999032	+	Human GRCh	OTTHUMG000001	LZTR1	**leucine zipper like post translational regulator 1**	Cytoplasm	transcription		1.044609443	0	0.83831443	0	1	0	11.0060014	11.3553884	11.4161528	11.0402467
2122	**ENSG0000009995**	MMP11	protein_coding	chr22	23768226	23784316	+	Human GRCh	OTTHUMG000001	MMP11	**matrix metallopeptidase 11**	Extracellular	peptidase	marimasta	-1.09590877	0	0.75842309	0	1	0	7.16481138	7.16666117	7.08270875	7.05114081
2123	**ENSG0000009995**	CECR2	protein_coding	chr22	17359949	17558151	+	Human GRCh	OTTHUMG000001	CECR2	**CECR2 histone acetyl-lysine reader**	Nucleus	other		-1.516703893	-1	0.42844491	0	1	0	9.72905919	9.58126551	9.31803091	9.08883859
2124	**ENSG0000009995**	SMARCB1	protein_coding	chr22	23786931	23838009	+	Human GRCh	OTTHUMG000001	SMARCB1	**SWI/SNF related, matrix associated, actin dependent regulato**	Nucleus	transcription		-1.141142668	0	0.52634813	0	1	0	11.725234	12.0057199	11.8502885	11.5877405
2125	**ENSG0000009995**	P2RX6	protein_coding	chr22	21009808	21028013	+	Human GRCh	OTTHUMG000001	P2RX6	**purinergic receptor P2X 6**	Plasma Mem	ion channel		-2.03769616	-1	0.28514222	0	1	0	2.56604668	3.75635819	3.32543729	2.02101747
2126	**ENSG0000009995**	DERL3	protein_coding	chr22	23834503	23839128	-	Human GRCh	OTTHUMG000001	DERL3	**derlin 3**	Cytoplasm	other		-1.114952706	0	0.73244473	0	1	0	11.0964949	11.4285059	11.0785649	11.2063155
2127	**ENSG0000009996**	SLC7A4	protein_coding	chr22	21028718	21032840	-	Human GRCh	OTTHUMG000001	SLC7A4	**solute carrier family 7 member 4**	Plasma Mem	transporter		1	0	1	0	1	0	0.14660785	0.24323371	0.22793176	0.1525611
2128	**ENSG0000009996**	BCL2L13	protein_coding	chr22	17628855	17730855	+	Human GRCh	OTTHUMG000001	BCL2L13	**BCL2 like 13**	Cytoplasm	other		-1.227014353	0	0.33739517	0	1	0	10.8406398	10.9561674	10.8018306	10.5194788
2129	**ENSG0000009997**	DDTL	protein_coding	chr22	23966888	23972556	+	Human GRCh	OTTHUMG000001	DDTL	**D-dopachrome tautomerase like**	Cytoplasm	other		-1.042810914	0	0.93381525	0	1	0	3.72049236	3.55449825	3.60647961	3.6084875
2130	**ENSG0000009997**	DDT	protein_coding	chr22	23970542	23980525	-	Human GRCh	OTTHUMG000001	DDT	**D-dopachrome tautomerase**	Cytoplasm	enzyme		-1.036674547	0	0.87035708	0	1	0	10.1678489	10.5495745	10.4107171	10.2493406
2131	**ENSG0000009998**	GSTT2	protein_coding	chr22	23980058	23983915	+	Human GRCh	OTTHUMG000001	GSTT2/GSTT2	**glutathione S-transferase theta 2B**	Cytoplasm	enzyme		1	0	1	0	1	0	0.75501108	0.82194167	0.8112007	0.75907311
2133	**ENSG0000009999**	CABIN1	protein_coding	chr22	24011192	24178628	+	Human GRCh	OTTHUMG000001	CABIN1	**calcineurin binding protein 1**	Nucleus	other		-1.074125465	0	0.70599516	0	1	0	11.9769977	12.2303715	12.148968	11.8993742
2134	**ENSG0000009999**	TBC1D10A	protein_coding	chr22	30291990	30326947	-	Human GRCh	OTTHUMG000001	TBC1D10A	**TBC1 domain family member 10A**	Plasma Mem	other		-1.017552245	0	0.94334172	0	1	0	8.95368872	9.20071628	9.16571973	8.95335437
2135	**ENSG0000009999**	SUSD2	protein_coding	chr22	24181487	24189106	+	Human GRCh	OTTHUMG000001	SUSD2	**sushi domain containing 2**	Extracellular	other		-1.091985304	0	0.85770046	0	1	0	7.37776118	7.94447251	7.3869805	7.79142806
2136	**ENSG0000009999**	SF3A1	protein_coding	chr22	30331988	30356919	-	Human GRCh	OTTHUMG000001	SF3A1	**splicing factor 3a subunit 1**	Nucleus	other		-1.124727266	0	0.54584197	0	1	0	12.3389012	12.7157107	12.4699101	12.3477086
2137	**ENSG0000009999**	GGT5	protein_coding	chr22	24219654	24245108	-	Human GRCh	OTTHUMG000001	GGT5	**gamma-glutamyltransferase 5**	Plasma Mem	enzyme		18.49115302	1	0.00947994	1	1	0	2.16474983	2.2565326	2.57544576	3.3569787
2138	**ENSG0000009999**	RNF215	protein_coding	chr22	30368811	30421771	-	Human GRCh	OTTHUMG000001	RNF215	**ring finger protein 215**	Other	other		-1.371767976	0	0.35171307	0	1	0	9.41002313	9.71227395	9.32922265	9.10490366
2139	**ENSG0000010000**	SEC14L2	protein_coding	chr22	30396941	30425303	+	Human GRCh	OTTHUMG000001	SEC14L2	**SEC14 like lipid binding 2**	Cytoplasm	transporter		-1.082439303	0	0.89844187	0	1	0	8.28447584	8.31581975	8.26870171	8.15843088
2141	**ENSG0000010001**	SPECC1L	protein_coding	chr22	24270817	24417739	+	Human GRCh	OTTHUMG000001	SPECC1L	**sperm antigen with calponin homology and coiled-coil domain**	Extracellular	other		-1.119965086	0	0.58190142	0	1	0	11.5670981	11.7416463	11.6638609	11.3841431
2142	**ENSG0000010002**	PPIL2	protein_coding	chr22	21666000	21700015	+	Human GRCh	OTTHUMG000001	PPIL2	**peptidylprolyl isomerase like 2**	Nucleus	enzyme		-1.005191399	0	0.9803218	0	1	0	11.4963076	11.8048715	11.7494958	11.5518912
2143	**ENSG0000010002**	UPB1	protein_coding	chr22	24494107	24528390	+	Human GRCh	OTTHUMG000001	UPB1	**beta-ureidopropionase 1**	Cytoplasm	enzyme		-2.905690089	-1	0.10221314	0	1	0	4.05573099	4.24583316	3.52780296	3.03241412
2144	**ENSG0000010002**	YPEL1	protein_coding	chr22	21697536	21735794	-	Human GRCh	OTTHUMG000001	YPEL1	**yippee like 1**	Nucleus	enzyme		-1.315281342	0	0.34181404	0	1	0	9.73039964	10.0107718	9.8076916	9.29233525
2145	**ENSG0000010002**	SNRPD3	protein_coding	chr22	24555958	24582052	+	Human GRCh	OTTHUMG000001	SNRPD3	**small nuclear ribonucleoprotein D3 polypeptide**	Nucleus	other		-1.04353827	0	0.85011836	0	1	0	11.7254172	12.0691187	11.9175263	11.8055949
2146	**ENSG0000010002**	PES1	protein_coding	chr22	30576625	30607083	-	Human GRCh	OTTHUMG000001	PES1	**pescadillo ribosomal biogenesis factor 1**	Nucleus	other		-1.027508289	0	0.87984559	0	1	0	11.0891939	11.4680684	11.3421464	11.1776505
2147	**ENSG0000010003**	MAPK1	protein_coding	chr22	21759657	21867680	-	Human GRCh	OTTHUMG000000	MAPK1	**mitogen-activated protein kinase 1**	Cytoplasm	kinase	pexmetinil	-1.070042139	0	0.76397053	0	1	0	11.8912072	12.2872723	12.1487359	11.8981958
2148	**ENSG0000010003**	GGT1	protein_coding	chr22	24594811	24629005	+	Human GRCh	OTTHUMG000000	GGT1	**gamma-glutamyltransferase 1**	Plasma Mem	enzyme		-1.104461979	0	0.74168377	0	1	0	8.67439199	8.66409062	8.54675073	8.57818725
2150	**ENSG0000010003**	PPM1F	protein_coding	chr22	21919425	21952848	-	Human GRCh	OTTHUMG000001	PPM1F	**protein phosphatase, Mg2+/Mn2+ dependent 1F**	Cytoplasm	phosphatase		1.104845347	0	0.64267798	0	1	0	9.90484218	10.0696134	10.2571649	9.92216427
2151	**ENSG0000010003**	SLC35E4	protein_coding	chr22	30635781	30669016	+	Human GRCh	OTTHUMG000001	SLC35E4	**solute carrier family 35 member E4**	Other	other		1.738739712	1	0.10440709	0	1	0	5.76135596	5.68362983	6.3147776	6.21016005
2152	**ENSG0000010003**	TOP3B	protein_coding	chr22	21957025	21982813	-	Human GRCh	OTTHUMG000001	TOP3B	**DNA topoisomerase III beta**	Nucleus	enzyme		-1.095034469	0	0.69340943	0	1	0	10.0118973	10.3476685	10.2469528	9.90922872
2153	**ENSG0000010005**	CRYBB3	protein_coding	chr22	25199858	25207359	+	Human GRCh	OTTHUMG000001	CRYBB3	**crystallin beta B3**	Other	other		1.171361724	0	0.81586123	0	1	0	4.37768925	3.83987843	4.6727206	3.63678266
2155	**ENSG0000010005**	ESS2	protein_coding	chr22	19130279	19144684	-	Human GRCh	OTTHUMG000001	ESS2	**ess-2 splicing factor homolog**	Nucleus	enzyme		-1.008751507	0	0.96830058	0	1	0	9.65275054	9.85867662	9.89001011	9.59221706
2156	**ENSG0000010006**	MFNG	protein_coding	chr22	37469063	37486393	-	Human GRCh	OTTHUMG000001	MFNG	**MFNG O-fucosylpeptide 3-beta-N-acetylglucosaminyltransfera**	Cytoplasm	enzyme		-65.23857501	-1	0.00016455	1	0.24	0	3.96913902	3.87310117	2.4237338	2.37835829
2157	**ENSG0000010006**	CARD10	protein_coding	chr22	37490362	37519542	-	Human GRCh	OTTHUMG000001	CARD10	**caspase recruitment domain family member 10**	Cytoplasm	other		1.345127858	0	0.24860004	0	1	0	6.94944215	7.28507753	7.36890595	7.47373391
2158	**ENSG0000010007**	SLC25A1	protein_coding	chr22	19175581	19178739	-	Human GRCh	OTTHUMG000001	SLC25A1	**solute carrier family 25 member 1**	Cytoplasm	transporter		1.101540754	0	0.67106665	0	1	0	10.9443468	11.3281927	11.1829683	11.3348693
2159	**ENSG0000010007**	GRK3	protein_coding	chr22	25564675	25729294	+	Human GRCh	OTTHUMG000001	GRK3	**G protein-coupled receptor kinase 3**	Cytoplasm	kinase	3-({[4-met	-1.052855937	0	0.83850427	0	1	0	8.84828559	9.17162751	8.95494037	8.97792443
2160	**ENSG0000010007**	PLA2G3	protein_coding	chr22	31134807	31140508	-	Human GRCh	OTTHUMG000001	PLA2G3	**phospholipase A2 group III**	Extracellular	enzyme		-1.160594174	0	0.70587282	0	1	0	8.78915389	9.37680642	8.935772	8.9599858
2162	**ENSG0000010008**	GGA1	protein_coding	chr22	37608725	37633564	+	Human GRCh	OTTHUMG000000	GGA1	**golgi associated, gamma adaptin ear containing, ARF binding p**	Cytoplasm	transporter		-1.044549232	0	0.84850248	0	1	0	11.2357417	11.5448505	11.484681	11.2016731
2163	**ENSG0000010008**	HIRA	protein_coding	chr22	19330698	19447450	-	Human GRCh	OTTHUMG000001	HIRA	**histone cell cycle regulator**	Nucleus	transcription		1.052323746	0	0.82019579	0	1	0	8.50529366	8.74975765	8.84310085	8.51477982
2164	**ENSG0000010009**	SH3BP1	protein_coding	chr22	37634654	37656117	+	Human GRCh	OTTHUMG000000	SH3BP1	**SH3 domain binding protein 1**	Plasma Mem	other		-1.027441063	0	0.9109266	0	1	0	9.21444606	9.62783648	9.35042437	9.46397566
2165	**ENSG0000010009**	SEZ6L	protein_coding	chr22	26169462	26383597	+	Human GRCh	OTTHUMG000001	SEZ6L	**seizure related 6 homolog like**	Plasma Mem	other		10.63476691	1	0.03951575	1	1	0	2.55909431	2.66851569	2.65094501	3.43285335
2166	**ENSG0000010009**	LGALS1	protein_coding	chr22	37675636	37679802	+	Human GRCh	OTTHUMG000001	LGALS1	**galectin 1**	Extracellular	other	OTX008	-1.010665225	0	0.98139513	0	1	0	7.7821496	8.41471427	8.03373335	8.21357652
2167	**ENSG0000010009**	HPS4	protein_coding	chr22	26443107	26483931	-	Human GRCh	OTTHUMG000000	HPS4	**HPS4 biogenesis of lysosomal organelles complex 3 subunit 2**	Cytoplasm	other		-1.145800606	0	0.50783166	0	1	0	10.9133841	11.0752697	11.0077406	10.6594404
2168	**ENSG0000010010**	PIK3IP1	protein_coding	chr22	31281594	31292534	-	Human GRCh	OTTHUMG000001	PIK3IP1	**phosphoinositide-3-kinase interacting protein 1**	Cytoplasm	other		-1.218212106	0	0.64249264	0	1	0	6.56452386	7.45711298	6.85217597	6.91957398

2169	**ENSG0000010010**	SRRD	protein_coding	chr22	26483877	26494658	+	Human GRCh	OTTHUMG000001	SRRD	**SRR1 domain containing**	Cytoplasm	other		-1.06658839	0	0.76910365	0	1	0	9.74853251	10.0140143	9.93205584	9.68820987
2170	**ENSG0000010010**	PATZ1	protein_coding	chr22	31325804	31346346	-	Human GRCh	OTTHUMG000001	PATZ1	**POZ/BTB and AT hook containing zinc finger 1**	Nucleus	transcription		1.096404648	0	0.67654159	0	1	0	10.7920635	11.1495276	11.187917	10.9757403
2171	**ENSG0000010010**	TRIOBP	protein_coding	chr22	37697048	37776556	+	Human GRCh	OTTHUMG000001	TRIOBP	**TRIO and F-actin binding protein**	Nucleus	other		-1.212656119	0	0.36115534	0	1	0	11.059902	11.4119594	11.0677913	11.0024765
2172	**ENSG0000010010**	TFIP11	protein_coding	chr22	26491225	26512505	-	Human GRCh	OTTHUMG000001	TFIP11	**tuftelin interacting protein 11**	Extracellular	other		-1.097895956	0	0.65467327	0	1	0	9.11312184	9.28854473	9.18860021	9.00690238
2173	**ENSG0000010011**	GCAT	protein_coding	chr22	37807905	37817176	+	Human GRCh	OTTHUMG000001	GCAT	**glycine C-acetyltransferase**	Cytoplasm	enzyme		1.02958814	0	0.92294295	0	1	0	9.41733138	9.63720876	9.72847145	9.3789907
2176	**ENSG0000010012**	ANKRD54	protein_coding	chr22	37830855	37849327	-	Human GRCh	OTTHUMG000001	ANKRD54	**ankyrin repeat domain 54**	Nucleus	transcription		-1.129632429	0	0.58381719	0	1	0	10.2149778	10.4718526	10.3370431	10.0721517
2177	**ENSG0000010012**	EIF3L	protein_coding	chr22	37848868	37889407	+	Human GRCh	OTTHUMG000001	EIF3L	**eukaryotic translation initiation factor 3 subunit L**	Cytoplasm	translation re		-1.134041555	0	0.60032286	0	1	0	11.9004834	12.2592373	12.153157	11.7013297
2178	**ENSG0000010013**	SNU13	protein_coding	chr22	41673933	41690504	-	Human GRCh	OTTHUMG000001	SNU13	**small nuclear ribonucleoprotein 13**	Nucleus	other		-1.005747575	0	0.97832612	0	1	0	11.2830537	11.6673152	11.6008241	11.3546029
2179	**ENSG0000010013**	MICALL1	protein_coding	chr22	37905657	37942822	+	Human GRCh	OTTHUMG000001	MICALL1	**MICAL like 1**	Cytoplasm	other		-1.112237406	0	0.57359001	0	1	0	10.2407352	10.3355914	10.2807205	10.0488033
2180	**ENSG0000010014**	POLR2F	protein_coding	chr22	37952607	38041915	+	Human GRCh	OTTHUMG000001	POLR2F	**RNA polymerase II, I and III subunit F**	Nucleus	enzyme		1.096784714	0	0.67621075	0	1	0	8.98332907	9.52126605	9.55828793	9.18271067
2182	**ENSG0000010014**	CCDC134	protein_coding	chr22	41800679	41832164	+	Human GRCh	OTTHUMG000001	CCDC134	**coiled-coil domain containing 134**	Cytoplasm	other		-1.068221639	0	0.75302777	0	1	0	10.910201	11.2628622	11.1372088	10.9021808
2183	**ENSG0000010015**	DEPDC5	protein_coding	chr22	31753867	31908033	+	Human GRCh	OTTHUMG000000	DEPDC5	**DEP domain containing 5, GATOR1 subcomplex subunit**	Cytoplasm	other		-1.07601658	0	0.72435533	0	1	0	10.1834047	10.4238708	10.3594907	10.0826682
2184	**ENSG0000010015**	PICK1	protein_coding	chr22	38056311	38075701	+	Human GRCh	OTTHUMG000001	PICK1	**protein interacting with PRKCA 1**	Cytoplasm	enzyme		1.065505638	0	0.74850234	0	1	0	10.4414113	10.5414821	10.6651472	10.4527461
2185	**ENSG0000010015**	TTC28	protein_coding	chr22	27978014	28679840	-	Human GRCh	OTTHUMG000001	TTC28	**tetratricopeptide repeat domain 28**	Cytoplasm	other		-1.046064437	0	0.89836948	0	1	0	8.45403467	8.41935078	8.56791227	8.17578311
2186	**ENSG0000010015**	SLC16A8	protein_coding	chr22	38078134	38084184	-	Human GRCh	OTTHUMG000001	SLC16A8	**solute carrier family 16 member 8**	Plasma Mem	transporter		1.253395941	0	0.68306891	0	1	0	5.17687723	5.93768521	5.97210653	5.73706323
2187	**ENSG0000010016**	CENPM	protein_coding	chr22	41938737	41947152	-	Human GRCh	OTTHUMG000001	CENPM	**centromere protein M**	Cytoplasm	other		-1.142178806	0	0.57262173	0	1	0	9.66843024	9.93099293	9.69610615	9.622203
2188	**ENSG0000010016**	SEPTIN3	protein_coding	chr22	41969443	41998221	+	Human GRCh	OTTHUMG000000	SEPTIN3	**septin 3**	Cytoplasm	enzyme		1.131728939	0	0.57354608	0	1	0	9.32087889	9.38644663	9.55971084	9.40904106
2189	**ENSG0000010017**	SLC5A1	protein_coding	chr22	32043261	32113029	+	Human GRCh	OTTHUMG000000	SLC5A1	**solute carrier family 5 member 1**	Plasma Mem	transporter	YG1699, sc	-3.15075627	-1	0.47841	0	NA	0	1.24715511	1.60934475	0.93697595	1.2520075
2191	**ENSG0000010019**	KDELR3	protein_coding	chr22	38468078	38483447	+	Human GRCh	OTTHUMG000001	KDELR3	**KDEL endoplasmic reticulum protein retention receptor 3**	Cytoplasm	transporter		-1.351488448	0	0.37657027	0	1	0	7.37813605	7.75769649	7.33898118	7.13380303
2192	**ENSG0000010019**	CYP2D6	protein_coding	chr22	42126499	42130865	-	Human GRCh	OTTHUMG000001	CYP2D6	**cytochrome P450 family 2 subfamily D member 6**	Cytoplasm	enzyme	quinidine,	-1.907007941	-1	0.09473615	0	1	0	6.91237163	7.11624293	6.31297961	6.37823604
2193	**ENSG0000010020**	DDX17	protein_coding	chr22	38483438	38507660	-	Human GRCh	OTTHUMG000001	DDX17	**DEAD-box helicase 17**	Nucleus	enzyme		-1.184415997	0	0.40574245	0	1	0	14.2119306	14.4337662	14.2753447	13.9863954
2194	**ENSG0000010020**	DMC1	protein_coding	chr22	38518948	38570204	-	Human GRCh	OTTHUMG000001	DMC1	**DNA meiotic recombinase 1**	Nucleus	enzyme		-1.079667057	0	0.82135849	0	1	0	7.20195194	7.31705779	7.39727695	6.90569225
2195	**ENSG0000010020**	TCF20	protein_coding	chr22	42160013	42343616	-	Human GRCh	OTTHUMG000001	TCF20	**transcription factor 20**	Nucleus	transcription		-1.149381104	0	0.44282134	0	1	0	12.2271815	12.5132589	12.3422881	12.091062
2196	**ENSG0000010020**	HSCB	protein_coding	chr22	28742039	28757515	+	Human GRCh	OTTHUMG000001	HSCB	**HscB mitochondrial iron-sulfur cluster cochaperone**	Cytoplasm	other		-1.124380542	0	0.62738803	0	1	0	7.70410179	8.07742646	7.9906673	7.51862186
2197	**ENSG0000010021**	CBY1	protein_coding	chr22	38656636	38673854	+	Human GRCh	OTTHUMG000001	CBY1	**chibby 1, beta catenin antagonist**	Cytoplasm	other		-1.153765869	0	0.49540275	0	1	0	9.34079563	9.4857078	9.27321449	9.23525537
2198	**ENSG0000010021**	TOMM22	protein_coding	chr22	38681957	38685421	+	Human GRCh	OTTHUMG000001	TOMM22	**translocase of outer mitochondrial membrane 22**	Cytoplasm	transporter		-1.105465222	0	0.6135238	0	1	0	11.1333012	11.4668682	11.3376388	11.0393484
2200	**ENSG0000010021**	XBP1	protein_coding	chr22	28794555	28800597	-	Human GRCh	OTTHUMG000001	XBP1	**X-box binding protein 1**	Nucleus	transcription		-1.148825136	0	0.52094267	0	1	0	11.6115436	11.7169273	11.6518447	11.345408
2201	**ENSG0000010022**	RTCB	protein_coding	chr22	32387582	32412248	-	Human GRCh	OTTHUMG000000	RTCB	**RNA 2',3'-cyclic phosphate and 5'-OH ligase**	Cytoplasm	enzyme		-1.070987183	0	0.77108227	0	1	0	10.2413517	10.6786706	10.4553429	10.3466513
2202	**ENSG0000010022**	JOSD1	protein_coding	chr22	38685543	38701556	-	Human GRCh	OTTHUMG000001	JOSD1	**Josephin domain containing 1**	Cytoplasm	peptidase		-1.047800938	0	0.80399984	0	1	0	10.3865294	10.6247322	10.6367285	10.2508977
2203	**ENSG0000010022**	FBXO7	protein_coding	chr22	32474676	32498829	+	Human GRCh	OTTHUMG000000	FBXO7	**F-box protein 7**	Cytoplasm	enzyme		-1.124457384	0	0.5263188	0	1	0	10.697622	10.9157126	10.7900711	10.5628675
2204	**ENSG0000010022**	GTPBP1	protein_coding	chr22	38705742	38738299	+	Human GRCh	OTTHUMG000001	GTPBP1	**GTP binding protein 1**	Cytoplasm	enzyme		-1.14375455	0	0.53689091	0	1	0	11.9468204	12.2757111	12.0625144	11.8697031
2205	**ENSG0000010022**	POLDIP3	protein_coding	chr22	42583721	42614962	-	Human GRCh	OTTHUMG000001	POLDIP3	**DNA polymerase delta interacting protein 3**	Nucleus	other		-1.02310005	0	0.90445989	0	1	0	10.8934587	11.2244282	11.1919043	10.8772331
2206	**ENSG0000010022**	RAB36	protein_coding	chr22	23145366	23165663	+	Human GRCh	OTTHUMG000001	RAB36	**RAB36, member RAS oncogene family**	Cytoplasm	enzyme		-1.204601326	0	0.41372004	0	1	0	9.18117344	9.53743088	9.34001143	8.9546946
2207	**ENSG0000010023**	TIMP3	protein_coding	chr22	32801705	32863041	+	Human GRCh	OTTHUMG000000	TIMP3	**TIMP metallopeptidase inhibitor 3**	Extracellular	other		1.877670899	1	0.11007354	0	1	0	6.4439696	6.2853627	7.16259344	7.01447445
2208	**ENSG0000010023**	PPP6R2	protein_coding	chr22	50343304	50445090	+	Human GRCh	OTTHUMG000001	PPP6R2	**protein phosphatase 6 regulatory subunit 2**	Cytoplasm	other		-1.0149756	0	0.94318951	0	1	0	11.0621435	11.4006851	11.3105956	11.1364994
2209	**ENSG0000010024**	SBF1	protein_coding	chr22	50443219	50483923	-	Human GRCh	OTTHUMG000001	SBF1	**SET binding factor 1**	Plasma Mem	phosphatase		-1.042667654	0	0.83761364	0	1	0	12.7768094	12.9677728	12.9141874	12.7378946
2210	**ENSG0000010024**	SUN2	protein_coding	chr22	38733290	38794143	-	Human GRCh	OTTHUMG000001	SUN2	**Sad1 and UNC84 domain containing 2**	Nucleus	other		-1.053233895	0	0.79708964	0	1	0	11.5669411	11.7821148	11.7011064	11.5383895
2211	**ENSG0000010024**	CYB5R3	protein_coding	chr22	42615730	42720870	-	Human GRCh	OTTHUMG000001	CYB5R3	**cytochrome b5 reductase 3**	Cytoplasm	enzyme		1.105058505	0	0.57070101	0	1	0	11.073947	11.3072382	11.3083767	11.3077621
2212	**ENSG0000010024**	DNAL4	protein_coding	chr22	38778508	38794198	-	Human GRCh	OTTHUMG000001	DNAL4	**dynein axonemal light chain 4**	Cytoplasm	enzyme		-1.147216886	0	0.53888985	0	1	0	8.7645878	8.7429737	8.75539258	8.43161574
2214	**ENSG0000010025**	MIOX	protein_coding	chr22	50486784	50490648	+	Human GRCh	OTTHUMG000001	MIOX	**myo-inositol oxygenase**	Cytoplasm	enzyme		1.981662549	1	0.3332297	0	1	0	2.89567989	3.02571841	3.45800283	3.33669315
2215	**ENSG0000010025**	LMF2	protein_coding	chr22	50502949	50507702	-	Human GRCh	OTTHUMG000001	LMF2	**lipase maturation factor 2**	Other	other		1.088309054	0	0.69269098	0	1	0	11.1784724	11.365974	11.5173105	11.2039889
2216	**ENSG0000010026**	RHBDD3	protein_coding	chr22	29259872	29268209	-	Human GRCh	OTTHUMG000001	RHBDD3	**rhomboid domain containing 3**	Other	other		1.08186819	0	0.7340113	0	1	0	9.51557805	9.65745404	9.88236533	9.42515797
2217	**ENSG0000010026**	PACSIN2	protein_coding	chr22	42835412	43015149	-	Human GRCh	OTTHUMG000001	PACSIN2	**protein kinase C and casein kinase substrate in neurons 2**	Cytoplasm	other		-1.004048327	0	0.984463	0	1	0	11.0248887	11.299549	11.2501777	11.0745058
2218	**ENSG0000010027**	TTLL1	protein_coding	chr22	43039516	43089419	-	Human GRCh	OTTHUMG000001	TTLL1	**TTL family tubulin polyglutamylase complex subunit L1**	Extracellular	enzyme		-1.365453698	0	0.34603202	0	1	0	6.15606073	6.05832591	5.75942916	5.84927925
2219	**ENSG0000010027**	RASL10A	protein_coding	chr22	29312933	29319679	-	Human GRCh	OTTHUMG000001	RASL10A	**RAS like family 10 member A**	Plasma Mem	enzyme		1.093200246	0	0.88306539	0	1	0	7.24909358	7.52187621	7.76505133	7.14587761
2220	**ENSG0000010028**	AP1B1	protein_coding	chr22	29327680	29388583	-	Human GRCh	OTTHUMG000001	AP1B1	**adaptor related protein complex 1 subunit beta 1**	Cytoplasm	other		1.098654442	0	0.63939128	0	1	0	11.1742696	11.4216988	11.5261124	11.2801333
2221	**ENSG0000010028**	HMGXB4	protein_coding	chr22	35257452	35295807	+	Human GRCh	OTTHUMG000001	HMGXB4	**HMG-box containing 4**	Nucleus	other		-1.154700547	0	0.46963403	0	1	0	10.3134612	10.5498965	10.3639891	10.1894509
2222	**ENSG0000010028**	TOM1	protein_coding	chr22	35299275	35347992	+	Human GRCh	OTTHUMG000001	TOM1	**target of myb1 membrane trafficking protein**	Cytoplasm	transporter		-1.049856231	0	0.83368117	0	1	0	9.86131719	10.1953267	10.0780725	9.88437132
2223	**ENSG0000010028**	NEFH	protein_coding	chr22	29480218	29491390	+	Human GRCh	OTTHUMG000001	NEFH	**neurofilament heavy chain**	Cytoplasm	other		2.292144027	1	0.23271487	0	1	0	4.47789749	4.67946635	5.34654248	5.48759796
2224	**ENSG0000010028**	CHKB	protein_coding	chr22	50578959	50601455	-	Human GRCh	OTTHUMG000001	CHKB	**choline kinase beta**	Cytoplasm	kinase		-1.098354473	0	0.66041262	0	1	0	10.1744361	10.3517579	10.263758	10.0510489
2225	**ENSG0000010029**	BIK	protein_coding	chr22	43110750	43129712	+	Human GRCh	OTTHUMG000001	BIK	**BCL2 interacting killer**	Cytoplasm	other		1.209827611	0	0.68008613	0	1	0	5.49907597	4.81802032	5.3309835	5.44369765
2226	**ENSG0000010029**	HMOX1	protein_coding	chr22	35380361	35394214	+	Human GRCh	OTTHUMG000001	HMOX1	**heme oxygenase 1**	Cytoplasm	enzyme	tin mesopo	1.135986765	0	0.71992835	0	1	0	6.9040335	6.65023781	6.87875673	6.95837182
2227	**ENSG0000010029**	MCAT	protein_coding	chr22	43132209	43143398	-	Human GRCh	OTTHUMG000001	MCAT	**malonyl-CoA-acyl carrier protein transacylase**	Cytoplasm	enzyme		1.025296178	0	0.90150113	0	1	0	9.37891546	9.54908757	9.6279405	9.3457177
2228	**ENSG0000010029**	THOC5	protein_coding	chr22	29505879	29555216	-	Human GRCh	OTTHUMG000001	THOC5	**THO complex subunit 5**	Cytoplasm	other		-1.158079264	0	0.53521856	0	1	0	11.8265852	12.2393577	12.0482002	11.6927803
2229	**ENSG0000010029**	MCM5	protein_coding	chr22	35400134	35425431	+	Human GRCh	OTTHUMG000001	MCM5	**minichromosome maintenance complex component 5**	Nucleus	enzyme		-1.182421537	0	0.43087133	0	1	0	12.0614448	12.4784547	12.1693073	12.0265589
2231	**ENSG0000010029**	ARSA	protein_coding	chr22	50622754	50628173	-	Human GRCh	OTTHUMG000001	ARSA	**arylsulfatase A**	Cytoplasm	enzyme		1.030989401	0	0.91031231	0	1	0	9.85090771	10.1847863	10.1664959	9.95057133
2232	**ENSG0000010030**	TSPO	protein_coding	chr22	43151547	43163242	+	Human GRCh	OTTHUMG000001	TSPO	**translocator protein**	Cytoplasm	transmembra	PK 11195,	1.119838674	0	0.66450674	0	1	0	8.52515578	8.76845509	8.80141023	8.75344592
2233	**ENSG0000010030**	RASD2	protein_coding	chr22	35540831	35553999	+	Human GRCh	OTTHUMG000001	RASD2	**RASD family member 2**	Cytoplasm	enzyme		2.921537174	1	0.0389294	1	1	0	4.09440573	4.50612621	5.24552381	5.19617985
2234	**ENSG0000010030**	TTLL12	protein_coding	chr22	43166622	43187134	-	Human GRCh	OTTHUMG000001	TTLL12	**tubulin tyrosine ligase like 12**	Cytoplasm	other		1.02872747	0	0.88720535	0	1	0	11.6717876	12.0598494	11.935578	11.8923058
2235	**ENSG0000010030**	CBX7	protein_coding	chr22	39120167	39152680	-	Human GRCh	OTTHUMG000001	CBX7	**chromobox 7**	Nucleus	other		-1.078787868	0	0.7386575	0	1	0	9.45226322	9.80356019	9.66155731	9.44307353
2236	**ENSG0000010031**	PDGFB	protein_coding	chr22	39223359	39244982	-	Human GRCh	OTTHUMG000001	PDGFB	**platelet derived growth factor subunit B**	Extracellular	growth facto	dasatinib, :	-1.112964445	0	0.83424801	0	1	0	5.0003831	5.22754559	5.02806543	5.01236152
2237	**ENSG0000010031**	ACR	protein_coding	chr22	50738196	50745339	+	Human GRCh	OTTHUMG000001	ACR	**acrosin**	Extracellular	peptidase		1.84867415	1	0.59539605	0	NA	0	1.83886425	2.55280862	2.53623387	2.45709009
2238	**ENSG0000010031**	CABP7	protein_coding	chr22	29720003	29731833	+	Human GRCh	OTTHUMG000001	CABP7	**calcium binding protein 7**	Cytoplasm	other		2.233103788	1	0.06481018	0	1	0	4.06667507	4.03400834	4.30113619	4.95317691
2239	**ENSG0000010031**	RPL3	protein_coding	chr22	39312882	39320389	-	Human GRCh	OTTHUMG000001	RPL3	**ribosomal protein L3**	Nucleus	other	cytarabine	-1.023378444	0	0.9154002	0	1	0	14.5563303	14.8273799	14.8661698	14.4427511
2240	**ENSG0000010031**	ZMAT5	protein_coding	chr22	29730956	29767011	-	Human GRCh	OTTHUMG000001	ZMAT5	**zinc finger matrin-type 5**	Nucleus	other		-1.077998189	0	0.77510802	0	1	0	8.14421924	8.37888918	8.17427131	8.19666321
2241	**ENSG0000010032**	RBFOX2	protein_coding	chr22	35738736	36028824	-	Human GRCh	OTTHUMG000001	RBFOX2	**RNA binding fox-1 homolog 2**	Nucleus	transcription		-1.152400499	0	0.52872623	0	1	0	11.3571649	11.7246447	11.5316481	11.2552364
2242	**ENSG0000010032**	SYNGR1	protein_coding	chr22	39349925	39385575	+	Human GRCh	OTTHUMG000000	SYNGR1	**synaptogyrin 1**	Plasma Mem	transporter		1.319157386	0	0.41401739	0	1	0	7.78444544	8.12510796	8.48017529	8.00359724
2243	**ENSG0000010032**	TAB1	protein_coding	chr22	39399778	39437060	+	Human GRCh	OTTHUMG000001	TAB1	**TGF-beta activated kinase 1 (MAP3K7) binding protein 1**	Cytoplasm	enzyme		-1.078424541	0	0.70802633	0	1	0	10.3105763	10.5723026	10.4364722	10.2868494
2244	**ENSG0000010032**	ASCC2	protein_coding	chr22	29788609	29838304	-	Human GRCh	OTTHUMG000000	ASCC2	**activating signal cointegrator 1 complex subunit 2**	Nucleus	other		-1.146342099	0	0.44348296	0	1	0	10.6821323	10.9556766	10.7670115	10.5723357
2245	**ENSG0000010033**	MTMR3	protein_coding	chr22	29883169	30030868	+	Human GRCh	OTTHUMG000001	MTMR3	**myotubularin related protein 3**	Cytoplasm	phosphatase		-1.277670162	0	0.22332728	0	1	0	12.0536687	12.3613599	12.0521134	11.8201021
2246	**ENSG0000010033**	MIEF1	protein_coding	chr22	39499432	39518132	+	Human GRCh	OTTHUMG000001	MIEF1	**mitochondrial elongation factor 1**	Cytoplasm	other		1.004319855	0	0.98344094	0	1	0	10.9416468	11.3621937	11.3038265	11.0309897
2247	**ENSG0000010033**	APOL4	protein_coding	chr22	36189124	36204840	-	Human GRCh	OTTHUMG000001	APOL4	**apolipoprotein L4**	Extracellular	other		8.003446611	1	0.09683582	0	1	0	0.85244976	0.95332424	1.45266123	1.69404223
2248	**ENSG0000010034**	PNPLA5	protein_coding	chr22	43879678	43892013	-	Human GRCh	OTTHUMG000000	PNPLA5	**patatin like phospholipase domain containing 5**	Cytoplasm	enzyme		5.942437975	1	0.33483401	0	NA	0	1.03462088	0.86004341	1.23737624	1.42992655
2249	**ENSG0000010034**	APOL1	protein_coding	chr22	36253071	36267530	+	Human GRCh	OTTHUMG000000	APOL1	**apolipoprotein L1**	Extracellular	transporter		-1.043003772	0	0.96035144	0	1	0	1.85370748	2.95383904	2.5405812	2.44024975
2250	**ENSG0000010034**	PNPLA3	protein_coding	chr22	43923792	43964488	+	Human GRCh	OTTHUMG000001	PNPLA3	**patatin like phospholipase domain containing 3**	Cytoplasm	enzyme		1.208370641	0	0.6753655	0	1	0	7.01165073	7.46881362	7.46314483	7.4769974
2251	**ENSG0000010034**	MYH9	protein_coding	chr22	36281280	36388010	-	Human GRCh	OTTHUMG000000	MYH9	**myosin heavy chain 9**	Cytoplasm	enzyme		1.045112517	0	0.8370663	0	1	0	13.4824891	13.8062037	13.7512829	13.6559852
2252	**ENSG0000010034**	CACNA1I	protein_coding	chr22	39570753	39689735	+	Human GRCh	OTTHUMG000001	CACNA1I	**calcium voltage-gated channel subunit alpha1 I**	Plasma Mem	ion channel	cinnarizine	-1.319487565	0	0.3813805	0	1	0	7.34453481	7.82138965	7.28254505	7.35137562
2253	**ENSG0000010034**	SAMM50	protein_coding	chr22	43955442	44010531	+	Human GRCh	OTTHUMG000001	SAMM50	**SAMM50 sorting and assembly machinery component**	Cytoplasm	other		-1.02618812	0	0.91141034	0	1	0	10.155069	10.6850909	10.4791682	10.3577912
2254	**ENSG0000010034**	TXN2	protein_coding	chr22	36467046	36481640	-	Human GRCh	OTTHUMG000001	TXN2	**thioredoxin 2**	Cytoplasm	enzyme		-1.122019495	0	0.58356687	0	1	0	10.6185394	10.9953707	10.7573667	10.6191478
2255	**ENSG0000010035**	FOXRED2	protein_coding	chr22	36487190	36507221	-	Human GRCh	OTTHUMG000000	FOXRED2	**FAD dependent oxidoreductase domain containing 2**	Cytoplasm	other		-1.103550521	0	0.62876336	0	1	0	11.9335287	12.1884282	12.0700281	11.8286434
2256	**ENSG0000010035**	GRAP2	protein_coding	chr22	39901084	39973721	+	Human GRCh	OTTHUMG000001	GRAP2	**GRB2 related adaptor protein 2**	Cytoplasm	other		10.7415139	1	0.04189133	1	1	0	1.53652711	1.64464125	2.3567701	2.23769837
2257	**ENSG0000010035**	EIF3D	protein_coding	chr22	36510855	36529184	-	Human GRCh	OTTHUMG000001	EIF3D	**eukaryotic translation initiation factor 3 subunit D**	Cytoplasm	translation re		-1.156861658	0	0.42487232	0	1	0	11.9185553	12.2716785	12.0507992	11.8227414
2258	**ENSG0000010035**	TNRC6B	protein_coding	chr22	40044817	40335808	+	Human GRCh	OTTHUMG000001	TNRC6B	**trinucleotide repeat containing adaptor 6B**	Cytoplasm	other		-1.087240583	0	0.6655898	0	1	0	12.8065788	13.0902186	12.992096	12.718788

2259	**ENSG0000010035**	SGSM3	protein_coding	chr22	40370591	40410289	+	Human GRCh	OTTHUMG000001	SGSM3	**small G protein signaling modulator 3**	Plasma Mem	other		-1.014232188	0	0.94582481	0	1	0	10.5869677	10.9406266	10.8580147	10.6548749
2260	**ENSG0000010036**	IFT27	protein_coding	chr22	36758202	36776256	-	Human GRCh	OTTHUMG000001	IFT27	**intraflagellar transport 27**	Extracellular	enzyme		-1.048248632	0	0.84685514	0	1	0	9.67917208	10.0465416	9.93663001	9.70030834
2261	**ENSG0000010036**	PVALB	protein_coding	chr22	36800684	36819479	-	Human GRCh	OTTHUMG000001	PVALB	**parvalbumin**	Cytoplasm	other		1	0	1	0	NA	0	-0.0043695	0.01939397	0.01553545	-0.0029473
2262	**ENSG0000010036**	KIAA0930	protein_coding	chr22	45192244	45240894	-	Human GRCh	OTTHUMG000001	KIAA0930	**KIAA0930**	Other	other		-1.078986128	0	0.69123947	0	1	0	10.1808019	10.4017487	10.2213921	10.2057534
2265	**ENSG0000010037**	SLC25A17	protein_coding	chr22	40769630	40819399	-	Human GRCh	OTTHUMG000001	SLC25A17	**solute carrier family 25 member 17**	Cytoplasm	transporter		1.006600765	0	0.97757118	0	1	0	9.12021953	9.53256178	9.6180436	9.006163
2267	**ENSG0000010037**	FAM118A	protein_coding	chr22	45308968	45341955	+	Human GRCh	OTTHUMG000001	FAM118A	**family with sequence similarity 118 member A**	Other	other		-1.026091824	0	0.9183864	0	1	0	10.0114245	10.3641346	10.3937249	9.89862078
2268	**ENSG0000010037**	KCTD17	protein_coding	chr22	37051739	37063390	+	Human GRCh	OTTHUMG000001	KCTD17	**potassium channel tetramerization domain containing 17**	Cytoplasm	other		1.200146445	0	0.45703018	0	1	0	8.07747081	7.93272853	8.31327575	8.07615898
2269	**ENSG0000010038**	ST13	protein_coding	chr22	40824535	40856639	-	Human GRCh	OTTHUMG000001	ST13	**ST13 Hsp70 interacting protein**	Cytoplasm	other		1.044095459	0	0.8361407	0	1	0	12.0217395	12.3586438	12.37327	12.113528
2270	**ENSG0000010038**	IL2RB	protein_coding	chr22	37118666	37175118	-	Human GRCh	OTTHUMG000001	IL2RB	**interleukin 2 receptor subunit beta**	Plasma Mem	transmembra	STK-012, n	1.002918631	0	0.99786493	0	1	0	2.91188208	2.01023221	1.99377859	2.92219816
2271	**ENSG0000010038**	RBX1	protein_coding	chr22	40951347	40973309	+	Human GRCh	OTTHUMG000001	RBX1	**ring-box 1**	Cytoplasm	enzyme		-1.00787012	0	0.97356978	0	1	0	9.76308259	9.97309509	10.0379169	9.66004522
2272	**ENSG0000010039**	EP300	protein_coding	chr22	41092592	41180077	+	Human GRCh	OTTHUMG000001	EP300	**E1A binding protein p300**	Nucleus	transcription	FT-7051, C	-1.139310267	0	0.50065945	0	1	0	12.9257303	13.2619887	13.0346301	12.8794806
2273	**ENSG0000010039**	L3MBTL2	protein_coding	chr22	41205282	41231271	+	Human GRCh	OTTHUMG000001	L3MBTL2	**L3MBTL histone methyl-lysine binding protein 2**	Nucleus	transcription		-1.020856588	0	0.91183053	0	1	0	10.3923427	10.7127918	10.6646441	10.400866
2274	**ENSG0000010039**	CHADL	protein_coding	chr22	41229510	41240931	-	Human GRCh	OTTHUMG000001	CHADL	**chondroadherin like**	Extracellular	other		1.151160279	0	0.7590614	0	1	0	4.96782667	4.84805282	5.01390132	5.05461071
2275	**ENSG0000010040**	RANGAP1	protein_coding	chr22	41244779	41286187	-	Human GRCh	OTTHUMG000001	RANGAP1	**Ran GTPase activating protein 1**	Nucleus	other		-1.010110261	0	0.95652978	0	1	0	12.1722901	12.391362	12.3761964	12.1654896
2276	**ENSG0000010040**	ZC3H7B	protein_coding	chr22	41301525	41360147	+	Human GRCh	OTTHUMG000001	ZC3H7B	**zinc finger CCCH-type containing 7B**	Nucleus	other		-1.053287466	0	0.7809603	0	1	0	11.7077868	11.9309227	11.921055	11.5908177
2277	**ENSG0000010041**	PHF5A	protein_coding	chr22	41459717	41468692	-	Human GRCh	OTTHUMG000001	PHF5A	**PHD finger protein 5A**	Nucleus	other		1.000989636	0	0.99635995	0	1	0	9.06817439	9.39338158	9.33225308	9.14605006
2278	**ENSG0000010041**	ACO2	protein_coding	chr22	41447830	41529273	+	Human GRCh	OTTHUMG000000	ACO2	**aconitase 2**	Cytoplasm	enzyme		-1.084862024	0	0.69601294	0	1	0	12.1314601	12.4567934	12.3111375	12.1047966
2279	**ENSG0000010041**	POLR3H	protein_coding	chr22	41525799	41544606	-	Human GRCh	OTTHUMG000001	POLR3H	**RNA polymerase III subunit H**	Nucleus	enzyme		-1.022766939	0	0.91474657	0	1	0	11.1329615	11.3919124	11.430808	11.0221842
2280	**ENSG0000010041**	TRMU	protein_coding	chr22	46330875	46357340	+	Human GRCh	OTTHUMG000001	TRMU	**tRNA mitochondrial 2-thiouridylase**	Cytoplasm	enzyme		1.015617008	0	0.94438546	0	1	0	10.8147232	11.2240437	11.2685751	10.7924938
2281	**ENSG0000010041**	PMM1	protein_coding	chr22	41576900	41589871	-	Human GRCh	OTTHUMG000001	PMM1	**phosphomannomutase 1**	Cytoplasm	enzyme		-1.149464752	0	0.56568705	0	1	0	9.63005267	9.91146556	9.68066127	9.55969694
2282	**ENSG0000010041**	DESI1	protein_coding	chr22	41598028	41621043	-	Human GRCh	OTTHUMG000000	DESI1	**desumoylating isopeptidase 1**	Cytoplasm	peptidase		-1.061305676	0	0.7722186	0	1	0	10.8035771	11.2170768	11.1050935	10.7936015
2283	**ENSG0000010042**	CERK	protein_coding	chr22	46684410	46738252	-	Human GRCh	OTTHUMG000001	CERK	**ceramide kinase**	Plasma Mem	kinase		-1.038406341	0	0.87362136	0	1	0	9.57773974	9.7070149	9.76932182	9.41071154
2284	**ENSG0000010042**	BRD1	protein_coding	chr22	49773283	49827873	-	Human GRCh	OTTHUMG000001	BRD1	**bromodomain containing 1**	Nucleus	transcription		-1.070423492	0	0.71902839	0	1	0	11.3463316	11.676557	11.5557295	11.3267843
2285	**ENSG0000010042**	ZBED4	protein_coding	chr22	49853844	49890080	+	Human GRCh	OTTHUMG000001	ZBED4	**zinc finger BED-type containing 4**	Nucleus	transcription		-1.101196042	0	0.64854619	0	1	0	11.2964504	11.6543695	11.5153823	11.2288616
2286	**ENSG0000010042**	MLC1	protein_coding	chr22	50059391	50085426	-	Human GRCh	OTTHUMG000001	MLC1	**modulator of VRAC current 1**	Plasma Mem	transporter		7.189861254	1	0.20568189	0	NA	0	1.40496395	1.00111389	1.77482959	2.22589137
2287	**ENSG0000010042**	HDAC10	protein_coding	chr22	50245183	50251405	-	Human GRCh	OTTHUMG000000	HDAC10	**histone deacetylase 10**	Nucleus	transcription	tributyrin,	1.50625135	1	0.20746993	0	1	0	7.28551949	7.62755877	8.09703704	7.7126831
2288	**ENSG0000010043**	KCNK10	protein_coding	chr14	88180103	88326907	-	Human GRCh	OTTHUMG000001	KCNK10	**potassium two pore domain channel subfamily K member 10**	Plasma Mem	ion channel		5.136913531	1	0.33351433	0	1	0	1.66863129	1.74644552	1.7338855	2.28105615
2289	**ENSG0000010043**	ABHD4	protein_coding	chr14	22598290	22612963	+	Human GRCh	OTTHUMG000000	ABHD4	**abhydrolase domain containing 4, N-acyl phospholipase B**	Cytoplasm	peptidase		-1.023856854	0	0.91820612	0	1	0	8.85478225	8.82924917	8.92168608	8.70185502
2290	**ENSG0000010044**	KHNYN	protein_coding	chr14	24429286	24441843	+	Human GRCh	OTTHUMG000001	KHNYN	**KH and NYN domain containing**	Other	other		-1.047375306	0	0.81617904	0	1	0	11.339111	11.6116984	11.5909046	11.2477207
2291	**ENSG0000010044**	FKBP3	protein_coding	chr14	45115599	45135319	-	Human GRCh	OTTHUMG000001	FKBP3	**FKBP prolyl isomerase 3**	Nucleus	enzyme		-1.008733618	0	0.96235016	0	1	0	9.72857353	10.023616	9.98961949	9.74938181
2292	**ENSG0000010044**	SDR39U1	protein_coding	chr14	24439766	24442905	-	Human GRCh	OTTHUMG000001	SDR39U1	**short chain dehydrogenase/reductase family 39U member 1**	Nucleus	other		-1.074920509	0	0.73838498	0	1	0	10.0522113	10.2541144	10.0817421	10.0746194
2296	**ENSG0000010046**	RBM23	protein_coding	chr14	22893204	22919182	-	Human GRCh	OTTHUMG000001	RBM23	**RNA binding motif protein 23**	Nucleus	other		-1.156252188	0	0.47410248	0	1	0	10.9335898	11.1327079	11.016347	10.7250709
2297	**ENSG0000010046**	PRMT5	protein_coding	chr14	22920525	22929408	-	Human GRCh	OTTHUMG000000	PRMT5	**protein arginine methyltransferase 5**	Cytoplasm	enzyme	AMG 193,	-1.150461137	0	0.48110143	0	1	0	11.2114135	11.6315184	11.3874126	11.1694118
2298	**ENSG0000010047**	COCH	protein_coding	chr14	30874514	30895065	+	Human GRCh	OTTHUMG000000	COCH	**cochlin**	Extracellular	other		-1.096925139	0	0.73430551	0	1	0	8.11511954	8.21914161	8.24893048	7.85482878
2299	**ENSG0000010047**	AP4S1	protein_coding	chr14	31025106	31130996	+	Human GRCh	OTTHUMG000001	AP4S1	**adaptor related protein complex 4 subunit sigma 1**	Cytoplasm	other		-1.269846399	0	0.28166281	0	1	0	8.16260352	8.447411	8.17439264	7.93670631
2300	**ENSG0000010047**	POLE2	protein_coding	chr14	49643555	49688422	-	Human GRCh	OTTHUMG000001	POLE2	**DNA polymerase epsilon 2, accessory subunit**	Nucleus	enzyme	bortezomi	1.01741819	0	0.94449835	0	1	0	8.80562304	9.33303567	9.22944693	8.99221917
2301	**ENSG0000010048**	VCPKMT	protein_coding	chr14	50108632	50116600	-	Human GRCh	OTTHUMG000000	VCPKMT	**valosin containing protein lysine methyltransferase**	Cytoplasm	enzyme		1.034200776	0	0.89401693	0	1	0	8.27753205	8.30884794	8.58131434	8.01506421
2302	**ENSG0000010048**	SOS2	protein_coding	chr14	50117130	50231578	-	Human GRCh	OTTHUMG000001	SOS2	**SOS Ras/Rho guanine nucleotide exchange factor 2**	Cytoplasm	other		-1.192765517	0	0.38520322	0	1	0	10.5334416	10.7721472	10.6156031	10.2984541
2303	**ENSG0000010049**	CDKL1	protein_coding	chr14	50326265	50416461	-	Human GRCh	OTTHUMG000001	CDKL1	**cyclin dependent kinase like 1**	Nucleus	kinase		1.227516918	0	0.57597123	0	1	0	6.54491654	6.80150949	7.16728595	6.5248137
2304	**ENSG0000010050**	NIN	protein_coding	chr14	50719763	50831162	-	Human GRCh	OTTHUMG000000	NIN	**ninein**	Cytoplasm	other		1.059497409	0	0.76797009	0	1	0	10.7660855	10.9030251	11.0027762	10.78346
2305	**ENSG0000010050**	PYGL	protein_coding	chr14	50857891	50944483	-	Human GRCh	OTTHUMG000001	PYGL	**glycogen phosphorylase L**	Cytoplasm	enzyme		-1.079681381	0	0.75875367	0	1	0	10.9479144	11.3127983	11.1694273	10.9315037
2306	**ENSG0000010050**	TRIM9	protein_coding	chr14	50975262	51096061	-	Human GRCh	OTTHUMG000001	TRIM9	**tripartite motif containing 9**	Cytoplasm	enzyme		1.062237225	0	0.92603988	0	1	0	5.30269809	6.06179617	5.97461762	5.57240439
2307	**ENSG0000010051**	PSMC6	protein_coding	chr14	52707178	52728590	+	Human GRCh	OTTHUMG000001	PSMC6	**proteasome 26S subunit, ATPase 6**	Nucleus	peptidase		-1.013752341	0	0.94587746	0	1	0	10.713611	10.9751918	11.0316755	10.6027798
2308	**ENSG0000010052**	GNPNAT1	protein_coding	chr14	52775193	52791668	-	Human GRCh	OTTHUMG000001	GNPNAT1	**glucosamine-phosphate N-acetyltransferase 1**	Cytoplasm	enzyme		1.033272651	0	0.86251149	0	1	0	10.4626258	10.5967393	10.7265237	10.3840105
2309	**ENSG0000010052**	DDHD1	protein_coding	chr14	53036745	53153323	-	Human GRCh	OTTHUMG000001	DDHD1	**DDHD domain containing 1**	Extracellular	enzyme		1.153082824	0	0.53403971	0	1	0	10.5753551	10.9417722	11.0844803	10.7516549
2310	**ENSG0000010052**	CDKN3	protein_coding	chr14	54396849	54420218	+	Human GRCh	OTTHUMG000001	CDKN3	**cyclin dependent kinase inhibitor 3**	Cytoplasm	phosphatase		-1.086957521	0	0.76780052	0	1	0	8.81522156	9.33245259	9.00566046	9.01485965
2311	**ENSG0000010052**	CNIH1	protein_coding	chr14	54423561	54441391	-	Human GRCh	OTTHUMG000001	CNIH1	**cornichon family AMPA receptor auxiliary protein 1**	Plasma Mem	other		1.03955678	0	0.85628245	0	1	0	10.5866026	10.7902482	10.8686139	10.5859043
2312	**ENSG0000010053**	CGRRF1	protein_coding	chr14	54509812	54539292	+	Human GRCh	OTTHUMG000001	CGRRF1	**cell growth regulator with ring finger domain 1**	Nucleus	other		-1.033493904	0	0.89775273	0	1	0	7.41602123	7.64813308	7.67457601	7.30274294
2313	**ENSG0000010055**	ATP6V1D	protein_coding	chr14	67294371	67360265	-	Human GRCh	OTTHUMG000001	ATP6V1D	**ATPase H+ transporting V1 subunit D**	Cytoplasm	transporter		-1.055306571	0	0.80968586	0	1	0	9.63254201	9.9126213	9.90550223	9.5054532
2315	**ENSG0000010055**	PLEK2	protein_coding	chr14	67386984	67412167	-	Human GRCh	OTTHUMG000001	PLEK2	**pleckstrin 2**	Plasma Mem	other		1.203555769	0	0.67454662	0	1	0	4.99313289	4.96971312	4.54771784	5.49114254
2316	**ENSG0000010056**	PIGH	protein_coding	chr14	67581955	67600286	-	Human GRCh	OTTHUMG000001	PIGH	**phosphatidylinositol glycan anchor biosynthesis class H**	Cytoplasm	enzyme		-1.121823366	0	0.67130727	0	1	0	8.43138083	8.82553785	8.66226687	8.35142058
2318	**ENSG0000010056**	PSMA3	protein_coding	chr14	58244843	58272012	+	Human GRCh	OTTHUMG000001	PSMA3	**proteasome 20S subunit alpha 3**	Cytoplasm	peptidase		-1.015886575	0	0.94204693	0	1	0	10.693914	10.9893215	10.9542066	10.6987967
2319	**ENSG0000010056**	VTI1B	protein_coding	chr14	67647085	67674820	-	Human GRCh	OTTHUMG000001	VTI1B	**vesicle transport through interaction with t-SNAREs 1B**	Plasma Mem	transporter		-1.074045787	0	0.72206923	0	1	0	10.0990086	10.4023659	10.2951627	10.0547477
2320	**ENSG0000010057**	TIMM9	protein_coding	chr14	58408495	58427531	-	Human GRCh	OTTHUMG000001	TIMM9	**translocase of inner mitochondrial membrane 9**	Cytoplasm	transporter		-1.046610371	0	0.83164122	0	1	0	9.18955118	9.43397528	9.4020276	9.11637938
2321	**ENSG0000010057**	GSTZ1	protein_coding	chr14	77320996	77331597	+	Human GRCh	OTTHUMG000001	GSTZ1	**glutathione S-transferase zeta 1**	Cytoplasm	enzyme		-1.092157449	0	0.69518766	0	1	0	8.3918381	8.82259731	8.73492144	8.29429373
2322	**ENSG0000010057**	KIAA0586	protein_coding	chr14	58427385	58551297	+	Human GRCh	OTTHUMG000001	KIAA0586	**KIAA0586**	Cytoplasm	other		-1.23732878	0	0.31342014	0	1	0	11.3766026	11.7065051	11.4793199	11.1270883
2323	**ENSG0000010058**	TMED8	protein_coding	chr14	77335029	77377094	-	Human GRCh	OTTHUMG000001	TMED8	**transmembrane p24 trafficking protein family member 8**	Other	other		-1.005363396	0	0.97899222	0	1	0	10.9491305	11.1896954	11.2222595	10.8944879
2324	**ENSG0000010058**	SAMD15	protein_coding	chr14	77376689	77392163	+	Human GRCh	OTTHUMG000001	SAMD15	**sterile alpha motif domain containing 15**	Other	other		-1.072226966	0	0.84449361	0	1	0	5.95224873	5.89557634	5.91795667	5.79319485
2325	**ENSG0000010059**	AHSA1	protein_coding	chr14	77457870	77469472	+	Human GRCh	OTTHUMG000001	AHSA1	**activator of HSP90 ATPase activity 1**	Cytoplasm	other		1.047555188	0	0.82563307	0	1	0	10.5050704	10.7329306	10.8180156	10.5148904
2326	**ENSG0000010059**	DAAM1	protein_coding	chr14	59188646	59371405	+	Human GRCh	OTTHUMG000001	DAAM1	**dishevelled associated activator of morphogenesis 1**	Cytoplasm	other		1.007902495	0	0.97132893	0	1	0	10.3473178	10.6865068	10.7148868	10.3289153
2327	**ENSG0000010059**	ISM2	protein_coding	chr14	77474394	77498816	-	Human GRCh	OTTHUMG000001	ISM2	**isthmin 2**	Other	other		4.598295989	1	0.02336756	1	1	0	2.94726944	2.05134481	3.81380547	3.47405121
2328	**ENSG0000010059**	SPTLC2	protein_coding	chr14	77505997	77616637	-	Human GRCh	OTTHUMG000001	SPTLC2	**serine palmitoyltransferase long chain base subunit 2**	Cytoplasm	enzyme		-1.03951232	0	0.85510005	0	1	0	11.2584612	11.4886997	11.5296411	11.1068042
2329	**ENSG0000010059**	RIN3	protein_coding	chr14	92513781	92688994	+	Human GRCh	OTTHUMG000001	RIN3	**Ras and Rab interactor 3**	Cytoplasm	other		-1.143448737	0	0.7512099	0	1	0	4.863954	4.66097527	4.29807924	4.90773902
2330	**ENSG0000010060**	LGMN	protein_coding	chr14	92703807	92748679	-	Human GRCh	OTTHUMG000001	LGMN	**legumain**	Cytoplasm	peptidase		-1.102781965	0	0.66307425	0	1	0	9.54705226	9.84075214	9.50579369	9.678604
2331	**ENSG0000010060**	ALKBH1	protein_coding	chr14	77672404	77708023	-	Human GRCh	OTTHUMG000001	ALKBH1	**alkB homolog 1, histone H2A dioxygenase**	Cytoplasm	enzyme		-1.096928173	0	0.69821021	0	1	0	8.70878985	9.10827972	9.03509573	8.57230101
2332	**ENSG0000010060**	SNW1	protein_coding	chr14	77717599	77761207	-	Human GRCh	OTTHUMG000001	SNW1	**SNW domain containing 1**	Nucleus	transcription		-1.05122782	0	0.81225267	0	1	0	10.4871551	10.818878	10.6763352	10.5389923
2333	**ENSG0000010060**	CHGA	protein_coding	chr14	92923150	92935285	+	Human GRCh	OTTHUMG000001	CHGA	**chromogranin A**	Cytoplasm	other		-1.522413744	-1	0.53598789	0	1	0	2.95603496	4.12527132	3.8486766	2.96213584
2334	**ENSG0000010060**	ITPK1	protein_coding	chr14	92936914	93116320	-	Human GRCh	OTTHUMG000001	ITPK1	**inositol-tetrakisphosphate 1-kinase**	Cytoplasm	kinase		-1.150518546	0	0.47491669	0	1	0	10.5134117	10.6821254	10.4827412	10.4100306
2335	**ENSG0000010061**	DHRS7	protein_coding	chr14	60144119	60169856	-	Human GRCh	OTTHUMG000001	DHRS7	**dehydrogenase/reductase 7**	Cytoplasm	enzyme		1.06159891	0	0.84125044	0	1	0	8.58204478	8.61952373	8.72028807	8.60847745
2336	**ENSG0000010061**	PPM1A	protein_coding	chr14	60245752	60299087	+	Human GRCh	OTTHUMG000001	PPM1A	**protein phosphatase, Mg2+/Mn2+ dependent 1A**	Cytoplasm	phosphatase		-1.046455389	0	0.8375986	0	1	0	10.959606	11.209493	11.1885504	10.8756508
2337	**ENSG0000010062**	SIX4	protein_coding	chr14	60709539	60724351	-	Human GRCh	OTTHUMG000000	SIX4	**SIX homeobox 4**	Nucleus	transcription		-1.179283893	0	0.4773163	0	1	0	10.8672757	11.0517588	10.9777186	10.5467053
2338	**ENSG0000010062**	GALNT16	protein_coding	chr14	69259277	69357033	+	Human GRCh	OTTHUMG000001	GALNT16	**polypeptide N-acetylgalactosaminyltransferase 16**	Cytoplasm	enzyme		1.586424433	1	0.2310467	0	1	0	5.57286143	4.97080457	5.65130022	5.70881653
2340	**ENSG0000010062**	CEP128	protein_coding	chr14	80476983	80959517	-	Human GRCh	OTTHUMG000001	CEP128	**centrosomal protein 128**	Cytoplasm	other		-1.044979713	0	0.84575602	0	1	0	9.57431998	9.70822158	9.64283706	9.54731445
2341	**ENSG0000010063**	ERH	protein_coding	chr14	69380128	69398299	-	Human GRCh	OTTHUMG000001	ERH	**ERH mRNA splicing and mitosis factor**	Nucleus	other		-1.079290426	0	0.74020323	0	1	0	10.915642	11.3076424	11.1339313	10.9435473
2342	**ENSG0000010064**	HIF1A	protein_coding	chr14	61695513	61748259	+	Human GRCh	OTTHUMG000001	HIF1A	**hypoxia inducible factor 1 subunit alpha**	Nucleus	transcription	BAY 87-22	-1.02745154	0	0.90645944	0	1	0	12.2095687	12.6410564	12.4866131	12.3375001
2343	**ENSG0000010064**	SUSD6	protein_coding	chr14	69611596	69715144	+	Human GRCh	OTTHUMG000001	SUSD6	**sushi domain containing 6**	Other	other		-1.094405173	0	0.73240012	0	1	0	9.45534634	9.83700224	9.70357689	9.41451971
2344	**ENSG0000010065**	SRSF5	protein_coding	chr14	69726900	69772005	+	Human GRCh	OTTHUMG000001	SRSF5	**serine and arginine rich splicing factor 5**	Nucleus	other		-1.067449129	0	0.75215736	0	1	0	11.9462509	12.1974052	12.0934939	11.9146046
2346	**ENSG0000010066**	EIF5	protein_coding	chr14	103333544	103345025	+	Human GRCh	OTTHUMG000001	EIF5	**eukaryotic translation initiation factor 5**	Cytoplasm	translation re		-1.170853054	0	0.4208294	0	1	0	11.9782198	12.2671074	12.0631794	11.8420342
2348	**ENSG0000010067**	SLC8A3	protein_coding	chr14	70044215	70189480	-	Human GRCh	OTTHUMG000001	SLC8A3	**solute carrier family 8 member A3**	Plasma Mem	transporter		1	0	1	0	1	0	1.69997284	1.81840971	1.79955893	1.70723231
2349	**ENSG0000010069**	DICER1	protein_coding	chr14	95086228	95158010	-	Human GRCh	OTTHUMG000001	DICER1	**dicer 1, ribonuclease III**	Cytoplasm	enzyme		-1.067479239	0	0.75428913	0	1	0	12.1361445	12.4290942	12.3655448	12.059343
2350	**ENSG0000010071**	ZFYVE21	protein_coding	chr14	103715730	103733668	+	Human GRCh	OTTHUMG000001	ZFYVE21	**zinc finger FYVE-type containing 21**	Other	other		1.037164421	0	0.87026577	0	1	0	9.49919722	9.84940951	9.89156832	9.53524707
2351	**ENSG0000010071**	MTHFD1	protein_coding	chr14	64388031	64463457	+	Human GRCh	OTTHUMG000001	MTHFD1	**methylenetetrahydrofolate dehydrogenase, cyclohydrolase an**	Cytoplasm	enzyme		-1.068019806	0	0.7851959	0	1	0	12.0494584	12.4373671	12.3424264	12.0073135
2353	**ENSG0000010072**	ZC3H14	protein_coding	chr14	88562970	88627596	+	Human GRCh	OTTHUMG000001	ZC3H14	**zinc finger CCCH-type containing 14**	Nucleus	other		-1.023914063	0	0.91526919	0	1	0	11.118763	11.4092942	11.3832508	11.0928113

2354	**ENSG0000010072**	TELO2	protein_coding	chr16	1493344	1510457	+	Human GRCh	OTTHUMG000000	TELO2	**telomere maintenance 2**	Cytoplasm	other		1.074267977	0	0.72682693	0	1	0	10.8895673	11.1886464	11.2751907	10.9606749
2355	**ENSG0000010073**	PCNX1	protein_coding	chr14	70907405	71115382	+	Human GRCh	OTTHUMG000001	PCNX1	**pecanex 1**	Plasma Mem	other		-1.059594431	0	0.79240239	0	1	0	11.359352	11.5984955	11.5990335	11.2117731
2356	**ENSG0000010073**	BDKRB1	protein_coding	chr14	96256210	96268967	+	Human GRCh	OTTHUMG000001	BDKRB1	**bradykinin receptor B1**	Plasma Mem	G-protein cou		1	0	1	0	1	0	1.69039368	1.75879479	1.74771107	1.69449947
2357	**ENSG0000010074**	GSKIP	protein_coding	chr14	96363452	96387288	+	Human GRCh	OTTHUMG000001	GSKIP	**GSK3B interacting protein**	Nucleus	other		1.026339427	0	0.91100386	0	1	0	8.71775025	8.89860647	8.93485431	8.73637505
2358	**ENSG0000010074**	VRK1	protein_coding	chr14	96797304	96954846	+	Human GRCh	OTTHUMG000001	VRK1	**VRK serine/threonine kinase 1**	Nucleus	kinase		1.11189153	0	0.64207387	0	1	0	11.1211774	11.4162973	11.593385	11.1529244
2359	**ENSG0000010076**	PSMC1	protein_coding	chr14	90256527	90275429	+	Human GRCh	OTTHUMG000001	PSMC1	**proteasome 26S subunit, ATPase 1**	Nucleus	peptidase		-1.168872445	0	0.44202935	0	1	0	10.1008367	10.3857718	10.1249427	10.0348006
2360	**ENSG0000010076**	PAPLN	protein_coding	chr14	73237497	73274640	+	Human GRCh	OTTHUMG000001	PAPLN	**papilin, proteoglycan like sulfated glycoprotein**	Extracellular	other		1.968425747	1	0.03444928	1	1	0	5.51835063	5.59439692	5.69366954	6.34944565
2361	**ENSG0000010078**	RPS6KA5	protein_coding	chr14	90847861	91060641	-	Human GRCh	OTTHUMG000001	RPS6KA5	**ribosomal protein S6 kinase A5**	Nucleus	kinase	H89, fasud	-1.204027328	0	0.45307642	0	1	0	10.7798989	11.1018137	10.936949	10.5236778
2362	**ENSG0000010079**	PPP4R3A	protein_coding	chr14	91457508	91510554	-	Human GRCh	OTTHUMG000001	PPP4R3A	**protein phosphatase 4 regulatory subunit 3A**	Plasma Mem	other		-1.072752235	0	0.717546	0	1	0	11.8203372	12.1445247	12.0741036	11.7327317
2363	**ENSG0000010080**	C14orf93	protein_coding	chr14	22985894	23010166	-	Human GRCh	OTTHUMG000000	C14orf93	**chromosome 14 open reading frame 93**	Other	other		-1.213585187	0	0.43590769	0	1	0	8.35990638	8.88184445	8.5054381	8.37668395
2364	**ENSG0000010080**	PSMB5	protein_coding	chr14	23016543	23035230	-	Human GRCh	OTTHUMG000000	PSMB5	**proteasome 20S subunit beta 5**	Cytoplasm	peptidase	bortezomi	-1.167047845	0	0.46133269	0	1	0	10.9640879	11.334371	11.0864386	10.8840932
2365	**ENSG0000010081**	YY1	protein_coding	chr14	100238298	100282788	+	Human GRCh	OTTHUMG000001	YY1	**YY1 transcription factor**	Nucleus	transcription		1.013707052	0	0.94471448	0	1	0	11.8488253	12.0987159	12.1237752	11.8509145
2366	**ENSG0000010081**	ACIN1	protein_coding	chr14	23058564	23095614	-	Human GRCh	OTTHUMG000000	ACIN1	**apoptotic chromatin condensation inducer 1**	Nucleus	enzyme		-1.102577774	0	0.59003265	0	1	0	12.9541682	13.1966184	13.0734672	12.8655352
2367	**ENSG0000010081**	CCNB1IP1	protein_coding	chr14	20311368	20333312	-	Human GRCh	OTTHUMG000000	CCNB1IP1	**cyclin B1 interacting protein 1**	Nucleus	enzyme		-1.237523825	0	0.37035413	0	1	0	10.4000874	10.7388025	10.4814005	10.1920486
2368	**ENSG0000010081**	TRIP11	protein_coding	chr14	91965991	92040896	-	Human GRCh	OTTHUMG000001	TRIP11	**thyroid hormone receptor interactor 11**	Cytoplasm	transcription		-1.151380585	0	0.46214048	0	1	0	11.1343401	11.2534229	11.1845018	10.8767324
2369	**ENSG0000010082**	APEX1	protein_coding	chr14	20455191	20457772	+	Human GRCh	OTTHUMG000000	APEX1	**apurinic/apyrimidinic endodeoxyribonuclease 1**	Nucleus	enzyme	lucanthone	-1.212742721	0	0.36619312	0	1	0	12.1056462	12.4534642	12.1596812	11.984512
2370	**ENSG0000010083**	PABPN1	protein_coding	chr14	23321457	23326163	+	Human GRCh	OTTHUMG000000	PABPN1	**poly(A) binding protein nuclear 1**	Nucleus	enzyme		1.045320049	0	0.814897	0	1	0	12.1145784	12.2793958	12.4441821	12.0137805
2371	**ENSG0000010084**	EFS	protein_coding	chr14	23356403	23365752	-	Human GRCh	OTTHUMG000000	EFS	**embryonal Fyn-associated substrate**	Cytoplasm	other		-1.176117786	0	0.84167899	0	1	0	2.60594228	3.38599979	3.56665746	1.42636923
2372	**ENSG0000010085**	ARHGAP5	protein_coding	chr14	32076114	32159728	+	Human GRCh	OTTHUMG000001	ARHGAP5	**Rho GTPase activating protein 5**	Cytoplasm	enzyme		1.024488566	0	0.91454064	0	1	0	12.8356909	13.0957514	13.215139	12.7347625
2373	**ENSG0000010086**	CINP	protein_coding	chr14	102341102	102362916	-	Human GRCh	OTTHUMG000001	CINP	**cyclin dependent kinase 2 interacting protein**	Nucleus	other		-1.146044095	0	0.54627609	0	1	0	9.32559659	9.45486503	9.20125236	9.28192698
2374	**ENSG0000010086**	DHRS2	protein_coding	chr14	23630115	23645639	+	Human GRCh	OTTHUMG000000	DHRS2	**dehydrogenase/reductase 2**	Nucleus	enzyme		-1.182417141	0	0.8004423	0	1	0	9.02118914	9.09435844	8.62050257	9.0634519
2375	**ENSG0000010088**	SRP54	protein_coding	chr14	34981957	35029686	+	Human GRCh	OTTHUMG000001	SRP54	**signal recognition particle 54**	Cytoplasm	enzyme		-1.069079526	0	0.75949866	0	1	0	10.8723952	11.2183065	11.1466648	10.7958754
2376	**ENSG0000010088**	CPNE6	protein_coding	chr14	24070837	24078100	+	Human GRCh	OTTHUMG000000	CPNE6	**copine 6**	Plasma Mem	enzyme		1	0	1	0	NA	0	0.86462656	0.87344572	0.87196248	0.86513365
2377	**ENSG0000010088**	CHD8	protein_coding	chr14	21385194	21456126	-	Human GRCh	OTTHUMG000001	CHD8	**chromodomain helicase DNA binding protein 8**	Nucleus	enzyme		-1.075140141	0	0.70372805	0	1	0	12.2108048	12.5858528	12.4420849	12.2146349
2378	**ENSG0000010088**	PCK2	protein_coding	chr14	24094053	24110598	+	Human GRCh	OTTHUMG000000	PCK2	**phosphoenolpyruvate carboxykinase 2, mitochondrial**	Cytoplasm	kinase		-1.531127725	-1	0.09608386	0	1	0	10.2885151	9.94721487	9.76096555	9.57576548
2379	**ENSG0000010089**	PRORP	protein_coding	chr14	35121846	35277622	+	Human GRCh	OTTHUMG000001	PRORP	**protein only RNase P catalytic subunit**	Cytoplasm	enzyme		-1.204611725	0	0.38384504	0	1	0	10.9670168	11.3491332	11.1687806	10.7161872
2380	**ENSG0000010089**	DCAF11	protein_coding	chr14	24114195	24125242	+	Human GRCh	OTTHUMG000000	DCAF11	**DDB1 and CUL4 associated factor 11**	Nucleus	other		-1.205084429	0	0.36964311	0	1	0	11.6546353	11.9445083	11.697444	11.4928086
2381	**ENSG0000010090**	PSMA6	protein_coding	chr14	35278633	35317493	+	Human GRCh	OTTHUMG000001	PSMA6	**proteasome 20S subunit alpha 6**	Cytoplasm	peptidase		-1.132453575	0	0.5927649	0	1	0	12.3233659	12.7192333	12.659033	12.0589866
2382	**ENSG0000010090**	NFKBIA	protein_coding	chr14	35401079	35404749	-	Human GRCh	OTTHUMG000001	NFKBIA	**NFKB inhibitor alpha**	Cytoplasm	transcription		-1.150853075	0	0.4519812	0	1	0	11.5710006	11.8107485	11.6775094	11.3847059
2383	**ENSG0000010090**	EMC9	protein_coding	chr14	24138959	24141591	-	Human GRCh	OTTHUMG000000	EMC9	**ER membrane protein complex subunit 9**	Plasma Mem	other		-1.072438451	0	0.75522036	0	1	0	9.26383518	9.37751177	9.30165802	9.1857923
2384	**ENSG0000010091**	PSME2	protein_coding	chr14	24143362	24147570	-	Human GRCh	OTTHUMG000000	PSME2	**proteasome activator subunit 2**	Cytoplasm	peptidase		-1.013363267	0	0.94342872	0	1	0	10.0680714	10.3193678	10.3045442	10.0534977
2385	**ENSG0000010091**	BRMS1L	protein_coding	chr14	35826338	35932325	+	Human GRCh	OTTHUMG000001	BRMS1L	**BRMS1 like transcriptional repressor**	Other	other		1.04825992	0	0.83017179	0	1	0	8.05343492	8.43364866	8.48509683	8.09897018
2386	**ENSG0000010091**	REC8	protein_coding	chr14	24171853	24180257	+	Human GRCh	OTTHUMG000001	REC8	**REC8 meiotic recombination protein**	Nucleus	other		-1.280546938	0	0.53721374	0	1	0	5.88084854	6.32869182	6.18136019	5.47771024
2387	**ENSG0000010092**	TM9SF1	protein_coding	chr14	24189149	24195687	-	Human GRCh	OTTHUMG000000	TM9SF1	**transmembrane 9 superfamily member 1**	Plasma Mem	transporter		-1.178703255	0	0.40356537	0	1	0	10.3677345	10.6047493	10.4120003	10.1976502
2388	**ENSG0000010093**	SEC23A	protein_coding	chr14	39031919	39109646	-	Human GRCh	OTTHUMG000000	SEC23A	**SEC23 homolog A, COPII coat complex component**	Cytoplasm	transporter		1.020212399	0	0.92904715	0	1	0	10.5812003	10.8826602	10.9226362	10.5802563
2389	**ENSG0000010093**	GMPR2	protein_coding	chr14	24232422	24239242	+	Human GRCh	OTTHUMG000001	GMPR2	**guanosine monophosphate reductase 2**	Cytoplasm	enzyme		-1.078710167	0	0.72124731	0	1	0	9.24111461	9.58524753	9.46627856	9.20379027
2390	**ENSG0000010094**	PNN	protein_coding	chr14	39175183	39183220	+	Human GRCh	OTTHUMG000000	PNN	**pinin, desmosome associated protein**	Plasma Mem	other		1.12570901	0	0.56981641	0	1	0	11.0866075	11.4903821	11.5388077	11.3245645
2391	**ENSG0000010094**	RABGGTA	protein_coding	chr14	24265538	24271611	-	Human GRCh	OTTHUMG000001	RABGGTA	**Rab geranylgeranyltransferase subunit alpha**	Extracellular	enzyme		-1.109090783	0	0.64014582	0	1	0	9.27198552	9.5734238	9.32624207	9.31050467
2392	**ENSG0000010096**	NFATC4	protein_coding	chr14	24365673	24379604	+	Human GRCh	OTTHUMG000000	NFATC4	**nuclear factor of activated T cells 4**	Nucleus	transcription		-1.012038135	0	0.96265153	0	1	0	10.6545024	10.9006286	10.8482988	10.6867032
2393	**ENSG0000010097**	PLTP	protein_coding	chr20	45898621	45912155	-	Human GRCh	OTTHUMG000000	PLTP	**phospholipid transfer protein**	Extracellular	enzyme		1.243365635	0	0.47654335	0	1	0	7.27270146	7.01866967	7.23450097	7.50966292
2394	**ENSG0000010098**	PCIF1	protein_coding	chr20	45934683	45948023	+	Human GRCh	OTTHUMG000000	PCIF1	**phosphorylated CTD interacting factor 1**	Nucleus	enzyme		-1.334530849	0	0.14734946	0	1	0	10.1297984	10.4292919	10.0790088	9.86082431
2395	**ENSG0000010098**	GSS	protein_coding	chr20	34928432	34956027	-	Human GRCh	OTTHUMG000000	GSS	**glutathione synthetase**	Cytoplasm	enzyme	N-acetyl-L-	-1.180270645	0	0.42060885	0	1	0	10.7432631	11.0783691	10.8560224	10.6074624
2396	**ENSG0000010098**	MMP9	protein_coding	chr20	46008908	46016561	+	Human GRCh	OTTHUMG000000	MMP9	**matrix metallopeptidase 9**	Extracellular	peptidase	MMP2 MN	8.186992193	1	0.13898673	0	1	0	1.642482	1.67069368	2.13392456	1.8813849
2397	**ENSG0000010098**	VSX1	protein_coding	chr20	25070885	25082141	-	Human GRCh	OTTHUMG000000	VSX1	**visual system homeobox 1**	Nucleus	transcription		-5.927359035	-1	0.37261645	0	NA	0	0.72045961	1.17396403	0.73006625	0.72112655
2398	**ENSG0000010099**	TRPC4AP	protein_coding	chr20	35002404	35092807	-	Human GRCh	OTTHUMG000000	TRPC4AP	**transient receptor potential cation channel subfamily C memb**	Cytoplasm	transporter		-1.101439049	0	0.63721079	0	1	0	10.9667545	11.2353294	11.1531556	10.8263233
2399	**ENSG0000010099**	PYGB	protein_coding	chr20	25248085	25298012	+	Human GRCh	OTTHUMG000000	PYGB	**glycogen phosphorylase B**	Cytoplasm	enzyme		1.029459087	0	0.90944417	0	1	0	12.2879497	12.6760672	12.4235499	12.6290596
2400	**ENSG0000010099**	ABHD12	protein_coding	chr20	25294742	25390835	-	Human GRCh	OTTHUMG000000	ABHD12	**abhydrolase domain containing 12, lysophospholipase**	Plasma Mem	enzyme		1.04064246	0	0.85019439	0	1	0	10.659612	10.9392626	10.9407069	10.755306
2401	**ENSG0000010100**	PROCR	protein_coding	chr20	35172072	35216240	+	Human GRCh	OTTHUMG000000	PROCR	**protein C receptor**	Plasma Mem	other		54.83506362	1	0.01066181	1	1	0	1.62376292	1.72038737	1.7049187	4.1673765
2402	**ENSG0000010100**	GINS1	protein_coding	chr20	25391008	25452700	+	Human GRCh	OTTHUMG000000	GINS1	**GINS complex subunit 1**	Nucleus	other		-1.006646032	0	0.97186965	0	1	0	10.6266059	10.8786052	10.8395342	10.6573977
2403	**ENSG0000010100**	NINL	protein_coding	chr20	25451594	25585531	-	Human GRCh	OTTHUMG000000	NINL	**ninein like**	Cytoplasm	other		1.197745917	0	0.51541226	0	1	0	9.47333224	9.40723112	9.8429931	9.35747926
2404	**ENSG0000010101**	CD40	protein_coding	chr20	46118271	46129863	+	Human GRCh	OTTHUMG000000	CD40	**CD40 molecule**	Plasma Mem	transmembra	anti-CD40	1.489659254	0	0.65541341	0	1	0	3.73459387	2.9740028	3.95353523	3.52344215
2405	**ENSG0000010101**	UQCC1	protein_coding	chr20	35302566	35412031	-	Human GRCh	OTTHUMG000000	UQCC1	**ubiquinol-cytochrome c reductase complex assembly factor 1**	Cytoplasm	other		-1.091341976	0	0.67825757	0	1	0	10.2318686	10.5003294	10.4624648	10.0553615
2406	**ENSG0000010104**	ZMYND8	protein_coding	chr20	47209214	47356889	-	Human GRCh	OTTHUMG000000	ZMYND8	**zinc finger MYND-type containing 8**	Nucleus	transcription		-1.265620967	0	0.25748448	0	1	0	11.9506782	12.2241657	11.9540282	11.7058358
2407	**ENSG0000010104**	SGK2	protein_coding	chr20	43558968	43588237	+	Human GRCh	OTTHUMG000000	SGK2	**serum/glucocorticoid regulated kinase 2**	Cytoplasm	kinase		-1.395696289	0	0.4391178	0	1	0	5.90782656	6.62142706	6.08349661	5.87280011
2408	**ENSG0000010105**	IFT52	protein_coding	chr20	43590937	43647299	+	Human GRCh	OTTHUMG000000	IFT52	**intraflagellar transport 52**	Cytoplasm	other		-1.192308906	0	0.48491472	0	1	0	8.92053378	9.38317699	9.13899752	8.80059388
2409	**ENSG0000010105**	MYBL2	protein_coding	chr20	43667019	43716495	+	Human GRCh	OTTHUMG000000	MYBL2	**MYB proto-oncogene like 2**	Nucleus	transcription		-1.185009442	0	0.45228693	0	1	0	11.1341483	11.6018173	11.2649797	11.1454301
2411	**ENSG0000010107**	HNF4A	protein_coding	chr20	44355699	44432845	+	Human GRCh	OTTHUMG000000	HNF4A	**hepatocyte nuclear factor 4 alpha**	Nucleus	transcription		-4.964745293	-1	0.76016999	0	1	0	0.55662481	-0.5229328	-0.5352303	-0.5968531
2412	**ENSG0000010107**	NDRG3	protein_coding	chr20	36651766	36746090	-	Human GRCh	OTTHUMG000000	NDRG3	**NDRG family member 3**	Cytoplasm	other		-1.085200766	0	0.72493007	0	1	0	10.1103471	10.5138242	10.3385677	10.1281051
2413	**ENSG0000010108**	SLA2	protein_coding	chr20	36612318	36646196	-	Human GRCh	OTTHUMG000000	SLA2	**Src like adaptor 2**	Plasma Mem	other		-1.660061556	-1	0.43712834	0	1	0	4.48641968	4.31501654	4.25390487	3.62168216
2414	**ENSG0000010108**	RAB5IF	protein_coding	chr20	36605779	36612557	+	Human GRCh	OTTHUMG000000	RAB5IF	**RAB5 interacting factor**	Cytoplasm	other		-1.025186625	0	0.90222611	0	1	0	9.47704951	9.86160054	9.69700552	9.61537397
2415	**ENSG0000010109**	NFATC2	protein_coding	chr20	51386957	51562831	-	Human GRCh	OTTHUMG000000	NFATC2	**nuclear factor of activated T cells 2**	Nucleus	transcription		1.225605057	0	0.51249007	0	1	0	8.00731987	8.49885203	7.9962125	8.83911152
2416	**ENSG0000010109**	RIMS4	protein_coding	chr20	44751808	44810546	-	Human GRCh	OTTHUMG000000	RIMS4	**regulating synaptic membrane exocytosis 4**	Plasma Mem	other		-1.351711069	0	0.36579805	0	1	0	5.36715856	5.98750371	5.59633449	5.20461267
2417	**ENSG0000010110**	PABPC1L	protein_coding	chr20	44910060	44959035	+	Human GRCh	OTTHUMG000000	PABPC1L	**poly(A) binding protein cytoplasmic 1 like**	Cytoplasm	other		-1.168591177	0	0.76880684	0	1	0	7.87375762	8.14856555	7.91347341	7.83157695
2418	**ENSG0000010110**	STK4	protein_coding	chr20	44966494	45080021	+	Human GRCh	OTTHUMG000000	STK4	**serine/threonine kinase 4**	Cytoplasm	kinase	XMU-MP-1	-1.06036761	0	0.77906232	0	1	0	10.6638698	11.0097543	10.8931582	10.6663366
2419	**ENSG0000010111**	SALL4	protein_coding	chr20	51782331	51802521	-	Human GRCh	OTTHUMG000000	SALL4	**spalt like transcription factor 4**	Nucleus	transcription		1.165238489	0	0.88942647	0	NA	0	2.50965886	2.63011103	2.78882702	2.51689837
2420	**ENSG0000010112**	ADNP	protein_coding	chr20	50888916	50931437	-	Human GRCh	OTTHUMG000000	ADNP	**activity dependent neuroprotector homeobox**	Nucleus	transcription		-1.112486963	0	0.58266865	0	1	0	12.4422902	12.6627463	12.5480551	12.3215985
2421	**ENSG0000010113**	PFDN4	protein_coding	chr20	54208087	54228052	+	Human GRCh	OTTHUMG000000	PFDN4	**prefoldin subunit 4**	Cytoplasm	other		-1.253828036	0	0.32058941	0	1	0	8.67474781	9.16053737	8.75983335	8.62843961
2422	**ENSG0000010113**	DOK5	protein_coding	chr20	54475593	54651169	+	Human GRCh	OTTHUMG000000	DOK5	**docking protein 5**	Plasma Mem	other		1	0	1	0	1	0	1.57124704	1.60889538	1.60269836	1.57346615
2423	**ENSG0000010113**	CSTF1	protein_coding	chr20	56392371	56406362	+	Human GRCh	OTTHUMG000000	CSTF1	**cleavage stimulation factor subunit 1**	Nucleus	other		-1.150634976	0	0.45707417	0	1	0	10.1966162	10.4133274	10.1849902	10.1238657
2424	**ENSG0000010114**	BMP7	protein_coding	chr20	57168753	57266641	-	Human GRCh	OTTHUMG000000	BMP7	**bone morphogenetic protein 7**	Extracellular	growth facto		-9.459775004	-1	0.01428922	1	1	0	3.77117518	3.08041437	1.8004098	2.36397679
2425	**ENSG0000010114**	RAE1	protein_coding	chr20	57351223	57379211	+	Human GRCh	OTTHUMG000000	RAE1	**ribonucleic acid export 1**	Nucleus	other		-1.092199234	0	0.65346341	0	1	0	10.0070079	10.3996575	10.2384091	9.9912216
2426	**ENSG0000010115**	TPD52L2	protein_coding	chr20	63865228	63891545	+	Human GRCh	OTTHUMG000000	TPD52L2	**TPD52 like 2**	Cytoplasm	other		-1.068694352	0	0.75672539	0	1	0	11.3598016	11.6839281	11.6130716	11.2810172
2427	**ENSG0000010115**	DNAJC5	protein_coding	chr20	63895126	63936031	+	Human GRCh	OTTHUMG000000	DNAJC5	**DnaJ heat shock protein family (Hsp40) member C5**	Plasma Mem	other		-1.062440289	0	0.77736547	0	1	0	11.8176692	12.1095526	12.0320212	11.7619129
2428	**ENSG0000010115**	NELFCD	protein_coding	chr20	58981208	58995133	+	Human GRCh	OTTHUMG000000	NELFCD	**negative elongation factor complex member C/D**	Nucleus	other		-1.160748243	0	0.47691499	0	1	0	11.5649284	11.8782631	11.6808716	11.437537
2429	**ENSG0000010116**	CTSZ	protein_coding	chr20	58985686	59008238	-	Human GRCh	OTTHUMG000000	CTSZ	**cathepsin Z**	Cytoplasm	peptidase		-1.004184898	0	0.98102731	0	1	0	10.0402184	10.1551487	10.1892822	9.99156982
2430	**ENSG0000010116**	PRPF6	protein_coding	chr20	63981132	64033100	+	Human GRCh	OTTHUMG000000	PRPF6	**pre-mRNA processing factor 6**	Nucleus	transcription		-1.02383627	0	0.91088447	0	1	0	11.0965853	11.4704915	11.4236058	11.098889
2431	**ENSG0000010116**	TUBB1	protein_coding	chr20	59019429	59026654	+	Human GRCh	OTTHUMG000000	TUBB1	**tubulin beta 1 class VI**	Cytoplasm	other	docetaxel/	1.615933417	1	0.73580121	0	NA	0	2.10223622	2.72476812	2.48451947	3.08251902
2432	**ENSG0000010116**	PRELID3B	protein_coding	chr20	59033145	59042809	-	Human GRCh	OTTHUMG000000	PRELID3B	**PRELI domain containing 3B**	Cytoplasm	other		1.051686362	0	0.81329486	0	1	0	11.2473539	11.4800645	11.5198924	11.3223146
2433	**ENSG0000010118**	HRH3	protein_coding	chr20	62214960	62220278	-	Human GRCh	OTTHUMG000000	HRH3	**histamine receptor H3**	Plasma Mem	G-protein cou	pitolisant,	1.620096024	1	0.39434873	0	1	0	4.09480111	3.80712788	3.9932362	4.62798748
2434	**ENSG0000010118**	MTG2	protein_coding	chr20	62183028	62203568	+	Human GRCh	OTTHUMG000000	MTG2	**mitochondrial ribosome associated GTPase 2**	Cytoplasm	enzyme		1.101322109	0	0.62457809	0	1	0	10.0634278	10.2433122	10.3585796	10.1596664
2435	**ENSG0000010118**	PSMA7	protein_coding	chr20	62136733	62143440	-	Human GRCh	OTTHUMG000000	PSMA7	**proteasome 20S subunit alpha 7**	Cytoplasm	peptidase		-1.064343878	0	0.77901777	0	1	0	11.106618	11.5485662	11.382577	11.1637973
2436	**ENSG0000010118**	SLCO4A1	protein_coding	chr20	62642503	62685785	+	Human GRCh	OTTHUMG000000	SLCO4A1	**solute carrier organic anion transporter family member 4A1**	Plasma Mem	transporter		1.124888977	0	0.71836131	0	1	0	9.07734436	9.28585914	9.5309724	9.05174599
2438	**ENSG0000010118**	MRGBP	protein_coding	chr20	62796473	62801729	+	Human GRCh	OTTHUMG000000	MRGBP	**MRG domain binding protein**	Nucleus	other		-1.011208271	0	0.95844313	0	1	0	9.42107224	9.76737129	9.71390326	9.4616347

2439	**ENSG0000010119**	TCFL5	protein_coding	chr20	62841005	62861822	-	Human GRCh	OTTHUMG000000	TCFL5	**transcription factor like 5**	Nucleus	transcription		-1.073058203	0	0.74567401	0	1	0	8.66411412	9.08164226	8.88919746	8.73347753
2440	**ENSG0000010119**	DIDO1	protein_coding	chr20	62877738	62937952	-	Human GRCh	OTTHUMG000000	DIDO1	**death inducer-obliterator 1**	Nucleus	other		-1.187909669	0	0.39378142	0	1	0	12.3559434	12.661195	12.4610244	12.1748628
2441	**ENSG0000010119**	GID8	protein_coding	chr20	62938147	62948475	+	Human GRCh	OTTHUMG000000	GID8	**GID complex subunit 8 homolog**	Nucleus	other		-1.125284311	0	0.57086456	0	1	0	10.859621	11.3083813	11.0662111	10.8770043
2442	**ENSG0000010119**	SLC17A9	protein_coding	chr20	62952707	62969585	+	Human GRCh	OTTHUMG000000	SLC17A9	**solute carrier family 17 member 9**	Cytoplasm	transporter		14.54242251	1	0.0001787	1	0.24	0	2.60666604	3.3771537	3.56805035	6.06162047
2444	**ENSG0000010119**	NKAIN4	protein_coding	chr20	63240784	63272694	-	Human GRCh	OTTHUMG000000	NKAIN4	**sodium/potassium transporting ATPase interacting 4**	Other	other		-5.887205899	-1	0.33256504	0	NA	0	1.28992836	1.71185161	1.3017262	1.29074697
2445	**ENSG0000010119**	ARFGAP1	protein_coding	chr20	63272785	63289790	+	Human GRCh	OTTHUMG000000	ARFGAP1	**ADP ribosylation factor GTPase activating protein 1**	Cytoplasm	enzyme		1.013385381	0	0.95284216	0	1	0	10.9778954	11.2843391	11.2949703	10.9979221
2446	**ENSG0000010120**	AVP	protein_coding	chr20	3082556	3084724	-	Human GRCh	OTTHUMG000000	AVP	**arginine vasopressin**	Plasma Mem	other		1	0	1	0	1	0	-0.0896151	-0.0123729	-0.024636	-0.0848704
2447	**ENSG0000010120**	COL20A1	protein_coding	chr20	63293186	63334851	+	Human GRCh	OTTHUMG000000	COL20A1	**collagen type XX alpha 1 chain**	Extracellular	other		1.149840157	0	0.8159004	0	1	0	5.54764461	5.48842359	5.82419487	5.46954185
2448	**ENSG0000010120**	CHRNA4	protein_coding	chr20	63343223	63378401	-	Human GRCh	OTTHUMG000000	CHRNA4	**cholinergic receptor nicotinic alpha 4 subunit**	Plasma Mem	transmembra	succinylch	-15.4423369	-1	0.03736539	1	1	0	3.08022484	2.1547015	2.14722362	2.11197823
2449	**ENSG0000010121**	EEF1A2	protein_coding	chr20	63488013	63499239	-	Human GRCh	OTTHUMG000000	EEF1A2	**eukaryotic translation elongation factor 1 alpha 2**	Cytoplasm	translation re		-1.387587162	0	0.49214769	0	1	0	10.5564435	10.6128097	10.3509046	10.0606305
2450	**ENSG0000010121**	PTK6	protein_coding	chr20	63528001	63537376	-	Human GRCh	OTTHUMG000000	PTK6	**protein tyrosine kinase 6**	Cytoplasm	kinase	vandetanib	2.873622629	1	0.08439668	0	1	0	5.23586426	5.18181943	5.48549435	6.72819728
2451	**ENSG0000010121**	GMEB2	protein_coding	chr20	63587602	63627101	-	Human GRCh	OTTHUMG000000	GMEB2	**glucocorticoid modulatory element binding protein 2**	Nucleus	transcription		-1.063144227	0	0.76317862	0	1	0	9.49495201	9.78006167	9.62380217	9.53377415
2452	**ENSG0000010122**	ADISSP	protein_coding	chr20	3753508	3767781	-	Human GRCh	OTTHUMG000000	ADISSP	**adipose secreted signaling protein**	Extracellular	other		1.318899359	0	0.18363733	0	1	0	9.725604	10.120935	10.3497357	10.1425769
2453	**ENSG0000010122**	SPEF1	protein_coding	chr20	3777504	3781448	-	Human GRCh	OTTHUMG000000	SPEF1	**sperm flagellar 1**	Cytoplasm	other		1.322549351	0	0.51513433	0	1	0	5.42684235	5.1612953	5.73340852	5.38467383
2454	**ENSG0000010122**	CDC25B	protein_coding	chr20	3786772	3806121	+	Human GRCh	OTTHUMG000000	CDC25B	**cell division cycle 25B**	Nucleus	phosphatase		-1.096798293	0	0.66732487	0	1	0	11.7028136	12.0426807	11.8443741	11.7224088
2455	**ENSG0000010123**	ISM1	protein_coding	chr20	13221274	13300651	+	Human GRCh	OTTHUMG000000	ISM1	**isthmin 1**	Other	other		1	0	1	0	1	0	1.55729299	1.65463707	1.63906215	1.56322289
2456	**ENSG0000010123**	RNF24	protein_coding	chr20	3927309	4015558	-	Human GRCh	OTTHUMG000000	RNF24	**ring finger protein 24**	Cytoplasm	other		-1.164687956	0	0.51898521	0	1	0	9.88888889	10.211239	9.94684168	9.83344852
2457	**ENSG0000010124**	ARFRP1	protein_coding	chr20	63698642	63708025	-	Human GRCh	OTTHUMG000000	ARFRP1	**ADP ribosylation factor related protein 1**	Cytoplasm	enzyme		1.093136828	0	0.68798487	0	1	0	10.4380827	10.6310534	10.7848697	10.4675567
2458	**ENSG0000010124**	NDUFAF5	protein_coding	chr20	13785007	13821580	+	Human GRCh	OTTHUMG000000	NDUFAF5	**NADH:ubiquinone oxidoreductase complex assembly factor 5**	Cytoplasm	other		1.112476623	0	0.62935888	0	1	0	8.77442444	9.1861991	9.26801771	8.93617696
2460	**ENSG0000010125**	TRIB3	protein_coding	chr20	362835	397559	+	Human GRCh	OTTHUMG000000	TRIB3	**tribbles pseudokinase 3**	Nucleus	kinase		-1.122259055	0	0.67216021	0	1	0	9.82650729	10.0922523	9.91401411	9.74838753
2461	**ENSG0000010126**	RASSF2	protein_coding	chr20	4780023	4823608	-	Human GRCh	OTTHUMG000000	RASSF2	**Ras association domain family member 2**	Nucleus	kinase		1.200691927	0	0.7411278	0	1	0	3.84194839	3.69443354	3.92789649	3.90328206
2462	**ENSG0000010126**	CSNK2A1	protein_coding	chr20	472498	543835	-	Human GRCh	OTTHUMG000000	CSNK2A1	**casein kinase 2 alpha 1**	Nucleus	kinase	SM1-71	-1.139496691	0	0.5058585	0	1	0	12.400927	12.6970338	12.5376331	12.2674947
2463	**ENSG0000010127**	SLC52A3	protein_coding	chr20	760080	776015	-	Human GRCh	OTTHUMG000000	SLC52A3	**solute carrier family 52 member 3**	Plasma Mem	transporter		2.609313968	1	0.05497143	0	1	0	3.93004656	3.93000941	4.82768275	4.63656961
2465	**ENSG0000010128**	RSPO4	protein_coding	chr20	958452	1002311	-	Human GRCh	OTTHUMG000000	RSPO4	**R-spondin 4**	Plasma Mem	other		-1.730194564	-1	0.27328205	0	1	0	4.27171243	4.36107789	3.72216824	3.91822793
2466	**ENSG0000010129**	CDS2	protein_coding	chr20	5126879	5197887	+	Human GRCh	OTTHUMG000000	CDS2	**CDP-diacylglycerol synthase 2**	Cytoplasm	enzyme		-1.213326337	0	0.33236528	0	1	0	11.0656045	11.3457545	11.1505074	10.8329869
2468	**ENSG0000010129**	HM13	protein_coding	chr20	31514428	31577923	+	Human GRCh	OTTHUMG000000	HM13	**histocompatibility minor 13**	Cytoplasm	peptidase		-1.136301054	0	0.53248057	0	1	0	11.9674449	12.1924794	12.0593394	11.808124
2469	**ENSG0000010129**	SNPH	protein_coding	chr20	1266280	1309328	+	Human GRCh	OTTHUMG000000	SNPH	**syntaphilin**	Plasma Mem	other		1.084183094	0	0.85841063	0	1	0	6.95972892	6.93899082	7.00161608	7.07019386
2470	**ENSG0000010130**	MYLK2	protein_coding	chr20	31819308	31834689	+	Human GRCh	OTTHUMG000000	MYLK2	**myosin light chain kinase 2**	Cytoplasm	kinase		-1.233316874	0	0.66215926	0	1	0	5.47397125	5.93826806	5.66308497	5.37660465
2472	**ENSG0000010131**	SEC23B	protein_coding	chr20	18507520	18561415	+	Human GRCh	OTTHUMG000000	SEC23B	**SEC23 homolog B, COPII coat complex component**	Extracellular	transporter		-1.081756362	0	0.73563457	0	1	0	11.5260325	11.9346129	11.7286912	11.5835894
2473	**ENSG0000010131**	FERMT1	protein_coding	chr20	6074845	6123214	-	Human GRCh	OTTHUMG000000	FERMT1	**FERM domain containing kindlin 1**	Plasma Mem	other		-1.537227704	-1	0.269807	0	1	0	5.55455567	5.7714679	4.92467884	5.56613353
2476	**ENSG0000010133**	CCM2L	protein_coding	chr20	32010438	32032180	+	Human GRCh	OTTHUMG000000	CCM2L	**CCM2 like scaffold protein**	Other	other		-33.46545045	-1	0.001497	1	0.82	0	3.11650173	3.75094088	2.32336788	2.26006787
2477	**ENSG0000010133**	PLCB4	protein_coding	chr20	9067825	9504593	+	Human GRCh	OTTHUMG000000	PLCB4	**phospholipase C beta 4**	Cytoplasm	enzyme		1.030270698	0	0.92808938	0	1	0	8.93048533	9.52954388	9.49864456	9.06365697
2478	**ENSG0000010133**	MYL9	protein_coding	chr20	36541497	36551447	+	Human GRCh	OTTHUMG000000	MYL9	**myosin light chain 9**	Cytoplasm	other		2.366596572	1	0.07855761	0	1	0	4.34013741	4.95495883	5.02967745	5.76040044
2480	**ENSG0000010133**	TM9SF4	protein_coding	chr20	32109714	32167258	+	Human GRCh	OTTHUMG000000	TM9SF4	**transmembrane 9 superfamily member 4**	Cytoplasm	transporter		-1.131226299	0	0.50992858	0	1	0	10.8611928	11.2120382	11.0299376	10.7833469
2481	**ENSG0000010134**	TLDC2	protein_coding	chr20	36876121	36894235	+	Human GRCh	OTTHUMG000000	TLDC2	**TBC/LysM-associated domain containing 2**	Other	other		-4.743476309	-1	0.04097779	1	1	0	4.13859341	4.04803111	3.10650627	3.02393489
2482	**ENSG0000010134**	CRNKL1	protein_coding	chr20	20034368	20056046	-	Human GRCh	OTTHUMG000000	CRNKL1	**crooked neck pre-mRNA splicing factor 1**	Nucleus	other		-1.065333663	0	0.79494159	0	1	0	9.46241736	9.87178038	9.76067555	9.45066474
2483	**ENSG0000010134**	POFUT1	protein_coding	chr20	32207855	32238658	+	Human GRCh	OTTHUMG000001	POFUT1	**protein O-fucosyltransferase 1**	Cytoplasm	enzyme		-1.084663023	0	0.69689531	0	1	0	10.8709546	11.1973343	11.1303921	10.7499457
2484	**ENSG0000010134**	SAMHD1	protein_coding	chr20	36890229	36951893	-	Human GRCh	OTTHUMG000000	SAMHD1	**SAM and HD domain containing deoxynucleoside triphosphate**	Nucleus	enzyme		1.067992109	0	0.78200827	0	1	0	9.12757741	9.52986177	9.43888851	9.39606945
2485	**ENSG0000010134**	PAK5	protein_coding	chr20	9537370	9839076	-	Human GRCh	OTTHUMG000000	PAK5	**p21 (RAC1) activated kinase 5**	Nucleus	kinase		1.40527906	0	0.69354164	0	1	0	1.53964708	2.65540607	2.23740776	2.51859158
2486	**ENSG0000010135**	KIF3B	protein_coding	chr20	32277651	32335011	+	Human GRCh	OTTHUMG000000	KIF3B	**kinesin family member 3B**	Cytoplasm	transporter		-1.185409613	0	0.38730352	0	1	0	11.4317973	11.6944165	11.4999985	11.2463672
2487	**ENSG0000010135**	MROH8	protein_coding	chr20	37101226	37179588	-	Human GRCh	OTTHUMG000000	MROH8	**maestro heat like repeat family member 8**	Other	other		-1.787311995	-1	0.23535944	0	1	0	5.28939885	5.83654946	5.24158552	4.83164058
2488	**ENSG0000010136**	NOP56	protein_coding	chr20	2652593	2658393	+	Human GRCh	OTTHUMG000000	NOP56	**NOP56 ribonucleoprotein**	Nucleus	other		1.037969386	0	0.83392042	0	1	0	11.9531745	12.150833	12.2552358	11.9164084
2489	**ENSG0000010136**	MANBAL	protein_coding	chr20	37289649	37317260	+	Human GRCh	OTTHUMG000000	MANBAL	**mannosidase beta like**	Other	other		-1.160904248	0	0.49511992	0	1	0	9.32110399	9.66225002	9.42772873	9.24561511
2490	**ENSG0000010136**	IDH3B	protein_coding	chr20	2658395	2664219	-	Human GRCh	OTTHUMG000000	IDH3B	**isocitrate dehydrogenase (NAD(+)) 3 non-catalytic subunit beta**	Cytoplasm	enzyme		-1.096254491	0	0.68780625	0	1	0	10.7750195	11.1886347	11.0212512	10.7548894
2491	**ENSG0000010136**	MAPRE1	protein_coding	chr20	32819954	32850405	+	Human GRCh	OTTHUMG000000	MAPRE1	**microtubule associated protein RP/EB family member 1**	Cytoplasm	other		-1.038371036	0	0.85289603	0	1	0	11.2823471	11.5658185	11.5443761	11.2145888
2492	**ENSG0000010138**	JAG1	protein_coding	chr20	10637684	10673999	-	Human GRCh	OTTHUMG000000	JAG1	**jagged canonical Notch ligand 1**	Extracellular	growth facto		-1.461414755	0	0.22360994	0	1	0	12.0066935	12.1589921	11.7064865	11.5794394
2493	**ENSG0000010139**	CDK5RAP1	protein_coding	chr20	33358839	33401561	-	Human GRCh	OTTHUMG000000	CDK5RAP1	**CDK5 regulatory subunit associated protein 1**	Extracellular	enzyme		-1.16013639	0	0.47521881	0	1	0	10.1036083	10.5297085	10.2708994	10.0623371
2494	**ENSG0000010140**	SNTA1	protein_coding	chr20	33407957	33443726	-	Human GRCh	OTTHUMG000000	SNTA1	**syntrophin alpha 1**	Plasma Mem	other		1.059962932	0	0.81990008	0	1	0	8.47413868	8.99447153	8.94479707	8.68309613
2496	**ENSG0000010140**	TTI1	protein_coding	chr20	37983007	38033461	-	Human GRCh	OTTHUMG000000	TTI1	**TELO2 interacting protein 1**	Cytoplasm	other		-1.113904747	0	0.58788884	0	1	0	10.2861083	10.4850306	10.421068	10.1017251
2497	**ENSG0000010141**	E2F1	protein_coding	chr20	33675477	33686385	-	Human GRCh	OTTHUMG000000	E2F1	**E2F transcription factor 1**	Nucleus	transcription		1.02478795	0	0.91024825	0	1	0	11.2336487	11.4630752	11.4008285	11.3607116
2498	**ENSG0000010141**	RPRD1B	protein_coding	chr20	38033725	38127780	+	Human GRCh	OTTHUMG000000	RPRD1B	**regulation of nuclear pre-mRNA domain containing 1B**	Nucleus	other		-1.147356535	0	0.51096856	0	1	0	10.6769411	10.9323997	10.7960542	10.5067573
2499	**ENSG0000010141**	PXMP4	protein_coding	chr20	33702758	33720319	-	Human GRCh	OTTHUMG000000	PXMP4	**peroxisomal membrane protein 4**	Cytoplasm	other		-1.089730661	0	0.71234898	0	1	0	9.51141478	9.89575789	9.68899635	9.54943097
2500	**ENSG0000010142**	CHMP4B	protein_coding	chr20	33811348	33854366	+	Human GRCh	OTTHUMG000000	CHMP4B	**charged multivesicular body protein 4B**	Cytoplasm	other		-1.037607171	0	0.86302674	0	1	0	9.8669563	10.2234261	10.0786851	9.95445876
2504	**ENSG0000010143**	CST3	protein_coding	chr20	23626706	23638473	-	Human GRCh	OTTHUMG000000	CST3	**cystatin C**	Extracellular	other		-1.135095813	0	0.58056797	0	1	0	10.401443	10.604913	10.4894778	10.2338753
2505	**ENSG0000010144**	ASIP	protein_coding	chr20	34194569	34269344	+	Human GRCh	OTTHUMG000000	ASIP	**agouti signaling protein**	Extracellular	other		1.060496581	0	0.96167651	0	NA	0	3.39662447	3.58220384	3.95186478	2.87782583
2506	**ENSG0000010144**	CST4	protein_coding	chr20	23685640	23689038	-	Human GRCh	OTTHUMG000000	CST4	**cystatin S**	Extracellular	other		1	0	1	0	NA	0	-0.3427941	-0.3365687	-0.3376038	-0.3424315
2507	**ENSG0000010144**	ACTR5	protein_coding	chr20	38748460	38772520	+	Human GRCh	OTTHUMG000000	ACTR5	**actin related protein 5**	Nucleus	other		-1.018533203	0	0.94221895	0	1	0	7.72713025	8.08700635	8.08424053	7.68381531
2508	**ENSG0000010144**	WFDC2	protein_coding	chr20	45469753	45481532	+	Human GRCh	OTTHUMG000000	WFDC2	**WAP four-disulfide core domain 2**	Extracellular	other		-1.001081321	0	0.99932049	0	1	0	3.15888878	2.76993111	2.75365513	3.16726139
2509	**ENSG0000010144**	AHCY	protein_coding	chr20	34280268	34311802	-	Human GRCh	OTTHUMG000001	AHCY	**adenosylhomocysteinase**	Cytoplasm	enzyme	9-(2',3'-dih	-1.156515852	0	0.49046096	0	1	0	11.9859782	12.2551284	12.1495463	11.7462081
2510	**ENSG0000010144**	PPP1R16B	protein_coding	chr20	38805697	38923024	+	Human GRCh	OTTHUMG000000	PPP1R16B	**protein phosphatase 1 regulatory subunit 16B**	Plasma Mem	phosphatase		1.304478953	0	0.7182818	0	1	0	3.94479479	3.34333009	2.15925982	4.52904956
2512	**ENSG0000010144**	FAM83D	protein_coding	chr20	38926312	38953106	+	Human GRCh	OTTHUMG000000	FAM83D	**family with sequence similarity 83 member D**	Cytoplasm	other		-1.128986853	0	0.62107942	0	1	0	10.0873945	10.4447814	10.2525803	10.0280769
2514	**ENSG0000010145**	DHX35	protein_coding	chr20	38962299	39039723	+	Human GRCh	OTTHUMG000000	DHX35	**DEAH-box helicase 35**	Nucleus	enzyme		-1.163730868	0	0.48553381	0	1	0	9.46518122	9.77801814	9.59082088	9.32781526
2515	**ENSG0000010145**	DNTTIP1	protein_coding	chr20	45791954	45811427	+	Human GRCh	OTTHUMG000000	DNTTIP1	**deoxynucleotidyltransferase terminal interacting protein 1**	Nucleus	ligand-depen		1.029850355	0	0.90274394	0	1	0	9.17039744	9.47860963	9.42967398	9.30100255
2516	**ENSG0000010146**	MAP1LC3A	protein_coding	chr20	34546854	34560345	+	Human GRCh	OTTHUMG000000	MAP1LC3A	**microtubule associated protein 1 light chain 3 alpha**	Cytoplasm	other		1.096419027	0	0.76108855	0	1	0	8.77303291	8.76703611	8.85263972	8.89076913
2517	**ENSG0000010146**	SYNDIG1	protein_coding	chr20	24469629	24666616	+	Human GRCh	OTTHUMG000000	SYNDIG1	**synapse differentiation inducing 1**	Plasma Mem	other		1	0	1	0	NA	0	0.69990064	0.73414778	0.7285586	0.70193892
2518	**ENSG0000010146**	PIGU	protein_coding	chr20	34560542	34698790	-	Human GRCh	OTTHUMG000000	PIGU	**phosphatidylinositol glycan anchor biosynthesis class U**	Cytoplasm	enzyme		-1.322419081	0	0.15480973	0	1	0	9.64200257	9.87915413	9.50000246	9.4174833
2519	**ENSG0000010147**	TNNC2	protein_coding	chr20	45823214	45833745	-	Human GRCh	OTTHUMG000000	TNNC2	**troponin C2, fast skeletal type**	Cytoplasm	other	Ca2+	2.48112127	1	0.3141819	0	1	0	2.88230277	2.33424503	2.98160377	3.25441125
2520	**ENSG0000010147**	ACOT8	protein_coding	chr20	45841721	45857405	-	Human GRCh	OTTHUMG000000	ACOT8	**acyl-CoA thioesterase 8**	Cytoplasm	enzyme		-1.16927324	0	0.48443884	0	1	0	8.82463888	9.26551545	8.92442799	8.86138431
2521	**ENSG0000010147**	APMAP	protein_coding	chr20	24962925	24992751	-	Human GRCh	OTTHUMG000000	APMAP	**adipocyte plasma membrane associated protein**	Plasma Mem	enzyme		-1.067868484	0	0.76977941	0	1	0	9.64829459	9.8785159	9.72107531	9.67994825
2522	**ENSG0000010148**	CELF4	protein_coding	chr18	37243040	37565827	-	Human GRCh	OTTHUMG000001	CELF4	**CUGBP Elav-like family member 4**	Nucleus	translation re		1.975909185	1	0.06827855	0	1	0	4.85828243	5.13771582	5.4410032	5.71902082
2523	**ENSG0000010149**	ZNF516	protein_coding	chr18	76357682	76495242	-	Human GRCh	OTTHUMG000001	ZNF516	**zinc finger protein 516**	Nucleus	transcription		1.304256837	0	0.63839268	0	1	0	2.5631115	3.25394932	2.72680727	3.5395432
2525	**ENSG0000010154**	ADNP2	protein_coding	chr18	80109262	80147523	+	Human GRCh	OTTHUMG000001	ADNP2	**ADNP homeobox 2**	Nucleus	transcription		-1.08995302	0	0.6548589	0	1	0	11.2858531	11.5097175	11.4474415	11.14586
2526	**ENSG0000010154**	RBFA	protein_coding	chr18	80034389	80050651	+	Human GRCh	OTTHUMG000001	RBFA	**ribosome binding factor A**	Cytoplasm	other		-1.086806086	0	0.68927701	0	1	0	10.0629149	10.3715496	10.2664904	9.98720599
2527	**ENSG0000010155**	USP14	protein_coding	chr18	158383	214629	+	Human GRCh	OTTHUMG000001	USP14	**ubiquitin specific peptidase 14**	Cytoplasm	peptidase	VLX1570	-1.080998663	0	0.68594698	0	1	0	11.7218764	12.0449901	11.9741059	11.6132817
2528	**ENSG0000010155**	VAPA	protein_coding	chr18	9914016	9960021	+	Human GRCh	OTTHUMG000001	VAPA	**VAMP associated protein A**	Plasma Mem	other		-1.006405247	0	0.97600038	0	1	0	11.7937809	12.0294202	12.0369147	11.7686831
2529	**ENSG0000010157**	METTL4	protein_coding	chr18	2537525	2571509	-	Human GRCh	OTTHUMG000001	METTL4	**methyltransferase 4, N6-adenosine**	Nucleus	enzyme		1.002311619	0	0.99251125	0	1	0	8.58163489	9.04634221	8.9521849	8.71391538
2530	**ENSG0000010157**	LPIN2	protein_coding	chr18	2885296	3013144	-	Human GRCh	OTTHUMG000001	LPIN2	**lipin 2**	Nucleus	phosphatase		-1.030176369	0	0.87621287	0	1	0	10.8754929	11.0827437	11.0205049	10.8765576
2531	**ENSG0000010159**	SMCHD1	protein_coding	chr18	2655726	2805017	+	Human GRCh	OTTHUMG000001	SMCHD1	**structural maintenance of chromosomes flexible hinge domain**	Nucleus	enzyme		1.027864688	0	0.89749374	0	1	0	12.5492608	12.8213149	12.9011862	12.5129834
2532	**ENSG0000010160**	MYOM1	protein_coding	chr18	3066807	3219968	-	Human GRCh	OTTHUMG000001	MYOM1	**myomesin 1**	Cytoplasm	other		1.54581643	1	0.21251071	0	1	0	5.6646425	5.65919056	6.15031539	6.07502174
2533	**ENSG0000010160**	MYL12A	protein_coding	chr18	3247481	3256236	+	Human GRCh	OTTHUMG000001	MYL12A	**myosin light chain 12A**	Cytoplasm	other		-1.119775793	0	0.63002947	0	1	0	10.9957479	11.3056766	11.0864286	10.9797222
2534	**ENSG0000010162**	CEP76	protein_coding	chr18	12661833	12702777	-	Human GRCh	OTTHUMG000001	CEP76	**centrosomal protein 76**	Cytoplasm	other		-1.14140941	0	0.56107524	0	1	0	8.62150431	8.78823651	8.64502633	8.48404888
2535	**ENSG0000010163**	ST8SIA5	protein_coding	chr18	46667821	46759257	-	Human GRCh	OTTHUMG000001	ST8SIA5	**ST8 alpha-N-acetyl-neuraminide alpha-2,8-sialyltransferase 5**	Cytoplasm	enzyme		-1.27436327	0	0.51517979	0	1	0	6.21820931	5.99153391	6.02418624	5.69076037
2536	**ENSG0000010163**	CEP192	protein_coding	chr18	12991362	13125052	+	Human GRCh	OTTHUMG000001	CEP192	**centrosomal protein 192**	Cytoplasm	other		-1.183995455	0	0.42353522	0	1	0	11.7219587	11.9702702	11.833391	11.4835352

2537	**ENSG0000010165**	RNMT	protein_coding	chr18	13726660	13764556	+	Human GRCh	OTTHUMG000001	RNMT	**RNA guanine-7 methyltransferase**	Nucleus	enzyme	5-azacytidi	-1.11977827	0	0.60051378	0	1	0	10.9221417	11.2947921	11.1464451	10.8272892
2538	**ENSG0000010166**	SMAD7	protein_coding	chr18	48919853	48950965	-	Human GRCh	OTTHUMG000001	SMAD7	**SMAD family member 7**	Nucleus	transcription		1.792819325	1	0.04590359	1	1	0	6.20075412	6.04662645	6.4498234	6.85675146
2539	**ENSG0000010167**	LIPG	protein_coding	chr18	49560699	49599185	+	Human GRCh	OTTHUMG000001	LIPG	**lipase G, endothelial type**	Extracellular	enzyme		1.113174886	0	0.75895997	0	1	0	7.53540048	8.00451658	8.02045018	7.77138674
2540	**ENSG0000010168**	LAMA1	protein_coding	chr18	6941742	7117797	-	Human GRCh	OTTHUMG000001	LAMA1	**laminin subunit alpha 1**	Extracellular	other		1.451475367	0	0.46640228	0	1	0	6.27875977	6.56355669	6.61413585	6.97077305
2541	**ENSG0000010169**	RNF125	protein_coding	chr18	32018825	32073219	+	Human GRCh	OTTHUMG000001	RNF125	**ring finger protein 125**	Cytoplasm	enzyme		-1.068061791	0	0.83815663	0	1	0	9.39138768	9.68440947	9.81146422	8.99432151
2542	**ENSG0000010174**	ANKRD12	protein_coding	chr18	9136228	9285985	+	Human GRCh	OTTHUMG000001	ANKRD12	**ankyrin repeat domain 12**	Nucleus	other		1.018134482	0	0.92445487	0	1	0	11.0074711	11.289467	11.2924915	11.0479472
2543	**ENSG0000010174**	NOL4	protein_coding	chr18	33851100	34224952	-	Human GRCh	OTTHUMG000001	NOL4	**nucleolar protein 4**	Nucleus	other		1.229012797	0	0.59239902	0	1	0	5.30261915	5.04212893	5.55911064	5.15075721
2544	**ENSG0000010175**	POLI	protein_coding	chr18	54269517	54321266	+	Human GRCh	OTTHUMG000001	POLI	**DNA polymerase iota**	Nucleus	enzyme		-1.154833392	0	0.51558841	0	1	0	10.2214531	10.3404243	10.3087024	9.90891197
2545	**ENSG0000010175**	MIB1	protein_coding	chr18	21704957	21870953	+	Human GRCh	OTTHUMG000001	MIB1	**MIB E3 ubiquitin protein ligase 1**	Cytoplasm	enzyme		1.025740483	0	0.89975279	0	1	0	11.7293021	11.8750875	12.0194021	11.6096745
2546	**ENSG0000010177**	RBBP8	protein_coding	chr18	22798261	23026488	+	Human GRCh	OTTHUMG000001	RBBP8	**RB binding protein 8, endonuclease**	Nucleus	enzyme		-1.016292631	0	0.93693081	0	1	0	10.0461624	10.2483469	10.2386304	10.0198939
2547	**ENSG0000010178**	RIOK3	protein_coding	chr18	23453287	23486603	+	Human GRCh	OTTHUMG000001	RIOK3	**RIO kinase 3**	Cytoplasm	kinase		1.017020186	0	0.93620452	0	1	0	10.7481281	11.0126274	11.0683234	10.7189459
2548	**ENSG0000010181**	CSTF2	protein_coding	chrX	100820391	100841520	+	Human GRCh	OTTHUMG000000	CSTF2	**cleavage stimulation factor subunit 2**	Nucleus	other		-1.082447159	0	0.71290467	0	1	0	9.38054854	9.62553738	9.60526738	9.20483894
2549	**ENSG0000010181**	H2BW2	protein_coding	chrX	104039956	104042454	+	Human GRCh	OTTHUMG000000	H2BW2	**H2B.W histone 2**	Nucleus	other		1	0	1	0	NA	0	0.8443751	0.8610224	0.85824972	0.84534303
2552	**ENSG0000010184**	PSMD10	protein_coding	chrX	108084207	108091549	-	Human GRCh	OTTHUMG000000	PSMD10	**proteasome 26S subunit, non-ATPase 10**	Cytoplasm	transcription		-1.122160441	0	0.62480702	0	1	0	9.54057541	9.74001292	9.65501961	9.36049436
2553	**ENSG0000010184**	ATG4A	protein_coding	chrX	108091668	108154671	+	Human GRCh	OTTHUMG000000	ATG4A	**autophagy related 4A cysteine peptidase**	Cytoplasm	peptidase		-1.092779195	0	0.7255604	0	1	0	8.32488118	8.56062573	8.43045889	8.26683621
2554	**ENSG0000010184**	STS	protein_coding	chrX	7147237	7804358	+	Human GRCh	OTTHUMG000000	STS	**steroid sulfatase**	Cytoplasm	enzyme	irosustat	-1.034314807	0	0.89713727	0	1	0	8.22283258	8.31568646	8.41045599	8.02798845
2555	**ENSG0000010184**	TBL1X	protein_coding	chrX	9463320	9741037	+	Human GRCh	OTTHUMG000000	TBL1X	**transducin beta like 1 X-linked**	Nucleus	transcription	tegavivint	-1.255502178	0	0.25904226	0	1	0	11.7325473	11.6391742	11.4783791	11.3848634
2556	**ENSG0000010185**	GPR143	protein_coding	chrX	9725346	9786297	-	Human GRCh	OTTHUMG000000	GPR143	**G protein-coupled receptor 143**	Plasma Mem	G-protein cou		4.108022001	1	0.02786589	1	1	0	3.52312597	3.12009416	4.45707401	4.11667346
2557	**ENSG0000010185**	PGRMC1	protein_coding	chrX	119236245	119244466	+	Human GRCh	OTTHUMG000000	PGRMC1	**progesterone receptor membrane component 1**	Plasma Mem	transmembra	CT1812	-1.017303901	0	0.93369293	0	1	0	10.5085533	10.731053	10.7634358	10.4244789
2558	**ENSG0000010186**	POLA1	protein_coding	chrX	24693873	24996986	+	Human GRCh	OTTHUMG000000	POLA1	**DNA polymerase alpha 1, catalytic subunit**	Nucleus	enzyme	clofarabine	-1.10660393	0	0.61527308	0	1	0	11.332879	11.550469	11.4589144	11.2072866
2559	**ENSG0000010187**	MID1	protein_coding	chrX	10445310	10833654	-	Human GRCh	OTTHUMG000000	MID1	**midline 1**	Nucleus	other		-1.235709924	0	0.47677376	0	1	0	8.75131001	9.05170439	8.8677801	8.47642637
2560	**ENSG0000010188**	NKAP	protein_coding	chrX	119920672	119943751	-	Human GRCh	OTTHUMG000000	NKAP	**NFKB activating protein**	Nucleus	transcription		-1.021910781	0	0.91755204	0	1	0	8.65418215	9.02576547	8.93181555	8.72015882
2561	**ENSG0000010188**	RHOXF1	protein_coding	chrX	120109051	120245267	-	Human GRCh		RHOXF1	**Rhox homeobox family member 1**	Nucleus	transcription		-1.032329316	0	0.92512159	0	1	0	6.30811282	6.42260063	6.53572789	6.09406506
2562	**ENSG0000010188**	NXT2	protein_coding	chrX	109535781	109544698	+	Human GRCh	OTTHUMG000000	NXT2	**nuclear transport factor 2 like export factor 2**	Nucleus	transporter		1.102775496	0	0.7408803	0	1	0	7.51740576	7.70389071	7.91379086	7.48097126
2566	**ENSG0000010190**	ALG13	protein_coding	chrX	111665811	111760649	+	Human GRCh	OTTHUMG000000	ALG13	**ALG13 UDP-N-acetylglucosaminyltransferase subunit**	Cytoplasm	enzyme		-1.042679023	0	0.84428299	0	1	0	10.3225072	10.4770773	10.5480945	10.1272427
2567	**ENSG0000010191**	PRPS2	protein_coding	chrX	12791355	12824222	+	Human GRCh	OTTHUMG000000	PRPS2	**phosphoribosyl pyrophosphate synthetase 2**	Cytoplasm	kinase		1.032510798	0	0.89336231	0	1	0	10.0098409	10.4482193	10.4581904	10.0789053
2569	**ENSG0000010192**	MOSPD1	protein_coding	chrX	134887632	134915257	-	Human GRCh	OTTHUMG000000	MOSPD1	**motile sperm domain containing 1**	Other	other		-1.095441256	0	0.70138529	0	1	0	7.9334528	8.0051113	8.09402459	7.61098444
2570	**ENSG0000010193**	AMMECR1	protein_coding	chrX	110194186	110440318	-	Human GRCh	OTTHUMG000000	AMMECR1	**AMMECR nuclear protein 1**	Nucleus	other		-1.021667062	0	0.92359194	0	1	0	9.87924422	10.207072	10.1210431	9.93339719
2571	**ENSG0000010193**	CHRDL1	protein_coding	chrX	110673856	110795819	-	Human GRCh	OTTHUMG000000	CHRDL1	**chordin like 1**	Extracellular	other		-2.000547996	-1	0.10556536	0	1	0	6.32698412	6.20393748	5.5355332	5.47390919
2572	**ENSG0000010194**	WDR13	protein_coding	chrX	48590042	48608869	+	Human GRCh	OTTHUMG000000	WDR13	**WD repeat domain 13**	Nucleus	other		-1.031277822	0	0.90294489	0	1	0	11.8496842	12.0326867	12.0225914	11.7842493
2573	**ENSG0000010194**	SUV39H1	protein_coding	chrX	48695554	48709016	+	Human GRCh	OTTHUMG000000	SUV39H1	**SUV39H1 histone lysine methyltransferase**	Nucleus	enzyme		-1.456305502	0	0.09485186	0	1	0	8.36637542	8.53270216	7.87867537	8.20592938
2574	**ENSG0000010195**	PAGE4	protein_coding	chrX	49829260	49834264	+	Human GRCh	OTTHUMG000000	PAGE4	**PAGE family member 4**	Nucleus	transcription		1.509179122	1	0.79422208	0	NA	0	1.70188012	2.72047655	3.22744101	1.04322999
2577	**ENSG0000010196**	XIAP	protein_coding	chrX	123859712	123913972	+	Human GRCh	OTTHUMG000000	XIAP	**X-linked inhibitor of apoptosis**	Cytoplasm	enzyme	TQB3728,	-1.056732659	0	0.78764727	0	1	0	11.1708639	11.2676302	11.3468223	10.9376605
2578	**ENSG0000010197**	STAG2	protein_coding	chrX	123960212	124422664	+	Human GRCh	OTTHUMG000000	STAG2	**STAG2 cohesin complex component**	Nucleus	other		-1.036619794	0	0.86795616	0	1	0	12.1723074	12.3930743	12.3996708	12.074668
2579	**ENSG0000010197**	ATP11C	protein_coding	chrX	139726346	139945276	-	Human GRCh	OTTHUMG000000	ATP11C	**ATPase phospholipid transporting 11C**	Plasma Mem	transporter		-1.044624886	0	0.84102517	0	1	0	10.668722	10.9350243	11.0012516	10.458847
2580	**ENSG0000010197**	MCF2	protein_coding	chrX	139581770	139708216	-	Human GRCh	OTTHUMG000000	MCF2	**MCF.2 cell line derived transforming sequence**	Cytoplasm	other		-1.630343473	-1	0.45304289	0	1	0	3.39192629	4.35208316	3.80091675	3.32051819
2582	**ENSG0000010198**	ABCD1	protein_coding	chrX	153724856	153744755	+	Human GRCh	OTTHUMG000000	ABCD1	**ATP binding cassette subfamily D member 1**	Plasma Mem	transporter		-1.004859798	0	0.9855907	0	1	0	8.04093792	8.35778403	8.35129414	8.03695438
2583	**ENSG0000010199**	CCDC22	protein_coding	chrX	49235470	49250520	+	Human GRCh	OTTHUMG000000	CCDC22	**coiled-coil domain containing 22**	Cytoplasm	other		1.007516751	0	0.97264338	0	1	0	8.65022788	8.97463145	8.84859873	8.81340604
2584	**ENSG0000010200**	CACNA1F	protein_coding	chrX	49205063	49233371	-	Human GRCh	OTTHUMG000000	CACNA1F	**calcium voltage-gated channel subunit alpha1 F**	Plasma Mem	ion channel	verapamil,	5.177283212	1	0.28961169	0	NA	0	1.59656733	1.61629208	1.61299925	1.97098202
2585	**ENSG0000010200**	SYP	protein_coding	chrX	49187815	49200218	-	Human GRCh	OTTHUMG000000	SYP	**synaptophysin**	Cytoplasm	transporter		1.229822539	0	0.44730909	0	1	0	7.24542917	7.50280583	7.63756739	7.53629214
2586	**ENSG0000010200**	PLP2	protein_coding	chrX	49171898	49175235	+	Human GRCh	OTTHUMG000000	PLP2	**proteolipid protein 2**	Cytoplasm	transporter		-1.482479881	0	0.5600548	0	1	0	5.27401316	5.52572709	4.77655304	5.42181422
2589	**ENSG0000010202**	PLS3	protein_coding	chrX	115561174	115650861	+	Human GRCh	OTTHUMG000000	PLS3	**plastin 3**	Cytoplasm	other		-1.130865197	0	0.60482986	0	1	0	9.88802138	10.2107881	9.90196087	9.9417431
2590	**ENSG0000010203**	NAA10	protein_coding	chrX	153929225	153935080	-	Human GRCh	OTTHUMG000000	NAA10	**N-alpha-acetyltransferase 10, NatA catalytic subunit**	Nucleus	enzyme		1.109077694	0	0.64610541	0	1	0	9.14939353	9.54521195	9.6855301	9.22701835
2591	**ENSG0000010203**	RENBP	protein_coding	chrX	153935269	153944687	-	Human GRCh	OTTHUMG000000	RENBP	**renin binding protein**	Cytoplasm	enzyme		1.286253642	0	0.31109834	0	1	0	8.06641261	8.42684152	8.55914434	8.51497123
2592	**ENSG0000010203**	ELF4	protein_coding	chrX	130063955	130110716	-	Human GRCh	OTTHUMG000000	ELF4	**E74 like ETS transcription factor 4**	Nucleus	transcription		-1.07830242	0	0.78359797	0	1	0	9.15678209	9.56942288	9.21977711	9.37794918
2593	**ENSG0000010203**	SMARCA1	protein_coding	chrX	129446501	129523500	-	Human GRCh	OTTHUMG000000	SMARCA1	**SWI/SNF related, matrix associated, actin dependent regulato**	Nucleus	transcription		-1.014524516	0	0.95680965	0	1	0	7.54354732	8.03019921	7.84173946	7.74864512
2594	**ENSG0000010204**	MTMR8	protein_coding	chrX	64268081	64395452	-	Human GRCh	OTTHUMG000000	MTMR8	**myotubularin related protein 8**	Nucleus	phosphatase		1.221571006	0	0.68881318	0	1	0	5.58197262	5.88720066	6.25591107	5.56773411
2595	**ENSG0000010204**	ASB9	protein_coding	chrX	15235288	15270467	-	Human GRCh	OTTHUMG000000	ASB9	**ankyrin repeat and SOCS box containing 9**	Nucleus	transcription		1.29367952	0	0.73017348	0	1	0	3.971413	3.46470169	3.92865664	4.02792816
2596	**ENSG0000010205**	ZC3H12B	protein_coding	chrX	65034788	65507887	+	Human GRCh	OTTHUMG000000	ZC3H12B	**zinc finger CCCH-type containing 12B**	Other	other		1.837424263	1	0.73795791	0	NA	0	0.5608539	1.06549306	1.05339039	0.99572753
2597	**ENSG0000010205**	RBBP7	protein_coding	chrX	16839283	16870362	-	Human GRCh	OTTHUMG000000	RBBP7	**RB binding protein 7, chromatin remodeling factor**	Nucleus	transcription		-1.139485733	0	0.53436601	0	1	0	11.583284	11.9851435	11.7473612	11.5602904
2599	**ENSG0000010205**	KCND1	protein_coding	chrX	48961378	48971844	-	Human GRCh	OTTHUMG000000	KCND1	**potassium voltage-gated channel subfamily D member 1**	Plasma Mem	ion channel	dalfampric	-1.399652685	0	0.33502788	0	1	0	6.95027127	7.39122226	6.82983503	6.84254448
2601	**ENSG0000010207**	SLC25A14	protein_coding	chrX	130339888	130373361	+	Human GRCh	OTTHUMG000000	SLC25A14	**solute carrier family 25 member 14**	Cytoplasm	transporter		-1.262548858	0	0.33480237	0	1	0	7.97656172	8.04098626	7.791083	7.72903844
2602	**ENSG0000010208**	FMR1	protein_coding	chrX	147911919	147951125	+	Human GRCh	OTTHUMG000000	FMR1	**fragile X messenger ribonucleoprotein 1**	Cytoplasm	translation re		1.015066612	0	0.94239424	0	1	0	10.8396192	11.0284235	11.1249014	10.7538913
2603	**ENSG0000010209**	PIM2	protein_coding	chrX	48913182	48919024	-	Human GRCh	OTTHUMG000000	PIM2	**Pim-2 proto-oncogene, serine/threonine kinase**	Nucleus	kinase	TP-3654, S	-1.051746583	0	0.8372397	0	1	0	9.39380406	9.86323653	9.70220973	9.47514336
2604	**ENSG0000010209**	SCML2	protein_coding	chrX	18239313	18354688	-	Human GRCh	OTTHUMG000000	SCML2	**Scm polycomb group protein like 2**	Nucleus	transcription		-1.03778974	0	0.88156455	0	1	0	10.0190492	10.3890402	10.4377334	9.84248317
2605	**ENSG0000010210**	SLC35A2	protein_coding	chrX	48903180	48911958	-	Human GRCh	OTTHUMG000000	SLC35A2	**solute carrier family 35 member A2**	Cytoplasm	enzyme		-1.022043044	0	0.92625655	0	1	0	9.21611879	9.42666838	9.46829201	9.10854978
2606	**ENSG0000010210**	PQBP1	protein_coding	chrX	48890197	48903402	+	Human GRCh	OTTHUMG000000	PQBP1	**polyglutamine binding protein 1**	Nucleus	transcription		-1.074494427	0	0.73840518	0	1	0	10.412624	10.7653799	10.6053232	10.4311313
2608	**ENSG0000010210**	PCSK1N	protein_coding	chrX	48831096	48835610	-	Human GRCh	OTTHUMG000000	PCSK1N	**proprotein convertase subtilisin/kexin type 1 inhibitor**	Extracellular	other		1.275508085	0	0.49496468	0	1	0	8.27280927	8.49606947	8.74664942	8.56049829
2609	**ENSG0000010211**	EMD	protein_coding	chrX	154379273	154381574	+	Human GRCh	OTTHUMG000000	EMD	**emerin**	Nucleus	other		-1.003205814	0	0.98784873	0	1	0	11.0219951	11.2958781	11.2135488	11.1106088
2610	**ENSG0000010212**	TAFAZZIN	protein_coding	chrX	154411524	154421726	+	Human GRCh	OTTHUMG000000	TAFAZZIN	**tafazzin, phospholipid-lysophospholipid transacylase**	Nucleus	enzyme		1.028564607	0	0.89166606	0	1	0	9.48309837	9.58472379	9.68851985	9.42845992
2611	**ENSG0000010212**	RAB40AL	protein_coding	chrX	102937272	102938300	+	Human GRCh	OTTHUMG000000	RAB40AL	**RAB40A like**	Plasma Mem	other		-1.352023043	0	0.47358576	0	1	0	5.52247548	5.90644607	5.49604128	5.38387209
2612	**ENSG0000010214**	PGK1	protein_coding	chrX	77910739	78129295	+	Human GRCh	OTTHUMG000000	PGK1	**phosphoglycerate kinase 1**	Cytoplasm	kinase	CBR-470-1	-1.054816848	0	0.82135795	0	1	0	12.9609783	13.4024556	13.2575046	13.0140744
2614	**ENSG0000010215**	MAGT1	protein_coding	chrX	77825747	77899271	-	Human GRCh	OTTHUMG000000	MAGT1	**magnesium transporter 1**	Plasma Mem	enzyme		-1.12185465	0	0.56839785	0	1	0	11.8666908	11.9851998	11.9462741	11.6293355
2615	**ENSG0000010217**	SMS	protein_coding	chrX	21940709	21994837	+	Human GRCh	OTTHUMG000000	SMS	**spermine synthase**	Cytoplasm	enzyme	(±)-2-hydr	-1.092040637	0	0.70126493	0	1	0	10.3296646	10.7081215	10.6003876	10.2459276
2616	**ENSG0000010217**	PHEX	protein_coding	chrX	22032325	22494713	+	Human GRCh	OTTHUMG000000	PHEX	**phosphate regulating endopeptidase X-linked**	Cytoplasm	peptidase		-1.268511929	0	0.57865526	0	1	0	5.94901734	6.87178802	6.35561213	6.14106681
2617	**ENSG0000010217**	UBL4A	protein_coding	chrX	154483717	154486615	-	Human GRCh	OTTHUMG000000	UBL4A	**ubiquitin like 4A**	Cytoplasm	enzyme		-1.175058674	0	0.44607236	0	1	0	10.1051279	10.4686161	10.2529395	9.98143122
2618	**ENSG0000010218**	CD99L2	protein_coding	chrX	150766336	150898816	-	Human GRCh	OTTHUMG000000	CD99L2	**CD99 molecule like 2**	Plasma Mem	other		-1.113928942	0	0.61177324	0	1	0	9.05912633	9.30516192	9.16975508	8.97192774
2619	**ENSG0000010218**	EEA1	protein_coding	chr12	92770637	92929331	-	Human GRCh	OTTHUMG000001	EEA1	**early endosome antigen 1**	Cytoplasm	other		1.105411053	0	0.64316899	0	1	0	10.552281	10.8046951	10.9552672	10.6132485
2620	**ENSG0000010219**	GPR50	protein_coding	chrX	151176584	151181465	+	Human GRCh	OTTHUMG000000	GPR50	**G protein-coupled receptor 50**	Plasma Mem	G-protein cou		9.939478631	1	0.60790778	0	NA	0	0.19034713	0.20141556	0.19957773	0.87929988
2621	**ENSG0000010221**	RP2	protein_coding	chrX	46837043	46882358	+	Human GRCh	OTTHUMG000000	RP2	**RP2 activator of ARL3 GTPase**	Cytoplasm	enzyme		-1.003066602	0	0.99062659	0	1	0	8.02290116	8.26662186	8.36985653	7.87373465
2622	**ENSG0000010222**	JADE3	protein_coding	chrX	46912276	47061242	+	Human GRCh	OTTHUMG000000	JADE3	**jade family PHD finger 3**	Nucleus	other		-1.111789174	0	0.6280942	0	1	0	9.76571912	9.8503072	9.85481618	9.51289003
2623	**ENSG0000010222**	CDK16	protein_coding	chrX	47217860	47229997	+	Human GRCh	OTTHUMG000000	CDK16	**cyclin dependent kinase 16**	Cytoplasm	kinase		-1.166873997	0	0.43491086	0	1	0	11.9447213	12.2746404	11.9938826	11.8992982
2624	**ENSG0000010222**	USP11	protein_coding	chrX	47232866	47248328	+	Human GRCh	OTTHUMG000000	USP11	**ubiquitin specific peptidase 11**	Nucleus	peptidase		-1.169749916	0	0.50494157	0	1	0	10.8084288	11.2432658	10.9631959	10.7754266
2625	**ENSG0000010223**	PCYT1B	protein_coding	chrX	24558087	24672677	-	Human GRCh	OTTHUMG000000	PCYT1B	**phosphate cytidylyltransferase 1B, choline**	Cytoplasm	enzyme		1.274791941	0	0.57701717	0	1	0	6.08229532	5.79565114	5.81331432	6.50622841
2626	**ENSG0000010223**	BRS3	protein_coding	chrX	136487947	136493780	+	Human GRCh	OTTHUMG000000	BRS3	**bombesin receptor subtype 3**	Plasma Mem	G-protein cou		1	0	1	0	NA	0	-0.8272473	-0.8254376	-0.8257424	-0.8271434
2627	**ENSG0000010224**	HTATSF1	protein_coding	chrX	136497079	136512346	+	Human GRCh	OTTHUMG000000	HTATSF1	**HIV-1 Tat specific factor 1**	Nucleus	transcription		-1.073537943	0	0.7322561	0	1	0	10.0433958	10.3506195	10.2943963	9.94365148
2630	**ENSG0000010226**	TIMP1	protein_coding	chrX	47582408	47586789	+	Human GRCh	OTTHUMG000000	TIMP1	**TIMP metallopeptidase inhibitor 1**	Extracellular	cytokine		-1.512650756	-1	0.64092012	0	1	0	6.12607574	6.07928917	4.8825151	6.10927256
2632	**ENSG0000010228**	GABRE	protein_coding	chrX	151953124	151974680	-	Human GRCh	OTTHUMG000000	GABRE	**gamma-aminobutyric acid type A receptor subunit epsilon**	Plasma Mem	ion channel	nitrazepan	1.664048515	1	0.15693042	0	1	0	6.94786477	7.2406227	7.34308157	7.97801007
2633	**ENSG0000010229**	PCDH11X	protein_coding	chrX	91779261	92623230	+	Human GRCh	OTTHUMG000000	PCDH11X	**protocadherin 11 X-linked**	Plasma Mem	other		1.523356833	1	0.61957579	0	1	0	4.66472904	4.13483468	5.43866903	3.86599145
2634	**ENSG0000010230**	FGD1	protein_coding	chrX	54445454	54496234	-	Human GRCh	OTTHUMG000000	FGD1	**FYVE, RhoGEF and PH domain containing 1**	Cytoplasm	other		1.441433066	0	0.15948759	0	1	0	7.95688651	8.28999025	8.71971741	8.25040619
2635	**ENSG0000010230**	PIN4	protein_coding	chrX	72181353	72302926	+	Human GRCh	OTTHUMG000000	PIN4	**peptidylprolyl cis/trans isomerase, NIMA-interacting 4**	Nucleus	enzyme		-1.007906023	0	0.97404281	0	1	0	7.73066499	8.22946462	8.02941272	7.96384193
2636	**ENSG0000010231**	PORCN	protein_coding	chrX	48508959	48520814	+	Human GRCh	OTTHUMG000000	PORCN	**porcupine O-acyltransferase**	Cytoplasm	enzyme	RXC004, XI	1.121614442	0	0.68859884	0	1	0	8.12359442	8.51299298	8.50221008	8.42045501
2638	**ENSG0000010231**	MAGED2	protein_coding	chrX	54807599	54816015	+	Human GRCh	OTTHUMG000000	MAGED2	**MAGE family member D2**	Plasma Mem	other		1.041682282	0	0.84788583	0	1	0	9.77426262	9.98831853	10.1368781	9.67729684

2639	**ENSG0000010231**	RBM3	protein_coding	chrX	48574449	48581162	+	Human GRCh	OTTHUMG000000	RBM3	**RNA binding motif protein 3**	Cytoplasm	other		1.030848542	0	0.89762165	0	1	0	12.4777548	12.854387	12.9421563	12.4316992
2640	**ENSG0000010234**	KLF8	protein_coding	chrX	56232356	56291531	+	Human GRCh	OTTHUMG000000	KLF8	**KLF transcription factor 8**	Nucleus	transcription		1.43756851	0	0.69063755	0	1	0	1.90838194	2.92568752	2.90315899	2.44698254
2642	**ENSG0000010236**	SYTL4	protein_coding	chrX	100671783	100732123	-	Human GRCh	OTTHUMG000000	SYTL4	**synaptotagmin like 4**	Cytoplasm	transporter		-1.246569302	0	0.49803885	0	1	0	10.52089	10.940802	10.8615998	9.98554847
2643	**ENSG0000010238**	ZDHHC15	protein_coding	chrX	75368427	75523502	-	Human GRCh	OTTHUMG000000	ZDHHC15	**zinc finger DHHC-type palmitoyltransferase 15**	Cytoplasm	enzyme		-1.476337668	0	0.55864058	0	1	0	3.67347006	3.32365815	3.71480194	2.05031839
2644	**ENSG0000010238**	CENPI	protein_coding	chrX	101098188	101166126	+	Human GRCh	OTTHUMG000000	CENPI	**centromere protein I**	Nucleus	other		-1.035232261	0	0.88537089	0	1	0	9.55831766	10.0657768	10.0118458	9.54571027
2645	**ENSG0000010238**	DRP2	protein_coding	chrX	101219769	101264502	+	Human GRCh	OTTHUMG000000	DRP2	**dystrophin related protein 2**	Plasma Mem	other		-1.261345616	0	0.55790001	0	1	0	5.08147531	6.15875842	5.62749423	5.40091123
2646	**ENSG0000010238**	TAF7L	protein_coding	chrX	101268253	101293057	-	Human GRCh	OTTHUMG000000	TAF7L	**TATA-box binding protein associated factor 7 like**	Nucleus	transcription		-7.229526334	-1	0.06745448	0	1	0	3.00876563	2.96088515	1.93754109	2.33163783
2647	**ENSG0000010239**	PBDC1	protein_coding	chrX	76173040	76178314	+	Human GRCh	OTTHUMG000000	PBDC1	**polysaccharide biosynthesis domain containing 1**	Other	other		1.032656448	0	0.88494785	0	1	0	7.89118941	8.2875789	8.3099335	7.94285023
2648	**ENSG0000010239**	GLA	protein_coding	chrX	101393273	101408012	-	Human GRCh	OTTHUMG000000	GLA	**galactosidase alpha**	Cytoplasm	enzyme	migalastat	-1.144977903	0	0.56959709	0	1	0	9.14599773	9.51809333	9.28528903	9.09809435
2649	**ENSG0000010240**	ARMCX3	protein_coding	chrX	101622797	101627843	+	Human GRCh	OTTHUMG000000	ARMCX3	**armadillo repeat containing X-linked 3**	Cytoplasm	other		1.147146016	0	0.5222385	0	1	0	9.52938635	9.65915211	9.93693873	9.52315633
2650	**ENSG0000010240**	BEX4	protein_coding	chrX	103215108	103217246	+	Human GRCh	OTTHUMG000000	BEX4	**brain expressed X-linked 4**	Cytoplasm	other		-1.096564614	0	0.72740768	0	1	0	9.14430416	9.11251028	9.31540301	8.64056649
2651	**ENSG0000010244**	RUBCNL	protein_coding	chr13	46334681	46438190	-	Human GRCh	OTTHUMG000000	RUBCNL	**rubicon like autophagy enhancer**	Cytoplasm	other		5.268614504	1	0.2187657	0	1	0	1.65686332	1.7204258	1.71010001	2.23111714
2652	**ENSG0000010245**	NALCN	protein_coding	chr13	101053776	101416508	-	Human GRCh	OTTHUMG000000	NALCN	**sodium leak channel, non-selective**	Plasma Mem	ion channel		-1.197735707	0	0.9388169	0	NA	0	0.75454357	1.67288732	0.77387475	1.56724941
2653	**ENSG0000010246**	FGF14	protein_coding	chr13	101710804	102402457	-	Human GRCh	OTTHUMG000000	FGF14	**fibroblast growth factor 14**	Extracellular	growth facto		1	0	1	0	1	0	0.67508427	0.72199297	0.71436483	0.67788793
2655	**ENSG0000010247**	NDFIP2	protein_coding	chr13	79481155	79556077	+	Human GRCh	OTTHUMG000000	NDFIP2	**Nedd4 family interacting protein 2**	Cytoplasm	other		1.039785053	0	0.86254594	0	1	0	10.2649526	10.5682423	10.7059682	10.1765146
2656	**ENSG0000010252**	TNFSF13B	protein_coding	chr13	108251240	108308484	+	Human GRCh	OTTHUMG000000	TNFSF13B	**TNF superfamily member 13b**	Extracellular	cytokine	belimumal	-1.316055633	0	0.65686328	0	1	0	3.72984983	3.88038627	3.69801131	3.53141386
2657	**ENSG0000010253**	FNDC3A	protein_coding	chr13	48975912	49209779	+	Human GRCh	OTTHUMG000000	FNDC3A	**fibronectin type III domain containing 3A**	Cytoplasm	other		-1.159075186	0	0.48885095	0	1	0	11.1423719	11.3866321	11.2999194	10.8738333
2659	**ENSG0000010254**	CDADC1	protein_coding	chr13	49247925	49293485	+	Human GRCh	OTTHUMG000000	CDADC1	**cytidine and dCMP deaminase domain containing 1**	Other	kinase		-1.193148259	0	0.43448874	0	1	0	7.77769916	7.85403341	7.76233396	7.47203358
2660	**ENSG0000010254**	CAB39L	protein_coding	chr13	49308650	49444064	-	Human GRCh	OTTHUMG000000	CAB39L	**calcium binding protein 39 like**	Cytoplasm	kinase		-1.410571602	0	0.3627722	0	1	0	6.77311034	6.90604386	6.67184113	6.26457153
2661	**ENSG0000010255**	KLF5	protein_coding	chr13	73054976	73077541	+	Human GRCh	OTTHUMG000000	KLF5	**KLF transcription factor 5**	Nucleus	transcription		-1.01743943	0	0.9560635	0	1	0	11.5402164	11.8597317	11.6400464	11.7401848
2662	**ENSG0000010257**	STK24	protein_coding	chr13	98445185	98577940	-	Human GRCh	OTTHUMG000000	STK24	**serine/threonine kinase 24**	Cytoplasm	kinase	bosutinib,	-1.145711689	0	0.48347291	0	1	0	11.2378882	11.5310017	11.3047162	11.1882962
2663	**ENSG0000010257**	ACP5	protein_coding	chr19	11574653	11579993	-	Human GRCh	OTTHUMG000001	ACP5	**acid phosphatase 5, tartrate resistant**	Cytoplasm	phosphatase		5.868615337	1	0.14256021	0	NA	0	1.82853904	1.89892601	2.69594249	2.81884763
2664	**ENSG0000010258**	DNAJC3	protein_coding	chr13	95677139	95794988	+	Human GRCh	OTTHUMG000000	DNAJC3	**DnaJ heat shock protein family (Hsp40) member C3**	Cytoplasm	other		-1.091734779	0	0.7351501	0	1	0	12.2565099	12.4358686	12.3266652	12.1670089
2665	**ENSG0000010259**	UGGT2	protein_coding	chr13	95801580	96053482	-	Human GRCh	OTTHUMG000000	UGGT2	**UDP-glucose glycoprotein glucosyltransferase 2**	Cytoplasm	enzyme		-1.03657952	0	0.86707895	0	1	0	10.631079	10.8343117	10.8788147	10.4838953
2666	**ENSG0000010260**	ARHGEF7	protein_coding	chr13	111114559	111305737	+	Human GRCh	OTTHUMG000000	ARHGEF7	**Rho guanine nucleotide exchange factor 7**	Cytoplasm	other		-1.149854875	0	0.50088557	0	1	0	11.227346	11.4871891	11.3496827	11.0586059
2667	**ENSG0000010267**	FGF9	protein_coding	chr13	21671073	21704498	+	Human GRCh	OTTHUMG000000	FGF9	**fibroblast growth factor 9**	Extracellular	growth facto		7.928379291	1	0.01474872	1	1	0	3.04352558	3.58011123	4.46724431	3.98845098
2669	**ENSG0000010269**	PARP4	protein_coding	chr13	24420931	24512778	-	Human GRCh	OTTHUMG000000	PARP4	**poly(ADP-ribose) polymerase family member 4**	Cytoplasm	enzyme		-1.149645657	0	0.49706611	0	1	0	10.7320897	10.9372895	10.7474796	10.6168798
2670	**ENSG0000010271**	SUPT20H	protein_coding	chr13	37009312	37059713	-	Human GRCh	OTTHUMG000000	SUPT20H	**SPT20 homolog, SAGA complex component**	Nucleus	other		-1.040170808	0	0.8496281	0	1	0	11.3153978	11.5440701	11.5675641	11.1841539
2671	**ENSG0000010273**	MRPS31	protein_coding	chr13	40729128	40771190	-	Human GRCh	OTTHUMG000000	MRPS31	**mitochondrial ribosomal protein S31**	Cytoplasm	other		-1.068122715	0	0.78534166	0	1	0	8.62120168	8.79592845	8.83207702	8.40921528
2672	**ENSG0000010274**	SLC25A15	protein_coding	chr13	40789412	40812460	+	Human GRCh	OTTHUMG000000	SLC25A15	**solute carrier family 25 member 15**	Cytoplasm	transporter		-1.197549729	0	0.38600182	0	1	0	9.87068932	10.1665271	9.89357883	9.75100489
2673	**ENSG0000010275**	KPNA3	protein_coding	chr13	49699320	49792682	-	Human GRCh	OTTHUMG000000	KPNA3	**karyopherin subunit alpha 3**	Nucleus	transporter		-1.039317893	0	0.84871729	0	1	0	10.6181375	11.0602959	10.9464753	10.6698415
2674	**ENSG0000010275**	FLT1	protein_coding	chr13	28300346	28495145	-	Human GRCh	OTTHUMG000000	FLT1	**fms related receptor tyrosine kinase 1**	Plasma Mem	kinase	imatinib/s	-1.009755875	0	0.99075323	0	1	0	4.38524994	3.71460182	3.82615669	4.30752689
2675	**ENSG0000010276**	RGCC	protein_coding	chr13	41457550	41470871	+	Human GRCh	OTTHUMG000000	RGCC	**regulator of cell cycle**	Cytoplasm	other		2.52686586	1	0.21253696	0	1	0	3.21506664	2.95123409	3.72449837	3.70186437
2676	**ENSG0000010276**	VWA8	protein_coding	chr13	41566835	41961120	-	Human GRCh	OTTHUMG000000	VWA8	**von Willebrand factor A domain containing 8**	Cytoplasm	enzyme		-1.135381938	0	0.53716547	0	1	0	10.3406216	10.5802045	10.4681072	10.1612906
2677	**ENSG0000010278**	DGKH	protein_coding	chr13	42040036	42256578	+	Human GRCh	OTTHUMG000000	DGKH	**diacylglycerol kinase eta**	Cytoplasm	kinase		1.155084238	0	0.60305453	0	1	0	9.83883009	9.93861813	10.1066234	9.9624585
2678	**ENSG0000010278**	KATNAL1	protein_coding	chr13	30202630	30307551	-	Human GRCh	OTTHUMG000000	KATNAL1	**katanin catalytic subunit A1 like 1**	Cytoplasm	enzyme		-1.106577772	0	0.70279672	0	1	0	8.14977242	8.29104377	8.14388909	8.0935463
2679	**ENSG0000010278**	INTS6	protein_coding	chr13	51354077	51454264	-	Human GRCh	OTTHUMG000000	INTS6	**integrator complex subunit 6**	Nucleus	enzyme		1.091657558	0	0.69536324	0	1	0	11.6977701	11.942635	12.0882954	11.7352628
2681	**ENSG0000010279**	DHRS12	protein_coding	chr13	51767993	51804163	-	Human GRCh	OTTHUMG000000	DHRS12	**dehydrogenase/reductase 12**	Nucleus	other		-1.490910822	0	0.17496733	0	1	0	7.16270732	7.5834875	7.17196707	6.73017488
2683	**ENSG0000010280**	TSC22D1	protein_coding	chr13	44432143	44577147	-	Human GRCh	OTTHUMG000000	TSC22D1	**TSC22 domain family member 1**	Nucleus	transcription		-1.197741478	0	0.47649717	0	1	0	12.7874502	12.9580537	12.9015936	12.3822692
2684	**ENSG0000010280**	CLN5	protein_coding	chr13	76990660	77019143	+	Human GRCh	OTTHUMG000000	CLN5	**CLN5 intracellular trafficking protein**	Cytoplasm	enzyme		1.080335171	0	0.76308244	0	1	0	9.11060705	9.34716755	9.40446236	9.22918601
2685	**ENSG0000010283**	OLFM4	protein_coding	chr13	53028813	53052057	+	Human GRCh	OTTHUMG000000	OLFM4	**olfactomedin 4**	Extracellular	other		1	0	1	0	NA	0	0.55599778	0.56607721	0.5643905	0.55658066
2686	**ENSG0000010285**	MSLN	protein_coding	chr16	760734	768865	+	Human GRCh	OTTHUMG000000	MSLN	**mesothelin**	Extracellular	other	amatuxima	-1.974215548	-1	0.52384281	0	NA	0	2.38360045	2.44183033	1.71331751	2.2331998
2687	**ENSG0000010285**	MGRN1	protein_coding	chr16	4616493	4690974	+	Human GRCh	OTTHUMG000001	MGRN1	**mahogunin ring finger 1**	Cytoplasm	enzyme		-1.052741053	0	0.80128016	0	1	0	11.9722177	12.1818949	12.1189863	11.9222881
2688	**ENSG0000010287**	ZNF629	protein_coding	chr16	30778456	30787205	-	Human GRCh	OTTHUMG000001	ZNF629	**zinc finger protein 629**	Nucleus	transcription		-1.120966713	0	0.54230074	0	1	0	10.1970101	10.3136725	10.2257548	10.0263575
2689	**ENSG0000010287**	TRADD	protein_coding	chr16	67154185	67159909	-	Human GRCh	OTTHUMG000001	TRADD	**TNFRSF1A associated via death domain**	Cytoplasm	other		-1.061239033	0	0.84574383	0	1	0	7.45535532	7.33912423	7.3503039	7.32129267
2690	**ENSG0000010287**	HSF4	protein_coding	chr16	67164681	67169945	+	Human GRCh	OTTHUMG000001	HSF4	**heat shock transcription factor 4**	Nucleus	transcription		-1.061022506	0	0.82952571	0	1	0	9.87670482	9.93569524	10.0007179	9.65379513
2691	**ENSG0000010287**	CORO1A	protein_coding	chr16	30182827	30189076	+	Human GRCh	OTTHUMG000001	CORO1A	**coronin 1A**	Cytoplasm	other		1.615193637	1	0.32356174	0	1	0	3.18861255	5.30926192	5.15935697	5.01173164
2692	**ENSG0000010288**	MAPK3	protein_coding	chr16	30114105	30123506	-	Human GRCh	OTTHUMG000001	MAPK3	**mitogen-activated protein kinase 3**	Cytoplasm	kinase	ulixertinib,	-1.080240379	0	0.72359342	0	1	0	10.0836516	10.4953984	10.3056198	10.132074
2693	**ENSG0000010288**	GDPD3	protein_coding	chr16	30104810	30113537	-	Human GRCh	OTTHUMG000001	GDPD3	**glycerophosphodiester phosphodiesterase domain containing**	Cytoplasm	enzyme		-1.0392876	0	0.9077096	0	1	0	6.09974042	6.76856259	6.44353133	6.43909659
2694	**ENSG0000010289**	ELMO3	protein_coding	chr16	67199111	67204029	+	Human GRCh	OTTHUMG000001	ELMO3	**engulfment and cell motility 3**	Cytoplasm	other		1.02855576	0	0.88671357	0	1	0	9.29056435	9.47318645	9.50159257	9.32427772
2696	**ENSG0000010289**	PHKB	protein_coding	chr16	47461123	47701523	+	Human GRCh	OTTHUMG000001	PHKB	**phosphorylase kinase regulatory subunit beta**	Cytoplasm	kinase		-1.112174405	0	0.58908401	0	1	0	10.7699491	11.0436901	10.8503552	10.7385381
2697	**ENSG0000010289**	LYRM1	protein_coding	chr16	20899868	20925006	+	Human GRCh	OTTHUMG000001	LYRM1	**LYR motif containing 1**	Cytoplasm	other		-1.051687478	0	0.82941757	0	1	0	9.4009734	9.55875415	9.609914	9.2086042
2698	**ENSG0000010289**	NUTF2	protein_coding	chr16	67846882	67872567	+	Human GRCh	OTTHUMG000001	NUTF2	**nuclear transport factor 2**	Nucleus	other		-1.157981578	0	0.48820249	0	1	0	11.4238093	11.7629681	11.5034734	11.3693625
2699	**ENSG0000010290**	NUP93	protein_coding	chr16	56730118	56850286	+	Human GRCh	OTTHUMG000001	NUP93	**nucleoporin 93**	Nucleus	other		-1.180880993	0	0.42513751	0	1	0	10.8667099	11.1943	10.9865556	10.7072604
2700	**ENSG0000010290**	CENPT	protein_coding	chr16	67828157	67847811	-	Human GRCh	OTTHUMG000001	CENPT	**centromere protein T**	Cytoplasm	other		1.03376415	0	0.86060819	0	1	0	10.7866447	10.9304027	10.9865288	10.8033565
2701	**ENSG0000010290**	TSNAXIP1	protein_coding	chr16	67806765	67832148	+	Human GRCh	OTTHUMG000001	TSNAXIP1	**translin associated factor X interacting protein 1**	Cytoplasm	other		1.215594063	0	0.72384771	0	1	0	4.72430023	5.0766994	5.26328162	4.92393074
2702	**ENSG0000010290**	NFAT5	protein_coding	chr16	69565094	69704666	+	Human GRCh	OTTHUMG000001	NFAT5	**nuclear factor of activated T cells 5**	Nucleus	transcription		-1.005860417	0	0.98296812	0	1	0	11.9602718	12.1461364	12.1698157	11.9173878
2703	**ENSG0000010291**	LONP2	protein_coding	chr16	48244300	48363122	+	Human GRCh	OTTHUMG000001	LONP2	**lon peptidase 2, peroxisomal**	Cytoplasm	peptidase		-1.110010143	0	0.62018158	0	1	0	11.8152596	12.038574	11.9509485	11.6609831
2704	**ENSG0000010292**	N4BP1	protein_coding	chr16	48538726	48620148	-	Human GRCh	OTTHUMG000001	N4BP1	**NEDD4 binding protein 1**	Cytoplasm	enzyme		-1.124808305	0	0.57443094	0	1	0	10.3237365	10.595484	10.4445482	10.2187873
2705	**ENSG0000010292**	CBLN1	protein_coding	chr16	49277917	49281838	-	Human GRCh	OTTHUMG000001	CBLN1	**cerebellin 1 precursor**	Cytoplasm	other		-9.976508466	-1	0.3895162	0	NA	0	1.88454807	0.96522484	0.9590233	0.92964717
2706	**ENSG0000010293**	ARL2BP	protein_coding	chr16	57245259	57253635	+	Human GRCh	OTTHUMG000001	ARL2BP	**ADP ribosylation factor like GTPase 2 binding protein**	Cytoplasm	transcription		-1.080506527	0	0.72430341	0	1	0	9.96348556	10.2034143	10.1344405	9.85401899
2707	**ENSG0000010293**	PLLP	protein_coding	chr16	57248547	57284672	-	Human GRCh	OTTHUMG000001	PLLP	**plasmolipin**	Plasma Mem	transporter		-1.300118928	0	0.48767746	0	1	0	5.55637452	5.60896751	5.508353	5.21560794
2708	**ENSG0000010293**	ZNF423	protein_coding	chr16	49487524	49857919	-	Human GRCh	OTTHUMG000001	ZNF423	**zinc finger protein 423**	Nucleus	transcription		1	0	1	0	1	0	0.64965719	0.7014949	0.69308169	0.65276244
2709	**ENSG0000010296**	CCL22	protein_coding	chr16	57358783	57366189	+	Human GRCh	OTTHUMG000001	CCL22	**C-C motif chemokine ligand 22**	Extracellular	cytokine		1.408286812	0	0.74742818	0	1	0	2.75511684	2.91767369	2.39425216	3.54163898
2710	**ENSG0000010296**	DHODH	protein_coding	chr16	72008588	72027664	+	Human GRCh	OTTHUMG000001	DHODH	**dihydroorotate dehydrogenase (quinone)**	Cytoplasm	enzyme	RP7214, le	1.070782505	0	0.75710717	0	1	0	9.2229475	9.57383748	9.65656867	9.29328561
2712	**ENSG0000010297**	CTCF	protein_coding	chr16	67562467	67639177	+	Human GRCh	OTTHUMG000001	CTCF	**CCCTC-binding factor**	Nucleus	transcription		-1.115941636	0	0.60769035	0	1	0	11.1514833	11.484357	11.3823477	11.0024699
2713	**ENSG0000010297**	ACD	protein_coding	chr16	67657512	67660810	-	Human GRCh	OTTHUMG000001	ACD	**ACD shelterin complex subunit and telomerase recruitment fa**	Nucleus	other		-1.020945857	0	0.92427995	0	1	0	9.44795369	9.8914124	9.77626729	9.54546864
2714	**ENSG0000010297**	POLR2C	protein_coding	chr16	57462660	57472009	+	Human GRCh	OTTHUMG000001	POLR2C	**RNA polymerase II subunit C**	Nucleus	enzyme		-1.06569156	0	0.77203012	0	1	0	10.2857496	10.7325054	10.5677353	10.3387264
2715	**ENSG0000010298**	PARD6A	protein_coding	chr16	67660946	67662774	+	Human GRCh	OTTHUMG000001	PARD6A	**par-6 family cell polarity regulator alpha**	Plasma Mem	other		1.242789298	0	0.59195339	0	1	0	7.82195542	8.29237136	8.4955973	8.1271595
2716	**ENSG0000010298**	ZNF821	protein_coding	chr16	71859680	71895336	-	Human GRCh	OTTHUMG000001	ZNF821	**zinc finger protein 821**	Other	other		-1.224512441	0	0.4227933	0	1	0	8.23552595	8.43189555	8.2110852	8.01605714
2717	**ENSG0000010299**	MMP15	protein_coding	chr16	58025754	58046901	+	Human GRCh	OTTHUMG000001	MMP15	**matrix metallopeptidase 15**	Extracellular	peptidase	marimasta	1.256967679	0	0.35814706	0	1	0	9.16298157	9.40583073	9.42195914	9.62090859
2718	**ENSG0000010300**	USB1	protein_coding	chr16	57999546	58021618	+	Human GRCh	OTTHUMG000001	USB1	**U6 snRNA biogenesis phosphodiesterase 1**	Nucleus	enzyme		1.081763152	0	0.72103749	0	1	0	9.34748375	9.75822333	9.58397057	9.72267928
2719	**ENSG0000010301**	CYB5B	protein_coding	chr16	69398402	69466264	+	Human GRCh	OTTHUMG000001	CYB5B	**cytochrome b5 type B**	Cytoplasm	enzyme		-1.133930311	0	0.5607028	0	1	0	11.2238459	11.6207098	11.3376736	11.2549524
2720	**ENSG0000010302**	CCDC113	protein_coding	chr16	58231157	58283836	+	Human GRCh	OTTHUMG000001	CCDC113	**coiled-coil domain containing 113**	Cytoplasm	other		-1.074952101	0	0.8027709	0	1	0	9.21598819	9.61598677	9.45164496	9.2390487
2722	**ENSG0000010302**	NME3	protein_coding	chr16	1770320	1771561	-	Human GRCh	OTTHUMG000001	NME3	**NME/NM23 nucleoside diphosphate kinase 3**	Cytoplasm	kinase		1.236508302	0	0.31765076	0	1	0	9.19832045	9.32713729	9.6404592	9.35327079
2723	**ENSG0000010303**	NDRG4	protein_coding	chr16	58462846	58513628	+	Human GRCh	OTTHUMG000001	NDRG4	**NDRG family member 4**	Plasma Mem	other		-1.115907447	0	0.72255803	0	1	0	11.7261603	11.9811266	11.7254322	11.7414409
2724	**ENSG0000010303**	PSMD7	protein_coding	chr16	74296814	74306288	+	Human GRCh	OTTHUMG000001	PSMD7	**proteasome 26S subunit, non-ATPase 7**	Cytoplasm	other		-1.194808059	0	0.36795049	0	1	0	10.228788	10.6845955	10.3579161	10.1961342
2725	**ENSG0000010303**	SETD6	protein_coding	chr16	58515479	58523842	+	Human GRCh	OTTHUMG000001	SETD6	**SET domain containing 6, protein lysine methyltransferase**	Nucleus	enzyme		-1.142389667	0	0.5138548	0	1	0	10.5283218	10.7135747	10.578154	10.3648127
2726	**ENSG0000010304**	SLC38A7	protein_coding	chr16	58665109	58684770	-	Human GRCh	OTTHUMG000001	SLC38A7	**solute carrier family 38 member 7**	Plasma Mem	transporter		-1.023168824	0	0.92025141	0	1	0	9.94388662	10.0868914	10.0861452	9.88892104
2727	**ENSG0000010304**	VAC14	protein_coding	chr16	70687439	70801160	-	Human GRCh	OTTHUMG000001	VAC14	**VAC14 component of PIKFYVE complex**	Cytoplasm	other		-1.042649449	0	0.84026813	0	1	0	10.6782676	10.9724338	10.8741413	10.6939004
2728	**ENSG0000010304**	HAS3	protein_coding	chr16	69105653	69118719	+	Human GRCh	OTTHUMG000001	HAS3	**hyaluronan synthase 3**	Plasma Mem	enzyme		-1.041913553	0	0.87947449	0	1	0	6.33870664	6.58967869	6.57218958	6.27028873
2729	**ENSG0000010304**	TANGO6	protein_coding	chr16	68843531	69085182	+	Human GRCh	OTTHUMG000001	TANGO6	**transport and golgi organization 6 homolog**	Other	other		-1.158693727	0	0.55097704	0	1	0	8.98560156	9.38619197	9.11702229	8.95861684
2730	**ENSG0000010305**	COG4	protein_coding	chr16	70480568	70523560	-	Human GRCh	OTTHUMG000001	COG4	**component of oligomeric golgi complex 4**	Cytoplasm	transporter		-1.209607843	0	0.35838111	0	1	0	10.6971478	11.0322541	10.7127853	10.6138199

2731	**ENSG0000010305**	SMPD3	protein_coding	chr16	68358327	68448508	-	Human GRCh	OTTHUMG000001	SMPD3	**sphingomyelin phosphodiesterase 3**	Cytoplasm	enzyme		1.203787284	0	0.54180347	0	1	0	8.18966382	8.22351229	8.44059367	8.3406255
2732	**ENSG0000010306**	SLC7A6OS	protein_coding	chr16	68284503	68310946	-	Human GRCh	OTTHUMG000001	SLC7A6OS	**solute carrier family 7 member 6 opposite strand**	Other	other		-1.129586603	0	0.55680166	0	1	0	9.64722294	9.83615247	9.71120624	9.50045874
2733	**ENSG0000010306**	SLC7A6	protein_coding	chr16	68264516	68301823	+	Human GRCh	OTTHUMG000001	SLC7A6	**solute carrier family 7 member 6**	Plasma Mem	transporter		1.066441627	0	0.77253141	0	1	0	10.3967668	10.5876191	10.6937073	10.4252215
2734	**ENSG0000010306**	PLA2G15	protein_coding	chr16	68245304	68261058	+	Human GRCh	OTTHUMG000001	PLA2G15	**phospholipase A2 group XV**	Cytoplasm	enzyme		1.085060719	0	0.7549061	0	1	0	8.12450039	8.3521378	8.43923217	8.21768048
2735	**ENSG0000010306**	ESRP2	protein_coding	chr16	68229033	68238102	-	Human GRCh	OTTHUMG000001	ESRP2	**epithelial splicing regulatory protein 2**	Nucleus	other		-1.138466241	0	0.59678723	0	1	0	12.0337407	12.3397128	12.1722329	11.912775
2736	**ENSG0000010308**	FA2H	protein_coding	chr16	74712955	74774831	-	Human GRCh	OTTHUMG000001	FA2H	**fatty acid 2-hydroxylase**	Cytoplasm	enzyme		-1.039719193	0	0.89851314	0	1	0	6.94784318	7.28176992	7.07961802	7.09120233
2737	**ENSG0000010309**	WDR59	protein_coding	chr16	74871362	75000173	-	Human GRCh	OTTHUMG000001	WDR59	**WD repeat domain 59**	Cytoplasm	transporter		-1.33102603	0	0.17145819	0	1	0	11.4157154	11.8514327	11.4836141	11.1415014
2738	**ENSG0000010311**	MON1B	protein_coding	chr16	77190835	77202398	+	Human GRCh	OTTHUMG000001	MON1B	**MON1 homolog B, secretory trafficking associated**	Cytoplasm	other		-1.110329885	0	0.70653125	0	1	0	10.6987013	10.6315018	10.6528479	10.4364098
2739	**ENSG0000010312**	CMC2	protein_coding	chr16	80966448	81020270	-	Human GRCh	OTTHUMG000001	CMC2	**C-X9-C motif containing 2**	Cytoplasm	other		-1.310677847	0	0.26602411	0	1	0	10.6655285	11.033322	10.7745077	10.3002844
2740	**ENSG0000010312**	AXIN1	protein_coding	chr16	287440	352723	-	Human GRCh	OTTHUMG000000	AXIN1	**axin 1**	Cytoplasm	other		1.055058925	0	0.77890566	0	1	0	10.8830005	11.0542241	11.1477974	10.9023756
2741	**ENSG0000010314**	HCFC1R1	protein_coding	chr16	3022625	3024286	-	Human GRCh	OTTHUMG000001	HCFC1R1	**host cell factor C1 regulator 1**	Nucleus	other		1.297682609	0	0.24847489	0	1	0	8.50757677	8.76964699	8.9593538	8.90275753
2742	**ENSG0000010314**	NPRL3	protein_coding	chr16	84271	138677	-	Human GRCh	OTTHUMG000000	NPRL3	**NPR3 like, GATOR1 complex subunit**	Cytoplasm	other		-1.090463055	0	0.6424355	0	1	0	10.6870004	10.9623751	10.6983643	10.7763202
2743	**ENSG0000010315**	MLYCD	protein_coding	chr16	83899115	83951445	+	Human GRCh	OTTHUMG000001	MLYCD	**malonyl-CoA decarboxylase**	Cytoplasm	enzyme		-1.100052967	0	0.70457594	0	1	0	9.51583084	9.42159754	9.3719392	9.35642641
2744	**ENSG0000010315**	MPG	protein_coding	chr16	77007	85851	+	Human GRCh	OTTHUMG000000	MPG	**N-methylpurine DNA glycosylase**	Nucleus	enzyme		1.163785343	0	0.64658616	0	1	0	8.4982345	8.7946265	8.82562313	8.80984699
2745	**ENSG0000010315**	NECAB2	protein_coding	chr16	83968244	84002776	+	Human GRCh	OTTHUMG000001	NECAB2	**N-terminal EF-hand calcium binding protein 2**	Cytoplasm	other		-2.557516797	-1	0.26228623	0	1	0	3.42173335	3.28798101	2.57410624	2.98803971
2746	**ENSG0000010316**	HSDL1	protein_coding	chr16	84122141	84145192	-	Human GRCh	OTTHUMG000001	HSDL1	**hydroxysteroid dehydrogenase like 1**	Cytoplasm	enzyme		-1.046557508	0	0.84793752	0	1	0	10.1259894	10.2339122	10.3598783	9.84585656
2747	**ENSG0000010316**	TAF1C	protein_coding	chr16	84177847	84187070	-	Human GRCh	OTTHUMG000001	TAF1C	**TATA-box binding protein associated factor, RNA polymerase I**	Nucleus	transcription		1.001182989	0	0.9954351	0	1	0	10.8678847	11.1676031	11.1619906	10.8780131
2748	**ENSG0000010317**	NAGPA	protein_coding	chr16	5024844	5034141	-	Human GRCh	OTTHUMG000000	NAGPA	**N-acetylglucosamine-1-phosphodiester alpha-N-acetylglucosa**	Cytoplasm	enzyme		-1.092756391	0	0.70960431	0	1	0	8.35239737	8.8123423	8.59527321	8.40872849
2750	**ENSG0000010318**	SEC14L5	protein_coding	chr16	4958330	5019157	+	Human GRCh	OTTHUMG000001	SEC14L5	**SEC14 like lipid binding 5**	Other	other		-1.116424844	0	0.85305079	0	1	0	5.44069334	6.06772224	5.48814866	5.85399828
2751	**ENSG0000010318**	COTL1	protein_coding	chr16	84565596	84618078	-	Human GRCh	OTTHUMG000001	COTL1	**coactosin like F-actin binding protein 1**	Cytoplasm	other		-1.189116498	0	0.54132798	0	1	0	8.00384514	8.40949385	8.12533446	8.02786909
2752	**ENSG0000010319**	USP10	protein_coding	chr16	84699986	84779922	+	Human GRCh	OTTHUMG000001	USP10	**ubiquitin specific peptidase 10**	Cytoplasm	peptidase		-1.200008039	0	0.35014608	0	1	0	11.4876028	11.7404803	11.5334678	11.2871512
2753	**ENSG0000010319**	CRISPLD2	protein_coding	chr16	84819985	84920768	+	Human GRCh	OTTHUMG000001	CRISPLD2	**cysteine rich secretory protein LCCL domain containing 2**	Cytoplasm	other		-1.241321556	0	0.4395249	0	1	0	6.99224176	6.82765074	6.81941885	6.56967038
2754	**ENSG0000010319**	TSC2	protein_coding	chr16	2047967	2089491	+	Human GRCh	OTTHUMG000001	TSC2	**TSC complex subunit 2**	Cytoplasm	other		-1.017517351	0	0.93793752	0	1	0	12.403635	12.6827183	12.6789601	12.3644312
2755	**ENSG0000010319**	ZNF500	protein_coding	chr16	4748239	4767624	-	Human GRCh	OTTHUMG000001	ZNF500	**zinc finger protein 500**	Nucleus	transcription		-1.121434013	0	0.58587479	0	1	0	9.91254642	10.2845157	10.1631784	9.77686364
2756	**ENSG0000010320**	NME4	protein_coding	chr16	396725	410367	+	Human GRCh	OTTHUMG000000	NME4	**NME/NM23 nucleoside diphosphate kinase 4**	Cytoplasm	kinase		1.140366096	0	0.66757652	0	1	0	5.96212911	6.1452527	5.98547859	6.31735034
2757	**ENSG0000010322**	ABCC1	protein_coding	chr16	15949138	16143257	+	Human GRCh	OTTHUMG000000	ABCC1	**ATP binding cassette subfamily C member 1**	Plasma Mem	transporter	sulfinpyraz	-1.2926232	0	0.63442386	0	1	0	13.8391239	14.0747685	13.8181823	13.4734297
2758	**ENSG0000010322**	NOMO3	protein_coding	chr16	16232506	16300806	+	Human GRCh	OTTHUMG000001	NOMO1 (incl	**NODAL modulator 1**	Plasma Mem	other		-1.076424367	0	0.77145065	0	1	0	7.9780565	8.25288021	8.27865069	7.74970072
2759	**ENSG0000010322**	LMF1	protein_coding	chr16	853634	981318	-	Human GRCh	OTTHUMG000000	LMF1	**lipase maturation factor 1**	Cytoplasm	other		-1.185780612	0	0.5598128	0	1	0	9.05924434	9.25216495	9.15804878	8.73840235
2760	**ENSG0000010324**	FOXF1	protein_coding	chr16	86510527	86515422	+	Human GRCh	OTTHUMG000001	FOXF1	**forkhead box F1**	Nucleus	transcription		1	0	1	0	1	0	1.64361557	1.69051224	1.68280134	1.64638286
2761	**ENSG0000010324**	CIAO3	protein_coding	chr16	729760	741329	-	Human GRCh	OTTHUMG000001	CIAO3	**cytosolic iron-sulfur assembly component 3**	Cytoplasm	other		1.092679139	0	0.71668568	0	1	0	10.4482558	10.6980551	10.8658396	10.4597216
2762	**ENSG0000010324**	MTHFSD	protein_coding	chr16	86530178	86555235	-	Human GRCh	OTTHUMG000001	MTHFSD	**methenyltetrahydrofolate synthetase domain containing**	Other	other		1.002912077	0	0.99010128	0	1	0	9.06422564	9.17909754	9.25775445	8.97833323
2763	**ENSG0000010324**	CLCN7	protein_coding	chr16	1444934	1475077	-	Human GRCh	OTTHUMG000000	CLCN7	**chloride voltage-gated channel 7**	Plasma Mem	ion channel		-1.080850609	0	0.70632911	0	1	0	11.4986709	11.716466	11.6704956	11.3631614
2764	**ENSG0000010325**	HAGHL	protein_coding	chr16	726936	735525	+	Human GRCh	OTTHUMG000001	HAGHL	**hydroxyacylglutathione hydrolase like**	Other	enzyme		1.080017743	0	0.745571	0	1	0	9.49561348	9.89193113	9.82250946	9.77391828
2765	**ENSG0000010325**	ANTKMT	protein_coding	chr16	720581	722590	+	Human GRCh	OTTHUMG000001	ANTKMT	**adenine nucleotide translocase lysine methyltransferase**	Cytoplasm	enzyme		1.337523847	0	0.23126636	0	1	0	7.75223139	7.91860185	8.29313578	7.98063694
2766	**ENSG0000010325**	SLC7A5	protein_coding	chr16	87830023	87869507	-	Human GRCh	OTTHUMG000001	SLC7A5	**solute carrier family 7 member 5**	Plasma Mem	transporter	QBS10072	-1.186479401	0	0.35975253	0	1	0	12.4685	12.163934	12.2401391	12.0095427
2767	**ENSG0000010326**	METRN	protein_coding	chr16	715118	719655	+	Human GRCh	OTTHUMG000000	METRN	**meteorin, glial cell differentiation regulator**	Extracellular	other		1.115212481	0	0.70659147	0	1	0	9.04126273	9.04380711	9.26835555	9.05442167
2768	**ENSG0000010326**	FBXO31	protein_coding	chr16	87326987	87392142	-	Human GRCh	OTTHUMG000001	FBXO31	**F-box protein 31**	Extracellular	enzyme		-1.03907038	0	0.85340997	0	1	0	10.3439031	10.6748025	10.4957983	10.4582618
2769	**ENSG0000010326**	STUB1	protein_coding	chr16	680224	682870	+	Human GRCh	OTTHUMG000001	STUB1	**STIP1 homology and U-box containing protein 1**	Cytoplasm	enzyme		1.060982957	0	0.77659151	0	1	0	11.4559834	11.7228845	11.732642	11.5925496
2770	**ENSG0000010326**	RHBDL1	protein_coding	chr16	675671	678268	+	Human GRCh	OTTHUMG000001	RHBDL1	**rhomboid like 1**	Plasma Mem	peptidase		1.469106087	0	0.2288379	0	1	0	8.10027601	8.48666891	8.72118267	8.75928726
2771	**ENSG0000010327**	NUBP1	protein_coding	chr16	10743786	10769351	+	Human GRCh	OTTHUMG000001	NUBP1	**NUBP iron-sulfur cluster assembly factor 1, cytosolic**	Cytoplasm	other		-1.108287036	0	0.70771549	0	1	0	8.84992126	9.3343799	9.19640071	8.77141177
2772	**ENSG0000010327**	UBE2I	protein_coding	chr16	1308880	1327018	+	Human GRCh	OTTHUMG000001	UBE2I	**ubiquitin conjugating enzyme E2 I**	Nucleus	enzyme		-1.117043882	0	0.57706683	0	1	0	12.0924437	12.4509884	12.2383881	12.0803705
2775	**ENSG0000010331**	CRYM	protein_coding	chr16	21238874	21303083	-	Human GRCh	OTTHUMG000000	CRYM	**crystallin mu**	Cytoplasm	enzyme		4.297454721	1	0.11035924	0	1	0	2.42484482	2.51042954	2.79626819	3.49203663
2776	**ENSG0000010331**	EEF2K	protein_coding	chr16	22206278	22288738	+	Human GRCh	OTTHUMG000000	EEF2K	**eukaryotic elongation factor 2 kinase**	Cytoplasm	kinase	TS-4, TX-1!	1.014643536	0	0.9446838	0	1	0	11.5686788	11.9192886	11.6031976	11.9220104
2777	**ENSG0000010332**	CAPN15	protein_coding	chr16	527712	554636	+	Human GRCh	OTTHUMG000001	CAPN15	**calpain 15**	Other	peptidase		1.121488584	0	0.57028468	0	1	0	11.6021089	11.9170548	11.9826368	11.8079557
2778	**ENSG0000010333**	PIEZO1	protein_coding	chr16	88715338	88785220	-	Human GRCh	OTTHUMG000001	PIEZO1	**piezo type mechanosensitive ion channel component 1**	Cytoplasm	ion channel		-1.381179223	0	0.4722028	0	1	0	10.5207764	10.4451473	10.2429656	10.0548441
2779	**ENSG0000010334**	GSPT1	protein_coding	chr16	11868128	11916082	-	Human GRCh	OTTHUMG000001	GSPT1	**G1 to S phase transition 1**	Cytoplasm	translation re		-1.099919736	0	0.61717958	0	1	0	12.4748566	12.8308381	12.6724878	12.4328007
2780	**ENSG0000010334**	ZNF174	protein_coding	chr16	3401215	3409364	+	Human GRCh	OTTHUMG000001	ZNF174	**zinc finger protein 174**	Nucleus	transcription		-1.167992871	0	0.50942594	0	1	0	8.88458382	9.07562737	8.88143911	8.73520198
2781	**ENSG0000010335**	CLUAP1	protein_coding	chr16	3500976	3539048	+	Human GRCh	OTTHUMG000001	CLUAP1	**clusterin associated protein 1**	Nucleus	other		-1.035402169	0	0.86644757	0	1	0	9.848398	10.0146962	10.0868588	9.6677484
2782	**ENSG0000010335**	UBFD1	protein_coding	chr16	23557721	23574389	+	Human GRCh	OTTHUMG000001	UBFD1	**ubiquitin family domain containing 1**	Other	other		-1.069017299	0	0.72870909	0	1	0	11.4543678	11.7485405	11.6260812	11.4378001
2784	**ENSG0000010335**	EARS2	protein_coding	chr16	23520754	23557731	-	Human GRCh	OTTHUMG000001	EARS2	**glutamyl-tRNA synthetase 2, mitochondrial**	Cytoplasm	enzyme		-1.11156534	0	0.6013926	0	1	0	10.6813402	11.0892374	10.9119806	10.6406069
2785	**ENSG0000010336**	ELOB	protein_coding	chr16	2771414	2777289	-	Human GRCh	OTTHUMG000001	ELOB	**elongin B**	Nucleus	transcription		-1.030047622	0	0.89381473	0	1	0	10.7328834	11.1216389	10.999358	10.8131626
2786	**ENSG0000010336**	GGA2	protein_coding	chr16	23463542	23521995	-	Human GRCh	OTTHUMG000000	GGA2	**golgi associated, gamma adaptin ear containing, ARF binding p**	Cytoplasm	transporter		-1.098645262	0	0.65847194	0	1	0	11.5290064	11.9461212	11.7828134	11.5030608
2788	**ENSG0000010338**	CPPED1	protein_coding	chr16	12659799	12803887	-	Human GRCh	OTTHUMG000001	CPPED1	**calcineurin like phosphoesterase domain containing 1**	Cytoplasm	enzyme		1.721376071	1	0.30540711	0	1	0	3.70863994	3.26658997	3.24557092	4.2340688
2789	**ENSG0000010340**	USP31	protein_coding	chr16	23061406	23149452	-	Human GRCh	OTTHUMG000000	USP31	**ubiquitin specific peptidase 31**	Nucleus	peptidase		1.002307488	0	0.9912759	0	1	0	10.4938001	10.7475411	10.7353343	10.5141364
2790	**ENSG0000010341**	HMOX2	protein_coding	chr16	4474690	4510347	+	Human GRCh	OTTHUMG000001	HMOX2	**heme oxygenase 2**	Cytoplasm	enzyme	tin mesop	-1.03054588	0	0.89567437	0	1	0	9.89294884	10.2571024	10.0520131	10.0572245
2791	**ENSG0000010342**	DNAJA3	protein_coding	chr16	4425805	4456775	+	Human GRCh	OTTHUMG000001	DNAJA3	**DnaJ heat shock protein family (Hsp40) member A3**	Cytoplasm	other		1.024195956	0	0.9100271	0	1	0	11.1970348	11.4164089	11.513677	11.1330575
2792	**ENSG0000010342**	BFAR	protein_coding	chr16	14632931	14669236	+	Human GRCh	OTTHUMG000001	BFAR	**bifunctional apoptosis regulator**	Cytoplasm	enzyme		-1.279336811	0	0.23803612	0	1	0	10.8367853	11.1546078	10.8538698	10.5860351
2793	**ENSG0000010344**	SALL1	protein_coding	chr16	51135982	51152334	-	Human GRCh	OTTHUMG000001	SALL1	**spalt like transcription factor 1**	Nucleus	transcription		1.915410267	1	0.54867841	0	1	0	1.05686019	2.15949464	2.42112698	1.6605295
2794	**ENSG0000010346**	TOX3	protein_coding	chr16	52436417	52547802	-	Human GRCh	OTTHUMG000001	TOX3	**TOX high mobility group box family member 3**	Nucleus	transcription		1.203379043	0	0.5247468	0	1	0	9.19923048	9.71389879	9.6966522	9.64904228
2795	**ENSG0000010347**	RBL2	protein_coding	chr16	53433977	53491648	+	Human GRCh	OTTHUMG000001	RBL2	**RB transcriptional corepressor like 2**	Nucleus	other		-1.077728026	0	0.71143922	0	1	0	11.019322	11.2672819	11.2022574	10.9136615
2796	**ENSG0000010348**	QPRT	protein_coding	chr16	29663279	29698699	+	Human GRCh	OTTHUMG000000	QPRT	**quinolinate phosphoribosyltransferase**	Cytoplasm	enzyme	atorvastati	3.90410479	1	0.12676719	0	1	0	2.78280124	2.33180231	2.31649301	3.63386266
2797	**ENSG0000010348**	XYLT1	protein_coding	chr16	17101769	17470960	-	Human GRCh	OTTHUMG000001	XYLT1	**xylosyltransferase 1**	Cytoplasm	enzyme		4.518789457	1	0.07205322	0	1	0	3.59841629	3.28211043	4.13438146	3.89679044
2799	**ENSG0000010349**	RPGRIP1L	protein_coding	chr16	53598153	53703938	-	Human GRCh	OTTHUMG000001	RPGRIP1L	**RPGRIP1 like**	Cytoplasm	other		-1.13203362	0	0.57221878	0	1	0	9.90228435	10.1352943	10.0462858	9.70109848
2800	**ENSG0000010349**	MAZ	protein_coding	chr16	29806106	29811164	+	Human GRCh	OTTHUMG000001	MAZ	**MYC associated zinc finger protein**	Nucleus	transcription		1.003571228	0	0.98520632	0	1	0	13.6496323	13.9702406	13.8786081	13.7680777
2801	**ENSG0000010349**	STX4	protein_coding	chr16	31032889	31042975	+	Human GRCh	OTTHUMG000001	STX4	**syntaxin 4**	Plasma Mem	transporter		1.105171622	0	0.73648469	0	1	0	8.9218189	9.62620401	9.58749569	9.26564399
2802	**ENSG0000010350**	CDIPT	protein_coding	chr16	29858357	29863414	-	Human GRCh	OTTHUMG000001	CDIPT	**CDP-diacylglycerol--inositol 3-phosphatidyltransferase**	Cytoplasm	enzyme		1.028462609	0	0.89924748	0	1	0	10.3435377	10.4155843	10.5548267	10.248537
2803	**ENSG0000010350**	BCKDK	protein_coding	chr16	31106107	31112791	+	Human GRCh	OTTHUMG000000	BCKDK	**branched chain keto acid dehydrogenase kinase**	Cytoplasm	kinase	BT2F, BT2	1.025227147	0	0.90244074	0	1	0	10.4583206	10.8381677	10.769964	10.6071748
2804	**ENSG0000010351**	KAT8	protein_coding	chr16	31114489	31131393	+	Human GRCh	OTTHUMG000001	KAT8	**lysine acetyltransferase 8**	Nucleus	enzyme		1.012712511	0	0.94784564	0	1	0	10.0342568	10.4432622	10.3835215	10.1447422
2805	**ENSG0000010351**	NOMO1	protein_coding	chr16	14833721	14896157	+	Human GRCh	OTTHUMG000000	NOMO1 (incl	**NODAL modulator 1**	Plasma Mem	other		-1.174937181	0	0.45167943	0	1	0	10.7963371	11.122306	10.9286613	10.6345102
2807	**ENSG0000010352**	SYT17	protein_coding	chr16	19167971	19268332	+	Human GRCh	OTTHUMG000001	SYT17	**synaptotagmin 17**	Plasma Mem	other		-1.099348202	0	0.80410231	0	1	0	8.76457934	8.62075338	8.87989739	8.20911125
2808	**ENSG0000010353**	TMC5	protein_coding	chr16	19410496	19499113	+	Human GRCh	OTTHUMG000001	TMC5	**transmembrane channel like 5**	Cytoplasm	other		1.615973205	1	0.30145674	0	1	0	6.46044302	6.86840799	7.27847638	7.10101976
2809	**ENSG0000010354**	CCP110	protein_coding	chr16	19523811	19553408	+	Human GRCh	OTTHUMG000001	CCP110	**centriolar coiled-coil protein 110**	Cytoplasm	other		-1.17320207	0	0.57487998	0	1	0	12.3635045	12.7252691	12.6568853	12.0075118
2810	**ENSG0000010354**	VPS35L	protein_coding	chr16	19555240	19706793	+	Human GRCh	OTTHUMG000001	VPS35L	**VPS35 endosomal protein sorting factor like**	Cytoplasm	other		1.049799216	0	0.8513752	0	1	0	9.30322071	9.88255466	9.6709042	9.68854159
2812	**ENSG0000010354**	RNF40	protein_coding	chr16	30761745	30776307	+	Human GRCh	OTTHUMG000001	RNF40	**ring finger protein 40**	Cytoplasm	enzyme		-1.017251276	0	0.92580748	0	1	0	12.0959658	12.4160031	12.3720277	12.1082599
2813	**ENSG0000010355**	KNOP1	protein_coding	chr16	19701937	19718235	-	Human GRCh	OTTHUMG000001	KNOP1	**lysine rich nucleolar protein 1**	Other	other		-1.044444766	0	0.81577429	0	1	0	10.6849467	11.0482664	10.9693587	10.6758214
2815	**ENSG0000010359**	AAGAB	protein_coding	chr15	67200667	67255195	-	Human GRCh	OTTHUMG000001	AAGAB	**alpha and gamma adaptin binding protein**	Cytoplasm	other		-1.175653833	0	0.44621712	0	1	0	10.487706	10.7956463	10.5712537	10.3621229
2816	**ENSG0000010359**	IQCH	protein_coding	chr15	67254786	67502260	+	Human GRCh	OTTHUMG000001	IQCH	**IQ motif containing H**	Other	other		-1.08446683	0	0.73704709	0	1	0	8.01064908	8.19699634	8.14968571	7.87453574
2817	**ENSG0000010364**	LACTB	protein_coding	chr15	63121833	63142061	+	Human GRCh	OTTHUMG000001	LACTB	**lactamase beta**	Cytoplasm	peptidase		1.022663815	0	0.93826842	0	1	0	8.4910106	8.9493619	8.91974498	8.59214115
2818	**ENSG0000010364**	CORO2B	protein_coding	chr15	68578993	68727806	+	Human GRCh	OTTHUMG000001	CORO2B	**coronin 2B**	Plasma Mem	other		1.955088592	1	0.28573783	0	1	0	3.45912165	3.26387495	3.24263891	4.08947976
2819	**ENSG0000010365**	CSK	protein_coding	chr15	74782080	74803197	+	Human GRCh	OTTHUMG000001	CSK	**C-terminal Src kinase**	Cytoplasm	kinase	SC-204303	-1.049046787	0	0.79116027	0	1	0	10.8276077	11.0176823	10.9985905	10.731511
2820	**ENSG0000010365**	HERC1	protein_coding	chr15	63608618	63833948	-	Human GRCh	OTTHUMG000001	HERC1	**HECT and RLD domain containing E3 ubiquitin protein ligase fa**	Cytoplasm	other		-1.095355011	0	0.6436216	0	1	0	12.1092304	12.4502191	12.3276826	12.0345576
2821	**ENSG0000010367**	TRIP4	protein_coding	chr15	64387748	64455303	+	Human GRCh	OTTHUMG000001	TRIP4	**thyroid hormone receptor interactor 4**	Nucleus	transcription		-1.093846554	0	0.68401991	0	1	0	8.89962135	9.25291302	9.06083651	8.9139754
2822	**ENSG0000010370**	MTFMT	protein_coding	chr15	65001512	65029639	-	Human GRCh	OTTHUMG000001	MTFMT	**mitochondrial methionyl-tRNA formyltransferase**	Cytoplasm	enzyme		-1.206777133	0	0.41151339	0	1	0	8.30157781	8.57478182	8.30868175	8.16403333

2824	**ENSG0000010372**	AP3B2	protein_coding	chr15	82659281	82710112	-	Human GRCh	OTTHUMG000001	AP3B2	**adaptor related protein complex 3 subunit beta 2**	Cytoplasm	other		1.421638389	0	0.52206589	0	1	0	5.2857388	5.91035556	5.1420205	6.40248274
2825	**ENSG0000010374**	ACSBG1	protein_coding	chr15	78167468	78245688	-	Human GRCh	OTTHUMG000001	ACSBG1	**acyl-CoA synthetase bubblegum family member 1**	Cytoplasm	enzyme		-1.06081859	0	0.92166632	0	1	0	4.41012321	5.11345063	4.72974968	4.78712074
2826	**ENSG0000010374**	IGDCC4	protein_coding	chr15	65381484	65422947	-	Human GRCh	OTTHUMG000001	IGDCC4	**immunoglobulin superfamily DCC subclass member 4**	Plasma Mem	other		1.200771817	0	0.74920287	0	1	0	4.22827508	3.61057163	4.17144432	4.04485079
2827	**ENSG0000010376**	RAB11A	protein_coding	chr15	65726054	65891989	+	Human GRCh	OTTHUMG000001	RAB11A	**RAB11A, member RAS oncogene family**	Cytoplasm	enzyme		-1.037658832	0	0.87336338	0	1	0	11.2630432	11.6259598	11.6294983	11.1576592
2828	**ENSG0000010381**	CTSH	protein_coding	chr15	78921058	78949574	-	Human GRCh	OTTHUMG000001	CTSH	**cathepsin H**	Cytoplasm	peptidase		1.182360326	0	0.56965549	0	1	0	7.1661772	7.17826589	7.6418629	6.9579231
2829	**ENSG0000010385**	TTC23	protein_coding	chr15	99136323	99251223	-	Human GRCh	OTTHUMG000001	TTC23	**tetratricopeptide repeat domain 23**	Extracellular	other		-1.063962269	0	0.79565895	0	1	0	8.84168421	9.22750541	9.04860198	8.90825098
2830	**ENSG0000010385**	CD276	protein_coding	chr15	73683966	73714514	+	Human GRCh	OTTHUMG000001	CD276	**CD276 molecule**	Plasma Mem	other	DS-5573a,	-1.120292588	0	0.53220157	0	1	0	11.0788051	11.2498143	11.1998418	10.8576226
2831	**ENSG0000010387**	FAH	protein_coding	chr15	80152490	80186946	+	Human GRCh	OTTHUMG000001	FAH	**fumarylacetoacetate hydrolase**	Cytoplasm	enzyme		-1.128389222	0	0.6244335	0	1	0	8.57078878	8.7530227	8.62226769	8.42882793
2832	**ENSG0000010388**	CEMIP	protein_coding	chr15	80779343	80951776	+	Human GRCh	OTTHUMG000001	CEMIP	**cell migration inducing hyaluronidase 1**	Cytoplasm	enzyme		-1.092176401	0	0.90719358	0	1	0	2.75165066	3.88385009	2.81589669	3.73980963
2833	**ENSG0000010393**	RPAP1	protein_coding	chr15	41517176	41544269	-	Human GRCh	OTTHUMG000001	RPAP1	**RNA polymerase II associated protein 1**	Other	other		-1.047819863	0	0.82692294	0	1	0	10.1733973	10.5355914	10.3917714	10.2329339
2834	**ENSG0000010394**	HOMER2	protein_coding	chr15	82836946	82986153	-	Human GRCh	OTTHUMG000001	HOMER2	**homer scaffold protein 2**	Plasma Mem	other		-1.1301368	0	0.6586594	0	1	0	13.1332828	13.468408	13.376349	12.9216992
2835	**ENSG0000010396**	EHD4	protein_coding	chr15	41895933	41972557	-	Human GRCh	OTTHUMG000001	EHD4	**EH domain containing 4**	Plasma Mem	enzyme		-1.007566579	0	0.96906038	0	1	0	10.4883242	10.7229171	10.7055766	10.4901581
2836	**ENSG0000010397**	TMEM87A	protein_coding	chr15	42210447	42273534	-	Human GRCh	OTTHUMG000001	TMEM87A	**transmembrane protein 87A**	Cytoplasm	other		-1.104154561	0	0.62278058	0	1	0	10.2705698	10.5539925	10.5144909	10.0619301
2837	**ENSG0000010399**	ZNF106	protein_coding	chr15	42412823	42491141	-	Human GRCh	OTTHUMG000001	ZNF106	**zinc finger protein 106**	Cytoplasm	other		-1.113689903	0	0.60278944	0	1	0	11.292106	11.6072444	11.4907165	11.1654243
2838	**ENSG0000010399**	CEP152	protein_coding	chr15	48712928	48811146	-	Human GRCh	OTTHUMG000001	CEP152	**centrosomal protein 152**	Cytoplasm	other		-1.162813817	0	0.4860212	0	1	0	10.4475207	10.7366358	10.5583424	10.2951072
2839	**ENSG0000010404**	ATP8B4	protein_coding	chr15	49858238	50182817	-	Human GRCh	OTTHUMG000001	ATP8B4	**ATPase phospholipid transporting 8B4 (putative)**	Plasma Mem	transporter		-1.020034517	0	0.95816079	0	1	0	6.41330933	6.2799679	6.54137231	6.07076028
2840	**ENSG0000010404**	OCA2	protein_coding	chr15	27754875	28099315	-	Human GRCh	OTTHUMG000001	OCA2	**OCA2 melanosomal transmembrane protein**	Plasma Mem	transporter		-1.223588404	0	0.661513	0	1	0	6.34198285	7.08209447	6.77365359	6.30316435
2841	**ENSG0000010404**	DTWD1	protein_coding	chr15	49621037	49656232	+	Human GRCh	OTTHUMG000001	DTWD1	**DTW domain containing 1**	Nucleus	enzyme		-1.050110429	0	0.83252127	0	1	0	9.22023919	9.44695839	9.46462218	9.07430563
2843	**ENSG0000010405**	ENTREP2	protein_coding	chr15	29120252	29570979	-	Human GRCh	OTTHUMG000001	ENTREP2	**endosomal transmembrane epsin interactor 2**	Other	other		-3.119806696	-1	0.19706742	0	1	0	3.99143346	3.07325746	2.94053378	2.58069422
2844	**ENSG0000010406**	GABPB1	protein_coding	chr15	50275389	50355408	-	Human GRCh	OTTHUMG000001	GABPB1	**GA binding protein transcription factor subunit beta 1**	Nucleus	transcription		-1.131990837	0	0.55531708	0	1	0	9.22500957	9.56182512	9.39089755	9.13867637
2845	**ENSG0000010406**	TJP1	protein_coding	chr15	29699367	29968915	-	Human GRCh	OTTHUMG000001	TJP1	**tight junction protein 1**	Plasma Mem	other		-1.095800846	0	0.63265902	0	1	0	11.2841295	11.4942627	11.3517085	11.2267696
2846	**ENSG0000010408**	BMF	protein_coding	chr15	40087890	40108892	-	Human GRCh	OTTHUMG000001	BMF	**Bcl2 modifying factor**	Cytoplasm	other		-1.529205785	-1	0.23666497	0	1	0	9.25888971	9.38401322	8.76001236	8.98445351
2847	**ENSG0000010409**	DMXL2	protein_coding	chr15	51447711	51622833	-	Human GRCh	OTTHUMG000001	DMXL2	**Dmx like 2**	Cytoplasm	other		-1.054946916	0	0.82946891	0	1	0	10.7455311	11.1727788	11.0547658	10.7666126
2848	**ENSG0000010411**	SCG3	protein_coding	chr15	51681492	51721026	+	Human GRCh	OTTHUMG000001	SCG3	**secretogranin III**	Extracellular	other		1	0	1	0	1	0	1.36933441	1.50013141	1.47941518	1.37739678
2849	**ENSG0000010412**	DNAJC17	protein_coding	chr15	40765161	40807478	-	Human GRCh	OTTHUMG000001	DNAJC17	**DnaJ heat shock protein family (Hsp40) member C17**	Other	other		1.400081717	0	0.18287291	0	1	0	7.87592243	8.28537091	8.64341675	8.24535864
2850	**ENSG0000010413**	EIF3J	protein_coding	chr15	44537125	44563029	+	Human GRCh	OTTHUMG000001	EIF3J	**eukaryotic translation initiation factor 3 subunit J**	Cytoplasm	translation re		-1.025045812	0	0.89410725	0	1	0	11.1454343	11.4426173	11.4318739	11.0941148
2851	**ENSG0000010413**	SPG11	protein_coding	chr15	44554818	44663688	-	Human GRCh	OTTHUMG000001	SPG11	**SPG11 vesicle trafficking associated, spatacsin**	Cytoplasm	other		-1.163652463	0	0.43931421	0	1	0	12.3248573	12.5654384	12.4439849	12.0866752
2852	**ENSG0000010414**	RHOV	protein_coding	chr15	40872214	40874234	-	Human GRCh	OTTHUMG000001	RHOV	**ras homolog family member V**	Plasma Mem	enzyme		1.564526765	1	0.23368193	0	1	0	5.7198393	5.65513661	5.77737121	6.42468154
2853	**ENSG0000010414**	VPS18	protein_coding	chr15	40894450	40903975	+	Human GRCh	OTTHUMG000001	VPS18	**VPS18 core subunit of CORVET and HOPS complexes**	Cytoplasm	transporter		-1.000376956	0	0.99872056	0	1	0	9.88262933	10.1521207	10.1115704	9.93046075
2854	**ENSG0000010414**	OIP5	protein_coding	chr15	41309273	41332591	-	Human GRCh	OTTHUMG000001	OIP5	**Opa interacting protein 5**	Nucleus	other		-1.21396014	0	0.48535789	0	1	0	8.41241173	8.88863275	8.59146524	8.30214126
2855	**ENSG0000010415**	SLC30A4	protein_coding	chr15	45479606	45522755	-	Human GRCh	OTTHUMG000001	SLC30A4	**solute carrier family 30 member 4**	Cytoplasm	transporter		1.000439125	0	0.99920378	0	1	0	6.19111555	6.24049347	6.64502792	5.5191692
2856	**ENSG0000010416**	BLOC1S6	protein_coding	chr15	45587214	45615945	+	Human GRCh	OTTHUMG000001	BLOC1S6	**biogenesis of lysosomal organelles complex 1 subunit 6**	Cytoplasm	other		1.019748201	0	0.92748869	0	1	0	10.3198931	10.4670888	10.5939117	10.2102444
2857	**ENSG0000010417**	MYEF2	protein_coding	chr15	48134632	48178353	-	Human GRCh	OTTHUMG000001	MYEF2	**myelin expression factor 2**	Nucleus	transcription		1.026542879	0	0.91384022	0	1	0	10.6793532	10.8908661	11.0700898	10.4955503
2858	**ENSG0000010420**	SGK3	protein_coding	chr8	66712734	66862022	+	Human GRCh	OTTHUMG000001	C8orf44-SGK	**serum/glucocorticoid regulated kinase family member 3**	Cytoplasm	kinase		-1.084854508	0	0.74747375	0	1	0	9.97114504	10.2110645	10.1359674	9.86272617
2859	**ENSG0000010421**	PDGFRL	protein_coding	chr8	17576433	17644071	+	Human GRCh	OTTHUMG000001	PDGFRL	**platelet derived growth factor receptor like**	Plasma Mem	kinase		1.278475859	0	0.65428397	0	1	0	4.17353551	4.58275341	4.71627888	4.4613995
2860	**ENSG0000010421**	CSPP1	protein_coding	chr8	67062417	67196778	+	Human GRCh	OTTHUMG000001	CSPP1	**centrosome and spindle pole associated protein 1**	Cytoplasm	other		-1.024412037	0	0.91000957	0	1	0	11.6816254	11.902561	11.9752854	11.5235747
2861	**ENSG0000010421**	ZDHHC2	protein_coding	chr8	17156482	17224799	+	Human GRCh	OTTHUMG000001	ZDHHC2	**zinc finger DHHC-type palmitoyltransferase 2**	Nucleus	enzyme		-1.035557986	0	0.89152279	0	1	0	9.10154969	9.21894244	9.31756204	8.89286418
2862	**ENSG0000010422**	BRF2	protein_coding	chr8	37843268	37849861	-	Human GRCh	OTTHUMG000001	BRF2	**BRF2 RNA polymerase III transcription initiation factor subunit**	Nucleus	transcription		-1.075858569	0	0.75619397	0	1	0	8.26458936	8.6480668	8.56216762	8.19330995
2863	**ENSG0000010422**	TRIM35	protein_coding	chr8	27284886	27311272	-	Human GRCh	OTTHUMG000001	TRIM35	**tripartite motif containing 35**	Cytoplasm	other		1.058683382	0	0.78617831	0	1	0	10.548871	10.6981577	10.891787	10.4479078
2864	**ENSG0000010423**	ZFAND1	protein_coding	chr8	81701334	81732903	-	Human GRCh	OTTHUMG000001	ZFAND1	**zinc finger AN1-type containing 1**	Cytoplasm	other		1.008172034	0	0.97432096	0	1	0	10.2813767	10.3902399	10.5174879	10.1441664
2865	**ENSG0000010423**	RP1	protein_coding	chr8	54509422	54871720	+	Human GRCh	OTTHUMG000001	RP1	**RP1 axonemal microtubule associated**	Cytoplasm	other		1	0	1	0	1	0	0.95953862	1.08383379	1.06419048	0.96721862
2866	**ENSG0000010426**	CA2	protein_coding	chr8	85463968	85481493	+	Human GRCh	OTTHUMG000001	CA2	**carbonic anhydrase 2**	Cytoplasm	enzyme	benzthiazi	-2.179286583	-1	0.34148114	0	1	0	3.69988659	3.24029482	3.46949335	2.33137294
2867	**ENSG0000010429**	FZD3	protein_coding	chr8	28494205	28574267	+	Human GRCh	OTTHUMG000001	FZD3	**frizzled class receptor 3**	Plasma Mem	G-protein cou		1.067481711	0	0.77949928	0	1	0	10.499521	10.5822973	10.8541138	10.3127076
2868	**ENSG0000010429**	INTS9	protein_coding	chr8	28767661	28890242	-	Human GRCh	OTTHUMG000001	INTS9	**integrator complex subunit 9**	Nucleus	other		-1.169257969	0	0.47601381	0	1	0	9.15068116	9.61102618	9.39086826	9.04468594
2869	**ENSG0000010431**	RIPK2	protein_coding	chr8	89757806	89791064	+	Human GRCh	OTTHUMG000001	RIPK2	**receptor interacting serine/threonine kinase 2**	Plasma Mem	kinase	GSK583, S(	-1.058363803	0	0.8235857	0	1	0	8.07199494	8.55277665	8.4371914	8.08946786
2870	**ENSG0000010431**	EYA1	protein_coding	chr8	71197433	71592025	-	Human GRCh	OTTHUMG000001	EYA1	**EYA transcriptional coactivator and phosphatase 1**	Nucleus	phosphatase		2.373268131	1	0.09046365	0	1	0	5.37607849	6.12977572	5.67326024	7.11516831
2871	**ENSG0000010432**	NBN	protein_coding	chr8	89924515	90003228	-	Human GRCh	OTTHUMG000001	NBN	**nibrin**	Nucleus	other		1.001723495	0	0.99285427	0	1	0	10.5208226	10.6953683	10.7506761	10.4573262
2872	**ENSG0000010432**	TRPA1	protein_coding	chr8	72019917	72075584	-	Human GRCh	OTTHUMG000001	TRPA1	**transient receptor potential cation channel subfamily A memb**	Plasma Mem	transporter	TRPA1-inh	1.455806916	0	0.55409188	0	1	0	4.34473818	4.08130149	4.35056399	4.68864957
2873	**ENSG0000010432**	CPQ	protein_coding	chr8	96645242	97149654	+	Human GRCh	OTTHUMG000001	CPQ	**carboxypeptidase Q**	Extracellular	peptidase		-1.205252414	0	0.78109609	0	1	0	4.42779314	4.0195136	4.1488726	4.00865889
2874	**ENSG0000010432**	DECR1	protein_coding	chr8	90001405	90053633	+	Human GRCh	OTTHUMG000001	DECR1	**2,4-dienoyl-CoA reductase 1**	Cytoplasm	enzyme		1.129922585	0	0.90889604	0	1	0	8.46227748	8.87263793	8.93055633	8.70234232
2875	**ENSG0000010432**	CALB1	protein_coding	chr8	90058608	90095475	-	Human GRCh	OTTHUMG000001	CALB1	**calbindin 1**	Cytoplasm	other		1.097790533	0	0.90606141	0	1	0	2.33892943	3.70813532	2.39140323	3.72172392
2876	**ENSG0000010433**	BPNT2	protein_coding	chr8	56957931	56993867	-	Human GRCh	OTTHUMG000001	BPNT2	**3'(2'), 5'-bisphosphate nucleotidase 2**	Cytoplasm	enzyme		1.065936324	0	0.76582484	0	1	0	12.2029411	12.476718	12.540262	12.2828762
2877	**ENSG0000010433**	SFRP1	protein_coding	chr8	41261962	41309473	-	Human GRCh	OTTHUMG000001	SFRP1	**secreted frizzled related protein 1**	Plasma Mem	transmembra		-1.185742844	0	0.75242149	0	1	0	8.56145354	8.49013777	8.53779678	8.10873856
2878	**ENSG0000010434**	LAPTM4B	protein_coding	chr8	97775057	97853013	+	Human GRCh	OTTHUMG000001	LAPTM4B	**lysosomal protein transmembrane 4 beta**	Cytoplasm	other		1.083609964	0	0.70350044	0	1	0	11.766247	11.9953307	11.9814347	11.9690634
2879	**ENSG0000010434**	UBE2W	protein_coding	chr8	73780097	73878910	-	Human GRCh	OTTHUMG000001	UBE2W	**ubiquitin conjugating enzyme E2 W**	Nucleus	enzyme		1.019956829	0	0.93233374	0	1	0	10.267101	10.4636479	10.6009359	10.133899
2880	**ENSG0000010435**	POP1	protein_coding	chr8	98117293	98159835	+	Human GRCh	OTTHUMG000001	POP1	**POP1 homolog, ribonuclease P/MRP subunit**	Nucleus	enzyme		-1.198217452	0	0.43218257	0	1	0	9.75024897	10.1885051	9.90233088	9.66241822
2881	**ENSG0000010436**	NIPAL2	protein_coding	chr8	98189826	98294393	-	Human GRCh	OTTHUMG000001	NIPAL2	**NIPA like domain containing 2**	Other	other		-1.052992575	0	0.90870924	0	1	0	6.11438849	6.20271558	6.36345189	5.78439657
2882	**ENSG0000010436**	IKBKB	protein_coding	chr8	42271302	42332460	+	Human GRCh	OTTHUMG000001	IKBKB	**inhibitor of nuclear factor kappa B kinase subunit beta**	Cytoplasm	kinase	auranofin,	-1.078523187	0	0.72921974	0	1	0	10.6618872	10.7796178	10.8522052	10.3795255
2883	**ENSG0000010436**	PLAT	protein_coding	chr8	42174718	42207709	-	Human GRCh	OTTHUMG000001	PLAT	**plasminogen activator, tissue type**	Extracellular	peptidase	6-aminoca	2.971942019	1	0.17865809	0	1	0	2.33896935	3.17806285	2.8427252	3.69884603
2884	**ENSG0000010436**	JPH1	protein_coding	chr8	74234700	74321540	-	Human GRCh	OTTHUMG000001	JPH1	**junctophilin 1**	Plasma Mem	other		1.209999325	0	0.40881069	0	1	0	8.87546221	9.21940533	9.45479612	9.03663871
2886	**ENSG0000010437**	STK3	protein_coding	chr8	98371228	98942827	-	Human GRCh	OTTHUMG000001	STK3	**serine/threonine kinase 3**	Cytoplasm	kinase	XMU-MP-1	-1.04736765	0	0.82666832	0	1	0	9.76262051	9.9795718	9.94506556	9.6945218
2887	**ENSG0000010438**	GDAP1	protein_coding	chr8	74320613	74518007	+	Human GRCh	OTTHUMG000001	GDAP1	**ganglioside induced differentiation associated protein 1**	Cytoplasm	other		1.255343572	0	0.3626114	0	1	0	7.93949542	8.23761418	8.25817112	8.36709602
2888	**ENSG0000010438**	RAB2A	protein_coding	chr8	60516936	60623644	+	Human GRCh	OTTHUMG000001	RAB2A	**RAB2A, member RAS oncogene family**	Cytoplasm	enzyme		1.016465309	0	0.93805445	0	1	0	10.8417814	11.0641479	11.1418887	10.7819641
2889	**ENSG0000010440**	EIF3E	protein_coding	chr8	108162787	108443496	-	Human GRCh	OTTHUMG000001	EIF3E	**eukaryotic translation initiation factor 3 subunit E**	Cytoplasm	translation re		-1.04510402	0	0.84143262	0	1	0	13.1490253	13.3675412	13.3870766	13.0116542
2890	**ENSG0000010441**	EMC2	protein_coding	chr8	108443601	108489196	+	Human GRCh	OTTHUMG000001	EMC2	**ER membrane protein complex subunit 2**	Nucleus	other		1.032305119	0	0.87415331	0	1	0	9.633582	9.90659266	9.98302838	9.61288088
2891	**ENSG0000010441**	ESRP1	protein_coding	chr8	94641074	94707466	+	Human GRCh	OTTHUMG000001	ESRP1	**epithelial splicing regulatory protein 1**	Nucleus	other		-1.187017509	0	0.41373868	0	1	0	12.2808897	12.6470019	12.3643069	12.2092671
2892	**ENSG0000010441**	CCN4	protein_coding	chr8	133191039	133231690	+	Human GRCh	OTTHUMG000001	CCN4	**cellular communication network factor 4**	Extracellular	other		-11.73437126	-1	0.08589172	0	1	0	1.6691031	2.64470585	1.70933959	1.67195539
2893	**ENSG0000010441**	NDRG1	protein_coding	chr8	133237175	133302022	-	Human GRCh	OTTHUMG000001	NDRG1	**N-myc downstream regulated 1**	Nucleus	kinase		1.405386164	0	0.45631829	0	1	0	11.5756005	11.7618547	11.8675077	12.1942734
2894	**ENSG0000010442**	ZC2HC1A	protein_coding	chr8	78666050	78719765	+	Human GRCh	OTTHUMG000001	ZC2HC1A	**zinc finger C2HC-type containing 1A**	Other	other		-1.190889645	0	0.57771085	0	1	0	8.46827246	8.56900709	8.58642482	8.02858791
2895	**ENSG0000010443**	IL7	protein_coding	chr8	78675743	78805523	-	Human GRCh	OTTHUMG000001	IL7	**interleukin 7**	Extracellular	cytokine	ritlecitinib,	-1.000877521	0	0.99945324	0	1	0	1.95953658	1.45052027	1.44241454	1.96606469
2896	**ENSG0000010443**	STMN2	protein_coding	chr8	79611117	79666158	+	Human GRCh	OTTHUMG000001	STMN2	**stathmin 2**	Plasma Mem	other		1	0	1	0	1	0	1.36210755	1.49176115	1.47122728	1.37009991
2897	**ENSG0000010444**	ARMC1	protein_coding	chr8	65602458	65634217	-	Human GRCh	OTTHUMG000001	ARMC1	**armadillo repeat containing 1**	Cytoplasm	other		-1.022399441	0	0.91526871	0	1	0	10.2212858	10.516753	10.5005148	10.1869354
2898	**ENSG0000010444**	TRPS1	protein_coding	chr8	115408496	115809673	-	Human GRCh	OTTHUMG000001	TRPS1	**transcriptional repressor GATA binding 1**	Nucleus	transcription		-1.007001529	0	0.96915124	0	1	0	11.7793934	12.1980744	12.1200577	11.863994
2899	**ENSG0000010445**	SPAG1	protein_coding	chr8	100157906	100259278	+	Human GRCh	OTTHUMG000001	SPAG1	**sperm associated antigen 1**	Cytoplasm	other		2.025985199	1	0.00477448	1	1	0	7.00476291	7.10552973	8.09486196	7.29756238
2900	**ENSG0000010447**	CHRAC1	protein_coding	chr8	140511311	140517154	+	Human GRCh	OTTHUMG000001	CHRAC1	**chromatin accessibility complex subunit 1**	Nucleus	enzyme		-1.040624973	0	0.85842246	0	1	0	9.63476132	9.92437428	9.82559771	9.66050717
2901	**ENSG0000010449**	NCALD	protein_coding	chr8	101686542	102124907	-	Human GRCh	OTTHUMG000001	NCALD	**neurocalcin delta**	Cytoplasm	other		-1.161411335	0	0.71487358	0	1	0	5.22843918	4.85218865	4.87207682	4.97737092
2902	**ENSG0000010449**	SNX16	protein_coding	chr8	81799581	81842866	-	Human GRCh	OTTHUMG000001	SNX16	**sorting nexin 16**	Cytoplasm	transporter		1.050123226	0	0.85110612	0	1	0	7.8770568	7.96741719	8.06622728	7.87097528
2904	**ENSG0000010451**	UBR5	protein_coding	chr8	102252273	102412759	-	Human GRCh	OTTHUMG000001	UBR5	**ubiquitin protein ligase E3 component n-recognin 5**	Nucleus	enzyme		-1.163333588	0	0.49436835	0	1	0	13.0538561	13.3929526	13.1653445	12.9678816
2905	**ENSG0000010451**	GSDMD	protein_coding	chr8	143553207	143563062	+	Human GRCh	OTTHUMG000001	GSDMD	**gasdermin D**	Extracellular	transporter		4.777085886	1	3.09E-05	1	0.07	1	5.82098566	6.03648844	6.78845079	7.48856443
2906	**ENSG0000010452**	GFUS	protein_coding	chr8	143612618	143618048	-	Human GRCh	OTTHUMG000001	GFUS	**GDP-L-fucose synthase**	Plasma Mem	enzyme		-1.027681892	0	0.9006639	0	1	0	10.810129	11.1742531	11.0578917	10.886379
2907	**ENSG0000010452**	PYCR3	protein_coding	chr8	143603210	143609773	-	Human GRCh	OTTHUMG000001	PYCR3	**pyrroline-5-carboxylate reductase 3**	Cytoplasm	enzyme		1.110159901	0	0.60949279	0	1	0	10.205399	10.5206812	10.6175759	10.3466362
2908	**ENSG0000010452**	EEF1D	protein_coding	chr8	143579697	143599541	-	Human GRCh	OTTHUMG000001	EEF1D	**eukaryotic translation elongation factor 1 delta**	Cytoplasm	translation re		1.103386879	0	0.63216861	0	1	0	12.697651	12.8861532	13.0214001	12.7709398
2910	**ENSG0000010454**	SQLE	protein_coding	chr8	124998497	125022283	+	Human GRCh	OTTHUMG000001	SQLE	**squalene epoxidase**	Cytoplasm	enzyme	tolnaftate,	-1.115926529	0	0.71286815	0	1	0	11.1740006	11.7831753	11.5786889	11.1714903

2911	**ENSG0000010461**	SH2D4A	protein_coding	chr8	19313693	19396218	+	Human GRCh	OTTHUMG000000	SH2D4A	**SH2 domain containing 4A**	Cytoplasm	other		-1.137841243	0	0.62997127	0	1	0	8.62275538	8.87789485	8.72545658	8.49227289
2912	**ENSG0000010461**	INTS10	protein_coding	chr8	19817391	19852083	+	Human GRCh	OTTHUMG000001	INTS10	**integrator complex subunit 10**	Nucleus	other		-1.143857362	0	0.51667453	0	1	0	10.5539367	10.8267639	10.6831925	10.3948398
2913	**ENSG0000010462**	ERI1	protein_coding	chr8	9002147	9116746	+	Human GRCh	OTTHUMG000001	ERI1	**exoribonuclease 1**	Nucleus	enzyme		1.284736382	0	0.26732864	0	1	0	8.71364392	8.92796742	9.19926631	8.99127864
2914	**ENSG0000010463**	SLC39A14	protein_coding	chr8	22367278	22434129	+	Human GRCh	OTTHUMG000000	SLC39A14	**solute carrier family 39 member 14**	Plasma Mem	transporter		-1.194182389	0	0.3918683	0	1	0	11.3614961	11.4715307	11.3422124	11.0693404
2915	**ENSG0000010464**	MTMR9	protein_coding	chr8	11284816	11328146	+	Human GRCh	OTTHUMG000000	MTMR9	**myotubularin related protein 9**	Cytoplasm	phosphatase		-1.112971817	0	0.66936868	0	1	0	10.5459933	10.8037226	10.7472337	10.3407667
2916	**ENSG0000010466**	LEPROTL1	protein_coding	chr8	30095408	30177208	+	Human GRCh	OTTHUMG000001	LEPROTL1	**leptin receptor overlapping transcript like 1**	Plasma Mem	other		-1.093197233	0	0.6538008	0	1	0	9.62824094	9.87682193	9.81422051	9.48297332
2917	**ENSG0000010467**	DCTN6	protein_coding	chr8	30156319	30183639	+	Human GRCh	OTTHUMG000001	DCTN6	**dynactin subunit 6**	Cytoplasm	enzyme		-1.140337854	0	0.59158801	0	1	0	8.62692513	8.87432872	8.7453249	8.46360336
2918	**ENSG0000010467**	R3HCC1	protein_coding	chr8	23270120	23296279	+	Human GRCh	OTTHUMG000001	R3HCC1	**R3H domain and coiled-coil containing 1**	Other	other		1.026129385	0	0.90599263	0	1	0	9.34953721	9.66564843	9.6284289	9.45905012
2919	**ENSG0000010468**	GSR	protein_coding	chr8	30678066	30727846	-	Human GRCh	OTTHUMG000001	GSR	**glutathione-disulfide reductase**	Cytoplasm	enzyme	carmustine	-1.088347183	0	0.69556885	0	1	0	11.5174376	11.9523106	11.7321096	11.5824153
2920	**ENSG0000010468**	TNFRSF10/	protein_coding	chr8	23190452	23225102	-	Human GRCh	OTTHUMG000000	TNFRSF10A	**TNF receptor superfamily member 10a**	Plasma Mem	transmembra	trm-1, map	-1.014492295	0	0.95868496	0	1	0	9.08478607	9.3446957	9.40513637	8.96540594
2921	**ENSG0000010469**	UBXN8	protein_coding	chr8	30729131	30767006	+	Human GRCh	OTTHUMG000001	UBXN8	**UBX domain protein 8**	Cytoplasm	other		-1.004517882	0	0.98609909	0	1	0	7.29866178	7.59949826	7.37888248	7.5216327
2922	**ENSG0000010469**	PPP2CB	protein_coding	chr8	30774457	30814314	-	Human GRCh	OTTHUMG000001	PPP2CB	**protein phosphatase 2 catalytic subunit beta**	Cytoplasm	phosphatase		-1.19080255	0	0.4603966	0	1	0	10.8188121	11.2078276	11.0389114	10.5773076
2923	**ENSG0000010471**	ERICH1	protein_coding	chr8	614746	738106	-	Human GRCh	OTTHUMG000001	ERICH1	**glutamate rich 1**	Other	other		1.019366388	0	0.94858269	0	1	0	9.23138878	9.35884945	9.47383168	9.13768478
2925	**ENSG0000010472**	TUSC3	protein_coding	chr8	15417215	15766649	+	Human GRCh	OTTHUMG000000	TUSC3	**tumor suppressor candidate 3**	Plasma Mem	transporter		1.074106151	0	0.7909501	0	1	0	9.49443531	9.5449415	9.70390305	9.47798943
2926	**ENSG0000010472**	ARHGEF10	protein_coding	chr8	1823926	1958641	+	Human GRCh	OTTHUMG000001	ARHGEF10	**Rho guanine nucleotide exchange factor 10**	Cytoplasm	enzyme		1.005505932	0	0.98575494	0	1	0	9.3572628	9.47623051	9.62698179	9.17836982
2927	**ENSG0000010473**	KLHDC4	protein_coding	chr16	87696485	87765992	-	Human GRCh	OTTHUMG000001	KLHDC4	**kelch domain containing 4**	Other	other		-1.031182212	0	0.87243411	0	1	0	11.1144607	11.3656245	11.4122305	10.9724188
2928	**ENSG0000010473**	MCM4	protein_coding	chr8	47960185	47978160	+	Human GRCh	OTTHUMG000001	MCM4	**minichromosome maintenance complex component 4**	Nucleus	enzyme		-1.117518841	0	0.61210379	0	1	0	12.8143651	13.2620942	13.0253292	12.8439118
2930	**ENSG0000010475**	KCTD9	protein_coding	chr8	25427847	25458476	-	Human GRCh	OTTHUMG000000	KCTD9	**potassium channel tetramerization domain containing 9**	Other	other		-1.114215162	0	0.60906682	0	1	0	10.791603	11.1102995	11.0097926	10.6418293
2931	**ENSG0000010476**	FGL1	protein_coding	chr8	17864380	17910365	-	Human GRCh	OTTHUMG000000	FGL1	**fibrinogen like 1**	Extracellular	other		11.66674929	1	0.63778314	0	NA	0	0.09299048	0.09993018	0.75165207	0.0933922
2932	**ENSG0000010476**	ASAH1	protein_coding	chr8	18055992	18084998	-	Human GRCh	OTTHUMG000000	ASAH1	**N-acylsphingosine amidohydrolase 1**	Cytoplasm	enzyme		-1.007710702	0	0.97466588	0	1	0	11.6532611	11.9019999	11.9957501	11.5048325
2933	**ENSG0000010476**	BNIP3L	protein_coding	chr8	26383054	26505636	+	Human GRCh	OTTHUMG000000	BNIP3L	**BCL2 interacting protein 3 like**	Cytoplasm	other		-1.167935089	0	0.53397442	0	1	0	12.2053874	12.6669121	12.3712204	12.185139
2934	**ENSG0000010477**	MAN2B1	protein_coding	chr19	12646511	12666742	-	Human GRCh	OTTHUMG000001	MAN2B1	**mannosidase alpha class 2B member 1**	Cytoplasm	enzyme		1.019009961	0	0.93435981	0	1	0	10.6071812	10.9245382	10.87036	10.7196819
2935	**ENSG0000010478**	KCNN4	protein_coding	chr19	43766533	43780976	-	Human GRCh	OTTHUMG000001	KCNN4	**potassium calcium-activated channel subfamily N member 4**	Plasma Mem	ion channel	betametha	-5.632364452	-1	0.20320012	0	1	0	1.65432427	2.33658314	1.70690268	1.65808002
2936	**ENSG0000010480**	TULP2	protein_coding	chr19	48880965	48898744	-	Human GRCh	OTTHUMG000001	TULP2	**TUB like protein 2**	Other	enzyme		-1.011191181	0	0.9916112	0	1	0	1.63094731	2.76278529	1.92805138	2.62102883
2937	**ENSG0000010480**	NUCB1	protein_coding	chr19	48900050	48923473	+	Human GRCh	OTTHUMG000001	NUCB1	**nucleobindin 1**	Cytoplasm	other		-1.05050044	0	0.80199968	0	1	0	11.9889204	12.1976939	12.1488467	11.9241971
2938	**ENSG0000010480**	DHDH	protein_coding	chr19	48933699	48944969	+	Human GRCh	OTTHUMG000001	DHDH	**dihydrodiol dehydrogenase**	Other	enzyme		-1.819564163	-1	0.44021574	0	1	0	3.95724029	4.24494551	3.93953883	3.0587484
2939	**ENSG0000010481**	GYS1	protein_coding	chr19	48968130	48993310	-	Human GRCh	OTTHUMG000001	GYS1	**glycogen synthase 1**	Cytoplasm	enzyme		-1.092077747	0	0.70519679	0	1	0	11.4923962	11.9114082	11.5985919	11.6371075
2940	**ENSG0000010481**	MAP4K1	protein_coding	chr19	38587641	38618882	-	Human GRCh	OTTHUMG000001	MAP4K1	**mitogen-activated protein kinase kinase kinase kinase 1**	Cytoplasm	kinase	CFI-40241:	1.525371492	1	0.32190786	0	1	0	5.25279332	5.53678003	5.81597219	5.80214729
2942	**ENSG0000010482**	ECH1	protein_coding	chr19	38815422	38831841	-	Human GRCh	OTTHUMG000001	ECH1	**enoyl-CoA hydratase 1**	Cytoplasm	enzyme		1.004837472	0	0.98299072	0	1	0	11.9302912	12.3710213	12.1901051	12.1622869
2943	**ENSG0000010482**	HNRNPL	protein_coding	chr19	38836370	38852347	-	Human GRCh	OTTHUMG000001	HNRNPL	**heterogeneous nuclear ribonucleoprotein L**	Nucleus	other		-1.064772054	0	0.75196089	0	1	0	13.4759444	13.7474337	13.682167	13.3989357
2944	**ENSG0000010482**	NFKBIB	protein_coding	chr19	38899700	38908893	+	Human GRCh	OTTHUMG000001	NFKBIB	**NFKB inhibitor beta**	Nucleus	transcription		1.129075975	0	0.57921796	0	1	0	9.43695122	9.85353374	9.97508202	9.59526528
2945	**ENSG0000010482**	LHB	protein_coding	chr19	49015980	49017091	-	Human GRCh	OTTHUMG000001	LHB	**luteinizing hormone subunit beta**	Extracellular	other		-1.522322936	-1	0.57289434	0	1	0	3.93761259	4.43250696	4.03119197	3.5792361
2947	**ENSG0000010483**	TUBB4A	protein_coding	chr19	6494319	6502848	-	Human GRCh	OTTHUMG000001	TUBB4A	**tubulin beta 4A class IVa**	Cytoplasm	other	docetaxel/	2.399194891	1	0.25925176	0	1	0	7.74711363	7.99010078	8.58289533	9.19796141
2948	**ENSG0000010483**	SARS2	protein_coding	chr19	38915266	38930763	-	Human GRCh	OTTHUMG000001	SARS2	**seryl-tRNA synthetase 2, mitochondrial**	Cytoplasm	enzyme		-1.108777496	0	0.6158632	0	1	0	10.0463759	10.3528282	10.2127512	9.96335409
2950	**ENSG0000010485**	SNRNP70	protein_coding	chr19	49085419	49108605	+	Human GRCh	OTTHUMG000001	SNRNP70	**small nuclear ribonucleoprotein U1 subunit 70**	Nucleus	other		1.04050349	0	0.82104104	0	1	0	12.2940971	12.4795658	12.5410809	12.3186494
2951	**ENSG0000010485**	CLPTM1	protein_coding	chr19	44954585	44993341	+	Human GRCh	OTTHUMG000001	CLPTM1	**CLPTM1 regulator of GABA type A receptor forward trafficking**	Plasma Mem	other		-1.070329881	0	0.74606125	0	1	0	11.5548484	11.8423391	11.6959276	11.5605051
2952	**ENSG0000010485**	RELB	protein_coding	chr19	45001461	45038192	+	Human GRCh	OTTHUMG000001	RELB	**RELB proto-oncogene, NF-kB subunit**	Nucleus	transcription		1.049779692	0	0.90855476	0	1	0	6.33802736	7.31327259	7.1681647	6.74788645
2953	**ENSG0000010485**	CLASRP	protein_coding	chr19	45039045	45070956	+	Human GRCh	OTTHUMG000001	CLASRP	**CLK4 associating serine/arginine rich protein**	Nucleus	other		-1.048415628	0	0.82518661	0	1	0	11.1403315	11.4400856	11.3356086	11.1506456
2954	**ENSG0000010486**	LIN7B	protein_coding	chr19	49114324	49118460	+	Human GRCh	OTTHUMG000001	LIN7B	**lin-7 homolog B, crumbs cell polarity complex component**	Cytoplasm	other		-1.034905163	0	0.91406375	0	1	0	6.7463332	7.03882425	6.60239145	7.07608114
2955	**ENSG0000010486**	PPP1R37	protein_coding	chr19	45091396	45148077	+	Human GRCh	OTTHUMG000001	PPP1R37	**protein phosphatase 1 regulatory subunit 37**	Other	other		1.072818117	0	0.73593233	0	1	0	10.5021114	10.5753803	10.7315072	10.4895629
2956	**ENSG0000010487**	FCGRT	protein_coding	chr19	49506816	49526428	+	Human GRCh	OTTHUMG000001	FCGRT	**Fc gamma receptor and transporter**	Plasma Mem	transmembra	efgartigim	-1.30715539	0	0.35917092	0	1	0	6.94011726	7.38862659	7.08784716	6.71827823
2957	**ENSG0000010487**	PIH1D1	protein_coding	chr19	49446298	49453497	-	Human GRCh	OTTHUMG000001	PIH1D1	**PIH1 domain containing 1**	Nucleus	other		-1.110152604	0	0.61571272	0	1	0	11.1311945	11.4034144	11.2730353	11.0256254
2958	**ENSG0000010487**	CKM	protein_coding	chr19	45306413	45322875	-	Human GRCh	OTTHUMG000001	CKM	**creatine kinase, M-type**	Cytoplasm	kinase		15.91487262	1	0.02654748	1	1	0	1.17683316	1.25103634	1.97158044	2.28501916
2959	**ENSG0000010488**	ARHGEF18	protein_coding	chr19	7348937	7472485	+	Human GRCh	OTTHUMG000001	ARHGEF18	**Rho/Rac guanine nucleotide exchange factor 18**	Cytoplasm	other		1.013707266	0	0.95385964	0	1	0	10.1960023	10.6563673	10.6152705	10.289231
2960	**ENSG0000010488**	PPP1R13L	protein_coding	chr19	45379638	45406349	-	Human GRCh	OTTHUMG000001	PPP1R13L	**protein phosphatase 1 regulatory subunit 13 like**	Nucleus	transcription		-1.074402435	0	0.74987681	0	1	0	10.6276291	11.1182483	10.7175022	10.9074934
2961	**ENSG0000010488**	PEX11G	protein_coding	chr19	7476875	7497449	-	Human GRCh	OTTHUMG000001	PEX11G	**peroxisomal biogenesis factor 11 gamma**	Cytoplasm	other		1.268161083	0	0.49704265	0	1	0	6.04721869	5.70166533	6.37711522	5.80459955
2962	**ENSG0000010488**	ERCC2	protein_coding	chr19	45349837	45370918	-	Human GRCh	OTTHUMG000000	ERCC2	**ERCC excision repair 2, TFIIH core complex helicase subunit**	Nucleus	enzyme		1.079916407	0	0.71323	0	1	0	10.8017076	11.1836792	11.2477247	10.9209525
2963	**ENSG0000010488**	DOT1L	protein_coding	chr19	2163933	2232578	+	Human GRCh	OTTHUMG000001	DOT1L	**DOT1 like histone lysine methyltransferase**	Nucleus	phosphatase	pinometos	-1.020012217	0	0.9194784	0	1	0	12.9103701	13.0632637	13.0467369	12.8824058
2964	**ENSG0000010488**	PLEKHJ1	protein_coding	chr19	2230084	2237704	-	Human GRCh	OTTHUMG000001	PLEKHJ1	**pleckstrin homology domain containing J1**	Other	other		1.107678303	0	0.62372881	0	1	0	10.5971589	10.928142	10.9736452	10.7927921
2965	**ENSG0000010488**	SLC17A7	protein_coding	chr19	49429401	49442360	-	Human GRCh	OTTHUMG000001	SLC17A7	**solute carrier family 17 member 7**	Plasma Mem	transporter		1.463423759	0	0.52828036	0	1	0	6.26775696	6.52214155	6.94147154	6.70260947
2966	**ENSG0000010488**	RNASEH2A	protein_coding	chr19	12806584	12813640	+	Human GRCh	OTTHUMG000001	RNASEH2A	**ribonuclease H2 subunit A**	Nucleus	enzyme		-1.189762594	0	0.62644956	0	1	0	6.00277875	5.87537629	5.92546843	5.59712201
2967	**ENSG0000010489**	KLC3	protein_coding	chr19	45333434	45351520	+	Human GRCh	OTTHUMG000001	KLC3	**kinesin light chain 3**	Cytoplasm	other		-1.096366657	0	0.81829126	0	1	0	8.02315154	8.56447992	8.15520371	8.28429373
2969	**ENSG0000010489**	SF3A2	protein_coding	chr19	2236824	2248655	+	Human GRCh	OTTHUMG000001	SF3A2	**splicing factor 3a subunit 2**	Nucleus	other		1.121572227	0	0.54126338	0	1	0	12.0469233	12.3397628	12.4094691	12.2430888
2970	**ENSG0000010489**	AMH	protein_coding	chr19	2249309	2252073	+	Human GRCh	OTTHUMG000001	AMH	**anti-Mullerian hormone**	Extracellular	growth facto		-1.121085633	0	0.66985491	0	1	0	10.0806551	10.3570993	10.3143108	9.84113408
2971	**ENSG0000010490**	DKKL1	protein_coding	chr19	49361783	49375116	+	Human GRCh	OTTHUMG000001	DKKL1	**dickkopf like acrosomal protein 1**	Extracellular	other		1.478957686	0	0.72960395	0	NA	0	2.46000505	2.26116191	3.12422986	1.64999913
2972	**ENSG0000010490**	LYL1	protein_coding	chr19	13099033	13103161	-	Human GRCh	OTTHUMG000001	LYL1	**LYL1 basic helix-loop-helix family member**	Nucleus	transcription		-5.651008577	-1	0.20873377	0	1	0	1.96557902	2.51077222	2.00101596	1.9680764
2973	**ENSG0000010490**	OAZ1	protein_coding	chr19	2269509	2273490	+	Human GRCh	OTTHUMG000001	OAZ1	**ornithine decarboxylase antizyme 1**	Cytoplasm	enzyme	eflornithin	1.08061357	0	0.68971851	0	1	0	12.3210036	12.506366	12.6136938	12.3817561
2974	**ENSG0000010490**	TRMT1	protein_coding	chr19	13104902	13117567	-	Human GRCh	OTTHUMG000001	TRMT1	**tRNA methyltransferase 1**	Extracellular	enzyme		-1.068334945	0	0.73862462	0	1	0	10.1940169	10.5015331	10.4539425	10.0841548
2975	**ENSG0000010491**	STX10	protein_coding	chr19	13144058	13150383	-	Human GRCh	OTTHUMG000001	STX10	**syntaxin 10**	Cytoplasm	other		-1.079987419	0	0.69897936	0	1	0	9.80452794	10.1801007	10.0168493	9.81692892
2978	**ENSG0000010493**	DMPK	protein_coding	chr19	45769709	45782552	-	Human GRCh	OTTHUMG000001	DMPK	**DM1 protein kinase**	Cytoplasm	kinase		-1.146157987	0	0.54513686	0	1	0	10.5636329	10.9821259	10.7592361	10.5047682
2980	**ENSG0000010494**	RSPH6A	protein_coding	chr19	45795713	45815308	-	Human GRCh	OTTHUMG000001	RSPH6A	**radial spoke head 6 homolog A**	Extracellular	other		-1.175051406	0	0.78751635	0	1	0	4.3802515	4.71052616	4.53116968	4.29267456
2981	**ENSG0000010494**	TBC1D17	protein_coding	chr19	49877666	49888750	+	Human GRCh	OTTHUMG000001	TBC1D17	**TBC1 domain family member 17**	Cytoplasm	other		-1.157676817	0	0.52583303	0	1	0	10.9788614	11.3626053	11.0352365	11.0052259
2982	**ENSG0000010495**	IL4I1	protein_coding	chr19	49889654	49929539	-	Human GRCh	OTTHUMG000001	IL4I1	**interleukin 4 induced 1**	Cytoplasm	enzyme		2.131802035	1	0.31148387	0	1	0	3.27158764	3.40673882	3.71584481	4.02802008
2983	**ENSG0000010495**	TLE6	protein_coding	chr19	2977538	2995179	+	Human GRCh	OTTHUMG000001	TLE6	**TLE family member 6, subcortical maternal complex member**	Nucleus	other		-1.196923134	0	0.5811347	0	1	0	6.10618383	6.36863118	6.10092141	6.02578903
2984	**ENSG0000010495**	YJU2B	protein_coding	chr19	13731760	13763296	+	Human GRCh	OTTHUMG000001	YJU2B	**YJU2 splicing factor homolog B**	Other	other		-1.079885082	0	0.73425641	0	1	0	9.72690858	9.95668873	9.81057849	9.71427424
2985	**ENSG0000010496**	PTOV1	protein_coding	chr19	49850735	49860744	+	Human GRCh	OTTHUMG000001	PTOV1	**PTOV1 extended AT-hook containing adaptor protein**	Nucleus	other		1.035471223	0	0.85605642	0	1	0	11.3177955	11.5150012	11.5789282	11.3263043
2986	**ENSG0000010496**	TLE5	protein_coding	chr19	3052910	3063107	-	Human GRCh	OTTHUMG000001	TLE5	**TLE family member 5, transcriptional modulator**	Nucleus	transcription		1.166526929	0	0.45171446	0	1	0	11.4460988	11.7525503	11.7442346	11.8115524
2988	**ENSG0000010496**	SGTA	protein_coding	chr19	2754715	2783362	-	Human GRCh	OTTHUMG000001	SGTA	**small glutamine rich tetratricopeptide repeat co-chaperone al**	Cytoplasm	other		-1.026582222	0	0.88274017	0	1	0	11.8984072	12.2136945	12.0502713	12.0231588
2990	**ENSG0000010497**	MED25	protein_coding	chr19	49818282	49840383	+	Human GRCh	OTTHUMG000001	MED25	**mediator complex subunit 25**	Nucleus	other		-1.051039016	0	0.81933303	0	1	0	10.4243636	10.6940325	10.6418011	10.3683327
2992	**ENSG0000010497**	SNAPC2	protein_coding	chr19	7920338	7923250	+	Human GRCh	OTTHUMG000001	SNAPC2	**small nuclear RNA activating complex polypeptide 2**	Nucleus	transcription		1.112452344	0	0.6904819	0	1	0	9.38128662	9.76323968	9.74703741	9.66839593
2993	**ENSG0000010497**	C19orf53	protein_coding	chr19	13774456	13778773	+	Human GRCh	OTTHUMG000001	C19orf53	**chromosome 19 open reading frame 53**	Nucleus	other		1.1909864	0	0.43414007	0	1	0	9.55484875	9.90801636	9.86635468	10.0110744
2994	**ENSG0000010498**	TIMM44	protein_coding	chr19	7926718	7943667	-	Human GRCh	OTTHUMG000001	TIMM44	**translocase of inner mitochondrial membrane 44**	Cytoplasm	transporter		1.107498163	0	0.63768814	0	1	0	10.7854698	11.0784149	11.2125959	10.8750818
2995	**ENSG0000010498**	CCDC61	protein_coding	chr19	45995461	46021318	+	Human GRCh	OTTHUMG000001	CCDC61	**coiled-coil domain containing 61**	Cytoplasm	other		1.222746206	0	0.37749506	0	1	0	7.61228287	7.76691145	7.92125981	7.88956868
2996	**ENSG0000010499**	IL27RA	protein_coding	chr19	14031762	14053218	+	Human GRCh	OTTHUMG000001	IL27RA	**interleukin 27 receptor subunit alpha**	Plasma Mem	transmembra		-1.185802649	0	0.77712148	0	1	0	4.40952547	4.07373731	4.28682648	3.91233128
2997	**ENSG0000010501**	ASF1B	protein_coding	chr19	14119512	14136613	-	Human GRCh	OTTHUMG000001	ASF1B	**anti-silencing function 1B histone chaperone**	Nucleus	other		-1.230551003	0	0.39999658	0	1	0	10.3191395	10.844104	10.4217671	10.3465695
2998	**ENSG0000010504**	TNNT1	protein_coding	chr19	55132698	55149206	-	Human GRCh	OTTHUMG000001	TNNT1	**troponin T1, slow skeletal type**	Cytoplasm	other		-1.094749914	0	0.71888404	0	1	0	8.6360259	8.69916068	8.67298796	8.45403565
2999	**ENSG0000010505**	VRK3	protein_coding	chr19	49976468	50025946	-	Human GRCh	OTTHUMG000001	VRK3	**VRK serine/threonine kinase 3**	Nucleus	kinase		-1.28234445	0	0.27803826	0	1	0	10.5334523	10.6878615	10.5182201	10.1139045
3000	**ENSG0000010505**	FAM32A	protein_coding	chr19	16185380	16192046	+	Human GRCh	OTTHUMG000001	FAM32A	**family with sequence similarity 32 member A**	Nucleus	other		-1.073604282	0	0.73376828	0	1	0	9.68152141	10.0158837	9.86493182	9.69235895
3001	**ENSG0000010506**	PPP6R1	protein_coding	chr19	55229779	55259017	-	Human GRCh	OTTHUMG000001	PPP6R1	**protein phosphatase 6 regulatory subunit 1**	Cytoplasm	other		1.09255905	0	0.65136234	0	1	0	11.8044219	12.1125759	12.1405623	11.9906978
3002	**ENSG0000010507**	C19orf44	protein_coding	chr19	16496394	16521352	+	Human GRCh	OTTHUMG000001	C19orf44	**chromosome 19 open reading frame 44**	Other	other		1.084352461	0	0.72156068	0	1	0	8.33054538	8.41162707	8.68313016	8.18252183
3003	**ENSG0000010508**	MED26	protein_coding	chr19	16574907	16629062	-	Human GRCh	OTTHUMG000001	MED26	**mediator complex subunit 26**	Nucleus	transcription		1.076917725	0	0.73371736	0	1	0	9.09777379	9.56762824	9.50601212	9.36446508
3004	**ENSG0000010508**	OLFM2	protein_coding	chr19	9853718	9936515	-	Human GRCh	OTTHUMG000001	OLFM2	**olfactomedin 2**	Cytoplasm	other		1.260226391	0	0.58679937	0	1	0	6.67839527	6.34921008	7.12801436	6.29739671
3005	**ENSG0000010512**	RASAL3	protein_coding	chr19	15451624	15464544	-	Human GRCh	OTTHUMG000001	RASAL3	**RAS protein activator like 3**	Cytoplasm	other		1.933263533	1	0.60723417	0	NA	0	2.35363107	2.78433168	2.93971863	3.28461042

3006	**ENSG0000010512**	AKAP8	protein_coding	chr19	15353385	15379815	-	Human GRCh	OTTHUMG000001	AKAP8	**A-kinase anchoring protein 8**	Nucleus	other		1.014486559	0	0.93470757	0	1	0	10.6107319	10.8465548	10.7979938	10.7005429
3007	**ENSG0000010513**	EPHX3	protein_coding	chr19	15226919	15233435	-	Human GRCh	OTTHUMG000001	EPHX3	**epoxide hydrolase 3**	Other	enzyme		-2.199012024	-1	0.14465951	0	1	0	5.05292802	5.78841583	4.82678774	4.68540473
3008	**ENSG0000010513**	ILVBL	protein_coding	chr19	15114984	15125786	-	Human GRCh	OTTHUMG000001	ILVBL	**ilvB acetolactate synthase like**	Cytoplasm	enzyme		-1.001706476	0	0.9944042	0	1	0	10.0371112	10.4154533	10.4218976	10.0235221
3009	**ENSG0000010513**	ZNF419	protein_coding	chr19	57487711	57496098	+	Human GRCh	OTTHUMG000000	ZNF419	**zinc finger protein 419**	Nucleus	transcription		-1.009115024	0	0.9742091	0	1	0	8.78238122	8.8973025	9.1087919	8.4673266
3010	**ENSG0000010513**	SYDE1	protein_coding	chr19	15107401	15114985	+	Human GRCh	OTTHUMG000001	SYDE1	**synapse defective Rho GTPase homolog 1**	Cytoplasm	other		-3.037049007	-1	0.44833809	0	NA	0	2.28737544	1.56789465	1.11662282	1.77051748
3013	**ENSG0000010514**	AURKC	protein_coding	chr19	57231009	57235548	+	Human GRCh	OTTHUMG000001	AURKC	**aurora kinase C**	Nucleus	kinase	tozasertib,	-1.049774418	0	0.9204949	0	1	0	4.81320971	5.4291894	5.28003671	4.92698689
3014	**ENSG0000010517**	POP4	protein_coding	chr19	29606283	29617237	+	Human GRCh	OTTHUMG000001	POP4	**POP4 homolog, ribonuclease P/MRP subunit**	Nucleus	enzyme		-1.080367475	0	0.7037771	0	1	0	8.93641426	9.19017394	9.10161883	8.85583396
3015	**ENSG0000010517**	CCNE1	protein_coding	chr19	29811991	29824312	+	Human GRCh	OTTHUMG000001	CCNE1	**cyclin E1**	Nucleus	transcription		-1.053702501	0	0.81611382	0	1	0	8.31908757	8.73871833	8.45016531	8.52710331
3016	**ENSG0000010517**	URI1	protein_coding	chr19	29923644	30016612	+	Human GRCh	OTTHUMG000001	URI1	**URI1 prefoldin like chaperone**	Nucleus	transcription		1.0669614	0	0.75123731	0	1	0	10.6157612	10.8818143	10.9713623	10.6646249
3017	**ENSG0000010518**	PDCD5	protein_coding	chr19	32581190	32587453	+	Human GRCh	OTTHUMG000001	PDCD5	**programmed cell death 5**	Nucleus	other		1.220553791	0	0.31264063	0	1	0	8.95719254	9.20369223	9.32712048	9.28298362
3018	**ENSG0000010518**	ANKRD27	protein_coding	chr19	32597006	32676597	-	Human GRCh	OTTHUMG000001	ANKRD27	**ankyrin repeat domain 27**	Cytoplasm	other		-1.039200285	0	0.84353644	0	1	0	10.8015268	11.1054131	11.0799914	10.7360394
3019	**ENSG0000010519**	RPS16	protein_coding	chr19	39433137	39435949	-	Human GRCh	OTTHUMG000001	RPS16	**ribosomal protein S16**	Cytoplasm	other		-1.029123602	0	0.88186527	0	1	0	13.4378599	13.6867342	13.6207431	13.4448024
3020	**ENSG0000010519**	TIMM50	protein_coding	chr19	39480412	39493785	+	Human GRCh	OTTHUMG000001	TIMM50	**translocase of inner mitochondrial membrane 50**	Cytoplasm	phosphatase		-1.011952367	0	0.95414273	0	1	0	10.3582147	10.9098185	10.7600829	10.5343134
3021	**ENSG0000010519**	LGALS13	protein_coding	chr19	39602524	39607474	+	Human GRCh	OTTHUMG000001	LGALS13	**galectin 13**	Nucleus	enzyme		1	0	1	0	NA	0	-1.1040467	-1.1016573	-1.1021149	-1.1039799
3022	**ENSG0000010520**	FBL	protein_coding	chr19	39834458	39846379	-	Human GRCh	OTTHUMG000001	FBL	**fibrillarin**	Nucleus	enzyme		-1.060395685	0	0.78040649	0	1	0	11.6823759	12.0683469	11.9353622	11.7016445
3023	**ENSG0000010520**	DYRK1B	protein_coding	chr19	39825350	39834201	-	Human GRCh	OTTHUMG000001	DYRK1B	**dual specificity tyrosine phosphorylation regulated kinase 1B**	Nucleus	kinase	GSK11738(	1.021140209	0	0.92597647	0	1	0	9.27675302	9.48363607	9.3357833	9.47443011
3025	**ENSG0000010521**	CCNP	protein_coding	chr19	40222208	40226697	-	Human GRCh	OTTHUMG000001	CCNP	**cyclin P**	Nucleus	other		1.112925152	0	0.77860702	0	1	0	6.68423799	7.2514901	6.64469621	7.44832382
3026	**ENSG0000010522**	GPI	protein_coding	chr19	34359480	34402413	+	Human GRCh	OTTHUMG000001	GPI	**glucose-6-phosphate isomerase**	Extracellular	enzyme		-1.090795351	0	0.69121148	0	1	0	14.0872951	14.5404157	14.3324402	14.1333564
3027	**ENSG0000010522**	AKT2	protein_coding	chr19	40230317	40285536	-	Human GRCh	OTTHUMG000001	AKT2	**AKT serine/threonine kinase 2**	Cytoplasm	kinase	capivaserti	1.013028363	0	0.94676902	0	1	0	12.2241583	12.5184532	12.5208207	12.2541527
3028	**ENSG0000010522**	PLD3	protein_coding	chr19	40348456	40389472	+	Human GRCh	OTTHUMG000001	PLD3	**phospholipase D family member 3**	Cytoplasm	enzyme		-1.018609818	0	0.93462995	0	1	0	11.5644665	11.7506204	11.7580141	11.5099918
3029	**ENSG0000010522**	PRX	protein_coding	chr19	40393766	40414793	-	Human GRCh	OTTHUMG000001	PRX	**periaxin**	Nucleus	other		1.07901063	0	0.83391586	0	1	0	6.99111844	7.48689507	7.43997148	7.24892093
3030	**ENSG0000010522**	PIAS4	protein_coding	chr19	4007736	4039386	+	Human GRCh	OTTHUMG000001	PIAS4	**protein inhibitor of activated STAT 4**	Nucleus	transcription		-1.066220051	0	0.75604119	0	1	0	10.2584997	10.5112153	10.3838229	10.2545542
3031	**ENSG0000010524**	NUMBL	protein_coding	chr19	40665905	40690972	-	Human GRCh	OTTHUMG000001	NUMBL	**NUMB like endocytic adaptor protein**	Cytoplasm	other		-1.012096389	0	0.96154941	0	1	0	9.66749221	10.0445973	9.98253048	9.71724096
3033	**ENSG0000010524**	YJU2	protein_coding	chr19	4247080	4269088	+	Human GRCh	OTTHUMG000001	YJU2	**YJU2 splicing factor homolog**	Nucleus	other		1.001268983	0	0.99560482	0	1	0	8.41582406	8.87544331	8.63775034	8.69829473
3034	**ENSG0000010525**	SHD	protein_coding	chr19	4279266	4290722	+	Human GRCh	OTTHUMG000001	SHD	**Src homology 2 domain containing transforming protein D**	Cytoplasm	other		-1.146276024	0	0.87780323	0	NA	0	3.56685405	2.76383417	2.74548679	3.43467798
3035	**ENSG0000010525**	TBCB	protein_coding	chr19	36114289	36125947	+	Human GRCh	OTTHUMG000000	TBCB	**tubulin folding cofactor B**	Cytoplasm	other		1.081350748	0	0.69094967	0	1	0	10.5554857	10.741995	10.8227377	10.6478515
3036	**ENSG0000010525**	FSD1	protein_coding	chr19	4304598	4323843	+	Human GRCh	OTTHUMG000001	FSD1	**fibronectin type III and SPRY domain containing 1**	Cytoplasm	other		-1.030885797	0	0.94355981	0	1	0	5.05365026	5.20018349	5.08002049	5.11759085
3037	**ENSG0000010525**	POLR2I	protein_coding	chr19	36113709	36115213	-	Human GRCh	OTTHUMG000001	POLR2I	**RNA polymerase II subunit I**	Nucleus	transcription		-1.035549537	0	0.86024977	0	1	0	9.55623324	9.85888013	9.67056261	9.68574782
3038	**ENSG0000010526**	OVOL3	protein_coding	chr19	36111143	36113711	+	Human GRCh	OTTHUMG000001	OVOL3	**ovo like zinc finger 3**	Nucleus	transcription		1.309791033	0	0.76672683	0	NA	0	2.15582969	2.62001467	2.35014249	2.62947879
3039	**ENSG0000010527**	CLIP3	protein_coding	chr19	36014660	36033343	-	Human GRCh	OTTHUMG000001	CLIP3	**CAP-Gly domain containing linker protein 3**	Plasma Mem	other		-1.043807437	0	0.88862182	0	1	0	6.36703198	6.82842953	6.63772172	6.51037324
3040	**ENSG0000010527**	ZFR2	protein_coding	chr19	3804024	3869038	-	Human GRCh	OTTHUMG000001	ZFR2	**zinc finger RNA binding protein 2**	Other	other		-1.300021407	0	0.75574393	0	1	0	2.45857717	1.34206564	2.39523758	1.23381038
3041	**ENSG0000010528**	SLC1A5	protein_coding	chr19	46774883	46788594	-	Human GRCh	OTTHUMG000001	SLC1A5	**solute carrier family 1 member 5**	Plasma Mem	transporter	MEDI7247	-1.498994169	0	0.15966489	0	1	0	10.2109434	9.69584089	9.45811629	9.66112525
3042	**ENSG0000010528**	PRKD2	protein_coding	chr19	46674275	46717127	-	Human GRCh	OTTHUMG000001	PRKD2	**protein kinase D2**	Cytoplasm	kinase	kb-NB 142	1.034007437	0	0.87533608	0	1	0	10.023385	10.2627152	10.167558	10.2045462
3043	**ENSG0000010528**	TJP3	protein_coding	chr19	3708362	3750813	+	Human GRCh	OTTHUMG000001	TJP3	**tight junction protein 3**	Cytoplasm	other		-1.129822293	0	0.74102907	0	1	0	10.0133511	10.4406395	10.2412461	9.95591921
3044	**ENSG0000010529**	APLP1	protein_coding	chr19	35867899	35879792	+	Human GRCh	OTTHUMG000001	APLP1	**amyloid beta precursor like protein 1**	Extracellular	other		1.125161967	0	0.84049056	0	1	0	7.47645483	7.62145437	7.84325297	7.46953332
3045	**ENSG0000010529**	CACTIN	protein_coding	chr19	3610645	3626815	-	Human GRCh	OTTHUMG000001	CACTIN	**cactin, spliceosome C complex subunit**	Nucleus	other		1.15198313	0	0.50860951	0	1	0	10.0241453	10.2944579	10.3232808	10.3249872
3046	**ENSG0000010532**	CCDC9	protein_coding	chr19	47255980	47273701	+	Human GRCh	OTTHUMG000001	CCDC9	**coiled-coil domain containing 9**	Extracellular	other		1.272856241	0	0.4107469	0	1	0	9.93093322	10.2499562	10.576139	10.1373639
3047	**ENSG0000010532**	HNRNPUL1	protein_coding	chr19	41262496	41307787	+	Human GRCh	OTTHUMG000001	HNRNPUL1	**heterogeneous nuclear ribonucleoprotein U like 1**	Nucleus	other		-1.076999811	0	0.7074278	0	1	0	13.0856632	13.4060332	13.2244309	13.1243352
3048	**ENSG0000010532**	FZR1	protein_coding	chr19	3506311	3538334	+	Human GRCh	OTTHUMG000001	FZR1	**fizzy and cell division cycle 20 related 1**	Nucleus	kinase		1.028805883	0	0.89797519	0	1	0	11.4389767	11.7290132	11.613684	11.6349017
3049	**ENSG0000010532**	BBC3	protein_coding	chr19	47220822	47232766	-	Human GRCh	OTTHUMG000001	BBC3	**BCL2 binding component 3**	Cytoplasm	other		1.228257857	0	0.48797217	0	1	0	8.32688516	8.64170645	8.53505938	8.88295818
3050	**ENSG0000010532**	TGFB1	protein_coding	chr19	41301587	41353922	-	Human GRCh	OTTHUMG000001	TGFB1	**transforming growth factor beta 1**	Extracellular	growth facto	STP707, YL	-1.018442287	0	0.95395603	0	1	0	7.08879391	6.96048319	7.14000694	6.86405003
3051	**ENSG0000010533**	DENND3	protein_coding	chr8	141117278	141195808	+	Human GRCh	OTTHUMG000001	DENND3	**DENN domain containing 3**	Cytoplasm	other		-1.40310748	0	0.36321067	0	1	0	6.53701114	7.08649281	6.6518586	6.4027047
3052	**ENSG0000010534**	DMAC2	protein_coding	chr19	41431318	41440717	-	Human GRCh	OTTHUMG000001	DMAC2	**distal membrane arm assembly component 2**	Cytoplasm	other		-1.090708823	0	0.65495995	0	1	0	9.68519185	9.70464438	9.66129415	9.53407325
3054	**ENSG0000010535**	PLIN3	protein_coding	chr19	4838341	4867694	-	Human GRCh	OTTHUMG000001	PLIN3	**perilipin 3**	Cytoplasm	other		-1.08792851	0	0.74974562	0	1	0	10.3788115	10.5977237	10.3251442	10.4655837
3055	**ENSG0000010535**	MYH14	protein_coding	chr19	50188186	50310542	+	Human GRCh	OTTHUMG000001	MYH14	**myosin heavy chain 14**	Extracellular	enzyme	mavacamt	1.253145579	0	0.20775595	0	1	0	11.0723339	11.059659	11.418786	11.1946842
3056	**ENSG0000010536**	MRPL4	protein_coding	chr19	10251901	10260055	+	Human GRCh	OTTHUMG000001	MRPL4	**mitochondrial ribosomal protein L4**	Cytoplasm	other		1.028922597	0	0.89275283	0	1	0	10.9966819	11.4243536	11.2859191	11.2374461
3058	**ENSG0000010536**	CD79A	protein_coding	chr19	41877279	41881372	+	Human GRCh	OTTHUMG000001	CD79A	**CD79a molecule**	Plasma Mem	transmembra		5.44755734	1	0.17898698	0	NA	0	1.94650347	1.47435481	2.14434183	2.68783962
3061	**ENSG0000010537**	RPS19	protein_coding	chr19	41860255	41872925	+	Human GRCh	OTTHUMG000001	RPS19	**ribosomal protein S19**	Cytoplasm	other		1.044130418	0	0.84098721	0	1	0	12.9511795	13.2419996	13.2865057	13.0047113
3062	**ENSG0000010537**	NOP53	protein_coding	chr19	47745546	47757058	+	Human GRCh	OTTHUMG000001	NOP53	**NOP53 ribosome biogenesis factor**	Cytoplasm	other		1.200633975	0	0.44529476	0	1	0	11.5737442	11.8580089	12.0484494	11.7995179
3064	**ENSG0000010537**	ICAM5	protein_coding	chr19	10289952	10296778	+	Human GRCh	OTTHUMG000001	ICAM5	**intercellular adhesion molecule 5**	Plasma Mem	other		1.065806612	0	0.82427208	0	1	0	10.3167613	10.6716368	10.7034104	10.4504047
3065	**ENSG0000010537**	ETFB	protein_coding	chr19	51345169	51366388	-	Human GRCh	OTTHUMG000001	ETFB	**electron transfer flavoprotein subunit beta**	Cytoplasm	transporter		1.160924741	0	0.4711322	0	1	0	9.78411364	10.2231662	10.2613004	10.1212468
3066	**ENSG0000010538**	CD33	protein_coding	chr19	51225064	51243860	+	Human GRCh	OTTHUMG000001	CD33	**CD33 molecule**	Plasma Mem	other	anti-CD33/	2.702753545	1	0.32703615	0	NA	0	1.76923753	2.18959217	2.51864656	2.65673412
3067	**ENSG0000010538**	CEACAM5	protein_coding	chr19	41708585	41730433	+	Human GRCh	OTTHUMG000001	CEACAM5	**CEA cell adhesion molecule 5**	Plasma Mem	other	F6-734, an	9.635560911	1	0.05515081	0	1	0	2.69361276	3.33227492	2.78448966	4.86409009
3069	**ENSG0000010539**	BABAM1	protein_coding	chr19	17267376	17281249	+	Human GRCh	OTTHUMG000001	BABAM1	**BRISC and BRCA1 A complex member 1**	Nucleus	other		-1.154543385	0	0.4650367	0	1	0	10.6266593	10.9354237	10.7304948	10.5231836
3070	**ENSG0000010539**	TYK2	protein_coding	chr19	10350533	10380608	-	Human GRCh	OTTHUMG000001	TYK2	**tyrosine kinase 2**	Cytoplasm	kinase	VTX958, ba	-1.057687772	0	0.79922975	0	1	0	11.2889481	11.6135951	11.4905902	11.3028477
3072	**ENSG0000010540**	CDC37	protein_coding	chr19	10391133	10420121	-	Human GRCh	OTTHUMG000001	CDC37	**cell division cycle 37, HSP90 cochaperone**	Cytoplasm	other		1.227434028	0	0.35848116	0	1	0	11.5553351	11.8877813	11.8412602	12.071884
3073	**ENSG0000010540**	NAPA	protein_coding	chr19	47487637	47515091	-	Human GRCh	OTTHUMG000001	NAPA	**NSF attachment protein alpha**	Cytoplasm	transporter		-1.04121883	0	0.84573634	0	1	0	11.4149991	11.7100607	11.6287015	11.4133771
3074	**ENSG0000010540**	RABAC1	protein_coding	chr19	41956681	41959321	-	Human GRCh	OTTHUMG000001	RABAC1	**Rab acceptor 1**	Cytoplasm	other		1.01704847	0	0.94521567	0	1	0	9.14172449	9.45941027	9.34350807	9.31572094
3075	**ENSG0000010540**	ATP1A3	protein_coding	chr19	41966582	41997497	-	Human GRCh	OTTHUMG000001	ATP1A3	**ATPase Na+/K+ transporting subunit alpha 3**	Plasma Mem	transporter	digoxin, et	1.150570559	0	0.65775759	0	1	0	9.56628614	9.67306744	9.58568673	9.9402598
3076	**ENSG0000010541**	MEIS3	protein_coding	chr19	47403124	47419527	-	Human GRCh	OTTHUMG000001	MEIS3	**Meis homeobox 3**	Nucleus	transcription		-1.249247711	0	0.41289338	0	1	0	7.68579532	7.60204363	7.65529795	7.09323987
3077	**ENSG0000010542**	PTPRS	protein_coding	chr19	5158495	5340812	-	Human GRCh	OTTHUMG000001	PTPRS	**protein tyrosine phosphatase receptor type S**	Plasma Mem	phosphatase		-1.030915776	0	0.88905675	0	1	0	12.7843879	12.8873936	12.9963281	12.5709707
3078	**ENSG0000010542**	CNFN	protein_coding	chr19	42387019	42390297	-	Human GRCh	OTTHUMG000001	CNFN	**cornifelin**	Cytoplasm	other		2.163123089	1	0.10306823	0	1	0	4.94176296	4.83314271	5.9461959	5.12924212
3080	**ENSG0000010542**	MEGF8	protein_coding	chr19	42325609	42378769	+	Human GRCh	OTTHUMG000001	MEGF8	**multiple EGF like domains 8**	Extracellular	other		1.006608682	0	0.97488751	0	1	0	11.7902314	11.9220138	12.0374679	11.6640167
3081	**ENSG0000010543**	KDELR1	protein_coding	chr19	48382575	48391551	-	Human GRCh	OTTHUMG000001	KDELR1	**KDEL endoplasmic reticulum protein retention receptor 1**	Cytoplasm	transporter		-1.043153073	0	0.82723448	0	1	0	12.1751812	12.4878795	12.3807596	12.2004531
3082	**ENSG0000010544**	CYTH2	protein_coding	chr19	48469208	48482314	+	Human GRCh	OTTHUMG000001	CYTH2	**cytohesin 2**	Cytoplasm	other		-1.012735039	0	0.95216758	0	1	0	12.6133727	12.8055774	12.8632072	12.5084763
3083	**ENSG0000010544**	GRWD1	protein_coding	chr19	48445841	48457022	+	Human GRCh	OTTHUMG000001	GRWD1	**glutamate rich WD repeat containing 1**	Nucleus	other		1.106004508	0	0.56661047	0	1	0	10.9504824	11.1930513	11.27852	11.0989992
3084	**ENSG0000010546**	GRIN2D	protein_coding	chr19	48393668	48444931	+	Human GRCh	OTTHUMG000001	GRIN2D	**glutamate ionotropic receptor NMDA type subunit 2D**	Plasma Mem	ion channel	dextromet	1.068758112	0	0.78424053	0	1	0	9.82176935	10.3233988	10.1822831	10.1685847
3085	**ENSG0000010546**	SYNGR4	protein_coding	chr19	48364367	48376377	+	Human GRCh	OTTHUMG000001	SYNGR4	**synaptogyrin 4**	Plasma Mem	other		-1.104785293	0	0.84858657	0	1	0	3.46028575	4.23835381	3.50195426	4.05589085
3086	**ENSG0000010547**	CLEC11A	protein_coding	chr19	50723364	50725708	+	Human GRCh	OTTHUMG000001	CLEC11A	**C-type lectin domain containing 11A**	Extracellular	growth facto		1.213183107	0	0.52544759	0	1	0	7.77668313	7.9724496	8.05265052	8.13466588
3087	**ENSG0000010547**	ODAD1	protein_coding	chr19	48296457	48321971	-	Human GRCh	OTTHUMG000001	ODAD1	**outer dynein arm docking complex subunit 1**	Extracellular	other		-1.323942592	0	0.32032244	0	1	0	8.48686654	8.75980752	8.64774659	7.89165754
3088	**ENSG0000010548**	CARD8	protein_coding	chr19	48180770	48255946	-	Human GRCh	OTTHUMG000001	CARD8	**caspase recruitment domain family member 8**	Nucleus	other		-1.071570117	0	0.77098438	0	1	0	9.62672614	9.85721335	9.89003984	9.41246851
3089	**ENSG0000010548**	LIG1	protein_coding	chr19	48115445	48170603	-	Human GRCh	OTTHUMG000001	LIG1	**DNA ligase 1**	Nucleus	enzyme		-1.075614809	0	0.71448677	0	1	0	11.5347993	11.8857706	11.811395	11.4470324
3091	**ENSG0000010549**	ZNF175	protein_coding	chr19	51571283	51592510	+	Human GRCh	OTTHUMG000001	ZNF175	**zinc finger protein 175**	Nucleus	transcription		-1.130381475	0	0.61000268	0	1	0	8.31018692	8.37212633	8.2944146	8.11868494
3092	**ENSG0000010549**	PLA2G4C	protein_coding	chr19	48047843	48110817	-	Human GRCh	OTTHUMG000001	PLA2G4C	**phospholipase A2 group IVC**	Plasma Mem	enzyme		-1.113830545	0	0.79650493	0	1	0	6.23118096	6.08130765	6.33839972	5.66521873
3095	**ENSG0000010551**	RAB3D	protein_coding	chr19	11322068	11346270	-	Human GRCh	OTTHUMG000001	RAB3D	**RAB3D, member RAS oncogene family**	Cytoplasm	enzyme		-1.254321755	0	0.34901941	0	1	0	11.0414254	11.4321429	11.1008088	10.8831321
3096	**ENSG0000010551**	DBP	protein_coding	chr19	48630030	48637379	-	Human GRCh	OTTHUMG000001	DBP	**D-box binding PAR bZIP transcription factor**	Nucleus	transcription		-1.035502331	0	0.89841898	0	1	0	9.19622562	9.22887041	9.1356273	9.21072181
3097	**ENSG0000010551**	TMEM205	protein_coding	chr19	11342776	11346518	-	Human GRCh	OTTHUMG000001	TMEM205	**transmembrane protein 205**	Cytoplasm	other		-1.14033314	0	0.58694868	0	1	0	10.6561036	10.9690702	10.760999	10.579628
3098	**ENSG0000010551**	CAPS	protein_coding	chr19	5912339	5916211	+	Human GRCh	OTTHUMG000001	CAPS	**calcyphosine**	Cytoplasm	other		-1.011138158	0	0.96919944	0	1	0	7.38218434	7.68331153	7.66989368	7.37221402
3099	**ENSG0000010552**	PLPPR2	protein_coding	chr19	11355386	11365698	+	Human GRCh	OTTHUMG000001	PLPPR2	**phospholipid phosphatase related 2**	Other	phosphatase		-1.048654896	0	0.83003562	0	1	0	9.50497277	10.0320648	9.82906746	9.65032923
3100	**ENSG0000010552**	FAM83E	protein_coding	chr19	48599961	48615076	-	Human GRCh	OTTHUMG000001	FAM83E	**family with sequence similarity 83 member E**	Other	other		1.210633507	0	0.53434951	0	1	0	5.87987385	5.97847053	6.10655801	6.11712122
3101	**ENSG0000010553**	RASIP1	protein_coding	chr19	48720585	48740610	-	Human GRCh	OTTHUMG000001	RASIP1	**Ras interacting protein 1**	Cytoplasm	other		1.148622177	0	0.77088722	0	1	0	7.57091507	7.95166523	7.98546825	7.87479745
3102	**ENSG0000010554**	THEG	protein_coding	chr19	361747	376026	-	Human GRCh	OTTHUMG000001	SPMAP2	**sperm microtubule associated protein 2**	Cytoplasm	other		1	0	1	0	1	0	1.79787558	1.92221794	1.90244178	1.80550375
3104	**ENSG0000010555**	BCAT2	protein_coding	chr19	48795062	48811029	-	Human GRCh	OTTHUMG000001	BCAT2	**branched chain amino acid transaminase 2**	Cytoplasm	enzyme		-1.008211575	0	0.97115538	0	1	0	10.7068542	11.0517249	11.014757	10.7333721

3105	**ENSG0000010555**	MIER2	protein_coding	chr19	305573	344815	-	Human GRCh	OTTHUMG000001	MIER2	**MIER family member 2**	Nucleus	transcription		1.018963821	0	0.93354117	0	1	0	9.38859113	9.7511456	9.78258876	9.393156
3106	**ENSG0000010555**	PLEKHA4	protein_coding	chr19	48837092	48868617	-	Human GRCh	OTTHUMG000001	PLEKHA4	**pleckstrin homology domain containing A4**	Cytoplasm	other		-1.051227747	0	0.86982741	0	1	0	8.08852135	8.48701002	8.27580263	8.21658767
3107	**ENSG0000010556**	PPP2R1A	protein_coding	chr19	52170936	52229518	+	Human GRCh	OTTHUMG000001	PPP2R1A	**protein phosphatase 2 scaffold subunit Aalpha**	Cytoplasm	phosphatase		-1.116867753	0	0.59574239	0	1	0	12.4829963	12.8177347	12.6879281	12.364227
3108	**ENSG0000010557**	TNPO2	protein_coding	chr19	12699201	12724011	-	Human GRCh	OTTHUMG000001	TNPO2	**transportin 2**	Nucleus	transporter		1.01995442	0	0.92326126	0	1	0	12.5776316	12.8118877	12.9136984	12.494503
3109	**ENSG0000010558**	WDR83OS	protein_coding	chr19	12668073	12669415	-	Human GRCh	OTTHUMG000001	WDR83OS	**WD repeat domain 83 opposite strand**	Cytoplasm	other		1.010450007	0	0.96293888	0	1	0	10.1871423	10.4060619	10.4204321	10.1953716
3111	**ENSG0000010560**	GCDH	protein_coding	chr19	12891160	12914207	+	Human GRCh	OTTHUMG000001	GCDH	**glutaryl-CoA dehydrogenase**	Cytoplasm	enzyme		-1.010978926	0	0.96034931	0	1	0	9.8527014	10.1026249	10.1133628	9.81182999
3114	**ENSG0000010561**	DNASE2	protein_coding	chr19	12875209	12881595	-	Human GRCh	OTTHUMG000001	DNASE2	**deoxyribonuclease 2, lysosomal**	Cytoplasm	enzyme		-1.066363094	0	0.78010755	0	1	0	10.199255	10.338036	10.2996603	10.0909889
3115	**ENSG0000010561**	MAST1	protein_coding	chr19	12833951	12874952	+	Human GRCh	OTTHUMG000001	MAST1	**microtubule associated serine/threonine kinase 1**	Cytoplasm	kinase		1.217907931	0	0.57262529	0	1	0	8.35810456	8.92747162	9.00407344	8.76339959
3116	**ENSG0000010561**	LENG1	protein_coding	chr19	54155161	54159721	-	Human GRCh	OTTHUMG000001	LENG1	**leukocyte receptor cluster member 1**	Nucleus	other		1.31912657	0	0.32606824	0	1	0	6.5873509	6.96523781	7.15755152	6.99784617
3117	**ENSG0000010561**	PRPF31	protein_coding	chr19	54115410	54131719	+	Human GRCh	OTTHUMG000000	PRPF31	**pre-mRNA processing factor 31**	Nucleus	other		-1.032415687	0	0.86982348	0	1	0	10.9153929	11.2402357	11.1154884	10.9858199
3118	**ENSG0000010561**	TFPT	protein_coding	chr19	54107020	54115657	-	Human GRCh	OTTHUMG000000	TFPT	**TCF3 fusion partner**	Nucleus	other		1.30084713	0	0.3040791	0	1	0	8.32149441	8.48852441	8.69531306	8.70125341
3119	**ENSG0000010563**	JAK3	protein_coding	chr19	17824780	17848071	-	Human GRCh	OTTHUMG000001	JAK3	**Janus kinase 3**	Cytoplasm	kinase	baricitinib,	-2.081306963	-1	0.2305223	0	1	0	5.52579427	5.42323369	3.94616863	5.20889962
3120	**ENSG0000010564**	RPL18A	protein_coding	chr19	17859910	17864153	+	Human GRCh	OTTHUMG000001	RPL18A	**ribosomal protein L18a**	Cytoplasm	other		1.062177949	0	0.7696182	0	1	0	13.1361596	13.3932673	13.4450536	13.2247012
3121	**ENSG0000010564**	SLC5A5	protein_coding	chr19	17871945	17895174	+	Human GRCh	OTTHUMG000001	SLC5A5	**solute carrier family 5 member 5**	Plasma Mem	transporter		-1.388707495	0	0.46942115	0	1	0	8.01095656	8.27709805	7.81259817	7.7227338
3122	**ENSG0000010564**	KCNN1	protein_coding	chr19	17951293	18000085	+	Human GRCh	OTTHUMG000001	KCNN1	**potassium calcium-activated channel subfamily N member 1**	Plasma Mem	ion channel		-1.071330292	0	0.87401628	0	1	0	7.28264053	7.63441376	7.45146788	7.33643325
3123	**ENSG0000010564**	ARRDC2	protein_coding	chr19	18001132	18014102	+	Human GRCh	OTTHUMG000001	ARRDC2	**arrestin domain containing 2**	Other	other		1.04072009	0	0.87458209	0	1	0	8.87972318	9.08299118	9.14003849	8.90458416
3124	**ENSG0000010564**	PIK3R2	protein_coding	chr19	18153163	18170532	+	Human GRCh	OTTHUMG000001	PIK3R2	**phosphoinositide-3-kinase regulatory subunit 2**	Cytoplasm	kinase		-1.022373285	0	0.90689401	0	1	0	11.3639277	11.6812928	11.5654684	11.4483259
3125	**ENSG0000010564**	RAB3A	protein_coding	chr19	18196784	18204042	-	Human GRCh	OTTHUMG000001	RAB3A	**RAB3A, member RAS oncogene family**	Cytoplasm	enzyme		1.127417981	0	0.68494652	0	1	0	9.2226355	9.54891125	9.49688429	9.57040517
3126	**ENSG0000010565**	PDE4C	protein_coding	chr19	18207965	18248200	-	Human GRCh	OTTHUMG000001	PDE4C	**phosphodiesterase 4C**	Cytoplasm	enzyme	caffeine, a	-1.932242458	-1	0.18609763	0	1	0	3.49220022	4.84530335	4.05968945	3.62603193
3127	**ENSG0000010565**	ISYNA1	protein_coding	chr19	18434388	18438167	-	Human GRCh	OTTHUMG000001	ISYNA1	**inositol-3-phosphate synthase 1**	Cytoplasm	enzyme		-1.085463084	0	0.74762263	0	1	0	11.2755761	11.6686592	11.3298456	11.4594171
3128	**ENSG0000010565**	ELL	protein_coding	chr19	18442663	18522116	-	Human GRCh	OTTHUMG000001	ELL	**elongation factor for RNA polymerase II**	Nucleus	transcription		1.024987494	0	0.90939506	0	1	0	9.51849847	10.0655824	9.84590029	9.8545527
3129	**ENSG0000010566**	CRTC1	protein_coding	chr19	18683678	18782733	+	Human GRCh	OTTHUMG000001	CRTC1	**CREB regulated transcription coactivator 1**	Nucleus	transcription	CC-115, sa	1.067307699	0	0.76351967	0	1	0	10.4118945	10.6313303	10.6149571	10.5783285
3130	**ENSG0000010566**	COMP	protein_coding	chr19	18782773	18791305	-	Human GRCh	OTTHUMG000001	COMP	**cartilage oligomeric matrix protein**	Extracellular	other		5.40483451	1	0.04181361	1	1	0	3.11595854	3.41658806	2.93945346	5.33073094
3132	**ENSG0000010566**	COPE	protein_coding	chr19	18899514	18919387	-	Human GRCh	OTTHUMG000001	COPE	**COPI coat complex subunit epsilon**	Cytoplasm	transporter		1.061046296	0	0.7877851	0	1	0	11.5252705	11.8733931	11.8598942	11.6917826
3133	**ENSG0000010567**	DDX49	protein_coding	chr19	18919705	18929189	+	Human GRCh	OTTHUMG000001	DDX49	**DEAD-box helicase 49**	Nucleus	enzyme		-1.015227558	0	0.94343244	0	1	0	10.4989666	10.8165829	10.7187031	10.5795596
3134	**ENSG0000010567**	ETV2	protein_coding	chr19	35641745	35644871	+	Human GRCh	OTTHUMG000001	ETV2	**ETS variant transcription factor 2**	Nucleus	transcription		1.236393209	0	0.68577493	0	1	0	4.68195943	4.73016059	5.06096776	4.6931213
3135	**ENSG0000010567**	ATP4A	protein_coding	chr19	35550031	35563658	-	Human GRCh	OTTHUMG000000	ATP4A	**ATPase H+/K+ transporting subunit alpha**	Plasma Mem	transporter	ilaprazole,	6.000680641	1	0.02409341	1	1	0	2.25001681	2.75039638	3.00504778	4.35291816
3136	**ENSG0000010567**	ARMC6	protein_coding	chr19	19033575	19060311	+	Human GRCh	OTTHUMG000001	ARMC6	**armadillo repeat containing 6**	Cytoplasm	other		-1.070496771	0	0.74300267	0	1	0	10.2408141	10.6343598	10.5396445	10.1912203
3137	**ENSG0000010567**	TMEM147	protein_coding	chr19	35545600	35547526	+	Human GRCh	OTTHUMG000000	TMEM147	**transmembrane protein 147**	Cytoplasm	other		-1.072183542	0	0.72272184	0	1	0	10.3531695	10.6300363	10.4086522	10.4349447
3140	**ENSG0000010569**	TMEM59L	protein_coding	chr19	18607430	18621039	+	Human GRCh	OTTHUMG000001	TMEM59L	**transmembrane protein 59 like**	Cytoplasm	other		1.180755689	0	0.77039905	0	1	0	5.06180275	5.24434726	5.23358608	5.39993273
3141	**ENSG0000010569**	HAMP	protein_coding	chr19	35280716	35285143	+	Human GRCh	OTTHUMG000001	HAMP	**hepcidin antimicrobial peptide**	Extracellular	other	lexaptepid	-1.822161014	-1	0.56940712	0	NA	0	2.10128606	3.04095702	2.46049509	2.25211408
3142	**ENSG0000010569**	USF2	protein_coding	chr19	35268962	35279821	+	Human GRCh	OTTHUMG000001	USF2	**upstream transcription factor 2, c-fos interacting**	Nucleus	transcription		-1.007047631	0	0.96871835	0	1	0	11.2856164	11.5177063	11.4152275	11.3838864
3143	**ENSG0000010569**	LSR	protein_coding	chr19	35248330	35267964	+	Human GRCh	OTTHUMG000001	LSR	**lipolysis stimulated lipoprotein receptor**	Nucleus	transcription		-1.009744922	0	0.96368927	0	1	0	11.3493648	11.626792	11.5798339	11.3830134
3144	**ENSG0000010570**	KXD1	protein_coding	chr19	18557762	18569387	+	Human GRCh	OTTHUMG000001	KXD1	**KxDL motif containing 1**	Cytoplasm	other		-1.108922294	0	0.5700577	0	1	0	10.6332971	10.8815834	10.7546786	10.5335085
3145	**ENSG0000010570**	FKBP8	protein_coding	chr19	18531751	18544077	-	Human GRCh	OTTHUMG000001	FKBP8	**FKBP prolyl isomerase 8**	Cytoplasm	other		1.195338796	0	0.41053178	0	1	0	11.5772328	11.9210041	12.0284976	11.8899492
3146	**ENSG0000010570**	SUGP1	protein_coding	chr19	19276018	19320509	-	Human GRCh	OTTHUMG000001	SUGP1	**SURP and G-patch domain containing 1**	Nucleus	other		-1.182385349	0	0.41379874	0	1	0	10.2006322	10.569102	10.2421497	10.1821468
3147	**ENSG0000010570**	HPN	protein_coding	chr19	35040506	35066573	+	Human GRCh	OTTHUMG000001	HPN	**hepsin**	Plasma Mem	peptidase		1.004987142	0	0.98764537	0	1	0	8.7743205	9.24425293	9.09874574	8.97353885
3148	**ENSG0000010570**	ZNF14	protein_coding	chr19	19710472	19733112	-	Human GRCh	OTTHUMG000001	ZNF14	**zinc finger protein 14**	Nucleus	transcription		-1.133667368	0	0.61334844	0	1	0	8.38672234	8.49282269	8.4955164	8.08220699
3149	**ENSG0000010571**	SCN1B	protein_coding	chr19	35030470	35040449	+	Human GRCh	OTTHUMG000001	SCN1B	**sodium voltage-gated channel beta subunit 1**	Plasma Mem	ion channel	procaine, r	-1.425527802	0	0.34090412	0	1	0	6.19688714	6.40364579	6.18065227	5.70239123
3150	**ENSG0000010571**	PBX4	protein_coding	chr19	19561707	19618693	-	Human GRCh	OTTHUMG000001	PBX4	**PBX homeobox 4**	Nucleus	transcription		-1.228349561	0	0.48582094	0	1	0	7.23014517	8.04165687	7.60312432	7.35531776
3151	**ENSG0000010572**	ERF	protein_coding	chr19	42247569	42255128	-	Human GRCh	OTTHUMG000001	ERF	**ETS2 repressor factor**	Nucleus	transcription		1.089386994	0	0.69943543	0	1	0	11.2269697	11.5332534	11.5424895	11.4310539
3152	**ENSG0000010572**	GSK3A	protein_coding	chr19	42226225	42242625	-	Human GRCh	OTTHUMG000001	GSK3A	**glycogen synthase kinase 3 alpha**	Nucleus	kinase	aloisine A,	1.02989736	0	0.8647701	0	1	0	11.0770931	11.4076491	11.309025	11.2643307
3153	**ENSG0000010572**	ATP13A1	protein_coding	chr19	19645198	19663676	-	Human GRCh	OTTHUMG000001	ATP13A1	**ATPase 13A1**	Extracellular	transporter		-1.122870288	0	0.56920651	0	1	0	12.0452986	12.4755611	12.2471171	12.0416652
3154	**ENSG0000010573**	ZNF574	protein_coding	chr19	42068477	42081552	+	Human GRCh	OTTHUMG000001	ZNF574	**zinc finger protein 574**	Other	other		-1.006328913	0	0.97530875	0	1	0	9.64305542	9.99884031	9.89047964	9.76001041
3155	**ENSG0000010573**	GRIK5	protein_coding	chr19	41998321	42070206	-	Human GRCh	OTTHUMG000000	GRIK5	**glutamate ionotropic receptor kainate type subunit 5**	Plasma Mem	ion channel		-1.244118445	0	0.4438945	0	1	0	6.08845729	6.6766057	5.80113881	6.50094395
3156	**ENSG0000010573**	SIPA1L3	protein_coding	chr19	37907208	38208369	+	Human GRCh	OTTHUMG000000	SIPA1L3	**signal induced proliferation associated 1 like 3**	Extracellular	other		1.01536045	0	0.93695935	0	1	0	11.0892814	11.2598297	11.2632547	11.1191669
3157	**ENSG0000010575**	ZNF85	protein_coding	chr19	20923222	20950697	+	Human GRCh	OTTHUMG000001	ZNF85	**zinc finger protein 85**	Nucleus	transcription		-1.032637151	0	0.89247215	0	1	0	8.90706224	9.13803673	9.20606456	8.7325595
3158	**ENSG0000010575**	ETHE1	protein_coding	chr19	43506719	43527230	-	Human GRCh	OTTHUMG000001	ETHE1	**ETHE1 persulfide dioxygenase**	Cytoplasm	enzyme		1.211603722	0	0.52058128	0	1	0	7.02507005	7.2449205	7.33989544	7.36515492
3159	**ENSG0000010576**	CADM4	protein_coding	chr19	43622368	43639850	-	Human GRCh	OTTHUMG000001	CADM4	**cell adhesion molecule 4**	Plasma Mem	other		1.160524845	0	0.61596861	0	1	0	8.20686858	8.3043138	8.659699	8.10923482
3160	**ENSG0000010577**	SMG9	protein_coding	chr19	43727983	43754962	-	Human GRCh	OTTHUMG000001	SMG9	**SMG9 nonsense mediated mRNA decay factor**	Cytoplasm	other		1.015257305	0	0.94280626	0	1	0	10.0076789	10.2593426	10.3017972	9.99154772
3161	**ENSG0000010577**	AVL9	protein_coding	chr7	32495426	32588726	+	Human GRCh	OTTHUMG000001	AVL9	**AVL9 cell migration associated**	Cytoplasm	other		-1.109689652	0	0.62812691	0	1	0	11.5435525	11.858889	11.8030623	11.3480279
3162	**ENSG0000010578**	RUNDC3B	protein_coding	chr7	87627548	87832296	+	Human GRCh	OTTHUMG000001	RUNDC3B	**RUN domain containing 3B**	Other	other		-1.107387883	0	0.81967337	0	1	0	7.28613408	7.62137935	7.47080598	7.21227551
3163	**ENSG0000010579**	CFAP69	protein_coding	chr7	90245174	90311063	+	Human GRCh	OTTHUMG000000	CFAP69	**cilia and flagella associated protein 69**	Cytoplasm	other		-1.215352441	0	0.53782817	0	1	0	8.54309204	8.5430876	8.56878076	8.02423573
3164	**ENSG0000010579**	GTPBP10	protein_coding	chr7	90335223	90391453	+	Human GRCh	OTTHUMG000000	GTPBP10	**GTP binding protein 10**	Cytoplasm	other		1.063000927	0	0.79084511	0	1	0	10.0197158	10.2496298	10.4738499	9.86415238
3165	**ENSG0000010580**	RASA4	protein_coding	chr7	102579646	102616756	-	Human GRCh	OTTHUMG000001	RASA4	**RAS p21 protein activator 4**	Cytoplasm	other		1.04161418	0	0.90117564	0	1	0	7.06968833	7.46651649	7.50822822	7.11589434
3166	**ENSG0000010581**	CDK6	protein_coding	chr7	92604921	92836573	-	Human GRCh	OTTHUMG000001	CDK6	**cyclin dependent kinase 6**	Nucleus	kinase	fulvestrant	2.030351704	1	0.3141749	0	1	0	8.52379014	8.50644648	8.69794564	9.68469959
3167	**ENSG0000010581**	PMPCB	protein_coding	chr7	103297407	103331818	+	Human GRCh	OTTHUMG000001	PMPCB	**peptidase, mitochondrial processing subunit beta**	Cytoplasm	peptidase		-1.076207158	0	0.725855	0	1	0	10.7081502	10.9954304	10.9308545	10.6047075
3168	**ENSG0000010582**	DNAJC2	protein_coding	chr7	103312289	103344830	-	Human GRCh	OTTHUMG000001	DNAJC2	**DnaJ heat shock protein family (Hsp40) member C2**	Nucleus	transcription		1.045356674	0	0.82193694	0	1	0	9.94276164	10.2645818	10.3229412	9.97983805
3170	**ENSG0000010582**	BET1	protein_coding	chr7	93962762	94004382	-	Human GRCh	OTTHUMG000000	BET1	**Bet1 golgi vesicular membrane trafficking protein**	Cytoplasm	transporter		1.115724693	0	0.65273806	0	1	0	8.76910474	9.01702616	9.2494493	8.73594717
3171	**ENSG0000010583**	NAMPT	protein_coding	chr7	106248298	106285966	-	Human GRCh	OTTHUMG000001	NAMPT	**nicotinamide phosphoribosyltransferase**	Extracellular	cytokine	OT-82, STF	1.019883685	0	0.92523816	0	1	0	11.9928298	12.1567816	12.2845882	11.8814196
3172	**ENSG0000010584**	POLR1F	protein_coding	chr7	19695461	19709037	-	Human GRCh	OTTHUMG000001	POLR1F	**RNA polymerase I subunit F**	Nucleus	other		-1.008314503	0	0.97274786	0	1	0	9.85201559	10.3531287	10.3375501	9.85214393
3175	**ENSG0000010585**	PON2	protein_coding	chr7	95404862	95435329	-	Human GRCh	OTTHUMG000001	PON2	**paraoxonase 2**	Plasma Mem	enzyme		-1.086602154	0	0.69127771	0	1	0	10.1957383	10.5098747	10.3574449	10.1771734
3176	**ENSG0000010585**	ITGB8	protein_coding	chr7	20330702	20415754	+	Human GRCh	OTTHUMG000000	ITGB8	**integrin subunit beta 8**	Plasma Mem	other		-1.108842365	0	0.76828434	0	1	0	7.93931945	8.32400475	8.38070543	7.53878851
3177	**ENSG0000010585**	HBP1	protein_coding	chr7	107168961	107202522	+	Human GRCh	OTTHUMG000001	HBP1	**HMG-box transcription factor 1**	Nucleus	transcription		-1.391466809	0	0.12788482	0	1	0	11.0531789	11.3839989	10.8888708	10.8260062
3178	**ENSG0000010586**	DUS4L	protein_coding	chr7	107563484	107578523	+	Human GRCh	OTTHUMG000001	DUS4L	**dihydrouridine synthase 4 like**	Other	other		1.036202899	0	0.87932379	0	1	0	8.58599283	8.65552708	8.75147039	8.56171145
3179	**ENSG0000010586**	SP4	protein_coding	chr7	21428043	21514822	+	Human GRCh	OTTHUMG000000	SP4	**Sp4 transcription factor**	Nucleus	transcription		-1.1946626	0	0.41295824	0	1	0	9.6594209	9.74787675	9.61419208	9.41850618
3180	**ENSG0000010587**	WDR91	protein_coding	chr7	135183839	135211555	-	Human GRCh	OTTHUMG000001	WDR91	**WD repeat domain 91**	Cytoplasm	other		-1.002366688	0	0.99325114	0	1	0	8.89465019	9.19357362	9.0276454	9.07321723
3181	**ENSG0000010587**	DNAH11	protein_coding	chr7	21543039	21901839	+	Human GRCh	OTTHUMG000001	DNAH11	**dynein axonemal heavy chain 11**	Cytoplasm	enzyme		-1.027446963	0	0.94570803	0	1	0	7.19365133	7.86205774	7.2430954	7.7841834
3182	**ENSG0000010587**	CBLL1	protein_coding	chr7	107743073	107761667	+	Human GRCh	OTTHUMG000001	CBLL1	**Cbl proto-oncogene like 1**	Nucleus	enzyme		1.004415225	0	0.98471392	0	1	0	10.8079581	11.0913587	11.1720281	10.7094055
3183	**ENSG0000010588**	DLX5	protein_coding	chr7	97020396	97024950	-	Human GRCh	OTTHUMG000001	DLX5	**distal-less homeobox 5**	Nucleus	transcription		1.058450264	0	0.82426826	0	1	0	9.43671944	9.73051854	9.84702365	9.43186863
3184	**ENSG0000010588**	MTPN	protein_coding	chr7	135926760	135977359	-	Human GRCh	OTTHUMG000001	MTPN	**myotrophin**	Nucleus	transcription		-1.005440601	0	0.97888954	0	1	0	11.8258478	12.1570337	12.1115789	11.8698784
3185	**ENSG0000010588**	STEAP1B	protein_coding	chr7	22419444	22727613	-	Human GRCh	OTTHUMG000001	STEAP1B	**STEAP family member 1B**	Other	other		-11.81756301	-1	0.10554515	0	1	0	1.42507055	2.41391876	1.46055441	1.42758461
3186	**ENSG0000010589**	PTN	protein_coding	chr7	137227341	137343774	-	Human GRCh	OTTHUMG000001	PTN	**pleiotrophin**	Extracellular	growth facto		2.092062486	1	0.58533272	0	1	0	3.08448357	2.6910442	3.19355254	3.09235969
3187	**ENSG0000010592**	PALS2	protein_coding	chr7	24573268	24694193	+	Human GRCh	OTTHUMG000000	PALS2	**protein associated with LIN7 2, MAGUK p55 family member**	Plasma Mem	kinase		1.144356422	0	0.6215687	0	1	0	10.1336679	10.2583911	10.4534617	10.2247031
3188	**ENSG0000010592**	GSDME	protein_coding	chr7	24698355	24757460	-	Human GRCh	OTTHUMG000000	GSDME	**gasdermin E**	Plasma Mem	transporter		-1.153162042	0	0.7951614	0	1	0	4.57944145	3.92838474	4.31762049	4.0703853
3190	**ENSG0000010593**	ZC3HAV1	protein_coding	chr7	139043515	139132122	-	Human GRCh	OTTHUMG000001	ZC3HAV1	**zinc finger CCCH-type containing, antiviral 1**	Plasma Mem	other		-1.25781494	0	0.3181514	0	1	0	11.4523009	11.7663701	11.3423687	11.3860073
3191	**ENSG0000010594**	TTC26	protein_coding	chr7	139133744	139191986	+	Human GRCh	OTTHUMG000001	IFT56	**intraflagellar transport 56**	Extracellular	other		-1.091119762	0	0.72674411	0	1	0	8.83312928	9.05599264	9.06344074	8.60123835
3192	**ENSG0000010595**	OGDH	protein_coding	chr7	44606572	44709066	+	Human GRCh	OTTHUMG000001	OGDH	**oxoglutarate dehydrogenase**	Cytoplasm	enzyme		-1.093985487	0	0.66513805	0	1	0	11.7281947	12.0081684	11.8923343	11.6483829
3194	**ENSG0000010596**	ADAP1	protein_coding	chr7	897900	955407	-	Human GRCh	OTTHUMG000000	ADAP1	**ArfGAP with dual PH domains 1**	Nucleus	other		1.468939052	0	0.4671918	0	1	0	4.09594451	3.6974211	4.60386169	3.63929547
3196	**ENSG0000010596**	H2AZ2	protein_coding	chr7	44826791	44848087	-	Human GRCh	OTTHUMG000001	H2AZ2	**H2A.Z variant histone 2**	Nucleus	other		-1.021450193	0	0.92410583	0	1	0	12.6760549	12.9066558	12.9586036	12.5556113
3197	**ENSG0000010597**	CAV2	protein_coding	chr7	116287380	116508541	+	Human GRCh	OTTHUMG000000	CAV2	**caveolin 2**	Plasma Mem	other		-1.408227844	0	0.66184736	0	1	0	3.68987018	3.15206957	3.12502728	3.21782388
3198	**ENSG0000010597**	CAV1	protein_coding	chr7	116524994	116561179	+	Human GRCh	OTTHUMG000000	CAV1	**caveolin 1**	Plasma Mem	transmembra	GSK12788(	1.037739412	0	0.97014033	0	1	0	3.36675267	4.56699042	4.31270745	3.97331769
3199	**ENSG0000010597**	MET	protein_coding	chr7	116672196	116798377	+	Human GRCh	OTTHUMG000000	MET	**MET proto-oncogene, receptor tyrosine kinase**	Plasma Mem	kinase	RXDX-106,	1.786519392	1	0.43485379	0	1	0	5.00704841	4.41233967	4.83786639	5.40195661

3200	**ENSG0000010598**	RNF32	protein_coding	chr7	156640281	156677130	+	Human GRCh	OTTHUMG000001	RNF32	**ring finger protein 32**	Cytoplasm	other		1.273447181	0	0.51818955	0	1	0	5.2627684	5.91059583	5.74168487	5.98800051
3201	**ENSG0000010598**	LMBR1	protein_coding	chr7	156668946	156893216	-	Human GRCh	OTTHUMG000001	LMBR1	**limb development membrane protein 1**	Plasma Mem	transmembra		-1.11830555	0	0.58873724	0	1	0	11.9583492	12.175121	12.0973978	11.770413
3203	**ENSG0000010599**	HOXA1	protein_coding	chr7	27092993	27096000	-	Human GRCh	OTTHUMG000000	HOXA1	**homeobox A1**	Nucleus	transcription		2.273695643	1	0.02341894	1	1	0	5.79257741	5.71443771	6.79331877	6.30544391
3204	**ENSG0000010599**	DNAJB6	protein_coding	chr7	157335381	157417439	+	Human GRCh	OTTHUMG000001	DNAJB6	**DnaJ heat shock protein family (Hsp40) member B6**	Nucleus	transcription		1.008478558	0	0.96425269	0	1	0	11.2915979	11.5320284	11.576181	11.2572411
3205	**ENSG0000010599**	HOXA2	protein_coding	chr7	27100354	27102686	-	Human GRCh	OTTHUMG000000	HOXA2	**homeobox A2**	Nucleus	transcription		-1.341962694	0	0.67618816	0	1	0	4.24040857	4.57048132	4.06949542	4.25147678
3206	**ENSG0000010599**	HOXA3	protein_coding	chr7	27106184	27152583	-	Human GRCh	OTTHUMG000000	HOXA3	**homeobox A3**	Nucleus	transcription		1.491172293	0	0.37568287	0	1	0	6.77966668	6.83592511	7.28618665	7.16040288
3207	**ENSG0000010600**	LFNG	protein_coding	chr7	2512529	2529177	+	Human GRCh	OTTHUMG000001	LFNG	**LFNG O-fucosylpeptide 3-beta-N-acetylglucosaminyltransferas**	Cytoplasm	enzyme		1.980604848	1	0.04556346	1	1	0	7.9518399	8.51038056	8.37791071	9.32893592
3208	**ENSG0000010600**	HOXA5	protein_coding	chr7	27141052	27143681	-	Human GRCh	OTTHUMG000000	HOXA5	**homeobox A5**	Nucleus	transcription		-1.035562919	0	0.92487713	0	1	0	7.4291748	7.42162151	7.31235117	7.46022484
3209	**ENSG0000010600**	HOXA6	protein_coding	chr7	27145396	27150603	-	Human GRCh	OTTHUMG000000	HOXA6	**homeobox A6**	Nucleus	transcription		1.220844242	0	0.6348148	0	1	0	7.15914246	6.82205928	7.35942493	7.04321722
3210	**ENSG0000010600**	BRAT1	protein_coding	chr7	2537810	2555694	-	Human GRCh	OTTHUMG000001	BRAT1	**BRCA1 associated ATM activator 1**	Nucleus	other		1.005986469	0	0.97536377	0	1	0	10.8206489	11.1187639	11.0292769	10.9401944
3211	**ENSG0000010601**	IQCE	protein_coding	chr7	2558972	2614733	+	Human GRCh	OTTHUMG000001	IQCE	**IQ motif containing E**	Cytoplasm	other		-1.044004216	0	0.82720957	0	1	0	10.7666239	11.0456213	10.9649257	10.7579874
3214	**ENSG0000010602**	TSPAN12	protein_coding	chr7	120787320	120858402	-	Human GRCh	OTTHUMG000001	TSPAN12	**tetraspanin 12**	Plasma Mem	other		1.297238515	0	0.37622721	0	1	0	7.35356323	7.639975	7.66669343	7.87310607
3215	**ENSG0000010602**	SSBP1	protein_coding	chr7	141738334	141787922	+	Human GRCh	OTTHUMG000001	SSBP1	**single stranded DNA binding protein 1**	Cytoplasm	other		1.035176736	0	0.87473895	0	1	0	10.1281488	10.5307212	10.4416652	10.3255228
3216	**ENSG0000010603**	HOXA13	protein_coding	chr7	27193503	27200091	-	Human GRCh	OTTHUMG000000	HOXA13	**homeobox A13**	Nucleus	transcription		-1.845528465	-1	0.60388326	0	1	0	4.53440354	3.8213505	4.15041992	3.37714949
3217	**ENSG0000010603**	CPED1	protein_coding	chr7	120988697	121297442	+	Human GRCh	OTTHUMG000001	CPED1	**cadherin like and PC-esterase domain containing 1**	Cytoplasm	other		1.110820145	0	0.94821184	0	NA	0	1.57532262	1.15764512	1.64373161	1.13717854
3218	**ENSG0000010603**	EVX1	protein_coding	chr7	27242700	27250493	+	Human GRCh	OTTHUMG000000	EVX1	**even-skipped homeobox 1**	Nucleus	transcription		-79619.94635	-1	4.62E-05	1	0.09	1	0.47660508	1.55962883	0.55923008	0.48265313
3219	**ENSG0000010604**	HIBADH	protein_coding	chr7	27525442	27662883	-	Human GRCh	OTTHUMG000000	HIBADH	**3-hydroxyisobutyrate dehydrogenase**	Cytoplasm	enzyme		-1.043800132	0	0.8614547	0	1	0	9.55119557	9.84840216	9.88757967	9.38228225
3220	**ENSG0000010605**	TAX1BP1	protein_coding	chr7	27739331	27844564	+	Human GRCh	OTTHUMG000000	TAX1BP1	**Tax1 binding protein 1**	Cytoplasm	other		-1.086497241	0	0.73292084	0	1	0	10.4840057	10.8717018	10.6987507	10.500868
3221	**ENSG0000010606**	CPVL	protein_coding	chr7	28995235	29195451	-	Human GRCh	OTTHUMG000000	CPVL	**carboxypeptidase vitellogenic like**	Cytoplasm	peptidase		1.943614177	1	0.27351977	0	1	0	3.06826479	3.52810855	3.50481359	3.83494606
3222	**ENSG0000010606**	CHN2	protein_coding	chr7	29146547	29514328	+	Human GRCh	OTTHUMG000000	CHN2	**chimerin 2**	Cytoplasm	other		1.264788224	0	0.49158555	0	1	0	8.48143131	8.4073408	8.00435879	9.10217429
3223	**ENSG0000010607**	GRB10	protein_coding	chr7	50590063	50793462	-	Human GRCh	OTTHUMG000001	GRB10	**growth factor receptor bound protein 10**	Cytoplasm	other		-1.145545323	0	0.69105633	0	1	0	10.0142563	9.58526799	9.46294293	9.86838441
3224	**ENSG0000010607**	ABHD11	protein_coding	chr7	73736094	73738852	-	Human GRCh	OTTHUMG000001	ABHD11	**abhydrolase domain containing 11**	Cytoplasm	enzyme		1.012682747	0	0.95559204	0	1	0	10.0367317	10.3942527	10.3338894	10.1440688
3225	**ENSG0000010607**	COBL	protein_coding	chr7	51016212	51316818	-	Human GRCh	OTTHUMG000001	COBL	**cordon-bleu WH2 repeat protein**	Plasma Mem	other		-1.11871939	0	0.60601542	0	1	0	10.2047639	10.5453754	10.2955766	10.2381518
3226	**ENSG0000010608**	FKBP14	protein_coding	chr7	30010587	30026702	-	Human GRCh	OTTHUMG000000	FKBP14	**FKBP prolyl isomerase 14**	Cytoplasm	enzyme		-1.219356944	0	0.48890617	0	1	0	10.5695054	10.8564106	10.7298671	10.1987758
3227	**ENSG0000010608**	PLEKHA8	protein_coding	chr7	30027404	30130483	+	Human GRCh	OTTHUMG000000	PLEKHA8	**pleckstrin homology domain containing A8**	Extracellular	transporter		-1.187839789	0	0.47881031	0	1	0	11.5286253	11.8515763	11.7412144	11.2151263
3228	**ENSG0000010608**	STX1A	protein_coding	chr7	73699206	73719672	-	Human GRCh	OTTHUMG000001	STX1A	**syntaxin 1A**	Cytoplasm	transporter		1.039600506	0	0.86756521	0	1	0	8.34341539	8.70569198	8.68124971	8.46858177
3229	**ENSG0000010610**	NOD1	protein_coding	chr7	30424527	30478784	-	Human GRCh	OTTHUMG000000	NOD1	**nucleotide binding oligomerization domain containing 1**	Cytoplasm	other		1.250558194	0	0.44267444	0	1	0	7.44006475	7.87570251	8.06981881	7.68953274
3230	**ENSG0000010610**	GARS1	protein_coding	chr7	30580533	30634033	+	Human GRCh	OTTHUMG000001	GARS1	**glycyl-tRNA synthetase 1**	Cytoplasm	enzyme		-1.200012073	0	0.34508274	0	1	0	12.1877844	12.1266534	12.1007814	11.7946041
3231	**ENSG0000010611**	CRHR2	protein_coding	chr7	30651942	30700129	-	Human GRCh	OTTHUMG000000	CRHR2	**corticotropin releasing hormone receptor 2**	Plasma Mem	G-protein cou		-5.802052482	-1	0.27210526	0	NA	0	1.02257165	1.64905737	1.04970797	1.02448804
3233	**ENSG0000010612**	MINDY4	protein_coding	chr7	30771417	30892387	+	Human GRCh	OTTHUMG000001	MINDY4	**MINDY lysine 48 deubiquitinase 4**	Other	other		1.092243332	0	0.72872725	0	1	0	8.25268338	8.38326495	8.64355871	8.12601623
3235	**ENSG0000010614**	CASP2	protein_coding	chr7	143288215	143307696	+	Human GRCh	OTTHUMG000000	CASP2	**caspase 2**	Cytoplasm	peptidase		-1.204786431	0	0.32857284	0	1	0	11.6607179	11.9760074	11.7515388	11.4702652
3236	**ENSG0000010615**	CHCHD2	protein_coding	chr7	56101573	56106476	-	Human GRCh	OTTHUMG000001	CHCHD2	**coiled-coil-helix-coiled-coil-helix domain containing 2**	Cytoplasm	other		-1.050400519	0	0.81329541	0	1	0	12.3144994	12.6158824	12.5424655	12.2830892
3238	**ENSG0000010621**	HSPB1	protein_coding	chr7	76302673	76304295	+	Human GRCh	OTTHUMG000000	HSPB1	**heat shock protein family B (small) member 1**	Cytoplasm	other		1.303386509	0	0.32212367	0	1	0	11.8460703	11.6194222	11.8405121	12.1923185
3239	**ENSG0000010623**	NPTX2	protein_coding	chr7	98617285	98629869	+	Human GRCh	OTTHUMG000001	NPTX2	**neuronal pentraxin 2**	Extracellular	other		1.397147424	0	0.66620527	0	1	0	9.51912736	10.0468946	9.97645424	10.4030337
3240	**ENSG0000010624**	PDAP1	protein_coding	chr7	99392048	99408597	-	Human GRCh	OTTHUMG000001	PDAP1	**PDGFA associated protein 1**	Cytoplasm	other		1.198609871	0	0.3485259	0	1	0	10.8954909	11.2125213	11.3842252	11.1369596
3241	**ENSG0000010624**	BUD31	protein_coding	chr7	99408641	99419616	+	Human GRCh	OTTHUMG000001	BUD31	**BUD31 homolog**	Nucleus	transcription		-1.064758052	0	0.77731985	0	1	0	9.65334817	9.85772038	9.80552107	9.56219778
3242	**ENSG0000010624**	PTCD1	protein_coding	chr7	99416739	99466163	-	Human GRCh	OTTHUMG000001	PTCD1	**pentatricopeptide repeat domain 1**	Cytoplasm	other		-1.018833993	0	0.92717015	0	1	0	10.0748076	10.4233562	10.4583921	9.97433643
3243	**ENSG0000010625**	CYP3A5	protein_coding	chr7	99648194	99679996	-	Human GRCh	OTTHUMG000001	CYP3A5	**cytochrome P450 family 3 subfamily A member 5**	Cytoplasm	enzyme		5.178548732	1	0.35146715	0	NA	0	1.22769175	1.27595157	2.13934082	1.40043115
3244	**ENSG0000010626**	ZKSCAN1	protein_coding	chr7	100015572	100041689	+	Human GRCh	OTTHUMG000001	ZKSCAN1	**zinc finger with KRAB and SCAN domains 1**	Nucleus	transcription		-1.075631565	0	0.71142824	0	1	0	12.2377213	12.4530237	12.3228942	12.2140596
3245	**ENSG0000010626**	EIF3B	protein_coding	chr7	2354086	2380745	+	Human GRCh	OTTHUMG000000	EIF3B	**eukaryotic translation initiation factor 3 subunit B**	Cytoplasm	translation re		-1.134591159	0	0.47316167	0	1	0	12.8156848	13.1689969	12.9289916	12.7944958
3246	**ENSG0000010626**	SNX8	protein_coding	chr7	2251770	2354318	-	Human GRCh	OTTHUMG000001	SNX8	**sorting nexin 8**	Cytoplasm	transporter		1.075157586	0	0.74528783	0	1	0	9.65864329	9.92514428	9.96996533	9.77701366
3247	**ENSG0000010626**	NUDT1	protein_coding	chr7	2242226	2251146	+	Human GRCh	OTTHUMG000000	NUDT1	**nudix hydrolase 1**	Extracellular	phosphatase	karonudib	1.445857058	0	0.17777281	0	1	0	7.05476069	7.3837667	7.70890617	7.49899033
3248	**ENSG0000010627**	PTPRZ1	protein_coding	chr7	121873089	122062036	+	Human GRCh	OTTHUMG000001	PTPRZ1	**protein tyrosine phosphatase receptor type Z1**	Plasma Mem	phosphatase		-1.079547059	0	0.92993903	0	1	0	3.45698118	3.21607198	3.57446265	2.95483769
3249	**ENSG0000010629**	TAF6	protein_coding	chr7	100106876	100119841	-	Human GRCh	OTTHUMG000001	TAF6	**TATA-box binding protein associated factor 6**	Nucleus	transcription		-1.147095793	0	0.42080553	0	1	0	10.9927835	11.2980739	11.0547052	10.9533478
3250	**ENSG0000010629**	WASL	protein_coding	chr7	123681943	123749003	-	Human GRCh	OTTHUMG000001	WASL	**WASP like actin nucleation promoting factor**	Cytoplasm	other		1.049769207	0	0.80593672	0	1	0	11.0775295	11.3989437	11.4753887	11.1026495
3253	**ENSG0000010630**	AIMP2	protein_coding	chr7	6009255	6023834	+	Human GRCh	OTTHUMG000001	AIMP2	**aminoacyl tRNA synthetase complex interacting multifunction**	Plasma Mem	other		-1.10144508	0	0.66216085	0	1	0	10.0797509	10.4454249	10.2759347	10.050888
3254	**ENSG0000010632**	TFR2	protein_coding	chr7	100620416	100642779	-	Human GRCh	OTTHUMG000001	TFR2	**transferrin receptor 2**	Plasma Mem	transporter		1.092896448	0	0.7467599	0	1	0	7.76561166	7.94525755	8.12238326	7.75747218
3255	**ENSG0000010632**	FSCN3	protein_coding	chr7	127591409	127602144	+	Human GRCh	OTTHUMG000000	FSCN3	**fascin actin-bundling protein 3**	Cytoplasm	other		-2.200807459	-1	0.25153087	0	1	0	4.38633592	4.413865	3.84328723	3.72724403
3256	**ENSG0000010633**	MOSPD3	protein_coding	chr7	100612102	100615384	+	Human GRCh	OTTHUMG000001	MOSPD3	**motile sperm domain containing 3**	Other	other		1.083808093	0	0.80684713	0	1	0	8.42184365	8.55799164	8.63729621	8.52084979
3258	**ENSG0000010633**	PCOLCE	protein_coding	chr7	100602363	100608175	+	Human GRCh	OTTHUMG000001	PCOLCE	**procollagen C-endopeptidase enhancer**	Extracellular	other		1.401206941	0	0.41273718	0	1	0	6.11246094	6.38610855	6.74856095	6.43021471
3259	**ENSG0000010633**	FBXO24	protein_coding	chr7	100583982	100601117	+	Human GRCh	OTTHUMG000001	FBXO24	**F-box protein 24**	Other	enzyme		1.225378835	0	0.69184109	0	1	0	4.70227563	5.60272855	5.48806822	5.37981057
3261	**ENSG0000010634**	RBM28	protein_coding	chr7	128297685	128343908	-	Human GRCh	OTTHUMG000001	RBM28	**RNA binding motif protein 28**	Nucleus	other		-1.054216647	0	0.78851624	0	1	0	11.2956411	11.6629873	11.5035252	11.3612438
3262	**ENSG0000010634**	USP42	protein_coding	chr7	6104949	6161564	+	Human GRCh	OTTHUMG000001	USP42	**ubiquitin specific peptidase 42**	Nucleus	peptidase		-1.141514174	0	0.54436339	0	1	0	10.396543	10.6201547	10.5124322	10.2054916
3263	**ENSG0000010634**	IMPDH1	protein_coding	chr7	128392277	128409986	-	Human GRCh	OTTHUMG000001	IMPDH1	**inosine monophosphate dehydrogenase 1**	Cytoplasm	enzyme	pegintron/	-1.134129744	0	0.5440679	0	1	0	10.2181909	10.4838557	10.3536886	10.0621268
3264	**ENSG0000010635**	AGFG2	protein_coding	chr7	100539203	100568220	+	Human GRCh	OTTHUMG000001	AGFG2	**ArfGAP with FG repeats 2**	Other	other		-1.073073691	0	0.74241486	0	1	0	9.36709694	9.6608759	9.5868381	9.28057584
3265	**ENSG0000010635**	LSM5	protein_coding	chr7	32485338	32495283	-	Human GRCh	OTTHUMG000000	LSM5	**LSM5 homolog, U6 small nuclear RNA and mRNA degradation**	Cytoplasm	other		1.063636944	0	0.79458772	0	1	0	9.50275119	9.74190499	9.90575216	9.43891515
3267	**ENSG0000010636**	AP1S1	protein_coding	chr7	101154456	101161596	+	Human GRCh	OTTHUMG000001	AP1S1	**adaptor related protein complex 1 subunit sigma 1**	Cytoplasm	transporter		1.073948561	0	0.74201323	0	1	0	9.63143643	9.88920379	9.86657308	9.8249712
3269	**ENSG0000010639**	C1GALT1	protein_coding	chr7	7156934	7248616	+	Human GRCh	OTTHUMG000001	C1GALT1	**core 1 synthase, glycoprotein-N-acetylgalactosamine 3-beta-ga**	Plasma Mem	enzyme		1.270867134	0	0.34766936	0	1	0	8.74473988	8.89473561	9.17056288	8.97261336
3270	**ENSG0000010639**	PLOD3	protein_coding	chr7	101205977	101218420	-	Human GRCh	OTTHUMG000001	PLOD3	**procollagen-lysine,2-oxoglutarate 5-dioxygenase 3**	Cytoplasm	enzyme		-1.102334755	0	0.63787366	0	1	0	11.0772717	11.3852879	11.286122	10.950945
3271	**ENSG0000010639**	RPA3	protein_coding	chr7	7636518	7718607	-	Human GRCh	OTTHUMG000000	RPA3	**replication protein A3**	Nucleus	other		1.06387628	0	0.80386063	0	1	0	9.0172411	9.18812397	9.374694	8.93008422
3272	**ENSG0000010640**	ZNHIT1	protein_coding	chr7	101218165	101224190	+	Human GRCh	OTTHUMG000001	ZNHIT1	**zinc finger HIT-type containing 1**	Nucleus	other		-1.146720436	0	0.50107091	0	1	0	10.3318195	10.6290583	10.4876812	10.1611152
3273	**ENSG0000010640**	CLDN15	protein_coding	chr7	101232092	101238820	-	Human GRCh	OTTHUMG000001	CLDN15	**claudin 15**	Plasma Mem	other		-1.062575819	0	0.80729134	0	1	0	8.77673161	8.88358068	8.89043311	8.62592342
3275	**ENSG0000010641**	GLCCI1	protein_coding	chr7	7968796	8094272	+	Human GRCh	OTTHUMG000001	GLCCI1	**glucocorticoid induced 1**	Cytoplasm	other		1.00430747	0	0.9875333	0	1	0	8.77994573	9.26424842	9.15052548	8.94332744
3277	**ENSG0000010644**	PHF14	protein_coding	chr7	10973336	11169623	+	Human GRCh	OTTHUMG000001	PHF14	**PHD finger protein 14**	Nucleus	other		1.084135656	0	0.70541708	0	1	0	10.937781	11.2324613	11.35171	10.9795949
3278	**ENSG0000010645**	NRF1	protein_coding	chr7	129611720	129757082	+	Human GRCh	OTTHUMG000001	NRF1	**nuclear respiratory factor 1**	Nucleus	transcription		-1.074942394	0	0.74318313	0	1	0	9.64312654	9.93535041	9.9226996	9.47236221
3279	**ENSG0000010646**	TMEM106	protein_coding	chr7	12211270	12243367	+	Human GRCh	OTTHUMG000001	TMEM106B	**transmembrane protein 106B**	Cytoplasm	other		1.353839482	0	0.23987279	0	1	0	10.2430406	10.516404	10.9627247	10.4019838
3280	**ENSG0000010646**	EZH2	protein_coding	chr7	148807257	148884321	-	Human GRCh	OTTHUMG000001	EZH2	**enhancer of zeste 2 polycomb repressive complex 2 subunit**	Nucleus	transcription	SHR2554, (	-1.064613791	0	0.76389157	0	1	0	11.9990717	12.3636028	12.3099276	11.9101208
3281	**ENSG0000010647**	CEP41	protein_coding	chr7	130393771	130442433	-	Human GRCh	OTTHUMG000001	CEP41	**centrosomal protein 41**	Cytoplasm	other		-1.090321497	0	0.73867942	0	1	0	7.92574695	7.94278772	7.99951242	7.66372928
3282	**ENSG0000010647**	ZNF862	protein_coding	chr7	149838375	149867479	+	Human GRCh	OTTHUMG000001	ZNF862	**zinc finger protein 862**	Other	other		-1.130088528	0	0.60319461	0	1	0	10.0320213	10.2129127	10.1730214	9.77181945
3284	**ENSG0000010648**	MEST	protein_coding	chr7	130486171	130506465	+	Human GRCh	OTTHUMG000001	MEST	**mesoderm specific transcript**	Cytoplasm	peptidase		-1.161342954	0	0.45678825	0	1	0	11.3178935	11.6064224	11.5029508	11.0672752
3286	**ENSG0000010652**	ANKMY2	protein_coding	chr7	16599779	16645817	-	Human GRCh	OTTHUMG000000	ANKMY2	**ankyrin repeat and MYND domain containing 2**	Plasma Mem	other		-1.01299915	0	0.96347493	0	1	0	7.68230856	8.03031648	7.97243872	7.72495222
3287	**ENSG0000010652**	ACTR3C	protein_coding	chr7	150243916	150323725	-	Human GRCh	OTTHUMG000001	ACTR3C	**actin related protein 3C**	Cytoplasm	other		-1.124477639	0	0.66698202	0	1	0	7.98428335	8.5502522	8.31054652	8.01630203
3288	**ENSG0000010653**	POU6F2	protein_coding	chr7	38977909	39493095	+	Human GRCh	OTTHUMG000001	POU6F2	**POU class 6 homeobox 2**	Nucleus	transcription		-1.679408553	-1	0.15354511	0	1	0	6.24651537	6.3402087	5.79206534	5.815121
3289	**ENSG0000010653**	TSPAN13	protein_coding	chr7	16753755	16784536	+	Human GRCh	OTTHUMG000000	TSPAN13	**tetraspanin 13**	Plasma Mem	other		1.532924761	1	0.04890137	1	1	0	7.92978924	8.13633592	8.2419018	8.66716957
3290	**ENSG0000010653**	RARRES2	protein_coding	chr7	150338317	150341662	-	Human GRCh	OTTHUMG000001	RARRES2	**retinoic acid receptor responder 2**	Plasma Mem	transmembra		1.678401897	1	0.63238169	0	NA	0	2.12892677	2.23888513	2.2210487	2.68690337
3291	**ENSG0000010654**	AGR2	protein_coding	chr7	16791811	16833433	-	Human GRCh	OTTHUMG000000	AGR2	**anterior gradient 2, protein disulphide isomerase family meml**	Extracellular	other		3.073840761	1	0.27160012	0	1	0	5.66001286	4.33830317	4.54934266	7.05434051
3292	**ENSG0000010654**	AHR	protein_coding	chr7	16916359	17346152	+	Human GRCh	OTTHUMG000001	AHR	**aryl hydrocarbon receptor**	Nucleus	ligand-depen	tapinarof,	1.278975976	0	0.33743544	0	1	0	9.37868867	9.46092484	9.55472167	9.80969168
3293	**ENSG0000010655**	CHCHD3	protein_coding	chr7	132784870	133082090	-	Human GRCh	OTTHUMG000001	CHCHD3	**coiled-coil-helix-coiled-coil-helix domain containing 3**	Cytoplasm	transcription		-1.09010226	0	0.66702239	0	1	0	10.3732335	10.6474113	10.5868698	10.2310504
3295	**ENSG0000010656**	TMEM176	protein_coding	chr7	150791285	150801360	-	Human GRCh	OTTHUMG000001	TMEM176B	**transmembrane protein 176B**	Other	other		-1.005498527	0	0.99594012	0	1	0	1.75282368	1.0302308	1.01303311	1.76264012
3296	**ENSG0000010657**	GLI3	protein_coding	chr7	41960949	42264100	-	Human GRCh	OTTHUMG000000	GLI3	**GLI family zinc finger 3**	Nucleus	transcription		1.3580369	0	0.66508975	0	1	0	8.18116745	8.12202665	7.91987421	8.91201697
3297	**ENSG0000010658**	PSMA2	protein_coding	chr7	42916828	42932185	-	Human GRCh	OTTHUMG000000	PSMA2	**proteasome 20S subunit alpha 2**	Cytoplasm	peptidase		-1.016187947	0	0.94269259	0	1	0	10.7755778	11.1150842	11.0377318	10.8328082
3298	**ENSG0000010659**	MRPL32	protein_coding	chr7	42932376	42948958	+	Human GRCh	OTTHUMG000001	MRPL32	**mitochondrial ribosomal protein L32**	Cytoplasm	translation re		-1.009718824	0	0.9653649	0	1	0	8.68580838	8.98061848	9.00973821	8.62216293
3299	**ENSG0000010660**	COA1	protein_coding	chr7	43608456	43729717	-	Human GRCh	OTTHUMG000001	COA1	**cytochrome c oxidase assembly factor 1**	Cytoplasm	other		1.017000871	0	0.94501863	0	1	0	11.093225	11.2839439	11.3684441	11.0284832

3300	**ENSG0000010660**	BLVRA	protein_coding	chr7	43758680	43807342	+	Human GRCh	OTTHUMG000001	BLVRA	**biliverdin reductase A**	Cytoplasm	enzyme		1.25757201	0	0.59487654	0	1	0	4.85234446	4.97785586	5.53920162	4.50202795
3301	**ENSG0000010660**	URGCP	protein_coding	chr7	43875894	43926411	-	Human GRCh	OTTHUMG000001	URGCP	**upregulator of cell proliferation**	Cytoplasm	other		-1.072302135	0	0.75351072	0	1	0	10.1348402	10.3721694	10.3960009	9.92237637
3302	**ENSG0000010660**	TMEM248	protein_coding	chr7	66921225	66958551	+	Human GRCh	OTTHUMG000001	TMEM248	**transmembrane protein 248**	Other	other		-1.055690282	0	0.79780058	0	1	0	11.0474871	11.3453875	11.234055	11.0514217
3303	**ENSG0000010661**	RHEB	protein_coding	chr7	151466012	151520120	-	Human GRCh	OTTHUMG000001	RHEB	**Ras homolog, mTORC1 binding**	Cytoplasm	enzyme		-1.189444082	0	0.38987155	0	1	0	11.4451035	11.7924214	11.5471718	11.3157386
3304	**ENSG0000010661**	PRKAG2	protein_coding	chr7	151556124	151877214	-	Human GRCh	OTTHUMG000001	PRKAG2	**protein kinase AMP-activated non-catalytic subunit gamma 2**	Cytoplasm	kinase		-1.190847463	0	0.46072725	0	1	0	11.2421344	11.413	11.2241242	11.0230789
3305	**ENSG0000010662**	AEBP1	protein_coding	chr7	44104345	44114562	+	Human GRCh	OTTHUMG000000	AEBP1	**AE binding protein 1**	Nucleus	peptidase		-1.124815963	0	0.7337045	0	1	0	5.95492351	6.61014606	6.1300607	6.2786366
3306	**ENSG0000010662**	POLD2	protein_coding	chr7	44114631	44124358	-	Human GRCh	OTTHUMG000000	POLD2	**DNA polymerase delta 2, accessory subunit**	Nucleus	enzyme		-1.108460083	0	0.58952434	0	1	0	12.54661	12.8019714	12.6435873	12.4797232
3307	**ENSG0000010663**	MYL7	protein_coding	chr7	44138864	44141332	-	Human GRCh	OTTHUMG000000	MYL7	**myosin light chain 7**	Cytoplasm	enzyme		1	0	1	0	NA	0	0.05911718	0.072887	0.07061769	0.05992744
3308	**ENSG0000010663**	GCK	protein_coding	chr7	44143213	44198170	-	Human GRCh	OTTHUMG000000	GCK	**glucokinase**	Cytoplasm	kinase		12.86464809	1	0.00037699	1	0.38	0	3.07715639	2.24315391	4.06534526	5.07402436
3309	**ENSG0000010663**	BCL7B	protein_coding	chr7	73536356	73557690	-	Human GRCh	OTTHUMG000000	BCL7B	**BAF chromatin remodeling complex subunit BCL7B**	Nucleus	other		-1.066855872	0	0.77475647	0	1	0	9.89365976	10.1866409	10.080333	9.86472434
3310	**ENSG0000010663**	YKT6	protein_coding	chr7	44200959	44214294	+	Human GRCh	OTTHUMG000001	YKT6	**YKT6 v-SNARE homolog**	Cytoplasm	enzyme		-1.040115446	0	0.83965049	0	1	0	11.2450152	11.4774685	11.4474352	11.1855649
3311	**ENSG0000010663**	TBL2	protein_coding	chr7	73567537	73578791	-	Human GRCh	OTTHUMG000000	TBL2	**transducin beta like 2**	Plasma Mem	other		-1.156169312	0	0.46338263	0	1	0	10.3784834	10.6192163	10.4473738	10.2276585
3313	**ENSG0000010666**	CLIP2	protein_coding	chr7	74289407	74405935	+	Human GRCh	OTTHUMG000000	CLIP2	**CAP-Gly domain containing linker protein 2**	Cytoplasm	transcription		-1.231582101	0	0.81020973	0	1	0	5.53070909	5.21624809	5.00439781	5.34793843
3314	**ENSG0000010668**	EIF4H	protein_coding	chr7	74174231	74197122	+	Human GRCh	OTTHUMG000000	EIF4H	**eukaryotic translation initiation factor 4H**	Cytoplasm	translation re		-1.093315768	0	0.65207898	0	1	0	12.7535502	13.1385657	12.9989901	12.7065902
3315	**ENSG0000010668**	LIMK1	protein_coding	chr7	74082933	74122525	+	Human GRCh	OTTHUMG000000	LIMK1	**LIM domain kinase 1**	Cytoplasm	kinase	dabrafenit	-1.03911519	0	0.84760826	0	1	0	9.99631081	10.1424502	10.125104	9.92553236
3316	**ENSG0000010668**	SPATA6L	protein_coding	chr9	4553386	4666674	-	Human GRCh	OTTHUMG000000	SPATA6L	**spermatogenesis associated 6 like**	Other	other		1.284755132	0	0.41001267	0	1	0	5.30460849	5.5613817	5.33605525	5.881791
3317	**ENSG0000010668**	SLC1A1	protein_coding	chr9	4490468	4587469	+	Human GRCh	OTTHUMG000000	SLC1A1	**solute carrier family 1 member 1**	Plasma Mem	transporter	riluzole	1.588080671	1	0.17005398	0	1	0	7.71001488	7.71065512	7.98890347	8.43668466
3318	**ENSG0000010668**	LHX2	protein_coding	chr9	124001670	124033301	+	Human GRCh	OTTHUMG000000	LHX2	**LIM homeobox 2**	Nucleus	transcription		1.159754666	0	0.64265522	0	1	0	6.96544112	7.31497693	7.32569373	7.29482488
3319	**ENSG0000010669**	FKTN	protein_coding	chr9	105558122	105653820	+	Human GRCh	OTTHUMG000000	FKTN	**fukutin**	Cytoplasm	enzyme		1.007612855	0	0.97188075	0	1	0	10.3334056	10.3734214	10.5301259	10.1655449
3320	**ENSG0000010670**	FSD1L	protein_coding	chr9	105447796	105552433	+	Human GRCh	OTTHUMG000000	FSD1L	**fibronectin type III and SPRY domain containing 1 like**	Other	other		-1.002968874	0	0.99219498	0	1	0	7.98615483	7.95589651	8.27884532	7.54246831
3321	**ENSG0000010671**	CNTNAP3	protein_coding	chr9	39064710	39288315	-	Human GRCh	OTTHUMG000000	CNTNAP3	**contactin associated protein family member 3**	Extracellular	other		-1.261848691	0	0.50683999	0	1	0	6.72444295	6.58363554	6.56710846	6.20881821
3322	**ENSG0000010672**	SPIN1	protein_coding	chr9	88388430	88478694	+	Human GRCh	OTTHUMG000000	SPIN1	**spindlin 1**	Nucleus	other		-1.194106525	0	0.36328962	0	1	0	11.4323264	11.5893296	11.385921	11.2672146
3323	**ENSG0000010673**	NMRK1	protein_coding	chr9	75060573	75088217	-	Human GRCh	OTTHUMG000000	NMRK1	**nicotinamide riboside kinase 1**	Cytoplasm	kinase		-1.0312901	0	0.9186202	0	1	0	7.04417361	7.11190924	7.01256348	7.07798239
3324	**ENSG0000010677**	TMEM245	protein_coding	chr9	109015135	109119947	-	Human GRCh	OTTHUMG000000	TMEM245	**transmembrane protein 245**	Other	other		-1.101363378	0	0.65450334	0	1	0	11.5682723	11.8748275	11.7672561	11.4635119
3325	**ENSG0000010677**	PRUNE2	protein_coding	chr9	76611376	76906114	-	Human GRCh	OTTHUMG000000	PRUNE2	**prune homolog 2 with BCH domain**	Cytoplasm	other		-1.481679995	0	0.40634897	0	1	0	7.51108416	8.1104585	7.46078634	7.50706746
3326	**ENSG0000010678**	MEGF9	protein_coding	chr9	120600811	120714470	-	Human GRCh	OTTHUMG000000	MEGF9	**multiple EGF like domains 9**	Extracellular	other		-1.041569555	0	0.84673773	0	1	0	10.6453258	10.8551004	10.7975594	10.613738
3327	**ENSG0000010678**	TRIM14	protein_coding	chr9	98069275	98119222	-	Human GRCh	OTTHUMG000000	TRIM14	**tripartite motif containing 14**	Cytoplasm	enzyme		1.271366568	0	0.42005332	0	1	0	7.93217923	8.48412208	8.34164658	8.58557752
3328	**ENSG0000010678**	CORO2A	protein_coding	chr9	98120975	98192637	-	Human GRCh	OTTHUMG000000	CORO2A	**coronin 2A**	Cytoplasm	other		1.248245231	0	0.54184608	0	1	0	5.77446432	5.57839883	5.95093484	5.75462439
3329	**ENSG0000010679**	TGFBR1	protein_coding	chr9	99104038	99154192	+	Human GRCh	OTTHUMG000000	TGFBR1	**transforming growth factor beta receptor 1**	Plasma Mem	kinase	YL-13027,	-1.104644587	0	0.6414744	0	1	0	10.4274219	10.7190953	10.7086626	10.1931006
3330	**ENSG0000010680**	SEC61B	protein_coding	chr9	99222064	99230615	+	Human GRCh	OTTHUMG000000	SEC61B	**SEC61 translocon subunit beta**	Cytoplasm	transporter		-1.042363217	0	0.86070933	0	1	0	10.2448239	10.5274039	10.4814239	10.1971511
3331	**ENSG0000010680**	C5	protein_coding	chr9	120932987	121075195	-	Human GRCh	OTTHUMG000000	C5	**complement C5**	Extracellular	cytokine	cemdisirar	-1.291376605	0	0.34347712	0	1	0	8.11386811	8.15647757	7.91672366	7.83562142
3335	**ENSG0000010682**	TLE4	protein_coding	chr9	79571773	79726882	+	Human GRCh	OTTHUMG000000	TLE4	**TLE family member 4, transcriptional corepressor**	Nucleus	transcription		1.080573904	0	0.76529925	0	1	0	10.2477677	10.5256271	10.7010074	10.2118914
3336	**ENSG0000010685**	LHX6	protein_coding	chr9	122202577	122229626	-	Human GRCh	OTTHUMG000000	LHX6	**LIM homeobox 6**	Nucleus	transcription		1.000910505	0	0.99898755	0	1	0	3.47933124	3.79421863	3.90908487	3.30342536
3337	**ENSG0000010685**	PTGR1	protein_coding	chr9	111549722	111599893	-	Human GRCh	OTTHUMG000000	PTGR1	**prostaglandin reductase 1**	Cytoplasm	enzyme		1.021331285	0	0.91652182	0	1	0	10.0607147	10.3156437	10.3636963	10.0528724
3338	**ENSG0000010686**	SUSD1	protein_coding	chr9	112040783	112175297	-	Human GRCh	OTTHUMG000000	SUSD1	**sushi domain containing 1**	Other	other		-1.023679311	0	0.93083882	0	1	0	6.14419266	6.02813363	6.24397318	5.86741417
3340	**ENSG0000010694**	AKNA	protein_coding	chr9	114334156	114394405	-	Human GRCh	OTTHUMG000000	AKNA	**AT-hook transcription factor**	Nucleus	transcription		-1.105040054	0	0.64501892	0	1	0	9.18491947	8.95120075	9.16795912	8.74088939
3342	**ENSG0000010697**	DNM1	protein_coding	chr9	128191655	128255248	+	Human GRCh	OTTHUMG000000	DNM1	**dynamin 1**	Cytoplasm	enzyme		1.17861404	0	0.45061364	0	1	0	9.821668	9.89802563	10.0809271	9.98132496
3343	**ENSG0000010699**	ENG	protein_coding	chr9	127815013	127854658	-	Human GRCh	OTTHUMG000000	ENG	**endoglin**	Plasma Mem	transmembra	carotuxima	-2.24825859	-1	0.54146575	0	NA	0	1.96291309	2.52985029	1.62011015	2.22190996
3344	**ENSG0000010699**	AK1	protein_coding	chr9	127866486	127877675	-	Human GRCh	OTTHUMG000000	AK1	**adenylate kinase 1**	Cytoplasm	kinase		-1.064531972	0	0.81539438	0	1	0	9.52371591	10.0027566	9.68110763	9.75621772
3345	**ENSG0000010699**	CDC37L1	protein_coding	chr9	4679559	4708399	+	Human GRCh	OTTHUMG000000	CDC37L1	**cell division cycle 37 like 1, HSP90 cochaperone**	Cytoplasm	other		1.187505374	0	0.40740083	0	1	0	7.79830864	7.83214019	8.11116868	7.87521705
3346	**ENSG0000010701**	RLN2	protein_coding	chr9	5299864	5304716	-	Human GRCh	OTTHUMG000000	RLN2	**relaxin 2**	Extracellular	other		1.196381522	0	0.80070135	0	1	0	3.02317732	3.47089635	3.29981373	3.46043432
3347	**ENSG0000010701**	RLN1	protein_coding	chr9	5334930	5339876	-	Human GRCh	OTTHUMG000000	RLN1	**relaxin 1**	Extracellular	other		-1.641570958	-1	0.53556285	0	NA	0	3.018339	3.42736885	2.92881914	2.90119791
3348	**ENSG0000010702**	PLGRKT	protein_coding	chr9	5357971	5437925	-	Human GRCh	OTTHUMG000000	PLGRKT	**plasminogen receptor with a C-terminal lysine**	Cytoplasm	other		-1.166713043	0	0.53196	0	1	0	7.87213706	8.21422721	8.0936302	7.6490775
3349	**ENSG0000010702**	TBC1D13	protein_coding	chr9	128787253	128810430	+	Human GRCh	OTTHUMG000000	TBC1D13	**TBC1 domain family member 13**	Cytoplasm	other		-1.167212137	0	0.48273491	0	1	0	9.89701031	10.1498062	9.9324239	9.78543414
3350	**ENSG0000010703**	RIC1	protein_coding	chr9	5629025	5776557	+	Human GRCh	OTTHUMG000000	RIC1	**RIC1 homolog, RAB6A GEF complex partner 1**	Cytoplasm	other		-1.208159366	0	0.32496701	0	1	0	10.4032687	10.718822	10.4449439	10.2762479
3351	**ENSG0000010707**	KDM4C	protein_coding	chr9	6720863	7175648	+	Human GRCh	OTTHUMG000000	KDM4C	**lysine demethylase 4C**	Nucleus	enzyme		-1.124959529	0	0.58737113	0	1	0	10.8031912	11.0842147	10.9789005	10.6417996
3352	**ENSG0000010709**	DOCK8	protein_coding	chr9	214854	465259	+	Human GRCh	OTTHUMG000000	DOCK8	**dedicator of cytokinesis 8**	Cytoplasm	other		-1.199312092	0	0.59957373	0	1	0	10.7017023	10.8062637	10.8557809	10.0991771
3353	**ENSG0000010710**	KANK1	protein_coding	chr9	470291	746105	+	Human GRCh	OTTHUMG000000	KANK1	**KN motif and ankyrin repeat domains 1**	Nucleus	transcription		-1.016347394	0	0.94200724	0	1	0	10.3133225	10.347919	10.3796411	10.2426859
3354	**ENSG0000010710**	ELAVL2	protein_coding	chr9	23690104	23826337	-	Human GRCh	OTTHUMG000000	ELAVL2	**ELAV like RNA binding protein 2**	Cytoplasm	other		-1.286770014	0	0.47347464	0	1	0	7.97177104	8.34070281	8.09349251	7.69779452
3355	**ENSG0000010713**	NCS1	protein_coding	chr9	130172404	130237303	+	Human GRCh	OTTHUMG000000	NCS1	**neuronal calcium sensor 1**	Plasma Mem	other		-1.096690207	0	0.59825991	0	1	0	10.171409	10.4859986	10.231769	10.2430992
3356	**ENSG0000010714**	TESK1	protein_coding	chr9	35605262	35610041	+	Human GRCh	OTTHUMG000000	TESK1	**testis associated actin remodelling kinase 1**	Nucleus	kinase		1.032664044	0	0.87660455	0	1	0	9.66398821	9.93486781	9.86437049	9.81995331
3357	**ENSG0000010714**	KCNT1	protein_coding	chr9	135702185	135795508	+	Human GRCh	OTTHUMG000000	KCNT1	**potassium sodium-activated channel subfamily T member 1**	Plasma Mem	ion channel		1.809404417	1	0.3678234	0	1	0	4.01796733	5.34156131	4.97949189	5.69711905
3358	**ENSG0000010715**	CA9	protein_coding	chr9	35673928	35681159	+	Human GRCh	OTTHUMG000000	CA9	**carbonic anhydrase 9**	Nucleus	enzyme	benzthiazi	1	0	1	0	NA	0	0.89281816	0.91671696	0.91277031	0.89422138
3359	**ENSG0000010716**	FUBP3	protein_coding	chr9	130578965	130638352	+	Human GRCh	OTTHUMG000000	FUBP3	**far upstream element binding protein 3**	Nucleus	transcription		1.05776458	0	0.77916916	0	1	0	11.0802088	11.4647463	11.4996377	11.1742911
3360	**ENSG0000010716**	TYRP1	protein_coding	chr9	12685439	12710285	+	Human GRCh	OTTHUMG000000	TYRP1	**tyrosinase related protein 1**	Cytoplasm	enzyme	RO729358	1	0	1	0	1	0	1.80520777	1.91998531	1.90166336	1.81221833
3361	**ENSG0000010717**	CREB3	protein_coding	chr9	35732598	35736999	+	Human GRCh	OTTHUMG000000	CREB3	**cAMP responsive element binding protein 3**	Nucleus	transcription		-1.086170909	0	0.69810565	0	1	0	9.56647066	9.93727776	9.79902987	9.5338265
3362	**ENSG0000010718**	RGP1	protein_coding	chr9	35749287	35758585	+	Human GRCh	OTTHUMG000000	RGP1	**RGP1 homolog, RAB6A GEF complex partner 1**	Cytoplasm	other		-1.12155797	0	0.55102766	0	1	0	12.1055037	12.3451363	12.150737	12.0535585
3363	**ENSG0000010718**	MPDZ	protein_coding	chr9	13105706	13279692	-	Human GRCh	OTTHUMG000000	MPDZ	**multiple PDZ domain crumbs cell polarity complex component**	Plasma Mem	other		-1.191066901	0	0.44246463	0	1	0	11.1660883	11.365846	11.2712386	10.8460772
3364	**ENSG0000010718**	LHX3	protein_coding	chr9	136196250	136205128	-	Human GRCh	OTTHUMG000000	LHX3	**LIM homeobox 3**	Nucleus	transcription		-1.002343868	0	0.99651296	0	1	0	3.1275432	4.0498799	3.51243072	3.7933062
3365	**ENSG0000010720**	RIGI	protein_coding	chr9	32455302	32526208	-	Human GRCh	OTTHUMG000000	RIGI	**RNA sensor RIG-I**	Cytoplasm	enzyme	MK-4621	-1.120688336	0	0.62725493	0	1	0	8.52964997	8.56182981	8.49964588	8.34948719
3366	**ENSG0000010722**	EDF1	protein_coding	chr9	136862119	136866308	-	Human GRCh	OTTHUMG000000	EDF1	**endothelial differentiation related factor 1**	Nucleus	transcription		1.091155816	0	0.70206779	0	1	0	11.2597549	11.4456797	11.5115252	11.3939964
3367	**ENSG0000010724**	PIP5K1B	protein_coding	chr9	68705240	69009176	+	Human GRCh	OTTHUMG000000	PIP5K1B	**phosphatidylinositol-4-phosphate 5-kinase type 1 beta**	Cytoplasm	kinase		4.310554735	1	0.01470747	1	1	0	5.01738926	5.27731405	5.51664983	6.61942588
3368	**ENSG0000010724**	GLIS3	protein_coding	chr9	3824127	4348392	-	Human GRCh	OTTHUMG000000	GLIS3	**GLIS family zinc finger 3**	Nucleus	transcription		-1.013322558	0	0.97118098	0	1	0	5.6241875	6.40847162	5.61432473	6.37486054
3369	**ENSG0000010726**	BAG1	protein_coding	chr9	33247820	33264720	-	Human GRCh	OTTHUMG000000	BAG1	**BAG cochaperone 1**	Cytoplasm	other		-1.065759758	0	0.73511058	0	1	0	11.2399607	11.6131958	11.4522453	11.278744
3370	**ENSG0000010726**	RAPGEF1	protein_coding	chr9	131576770	131740076	-	Human GRCh	OTTHUMG000000	RAPGEF1	**Rap guanine nucleotide exchange factor 1**	Cytoplasm	other		-1.129500354	0	0.50287512	0	1	0	11.6354957	11.8724733	11.7805527	11.4466976
3371	**ENSG0000010728**	NPDC1	protein_coding	chr9	137039463	137046179	-	Human GRCh	OTTHUMG000000	NPDC1	**neural proliferation, differentiation and control 1**	Extracellular	other		1.196032885	0	0.47753623	0	1	0	10.6528897	10.76903	11.0681713	10.7302517
3372	**ENSG0000010728**	APBA1	protein_coding	chr9	69427532	69672371	-	Human GRCh	OTTHUMG000000	APBA1	**amyloid beta precursor protein binding family A member 1**	Cytoplasm	transporter		3.950107785	1	0.09457835	0	1	0	3.18341891	3.70960983	3.99352707	3.88201821
3373	**ENSG0000010729**	SETX	protein_coding	chr9	132261356	132354986	-	Human GRCh	OTTHUMG000000	SETX	**senataxin**	Nucleus	enzyme		-1.066129941	0	0.76404513	0	1	0	12.4444264	12.7588187	12.6699661	12.3952066
3374	**ENSG0000010729**	SH3GL2	protein_coding	chr9	17579066	17797124	+	Human GRCh	OTTHUMG000000	SH3GL2	**SH3 domain containing GRB2 like 2, endophilin A1**	Plasma Mem	enzyme		1.007224496	0	0.98516236	0	1	0	8.75792567	8.63284803	8.61417774	8.78911948
3375	**ENSG0000010731**	PTGDS	protein_coding	chr9	136975092	136981742	+	Human GRCh	OTTHUMG000000	PTGDS	**prostaglandin D2 synthase**	Cytoplasm	enzyme		1.318137873	0	0.6337653	0	1	0	6.00155484	5.88877772	6.39867072	6.03915216
3376	**ENSG0000010733**	ABCA2	protein_coding	chr9	137007234	137028915	-	Human GRCh	OTTHUMG000000	ABCA2	**ATP binding cassette subfamily A member 2**	Plasma Mem	transporter		-1.021107878	0	0.91567458	0	1	0	12.1044896	12.2512212	12.3323443	11.9526774
3377	**ENSG0000010733**	SHB	protein_coding	chr9	37915898	38069227	-	Human GRCh	OTTHUMG000000	SHB	**SH2 domain containing adaptor protein B**	Cytoplasm	other		1.100235833	0	0.7180074	0	1	0	8.13529107	7.8847549	7.99632452	8.22024044
3378	**ENSG0000010734**	UBE2R2	protein_coding	chr9	33817160	33920399	+	Human GRCh	OTTHUMG000000	UBE2R2	**ubiquitin conjugating enzyme E2 R2**	Cytoplasm	enzyme		-1.034248648	0	0.86347521	0	1	0	11.6555241	11.9118835	11.9052406	11.5780671
3379	**ENSG0000010736**	ABHD17B	protein_coding	chr9	71862452	71911193	-	Human GRCh	OTTHUMG000000	ABHD17B	**abhydrolase domain containing 17B, depalmitoylase**	Plasma Mem	peptidase		1.197010716	0	0.40876673	0	1	0	8.89017858	9.13844226	9.35450966	9.06073687
3380	**ENSG0000010737**	EXOSC3	protein_coding	chr9	37759234	37832117	-	Human GRCh	OTTHUMG000000	EXOSC3	**exosome component 3**	Nucleus	enzyme		-1.041895031	0	0.84938934	0	1	0	9.52718747	9.80679551	9.73330486	9.51669762
3381	**ENSG0000010737**	ZFAND5	protein_coding	chr9	72351413	72365235	-	Human GRCh	OTTHUMG000000	ZFAND5	**zinc finger AN1-type containing 5**	Nucleus	other		-1.052114387	0	0.79370494	0	1	0	11.6263408	11.7539399	11.7539475	11.5055919
3382	**ENSG0000010740**	DVL1	protein_coding	chr1	1335276	1349418	-	Human GRCh	OTTHUMG000000	DVL1	**dishevelled segment polarity protein 1**	Cytoplasm	other		1.105879885	0	0.59423117	0	1	0	11.9828498	12.2804822	12.3542517	12.1472752
3383	**ENSG0000010743**	PDLIM1	protein_coding	chr10	95237572	95291012	-	Human GRCh	OTTHUMG000000	PDLIM1	**PDZ and LIM domain 1**	Cytoplasm	transcription		-1.14338283	0	0.66540879	0	1	0	8.09187512	7.9610606	7.16599926	8.32759637
3384	**ENSG0000010744**	CCNJ	protein_coding	chr10	96043394	96060870	+	Human GRCh	OTTHUMG000000	CCNJ	**cyclin J**	Nucleus	other		1.004777614	0	0.98262	0	1	0	9.3032846	9.51582682	9.63416408	9.15721723
3386	**ENSG0000010748**	GATA3	protein_coding	chr10	8045378	8075198	+	Human GRCh	OTTHUMG000000	GATA3	**GATA binding protein 3**	Nucleus	transcription		-1.154871444	0	0.84394045	0	1	0	8.12848384	8.29982972	7.78235181	8.26207617
3387	**ENSG0000010751**	ATRNL1	protein_coding	chr10	115093365	115948999	+	Human GRCh	OTTHUMG000000	ATRNL1	**attractin like 1**	Other	other		-1.029274823	0	0.93960996	0	1	0	7.17069767	7.44619874	7.22212472	7.34576978
3388	**ENSG0000010752**	HPS1	protein_coding	chr10	98410939	98446963	-	Human GRCh	OTTHUMG000000	HPS1	**HPS1 biogenesis of lysosomal organelles complex 3 subunit 1**	Cytoplasm	other		-1.049451946	0	0.83403786	0	1	0	9.90811828	10.2778083	10.1887274	9.89875849
3389	**ENSG0000010753**	PHYH	protein_coding	chr10	13277796	13302412	-	Human GRCh	OTTHUMG000000	PHYH	**phytanoyl-CoA 2-hydroxylase**	Cytoplasm	enzyme		1.114116273	0	0.67347863	0	1	0	8.31307314	8.76797176	8.85732407	8.46794835

3390	ENSG0000010755	RASSF4	protein_coding	chr10	44959407	44995891	+	Human GRCh	OTTHUMG000000	RASSF4	Ras association domain family member 4	Other	other		1.199689378	0	0.6696693	0	1	0	4.93202106	5.24666203	5.01499159	5.47121744
3391	ENSG0000010755	DNMBP	protein_coding	chr10	99875577	100009947	-	Human GRCh	OTTHUMG000000	DNMBP	dynamin binding protein	Cytoplasm	other		1.058015887	0	0.85189883	0	1	0	7.40783138	7.34830908	7.48145044	7.38153342
3392	ENSG0000010756	RAB11FIP2	protein_coding	chr10	118004916	118046941	-	Human GRCh	OTTHUMG000000	RAB11FIP2	RAB11 family interacting protein 2	Cytoplasm	other		1.180920517	0	0.49999852	0	1	0	9.08640399	9.28317259	9.53563082	9.17310132
3393	ENSG0000010756	CXCL12	protein_coding	chr10	44370165	44386493	-	Human GRCh	OTTHUMG000000	CXCL12	C-X-C motif chemokine ligand 12	Extracellular	cytokine	NOX-A12,	5.007656292	1	0.59823585	0	NA	0	0.31438953	0.34646563	0.34125291	0.94283215
3394	ENSG0000010756	ERLIN1	protein_coding	chr10	100150094	100186033	-	Human GRCh	OTTHUMG000000	ERLIN1	ER lipid raft associated 1	Plasma Mem	other		-1.131412577	0	0.54313461	0	1	0	9.73640007	10.0640954	9.93872197	9.58268346
3395	ENSG0000010758	EIF3A	protein_coding	chr10	119033670	119080817	-	Human GRCh	OTTHUMG000000	EIF3A	eukaryotic translation initiation factor 3 subunit A	Cytoplasm	translation re		-1.028561481	0	0.88459628	0	1	0	12.9122631	13.224775	13.1688927	12.9114116
3396	ENSG0000010759	PKD2L1	protein_coding	chr10	100288149	100330264	-	Human GRCh	OTTHUMG000000	PKD2L1	polycystin 2 like 1, transient receptor potential cation channel	Plasma Mem	ion channel		-1.673480643	-1	0.5141022	0	1	0	3.30548612	3.54895026	3.52259763	2.24898584
3397	ENSG0000010761	CUBN	protein_coding	chr10	16823966	17129811	-	Human GRCh	OTTHUMG000000	CUBN	cubilin	Plasma Mem	transmembra		-1.510586055	-1	0.34726923	0	1	0	6.84708584	7.27566531	6.98451925	6.14578243
3398	ENSG0000010761	TRDMT1	protein_coding	chr10	17137336	17202054	-	Human GRCh	OTTHUMG000000	TRDMT1	tRNA aspartic acid methyltransferase 1	Nucleus	enzyme		-1.140882029	0	0.63218323	0	1	0	9.11525634	9.39924623	9.29763372	8.92140569
3399	ENSG0000010762	DDX50	protein_coding	chr10	68901286	68946847	+	Human GRCh	OTTHUMG000000	DDX50	DExD-box helicase 50	Nucleus	enzyme		-1.023053964	0	0.90427304	0	1	0	10.048185	10.3464808	10.3109528	10.0366618
3400	ENSG0000010764	MAPK8	protein_coding	chr10	48306639	48439360	+	Human GRCh	OTTHUMG000000	MAPK8	mitogen-activated protein kinase 8	Cytoplasm	kinase	aplidine, S	-1.085741783	0	0.70152016	0	1	0	10.3880978	10.6482705	10.6110284	10.2338925
3401	ENSG0000010765	SEC23IP	protein_coding	chr10	119892707	119944657	+	Human GRCh	OTTHUMG000000	SEC23IP	SEC23 interacting protein	Cytoplasm	other		1.043710694	0	0.83059511	0	1	0	10.2491799	10.3976675	10.4582311	10.280735
3402	ENSG0000010766	ATE1	protein_coding	chr10	121709393	121928801	-	Human GRCh	OTTHUMG000000	ATE1	arginyltransferase 1	Nucleus	enzyme		-1.054389503	0	0.84121735	0	1	0	11.5627758	11.9327843	11.9244252	11.4283836
3403	ENSG0000010767	NSMCE4A	protein_coding	chr10	121957091	121975217	-	Human GRCh	OTTHUMG000000	NSMCE4A	NSE4 homolog A, SMC5-SMC6 complex component	Nucleus	other		-1.083899367	0	0.77184782	0	1	0	11.3756758	11.6686021	11.5806449	11.2767636
3404	ENSG0000010767	PLEKHA1	protein_coding	chr10	122374696	122432354	+	Human GRCh	OTTHUMG000000	PLEKHA1	pleckstrin homology domain containing A1	Cytoplasm	other		1.208206657	0	0.38816352	0	1	0	10.1726105	10.3687726	10.6745347	10.2421449
3405	ENSG0000010771	PALD1	protein_coding	chr10	70478767	70668754	+	Human GRCh	OTTHUMG000000	PALD1	phosphatase domain containing paladin 1	Cytoplasm	phosphatase		-1.057012915	0	0.82330623	0	1	0	6.94644637	6.66315127	6.82493677	6.69554702
3406	ENSG0000010773	UNC5B	protein_coding	chr10	71212570	71302864	+	Human GRCh	OTTHUMG000000	UNC5B	unc-5 netrin receptor B	Plasma Mem	transmembra		2.112178931	1	0.12956725	0	1	0	6.92962546	6.80397719	7.21865491	7.7930985
3407	ENSG0000010773	CDH23	protein_coding	chr10	71396920	71815947	+	Human GRCh	OTTHUMG000000	CDH23	cadherin related 23	Plasma Mem	transporter		-2.523667015	-1	0.29439377	0	1	0	6.16794908	6.50296242	5.64787525	5.80765058
3408	ENSG0000010773	VSIR	protein_coding	chr10	71747556	71773520	-	Human GRCh	OTTHUMG000000	VSIR	V-set immunoregulatory receptor	Plasma Mem	other	HMBD-002	1.320663109	0	0.66930566	0	1	0	3.54949134	3.10666311	4.05649025	2.52000582
3409	ENSG0000010774	SPOCK2	protein_coding	chr10	72059034	72089032	-	Human GRCh	OTTHUMG000000	SPOCK2	SPARC (osteonectin), cwcv and kazal like domains proteoglyca	Extracellular	other		-1.462752632	0	0.27226065	0	1	0	6.76161764	7.14900215	6.6202582	6.52010104
3410	ENSG0000010774	MICU1	protein_coding	chr10	72367340	72626131	-	Human GRCh	OTTHUMG000000	MICU1	mitochondrial calcium uptake 1	Cytoplasm	other		-1.129433152	0	0.55725245	0	1	0	10.4601059	10.8107077	10.6724139	10.3322612
3411	ENSG0000010775	PPP3CB	protein_coding	chr10	73436433	73496024	-	Human GRCh	OTTHUMG000000	PPP3CB	protein phosphatase 3 catalytic subunit beta	Plasma Mem	phosphatase	voclospori	1.099815682	0	0.68594283	0	1	0	9.72070486	9.81032563	10.0833239	9.60335551
3412	ENSG0000010777	CCSER2	protein_coding	chr10	84328586	84518521	+	Human GRCh	OTTHUMG000000	CCSER2	coiled-coil serine rich protein 2	Other	other		-1.041943281	0	0.84918749	0	1	0	10.1901454	10.6272131	10.5182896	10.2327253
3413	ENSG0000010777	BMPR1A	protein_coding	chr10	86756596	86932825	+	Human GRCh	OTTHUMG000000	BMPR1A	bone morphogenetic protein receptor type 1A	Plasma Mem	kinase	LDN-19318	-1.081727504	0	0.70852174	0	1	0	10.2015207	10.294978	10.3102855	9.99263453
3414	ENSG0000010778	MINPP1	protein_coding	chr10	87504875	87553461	+	Human GRCh	OTTHUMG000000	MINPP1	multiple inositol-polyphosphate phosphatase 1	Cytoplasm	phosphatase		1.06990373	0	0.78110652	0	1	0	8.86669062	9.05107555	9.1643442	8.89920654
3415	ENSG0000010779	ACTA2	protein_coding	chr10	88935074	88991339	-	Human GRCh	OTTHUMG000000	ACTA2	actin alpha 2, smooth muscle	Cytoplasm	other		-1.850514055	-1	0.62940856	0	NA	0	2.33984413	1.50380321	1.84399048	1.79733506
3416	ENSG0000010779	LIPA	protein_coding	chr10	89213569	89414557	-	Human GRCh	OTTHUMG000000	LIPA	lipase A, lysosomal acid type	Cytoplasm	enzyme		-1.218694896	0	0.43272107	0	1	0	8.48904131	8.96787237	8.56479401	8.49632329
3417	ENSG0000010780	TLX1	protein_coding	chr10	101131300	101137789	+	Human GRCh	OTTHUMG000000	TLX1	T cell leukemia homeobox 1	Nucleus	transcription		15.32852178	1	0.04019854	1	NA	0	1.9104258	1.93464999	1.93061271	2.74429787
3418	ENSG0000010781	TWNK	protein_coding	chr10	100987367	100994403	+	Human GRCh	OTTHUMG000000	TWNK	twinkle mtDNA helicase	Cytoplasm	enzyme		1.084170029	0	0.71591721	0	1	0	9.94932992	10.3088535	10.3860622	10.0616538
3419	ENSG0000010781	LZTS2	protein_coding	chr10	100996618	101007836	+	Human GRCh	OTTHUMG000000	LZTS2	leucine zipper tumor suppressor 2	Cytoplasm	other		1.108920882	0	0.65342144	0	1	0	10.5745344	10.9420213	10.9968064	10.7660594
3420	ENSG0000010781	SFXN3	protein_coding	chr10	101031234	101041241	+	Human GRCh	OTTHUMG000000	SFXN3	sideroflexin 3	Cytoplasm	transporter		-1.139572828	0	0.67309496	0	1	0	9.3574397	9.92539917	9.65574258	9.38721348
3421	ENSG0000010782	KAZALD1	protein_coding	chr10	101061989	101068131	+	Human GRCh	OTTHUMG000000	KAZALD1	Kazal type serine peptidase inhibitor domain 1	Extracellular	other		-1.059737167	0	0.90067868	0	1	0	5.77879251	6.47231073	6.17932845	6.06260306
3422	ENSG0000010782	FBXW4	protein_coding	chr10	101610664	101695295	-	Human GRCh	OTTHUMG000000	FBXW4	F-box and WD repeat domain containing 4	Cytoplasm	other		-1.121996828	0	0.59885755	0	1	0	9.41929775	9.74533149	9.56642285	9.35726148
3424	ENSG0000010783	NPM3	protein_coding	chr10	101781325	101783446	-	Human GRCh	OTTHUMG000000	NPM3	nucleophosmin/nucleoplasmin 3	Nucleus	other		1.001392285	0	0.99484476	0	1	0	9.13745631	9.52405738	9.41277079	9.27854758
3425	ENSG0000010785	TNKS2	protein_coding	chr10	91798426	91865475	+	Human GRCh	OTTHUMG000000	TNKS2	tankyrase 2	Nucleus	enzyme		-1.220599806	0	0.34792731	0	1	0	11.6684528	12.0103228	11.7632914	11.4743123
3427	ENSG0000010786	GBF1	protein_coding	chr10	102245371	102382899	+	Human GRCh	OTTHUMG000000	GBF1	golgi brefeldin A resistant guanine nucleotide exchange factor	Cytoplasm	other		-1.102211401	0	0.6090239	0	1	0	12.3029845	12.6191448	12.4292736	12.2899527
3428	ENSG0000010786	ARHGAP21	protein_coding	chr10	24583609	24723887	-	Human GRCh	OTTHUMG000000	ARHGAP21	Rho GTPase activating protein 21	Cytoplasm	other		-1.279057558	0	0.210438	0	1	0	11.4493427	11.7039594	11.3705453	11.2530889
3429	ENSG0000010786	CPEB3	protein_coding	chr10	92046692	92291078	-	Human GRCh	OTTHUMG000000	CPEB3	cytoplasmic polyadenylation element binding protein 3	Cytoplasm	translation re		-1.142451544	0	0.6191165	0	1	0	9.20297469	9.77718601	9.44784399	9.30073485
3430	ENSG0000010787	FBXL15	protein_coding	chr10	102419189	102423136	+	Human GRCh	OTTHUMG000000	FBXL15	F-box and leucine rich repeat protein 15	Cytoplasm	other		1.088046922	0	0.72315428	0	1	0	8.20034285	8.42756791	8.42345764	8.39742519
3431	ENSG0000010787	CUEDC2	protein_coding	chr10	102423245	102432584	-	Human GRCh	OTTHUMG000000	CUEDC2	CUE domain containing 2	Extracellular	other		1.036606393	0	0.86740356	0	1	0	9.78121006	10.119875	10.1021238	9.8944886
3432	ENSG0000010788	SUFU	protein_coding	chr10	102503972	102633535	+	Human GRCh	OTTHUMG000000	SUFU	SUFU negative regulator of hedgehog signaling	Nucleus	transcription		1.014222539	0	0.94765828	0	1	0	9.47428557	9.86208573	9.7754281	9.61855486
3433	ENSG0000010789	ANKRD26	protein_coding	chr10	26973793	27100494	-	Human GRCh	OTTHUMG000000	ANKRD26	ankyrin repeat domain containing 26	Nucleus	transcription		1.021092045	0	0.93149496	0	1	0	10.5845302	10.9342409	11.0239038	10.5111679
3434	ENSG0000010789	ACBD5	protein_coding	chr10	27168135	27243046	-	Human GRCh	OTTHUMG000000	ACBD5	acyl-CoA binding domain containing 5	Nucleus	other		1.018555447	0	0.93423834	0	1	0	9.44300195	9.67575803	9.73530873	9.41245291
3435	ENSG0000010790	LHPP	protein_coding	chr10	124461823	124617888	+	Human GRCh	OTTHUMG000000	LHPP	phospholysine phosphohistidine inorganic pyrophosphate pho	Cytoplasm	phosphatase		1.164635053	0	0.59399596	0	1	0	6.97330044	7.56911538	7.27570377	7.62628552
3436	ENSG0000010792	LARP4B	protein_coding	chr10	806933	931821	-	Human GRCh	OTTHUMG000000	LARP4B	La ribonucleoprotein 4B	Cytoplasm	other		-1.005108489	0	0.97997992	0	1	0	12.1239802	12.3645882	12.4072602	12.0562365
3437	ENSG0000010793	GTPBP4	protein_coding	chr10	988434	1019932	+	Human GRCh	OTTHUMG000000	GTPBP4	GTP binding protein 4	Nucleus	enzyme		1.014033276	0	0.94447114	0	1	0	10.9421521	11.2469957	11.2983627	10.9096388
3438	ENSG0000010793	EDRF1	protein_coding	chr10	125719515	125764143	+	Human GRCh	OTTHUMG000000	EDRF1	erythroid differentiation regulatory factor 1	Other	other		-1.090168896	0	0.71534563	0	1	0	10.6382287	10.9775762	10.9130066	10.5002929
3439	ENSG0000010794	BCCIP	protein_coding	chr10	125823546	125853695	+	Human GRCh	OTTHUMG000000	BCCIP	BRCA2 and CDKN1A interacting protein	Nucleus	other		-1.130803316	0	0.54748289	0	1	0	10.2120673	10.625435	10.4023128	10.1884412
3440	ENSG0000010795	MTPAP	protein_coding	chr10	30309801	30374448	-	Human GRCh	OTTHUMG000000	MTPAP	mitochondrial poly(A) polymerase	Cytoplasm	enzyme		-1.118878057	0	0.59965928	0	1	0	10.3270629	10.7617325	10.6209077	10.2270306
3441	ENSG0000010795	NEURL1	protein_coding	chr10	103493705	103592546	+	Human GRCh	OTTHUMG000000	NEURL1	neuralized E3 ubiquitin protein ligase 1	Cytoplasm	enzyme		1.235323736	0	0.62112757	0	1	0	5.45290251	5.56799287	5.00170369	6.09882934
3442	ENSG0000010795	SH3PXD2A	protein_coding	chr10	103594027	103855584	-	Human GRCh	OTTHUMG000000	SH3PXD2A	SH3 and PX domains 2A	Cytoplasm	other		1.530588455	1	0.44367789	0	1	0	6.96208949	7.15443338	7.3544018	7.44679606
3443	ENSG0000010795	PITRM1	protein_coding	chr10	3137728	3172841	-	Human GRCh	OTTHUMG000000	PITRM1	pitrilysin metallopeptidase 1	Cytoplasm	peptidase		-1.094491161	0	0.65419171	0	1	0	11.3976592	11.8316564	11.5972201	11.4639336
3444	ENSG0000010796	STN1	protein_coding	chr10	103856806	103918332	-	Human GRCh	OTTHUMG000000	STN1	STN1 subunit of CST complex	Nucleus	other		-1.086730199	0	0.71092611	0	1	0	8.95020008	9.18437338	9.12694833	8.8150462
3445	ENSG0000010796	MAP3K8	protein_coding	chr10	30434021	30461833	+	Human GRCh	OTTHUMG000000	MAP3K8	mitogen-activated protein kinase kinase kinase 8	Cytoplasm	kinase	Tpl2 kinase	1.41478619	0	0.53633905	0	1	0	2.45748655	4.17409965	3.99823914	3.69881983
3446	ENSG0000010798	DKK1	protein_coding	chr10	52314281	52318042	+	Human GRCh	OTTHUMG000000	DKK1	dickkopf WNT signaling pathway inhibitor 1	Extracellular	growth facto	BHQ880, D	1.168410786	0	0.93458119	0	1	0	3.30032405	1.97051416	2.44770173	3.31343287
3447	ENSG0000010800	EBF3	protein_coding	chr10	129835233	129973053	-	Human GRCh	OTTHUMG000000	EBF3	EBF transcription factor 3	Nucleus	transcription		1	0	1	0	1	0	1.42689217	1.50458453	1.49208074	1.43159271
3448	ENSG0000010801	GLRX3	protein_coding	chr10	130136391	130184521	+	Human GRCh	OTTHUMG000000	GLRX3	glutaredoxin 3	Cytoplasm	enzyme		-1.099937737	0	0.67401692	0	1	0	10.6107248	11.0766497	10.867024	10.6446236
3449	ENSG0000010801	SORCS1	protein_coding	chr10	106573663	107164706	-	Human GRCh	OTTHUMG000000	SORCS1	sortilin related VPS10 domain containing receptor 1	Plasma Mem	transporter		-3.145471187	-1	0.41416645	0	NA	0	2.36324276	2.83229353	1.74887549	1.89566349
3450	ENSG0000010802	TASOR2	protein_coding	chr10	5684731	5763779	+	Human GRCh	OTTHUMG000000	TASOR2	transcription activation suppressor family member 2	Nucleus	other		1.018192095	0	0.92494982	0	1	0	12.6482149	13.0241427	12.9975317	12.7300453
3451	ENSG0000010803	XPNPEP1	protein_coding	chr10	109864766	109923553	-	Human GRCh	OTTHUMG000000	XPNPEP1	X-prolyl aminopeptidase 1	Cytoplasm	peptidase		-1.150405089	0	0.50019302	0	1	0	10.2795406	10.651163	10.5030993	10.1098296
3452	ENSG0000010805	SMC3	protein_coding	chr10	110567684	110606048	+	Human GRCh	OTTHUMG000000	SMC3	structural maintenance of chromosomes 3	Nucleus	enzyme		-1.002573373	0	0.98981534	0	1	0	11.3794015	11.7818088	11.6499469	11.5371835
3453	ENSG0000010806	SHOC2	protein_coding	chr10	110919367	111017307	+	Human GRCh	OTTHUMG000000	SHOC2	SHOC2 leucine rich repeat scaffold protein	Cytoplasm	other		1.129989994	0	0.58725207	0	1	0	10.649911	10.9624086	11.1776034	10.6742863
3454	ENSG0000010806	TFAM	protein_coding	chr10	58385345	58399220	+	Human GRCh	OTTHUMG000000	TFAM	transcription factor A, mitochondrial	Cytoplasm	transcription		1.017564659	0	0.92933262	0	1	0	10.0354496	10.2396749	10.3062356	9.99482314
3455	ENSG0000010809	CCDC6	protein_coding	chr10	59788747	59906556	-	Human GRCh	OTTHUMG000000	CCDC6	coiled-coil domain containing 6	Cytoplasm	other		-1.042526608	0	0.83934097	0	1	0	11.5607349	11.9281662	11.7688877	11.6553397
3456	ENSG0000010809	CUL2	protein_coding	chr10	35008504	35127006	-	Human GRCh	OTTHUMG000000	CUL2	cullin 2	Nucleus	enzyme		-1.082816738	0	0.69772944	0	1	0	10.4324295	10.768488	10.6883723	10.3341315
3457	ENSG0000010810	CCNY	protein_coding	chr10	35247025	35572669	+	Human GRCh	OTTHUMG000000	CCNY	cyclin Y	Nucleus	other		-1.047444277	0	0.8258701	0	1	0	10.687779	11.0452908	10.9823982	10.6523747
3458	ENSG0000010810	UBE2S	protein_coding	chr19	55399745	55407788	-	Human GRCh	OTTHUMG000001	UBE2S	ubiquitin conjugating enzyme E2 S	Nucleus	enzyme		1.166299162	0	0.43998363	0	1	0	12.0152043	12.1559016	12.3289947	12.188114
3459	ENSG0000010810	RPL28	protein_coding	chr19	55385932	55403250	+	Human GRCh	OTTHUMG000001	RPL28	ribosomal protein L28	Cytoplasm	other		1.02776835	0	0.88543347	0	1	0	13.5535171	13.7900949	13.7670021	13.6478603
3460	ENSG0000010817	ZMIZ1	protein_coding	chr10	79068966	79316519	+	Human GRCh	OTTHUMG000000	ZMIZ1	zinc finger MIZ-type containing 1	Nucleus	transcription		-1.049443272	0	0.82871588	0	1	0	11.7940882	11.8992099	11.7741322	11.8191822
3461	ENSG0000010817	DNAJC12	protein_coding	chr10	67796669	67838188	-	Human GRCh	OTTHUMG000000	DNAJC12	DnaJ heat shock protein family (Hsp40) member C12	Cytoplasm	other		1.492623852	0	0.55464034	0	1	0	3.38541332	2.10923863	3.41559441	2.99501177
3462	ENSG0000010817	PPIF	protein_coding	chr10	79347469	79355334	+	Human GRCh	OTTHUMG000000	PPIF	peptidylprolyl isomerase F	Cytoplasm	enzyme		-1.050842564	0	0.83354996	0	1	0	11.8904501	12.3900974	12.2542126	11.9462899
3463	ENSG0000010818	PBLD	protein_coding	chr10	68282660	68333049	-	Human GRCh	OTTHUMG000000	PBLD	phenazine biosynthesis like protein domain containing	Cytoplasm	enzyme		-1.157880218	0	0.5787402	0	1	0	7.69219262	7.46442733	7.46496434	7.39938599
3464	ENSG0000010821	TSPAN14	protein_coding	chr10	80454265	80533124	+	Human GRCh	OTTHUMG000000	TSPAN14	tetraspanin 14	Plasma Mem	other		-1.125007164	0	0.54224949	0	1	0	10.8083276	11.0650064	10.9516557	10.6637072
3466	ENSG0000010823	TBC1D12	protein_coding	chr10	94402541	94536332	+	Human GRCh	OTTHUMG000000	TBC1D12	TBC1 domain family member 12	Extracellular	other		1.046216288	0	0.8472906	0	1	0	9.66720816	10.0162743	10.1142337	9.6513354
3467	ENSG0000010824	CYP2C18	protein_coding	chr10	94683729	94736190	+	Human GRCh	OTTHUMG000000	CYP2C18	cytochrome P450 family 2 subfamily C member 18	Cytoplasm	enzyme		-1.342173587	0	0.5660939	0	1	0	4.73741955	5.13376129	4.60842936	4.72623703
3470	ENSG0000010825	NUFIP2	protein_coding	chr17	29255839	29294148	-	Human GRCh	OTTHUMG000001	NUFIP2	nuclear FMR1 interacting protein 2	Cytoplasm	other		-1.080230667	0	0.70129202	0	1	0	12.7710863	13.1063115	13.0181657	12.687461
3471	ENSG0000010826	GIT1	protein_coding	chr17	29573475	29594054	-	Human GRCh	OTTHUMG000001	GIT1	GIT ArfGAP 1	Cytoplasm	other		-1.069424942	0	0.72824419	0	1	0	11.5489607	11.9061878	11.7575819	11.5656527
3472	ENSG0000010829	RPL19	protein_coding	chr17	39200283	39204840	+	Human GRCh	OTTHUMG000001	RPL19	ribosomal protein L19	Cytoplasm	other		1.026743552	0	0.89770106	0	1	0	13.2013754	13.5861465	13.5065374	13.3678871
3473	ENSG0000010830	FBXL20	protein_coding	chr17	39252663	39402523	-	Human GRCh	OTTHUMG000001	FBXL20	F-box and leucine rich repeat protein 20	Cytoplasm	other		-1.342185665	0	0.17438362	0	1	0	11.489081	11.8298936	11.4658942	11.2039344
3474	ENSG0000010830	RUNDC3A	protein_coding	chr17	44308413	44318670	+	Human GRCh	OTTHUMG000001	RUNDC3A	RUN domain containing 3A	Cytoplasm	other		1.253980275	0	0.55962984	0	1	0	5.14490942	5.37330665	5.30380126	5.59789874
3475	ENSG0000010831	UBTF	protein_coding	chr17	44205033	44221626	-	Human GRCh	OTTHUMG000001	UBTF	upstream binding transcription factor	Nucleus	transcription		-1.016933835	0	0.9319204	0	1	0	11.8972438	12.1443424	12.1183232	11.887575
3476	ENSG0000010834	CSF3	protein_coding	chr17	40015361	40017813	+	Human GRCh	OTTHUMG000001	CSF3	colony stimulating factor 3	Extracellular	cytokine		1.523057081	1	0.64375813	0	1	0	3.55679698	3.0163374	3.5915863	3.76432206
3477	ENSG0000010834	PSMD3	protein_coding	chr17	39980807	39997959	+	Human GRCh	OTTHUMG000001	PSMD3	proteasome 26S subunit, non-ATPase 3	Cytoplasm	other		-1.103664444	0	0.60464924	0	1	0	11.7758824	12.0913329	11.831721	11.8352328

3478	**ENSG0000010834**	CASC3	protein_coding	chr17	40140318	40172171	+	Human GRCh	OTTHUMG000001	CASC3	**CASC3 exon junction complex subunit**	Nucleus	other		-1.035798113	0	0.84521604	0	1	0	12.2900892	12.6201229	12.5371227	12.306215
3479	**ENSG0000010835**	RAPGEFL1	protein_coding	chr17	40177010	40195656	+	Human GRCh	OTTHUMG000001	RAPGEFL1	**Rap guanine nucleotide exchange factor like 1**	Other	other		-1.131534161	0	0.59547861	0	1	0	8.91535881	9.13118031	8.88465686	8.90176049
3480	**ENSG0000010837**	RGS9	protein_coding	chr17	65100812	65227703	+	Human GRCh	OTTHUMG000001	RGS9	**regulator of G protein signaling 9**	Cytoplasm	enzyme		-1.259135696	0	0.55266551	0	1	0	5.93127449	6.55838667	6.04555201	6.04979184
3481	**ENSG0000010837**	RNF43	protein_coding	chr17	58353676	58417595	-	Human GRCh	OTTHUMG000001	RNF43	**ring finger protein 43**	Plasma Mem	enzyme		3.015634609	1	0.25406996	0	1	0	3.80555609	3.20634635	3.62110711	4.36935728
3482	**ENSG0000010837**	WNT3	protein_coding	chr17	46762506	46833154	-	Human GRCh	OTTHUMG000001	WNT3	**Wnt family member 3**	Extracellular	other		8.217636274	1	1.30E-05	1	0.03	1	3.72734781	3.85699304	5.4438896	5.23321626
3483	**ENSG0000010838**	ASPA	protein_coding	chr17	3472374	3503405	+	Human GRCh	OTTHUMG000000	ASPA	**aspartoacylase**	Cytoplasm	enzyme		1	0	1	0	NA	0	0.47926892	0.49632703	0.49350697	0.48026917
3484	**ENSG0000010838**	RAD51C	protein_coding	chr17	58692573	58735611	+	Human GRCh	OTTHUMG000001	RAD51C	**RAD51 paralog C**	Nucleus	enzyme		1.088860755	0	0.69175391	0	1	0	8.93312296	9.11689127	9.21709862	9.01477462
3485	**ENSG0000010838**	SEPTIN4	protein_coding	chr17	58520250	58544368	-	Human GRCh	OTTHUMG000001	SEPTIN4	**septin 4**	Cytoplasm	enzyme		1.765078562	1	0.47034773	0	1	0	3.89531288	2.34038971	4.03513498	3.52979274
3486	**ENSG0000010838**	MTMR4	protein_coding	chr17	58489529	58517905	-	Human GRCh	OTTHUMG000001	MTMR4	**myotubularin related protein 4**	Cytoplasm	phosphatase		-1.101360505	0	0.63806805	0	1	0	11.6197827	12.0142624	11.8060208	11.6358552
3487	**ENSG0000010839**	TRIM37	protein_coding	chr17	58982638	59106921	-	Human GRCh	OTTHUMG000001	TRIM37	**tripartite motif containing 37**	Cytoplasm	enzyme		-1.052342167	0	0.79354888	0	1	0	10.593006	10.8065144	10.8390449	10.4219162
3488	**ENSG0000010840**	P2RX1	protein_coding	chr17	3896592	3916476	-	Human GRCh	OTTHUMG000001	P2RX1	**purinergic receptor P2X 1**	Plasma Mem	ion channel		13.6061771	1	0.05628467	0	1	0	1.5181144	1.56180468	2.31246187	2.03971699
3489	**ENSG0000010840**	DHX40	protein_coding	chr17	59565558	59608345	+	Human GRCh	OTTHUMG000001	DHX40	**DEAH-box helicase 40**	Other	enzyme		-1.168865643	0	0.52406399	0	1	0	10.5583564	10.8506253	10.6539223	10.4163887
3491	**ENSG0000010842**	TUBD1	protein_coding	chr17	59859479	59892945	-	Human GRCh	OTTHUMG000001	TUBD1	**tubulin delta 1**	Cytoplasm	other	docetaxel/	1.040659008	0	0.8678223	0	1	0	8.32224358	8.63569743	8.70762044	8.32770973
3492	**ENSG0000010842**	KPNB1	protein_coding	chr17	47649476	47685505	+	Human GRCh	OTTHUMG000000	KPNB1	**karyopherin subunit beta 1**	Nucleus	other		-1.148009785	0	0.47106759	0	1	0	13.6270969	13.9287565	13.7523687	13.5012114
3493	**ENSG0000010843**	GOSR2	protein_coding	chr17	46923075	46975524	+	Human GRCh	OTTHUMG000001	GOSR2	**golgi SNAP receptor complex member 2**	Cytoplasm	transporter		-1.148326497	0	0.53448085	0	1	0	11.015309	11.3425085	11.2244449	10.8157208
3494	**ENSG0000010843**	PNPO	protein_coding	chr17	47941506	47949308	+	Human GRCh	OTTHUMG000001	PNPO	**pyridoxamine 5'-phosphate oxidase**	Cytoplasm	enzyme		-1.112414815	0	0.60443187	0	1	0	10.8315171	11.0315327	10.9373839	10.6774265
3495	**ENSG0000010844**	RPS6KB1	protein_coding	chr17	59893046	59950574	+	Human GRCh	OTTHUMG000001	RPS6KB1	**ribosomal protein S6 kinase B1**	Cytoplasm	kinase	PF-470867	1.114477095	0	0.60587448	0	1	0	10.4031222	10.7965114	10.8678186	10.5898856
3496	**ENSG0000010846**	CDK5RAP3	protein_coding	chr17	47967810	47981781	+	Human GRCh	OTTHUMG000001	CDK5RAP3	**CDK5 regulatory subunit associated protein 3**	Nucleus	other		-1.066299285	0	0.73165706	0	1	0	11.5450824	11.7509373	11.7584952	11.372684
3497	**ENSG0000010846**	CBX1	protein_coding	chr17	48070052	48101478	-	Human GRCh	OTTHUMG000001	CBX1	**chromobox 1**	Nucleus	transcription		-1.036530077	0	0.86186956	0	1	0	11.1467529	11.4104319	11.3597346	11.1200639
3498	**ENSG0000010846**	RECQL5	protein_coding	chr17	75626845	75667189	-	Human GRCh	OTTHUMG000001	RECQL5	**RecQ like helicase 5**	Nucleus	enzyme		-1.087749607	0	0.70515276	0	1	0	10.2939016	10.3182489	10.3603317	10.0450026
3499	**ENSG0000010847**	PIGL	protein_coding	chr17	16217191	16351797	+	Human GRCh	OTTHUMG000000	PIGL	**phosphatidylinositol glycan anchor biosynthesis class L**	Cytoplasm	enzyme		-1.221804409	0	0.40491467	0	1	0	9.24501051	9.47565238	9.28778648	8.98776169
3500	**ENSG0000010847**	GALK1	protein_coding	chr17	75751594	75765236	-	Human GRCh	OTTHUMG000001	GALK1	**galactokinase 1**	Cytoplasm	kinase		1.042528595	0	0.88004557	0	1	0	7.97135369	8.02319269	8.13429382	7.94593531
3501	**ENSG0000010850**	INTS2	protein_coding	chr17	61865367	61928016	-	Human GRCh	OTTHUMG000001	INTS2	**integrator complex subunit 2**	Nucleus	other		-1.083230541	0	0.71377414	0	1	0	9.84036026	10.1472266	10.0271507	9.79194815
3502	**ENSG0000010850**	CAMTA2	protein_coding	chr17	4967992	4987675	-	Human GRCh	OTTHUMG000000	CAMTA2	**calmodulin binding transcription activator 2**	Nucleus	transcription		-1.101135251	0	0.64225084	0	1	0	11.7745955	12.0785487	11.8766045	11.7751988
3503	**ENSG0000010851**	MED13	protein_coding	chr17	61942605	62065278	-	Human GRCh	OTTHUMG000001	MED13	**mediator complex subunit 13**	Nucleus	transcription		-1.111407436	0	0.60074488	0	1	0	12.7359663	13.0219088	12.9104129	12.6097587
3504	**ENSG0000010851**	HOXB6	protein_coding	chr17	48595751	48604992	-	Human GRCh	OTTHUMG000001	HOXB6	**homeobox B6**	Nucleus	transcription		-1.969663381	-1	0.10932327	0	1	0	5.58312287	5.82917478	5.18494561	5.15431304
3505	**ENSG0000010851**	ENO3	protein_coding	chr17	4948092	4957131	+	Human GRCh	OTTHUMG000000	ENO3	**enolase 3**	Cytoplasm	enzyme		-1.112612357	0	0.62595706	0	1	0	8.57503155	8.80479259	8.74379	8.39812375
3506	**ENSG0000010851**	PFN1	protein_coding	chr17	4945652	4949061	-	Human GRCh	OTTHUMG000000	PFN1	**profilin 1**	Cytoplasm	other		-1.022696265	0	0.90996909	0	1	0	13.4616463	13.8299973	13.5820163	13.6847186
3507	**ENSG0000010852**	RNF167	protein_coding	chr17	4940008	4945222	+	Human GRCh	OTTHUMG000000	RNF167	**ring finger protein 167**	Cytoplasm	enzyme		-1.045130864	0	0.82301258	0	1	0	11.1775084	11.4844404	11.2939968	11.288172
3508	**ENSG0000010852**	SLC25A11	protein_coding	chr17	4937130	4940053	-	Human GRCh	OTTHUMG000000	SLC25A11	**solute carrier family 25 member 11**	Cytoplasm	transporter		1.092236953	0	0.68902995	0	1	0	10.6970921	11.010702	11.0046311	10.9192845
3509	**ENSG0000010855**	RASD1	protein_coding	chr17	17494437	17496395	-	Human GRCh	OTTHUMG000000	RASD1	**ras related dexamethasone induced 1**	Cytoplasm	enzyme		1.223116583	0	0.52177909	0	1	0	6.76272881	6.58385995	6.49030507	7.1590669
3510	**ENSG0000010855**	CHRNE	protein_coding	chr17	4897771	4934438	-	Human GRCh	OTTHUMG000000	CHRNE	**cholinergic receptor nicotinic epsilon subunit**	Plasma Mem	transmembra	succinylch	-1.00569784	0	0.98438554	0	1	0	9.59212971	9.81782576	9.63901754	9.76451106
3511	**ENSG0000010855**	RAI1	protein_coding	chr17	17681458	17811453	+	Human GRCh	OTTHUMG000000	RAI1	**retinoic acid induced 1**	Nucleus	transcription		1.106729418	0	0.58478687	0	1	0	11.6384562	11.7693459	11.9222516	11.7019001
3512	**ENSG0000010855**	NUP88	protein_coding	chr17	5359668	5420164	-	Human GRCh	OTTHUMG000000	NUP88	**nucleoporin 88**	Nucleus	other		-1.137473384	0	0.52201668	0	1	0	10.8114478	11.1713758	10.9798617	10.7346335
3513	**ENSG0000010856**	C1QBP	protein_coding	chr17	5432777	5448830	-	Human GRCh	OTTHUMG000001	C1QBP	**complement C1q binding protein**	Cytoplasm	transcription		-1.043429717	0	0.84842357	0	1	0	11.7246706	12.146639	11.9578118	11.8500464
3515	**ENSG0000010857**	BLMH	protein_coding	chr17	30248203	30292056	-	Human GRCh	OTTHUMG000001	BLMH	**bleomycin hydrolase**	Cytoplasm	peptidase		-1.09477625	0	0.64508027	0	1	0	9.80828697	10.0995597	10.0023107	9.70375617
3516	**ENSG0000010858**	CPD	protein_coding	chr17	30378927	30469989	+	Human GRCh	OTTHUMG000001	CPD	**carboxypeptidase D**	Extracellular	peptidase		-1.038059164	0	0.86046696	0	1	0	11.6832653	11.8740258	11.9064655	11.5513616
3517	**ENSG0000010858**	GOSR1	protein_coding	chr17	30477362	30527592	+	Human GRCh	OTTHUMG000001	GOSR1	**golgi SNAP receptor complex member 1**	Cytoplasm	transporter		-1.093571551	0	0.6338219	0	1	0	10.8225033	11.0896059	11.0000808	10.7130176
3518	**ENSG0000010858**	CCDC47	protein_coding	chr17	63745255	63776351	-	Human GRCh	OTTHUMG000001	CCDC47	**coiled-coil domain containing 47**	Extracellular	other		-1.003900795	0	0.98496874	0	1	0	11.4762764	11.9071982	11.8264838	11.5733846
3519	**ENSG0000010859**	MED31	protein_coding	chr17	6643311	6651634	-	Human GRCh	OTTHUMG000001	MED31	**mediator complex subunit 31**	Nucleus	transcription		1.094232618	0	0.76565587	0	1	0	7.88415612	7.76735413	8.02855964	7.81084559
3520	**ENSG0000010859**	DRG2	protein_coding	chr17	18087892	18107970	+	Human GRCh	OTTHUMG000000	DRG2	**developmentally regulated GTP binding protein 2**	Cytoplasm	enzyme		-1.112510932	0	0.63488967	0	1	0	10.1886198	10.5770583	10.4226486	10.1163023
3521	**ENSG0000010859**	FTSJ3	protein_coding	chr17	63819433	63830012	-	Human GRCh	OTTHUMG000001	FTSJ3	**FtsJ RNA 2'-O-methyltransferase 3**	Nucleus	enzyme		1.01489665	0	0.94420844	0	1	0	10.2593865	10.700224	10.6740062	10.3353734
3522	**ENSG0000010859**	AKAP10	protein_coding	chr17	19904302	19978343	-	Human GRCh	OTTHUMG000000	AKAP10	**A-kinase anchoring protein 10**	Cytoplasm	other		-1.1806425	0	0.36997264	0	1	0	10.1254863	10.3995256	10.2147034	9.94243052
3523	**ENSG0000010860**	ALDH3A1	protein_coding	chr17	19737984	19748943	-	Human GRCh	OTTHUMG000000	ALDH3A1	**aldehyde dehydrogenase 3 family member A1**	Cytoplasm	enzyme		10.77496313	1	0.10572307	0	NA	0	1.54729355	1.55914335	1.9026587	1.85528986
3524	**ENSG0000010860**	SMARCD2	protein_coding	chr17	63832081	63843065	-	Human GRCh	OTTHUMG000001	SMARCD2	**SWI/SNF related, matrix associated, actin dependent regulato**	Nucleus	transcription		-1.077857154	0	0.73923257	0	1	0	11.5221062	11.8987248	11.7741217	11.4880829
3526	**ENSG0000010863**	SYNGR2	protein_coding	chr17	78168581	78173527	+	Human GRCh	OTTHUMG000001	SYNGR2	**synaptogyrin 2**	Cytoplasm	other		-1.083234428	0	0.73864635	0	1	0	11.0081991	11.3981794	11.2136157	11.0380351
3527	**ENSG0000010864**	B9D1	protein_coding	chr17	19334308	19378193	-	Human GRCh	OTTHUMG000000	B9D1	**B9 domain containing 1**	Plasma Mem	other		-1.164253649	0	0.49054151	0	1	0	8.84082894	9.0473244	8.83258959	8.73128816
3528	**ENSG0000010865**	UTP6	protein_coding	chr17	31860904	31901708	-	Human GRCh	OTTHUMG000001	UTP6	**UTP6 small subunit processome component**	Nucleus	other		-1.051945	0	0.79932084	0	1	0	9.89036959	10.2692224	10.2260414	9.81647487
3529	**ENSG0000010865**	DDX5	protein_coding	chr17	64498254	64508199	-	Human GRCh	OTTHUMG000001	DDX5	**DEAD-box helicase 5**	Nucleus	enzyme		-1.075551533	0	0.6988885	0	1	0	13.55872	13.8655183	13.7807434	13.4824829
3530	**ENSG0000010866**	C17orf75	protein_coding	chr17	32324565	32350023	-	Human GRCh	OTTHUMG000001	C17orf75	**chromosome 17 open reading frame 75**	Extracellular	other		-1.060643555	0	0.78423601	0	1	0	9.38296255	9.41784916	9.42378999	9.24243874
3531	**ENSG0000010866**	CYTH1	protein_coding	chr17	78674048	78782297	-	Human GRCh	OTTHUMG000001	CYTH1	**cytohesin 1**	Cytoplasm	other		-1.282591493	0	0.22516204	0	1	0	10.4743219	10.7532947	10.4504007	10.2349669
3532	**ENSG0000010867**	PSMD11	protein_coding	chr17	32444379	32483319	+	Human GRCh	OTTHUMG000001	PSMD11	**proteasome 26S subunit, non-ATPase 11**	Cytoplasm	other		-1.151351914	0	0.42455267	0	1	0	11.8221065	12.1220349	11.8767467	11.771297
3533	**ENSG0000010867**	LGALS3BP	protein_coding	chr17	78971238	78979947	-	Human GRCh	OTTHUMG000001	LGALS3BP	**galectin 3 binding protein**	Plasma Mem	transmembra		1.21184874	0	0.667992	0	1	0	8.5440322	9.10415507	9.51893594	8.27745624
3534	**ENSG0000010868**	ASIC2	protein_coding	chr17	33013087	34174964	-	Human GRCh	OTTHUMG000001	ASIC2	**acid sensing ion channel subunit 2**	Plasma Mem	ion channel	amiloride,	5.494599502	1	0.21130362	0	1	0	1.12225576	1.21576145	1.99307556	1.12797136
3536	**ENSG0000010869**	CCL2	protein_coding	chr17	34255274	34257208	+	Human GRCh	OTTHUMG000001	CCL2	**C-C motif chemokine ligand 2**	Extracellular	cytokine	CNTO 888,	1	0	1	0	NA	0	-0.1641034	-0.1560714	-0.1574024	-0.1636338
3539	**ENSG0000010873**	PEX12	protein_coding	chr17	35574795	35578863	-	Human GRCh	OTTHUMG000001	PEX12	**peroxisomal biogenesis factor 12**	Cytoplasm	other		1.300057308	0	0.33098883	0	1	0	7.28307178	7.51366826	7.80373119	7.55813187
3541	**ENSG0000010877**	DHX58	protein_coding	chr17	42101404	42112714	-	Human GRCh	OTTHUMG000001	DHX58	**DExH-box helicase 58**	Cytoplasm	enzyme		1.159972997	0	0.62109148	0	1	0	7.11408685	7.54294335	7.71095979	7.25829091
3542	**ENSG0000010877**	KAT2A	protein_coding	chr17	42113111	42121367	-	Human GRCh	OTTHUMG000001	KAT2A	**lysine acetyltransferase 2A**	Cytoplasm	enzyme		1.037061094	0	0.83533867	0	1	0	11.3531711	11.5711012	11.6160974	11.3894908
3543	**ENSG0000010877**	RAB5C	protein_coding	chr17	42124978	42155044	-	Human GRCh	OTTHUMG000001	RAB5C	**RAB5C, member RAS oncogene family**	Cytoplasm	enzyme		-1.031486683	0	0.88346607	0	1	0	10.9069988	11.1503965	11.0154855	10.9847045
3544	**ENSG0000010878**	NAGLU	protein_coding	chr17	42536241	42544449	+	Human GRCh	OTTHUMG000001	NAGLU	**N-acetyl-alpha-glucosaminidase**	Cytoplasm	enzyme		-1.006371958	0	0.97786903	0	1	0	9.0281679	9.13205321	9.06480055	9.08340739
3545	**ENSG0000010878**	HSD17B1	protein_coding	chr17	42552922	42555214	+	Human GRCh	OTTHUMG000001	HSD17B1	**hydroxysteroid 17-beta dehydrogenase 1**	Cytoplasm	enzyme	OG-6219	1.418102895	0	0.49789868	0	1	0	4.35587798	5.0991294	4.86947795	5.28411178
3546	**ENSG0000010878**	MLX	protein_coding	chr17	42567072	42573239	+	Human GRCh	OTTHUMG000001	MLX	**MAX dimerization protein MLX**	Nucleus	transcription		-1.080544689	0	0.73482444	0	1	0	10.4081628	10.8495809	10.6290653	10.4909246
3547	**ENSG0000010879**	CNTNAP1	protein_coding	chr17	42682531	42699993	+	Human GRCh	OTTHUMG000001	CNTNAP1	**contactin associated protein 1**	Plasma Mem	other		1.019223654	0	0.93826355	0	1	0	8.63562536	8.83475912	8.89407842	8.6098217
3549	**ENSG0000010879**	EZH1	protein_coding	chr17	42700275	42745049	-	Human GRCh	OTTHUMG000001	EZH1	**enhancer of zeste 1 polycomb repressive complex 2 subunit**	Nucleus	enzyme	tazemetos	-1.08923148	0	0.70837156	0	1	0	10.1555043	10.355802	10.3147718	9.99520573
3550	**ENSG0000010881**	DLX4	protein_coding	chr17	49968588	49974959	+	Human GRCh	OTTHUMG000001	DLX4	**distal-less homeobox 4**	Nucleus	transcription		1.716697273	1	0.41044294	0	1	0	4.08809092	3.24098649	3.85035642	4.45824912
3551	**ENSG0000010881**	PPP1R9B	protein_coding	chr17	50133737	50150677	-	Human GRCh	OTTHUMG000001	PPP1R9B	**protein phosphatase 1 regulatory subunit 9B**	Cytoplasm	enzyme		1.193671637	0	0.28857644	0	1	0	10.8143477	10.9023714	11.0636463	11.0530401
3552	**ENSG0000010882**	COL1A1	protein_coding	chr17	50184101	50201632	-	Human GRCh	OTTHUMG000001	COL1A1	**collagen type I alpha 1 chain**	Extracellular	other	collagenas	-1.510205501	-1	0.29263426	0	1	0	9.28357026	9.45442336	8.75669182	9.01741591
3554	**ENSG0000010882**	MRPL27	protein_coding	chr17	50367857	50373207	-	Human GRCh	OTTHUMG000001	MRPL27	**mitochondrial ribosomal protein L27**	Cytoplasm	other		-1.144973286	0	0.53450292	0	1	0	9.37374067	9.83480261	9.53991649	9.41071164
3555	**ENSG0000010882**	VAT1	protein_coding	chr17	43014607	43025123	-	Human GRCh	OTTHUMG000001	VAT1	**vesicle amine transport 1**	Plasma Mem	transporter		-1.081861554	0	0.70613629	0	1	0	11.5517576	11.8685697	11.7636681	11.4830366
3556	**ENSG0000010882**	LRRC59	protein_coding	chr17	50375059	50397523	-	Human GRCh	OTTHUMG000001	LRRC59	**leucine rich repeat containing 59**	Cytoplasm	other		-1.119183702	0	0.59008086	0	1	0	12.2870435	12.7412193	12.5178441	12.2857568
3557	**ENSG0000010883**	RND2	protein_coding	chr17	43025231	43032041	+	Human GRCh	OTTHUMG000001	RND2	**Rho family GTPase 2**	Cytoplasm	enzyme		1.278056124	0	0.47396989	0	1	0	7.95602927	8.21367049	8.42137742	8.31205749
3559	**ENSG0000010884**	HDAC5	protein_coding	chr17	44076746	44123702	-	Human GRCh	OTTHUMG000001	HDAC5	**histone deacetylase 5**	Nucleus	transcription	tributyrin,	-1.142452947	0	0.52685124	0	1	0	11.5891299	11.7303959	11.582108	11.4375238
3560	**ENSG0000010884**	ABCC3	protein_coding	chr17	50634777	50692253	+	Human GRCh	OTTHUMG000001	ABCC3	**ATP binding cassette subfamily C member 3**	Plasma Mem	transporter		1.393182806	0	0.60972479	0	1	0	2.07016707	3.53781769	3.03493588	3.38341909
3561	**ENSG0000010884**	LUC7L3	protein_coding	chr17	50719565	50756219	+	Human GRCh	OTTHUMG000001	LUC7L3	**LUC7 like 3 pre-mRNA splicing factor**	Nucleus	other		-1.007625815	0	0.97036528	0	1	0	11.7726888	12.0265554	12.0515052	11.721619
3563	**ENSG0000010885**	MPP2	protein_coding	chr17	43875357	43909711	-	Human GRCh	OTTHUMG000001	MPP2	**MAGUK p55 scaffold protein 2**	Plasma Mem	kinase		-1.009445676	0	0.97228025	0	1	0	7.11800371	7.39432589	7.21947598	7.28590094
3564	**ENSG0000010885**	SMURF2	protein_coding	chr17	64542282	64662307	-	Human GRCh	OTTHUMG000001	SMURF2	**SMAD specific E3 ubiquitin protein ligase 2**	Cytoplasm	enzyme		-1.170145998	0	0.45951976	0	1	0	10.1809999	10.4180951	10.2857613	9.95796883
3565	**ENSG0000010886**	DUSP3	protein_coding	chr17	43766125	43778977	-	Human GRCh	OTTHUMG000001	DUSP3	**dual specificity phosphatase 3**	Cytoplasm	phosphatase		-1.136659838	0	0.59831531	0	1	0	10.7773092	11.132723	10.8718597	10.7727033
3567	**ENSG0000010888**	EFTUD2	protein_coding	chr17	44849948	44899445	-	Human GRCh	OTTHUMG000001	EFTUD2	**elongation factor Tu GTP binding domain containing 2**	Nucleus	enzyme		-1.222960119	0	0.30466404	0	1	0	11.7463355	12.106172	11.8251164	11.5930429
3568	**ENSG0000010892**	HLF	protein_coding	chr17	55264960	55325187	+	Human GRCh	OTTHUMG000001	HLF	**HLF transcription factor, PAR bZIP family member**	Nucleus	transcription		1.37901449	0	0.50589117	0	1	0	5.18515799	5.33244834	5.5442186	5.59831296
3569	**ENSG0000010893**	SLC16A6	protein_coding	chr17	68267026	68291267	-	Human GRCh	OTTHUMG000001	SLC16A6	**solute carrier family 16 member 6**	Plasma Mem	transporter		-1.122927604	0	0.82910805	0	1	0	5.57476367	6.12568157	5.43431606	6.00806185
3570	**ENSG0000010894**	PRKAR1A	protein_coding	chr17	68511780	68551319	+	Human GRCh	OTTHUMG000001	PRKAR1A	**protein kinase cAMP-dependent type I regulatory subunit alph**	Cytoplasm	kinase		-1.032171954	0	0.88147897	0	1	0	12.022193	12.2647311	12.2800975	11.9216104
3571	**ENSG0000010894**	EFNB3	protein_coding	chr17	7705202	7711372	+	Human GRCh	OTTHUMG000001	EFNB3	**ephrin B3**	Plasma Mem	kinase		-1.145784368	0	0.63860635	0	1	0	8.74685018	8.94940995	8.97539076	8.34891169
3572	**ENSG0000010895**	FAM20A	protein_coding	chr17	68535113	68601367	-	Human GRCh	OTTHUMG000001	FAM20A	**FAM20A golgi associated secretory pathway pseudokinase**	Extracellular	other		1.360146417	0	0.73691165	0	1	0	2.89810875	3.37475577	3.02802941	3.68987632

3573	**ENSG0000010895**	YWHAE	protein_coding	chr17	1344275	1400222	-	Human GRCh	OTTHUMG000001	YWHAE	**tyrosine 3-monooxygenase/tryptophan 5-monooxygenase acti**	Cytoplasm	other		-1.20761669	0	0.38438829	0	1	0	13.9785371	14.2821725	14.0282927	13.8164743
3574	**ENSG0000010896**	MMD	protein_coding	chr17	55392622	55421924	-	Human GRCh	OTTHUMG000001	MMD	**monocyte to macrophage differentiation associated**	Plasma Mem	kinase		1.06323072	0	0.77831887	0	1	0	8.1403426	8.0407467	8.25930429	8.04696718
3575	**ENSG0000010896**	RANGRF	protein_coding	chr17	8288654	8290092	+	Human GRCh	OTTHUMG000001	RANGRF	**RAN guanine nucleotide release factor**	Nucleus	other		1.011044	0	0.96713703	0	1	0	8.28043418	8.72432614	8.62478573	8.43753092
3576	**ENSG0000010896**	DPH1	protein_coding	chr17	2030137	2043898	+	Human GRCh	OTTHUMG000001	DPH1	**diphthamide biosynthesis 1**	Cytoplasm	enzyme		-1.112761449	0	0.58948254	0	1	0	10.3706005	10.7730401	10.5690335	10.3560579
3577	**ENSG0000010898**	MAP2K6	protein_coding	chr17	69414697	69553865	+	Human GRCh	OTTHUMG000001	MAP2K6	**mitogen-activated protein kinase kinase 6**	Cytoplasm	kinase		1.093936531	0	0.85022294	0	1	0	6.58474688	7.05578895	7.20438033	6.59847386
3578	**ENSG0000010901**	DHRS7B	protein_coding	chr17	21123364	21193265	+	Human GRCh	OTTHUMG000001	DHRS7B	**dehydrogenase/reductase 7B**	Cytoplasm	enzyme		-1.16930314	0	0.55052049	0	1	0	8.46674912	8.8010082	8.67958797	8.22157961
3579	**ENSG0000010904**	WSB1	protein_coding	chr17	27294076	27315926	+	Human GRCh	OTTHUMG000001	WSB1	**WD repeat and SOCS box containing 1**	Cytoplasm	enzyme		-1.02529644	0	0.90918961	0	1	0	11.8928841	12.0575477	12.1168017	11.7570878
3582	**ENSG0000010906**	NHERF1	protein_coding	chr17	74748628	74769353	+	Human GRCh	OTTHUMG000001	NHERF1	**NHERF family PDZ scaffold protein 1**	Plasma Mem	transporter		1.213565723	0	0.40934337	0	1	0	9.052887	9.27309564	9.45485789	9.29812442
3583	**ENSG0000010906**	MYH3	protein_coding	chr17	10628526	10657309	-	Human GRCh	OTTHUMG000001	MYH3	**myosin heavy chain 3**	Cytoplasm	enzyme		-1.260793102	0	0.47808081	0	1	0	7.35980248	7.69437072	7.461913	7.08649116
3584	**ENSG0000010906**	NAT9	protein_coding	chr17	74770529	74776367	-	Human GRCh	OTTHUMG000001	NAT9	**N-acetyltransferase 9 (putative)**	Other	other		1.034833781	0	0.88745644	0	1	0	9.52272369	9.77024334	9.86012298	9.49315283
3585	**ENSG0000010906**	TMEM104	protein_coding	chr17	74776499	74839753	+	Human GRCh	OTTHUMG000001	TMEM104	**transmembrane protein 104**	Other	transporter		1.055359664	0	0.81545321	0	1	0	9.55528097	9.91311172	9.96953882	9.62081764
3587	**ENSG0000010907**	TNFAIP1	protein_coding	chr17	28335602	28347009	+	Human GRCh	OTTHUMG000001	TNFAIP1	**TNF alpha induced protein 1**	Plasma Mem	ion channel		-1.117009196	0	0.62838247	0	1	0	9.78849177	10.129912	9.96094927	9.72783957
3588	**ENSG0000010908**	IFT20	protein_coding	chr17	28328325	28335489	-	Human GRCh	OTTHUMG000001	IFT20	**intraflagellar transport 20**	Cytoplasm	other		1.195051451	0	0.46983009	0	1	0	8.21078531	8.42401265	8.52232685	8.50744213
3589	**ENSG0000010908**	TMEM97	protein_coding	chr17	28319200	28328685	+	Human GRCh	OTTHUMG000001	TMEM97	**transmembrane protein 97**	Extracellular	other	CT1812	-1.172611706	0	0.50319861	0	1	0	11.1768791	11.4851948	11.2632133	11.0537107
3590	**ENSG0000010908**	CDR2L	protein_coding	chr17	74987632	75005800	+	Human GRCh	OTTHUMG000001	CDR2L	**cerebellar degeneration related protein 2 like**	Other	other		1.198704343	0	0.38670813	0	1	0	9.26535865	9.41432938	9.66860756	9.40628892
3591	**ENSG0000010909**	PMP22	protein_coding	chr17	15229773	15272292	-	Human GRCh	OTTHUMG000000	PMP22	**peripheral myelin protein 22**	Plasma Mem	other		1.44148397	0	0.5186382	0	1	0	3.89060654	4.04090731	3.83135676	4.49878874
3592	**ENSG0000010910**	FOXN1	protein_coding	chr17	28506243	28538900	+	Human GRCh	OTTHUMG000001	FOXN1	**forkhead box N1**	Nucleus	transcription		1.751397251	1	0.59772936	0	NA	0	2.8321472	2.70247284	2.82662841	3.42031383
3593	**ENSG0000010910**	UNC119	protein_coding	chr17	28546708	28552631	-	Human GRCh	OTTHUMG000001	UNC119	**unc-119 lipid binding chaperone**	Cytoplasm	other		1.209164691	0	0.43075902	0	1	0	9.49515003	10.0693119	10.0547851	9.99858614
3594	**ENSG0000010910**	ALDOC	protein_coding	chr17	28573115	28576948	-	Human GRCh	OTTHUMG000001	ALDOC	**aldolase, fructose-bisphosphate C**	Cytoplasm	enzyme		1.13509899	0	0.65335835	0	1	0	9.97319851	10.8570508	10.6114285	10.6701329
3595	**ENSG0000010911**	SUPT6H	protein_coding	chr17	28662198	28702679	+	Human GRCh	OTTHUMG000001	SUPT6H	**SPT6 homolog, histone chaperone and transcription elongatio**	Nucleus	transcription		-1.081747464	0	0.67991142	0	1	0	12.0958115	12.4342695	12.2369276	12.1396582
3596	**ENSG0000010911**	RAB34	protein_coding	chr17	28714281	28718429	-	Human GRCh	OTTHUMG000001	RAB34	**RAB34, member RAS oncogene family**	Cytoplasm	enzyme		-1.228965863	0	0.41991393	0	1	0	8.66983823	9.48821466	9.11546321	8.69348368
3597	**ENSG0000010911**	PHF12	protein_coding	chr17	28905250	28951771	-	Human GRCh	OTTHUMG000001	PHF12	**PHD finger protein 12**	Nucleus	transcription		-1.13045253	0	0.53395549	0	1	0	11.3917528	11.6856149	11.465948	11.3564433
3599	**ENSG0000010913**	TMEM33	protein_coding	chr4	41935129	41960803	+	Human GRCh	OTTHUMG000000	TMEM33	**transmembrane protein 33**	Cytoplasm	other		1.054664223	0	0.78677942	0	1	0	12.4341269	12.6210892	12.7236747	12.4427974
3602	**ENSG0000010917**	SLAIN2	protein_coding	chr4	48341529	48426201	+	Human GRCh	OTTHUMG000001	SLAIN2	**SLAIN motif family member 2**	Cytoplasm	other		-1.00538551	0	0.97900077	0	1	0	10.7279247	10.9579632	10.9598738	10.712222
3603	**ENSG0000010918**	OCIAD1	protein_coding	chr4	48805212	48861817	+	Human GRCh	OTTHUMG000001	OCIAD1	**OCIA domain containing 1**	Cytoplasm	other		-1.014195969	0	0.94699633	0	1	0	10.616757	10.9816558	10.9403892	10.635345
3606	**ENSG0000010918**	DCUN1D4	protein_coding	chr4	51843000	51916837	+	Human GRCh	OTTHUMG000001	DCUN1D4	**defective in cullin neddylation 1 domain containing 4**	Nucleus	other		-1.037737286	0	0.8719894	0	1	0	10.6392934	10.9529874	10.9819223	10.5000416
3607	**ENSG0000010918**	USP46	protein_coding	chr4	52590960	52659301	-	Human GRCh	OTTHUMG000001	USP46	**ubiquitin specific peptidase 46**	Plasma Mem	peptidase		1.007259985	0	0.97530346	0	1	0	9.60834363	9.77750717	9.89559118	9.47507102
3608	**ENSG0000010919**	SULT1E1	protein_coding	chr4	69841212	69860145	-	Human GRCh	OTTHUMG000001	SULT1E1	**sulfotransferase family 1E member 1**	Cytoplasm	enzyme	estrogen s	-5.686004848	-1	0.22020107	0	1	0	0.42794616	1.46856121	0.4974071	0.43300362
3611	**ENSG0000010922**	CHIC2	protein_coding	chr4	54009789	54064605	-	Human GRCh	OTTHUMG000001	CHIC2	**cysteine rich hydrophobic domain 2**	Plasma Mem	other		1.10182141	0	0.67261816	0	1	0	8.13926932	8.42933802	8.58381159	8.17697291
3612	**ENSG0000010925**	NMU	protein_coding	chr4	55595229	55636698	-	Human GRCh	OTTHUMG000001	NMU	**neuromedin U**	Extracellular	other		-1.073531816	0	0.83991818	0	1	0	6.51582126	6.42416842	6.41661536	6.38265021
3613	**ENSG0000010926**	CRACD	protein_coding	chr4	56049098	56330609	+	Human GRCh	OTTHUMG000001	CRACD	**capping protein inhibiting regulator of actin dynamics**	Other	other		-1.102346331	0	0.61874653	0	1	0	10.1911875	10.4497442	10.4126419	9.98744033
3614	**ENSG0000010927**	LAMTOR3	protein_coding	chr4	99878336	99894428	-	Human GRCh	OTTHUMG000001	LAMTOR3	**late endosomal/lysosomal adaptor, MAPK and MTOR activato**	Cytoplasm	other		1.157552876	0	0.53057948	0	1	0	9.27746725	9.56988904	9.75843478	9.40436018
3616	**ENSG0000010932**	NFKB1	protein_coding	chr4	102501330	102617302	+	Human GRCh	OTTHUMG000001	NFKB1	**nuclear factor kappa B subunit 1**	Nucleus	transcription	dexametha	1.061261604	0	0.80827228	0	1	0	9.34773345	9.65702608	9.78937735	9.32038205
3618	**ENSG0000010932**	MANBA	protein_coding	chr4	102630770	102760994	-	Human GRCh	OTTHUMG000001	MANBA	**mannosidase beta**	Cytoplasm	enzyme		1.089781313	0	0.69803449	0	1	0	9.71472812	10.023651	10.0770365	9.86208582
3619	**ENSG0000010933**	UBE2D3	protein_coding	chr4	102794383	102868896	-	Human GRCh	OTTHUMG000001	UBE2D3	**ubiquitin conjugating enzyme E2 D3**	Cytoplasm	enzyme		-1.043202384	0	0.82965167	0	1	0	12.6321283	12.88485	12.8594792	12.5565966
3620	**ENSG0000010933**	MAPK10	protein_coding	chr4	85990007	86594625	-	Human GRCh	OTTHUMG000001	MAPK10	**mitogen-activated protein kinase 10**	Cytoplasm	kinase	SP600125,	1.079218775	0	0.83316819	0	1	0	7.11558056	6.81296619	6.94922085	7.15060199
3621	**ENSG0000010938**	ELF2	protein_coding	chr4	139028112	139177218	-	Human GRCh	OTTHUMG000001	ELF2	**E74 like ETS transcription factor 2**	Nucleus	transcription		-1.167298635	0	0.47537912	0	1	0	10.6634915	10.9595992	10.6823413	10.6190633
3622	**ENSG0000010939**	NDUFC1	protein_coding	chr4	139266880	139302551	-	Human GRCh	OTTHUMG000001	NDUFC1	**NADH:ubiquinone oxidoreductase subunit C1**	Cytoplasm	enzyme		1.113576106	0	0.66730035	0	1	0	8.91187531	9.43247013	9.35157997	9.28957921
3624	**ENSG0000010943**	TBC1D9	protein_coding	chr4	140620782	140756385	-	Human GRCh	OTTHUMG000001	TBC1D9	**TBC1 domain family member 9**	Plasma Mem	other		1.221673466	0	0.3943824	0	1	0	8.57678833	8.50612848	8.83626502	8.67002812
3625	**ENSG0000010944**	ZNF330	protein_coding	chr4	141220887	141234697	+	Human GRCh	OTTHUMG000001	ZNF330	**zinc finger protein 330**	Nucleus	other		1.103213246	0	0.68558824	0	1	0	9.05258543	9.0833355	9.33607706	8.99426932
3626	**ENSG0000010945**	INPP4B	protein_coding	chr4	142023160	142847432	-	Human GRCh	OTTHUMG000001	INPP4B	**inositol polyphosphate-4-phosphatase type II B**	Cytoplasm	phosphatase		1.238407186	0	0.42105897	0	1	0	9.10555623	9.49295133	9.57864534	9.51355313
3627	**ENSG0000010945**	GAB1	protein_coding	chr4	143336876	143474565	+	Human GRCh	OTTHUMG000001	GAB1	**GRB2 associated binding protein 1**	Cytoplasm	other		-1.031788277	0	0.90007565	0	1	0	8.85416845	9.14837027	9.20433089	8.69245663
3628	**ENSG0000010946**	KLHL2	protein_coding	chr4	165207561	165323156	+	Human GRCh	OTTHUMG000001	KLHL2	**kelch like family member 2**	Cytoplasm	other		1.082490166	0	0.73916656	0	1	0	8.05181604	8.13592306	8.11980963	8.23021606
3630	**ENSG0000010947**	CPE	protein_coding	chr4	165361194	165498547	+	Human GRCh	OTTHUMG000001	CPE	**carboxypeptidase E**	Cytoplasm	peptidase		1.005556808	0	0.98013803	0	1	0	9.43620229	9.89203098	9.81081588	9.55980959
3631	**ENSG0000010947**	RPL34	protein_coding	chr4	108620569	108630412	+	Human GRCh	OTTHUMG000001	RPL34	**ribosomal protein L34**	Cytoplasm	other		-1.010095198	0	0.96270362	0	1	0	12.1352247	12.4531524	12.4291254	12.1399156
3632	**ENSG0000010950**	WFS1	protein_coding	chr4	6269849	6303265	+	Human GRCh	OTTHUMG000000	WFS1	**wolframin ER transmembrane glycoprotein**	Cytoplasm	enzyme		-1.090490609	0	0.76286508	0	1	0	9.65147173	9.81410921	9.70217253	9.56724306
3634	**ENSG0000010951**	GRPEL1	protein_coding	chr4	7058671	7068085	-	Human GRCh	OTTHUMG000000	GRPEL1	**GrpE like 1, mitochondrial**	Cytoplasm	other		1.095167074	0	0.65976645	0	1	0	10.0218399	10.3354294	10.381128	10.1966854
3635	**ENSG0000010953**	GAR1	protein_coding	chr4	109815510	109824740	+	Human GRCh	OTTHUMG000001	GAR1	**GAR1 ribonucleoprotein**	Nucleus	other		1.161485854	0	0.49196113	0	1	0	9.03651102	9.43336771	9.55466723	9.26688884
3636	**ENSG0000010953**	FRG1	protein_coding	chr4	189940870	189963202	+	Human GRCh	OTTHUMG000001	FRG1	**FSHD region gene 1**	Nucleus	transporter		-1.095304955	0	0.66697199	0	1	0	9.12583885	9.47373372	9.27887944	9.13722615
3637	**ENSG0000010957**	CLCN3	protein_coding	chr4	169612633	169723673	+	Human GRCh	OTTHUMG000001	CLCN3	**chloride voltage-gated channel 3**	Plasma Mem	ion channel		1.009508769	0	0.96495201	0	1	0	10.2482123	10.5085101	10.5273344	10.2479378
3638	**ENSG0000010957**	AADAT	protein_coding	chr4	170060222	170091699	-	Human GRCh	OTTHUMG000001	AADAT	**aminoadipate aminotransferase**	Cytoplasm	enzyme		-1.357718729	0	0.16477973	0	1	0	8.89341603	9.15965353	8.68644799	8.712247
3639	**ENSG0000010958**	GALNT7	protein_coding	chr4	173168811	173323967	+	Human GRCh	OTTHUMG000001	GALNT7	**polypeptide N-acetylgalactosaminyltransferase 7**	Cytoplasm	enzyme		1.049143877	0	0.84575581	0	1	0	9.13867172	9.10683439	9.26147748	9.08172908
3640	**ENSG0000010960**	DHX15	protein_coding	chr4	24517441	24584554	-	Human GRCh	OTTHUMG000001	DHX15	**DEAH-box helicase 15**	Nucleus	enzyme		-1.160339656	0	0.44492865	0	1	0	12.945961	13.1944177	13.0535271	12.7482775
3641	**ENSG0000010961**	SOD3	protein_coding	chr4	24789912	24800842	+	Human GRCh	OTTHUMG000001	SOD3	**superoxide dismutase 3**	Extracellular	enzyme		-1.050906122	0	0.87948095	0	1	0	10.2547167	10.5642035	10.4812452	10.2302274
3642	**ENSG0000010961**	SEPSECS	protein_coding	chr4	25120014	25160550	-	Human GRCh	OTTHUMG000001	SEPSECS	**Sep (O-phosphoserine) tRNA:Sec (selenocysteine) tRNA syntha**	Cytoplasm	enzyme		-1.191591382	0	0.45536738	0	1	0	9.91941147	10.2186434	9.96185571	9.80012623
3644	**ENSG0000010965**	TRIM2	protein_coding	chr4	153152163	153339319	+	Human GRCh	OTTHUMG000001	TRIM2	**tripartite motif containing 2**	Cytoplasm	enzyme		1.558652217	1	0.53618129	0	1	0	5.16168319	5.80838269	5.60477066	5.97277508
3645	**ENSG0000010966**	SLC2A9	protein_coding	chr4	9771153	10054936	-	Human GRCh	OTTHUMG000000	SLC2A9	**solute carrier family 2 member 9**	Plasma Mem	transporter		-1.281319801	0	0.67972403	0	1	0	5.15569069	5.11509714	5.23141764	4.43300043
3646	**ENSG0000010967**	FBXW7	protein_coding	chr4	152320544	152536092	-	Human GRCh	OTTHUMG000001	FBXW7	**F-box and WD repeat domain containing 7**	Nucleus	enzyme		1.137261618	0	0.55584712	0	1	0	9.73346296	9.98635108	10.1925637	9.78923612
3647	**ENSG0000010967**	NEIL3	protein_coding	chr4	177309874	177362936	+	Human GRCh	OTTHUMG000001	NEIL3	**nei like DNA glycosylase 3**	Nucleus	enzyme		-1.109538702	0	0.67468265	0	1	0	8.35442734	8.75776802	8.54876971	8.36817483
3648	**ENSG0000010968**	TBC1D19	protein_coding	chr4	26576437	26756223	+	Human GRCh	OTTHUMG000000	TBC1D19	**TBC1 domain family member 19**	Other	other		-1.268452692	0	0.42394295	0	1	0	8.01959686	7.9895134	7.89465623	7.6018546
3649	**ENSG0000010968**	CLNK	protein_coding	chr4	10486395	10684768	-	Human GRCh	OTTHUMG000001	CLNK	**cytokine dependent hematopoietic cell linker**	Cytoplasm	other		-1.127335963	0	0.9012199	0	1	0	3.03275072	4.06017739	3.80088063	3.27792838
3650	**ENSG0000010968**	NSD2	protein_coding	chr4	1871393	1982207	+	Human GRCh	OTTHUMG000001	NSD2	**nuclear receptor binding SET domain protein 2**	Nucleus	transcription	KTX-1001	-1.148948876	0	0.50683138	0	1	0	13.27956	13.5365144	13.459798	13.0295298
3651	**ENSG0000010968**	SH3D19	protein_coding	chr4	151102751	151325605	-	Human GRCh	OTTHUMG000001	SH3D19	**SH3 domain containing 19**	Plasma Mem	other		-1.151193889	0	0.51809155	0	1	0	11.1809775	11.1684266	11.0870052	10.9455842
3652	**ENSG0000010968**	STIM2	protein_coding	chr4	26857601	27025381	+	Human GRCh	OTTHUMG000000	STIM2	**stromal interaction molecule 2**	Plasma Mem	transporter		-1.094378976	0	0.64045821	0	1	0	9.95673118	10.3478215	10.1429669	9.9891128
3653	**ENSG0000010970**	NKX3-2	protein_coding	chr4	13540830	13544508	-	Human GRCh	OTTHUMG000000	NKX3-2	**NK3 homeobox 2**	Nucleus	transcription		-1.514409609	-1	0.46150867	0	1	0	5.21062888	5.38234002	5.21453284	4.45204663
3654	**ENSG0000010973**	MFSD10	protein_coding	chr4	2930561	2934834	-	Human GRCh	OTTHUMG000001	MFSD10	**major facilitator superfamily domain containing 10**	Nucleus	transporter		-1.043285539	0	0.84461466	0	1	0	9.89982323	10.1568137	10.1420227	9.81251686
3655	**ENSG0000010973**	GLRB	protein_coding	chr4	157076125	157172090	+	Human GRCh	OTTHUMG000001	GLRB	**glycine receptor beta**	Plasma Mem	ion channel		1.119125164	0	0.78943891	0	1	0	7.44471526	7.74847354	7.88489693	7.55089239
3656	**ENSG0000010974**	BST1	protein_coding	chr4	15703065	15738313	+	Human GRCh	OTTHUMG000000	BST1	**bone marrow stromal cell antigen 1**	Plasma Mem	enzyme	MEN1112	-1.550197474	-1	0.60384767	0	1	0	3.39086983	4.1167316	3.79151104	3.10593683
3657	**ENSG0000010975**	RAPGEF2	protein_coding	chr4	159103013	159360174	+	Human GRCh	OTTHUMG000001	RAPGEF2	**Rap guanine nucleotide exchange factor 2**	Cytoplasm	other		-1.020203342	0	0.92523275	0	1	0	10.7197956	11.0079188	10.9648914	10.7232325
3658	**ENSG0000010975**	HGFAC	protein_coding	chr4	3441968	3449486	+	Human GRCh	OTTHUMG000000	HGFAC	**HGF activator**	Extracellular	peptidase		-1.023166102	0	0.95762441	0	1	0	6.12184111	6.1093134	6.46889803	5.53197598
3659	**ENSG0000010976**	SNX25	protein_coding	chr4	185204237	185370185	+	Human GRCh	OTTHUMG000001	SNX25	**sorting nexin 25**	Cytoplasm	other		-1.078251849	0	0.73867321	0	1	0	9.75234104	9.95860679	9.88507923	9.65373429
3660	**ENSG0000010977**	LRP2BP	protein_coding	chr4	185363872	185395924	-	Human GRCh	OTTHUMG000001	LRP2BP	**LRP2 binding protein**	Cytoplasm	other		1.183903516	0	0.61510367	0	1	0	6.49458724	6.80696008	7.079648	6.52759221
3661	**ENSG0000010977**	UFSP2	protein_coding	chr4	185399537	185425979	-	Human GRCh	OTTHUMG000001	UFSP2	**UFM1 specific peptidase 2**	Other	peptidase		-1.055461225	0	0.81762251	0	1	0	8.82355823	9.21124736	9.06840067	8.8695655
3662	**ENSG0000010978**	KLF3	protein_coding	chr4	38664197	38701517	+	Human GRCh	OTTHUMG000000	KLF3	**KLF transcription factor 3**	Nucleus	transcription		-1.145492917	0	0.47823059	0	1	0	11.7992643	12.0009349	11.8058054	11.6973132
3663	**ENSG0000010979**	KLHL5	protein_coding	chr4	39045039	39126857	+	Human GRCh	OTTHUMG000000	KLHL5	**kelch like family member 5**	Extracellular	other		-1.266880715	0	0.36455562	0	1	0	8.46699859	8.79088112	8.46853859	8.27917998
3664	**ENSG0000010979**	FAM149A	protein_coding	chr4	186104419	186175337	+	Human GRCh	OTTHUMG000001	FAM149A	**family with sequence similarity 149 member A**	Other	other		-1.534405992	-1	0.27573282	0	1	0	4.55293385	5.03059474	4.19193981	4.67011924
3665	**ENSG0000010980**	NCAPG	protein_coding	chr4	17810979	17844865	+	Human GRCh	OTTHUMG000001	NCAPG	**non-SMC condensin I complex subunit G**	Nucleus	other		-1.085454511	0	0.7013878	0	1	0	10.9067381	11.3648225	11.1188839	11.0151944
3666	**ENSG0000010981**	UGDH	protein_coding	chr4	39498755	39528311	-	Human GRCh	OTTHUMG000000	UGDH	**UDP-glucose 6-dehydrogenase**	Nucleus	enzyme		1.11738301	0	0.73832092	0	1	0	11.0008365	11.3616876	11.4198936	11.2110839
3667	**ENSG0000010981**	PPARGC1A	protein_coding	chr4	23755041	23904089	-	Human GRCh	OTTHUMG000000	PPARGC1A	**PPARG coactivator 1 alpha**	Nucleus	transcription		2.803221506	1	0.05085054	0	1	0	6.06264402	6.20074138	6.36313218	7.70307825
3668	**ENSG0000010983**	DDX25	protein_coding	chr11	125903348	125943702	+	Human GRCh	OTTHUMG000001	DDX25	**DEAD-box helicase 25**	Nucleus	enzyme		1.966985329	1	0.36375483	0	1	0	2.61468629	2.9370551	3.22571715	3.10686317
3669	**ENSG0000010984**	CRYAB	protein_coding	chr11	111908564	111923722	-	Human GRCh	OTTHUMG000001	CRYAB	**crystallin alpha B**	Nucleus	other		10.49494433	1	0.04598433	1	1	0	2.4792697	1.76872238	3.54917403	3.75806252
3671	**ENSG0000010985**	HTATIP2	protein_coding	chr11	20363685	20383782	+	Human GRCh	OTTHUMG000001	HTATIP2	**HIV-1 Tat interactive protein 2**	Nucleus	transcription		-1.560054363	-1	0.1295207	0	1	0	6.39434824	6.36189438	5.87216979	6.06076672
3672	**ENSG0000010986**	CTSC	protein_coding	chr11	88265069	88359684	-	Human GRCh	OTTHUMG000001	CTSC	**cathepsin C**	Cytoplasm	peptidase		1.145124904	0	0.83516231	0	1	0	8.58989694	8.80112515	8.6525284	8.99940817

3673	**ENSG0000010988**	CCDC34	protein_coding	chr11	27330827	27363234	-	Human GRCh	OTTHUMG000001	CCDC34	**coiled-coil domain containing 34**	Cytoplasm	other		1.086304471	0	0.73780609	0	1	0	7.96141242	8.25745676	8.31765988	8.07987025
3674	**ENSG0000010990**	ZBTB16	protein_coding	chr11	114059041	114256765	+	Human GRCh	OTTHUMG000001	ZBTB16	**zinc finger and BTB domain containing 16**	Nucleus	transcription		-1.086263023	0	0.7573661	0	1	0	14.0218678	14.1692115	14.1387607	13.8409715
3675	**ENSG0000010991**	ELP4	protein_coding	chr11	31509755	31790324	+	Human GRCh	OTTHUMG000001	ELP4	**elongator acetyltransferase complex subunit 4**	Cytoplasm	other		1.035902254	0	0.86731991	0	1	0	9.5319073	9.64559273	9.74750362	9.49639877
3676	**ENSG0000010991**	ZPR1	protein_coding	chr11	116773799	116788039	-	Human GRCh	OTTHUMG000000	ZPR1	**ZPR1 zinc finger**	Nucleus	other		1.075547991	0	0.73781562	0	1	0	9.75181859	10.1320449	10.1590925	9.90208947
3677	**ENSG0000010991**	MTCH2	protein_coding	chr11	47617315	47642607	-	Human GRCh	OTTHUMG000001	MTCH2	**mitochondrial carrier 2**	Cytoplasm	other		-1.115193105	0	0.67773518	0	1	0	11.2758321	11.7097991	11.5172394	11.2477163
3678	**ENSG0000010992**	FNBP4	protein_coding	chr11	47716494	47767443	-	Human GRCh	OTTHUMG000001	FNBP4	**formin binding protein 4**	Nucleus	other		-1.118603582	0	0.59192757	0	1	0	12.0329051	12.2720016	12.2005098	11.8423231
3679	**ENSG0000010992**	TECTA	protein_coding	chr11	121101243	121191490	+	Human GRCh	OTTHUMG000001	TECTA	**tectorin alpha**	Extracellular	enzyme		-1.491560897	0	0.28563648	0	1	0	5.88707452	5.98619133	5.67785885	5.42501348
3680	**ENSG0000010992**	SC5D	protein_coding	chr11	121292681	121313410	+	Human GRCh	OTTHUMG000001	SC5D	**sterol-C5-desaturase**	Cytoplasm	enzyme		-1.081637792	0	0.7254234	0	1	0	12.0179761	12.2548509	12.2429428	11.8257535
3682	**ENSG0000010994**	JHY	protein_coding	chr11	122882683	122963862	+	Human GRCh	OTTHUMG000001	JHY	**junctional cadherin complex regulator**	Extracellular	other		-2.813609287	-1	0.24871461	0	1	0	4.25254438	4.43896903	3.65761861	2.89448545
3683	**ENSG0000010995**	B3GAT1	protein_coding	chr11	134378504	134412242	-	Human GRCh	OTTHUMG000001	B3GAT1	**beta-1,3-glucuronyltransferase 1**	Cytoplasm	enzyme	aprepitant	-1.081038793	0	0.88440096	0	1	0	13.5179106	13.6731389	13.6384725	13.3559762
3684	**ENSG0000010997**	HSPA8	protein_coding	chr11	123057489	123063230	-	Human GRCh	OTTHUMG000001	HSPA8	**heat shock protein family A (Hsp70) member 8**	Cytoplasm	enzyme	SHetA2	1.125898153	0	0.59653735	0	1	0	14.3330875	14.7834062	14.9184433	14.4663595
3686	**ENSG0000011000**	VWA5A	protein_coding	chr11	124115404	124147721	+	Human GRCh	OTTHUMG000001	VWA5A	**von Willebrand factor A domain containing 5A**	Nucleus	other		3.832207507	1	0.12347811	0	1	0	3.52136048	3.14473397	4.72856307	4.5429949
3687	**ENSG0000011001**	DNAJC4	protein_coding	chr11	64230278	64234286	+	Human GRCh	OTTHUMG000001	DNAJC4	**DnaJ heat shock protein family (Hsp40) member C4**	Cytoplasm	other		1.093839366	0	0.70652352	0	1	0	9.23731936	9.44701349	9.46768088	9.4235026
3688	**ENSG0000011001**	SIAE	protein_coding	chr11	124633113	124695707	-	Human GRCh	OTTHUMG000001	SIAE	**sialic acid acetylesterase**	Cytoplasm	enzyme		-1.239406903	0	0.37991292	0	1	0	8.26454727	8.5701204	8.33890391	8.02283563
3689	**ENSG0000011002**	SNX15	protein_coding	chr11	65027439	65040572	+	Human GRCh	OTTHUMG000000	SNX15	**sorting nexin 15**	Cytoplasm	transporter		-1.181570092	0	0.48303761	0	1	0	8.45439439	8.65754197	8.46636461	8.28722617
3690	**ENSG0000011003**	LPXN	protein_coding	chr11	58526871	58578220	-	Human GRCh	OTTHUMG000001	LPXN	**leupaxin**	Cytoplasm	transcription		-1.350037395	0	0.227703	0	1	0	8.32814635	8.53381903	8.25591235	7.94682297
3691	**ENSG0000011004**	DTX4	protein_coding	chr11	59171430	59208588	+	Human GRCh	OTTHUMG000001	DTX4	**deltex E3 ubiquitin ligase 4**	Cytoplasm	enzyme		-1.136453674	0	0.79391474	0	1	0	5.66784908	5.36331276	5.59476501	5.1757021
3692	**ENSG0000011004**	ATG2A	protein_coding	chr11	64894546	64917209	-	Human GRCh	OTTHUMG000000	ATG2A	**autophagy related 2A**	Cytoplasm	transporter		-1.099313301	0	0.72952417	0	1	0	10.9859113	11.3334765	11.0935404	11.0380165
3693	**ENSG0000011004**	EHD1	protein_coding	chr11	64851642	64888296	-	Human GRCh	OTTHUMG000000	EHD1	**EH domain containing 1**	Cytoplasm	other		1.099813182	0	0.71555566	0	1	0	9.91853835	10.122964	10.2800071	9.96559917
3694	**ENSG0000011004**	OSBP	protein_coding	chr11	59574398	59615774	-	Human GRCh	OTTHUMG000001	OSBP	**oxysterol binding protein**	Cytoplasm	transporter		-1.112329011	0	0.58598208	0	1	0	11.0824866	11.4603888	11.2761066	11.0495687
3695	**ENSG0000011005**	UNC93B1	protein_coding	chr11	67991100	68004982	-	Human GRCh	OTTHUMG000001	UNC93B1	**unc-93 homolog B1, TLR signaling regulator**	Cytoplasm	other		-1.032312218	0	0.89360473	0	1	0	9.08658377	9.58491889	9.38948719	9.26135509
3696	**ENSG0000011006**	PUS3	protein_coding	chr11	125893485	125903221	-	Human GRCh	OTTHUMG000001	PUS3	**pseudouridine synthase 3**	Nucleus	enzyme		1.194951411	0	0.55293393	0	1	0	7.26769012	7.55455964	7.64628842	7.57773224
3697	**ENSG0000011006**	DCPS	protein_coding	chr11	126304060	126350005	+	Human GRCh	OTTHUMG000001	DCPS	**decapping enzyme, scavenger**	Nucleus	enzyme		-1.023452818	0	0.91910739	0	1	0	9.52728289	9.96404227	9.90704408	9.54539196
3698	**ENSG0000011006**	KMT5B	protein_coding	chr11	68154863	68213852	-	Human GRCh	OTTHUMG000001	KMT5B	**lysine methyltransferase 5B**	Nucleus	enzyme		-1.06617665	0	0.75521407	0	1	0	10.6967534	10.8519673	10.8947051	10.4897662
3699	**ENSG0000011007**	FOXRED1	protein_coding	chr11	126269024	126278131	+	Human GRCh	OTTHUMG000001	FOXRED1	**FAD dependent oxidoreductase domain containing 1**	Cytoplasm	other		-1.000807985	0	0.99696325	0	1	0	10.2687326	10.4149094	10.5064479	10.1538086
3700	**ENSG0000011007**	PPP6R3	protein_coding	chr11	68460731	68615334	+	Human GRCh	OTTHUMG000001	PPP6R3	**protein phosphatase 6 regulatory subunit 3**	Cytoplasm	other		-1.172579057	0	0.43217467	0	1	0	12.1523685	12.3801434	12.2397713	11.9370019
3701	**ENSG0000011007**	NRXN2	protein_coding	chr11	64606174	64723197	-	Human GRCh	OTTHUMG000000	NRXN2	**neurexin 2**	Plasma Mem	transmembra		-1.323689297	0	0.69741695	0	1	0	5.13906648	4.40563739	2.97678353	5.17732139
3704	**ENSG0000011008**	ST3GAL4	protein_coding	chr11	126355640	126440344	+	Human GRCh	OTTHUMG000001	ST3GAL4	**ST3 beta-galactoside alpha-2,3-sialyltransferase 4**	Cytoplasm	enzyme		1.699777177	1	0.05794688	0	1	0	9.80134794	10.2158788	10.2671589	10.9213374
3705	**ENSG0000011009**	CPT1A	protein_coding	chr11	68754620	68844410	-	Human GRCh	OTTHUMG000001	CPT1A	**carnitine palmitoyltransferase 1A**	Cytoplasm	enzyme	perhexiline	-1.121541647	0	0.52635775	0	1	0	10.8983424	10.9284837	10.698736	10.8704619
3706	**ENSG0000011009**	CCND1	protein_coding	chr11	69641156	69654474	+	Human GRCh	OTTHUMG000001	CCND1	**cyclin D1**	Nucleus	transcription	arsenic tric	-1.156633983	0	0.81124548	0	1	0	14.0738141	14.3175793	14.2000279	13.8180158
3707	**ENSG0000011010**	CCDC86	protein_coding	chr11	60842113	60851081	+	Human GRCh	OTTHUMG000001	CCDC86	**coiled-coil domain containing 86**	Nucleus	other		1.070432759	0	0.77281024	0	1	0	10.9967493	11.439775	11.3542837	11.2774792
3708	**ENSG0000011010**	PRPF19	protein_coding	chr11	60890547	60906585	-	Human GRCh	OTTHUMG000001	PRPF19	**pre-mRNA processing factor 19**	Nucleus	enzyme		1.065298261	0	0.78001784	0	1	0	12.1292036	12.4636161	12.4703869	12.281232
3709	**ENSG0000011010**	TMEM109	protein_coding	chr11	60914158	60923443	+	Human GRCh	OTTHUMG000001	TMEM109	**transmembrane protein 109**	Cytoplasm	ion channel		1.04902048	0	0.83169084	0	1	0	11.024145	11.3652357	11.3242689	11.1947042
3710	**ENSG0000011014**	CCKBR	protein_coding	chr11	6259806	6272127	+	Human GRCh	OTTHUMG000001	CCKBR	**cholecystokinin B receptor**	Plasma Mem	G-protein co	triglumide	3.110434928	1	0.27063082	0	1	0	2.23710308	1.78483803	2.67193544	2.24327723
3711	**ENSG0000011016**	HPX	protein_coding	chr11	6431049	6442617	-	Human GRCh	OTTHUMG000001	HPX	**hemopexin**	Extracellular	transporter		1.666876438	1	0.28882344	0	1	0	5.07613255	4.72246342	5.52849316	5.20053081
3712	**ENSG0000011017**	TRIM3	protein_coding	chr11	6448613	6474459	-	Human GRCh	OTTHUMG000001	TRIM3	**tripartite motif containing 3**	Cytoplasm	enzyme		1.204305054	0	0.44719211	0	1	0	8.43520828	8.75134578	8.98865564	8.58177672
3713	**ENSG0000011017**	CHORDC1	protein_coding	chr11	90200429	90223077	-	Human GRCh	OTTHUMG000001	CHORDC1	**cysteine and histidine rich domain containing 1**	Other	other		1.239957597	0	0.31664524	0	1	0	10.9720653	11.074704	11.496342	10.9629017
3714	**ENSG0000011019**	FOLR1	protein_coding	chr11	72189558	72196323	+	Human GRCh	OTTHUMG000001	FOLR1	**folate receptor alpha**	Plasma Mem	transporter	STRO-002,	1	0	1	0	NA	0	1.05315163	1.06739428	1.06501226	1.05397583
3715	**ENSG0000011020**	ANAPC15	protein_coding	chr11	72106378	72112780	-	Human GRCh	OTTHUMG000001	ANAPC15	**anaphase promoting complex subunit 15**	Nucleus	other		-1.135820631	0	0.58594756	0	1	0	8.71346802	9.15698383	8.86042318	8.77356661
3717	**ENSG0000011021**	PANX1	protein_coding	chr11	94128841	94181968	+	Human GRCh	OTTHUMG000001	PANX1	**pannexin 1**	Plasma Mem	transporter		-1.337056891	0	0.1559318	0	1	0	9.46721041	9.79857299	9.43812455	9.19868513
3718	**ENSG0000011023**	ARHGEF17	protein_coding	chr11	73308276	73369388	+	Human GRCh	OTTHUMG000001	ARHGEF17	**Rho guanine nucleotide exchange factor 17**	Cytoplasm	other		-1.062366041	0	0.79934265	0	1	0	8.85777396	8.96571874	8.90488311	8.79110002
3722	**ENSG0000011027**	CEP164	protein_coding	chr11	117314557	117413266	+	Human GRCh	OTTHUMG000001	CEP164	**centrosomal protein 164**	Cytoplasm	other		-1.096584298	0	0.64601775	0	1	0	10.8408328	11.0420746	11.020417	10.636631
3723	**ENSG0000011031**	RNF141	protein_coding	chr11	10511673	10541230	-	Human GRCh	OTTHUMG000001	RNF141	**ring finger protein 141**	Other	enzyme		1.125063342	0	0.6227696	0	1	0	9.21479477	9.44856553	9.53852871	9.38269951
3724	**ENSG0000011031**	CEP126	protein_coding	chr11	101915010	102001062	+	Human GRCh	OTTHUMG000001	CEP126	**centrosomal protein 126**	Cytoplasm	other		1.118941895	0	0.69585145	0	1	0	6.99970977	7.13090543	6.97316266	7.35783349
3725	**ENSG0000011032**	EIF4G2	protein_coding	chr11	10797050	10808940	-	Human GRCh	OTTHUMG000001	EIF4G2	**eukaryotic translation initiation factor 4 gamma 2**	Cytoplasm	translation re		-1.156606318	0	0.51323656	0	1	0	14.4225908	14.7081552	14.5361429	14.2772954
3726	**ENSG0000011032**	IL10RA	protein_coding	chr11	117986370	118003037	+	Human GRCh	OTTHUMG000001	IL10RA	**interleukin 10 receptor subunit alpha**	Plasma Mem	transmembra		1.649963001	1	0.54220271	0	1	0	3.20719768	2.47743947	3.69176751	2.39731926
3727	**ENSG0000011032**	GALNT18	protein_coding	chr11	11270877	11622005	-	Human GRCh	OTTHUMG000001	GALNT18	**polypeptide N-acetylgalactosaminyltransferase 18**	Cytoplasm	enzyme		1.013722276	0	0.95969677	0	1	0	9.84990833	9.9180729	9.67665339	10.0868165
3728	**ENSG0000011033**	BIRC2	protein_coding	chr11	102347211	102378670	+	Human GRCh	OTTHUMG000001	BIRC2	**baculoviral IAP repeat containing 2**	Cytoplasm	enzyme	TQB3728,	-1.046865227	0	0.81644291	0	1	0	10.1626841	10.4744421	10.4501163	10.076797
3729	**ENSG0000011034**	UBE4A	protein_coding	chr11	118359600	118399211	+	Human GRCh	OTTHUMG000001	UBE4A	**ubiquitination factor E4A**	Cytoplasm	enzyme		-1.105005376	0	0.66076837	0	1	0	11.6484502	11.9242693	11.8036434	11.5492548
3730	**ENSG0000011036**	DDX6	protein_coding	chr11	118747763	118791164	-	Human GRCh	OTTHUMG000001	DDX6	**DEAD-box helicase 6**	Nucleus	enzyme		1.027402004	0	0.89780554	0	1	0	12.8127523	12.971079	13.1023522	12.7152239
3731	**ENSG0000011037**	UPK2	protein_coding	chr11	118925164	118958559	+	Human GRCh	OTTHUMG000001	UPK2	**uroplakin 2**	Plasma Mem	other		-1.130983931	0	0.88228018	0	1	0	3.91531575	4.34781912	3.89467617	4.16157707
3732	**ENSG0000011039**	CBL	protein_coding	chr11	119206298	119313926	+	Human GRCh	OTTHUMG000001	CBL	**Cbl proto-oncogene**	Nucleus	enzyme		-1.10762807	0	0.60213866	0	1	0	11.2052862	11.4615886	11.3361056	11.1103926
3733	**ENSG0000011040**	NECTIN1	protein_coding	chr11	119623408	119729200	-	Human GRCh	OTTHUMG000001	NECTIN1	**nectin cell adhesion molecule 1**	Plasma Mem	other		1.043795837	0	0.81657949	0	1	0	9.64096962	9.86834652	9.82354177	9.78596068
3734	**ENSG0000011042**	HIPK3	protein_coding	chr11	33256672	33357023	+	Human GRCh	OTTHUMG000001	HIPK3	**homeodomain interacting protein kinase 3**	Nucleus	kinase		-1.083640263	0	0.72766348	0	1	0	11.5743424	11.9200193	11.8334418	11.4800494
3735	**ENSG0000011042**	KIAA1549L	protein_coding	chr11	33376108	33674102	+	Human GRCh	OTTHUMG000001	KIAA1549L	**KIAA1549 like**	Cytoplasm	other		-2.648138036	-1	0.23819253	0	1	0	3.14496138	3.64991292	2.41362463	2.94435751
3736	**ENSG0000011042**	FBXO3	protein_coding	chr11	33740939	33774543	-	Human GRCh	OTTHUMG000001	FBXO3	**F-box protein 3**	Cytoplasm	enzyme		1.029434009	0	0.8972759	0	1	0	10.017451	10.2267704	10.237059	10.0746035
3737	**ENSG0000011043**	PDHX	protein_coding	chr11	34915829	35020591	+	Human GRCh	OTTHUMG000001	PDHX	**pyruvate dehydrogenase complex component X**	Cytoplasm	enzyme		-1.03754981	0	0.88335044	0	1	0	11.3935346	11.6868884	11.7132733	11.2576499
3738	**ENSG0000011043**	SLC1A2	protein_coding	chr11	35251205	35420063	-	Human GRCh	OTTHUMG000000	SLC1A2	**solute carrier family 1 member 2**	Plasma Mem	transporter	riluzole	1.309169128	0	0.69568312	0	1	0	6.80899824	6.8798016	7.38352518	6.87032477
3739	**ENSG0000011044**	COMMD9	protein_coding	chr11	36269284	36289449	-	Human GRCh	OTTHUMG000001	COMMD9	**COMM domain containing 9**	Cytoplasm	other		-1.15770193	0	0.56058972	0	1	0	9.73783224	10.0631755	9.8799567	9.59894339
3740	**ENSG0000011044**	SLC15A3	protein_coding	chr11	60937060	60952653	-	Human GRCh	OTTHUMG000001	SLC15A3	**solute carrier family 15 member 3**	Cytoplasm	transporter		-5.293350144	-1	0.24082736	0	1	0	2.11127523	1.64033962	1.63271272	1.59668243
3742	**ENSG0000011045**	ACCS	protein_coding	chr11	44065925	44084237	+	Human GRCh	OTTHUMG000001	ACCS	**1-aminocyclopropane-1-carboxylate synthase homolog (inacti**	Other	other		-1.022712041	0	0.93084323	0	1	0	8.53256409	8.98085195	9.00036848	8.44054843
3744	**ENSG0000011049**	MDK	protein_coding	chr11	46380756	46383837	+	Human GRCh	OTTHUMG000001	MDK	**midkine**	Extracellular	growth facto		1.194707138	0	0.44983886	0	1	0	10.2815333	10.4226391	10.4217708	10.6101246
3745	**ENSG0000011049**	AMBRA1	protein_coding	chr11	46396414	46594125	-	Human GRCh	OTTHUMG000001	AMBRA1	**autophagy and beclin 1 regulator 1**	Cytoplasm	other		-1.069791048	0	0.73850608	0	1	0	11.0594454	11.1860494	11.2064889	10.8696957
3746	**ENSG0000011051**	MADD	protein_coding	chr11	47269161	47330031	+	Human GRCh	OTTHUMG000001	MADD	**MAP kinase activating death domain**	Cytoplasm	other		-1.135524302	0	0.55886767	0	1	0	10.8585093	11.1123107	11.0225729	10.6567081
3747	**ENSG0000011053**	PTPMT1	protein_coding	chr11	47565430	47573461	+	Human GRCh	OTTHUMG000001	PTPMT1	**protein tyrosine phosphatase mitochondrial 1**	Cytoplasm	phosphatase		-1.378071748	0	0.21802307	0	1	0	8.55399042	8.68645129	8.41873323	8.10518757
3748	**ENSG0000011058**	NAA40	protein_coding	chr11	63938959	63957319	+	Human GRCh	OTTHUMG000001	NAA40	**N-alpha-acetyltransferase 40, NatD catalytic subunit**	Cytoplasm	enzyme		-1.017532512	0	0.93563277	0	1	0	10.5883001	10.925044	10.9300111	10.5365292
3749	**ENSG0000011061**	CARS1	protein_coding	chr11	3000922	3057613	-	Human GRCh	OTTHUMG000000	CARS1	**cysteinyl-tRNA synthetase 1**	Cytoplasm	enzyme		-1.106511257	0	0.6175646	0	1	0	10.9788911	10.9134483	11.0196987	10.6172003
3750	**ENSG0000011062**	SLC22A18	protein_coding	chr11	2899721	2925246	+	Human GRCh	OTTHUMG000000	SLC22A18	**solute carrier family 22 member 18**	Plasma Mem	transporter		-1.030217978	0	0.92177922	0	1	0	7.12544946	7.45226635	6.85421063	7.56817059
3751	**ENSG0000011065**	CD81	protein_coding	chr11	2376177	2397802	+	Human GRCh	OTTHUMG000000	CD81	**CD81 molecule**	Plasma Mem	other		1.037144848	0	0.87649457	0	1	0	11.2057451	11.5023625	11.4897097	11.3143912
3752	**ENSG0000011066**	SLC35F2	protein_coding	chr11	107790991	107928293	-	Human GRCh	OTTHUMG000001	SLC35F2	**solute carrier family 35 member F2**	Other	other		-1.175360897	0	0.45091068	0	1	0	10.577048	10.71802	10.5324549	10.3951058
3754	**ENSG0000011067**	ELMOD1	protein_coding	chr11	107591091	107666779	+	Human GRCh	OTTHUMG000001	ELMOD1	**ELMO domain containing 1**	Other	other		-11.82087394	-1	0.10649568	0	NA	0	1.2340089	2.12680564	1.25688552	1.2356155
3755	**ENSG0000011068**	CALCA	protein_coding	chr11	14966622	14972354	-	Human GRCh	OTTHUMG000001	CALCA	**calcitonin related polypeptide alpha**	Plasma Mem	other	eptinezum	1	0	1	0	1	0	1.46524596	1.58177602	1.56324432	1.47239458
3756	**ENSG0000011069**	SOX6	protein_coding	chr11	15966449	16739591	-	Human GRCh	OTTHUMG000001	SOX6	**SRY-box transcription factor 6**	Nucleus	transcription		1.029611694	0	0.93099817	0	1	0	7.50397282	7.74565173	7.58133316	7.73143349
3757	**ENSG0000011069**	C11orf58	protein_coding	chr11	16613132	16758340	+	Human GRCh	OTTHUMG000001	C11orf58	**chromosome 11 open reading frame 58**	Other	other		-1.01464787	0	0.93922176	0	1	0	11.4242007	11.6980963	11.7041028	11.3797623
3758	**ENSG0000011069**	PITPNM1	protein_coding	chr11	67491768	67506263	-	Human GRCh	OTTHUMG000001	PITPNM1	**phosphatidylinositol transfer protein membrane associated 1**	Cytoplasm	transporter		1.116932984	0	0.58975487	0	1	0	11.344537	11.4267754	11.6354737	11.374557
3759	**ENSG0000011070**	RPS13	protein_coding	chr11	17074388	17077715	-	Human GRCh	OTTHUMG000001	RPS13	**ribosomal protein S13**	Cytoplasm	other		1.001380963	0	0.99494273	0	1	0	12.5034041	12.8248855	12.80086	12.5376669
3760	**ENSG0000011071**	AIP	protein_coding	chr11	67468174	67491154	+	Human GRCh	OTTHUMG000001	AIP	**aryl hydrocarbon receptor interacting protein**	Nucleus	transcription		1.210063155	0	0.35089779	0	1	0	8.54716005	8.74544762	8.87806121	8.82983861
3761	**ENSG0000011071**	NUP98	protein_coding	chr11	3671083	3797792	-	Human GRCh	OTTHUMG000000	NUP98	**nucleoporin 98 and 96 precursor**	Nucleus	transporter		-1.168082468	0	0.46802788	0	1	0	12.87178	13.1948757	12.9316704	12.8041131
3762	**ENSG0000011071**	NDUFS8	protein_coding	chr11	68030617	68036644	+	Human GRCh	OTTHUMG000001	NDUFS8	**NADH:ubiquinone oxidoreductase core subunit S8**	Cytoplasm	enzyme		-1.03197257	0	0.89297255	0	1	0	11.1562403	11.4708989	11.3192023	11.2573387
3763	**ENSG0000011071**	TCIRG1	protein_coding	chr11	68039025	68050895	+	Human GRCh	OTTHUMG000001	TCIRG1	**T cell immune regulator 1, ATPase H+ transporting V0 subunit**	Plasma Mem	enzyme		1.264601448	0	0.24281138	0	1	0	8.67413803	8.91320145	9.0376488	9.04224919
3764	**ENSG0000011072**	CHKA	protein_coding	chr11	68052859	68121444	-	Human GRCh	OTTHUMG000001	CHKA	**choline kinase alpha**	Cytoplasm	kinase	TCD-717, N	-1.010878918	0	0.96490029	0	1	0	10.4706148	10.8035258	10.7135566	10.5543322
3765	**ENSG0000011072**	EXPH5	protein_coding	chr11	108505435	108593768	-	Human GRCh	OTTHUMG000001	EXPH5	**exophilin 5**	Cytoplasm	other		-1.142044229	0	0.57017271	0	1	0	11.168972	11.5794988	11.3208344	11.1627803
3766	**ENSG0000011075**	HPS5	protein_coding	chr11	18278668	18322198	-	Human GRCh	OTTHUMG000001	HPS5	**HPS5 biogenesis of lysosomal organelles complex 2 subunit 2**	Cytoplasm	other		1.098552703	0	0.66221353	0	1	0	9.43388341	9.73657409	9.80681368	9.57681983

3767	**ENSG0000011076**	GTF2H1	protein_coding	chr11	18322295	18367045	+	Human GRCh	OTTHUMG000001	GTF2H1	**general transcription factor IIH subunit 1**	Nucleus	transcription		-1.059169244	0	0.80066968	0	1	0	10.2503311	10.5679365	10.5140005	10.1740203
3768	**ENSG0000011077**	POU2AF1	protein_coding	chr11	111352255	111455630	-	Human GRCh	OTTHUMG000001	POU2AF1	**POU class 2 homeobox associating factor 1**	Nucleus	transcription		-5.495307611	-1	0.17165328	0	1	0	2.73227128	3.35985572	2.82089558	2.73866335
3770	**ENSG0000011079**	VWF	protein_coding	chr12	5948877	6124770	-	Human GRCh	OTTHUMG000001	VWF	**von Willebrand factor**	Extracellular	other	susoctoco|	-1.047410168	0	0.85767857	0	1	0	9.27003577	9.07919138	9.32936715	8.87903323
3771	**ENSG0000011080**	PSMD9	protein_coding	chr12	121888732	121918297	+	Human GRCh	OTTHUMG000001	PSMD9	**proteasome 26S subunit, non-ATPase 9**	Cytoplasm	transcription		-1.081460974	0	0.71021718	0	1	0	10.2718428	10.4287741	10.3510403	10.1729539
3772	**ENSG0000011081**	P3H3	protein_coding	chr12	6828407	6839847	+	Human GRCh	OTTHUMG000001	P3H3	**prolyl 3-hydroxylase 3**	Nucleus	enzyme		1.286948764	0	0.31438187	0	1	0	9.03809701	9.01584483	9.48175927	9.10055563
3773	**ENSG0000011084**	PPFIBP1	protein_coding	chr12	27523431	27695564	+	Human GRCh	OTTHUMG000001	PPFIBP1	**PPFIA binding protein 1**	Plasma Mem	other		-1.142398659	0	0.58517178	0	1	0	12.0016104	12.2385724	12.1951731	11.6886502
3774	**ENSG0000011084**	PRPF40B	protein_coding	chr12	49568218	49644666	+	Human GRCh	OTTHUMG000001	PRPF40B	**pre-mRNA processing factor 40 homolog B**	Nucleus	other		1.030190437	0	0.89102201	0	1	0	9.78310242	9.89789435	9.99249102	9.74329207
3776	**ENSG0000011085**	PRDM4	protein_coding	chr12	107732871	107761272	-	Human GRCh	OTTHUMG000001	PRDM4	**PR/SET domain 4**	Nucleus	transcription		-1.116800953	0	0.55393225	0	1	0	10.0168041	10.1999835	10.076235	9.89936096
3777	**ENSG0000011085**	CLEC2B	protein_coding	chr12	9852369	9869386	-	Human GRCh	OTTHUMG000001	CLEC2B	**C-type lectin domain family 2 member B**	Plasma Mem	other		3.164064242	1	0.62798277	0	NA	0	0.37344637	0.05275778	0.42104713	0.60148072
3778	**ENSG0000011087**	COQ5	protein_coding	chr12	120503279	120534434	-	Human GRCh	OTTHUMG000001	COQ5	**coenzyme Q5, methyltransferase**	Cytoplasm	enzyme		-1.247252339	0	0.3008823	0	1	0	9.10369661	9.35709031	9.15846995	8.81083129
3780	**ENSG0000011088**	CORO1C	protein_coding	chr12	108645109	108731526	-	Human GRCh	OTTHUMG000001	CORO1C	**coronin 1C**	Cytoplasm	other		-1.100437776	0	0.64922911	0	1	0	10.9463385	11.2740268	11.1324776	10.8902982
3781	**ENSG0000011088**	ASIC1	protein_coding	chr12	50057548	50083611	+	Human GRCh	OTTHUMG000001	ASIC1	**acid sensing ion channel subunit 1**	Plasma Mem	ion channel	diclofenac,	1.026374318	0	0.92918474	0	1	0	8.66201959	8.82612535	8.82579423	8.72522308
3783	**ENSG0000011088**	CAPRIN2	protein_coding	chr12	30709552	30823302	-	Human GRCh	OTTHUMG000001	CAPRIN2	**caprin family member 2**	Cytoplasm	other		1.101551136	0	0.73645706	0	1	0	8.88157138	9.02602233	9.2211456	8.870653
3784	**ENSG0000011090**	TSPAN11	protein_coding	chr12	30926428	30996602	+	Human GRCh	OTTHUMG000001	TSPAN11	**tetraspanin 11**	Other	other		1	0	1	0	1	0	0.76043446	0.86132415	0.84531071	0.76663659
3785	**ENSG0000011090**	KCTD10	protein_coding	chr12	109448655	109477359	-	Human GRCh	OTTHUMG000001	KCTD10	**potassium channel tetramerization domain containing 10**	Nucleus	ion channel		-1.102929779	0	0.63268358	0	1	0	10.5513681	10.9377895	10.7830778	10.5032425
3786	**ENSG0000011091**	SLC11A2	protein_coding	chr12	50979401	51028566	-	Human GRCh	OTTHUMG000001	SLC11A2	**solute carrier family 11 member 2**	Plasma Mem	transporter		-1.231608948	0	0.35217286	0	1	0	11.5576847	11.8417475	11.6372476	11.2836743
3787	**ENSG0000011091**	MLEC	protein_coding	chr12	120687149	120701859	+	Human GRCh	OTTHUMG000001	MLEC	**malectin**	Plasma Mem	other		-1.051957469	0	0.79748916	0	1	0	12.9356358	13.1548159	13.1415221	12.8237136
3788	**ENSG0000011092**	MVK	protein_coding	chr12	109573255	109598125	+	Human GRCh	OTTHUMG000001	MVK	**mevalonate kinase**	Cytoplasm	kinase		1.010727018	0	0.96705363	0	1	0	10.2079449	10.8165793	10.6564353	10.4612437
3789	**ENSG0000011092**	CSRNP2	protein_coding	chr12	51061205	51083664	-	Human GRCh	OTTHUMG000001	CSRNP2	**cysteine and serine rich nuclear protein 2**	Nucleus	transcription		-1.046193229	0	0.83977185	0	1	0	11.2960149	11.4812438	11.6099866	10.9941206
3790	**ENSG0000011093**	CAMKK2	protein_coding	chr12	121237675	121298308	-	Human GRCh	OTTHUMG000001	CAMKK2	**calcium/calmodulin dependent protein kinase kinase 2**	Cytoplasm	kinase		1.027350975	0	0.91712068	0	1	0	11.6195183	11.9944241	12.0655203	11.5921586
3791	**ENSG0000011093**	BIN2	protein_coding	chr12	51281038	51324668	-	Human GRCh	OTTHUMG000001	BIN2	**bridging integrator 2**	Plasma Mem	other		-1.209581298	0	0.97078674	0	NA	0	-0.0341094	0.45929456	-0.0254997	0.39556348
3792	**ENSG0000011094**	IL23A	protein_coding	chr12	56334174	56340410	+	Human GRCh	OTTHUMG000001	IL23A	**interleukin 23 subunit alpha**	Extracellular	cytokine	tildrakizum	1.722137725	1	0.4097837	0	1	0	3.60937678	3.68061611	4.07005164	3.99097256
3793	**ENSG0000011095**	ATP5F1B	protein_coding	chr12	56638175	56645984	-	Human GRCh	OTTHUMG000001	ATP5F1B	**ATP synthase F1 subunit beta**	Cytoplasm	transporter		-1.065131533	0	0.79117644	0	1	0	14.2172439	14.6366304	14.5084353	14.2216115
3794	**ENSG0000011095**	PTGES3	protein_coding	chr12	56663341	56688408	-	Human GRCh	OTTHUMG000001	PTGES3	**prostaglandin E synthase 3**	Cytoplasm	enzyme		1.096969355	0	0.6413664	0	1	0	12.500482	12.7838453	12.9459805	12.5195067
3795	**ENSG0000011097**	SYT10	protein_coding	chr12	33374238	33439819	-	Human GRCh	OTTHUMG000001	SYT10	**synaptotagmin 10**	Cytoplasm	transporter		1.019525059	0	0.97716697	0	1	0	8.26132913	8.18349119	8.6035783	7.74206426
3796	**ENSG0000011098**	BCL7A	protein_coding	chr12	122019422	122062044	+	Human GRCh	OTTHUMG000001	BCL7A	**BAF chromatin remodeling complex subunit BCL7A**	Nucleus	other		-1.098223608	0	0.62541021	0	1	0	10.636245	10.7231499	10.6121002	10.5371674
3797	**ENSG0000011101**	RSRC2	protein_coding	chr12	122503454	122527000	-	Human GRCh	OTTHUMG000001	RSRC2	**arginine and serine rich coiled-coil 2**	Other	other		1.098000376	0	0.64390353	0	1	0	11.0945564	11.2467121	11.4148495	11.1141641
3798	**ENSG0000011101**	CYP27B1	protein_coding	chr12	57762334	57768986	-	Human GRCh	OTTHUMG000001	CYP27B1	**cytochrome P450 family 27 subfamily B member 1**	Cytoplasm	enzyme		-1.114025698	0	0.70597056	0	1	0	7.41082645	7.73204354	7.61646652	7.29354728
3801	**ENSG0000011105**	LIN7A	protein_coding	chr12	80792520	80937934	-	Human GRCh	OTTHUMG000001	LIN7A	**lin-7 homolog A, crumbs cell polarity complex component**	Plasma Mem	other		-1.0272222	0	0.93937275	0	1	0	7.1853181	7.12271132	6.9973249	7.24306014
3802	**ENSG0000011105**	KRT18	protein_coding	chr12	52948871	52952906	+	Human GRCh	OTTHUMG000001	KRT18	**keratin 18**	Cytoplasm	other		1.016362447	0	0.9490531	0	1	0	12.148911	12.4438737	12.3360063	12.3111172
3803	**ENSG0000011105**	ACSS3	protein_coding	chr12	80936414	81261210	+	Human GRCh	OTTHUMG000001	ACSS3	**acyl-CoA synthetase short chain family member 3**	Cytoplasm	enzyme		-2.245816093	-1	0.33408448	0	1	0	3.61156143	3.1043107	3.08009785	2.60158009
3804	**ENSG0000011107**	TNS2	protein_coding	chr12	53046969	53064379	+	Human GRCh	OTTHUMG000001	TNS2	**tensin 2**	Plasma Mem	enzyme		1.041545032	0	0.89841553	0	1	0	8.0979904	8.09732997	7.86574989	8.35631028
3805	**ENSG0000011108**	GLI1	protein_coding	chr12	57459785	57472268	+	Human GRCh	OTTHUMG000001	GLI1	**GLI family zinc finger 1**	Nucleus	transcription		5.371293732	1	0.05154208	0	1	0	2.57155709	2.12607669	3.56520789	3.3093781
3806	**ENSG0000011111**	PPM1H	protein_coding	chr12	62643994	62935150	-	Human GRCh	OTTHUMG000001	PPM1H	**protein phosphatase, Mg2+/Mn2+ dependent 1H**	Nucleus	phosphatase		-1.234673786	0	0.33604718	0	1	0	11.1614141	11.2215419	11.0299035	10.8787105
3807	**ENSG0000011114**	METAP2	protein_coding	chr12	95473520	95515839	+	Human GRCh	OTTHUMG000001	METAP2	**methionyl aminopeptidase 2**	Cytoplasm	peptidase	APL-1202,	1.072409363	0	0.72645612	0	1	0	10.5834396	10.9441353	10.9640991	10.7346038
3808	**ENSG0000011114**	LTA4H	protein_coding	chr12	96000753	96043520	-	Human GRCh	OTTHUMG000001	LTA4H	**leukotriene A4 hydrolase**	Cytoplasm	enzyme		1.076727707	0	0.76449627	0	1	0	10.0631919	10.3635313	10.4992633	10.0688002
3809	**ENSG0000011114**	ELK3	protein_coding	chr12	96194375	96269824	+	Human GRCh	OTTHUMG000001	ELK3	**ETS transcription factor ELK3**	Nucleus	transcription		1.066841722	0	0.85394106	0	1	0	7.03633441	6.97281458	6.83548926	7.26459853
3810	**ENSG0000011118**	SLC6A12	protein_coding	chr12	190077	214570	-	Human GRCh	OTTHUMG000000	SLC6A12	**solute carrier family 6 member 12**	Plasma Mem	transporter	tiagabine	-1.366892329	0	0.69428464	0	1	0	3.24348706	3.39581314	3.02253458	3.17936742
3811	**ENSG0000011118**	WNT5B	protein_coding	chr12	1529891	1647212	+	Human GRCh	OTTHUMG000000	WNT5B	**Wnt family member 5B**	Extracellular	other		2.422920078	1	0.37501251	0	NA	0	2.01609377	2.71117964	3.07522632	2.36726874
3812	**ENSG0000011119**	MAGOHB	protein_coding	chr12	10604193	10613609	-	Human GRCh	OTTHUMG000001	MAGOHB	**mago homolog B, exon junction complex subunit**	Nucleus	other		1.084603517	0	0.74480484	0	1	0	9.62507502	9.95704594	10.1176726	9.61350284
3813	**ENSG0000011119**	TRPV4	protein_coding	chr12	109783087	109833406	-	Human GRCh	OTTHUMG000001	TRPV4	**transient receptor potential cation channel subfamily V memb**	Plasma Mem	ion channel	GSK27987	1.747688627	1	0.3962696	0	1	0	3.43289757	3.99982487	3.57416313	4.65192548
3814	**ENSG0000011120**	ITFG2	protein_coding	chr12	2812622	2859791	+	Human GRCh	OTTHUMG000001	ITFG2	**integrin alpha FG-GAP repeat containing 2**	Cytoplasm	other		1.132257595	0	0.61762872	0	1	0	9.17985273	9.40881865	9.60349851	9.23770122
3815	**ENSG0000011120**	FOXM1	protein_coding	chr12	2857680	2877174	-	Human GRCh	OTTHUMG000001	FOXM1	**forkhead box M1**	Nucleus	transcription		-1.313841368	0	0.15809507	0	1	0	12.1792284	12.546932	12.1432788	11.9933704
3816	**ENSG0000011121**	PRR4	protein_coding	chr12	10845849	10849475	-	Human GRCh	OTTHUMG000001	PRR4	**proline rich 4**	Extracellular	other		-1.514404946	-1	0.37169894	0	1	0	5.63354129	5.62028209	5.36145741	5.0904911
3817	**ENSG0000011121**	PRMT8	protein_coding	chr12	3381349	3593973	+	Human GRCh	OTTHUMG000001	PRMT8	**protein arginine methyltransferase 8**	Nucleus	enzyme		1	0	1	0	1	0	1.87975829	1.99246345	1.9744646	1.88663939
3818	**ENSG0000011122**	PARP11	protein_coding	chr12	3791047	3873448	-	Human GRCh	OTTHUMG000001	PARP11	**poly(ADP-ribose) polymerase family member 11**	Nucleus	enzyme		73.9846976	1	6.56E-05	1	0.11	0	2.90640036	2.99018017	4.85496449	4.15674203
3819	**ENSG0000011122**	ARPC3	protein_coding	chr12	110434823	110450422	-	Human GRCh	OTTHUMG000001	ARPC3	**actin related protein 2/3 complex subunit 3**	Cytoplasm	other		-1.002028379	0	0.99278017	0	1	0	9.9807169	10.3441681	10.2423913	10.1012479
3820	**ENSG0000011123**	GPN3	protein_coding	chr12	110452486	110469268	-	Human GRCh	OTTHUMG000001	GPN3	**GPN-loop GTPase 3**	Cytoplasm	other		-1.133766156	0	0.57681904	0	1	0	8.78643096	9.1116844	8.98250039	8.6337906
3821	**ENSG0000011123**	VPS29	protein_coding	chr12	110491083	110502111	-	Human GRCh	OTTHUMG000001	VPS29	**VPS29 retromer complex component**	Cytoplasm	transporter		-1.061775072	0	0.78300957	0	1	0	10.3348992	10.6695969	10.5920627	10.2833723
3824	**ENSG0000011124**	RAD51AP1	protein_coding	chr12	4538798	4560047	+	Human GRCh	OTTHUMG000001	RAD51AP1	**RAD51 associated protein 1**	Nucleus	other		1.039641236	0	0.86804794	0	1	0	9.36990506	9.66286262	9.68292061	9.4398389
3825	**ENSG0000011124**	CUX2	protein_coding	chr12	111034165	111350554	+	Human GRCh	OTTHUMG000001	CUX2	**cut like homeobox 2**	Nucleus	transcription		-1.133005979	0	0.70180812	0	1	0	10.6461984	10.6767856	10.6312228	10.3723726
3826	**ENSG0000011125**	SH2B3	protein_coding	chr12	111405923	111451623	+	Human GRCh	OTTHUMG000001	SH2B3	**SH2B adaptor protein 3**	Plasma Mem	other		-1.085588684	0	0.83197681	0	1	0	8.5540815	8.57611477	8.52327858	8.42051223
3827	**ENSG0000011125**	AKAP3	protein_coding	chr12	4615508	4649051	-	Human GRCh	OTTHUMG000001	AKAP3	**A-kinase anchoring protein 3**	Cytoplasm	other		-1.174212997	0	0.7729003	0	1	0	4.72461852	4.12490141	4.58160983	4.03595617
3828	**ENSG0000011126**	MANSC1	protein_coding	chr12	12326056	12350242	-	Human GRCh	OTTHUMG000001	MANSC1	**MANSC domain containing 1**	Other	other		-1.019939893	0	0.948799	0	1	0	8.30699267	8.44525231	8.39955333	8.31217729
3829	**ENSG0000011126**	KCNA1	protein_coding	chr12	4909905	4918256	+	Human GRCh	OTTHUMG000000	KCNA1	**potassium voltage-gated channel subfamily A member 1**	Plasma Mem	ion channel	dalfampric	1	0	1	0	NA	0	0.11032699	0.126221	0.12360732	0.11126458
3830	**ENSG0000011126**	DUSP16	protein_coding	chr12	12473282	12562863	-	Human GRCh	OTTHUMG000001	DUSP16	**dual specificity phosphatase 16**	Nucleus	phosphatase		-1.006209186	0	0.98190205	0	1	0	9.69991485	9.57239415	9.51542141	9.73642038
3831	**ENSG0000011126**	CREBL2	protein_coding	chr12	12611827	12645108	+	Human GRCh	OTTHUMG000001	CREBL2	**cAMP responsive element binding protein like 2**	Nucleus	transcription		1.053932553	0	0.8339505	0	1	0	9.25684017	9.41465295	9.55552798	9.21058674
3832	**ENSG0000011127**	ACAD10	protein_coding	chr12	111686053	111757107	+	Human GRCh	OTTHUMG000001	ACAD10	**acyl-CoA dehydrogenase family member 10**	Cytoplasm	enzyme		-1.207990479	0	0.43252927	0	1	0	10.0071139	10.192039	10.1046895	9.64666939
3833	**ENSG0000011127**	ALDH2	protein_coding	chr12	111766887	111817532	+	Human GRCh	OTTHUMG000001	ALDH2	**aldehyde dehydrogenase 2 family member**	Cytoplasm	enzyme	disulfiram/	-1.365730963	0	0.52947489	0	1	0	6.56099915	6.22005649	5.80470296	6.31520126
3834	**ENSG0000011127**	CDKN1B	protein_coding	chr12	12685498	12722369	+	Human GRCh	OTTHUMG000001	CDKN1B	**cyclin dependent kinase inhibitor 1B**	Nucleus	other		-1.109142556	0	0.63712376	0	1	0	11.0379281	11.2940845	11.106623	11.0157987
3836	**ENSG0000011130**	NAA25	protein_coding	chr12	112026689	112108796	-	Human GRCh	OTTHUMG000001	NAA25	**N-alpha-acetyltransferase 25, NatB auxiliary subunit**	Cytoplasm	other		-1.056569048	0	0.77588872	0	1	0	11.8122272	11.9796121	11.9936988	11.6571846
3838	**ENSG0000011131**	SCNN1A	protein_coding	chr12	6346843	6377730	-	Human GRCh	OTTHUMG000001	SCNN1A	**sodium channel epithelial 1 subunit alpha**	Plasma Mem	ion channel	hydrochlo	1.056286097	0	0.85241031	0	1	0	11.4804031	12.0663454	11.7409578	11.9838145
3839	**ENSG0000011132**	LTBR	protein_coding	chr12	6375045	6391571	+	Human GRCh	OTTHUMG000001	LTBR	**lymphotoxin beta receptor**	Plasma Mem	transmembra		-1.098214267	0	0.61493351	0	1	0	11.2958432	11.47162	11.3255348	11.2337288
3840	**ENSG0000011132**	OGFOD2	protein_coding	chr12	122974580	122980043	+	Human GRCh	OTTHUMG000001	OGFOD2	**2-oxoglutarate and iron dependent oxygenase domain contain**	Other	other		-1.069433175	0	0.78339477	0	1	0	9.02258544	9.59217327	9.33026182	9.2013179
3841	**ENSG0000011132**	CDK2AP1	protein_coding	chr12	123250112	123272334	-	Human GRCh	OTTHUMG000001	CDK2AP1	**cyclin dependent kinase 2 associated protein 1**	Nucleus	other		-1.233902075	0	0.36461493	0	1	0	11.0365575	11.393369	11.0905508	10.8859583
3842	**ENSG0000011133**	OAS3	protein_coding	chr12	112938051	112976460	+	Human GRCh	OTTHUMG000001	OAS3	**2'-5'-oligoadenylate synthetase 3**	Cytoplasm	enzyme		1.597988561	1	0.17056844	0	1	0	6.51667145	6.33532532	7.09061101	6.45190609
3843	**ENSG0000011133**	OAS2	protein_coding	chr12	112978395	113011723	+	Human GRCh	OTTHUMG000001	OAS2	**2'-5'-oligoadenylate synthetase 2**	Cytoplasm	enzyme		1	0	1	0	NA	0	0.92776613	0.94657534	0.94344765	0.9288618
3846	**ENSG0000011134**	RASAL1	protein_coding	chr12	113098819	113136239	-	Human GRCh	OTTHUMG000001	RASAL1	**RAS protein activator like 1**	Cytoplasm	other		1.282457182	0	0.49237808	0	1	0	6.61104003	6.45334666	6.44601855	6.99233643
3848	**ENSG0000011135**	GTF2H3	protein_coding	chr12	123633739	123662604	+	Human GRCh	OTTHUMG000001	GTF2H3	**general transcription factor IIH subunit 3**	Nucleus	transcription		1.082092121	0	0.70877359	0	1	0	9.67356248	9.93195846	10.1133546	9.63004223
3849	**ENSG0000011136**	EIF2B1	protein_coding	chr12	123620406	123633766	-	Human GRCh	OTTHUMG000001	EIF2B1	**eukaryotic translation initiation factor 2B subunit alpha**	Cytoplasm	translation re		-1.107172979	0	0.6257068	0	1	0	10.5590771	10.983529	10.7227476	10.6271628
3850	**ENSG0000011136**	DDX55	protein_coding	chr12	123602077	123620943	+	Human GRCh	OTTHUMG000001	DDX55	**DEAD-box helicase 55**	Nucleus	enzyme		1.054385647	0	0.78931652	0	1	0	10.118357	10.4360656	10.4682102	10.2086426
3851	**ENSG0000011137**	SLC38A1	protein_coding	chr12	46183063	46270017	-	Human GRCh	OTTHUMG000001	SLC38A1	**solute carrier family 38 member 1**	Plasma Mem	transporter		-1.191733625	0	0.42688527	0	1	0	13.2473487	13.3002717	13.1626129	12.9936053
3852	**ENSG0000011140**	RERGL	protein_coding	chr12	18080869	18320107	-	Human GRCh	OTTHUMG000001	RERGL	**RERG like**	Other	other		1	0	1	0	1	0	0.70926277	0.81151764	0.795302	0.71555521
3853	**ENSG0000011140**	ENDOU	protein_coding	chr12	47709734	47725567	-	Human GRCh	OTTHUMG000001	ENDOU	**endonuclease, poly(U) specific**	Cytoplasm	peptidase		2.110412742	1	0.6498426	0	NA	0	1.2915389	1.13176047	1.4814975	1.1231773
3854	**ENSG0000011141**	SPRING1	protein_coding	chr12	116710171	116738070	-	Human GRCh	OTTHUMG000001	SPRING1	**SREBF pathway regulator in golgi 1**	Cytoplasm	other		-1.06519466	0	0.77416545	0	1	0	10.5418237	10.755843	10.6359774	10.5291728
3855	**ENSG0000011142**	VDR	protein_coding	chr12	47841537	47943048	-	Human GRCh	OTTHUMG000001	VDR	**vitamin D receptor**	Nucleus	transcription	cholecalcif	1.602610203	1	0.04656015	1	1	0	7.14938016	7.36081861	7.51685906	7.95544824
3857	**ENSG0000011144**	RFC5	protein_coding	chr12	118013588	118033130	+	Human GRCh	OTTHUMG000001	RFC5	**replication factor C subunit 5**	Nucleus	enzyme		-1.145710677	0	0.51800235	0	1	0	10.2583911	10.6948709	10.4738833	10.2088065
3858	**ENSG0000011145**	STX2	protein_coding	chr12	130789600	130839254	-	Human GRCh	OTTHUMG000001	STX2	**syntaxin 2**	Cytoplasm	transporter		-1.036795041	0	0.90756719	0	1	0	8.06011891	8.13311547	8.20628254	7.89269562
3859	**ENSG0000011145**	ADGRD1	protein_coding	chr12	130953907	131141469	+	Human GRCh	OTTHUMG000001	ADGRD1	**adhesion G protein-coupled receptor D1**	Plasma Mem	G-protein cou		1.046407397	0	0.86804298	0	1	0	7.85155033	7.92564067	8.09178537	7.77116115
3860	**ENSG0000011148**	COPZ1	protein_coding	chr12	54301202	54351846	+	Human GRCh	OTTHUMG000001	COPZ1	**COPI coat complex subunit zeta 1**	Cytoplasm	transporter		-1.154388932	0	0.46263426	0	1	0	11.8492886	12.0860074	11.907486	11.7118681
3861	**ENSG0000011149**	TBC1D30	protein_coding	chr12	64759484	64881033	+	Human GRCh	OTTHUMG000001	TBC1D30	**TBC1 domain family member 30**	Cytoplasm	other		1.271698711	0	0.42440479	0	1	0	6.18252708	6.61810635	6.82688331	6.41867901
3862	**ENSG0000011153**	CAND1	protein_coding	chr12	67269358	67319953	+	Human GRCh	OTTHUMG000001	CAND1	**cullin associated and neddylation dissociated 1**	Cytoplasm	transcription		-1.229637001	0	0.30276364	0	1	0	12.4982173	12.837468	12.603392	12.2783562
3865	**ENSG0000011154**	RAB5B	protein_coding	chr12	55973913	55996683	+	Human GRCh	OTTHUMG000001	RAB5B	**RAB5B, member RAS oncogene family**	Cytoplasm	enzyme		-1.180579423	0	0.42231967	0	1	0	11.8932312	12.2222005	11.9507783	11.815895

3866	ENSG0000011155	MDM1	protein_coding	chr12	68272443	68332381	-	Human GRCh	OTTHUMG000001	MDM1	Mdm1 nuclear protein	Cytoplasm	other		-1.091899251	0	0.684989	0	1	0	8.91846198	9.29900483	9.17093989	8.87196138
3867	ENSG0000011158	NUP107	protein_coding	chr12	68686951	68745809	+	Human GRCh	OTTHUMG000001	NUP107	nucleoporin 107	Nucleus	other		-1.099001345	0	0.65893612	0	1	0	10.9361565	11.3150049	11.1944458	10.8574905
3868	ENSG0000011159	CNOT2	protein_coding	chr12	70243002	70354993	+	Human GRCh	OTTHUMG000001	CNOT2	CCR4-NOT transcription complex subunit 2	Nucleus	transcription		1.000715553	0	0.99734407	0	1	0	11.0351295	11.2311105	11.3124984	10.935495
3869	ENSG0000011160	TIMELESS	protein_coding	chr12	56416363	56449426	-	Human GRCh	OTTHUMG000001	TIMELESS	timeless circadian regulator	Nucleus	other		-1.272816957	0	0.24474611	0	1	0	11.3277156	11.4784178	11.3393894	10.9200369
3870	ENSG0000011160	CPSF6	protein_coding	chr12	69239569	69274358	+	Human GRCh	OTTHUMG000001	CPSF6	cleavage and polyadenylation specific factor 6	Nucleus	other		-1.004550408	0	0.98239975	0	1	0	12.255783	12.465823	12.5015893	12.2019079
3871	ENSG0000011161	KRR1	protein_coding	chr12	75490863	75511636	-	Human GRCh	OTTHUMG000001	KRR1	KRR1 small subunit processome component homolog	Nucleus	other		-1.029010086	0	0.90246383	0	1	0	10.6535732	10.9886732	10.9749171	10.5984289
3872	ENSG0000011163	MRPL51	protein_coding	chr12	6491886	6493841	-	Human GRCh	OTTHUMG000001	MRPL51	mitochondrial ribosomal protein L51	Cytoplasm	other		1.038886392	0	0.83374872	0	1	0	10.1315583	10.4986208	10.428167	10.3123517
3873	ENSG0000011164	GAPDH	protein_coding	chr12	6534512	6538374	+	Human GRCh	OTTHUMG000001	GAPDH	glyceraldehyde-3-phosphate dehydrogenase	Cytoplasm	enzyme		-1.020498221	0	0.92853892	0	1	0	15.6853935	16.1602073	15.9250931	15.920661
3874	ENSG0000011164	NOP2	protein_coding	chr12	6556863	6568691	-	Human GRCh	OTTHUMG000001	NOP2	NOP2 nucleolar protein	Nucleus	other		-1.044678048	0	0.82716074	0	1	0	10.872395	11.2959733	11.1625078	10.9328356
3875	ENSG0000011164	CHD4	protein_coding	chr12	6570082	6614524	-	Human GRCh	OTTHUMG000001	CHD4	chromodomain helicase DNA binding protein 4	Nucleus	enzyme		-1.084461154	0	0.68016091	0	1	0	13.1477838	13.3908845	13.2917519	13.0699861
3876	ENSG0000011164	ACRBP	protein_coding	chr12	6638075	6647433	-	Human GRCh	OTTHUMG000001	ACRBP	acrosin binding protein	Extracellular	other		-1.445612114	0	0.27023565	0	1	0	6.38544808	6.06347286	6.03081475	5.69652725
3877	ENSG0000011164	BLTP3B	protein_coding	chr12	100028455	100142874	-	Human GRCh	OTTHUMG000001	BLTP3B	bridge-like lipid transfer protein family member 3B	Cytoplasm	transporter		-1.170430887	0	0.43708812	0	1	0	11.3321094	11.6343986	11.4489588	11.1641225
3878	ENSG0000011165	COPS7A	protein_coding	chr12	6724014	6731875	+	Human GRCh	OTTHUMG000001	COPS7A	COP9 signalosome subunit 7A	Cytoplasm	other		1.02822029	0	0.89340918	0	1	0	9.82328158	10.1101782	10.0983684	9.90691392
3879	ENSG0000011165	ING4	protein_coding	chr12	6650301	6663142	-	Human GRCh	OTTHUMG000001	ING4	inhibitor of growth family member 4	Nucleus	transcription		-1.055259718	0	0.82146704	0	1	0	8.82550441	8.93192161	8.93801368	8.6928673
3880	ENSG0000011166	GNB3	protein_coding	chr12	6839954	6847393	+	Human GRCh	OTTHUMG000001	GNB3	G protein subunit beta 3	Plasma Mem	other		1.191021627	0	0.59812811	0	1	0	8.68630222	8.66377406	8.98616186	8.74056614
3881	ENSG0000011166	CDCA3	protein_coding	chr12	6844793	6851292	-	Human GRCh	OTTHUMG000001	CDCA3	cell division cycle associated 3	Cytoplasm	other		-1.264566249	0	0.26994391	0	1	0	10.4530581	10.6837841	10.3909161	10.2321757
3882	ENSG0000011166	CHPT1	protein_coding	chr12	101696947	101744140	+	Human GRCh	OTTHUMG000001	CHPT1	choline phosphotransferase 1	Cytoplasm	enzyme		-1.044796742	0	0.85289897	0	1	0	11.6089445	11.7945061	11.8889927	11.3639713
3883	ENSG0000011166	USP5	protein_coding	chr12	6852148	6866632	+	Human GRCh	OTTHUMG000001	USP5	ubiquitin specific peptidase 5	Cytoplasm	peptidase		-1.082038135	0	0.68777174	0	1	0	11.570155	11.9309468	11.7733206	11.5672871
3884	ENSG0000011166	TPI1	protein_coding	chr12	6867119	6870948	+	Human GRCh	OTTHUMG000001	TPI1	triosephosphate isomerase 1	Cytoplasm	enzyme		-1.078715229	0	0.73162692	0	1	0	13.7800037	14.1298735	13.9875679	13.7636706
3885	ENSG0000011167	GNPTAB	protein_coding	chr12	101745499	101830959	-	Human GRCh	OTTHUMG000001	GNPTAB	N-acetylglucosamine-1-phosphate transferase subunits alpha a	Cytoplasm	enzyme		-1.138296293	0	0.5539928	0	1	0	10.7929507	11.174746	11.0536894	10.6177339
3886	ENSG0000011167	SPSB2	protein_coding	chr12	6870935	6889358	-	Human GRCh	OTTHUMG000001	SPSB2	splA/ryanodine receptor domain and SOCS box containing 2	Cytoplasm	other		1.114574109	0	0.70342307	0	1	0	7.18759692	7.15218329	7.47624063	7.05722977
3887	ENSG0000011167	ENO2	protein_coding	chr12	6913745	6923698	+	Human GRCh	OTTHUMG000001	ENO2	enolase 2	Cytoplasm	enzyme		1.081564775	0	0.75959462	0	1	0	10.6125741	10.9005723	10.953565	10.739514
3888	ENSG0000011167	ATN1	protein_coding	chr12	6924463	6942321	+	Human GRCh	OTTHUMG000001	ATN1	atrophin 1	Nucleus	transcription		1.031618442	0	0.88410731	0	1	0	12.9526828	13.2076026	13.2061419	13.0316262
3889	ENSG0000011167	C12orf57	protein_coding	chr12	6942978	6946003	+	Human GRCh	OTTHUMG000001	C12orf57	chromosome 12 open reading frame 57	Cytoplasm	other		-1.154291229	0	0.51250259	0	1	0	10.8537709	11.1483945	10.9632579	10.7181872
3890	ENSG0000011167	PTPN6	protein_coding	chr12	6946468	6961316	+	Human GRCh	OTTHUMG000001	PTPN6	protein tyrosine phosphatase non-receptor type 6	Cytoplasm	phosphatase	SC-43	-1.001713358	0	0.99367117	0	1	0	8.83276399	9.00635842	9.01638803	8.81684986
3891	ENSG0000011168	LPCAT3	protein_coding	chr12	6976185	7018477	-	Human GRCh	OTTHUMG000001	LPCAT3	lysophosphatidylcholine acyltransferase 3	Plasma Mem	enzyme		1.022309142	0	0.92010403	0	1	0	8.65042658	9.14449908	8.8990337	8.99190163
3892	ENSG0000011169	NT5DC3	protein_coding	chr12	103770453	103841234	-	Human GRCh	OTTHUMG000001	NT5DC3	5'-nucleotidase domain containing 3	Cytoplasm	other		-1.183270383	0	0.49927993	0	1	0	12.0511933	12.3336339	12.2724852	11.6732599
3893	ENSG0000011170	SLCO1B3	protein_coding	chr12	20810702	20916911	+	Human GRCh	OTTHUMG000001	SLCO1B3	solute carrier organic anion transporter family member 1B3	Plasma Mem	transporter		2.90111398	1	0.40625677	0	NA	0	1.05426163	2.03420188	2.0144747	2.44828078
3896	ENSG0000011170	SUDS3	protein_coding	chr12	118376555	118418033	+	Human GRCh	OTTHUMG000001	SUDS3	SDS3 homolog, SIN3A corepressor complex component	Nucleus	transcription		-1.118611085	0	0.59535395	0	1	0	11.1076714	11.3577456	11.2906342	10.9145309
3897	ENSG0000011171	GOLT1B	protein_coding	chr12	21501781	21518408	+	Human GRCh	OTTHUMG000001	GOLT1B	golgi transport 1B	Cytoplasm	other		1.0181702	0	0.93706297	0	1	0	9.55714782	9.72738042	9.89923861	9.3725143
3898	ENSG0000011171	GYS2	protein_coding	chr12	21536107	21604847	-	Human GRCh	OTTHUMG000001	GYS2	glycogen synthase 2	Cytoplasm	enzyme		-1.689645047	-1	0.66407633	0	NA	0	2.64265091	2.53835538	2.32038636	2.14833281
3899	ENSG0000011171	LDHB	protein_coding	chr12	21635342	21757857	-	Human GRCh	OTTHUMG000001	LDHB	lactate dehydrogenase B	Cytoplasm	enzyme		-1.118531262	0	0.64641965	0	1	0	13.0238199	13.5406576	13.3468378	12.9730019
3900	ENSG0000011172	PRKAB1	protein_coding	chr12	119667864	119681624	+	Human GRCh	OTTHUMG000001	PRKAB1	protein kinase AMP-activated non-catalytic subunit beta 1	Nucleus	kinase		1.006904576	0	0.97461939	0	1	0	9.30690878	9.39078457	9.49330302	9.20241482
3901	ENSG0000011172	CMAS	protein_coding	chr12	22046218	22065674	+	Human GRCh	OTTHUMG000001	CMAS	cytidine monophosphate N-acetylneuraminic acid synthetase	Nucleus	enzyme		-1.055355566	0	0.81412798	0	1	0	9.74836638	10.1659562	10.0947298	9.70400633
3902	ENSG0000011172	HCFC2	protein_coding	chr12	104064531	104106524	+	Human GRCh	OTTHUMG000001	HCFC2	host cell factor C2	Nucleus	transcription		-1.167999932	0	0.51099555	0	1	0	8.65387418	8.9677747	8.79630758	8.49805626
3903	ENSG0000011172	ST8SIA1	protein_coding	chr12	22063773	22437041	-	Human GRCh	OTTHUMG000001	ST8SIA1	ST8 alpha-N-acetyl-neuraminide alpha-2,8-sialyltransferase 1	Cytoplasm	enzyme		1.277193336	0	0.7565257	0	1	0	7.55382198	7.9388455	8.38511641	7.59817602
3904	ENSG0000011172	CLEC4A	protein_coding	chr12	8123617	8138607	+	Human GRCh	OTTHUMG000001	CLEC4A	C-type lectin domain family 4 member A	Plasma Mem	transmembra		1.028467669	0	0.96509705	0	1	0	3.31522824	4.32695077	4.04124556	3.87243243
3905	ENSG0000011173	C2CD5	protein_coding	chr12	22448583	22544546	-	Human GRCh	OTTHUMG000001	C2CD5	C2 calcium dependent domain containing 5	Cytoplasm	other		-1.162857645	0	0.49608293	0	1	0	10.9986812	11.3334091	11.2179093	10.7665909
3907	ENSG0000011173	RAB35	protein_coding	chr12	120095099	120117502	-	Human GRCh	OTTHUMG000001	RAB35	RAB35, member RAS oncogene family	Cytoplasm	enzyme		-1.070562078	0	0.7256619	0	1	0	10.5659376	10.8738325	10.7625345	10.5324165
3908	ENSG0000011175	PHC1	protein_coding	chr12	8913896	8941467	+	Human GRCh	OTTHUMG000001	PHC1	polyhomeotic homolog 1	Nucleus	transcription		-1.090693144	0	0.70205944	0	1	0	8.285113	8.39170406	8.24215124	8.25266496
3909	ENSG0000011177	COX6A1	protein_coding	chr12	120438090	120440737	+	Human GRCh	OTTHUMG000000	COX6A1	cytochrome c oxidase subunit 6A1	Cytoplasm	enzyme		-1.056421621	0	0.80497217	0	1	0	11.9645606	12.283548	12.1318882	12.0104432
3910	ENSG0000011178	RFX4	protein_coding	chr12	106583004	106762803	+	Human GRCh	OTTHUMG000001	RFX4	regulatory factor X4	Nucleus	transcription		-1.558721836	-1	0.5580449	0	1	0	2.85655482	2.34300994	2.65103269	1.72561052
3911	ENSG0000011178	RIC8B	protein_coding	chr12	106774621	106889316	+	Human GRCh	OTTHUMG000001	RIC8B	RIC8 guanine nucleotide exchange factor B	Cytoplasm	other		-1.004449267	0	0.98466798	0	1	0	10.6378296	10.9056226	10.9857115	10.5167215
3912	ENSG0000011178	SRSF9	protein_coding	chr12	120461672	120495946	-	Human GRCh	OTTHUMG000000	SRSF9	serine and arginine rich splicing factor 9	Nucleus	enzyme		-1.015507506	0	0.93717778	0	1	0	11.4380847	11.7347898	11.7185502	11.4195598
3913	ENSG0000011179	FGFR1OP2	protein_coding	chr12	26938470	26966650	+	Human GRCh	OTTHUMG000001	FGFR1OP2	FGFR1 oncogene partner 2	Cytoplasm	other		1.190085786	0	0.46019462	0	1	0	9.37769383	9.49177927	9.87309791	9.31148245
3915	ENSG0000011179	COL12A1	protein_coding	chr6	75084326	75206267	-	Human GRCh	OTTHUMG000000	COL12A1	collagen type XII alpha 1 chain	Extracellular	other	collagenas	1.499914419	0	0.19340957	0	1	0	8.36078804	7.88019507	8.64469791	8.51208263
3916	ENSG0000011180	BTN3A3	protein_coding	chr6	26440472	26453415	+	Human GRCh	OTTHUMG000000	BTN3A3	butyrophilin subfamily 3 member A3	Plasma Mem	other		-2.10212585	-1	0.08317115	0	1	0	5.63391236	6.57864173	5.62198623	5.35787846
3917	ENSG0000011180	TDP2	protein_coding	chr6	24649979	24666930	-	Human GRCh	OTTHUMG000000	TDP2	tyrosyl-DNA phosphodiesterase 2	Cytoplasm	transcription		-1.038895515	0	0.85198943	0	1	0	9.46387973	9.75845827	9.73238611	9.40144337
3918	ENSG0000011181	FRK	protein_coding	chr6	115931149	116060891	-	Human GRCh	OTTHUMG000000	FRK	fyn related Src family tyrosine kinase	Nucleus	kinase	regorafeni	-1.012991124	0	0.96442847	0	1	0	10.3830616	10.6611756	10.6582737	10.3535079
3919	ENSG0000011181	DSE	protein_coding	chr6	116254173	116444861	+	Human GRCh	OTTHUMG000000	DSE	dermatan sulfate epimerase	Cytoplasm	enzyme		-1.163717433	0	0.56336051	0	1	0	9.27764789	9.43205784	9.41585518	8.91792325
3920	ENSG0000011183	RWDD1	protein_coding	chr6	116571409	116597675	+	Human GRCh	OTTHUMG000000	RWDD1	RWD domain containing 1	Cytoplasm	other		-1.041155221	0	0.85504056	0	1	0	9.75047563	10.0833449	9.95432568	9.80902749
3921	ENSG0000011183	RSPH4A	protein_coding	chr6	116616479	116632985	+	Human GRCh	OTTHUMG000000	RSPH4A	radial spoke head component 4A	Nucleus	other		-2.25456403	-1	0.04820333	1	1	0	5.79003586	6.28385058	5.31017677	5.27433946
3922	ENSG0000011183	MAK	protein_coding	chr6	10762723	10838553	-	Human GRCh	OTTHUMG000000	MAK	male germ cell associated kinase	Cytoplasm	kinase		1.392895546	0	0.59669638	0	1	0	5.62338027	6.06808223	6.54262393	5.60607915
3923	ENSG0000011184	TMEM14C	protein_coding	chr6	10722915	10731129	+	Human GRCh	OTTHUMG000000	TMEM14C	transmembrane protein 14C	Plasma Mem	other		1.076408661	0	0.76410107	0	1	0	9.76156119	10.032344	10.1986062	9.72680117
3924	ENSG0000011184	PAK1IP1	protein_coding	chr6	10694972	10709782	+	Human GRCh	OTTHUMG000000	PAK1IP1	PAK1 interacting protein 1	Nucleus	other		1.091226041	0	0.66707799	0	1	0	8.95192142	9.20940178	9.30204852	9.05274716
3925	ENSG0000011184	GCNT2	protein_coding	chr6	10492223	10629368	+	Human GRCh	OTTHUMG000000	GCNT2	glucosaminyl (N-acetyl) transferase 2 (I blood group)	Cytoplasm	enzyme		-1.476926899	0	0.42537001	0	1	0	3.24844929	4.58957352	3.37694891	4.04922936
3926	ENSG0000011185	SMIM8	protein_coding	chr6	87322583	87399749	+	Human GRCh	OTTHUMG000000	SMIM8	small integral membrane protein 8	Other	other		1.090825022	0	0.74416512	0	1	0	8.5957802	8.98087403	9.09519989	8.66057358
3927	ENSG0000011185	NEDD9	protein_coding	chr6	11183298	11382348	-	Human GRCh	OTTHUMG000000	NEDD9	neural precursor cell expressed, developmentally down-regula	Nucleus	other		1.163579431	0	0.81978183	0	1	0	4.89319168	4.55256135	4.59685336	5.12574682
3928	ENSG0000011186	CEP85L	protein_coding	chr6	118460772	118710075	-	Human GRCh	OTTHUMG000000	CEP85L	centrosomal protein 85 like	Cytoplasm	other		1.122331623	0	0.58385939	0	1	0	9.06442121	9.231755	9.38285593	9.1599312
3930	ENSG0000011187	ASF1A	protein_coding	chr6	118894152	118909171	+	Human GRCh	OTTHUMG000000	ASF1A	anti-silencing function 1A histone chaperone	Nucleus	other		-1.022488406	0	0.92466076	0	1	0	9.55069717	9.83566365	9.88357912	9.42684591
3931	ENSG0000011187	MCM9	protein_coding	chr6	118813442	118935162	-	Human GRCh	OTTHUMG000000	MCM9	minichromosome maintenance 9 homologous recombination r	Nucleus	enzyme		-1.115062022	0	0.63268175	0	1	0	9.44431913	9.7302111	9.64904302	9.28351412
3932	ENSG0000011187	FAM184A	protein_coding	chr6	118959763	119149387	-	Human GRCh	OTTHUMG000000	FAM184A	family with sequence similarity 184 member A	Extracellular	other		-1.000911621	0	0.99680333	0	1	0	8.48903676	8.99560096	8.97870276	8.51150833
3933	ENSG0000011188	RNGTT	protein_coding	chr6	88609897	88963618	-	Human GRCh	OTTHUMG000000	RNGTT	RNA guanylyltransferase and 5'-phosphatase	Nucleus	phosphatase		-1.005308877	0	0.97855482	0	1	0	9.9253244	10.0679707	10.2028616	9.73761711
3934	ENSG0000011188	MAN1A1	protein_coding	chr6	119177205	119349761	-	Human GRCh	OTTHUMG000000	MAN1A1	mannosidase alpha class 1A member 1	Cytoplasm	enzyme		2.380746397	1	0.02054126	1	1	0	6.1235429	5.99485973	6.75190993	7.08875721
3935	ENSG0000011188	GABRR2	protein_coding	chr6	89254464	89315299	-	Human GRCh	OTTHUMG000000	GABRR2	gamma-aminobutyric acid type A receptor subunit rho2	Plasma Mem	ion channel	triazolam,	-1.366696431	0	0.56187232	0	1	0	4.93618692	5.22242753	4.77240367	4.82606809
3936	ENSG0000011189	SERINC1	protein_coding	chr6	122443351	122471807	-	Human GRCh	OTTHUMG000000	SERINC1	serine incorporator 1	Plasma Mem	transporter		-1.016260321	0	0.93766265	0	1	0	11.591196	11.8048297	11.865683	11.4712333
3937	ENSG0000011190	HDDC2	protein_coding	chr6	125219962	125302078	-	Human GRCh	OTTHUMG000000	HDDC2	HD domain containing 2	Cytoplasm	other		-1.055104901	0	0.81083761	0	1	0	9.25872009	9.6400224	9.55628373	9.23015873
3938	ENSG0000011190	TPD52L1	protein_coding	chr6	125119049	125264407	+	Human GRCh	OTTHUMG000000	TPD52L1	TPD52 like 1	Cytoplasm	other		1.108821765	0	0.76000906	0	1	0	9.67951054	10.2340047	10.1788514	10.0317716
3939	ENSG0000011191	HINT3	protein_coding	chr6	125956770	125980244	+	Human GRCh	OTTHUMG000000	HINT3	histidine triad nucleotide binding protein 3	Cytoplasm	enzyme		1.290931044	0	0.25331856	0	1	0	9.8338634	9.97816613	10.3919878	9.94375757
3940	ENSG0000011191	NCOA7	protein_coding	chr6	125781161	125932034	+	Human GRCh	OTTHUMG000000	NCOA7	nuclear receptor coactivator 7	Nucleus	transcription		-1.146486867	0	0.62377084	0	1	0	9.09571584	8.83856303	8.98144937	8.66331326
3941	ENSG0000011191	RIPOR2	protein_coding	chr6	24804282	25042170	-	Human GRCh	OTTHUMG000001	RIPOR2	RHO family interacting cell polarization regulator 2	Cytoplasm	other		-1.791142413	-1	0.4476009	0	1	0	5.02073787	5.29154519	4.56370905	4.8341076
3942	ENSG0000011196	SASH1	protein_coding	chr6	148272304	148552044	+	Human GRCh	OTTHUMG000000	SASH1	SAM and SH3 domain containing 1	Extracellular	other		-1.601259906	-1	0.1073502	0	1	0	12.2047571	12.4391354	11.9483441	11.6105011
3943	ENSG0000011196	UST	protein_coding	chr6	148747030	149076990	+	Human GRCh	OTTHUMG000000	UST	uronyl 2-sulfotransferase	Cytoplasm	enzyme		1.037962872	0	0.92953619	0	1	0	6.50555265	5.78884509	5.95139652	6.47258924
3944	ENSG0000011198	ULBP1	protein_coding	chr6	149963943	149973715	+	Human GRCh	OTTHUMG000000	ULBP1	UL16 binding protein 1	Plasma Mem	transmembra		-1.200834845	0	0.55834444	0	1	0	6.06306006	5.9155932	5.84413303	5.77156508
3945	ENSG0000011202	FBXO5	protein_coding	chr6	152970519	152983579	-	Human GRCh	OTTHUMG000000	FBXO5	F-box protein 5	Nucleus	enzyme		-1.20771388	0	0.48189481	0	1	0	9.42867428	9.7953512	9.47137063	9.37666679
3946	ENSG0000011203	MTRF1L	protein_coding	chr6	152987362	153002709	-	Human GRCh	OTTHUMG000000	MTRF1L	mitochondrial translation release factor 1 like	Cytoplasm	translation re		-1.144781043	0	0.52668206	0	1	0	8.67076872	8.71046783	8.60356376	8.4795986
3947	ENSG0000011203	PPARD	protein_coding	chr6	35342558	35428191	+	Human GRCh	OTTHUMG000000	PPARD	peroxisome proliferator activated receptor delta	Nucleus	ligand-depen	treprostini	1.170555604	0	0.47694631	0	1	0	9.5348063	9.90370821	9.99700273	9.81082969
3949	ENSG0000011203	FANCE	protein_coding	chr6	35452338	35467104	+	Human GRCh	OTTHUMG000000	FANCE	FA complementation group E	Nucleus	other		1.028412148	0	0.90013739	0	1	0	8.8004552	9.01045656	9.14213481	8.69950837
3952	ENSG0000011206	MAPK14	protein_coding	chr6	36027782	36111236	+	Human GRCh	OTTHUMG000001	MAPK14	mitogen-activated protein kinase 14	Cytoplasm	kinase	pamapimo	-1.084798674	0	0.70155595	0	1	0	10.8399177	11.2378081	11.1292159	10.7786017
3954	ENSG0000011207	KCTD20	protein_coding	chr6	36442767	36491143	+	Human GRCh	OTTHUMG000000	KCTD20	potassium channel tetramerization domain containing 20	Cytoplasm	other		-1.13720095	0	0.51891721	0	1	0	11.1945153	11.5177017	11.3494033	11.0864964
3955	ENSG0000011207	STK38	protein_coding	chr6	36493892	36547479	-	Human GRCh	OTTHUMG000000	STK38	serine/threonine kinase 38	Nucleus	kinase		1.027491401	0	0.88142197	0	1	0	10.0200223	10.2621787	10.1937506	10.1576001
3956	ENSG0000011208	SRSF3	protein_coding	chr6	36594353	36605600	+	Human GRCh	OTTHUMG000000	SRSF3	serine and arginine rich splicing factor 3	Nucleus	other		-1.065832431	0	0.73697643	0	1	0	12.6625494	12.8942815	12.8675455	12.5380186
3957	ENSG0000011211	MRPL18	protein_coding	chr6	159789812	159798436	+	Human GRCh	OTTHUMG000000	MRPL18	mitochondrial ribosomal protein L18	Cytoplasm	translation re		-1.101492347	0	0.63635036	0	1	0	10.1118089	10.5042663	10.2736177	10.1572412

3960	ENSG0000011211	MCM3	protein_coding	chr6	52264014	52284881	-	Human GRCh	OTTHUMG000000	MCM3	minichromosome maintenance complex component 3	Nucleus	enzyme		-1.19320044	0	0.38760717	0	1	0	11.5546859	11.8721152	11.6840419	11.3569855
3961	ENSG0000011213	RNF8	protein_coding	chr6	37353979	37394734	+	Human GRCh	OTTHUMG000000	RNF8	ring finger protein 8	Nucleus	enzyme		1.026516298	0	0.90144712	0	1	0	8.71901764	8.80790691	8.92445955	8.64443193
3962	ENSG0000011213	PHACTR1	protein_coding	chr6	12716312	13290446	+	Human GRCh	OTTHUMG000000	PHACTR1	phosphatase and actin regulator 1	Cytoplasm	other		-1.063256865	0	0.93971405	0	1	0	6.58334066	7.73632014	7.51178544	6.84471223
3963	ENSG0000011213	MDGA1	protein_coding	chr6	37630679	37699306	-	Human GRCh	OTTHUMG000000	MDGA1	MAM domain containing glycosylphosphatidylinositol anchor	Plasma Mem	other		-1.328600119	0	0.68793733	0	1	0	4.49693127	2.98751661	3.75338392	3.87648164
3964	ENSG0000011214	CILK1	protein_coding	chr6	53001279	53061824	-	Human GRCh	OTTHUMG000000	CILK1	ciliogenesis associated kinase 1	Cytoplasm	kinase		-1.127699156	0	0.59284351	0	1	0	10.7065637	11.0015125	10.8499775	10.597545
3965	ENSG0000011214	FBXO9	protein_coding	chr6	53051991	53100873	+	Human GRCh	OTTHUMG000000	FBXO9	F-box protein 9	Cytoplasm	enzyme		-1.025962274	0	0.90398239	0	1	0	10.5163972	10.7897396	10.8085834	10.4258542
3966	ENSG0000011214	CD83	protein_coding	chr6	14117256	14136918	+	Human GRCh	OTTHUMG000000	CD83	CD83 molecule	Plasma Mem	transmembra		1.845654	1	0.15751536	0	1	0	3.56624348	4.47538061	4.3012538	4.78064353
3967	ENSG0000011215	MDN1	protein_coding	chr6	89642498	89819794	-	Human GRCh	OTTHUMG000000	MDN1	midasin AAA ATPase 1	Nucleus	other		-1.090122477	0	0.67072875	0	1	0	13.077717	13.3982399	13.3210633	12.9558913
3968	ENSG0000011216	GLP1R	protein_coding	chr6	39048781	39091303	+	Human GRCh	OTTHUMG000000	GLP1R	glucagon like peptide 1 receptor	Plasma Mem	G-protein cou	68Ga-exen	-10.17341586	-1	0.15740318	0	1	0	2.29897672	1.23109003	1.21751704	1.15144627
3969	ENSG0000011216	SAYSD1	protein_coding	chr6	39104063	39115193	-	Human GRCh	OTTHUMG000000	SAYSD1	SAYSVFN motif domain containing 1	Other	other		-1.100808948	0	0.68108813	0	1	0	8.44271617	8.72681205	8.66047374	8.3015064
3970	ENSG0000011217	BMP5	protein_coding	chr6	55753653	55875590	-	Human GRCh	OTTHUMG000000	BMP5	bone morphogenetic protein 5	Extracellular	growth facto		1.003089528	0	0.99776191	0	1	0	2.66747341	1.83719707	1.82717357	2.6759284
3971	ENSG0000011218	BACH2	protein_coding	chr6	89926528	90296843	-	Human GRCh	OTTHUMG000000	BACH2	BTB domain and CNC homolog 2	Nucleus	transcription		-1.00017991	0	0.99970316	0	1	0	7.02511448	6.96288946	6.91027416	7.07230107
3973	ENSG0000011218	CAP2	protein_coding	chr6	17393595	17557780	+	Human GRCh	OTTHUMG000000	CAP2	cyclase associated actin cytoskeleton regulatory protein 2	Plasma Mem	other		1.244663805	0	0.59449909	0	1	0	7.11333819	7.78023929	7.75397025	7.74752313
3975	ENSG0000011220	ZNF451	protein_coding	chr6	57086844	57170305	+	Human GRCh	OTTHUMG000000	ZNF451	zinc finger protein 451	Nucleus	enzyme		-1.008050103	0	0.96963765	0	1	0	11.9172976	12.2208507	12.2737978	11.8231944
3976	ENSG0000011220	BAG2	protein_coding	chr6	57172326	57189833	+	Human GRCh	OTTHUMG000000	BAG2	BAG cochaperone 2	Cytoplasm	other		1.093998068	0	0.76604283	0	1	0	9.32558039	9.76530157	9.88592805	9.39811793
3977	ENSG0000011221	RAB23	protein_coding	chr6	57186992	57222307	-	Human GRCh	OTTHUMG000000	RAB23	RAB23, member RAS oncogene family	Cytoplasm	enzyme		1.0851919	0	0.70480217	0	1	0	8.50576569	8.83019697	8.80754944	8.72295277
3980	ENSG0000011221	GPR63	protein_coding	chr6	96794125	96837477	-	Human GRCh	OTTHUMG000000	GPR63	G protein-coupled receptor 63	Plasma Mem	G-protein cou		1.249694718	0	0.4666489	0	1	0	6.98935234	7.18373287	7.16324447	7.47377986
3981	ENSG0000011223	KHDRBS2	protein_coding	chr6	61679961	62286225	-	Human GRCh	OTTHUMG000000	KHDRBS2	KH RNA binding domain containing, signal transduction associ	Nucleus	other		1.105450359	0	0.76847668	0	1	0	8.7047924	8.9942559	9.206693	8.70033276
3982	ENSG0000011223	FBXL4	protein_coding	chr6	98868535	98948006	-	Human GRCh	OTTHUMG000000	FBXL4	F-box and leucine rich repeat protein 4	Nucleus	other		-1.038971021	0	0.86474027	0	1	0	9.97959647	10.0609789	10.1336968	9.80384447
3983	ENSG0000011223	CCNC	protein_coding	chr6	99542387	99568825	-	Human GRCh	OTTHUMG000000	CCNC	cyclin C	Nucleus	other		1.039645957	0	0.8599612	0	1	0	10.7141084	11.0793703	11.1594935	10.7037941
3984	ENSG0000011223	PRDM13	protein_coding	chr6	99606774	99615578	+	Human GRCh	OTTHUMG000000	PRDM13	PR/SET domain 13	Nucleus	enzyme		1.530698632	1	0.26690277	0	1	0	5.51883565	5.1682529	5.77603078	5.78808504
3985	ENSG0000011224	E2F3	protein_coding	chr6	20401879	20493714	+	Human GRCh	OTTHUMG000000	E2F3	E2F transcription factor 3	Nucleus	transcription		-1.039522307	0	0.83783844	0	1	0	10.6241566	10.8673887	10.8810944	10.5081345
3986	ENSG0000011224	PTP4A1	protein_coding	chr6	63521746	63583588	+	Human GRCh	OTTHUMG000000	PTP4A1	protein tyrosine phosphatase 4A1	Cytoplasm	phosphatase		2.977412098	1	0.02400682	1	1	0	4.25066089	4.30332925	5.48772037	4.78846573
3987	ENSG0000011224	SIM1	protein_coding	chr6	100385009	100464921	-	Human GRCh	OTTHUMG000000	SIM1	SIM bHLH transcription factor 1	Nucleus	transcription		-5.964791089	-1	0.42046586	0	NA	0	0.53169813	1.00509981	0.5411204	0.53235291
3988	ENSG0000011224	ASCC3	protein_coding	chr6	100508194	100881372	-	Human GRCh	OTTHUMG000000	ASCC3	activating signal cointegrator 1 complex subunit 3	Cytoplasm	enzyme		-1.1179813	0	0.57441221	0	1	0	11.7677439	12.1585074	11.9734673	11.7255653
3990	ENSG0000011227	BVES	protein_coding	chr6	105096822	105137157	-	Human GRCh	OTTHUMG000000	BVES	blood vessel epicardial substance	Plasma Mem	other		1.246148652	0	0.60239376	0	1	0	6.44102041	6.12255572	6.13270757	6.78449444
3991	ENSG0000011228	COL9A1	protein_coding	chr6	70216040	70303084	-	Human GRCh	OTTHUMG000000	COL9A1	collagen type IX alpha 1 chain	Extracellular	other	collagenas	-1.847639777	-1	0.57350799	0	NA	0	2.73814377	2.36318607	2.62527413	1.94292056
3992	ENSG0000011228	MED23	protein_coding	chr6	131573966	131628242	-	Human GRCh	OTTHUMG000000	MED23	mediator complex subunit 23	Nucleus	transcription		-1.162856926	0	0.51590588	0	1	0	10.8951797	11.1527395	11.0285364	10.6713312
3993	ENSG0000011229	WASF1	protein_coding	chr6	110099819	110180004	-	Human GRCh	OTTHUMG000000	WASF1	WASP family member 1	Nucleus	other		1.211606272	0	0.43097656	0	1	0	9.14601986	9.32464747	9.52840947	9.356522
3994	ENSG0000011229	GPLD1	protein_coding	chr6	24424565	24495205	-	Human GRCh	OTTHUMG000000	GPLD1	glycosylphosphatidylinositol specific phospholipase D1	Cytoplasm	enzyme		1.313953333	0	0.39001157	0	1	0	6.78376159	6.91830608	7.38117751	6.83181008
3995	ENSG0000011229	ALDH5A1	protein_coding	chr6	24494867	24537207	+	Human GRCh	OTTHUMG000000	ALDH5A1	aldehyde dehydrogenase 5 family member A1	Cytoplasm	enzyme	theophyllii	1.051180416	0	0.86408941	0	1	0	9.46736968	9.74757539	9.77728799	9.55033098
3996	ENSG0000011229	CRYBG1	protein_coding	chr6	106360717	106572017	+	Human GRCh		CRYBG1	crystallin beta-gamma domain containing 1	Extracellular	other		1.0347125	0	0.90316237	0	1	0	9.73487526	9.86615133	9.92367219	9.75036755
3999	ENSG0000011230	ACOT13	protein_coding	chr6	24667035	24705065	+	Human GRCh	OTTHUMG000000	ACOT13	acyl-CoA thioesterase 13	Cytoplasm	enzyme		1.016327696	0	0.95122543	0	1	0	8.66644684	9.02966487	8.85784003	8.90060737
4000	ENSG0000011230	SMAP1	protein_coding	chr6	70667776	70862011	+	Human GRCh	OTTHUMG000000	SMAP1	small ArfGAP 1	Cytoplasm	other		-1.008784864	0	0.96995065	0	1	0	9.42557849	9.8406432	9.76638921	9.5024879
4001	ENSG0000011230	RPS12	protein_coding	chr6	132814569	132817564	+	Human GRCh	OTTHUMG000000	RPS12	ribosomal protein S12	Cytoplasm	other	neomycin	1.087515684	0	0.70595456	0	1	0	12.0771556	12.3478088	12.4943188	12.1042396
4002	ENSG0000011230	C6orf62	protein_coding	chr6	24704861	24720825	-	Human GRCh	OTTHUMG000000	C6orf62	chromosome 6 open reading frame 62	Other	other		-1.141330821	0	0.52521835	0	1	0	12.0019133	12.2490563	12.0767658	11.8900873
4003	ENSG0000011230	B3GAT2	protein_coding	chr6	70856679	70957060	-	Human GRCh	OTTHUMG000000	B3GAT2	beta-1,3-glucuronyltransferase 2	Cytoplasm	enzyme		4.308098302	1	0.15312683	0	1	0	2.72006782	2.33644639	3.55425812	2.28999595
4004	ENSG0000011231	GMNN	protein_coding	chr6	24774931	24786099	+	Human GRCh	OTTHUMG000000	GMNN	geminin DNA replication inhibitor	Nucleus	transcription		-1.120216553	0	0.53805517	0	1	0	9.77224812	10.0305937	9.93646162	9.61114497
4005	ENSG0000011231	EYA4	protein_coding	chr6	133240514	133532128	+	Human GRCh	OTTHUMG000000	EYA4	EYA transcriptional coactivator and phosphatase 4	Cytoplasm	phosphatase		-12.11214375	-1	0.36090525	0	NA	0	0.57371361	1.48016374	0.59114221	0.57494157
4006	ENSG0000011232	SOBP	protein_coding	chr6	107490106	107661306	+	Human GRCh	OTTHUMG000000	SOBP	sine oculis binding protein homolog	Nucleus	other		1.296877379	0	0.53430303	0	1	0	5.26838997	5.16669215	5.49320481	5.35441775
4008	ENSG0000011233	SNX3	protein_coding	chr6	108211222	108261246	-	Human GRCh	OTTHUMG000000	SNX3	sorting nexin 3	Cytoplasm	transporter		-1.186459929	0	0.37845905	0	1	0	10.4412446	10.7205097	10.4636852	10.3333879
4010	ENSG0000011233	HBS1L	protein_coding	chr6	134960378	135103056	-	Human GRCh	OTTHUMG000000	HBS1L	HBS1 like translational GTPase	Cytoplasm	translation re		-1.204353122	0	0.34351343	0	1	0	11.5513995	11.8284846	11.6161219	11.34962
4011	ENSG0000011234	TRIM38	protein_coding	chr6	25962802	25991231	+	Human GRCh	OTTHUMG000000	TRIM38	tripartite motif containing 38	Cytoplasm	enzyme		1	0	1	0	1	0	2.65959527	2.75783809	2.74198423	2.66552128
4012	ENSG0000011235	PEX7	protein_coding	chr6	136822564	136913934	+	Human GRCh	OTTHUMG000000	PEX7	peroxisomal biogenesis factor 7	Cytoplasm	transporter		1.155559933	0	0.56493658	0	1	0	7.77222507	7.79454649	8.11946953	7.72208036
4013	ENSG0000011236	ZBTB24	protein_coding	chr6	109460632	109483239	-	Human GRCh	OTTHUMG000000	ZBTB24	zinc finger and BTB domain containing 24	Nucleus	transcription		-1.089337455	0	0.71122663	0	1	0	9.68019992	9.88505188	9.82860437	9.54382219
4014	ENSG0000011236	FIG4	protein_coding	chr6	109690609	109878098	+	Human GRCh	OTTHUMG000000	FIG4	FIG4 phosphoinositide 5-phosphatase	Cytoplasm	enzyme		-1.139461953	0	0.53376214	0	1	0	9.88190216	10.0747122	10.0066269	9.65108726
4015	ENSG0000011237	PERP	protein_coding	chr6	138088505	138107419	-	Human GRCh	OTTHUMG000000	PERP	p53 apoptosis effector related to PMP22	Plasma Mem	other		-1.02525422	0	0.93746058	0	1	0	11.8704395	12.1660363	12.1478084	11.8301526
4016	ENSG0000011237	ARFGEF3	protein_coding	chr6	138161939	138344663	+	Human GRCh	OTTHUMG000000	ARFGEF3	ARFGEF family member 3	Other	other		-1.145362593	0	0.62833209	0	1	0	13.5165159	13.8827188	13.7146767	13.3788491
4017	ENSG0000011239	SLC16A10	protein_coding	chr6	111087503	111231194	+	Human GRCh	OTTHUMG000000	SLC16A10	solute carrier family 16 member 10	Plasma Mem	transporter		1.017047259	0	0.94963127	0	1	0	8.82155615	8.91551855	9.0368666	8.71795517
4018	ENSG0000011240	HECA	protein_coding	chr6	139135080	139180802	+	Human GRCh	OTTHUMG000000	HECA	hdc homolog, cell cycle regulator	Nucleus	other		-1.208395912	0	0.43173756	0	1	0	10.5508228	10.9202181	10.6313325	10.4395628
4019	ENSG0000011241	ADGRG6	protein_coding	chr6	142301854	142446266	+	Human GRCh	OTTHUMG000000	ADGRG6	adhesion G protein-coupled receptor G6	Plasma Mem	G-protein cou		-1.051887521	0	0.89506433	0	1	0	8.72696062	8.9754617	8.84612216	8.75096045
4020	ENSG0000011241	PHACTR2	protein_coding	chr6	143536845	143831185	+	Human GRCh	OTTHUMG000000	PHACTR2	phosphatase and actin regulator 2	Other	other		1.058859783	0	0.87725532	0	1	0	9.2802846	9.66091281	9.62026875	9.47152694
4021	ENSG0000011242	EPM2A	protein_coding	chr6	145382535	145736023	-	Human GRCh	OTTHUMG000000	EPM2A	EPM2A glucan phosphatase, laforin	Cytoplasm	phosphatase		-1.203459199	0	0.51410022	0	1	0	7.4464448	8.15190981	7.82625456	7.46579868
4023	ENSG0000011247	SLC39A7	protein_coding	chr6	33200305	33204439	+	Human GRCh	OTTHUMG000000	SLC39A7	solute carrier family 39 member 7	Plasma Mem	transporter		-1.023214162	0	0.9134704	0	1	0	11.6891053	11.9223959	11.8848116	11.6771355
4024	ENSG0000011248	CCR6	protein_coding	chr6	167111807	167139141	+	Human GRCh	OTTHUMG000000	CCR6	C-C motif chemokine receptor 6	Plasma Mem	G-protein cou		1.652832151	1	0.48489243	0	1	0	2.87069045	2.98140357	3.06230188	3.33324697
4027	ENSG0000011251	PHF1	protein_coding	chr6	33410399	33416453	+	Human GRCh	OTTHUMG000000	PHF1	PHD finger protein 1	Nucleus	transcription		1.054015262	0	0.8173302	0	1	0	10.2200573	10.5330307	10.5806642	10.2907989
4028	ENSG0000011251	CUTA	protein_coding	chr6	33416442	33418317	-	Human GRCh	OTTHUMG000000	CUTA	cutA divalent cation tolerance homolog	Cytoplasm	other		-1.049479261	0	0.81104158	0	1	0	10.8669408	11.2150335	11.0436281	10.953161
4030	ENSG0000011253	QKI	protein_coding	chr6	163414000	163578592	+	Human GRCh	OTTHUMG000000	QKI	QKI, KH domain containing RNA binding	Nucleus	other		1.083828399	0	0.71705423	0	1	0	11.1299134	11.2244472	11.3185014	11.2197717
4032	ENSG0000011254	PDE10A	protein_coding	chr6	165327287	165988117	-	Human GRCh	OTTHUMG000000	PDE10A	phosphodiesterase 10A	Cytoplasm	enzyme	aspirin/dip	2.467224324	1	0.18787812	0	1	0	8.06409447	8.48806117	9.21427691	9.53579777
4033	ENSG0000011255	MDFI	protein_coding	chr6	41636882	41654244	+	Human GRCh	OTTHUMG000000	MDFI	MyoD family inhibitor	Cytoplasm	other		-3.147980011	-1	0.17799358	0	1	0	2.98776612	3.103394	2.25107417	2.80376549
4034	ENSG0000011256	TFEB	protein_coding	chr6	41683978	41736259	-	Human GRCh	OTTHUMG000000	TFEB	transcription factor EB	Nucleus	transcription		1.385816356	0	0.19493906	0	1	0	9.2801277	9.71024746	9.80018537	9.96300997
4035	ENSG0000011256	SMOC2	protein_coding	chr6	168441151	168673445	+	Human GRCh	OTTHUMG000000	SMOC2	SPARC related modular calcium binding 2	Extracellular	other		-16.1405219	-1	0.03356698	1	1	0	3.22071978	3.00074001	2.38602168	2.29988677
4036	ENSG0000011257	CCND3	protein_coding	chr6	41934934	42050357	-	Human GRCh	OTTHUMG000000	CCND3	cyclin D3	Nucleus	kinase		1.15155744	0	0.56020016	0	1	0	9.30157745	9.53449994	9.58235012	9.58077028
4037	ENSG0000011257	BYSL	protein_coding	chr6	41921499	41933046	+	Human GRCh	OTTHUMG000000	BYSL	bystin like	Nucleus	other		-1.011667584	0	0.95689339	0	1	0	9.32893157	9.53337274	9.62947573	9.17187853
4038	ENSG0000011258	FAM120B	protein_coding	chr6	170290703	170407067	+	Human GRCh	OTTHUMG000000	FAM120B	family with sequence similarity 120 member B	Nucleus	other		-1.152756358	0	0.48854269	0	1	0	10.1713394	10.4512836	10.2860645	10.0331526
4039	ENSG0000011259	TBP	protein_coding	chr6	170554302	170572870	+	Human GRCh	OTTHUMG000000	TBP	TATA-box binding protein	Nucleus	transcription		-1.208671916	0	0.40001613	0	1	0	9.2828874	9.45504509	9.21699965	9.11004937
4040	ENSG0000011259	GUCA1B	protein_coding	chr6	42183284	42194956	-	Human GRCh	OTTHUMG000000	GUCA1B	guanylate cyclase activator 1B	Cytoplasm	other		1.15269511	0	0.74072008	0	1	0	5.36017314	6.32888101	6.42395992	5.58690101
4041	ENSG0000011261	PRPH2	protein_coding	chr6	42696598	42722597	-	Human GRCh	OTTHUMG000000	PRPH2	peripherin 2	Plasma Mem	transmembra		3.874693265	1	0.02680999	1	1	0	3.31774942	3.44214491	4.00172095	4.49835012
4042	ENSG0000011262	BICRAL	protein_coding	chr6	42782052	42868558	+	Human GRCh	OTTHUMG000000	BICRAL	BICRA like chromatin remodeling complex associated protein	Nucleus	other		-1.186056091	0	0.44161447	0	1	0	10.5407119	10.8430727	10.5651015	10.4622978
4043	ENSG0000011264	PPP2R5D	protein_coding	chr6	42984553	43012342	+	Human GRCh	OTTHUMG000000	PPP2R5D	protein phosphatase 2 regulatory subunit B'delta	Nucleus	phosphatase		-1.123752419	0	0.58327456	0	1	0	11.2636084	11.6827055	11.4689677	11.2398629
4044	ENSG0000011265	MRPL2	protein_coding	chr6	43054029	43059438	-	Human GRCh	OTTHUMG000000	MRPL2	mitochondrial ribosomal protein L2	Extracellular	other		1.027845475	0	0.90175182	0	1	0	9.77600686	10.2740602	10.1431449	10.0167133
4045	ENSG0000011265	PTK7	protein_coding	chr6	43076307	43161719	+	Human GRCh	OTTHUMG000000	PTK7	protein tyrosine kinase 7 (inactive)	Plasma Mem	kinase	cofetuzum	-1.080995005	0	0.73554363	0	1	0	10.4604749	10.5222916	10.569129	10.2241152
4046	ENSG0000011265	SRF	protein_coding	chr6	43171269	43181506	+	Human GRCh	OTTHUMG000000	SRF	serum response factor	Nucleus	transcription		1.060088733	0	0.77575172	0	1	0	10.905495	10.9754625	11.1524408	10.8414994
4047	ENSG0000011265	CUL9	protein_coding	chr6	43182184	43224587	+	Human GRCh	OTTHUMG000000	CUL9	cullin 9	Cytoplasm	enzyme		-1.107986047	0	0.62883676	0	1	0	10.9635456	11.2276082	11.1295852	10.8318419
4048	ENSG0000011266	DNPH1	protein_coding	chr6	43225629	43229481	-	Human GRCh	OTTHUMG000000	DNPH1	2'-deoxynucleoside 5'-phosphate N-hydrolase 1	Nucleus	enzyme		1.379297672	0	0.19460332	0	1	0	9.68630247	9.92791575	10.2666169	10.0778769
4049	ENSG0000011267	DUSP22	protein_coding	chr6	291630	351355	+	Human GRCh	OTTHUMG000000	DUSP22	dual specificity phosphatase 22	Cytoplasm	phosphatase		-1.168406371	0	0.5018905	0	1	0	8.71637989	8.97102217	8.76924297	8.57876958
4050	ENSG0000011268	EXOC2	protein_coding	chr6	485154	693139	-	Human GRCh	OTTHUMG000001	EXOC2	exocyst complex component 2	Cytoplasm	transporter		-1.116540608	0	0.61288606	0	1	0	9.809001	10.1757185	10.0665971	9.67161888
4051	ENSG0000011269	COX7A2	protein_coding	chr6	75237675	75250323	-	Human GRCh	OTTHUMG000000	COX7A2	cytochrome c oxidase subunit 7A2	Cytoplasm	enzyme		-1.110840058	0	0.63461063	0	1	0	10.9915919	11.2659569	11.0723531	10.9619487
4052	ENSG0000011269	TMEM30A	protein_coding	chr6	75252924	75284948	-	Human GRCh	OTTHUMG000000	TMEM30A	transmembrane protein 30A	Cytoplasm	transporter		1.088564483	0	0.71942505	0	1	0	10.4459947	10.7918697	10.9449022	10.4472963
4053	ENSG0000011269	GMDS	protein_coding	chr6	1623806	2245605	-	Human GRCh	OTTHUMG000000	GMDS	GDP-mannose 4,6-dehydratase	Cytoplasm	enzyme		1.069618654	0	0.76291535	0	1	0	9.30208732	9.57795875	9.57488546	9.46766272
4054	ENSG0000011270	SENP6	protein_coding	chr6	75601509	75718281	+	Human GRCh	OTTHUMG000000	SENP6	SUMO specific peptidase 6	Cytoplasm	peptidase		-1.000142829	0	0.99946406	0	1	0	11.7748139	11.8891019	12.0403252	11.5814329
4055	ENSG0000011270	IMPG1	protein_coding	chr6	75921114	76072678	-	Human GRCh	OTTHUMG000000	IMPG1	interphotoreceptor matrix proteoglycan 1	Extracellular	other		-1.428649846	0	0.62921883	0	1	0	4.42683633	4.75889273	4.60129002	3.73809696
4056	ENSG0000011271	VEGFA	protein_coding	chr6	43770184	43786487	+	Human GRCh	OTTHUMG000000	VEGFA	vascular endothelial growth factor A	Extracellular	growth facto	bevacizum	1.203310272	0	0.44401732	0	1	0	12.6423254	12.5518578	12.9067248	12.6902702

4057	**ENSG0000011273**	PRPF4B	protein_coding	chr6	4021267	4064983	+	Human GRCh	OTTHUMG000000	PRPF4B	**pre-mRNA processing factor 4B**	Nucleus	kinase		1.021408169	0	0.92096714	0	1	0	11.5117813	11.8064507	11.9363649	11.381783
4058	**ENSG0000011274**	TTK	protein_coding	chr6	80003887	80042527	+	Human GRCh	OTTHUMG000000	TTK	**TTK protein kinase**	Nucleus	kinase	NMS-0194	-1.323735391	0	0.20399497	0	1	0	10.1888619	10.5874732	10.2052913	9.99560561
4059	**ENSG0000011275**	SLC29A1	protein_coding	chr6	44219553	44234142	+	Human GRCh	OTTHUMG000000	SLC29A1	**solute carrier family 29 member 1 (Augustine blood group)**	Plasma Mem	transporter	ticagrelor,	-1.051548418	0	0.82108131	0	1	0	10.2620921	10.4737112	10.3030131	10.3309408
4060	**ENSG0000011276**	CCN6	protein_coding	chr6	112054075	112069686	+	Human GRCh	OTTHUMG000001	CCN6	**cellular communication network factor 6**	Extracellular	growth facto		1	0	1	0	NA	0	0.50413452	0.51467534	0.51291418	0.50474517
4061	**ENSG0000011276**	BTN2A1	protein_coding	chr6	26457904	26476621	+	Human GRCh	OTTHUMG000000	BTN2A1	**butyrophilin subfamily 2 member A1**	Plasma Mem	other		-1.02155989	0	0.92262759	0	1	0	9.26609665	9.50821139	9.49204255	9.23381613
4062	**ENSG0000011276**	LAMA4	protein_coding	chr6	112107931	112254939	-	Human GRCh	OTTHUMG000000	LAMA4	**laminin subunit alpha 4**	Extracellular	enzyme		1.4862422	0	0.48863518	0	1	0	4.21812498	3.46825314	4.052194	4.35467989
4063	**ENSG0000011277**	TENT5A	protein_coding	chr6	81491439	81752774	-	Human GRCh	OTTHUMG000000	TENT5A	**terminal nucleotidyltransferase 5A**	Cytoplasm	enzyme		1.375691756	0	0.35076197	0	1	0	7.85067737	8.01888729	8.09472159	8.40672198
4064	**ENSG0000011278**	CLIC5	protein_coding	chr6	45880827	46080348	-	Human GRCh	OTTHUMG000000	CLIC5	**chloride intracellular channel 5**	Cytoplasm	ion channel		1.140265164	0	0.87374758	0	1	0	3.30273254	2.4397101	3.22340358	2.85691851
4065	**ENSG0000011278**	FBRSL1	protein_coding	chr12	132489551	132585188	+	Human GRCh	OTTHUMG000001	FBRSL1	**fibrosin like 1**	Nucleus	other		1.126696856	0	0.55705635	0	1	0	12.2519359	12.4883562	12.5772125	12.4375422
4066	**ENSG0000011279**	ENPP5	protein_coding	chr6	46159185	46170980	-	Human GRCh	OTTHUMG000000	ENPP5	**ectonucleotide pyrophosphatase/phosphodiesterase family m**	Extracellular	enzyme		1.06501163	0	0.79711177	0	1	0	7.47762568	8.08532605	7.90364384	7.86712245
4068	**ENSG0000011281**	PRSS16	protein_coding	chr6	27247701	27256624	+	Human GRCh	OTTHUMG000000	PRSS16	**serine protease 16**	Extracellular	peptidase		-1.336739914	0	0.20542188	0	1	0	9.43242363	9.58975342	9.28007951	9.10094304
4070	**ENSG0000011283**	TBX18	protein_coding	chr6	84687351	84764598	-	Human GRCh	OTTHUMG000000	TBX18	**T-box transcription factor 18**	Nucleus	transcription		1.244613816	0	0.78647826	0	1	0	3.43130355	4.39914776	4.02492118	4.41178421
4071	**ENSG0000011285**	ERBIN	protein_coding	chr5	65883128	66082546	+	Human GRCh	OTTHUMG000000	ERBIN	**erbb2 interacting protein**	Cytoplasm	other		-1.076503539	0	0.70404637	0	1	0	12.2259346	12.5300408	12.4199313	12.1793685
4072	**ENSG0000011285**	PCDHB2	protein_coding	chr5	141094606	141098703	+	Human GRCh	OTTHUMG000001	PCDHB2	**protocadherin beta 2**	Plasma Mem	other		-1.038467108	0	0.91675824	0	1	0	9.87865322	10.0388349	10.0971165	9.70961744
4073	**ENSG0000011285**	HARS2	protein_coding	chr5	140691430	140699305	+	Human GRCh	OTTHUMG000001	HARS2	**histidyl-tRNA synthetase 2, mitochondrial**	Cytoplasm	enzyme		1.020918612	0	0.9184852	0	1	0	9.72868555	10.1657317	10.066362	9.90862293
4074	**ENSG0000011287**	NUDT12	protein_coding	chr5	103548855	103562790	-	Human GRCh	OTTHUMG000001	NUDT12	**nudix hydrolase 12**	Cytoplasm	phosphatase		1	0	1	0	1	0	0.66299648	0.75359228	0.7392121	0.6685649
4075	**ENSG0000011287**	CEP72	protein_coding	chr5	612340	667168	+	Human GRCh	OTTHUMG000001	CEP72	**centrosomal protein 72**	Cytoplasm	other		-1.082431652	0	0.72925098	0	1	0	10.1602037	10.543483	10.4830221	10.0386376
4076	**ENSG0000011289**	MAN2A1	protein_coding	chr5	109689927	109869625	+	Human GRCh	OTTHUMG000001	MAN2A1	**mannosidase alpha class 2A member 1**	Cytoplasm	enzyme		-1.024427457	0	0.91310217	0	1	0	10.7972511	10.9558542	11.0345897	10.6362799
4077	**ENSG0000011290**	SEMA5A	protein_coding	chr5	9035033	9546075	-	Human GRCh	OTTHUMG000000	SEMA5A	**semaphorin 5A**	Plasma Mem	transmembra		6.560934221	1	0.0468452	1	1	0	2.83145132	2.58212005	3.26479578	4.74948853
4078	**ENSG0000011293**	C7	protein_coding	chr5	40909492	41020216	+	Human GRCh	OTTHUMG000001	C7	**complement C7**	Extracellular	other		5.525762982	1	0.22508462	0	1	0	1.37276948	1.42227912	2.02613579	1.37571863
4079	**ENSG0000011294**	TENT4A	protein_coding	chr5	6713432	6757044	+	Human GRCh	OTTHUMG000000	TENT4A	**terminal nucleotidyltransferase 4A**	Nucleus	enzyme		-1.095704597	0	0.64879951	0	1	0	11.0108837	11.3188383	11.2019985	10.9296213
4080	**ENSG0000011296**	GHR	protein_coding	chr5	42423439	42721878	+	Human GRCh	OTTHUMG000000	GHR	**growth hormone receptor**	Plasma Mem	transmembra	lonapegso	-1.048602378	0	0.87415122	0	1	0	9.96700253	10.433969	10.0368562	10.2830154
4081	**ENSG0000011297**	HMGCS1	protein_coding	chr5	43287470	43313512	-	Human GRCh	OTTHUMG000001	HMGCS1	**3-hydroxy-3-methylglutaryl-CoA synthase 1**	Cytoplasm	enzyme		-1.087990271	0	0.75758156	0	1	0	12.9329004	13.5671103	13.2878805	13.093463
4082	**ENSG0000011297**	DAP	protein_coding	chr5	10679230	10761234	-	Human GRCh	OTTHUMG000001	DAP	**death associated protein**	Cytoplasm	transcription		-1.070313566	0	0.74614806	0	1	0	10.6416352	10.9093122	10.8119417	10.5935687
4083	**ENSG0000011298**	NME5	protein_coding	chr5	138115175	138139443	-	Human GRCh	OTTHUMG000001	NME5	**NME/NM23 family member 5**	Cytoplasm	kinase		1.011365239	0	0.97496527	0	1	0	6.45645886	6.63027175	6.78727419	6.27399085
4084	**ENSG0000011298**	BRD8	protein_coding	chr5	138139770	138178953	-	Human GRCh	OTTHUMG000001	BRD8	**bromodomain containing 8**	Nucleus	transcription		-1.32439993	0	0.13953596	0	1	0	11.5403762	11.8800988	11.4285545	11.3901499
4085	**ENSG0000011298**	KIF20A	protein_coding	chr5	138178719	138187723	+	Human GRCh	OTTHUMG000001	KIF20A	**kinesin family member 20A**	Cytoplasm	other		-1.217018866	0	0.40655431	0	1	0	10.8134369	11.2059561	10.8045188	10.812349
4086	**ENSG0000011299**	NNT	protein_coding	chr5	43602692	43707405	+	Human GRCh	OTTHUMG000000	NNT	**nicotinamide nucleotide transhydrogenase**	Cytoplasm	enzyme		-1.122056417	0	0.63240072	0	1	0	9.10050716	9.60985824	9.48933838	8.99357401
4087	**ENSG0000011299**	MRPS30	protein_coding	chr5	44808947	44820428	+	Human GRCh	OTTHUMG000000	MRPS30	**mitochondrial ribosomal protein S30**	Cytoplasm	enzyme		1.033868301	0	0.87345419	0	1	0	10.691477	10.8754961	11.0028845	10.6125949
4088	**ENSG0000011301**	HSPA9	protein_coding	chr5	138553756	138575675	-	Human GRCh	OTTHUMG000001	HSPA9	**heat shock protein family A (Hsp70) member 9**	Cytoplasm	other	SHetA2	-1.110738167	0	0.61585524	0	1	0	13.3064573	13.5818627	13.4403071	13.2173818
4089	**ENSG0000011304**	MRPS27	protein_coding	chr5	72214953	72321717	-	Human GRCh	OTTHUMG000001	MRPS27	**mitochondrial ribosomal protein S27**	Cytoplasm	other		-1.151297118	0	0.51560257	0	1	0	11.8291308	12.2072134	12.0486293	11.6634744
4090	**ENSG0000011306**	PFDN1	protein_coding	chr5	140245035	140303113	-	Human GRCh	OTTHUMG000001	PFDN1	**prefoldin subunit 1**	Cytoplasm	other		-1.230721251	0	0.30032124	0	1	0	9.81231573	10.1333695	9.82544202	9.67659791
4091	**ENSG0000011307**	HBEGF	protein_coding	chr5	140332843	140346603	-	Human GRCh	OTTHUMG000001	HBEGF	**heparin binding EGF like growth factor**	Extracellular	growth facto	KHK2866,	1.125182928	0	0.76778583	0	1	0	6.11973424	5.85964701	6.10262734	6.1330147
4092	**ENSG0000011307**	SLC4A9	protein_coding	chr5	140360194	140375141	+	Human GRCh	OTTHUMG000001	SLC4A9	**solute carrier family 4 member 9**	Plasma Mem	transporter		1.750259813	1	0.53103199	0	NA	0	3.19746508	3.2155033	3.58218354	3.65826173
4093	**ENSG0000011308**	LOX	protein_coding	chr5	122063195	122078413	-	Human GRCh	OTTHUMG000001	LOX	**lysyl oxidase**	Extracellular	enzyme	PXS-5505,	1.214885956	0	0.76119907	0	1	0	9.62686034	10.2111704	10.5139214	9.74385535
4095	**ENSG0000011310**	CDH9	protein_coding	chr5	26880597	27121150	-	Human GRCh	OTTHUMG000000	CDH9	**cadherin 9**	Plasma Mem	other		1	0	1	0	NA	0	-0.0460605	-0.0183063	-0.0227841	-0.0443874
4096	**ENSG0000011310**	APBB3	protein_coding	chr5	140558268	140564781	-	Human GRCh	OTTHUMG000001	APBB3	**amyloid beta precursor protein binding family B member 3**	Cytoplasm	other		1.039091269	0	0.86890664	0	1	0	9.25613533	9.48913568	9.6608176	9.11878567
4097	**ENSG0000011311**	TMCO6	protein_coding	chr5	140639435	140645408	+	Human GRCh	OTTHUMG000001	TMCO6	**transmembrane and coiled-coil domains 6**	Other	other		-1.058236743	0	0.7903916	0	1	0	9.04424503	9.18276136	9.20827485	8.87476931
4098	**ENSG0000011314**	SPARC	protein_coding	chr5	151661096	151686975	-	Human GRCh	OTTHUMG000001	SPARC	**secreted protein acidic and cysteine rich**	Extracellular	other		35.91479013	1	0.00828161	1	1	0	1.86615173	1.96064249	2.58979489	3.72354336
4099	**ENSG0000011314**	IK	protein_coding	chr5	140647058	140662480	+	Human GRCh	OTTHUMG000001	IK	**IK cytokine**	Extracellular	cytokine		-1.154509022	0	0.4983889	0	1	0	10.8408175	11.19481	10.9054042	10.8310393
4100	**ENSG0000011316**	HMGCR	protein_coding	chr5	75336329	75364001	+	Human GRCh	OTTHUMG000001	HMGCR	**3-hydroxy-3-methylglutaryl-CoA reductase**	Cytoplasm	enzyme	atorvastati	-1.131931005	0	0.58969287	0	1	0	12.0648779	12.5993907	12.3524037	12.0694945
4101	**ENSG0000011316**	CERT1	protein_coding	chr5	75356345	75512138	-	Human GRCh	OTTHUMG000001	CERT1	**ceramide transporter 1**	Cytoplasm	kinase		1.032325892	0	0.88616875	0	1	0	10.4177841	10.7833717	10.8736323	10.3741112
4102	**ENSG0000011319**	FAF2	protein_coding	chr5	176447628	176510074	+	Human GRCh	OTTHUMG000001	FAF2	**Fas associated factor family member 2**	Cytoplasm	other		-1.158963034	0	0.45616611	0	1	0	11.2158811	11.5465129	11.2938207	11.1587852
4104	**ENSG0000011320**	PCDHB3	protein_coding	chr5	141100473	141103827	+	Human GRCh	OTTHUMG000001	PCDHB3	**protocadherin beta 3**	Plasma Mem	other		1.024367314	0	0.93532521	0	1	0	7.59417115	7.97958299	8.03090107	7.58320989
4105	**ENSG0000011320**	PCDHB5	protein_coding	chr5	141135206	141138615	+	Human GRCh	OTTHUMG000001	PCDHB5	**protocadherin beta 5**	Plasma Mem	other		1.394171336	0	0.16462896	0	1	0	8.63267428	8.78442078	9.27509037	8.84581282
4106	**ENSG0000011321**	PCDHB6	protein_coding	chr5	141150057	141153287	+	Human GRCh	OTTHUMG000001	PCDHB6	**protocadherin beta 6**	Plasma Mem	other		1.382728835	0	0.44691465	0	1	0	5.19503517	5.70023013	6.05541733	5.43269588
4107	**ENSG0000011321**	PCDHB7	protein_coding	chr5	141172644	141176383	+	Human GRCh	OTTHUMG000001	PCDHB7	**protocadherin beta 7**	Plasma Mem	other		1.702474876	1	0.32179058	0	1	0	5.20744181	4.1689244	5.69387654	4.71102282
4108	**ENSG0000011323**	PDE8B	protein_coding	chr5	77210449	77428256	+	Human GRCh	OTTHUMG000001	PDE8B	**phosphodiesterase 8B**	Cytoplasm	enzyme	ketotifen	-1.664447368	-1	0.11460854	0	1	0	7.52241373	7.29542489	6.88989505	6.87547294
4109	**ENSG0000011324**	CLK4	protein_coding	chr5	178602664	178630615	-	Human GRCh	OTTHUMG000001	CLK4	**CDC like kinase 4**	Nucleus	kinase	KH-CB19	1.082469906	0	0.73517536	0	1	0	9.10648408	9.3527387	9.57805504	8.99800751
4110	**ENSG0000011324**	PCDHB15	protein_coding	chr5	141245395	141249365	+	Human GRCh	OTTHUMG000001	PCDHB15	**protocadherin beta 15**	Plasma Mem	other		1.477873623	0	0.31056354	0	1	0	6.64951205	6.86727343	7.46218104	6.7918397
4114	**ENSG0000011326**	RNF130	protein_coding	chr5	179911651	180072113	-	Human GRCh	OTTHUMG000001	RNF130	**ring finger protein 130**	Cytoplasm	peptidase		-1.202385984	0	0.46547174	0	1	0	10.4392524	10.6934354	10.5317119	10.1780441
4115	**ENSG0000011327**	THG1L	protein_coding	chr5	157731420	157741449	+	Human GRCh	OTTHUMG000001	THG1L	**tRNA-histidine guanylyltransferase 1 like**	Cytoplasm	enzyme		-1.034541084	0	0.9028727	0	1	0	7.04512484	7.04816447	7.10117707	6.91493438
4116	**ENSG0000011327**	ARSB	protein_coding	chr5	78777209	78986087	-	Human GRCh	OTTHUMG000001	ARSB	**arylsulfatase B**	Cytoplasm	enzyme		1.139840799	0	0.59827373	0	1	0	7.2044564	7.2771826	7.44904507	7.29881558
4117	**ENSG0000011328**	CLINT1	protein_coding	chr5	157785743	157859145	-	Human GRCh	OTTHUMG000001	CLINT1	**clathrin interactor 1**	Cytoplasm	other		-1.033057299	0	0.8753581	0	1	0	11.163269	11.484986	11.4933223	11.0650468
4118	**ENSG0000011329**	THBS4	protein_coding	chr5	79991311	80083287	+	Human GRCh	OTTHUMG000001	THBS4	**thrombospondin 4**	Extracellular	other		-1.078165806	0	0.88638012	0	1	0	4.57532668	4.09421615	4.77759187	3.46946516
4119	**ENSG0000011330**	CNOT6	protein_coding	chr5	180494379	180578358	+	Human GRCh	OTTHUMG000001	CNOT6	**CCR4-NOT transcription complex subunit 6**	Nucleus	enzyme		-1.095548543	0	0.65512469	0	1	0	11.4787266	11.7998344	11.6886718	11.3918324
4122	**ENSG0000011331**	TTC1	protein_coding	chr5	160009113	160065543	+	Human GRCh	OTTHUMG000001	TTC1	**tetratricopeptide repeat domain 1**	Cytoplasm	other		-1.000448371	0	0.99815489	0	1	0	9.60351385	9.99816825	9.90245477	9.72387961
4123	**ENSG0000011331**	MSH3	protein_coding	chr5	80654652	80876815	+	Human GRCh	OTTHUMG000001	MSH3	**mutS homolog 3**	Nucleus	enzyme		-1.071425218	0	0.78124729	0	1	0	9.37546045	9.45802112	9.55014316	9.08998542
4124	**ENSG0000011331**	RASGRF2	protein_coding	chr5	80960363	81230162	+	Human GRCh	OTTHUMG000001	RASGRF2	**Ras protein specific guanine nucleotide releasing factor 2**	Cytoplasm	other		-1.029673149	0	0.96030196	0	1	0	4.59963247	5.21584531	5.18478891	4.57977267
4125	**ENSG0000011332**	GABRG2	protein_coding	chr5	162000057	162162977	+	Human GRCh	OTTHUMG000001	GABRG2	**gamma-aminobutyric acid type A receptor subunit gamma2**	Plasma Mem	ion channel	triazolam,	2.942020474	1	0.38685869	0	1	0	2.31894874	1.7794775	1.76493964	3.01357455
4126	**ENSG0000011332**	CCNG1	protein_coding	chr5	163437569	163446151	+	Human GRCh	OTTHUMG000001	CCNG1	**cyclin G1**	Nucleus	other		-1.090730646	0	0.70408663	0	1	0	10.9068833	11.2161345	11.1319657	10.7925913
4127	**ENSG0000011335**	POLR3G	protein_coding	chr5	90471748	90514557	+	Human GRCh	OTTHUMG000001	POLR3G	**RNA polymerase III subunit G**	Nucleus	enzyme		1.046689504	0	0.8844042	0	1	0	7.33074187	7.77198386	7.7788838	7.44142963
4128	**ENSG0000011336**	DROSHA	protein_coding	chr5	31400494	31532196	-	Human GRCh	OTTHUMG000001	DROSHA	**drosha ribonuclease III**	Nucleus	enzyme		-1.227625497	0	0.31408847	0	1	0	11.9650048	12.3311899	12.0242744	11.8367735
4129	**ENSG0000011336**	CDH6	protein_coding	chr5	31193686	31329146	+	Human GRCh	OTTHUMG000000	CDH6	**cadherin 6**	Plasma Mem	other	DS-6000a,	1.692171644	1	0.60098722	0	1	0	3.07539121	3.80200584	4.40805247	3.29688555
4130	**ENSG0000011336**	LMNB1	protein_coding	chr5	126776623	126837020	+	Human GRCh	OTTHUMG000001	LMNB1	**lamin B1**	Nucleus	other		-1.066821804	0	0.75581241	0	1	0	12.3815708	12.7034757	12.6193815	12.323739
4131	**ENSG0000011336**	ARRDC3	protein_coding	chr5	91368631	91383317	-	Human GRCh	OTTHUMG000001	ARRDC3	**arrestin domain containing 3**	Plasma Mem	other		1.19799759	0	0.43938993	0	1	0	10.0908138	10.3324318	10.5412853	10.2769293
4132	**ENSG0000011338**	GOLPH3	protein_coding	chr5	32124716	32174319	-	Human GRCh	OTTHUMG000000	GOLPH3	**golgi phosphoprotein 3**	Cytoplasm	other		-1.082946113	0	0.71400833	0	1	0	11.0157399	11.3264398	11.2101986	10.9652107
4133	**ENSG0000011338**	SUB1	protein_coding	chr5	32531633	32604079	+	Human GRCh	OTTHUMG000001	SUB1	**SUB1 regulator of transcription**	Nucleus	transcription		1.120903022	0	0.59150142	0	1	0	11.2216634	11.501755	11.63929	11.3324398
4134	**ENSG0000011338**	NPR3	protein_coding	chr5	32689070	32791724	+	Human GRCh	OTTHUMG000001	NPR3	**natriuretic peptide receptor 3**	Plasma Mem	G-protein cou	linaclotide	1.178039513	0	0.70382626	0	1	0	6.36280628	6.63242011	6.5863547	6.78027875
4135	**ENSG0000011339**	FAM172A	protein_coding	chr5	93617725	94111699	-	Human GRCh	OTTHUMG000001	FAM172A	**family with sequence similarity 172 member A**	Cytoplasm	transcription		-1.15959706	0	0.53878999	0	1	0	10.4928499	10.7760102	10.6667369	10.245676
4136	**ENSG0000011339**	SLC27A6	protein_coding	chr5	128538013	129033642	+	Human GRCh	OTTHUMG000001	SLC27A6	**solute carrier family 27 member 6**	Plasma Mem	transporter		-1.344614017	0	0.72343314	0	1	0	2.49410141	3.9766079	3.04291234	3.40125177
4137	**ENSG0000011340**	TARS1	protein_coding	chr5	33440696	33468091	+	Human GRCh	OTTHUMG000000	TARS1	**threonyl-tRNA synthetase 1**	Nucleus	enzyme		-1.183303474	0	0.35363323	0	1	0	11.5325768	11.6053957	11.5212358	11.229681
4138	**ENSG0000011343**	IRX4	protein_coding	chr5	1877413	1887236	-	Human GRCh	OTTHUMG000000	IRX4	**iroquois homeobox 4**	Nucleus	transcription		2.971595864	1	0.01990488	1	1	0	4.62064684	4.857402	4.97040704	6.21179422
4139	**ENSG0000011344**	LNPEP	protein_coding	chr5	96935394	97037513	+	Human GRCh	OTTHUMG000001	LNPEP	**leucyl and cystinyl aminopeptidase**	Cytoplasm	peptidase		1.048716367	0	0.85424678	0	1	0	12.6602511	12.9468452	13.1165736	12.5523491
4140	**ENSG0000011344**	PDE4D	protein_coding	chr5	58969038	60522120	-	Human GRCh	OTTHUMG000001	PDE4D	**phosphodiesterase 4D**	Cytoplasm	enzyme	dyphylline,	1.430592304	0	0.47777803	0	1	0	9.35731896	9.65285074	10.0664621	9.70355092
4141	**ENSG0000011345**	RAD1	protein_coding	chr5	34905260	34918989	-	Human GRCh	OTTHUMG000000	RAD1	**RAD1 checkpoint DNA exonuclease**	Nucleus	enzyme		-1.05730404	0	0.80191908	0	1	0	10.010758	10.2831088	10.2818888	9.87157495
4142	**ENSG0000011346**	BRIX1	protein_coding	chr5	34915677	34925996	+	Human GRCh	OTTHUMG000001	BRIX1	**biogenesis of ribosomes BRX1**	Nucleus	other		-1.119069921	0	0.6005207	0	1	0	10.1485477	10.4645885	10.3440717	10.0242426
4144	**ENSG0000011349**	PRLR	protein_coding	chr5	35048756	35230487	-	Human GRCh	OTTHUMG000000	PRLR	**prolactin receptor**	Plasma Mem	transmembra	fluoxymest	-19.51499246	-1	0.04885082	1	1	0	2.18452329	2.58594539	1.22376927	1.12914513
4145	**ENSG0000011350**	SLC12A7	protein_coding	chr5	1050384	1112063	-	Human GRCh	OTTHUMG000001	SLC12A7	**solute carrier family 12 member 7**	Plasma Mem	transporter		-1.01119877	0	0.9562472	0	1	0	11.0597145	11.1639104	11.0991509	11.102591
4147	**ENSG0000011352**	RAD50	protein_coding	chr5	132556019	132646349	+	Human GRCh	OTTHUMG000000	RAD50	**RAD50 double strand break repair protein**	Nucleus	enzyme		-1.059531331	0	0.76117731	0	1	0	11.8360038	12.1401125	12.1149338	11.7177381
4149	**ENSG0000011353**	ST8SIA4	protein_coding	chr5	100806933	100903282	-	Human GRCh	OTTHUMG000001	ST8SIA4	**ST8 alpha-N-acetyl-neuraminide alpha-2,8-sialyltransferase 4**	Cytoplasm	enzyme		1.233583355	0	0.48919094	0	1	0	6.48755859	6.72003064	6.78112629	6.88154186
4150	**ENSG0000011355**	GNPDA1	protein_coding	chr5	141991749	142013041	-	Human GRCh	OTTHUMG000001	GNPDA1	**glucosamine-6-phosphate deaminase 1**	Cytoplasm	enzyme		1.309444731	0	0.25687061	0	1	0	8.53178946	8.72894461	9.08780684	8.73137691
4151	**ENSG0000011355**	PCDH12	protein_coding	chr5	141943581	141969741	-	Human GRCh	OTTHUMG000001	PCDH12	**protocadherin 12**	Plasma Mem	other		1	0	1	0	NA	0	0.90457179	0.91783741	0.91561852	0.90533934

4152	**ENSG0000011355**	SKP1	protein_coding	chr5	134148935	134176964	-	Human GRCh	OTTHUMG000001	SKP1	**S-phase kinase associated protein 1**	Nucleus	transcription		-1.082436199	0	0.69033493	0	1	0	11.8856041	12.2769821	12.1487386	11.8511928
4153	**ENSG0000011356**	NUP155	protein_coding	chr5	37288137	37371106	-	Human GRCh	OTTHUMG000000	NUP155	**nucleoporin 155**	Nucleus	other		-1.086283163	0	0.68014171	0	1	0	12.536623	12.8407551	12.7491401	12.4459842
4154	**ENSG0000011357**	PPP2CA	protein_coding	chr5	134194035	134226073	-	Human GRCh	OTTHUMG000001	PPP2CA	**protein phosphatase 2 catalytic subunit alpha**	Cytoplasm	phosphatase		-1.009251125	0	0.96316708	0	1	0	11.395156	11.8235244	11.7124862	11.5163274
4155	**ENSG0000011357**	FGF1	protein_coding	chr5	142592178	142698070	-	Human GRCh	OTTHUMG000000	FGF1	**fibroblast growth factor 1**	Extracellular	growth facto	pentosan p	1	0	1	0	NA	0	0.4146332	0.4255852	0.42375798	0.41526872
4156	**ENSG0000011358**	NR3C1	protein_coding	chr5	143277931	143435512	-	Human GRCh	OTTHUMG000001	NR3C1	**nuclear receptor subfamily 3 group C member 1**	Nucleus	ligand-depen	dexametha	1.353903682	0	0.52516222	0	1	0	7.73034235	8.08653555	8.030594	8.36011207
4157	**ENSG0000011358**	C5orf15	protein_coding	chr5	133955510	133968674	-	Human GRCh	OTTHUMG000001	C5orf15	**chromosome 5 open reading frame 15**	Other	other		1.040132261	0	0.85254432	0	1	0	10.2825295	10.4486259	10.5016341	10.3154851
4158	**ENSG0000011359**	PPWD1	protein_coding	chr5	65563236	65587549	+	Human GRCh	OTTHUMG000001	PPWD1	**peptidylprolyl isomerase domain and WD repeat containing 1**	Nucleus	enzyme		1.032882574	0	0.88570003	0	1	0	9.21997538	9.49376162	9.55284385	9.22388729
4159	**ENSG0000011359**	LIFR	protein_coding	chr5	38474668	38608354	-	Human GRCh	OTTHUMG000001	LIFR	**LIF receptor subunit alpha**	Plasma Mem	transmembra		-1.080249236	0	0.80136345	0	1	0	12.5794972	12.7035003	12.7241812	12.366
4160	**ENSG0000011359**	TRIM23	protein_coding	chr5	65589690	65625975	-	Human GRCh	OTTHUMG000000	TRIM23	**tripartite motif containing 23**	Nucleus	enzyme		1.0728981	0	0.73509803	0	1	0	9.0864781	9.2111096	9.34492336	9.09916026
4161	**ENSG0000011359**	TRAPPC13	protein_coding	chr5	65625004	65666233	+	Human GRCh	OTTHUMG000001	TRAPPC13	**trafficking protein particle complex subunit 13**	Cytoplasm	other		-1.048400107	0	0.84894468	0	1	0	8.4780101	8.91457835	8.90400239	8.36541701
4163	**ENSG0000011361**	SEC24A	protein_coding	chr5	134648785	134727909	+	Human GRCh	OTTHUMG000001	SEC24A	**SEC24 homolog A, COPII coat complex component**	Cytoplasm	transporter		-1.03771136	0	0.85483384	0	1	0	10.9112894	11.2270732	11.1097491	10.9611381
4164	**ENSG0000011362**	TXNDC15	protein_coding	chr5	134874371	134901635	+	Human GRCh	OTTHUMG000001	TXNDC15	**thioredoxin domain containing 15**	Extracellular	enzyme		-1.023564688	0	0.90163754	0	1	0	9.52422211	9.71421939	9.70482415	9.47942228
4165	**ENSG0000011363**	TTC33	protein_coding	chr5	40512333	40755963	-	Human GRCh	OTTHUMG000001	TTC33	**tetratricopeptide repeat domain 33**	Other	other		-1.142228814	0	0.56930448	0	1	0	9.35896385	9.54252889	9.56466905	8.9786529
4166	**ENSG0000011364**	RARS1	protein_coding	chr5	168486451	168519301	+	Human GRCh	OTTHUMG000001	RARS1	**arginyl-tRNA synthetase 1**	Cytoplasm	enzyme		-1.068109565	0	0.74637908	0	1	0	10.9108039	11.2903473	11.1683156	10.8995278
4167	**ENSG0000011364**	WWC1	protein_coding	chr5	168291645	168472303	+	Human GRCh	OTTHUMG000001	WWC1	**WW and C2 domain containing 1**	Cytoplasm	transcription		-1.224953059	0	0.47585861	0	1	0	12.757188	12.9589339	12.7924509	12.4382234
4168	**ENSG0000011364**	MACROH2	protein_coding	chr5	135334381	135399914	-	Human GRCh	OTTHUMG000001	MACROH2A1	**macroH2A.1 histone**	Nucleus	other		-1.061349523	0	0.7748086	0	1	0	12.7747473	13.0342132	13.0182005	12.6459733
4169	**ENSG0000011364**	TCERG1	protein_coding	chr5	146447311	146511961	+	Human GRCh	OTTHUMG000001	TCERG1	**transcription elongation regulator 1**	Nucleus	transcription		-1.102015407	0	0.62457848	0	1	0	11.7424043	12.0667044	11.9442624	11.6554861
4170	**ENSG0000011365**	DPYSL3	protein_coding	chr5	147390808	147510068	-	Human GRCh	OTTHUMG000001	DPYSL3	**dihydropyrimidinase like 3**	Cytoplasm	enzyme		-1.140284997	0	0.77874267	0	1	0	9.52839877	10.0511045	9.19968219	10.0277416
4171	**ENSG0000011365**	SMAD5	protein_coding	chr5	136132845	136188747	+	Human GRCh	OTTHUMG000001	SMAD5	**SMAD family member 5**	Nucleus	transcription		1.056159771	0	0.80611685	0	1	0	11.1624453	11.3897061	11.5241041	11.1246477
4172	**ENSG0000011371**	CSNK1A1	protein_coding	chr5	149492982	149551471	-	Human GRCh	OTTHUMG000001	CSNK1A1	**casein kinase 1 alpha 1**	Cytoplasm	kinase	BTX-A51, C	-1.012042857	0	0.95801661	0	1	0	13.3824685	13.6479473	13.7351769	13.2295021
4173	**ENSG0000011371**	HMGXB3	protein_coding	chr5	150000046	150053142	+	Human GRCh	OTTHUMG000001	HMGXB3	**HMG-box containing 3**	Nucleus	transcription		-1.053875885	0	0.87172096	0	1	0	11.5002048	11.7975076	11.7594299	11.4176816
4174	**ENSG0000011371**	ERGIC1	protein_coding	chr5	172834251	172952792	+	Human GRCh	OTTHUMG000001	ERGIC1	**endoplasmic reticulum-golgi intermediate compartment 1**	Cytoplasm	other		-1.081678999	0	0.67584425	0	1	0	11.7587986	11.9100979	11.8522147	11.6368482
4175	**ENSG0000011372**	PDGFRB	protein_coding	chr5	150113839	150155872	-	Human GRCh	OTTHUMG000001	PDGFRB	**platelet derived growth factor receptor beta**	Plasma Mem	kinase	midostauri	1.341865833	0	0.84352292	0	NA	0	2.02471682	1.11536218	1.11215627	2.21061942
4177	**ENSG0000011373**	ATP6V0E1	protein_coding	chr5	172983771	173035445	+	Human GRCh	OTTHUMG000001	ATP6V0E1	**ATPase H+ transporting V0 subunit e1**	Cytoplasm	transporter		-1.208817449	0	0.39865304	0	1	0	9.16447072	9.55576963	9.26894273	9.04785502
4178	**ENSG0000011373**	BNIP1	protein_coding	chr5	173144442	173164387	+	Human GRCh	OTTHUMG000001	BNIP1	**BCL2 interacting protein 1**	Cytoplasm	transporter		-1.17573058	0	0.56420236	0	1	0	7.90973397	8.35181576	8.12498212	7.79909956
4179	**ENSG0000011373**	STC2	protein_coding	chr5	173314273	173328447	-	Human GRCh	OTTHUMG000001	STC2	**stanniocalcin 2**	Extracellular	other		-1.028828831	0	0.91317609	0	1	0	11.2202874	11.2272296	11.405229	10.9305669
4180	**ENSG0000011374**	CPEB4	protein_coding	chr5	173888349	173961980	+	Human GRCh	OTTHUMG000001	CPEB4	**cytoplasmic polyadenylation element binding protein 4**	Plasma Mem	other		1.13333476	0	0.73068127	0	1	0	10.9957354	11.1356242	11.3014708	11.116056
4181	**ENSG0000011374**	HRH2	protein_coding	chr5	175658030	175710756	+	Human GRCh	OTTHUMG000001	HRH2	**histamine receptor H2**	Plasma Mem	G-protein co	epinastine	1	0	1	0	1	0	0.8534901	0.90755753	0.89882098	0.85674544
4182	**ENSG0000011375**	DBN1	protein_coding	chr5	177456608	177474401	-	Human GRCh	OTTHUMG000001	DBN1	**drebrin 1**	Cytoplasm	other		-1.124641579	0	0.65314628	0	1	0	11.8530142	12.1662262	12.0663808	11.6785423
4183	**ENSG0000011376**	ZNF346	protein_coding	chr5	177022696	177081189	+	Human GRCh	OTTHUMG000001	ZNF346	**zinc finger protein 346**	Nucleus	other		-1.137871261	0	0.58158984	0	1	0	9.64402604	10.1787993	9.93429513	9.64712908
4184	**ENSG0000011376**	UNC5A	protein_coding	chr5	176810519	176880898	+	Human GRCh	OTTHUMG000001	UNC5A	**unc-5 netrin receptor A**	Plasma Mem	transmembra		1.177911914	0	0.75281991	0	1	0	4.45146225	2.89445233	3.9551321	4.19215746
4185	**ENSG0000011379**	EHHADH	protein_coding	chr3	185190624	185281990	-	Human GRCh	OTTHUMG000001	EHHADH	**enoyl-CoA hydratase and 3-hydroxyacyl CoA dehydrogenase**	Cytoplasm	enzyme		1.222264887	0	0.62611462	0	1	0	5.83958048	6.08072113	6.22929206	6.08470028
4186	**ENSG0000011380**	CNTN3	protein_coding	chr3	74262568	74616459	-	Human GRCh	OTTHUMG000001	CNTN3	**contactin 3**	Plasma Mem	other		3.057663787	1	0.00670241	1	1	0	4.30219395	4.94043582	5.67979933	6.04533873
4187	**ENSG0000011381**	SMC4	protein_coding	chr3	160399274	160434954	+	Human GRCh	OTTHUMG000001	SMC4	**structural maintenance of chromosomes 4**	Nucleus	transporter		-1.097668784	0	0.62362666	0	1	0	12.4249142	12.7181027	12.5949576	12.354592
4188	**ENSG0000011381**	SELENOK	protein_coding	chr3	53884417	53891885	-	Human GRCh	OTTHUMG000001	SELENOK	**selenoprotein K**	Cytoplasm	other		-1.110445152	0	0.65263738	0	1	0	9.65426241	10.0201742	9.82487343	9.63449476
4189	**ENSG0000011381**	ACTR8	protein_coding	chr3	53867066	53882152	-	Human GRCh	OTTHUMG000001	ACTR8	**actin related protein 8**	Nucleus	other		-1.098152924	0	0.65794318	0	1	0	9.02153249	9.29960237	9.22570068	8.88571549
4190	**ENSG0000011383**	TBCCD1	protein_coding	chr3	186546067	186570543	-	Human GRCh	OTTHUMG000001	TBCCD1	**TBCC domain containing 1**	Cytoplasm	other		-1.119385998	0	0.63374857	0	1	0	9.10955128	9.48646113	9.31204576	9.05136542
4191	**ENSG0000011384**	TIMMDC1	protein_coding	chr3	119498547	119525090	+	Human GRCh	OTTHUMG000001	TIMMDC1	**translocase of inner mitochondrial membrane domain contain**	Cytoplasm	other		-1.077165134	0	0.71632756	0	1	0	9.81068912	9.99541239	9.99875293	9.62057649
4192	**ENSG0000011385**	CRBN	protein_coding	chr3	3144628	3179727	-	Human GRCh	OTTHUMG000000	CRBN	**cereblon**	Cytoplasm	enzyme	HSK29116,	-1.039885875	0	0.85653387	0	1	0	9.8846754	10.0412039	10.0886181	9.72982806
4195	**ENSG0000011391**	BCL6	protein_coding	chr3	187721377	187745725	-	Human GRCh	OTTHUMG000001	BCL6	**BCL6 transcription repressor**	Nucleus	transcription		-1.230521507	0	0.40850499	0	1	0	10.4709899	10.466338	10.2237586	10.2484333
4196	**ENSG0000011392**	HGD	protein_coding	chr3	120628172	120682269	-	Human GRCh	OTTHUMG000001	HGD	**homogentisate 1,2-dioxygenase**	Cytoplasm	enzyme		-2.011389228	-1	0.34452156	0	1	0	3.22192017	3.36684623	2.99984489	2.76535277
4198	**ENSG0000011396**	ARL6	protein_coding	chr3	97764521	97801229	+	Human GRCh	OTTHUMG000001	ARL6	**ADP ribosylation factor like GTPase 6**	Cytoplasm	transporter		1.168267796	0	0.53042796	0	1	0	8.2281563	8.24686362	8.56525915	8.2192568
4199	**ENSG0000011397**	NPHP3	protein_coding	chr3	132680609	132722432	-	Human GRCh	OTTHUMG000001	NPHP3	**nephrocystin 3**	Extracellular	other		-1.011939125	0	0.96117366	0	1	0	9.35680185	9.64026841	9.6882762	9.2613048
4200	**ENSG0000011401**	CD86	protein_coding	chr3	122055362	122121139	+	Human GRCh	OTTHUMG000001	CD86	**CD86 molecule**	Plasma Mem	transmembra	abatacept,	-1.630924911	-1	0.50048304	0	1	0	5.7621871	5.18618809	5.31779203	4.53279687
4201	**ENSG0000011401**	AMOTL2	protein_coding	chr3	134355347	134375479	-	Human GRCh	OTTHUMG000001	AMOTL2	**angiomotin like 2**	Plasma Mem	other		1.857192352	1	0.05779896	0	1	0	6.6748967	6.16313565	6.93276913	7.08036955
4202	**ENSG0000011402**	NIT2	protein_coding	chr3	100334739	100361635	+	Human GRCh	OTTHUMG000001	NIT2	**nitrilase family member 2**	Cytoplasm	enzyme		-1.130670497	0	0.5496531	0	1	0	9.70353968	10.0136366	9.90936068	9.53012619
4203	**ENSG0000011402**	FAM162A	protein_coding	chr3	122384161	122412334	+	Human GRCh	OTTHUMG000001	FAM162A	**family with sequence similarity 162 member A**	Cytoplasm	other		1.057775883	0	0.78791713	0	1	0	10.8095789	11.2349768	11.1894146	11.0144928
4204	**ENSG0000011402**	OGG1	protein_coding	chr3	9749944	9788219	+	Human GRCh	OTTHUMG000000	OGG1	**8-oxoguanine DNA glycosylase**	Nucleus	enzyme		-1.018545072	0	0.93154472	0	1	0	9.6219465	10.1718515	9.98072047	9.83143612
4205	**ENSG0000011403**	KPNA1	protein_coding	chr3	122421902	122514945	-	Human GRCh	OTTHUMG000001	KPNA1	**karyopherin subunit alpha 1**	Nucleus	transporter		-1.090699813	0	0.68005062	0	1	0	11.0558213	11.370263	11.266624	10.9684271
4206	**ENSG0000011405**	PCCB	protein_coding	chr3	136250340	136337896	+	Human GRCh	OTTHUMG000001	PCCB	**propionyl-CoA carboxylase subunit beta**	Cytoplasm	enzyme		1.058853512	0	0.82096123	0	1	0	10.1269866	10.5794374	10.6213546	10.2235682
4207	**ENSG0000011406**	UBE3A	protein_coding	chr15	25333728	25439051	-	Human GRCh	OTTHUMG000001	UBE3A	**ubiquitin protein ligase E3A**	Nucleus	enzyme		1.000084656	0	0.99965781	0	1	0	12.0755581	12.2350462	12.3233334	11.9659623
4208	**ENSG0000011409**	ARMC8	protein_coding	chr3	138187248	138298384	+	Human GRCh	OTTHUMG000001	ARMC8	**armadillo repeat containing 8**	Nucleus	other		-1.04270517	0	0.85626311	0	1	0	10.0804189	10.3840317	10.3764614	9.98496948
4209	**ENSG0000011410**	CEP70	protein_coding	chr3	138494344	138594538	-	Human GRCh	OTTHUMG000001	CEP70	**centrosomal protein 70**	Cytoplasm	other		-1.086040114	0	0.69950452	0	1	0	9.74065878	9.94339513	9.93586148	9.54780649
4210	**ENSG0000011411**	RBP2	protein_coding	chr3	139452884	139480747	-	Human GRCh	OTTHUMG000001	RBP2	**retinol binding protein 2**	Cytoplasm	transporter		1	0	1	0	NA	0	0.49298883	0.50317732	0.5014713	0.4935776
4211	**ENSG0000011411**	RBP1	protein_coding	chr3	139517434	139539829	-	Human GRCh	OTTHUMG000001	RBP1	**retinol binding protein 1**	Extracellular	transporter		2.007720545	1	0.19838362	0	1	0	3.65594413	2.63353407	3.80243161	3.66660618
4212	**ENSG0000011412**	SLC25A36	protein_coding	chr3	140941830	140980995	+	Human GRCh	OTTHUMG000001	SLC25A36	**solute carrier family 25 member 36**	Cytoplasm	transporter		1.048971374	0	0.84667945	0	1	0	11.4318828	11.5749361	11.7406443	11.3392795
4213	**ENSG0000011412**	GRK7	protein_coding	chr3	141763408	141819352	+	Human GRCh	OTTHUMG000001	GRK7	**G protein-coupled receptor kinase 7**	Cytoplasm	kinase		-1.467790188	0	0.44964923	0	1	0	4.28713921	4.73364711	4.39263502	3.96431617
4214	**ENSG0000011412**	RNF7	protein_coding	chr3	141738249	141747560	+	Human GRCh	OTTHUMG000001	RNF7	**ring finger protein 7**	Nucleus	enzyme		1.052565773	0	0.81248754	0	1	0	9.07950908	9.18373676	9.26648147	9.10584739
4215	**ENSG0000011412**	TFDP2	protein_coding	chr3	141944428	142149544	-	Human GRCh	OTTHUMG000001	TFDP2	**transcription factor Dp-2**	Nucleus	transcription		-1.207612687	0	0.42252423	0	1	0	11.3407349	11.6889186	11.4771188	11.1438837
4216	**ENSG0000011412**	XRN1	protein_coding	chr3	142306607	142448062	-	Human GRCh	OTTHUMG000001	XRN1	**5'-3' exoribonuclease 1**	Cytoplasm	enzyme		-1.072485085	0	0.74740706	0	1	0	11.273192	11.5305269	11.4620853	11.1916081
4217	**ENSG0000011416**	KAT2B	protein_coding	chr3	20040446	20154404	+	Human GRCh	OTTHUMG000001	KAT2B	**lysine acetyltransferase 2B**	Nucleus	enzyme		-1.105232046	0	0.70059015	0	1	0	8.39171314	8.66004604	8.37024326	8.47352492
4218	**ENSG0000011420**	BCHE	protein_coding	chr3	165772904	165837462	-	Human GRCh	OTTHUMG000001	BCHE	**butyrylcholinesterase**	Plasma Mem	enzyme	hexafluore	10.77931704	1	0.12856365	0	1	0	0.90316185	0.95645725	1.6702876	1.57767956
4219	**ENSG0000011420**	SERPINI2	protein_coding	chr3	167441914	167478192	+	Human GRCh	OTTHUMG000001	SERPINI2	**serpin family I member 2**	Extracellular	other		1.13842463	0	0.88903607	0	1	0	1.98850766	2.43834672	2.41824182	2.17158469
4220	**ENSG0000011420**	PDCD10	protein_coding	chr3	167683298	167734939	-	Human GRCh	OTTHUMG000001	PDCD10	**programmed cell death 10**	Cytoplasm	other		-1.009213167	0	0.96806597	0	1	0	9.02471714	9.51245735	9.3465478	9.21675832
4222	**ENSG0000011425**	WNT5A	protein_coding	chr3	55465715	55490539	-	Human GRCh	OTTHUMG000001	WNT5A	**Wnt family member 5A**	Extracellular	growth facto		-2.08373441	-1	0.06727999	0	1	0	7.66013715	7.82883562	6.75817054	7.15317408
4223	**ENSG0000011426**	PFKFB4	protein_coding	chr3	48517684	48562015	-	Human GRCh	OTTHUMG000001	PFKFB4	**6-phosphofructo-2-kinase/fructose-2,6-biphosphatase 4**	Cytoplasm	kinase		-1.090558994	0	0.74901152	0	1	0	10.5238365	11.1853852	10.9307456	10.6582459
4224	**ENSG0000011427**	COL7A1	protein_coding	chr3	48564073	48595329	-	Human GRCh	OTTHUMG000001	COL7A1	**collagen type VII alpha 1 chain**	Extracellular	other	collagenas	-1.147629506	0	0.63293069	0	1	0	10.196634	10.2484571	10.3252281	9.74690901
4225	**ENSG0000011427**	FGF12	protein_coding	chr3	192139390	192767764	-	Human GRCh	OTTHUMG000001	FGF12	**fibroblast growth factor 12**	Extracellular	other		-3.895240829	-1	0.0024468	1	0.96	0	5.64603504	5.9922296	4.71009405	4.64888392
4226	**ENSG0000011430**	PRKAR2A	protein_coding	chr3	48744597	48847874	-	Human GRCh	OTTHUMG000001	PRKAR2A	**protein kinase cAMP-dependent type II regulatory subunit alp**	Cytoplasm	kinase		-1.024144839	0	0.91120611	0	1	0	12.05092	12.3783014	12.3831861	11.9804334
4227	**ENSG0000011431**	HES1	protein_coding	chr3	194136148	194138732	+	Human GRCh	OTTHUMG000001	HES1	**hes family bHLH transcription factor 1**	Nucleus	transcription		-1.051185843	0	0.85104351	0	1	0	9.55325628	9.83786276	9.58982328	9.70121631
4228	**ENSG0000011431**	USP4	protein_coding	chr3	49277144	49340712	-	Human GRCh	OTTHUMG000001	USP4	**ubiquitin specific peptidase 4**	Nucleus	peptidase		-1.158659706	0	0.47818418	0	1	0	10.3095945	10.4931671	10.4140932	10.0494289
4229	**ENSG0000011433**	ACAP2	protein_coding	chr3	195274745	195443044	-	Human GRCh	OTTHUMG000001	ACAP2	**ArfGAP with coiled-coil, ankyrin repeat and PH domains 2**	Nucleus	other		1.084215432	0	0.71299036	0	1	0	10.9643831	11.1746975	11.350605	10.937251
4230	**ENSG0000011434**	ECT2	protein_coding	chr3	172750682	172824174	+	Human GRCh	OTTHUMG000001	ECT2	**epithelial cell transforming 2**	Cytoplasm	other		-1.214050112	0	0.41444682	0	1	0	11.6760159	12.0484861	11.8161323	11.4942038
4231	**ENSG0000011434**	GNAT1	protein_coding	chr3	50191610	50197696	+	Human GRCh	OTTHUMG000001	GNAT1	**G protein subunit alpha transducin 1**	Plasma Mem	enzyme		-1.66946234	-1	0.60501417	0	1	0	3.0619017	2.41801871	1.65413761	2.90580154
4232	**ENSG0000011435**	GNAI2	protein_coding	chr3	50226292	50259362	+	Human GRCh	OTTHUMG000001	GNAI2	**G protein subunit alpha i2**	Plasma Mem	enzyme		-1.002719856	0	0.98856341	0	1	0	10.4539804	10.6732035	10.6805168	10.4384869
4233	**ENSG0000011435**	TFG	protein_coding	chr3	100709295	100748964	+	Human GRCh	OTTHUMG000001	TFG	**trafficking from ER to golgi regulator**	Cytoplasm	other		-1.084511458	0	0.69401237	0	1	0	11.4998359	11.7666087	11.6818313	11.401891
4234	**ENSG0000011437**	USP9Y	protein_coding	chrY	12537650	12860839	+	Human GRCh	OTTHUMG000000	USP9Y	**ubiquitin specific peptidase 9 Y-linked**	Cytoplasm	peptidase		-1.173726058	0	0.50862942	0	1	0	10.5089896	10.6934609	10.5554582	10.2449245
4235	**ENSG0000011437**	HYAL1	protein_coding	chr3	50299890	50312381	-	Human GRCh	OTTHUMG000001	HYAL1	**hyaluronidase 1**	Cytoplasm	enzyme	hyaluronic	1.754183507	1	0.08519935	0	1	0	7.92711098	8.62007416	8.62702099	9.20473544
4236	**ENSG0000011438**	TUSC2	protein_coding	chr3	50320027	50328251	-	Human GRCh	OTTHUMG000001	TUSC2	**tumor suppressor 2, mitochondrial calcium regulator**	Nucleus	other		-1.08371465	0	0.73446514	0	1	0	9.48991027	9.58243872	9.48812811	9.40563173
4237	**ENSG0000011438**	NPRL2	protein_coding	chr3	50347330	50350826	-	Human GRCh	OTTHUMG000001	NPRL2	**NPR2 like, GATOR1 complex subunit**	Cytoplasm	other		-1.104832777	0	0.67750199	0	1	0	9.5650011	9.9945931	9.78062528	9.58786682
4238	**ENSG0000011439**	RPL24	protein_coding	chr3	101681091	101686733	-	Human GRCh	OTTHUMG000001	RPL24	**ribosomal protein L24**	Cytoplasm	other		1.067276127	0	0.72850009	0	1	0	12.4650423	12.7324707	12.6808324	12.6788249
4239	**ENSG0000011439**	CYB561D2	protein_coding	chr3	50350845	50358460	+	Human GRCh	OTTHUMG000001	CYB561D2	**cytochrome b561 family member D2**	Cytoplasm	enzyme		1.021420846	0	0.93709054	0	1	0	8.25925197	8.46516268	8.49331721	8.27634869
4240	**ENSG0000011440**	C3orf14	protein_coding	chr3	62319015	62336213	+	Human GRCh	OTTHUMG000001	CEP15	**centrosomal protein 15**	Other	other		1.741789897	1	0.34654838	0	1	0	4.47369722	4.60040288	4.94183764	5.24887192

4241	**ENSG0000011441**	FXR1	protein_coding	chr3	180868141	180982753	+	Human GRCh	OTTHUMG000001	FXR1	**FMR1 autosomal homolog 1**	Cytoplasm	other		1.020155196	0	0.91553232	0	1	0	11.507905	11.7639359	11.7963388	11.5167329
4242	**ENSG0000011442**	CBLB	protein_coding	chr3	105655461	105869552	-	Human GRCh	OTTHUMG000001	CBLB	**Cbl proto-oncogene B**	Nucleus	enzyme	NX-1607	-1.173993511	0	0.42599235	0	1	0	10.5555244	10.7472283	10.5711603	10.3735249
4243	**ENSG0000011443**	BBX	protein_coding	chr3	107522936	107811339	+	Human GRCh	OTTHUMG000001	BBX	**BBX high mobility group box domain containing**	Nucleus	transcription		-1.212842915	0	0.35267138	0	1	0	11.9620266	12.2897623	11.9830458	11.8588595
4244	**ENSG0000011444**	IFT57	protein_coding	chr3	108160812	108222435	-	Human GRCh	OTTHUMG000001	IFT57	**intraflagellar transport 57**	Cytoplasm	other		-1.106007443	0	0.64619958	0	1	0	8.76690591	9.13031525	8.95268959	8.7429106
4245	**ENSG0000011445**	GNB4	protein_coding	chr3	179396088	179451476	-	Human GRCh	OTTHUMG000001	GNB4	**G protein subunit beta 4**	Plasma Mem	other		-1.575793465	-1	0.64270741	0	1	0	3.64584539	2.90485738	3.27890391	2.76053251
4247	**ENSG0000011447**	IQCG	protein_coding	chr3	197889077	197960142	-	Human GRCh	OTTHUMG000001	IQCG	**IQ motif containing G**	Cytoplasm	other		-1.276646059	0	0.31765345	0	1	0	9.31217088	9.6230888	9.38075229	9.0105055
4248	**ENSG0000011448**	GBE1	protein_coding	chr3	81489703	81761645	-	Human GRCh	OTTHUMG000001	GBE1	**1,4-alpha-glucan branching enzyme 1**	Cytoplasm	enzyme		-1.030544009	0	0.90467091	0	1	0	9.88911233	10.5216149	10.2146936	10.2115317
4250	**ENSG0000011449**	UMPS	protein_coding	chr3	124730433	124749273	+	Human GRCh	OTTHUMG000001	UMPS	**uridine monophosphate synthetase**	Cytoplasm	enzyme	pyrazofuri	1.006393906	0	0.97610086	0	1	0	10.4593266	10.7632361	10.8415153	10.3673929
4251	**ENSG0000011450**	NCBP2	protein_coding	chr3	196935402	196942594	-	Human GRCh	OTTHUMG000001	NCBP2	**nuclear cap binding protein subunit 2**	Nucleus	other		1.077740364	0	0.7270819	0	1	0	10.3668129	10.4730081	10.7052817	10.2570344
4252	**ENSG0000011452**	SNX4	protein_coding	chr3	125446650	125520202	-	Human GRCh	OTTHUMG000001	SNX4	**sorting nexin 4**	Cytoplasm	transporter		1.057217346	0	0.79117825	0	1	0	9.75609106	10.0303606	10.167619	9.71221146
4253	**ENSG0000011452**	C3orf52	protein_coding	chr3	112086335	112131004	+	Human GRCh	OTTHUMG000001	C3orf52	**chromosome 3 open reading frame 52**	Plasma Mem	other		-1.48126529	0	0.55950588	0	1	0	11.9243197	12.1993989	11.9034857	11.1977085
4254	**ENSG0000011454**	FRMD4B	protein_coding	chr3	69168782	69542583	-	Human GRCh	OTTHUMG000001	FRMD4B	**FERM domain containing 4B**	Cytoplasm	other		-1.547134983	-1	0.27179989	0	1	0	6.44090167	6.83795465	6.36934577	6.0529481
4255	**ENSG0000011454**	SLC41A3	protein_coding	chr3	126006357	126101561	-	Human GRCh	OTTHUMG000001	SLC41A3	**solute carrier family 41 member 3**	Plasma Mem	transporter		-1.226808604	0	0.33807147	0	1	0	9.39895906	9.47510879	9.35787429	9.05465324
4256	**ENSG0000011454**	ROPN1B	protein_coding	chr3	125969160	125983454	+	Human GRCh	OTTHUMG000001	ROPN1B	**rhophilin associated tail protein 1B**	Cytoplasm	other		2.077151662	1	0.37252388	0	NA	0	2.38562234	2.70827634	2.68885744	3.23875213
4257	**ENSG0000011455**	PLXNA1	protein_coding	chr3	126982693	127037389	+	Human GRCh	OTTHUMG000000	PLXNA1	**plexin A1**	Plasma Mem	transmembra		-1.061302651	0	0.76104427	0	1	0	11.8302344	11.9984353	12.0329248	11.640649
4258	**ENSG0000011457**	ATP6V1A	protein_coding	chr3	113746770	113812056	+	Human GRCh	OTTHUMG000001	ATP6V1A	**ATPase H+ transporting V1 subunit A**	Plasma Mem	transporter	bafilomyci	-1.019007113	0	0.93155204	0	1	0	11.5721077	11.9611611	11.9429016	11.5469827
4259	**ENSG0000011462**	ABTB1	protein_coding	chr3	127672935	127680926	+	Human GRCh	OTTHUMG000001	ABTB1	**ankyrin repeat and BTB domain containing 1**	Cytoplasm	translation re		-1.107859586	0	0.64049204	0	1	0	8.31050098	8.41540705	8.20664765	8.30175423
4260	**ENSG0000011463**	PODXL2	protein_coding	chr3	127629185	127672802	+	Human GRCh	OTTHUMG000001	PODXL2	**podocalyxin like 2**	Plasma Mem	other		-1.011294052	0	0.95839234	0	1	0	11.1973934	11.5114314	11.4718019	11.2189857
4261	**ENSG0000011463**	UPK1B	protein_coding	chr3	119173517	119205143	+	Human GRCh	OTTHUMG000001	UPK1B	**uroplakin 1B**	Plasma Mem	other		-5.013120208	-1	0.58374092	0	NA	0	0.98066167	0.46395724	0.45994563	0.4409336
4262	**ENSG0000011464**	CSPG5	protein_coding	chr3	47562238	47580792	-	Human GRCh	OTTHUMG000001	CSPG5	**chondroitin sulfate proteoglycan 5**	Extracellular	growth facto		-1.006238081	0	0.98396687	0	1	0	8.26049364	8.74013125	8.56603942	8.46701439
4263	**ENSG0000011464**	KLHL18	protein_coding	chr3	47282917	47346816	+	Human GRCh	OTTHUMG000001	KLHL18	**kelch like family member 18**	Other	other		-1.036926155	0	0.84694589	0	1	0	10.3498155	10.6048897	10.553747	10.322237
4264	**ENSG0000011465**	SCAP	protein_coding	chr3	47413681	47477126	-	Human GRCh	OTTHUMG000001	SCAP	**SREBF chaperone**	Cytoplasm	other		-1.155111092	0	0.51470521	0	1	0	12.9835431	13.1485596	12.9484905	12.8556258
4266	**ENSG0000011465**	CFAP92	protein_coding	chr3	128909866	129002690	-	Human GRCh	OTTHUMG000001	CFAP92	**cilia and flagella associated protein 92 (putative)**	Other	other		-1.022737124	0	0.9420332	0	1	0	7.7201485	7.80082932	7.96958048	7.4462485
4267	**ENSG0000011467**	NEK11	protein_coding	chr3	131026850	131350465	+	Human GRCh	OTTHUMG000001	NEK11	**NIMA related kinase 11**	Nucleus	kinase	dabrafenit	-1.097684329	0	0.68908267	0	1	0	8.61657939	8.71639893	8.79267255	8.28904305
4268	**ENSG0000011468**	MRPL3	protein_coding	chr3	131462212	131502983	-	Human GRCh	OTTHUMG000001	MRPL3	**mitochondrial ribosomal protein L3**	Cytoplasm	other		-1.006678592	0	0.97409148	0	1	0	11.1208608	11.4341615	11.4099166	11.1349014
4269	**ENSG0000011469**	PLSCR4	protein_coding	chr3	146192335	146251179	-	Human GRCh	OTTHUMG000001	PLSCR4	**phospholipid scramblase 4**	Plasma Mem	enzyme		2.516679161	1	0.09916643	0	1	0	3.23397025	3.52075066	4.08231803	4.01366974
4270	**ENSG0000011473**	HEMK1	protein_coding	chr3	50569152	50596168	+	Human GRCh	OTTHUMG000001	HEMK1	**HemK methyltransferase family member 1**	Nucleus	enzyme		-1.000520648	0	0.99821067	0	1	0	10.2284776	10.3954347	10.4349685	10.1798616
4271	**ENSG0000011473**	CISH	protein_coding	chr3	50606489	50611774	-	Human GRCh	OTTHUMG000001	CISH	**cytokine inducible SH2 containing protein**	Cytoplasm	other		1.667527505	1	0.17210649	0	1	0	6.51826984	6.21169518	7.27587186	6.30666811
4272	**ENSG0000011473**	MAPKAPK3	protein_coding	chr3	50611520	50649291	+	Human GRCh	OTTHUMG000001	MAPKAPK3	**MAPK activated protein kinase 3**	Nucleus	kinase		1.016233993	0	0.93817983	0	1	0	9.99092133	10.4541169	10.3492933	10.1698121
4273	**ENSG0000011473**	ACVR2B	protein_coding	chr3	38453890	38493142	+	Human GRCh	OTTHUMG000001	ACVR2B	**activin A receptor type 2B**	Plasma Mem	kinase		-1.212138916	0	0.35803992	0	1	0	11.4442512	11.6355949	11.4955082	11.1446067
4274	**ENSG0000011474**	WDR48	protein_coding	chr3	39052013	39096671	+	Human GRCh	OTTHUMG000001	WDR48	**WD repeat domain 48**	Cytoplasm	other		-1.04581049	0	0.81714657	0	1	0	10.4910097	10.6582616	10.6458084	10.3972916
4275	**ENSG0000011474**	COMMD2	protein_coding	chr3	149738472	149752495	-	Human GRCh	OTTHUMG000001	COMMD2	**COMM domain containing 2**	Other	other		1.268444313	0	0.3100564	0	1	0	9.20375113	9.52338796	9.82102652	9.40115632
4276	**ENSG0000011474**	GORASP1	protein_coding	chr3	39095222	39108369	-	Human GRCh	OTTHUMG000001	GORASP1	**golgi reassembly stacking protein 1**	Cytoplasm	other		-1.024886595	0	0.90093527	0	1	0	10.0200383	10.3087852	10.3021135	9.96628203
4277	**ENSG0000011475**	PEX5L	protein_coding	chr3	179794958	180037053	-	Human GRCh	OTTHUMG000001	PEX5L	**peroxisomal biogenesis factor 5 like**	Cytoplasm	ion channel		1.225592394	0	0.59552285	0	1	0	4.81618255	5.57816503	5.25922397	5.49894889
4278	**ENSG0000011476**	RRP9	protein_coding	chr3	51933429	51941904	-	Human GRCh	OTTHUMG000001	RRP9	**ribosomal RNA processing 9, U3 small nucleolar RNA binding p**	Nucleus	other		1.125561576	0	0.56113858	0	1	0	9.69293586	10.1313245	10.1335399	9.99215004
4279	**ENSG0000011477**	ABCC5	protein_coding	chr3	183919934	184017939	-	Human GRCh	OTTHUMG000001	ABCC5	**ATP binding cassette subfamily C member 5**	Plasma Mem	transporter		-1.20007967	0	0.40828569	0	1	0	10.3768113	10.7017899	10.504284	10.1801534
4281	**ENSG0000011477**	ABHD14B	protein_coding	chr3	51968510	51983409	-	Human GRCh	OTTHUMG000001	ABHD14B	**abhydrolase domain containing 14B**	Cytoplasm	enzyme		1.010811828	0	0.96270273	0	1	0	9.73470118	9.85947686	9.82943923	9.79157249
4282	**ENSG0000011478**	EIF1B	protein_coding	chr3	40309707	40312424	+	Human GRCh	OTTHUMG000001	EIF1B	**eukaryotic translation initiation factor 1B**	Cytoplasm	translation re		1.063114392	0	0.78956551	0	1	0	8.80834277	9.00154035	9.04948341	8.89797996
4283	**ENSG0000011479**	ARHGEF26	protein_coding	chr3	154121003	154257827	+	Human GRCh	OTTHUMG000001	ARHGEF26	**Rho guanine nucleotide exchange factor 26**	Plasma Mem	other		-1.029780562	0	0.87997918	0	1	0	9.10207492	9.34521328	9.25263434	9.14225247
4284	**ENSG0000011479**	KLHL24	protein_coding	chr3	183635610	183684519	+	Human GRCh	OTTHUMG000001	KLHL24	**kelch like family member 24**	Plasma Mem	other		-1.334019717	0	0.24157354	0	1	0	11.994543	12.3499966	11.9899315	11.7390216
4285	**ENSG0000011480**	PLCH1	protein_coding	chr3	155575580	155745071	-	Human GRCh	OTTHUMG000001	PLCH1	**phospholipase C eta 1**	Cytoplasm	enzyme		-1.166335718	0	0.56493523	0	1	0	9.94670754	10.1428765	10.2777846	9.26216505
4286	**ENSG0000011481**	VIPR1	protein_coding	chr3	42489299	42537573	+	Human GRCh	OTTHUMG000001	VIPR1	**vasoactive intestinal peptide receptor 1**	Plasma Mem	G-protein cou		1.107631094	0	0.81464696	0	1	0	7.4622944	7.47918068	7.85606246	7.21212173
4287	**ENSG0000011484**	DNAH1	protein_coding	chr3	52316319	52400492	+	Human GRCh	OTTHUMG000001	DNAH1	**dynein axonemal heavy chain 1**	Extracellular	other		-1.237409975	0	0.47298656	0	1	0	9.62375163	9.76478689	9.65721705	9.23369931
4288	**ENSG0000011485**	SSR3	protein_coding	chr3	156539553	156555149	-	Human GRCh	OTTHUMG000001	SSR3	**signal sequence receptor subunit 3**	Cytoplasm	other		-1.137429963	0	0.56935666	0	1	0	12.6543561	12.9431167	12.8367614	12.4544482
4289	**ENSG0000011485**	ZBTB47	protein_coding	chr3	42653697	42667580	+	Human GRCh	OTTHUMG000001	ZBTB47	**zinc finger and BTB domain containing 47**	Other	other		-1.064347485	0	0.77051954	0	1	0	9.63525967	9.98125924	9.81791556	9.68043858
4290	**ENSG0000011485**	TNNC1	protein_coding	chr3	52451100	52454041	-	Human GRCh	OTTHUMG000001	TNNC1	**troponin C1, slow skeletal and cardiac type**	Cytoplasm	other	levosimen	2.471361701	1	0.21462817	0	1	0	3.14900797	3.55561526	4.01605019	3.8911369
4291	**ENSG0000011485**	NKTR	protein_coding	chr3	42600655	42648735	+	Human GRCh	OTTHUMG000001	NKTR	**natural killer cell triggering receptor**	Plasma Mem	enzyme		1.021616253	0	0.9250285	0	1	0	12.6189229	12.9357751	12.9697111	12.6286983
4292	**ENSG0000011485**	CLCN2	protein_coding	chr3	184346185	184361650	-	Human GRCh	OTTHUMG000001	CLCN2	**chloride voltage-gated channel 2**	Plasma Mem	ion channel	lubiprosto	-1.101610644	0	0.6850893	0	1	0	10.1217073	10.4097205	10.3385599	9.96205248
4293	**ENSG0000011486**	FOXP1	protein_coding	chr3	70954693	71583978	-	Human GRCh	OTTHUMG000001	FOXP1	**forkhead box P1**	Nucleus	transcription		1.056328752	0	0.80401545	0	1	0	11.3295908	11.4646568	11.5466331	11.365761
4294	**ENSG0000011486**	EIF4G1	protein_coding	chr3	184314495	184335358	+	Human GRCh	OTTHUMG000001	EIF4G1	**eukaryotic translation initiation factor 4 gamma 1**	Cytoplasm	translation re		-1.125340908	0	0.55676545	0	1	0	14.1886957	14.53392	14.3199492	14.1548571
4295	**ENSG0000011490**	SPCS1	protein_coding	chr3	52704955	52711148	+	Human GRCh	OTTHUMG000001	SPCS1	**signal peptidase complex subunit 1**	Cytoplasm	peptidase		-1.054466863	0	0.81195298	0	1	0	10.5409908	10.9086788	10.7434953	10.6120826
4296	**ENSG0000011490**	NEK4	protein_coding	chr3	52708444	52770946	-	Human GRCh	OTTHUMG000001	NEK4	**NIMA related kinase 4**	Nucleus	kinase		1.07193874	0	0.72337144	0	1	0	10.7459491	10.9761268	11.1083763	10.7517968
4297	**ENSG0000011492**	SLC4A3	protein_coding	chr2	219627394	219641980	+	Human GRCh	OTTHUMG000000	SLC4A3	**solute carrier family 4 member 3**	Plasma Mem	transporter		1.663243267	1	0.13569937	0	1	0	5.55274675	4.88345805	5.72167206	5.77736685
4298	**ENSG0000011493**	INO80D	protein_coding	chr2	205993721	206086303	-	Human GRCh	OTTHUMG000001	INO80D	**INO80 complex subunit D**	Nucleus	other		-1.162627808	0	0.44348014	0	1	0	11.560781	11.8445844	11.6596233	11.418331
4299	**ENSG0000011494**	EEF1B2	protein_coding	chr2	206159585	206162928	+	Human GRCh	OTTHUMG000001	EEF1B2	**eukaryotic translation elongation factor 1 beta 2**	Cytoplasm	translation re		-1.116388647	0	0.6163862	0	1	0	11.9613602	12.3801173	12.1677826	11.948952
4300	**ENSG0000011494**	ADAM23	protein_coding	chr2	206443532	206621127	+	Human GRCh	OTTHUMG000001	ADAM23	**ADAM metallopeptidase domain 23**	Plasma Mem	peptidase		-1.95372891	-1	0.16201671	0	1	0	5.08167603	5.27473106	4.65370694	4.57701446
4301	**ENSG0000011495**	DGUOK	protein_coding	chr2	73926826	73958961	+	Human GRCh	OTTHUMG000000	DGUOK	**deoxyguanosine kinase**	Cytoplasm	kinase	fluorine F	-1.026760297	0	0.90936119	0	1	0	9.28203452	9.73371388	9.58277791	9.41097438
4302	**ENSG0000011497**	MOB1A	protein_coding	chr2	74152528	74178898	-	Human GRCh	OTTHUMG000001	MOB1A	**MOB kinase activator 1A**	Plasma Mem	other		-1.023076418	0	0.91739934	0	1	0	11.2678881	11.4586956	11.5538948	11.0837037
4303	**ENSG0000011498**	KANSL3	protein_coding	chr2	96593170	96642787	-	Human GRCh	OTTHUMG000001	KANSL3	**KAT8 regulatory NSL complex subunit 3**	Nucleus	other		-1.103564344	0	0.65750126	0	1	0	11.2221426	11.4855836	11.4827639	10.9679913
4304	**ENSG0000011498**	LMAN2L	protein_coding	chr2	96705929	96740064	-	Human GRCh	OTTHUMG000001	LMAN2L	**lectin, mannose binding 2 like**	Cytoplasm	other		-1.052117176	0	0.80643439	0	1	0	9.09806603	9.3615259	9.36574306	8.96231717
4305	**ENSG0000011499**	RTKN	protein_coding	chr2	74425835	74442422	-	Human GRCh	OTTHUMG000001	RTKN	**rhotekin**	Cytoplasm	other		-1.038342891	0	0.84221962	0	1	0	11.1662491	11.3293263	11.3731591	11.0166211
4306	**ENSG0000011499**	TTL	protein_coding	chr2	112482156	112541739	+	Human GRCh	OTTHUMG000001	TTL	**tubulin tyrosine ligase**	Cytoplasm	enzyme		-1.047651047	0	0.81432881	0	1	0	10.9178966	11.2000824	11.1486285	10.8672968
4309	**ENSG0000011502**	PIKFYVE	protein_coding	chr2	208266255	208358746	+	Human GRCh	OTTHUMG000001	PIKFYVE	**phosphoinositide kinase, FYVE-type zinc finger containing**	Cytoplasm	kinase	APY0201, a	-1.193280777	0	0.39336105	0	1	0	11.2899341	11.6523087	11.3944267	11.1737781
4310	**ENSG0000011504**	KCNIP3	protein_coding	chr2	95297327	95386077	+	Human GRCh	OTTHUMG000001	KCNIP3	**potassium voltage-gated channel interacting protein 3**	Nucleus	transcription		3.021864958	1	0.01107198	1	1	0	4.62194117	4.71765469	5.97560175	4.95898435
4311	**ENSG0000011504**	FAHD2A	protein_coding	chr2	95402708	95416616	+	Human GRCh	OTTHUMG000001	FAHD2A	**fumarylacetoacetate hydrolase domain containing 2A**	Other	enzyme		1.116842405	0	0.64701295	0	1	0	9.01313136	9.2524037	9.357716	9.15395379
4312	**ENSG0000011505**	NCL	protein_coding	chr2	231453531	231483641	-	Human GRCh	OTTHUMG000001	NCL	**nucleolin**	Nucleus	other	AGRO 100,	1.021122612	0	0.91593293	0	1	0	13.7375872	14.1006223	14.0591748	13.8440344
4313	**ENSG0000011507**	ACTR1B	protein_coding	chr2	97655939	97664044	-	Human GRCh	OTTHUMG000001	ACTR1B	**actin related protein 1B**	Cytoplasm	other		-1.077178808	0	0.73904965	0	1	0	10.4860636	10.7697083	10.7065222	10.3804655
4314	**ENSG0000011508**	SLC35F5	protein_coding	chr2	113705011	113756693	-	Human GRCh	OTTHUMG000001	SLC35F5	**solute carrier family 35 member F5**	Other	other		1.023293536	0	0.91715697	0	1	0	11.1624743	11.5162244	11.6087534	11.0913052
4315	**ENSG0000011508**	ZAP70	protein_coding	chr2	97713576	97739862	+	Human GRCh	OTTHUMG000001	ZAP70	**zeta chain of T cell receptor associated protein kinase 70**	Plasma Mem	kinase		-1.128743353	0	0.87097168	0	1	0	4.20011847	3.62563072	3.89909701	3.82336036
4316	**ENSG0000011509**	ACTR3	protein_coding	chr2	113890063	113962596	+	Human GRCh	OTTHUMG000001	ACTR3	**actin related protein 3**	Plasma Mem	other		1.059460615	0	0.77295409	0	1	0	11.3729044	11.6562839	11.6861817	11.479784
4317	**ENSG0000011510**	STEAP3	protein_coding	chr2	119223831	119265652	+	Human GRCh	OTTHUMG000001	STEAP3	**STEAP3 metalloreductase**	Cytoplasm	transporter		1.052506923	0	0.8093931	0	1	0	10.3683123	10.7862908	10.756492	10.5377971
4318	**ENSG0000011510**	EPB41L5	protein_coding	chr2	120013077	120179119	+	Human GRCh	OTTHUMG000001	EPB41L5	**erythrocyte membrane protein band 4.1 like 5**	Plasma Mem	other		-1.052127829	0	0.82400496	0	1	0	9.63001651	9.97720575	9.93157293	9.56073679
4319	**ENSG0000011511**	TFCP2L1	protein_coding	chr2	121216587	121285202	-	Human GRCh	OTTHUMG000001	TFCP2L1	**transcription factor CP2 like 1**	Nucleus	transcription		2.017037896	1	0.00388037	1	1	0	7.8247686	7.90660384	8.4062911	8.6659584
4320	**ENSG0000011512**	SF3B6	protein_coding	chr2	24067586	24076373	-	Human GRCh	OTTHUMG000001	SF3B6	**splicing factor 3b subunit 6**	Nucleus	other		-1.051007507	0	0.83499491	0	1	0	9.96513757	10.2684069	10.1408285	9.99566159
4321	**ENSG0000011512**	TP53I3	protein_coding	chr2	24077433	24085861	-	Human GRCh	OTTHUMG000000	TP53I3	**tumor protein p53 inducible protein 3**	Cytoplasm	enzyme		-1.329636674	0	0.34290058	0	1	0	6.7229559	7.05600408	6.56900546	6.66012015
4322	**ENSG0000011513**	DNAJC27	protein_coding	chr2	24943636	24972094	-	Human GRCh	OTTHUMG000001	DNAJC27	**DnaJ heat shock protein family (Hsp40) member C27**	Cytoplasm	enzyme		-1.05792938	0	0.81283907	0	1	0	8.3555451	8.63770382	8.61508478	8.24307813
4323	**ENSG0000011513**	POMC	protein_coding	chr2	25160853	25168903	-	Human GRCh	OTTHUMG000000	POMC	**proopiomelanocortin**	Extracellular	other		1	0	1	0	1	0	1.63760334	1.73863025	1.72247006	1.64375931
4324	**ENSG0000011514**	STAM2	protein_coding	chr2	152116801	152175763	-	Human GRCh	OTTHUMG000001	STAM2	**signal transducing adaptor molecule 2**	Cytoplasm	other		-1.144581956	0	0.56610261	0	1	0	8.57597244	8.83618697	8.74769579	8.36494704
4325	**ENSG0000011515**	OTOF	protein_coding	chr2	26457203	26558756	-	Human GRCh	OTTHUMG000000	OTOF	**otoferlin**	Plasma Mem	other		1.572944572	1	0.74683242	0	1	0	2.06786907	0.82254044	1.70644546	2.07872439
4326	**ENSG0000011515**	GPD2	protein_coding	chr2	156435290	156613735	+	Human GRCh	OTTHUMG000001	GPD2	**glycerol-3-phosphate dehydrogenase 2**	Cytoplasm	enzyme		-1.216585783	0	0.48589059	0	1	0	9.0804608	9.55143317	9.43687368	8.71791469
4327	**ENSG0000011516**	CENPA	protein_coding	chr2	26764289	26801067	+	Human GRCh	OTTHUMG000000	CENPA	**centromere protein A**	Nucleus	other		-1.158709675	0	0.53477287	0	1	0	9.19254526	9.63155938	9.30979947	9.23555188
4329	**ENSG0000011517**	ACVR1	protein_coding	chr2	157736251	157876330	-	Human GRCh	OTTHUMG000001	ACVR1	**activin A receptor type 1**	Plasma Mem	kinase	itacnoserti	1.024576074	0	0.91578757	0	1	0	8.05313095	8.28974607	8.31378365	8.08137685
4330	**ENSG0000011518**	TANC1	protein_coding	chr2	158968640	159232659	+	Human GRCh	OTTHUMG000001	TANC1	**tetratricopeptide repeat, ankyrin repeat and coiled-coil contai**	Plasma Mem	other		-1.149290708	0	0.41853862	0	1	0	10.1972292	10.1566068	10.0304631	10.0195951

4331	**ENSG0000011519**	SLC30A3	protein_coding	chr2	27253684	27275817	-	Human GRCh	OTTHUMG000001	SLC30A3	**solute carrier family 30 member 3**	Plasma Mem	transporter		-1.114094408	0	0.91051079	0	1	0	10.2027968	10.5834485	10.4413607	10.0818815
4332	**ENSG0000011520**	MPV17	protein_coding	chr2	27309492	27325680	-	Human GRCh	OTTHUMG000000	MPV17	**mitochondrial inner membrane protein MPV17**	Cytoplasm	ion channel		-1.071140942	0	0.78108159	0	1	0	10.0347342	10.2960602	10.1872586	9.99385976
4333	**ENSG0000011520**	GTF3C2	protein_coding	chr2	27325849	27356999	-	Human GRCh	OTTHUMG000000	GTF3C2	**general transcription factor IIIC subunit 2**	Nucleus	transcription		-1.149566454	0	0.51081672	0	1	0	11.6035737	11.9579627	11.7591548	11.4994994
4334	**ENSG0000011521**	EIF2B4	protein_coding	chr2	27364352	27370338	-	Human GRCh	OTTHUMG000001	EIF2B4	**eukaryotic translation initiation factor 2B subunit delta**	Cytoplasm	translation re		-1.113496832	0	0.60839267	0	1	0	10.2563238	10.4078353	10.3504761	10.056354
4335	**ENSG0000011521**	NRBP1	protein_coding	chr2	27427790	27442259	+	Human GRCh	OTTHUMG000000	NRBP1	**nuclear receptor binding protein 1**	Nucleus	kinase		1.018140935	0	0.92487787	0	1	0	9.95891763	10.0432954	10.1538135	9.87164654
4336	**ENSG0000011522**	ITGB6	protein_coding	chr2	160099667	160200313	-	Human GRCh	OTTHUMG000001	ITGB6	**integrin subunit beta 6**	Plasma Mem	other		1	0	1	0	1	0	1.40357433	1.47472553	1.4631872	1.40784012
4337	**ENSG0000011522**	FNDC4	protein_coding	chr2	27491883	27495200	-	Human GRCh	OTTHUMG000000	FNDC4	**fibronectin type III domain containing 4**	Extracellular	other		4.170535685	1	0.01680023	1	1	0	3.3981568	3.84977335	4.91197175	4.55606249
4338	**ENSG0000011523**	ITGA4	protein_coding	chr2	181457202	181538940	+	Human GRCh	OTTHUMG000001	ITGA4	**integrin subunit alpha 4**	Plasma Mem	transmembra	natalizuma	5.234913261	1	0.11378293	0	1	0	1.63011722	1.72978459	2.22306302	3.03281242
4339	**ENSG0000011523**	PSMD14	protein_coding	chr2	161308425	161411717	+	Human GRCh	OTTHUMG000001	PSMD14	**proteasome 26S subunit, non-ATPase 14**	Cytoplasm	peptidase		-1.238433224	0	0.26262471	0	1	0	10.1017194	10.3725237	10.0960883	9.92024557
4340	**ENSG0000011523**	SNX17	protein_coding	chr2	27370496	27377535	+	Human GRCh	OTTHUMG000000	SNX17	**sorting nexin 17**	Cytoplasm	transporter		-1.079163246	0	0.69966128	0	1	0	10.8258276	11.043986	11.0466817	10.6243881
4341	**ENSG0000011523**	ASB3	protein_coding	chr2	53532672	53860160	-	Human GRCh	OTTHUMG000001	ASB3/GPR75-	**ankyrin repeat and SOCS box containing 3**	Cytoplasm	transcription		-1.130373468	0	0.63147293	0	1	0	8.82090356	9.10823441	9.0575067	8.5734693
4342	**ENSG0000011524**	PPM1G	protein_coding	chr2	27381195	27409591	-	Human GRCh	OTTHUMG000000	PPM1G	**protein phosphatase, Mg2+/Mn2+ dependent 1G**	Nucleus	phosphatase		-1.048808501	0	0.82317041	0	1	0	11.7616943	12.1598845	12.0212096	11.8158804
4343	**ENSG0000011525**	PDE1A	protein_coding	chr2	182139968	182523359	-	Human GRCh	OTTHUMG000001	PDE1A	**phosphodiesterase 1A**	Cytoplasm	enzyme		-1.407168802	0	0.59127277	0	1	0	3.71470474	3.86555213	3.43083993	3.66862804
4344	**ENSG0000011525**	REEP6	protein_coding	chr19	1491166	1497927	+	Human GRCh	OTTHUMG000001	REEP6	**receptor accessory protein 6**	Plasma Mem	other		1.302289	0	0.58488507	0	1	0	7.68614845	7.66568977	8.13680303	7.78225492
4345	**ENSG0000011525**	PCSK4	protein_coding	chr19	1481428	1490752	-	Human GRCh	OTTHUMG000001	PCSK4	**proprotein convertase subtilisin/kexin type 4**	Extracellular	peptidase		1.131249876	0	0.69075671	0	1	0	8.41766137	8.52779486	8.83293325	8.34079982
4347	**ENSG0000011526**	APC2	protein_coding	chr19	1446302	1473244	+	Human GRCh	OTTHUMG000001	APC2	**APC regulator of WNT signaling pathway 2**	Cytoplasm	enzyme		1.274225621	0	0.63730464	0	1	0	6.62832779	6.47009166	6.60263167	6.97773551
4348	**ENSG0000011526**	IFIH1	protein_coding	chr2	162267074	162318684	-	Human GRCh	OTTHUMG000001	IFIH1	**interferon induced with helicase C domain 1**	Nucleus	enzyme		1.072608364	0	0.86698712	0	1	0	4.34474119	4.85084049	4.72158516	4.63099499
4349	**ENSG0000011526**	RPS15	protein_coding	chr19	1438358	1440495	+	Human GRCh	OTTHUMG000001	RPS15	**ribosomal protein S15**	Cytoplasm	other		1.12517743	0	0.53536658	0	1	0	12.8834876	13.1587312	13.2081511	13.114579
4350	**ENSG0000011527**	GCA	protein_coding	chr2	162318840	162371595	+	Human GRCh	OTTHUMG000001	GCA	**grancalcin**	Cytoplasm	other		1.332407813	0	0.28889507	0	1	0	7.54627749	7.50661219	8.06883914	7.52022352
4351	**ENSG0000011527**	INO80B	protein_coding	chr2	74455087	74457944	+	Human GRCh	OTTHUMG000001	INO80B	**INO80 complex subunit B**	Nucleus	other		1.151547804	0	0.51347101	0	1	0	9.43909407	9.63305459	9.7848367	9.60341154
4352	**ENSG0000011527**	MOGS	protein_coding	chr2	74461057	74465410	-	Human GRCh	OTTHUMG000001	MOGS	**mannosyl-oligosaccharide glucosidase**	Cytoplasm	enzyme		-1.015203777	0	0.93827283	0	1	0	10.7420916	10.8899313	10.8933409	10.7016391
4353	**ENSG0000011528**	TTC31	protein_coding	chr2	74483073	74494559	+	Human GRCh	OTTHUMG000001	TTC31	**tetratricopeptide repeat domain 31**	Other	other		-1.055595867	0	0.78928435	0	1	0	10.1529078	10.2650221	10.2881849	9.99480004
4354	**ENSG0000011528**	NDUFS7	protein_coding	chr19	1383527	1395589	+	Human GRCh	OTTHUMG000001	NDUFS7	**NADH:ubiquinone oxidoreductase core subunit S7**	Cytoplasm	enzyme		1.027368805	0	0.90279643	0	1	0	10.7395052	11.0966482	11.0523844	10.8639184
4355	**ENSG0000011528**	PCGF1	protein_coding	chr2	74505043	74507696	-	Human GRCh	OTTHUMG000001	PCGF1	**polycomb group ring finger 1**	Nucleus	other		1.102136455	0	0.6641537	0	1	0	8.52649922	8.57790875	8.7930276	8.50796727
4356	**ENSG0000011529**	GRB14	protein_coding	chr2	164492417	164621482	-	Human GRCh	OTTHUMG000001	GRB14	**growth factor receptor bound protein 14**	Plasma Mem	other		-1.063520645	0	0.86460167	0	1	0	6.16735623	6.30992133	6.36993156	5.95260767
4357	**ENSG0000011529**	CLIP4	protein_coding	chr2	29097705	29199777	+	Human GRCh	OTTHUMG000000	CLIP4	**CAP-Gly domain containing linker protein family member 4**	Other	other		-1.135857731	0	0.66431328	0	1	0	8.79232024	9.07984994	9.05771735	8.46420731
4358	**ENSG0000011529**	TLX2	protein_coding	chr2	74513463	74517148	+	Human GRCh	OTTHUMG000001	TLX2	**T cell leukemia homeobox 2**	Nucleus	transcription		2.213301789	1	0.29261361	0	1	0	3.94781164	3.21088862	4.73024326	3.53235714
4359	**ENSG0000011530**	SPTBN1	protein_coding	chr2	54456317	54671446	+	Human GRCh	OTTHUMG000001	SPTBN1	**spectrin beta, non-erythrocytic 1**	Plasma Mem	other		-1.099846734	0	0.63415946	0	1	0	13.0726332	13.29863	13.1650105	12.9981205
4360	**ENSG0000011530**	AUP1	protein_coding	chr2	74526645	74529760	-	Human GRCh	OTTHUMG000001	AUP1	**AUP1 lipid droplet regulating VLDL assembly factor**	Cytoplasm	other		-1.094812665	0	0.59571372	0	1	0	11.3701061	11.5254791	11.3566708	11.3397351
4361	**ENSG0000011531**	RTN4	protein_coding	chr2	54972187	55112621	-	Human GRCh	OTTHUMG000001	RTN4	**reticulon 4**	Cytoplasm	other		1.277008807	0	0.24992674	0	1	0	10.429672	10.6005753	10.7473848	10.81713
4362	**ENSG0000011531**	HTRA2	protein_coding	chr2	74529596	74533350	+	Human GRCh	OTTHUMG000001	HTRA2	**HtrA serine peptidase 2**	Cytoplasm	peptidase		-1.133513024	0	0.56764411	0	1	0	9.85381407	10.386184	10.1673647	9.82564583
4363	**ENSG0000011531**	LOXL3	protein_coding	chr2	74532258	74555690	-	Human GRCh	OTTHUMG000001	LOXL3	**lysyl oxidase like 3**	Extracellular	enzyme		1.100855048	0	0.79341962	0	1	0	6.6272931	6.77772655	7.0555077	6.47218576
4364	**ENSG0000011532**	DOK1	protein_coding	chr2	74549026	74557551	+	Human GRCh	OTTHUMG000001	DOK1	**docking protein 1**	Plasma Mem	kinase		1.168735254	0	0.69165785	0	1	0	6.24628226	6.17918117	6.53206871	6.16690123
4365	**ENSG0000011533**	GALNT3	protein_coding	chr2	165747588	165794659	-	Human GRCh	OTTHUMG000001	GALNT3	**polypeptide N-acetylgalactosaminyltransferase 3**	Cytoplasm	enzyme		-1.136121235	0	0.63639538	0	1	0	7.82814186	8.03842144	7.88512042	7.70972894
4366	**ENSG0000011535**	POLE4	protein_coding	chr2	74958643	74970128	+	Human GRCh	OTTHUMG000001	POLE4	**DNA polymerase epsilon 4, accessory subunit**	Nucleus	enzyme	bortezomi	1.09474922	0	0.7007349	0	1	0	7.32589558	7.55167379	7.57544422	7.50028848
4368	**ENSG0000011535**	CCDC88A	protein_coding	chr2	55287842	55419895	-	Human GRCh	OTTHUMG000001	CCDC88A	**coiled-coil domain containing 88A**	Cytoplasm	other		1.193621102	0	0.55530752	0	1	0	9.37434016	9.31396682	9.43266971	9.59977898
4369	**ENSG0000011536**	ACADL	protein_coding	chr2	210187126	210225447	-	Human GRCh	OTTHUMG000001	ACADL	**acyl-CoA dehydrogenase long chain**	Cytoplasm	enzyme		-1.076768179	0	0.84243469	0	1	0	9.6895849	9.86010114	9.82165971	9.54280886
4370	**ENSG0000011536**	EVA1A	protein_coding	chr2	75469302	75569722	-	Human GRCh	OTTHUMG000001	EVA1A	**eva-1 homolog A, regulator of programmed cell death**	Plasma Mem	other		-6.771711187	-1	0.06787966	0	1	0	2.0600053	3.18210946	2.13778899	1.59418711
4371	**ENSG0000011536**	MRPL19	protein_coding	chr2	75646783	75690811	+	Human GRCh	OTTHUMG000001	MRPL19	**mitochondrial ribosomal protein L19**	Cytoplasm	other		1.053048711	0	0.81307814	0	1	0	10.5494142	10.8305619	10.9408055	10.5363819
4372	**ENSG0000011536**	LANCL1	protein_coding	chr2	210427131	210477652	-	Human GRCh	OTTHUMG000001	LANCL1	**LanC like glutathione S-transferase 1**	Plasma Mem	enzyme		-1.021217244	0	0.92449896	0	1	0	10.3696352	10.6584243	10.6406935	10.3403992
4373	**ENSG0000011536**	WDR75	protein_coding	chr2	189441446	189475552	+	Human GRCh	OTTHUMG000001	WDR75	**WD repeat domain 75**	Nucleus	other		1.042114769	0	0.84033426	0	1	0	10.6418322	10.9153449	11.0038134	10.6322343
4374	**ENSG0000011538**	EFEMP1	protein_coding	chr2	55865967	55924139	-	Human GRCh	OTTHUMG000001	EFEMP1	**EGF containing fibulin extracellular matrix protein 1**	Extracellular	enzyme		-1.340741029	0	0.56911491	0	1	0	5.03680583	5.48609375	4.88674352	5.16779669
4375	**ENSG0000011538**	REG1A	protein_coding	chr2	79120362	79123409	+	Human GRCh	OTTHUMG000001	REG1A	**regenerating family member 1 alpha**	Extracellular	growth facto		1	0	1	0	NA	0	0.90510496	0.92552341	0.92213422	0.90629682
4376	**ENSG0000011539**	FANCL	protein_coding	chr2	58159243	58241010	-	Human GRCh	OTTHUMG000001	FANCL	**FA complementation group L**	Nucleus	enzyme		1.081634731	0	0.74827216	0	1	0	11.3940176	11.4060566	11.627208	11.316856
4377	**ENSG0000011541**	FN1	protein_coding	chr2	215360440	215436073	-	Human GRCh	OTTHUMG000001	FN1	**fibronectin 1**	Extracellular	other	ocriplasmi	-1.136612768	0	0.67433898	0	1	0	9.80670841	10.159156	10.0573203	9.59855926
4378	**ENSG0000011541**	STAT1	protein_coding	chr2	190908460	191020960	-	Human GRCh	OTTHUMG000001	STAT1	**signal transducer and activator of transcription 1**	Nucleus	transcription		-1.030063585	0	0.89071766	0	1	0	10.4478678	10.8365837	10.7936248	10.429599
4379	**ENSG0000011541**	GLS	protein_coding	chr2	190880821	190965552	+	Human GRCh	OTTHUMG000001	GLS	**glutaminase**	Cytoplasm	enzyme	telaglenast	1.202306737	0	0.37752546	0	1	0	10.91275	11.0424033	11.3690628	10.9601369
4380	**ENSG0000011542**	PAPOLG	protein_coding	chr2	60756253	60802086	+	Human GRCh	OTTHUMG000001	PAPOLG	**poly(A) polymerase gamma**	Nucleus	enzyme		-1.037031618	0	0.85709179	0	1	0	10.2210315	10.4260491	10.4229437	10.1345343
4381	**ENSG0000011542**	DNAH6	protein_coding	chr2	84516455	84819589	+	Human GRCh	OTTHUMG000001	DNAH6	**dynein axonemal heavy chain 6**	Extracellular	other		1.052002122	0	0.8532253	0	1	0	8.35760241	8.45965535	8.65886196	8.23162074
4382	**ENSG0000011542**	PECR	protein_coding	chr2	215996329	216082955	-	Human GRCh	OTTHUMG000001	PECR	**peroxisomal trans-2-enoyl-CoA reductase**	Cytoplasm	enzyme		1.010411891	0	0.97495835	0	1	0	5.99902805	6.21869418	6.16991719	6.07421002
4383	**ENSG0000011544**	UNC50	protein_coding	chr2	98608579	98618515	+	Human GRCh	OTTHUMG000001	UNC50	**unc-50 inner nuclear membrane RNA binding protein**	Cytoplasm	other		1.032870464	0	0.88558471	0	1	0	8.76435911	9.03434762	9.09613465	8.76493388
4384	**ENSG0000011545**	IGFBP2	protein_coding	chr2	216632828	216664436	+	Human GRCh	OTTHUMG000001	IGFBP2	**insulin like growth factor binding protein 2**	Extracellular	other		-1.136693969	0	0.68311831	0	1	0	8.90666372	8.83736814	8.66122377	8.83805865
4385	**ENSG0000011545**	ELMOD3	protein_coding	chr2	85354394	85391752	+	Human GRCh	OTTHUMG000001	ELMOD3	**ELMO domain containing 3**	Other	other		-1.043106467	0	0.84535152	0	1	0	9.0147009	9.14827181	9.12598807	8.94150487
4386	**ENSG0000011546**	IGFBP5	protein_coding	chr2	216672105	216695549	-	Human GRCh	OTTHUMG000001	IGFBP5	**insulin like growth factor binding protein 5**	Extracellular	other		-1.943367727	-1	0.18861923	0	1	0	10.5985901	10.2916161	9.66675339	9.8483274
4387	**ENSG0000011546**	USP34	protein_coding	chr2	61187463	61471087	-	Human GRCh	OTTHUMG000001	USP34	**ubiquitin specific peptidase 34**	Extracellular	peptidase		-1.103313427	0	0.62471929	0	1	0	12.8877405	13.1417508	13.0970027	12.694644
4388	**ENSG0000011546**	EFHD1	protein_coding	chr2	232606017	232682776	+	Human GRCh	OTTHUMG000001	EFHD1	**EF-hand domain family member D1**	Cytoplasm	other		1.092394587	0	0.80451256	0	1	0	7.13038038	7.73016948	7.35535378	7.74419926
4390	**ENSG0000011548**	CCT4	protein_coding	chr2	61868085	61888671	-	Human GRCh	OTTHUMG000001	CCT4	**chaperonin containing TCP1 subunit 4**	Cytoplasm	other		1.00716603	0	0.9732954	0	1	0	11.8367126	12.1405147	12.1523945	11.8397496
4391	**ENSG0000011548**	GGCX	protein_coding	chr2	85544720	85561532	-	Human GRCh	OTTHUMG000001	GGCX	**gamma-glutamyl carboxylase**	Cytoplasm	enzyme	anisindion	-1.015284189	0	0.93715296	0	1	0	10.3937205	10.4924241	10.5815132	10.2502001
4393	**ENSG0000011550**	EHBP1	protein_coding	chr2	62673851	63046487	+	Human GRCh	OTTHUMG000001	EHBP1	**EH domain binding protein 1**	Cytoplasm	other		-1.085743849	0	0.68382692	0	1	0	11.0988025	11.2590338	11.2090888	10.960486
4394	**ENSG0000011550**	OTX1	protein_coding	chr2	63050057	63057836	+	Human GRCh	OTTHUMG000001	OTX1	**orthodenticle homeobox 1**	Nucleus	transcription		1.195356167	0	0.56973674	0	1	0	9.52991449	9.74444589	9.97303429	9.69356222
4395	**ENSG0000011551**	TXNDC9	protein_coding	chr2	99318982	99340702	-	Human GRCh	OTTHUMG000001	TXNDC9	**thioredoxin domain containing 9**	Cytoplasm	other		-1.046733811	0	0.82370634	0	1	0	8.69047792	8.96921828	8.99253492	8.54160391
4396	**ENSG0000011552**	COQ10B	protein_coding	chr2	197453493	197475310	+	Human GRCh	OTTHUMG000001	COQ10B	**coenzyme Q10B**	Cytoplasm	other		1.182185122	0	0.52631709	0	1	0	9.16073912	9.41667082	9.5937227	9.36089467
4398	**ENSG0000011552**	SF3B1	protein_coding	chr2	197388515	197435079	-	Human GRCh	OTTHUMG000001	SF3B1	**splicing factor 3b subunit 1**	Nucleus	other	H3B-8800	-1.096803251	0	0.66013699	0	1	0	13.147856	13.4649742	13.3948266	13.0001506
4399	**ENSG0000011552**	ST3GAL5	protein_coding	chr2	85837120	85905199	-	Human GRCh	OTTHUMG000001	ST3GAL5	**ST3 beta-galactoside alpha-2,3-sialyltransferase 5**	Cytoplasm	enzyme		2.06330465	1	0.05844709	0	1	0	6.03872557	6.09178319	6.43706895	7.07937772
4400	**ENSG0000011552**	CHST10	protein_coding	chr2	100391860	100417668	-	Human GRCh	OTTHUMG000001	CHST10	**carbohydrate sulfotransferase 10**	Cytoplasm	enzyme		-1.080095224	0	0.75545549	0	1	0	6.88791463	7.06503324	7.01354487	6.77449609
4401	**ENSG0000011553**	PDCL3	protein_coding	chr2	100562993	100576739	+	Human GRCh	OTTHUMG000001	PDCL3	**phosducin like 3**	Cytoplasm	other		1.218391796	0	0.38866245	0	1	0	7.76751594	7.97249912	8.13513863	8.03315187
4402	**ENSG0000011554**	MOB4	protein_coding	chr2	197515571	197553699	+	Human GRCh	OTTHUMG000001	MOB4	**MOB family member 4, phocein**	Cytoplasm	other		1.181304545	0	0.47885276	0	1	0	10.2288944	10.5558661	10.8503696	10.2527124
4403	**ENSG0000011554**	HSPE1	protein_coding	chr2	197500140	197503449	+	Human GRCh	OTTHUMG000001	HSPE1	**heat shock protein family E (Hsp10) member 1**	Cytoplasm	enzyme		1.243886794	0	0.33714828	0	1	0	11.479051	11.7258897	11.9680425	11.7373314
4404	**ENSG0000011554**	KDM3A	protein_coding	chr2	86440647	86492716	+	Human GRCh	OTTHUMG000001	KDM3A	**lysine demethylase 3A**	Nucleus	transcription		-1.075489282	0	0.74620346	0	1	0	12.3372541	12.7186814	12.6348753	12.2584195
4405	**ENSG0000011555**	PLCD4	protein_coding	chr2	218607855	218637184	+	Human GRCh	OTTHUMG000001	PLCD4	**phospholipase C delta 4**	Cytoplasm	enzyme		2.720313022	1	0.14360857	0	1	0	3.17685645	2.98357282	3.88209344	3.41336935
4406	**ENSG0000011556**	CHMP3	protein_coding	chr2	86503430	86563479	-	Human GRCh	OTTHUMG000001	CHMP3	**charged multivesicular body protein 3**	Cytoplasm	other		-1.0975745	0	0.66388743	0	1	0	10.8282997	11.0928164	11.0482006	10.6490495
4407	**ENSG0000011556**	ZNF142	protein_coding	chr2	218633329	218659655	-	Human GRCh	OTTHUMG000001	ZNF142	**zinc finger protein 142**	Nucleus	transcription		-1.111787572	0	0.58771751	0	1	0	11.5978825	11.9139189	11.7541236	11.5297651
4411	**ENSG0000011559**	IL1R1	protein_coding	chr2	102064541	102179874	+	Human GRCh	OTTHUMG000001	IL1R1	**interleukin 1 receptor type 1**	Plasma Mem	transmembra	anakinra/c	-1.876129406	-1	0.13147285	0	1	0	6.510859	6.46473547	5.6379944	5.88642129
4412	**ENSG0000011559**	WNT6	protein_coding	chr2	218859805	218874233	+	Human GRCh	OTTHUMG000001	WNT6	**Wnt family member 6**	Extracellular	other		2.632413554	1	0.73888458	0	NA	0	0.4620016	0.47750491	0.4749377	0.72000568
4415	**ENSG0000011560**	IL18R1	protein_coding	chr2	102311529	102398777	+	Human GRCh	OTTHUMG000001	IL18R1	**interleukin 18 receptor 1**	Plasma Mem	transmembra		3.800733114	1	0.40451407	0	NA	0	0.95508056	0.71258528	0.70943839	1.4567928
4417	**ENSG0000011561**	SLC9A2	protein_coding	chr2	102619553	102711355	+	Human GRCh	OTTHUMG000001	SLC9A2	**solute carrier family 9 member A2**	Plasma Mem	transporter		-2.935904729	-1	0.09405357	0	1	0	4.31357856	4.60965571	3.43229229	3.76803546
4418	**ENSG0000011564**	FHL2	protein_coding	chr2	105357712	105438513	-	Human GRCh	OTTHUMG000001	FHL2	**four and a half LIM domains 2**	Nucleus	transcription		-1.221180179	0	0.78886638	0	1	0	3.6481956	3.73285315	3.7780495	3.26713396
4419	**ENSG0000011564**	MLPH	protein_coding	chr2	237485428	237555322	+	Human GRCh	OTTHUMG000001	MLPH	**melanophilin**	Cytoplasm	other		-1.096182692	0	0.78909684	0	1	0	12.4981047	12.5863912	12.5871905	12.2585788
4420	**ENSG0000011564**	CNPPD1	protein_coding	chr2	219171897	219178106	-	Human GRCh	OTTHUMG000001	CNPPD1	**cyclin Pas1/PHO80 domain containing 1**	Other	other		-1.076403262	0	0.72733964	0	1	0	10.7449615	10.9875719	10.8839704	10.6878607
4421	**ENSG0000011565**	UXS1	protein_coding	chr2	106093308	106194301	-	Human GRCh	OTTHUMG000001	UXS1	**UDP-glucuronate decarboxylase 1**	Cytoplasm	enzyme		-1.099780582	0	0.65790035	0	1	0	9.22097377	9.75419406	9.52744505	9.2868351
4422	**ENSG0000011565**	ABCB6	protein_coding	chr2	219209772	219218994	-	Human GRCh	OTTHUMG000001	ABCB6	**ATP binding cassette subfamily B member 6 (Langereis blood g**	Cytoplasm	transporter		-1.135264208	0	0.54924035	0	1	0	11.2281316	11.5071702	11.443711	10.9714484
4423	**ENSG0000011566**	STK16	protein_coding	chr2	219245455	219250337	+	Human GRCh	OTTHUMG000001	STK16	**serine/threonine kinase 16**	Cytoplasm	kinase	SM1-71	-1.169480244	0	0.46741786	0	1	0	8.94149367	9.15188801	8.96369764	8.78918561
4425	**ENSG0000011567**	HDLBP	protein_coding	chr2	241227264	241317061	-	Human GRCh	OTTHUMG000001	HDLBP	**high density lipoprotein binding protein**	Nucleus	transporter		-1.187232728	0	0.40980863	0	1	0	14.1224425	14.4989887	14.2294126	14.0183437

4426	**ENSG0000011568**	PPP1R7	protein_coding	chr2	241149576	241183652	+	Human GRCh	OTTHUMG000001	PPP1R7	**protein phosphatase 1 regulatory subunit 7**	Nucleus	phosphatase		-1.039350911	0	0.87288141	0	1	0	9.63043401	10.0819459	9.85604727	9.81175127
4427	**ENSG0000011568**	PASK	protein_coding	chr2	241106099	241150264	-	Human GRCh	OTTHUMG000001	PASK	**PAS domain containing serine/threonine kinase**	Cytoplasm	kinase	BioE-1197	-1.028590131	0	0.89643822	0	1	0	10.6896641	11.0877168	10.9750117	10.7638669
4428	**ENSG0000011569**	STK25	protein_coding	chr2	241492670	241509730	-	Human GRCh	OTTHUMG000001	STK25	**serine/threonine kinase 25**	Cytoplasm	kinase		-1.099810611	0	0.6329441	0	1	0	11.7420501	12.0986862	12.0083926	11.6123552
4430	**ENSG0000011571**	PROC	protein_coding	chr2	127418427	127429242	+	Human GRCh	OTTHUMG000001	PROC	**protein C, inactivator of coagulation factors Va and VIIIa**	Extracellular	peptidase	argatrobar	4.089331831	1	0.10392293	0	1	0	3.00864068	2.04554756	3.56460835	3.49628929
4431	**ENSG0000011573**	ID2	protein_coding	chr2	8678845	8684461	+	Human GRCh	OTTHUMG000001	ID2	**inhibitor of DNA binding 2**	Nucleus	transcription		-1.040532295	0	0.91567975	0	1	0	8.90633456	9.03785628	8.85738184	8.99881603
4432	**ENSG0000011575**	TAF1B	protein_coding	chr2	9843443	9934416	+	Human GRCh	OTTHUMG000001	TAF1B	**TATA-box binding protein associated factor, RNA polymerase I**	Nucleus	transcription		1.154951422	0	0.51256218	0	1	0	8.94166574	9.24388523	9.47281867	8.99585574
4433	**ENSG0000011575**	HPCAL1	protein_coding	chr2	10302889	10427617	+	Human GRCh	OTTHUMG000000	HPCAL1	**hippocalcin like 1**	Cytoplasm	other		1.226127871	0	0.26073221	0	1	0	9.24741143	9.31091191	9.51926794	9.48356005
4434	**ENSG0000011575**	ODC1	protein_coding	chr2	10439968	10448327	-	Human GRCh	OTTHUMG000000	ODC1	**ornithine decarboxylase 1**	Cytoplasm	enzyme	tazarotene	-1.240252242	0	0.35443698	0	1	0	12.8017341	13.072145	12.6977278	12.7023056
4435	**ENSG0000011576**	BIRC6	protein_coding	chr2	32357023	32619571	+	Human GRCh	OTTHUMG000001	BIRC6	**baculoviral IAP repeat containing 6**	Cytoplasm	enzyme		-1.1063025	0	0.62848248	0	1	0	13.1871764	13.4898495	13.3727985	13.0797164
4436	**ENSG0000011576**	NOL10	protein_coding	chr2	10562347	10689987	-	Human GRCh	OTTHUMG000001	NOL10	**nucleolar protein 10**	Nucleus	other		-1.05228418	0	0.81114208	0	1	0	11.0814735	11.4955429	11.4031463	11.0728398
4437	**ENSG0000011576**	PLEKHB2	protein_coding	chr2	131104847	131353709	+	Human GRCh	OTTHUMG000001	PLEKHB2	**pleckstrin homology domain containing B2**	Other	other		-1.058601153	0	0.79102286	0	1	0	10.6020589	10.9211786	10.7850957	10.6272206
4438	**ENSG0000011580**	GORASP2	protein_coding	chr2	170928464	170967130	+	Human GRCh	OTTHUMG000001	GORASP2	**golgi reassembly stacking protein 2**	Cytoplasm	other		-1.096687964	0	0.6616775	0	1	0	10.4803881	10.8247803	10.6011582	10.5212619
4439	**ENSG0000011580**	STRN	protein_coding	chr2	36837698	36966536	-	Human GRCh	OTTHUMG000001	STRN	**striatin**	Cytoplasm	other		-1.11344876	0	0.59356868	0	1	0	11.3618078	11.5347092	11.3972375	11.2653711
4440	**ENSG0000011581**	CEBPZ	protein_coding	chr2	37201612	37231596	-	Human GRCh	OTTHUMG000001	CEBPZ	**CCAAT enhancer binding protein zeta**	Nucleus	transcription		-1.018313066	0	0.92971695	0	1	0	10.4612036	10.7606849	10.7114532	10.4775546
4441	**ENSG0000011582**	PRKD3	protein_coding	chr2	37250502	37324833	-	Human GRCh	OTTHUMG000001	PRKD3	**protein kinase D3**	Nucleus	kinase	SM1-71, kI	-1.061782056	0	0.79137072	0	1	0	10.0803387	10.3882757	10.2801728	10.07409
4442	**ENSG0000011582**	DCAF17	protein_coding	chr2	171434217	171485052	+	Human GRCh	OTTHUMG000001	DCAF17	**DDB1 and CUL4 associated factor 17**	Nucleus	other		1.172985287	0	0.52113256	0	1	0	8.41176931	8.76512549	9.0045639	8.47925509
4443	**ENSG0000011582**	QPCT	protein_coding	chr2	37342827	37373322	+	Human GRCh	OTTHUMG000001	QPCT	**glutaminyl-peptide cyclotransferase**	Cytoplasm	enzyme	varoglutan	-10.14183425	-1	0.17680293	0	NA	0	1.90012475	1.30889852	1.30650203	1.29541806
4444	**ENSG0000011583**	RAB3GAP1	protein_coding	chr2	135052289	135176667	+	Human GRCh	OTTHUMG000001	RAB3GAP1	**RAB3 GTPase activating protein catalytic subunit 1**	Cytoplasm	other		-1.11355821	0	0.58952949	0	1	0	9.72000836	9.94432469	9.82028956	9.61739297
4445	**ENSG0000011584**	SLC25A12	protein_coding	chr2	171783405	171999859	-	Human GRCh	OTTHUMG000001	SLC25A12	**solute carrier family 25 member 12**	Cytoplasm	transporter		1.027913701	0	0.91643323	0	1	0	8.01262696	8.59022489	8.53952213	8.16224441
4446	**ENSG0000011584**	RMDN2	protein_coding	chr2	37923187	38067142	+	Human GRCh	OTTHUMG000001	RMDN2	**regulator of microtubule dynamics 2**	Cytoplasm	other		1.377121837	0	0.37215724	0	1	0	4.78359836	5.00684478	5.33722444	4.99047606
4447	**ENSG0000011584**	DLX2	protein_coding	chr2	172099438	172102900	-	Human GRCh	OTTHUMG000001	DLX2	**distal-less homeobox 2**	Nucleus	transcription		-1.305382396	0	0.52340764	0	1	0	5.74495315	6.14728137	5.72173338	5.65403095
4448	**ENSG0000011585**	LCT	protein_coding	chr2	135787850	135837184	-	Human GRCh	OTTHUMG000001	LCT	**lactase**	Plasma Mem	enzyme		8.943321216	1	0.06187158	0	1	0	2.31552584	2.09953871	2.95567267	2.98228278
4449	**ENSG0000011586**	DARS1	protein_coding	chr2	135905881	135986100	-	Human GRCh	OTTHUMG000001	DARS1	**aspartyl-tRNA synthetase 1**	Cytoplasm	enzyme		-1.014461226	0	0.95103044	0	1	0	10.0666427	10.426813	10.3887121	10.0806874
4450	**ENSG0000011587**	SRSF7	protein_coding	chr2	38743599	38751494	-	Human GRCh	OTTHUMG000001	SRSF7	**serine and arginine rich splicing factor 7**	Nucleus	other		-1.037049031	0	0.86551152	0	1	0	11.3097053	11.4541071	11.5304277	11.1224988
4451	**ENSG0000011588**	SDC1	protein_coding	chr2	20200797	20225433	-	Human GRCh	OTTHUMG000000	SDC1	**syndecan 1**	Plasma Mem	enzyme	indatuxima	1.608876881	1	0.20273898	0	1	0	7.7727078	7.07556412	7.46056952	8.26069689
4452	**ENSG0000011589**	PLCL1	protein_coding	chr2	197804593	198572581	+	Human GRCh	OTTHUMG000001	PLCL1	**phospholipase C like 1 (inactive)**	Cytoplasm	enzyme	quinacrine	-1.163326137	0	0.76438833	0	1	0	4.68262632	5.15828867	4.50972823	5.03303194
4453	**ENSG0000011590**	SLC1A4	protein_coding	chr2	64988477	65023865	+	Human GRCh	OTTHUMG000001	SLC1A4	**solute carrier family 1 member 4**	Plasma Mem	transporter	riluzole	-1.371234644	0	0.17049299	0	1	0	10.7087823	10.4428108	10.42508	10.0151092
4454	**ENSG0000011590**	SOS1	protein_coding	chr2	38962206	39124345	-	Human GRCh	OTTHUMG000001	SOS1	**SOS Ras/Rac guanine nucleotide exchange factor 1**	Cytoplasm	other	MRTX0902	-1.049248805	0	0.82591433	0	1	0	11.3852557	11.726231	11.7105892	11.279982
4455	**ENSG0000011591**	KYNU	protein_coding	chr2	142877657	143055833	+	Human GRCh	OTTHUMG000001	KYNU	**kynureninase**	Cytoplasm	enzyme		-2.093824651	-1	0.58759454	0	1	0	1.5917864	2.2820622	1.64391743	1.9248346
4457	**ENSG0000011593**	WIPF1	protein_coding	chr2	174559572	174682916	-	Human GRCh	OTTHUMG000001	WIPF1	**WAS/WASL interacting protein family member 1**	Cytoplasm	other		1.112290739	0	0.83662844	0	1	0	5.15884319	4.58734521	4.47150758	5.32729389
4458	**ENSG0000011594**	ORC2	protein_coding	chr2	200908977	200963680	-	Human GRCh	OTTHUMG000001	ORC2	**origin recognition complex subunit 2**	Nucleus	other		-1.040310504	0	0.85534861	0	1	0	10.2653117	10.4053124	10.4627133	10.0968647
4459	**ENSG0000011594**	COX7A2L	protein_coding	chr2	42333546	42425088	-	Human GRCh	OTTHUMG000001	COX7A2L	**cytochrome c oxidase subunit 7A2 like**	Cytoplasm	enzyme		-1.046380165	0	0.83695365	0	1	0	9.59334496	9.9939022	9.92246827	9.57497911
4460	**ENSG0000011594**	PNO1	protein_coding	chr2	68157888	68176238	+	Human GRCh	OTTHUMG000001	PNO1	**partner of NOB1 homolog**	Nucleus	other		1.228032156	0	0.35841917	0	1	0	8.80983456	9.15250532	9.38224049	9.03430822
4461	**ENSG0000011594**	ORC4	protein_coding	chr2	147930396	148021604	-	Human GRCh	OTTHUMG000001	ORC4	**origin recognition complex subunit 4**	Nucleus	other		-1.120794329	0	0.62479151	0	1	0	9.28882507	9.62132304	9.57023166	9.06231626
4463	**ENSG0000011596**	RND3	protein_coding	chr2	150468195	150539011	-	Human GRCh	OTTHUMG000001	RND3	**Rho family GTPase 3**	Cytoplasm	enzyme		-2.190806064	-1	0.12850113	0	1	0	5.34583799	5.53945336	4.79936805	4.7828263
4464	**ENSG0000011596**	ATF2	protein_coding	chr2	175072250	175168382	-	Human GRCh	OTTHUMG000001	ATF2	**activating transcription factor 2**	Nucleus	transcription		-1.008425416	0	0.96812467	0	1	0	9.9018529	10.2201052	10.1398559	9.97965623
4465	**ENSG0000011597**	THADA	protein_coding	chr2	43230851	43596038	-	Human GRCh	OTTHUMG000001	THADA	**THADA armadillo repeat containing**	Cytoplasm	other		-1.037172268	0	0.85906071	0	1	0	9.88939524	10.1897444	10.2032317	9.77802103
4466	**ENSG0000011597**	AAK1	protein_coding	chr2	69457997	69674349	-	Human GRCh	OTTHUMG000001	AAK1	**AP2 associated kinase 1**	Cytoplasm	kinase	LP-935509	1.071929494	0	0.79506554	0	1	0	11.9251924	12.1911036	12.1940431	12.0918315
4467	**ENSG0000011599**	TRAK2	protein_coding	chr2	201377207	201451500	-	Human GRCh	OTTHUMG000001	TRAK2	**trafficking kinesin protein 2**	Plasma Mem	transporter		1.031865764	0	0.89613993	0	1	0	10.4824538	10.4745931	10.6559627	10.3501249
4468	**ENSG0000011599**	C2orf42	protein_coding	chr2	70149885	70248615	-	Human GRCh	OTTHUMG000001	C2orf42	**chromosome 2 open reading frame 42**	Nucleus	other		1.002649744	0	0.99215998	0	1	0	7.88873101	7.77560497	7.98950151	7.66228913
4469	**ENSG0000011600**	TIA1	protein_coding	chr2	70209444	70248660	-	Human GRCh	OTTHUMG000001	TIA1	**TIA1 cytotoxic granule associated RNA binding protein**	Nucleus	other		1.005459523	0	0.9805615	0	1	0	11.235727	11.5787474	11.6361524	11.1708905
4470	**ENSG0000011600**	PCYOX1	protein_coding	chr2	70257386	70281185	+	Human GRCh	OTTHUMG000001	PCYOX1	**prenylcysteine oxidase 1**	Cytoplasm	enzyme		1.084302286	0	0.72517245	0	1	0	9.81237067	10.1002371	10.048045	10.0603242
4471	**ENSG0000011601**	KISS1R	protein_coding	chr19	917287	921005	+	Human GRCh	OTTHUMG000001	KISS1R	**KISS1 receptor**	Plasma Mem	G-protein co		1.300443115	0	0.51253238	0	1	0	7.87396335	8.08198413	8.42971003	8.10748809
4472	**ENSG0000011601**	EPAS1	protein_coding	chr2	46293667	46386697	+	Human GRCh	OTTHUMG000001	EPAS1	**endothelial PAS domain protein 1**	Nucleus	transcription	DFF332, N	1.159008535	0	0.68087047	0	1	0	6.79836428	7.03020387	7.08328028	7.05415476
4473	**ENSG0000011601**	ARID3A	protein_coding	chr19	925781	975939	+	Human GRCh	OTTHUMG000001	ARID3A	**AT-rich interaction domain 3A**	Nucleus	transcription		-1.009667998	0	0.96759723	0	1	0	10.6138902	10.9301861	10.9340655	10.5835479
4474	**ENSG0000011603**	SUMO1	protein_coding	chr2	202206182	202238599	-	Human GRCh	OTTHUMG000001	SUMO1	**small ubiquitin like modifier 1**	Nucleus	enzyme		-1.077366554	0	0.73066477	0	1	0	10.4756297	10.7757818	10.639717	10.4591676
4475	**ENSG0000011603**	CD207	protein_coding	chr2	70830211	70835816	-	Human GRCh	OTTHUMG000001	CD207	**CD207 molecule**	Plasma Mem	other		1	0	1	0	1	0	1.00665248	1.07540671	1.06433858	1.01081023
4476	**ENSG0000011603**	GRIN3B	protein_coding	chr19	1000419	1009732	+	Human GRCh	OTTHUMG000001	GRIN3B	**glutamate ionotropic receptor NMDA type subunit 3B**	Plasma Mem	ion channel	dextromet	1.177218297	0	0.7705711	0	1	0	4.37459959	4.14543173	4.43323442	4.38596371
4477	**ENSG0000011603**	VAX2	protein_coding	chr2	70900576	70965373	+	Human GRCh	OTTHUMG000001	VAX2	**ventral anterior homeobox 2**	Nucleus	transcription		2.170763374	1	0.1557295	0	1	0	4.29237151	3.87359969	4.82853987	4.71533409
4478	**ENSG0000011603**	ATP6V1B1	protein_coding	chr2	70935900	70965431	+	Human GRCh	OTTHUMG000001	ATP6V1B1	**ATPase H+ transporting V1 subunit B1**	Cytoplasm	transporter		1.163892505	0	0.87736831	0	1	0	1.98853211	2.54961136	2.53201368	2.17055854
4479	**ENSG0000011604**	NFE2L2	protein_coding	chr2	177218667	177392756	-	Human GRCh	OTTHUMG000001	NFE2L2	**NFE2 like bZIP transcription factor 2**	Nucleus	transcription	omaveloxc	-1.226009852	0	0.31555559	0	1	0	10.2582736	10.3101103	10.1985943	9.91376199
4480	**ENSG0000011606**	MSH6	protein_coding	chr2	47695530	47810063	+	Human GRCh	OTTHUMG000001	MSH6	**mutS homolog 6**	Nucleus	enzyme		-1.070662372	0	0.76241887	0	1	0	10.9845522	11.3333666	11.2849521	10.8763516
4481	**ENSG0000011609**	PLEKHA3	protein_coding	chr2	178480457	178516463	+	Human GRCh	OTTHUMG000001	PLEKHA3	**pleckstrin homology domain containing A3**	Cytoplasm	other		-1.014704098	0	0.94944677	0	1	0	10.5031383	10.7157718	10.8125566	10.335466
4482	**ENSG0000011609**	SPR	protein_coding	chr2	72887382	72892158	+	Human GRCh	OTTHUMG000001	SPR	**sepiapterin reductase**	Cytoplasm	enzyme		-1.091766074	0	0.70871723	0	1	0	7.69478104	7.95981671	7.70321702	7.77673005
4483	**ENSG0000011610**	EPHA4	protein_coding	chr2	221418027	221574202	-	Human GRCh	OTTHUMG000001	EPHA4	**EPH receptor A4**	Plasma Mem	kinase		1.474207761	0	0.41665343	0	1	0	8.69201954	9.085816	9.23424301	9.41860836
4484	**ENSG0000011611**	PARD3B	protein_coding	chr2	204545475	205620162	+	Human GRCh	OTTHUMG000001	PARD3B	**par-3 family cell polarity regulator beta**	Plasma Mem	other		1.058399338	0	0.78519954	0	1	0	9.27260667	9.34537207	9.57877073	9.11970876
4485	**ENSG0000011612**	FARSB	protein_coding	chr2	222566899	222656092	-	Human GRCh	OTTHUMG000001	FARSB	**phenylalanyl-tRNA synthetase subunit beta**	Cytoplasm	enzyme		-1.128684995	0	0.55132536	0	1	0	11.652659	12.060577	11.8006863	11.6703562
4486	**ENSG0000011612**	ALMS1	protein_coding	chr2	73385758	73625166	+	Human GRCh	OTTHUMG000001	ALMS1	**ALMS1 centrosome and basal body associated protein**	Cytoplasm	other		-1.194796179	0	0.36654521	0	1	0	12.4889061	12.7357697	12.5457542	12.2902716
4487	**ENSG0000011612**	BCL9	protein_coding	chr1	147541501	147626216	+	Human GRCh	OTTHUMG000000	BCL9	**BCL9 transcription coactivator**	Nucleus	other		-1.154415803	0	0.46224406	0	1	0	11.1952128	11.493451	11.2823512	11.09726
4488	**ENSG0000011613**	PRRX1	protein_coding	chr1	170662728	170739421	+	Human GRCh	OTTHUMG000000	PRRX1	**paired related homeobox 1**	Nucleus	transcription		-2.031979217	-1	0.42828205	0	1	0	2.79747734	2.26414558	1.59416759	2.52870522
4489	**ENSG0000011613**	DHCR24	protein_coding	chr1	54849627	54887195	-	Human GRCh	OTTHUMG000000	DHCR24	**24-dehydrocholesterol reductase**	Cytoplasm	enzyme		-1.499777397	0	0.11236513	0	1	0	14.9729029	15.2243951	14.7129389	14.5402221
4490	**ENSG0000011613**	DNAJC16	protein_coding	chr1	15526813	15592379	+	Human GRCh	OTTHUMG000000	DNAJC16	**DnaJ heat shock protein family (Hsp40) member C16**	Cytoplasm	other		-1.249806977	0	0.26763193	0	1	0	10.7053872	10.9675663	10.6968147	10.4880138
4491	**ENSG0000011614**	MARK1	protein_coding	chr1	220528136	220664461	+	Human GRCh	OTTHUMG000000	MARK1	**microtubule affinity regulating kinase 1**	Cytoplasm	kinase		-1.550351595	-1	0.42560203	0	1	0	8.75386759	8.70293088	8.237635	8.37351993
4492	**ENSG0000011614**	TNR	protein_coding	chr1	175315194	175743595	-	Human GRCh	OTTHUMG000000	TNR	**tenascin R**	Plasma Mem	other		1.029591749	0	0.97924989	0	1	0	1.67816681	0.96082658	1.80476329	0.87144647
4493	**ENSG0000011615**	MORN1	protein_coding	chr1	2321253	2391707	-	Human GRCh	OTTHUMG000000	MORN1	**MORN repeat containing 1**	Other	other		-1.062886157	0	0.77632755	0	1	0	10.2758426	10.3901953	10.3328941	10.1976973
4494	**ENSG0000011615**	GPX7	protein_coding	chr1	52602371	52609051	+	Human GRCh	OTTHUMG000000	GPX7	**glutathione peroxidase 7**	Cytoplasm	enzyme		1	0	1	0	1	0	1.39366885	1.44367847	1.43552221	1.3966481
4495	**ENSG0000011616**	CACYBP	protein_coding	chr1	174999163	175012027	+	Human GRCh	OTTHUMG000000	CACYBP	**calcyclin binding protein**	Nucleus	other		-1.032699611	0	0.86191845	0	1	0	11.5351393	11.8106972	11.7608967	11.5163033
4496	**ENSG0000011617**	SCP2	protein_coding	chr1	52927276	53051698	+	Human GRCh	OTTHUMG000000	SCP2	**sterol carrier protein 2**	Cytoplasm	transporter		1.111741667	0	0.61505591	0	1	0	10.0128786	10.4529045	10.5211513	10.1956645
4498	**ENSG0000011618**	PAPPA2	protein_coding	chr1	176463171	176845601	+	Human GRCh	OTTHUMG000000	PAPPA2	**pappalysin 2**	Extracellular	peptidase		2.084673957	1	0.19154753	0	1	0	4.05214976	5.56350439	5.94281253	5.59469589
4499	**ENSG0000011619**	RALGPS2	protein_coding	chr1	178725165	178921842	+	Human GRCh	OTTHUMG000000	RALGPS2	**Ral GEF with PH domain and SH3 binding motif 2**	Other	other		-1.008194347	0	0.97379009	0	1	0	11.7008085	11.9395295	12.0350855	11.5494594
4501	**ENSG0000011619**	CEP104	protein_coding	chr1	3812086	3857396	-	Human GRCh	OTTHUMG000000	CEP104	**centrosomal protein 104**	Cytoplasm	other		-1.201728656	0	0.37967066	0	1	0	10.9842402	11.2760832	11.0764198	10.7767857
4502	**ENSG0000011619**	FAM20B	protein_coding	chr1	179025804	179076567	+	Human GRCh	OTTHUMG000000	FAM20B	**FAM20B glycosaminoglycan xylosylkinase**	Extracellular	enzyme		-1.091324066	0	0.65648163	0	1	0	11.3172146	11.6235681	11.4836833	11.2777468
4503	**ENSG0000011620**	TCEANC2	protein_coding	chr1	54053584	54112519	+	Human GRCh	OTTHUMG000000	TCEANC2	**transcription elongation factor A N-terminal and central doma**	Other	other		-1.245202066	0	0.29446016	0	1	0	9.606626	9.82403654	9.64420024	9.29077413
4504	**ENSG0000011620**	TMEM59	protein_coding	chr1	54026681	54053504	-	Human GRCh	OTTHUMG000000	TMEM59	**transmembrane protein 59**	Plasma Mem	peptidase		-1.174218132	0	0.46476398	0	1	0	10.9701017	11.1925736	11.0629101	10.7337976
4505	**ENSG0000011621**	LRRC42	protein_coding	chr1	53946085	53968168	+	Human GRCh	OTTHUMG000000	LRRC42	**leucine rich repeat containing 42**	Other	other		-1.152096031	0	0.58215443	0	1	0	8.74860854	9.09480998	8.90238	8.64366355
4506	**ENSG0000011621**	WRAP73	protein_coding	chr1	3630767	3652761	-	Human GRCh	OTTHUMG000000	WRAP73	**WD repeat containing, antisense to TP73**	Nucleus	other		-1.0779713	0	0.71069348	0	1	0	9.97739261	10.3154737	10.261415	9.85735235
4508	**ENSG0000011622**	MRPL37	protein_coding	chr1	54184041	54225464	+	Human GRCh	OTTHUMG000000	MRPL37	**mitochondrial ribosomal protein L37**	Cytoplasm	enzyme		-1.100951042	0	0.62740223	0	1	0	11.3772156	11.7551404	11.4993489	11.4460729
4509	**ENSG0000011623**	ICMT	protein_coding	chr1	6221193	6235972	-	Human GRCh	OTTHUMG000000	ICMT	**isoprenylcysteine carboxyl methyltransferase**	Cytoplasm	enzyme		-1.139389634	0	0.49656874	0	1	0	11.1078495	11.3652446	11.2408865	10.9427983
4510	**ENSG0000011625**	RPL22	protein_coding	chr1	6185020	6209389	-	Human GRCh	OTTHUMG000001	RPL22	**ribosomal protein L22**	Cytoplasm	translation re		1.155737474	0	0.4882379	0	1	0	11.7172533	11.9737646	12.1420005	11.8647121
4511	**ENSG0000011625**	CHD5	protein_coding	chr1	6101787	6180321	-	Human GRCh	OTTHUMG000000	CHD5	**chromodomain helicase DNA binding protein 5**	Nucleus	enzyme		-1.339146278	0	0.44517662	0	1	0	7.97229509	8.08856808	7.35570583	8.02208722
4512	**ENSG0000011626**	QSOX1	protein_coding	chr1	180154869	180204030	+	Human GRCh	OTTHUMG000000	QSOX1	**quiescin sulfhydryl oxidase 1**	Cytoplasm	enzyme		1.224215892	0	0.42915284	0	1	0	11.5254822	11.9076572	12.0280017	11.878492
4513	**ENSG0000011626**	STXBP3	protein_coding	chr1	108746674	108809523	+	Human GRCh	OTTHUMG000000	STXBP3	**syntaxin binding protein 3**	Plasma Mem	transporter		-1.072528896	0	0.7540002	0	1	0	9.61135617	9.75425809	9.8270152	9.34059661
4514	**ENSG0000011627**	PHF13	protein_coding	chr1	6613731	6624030	+	Human GRCh	OTTHUMG000000	PHF13	**PHD finger protein 13**	Nucleus	other		-1.017591306	0	0.94149181	0	1	0	10.0094073	10.391402	10.3394055	10.0339794

4515	**ENSG0000011628**	ERRFI1	protein_coding	chr1	8004404	8026309	-	Human GRCh	OTTHUMG000000	ERRFI1	**ERBB receptor feedback inhibitor 1**	Cytoplasm	other		-1.550201288	-1	0.20608043	0	1	0	13.3509396	13.5158869	12.8884671	12.944533
4516	**ENSG0000011628**	PARK7	protein_coding	chr1	7954291	7985505	+	Human GRCh	OTTHUMG000000	PARK7	**Parkinsonism associated deglycase**	Nucleus	enzyme		-1.142147351	0	0.65604139	0	1	0	11.3395369	11.7555734	11.519648	11.3059594
4517	**ENSG0000011629**	ELAPOR1	protein_coding	chr1	109113679	109206781	+	Human GRCh	OTTHUMG000000	ELAPOR1	**endosome-lysosome associated apoptosis and autophagy regu**	Plasma Mem	other		1.036276125	0	0.9404534	0	1	0	5.30508171	5.58609693	5.6343023	5.31701736
4518	**ENSG0000011632**	OPRD1	protein_coding	chr1	28812170	28871267	+	Human GRCh	OTTHUMG000000	OPRD1	**opioid receptor delta 1**	Plasma Mem	G-protein co	dextromet	1.022066336	0	0.96768512	0	1	0	4.29859988	3.34000435	3.92027595	3.99240815
4519	**ENSG0000011633**	AMPD2	protein_coding	chr1	109616104	109632051	+	Human GRCh	OTTHUMG000000	AMPD2	**adenosine monophosphate deaminase 2**	Cytoplasm	enzyme		-1.038469407	0	0.86118839	0	1	0	10.2370335	10.5691869	10.4954038	10.2336594
4520	**ENSG0000011635**	SRSF4	protein_coding	chr1	29147743	29181900	-	Human GRCh	OTTHUMG000000	SRSF4	**serine and arginine rich splicing factor 4**	Nucleus	other		-1.014510188	0	0.94071905	0	1	0	11.3690596	11.7022724	11.6407871	11.4116172
4521	**ENSG0000011635**	MECR	protein_coding	chr1	29192657	29230942	-	Human GRCh	OTTHUMG000000	MECR	**mitochondrial trans-2-enoyl-CoA reductase**	Cytoplasm	enzyme		-1.039863605	0	0.86198232	0	1	0	9.29008285	9.50170522	9.47150147	9.23195271
4522	**ENSG0000011639**	KCNC4	protein_coding	chr1	110210314	110283100	+	Human GRCh	OTTHUMG000000	KCNC4	**potassium voltage-gated channel subfamily C member 4**	Plasma Mem	ion channel		1.122427008	0	0.59974911	0	1	0	7.67043875	7.91853296	8.15330112	7.60623785
4523	**ENSG0000011640**	EDEM3	protein_coding	chr1	184690237	184754907	-	Human GRCh	OTTHUMG000000	EDEM3	**ER degradation enhancing alpha-mannosidase like protein 3**	Cytoplasm	enzyme		1.016990759	0	0.92914641	0	1	0	11.9756762	12.0981033	12.1926735	11.9045315
4524	**ENSG0000011645**	WDR77	protein_coding	chr1	111439890	111449256	-	Human GRCh	OTTHUMG000000	WDR77	**WD repeat domain 77**	Nucleus	transcription		-1.054369847	0	0.80206427	0	1	0	9.50034504	9.87400442	9.73827547	9.53838014
4525	**ENSG0000011645**	ATP5PB	protein_coding	chr1	111448864	111462773	+	Human GRCh	OTTHUMG000000	ATP5PB	**ATP synthase peripheral stalk-membrane subunit b**	Cytoplasm	transporter		-1.047910778	0	0.83765453	0	1	0	11.5768195	11.9130809	11.8189938	11.576186
4526	**ENSG0000011647**	RAP1A	protein_coding	chr1	111542218	111716691	+	Human GRCh	OTTHUMG000000	RAP1A	**RAP1A, member of RAS oncogene family**	Cytoplasm	enzyme		1.106123947	0	0.6732156	0	1	0	9.18298624	9.3165054	9.56138398	9.11994812
4527	**ENSG0000011647**	HDAC1	protein_coding	chr1	32292083	32333635	+	Human GRCh	OTTHUMG000000	HDAC1	**histone deacetylase 1**	Nucleus	enzyme	pyroxamid	-1.137056775	0	0.54840773	0	1	0	11.4560533	11.7057945	11.5546244	11.3263726
4528	**ENSG0000011648**	CAPZA1	protein_coding	chr1	112619805	112671616	+	Human GRCh	OTTHUMG000000	CAPZA1	**capping actin protein of muscle Z-line subunit alpha 1**	Cytoplasm	other		-1.021896143	0	0.91663285	0	1	0	11.8402476	12.2275463	12.0829524	11.9651866
4529	**ENSG0000011649**	S100PBP	protein_coding	chr1	32816767	32858875	+	Human GRCh	OTTHUMG000000	S100PBP	**S100P binding protein**	Nucleus	other		-1.085463904	0	0.70682073	0	1	0	10.756094	11.0369265	11.0123991	10.5807203
4530	**ENSG0000011651**	RNF19B	protein_coding	chr1	32936445	32964809	-	Human GRCh	OTTHUMG000000	RNF19B	**ring finger protein 19B**	Cytoplasm	enzyme		-1.130526112	0	0.5763725	0	1	0	9.24118257	9.44297637	9.18722206	9.24018415
4531	**ENSG0000011652**	SCAMP3	protein_coding	chr1	155255979	155262430	-	Human GRCh	OTTHUMG000000	SCAMP3	**secretory carrier membrane protein 3**	Cytoplasm	transporter		-1.098296803	0	0.67226356	0	1	0	11.3549952	11.7160341	11.5136088	11.3661518
4532	**ENSG0000011652**	TRIM62	protein_coding	chr1	33145399	33182059	-	Human GRCh	OTTHUMG000000	TRIM62	**tripartite motif containing 62**	Cytoplasm	enzyme		-1.029570195	0	0.90501193	0	1	0	7.86873256	8.09370105	7.82783017	8.06882772
4533	**ENSG0000011653**	ASH1L	protein_coding	chr1	155335268	155563162	-	Human GRCh	OTTHUMG000000	ASH1L	**ASH1 like histone lysine methyltransferase**	Nucleus	enzyme		-1.204853751	0	0.34574537	0	1	0	13.4865156	13.8447664	13.5711842	13.3610394
4534	**ENSG0000011654**	DLGAP3	protein_coding	chr1	34865436	34929650	-	Human GRCh	OTTHUMG000000	DLGAP3	**DLG associated protein 3**	Cytoplasm	other		2.169095287	1	0.0200973	1	1	0	4.85572996	5.0204976	5.43417716	5.65210482
4535	**ENSG0000011656**	SFPQ	protein_coding	chr1	35176378	35193446	-	Human GRCh	OTTHUMG000000	SFPQ	**splicing factor proline and glutamine rich**	Nucleus	other		1.098833178	0	0.65880315	0	1	0	13.6434757	13.90512	14.0687364	13.6668187
4536	**ENSG0000011657**	RHOU	protein_coding	chr1	228735479	228746664	+	Human GRCh	OTTHUMG000000	RHOU	**ras homolog family member U**	Cytoplasm	enzyme		1.125066107	0	0.67624732	0	1	0	10.1860247	10.1398691	10.5531595	9.96624096
4537	**ENSG0000011658**	GON4L	protein_coding	chr1	155749659	155859400	-	Human GRCh	OTTHUMG000000	GON4L	**gon-4 like**	Cytoplasm	transcription		-1.125840428	0	0.51554953	0	1	0	11.6886815	11.9119088	11.792051	11.5437015
4538	**ENSG0000011658**	ARHGEF2	protein_coding	chr1	155946851	156007070	-	Human GRCh	OTTHUMG000000	ARHGEF2	**Rho/Rac guanine nucleotide exchange factor 2**	Cytoplasm	other	paclitaxel/	-1.107965767	0	0.70046788	0	1	0	10.9661966	11.2111976	11.1146989	10.8347656
4539	**ENSG0000011658**	LAMTOR2	protein_coding	chr1	156054782	156058506	+	Human GRCh	OTTHUMG000000	LAMTOR2	**late endosomal/lysosomal adaptor, MAPK and MTOR activato**	Cytoplasm	other		1.186985528	0	0.40867993	0	1	0	8.39413415	8.56625019	8.75032884	8.58318393
4540	**ENSG0000011660**	MEF2D	protein_coding	chr1	156463727	156500779	-	Human GRCh	OTTHUMG000000	MEF2D	**myocyte enhancer factor 2D**	Nucleus	transcription		1.053660513	0	0.77099655	0	1	0	11.4175569	11.6558893	11.6004723	11.6028556
4541	**ENSG0000011664**	DOCK7	protein_coding	chr1	62454298	62688386	-	Human GRCh	OTTHUMG000000	DOCK7	**dedicator of cytokinesis 7**	Plasma Mem	other		-1.211228459	0	0.35707052	0	1	0	11.3975222	11.6077122	11.4445563	11.1224768
4542	**ENSG0000011664**	SRM	protein_coding	chr1	11054584	11060020	-	Human GRCh	OTTHUMG000000	SRM	**spermidine synthase**	Cytoplasm	enzyme		-1.078895162	0	0.71430429	0	1	0	11.4750402	11.9486792	11.6294277	11.6704172
4544	**ENSG0000011666**	FBXO2	protein_coding	chr1	11637018	11655785	-	Human GRCh	OTTHUMG000000	FBXO2	**F-box protein 2**	Cytoplasm	enzyme		-3.801031616	-1	0.14480032	0	1	0	5.53342318	6.5199823	5.02313842	4.14666538
4545	**ENSG0000011666**	FBXO6	protein_coding	chr1	11664200	11674354	+	Human GRCh	OTTHUMG000000	FBXO6	**F-box protein 6**	Cytoplasm	enzyme		1.2046417	0	0.59329809	0	1	0	6.91638457	6.86183462	6.99122619	7.18281635
4546	**ENSG0000011666**	C1orf21	protein_coding	chr1	184387029	184629019	+	Human GRCh	OTTHUMG000000	C1orf21	**chromosome 1 open reading frame 21**	Other	other		-1.053522462	0	0.8617745	0	1	0	12.3680716	12.7334	12.8364236	12.0443257
4547	**ENSG0000011666**	SWT1	protein_coding	chr1	185157080	185291781	+	Human GRCh	OTTHUMG000000	SWT1	**SWT1 RNA endoribonuclease homolog**	Extracellular	other		-1.150215809	0	0.59591389	0	1	0	8.63671912	8.89090938	8.7121284	8.5198655
4548	**ENSG0000011667**	MAD2L2	protein_coding	chr1	11658918	11691811	-	Human GRCh	OTTHUMG000000	MAD2L2	**mitotic arrest deficient 2 like 2**	Nucleus	enzyme		1.150584713	0	0.53478157	0	1	0	9.02658409	9.31909547	9.39686266	9.25355869
4549	**ENSG0000011667**	DNAJC6	protein_coding	chr1	65248219	65415871	+	Human GRCh	OTTHUMG000000	DNAJC6	**DnaJ heat shock protein family (Hsp40) member C6**	Cytoplasm	other		-1.158227029	0	0.57241931	0	1	0	9.20914643	9.08122394	9.11429118	8.87249261
4550	**ENSG0000011667**	LEPR	protein_coding	chr1	65420652	65641559	+	Human GRCh	OTTHUMG000000	LEPR	**leptin receptor**	Plasma Mem	transmembra	recombina	1.069813897	0	0.87138597	0	1	0	6.26065168	6.1199434	6.48212305	5.99572116
4551	**ENSG0000011667**	IVNS1ABP	protein_coding	chr1	185296388	185317273	-	Human GRCh	OTTHUMG000000	IVNS1ABP	**influenza virus NS1A binding protein**	Nucleus	other		-1.068395146	0	0.74824972	0	1	0	12.1146838	12.4096912	12.410968	11.9404589
4552	**ENSG0000011668**	KIAA2013	protein_coding	chr1	11919591	11926428	-	Human GRCh	OTTHUMG000000	KIAA2013	**KIAA2013**	Other	other		-1.026040495	0	0.90160204	0	1	0	11.0186653	11.2568498	11.2485347	10.9646689
4553	**ENSG0000011668**	MFN2	protein_coding	chr1	11980181	12015211	+	Human GRCh	OTTHUMG000000	MFN2	**mitofusin 2**	Cytoplasm	enzyme		-1.07594784	0	0.72126281	0	1	0	12.2094917	12.5803337	12.4613452	12.1766265
4555	**ENSG0000011669**	MIIP	protein_coding	chr1	12019466	12032045	+	Human GRCh	OTTHUMG000000	MIIP	**migration and invasion inhibitory protein**	Other	other		1.00720277	0	0.97322916	0	1	0	9.62690041	9.85588917	9.90357147	9.58463053
4556	**ENSG0000011669**	SMG7	protein_coding	chr1	183472216	183598246	+	Human GRCh	OTTHUMG000000	SMG7	**SMG7 nonsense mediated mRNA decay factor**	Nucleus	other		-1.125090866	0	0.51534075	0	1	0	12.0809	12.3837011	12.2014593	12.0147893
4557	**ENSG0000011670**	NCF2	protein_coding	chr1	183554461	183590905	-	Human GRCh	OTTHUMG000000	NCF2	**neutrophil cytosolic factor 2**	Cytoplasm	enzyme		5.762155109	1	0.4795423	0	NA	0	0.1742752	0.18143007	0.57999398	0.17468892
4559	**ENSG0000011670**	SLC35D1	protein_coding	chr1	66999350	67054148	-	Human GRCh	OTTHUMG000000	SLC35D1	**solute carrier family 35 member D1**	Cytoplasm	transporter		-1.022369547	0	0.92784243	0	1	0	8.63215496	8.64757181	8.82395045	8.36352239
4560	**ENSG0000011671**	PLA2G4A	protein_coding	chr1	186828949	186988981	+	Human GRCh	OTTHUMG000000	PLA2G4A	**phospholipase A2 group IVA**	Cytoplasm	enzyme	cytosolic p	-2.890648892	-1	0.65431602	0	1	0	0.64603841	1.18620039	0.69573092	0.64961081
4561	**ENSG0000011671**	GADD45A	protein_coding	chr1	67685201	67688334	+	Human GRCh	OTTHUMG000000	GADD45A	**growth arrest and DNA damage inducible alpha**	Nucleus	other		2.110163458	1	0.02331476	1	1	0	6.10833513	6.5396385	7.14099721	7.05194858
4563	**ENSG0000011672**	PRAMEF12	protein_coding	chr1	12773738	12777906	+	Human GRCh	OTTHUMG000000	PRAMEF12	**PRAME family member 12**	Nucleus	other		1.012093294	0	0.99815748	0	NA	0	-0.4676157	-0.7423562	-0.7427357	-0.4649435
4564	**ENSG0000011672**	WLS	protein_coding	chr1	68098473	68233120	-	Human GRCh	OTTHUMG000000	WLS	**Wnt ligand secretion mediator**	Cytoplasm	other		-1.067736249	0	0.83311944	0	1	0	9.46624123	9.75294602	9.57067909	9.51900956
4565	**ENSG0000011673**	PRDM2	protein_coding	chr1	13700188	13825079	+	Human GRCh	OTTHUMG000000	PRDM2	**PR/SET domain 2**	Nucleus	transcription		-1.108966185	0	0.57663299	0	1	0	10.9796897	11.2019881	11.1348282	10.806213
4566	**ENSG0000011674**	RGS2	protein_coding	chr1	192809039	192812275	+	Human GRCh	OTTHUMG000000	RGS2	**regulator of G protein signaling 2**	Nucleus	enzyme		-1.07452218	0	0.80910202	0	1	0	10.4236914	10.5185073	10.49655	10.2687084
4568	**ENSG0000011674**	RO60	protein_coding	chr1	193059454	193091777	+	Human GRCh	OTTHUMG000000	RO60	**Ro60, Y RNA binding protein**	Nucleus	other		1.211303037	0	0.39493913	0	1	0	11.916592	12.0979669	12.4645863	11.9157949
4570	**ENSG0000011675**	UCHL5	protein_coding	chr1	193012250	193060080	-	Human GRCh	OTTHUMG000000	UCHL5	**ubiquitin C-terminal hydrolase L5**	Cytoplasm	peptidase	VLX1570	1.113459385	0	0.60175363	0	1	0	11.2347521	11.6778538	11.7412834	11.4338623
4571	**ENSG0000011675**	BCAS2	protein_coding	chr1	114567557	114581629	-	Human GRCh	OTTHUMG000000	BCAS2	**BCAS2 pre-mRNA processing factor**	Nucleus	other		1.204344752	0	0.39099313	0	1	0	9.05323482	9.32016559	9.41025198	9.38542526
4572	**ENSG0000011675**	SRSF11	protein_coding	chr1	70205682	70253052	+	Human GRCh	OTTHUMG000000	SRSF11	**serine and arginine rich splicing factor 11**	Nucleus	other		1.030818782	0	0.88115119	0	1	0	12.0595848	12.3382152	12.4328217	12.0101501
4573	**ENSG0000011676**	CTH	protein_coding	chr1	70411218	70439851	+	Human GRCh	OTTHUMG000000	CTH	**cystathionine gamma-lyase**	Cytoplasm	enzyme		-1.053095867	0	0.8815279	0	1	0	6.98001739	7.3156954	7.2162974	6.97614879
4574	**ENSG0000011677**	AGMAT	protein_coding	chr1	15571699	15585051	-	Human GRCh	OTTHUMG000000	AGMAT	**agmatinase**	Cytoplasm	enzyme		-5.330687227	-1	0.15785882	0	NA	0	2.72911013	2.74520613	2.35709436	1.97053945
4575	**ENSG0000011677**	OLFML3	protein_coding	chr1	113979391	114035572	+	Human GRCh	OTTHUMG000000	OLFML3	**olfactomedin like 3**	Extracellular	other		-7.597592615	-1	0.19406966	0	NA	0	2.09616163	1.75199414	1.58493077	1.38352651
4576	**ENSG0000011678**	TNNI3K	protein_coding	chr1	74235387	74544428	+	Human GRCh	OTTHUMG000001	TNNI3K	**TNNI3 interacting kinase**	Cytoplasm	kinase		-1.086906688	0	0.86609611	0	1	0	4.42276622	5.04369014	4.83259929	4.54858972
4577	**ENSG0000011678**	CFHR3	protein_coding	chr1	196774813	196795407	+	Human GRCh	OTTHUMG000000	CFHR3	**complement factor H related 3**	Extracellular	other		-5.59312723	-1	0.1926703	0	1	0	1.79463763	2.55515333	1.87434876	1.80040142
4578	**ENSG0000011678**	PLEKHM2	protein_coding	chr1	15684320	15734769	+	Human GRCh	OTTHUMG000000	PLEKHM2	**pleckstrin homology and RUN domain containing M2**	Extracellular	other		-1.071057657	0	0.73295526	0	1	0	10.1306186	10.3699948	10.3186181	10.027751
4579	**ENSG0000011679**	CRYZ	protein_coding	chr1	74705482	74733408	-	Human GRCh	OTTHUMG000000	CRYZ	**crystallin zeta**	Cytoplasm	enzyme		1.078489449	0	0.74988346	0	1	0	8.3557423	8.59334422	8.79200457	8.27526241
4580	**ENSG0000011679**	PHTF1	protein_coding	chr1	113696831	113759489	-	Human GRCh	OTTHUMG000000	PHTF1	**putative homeodomain transcription factor 1**	Nucleus	transcription		1.154615833	0	0.53970389	0	1	0	8.16379748	8.28504885	8.62985414	8.04392062
4581	**ENSG0000011680**	ZBTB17	protein_coding	chr1	15941869	15976132	-	Human GRCh	OTTHUMG000000	ZBTB17	**zinc finger and BTB domain containing 17**	Nucleus	transcription		-1.048090023	0	0.8233479	0	1	0	9.90112517	9.99199199	10.0105836	9.76831236
4582	**ENSG0000011681**	CD58	protein_coding	chr1	116514534	116571039	-	Human GRCh	OTTHUMG000000	CD58	**CD58 molecule**	Plasma Mem	transmembra		1.320613038	0	0.37725205	0	1	0	6.29599417	6.20494432	6.64139291	6.39019904
4583	**ENSG0000011681**	TFAP2E	protein_coding	chr1	35573314	35595328	+	Human GRCh	OTTHUMG000000	TFAP2E	**transcription factor AP-2 epsilon**	Nucleus	transcription		-1.012868656	0	0.97738575	0	1	0	6.24277896	6.56012821	6.73637119	5.91710324
4585	**ENSG0000011683**	TTF2	protein_coding	chr1	117060326	117107453	+	Human GRCh	OTTHUMG000000	TTF2	**transcription termination factor 2**	Cytoplasm	transcription		-1.130707728	0	0.53304812	0	1	0	11.3862454	11.7553958	11.5331031	11.3547067
4586	**ENSG0000011683**	NR5A2	protein_coding	chr1	200027614	200177420	+	Human GRCh	OTTHUMG000000	NR5A2	**nuclear receptor subfamily 5 group A member 2**	Nucleus	ligand-depen		-1.012047705	0	0.98068171	0	1	0	11.3110307	11.7170068	11.7042355	11.2950478
4587	**ENSG0000011685**	KIF21B	protein_coding	chr1	200969390	201023714	-	Human GRCh	OTTHUMG000000	KIF21B	**kinesin family member 21B**	Cytoplasm	other		-1.186683196	0	0.53659673	0	1	0	7.28833385	7.35006415	7.09109593	7.20550638
4588	**ENSG0000011685**	TMEM9	protein_coding	chr1	201134772	201171574	-	Human GRCh	OTTHUMG000000	TMEM9	**transmembrane protein 9**	Cytoplasm	other		-1.0786565	0	0.70861436	0	1	0	10.9088818	11.2581203	11.1350732	10.8749803
4589	**ENSG0000011686**	ADPRS	protein_coding	chr1	36088892	36093932	+	Human GRCh	OTTHUMG000000	ADPRS	**ADP-ribosylserine hydrolase**	Cytoplasm	enzyme		1.141900989	0	0.51786862	0	1	0	8.99241972	9.20100809	9.3802544	9.09651002
4590	**ENSG0000011687**	MAP7D1	protein_coding	chr1	36155579	36180849	+	Human GRCh	OTTHUMG000000	MAP7D1	**MAP7 domain containing 1**	Cytoplasm	other		-1.044628102	0	0.85999996	0	1	0	11.7970703	12.0327404	11.9159161	11.8240795
4591	**ENSG0000011687**	WARS2	protein_coding	chr1	119031216	119140654	-	Human GRCh	OTTHUMG000000	WARS2	**tryptophanyl tRNA synthetase 2, mitochondrial**	Cytoplasm	enzyme		-1.355848883	0	0.22345964	0	1	0	8.77312157	9.26683449	8.68570279	8.73719607
4592	**ENSG0000011688**	HAO2	protein_coding	chr1	119368779	119394130	+	Human GRCh	OTTHUMG000000	HAO2	**hydroxyacid oxidase 2**	Cytoplasm	enzyme		-1.417814231	0	0.75379683	0	NA	0	3.19647027	3.60081688	3.14590797	3.11778589
4593	**ENSG0000011688**	OSCP1	protein_coding	chr1	36415827	36450451	-	Human GRCh	OTTHUMG000000	OSCP1	**organic solute carrier partner 1**	Plasma Mem	transporter		-1.136230035	0	0.63877202	0	1	0	7.99552524	8.45770606	8.2165568	7.98896791
4594	**ENSG0000011689**	MRPS15	protein_coding	chr1	36455718	36464384	-	Human GRCh	OTTHUMG000000	MRPS15	**mitochondrial ribosomal protein S15**	Cytoplasm	other		-1.085396351	0	0.70824815	0	1	0	10.1982729	10.5350766	10.3559115	10.21282
4595	**ENSG0000011690**	EXOC8	protein_coding	chr1	231332753	231337852	-	Human GRCh	OTTHUMG000000	EXOC8	**exocyst complex component 8**	Plasma Mem	other		1.055123691	0	0.80574703	0	1	0	10.3830218	10.6936936	10.7839751	10.4029881
4596	**ENSG0000011690**	GNPAT	protein_coding	chr1	231241207	231277973	+	Human GRCh	OTTHUMG000000	GNPAT	**glyceronephosphate O-acyltransferase**	Cytoplasm	enzyme		-1.13094554	0	0.53010898	0	1	0	10.4418454	10.8392034	10.6973412	10.3124129
4597	**ENSG0000011691**	TSNAX	protein_coding	chr1	231528541	231566524	+	Human GRCh	OTTHUMG000000	TSNAX	**translin associated factor X**	Nucleus	other		1.195111705	0	0.45210043	0	1	0	10.7574489	11.0669861	11.3750768	10.7914795
4598	**ENSG0000011692**	C1orf109	protein_coding	chr1	37681570	37692249	-	Human GRCh	OTTHUMG000000	AIRIM	**AFG2 interacting ribosome maturation factor**	Nucleus	other		-1.097351044	0	0.65745356	0	1	0	9.96641414	10.333873	10.2723359	9.80292262
4599	**ENSG0000011695**	RRAGC	protein_coding	chr1	38838198	38859772	-	Human GRCh	OTTHUMG000000	RRAGC	**Ras related GTP binding C**	Cytoplasm	enzyme		-1.14952992	0	0.54411266	0	1	0	9.95178235	10.3384349	10.1531681	9.8352574
4600	**ENSG0000011696**	NID1	protein_coding	chr1	235975830	236065109	-	Human GRCh	OTTHUMG000000	NID1	**nidogen 1**	Extracellular	other		-1.300281471	0	0.36148534	0	1	0	8.38939904	8.37858379	8.00867531	8.22982074
4601	**ENSG0000011697**	LGALS8	protein_coding	chr1	236518000	236552981	+	Human GRCh	OTTHUMG000000	LGALS8	**galectin 8**	Extracellular	other		-1.275389144	0	0.33067859	0	1	0	10.9519485	11.3650081	11.0093183	10.8006405
4602	**ENSG0000011698**	NT5C1A	protein_coding	chr1	39651229	39672107	-	Human GRCh	OTTHUMG000000	NT5C1A	**5'-nucleotidase, cytosolic IA**	Cytoplasm	phosphatase		-1.500296954	-1	0.71026109	0	1	0	2.60959197	2.94661298	2.92720061	2.33787041
4603	**ENSG0000011698**	HPCAL4	protein_coding	chr1	39678648	39691485	-	Human GRCh	OTTHUMG000000	HPCAL4	**hippocalcin like 4**	Other	transporter		-1.61160077	-1	0.63154808	0	1	0	3.39727942	4.0256629	3.17603649	3.65909163
4604	**ENSG0000011698**	MTR	protein_coding	chr1	236795260	236921278	+	Human GRCh	OTTHUMG000000	MTR	**5-methyltetrahydrofolate-homocysteine methyltransferase**	Cytoplasm	enzyme	hydroxoco	-1.165778702	0	0.47908546	0	1	0	12.6342444	12.87778	12.7550136	12.3972365

4605	**ENSG0000011698**	BMP8B	protein_coding	chr1	39757182	39788865	-	Human GRCh	OTTHUMG000000	BMP8B	**bone morphogenetic protein 8b**	Extracellular	growth facto		1.545316955	1	0.15149095	0	1	0	6.72761608	6.3270313	7.22337066	6.67260594
4606	**ENSG0000011699**	MYCL	protein_coding	chr1	39895428	39902256	-	Human GRCh	OTTHUMG000000	MYCL	**MYCL proto-oncogene, bHLH transcription factor**	Nucleus	transcription		1.074823444	0	0.78329418	0	1	0	6.9957163	7.67077417	7.45080574	7.44316016
4607	**ENSG0000011699**	SIPA1L2	protein_coding	chr1	232397965	232630571	-	Human GRCh	OTTHUMG000000	SIPA1L2	**signal induced proliferation associated 1 like 2**	Other	other		1.38755261	0	0.2860712	0	1	0	11.843954	11.9779263	12.3635637	12.1984011
4609	**ENSG0000011700**	RLF	protein_coding	chr1	40161387	40240921	+	Human GRCh	OTTHUMG000000	RLF	**RLF zinc finger**	Nucleus	transcription		-1.011084936	0	0.95922085	0	1	0	10.6878701	11.0175323	11.0083999	10.6715847
4611	**ENSG0000011701**	ZNF684	protein_coding	chr1	40531573	40548167	+	Human GRCh	OTTHUMG000000	ZNF684	**zinc finger protein 684**	Nucleus	transcription		1.483388591	0	0.29948501	0	1	0	5.46988553	5.66502534	6.23433055	5.55489299
4612	**ENSG0000011701**	KCNQ4	protein_coding	chr1	40783787	40840452	+	Human GRCh	OTTHUMG000000	KCNQ4	**potassium voltage-gated channel subfamily Q member 4**	Plasma Mem	ion channel		1.222664321	0	0.48689136	0	1	0	6.91844709	7.40632118	7.45575445	7.3424146
4613	**ENSG0000011701**	RIMS3	protein_coding	chr1	40620680	40665682	-	Human GRCh	OTTHUMG000000	RIMS3	**regulating synaptic membrane exocytosis 3**	Plasma Mem	other		-1.209073635	0	0.70484578	0	1	0	3.72038769	4.10147564	3.24913843	4.10766946
4614	**ENSG0000011702**	AKT3	protein_coding	chr1	243488233	243851079	-	Human GRCh	OTTHUMG000000	AKT3	**AKT serine/threonine kinase 3**	Cytoplasm	kinase	triciribine,	1.630465189	1	0.44661546	0	1	0	5.64120689	4.16761696	5.94177886	4.92843474
4615	**ENSG0000011703**	ETV3	protein_coding	chr1	157121191	157138474	-	Human GRCh	OTTHUMG000000	ETV3	**ETS variant transcription factor 3**	Nucleus	transcription		1.089681469	0	0.65166634	0	1	0	10.4729868	10.7732035	10.8598592	10.5794841
4616	**ENSG0000011705**	ACADM	protein_coding	chr1	75724431	75787575	+	Human GRCh	OTTHUMG000000	ACADM	**acyl-CoA dehydrogenase medium chain**	Cytoplasm	enzyme		1.079501208	0	0.71914105	0	1	0	10.6303926	10.8603003	11.0545784	10.5643929
4617	**ENSG0000011706**	ST6GALNA	protein_coding	chr1	76867480	77067546	+	Human GRCh	OTTHUMG000000	ST6GALNAC5	**ST6 N-acetylgalactosaminide alpha-2,6-sialyltransferase 5**	Cytoplasm	enzyme		-2.611017355	-1	0.31220124	0	1	0	2.71313738	2.84426992	2.6642302	1.89298887
4620	**ENSG0000011711**	ADGRL2	protein_coding	chr1	81306147	81993932	+	Human GRCh	OTTHUMG000000	ADGRL2	**adhesion G protein-coupled receptor L2**	Plasma Mem	G-protein cou		1.064565868	0	0.83996914	0	1	0	9.72790431	9.79258828	9.96292781	9.68376631
4621	**ENSG0000011711**	PADI2	protein_coding	chr1	17066761	17119451	-	Human GRCh	OTTHUMG000000	PADI2	**peptidyl arginine deiminase 2**	Cytoplasm	enzyme		-2.06554646	-1	0.49291918	0	1	0	2.42455664	2.52031194	2.02859164	2.43025616
4622	**ENSG0000011711**	SDHB	protein_coding	chr1	17018664	17054032	-	Human GRCh	OTTHUMG000000	SDHB	**succinate dehydrogenase complex iron sulfur subunit B**	Cytoplasm	enzyme		-1.138896222	0	0.58163692	0	1	0	10.8737521	11.2903507	10.9649053	10.9415357
4623	**ENSG0000011712**	MFAP2	protein_coding	chr1	16974502	16980632	-	Human GRCh	OTTHUMG000000	MFAP2	**microfibril associated protein 2**	Extracellular	other		5.548858562	1	0.23672791	0	1	0	2.23442743	2.34359214	2.92560249	2.24106468
4624	**ENSG0000011713**	RPF1	protein_coding	chr1	84479259	84498352	+	Human GRCh	OTTHUMG000000	RPF1	**ribosome production factor 1 homolog**	Nucleus	other		-1.039429084	0	0.85566824	0	1	0	9.26795	9.63520411	9.5490917	9.28084949
4625	**ENSG0000011713**	KDM5B	protein_coding	chr1	202724495	202808487	-	Human GRCh	OTTHUMG000000	KDM5B	**lysine demethylase 5B**	Nucleus	enzyme	CPI-455	-1.137930902	0	0.61045317	0	1	0	12.0315324	12.3118611	12.2077185	11.8328972
4626	**ENSG0000011714**	UAP1	protein_coding	chr1	162561722	162601240	+	Human GRCh	OTTHUMG000000	UAP1	**UDP-N-acetylglucosamine pyrophosphorylase 1**	Nucleus	enzyme		-1.075684173	0	0.78882452	0	1	0	11.572644	11.9663709	11.8093152	11.5843525
4627	**ENSG0000011714**	ACTL8	protein_coding	chr1	17755333	17827063	+	Human GRCh	OTTHUMG000000	ACTL8	**actin like 8**	Cytoplasm	other		1.203827963	0	0.81527159	0	1	0	3.33483714	3.14641624	3.45705012	3.2739986
4628	**ENSG0000011715**	CTBS	protein_coding	chr1	84549611	84574480	-	Human GRCh	OTTHUMG000000	CTBS	**chitobiase**	Cytoplasm	enzyme		1.605748255	1	0.09972789	0	1	0	7.74035079	7.84311284	8.53531885	8.01737664
4630	**ENSG0000011715**	KLHL12	protein_coding	chr1	202891116	202928636	-	Human GRCh	OTTHUMG000000	KLHL12	**kelch like family member 12**	Cytoplasm	other		-1.002008341	0	0.99127984	0	1	0	10.0911458	10.3241127	10.3577181	10.0436827
4631	**ENSG0000011715**	IGSF21	protein_coding	chr1	18107798	18378483	+	Human GRCh	OTTHUMG000000	IGSF21	**immunoglobin superfamily member 21**	Extracellular	other		1	0	1	0	1	0	1.81179169	1.92911793	1.9104225	1.8189737
4632	**ENSG0000011715**	SSX2IP	protein_coding	chr1	84643706	84690803	-	Human GRCh	OTTHUMG000000	SSX2IP	**SSX family member 2 interacting protein**	Plasma Mem	other		1.09043537	0	0.69200537	0	1	0	10.6839255	10.8565127	11.059965	10.6418765
4633	**ENSG0000011717**	ZNHIT6	protein_coding	chr1	85649417	85708433	-	Human GRCh	OTTHUMG000000	ZNHIT6	**zinc finger HIT-type containing 6**	Cytoplasm	other		1.19302889	0	0.42131396	0	1	0	9.19904786	9.47757209	9.71255401	9.34005572
4635	**ENSG0000011722**	RBBP5	protein_coding	chr1	205086142	205122015	-	Human GRCh	OTTHUMG000000	RBBP5	**RB binding protein 5, histone lysine methyltransferase comple**	Nucleus	transcription		-1.086634548	0	0.65271609	0	1	0	10.11227	10.4986738	10.3601032	10.0833067
4636	**ENSG0000011722**	GBP3	protein_coding	chr1	89006679	89022874	-	Human GRCh	OTTHUMG000000	GBP3	**guanylate binding protein 3**	Cytoplasm	enzyme		1	0	1	0	NA	0	0.47739416	0.48913308	0.48717705	0.47807632
4637	**ENSG0000011722**	GBP1	protein_coding	chr1	89051882	89065360	-	Human GRCh	OTTHUMG000000	GBP1	**guanylate binding protein 1**	Cytoplasm	enzyme		1	0	1	0	NA	0	1.02823167	1.04384659	1.04123948	1.02913703
4639	**ENSG0000011724**	KIF17	protein_coding	chr1	20664014	20718017	-	Human GRCh	OTTHUMG000000	KIF17	**kinesin family member 17**	Cytoplasm	transporter		1.206226019	0	0.75706336	0	1	0	4.36856154	4.4589077	4.6819752	4.46095393
4640	**ENSG0000011726**	GPR89A	protein_coding	chr1	145607798	145670650	+	Human GRCh	OTTHUMG000000	GPR89A/GPR	**G protein-coupled receptor 89A**	Cytoplasm	ion channel		1.014673895	0	0.95073259	0	1	0	8.81098544	9.04780879	9.17020576	8.68237192
4641	**ENSG0000011726**	CDK18	protein_coding	chr1	205504596	205532793	+	Human GRCh	OTTHUMG000000	CDK18	**cyclin dependent kinase 18**	Cytoplasm	kinase		1.756176566	1	0.21352806	0	1	0	9.1372755	9.27608332	9.78736045	9.81935659
4642	**ENSG0000011728**	RAB29	protein_coding	chr1	205767986	205775482	-	Human GRCh	OTTHUMG000000	RAB29	**RAB29, member RAS oncogene family**	Plasma Mem	enzyme		1.174610498	0	0.50754275	0	1	0	9.04570926	9.21844621	9.42557349	9.19059572
4644	**ENSG0000011729**	ECE1	protein_coding	chr1	21217247	21345572	-	Human GRCh	OTTHUMG000000	ECE1	**endothelin converting enzyme 1**	Plasma Mem	peptidase		-1.103223505	0	0.69466763	0	1	0	9.78590017	9.90709084	9.69098498	9.80448959
4645	**ENSG0000011730**	HMGCL	protein_coding	chr1	23801885	23838620	-	Human GRCh	OTTHUMG000000	HMGCL	**3-hydroxy-3-methylglutaryl-CoA lyase**	Cytoplasm	enzyme		-1.186728528	0	0.46002334	0	1	0	9.11573208	9.25361319	9.09202559	8.8967712
4646	**ENSG0000011730**	GALE	protein_coding	chr1	23795599	23800781	-	Human GRCh	OTTHUMG000000	GALE	**UDP-galactose-4-epimerase**	Cytoplasm	enzyme		1.079663447	0	0.74893478	0	1	0	9.56411443	10.0134577	9.98682318	9.79785839
4647	**ENSG0000011731**	ID3	protein_coding	chr1	23557926	23559501	-	Human GRCh	OTTHUMG000000	ID3	**inhibitor of DNA binding 3**	Nucleus	transcription		2.443263228	1	0.04586808	1	1	0	5.6478922	5.48875414	5.98150023	6.7837224
4648	**ENSG0000011732**	CR2	protein_coding	chr1	207453024	207489895	+	Human GRCh	OTTHUMG000000	CR2	**complement C3d receptor 2**	Plasma Mem	transmembra		-6.903195128	-1	0.03474973	1	1	0	3.37793788	3.3300849	2.69617026	2.10679762
4649	**ENSG0000011733**	CD46	protein_coding	chr1	207752037	207795513	+	Human GRCh	OTTHUMG000000	CD46	**CD46 molecule**	Plasma Mem	transmembra	FOR46	-1.01605138	0	0.94380573	0	1	0	12.8399282	13.1047655	13.1982196	12.6661584
4650	**ENSG0000011736**	PRPF3	protein_coding	chr1	150321479	150353233	+	Human GRCh	OTTHUMG000000	PRPF3	**pre-mRNA processing factor 3**	Nucleus	other		-1.060436703	0	0.74952213	0	1	0	11.3096911	11.6183879	11.5578685	11.2402848
4651	**ENSG0000011736**	APH1A	protein_coding	chr1	150265399	150269580	-	Human GRCh	OTTHUMG000000	APH1A	**aph-1 homolog A, gamma-secretase subunit**	Cytoplasm	peptidase		1.00619816	0	0.97560807	0	1	0	11.7351155	11.9457258	11.9857008	11.7012223
4652	**ENSG0000011738**	P3H1	protein_coding	chr1	42746335	42767084	-	Human GRCh	OTTHUMG000000	P3H1	**prolyl 3-hydroxylase 1**	Nucleus	enzyme		-1.090164361	0	0.72302999	0	1	0	8.65508305	9.21625346	8.89419157	8.85462136
4653	**ENSG0000011739**	SLC2A1	protein_coding	chr1	42925353	42958893	-	Human GRCh	OTTHUMG000000	SLC2A1	**solute carrier family 2 member 1**	Plasma Mem	transporter	canakinum	-1.009481505	0	0.97501942	0	1	0	11.3321093	11.5419623	11.5485126	11.300637
4654	**ENSG0000011739**	EBNA1BP2	protein_coding	chr1	43164175	43270936	-	Human GRCh	OTTHUMG000000	EBNA1BP2	**EBNA1 binding protein 2**	Nucleus	other		-1.085534312	0	0.70469504	0	1	0	10.2016118	10.6921785	10.4253053	10.3311913
4655	**ENSG0000011739**	CDC20	protein_coding	chr1	43358981	43363203	+	Human GRCh	OTTHUMG000000	CDC20	**cell division cycle 20**	Nucleus	other		-1.191485731	0	0.46266402	0	1	0	11.5774342	11.9478026	11.6509256	11.5078562
4656	**ENSG0000011740**	MPL	protein_coding	chr1	43337818	43354466	+	Human GRCh	OTTHUMG000000	MPL	**MPL proto-oncogene, thrombopoietin receptor**	Plasma Mem	transmembra	eltrombop	1.035160577	0	0.94844821	0	1	0	4.58420568	4.73307053	4.85374191	4.50012576
4657	**ENSG0000011740**	ARTN	protein_coding	chr1	43933320	43937240	+	Human GRCh	OTTHUMG000000	ARTN	**artemin**	Extracellular	growth facto		1.127783934	0	0.76411955	0	1	0	7.0394828	7.31602222	7.30039492	7.32666555
4658	**ENSG0000011740**	IPO13	protein_coding	chr1	43946950	43968022	+	Human GRCh	OTTHUMG000000	IPO13	**importin 13**	Nucleus	transporter		-1.089819515	0	0.76715793	0	1	0	10.4339691	10.6572984	10.5386225	10.3676414
4659	**ENSG0000011741**	ATP6V0B	protein_coding	chr1	43974487	43978295	+	Human GRCh	OTTHUMG000000	ATP6V0B	**ATPase H+ transporting V0 subunit b**	Cytoplasm	transporter		1.037118675	0	0.86555716	0	1	0	11.3244801	11.6246257	11.6462561	11.3897046
4660	**ENSG0000011741**	B4GALT2	protein_coding	chr1	43978943	43991170	+	Human GRCh	OTTHUMG000000	B4GALT2	**beta-1,4-galactosyltransferase 2**	Cytoplasm	enzyme		-1.003606658	0	0.98634543	0	1	0	11.3148013	11.6237223	11.6022282	11.3329378
4661	**ENSG0000011741**	ERI3	protein_coding	chr1	44221070	44355260	-	Human GRCh	OTTHUMG000000	ERI3	**ERI1 exoribonuclease family member 3**	Plasma Mem	other		-1.045934641	0	0.81634241	0	1	0	10.5517052	10.7800559	10.7242695	10.510091
4662	**ENSG0000011742**	PTCH2	protein_coding	chr1	44819844	44843253	-	Human GRCh	OTTHUMG000000	PTCH2	**patched 2**	Plasma Mem	transmembra		1.079704864	0	0.82982934	0	1	0	7.37529213	7.67985026	7.65838906	7.58176801
4663	**ENSG0000011744**	AKR1A1	protein_coding	chr1	45550543	45570049	+	Human GRCh	OTTHUMG000000	AKR1A1	**aldo-keto reductase family 1 member A1**	Cytoplasm	enzyme	aldose red	-1.052639425	0	0.79004091	0	1	0	10.5852704	10.8794034	10.8418181	10.5045223
4664	**ENSG0000011745**	PRDX1	protein_coding	chr1	45510914	45542732	-	Human GRCh	OTTHUMG000000	PRDX1	**peroxiredoxin 1**	Cytoplasm	enzyme		-1.043361342	0	0.84732631	0	1	0	11.7013591	12.2341491	12.0671061	11.8197159
4665	**ENSG0000011746**	PIK3R3	protein_coding	chr1	46040140	46133036	-	Human GRCh	OTTHUMG000000	PIK3R3	**phosphoinositide-3-kinase regulatory subunit 3**	Cytoplasm	kinase		1.006585353	0	0.97711262	0	1	0	10.0229558	10.4089786	10.3884328	10.0671642
4666	**ENSG0000011747**	TSPAN1	protein_coding	chr1	46175073	46185962	+	Human GRCh	OTTHUMG000000	TSPAN1	**tetraspanin 1**	Cytoplasm	other		1.009256971	0	0.98734366	0	1	0	6.21860445	6.12544448	6.09379692	6.26287446
4667	**ENSG0000011747**	BLZF1	protein_coding	chr1	169367970	169396540	+	Human GRCh	OTTHUMG000000	BLZF1	**basic leucine zipper nuclear factor 1**	Cytoplasm	transcription		1.013510161	0	0.94968348	0	1	0	9.98903747	10.1934317	10.1887452	10.0267703
4668	**ENSG0000011747**	CCDC181	protein_coding	chr1	169394870	169460669	-	Human GRCh	OTTHUMG000000	CCDC181	**coiled-coil domain containing 181**	Nucleus	other		-2.039413746	-1	0.49100931	0	NA	0	2.85231848	2.73973204	2.72154871	2.05960254
4669	**ENSG0000011747**	SLC19A2	protein_coding	chr1	169463909	169485944	-	Human GRCh	OTTHUMG000000	SLC19A2	**solute carrier family 19 member 2**	Plasma Mem	transporter		-1.043984909	0	0.85917212	0	1	0	10.3351949	10.4934747	10.6614255	9.97981662
4670	**ENSG0000011748**	FAAH	protein_coding	chr1	46394317	46413848	+	Human GRCh	OTTHUMG000000	FAAH	**fatty acid amide hydrolase**	Plasma Mem	enzyme		1.056516665	0	0.80862349	0	1	0	8.95553306	9.24558407	9.18096091	9.15735015
4671	**ENSG0000011748**	NSUN4	protein_coding	chr1	46340789	46365152	+	Human GRCh	OTTHUMG000000	NSUN4	**NOP2/Sun RNA methyltransferase 4**	Cytoplasm	enzyme		-1.039686283	0	0.84031794	0	1	0	10.3884129	10.5823117	10.6116927	10.2540316
4672	**ENSG0000011750**	TMED5	protein_coding	chr1	93149742	93180516	-	Human GRCh	OTTHUMG000000	TMED5	**transmembrane p24 trafficking protein 5**	Cytoplasm	other		1.031375944	0	0.8903151	0	1	0	11.5199777	11.6602996	11.8013382	11.4235599
4674	**ENSG0000011750**	DR1	protein_coding	chr1	93345907	93369493	+	Human GRCh	OTTHUMG000000	DR1	**down-regulator of transcription 1**	Nucleus	transcription		1.051066679	0	0.79898397	0	1	0	11.0996929	11.2962362	11.4068633	11.0856916
4675	**ENSG0000011751**	CNN3	protein_coding	chr1	94896949	94927223	-	Human GRCh	OTTHUMG000000	CNN3	**calponin 3**	Cytoplasm	other		-1.094623225	0	0.69995932	0	1	0	12.5240996	12.7887535	12.7298958	12.3631106
4676	**ENSG0000011752**	PRRC2C	protein_coding	chr1	171485530	171593511	+	Human GRCh	OTTHUMG000000	PRRC2C	**proline rich coiled-coil 2C**	Cytoplasm	other		-1.11500707	0	0.54492311	0	1	0	14.0710364	14.3378443	14.1590986	14.0171631
4677	**ENSG0000011752**	F3	protein_coding	chr1	94529173	94541759	-	Human GRCh	OTTHUMG000000	F3	**coagulation factor III, tissue factor**	Plasma Mem	transmembra	MRG004A,	-1.39297222	0	0.52194268	0	1	0	5.62713786	6.16762555	5.4570861	5.72078215
4678	**ENSG0000011752**	ABCD3	protein_coding	chr1	94418389	94518666	+	Human GRCh	OTTHUMG000000	ABCD3	**ATP binding cassette subfamily D member 3**	Cytoplasm	transporter		-1.073090965	0	0.76049951	0	1	0	10.2542879	10.6001059	10.4675604	10.2489119
4679	**ENSG0000011753**	VAMP4	protein_coding	chr1	171700160	171742074	-	Human GRCh	OTTHUMG000000	VAMP4	**vesicle associated membrane protein 4**	Cytoplasm	other		1.074393023	0	0.7539551	0	1	0	8.70362944	8.96580831	9.09302093	8.714948
4680	**ENSG0000011754**	DPH5	protein_coding	chr1	100989623	101026088	-	Human GRCh	OTTHUMG000000	DPH5	**diphthamide biosynthesis 5**	Cytoplasm	enzyme		-1.098679909	0	0.69842016	0	1	0	9.61360112	9.77659908	9.79068712	9.35655403
4682	**ENSG0000011756**	PTBP2	protein_coding	chr1	96721665	96823738	+	Human GRCh	OTTHUMG000000	PTBP2	**polypyrimidine tract binding protein 2**	Nucleus	other		1.036647117	0	0.87194484	0	1	0	11.2094461	11.4042076	11.5911455	11.0460253
4683	**ENSG0000011758**	TNFSF4	protein_coding	chr1	173183731	173207331	-	Human GRCh	OTTHUMG000000	TNFSF4	**TNF superfamily member 4**	Extracellular	cytokine		-1.179053536	0	0.80864086	0	1	0	5.25856471	4.89688304	5.07476521	4.76107237
4684	**ENSG0000011759**	PRDX6	protein_coding	chr1	173477330	173488815	+	Human GRCh	OTTHUMG000000	PRDX6	**peroxiredoxin 6**	Cytoplasm	enzyme		-1.158414062	0	0.48756231	0	1	0	12.1165632	12.4664068	12.2802452	11.9737835
4685	**ENSG0000011759**	DARS2	protein_coding	chr1	173824653	173858808	+	Human GRCh	OTTHUMG000000	DARS2	**aspartyl-tRNA synthetase 2, mitochondrial**	Cytoplasm	enzyme		-1.235912131	0	0.34246059	0	1	0	11.430089	11.7360169	11.5324254	11.1485043
4687	**ENSG0000011759**	IRF6	protein_coding	chr1	209785617	209806175	-	Human GRCh	OTTHUMG000000	IRF6	**interferon regulatory factor 6**	Nucleus	transcription		-1.011089862	0	0.95165317	0	1	0	10.587108	10.8778012	10.8466124	10.5984478
4688	**ENSG0000011759**	UTP25	protein_coding	chr1	209827972	209857565	+	Human GRCh	OTTHUMG000000	UTP25	**UTP25 small subunit processome component**	Nucleus	other		-1.283304055	0	0.22115956	0	1	0	11.11648	11.3985248	11.0798049	10.888118
4689	**ENSG0000011759**	PLPPR5	protein_coding	chr1	98890245	99227929	-	Human GRCh	OTTHUMG000000	PLPPR5	**phospholipid phosphatase related 5**	Plasma Mem	other		-29.18587625	-1	0.06593926	0	NA	0	0.87267083	1.58913792	0.48851158	0.47978312
4690	**ENSG0000011760**	PLPPR4	protein_coding	chr1	99264292	99309590	+	Human GRCh	OTTHUMG000000	PLPPR4	**phospholipid phosphatase related 4**	Plasma Mem	phosphatase		2.631235873	1	0.24514171	0	1	0	2.44833954	2.79383872	3.30105061	2.89455611
4692	**ENSG0000011760**	RCAN3	protein_coding	chr1	24502351	24541040	+	Human GRCh	OTTHUMG000000	RCAN3	**RCAN family member 3**	Cytoplasm	other		1.343174999	0	0.37300364	0	1	0	9.18882429	9.27654138	9.73365646	9.27175716
4693	**ENSG0000011761**	SYF2	protein_coding	chr1	25222276	25232502	-	Human GRCh	OTTHUMG000000	SYF2	**SYF2 pre-mRNA splicing factor**	Nucleus	other		-1.061713334	0	0.77921115	0	1	0	9.22569655	9.39556102	9.37086876	9.11119314
4694	**ENSG0000011761**	RSRP1	protein_coding	chr1	25242249	25338213	-	Human GRCh	OTTHUMG000000	RSRP1	**arginine and serine rich protein 1**	Nucleus	other		1.12797452	0	0.62606345	0	1	0	10.2220004	10.3588886	10.6630075	10.1230906
4695	**ENSG0000011762**	SLC35A3	protein_coding	chr1	99969351	100035634	+	Human GRCh	OTTHUMG000000	SLC35A3	**solute carrier family 35 member A3**	Cytoplasm	transporter		1.100930695	0	0.72070854	0	1	0	10.2283204	10.5809228	10.7499702	10.2387385
4696	**ENSG0000011762**	RCOR3	protein_coding	chr1	211258377	211316385	+	Human GRCh	OTTHUMG000000	RCOR3	**REST corepressor 3**	Nucleus	transcription		-1.029194408	0	0.89581974	0	1	0	11.7211533	12.0138401	11.9159917	11.7688629
4697	**ENSG0000011763**	STMN1	protein_coding	chr1	25884181	25906991	-	Human GRCh	OTTHUMG000000	STMN1	**stathmin 1**	Cytoplasm	other		-1.130937476	0	0.55643491	0	1	0	13.1323949	13.4438708	13.3301146	12.9689526
4698	**ENSG0000011764**	MTFR1L	protein_coding	chr1	25818640	25832942	+	Human GRCh	OTTHUMG000000	MTFR1L	**mitochondrial fission regulator 1 like**	Other	other		-1.052529782	0	0.80350409	0	1	0	9.96133637	10.2809016	10.1592853	9.98410571
4699	**ENSG0000011764**	MAN1C1	protein_coding	chr1	25616791	25786206	+	Human GRCh	OTTHUMG000000	MAN1C1	**mannosidase alpha class 1C member 1**	Cytoplasm	enzyme		1.115155188	0	0.70334166	0	1	0	7.85085467	8.2359653	8.06389998	8.26524521

4700	**ENSG0000011765**	NEK2	protein_coding	chr1	211658657	211675630	-	Human GRCh	OTTHUMG000000	NEK2	**NIMA related kinase 2**	Cytoplasm	kinase		-1.16963794	0	0.49916182	0	1	0	10.6967037	11.0762004	10.8543133	10.583136
4701	**ENSG0000011767**	RPS6KA1	protein_coding	chr1	26529761	26575030	+	Human GRCh	OTTHUMG000000	RPS6KA1	**ribosomal protein S6 kinase A1**	Cytoplasm	kinase	APIO-EE-0	-1.07252043	0	0.743728	0	1	0	9.33149408	9.88262576	9.58537391	9.54329038
4702	**ENSG0000011768**	DHDDS	protein_coding	chr1	26432282	26471306	+	Human GRCh	OTTHUMG000000	DHDDS	**dehydrodolichyl diphosphate synthase subunit**	Cytoplasm	enzyme		-1.105650188	0	0.65347094	0	1	0	10.389546	10.7998811	10.5912283	10.4011761
4703	**ENSG0000011769**	NENF	protein_coding	chr1	212432920	212446379	+	Human GRCh	OTTHUMG000000	NENF	**neudesin neurotrophic factor**	Extracellular	growth facto		1.078174712	0	0.73907938	0	1	0	8.7612537	9.22578126	9.01741198	9.17171726
4704	**ENSG0000011769**	NSL1	protein_coding	chr1	212726153	212791782	-	Human GRCh	OTTHUMG000000	NSL1	**NSL1 component of MIS12 kinetochore complex**	Nucleus	other		1.011144955	0	0.96078056	0	1	0	10.4978024	10.7910159	10.8657037	10.4241618
4705	**ENSG0000011770**	PROX1	protein_coding	chr1	213983181	214041510	+	Human GRCh	OTTHUMG000000	PROX1	**prospero homeobox 1**	Nucleus	transcription		1.359933011	0	0.38216691	0	1	0	5.66337456	5.96710596	5.66643363	6.36828327
4706	**ENSG0000011771**	ARID1A	protein_coding	chr1	26693236	26782104	+	Human GRCh	OTTHUMG000000	ARID1A	**AT-rich interaction domain 1A**	Nucleus	transcription		-1.191590275	0	0.35865896	0	1	0	13.5041389	13.7432296	13.554149	13.3068125
4707	**ENSG0000011772**	CENPF	protein_coding	chr1	214603185	214664574	+	Human GRCh	OTTHUMG000000	CENPF	**centromere protein F**	Nucleus	other		-1.167263064	0	0.40328568	0	1	0	13.5029513	13.8398413	13.6193957	13.3979004
4708	**ENSG0000011774**	RPA2	protein_coding	chr1	27891524	27914746	-	Human GRCh	OTTHUMG000000	RPA2	**replication protein A2**	Nucleus	other		-1.028687201	0	0.90963622	0	1	0	9.82699271	10.2970325	10.2451744	9.82538619
4709	**ENSG0000011775**	PPP1R8	protein_coding	chr1	27830782	27851676	+	Human GRCh	OTTHUMG000000	PPP1R8	**protein phosphatase 1 regulatory subunit 8**	Nucleus	phosphatase		1.004772839	0	0.98254104	0	1	0	9.98925783	10.1960299	10.2657174	9.91284331
4710	**ENSG0000011775**	STX12	protein_coding	chr1	27773219	27824443	+	Human GRCh	OTTHUMG000000	STX12	**syntaxin 12**	Cytoplasm	other		-1.135769745	0	0.58084991	0	1	0	9.74596264	10.1207348	9.94307945	9.65279341
4711	**ENSG0000011779**	MTARC2	protein_coding	chr1	220748225	220784815	+	Human GRCh	OTTHUMG000000	MTARC2	**mitochondrial amidoxime reducing component 2**	Cytoplasm	enzyme		-1.121622155	0	0.68279086	0	1	0	8.35949619	8.72317925	8.55271242	8.27536557
4712	**ENSG0000011783**	SLC5A9	protein_coding	chr1	48222685	48248644	+	Human GRCh	OTTHUMG000000	SLC5A9	**solute carrier family 5 member 9**	Plasma Mem	transporter		1	0	1	0	1	0	1.47114864	1.55671217	1.54297649	1.47634066
4713	**ENSG0000011785**	OSBPL9	protein_coding	chr1	51577179	51798427	+	Human GRCh	OTTHUMG000000	OSBPL9	**oxysterol binding protein like 9**	Cytoplasm	transporter		-1.090022939	0	0.67908446	0	1	0	10.1573233	10.4121556	10.2767795	10.1132661
4714	**ENSG0000011786**	TXNDC12	protein_coding	chr1	52020131	52055191	-	Human GRCh	OTTHUMG000000	TXNDC12	**thioredoxin domain containing 12**	Cytoplasm	enzyme		-1.083612224	0	0.69778348	0	1	0	9.81116551	10.1674393	10.0540849	9.75664281
4715	**ENSG0000011786**	ESYT2	protein_coding	chr7	158730995	158830253	-	Human GRCh	OTTHUMG000001	ESYT2	**extended synaptotagmin 2**	Plasma Mem	other		-1.055027263	0	0.80277687	0	1	0	11.8309677	12.2250265	12.1868844	11.739282
4716	**ENSG0000011787**	POLR1G	protein_coding	chr19	45406644	45410737	+	Human GRCh	OTTHUMG000001	POLR1G	**RNA polymerase I subunit G**	Nucleus	other		1.131926881	0	0.5686836	0	1	0	9.72203339	10.0901103	10.1277844	9.99150528
4717	**ENSG0000011789**	MESD	protein_coding	chr15	80946289	80989828	-	Human GRCh	OTTHUMG000001	MESD	**mesoderm development LRP chaperone**	Extracellular	other		-1.030819436	0	0.87049631	0	1	0	10.556134	10.8397775	10.8620927	10.4464549
4718	**ENSG0000011790**	RCN2	protein_coding	chr15	76931738	76954393	+	Human GRCh	OTTHUMG000001	RCN2	**reticulocalbin 2**	Cytoplasm	other		-1.081607987	0	0.7149599	0	1	0	11.0519411	11.2456052	11.2621924	10.8294657
4719	**ENSG0000011797**	CHRNB4	protein_coding	chr15	78624111	78727754	-	Human GRCh	OTTHUMG000001	CHRNB4	**cholinergic receptor nicotinic beta 4 subunit**	Plasma Mem	transmembra	succinylch	-2.206323911	-1	0.13618262	0	1	0	4.35114351	4.09147168	2.68784984	4.05141422
4720	**ENSG0000011798**	MUC5B	protein_coding	chr11	1223066	1262172	+	Human GRCh	OTTHUMG000001	MUC5B	**mucin 5B, oligomeric mucus/gel-forming**	Extracellular	other		2.233207983	1	0.36648433	0	1	0	2.68602182	2.99231744	3.47695871	3.35049183
4721	**ENSG0000011798**	CTSD	protein_coding	chr11	1752752	1764573	-	Human GRCh	OTTHUMG000000	CTSD	**cathepsin D**	Cytoplasm	peptidase		1.00299297	0	0.99058029	0	1	0	11.8714463	12.1301174	12.0721641	11.9468707
4722	**ENSG0000011800**	COLEC11	protein_coding	chr2	3594832	3644644	+	Human GRCh	OTTHUMG000000	COLEC11	**collectin subfamily member 11**	Extracellular	other		1.364225777	0	0.40796932	0	1	0	5.97347084	5.81594307	6.3884784	5.96910094
4723	**ENSG0000011800**	STAG1	protein_coding	chr3	136336236	136752403	-	Human GRCh	OTTHUMG000001	STAG1	**STAG1 cohesin complex component**	Nucleus	other		-1.116926216	0	0.58393976	0	1	0	11.2688481	11.5855605	11.4528121	11.1643085
4725	**ENSG0000011804**	STK11	protein_coding	chr19	1177558	1228431	+	Human GRCh	OTTHUMG000001	STK11	**serine/threonine kinase 11**	Cytoplasm	kinase		1.086689603	0	0.68732319	0	1	0	10.8504982	11.199464	11.226944	11.02478
4726	**ENSG0000011805**	KMT2A	protein_coding	chr11	118436456	118526832	+	Human GRCh	OTTHUMG000001	KMT2A	**lysine methyltransferase 2A**	Nucleus	enzyme	ziftomenib	-1.180916715	0	0.40682797	0	1	0	13.3370772	13.5460559	13.3296523	13.193414
4727	**ENSG0000011809**	TREH	protein_coding	chr11	118657316	118679690	-	Human GRCh	OTTHUMG000001	TREH	**trehalase**	Plasma Mem	enzyme		-5.67693042	-1	0.21708402	0	1	0	1.71528889	2.32319338	1.75476913	1.71807608
4728	**ENSG0000011809**	IFT46	protein_coding	chr11	118544528	118572970	-	Human GRCh	OTTHUMG000001	IFT46	**intraflagellar transport 46**	Cytoplasm	other		-1.093562243	0	0.69201481	0	1	0	9.24769367	9.46112041	9.47906763	8.99388616
4731	**ENSG0000011815**	ZNF541	protein_coding	chr19	47520690	47573148	-	Human GRCh	OTTHUMG000001	ZNF541	**zinc finger protein 541**	Nucleus	transcription		1.246879194	0	0.65791951	0	1	0	4.43884139	4.50322499	4.58252187	4.70721576
4732	**ENSG0000011816**	SLC8A2	protein_coding	chr19	47428017	47471893	-	Human GRCh	OTTHUMG000001	SLC8A2	**solute carrier family 8 member A2**	Cytoplasm	transporter		1.098500741	0	0.83104884	0	1	0	5.00291801	5.78698823	5.79532871	5.23449491
4733	**ENSG0000011816**	KPTN	protein_coding	chr19	47475150	47484265	-	Human GRCh	OTTHUMG000001	KPTN	**kaptin, actin binding protein**	Plasma Mem	other		-1.017364982	0	0.94745011	0	1	0	6.69182463	6.86676047	6.93802079	6.56288363
4734	**ENSG0000011818**	RPS25	protein_coding	chr11	119015712	119018691	-	Human GRCh	OTTHUMG000001	RPS25	**ribosomal protein S25**	Cytoplasm	other		1.065980114	0	0.74428123	0	1	0	12.4089917	12.7619837	12.8146823	12.5079041
4735	**ENSG0000011819**	KIF14	protein_coding	chr1	200551497	200620751	-	Human GRCh	OTTHUMG000000	KIF14	**kinesin family member 14**	Cytoplasm	enzyme		-1.26308105	0	0.25048353	0	1	0	10.9425394	11.1761344	10.8688731	10.7466079
4736	**ENSG0000011819**	TNNT2	protein_coding	chr1	201359008	201377764	-	Human GRCh	OTTHUMG000000	TNNT2	**troponin T2, cardiac type**	Cytoplasm	other		-2.715410424	-1	0.12454571	0	1	0	3.9597413	5.17058179	4.15753235	3.6032847
4737	**ENSG0000011819**	DDX59	protein_coding	chr1	200623896	200669907	-	Human GRCh	OTTHUMG000000	DDX59	**DEAD-box helicase 59**	Other	enzyme		-1.168211921	0	0.42220584	0	1	0	9.16384733	9.35975591	9.10954497	9.08625208
4738	**ENSG0000011820**	CAMSAP2	protein_coding	chr1	200738893	200860704	+	Human GRCh	OTTHUMG000000	CAMSAP2	**calmodulin regulated spectrin associated protein family memb**	Cytoplasm	other		-1.040353597	0	0.82543686	0	1	0	11.7997268	12.019569	12.0353464	11.6782323
4739	**ENSG0000011821**	ATF6	protein_coding	chr1	161766298	161977574	+	Human GRCh	OTTHUMG000000	ATF6	**activating transcription factor 6**	Cytoplasm	transcription		-1.204645864	0	0.32373533	0	1	0	11.7138646	11.8250167	11.7239392	11.3756557
4741	**ENSG0000011824**	MREG	protein_coding	chr2	215942584	216034096	-	Human GRCh	OTTHUMG000001	MREG	**melanoregulin**	Cytoplasm	other		-1.140396375	0	0.62295157	0	1	0	7.04096611	7.30953732	7.15741958	6.91907528
4743	**ENSG0000011824**	FASTKD2	protein_coding	chr2	206765357	206796189	+	Human GRCh	OTTHUMG000001	FASTKD2	**FAST kinase domains 2**	Cytoplasm	other		-1.01657216	0	0.93964177	0	1	0	10.6408297	11.0070148	11.0233645	10.5734056
4744	**ENSG0000011825**	NRP2	protein_coding	chr2	205681990	205798133	+	Human GRCh	OTTHUMG000001	NRP2	**neuropilin 2**	Plasma Mem	kinase		-1.223370989	0	0.68631399	0	1	0	3.71310072	4.47803414	3.516515	4.31275315
4745	**ENSG0000011826**	CREB1	protein_coding	chr2	207529737	207605988	+	Human GRCh	OTTHUMG000001	CREB1	**cAMP responsive element binding protein 1**	Nucleus	transcription		1.142421627	0	0.53058146	0	1	0	10.7870027	11.0626643	11.2964998	10.8073063
4746	**ENSG0000011826**	KLF7	protein_coding	chr2	207074137	207173856	-	Human GRCh	OTTHUMG000001	KLF7	**KLF transcription factor 7**	Nucleus	transcription		-1.409735875	0	0.32732957	0	1	0	12.1645492	12.5168116	11.992644	11.9220312
4748	**ENSG0000011827**	B4GALT6	protein_coding	chr18	31622246	31685836	-	Human GRCh	OTTHUMG000001	B4GALT6	**beta-1,4-galactosyltransferase 6**	Cytoplasm	enzyme		-1.193117618	0	0.66686027	0	1	0	7.1904531	6.99278805	7.14188458	6.64313263
4749	**ENSG0000011829**	C1orf54	protein_coding	chr1	150268200	150280916	+	Human GRCh	OTTHUMG000000	C1orf54	**chromosome 1 open reading frame 54**	Other	other		1.924831915	1	0.19379171	0	1	0	4.71168909	5.24571558	6.02014247	5.0377047
4750	**ENSG0000011829**	CA14	protein_coding	chr1	150257251	150265078	+	Human GRCh	OTTHUMG000000	CA14	**carbonic anhydrase 14**	Plasma Mem	enzyme	hydrochlor	-5.316467216	-1	0.22771977	0	1	0	1.68434202	1.02064932	1.00875672	0.95111976
4751	**ENSG0000011830**	DNAI7	protein_coding	chr12	25108289	25195162	-	Human GRCh	OTTHUMG000001	DNAI7	**dynein axonemal intermediate chain 7**	Cytoplasm	other		-1.070974103	0	0.86785032	0	1	0	6.18308553	6.64746519	6.46320491	6.26309225
4752	**ENSG0000011830**	IRAG2	protein_coding	chr12	25004342	25108335	+	Human GRCh	OTTHUMG000001	IRAG2	**inositol 1,4,5-triphosphate receptor associated 2**	Cytoplasm	other		1	0	1	0	NA	0	1.09712234	1.11902876	1.11539215	1.0984009
4754	**ENSG0000011836**	SPCS2	protein_coding	chr11	74949261	74979033	+	Human GRCh	OTTHUMG000001	SPCS2	**signal peptidase complex subunit 2**	Cytoplasm	other		-1.024146887	0	0.92137761	0	1	0	10.1845267	10.445305	10.4801214	10.0769098
4755	**ENSG0000011836**	USP35	protein_coding	chr11	78188812	78215232	+	Human GRCh	OTTHUMG000001	USP35	**ubiquitin specific peptidase 35**	Extracellular	peptidase		-1.105913231	0	0.68938932	0	1	0	8.82034235	9.17864358	8.92181621	8.87846869
4756	**ENSG0000011840**	ELOVL4	protein_coding	chr6	79914814	79947553	-	Human GRCh	OTTHUMG000000	ELOVL4	**ELOVL fatty acid elongase 4**	Cytoplasm	enzyme		1.48694656	0	0.21048859	0	1	0	6.30324236	6.6605895	6.99668484	6.76275588
4757	**ENSG0000011840**	FILIP1	protein_coding	chr6	75291859	75493800	-	Human GRCh	OTTHUMG000000	FILIP1	**filamin A interacting protein 1**	Cytoplasm	other		-1.757453318	-1	0.36093644	0	1	0	4.29489294	3.96721293	3.17750685	3.9048944
4758	**ENSG0000011841**	CASP8AP2	protein_coding	chr6	89829894	89874436	+	Human GRCh	OTTHUMG000000	CASP8AP2	**caspase 8 associated protein 2**	Nucleus	transcription		-1.124320048	0	0.54119097	0	1	0	11.0547566	11.2659124	11.1822312	10.8746399
4759	**ENSG0000011841**	HMGN3	protein_coding	chr6	79201245	79234689	-	Human GRCh	OTTHUMG000000	HMGN3	**high mobility group nucleosomal binding domain 3**	Nucleus	other		1.121501633	0	0.5949358	0	1	0	9.34002877	9.71494471	9.81845572	9.48408269
4760	**ENSG0000011842**	UBE3D	protein_coding	chr6	82892390	83065841	-	Human GRCh	OTTHUMG000000	UBE3D	**ubiquitin protein ligase E3D**	Other	enzyme		1.042176644	0	0.86330538	0	1	0	8.77863112	8.94263802	9.10541436	8.67214415
4761	**ENSG0000011843**	CNR1	protein_coding	chr6	88139864	88166347	-	Human GRCh	OTTHUMG000000	CNR1	**cannabinoid receptor 1**	Plasma Mem	G-protein cou	INV-202, t	-5.896294495	-1	0.34080591	0	1	0	1.67189875	2.47921024	1.75935365	1.6782488
4763	**ENSG0000011845**	ANKRD13C	protein_coding	chr1	70258999	70354734	-	Human GRCh	OTTHUMG000000	ANKRD13C	**ankyrin repeat domain 13C**	Cytoplasm	other		1.146594233	0	0.53276629	0	1	0	10.0134286	10.1842715	10.4269983	10.0472575
4764	**ENSG0000011847**	SGIP1	protein_coding	chr1	66533267	66751139	+	Human GRCh	OTTHUMG000000	SGIP1	**SH3GL interacting endocytic adaptor 1**	Cytoplasm	other		1.936608744	1	0.3288768	0	1	0	3.91711867	3.44966204	4.28011096	4.22847488
4765	**ENSG0000011848**	PHF3	protein_coding	chr6	63635802	63779336	+	Human GRCh	OTTHUMG000000	PHF3	**PHD finger protein 3**	Nucleus	other		-1.144836241	0	0.48993558	0	1	0	12.6071564	12.8862591	12.710375	12.4867689
4768	**ENSG0000011849**	PLAGL1	protein_coding	chr6	143940300	144064599	-	Human GRCh	OTTHUMG000000	PLAGL1	**PLAG1 like zinc finger 1**	Nucleus	transcription		-1.124238413	0	0.77485404	0	1	0	7.60734761	7.55576481	7.66936	7.19152883
4769	**ENSG0000011849**	FBXO30	protein_coding	chr6	145793502	145814795	-	Human GRCh	OTTHUMG000000	FBXO30	**F-box protein 30**	Extracellular	enzyme		1.212807558	0	0.4113458	0	1	0	9.70628044	9.67841636	10.0524984	9.7287162
4770	**ENSG0000011850**	TNFAIP3	protein_coding	chr6	137867214	137883314	+	Human GRCh	OTTHUMG000000	TNFAIP3	**TNF alpha induced protein 3**	Nucleus	enzyme		-1.229993932	0	0.48685836	0	1	0	10.2726704	10.5006324	10.362819	9.89621189
4771	**ENSG0000011850**	AKAP7	protein_coding	chr6	131135467	131283535	+	Human GRCh	OTTHUMG000000	AKAP7	**A-kinase anchoring protein 7**	Plasma Mem	other		1.085727156	0	0.86081237	0	1	0	5.31758485	5.63845277	5.85854645	5.2126068
4772	**ENSG0000011850**	RAB32	protein_coding	chr6	146543833	146554953	+	Human GRCh	OTTHUMG000000	RAB32	**RAB32, member RAS oncogene family**	Cytoplasm	enzyme		-1.218819745	0	0.55772719	0	1	0	6.50398974	6.87189569	6.60176631	6.36593055
4773	**ENSG0000011851**	MYB	protein_coding	chr6	135181308	135219173	+	Human GRCh	OTTHUMG000000	MYB	**MYB proto-oncogene, transcription factor**	Nucleus	transcription		-1.261916611	0	0.42929902	0	1	0	10.7026634	11.0378349	10.803776	10.3951063
4774	**ENSG0000011851**	ALDH8A1	protein_coding	chr6	134917393	134950115	-	Human GRCh	OTTHUMG000000	ALDH8A1	**aldehyde dehydrogenase 8 family member A1**	Cytoplasm	enzyme		-1.704984058	-1	0.21401392	0	1	0	6.35551384	6.73577917	6.03458769	5.95658295
4775	**ENSG0000011851**	SGK1	protein_coding	chr6	134169248	134318112	-	Human GRCh	OTTHUMG000000	SGK1	**serum/glucocorticoid regulated kinase 1**	Cytoplasm	kinase		-1.384160441	0	0.19221041	0	1	0	10.4133912	10.3281077	10.2332474	9.73053532
4776	**ENSG0000011851**	RNF146	protein_coding	chr6	127266726	127288567	+	Human GRCh	OTTHUMG000000	RNF146	**ring finger protein 146**	Cytoplasm	enzyme		-1.10810327	0	0.62958572	0	1	0	8.90627111	9.05960022	9.01254197	8.72220673
4778	**ENSG0000011852**	CCN2	protein_coding	chr6	131948176	131951372	-	Human GRCh	OTTHUMG000000	CCN2	**cellular communication network factor 2**	Extracellular	growth facto	pamrevlun	2.371316916	1	0.08638976	0	1	0	5.30128494	5.03000125	6.0801052	5.96305241
4780	**ENSG0000011855**	PMFBP1	protein_coding	chr16	72112157	72176878	-	Human GRCh	OTTHUMG000001	PMFBP1	**polyamine modulated factor 1 binding protein 1**	Cytoplasm	other		-1.421071241	0	0.15258005	0	1	0	9.47073151	9.84261629	9.32765548	9.22164765
4781	**ENSG0000011856**	FBXL5	protein_coding	chr4	15604381	15681679	-	Human GRCh	OTTHUMG000000	FBXL5	**F-box and leucine rich repeat protein 5**	Cytoplasm	enzyme		-1.011055701	0	0.96361947	0	1	0	10.5983093	10.7861366	10.8466757	10.49487
4782	**ENSG0000011857**	MED28	protein_coding	chr4	17614641	17634105	+	Human GRCh	OTTHUMG000001	MED28	**mediator complex subunit 28**	Nucleus	other		-1.088344352	0	0.70792988	0	1	0	11.5131275	11.8009703	11.7146685	11.4113661
4783	**ENSG0000011859**	SLC16A7	protein_coding	chr12	59596029	59789855	+	Human GRCh	OTTHUMG000001	SLC16A7	**solute carrier family 16 member 7**	Plasma Mem	transporter		1.088274982	0	0.78539433	0	1	0	8.34331326	8.71797511	8.51702877	8.74022409
4784	**ENSG0000011860**	RXYLT1	protein_coding	chr12	63779833	63809792	+	Human GRCh	OTTHUMG000001	RXYLT1	**ribitol xylosyltransferase 1**	Plasma Mem	enzyme		1.067921745	0	0.78870455	0	1	0	8.68021966	8.83211511	9.05411427	8.55199854
4785	**ENSG0000011862**	ZNF430	protein_coding	chr19	21020634	21060050	+	Human GRCh	OTTHUMG000001	ZNF430	**zinc finger protein 430**	Nucleus	transcription		-1.051281033	0	0.81315802	0	1	0	9.12226672	9.39086193	9.30929084	9.10082057
4786	**ENSG0000011864**	VAMP8	protein_coding	chr2	85561562	85582031	+	Human GRCh	OTTHUMG000001	VAMP8	**vesicle associated membrane protein 8**	Plasma Mem	transporter		-1.131237943	0	0.60298379	0	1	0	9.59046996	9.94989016	9.69338108	9.59673043
4787	**ENSG0000011865**	DCLRE1B	protein_coding	chr1	113904619	113914086	+	Human GRCh	OTTHUMG000000	DCLRE1B	**DNA cross-link repair 1B**	Nucleus	enzyme		-1.060485719	0	0.77658574	0	1	0	9.34418562	9.61469408	9.51383235	9.32255321
4788	**ENSG0000011868**	MYL12B	protein_coding	chr18	3261479	3278461	+	Human GRCh	OTTHUMG000001	MYL12B	**myosin light chain 12B**	Cytoplasm	other		-1.112871356	0	0.60553154	0	1	0	11.0870978	11.5096613	11.2652827	11.1324584
4789	**ENSG0000011868**	FOXO3	protein_coding	chr6	108559835	108684774	+	Human GRCh	OTTHUMG000000	FOXO3	**forkhead box O3**	Nucleus	transcription		-1.175606093	0	0.46836382	0	1	0	12.0021382	12.340343	12.1404009	11.84472
4790	**ENSG0000011869**	ARMC2	protein_coding	chr6	108848416	108974476	+	Human GRCh	OTTHUMG000000	ARMC2	**armadillo repeat containing 2**	Extracellular	other		-1.217139789	0	0.52317331	0	1	0	6.69023057	6.82125753	6.77628318	6.30689841
4792	**ENSG0000011870**	RPN2	protein_coding	chr20	37178410	37241619	+	Human GRCh	OTTHUMG000000	RPN2	**ribophorin II**	Cytoplasm	enzyme		-1.24240518	0	0.27124841	0	1	0	12.5676097	12.8531747	12.5415128	12.4058434
4793	**ENSG0000011870**	TGIF2	protein_coding	chr20	36573488	36593950	+	Human GRCh	OTTHUMG000001	TGIF2	**TGFB induced factor homeobox 2**	Nucleus	transcription		-1.025571315	0	0.92523171	0	1	0	10.0024358	10.3279854	10.4260102	9.78322538
4796	**ENSG0000011876**	PKD2	protein_coding	chr4	88007635	88077777	+	Human GRCh	OTTHUMG000001	PKD2	**polycystin 2, transient receptor potential cation channel**	Plasma Mem	ion channel		-1.03600634	0	0.87944179	0	1	0	9.08140218	9.23778023	9.18803259	9.05624771
4797	**ENSG0000011877**	ABCG2	protein_coding	chr4	88090150	88231628	-	Human GRCh	OTTHUMG000001	ABCG2	**ATP binding cassette subfamily G member 2 (Junior blood grou**	Plasma Mem	transporter		-1.055627043	0	0.94118145	0	1	0	3.88198241	3.28022598	3.66985257	3.48984115

4799	**ENSG0000011880**	STBD1	protein_coding	chr4	76306733	76311130	+	Human GRCh	OTTHUMG000001	STBD1	**starch binding domain 1**	Cytoplasm	other		-1.024550704	0	0.93486403	0	1	0	7.92283631	8.13754045	8.12317818	7.88152477
4800	**ENSG0000011881**	CCNI	protein_coding	chr4	77047155	77076309	-	Human GRCh	OTTHUMG000001	CCNI	**cyclin I**	Other	other		-1.120417293	0	0.59339144	0	1	0	12.6388922	12.9120858	12.7755353	12.5284056
4802	**ENSG0000011885**	MFSD1	protein_coding	chr3	158732198	158829719	+	Human GRCh	OTTHUMG000001	MFSD1	**major facilitator superfamily domain containing 1**	Cytoplasm	transporter		-1.132043907	0	0.58705529	0	1	0	9.72562346	9.89230115	9.86100743	9.45222426
4803	**ENSG0000011887**	RAB3GAP2	protein_coding	chr1	220148923	220272529	-	Human GRCh	OTTHUMG000000	RAB3GAP2	**RAB3 GTPase activating non-catalytic protein subunit 2**	Cytoplasm	other		-1.027465623	0	0.89864003	0	1	0	11.4377747	11.5870703	11.624023	11.3272076
4804	**ENSG0000011889**	EEF2KMT	protein_coding	chr16	5084284	5097795	-	Human GRCh	OTTHUMG000001	EEF2KMT	**eukaryotic elongation factor 2 lysine methyltransferase**	Cytoplasm	enzyme		-1.078356712	0	0.76631421	0	1	0	8.73082447	9.25423646	9.07065081	8.78603859
4805	**ENSG0000011889**	PPL	protein_coding	chr16	4882507	4960741	-	Human GRCh	OTTHUMG000001	PPL	**periplakin**	Cytoplasm	other		-1.281973923	0	0.42571826	0	1	0	7.35722774	7.24534117	6.73943496	7.30456135
4806	**ENSG0000011890**	UBN1	protein_coding	chr16	4846665	4882401	+	Human GRCh	OTTHUMG000001	UBN1	**ubinuclein 1**	Nucleus	transcription		-1.076753336	0	0.70837727	0	1	0	11.6082149	11.8931924	11.8043653	11.5332539
4807	**ENSG0000011892**	KLF12	protein_coding	chr13	73686089	74134404	-	Human GRCh	OTTHUMG000000	KLF12	**KLF transcription factor 12**	Nucleus	transcription		-1.013941538	0	0.97576647	0	1	0	10.797924	11.2791469	11.2177036	10.8483224
4808	**ENSG0000011893**	UCHL3	protein_coding	chr13	75549483	75606020	+	Human GRCh	OTTHUMG000001	UCHL3	**ubiquitin C-terminal hydrolase L3**	Cytoplasm	peptidase		-1.036010695	0	0.86950883	0	1	0	9.51357571	9.92836299	9.74893956	9.64684368
4809	**ENSG0000011894**	PCDH17	protein_coding	chr13	57631744	57729311	+	Human GRCh	OTTHUMG000000	PCDH17	**protocadherin 17**	Plasma Mem	other		1.028540907	0	0.95365516	0	1	0	5.8160158	5.84924343	5.51377168	6.12657053
4810	**ENSG0000011896**	HS1BP3	protein_coding	chr2	20560448	20651130	-	Human GRCh	OTTHUMG000001	HS1BP3	**HCLS1 binding protein 3**	Other	other		1.120879598	0	0.57923511	0	1	0	10.0905359	10.1802862	10.4076327	10.101864
4811	**ENSG0000011896**	LDAH	protein_coding	chr2	20684014	20823130	-	Human GRCh	OTTHUMG000001	LDAH	**lipid droplet associated hydrolase**	Cytoplasm	enzyme		-11.34969589	-1	0.046294	1	1	0	0.92196424	2.34232244	0.99813813	0.92749073
4812	**ENSG0000011896**	WDR35	protein_coding	chr2	19910263	19990105	-	Human GRCh	OTTHUMG000000	WDR35	**WD repeat domain 35**	Cytoplasm	other		-1.004796133	0	0.9858668	0	1	0	9.16858143	9.19305553	9.42430922	8.86286433
4813	**ENSG0000011897**	CCND2	protein_coding	chr12	4269771	4305353	+	Human GRCh	OTTHUMG000001	CCND2	**cyclin D2**	Nucleus	other		5.115352464	1	0.3616505	0	1	0	0.3885929	0.47421247	0.46060738	1.30617468
4815	**ENSG0000011898**	ELL2	protein_coding	chr5	95885098	95961851	-	Human GRCh	OTTHUMG000001	ELL2	**elongation factor for RNA polymerase II 2**	Nucleus	transcription		1.171101167	0	0.48993439	0	1	0	10.7752134	11.2985816	11.2424984	11.2585926
4816	**ENSG0000011899**	DNAH7	protein_coding	chr2	195737703	196068837	-	Human GRCh	OTTHUMG000001	DNAH7	**dynein axonemal heavy chain 7**	Cytoplasm	other		-1.108282492	0	0.71731681	0	1	0	8.97361186	8.90977179	8.93299168	8.72123676
4817	**ENSG0000011900**	CYP20A1	protein_coding	chr2	203238977	203306026	+	Human GRCh	OTTHUMG000001	CYP20A1	**cytochrome P450 family 20 subfamily A member 1**	Other	enzyme		-1.088058737	0	0.67812674	0	1	0	10.0600807	10.2134266	10.2405764	9.82718568
4818	**ENSG0000011901**	NDUFB3	protein_coding	chr2	201071433	201085750	+	Human GRCh	OTTHUMG000001	NDUFB3	**NADH:ubiquinone oxidoreductase subunit B3**	Cytoplasm	enzyme		1.110823556	0	0.69843356	0	1	0	9.3805538	9.66234631	9.58193795	9.70770707
4819	**ENSG0000011904**	GTF3C3	protein_coding	chr2	196763035	196799725	-	Human GRCh	OTTHUMG000001	GTF3C3	**general transcription factor IIIC subunit 3**	Nucleus	transcription		-1.067323188	0	0.75356265	0	1	0	10.4353239	10.7147787	10.6537194	10.3489001
4820	**ENSG0000011904**	SATB2	protein_coding	chr2	199269505	199471266	-	Human GRCh	OTTHUMG000001	SATB2	**SATB homeobox 2**	Nucleus	transcription		-1.056802158	0	0.83715566	0	1	0	9.98098339	10.1192184	10.0946112	9.86926746
4821	**ENSG0000011904**	UBE2B	protein_coding	chr5	134371184	134392108	+	Human GRCh	OTTHUMG000001	UBE2B	**ubiquitin conjugating enzyme E2 B**	Cytoplasm	enzyme		1.022841524	0	0.91920682	0	1	0	9.74421817	9.89529379	9.95649203	9.72667691
4822	**ENSG0000011912**	TRPM6	protein_coding	chr9	74722495	74888094	-	Human GRCh	OTTHUMG000000	TRPM6	**transient receptor potential cation channel subfamily M meml**	Plasma Mem	kinase		-1.261563619	0	0.84584178	0	NA	0	2.49669306	1.80755645	2.0546387	2.20359583
4823	**ENSG0000011912**	GDA	protein_coding	chr9	72114595	72257193	+	Human GRCh	OTTHUMG000000	GDA	**guanine deaminase**	Cytoplasm	enzyme		3.81116915	1	0.48381301	0	NA	0	0.05305674	0.36443735	0.56662129	0.51051828
4824	**ENSG0000011913**	KLF9	protein_coding	chr9	70384604	70414657	-	Human GRCh	OTTHUMG000000	KLF9	**KLF transcription factor 9**	Nucleus	transcription		-1.089371259	0	0.7621763	0	1	0	8.60031996	9.47125273	8.58538302	9.33195219
4825	**ENSG0000011913**	TJP2	protein_coding	chr9	69121264	69274615	+	Human GRCh	OTTHUMG000000	TJP2	**tight junction protein 2**	Plasma Mem	kinase		1.048555928	0	0.83460239	0	1	0	10.0736036	10.5241314	10.3031816	10.4311838
4827	**ENSG0000011918**	ITGB1BP1	protein_coding	chr2	9403475	9423528	-	Human GRCh	OTTHUMG000000	ITGB1BP1	**integrin subunit beta 1 binding protein 1**	Plasma Mem	other		1.046773618	0	0.83696482	0	1	0	9.58745446	9.96435244	9.96596971	9.70234092
4828	**ENSG0000011920**	CPSF3	protein_coding	chr2	9423651	9473101	+	Human GRCh	OTTHUMG000000	CPSF3	**cleavage and polyadenylation specific factor 3**	Nucleus	enzyme		-1.229802991	0	0.3244952	0	1	0	10.6744698	11.0954671	10.7814302	10.5500023
4829	**ENSG0000011922**	PIGZ	protein_coding	chr3	196946356	196969060	-	Human GRCh	OTTHUMG000001	PIGZ	**phosphatidylinositol glycan anchor biosynthesis class Z**	Cytoplasm	enzyme		-1.041891817	0	0.92472893	0	1	0	5.88315168	5.74821598	6.14651103	5.23852763
4830	**ENSG0000011923**	SENP5	protein_coding	chr3	196867856	196934714	+	Human GRCh	OTTHUMG000001	SENP5	**SUMO specific peptidase 5**	Nucleus	peptidase		-1.008541418	0	0.96945268	0	1	0	10.9762837	11.2792162	11.3623242	10.8396477
4831	**ENSG0000011924**	CCDC92	protein_coding	chr12	123918660	123972831	-	Human GRCh	OTTHUMG000001	CCDC92	**coiled-coil domain containing 92**	Cytoplasm	other		-1.386202415	0	0.15630101	0	1	0	9.42615951	9.68493264	9.33861047	9.05706765
4832	**ENSG0000011928**	C1orf198	protein_coding	chr1	230837119	230869589	-	Human GRCh	OTTHUMG000000	C1orf198	**chromosome 1 open reading frame 198**	Cytoplasm	other		1.115292707	0	0.63569402	0	1	0	10.9805046	11.5068298	11.5666696	11.1947904
4833	**ENSG0000011928**	TRIM67	protein_coding	chr1	231162058	231221565	+	Human GRCh	OTTHUMG000000	TRIM67	**tripartite motif containing 67**	Cytoplasm	other		-5.34097865	-1	0.21496908	0	1	0	2.61808845	2.1190158	2.10070305	2.0113386
4834	**ENSG0000011928**	HEATR1	protein_coding	chr1	236549005	236604516	-	Human GRCh	OTTHUMG000000	HEATR1	**HEAT repeat containing 1**	Nucleus	other		-1.102620553	0	0.62599236	0	1	0	12.1080769	12.4543202	12.3609976	11.9755536
4835	**ENSG0000011931**	PTBP3	protein_coding	chr9	112217716	112333664	-	Human GRCh	OTTHUMG000000	PTBP3	**polypyrimidine tract binding protein 3**	Nucleus	other		1.05228268	0	0.81761099	0	1	0	11.9527291	12.2643008	12.3467315	11.970165
4836	**ENSG0000011931**	RAD23B	protein_coding	chr9	107283137	107332192	+	Human GRCh	OTTHUMG000000	RAD23B	**RAD23 homolog B, nucleotide excision repair protein**	Nucleus	other		-1.038828644	0	0.84661027	0	1	0	12.547427	12.7855161	12.7250538	12.5268076
4837	**ENSG0000011932**	FKBP15	protein_coding	chr9	113161006	113221458	-	Human GRCh	OTTHUMG000000	FKBP15	**FKBP prolyl isomerase family member 15**	Plasma Mem	enzyme		1.012511872	0	0.95077566	0	1	0	10.4178646	10.7111012	10.694319	10.4693229
4838	**ENSG0000011932**	CTNNAL1	protein_coding	chr9	108942569	109013522	-	Human GRCh	OTTHUMG000000	CTNNAL1	**catenin alpha like 1**	Plasma Mem	other		-1.073722204	0	0.73763989	0	1	0	10.3108471	10.6151374	10.5310865	10.2389313
4839	**ENSG0000011932**	ABITRAM	protein_coding	chr9	108934400	108950744	+	Human GRCh	OTTHUMG000000	ABITRAM	**actin binding transcription modulator**	Extracellular	other		-1.141068403	0	0.55100251	0	1	0	9.33514238	9.65314946	9.3606347	9.35513477
4840	**ENSG0000011933**	DYNC2I2	protein_coding	chr9	128633653	128656847	-	Human GRCh	OTTHUMG000000	DYNC2I2	**dynein 2 intermediate chain 2**	Cytoplasm	other		1.006079117	0	0.9754852	0	1	0	11.5178512	11.7382689	11.70867	11.5667391
4841	**ENSG0000011933**	SET	protein_coding	chr9	128683424	128696400	+	Human GRCh	OTTHUMG000000	SET	**SET nuclear proto-oncogene**	Nucleus	phosphatase		-1.037441081	0	0.84821517	0	1	0	13.604608	13.8665394	13.8377395	13.5494608
4842	**ENSG0000011938**	PTPA	protein_coding	chr9	129110950	129148946	+	Human GRCh	OTTHUMG000000	PTPA	**protein phosphatase 2 phosphatase activator**	Cytoplasm	phosphatase		1.029592245	0	0.88671848	0	1	0	11.7781563	12.0169025	12.0433734	11.8188837
4843	**ENSG0000011939**	GLE1	protein_coding	chr9	128504655	128543874	+	Human GRCh	OTTHUMG000000	GLE1	**GLE1 RNA export mediator**	Nucleus	other		-1.16588028	0	0.43502484	0	1	0	10.8996391	11.2849492	11.0191383	10.8533482
4844	**ENSG0000011939**	RAB14	protein_coding	chr9	121178133	121223014	-	Human GRCh	OTTHUMG000000	RAB14	**RAB14, member RAS oncogene family**	Cytoplasm	enzyme		-1.040614063	0	0.84512773	0	1	0	11.5429269	11.8184268	11.7749538	11.498333
4845	**ENSG0000011939**	CNTRL	protein_coding	chr9	121074660	121177729	+	Human GRCh	OTTHUMG000000	CNTRL	**centriolin**	Nucleus	transcription		-1.19064057	0	0.38126135	0	1	0	9.98210924	10.136823	10.0214551	9.72502355
4846	**ENSG0000011940**	TRIM32	protein_coding	chr9	116687305	116701300	+	Human GRCh	OTTHUMG000000	TRIM32	**tripartite motif containing 32**	Nucleus	transcription		-1.066150506	0	0.77944199	0	1	0	9.15672046	9.39147799	9.32897104	9.07890393
4847	**ENSG0000011940**	FBXW2	protein_coding	chr9	120751978	120793416	-	Human GRCh	OTTHUMG000000	FBXW2	**F-box and WD repeat domain containing 2**	Cytoplasm	enzyme		-1.075714482	0	0.73796916	0	1	0	11.5444294	11.7379067	11.794448	11.2791157
4848	**ENSG0000011940**	PHF19	protein_coding	chr9	120855651	120894896	-	Human GRCh	OTTHUMG000000	PHF19	**PHD finger protein 19**	Nucleus	other		-1.228277145	0	0.39456754	0	1	0	10.6862847	10.8664538	10.6626662	10.4388358
4849	**ENSG0000011940**	NEK6	protein_coding	chr9	124257606	124353307	+	Human GRCh	OTTHUMG000000	NEK6	**NIMA related kinase 6**	Nucleus	kinase		1.020806529	0	0.91552056	0	1	0	10.4673418	10.7257346	10.6203715	10.6312067
4850	**ENSG0000011941**	BSPRY	protein_coding	chr9	113349541	113371233	+	Human GRCh	OTTHUMG000000	BSPRY	**B-box and SPRY domain containing**	Cytoplasm	other		-1.072309081	0	0.82845357	0	1	0	9.34665495	9.58039238	9.53942755	9.2250369
4851	**ENSG0000011941**	PPP6C	protein_coding	chr9	125146573	125189939	-	Human GRCh	OTTHUMG000000	PPP6C	**protein phosphatase 6 catalytic subunit**	Nucleus	phosphatase		-1.079225472	0	0.72097921	0	1	0	10.7395297	11.0406402	10.9294372	10.6893602
4852	**ENSG0000011942**	NDUFA8	protein_coding	chr9	122144058	122159779	-	Human GRCh	OTTHUMG000000	NDUFA8	**NADH:ubiquinone oxidoreductase subunit A8**	Cytoplasm	enzyme		-1.010107206	0	0.97135205	0	1	0	8.69091299	9.31922847	9.11049892	8.95451345
4853	**ENSG0000011943**	HDHD3	protein_coding	chr9	113373419	113376986	-	Human GRCh	OTTHUMG000000	HDHD3	**haloacid dehalogenase like hydrolase domain containing 3**	Cytoplasm	enzyme		1.047261122	0	0.8382802	0	1	0	8.13378523	8.08413263	8.25524986	8.05424863
4854	**ENSG0000011944**	RBM18	protein_coding	chr9	122237622	122264840	-	Human GRCh	OTTHUMG000000	RBM18	**RNA binding motif protein 18**	Other	other		1.3378047	0	0.18168471	0	1	0	8.96537503	9.04494158	9.51472193	9.08719828
4856	**ENSG0000011947**	HSDL2	protein_coding	chr9	112380080	112472405	+	Human GRCh	OTTHUMG000000	HSDL2	**hydroxysteroid dehydrogenase like 2**	Cytoplasm	transporter		1.002702378	0	0.99110753	0	1	0	9.92597962	10.3595561	10.2889371	10.0275912
4857	**ENSG0000011948**	MAPKAP1	protein_coding	chr9	125437393	125707234	-	Human GRCh	OTTHUMG000000	MAPKAP1	**MAPK associated protein 1**	Cytoplasm	other		-1.078461627	0	0.68131075	0	1	0	10.9057131	11.1010488	11.0282741	10.8110169
4858	**ENSG0000011950**	NR4A3	protein_coding	chr9	99821855	99866891	+	Human GRCh	OTTHUMG000000	NR4A3	**nuclear receptor subfamily 4 group A member 3**	Nucleus	ligand-depen		2.160348211	1	0.11103224	0	1	0	4.33814293	4.35592899	4.95675521	5.14380234
4859	**ENSG0000011950**	INVS	protein_coding	chr9	100099243	100302175	+	Human GRCh	OTTHUMG000000	INVS	**inversin**	Nucleus	transcription		-1.190538508	0	0.39403733	0	1	0	10.0784521	10.2701765	10.1420988	9.80725304
4860	**ENSG0000011951**	GALNT12	protein_coding	chr9	98807670	98850081	+	Human GRCh	OTTHUMG000000	GALNT12	**polypeptide N-acetylgalactosaminyltransferase 12**	Cytoplasm	enzyme		-1.793564621	-1	0.42138902	0	1	0	3.77873389	3.73941897	3.17179588	3.60472096
4861	**ENSG0000011952**	DENND1A	protein_coding	chr9	123379654	123930152	-	Human GRCh	OTTHUMG000000	DENND1A	**DENN domain containing 1A**	Plasma Mem	other		-1.050518101	0	0.81857791	0	1	0	11.0701511	11.3302379	11.2111979	11.0902422
4862	**ENSG0000011952**	ALG2	protein_coding	chr9	99216425	99221942	-	Human GRCh	OTTHUMG000000	ALG2	**ALG2 alpha-1,3/1,6-mannosyltransferase**	Cytoplasm	enzyme		-1.009300467	0	0.97020275	0	1	0	9.77762096	9.89788348	10.0248013	9.59422234
4863	**ENSG0000011953**	CSF3R	protein_coding	chr1	36466043	36483278	-	Human GRCh	OTTHUMG000000	CSF3R	**colony stimulating factor 3 receptor**	Plasma Mem	transmembra	cytarabine	1	0	1	0	NA	0	0.22110799	0.25123519	0.2463432	0.22291123
4864	**ENSG0000011953**	KDSR	protein_coding	chr18	63327726	63367228	-	Human GRCh	OTTHUMG000001	KDSR	**3-ketodihydrosphingosine reductase**	Plasma Mem	enzyme		-1.113585616	0	0.58116765	0	1	0	10.3880422	10.6355356	10.4615154	10.3327353
4865	**ENSG0000011954**	VPS4B	protein_coding	chr18	63389190	63422483	-	Human GRCh	OTTHUMG000001	VPS4B	**vacuolar protein sorting 4 homolog B**	Cytoplasm	transporter		-1.020960405	0	0.9144641	0	1	0	10.00806	10.3352082	10.3155034	9.98118043
4866	**ENSG0000011954**	ONECUT2	protein_coding	chr18	57435374	57491298	+	Human GRCh	OTTHUMG000001	ONECUT2	**one cut homeobox 2**	Nucleus	transcription		-1.058387842	0	0.80707844	0	1	0	11.71111	11.8352466	11.8303939	11.5804088
4867	**ENSG0000011955**	C19orf25	protein_coding	chr19	1461143	1479219	-	Human GRCh	OTTHUMG000001	C19orf25	**chromosome 19 open reading frame 25**	Other	other		1.169906621	0	0.45335464	0	1	0	9.57837301	9.71931803	9.97136303	9.65488107
4868	**ENSG0000011957**	ZBTB45	protein_coding	chr19	58513530	58538911	-	Human GRCh	OTTHUMG000001	ZBTB45	**zinc finger and BTB domain containing 45**	Nucleus	transcription		1.064991114	0	0.77609097	0	1	0	9.38868417	9.7066872	9.59834506	9.65570358
4869	**ENSG0000011959**	YLPM1	protein_coding	chr14	74763316	74859435	+	Human GRCh	OTTHUMG000001	YLPM1	**YLP motif containing 1**	Nucleus	transcription		-1.070774968	0	0.73613706	0	1	0	12.4084593	12.7378431	12.5857204	12.4283548
4870	**ENSG0000011959**	DCAF4	protein_coding	chr14	72926377	72959703	+	Human GRCh	OTTHUMG000001	DCAF4	**DDB1 and CUL4 associated factor 4**	Nucleus	other		-1.280277308	0	0.35989659	0	1	0	9.10062252	9.5635712	9.16725799	8.98661894
4873	**ENSG0000011961**	FCF1	protein_coding	chr14	74713144	74738620	+	Human GRCh	OTTHUMG000001	FCF1	**FCF1 rRNA-processing protein**	Nucleus	other		-1.024734496	0	0.89211419	0	1	0	9.74962855	9.8347756	9.80905119	9.72208771
4874	**ENSG0000011963**	PGF	protein_coding	chr14	74941834	74955626	-	Human GRCh	OTTHUMG000001	PGF	**placental growth factor**	Extracellular	growth facto	aflibercept	1.268369718	0	0.60600618	0	1	0	4.99073236	4.94367583	5.03382703	5.25941463
4875	**ENSG0000011963**	IFI27L2	protein_coding	chr14	94127779	94130253	-	Human GRCh	OTTHUMG000001	IFI27L2	**interferon alpha inducible protein 27 like 2**	Cytoplasm	other		1	0	1	0	NA	0	0.99143932	1.00931003	1.00633414	0.99247862
4876	**ENSG0000011963**	BBOF1	protein_coding	chr14	74019349	74082863	+	Human GRCh	OTTHUMG000001	BBOF1	**basal body orientation factor 1**	Cytoplasm	other		-1.343830128	0	0.33284725	0	1	0	7.66537936	7.983688	7.71704051	7.27376356
4877	**ENSG0000011963**	NEK9	protein_coding	chr14	75079353	75127344	-	Human GRCh	OTTHUMG000001	NEK9	**NIMA related kinase 9**	Nucleus	kinase		-1.109451818	0	0.6015821	0	1	0	11.4842531	11.7447707	11.6786275	11.3062102
4878	**ENSG0000011964**	ACYP1	protein_coding	chr14	75053237	75069483	-	Human GRCh	OTTHUMG000001	ACYP1	**acylphosphatase 1**	Cytoplasm	enzyme		-1.107982673	0	0.68583461	0	1	0	8.35060688	8.45754173	8.5690107	7.94744197
4879	**ENSG0000011965**	IFT43	protein_coding	chr14	75902136	76084585	+	Human GRCh	OTTHUMG000001	IFT43	**intraflagellar transport 43**	Cytoplasm	other		1.040632226	0	0.8595505	0	1	0	8.37026541	8.70920104	8.62680155	8.5614301
4880	**ENSG0000011965**	NPC2	protein_coding	chr14	74476192	74494177	-	Human GRCh	OTTHUMG000001	NPC2	**NPC intracellular cholesterol transporter 2**	Extracellular	transporter		1.00645585	0	0.97823856	0	1	0	10.0946595	10.4230369	10.3656913	10.1817353
4881	**ENSG0000011966**	DNAL1	protein_coding	chr14	73644875	73703732	+	Human GRCh	OTTHUMG000001	DNAL1	**dynein axonemal light chain 1**	Cytoplasm	other		-1.075124956	0	0.73200798	0	1	0	9.97486312	10.3119012	10.3097995	9.78527792
4882	**ENSG0000011966**	IRF2BPL	protein_coding	chr14	77024543	77028708	-	Human GRCh	OTTHUMG000001	IRF2BPL	**interferon regulatory factor 2 binding protein like**	Nucleus	enzyme		1.610950858	1	0.0873309	0	1	0	8.71422313	8.70262275	9.09371049	9.24115631
4883	**ENSG0000011967**	ACOT2	protein_coding	chr14	73567620	73575658	+	Human GRCh	OTTHUMG000001	ACOT2	**acyl-CoA thioesterase 2**	Cytoplasm	enzyme		1.057158463	0	0.85041824	0	1	0	8.17152396	8.27302433	8.57654063	7.89114669
4884	**ENSG0000011968**	LTBP2	protein_coding	chr14	74498183	74612378	-	Human GRCh	OTTHUMG000001	LTBP2	**latent transforming growth factor beta binding protein 2**	Extracellular	other		1.290090326	0	0.33910899	0	1	0	7.77757413	7.76150803	8.17141625	7.84701671
4885	**ENSG0000011968**	AREL1	protein_coding	chr14	74653437	74713117	-	Human GRCh	OTTHUMG000001	AREL1	**apoptosis resistant E3 ubiquitin protein ligase 1**	Cytoplasm	enzyme		-1.151919067	0	0.49735451	0	1	0	10.7962963	11.1043756	10.8980734	10.7068229
4886	**ENSG0000011968**	MLH3	protein_coding	chr14	75013769	75051532	-	Human GRCh	OTTHUMG000001	MLH3	**mutL homolog 3**	Nucleus	other		1.021154731	0	0.92004785	0	1	0	10.1152186	10.2335583	10.4019431	9.95273683
4887	**ENSG0000011968**	TTLL5	protein_coding	chr14	75633625	75955079	+	Human GRCh	OTTHUMG000001	TTLL5	**tubulin tyrosine ligase like 5**	Cytoplasm	enzyme		-1.104289329	0	0.62824111	0	1	0	10.7329249	11.1092906	10.9491334	10.6936555

4888	ENSG0000011968	FLVCR2	protein_coding	chr14	75578620	75663214	+	Human GRCh	OTTHUMG000001	FLVCR2	FLVCR choline and putative heme transporter 2	Plasma Mem	transporter		1.123305862	0	0.83913595	0	1	0	4.63872681	4.8379622	4.39896705	5.17180805
4889	ENSG0000011968	ABCD4	protein_coding	chr14	74285269	74303055	-	Human GRCh	OTTHUMG000001	ABCD4	ATP binding cassette subfamily D member 4	Cytoplasm	transporter		-1.101638273	0	0.68656422	0	1	0	9.96023975	10.0274859	10.0599065	9.69244742
4890	ENSG0000011968	DLST	protein_coding	chr14	74881891	74903743	+	Human GRCh	OTTHUMG000001	DLST	dihydrolipoamide S-succinyltransferase	Cytoplasm	enzyme		-1.052054217	0	0.8133865	0	1	0	11.4173768	11.864134	11.7755304	11.4078925
4891	ENSG0000011969	PPP4R4	protein_coding	chr14	94146128	94279734	+	Human GRCh	OTTHUMG000001	PPP4R4	protein phosphatase 4 regulatory subunit 4	Cytoplasm	other		-1.783903671	-1	0.08078688	0	1	0	7.01805258	7.43980022	6.42361263	6.8076259
4892	ENSG0000011969	TGFB3	protein_coding	chr14	75958097	75983011	-	Human GRCh	OTTHUMG000001	TGFB3	transforming growth factor beta 3	Extracellular	growth facto	fresolimun	-1.908420044	-1	0.07238872	0	1	0	6.38743684	6.76661523	5.93384185	5.91635924
4893	ENSG0000011970	ZC2HC1C	protein_coding	chr14	75064109	75119502	+	Human GRCh	OTTHUMG000001	ZC2HC1C	zinc finger C2HC-type containing 1C	Other	other		-1.148282527	0	0.67815782	0	1	0	6.19920398	6.35800765	6.32472229	5.93104261
4894	ENSG0000011970	SLIRP	protein_coding	chr14	77708071	77761104	+	Human GRCh	OTTHUMG000001	SLIRP	SRA stem-loop interacting RNA binding protein	Cytoplasm	other		1.02267714	0	0.92937789	0	1	0	9.38857527	9.91640365	9.77439974	9.63385256
4895	ENSG0000011970	RBM25	protein_coding	chr14	73058532	73123899	+	Human GRCh	OTTHUMG000001	RBM25	RNA binding motif protein 25	Nucleus	other		1.020848449	0	0.91038492	0	1	0	12.0255269	12.2866032	12.3324745	12.0183069
4896	ENSG0000011971	ALDH6A1	protein_coding	chr14	74056847	74084492	-	Human GRCh	OTTHUMG000001	ALDH6A1	aldehyde dehydrogenase 6 family member A1	Cytoplasm	enzyme		-1.158588191	0	0.52548569	0	1	0	11.4977771	11.7327423	11.6215898	11.2556449
4897	ENSG0000011971	GPR68	protein_coding	chr14	91232532	91253925	-	Human GRCh	OTTHUMG000001	GPR68	G protein-coupled receptor 68	Plasma Mem	G-protein cou		-6.074077748	-1	0.0355161	1	1	0	2.86933516	3.05138977	1.94513781	1.84343178
4898	ENSG0000011971	ESRRB	protein_coding	chr14	76310712	76501837	+	Human GRCh	OTTHUMG000001	ESRRB	estrogen related receptor beta	Nucleus	transcription		-2.468399853	-1	0.14040015	0	1	0	5.0363721	4.91115391	4.26506546	4.0774265
4899	ENSG0000011971	EIF2B2	protein_coding	chr14	75002921	75012366	+	Human GRCh	OTTHUMG000001	EIF2B2	eukaryotic translation initiation factor 2B subunit beta	Cytoplasm	translation re		-1.280180883	0	0.23513803	0	1	0	9.66750512	9.86954072	9.5775573	9.41624648
4900	ENSG0000011972	NRDE2	protein_coding	chr14	90267860	90331969	-	Human GRCh	OTTHUMG000001	NRDE2	NRDE-2, necessary for RNA interference, domain containing	Nucleus	other		-1.123929734	0	0.56691844	0	1	0	9.66672684	9.84294196	9.767417	9.48249676
4901	ENSG0000011972	COQ6	protein_coding	chr14	73949926	73963670	+	Human GRCh	OTTHUMG000001	COQ6	coenzyme Q6, monooxygenase	Cytoplasm	enzyme		-1.070971417	0	0.77149003	0	1	0	9.60022764	9.99789847	9.89689213	9.5571203
4902	ENSG0000011972	ZNF410	protein_coding	chr14	73886617	73932521	+	Human GRCh	OTTHUMG000001	ZNF410	zinc finger protein 410	Nucleus	transcription		-1.190345661	0	0.39274633	0	1	0	10.1945019	10.379208	10.2722724	9.90674198
4903	ENSG0000011972	RHOQ	protein_coding	chr2	46541806	46584688	+	Human GRCh	OTTHUMG000001	RHOQ	ras homolog family member Q	Plasma Mem	enzyme		-1.061841832	0	0.83100165	0	1	0	9.17698652	9.4190099	9.36399958	9.09915467
4904	ENSG0000011973	GPR75	protein_coding	chr2	53852912	53859967	-	Human GRCh	OTTHUMG000001	GPR75	G protein-coupled receptor 75	Plasma Mem	G-protein cou		-1.272823109	0	0.37623388	0	1	0	8.27964899	8.64447886	8.23872819	8.1851168
4905	ENSG0000011976	SUPT7L	protein_coding	chr2	27650809	27663840	-	Human GRCh	OTTHUMG000001	SUPT7L	SPT7 like, STAGA complex subunit gamma	Nucleus	transcription		-1.129846176	0	0.5332752	0	1	0	10.249328	10.373201	10.2385829	10.113135
4906	ENSG0000011977	KLHL29	protein_coding	chr2	23385179	23708611	+	Human GRCh	OTTHUMG000001	KLHL29	kelch like family member 29	Other	other		1.254733042	0	0.45027128	0	1	0	9.25090678	9.37133881	9.5481868	9.5867518
4907	ENSG0000011977	DNMT3A	protein_coding	chr2	25227855	25342590	-	Human GRCh	OTTHUMG000000	DNMT3A	DNA methyltransferase 3 alpha	Nucleus	enzyme		-1.081011601	0	0.71108421	0	1	0	11.1659218	11.2456335	11.2481702	10.9840491
4908	ENSG0000011977	TMEM214	protein_coding	chr2	27032910	27041694	+	Human GRCh	OTTHUMG000001	TMEM214	transmembrane protein 214	Extracellular	other		-1.070499356	0	0.75541021	0	1	0	11.2159501	11.5573419	11.4541773	11.1712104
4909	ENSG0000011977	ATAD2B	protein_coding	chr2	23748664	23927123	-	Human GRCh	OTTHUMG000001	ATAD2B	ATPase family AAA domain containing 2B	Nucleus	enzyme		-1.055405573	0	0.79875573	0	1	0	11.5726907	11.8113369	11.7873461	11.4647133
4910	ENSG0000011978	FKBP1B	protein_coding	chr2	24049701	24063681	+	Human GRCh	OTTHUMG000001	FKBP1B	FKBP prolyl isomerase 1B	Cytoplasm	enzyme		-1.015960518	0	0.97124734	0	1	0	6.20037184	6.50177352	6.42653299	6.25507263
4911	ENSG0000011978	ATL2	protein_coding	chr2	38293954	38377285	-	Human GRCh	OTTHUMG000001	ATL2	atlastin GTPase 2	Cytoplasm	other		-1.000793789	0	0.99681788	0	1	0	10.6176523	10.7638663	10.8811788	10.4668703
4912	ENSG0000011980	YPEL5	protein_coding	chr2	30146941	30160533	+	Human GRCh	OTTHUMG000000	YPEL5	yippee like 5	Nucleus	other		-1.304674619	0	0.24800959	0	1	0	9.56967571	9.86003622	9.56067838	9.27848125
4913	ENSG0000011981	FAM98A	protein_coding	chr2	33532744	33599347	-	Human GRCh	OTTHUMG000001	FAM98A	family with sequence similarity 98 member A	Cytoplasm	enzyme		-1.018293446	0	0.93137479	0	1	0	10.4378094	10.7363477	10.6247101	10.524282
4914	ENSG0000011982	YIPF4	protein_coding	chr2	32277904	32316594	+	Human GRCh	OTTHUMG000001	YIPF4	Yip1 domain family member 4	Cytoplasm	other		-1.147220863	0	0.4935222	0	1	0	10.6429157	10.8979039	10.743513	10.4939071
4915	ENSG0000011984	AFTPH	protein_coding	chr2	64524299	64593005	+	Human GRCh	OTTHUMG000001	AFTPH	aftiphilin	Cytoplasm	other		1.053810445	0	0.80556065	0	1	0	10.4204095	10.6273634	10.6935744	10.4668212
4916	ENSG0000011986	LGALSL	protein_coding	chr2	64453969	64461381	+	Human GRCh	OTTHUMG000001	LGALSL	galectin like	Other	other		1.040798062	0	0.86813736	0	1	0	9.227509	9.56230992	9.62558224	9.24644372
4917	ENSG0000011986	CNRIP1	protein_coding	chr2	68284171	68320051	-	Human GRCh	OTTHUMG000001	CNRIP1	cannabinoid receptor interacting protein 1	Extracellular	other		1	0	1	0	1	0	1.53946588	1.60980674	1.5984383	1.54370093
4918	ENSG0000011986	BCL11A	protein_coding	chr2	60450520	60554467	-	Human GRCh	OTTHUMG000001	BCL11A	BCL11 transcription factor A	Nucleus	transcription		-1.211134151	0	0.41541034	0	1	0	9.13153526	9.2250922	9.01640226	8.91716469
4919	ENSG0000011987	CRIPT	protein_coding	chr2	46616416	46630176	+	Human GRCh	OTTHUMG000001	CRIPT	CXXC repeat containing interactor of PDZ3 domain	Cytoplasm	other		-1.199720787	0	0.46402492	0	1	0	8.7930274	9.15945722	8.80245349	8.79451546
4920	ENSG0000011988	EPCAM	protein_coding	chr2	47345158	47387601	+	Human GRCh	OTTHUMG000001	EPCAM	epithelial cell adhesion molecule	Plasma Mem	other	monoclon	1.023771235	0	0.9257178	0	1	0	10.4948082	10.7640309	10.8823991	10.3892989
4921	ENSG0000011989	SLC17A5	protein_coding	chr6	73593379	73653992	-	Human GRCh	OTTHUMG000000	SLC17A5	solute carrier family 17 member 5	Plasma Mem	transporter		-1.02341317	0	0.92370087	0	1	0	9.41960845	9.72798247	9.66054919	9.44631929
4922	ENSG0000011990	OGFRL1	protein_coding	chr6	71288811	71309059	+	Human GRCh	OTTHUMG000000	OGFRL1	opioid growth factor receptor like 1	Other	other		1.130300647	0	0.5414504	0	1	0	9.77131906	10.0217155	10.1784415	9.86877174
4923	ENSG0000011990	SLF2	protein_coding	chr10	100912963	100965134	+	Human GRCh	OTTHUMG000000	SLF2	SMC5-SMC6 complex localization factor 2	Extracellular	other		-1.048955822	0	0.83089693	0	1	0	10.9165964	11.2367755	11.1564534	10.8984119
4924	ENSG0000011991	IDE	protein_coding	chr10	92451684	92574096	-	Human GRCh	OTTHUMG000000	IDE	insulin degrading enzyme	Extracellular	peptidase	bacitracin	-1.139233181	0	0.53793907	0	1	0	10.5485751	10.885764	10.7041121	10.4514074
4927	ENSG0000011991	IFIT3	protein_coding	chr10	89327307	89377473	+	Human GRCh	OTTHUMG000000	IFIT3	interferon induced protein with tetratricopeptide repeats 3	Cytoplasm	other		1	0	1	0	1	0	1.36889267	1.45470537	1.44094141	1.37410469
4928	ENSG0000011991	NKX2-3	protein_coding	chr10	99532942	99536524	+	Human GRCh	OTTHUMG000000	NKX2-3	NK2 homeobox 3	Nucleus	transcription		1.032144396	0	0.96176121	0	1	0	4.33677239	4.59201014	4.81527643	4.07035446
4929	ENSG0000011992	IFIT2	protein_coding	chr10	89283694	89309271	+	Human GRCh	OTTHUMG000000	IFIT2	interferon induced protein with tetratricopeptide repeats 2	Cytoplasm	other		1	0	1	0	1	0	1.32996235	1.36748161	1.36131766	1.33217868
4930	ENSG0000011992	GPAM	protein_coding	chr10	112149865	112215377	-	Human GRCh	OTTHUMG000000	GPAM	glycerol-3-phosphate acyltransferase, mitochondrial	Cytoplasm	enzyme		-1.127107036	0	0.60343775	0	1	0	9.40256521	9.73092296	9.54654032	9.33935306
4931	ENSG0000011992	CUTC	protein_coding	chr10	99702558	99756134	+	Human GRCh	OTTHUMG000000	CUTC	cutC copper transporter	Cytoplasm	other		1.0215378	0	0.92918891	0	1	0	7.86374508	8.13765314	8.13930244	7.91272435
4932	ENSG0000011993	PPP1R3C	protein_coding	chr10	91628442	91633071	-	Human GRCh	OTTHUMG000000	PPP1R3C	protein phosphatase 1 regulatory subunit 3C	Cytoplasm	phosphatase		5.472793673	1	0.2027713	0	1	0	1.75922773	1.83739941	2.45686341	1.76393927
4933	ENSG0000011994	PYROXD2	protein_coding	chr10	98383565	98415182	-	Human GRCh	OTTHUMG000000	PYROXD2	pyridine nucleotide-disulphide oxidoreductase domain 2	Cytoplasm	other		1.177173069	0	0.68728397	0	1	0	5.91146859	5.84066338	6.31406777	5.65787292
4934	ENSG0000011994	CNNM1	protein_coding	chr10	99329356	99394330	+	Human GRCh	OTTHUMG000000	CNNM1	cyclin and CBS domain divalent metal cation transport mediat	Plasma Mem	other		1.216339476	0	0.49760701	0	1	0	9.01920703	9.3502276	9.52913626	9.31002838
4935	ENSG0000011995	MXI1	protein_coding	chr10	110207605	110287365	+	Human GRCh	OTTHUMG000000	MXI1	MAX interactor 1, dimerization protein	Nucleus	transcription		-1.141781877	0	0.5576462	0	1	0	11.1870583	11.6260713	11.3536143	11.1937058
4936	ENSG0000011995	SMNDC1	protein_coding	chr10	110290730	110304938	-	Human GRCh	OTTHUMG000000	SMNDC1	survival motor neuron domain containing 1	Nucleus	other		1.092781308	0	0.65214623	0	1	0	9.68170145	9.69777457	9.9067826	9.65535288
4937	ENSG0000011996	C10orf88	protein_coding	chr10	122930901	122954311	-	Human GRCh	OTTHUMG000000	C10orf88	chromosome 10 open reading frame 88	Other	enzyme		-1.009611844	0	0.96807314	0	1	0	8.59698337	8.72904068	8.93840941	8.2798869
4938	ENSG0000011996	HELLS	protein_coding	chr10	94545329	94613905	+	Human GRCh	OTTHUMG000000	HELLS	helicase, lymphoid specific	Nucleus	enzyme		-1.039431717	0	0.86240351	0	1	0	12.7218043	13.0412464	13.024231	12.6450216
4939	ENSG0000011997	PRLHR	protein_coding	chr10	118589997	118595648	-	Human GRCh	OTTHUMG000000	PRLHR	prolactin releasing hormone receptor	Plasma Mem	G-protein cou		3.515599638	1	0.17796562	0	1	0	3.69619847	3.98020364	5.26298237	4.19433066
4940	ENSG0000011997	TCTN3	protein_coding	chr10	95659823	95694143	-	Human GRCh	OTTHUMG000000	TCTN3	tectonic family member 3	Cytoplasm	other		-1.129853468	0	0.53664727	0	1	0	9.87365074	10.0145012	9.89381216	9.71807669
4941	ENSG0000011997	DENND10	protein_coding	chr10	119104086	119137984	+	Human GRCh	OTTHUMG000000	DENND10	DENN domain containing 10	Cytoplasm	other		-1.102242669	0	0.69134203	0	1	0	9.02923247	9.44823176	9.19922423	9.09570634
4942	ENSG0000011998	AVPI1	protein_coding	chr10	97677424	97687241	-	Human GRCh	OTTHUMG000000	AVPI1	arginine vasopressin induced 1	Other	other		1.147832969	0	0.69636814	0	1	0	6.33275376	6.18283233	6.55686211	6.22677183
4943	ENSG0000012000	WDR11	protein_coding	chr10	120851305	120909524	+	Human GRCh	OTTHUMG000000	WDR11	WD repeat domain 11	Cytoplasm	other		-1.147597618	0	0.59763074	0	1	0	10.7733011	11.0878341	10.9588958	10.5983681
4944	ENSG0000012002	ARMH3	protein_coding	chr10	101845599	102056193	-	Human GRCh	OTTHUMG000000	ARMH3	armadillo like helical domain containing 3	Other	other		-1.172273291	0	0.42745705	0	1	0	9.63134067	9.94560094	9.70539232	9.53660095
4945	ENSG0000012004	KCNIP2	protein_coding	chr10	101825974	101843920	-	Human GRCh	OTTHUMG000000	KCNIP2	potassium voltage-gated channel interacting protein 2	Plasma Mem	transporter		-1.054735179	0	0.86439077	0	1	0	7.43509511	7.27440335	7.21826668	7.37798376
4946	ENSG0000012005	CFAP58	protein_coding	chr10	104353833	104455102	+	Human GRCh	OTTHUMG000000	CFAP58	cilia and flagella associated protein 58	Extracellular	other		-1.202342825	0	0.89228628	0	NA	0	1.42524354	2.51296451	2.32051651	1.62298545
4947	ENSG0000012005	GOT1	protein_coding	chr10	99396870	99430624	-	Human GRCh	OTTHUMG000000	GOT1	glutamic-oxaloacetic transaminase 1	Cytoplasm	enzyme		-1.133577735	0	0.59369706	0	1	0	11.1450917	11.4482822	11.226041	11.0960185
4949	ENSG0000012005	C10orf95	protein_coding	chr10	102449837	102451543	-	Human GRCh	OTTHUMG000000	C10orf95	chromosome 10 open reading frame 95	Extracellular	other		1.203038355	0	0.68675458	0	1	0	4.84250167	5.58551908	5.60893408	5.25896556
4950	ENSG0000012005	SFRP5	protein_coding	chr10	97766751	97771999	-	Human GRCh	OTTHUMG000000	SFRP5	secreted frizzled related protein 5	Plasma Mem	transmembra		1	0	1	0	1	0	2.56282582	2.67285303	2.65518119	2.56949949
4951	ENSG0000012006	GNA13	protein_coding	chr17	65009289	65056740	-	Human GRCh	OTTHUMG000001	GNA13	G protein subunit alpha 13	Plasma Mem	enzyme		1.000026329	0	0.99989989	0	1	0	10.7647549	10.920384	11.0231048	10.6354017
4952	ENSG0000012006	HOXB8	protein_coding	chr17	48611377	48615292	-	Human GRCh	OTTHUMG000001	HOXB8	homeobox B8	Nucleus	transcription		1	0	1	0	1	0	2.3168062	2.43077423	2.41253337	2.32374748
4953	ENSG0000012007	KANSL1	protein_coding	chr17	46029916	46225389	-	Human GRCh	OTTHUMG000001	KANSL1	KAT8 regulatory NSL complex subunit 1	Nucleus	other		-1.059299687	0	0.77494036	0	1	0	12.6143597	12.7982771	12.7427062	12.5416582
4954	ENSG0000012007	HOXB5	protein_coding	chr17	48591257	48593779	-	Human GRCh	OTTHUMG000001	HOXB5	homeobox B5	Nucleus	transcription		1.469384325	0	0.54516137	0	1	0	4.98108584	4.70486244	4.95738761	5.29491757
4955	ENSG0000012008	CRHR1	protein_coding	chr17	45784277	45835828	+	Human GRCh	OTTHUMG000001	CRHR1	corticotropin releasing hormone receptor 1	Plasma Mem	G-protein cou	tildacerfor	-3.785717542	-1	0.07569683	0	1	0	1.65954441	3.86814515	2.39575793	2.29080785
4956	ENSG0000012009	HOXB3	protein_coding	chr17	48548870	48604912	-	Human GRCh	OTTHUMG000001	HOXB3	homeobox B3	Nucleus	transcription		-1.098198092	0	0.85455245	0	1	0	6.58523322	6.36870989	6.47250255	6.33213681
4958	ENSG0000012012	DUSP1	protein_coding	chr5	172768096	172771195	-	Human GRCh	OTTHUMG000001	DUSP1	dual specificity phosphatase 1	Nucleus	phosphatase		1.085153502	0	0.73864104	0	1	0	11.3235437	11.4138673	11.5362703	11.388754
4959	ENSG0000012013	PANK3	protein_coding	chr5	168548495	168579368	-	Human GRCh	OTTHUMG000001	PANK3	pantothenate kinase 3	Cytoplasm	kinase		1.00761707	0	0.97159953	0	1	0	12.5187425	12.7305193	12.814016	12.4303534
4960	ENSG0000012014	MSX2	protein_coding	chr5	174724582	174730896	+	Human GRCh	OTTHUMG000001	MSX2	msh homeobox 2	Nucleus	transcription		-1.37137855	0	0.7545643	0	1	0	1.75474981	2.68052219	1.83520859	2.34011158
4962	ENSG0000012015	RCL1	protein_coding	chr9	4792944	4885917	+	Human GRCh	OTTHUMG000000	RCL1	RNA terminal phosphate cyclase like 1	Nucleus	enzyme		-1.016979928	0	0.93938776	0	1	0	9.58343184	9.98154356	9.90334638	9.64296669
4963	ENSG0000012015	CAAP1	protein_coding	chr9	26840685	26892803	-	Human GRCh	OTTHUMG000000	CAAP1	caspase activity and apoptosis inhibitor 1	Nucleus	other		1.074534464	0	0.75545592	0	1	0	8.53796813	8.70259609	8.94234627	8.39486022
4965	ENSG0000012016	MOB3B	protein_coding	chr9	27325209	27529814	-	Human GRCh	OTTHUMG000000	MOB3B	MOB kinase activator 3B	Other	other		-1.243119345	0	0.43889393	0	1	0	8.75483527	9.2031368	8.70262314	8.77187711
4969	ENSG0000012021	CD274	protein_coding	chr9	5450503	5470566	+	Human GRCh	OTTHUMG000000	CD274	CD274 molecule-PD-L1 (Programmed Death-Ligand 1)	Plasma Mem	transmembra	GNC-038, (	-1.647532362	-1	0.50854515	0	1	0	2.96204254	3.08701501	2.53307138	2.96958051
4972	ENSG0000012025	GRIA2	protein_coding	chr4	157204182	157387146	+	Human GRCh	OTTHUMG000001	GRIA2	glutamate ionotropic receptor AMPA type subunit 2	Plasma Mem	ion channel	talampane	2.632744335	1	0.12631137	0	1	0	3.52759067	4.40257017	5.26070817	4.32339191
4973	ENSG0000012025	NUP43	protein_coding	chr6	149724315	149749665	-	Human GRCh	OTTHUMG000000	NUP43	nucleoporin 43	Nucleus	transporter		-1.108006285	0	0.58245198	0	1	0	11.1468151	11.4248048	11.2717033	11.0798805
4974	ENSG0000012025	MTHFD1L	protein_coding	chr6	150865679	151101887	+	Human GRCh	OTTHUMG000000	MTHFD1L	methylenetetrahydrofolate dehydrogenase (NADP+ dependen	Cytoplasm	enzyme		-1.240258326	0	0.26190576	0	1	0	10.9163745	11.0367101	10.9049261	10.5426525
4975	ENSG0000012025	LRP11	protein_coding	chr6	149818757	149864359	-	Human GRCh	OTTHUMG000000	LRP11	LDL receptor related protein 11	Other	other		1.063051028	0	0.81198454	0	1	0	9.03980194	9.29892578	9.27216081	9.20778363
4977	ENSG0000012026	PCMT1	protein_coding	chr6	149749444	149811420	+	Human GRCh	OTTHUMG000000	PCMT1	protein-L-isoaspartate (D-aspartate) O-methyltransferase	Cytoplasm	enzyme		-1.064376313	0	0.77736478	0	1	0	9.82244989	10.2718789	10.1738456	9.79461188
4978	ENSG0000012027	PLEKHG1	protein_coding	chr6	150599883	150843665	+	Human GRCh	OTTHUMG000000	PLEKHG1	pleckstrin homology and RhoGEF domain containing G1	Extracellular	other		2.005990263	1	0.30600024	0	1	0	3.07780277	3.21827161	3.82167831	3.08634747
4982	ENSG0000012030	CYSTM1	protein_coding	chr5	140175156	140282052	+	Human GRCh	OTTHUMG000001	CYSTM1	cysteine rich transmembrane module containing 1	Cytoplasm	other		1.195254852	0	0.56056173	0	1	0	9.00790593	9.31289925	9.40318171	9.33290939
4983	ENSG0000012031	WDR55	protein_coding	chr5	140664868	140674124	+	Human GRCh	OTTHUMG000001	WDR55	WD repeat domain 55	Nucleus	other		-1.109565984	0	0.63494733	0	1	0	10.7151805	10.9500438	10.8642602	10.558858
4984	ENSG0000012031	ARAP3	protein_coding	chr5	141653401	141682230	-	Human GRCh	OTTHUMG000001	ARAP3	ArfGAP with RhoGAP domain, ankyrin repeat and PH domain 3	Cytoplasm	other		-1.494943678	0	0.59311284	0	1	0	3.60677585	2.59016272	2.57424987	3.35708651
4985	ENSG0000012032	PCDHB8	protein_coding	chr5	141177790	141180539	+	Human GRCh	OTTHUMG000001	PCDHB8	protocadherin beta 8	Plasma Mem	other		1.372412055	0	0.38251223	0	1	0	7.15024974	7.27652442	7.98947077	6.81416653

4986	ENSG0000012032	PCDHB10	protein_coding	chr5	141192353	141195647	+	Human GRCh	OTTHUMG000001	PCDHB10	protocadherin beta 10	Other	other		2.072279877	1	0.17488607	0	1	0	4.17443732	4.84512049	5.56235605	4.60281199
4987	ENSG0000012032	PCDHB14	protein_coding	chr5	141223343	141227759	+	Human GRCh	OTTHUMG000001	PCDHB14	protocadherin beta 14	Plasma Mem	other		1.176096387	0	0.60705707	0	1	0	7.29603336	7.24952418	7.67022481	7.16507322
4988	ENSG0000012032	PCDHB12	protein_coding	chr5	141208697	141212571	+	Human GRCh	OTTHUMG000001	PCDHB12	protocadherin beta 12	Plasma Mem	other		1.46511427	0	0.37157041	0	1	0	5.47637191	5.53281215	6.20070094	5.39877456
4989	ENSG0000012032	SLC25A2	protein_coding	chr5	141302635	141304049	-	Human GRCh	OTTHUMG000001	SLC25A2	solute carrier family 25 member 2	Cytoplasm	transporter		-2.060986479	-1	0.33471726	0	1	0	3.52370071	2.87207099	2.85220801	2.75649414
4991	ENSG0000012033	MRPS14	protein_coding	chr1	175010789	175023425	-	Human GRCh	OTTHUMG000000	MRPS14	mitochondrial ribosomal protein S14	Cytoplasm	other		1.060300377	0	0.79386037	0	1	0	9.46033265	9.80013471	9.75337773	9.65804411
4992	ENSG0000012033	CENPL	protein_coding	chr1	173799550	173824883	-	Human GRCh	OTTHUMG000000	CENPL	centromere protein L	Cytoplasm	other		-1.064591747	0	0.77027529	0	1	0	9.63756683	10.0221583	9.91110531	9.62544116
4994	ENSG0000012034	SEC16B	protein_coding	chr1	177923956	177984303	-	Human GRCh	OTTHUMG000001	SEC16B	SEC16 homolog B, endoplasmic reticulum export factor	Nucleus	other		-1.541526974	-1	0.55413542	0	1	0	3.01811464	2.74899423	2.02792346	3.02690792
4995	ENSG0000012037	GORAB	protein_coding	chr1	170531819	170553834	+	Human GRCh	OTTHUMG000000	GORAB	golgin, RAB6 interacting	Cytoplasm	other		-1.125218289	0	0.59461553	0	1	0	8.87510068	9.0977938	8.97961348	8.73998806
4997	ENSG0000012043	ACAT2	protein_coding	chr6	159762045	159779112	+	Human GRCh	OTTHUMG000000	ACAT2	acetyl-CoA acetyltransferase 2	Cytoplasm	enzyme		-1.157058824	0	0.57754281	0	1	0	11.0595336	11.6619582	11.3176542	11.1362558
4998	ENSG0000012043	TCP1	protein_coding	chr6	159778498	159789703	-	Human GRCh	OTTHUMG000000	TCP1	t-complex 1	Cytoplasm	other		-1.074432502	0	0.72552921	0	1	0	12.4866173	12.8562856	12.7094311	12.4906933
5000	ENSG0000012045	SNX19	protein_coding	chr11	130866250	130916479	-	Human GRCh	OTTHUMG000001	SNX19	sorting nexin 19	Cytoplasm	transporter		-1.083838665	0	0.70995021	0	1	0	11.6138394	11.9202122	11.8479053	11.5018587
5001	ENSG0000012045	KCNJ5	protein_coding	chr11	128891356	128921163	+	Human GRCh	OTTHUMG000001	KCNJ5	potassium inwardly rectifying channel subfamily J member 5	Plasma Mem	ion channel	minoxidil,	-5.773847833	-1	0.25702509	0	NA	0	1.60078827	2.0576142	1.6184164	1.60201595
5002	ENSG0000012045	MSANTD2	protein_coding	chr11	124766498	124800706	-	Human GRCh	OTTHUMG000001	MSANTD2	Myb/SANT DNA binding domain containing 2	Other	other		1.055707993	0	0.8352836	0	1	0	8.8709887	8.74042097	9.08319092	8.59971816
5003	ENSG0000012047	TP53AIP1	protein_coding	chr11	128934731	128943399	-	Human GRCh	OTTHUMG000001	TP53AIP1	tumor protein p53 regulated apoptosis inducing protein 1	Cytoplasm	other		-1.077788764	0	0.92235604	0	1	0	3.09890595	4.22453908	3.70355594	3.79103556
5006	ENSG0000012050	PDZD11	protein_coding	chrX	70281118	70290514	-	Human GRCh	OTTHUMG000000	PDZD11	PDZ domain containing 11	Extracellular	other		-1.162319028	0	0.55953996	0	1	0	9.69644096	10.0967172	9.87646864	9.59900172
5007	ENSG0000012051	SLC10A7	protein_coding	chr4	146253975	146522372	-	Human GRCh	OTTHUMG000001	SLC10A7	solute carrier family 10 member 7	Cytoplasm	transporter		-1.024922706	0	0.91777486	0	1	0	9.5077649	9.70969286	9.76219348	9.37777616
5008	ENSG0000012052	NUDCD1	protein_coding	chr8	109240919	109334121	-	Human GRCh	OTTHUMG000001	NUDCD1	NudC domain containing 1	Nucleus	other		-1.003607135	0	0.98760799	0	1	0	10.0077071	10.268447	10.3353891	9.90893942
5009	ENSG0000012053	ENY2	protein_coding	chr8	109334324	109345954	+	Human GRCh	OTTHUMG000001	ENY2	ENY2 transcription and export complex 2 subunit	Nucleus	transcription		1.033944314	0	0.87585572	0	1	0	9.6235048	9.94699077	9.96042035	9.6870109
5010	ENSG0000012053	MASTL	protein_coding	chr10	27154824	27187953	+	Human GRCh	OTTHUMG000000	MASTL	microtubule associated serine/threonine kinase like	Cytoplasm	kinase	greatwall k	-1.210048048	0	0.38659043	0	1	0	10.0568437	10.5526999	10.223546	10.0057334
5011	ENSG0000012054	KIAA1217	protein_coding	chr10	23694746	24547848	+	Human GRCh	OTTHUMG000000	KIAA1217	KIAA1217	Cytoplasm	other		1.003521051	0	0.99690544	0	1	0	5.75993242	6.68226443	5.21550371	6.84819628
5013	ENSG0000012059	PLXDC2	protein_coding	chr10	19816239	20289856	+	Human GRCh	OTTHUMG000000	PLXDC2	plexin domain containing 2	Extracellular	other		1.073092108	0	0.94287526	0	1	0	7.28309327	7.34774942	6.96253455	7.7131459
5014	ENSG0000012061	EPC1	protein_coding	chr10	32267751	32378798	-	Human GRCh	OTTHUMG000000	EPC1	enhancer of polycomb homolog 1	Nucleus	transcription		-1.050764521	0	0.81833946	0	1	0	10.7009581	10.9789515	10.8897997	10.6907651
5015	ENSG0000012064	IQSEC3	protein_coding	chr12	66767	178455	+	Human GRCh	OTTHUMG000001	IQSEC3	IQ motif and Sec7 domain ArfGEF 3	Cytoplasm	other		-1.144000552	0	0.92895494	0	1	0	2.98854457	1.17880879	2.88850217	1.06264261
5016	ENSG0000012064	CCDC77	protein_coding	chr12	389273	442642	+	Human GRCh	OTTHUMG000001	CCDC77	coiled-coil domain containing 77	Cytoplasm	other		-1.24814831	0	0.32319091	0	1	0	8.63598944	9.04253462	8.76490483	8.44323591
5017	ENSG0000012065	TAF12	protein_coding	chr1	28587829	28648291	-	Human GRCh	OTTHUMG000000	TAF12	TATA-box binding protein associated factor 12	Nucleus	transcription		1.014512243	0	0.96142471	0	1	0	8.82953491	9.1455513	9.0221024	9.00479866
5018	ENSG0000012065	ENOX1	protein_coding	chr13	43213130	43786972	-	Human GRCh	OTTHUMG000000	ENOX1	ecto-NOX disulfide-thiol exchanger 1	Plasma Mem	enzyme		-1.202473395	0	0.54475128	0	1	0	7.2042638	7.44093295	7.26171509	6.98779196
5019	ENSG0000012065	TNFSF11	protein_coding	chr13	42562736	42608013	+	Human GRCh	OTTHUMG000000	TNFSF11	TNF superfamily member 11	Extracellular	cytokine	JMT103, d	1	0	1	0	NA	0	0.45777152	0.47381908	0.47116387	0.4587116
5020	ENSG0000012066	MTRF1	protein_coding	chr13	41216369	41263577	-	Human GRCh	OTTHUMG000000	MTRF1	mitochondrial translation release factor 1	Cytoplasm	translation re		-1.025003291	0	0.92186293	0	1	0	7.16996279	7.5640376	7.41557241	7.29199102
5022	ENSG0000012066	SOHLH2	protein_coding	chr13	36168217	36214588	-	Human GRCh	OTTHUMG000000	SOHLH2	spermatogenesis and oogenesis specific basic helix-loop-helix	Nucleus	transcription		-1.001701178	0	0.99546099	0	1	0	9.15997825	9.57644948	9.51865757	9.23194506
5024	ENSG0000012068	PROSER1	protein_coding	chr13	39009865	39038089	-	Human GRCh	OTTHUMG000000	PROSER1	proline and serine rich 1	Other	other		-1.068318779	0	0.71363418	0	1	0	11.6304769	11.8951954	11.7294815	11.663007
5025	ENSG0000012068	UFM1	protein_coding	chr13	38349849	38363619	+	Human GRCh	OTTHUMG000000	UFM1	ubiquitin fold modifier 1	Cytoplasm	other		-1.012399807	0	0.95387091	0	1	0	10.589542	10.76198	10.8395384	10.4605933
5026	ENSG0000012068	WBP4	protein_coding	chr13	41061509	41084006	+	Human GRCh	OTTHUMG000000	WBP4	WW domain binding protein 4	Cytoplasm	other		1.237185443	0	0.37937866	0	1	0	7.59623418	7.69770462	7.92549021	7.82201123
5027	ENSG0000012069	ELF1	protein_coding	chr13	40931924	41061440	-	Human GRCh	OTTHUMG000000	ELF1	E74 like ETS transcription factor 1	Nucleus	transcription		-1.036579891	0	0.87167796	0	1	0	8.45556746	8.80946476	8.72316687	8.47798647
5028	ENSG0000012069	SMAD9	protein_coding	chr13	36844831	36920887	-	Human GRCh	OTTHUMG000000	SMAD9	SMAD family member 9	Nucleus	transcription		-1.166722239	0	0.58045448	0	1	0	9.48676227	10.0876495	9.40776497	9.86735814
5029	ENSG0000012069	HSPH1	protein_coding	chr13	31134973	31162388	-	Human GRCh	OTTHUMG000000	HSPH1	heat shock protein family H (Hsp110) member 1	Cytoplasm	other		1.01980448	0	0.91897411	0	1	0	11.2617049	11.2849826	11.428576	11.1448637
5030	ENSG0000012069	KBTBD7	protein_coding	chr13	41189834	41194569	-	Human GRCh	OTTHUMG000000	KBTBD7	kelch repeat and BTB domain containing 7	Cytoplasm	other		1.176732507	0	0.52754476	0	1	0	9.04079842	9.24205774	9.52846077	9.08413864
5031	ENSG0000012069	ALG5	protein_coding	chr13	36949738	37000763	-	Human GRCh	OTTHUMG000000	ALG5	ALG5 dolichyl-phosphate beta-glucosyltransferase	Cytoplasm	enzyme		-1.132230984	0	0.53378705	0	1	0	10.2565827	10.4843258	10.2804352	10.1922804
5032	ENSG0000012069	EXOSC8	protein_coding	chr13	36998816	37025881	+	Human GRCh	OTTHUMG000000	EXOSC8	exosome component 8	Nucleus	enzyme		1.010055279	0	0.96255668	0	1	0	10.1525436	10.2993456	10.3910113	10.0651129
5033	ENSG0000012070	ETF1	protein_coding	chr5	138506095	138543236	-	Human GRCh	OTTHUMG000001	ETF1	eukaryotic translation termination factor 1	Cytoplasm	translation re		-1.109637603	0	0.60323841	0	1	0	11.7460201	12.143539	11.9720522	11.6988215
5034	ENSG0000012070	TGFBI	protein_coding	chr5	136028988	136063818	+	Human GRCh	OTTHUMG000001	TGFBI	transforming growth factor beta induced	Extracellular	other		-1.081508133	0	0.95025023	0	1	0	1.56865343	2.15702529	1.60196429	2.06553091
5035	ENSG0000012070	FAM53C	protein_coding	chr5	138331935	138349729	+	Human GRCh	OTTHUMG000001	FAM53C	family with sequence similarity 53 member C	Other	other		-1.116322068	0	0.58755511	0	1	0	10.7603694	11.0158994	10.8507483	10.6863335
5036	ENSG0000012072	SIL1	protein_coding	chr5	138946724	139293557	-	Human GRCh	OTTHUMG000001	SIL1	SIL1 nucleotide exchange factor	Cytoplasm	transporter		-1.010690926	0	0.95897729	0	1	0	9.65686375	9.78332806	9.82759055	9.57988026
5037	ENSG0000012072	PAIP2	protein_coding	chr5	139341587	139369720	+	Human GRCh	OTTHUMG000001	PAIP2	poly(A) binding protein interacting protein 2	Cytoplasm	translation re		-1.145607501	0	0.53832273	0	1	0	10.629915	10.9593542	10.722922	10.5843771
5039	ENSG0000012073	KDM3B	protein_coding	chr5	138352685	138437028	+	Human GRCh	OTTHUMG000001	KDM3B	lysine demethylase 3B	Nucleus	enzyme		-1.141459901	0	0.54868145	0	1	0	11.8320727	12.1364903	11.9786097	11.7017799
5040	ENSG0000012073	EGR1	protein_coding	chr5	138465479	138469303	+	Human GRCh	OTTHUMG000001	EGR1	early growth response 1	Nucleus	transcription		-1.072581416	0	0.82808011	0	1	0	9.99605618	9.94030588	9.81110607	9.96169997
5041	ENSG0000012074	SERP1	protein_coding	chr3	150541998	150603228	-	Human GRCh	OTTHUMG000001	SERP1	stress associated endoplasmic reticulum protein 1	Cytoplasm	other		1.158132378	0	0.50321493	0	1	0	11.9917103	12.1410253	12.4160261	12.0173213
5042	ENSG0000012075	PLS1	protein_coding	chr3	142596393	142713664	+	Human GRCh	OTTHUMG000001	PLS1	plastin 1	Plasma Mem	other		1.383345233	0	0.22514298	0	1	0	8.42271501	8.32145352	8.89618832	8.47865634
5043	ENSG0000012078	ZFP30	protein_coding	chr19	37613749	37692337	-	Human GRCh	OTTHUMG000000	ZFP30	ZFP30 zinc finger protein	Nucleus	transcription		-1.083183693	0	0.72115457	0	1	0	9.32939971	9.40727649	9.5033669	9.01189097
5044	ENSG0000012079	NR2C1	protein_coding	chr12	95020229	95073628	-	Human GRCh	OTTHUMG000001	NR2C1	nuclear receptor subfamily 2 group C member 1	Nucleus	transcription		-1.101786075	0	0.65484392	0	1	0	10.3071677	10.5380658	10.5329794	10.0682348
5045	ENSG0000012080	UTP20	protein_coding	chr12	101280105	101386618	+	Human GRCh	OTTHUMG000001	UTP20	UTP20 small subunit processome component	Nucleus	other		-1.097434321	0	0.66343453	0	1	0	11.0064266	11.437544	11.2921786	10.9614089
5046	ENSG0000012080	TMPO	protein_coding	chr12	98515579	98550351	+	Human GRCh	OTTHUMG000001	TMPO	thymopoietin	Nucleus	other		1.144955976	0	0.53976603	0	1	0	13.147341	13.5410865	13.7276444	13.2375548
5047	ENSG0000012080	ARL1	protein_coding	chr12	101393116	101407772	-	Human GRCh	OTTHUMG000001	ARL1	ADP ribosylation factor like GTPase 1	Cytoplasm	enzyme		1.045053345	0	0.84014997	0	1	0	10.9633701	11.2502643	11.3732675	10.9116188
5048	ENSG0000012082	GLT8D2	protein_coding	chr12	103988987	104064183	-	Human GRCh	OTTHUMG000001	GLT8D2	glycosyltransferase 8 domain containing 2	Other	enzyme		30.30405637	1	0.00218806	1	0.96	0	2.12278061	2.22085292	3.80894171	2.70008588
5049	ENSG0000012083	MTERF2	protein_coding	chr12	106977277	106987160	-	Human GRCh	OTTHUMG000001	MTERF2	mitochondrial transcription termination factor 2	Cytoplasm	transcription		-1.06434644	0	0.78918792	0	1	0	8.31336115	8.36868968	8.50968063	7.98879129
5050	ENSG0000012083	SOCS2	protein_coding	chr12	93569814	93583487	+	Human GRCh	OTTHUMG000001	SOCS2	suppressor of cytokine signaling 2	Cytoplasm	other		-1.000275978	0	0.99975007	0	1	0	3.96681354	3.14900809	3.1233628	3.97890038
5051	ENSG0000012083	NFYB	protein_coding	chr12	104117086	104138241	-	Human GRCh	OTTHUMG000001	NFYB	nuclear transcription factor Y subunit beta	Nucleus	transcription		1.02915875	0	0.90354481	0	1	0	9.4182097	9.58260285	9.75975459	9.26024521
5052	ENSG0000012086	WASHC3	protein_coding	chr12	102012840	102062149	-	Human GRCh	OTTHUMG000001	WASHC3	WASH complex subunit 3	Cytoplasm	other		-1.101232485	0	0.69677284	0	1	0	8.78504177	9.07567109	8.87091467	8.79497131
5053	ENSG0000012086	APAF1	protein_coding	chr12	98645290	98735433	+	Human GRCh	OTTHUMG000001	APAF1	apoptotic peptidase activating factor 1	Cytoplasm	other		-1.015636296	0	0.9461172	0	1	0	9.48186932	9.79908653	9.81109894	9.42722795
5054	ENSG0000012087	DUSP4	protein_coding	chr8	29333064	29350684	-	Human GRCh	OTTHUMG000001	DUSP4	dual specificity phosphatase 4	Nucleus	phosphatase		1.17086367	0	0.65205924	0	1	0	10.3325279	10.5089	10.9952512	9.99112225
5055	ENSG0000012088	CLU	protein_coding	chr8	27596917	27614700	-	Human GRCh	OTTHUMG000001	CLU	clusterin	Cytoplasm	other	clusterin a	-1.29512203	0	0.63563076	0	1	0	7.80626054	6.96759143	6.44035145	7.64944965
5056	ENSG0000012088	TNFRSF10I	protein_coding	chr8	23020133	23069031	-	Human GRCh	OTTHUMG000000	TNFRSF10B	TNF receptor superfamily member 10b	Plasma Mem	transmembra	dulanermi	-1.010163246	0	0.97238727	0	1	0	11.5500541	11.7311939	11.7283478	11.5273544
5057	ENSG0000012089	SORBS3	protein_coding	chr8	22544986	22575788	+	Human GRCh	OTTHUMG000001	SORBS3	sorbin and SH3 domain containing 3	Cytoplasm	other		1.935628125	1	0.31725489	0	1	0	6.92485241	6.96884397	8.06785354	6.89886117
5058	ENSG0000012089	PTK2B	protein_coding	chr8	27311482	27459391	+	Human GRCh	OTTHUMG000001	PTK2B	protein tyrosine kinase 2 beta	Cytoplasm	kinase	PF-431396	-1.054958318	0	0.84044448	0	1	0	9.50820954	9.89731904	9.79618665	9.50363077
5059	ENSG0000012090	CHRNA2	protein_coding	chr8	27459756	27479883	-	Human GRCh	OTTHUMG000001	CHRNA2	cholinergic receptor nicotinic alpha 2 subunit	Plasma Mem	transmembra	carbamylcl	-1.364230142	0	0.59442787	0	1	0	9.71905732	10.1032194	9.78745006	9.24265006
5060	ENSG0000012090	ADRA1A	protein_coding	chr8	26748150	26867278	-	Human GRCh	OTTHUMG000000	ADRA1A	adrenoceptor alpha 1A	Plasma Mem	G-protein cou	epinephrin	1.210720059	0	0.7989581	0	1	0	8.81077309	8.02393381	8.86083389	8.53986864
5061	ENSG0000012091	PPP3CC	protein_coding	chr8	22440819	22541142	+	Human GRCh	OTTHUMG000001	PPP3CC	protein phosphatase 3 catalytic subunit gamma	Cytoplasm	phosphatase	voclospori	-1.015932739	0	0.95080362	0	1	0	7.84192579	8.3183952	8.20548668	7.95258292
5062	ENSG0000012091	PDLIM2	protein_coding	chr8	22578279	22598025	+	Human GRCh	OTTHUMG000001	PDLIM2	PDZ and LIM domain 2	Cytoplasm	other		1.827764214	1	0.17623534	0	1	0	4.43356817	4.18918688	5.12842722	4.48666719
5063	ENSG0000012091	EPHX2	protein_coding	chr8	27490781	27545564	+	Human GRCh	OTTHUMG000001	EPHX2	epoxide hydrolase 2	Cytoplasm	enzyme	EC5026	-1.186317807	0	0.49607424	0	1	0	9.49314891	9.72195628	9.68449352	9.06820709
5064	ENSG0000012092	RNF170	protein_coding	chr8	42849637	42897290	-	Human GRCh	OTTHUMG000001	RNF170	ring finger protein 170	Other	other		-1.032707979	0	0.90876292	0	1	0	9.01640329	9.1812968	9.28300423	8.80063165
5065	ENSG0000012093	NPPB	protein_coding	chr1	11857464	11858945	-	Human GRCh	OTTHUMG000000	NPPB	natriuretic peptide B	Extracellular	other		1	0	1	0	NA	0	1.08498792	1.09950722	1.09707918	1.08582823
5066	ENSG0000012094	UBIAD1	protein_coding	chr1	11273198	11296049	+	Human GRCh	OTTHUMG000000	UBIAD1	UbiA prenyltransferase domain containing 1	Nucleus	enzyme		-1.125362986	0	0.55492694	0	1	0	10.0051295	10.2526348	10.09179	9.90918483
5067	ENSG0000012094	TARDBP	protein_coding	chr1	11012344	11030528	+	Human GRCh	OTTHUMG000001	TARDBP	TAR DNA binding protein	Nucleus	transcription		1.088830051	0	0.65990899	0	1	0	11.9459523	12.2281023	12.3724184	11.9695478
5068	ENSG0000012094	TNFRSF8	protein_coding	chr1	12063303	12144207	+	Human GRCh	OTTHUMG000000	TNFRSF8	TNF receptor superfamily member 8	Plasma Mem	transmembra	brentuxim	-1.825893566	-1	0.50170606	0	1	0	2.86056041	3.47192934	2.87510488	2.78071227
5070	ENSG0000012096	ZNF706	protein_coding	chr8	101177878	101206193	-	Human GRCh	OTTHUMG000001	ZNF706	zinc finger protein 706	Other	other		-1.061288246	0	0.77824097	0	1	0	10.5185114	10.6487993	10.6711552	10.3503289
5071	ENSG0000012099	LYPLA1	protein_coding	chr8	54046367	54102017	-	Human GRCh	OTTHUMG000001	LYPLA1	lysophospholipase 1	Cytoplasm	enzyme		1.118042152	0	0.61579956	0	1	0	10.8801909	11.0194306	11.2874676	10.8129128
5072	ENSG0000012100	CRISPLD1	protein_coding	chr8	74984505	75034558	+	Human GRCh	OTTHUMG000001	CRISPLD1	cysteine rich secretory protein LCCL domain containing 1	Cytoplasm	other		1.387123563	0	0.42467635	0	1	0	5.31616916	5.51997328	5.53630682	5.77512162
5073	ENSG0000012102	COPS5	protein_coding	chr8	67043079	67083783	-	Human GRCh	OTTHUMG000001	COPS5	COP9 signalosome subunit 5	Nucleus	transcription		-1.073421042	0	0.70979744	0	1	0	10.3064388	10.5671804	10.5105554	10.2027612
5074	ENSG0000012103	RDH10	protein_coding	chr8	73294602	73325281	+	Human GRCh	OTTHUMG000001	RDH10	retinol dehydrogenase 10	Nucleus	enzyme		-1.047234849	0	0.91891927	0	1	0	11.2777688	11.5663791	11.6202389	11.0725044
5076	ENSG0000012105	AKAP1	protein_coding	chr17	57085092	57121346	+	Human GRCh	OTTHUMG000001	AKAP1	A-kinase anchoring protein 1	Cytoplasm	other		-1.050235717	0	0.83865085	0	1	0	12.0037065	12.3104819	12.1872302	12.0339019
5077	ENSG0000012105	COIL	protein_coding	chr17	56938199	56961050	-	Human GRCh	OTTHUMG000001	COIL	coilin	Nucleus	enzyme		-1.034327875	0	0.87464373	0	1	0	9.52952966	9.88484093	9.84798457	9.49144544
5078	ENSG0000012106	TRIM25	protein_coding	chr17	56836387	56914080	-	Human GRCh	OTTHUMG000001	TRIM25	tripartite motif containing 25	Cytoplasm	enzyme		-1.019652589	0	0.92351465	0	1	0	10.9687082	11.3451808	11.2495117	11.0411737
5079	ENSG0000012106	SCPEP1	protein_coding	chr17	56978129	57006768	+	Human GRCh	OTTHUMG000001	SCPEP1	serine carboxypeptidase 1	Cytoplasm	peptidase		1.139346268	0	0.57137315	0	1	0	8.1748372	8.30873251	8.37277416	8.37457776
5080	ENSG0000012106	SPOP	protein_coding	chr17	49598884	49678163	-	Human GRCh	OTTHUMG000001	SPOP	speckle type BTB/POZ protein	Extracellular	other		-1.089930992	0	0.689761	0	1	0	9.80536007	10.1539508	10.0890295	9.67023918

5081	**ENSG0000012106**	TBX2	protein_coding	chr17	61399843	61409466	+	Human GRCh	OTTHUMG000001	TBX2	**T-box transcription factor 2**	Nucleus	transcription		1.005241141	0	0.99108257	0	1	0	7.62159695	7.50236786	7.53000575	7.60747535
5082	**ENSG0000012107**	SLC35B1	protein_coding	chr17	49700934	49709014	-	Human GRCh	OTTHUMG000001	SLC35B1	**solute carrier family 35 member B1**	Cytoplasm	transporter		-1.219756286	0	0.36371263	0	1	0	10.4297087	10.7504034	10.4961288	10.2473802
5084	**ENSG0000012110**	TEX14	protein_coding	chr17	58556678	58692055	-	Human GRCh	OTTHUMG000001	TEX14	**testis expressed 14, intercellular bridge forming factor**	Plasma Mem	kinase		-1.653105253	-1	0.34685053	0	1	0	4.57985846	4.61982801	4.25407993	4.07542566
5085	**ENSG0000012110**	FAM117A	protein_coding	chr17	49710332	49789180	-	Human GRCh	OTTHUMG000001	FAM117A	**family with sequence similarity 117 member A**	Other	transporter		-1.051653468	0	0.86433688	0	1	0	8.61933235	8.97784858	8.82606126	8.6800536
5086	**ENSG0000012115**	NCAPH	protein_coding	chr2	96335766	96377091	+	Human GRCh	OTTHUMG000001	NCAPH	**non-SMC condensin I complex subunit H**	Nucleus	other		-1.289799233	0	0.25031357	0	1	0	10.9718994	11.3926303	11.011998	10.8079906
5087	**ENSG0000012120**	LRAT	protein_coding	chr4	154626945	154753120	+	Human GRCh	OTTHUMG000001	LRAT	**lecithin retinol acyltransferase**	Cytoplasm	enzyme		1.061988178	0	0.88700039	0	1	0	6.69868266	6.91507057	7.16092412	6.51792746
5088	**ENSG0000012121**	TMEM131	protein_coding	chr4	153466346	153636711	+	Human GRCh	OTTHUMG000001	TMEM131L	**transmembrane 131 like**	Other	other		-1.104947284	0	0.70786316	0	1	0	8.47466431	8.98983262	8.50809911	8.78384563
5089	**ENSG0000012121**	MND1	protein_coding	chr4	153344649	153415118	+	Human GRCh	OTTHUMG000001	MND1	**meiotic nuclear divisions 1**	Nucleus	other		-1.085142472	0	0.77850548	0	1	0	7.51295733	7.75832118	7.71758758	7.36519065
5091	**ENSG0000012127**	ABCC11	protein_coding	chr16	48165773	48247568	-	Human GRCh	OTTHUMG000001	ABCC11	**ATP binding cassette subfamily C member 11**	Plasma Mem	transporter		-1.083888814	0	0.89712826	0	1	0	3.72455653	3.98773008	4.23329458	3.07986504
5092	**ENSG0000012127**	TENT4B	protein_coding	chr16	50152911	50235310	+	Human GRCh	OTTHUMG000001	TENT4B	**terminal nucleotidyltransferase 4B**	Nucleus	enzyme		1.17963329	0	0.41476402	0	1	0	9.22775863	9.66963424	9.79974742	9.4873036
5093	**ENSG0000012128**	ADCY7	protein_coding	chr16	50246137	50318135	+	Human GRCh	OTTHUMG000001	ADCY7	**adenylate cyclase 7**	Plasma Mem	enzyme		-1.010339679	0	0.9803832	0	1	0	5.34033725	4.76215068	5.15943566	5.02286212
5094	**ENSG0000012128**	CEP89	protein_coding	chr19	32875925	32971991	-	Human GRCh	OTTHUMG000001	CEP89	**centrosomal protein 89**	Cytoplasm	other		-1.084235047	0	0.68065471	0	1	0	10.6148574	10.7960576	10.7649666	10.4536567
5095	**ENSG0000012129**	TSHZ3	protein_coding	chr19	31149979	31349436	-	Human GRCh	OTTHUMG000001	TSHZ3	**teashirt zinc finger homeobox 3**	Nucleus	transcription		1.237446357	0	0.84876222	0	NA	0	2.29612703	1.87698898	1.8639194	2.48065633
5096	**ENSG0000012131**	ECHDC2	protein_coding	chr1	52895910	52927212	-	Human GRCh	OTTHUMG000000	ECHDC2	**enoyl-CoA hydratase domain containing 2**	Other	other		1.028197049	0	0.90047701	0	1	0	10.958473	10.999845	11.1610292	10.8354065
5099	**ENSG0000012131**	TAS2R10	protein_coding	chr12	10825219	10826358	-	Human GRCh	OTTHUMG000001	TAS2R10	**taste 2 receptor member 10**	Plasma Mem	G-protein cou	denatoniu	-1.387079377	0	0.50189823	0	1	0	4.82099831	4.9565417	4.77975202	4.39665577
5101	**ENSG0000012135**	PYROXD1	protein_coding	chr12	21437615	21471250	+	Human GRCh	OTTHUMG000001	PYROXD1	**pyridine nucleotide-disulphide oxidoreductase domain 1**	Nucleus	other		-1.114824598	0	0.59447987	0	1	0	9.86594203	9.83633423	9.84527809	9.60334845
5102	**ENSG0000012135**	IAPP	protein_coding	chr12	21354959	21379980	+	Human GRCh	OTTHUMG000001	IAPP	**islet amyloid polypeptide**	Extracellular	other		-1.657735783	-1	0.6624807	0	1	0	3.98547356	4.14725413	3.96808041	2.95779328
5106	**ENSG0000012138**	TAS2R9	protein_coding	chr12	10809094	10810168	-	Human GRCh	OTTHUMG000001	TAS2R9	**taste 2 receptor member 9**	Plasma Mem	G-protein cou		-1.53636565	-1	0.69404836	0	NA	0	2.80408206	2.95127817	2.81757795	2.29701424
5107	**ENSG0000012139**	PSPC1	protein_coding	chr13	19674752	19783019	-	Human GRCh	OTTHUMG000000	PSPC1	**paraspeckle component 1**	Nucleus	transcription		1.035002531	0	0.8660814	0	1	0	10.7721581	11.0811561	11.0835103	10.8551848
5108	**ENSG0000012140**	ZNF549	protein_coding	chr19	57527325	57557542	+	Human GRCh	OTTHUMG000001	ZNF549	**zinc finger protein 549**	Other	other		-1.04439157	0	0.87467335	0	1	0	8.55133559	8.68843022	8.74205879	8.37708737
5109	**ENSG0000012141**	A1BG	protein_coding	chr19	58345178	58353492	-	Human GRCh	OTTHUMG000001	A1BG	**alpha-1-B glycoprotein**	Extracellular	other		-1.095952218	0	0.84339014	0	1	0	5.66060477	6.16631993	6.11752926	5.49443284
5110	**ENSG0000012141**	ZSCAN18	protein_coding	chr19	58083838	58118427	-	Human GRCh	OTTHUMG000001	ZSCAN18	**zinc finger and SCAN domain containing 18**	Other	other		2.215220419	1	0.01323494	1	1	0	7.72103527	7.744026	9.07894387	7.78670581
5111	**ENSG0000012141**	ZNF211	protein_coding	chr19	57630395	57644041	+	Human GRCh	OTTHUMG000001	ZNF211	**zinc finger protein 211**	Nucleus	transcription		1.014395314	0	0.95514283	0	1	0	8.08217681	8.18329498	8.38545281	7.85273146
5112	**ENSG0000012144**	PDZRN3	protein_coding	chr3	73382431	73624941	-	Human GRCh	OTTHUMG000001	PDZRN3	**PDZ domain containing ring finger 3**	Extracellular	enzyme		1.176515534	0	0.73572484	0	1	0	6.19929822	5.97197138	6.01939146	6.44792736
5114	**ENSG0000012145**	LHX4	protein_coding	chr1	180230264	180278984	+	Human GRCh	OTTHUMG000000	LHX4	**LIM homeobox 4**	Nucleus	transcription		-1.124408725	0	0.87755933	0	1	0	2.715469	3.58048039	3.55617868	2.52629754
5115	**ENSG0000012148**	RNF2	protein_coding	chr1	185045526	185102603	+	Human GRCh	OTTHUMG000000	RNF2	**ring finger protein 2**	Nucleus	transcription		-1.057401272	0	0.81932031	0	1	0	10.3700256	10.5940405	10.5340617	10.3072656
5116	**ENSG0000012148**	TRMT1L	protein_coding	chr1	185118101	185157072	-	Human GRCh	OTTHUMG000000	TRMT1L	**tRNA methyltransferase 1 like**	Other	enzyme		1.005053927	0	0.98240245	0	1	0	9.39434603	9.622909	9.69723115	9.31061146
5117	**ENSG0000012154**	SEC22A	protein_coding	chr3	123201927	123274136	+	Human GRCh	OTTHUMG000001	SEC22A	**SEC22 homolog A, vesicle trafficking protein**	Cytoplasm	transporter		1.066030945	0	0.79505147	0	1	0	8.45789597	8.64362992	8.80191532	8.41254737
5120	**ENSG0000012157**	POPDC2	protein_coding	chr3	119636457	119665324	-	Human GRCh	OTTHUMG000001	POPDC2	**popeye domain containing 2**	Plasma Mem	other		-2.649725623	-1	0.057428	0	1	0	4.40684255	4.38763372	3.59051543	3.76861884
5121	**ENSG0000012157**	B4GALT4	protein_coding	chr3	119211732	119240946	-	Human GRCh	OTTHUMG000001	B4GALT4	**beta-1,4-galactosyltransferase 4**	Cytoplasm	enzyme		-1.190959676	0	0.39072382	0	1	0	8.43045638	8.8125207	8.49597857	8.40440713
5122	**ENSG0000012157**	NAA50	protein_coding	chr3	113716458	113746300	-	Human GRCh	OTTHUMG000001	NAA50	**N-alpha-acetyltransferase 50, NatE catalytic subunit**	Cytoplasm	enzyme		1.113647269	0	0.57872462	0	1	0	11.834246	12.1796825	12.3205902	11.922384
5124	**ENSG0000012162**	KIF18A	protein_coding	chr11	28020619	28108156	-	Human GRCh	OTTHUMG000001	KIF18A	**kinesin family member 18A**	Cytoplasm	enzyme	sovilnesib	-1.204873945	0	0.44528956	0	1	0	10.1311889	10.5429087	10.3091501	9.96367045
5126	**ENSG0000012164**	DESI2	protein_coding	chr1	244653103	244709033	+	Human GRCh	OTTHUMG000000	DESI2	**desumoylating isopeptidase 2**	Cytoplasm	enzyme		1.020115844	0	0.92240202	0	1	0	11.1964402	11.3477494	11.4675601	11.0964547
5127	**ENSG0000012165**	MAPK8IP1	protein_coding	chr11	45885651	45906465	+	Human GRCh	OTTHUMG000001	MAPK8IP1	**mitogen-activated protein kinase 8 interacting protein 1**	Cytoplasm	other		1.104379441	0	0.67544054	0	1	0	9.89690654	10.0365875	10.1690031	9.97705869
5128	**ENSG0000012167**	CRY2	protein_coding	chr11	45847118	45883248	+	Human GRCh	OTTHUMG000001	CRY2	**cryptochrome circadian regulator 2**	Nucleus	enzyme		-1.13733466	0	0.60022003	0	1	0	10.4588751	10.8211149	10.6606821	10.3354612
5129	**ENSG0000012168**	PEX16	protein_coding	chr11	45909663	45918812	-	Human GRCh	OTTHUMG000001	PEX16	**peroxisomal biogenesis factor 16**	Cytoplasm	other		1.199042026	0	0.47823342	0	1	0	8.82425428	9.1331595	9.34803688	9.00543472
5130	**ENSG0000012169**	DEPDC7	protein_coding	chr11	33015876	33033582	+	Human GRCh	OTTHUMG000001	DEPDC7	**DEP domain containing 7**	Cytoplasm	other		-6.077896619	-1	0.730521	0	NA	0	0.21564083	0.82678099	0.2327063	0.21684964
5131	**ENSG0000012169**	CAT	protein_coding	chr11	34438934	34472060	+	Human GRCh	OTTHUMG000000	CAT	**catalase**	Cytoplasm	enzyme	fomepizole	1.010280739	0	0.96736582	0	1	0	9.61706384	9.95718961	9.92148383	9.68813009
5132	**ENSG0000012171**	PILRB	protein_coding	chr7	100352176	100367831	+	Human GRCh	OTTHUMG000001	PILRB	**paired immunoglobin like type 2 receptor beta**	Plasma Mem	other		-1.119699635	0	0.57487871	0	1	0	11.9034864	12.0459202	11.9920317	11.6901348
5133	**ENSG0000012174**	ZMYM2	protein_coding	chr13	19958677	20091829	+	Human GRCh	OTTHUMG000000	ZMYM2	**zinc finger MYM-type containing 2**	Nucleus	transcription		-1.167149379	0	0.45257806	0	1	0	12.3911054	12.6403041	12.5050449	12.1710656
5135	**ENSG0000012174**	GJA3	protein_coding	chr13	20138255	20161052	-	Human GRCh	OTTHUMG000000	GJA3	**gap junction protein alpha 3**	Plasma Mem	transporter		1.02883524	0	0.98338099	0	1	0	2.96333573	1.94897069	2.36573646	2.78088364
5136	**ENSG0000012174**	TBC1D15	protein_coding	chr12	71839707	71927248	+	Human GRCh	OTTHUMG000001	TBC1D15	**TBC1 domain family member 15**	Cytoplasm	other		-1.128916539	0	0.57328403	0	1	0	10.4097378	10.6746202	10.4517954	10.3826694
5137	**ENSG0000012175**	ADGRB2	protein_coding	chr1	31727117	31764893	-	Human GRCh	OTTHUMG000000	ADGRB2	**adhesion G protein-coupled receptor B2**	Plasma Mem	G-protein cou		1.40006705	0	0.2484647	0	1	0	6.58742455	6.63474685	6.90030484	6.93329796
5138	**ENSG0000012176**	HCRTR1	protein_coding	chr1	31617686	31632518	+	Human GRCh	OTTHUMG000000	HCRTR1	**hypocretin receptor 1**	Plasma Mem	G-protein cou	daridorexa	1	0	1	0	NA	0	0.88562977	0.89964062	0.89729947	0.88644138
5139	**ENSG0000012176**	ZCCHC17	protein_coding	chr1	31296982	31364953	+	Human GRCh	OTTHUMG000000	ZCCHC17	**zinc finger CCHC-type containing 17**	Nucleus	other		-1.00239142	0	0.9912901	0	1	0	8.96596069	9.29055378	9.19484113	9.07512306
5141	**ENSG0000012177**	KHDRBS1	protein_coding	chr1	32013868	32060850	+	Human GRCh	OTTHUMG000000	KHDRBS1	**KH RNA binding domain containing, signal transduction associ**	Nucleus	transcription	CWP23229	-1.026515557	0	0.89930714	0	1	0	12.3597028	12.6731991	12.65587	12.3156943
5142	**ENSG0000012177**	TMEM39B	protein_coding	chr1	32072031	32102866	+	Human GRCh	OTTHUMG000000	TMEM39B	**transmembrane protein 39B**	Cytoplasm	other		-1.105531118	0	0.66053891	0	1	0	8.94295352	8.93569501	8.80675576	8.85494025
5145	**ENSG0000012185**	POLR3GL	protein_coding	chr1	145964690	145978848	+	Human GRCh	OTTHUMG000000	POLR3GL	**RNA polymerase III subunit GL**	Nucleus	other		1.24172925	0	0.50344694	0	1	0	5.92751459	6.53923195	6.69729118	6.21570055
5147	**ENSG0000012185**	TNFSF10	protein_coding	chr3	172505508	172523475	-	Human GRCh	OTTHUMG000001	TNFSF10	**TNF superfamily member 10**	Extracellular	cytokine	ABBV-621	-22.71621603	-1	0.01138902	1	1	0	2.22227416	3.0149248	1.70498454	1.65483072
5148	**ENSG0000012186**	ZNF639	protein_coding	chr3	179323031	179338583	+	Human GRCh	OTTHUMG000001	ZNF639	**zinc finger protein 639**	Nucleus	transcription		-1.279091004	0	0.22022922	0	1	0	10.1097658	10.3920712	10.1613832	9.78637577
5149	**ENSG0000012187**	SLITRK3	protein_coding	chr3	165186720	165197109	-	Human GRCh	OTTHUMG000001	SLITRK3	**SLIT and NTRK like family member 3**	Plasma Mem	other		2.134022594	1	0.13392599	0	1	0	4.14244115	3.31127867	4.54402542	4.25994064
5150	**ENSG0000012187**	PIK3CA	protein_coding	chr3	179148114	179240093	+	Human GRCh	OTTHUMG000001	PIK3CA	**phosphatidylinositol-4,5-bisphosphate 3-kinase catalytic subu**	Cytoplasm	kinase	NVS-PI3-2,	-1.194392544	0	0.3986302	0	1	0	10.3459389	10.568241	10.3710789	10.1571539
5151	**ENSG0000012189**	PDS5A	protein_coding	chr4	39822863	39977956	-	Human GRCh	OTTHUMG000001	PDS5A	**PDS5 cohesin associated factor A**	Nucleus	other		-1.077724857	0	0.71700394	0	1	0	12.9908088	13.2932834	13.2381101	12.8692842
5153	**ENSG0000012189**	LIAS	protein_coding	chr4	39459004	39485109	+	Human GRCh	OTTHUMG000000	LIAS	**lipoic acid synthetase**	Cytoplasm	enzyme		-1.04821284	0	0.84294833	0	1	0	9.52969579	9.57766116	9.7223277	9.23044491
5155	**ENSG0000012190**	TMEM54	protein_coding	chr1	32894594	32901438	-	Human GRCh	OTTHUMG000000	TMEM54	**transmembrane protein 54**	Other	other		-1.074107479	0	0.7764621	0	1	0	8.59175465	8.73178146	8.66017905	8.50778612
5156	**ENSG0000012190**	ZSCAN20	protein_coding	chr1	33472645	33501643	+	Human GRCh	OTTHUMG000000	ZSCAN20	**zinc finger and SCAN domain containing 20**	Nucleus	transcription		1.062480676	0	0.80627127	0	1	0	8.77286268	9.07763122	9.31523974	8.56795455
5157	**ENSG0000012190**	CSMD2	protein_coding	chr1	33513998	34165842	-	Human GRCh	OTTHUMG000000	CSMD2	**CUB and Sushi multiple domains 2**	Other	other		5.333741473	1	0.1836555	0	1	0	2.05380131	2.17120251	2.15240316	2.6691957
5158	**ENSG0000012190**	HPCA	protein_coding	chr1	32885994	32898441	+	Human GRCh	OTTHUMG000000	HPCA	**hippocalcin**	Cytoplasm	other		-2.381667565	-1	0.20115729	0	1	0	3.23980276	4.29223718	2.80942402	3.58344648
5159	**ENSG0000012193**	LRIF1	protein_coding	chr1	110947190	110963965	-	Human GRCh	OTTHUMG000000	LRIF1	**ligand dependent nuclear receptor interacting factor 1**	Nucleus	other		1.2642082	0	0.34986271	0	1	0	9.04720214	9.15769608	9.650004	8.97712104
5161	**ENSG0000012194**	CLCC1	protein_coding	chr1	108881885	108963527	-	Human GRCh	OTTHUMG000000	CLCC1	**chloride channel CLIC like 1**	Cytoplasm	ion channel		-1.03883427	0	0.85193702	0	1	0	11.1690696	11.4534659	11.4314235	11.1007033
5162	**ENSG0000012195**	GPSM2	protein_coding	chr1	108875350	108934545	+	Human GRCh	OTTHUMG000000	GPSM2	**G protein signaling modulator 2**	Nucleus	other		1.032328474	0	0.87622259	0	1	0	10.5382023	10.8348645	10.8652621	10.5775351
5163	**ENSG0000012196**	GTDC1	protein_coding	chr2	143938068	144332568	-	Human GRCh	OTTHUMG000001	GTDC1	**glycosyltransferase like domain containing 1**	Other	other		-1.050339993	0	0.85683616	0	1	0	8.42380471	8.9216263	8.87605812	8.35912565
5164	**ENSG0000012196**	CXCR4	protein_coding	chr2	136114349	136119177	-	Human GRCh	OTTHUMG000001	CXCR4	**C-X-C motif chemokine receptor 4**	Plasma Mem	G-protein cou	USL311, PF	1.002883581	0	0.99612659	0	1	0	3.77957455	3.92281846	4.13806161	3.49438687
5165	**ENSG0000012198**	ZRANB3	protein_coding	chr2	135136916	135531218	-	Human GRCh	OTTHUMG000001	ZRANB3	**zinc finger RANBP2-type containing 3**	Nucleus	enzyme		1.020863827	0	0.93590363	0	1	0	8.21701065	8.27893579	8.46120005	8.0434673
5166	**ENSG0000012198**	ACVR2A	protein_coding	chr2	147844517	147930826	+	Human GRCh	OTTHUMG000001	ACVR2A	**activin A receptor type 2A**	Plasma Mem	kinase	bimagrum	-1.210730676	0	0.39356574	0	1	0	9.06894465	9.33101043	9.12214823	8.85979999
5167	**ENSG0000012200**	POLK	protein_coding	chr5	75511845	75609991	+	Human GRCh	OTTHUMG000001	POLK	**DNA polymerase kappa**	Nucleus	enzyme		1.043074116	0	0.86244147	0	1	0	9.67906257	9.81900597	10.0131198	9.53111569
5168	**ENSG0000012201**	SV2C	protein_coding	chr5	76083383	76353939	+	Human GRCh	OTTHUMG000001	SV2C	**synaptic vesicle glycoprotein 2C**	Plasma Mem	other		-1.963143428	-1	0.58984392	0	1	0	3.07050296	1.55021446	1.53604503	2.54939647
5170	**ENSG0000012202**	RPL21	protein_coding	chr13	27251362	27256691	+	Human GRCh	OTTHUMG000000	RPL21	**ribosomal protein L21**	Cytoplasm	other		-1.048210163	0	0.82607628	0	1	0	12.3077674	12.6364162	12.5653372	12.2774844
5171	**ENSG0000012203**	MTIF3	protein_coding	chr13	27435643	27450591	-	Human GRCh	OTTHUMG000000	MTIF3	**mitochondrial translational initiation factor 3**	Cytoplasm	translation re		-1.230276732	0	0.32325079	0	1	0	8.53982542	8.90246687	8.59186469	8.41567334
5172	**ENSG0000012203**	GTF3A	protein_coding	chr13	27424619	27435823	+	Human GRCh	OTTHUMG000000	GTF3A	**general transcription factor IIIA**	Nucleus	transcription		-1.072054103	0	0.72830834	0	1	0	10.8291011	11.1779894	11.0125373	10.8567174
5173	**ENSG0000012203**	RASL11A	protein_coding	chr13	27270830	27275192	+	Human GRCh	OTTHUMG000000	RASL11A	**RAS like family 11 member A**	Nucleus	other		1.848806383	1	0.2455953	0	1	0	3.76990594	4.5709998	4.7746684	4.74067778
5174	**ENSG0000012204**	UBL3	protein_coding	chr13	29764371	29850617	-	Human GRCh	OTTHUMG000000	UBL3	**ubiquitin like 3**	Cytoplasm	other		1.038784854	0	0.86642811	0	1	0	9.62916735	9.79231681	9.87859465	9.61884053
5176	**ENSG0000012206**	FYTTD1	protein_coding	chr3	197737179	197787596	+	Human GRCh	OTTHUMG000001	FYTTD1	**forty-two-three domain containing 1**	Nucleus	other		1.032929786	0	0.8854896	0	1	0	11.0598523	11.427446	11.5173194	11.017946
5177	**ENSG0000012208**	MTERF4	protein_coding	chr2	241072169	241102332	-	Human GRCh	OTTHUMG000001	MTERF4	**mitochondrial transcription termination factor 4**	Cytoplasm	other		-1.02016986	0	0.92905366	0	1	0	10.0635662	10.186474	10.2142175	9.98371303
5178	**ENSG0000012212**	XPNPEP2	protein_coding	chrX	129738949	129769536	+	Human GRCh	OTTHUMG000000	XPNPEP2	**X-prolyl aminopeptidase 2**	Plasma Mem	peptidase		1	0	1	0	NA	0	-0.1940771	-0.1834848	-0.1852391	-0.1934573
5179	**ENSG0000012212**	SASH3	protein_coding	chrX	129779949	129795201	+	Human GRCh	OTTHUMG000000	SASH3	**SAM and SH3 domain containing 3**	Cytoplasm	other		1	0	1	0	NA	0	0.68634265	0.69978815	0.69754318	0.68712218
5180	**ENSG0000012212**	OCRL	protein_coding	chrX	129539849	129592561	+	Human GRCh	OTTHUMG000000	OCRL	**OCRL inositol polyphosphate-5-phosphatase**	Cytoplasm	phosphatase		-1.109813672	0	0.64347829	0	1	0	10.6908568	10.8795164	10.796001	10.5416967
5181	**ENSG0000012213**	PAEP	protein_coding	chr9	135561756	135566955	+	Human GRCh	OTTHUMG000000	PAEP	**progestagen associated endometrial protein**	Cytoplasm	other		5.664659277	1	0.32383272	0	NA	0	0.77402668	0.79959581	1.35789122	0.77553305
5183	**ENSG0000012214**	MRPS2	protein_coding	chr9	135499984	135504673	+	Human GRCh	OTTHUMG000000	MRPS2	**mitochondrial ribosomal protein S2**	Cytoplasm	other		-1.053697583	0	0.80082859	0	1	0	10.7819695	10.9740988	10.9112334	10.7260015
5189	**ENSG0000012220**	KIAA1191	protein_coding	chr5	176346062	176361807	-	Human GRCh	OTTHUMG000001	KIAA1191	**KIAA1191**	Cytoplasm	other		-1.140111512	0	0.54611035	0	1	0	11.3176178	11.5553381	11.3834472	11.200178
5190	**ENSG0000012221**	COPA	protein_coding	chr1	160288594	160343566	-	Human GRCh	OTTHUMG000000	COPA	**COPI coat complex subunit alpha**	Cytoplasm	transporter		-1.119250097	0	0.59943108	0	1	0	13.3373166	13.6148931	13.4837373	13.2174946
5193	**ENSG0000012225**	HS3ST2	protein_coding	chr16	22814162	22916338	+	Human GRCh	OTTHUMG000000	HS3ST2	**heparan sulfate-glucosamine 3-sulfotransferase 2**	Cytoplasm	enzyme		-9.957403795	-1	0.4412412	0	NA	0	1.40001542	0.60171289	0.59799759	0.580465
5194	**ENSG0000012225**	RBBP6	protein_coding	chr16	24537693	24572863	+	Human GRCh	OTTHUMG000000	RBBP6	**RB binding protein 6, ubiquitin ligase**	Nucleus	enzyme		-1.163791714	0	0.39585688	0	1	0	11.8026271	12.101497	11.8520311	11.7303856

5195	ENSG0000012229	ZC3H7A	protein_coding	chr16	11750586	11797258	-	Human GRCh	OTTHUMG000001	ZC3H7A	zinc finger CCCH-type containing 7A	Extracellular	other		-1.171008996	0	0.41516491	0	1	0	10.9994217	11.1758155	11.0985001	10.701744
5197	ENSG0000012233	SERAC1	protein_coding	chr6	158109519	158168280	-	Human GRCh	OTTHUMG000000	SERAC1	serine active site containing 1	Extracellular	other		-1.135546785	0	0.54068375	0	1	0	8.93184219	9.10852695	9.00961149	8.74539479
5198	ENSG0000012235	ANXA11	protein_coding	chr10	80150889	80205572	-	Human GRCh	OTTHUMG000000	ANXA11	annexin A11	Nucleus	other		1.005059759	0	0.9818001	0	1	0	9.81514224	10.0687381	10.0190937	9.88524296
5199	ENSG0000012236	LDB3	protein_coding	chr10	86666785	86736072	+	Human GRCh	OTTHUMG000000	LDB3	LIM domain binding 3	Cytoplasm	transporter		-1.059131304	0	0.92646176	0	1	0	4.35699254	4.86276609	4.25817927	4.83417744
5201	ENSG0000012237	SHLD2	protein_coding	chr10	87094161	87191465	+	Human GRCh	OTTHUMG000000	SHLD2	shieldin complex subunit 2	Extracellular	other		-1.181789382	0	0.46725323	0	1	0	10.4094907	10.7593543	10.5608418	10.2225656
5202	ENSG0000012237	PRXL2A	protein_coding	chr10	80407829	80437115	+	Human GRCh	OTTHUMG000000	PRXL2A	peroxiredoxin like 2A	Extracellular	other		-1.051625667	0	0.80131212	0	1	0	10.7903453	11.1121411	11.0040465	10.8007061
5203	ENSG0000012238	ZNF205	protein_coding	chr16	3112560	3120517	+	Human GRCh	OTTHUMG000001	ZNF205	zinc finger protein 205	Nucleus	transcription		1.130951039	0	0.56065747	0	1	0	8.8991594	9.05015001	9.16317397	9.06087639
5204	ENSG0000012239	NAA60	protein_coding	chr16	3443649	3486953	+	Human GRCh	OTTHUMG000001	NAA60	N-alpha-acetyltransferase 60, NatF catalytic subunit	Cytoplasm	enzyme		-1.20597899	0	0.37072803	0	1	0	10.6347094	11.0800378	10.7505704	10.5792606
5205	ENSG0000012240	RPL5	protein_coding	chr1	92832013	92841924	+	Human GRCh	OTTHUMG000000	RPL5	ribosomal protein L5	Cytoplasm	other		-1.019471148	0	0.92391019	0	1	0	13.3744224	13.6723769	13.6605404	13.3395649
5206	ENSG0000012241	ODF2L	protein_coding	chr1	86346824	86396342	-	Human GRCh	OTTHUMG000000	ODF2L	outer dense fiber of sperm tails 2 like	Cytoplasm	other		-1.229213827	0	0.35803857	0	1	0	10.1173139	10.2718277	10.1743102	9.72825336
5207	ENSG0000012242	PTGFR	protein_coding	chr1	78303884	78540701	+	Human GRCh	OTTHUMG000000	PTGFR	prostaglandin F receptor	Plasma Mem	G-protein co	timolol/tra	-2.105790968	-1	0.37406537	0	1	0	4.25814332	4.9691155	3.74184022	4.03291349
5208	ENSG0000012243	SPATA1	protein_coding	chr1	84506300	84567379	+	Human GRCh		SPATA1	spermatogenesis associated 1	Cytoplasm	other		1.125405583	0	0.85008943	0	1	0	3.79338808	3.73497575	4.06954813	3.59112935
5209	ENSG0000012243	TRMT13	protein_coding	chr1	100133150	100150496	+	Human GRCh	OTTHUMG000000	TRMT13	tRNA methyltransferase 13 homolog	Cytoplasm	other		1.201863236	0	0.43863782	0	1	0	8.87228493	8.97606621	9.35812023	8.83170456
5210	ENSG0000012247	LRRC39	protein_coding	chr1	100148448	100178267	-	Human GRCh	OTTHUMG000000	LRRC39	leucine rich repeat containing 39	Other	other		-1.072288439	0	0.87338837	0	1	0	5.37237054	5.72661753	5.58044306	5.40474347
5211	ENSG0000012248	RWDD3	protein_coding	chr1	95234210	95247225	+	Human GRCh	OTTHUMG000000	RWDD3	RWD domain containing 3	Other	other		1.158869383	0	0.58597556	0	1	0	7.98141741	8.01788069	8.36652432	7.90566574
5212	ENSG0000012248	ZNF644	protein_coding	chr1	90915298	91022272	-	Human GRCh	OTTHUMG000000	ZNF644	zinc finger protein 644	Nucleus	transcription		-1.080325212	0	0.713788	0	1	0	10.8263258	11.0308396	10.9951844	10.6823636
5213	ENSG0000012248	CCDC18	protein_coding	chr1	93179919	93278730	+	Human GRCh	OTTHUMG000000	CCDC18	coiled-coil domain containing 18	Cytoplasm	other		-1.187238278	0	0.3757982	0	1	0	10.4448495	10.7102513	10.5048004	10.2745691
5214	ENSG0000012248	RPAP2	protein_coding	chr1	92299059	92402056	+	Human GRCh	OTTHUMG000000	RPAP2	RNA polymerase II associated protein 2	Nucleus	enzyme		-1.119306363	0	0.59733064	0	1	0	10.5558546	10.8080671	10.7692173	10.3145836
5215	ENSG0000012249	SLC66A2	protein_coding	chr18	79902420	79951657	-	Human GRCh	OTTHUMG000001	SLC66A2	solute carrier family 66 member 2	Other	other		-1.007635272	0	0.97035247	0	1	0	9.76844302	9.80611704	9.79273456	9.76505
5216	ENSG0000012250	BBS9	protein_coding	chr7	33109557	33877180	+	Human GRCh	OTTHUMG000001	BBS9	Bardet-Biedl syndrome 9	Extracellular	other		-1.222827619	0	0.41715637	0	1	0	9.12240329	9.37554036	9.17621147	8.88154251
5217	ENSG0000012251	PMS2	protein_coding	chr7	5970925	6009130	-	Human GRCh	OTTHUMG000000	PMS2	PMS1 homolog 2, mismatch repair system component	Nucleus	enzyme		-1.134560337	0	0.54993754	0	1	0	9.9595997	10.267071	10.1685795	9.77057326
5218	ENSG0000012251	ZMIZ2	protein_coding	chr7	44748581	44769881	+	Human GRCh	OTTHUMG000001	ZMIZ2	zinc finger MIZ-type containing 2	Nucleus	transcription		-1.028026863	0	0.89707347	0	1	0	12.5741088	12.8615066	12.8582016	12.5084577
5220	ENSG0000012254	SEPTIN7	protein_coding	chr7	35800932	35907110	+	Human GRCh	OTTHUMG000001	SEPTIN7	septin 7	Cytoplasm	other		1.043553719	0	0.83900875	0	1	0	11.4208346	11.7491832	11.8009873	11.4584764
5221	ENSG0000012254	EEPD1	protein_coding	chr7	36153254	36301538	+	Human GRCh	OTTHUMG000001	EEPD1	endonuclease/exonuclease/phosphatase family domain conta	Plasma Mem	other		-1.02348868	0	0.93626443	0	1	0	8.70960155	9.04156671	8.83674428	8.88585312
5223	ENSG0000012255	KLHL7	protein_coding	chr7	23105758	23177914	+	Human GRCh	OTTHUMG000000	KLHL7	kelch like family member 7	Nucleus	other		1.041988987	0	0.84616726	0	1	0	8.96933109	9.29324747	9.32038466	9.03509775
5224	ENSG0000012255	HERPUD2	protein_coding	chr7	35632659	35697459	-	Human GRCh	OTTHUMG000001	HERPUD2	HERPUD family member 2	Other	other		-1.131123034	0	0.53753641	0	1	0	9.63771434	9.89757897	9.82791877	9.41844972
5225	ENSG0000012256	CBX3	protein_coding	chr7	26201162	26213607	+	Human GRCh	OTTHUMG000000	CBX3	chromobox 3	Nucleus	transcription		1.013335892	0	0.94841527	0	1	0	12.0280226	12.2964278	12.38346	11.94465
5226	ENSG0000012256	HNRNPA2I	protein_coding	chr7	26171151	26201529	-	Human GRCh	OTTHUMG000000	HNRNPA2B1	heterogeneous nuclear ribonucleoprotein A2/B1	Nucleus	other		1.132028901	0	0.5041035	0	1	0	14.0871703	14.3436655	14.4814243	14.2205739
5227	ENSG0000012257	WIPF3	protein_coding	chr7	29806486	29917061	+	Human GRCh	OTTHUMG000001	WIPF3	WAS/WASL interacting protein family member 3	Plasma Mem	other		1.492730795	0	0.36111503	0	1	0	5.48089415	5.7753089	5.89784502	6.12728738
5228	ENSG0000012258	NXPH1	protein_coding	chr7	8433609	8752961	+	Human GRCh	OTTHUMG000001	NXPH1	neurexophilin 1	Extracellular	other		1.28723683	0	0.79392796	0	1	0	2.76318293	1.77174677	2.88084005	2.00418597
5229	ENSG0000012258	NPY	protein_coding	chr7	24284188	24291862	+	Human GRCh	OTTHUMG000000	NPY	neuropeptide Y	Plasma Mem	other		1	0	1	0	1	0	0.99407924	1.06694746	1.05524734	0.99849929
5230	ENSG0000012259	HYCC1	protein_coding	chr7	22889371	23014130	-	Human GRCh	OTTHUMG000001	HYCC1	hyccin PI4KA lipid kinase complex subunit 1	Cytoplasm	other		1.023852953	0	0.91672893	0	1	0	9.92773328	10.2486428	10.3248798	9.88139048
5231	ENSG0000012259	HOXA7	protein_coding	chr7	27153716	27157936	-	Human GRCh	OTTHUMG000000	HOXA7	homeobox A7	Nucleus	transcription		1.351553732	0	0.3139246	0	1	0	7.08309135	7.79074256	7.87135817	7.77594129
5232	ENSG0000012264	INHBA	protein_coding	chr7	41667168	41705834	-	Human GRCh	OTTHUMG000000	INHBA	inhibin subunit beta A	Extracellular	growth facto	activin typ	-7.139683017	-1	0.43462195	0	1	0	3.23211477	4.03467055	0.8405553	2.42902447
5233	ENSG0000012264	FKBP9	protein_coding	chr7	32957404	33006930	+	Human GRCh	OTTHUMG000000	FKBP9	FKBP prolyl isomerase 9	Cytoplasm	enzyme		-1.059937475	0	0.82026843	0	1	0	8.3615548	8.7498826	8.50927929	8.51919718
5234	ENSG0000012264	NT5C3A	protein_coding	chr7	33014130	33062796	-	Human GRCh	OTTHUMG000001	NT5C3A	5'-nucleotidase, cytosolic IIIA	Cytoplasm	phosphatase		-1.016470161	0	0.94191632	0	1	0	9.44070186	9.66885099	9.75565899	9.28261139
5235	ENSG0000012264	ARL4A	protein_coding	chr7	12686856	12690958	+	Human GRCh	OTTHUMG000000	ARL4A	ADP ribosylation factor like GTPase 4A	Nucleus	enzyme		-1.176455088	0	0.52810193	0	1	0	8.28065515	8.58809278	8.35392796	8.19430174
5236	ENSG0000012267	CCZ1	protein_coding	chr7	5898725	5926550	+	Human GRCh	OTTHUMG000001	CCZ1/CCZ1B	CCZ1 homolog, vacuolar protein trafficking and biogenesis ass	Cytoplasm	other		-1.078186886	0	0.73033765	0	1	0	8.74654475	8.98617967	8.85940044	8.71740132
5237	ENSG0000012267	POLM	protein_coding	chr7	44072062	44082530	-	Human GRCh	OTTHUMG000001	POLM	DNA polymerase mu	Nucleus	enzyme		1.069120054	0	0.7638456	0	1	0	10.0265605	10.217361	10.3576362	10.0164448
5239	ENSG0000012268	MRM2	protein_coding	chr7	2234195	2242205	-	Human GRCh	OTTHUMG000000	MRM2	mitochondrial rRNA methyltransferase 2	Nucleus	enzyme		1.048477321	0	0.81979152	0	1	0	9.67534985	9.90347193	9.97835087	9.69895172
5240	ENSG0000012269	TWIST1	protein_coding	chr7	19020991	19117636	-	Human GRCh	OTTHUMG000000	TWIST1	twist family bHLH transcription factor 1	Nucleus	transcription		-1.248797823	0	0.64755984	0	1	0	6.8775071	6.63889315	6.69403192	6.33311215
5241	ENSG0000012269	SMU1	protein_coding	chr9	33041765	33076674	-	Human GRCh	OTTHUMG000000	SMU1	SMU1 DNA replication regulator and spliceosomal factor	Nucleus	other		-1.094824638	0	0.65060355	0	1	0	11.506661	11.8351674	11.6627512	11.4932654
5242	ENSG0000012269	GLIPR2	protein_coding	chr9	36136536	36163913	+	Human GRCh	OTTHUMG000000	GLIPR2	GLI pathogenesis related 2	Cytoplasm	other		1.973956217	1	0.07257724	0	1	0	5.3115476	5.60798118	6.1002452	6.20078034
5243	ENSG0000012269	SLC25A51	protein_coding	chr9	37879400	37904353	-	Human GRCh	OTTHUMG000000	SLC25A51	solute carrier family 25 member 51	Cytoplasm	transporter		-1.031148562	0	0.88321199	0	1	0	8.89041112	9.13066405	9.11570941	8.83336595
5244	ENSG0000012270	CLTA	protein_coding	chr9	36190856	36304781	+	Human GRCh	OTTHUMG000000	CLTA	clathrin light chain A	Plasma Mem	other		-1.139914375	0	0.50772219	0	1	0	10.9975187	11.2904085	11.1169782	10.886703
5245	ENSG0000012270	RECK	protein_coding	chr9	36036913	36124455	+	Human GRCh	OTTHUMG000000	RECK	reversion inducing cysteine rich protein with kazal motifs	Plasma Mem	other		-1.30761783	0	0.31773638	0	1	0	7.20878112	7.30660174	7.0903298	6.8531841
5247	ENSG0000012272	TAF1L	protein_coding	chr9	32629454	32635669	-	Human GRCh	OTTHUMG000000	TAF1L	TATA-box binding protein associated factor 1 like	Nucleus	transcription		-1.509468904	-1	0.52096071	0	1	0	4.65323942	4.59542784	4.0739492	4.42168332
5248	ENSG0000012272	ACO1	protein_coding	chr9	32384603	32454769	+	Human GRCh	OTTHUMG000000	ACO1	aconitase 1	Cytoplasm	enzyme		-1.088643415	0	0.75345219	0	1	0	9.53673997	10.0836409	9.84914644	9.63611633
5249	ENSG0000012273	PHF24	protein_coding	chr9	34957608	34982544	+	Human GRCh	OTTHUMG000000	PHF24	PHD finger protein 24	Other	other		-11.26861721	-1	0.13817463	0	NA	0	1.36422824	2.51290002	1.37393224	1.5414999
5250	ENSG0000012273	DNAI1	protein_coding	chr9	34457414	34520988	+	Human GRCh	OTTHUMG000000	DNAI1	dynein axonemal intermediate chain 1	Extracellular	other		-1.007655169	0	0.98756947	0	1	0	4.51671236	4.36552541	4.33988528	4.52734691
5251	ENSG0000012274	DCAF10	protein_coding	chr9	37800554	37867666	+	Human GRCh	OTTHUMG000000	DCAF10	DDB1 and CUL4 associated factor 10	Nucleus	other		-1.019653892	0	0.93268264	0	1	0	10.6045663	10.8311543	10.923567	10.4285089
5252	ENSG0000012275	CNTFR	protein_coding	chr9	34551432	34590140	-	Human GRCh	OTTHUMG000000	CNTFR	ciliary neurotrophic factor receptor	Plasma Mem	transmembra		-1.721517088	-1	0.09380032	0	1	0	6.90051447	6.47712113	5.96976988	6.38580582
5253	ENSG0000012277	KIAA1549	protein_coding	chr7	138831381	138981389	-	Human GRCh	OTTHUMG000001	KIAA1549	KIAA1549	Plasma Mem	other		-1.155497304	0	0.58074149	0	1	0	9.69925406	10.0578252	9.84241883	9.6175676
5254	ENSG0000012277	TRIM24	protein_coding	chr7	138460259	138589996	+	Human GRCh	OTTHUMG000001	TRIM24	tripartite motif containing 24	Nucleus	transcription		-1.237443663	0	0.34761377	0	1	0	11.6825649	12.0411365	11.7594979	11.4967002
5255	ENSG0000012278	CYREN	protein_coding	chr7	135092363	135170795	-	Human GRCh	OTTHUMG000001	CYREN	cell cycle regulator of NHEJ	Nucleus	other		-1.085031276	0	0.69541133	0	1	0	9.67961962	9.98599486	9.94255418	9.52630373
5256	ENSG0000012278	CALD1	protein_coding	chr7	134744252	134970729	+	Human GRCh	OTTHUMG000001	CALD1	caldesmon 1	Cytoplasm	other		-1.204727884	0	0.48621468	0	1	0	9.13984111	9.4603625	9.17988988	8.99441277
5257	ENSG0000012278	AKR1D1	protein_coding	chr7	138002324	138118305	+	Human GRCh	OTTHUMG000001	AKR1D1	aldo-keto reductase family 1 member D1	Cytoplasm	enzyme	azelaic aci	-1.382484196	0	0.61862614	0	1	0	6.54496541	6.08181375	6.13272142	5.77630739
5258	ENSG0000012282	NUDT10	protein_coding	chrX	51332231	51337525	+	Human GRCh	OTTHUMG000000	NUDT10	nudix hydrolase 10	Cytoplasm	enzyme		-1.355046979	0	0.52206401	0	1	0	10.0975207	10.3977647	10.0021666	9.75329983
5259	ENSG0000012285	SFTPA1	protein_coding	chr10	79610939	79615455	+	Human GRCh	OTTHUMG000000	SFTPA1	surfactant protein A1	Extracellular	transporter		1	0	1	0	1	0	0.76962337	0.871275	0.85516053	0.7758811
5260	ENSG0000012285	NEUROG3	protein_coding	chr10	69571698	69573422	-	Human GRCh	OTTHUMG000000	NEUROG3	neurogenin 3	Nucleus	transcription		5.383560564	1	0.17503789	0	1	0	3.02138124	3.12102964	3.57427374	3.0273879
5261	ENSG0000012286	PLAU	protein_coding	chr10	73909177	73917496	+	Human GRCh	OTTHUMG000000	PLAU	plasminogen activator, urokinase	Extracellular	peptidase		1	0	1	0	NA	0	1.41040178	1.4358447	1.43162391	1.41188798
5263	ENSG0000012286	CHST3	protein_coding	chr10	71964395	72013558	+	Human GRCh	OTTHUMG000000	CHST3	carbohydrate sulfotransferase 3	Cytoplasm	enzyme		1.553505007	1	0.11768541	0	1	0	7.5405841	7.67066345	7.76672612	8.33788279
5264	ENSG0000012287	BICC1	protein_coding	chr10	58512872	58831435	+	Human GRCh	OTTHUMG000000	BICC1	BicC family RNA binding protein 1	Other	other		-1.168002957	0	0.71481636	0	1	0	7.26035252	7.50618884	6.71584017	7.57797981
5265	ENSG0000012287	CISD1	protein_coding	chr10	58269162	58289586	+	Human GRCh	OTTHUMG000000	CISD1	CDGSH iron sulfur domain 1	Cytoplasm	enzyme		1.081905449	0	0.73363423	0	1	0	8.99098055	9.35009682	9.42976417	9.08672661
5266	ENSG0000012287	EGR2	protein_coding	chr10	62811996	62819167	-	Human GRCh	OTTHUMG000000	EGR2	early growth response 2	Nucleus	transcription		-1.081185643	0	0.9202046	0	NA	0	3.55335084	2.72293466	3.34596924	2.98042086
5267	ENSG0000012288	ECD	protein_coding	chr10	73130155	73169055	-	Human GRCh	OTTHUMG000000	ECD	ecdysoneless cell cycle regulator	Nucleus	transcription		-1.116946694	0	0.60215652	0	1	0	10.1310161	10.4063592	10.2315869	10.0742818
5268	ENSG0000012288	P4HA1	protein_coding	chr10	73007217	73096974	-	Human GRCh	OTTHUMG000000	P4HA1	prolyl 4-hydroxylase subunit alpha 1	Cytoplasm	enzyme		-1.001564744	0	0.99629529	0	1	0	11.2680884	11.533729	11.6019052	11.1727179
5269	ENSG0000012291	SLC25A16	protein_coding	chr10	68477998	68527523	-	Human GRCh	OTTHUMG000000	SLC25A16	solute carrier family 25 member 16	Cytoplasm	transporter		1.050163514	0	0.82670298	0	1	0	9.58331414	9.96480064	10.0047802	9.65432051
5270	ENSG0000012295	ZWINT	protein_coding	chr10	56357227	56361273	-	Human GRCh	OTTHUMG000000	ZWINT	ZW10 interacting kinetochore protein	Nucleus	other		-1.120940939	0	0.57209523	0	1	0	11.1422003	11.5527359	11.2783692	11.2006194
5271	ENSG0000012295	VPS26A	protein_coding	chr10	69123512	69174412	+	Human GRCh	OTTHUMG000000	VPS26A	VPS26 retromer complex component A	Cytoplasm	transporter		1.096881837	0	0.67047149	0	1	0	9.98077172	10.3129572	10.4469623	10.0367665
5272	ENSG0000012296	RBM19	protein_coding	chr12	113816738	113966325	-	Human GRCh	OTTHUMG000001	RBM19	RNA binding motif protein 19	Nucleus	other		1.024584602	0	0.91419598	0	1	0	10.3729966	10.8123803	10.7636941	10.502136
5273	ENSG0000012296	CIT	protein_coding	chr12	119685791	119877320	-	Human GRCh	OTTHUMG000001	CIT	citron rho-interacting serine/threonine kinase	Cytoplasm	kinase		-1.341641364	0	0.13552764	0	1	0	12.1076853	12.4282259	12.1231578	11.7775203
5274	ENSG0000012297	IFT81	protein_coding	chr12	110124335	110218793	+	Human GRCh	OTTHUMG000001	IFT81	intraflagellar transport 81	Extracellular	other		1.004425282	0	0.98299874	0	1	0	9.09571445	9.0548243	9.28302482	8.83588671
5275	ENSG0000012297	ACADS	protein_coding	chr12	120725774	120740008	+	Human GRCh	OTTHUMG000001	ACADS	acyl-CoA dehydrogenase short chain	Cytoplasm	enzyme		1.338026928	0	0.29382007	0	1	0	8.07327535	8.49246732	8.81087006	8.38605947
5276	ENSG0000012298	HVCN1	protein_coding	chr12	110627841	110704950	-	Human GRCh	OTTHUMG000001	HVCN1	hydrogen voltage gated channel 1	Plasma Mem	ion channel		-3.241339814	-1	0.35168156	0	NA	0	2.04075326	2.41583867	1.67093794	2.04537288
5277	ENSG0000012306	DDX54	protein_coding	chr12	113157173	113185479	-	Human GRCh	OTTHUMG000001	DDX54	DEAD-box helicase 54	Nucleus	transcription		1.012809117	0	0.94604068	0	1	0	11.6230688	11.9790944	11.9441322	11.7008815
5278	ENSG0000012306	MED13L	protein_coding	chr12	115957905	116277693	-	Human GRCh	OTTHUMG000001	MED13L	mediator complex subunit 13L	Nucleus	other		-1.175666731	0	0.44808818	0	1	0	12.7818967	13.1074675	12.8628393	12.6850733
5279	ENSG0000012308	CDKN2C	protein_coding	chr1	50960745	50974634	+	Human GRCh	OTTHUMG000000	CDKN2C	cyclin dependent kinase inhibitor 2C	Nucleus	transcription		-1.043090285	0	0.85157419	0	1	0	9.56528804	9.9843474	9.76495415	9.73584005
5280	ENSG0000012309	RNF11	protein_coding	chr1	51236273	51273447	+	Human GRCh	OTTHUMG000000	RNF11	ring finger protein 11	Cytoplasm	enzyme		1.118832938	0	0.57897326	0	1	0	9.95024069	10.0746489	10.1791275	10.0899517
5281	ENSG0000012309	RASSF8	protein_coding	chr12	25958232	26079892	+	Human GRCh	OTTHUMG000001	RASSF8	Ras association domain family member 8	Extracellular	other		1.393057934	0	0.2921343	0	1	0	8.37125306	8.61901645	9.07028961	8.61232853
5282	ENSG0000012309	BHLHE41	protein_coding	chr12	26120030	26125037	-	Human GRCh	OTTHUMG000001	BHLHE41	basic helix-loop-helix family member e41	Nucleus	transcription		1.143910943	0	0.75477237	0	1	0	5.91539735	6.51141612	6.59112471	6.15383685
5284	ENSG0000012310	ITPR2	protein_coding	chr12	26335352	26833194	-	Human GRCh	OTTHUMG000001	ITPR2	inositol 1,4,5-trisphosphate receptor type 2	Cytoplasm	ion channel		-1.087168564	0	0.7625677	0	1	0	10.9995695	11.3139826	11.4145711	10.5943134
5285	ENSG0000012310	CCDC91	protein_coding	chr12	28133249	28581511	+	Human GRCh	OTTHUMG000001	CCDC91	coiled-coil domain containing 91	Cytoplasm	other		-1.062553184	0	0.78910443	0	1	0	9.65243562	9.9113504	9.85415269	9.57589644

5286	**ENSG0000012311**	NECAB1	protein_coding	chr8	90791741	90959393	+	Human GRCh	OTTHUMG000001	NECAB1	**N-terminal EF-hand calcium binding protein 1**	Cytoplasm	other		1.24828644	0	0.77555867	0	1	0	2.69332287	3.44578773	3.41937365	3.1727906
5287	**ENSG0000012312**	WWP1	protein_coding	chr8	86342547	86478420	+	Human GRCh	OTTHUMG000001	WWP1	**WW domain containing E3 ubiquitin protein ligase 1**	Cytoplasm	enzyme		-1.221530962	0	0.38483244	0	1	0	11.9753621	12.1492546	11.9817314	11.6802081
5288	**ENSG0000012313**	ACOT9	protein_coding	chrX	23701055	23766475	-	Human GRCh	OTTHUMG000000	ACOT9	**acyl-CoA thioesterase 9**	Cytoplasm	enzyme		1.028144068	0	0.92887258	0	1	0	6.77562225	6.89438098	7.13130663	6.51608726
5289	**ENSG0000012313**	PRDX4	protein_coding	chrX	23664262	23686397	+	Human GRCh	OTTHUMG000000	PRDX4	**peroxiredoxin 4**	Cytoplasm	enzyme		-1.070675962	0	0.75036872	0	1	0	10.3758901	10.8303992	10.6969551	10.376832
5290	**ENSG0000012313**	DDX39A	protein_coding	chr19	14408798	14419383	-	Human GRCh	OTTHUMG000001	DDX39A	**DExD-box helicase 39A**	Nucleus	enzyme		-1.064020323	0	0.77724353	0	1	0	11.933763	12.3940642	12.22426	11.9974327
5291	**ENSG0000012314**	PKN1	protein_coding	chr19	14433053	14471867	+	Human GRCh	OTTHUMG000000	PKN1	**protein kinase N1**	Cytoplasm	kinase		1.033092815	0	0.88363206	0	1	0	12.0403574	12.2624111	12.3453172	12.0169135
5292	**ENSG0000012314**	TRIR	protein_coding	chr19	12730640	12734684	-	Human GRCh	OTTHUMG000001	TRIR	**telomerase RNA component interacting RNase**	Nucleus	enzyme		1.036674612	0	0.84421533	0	1	0	11.595823	11.8217993	11.8022218	11.7046724
5293	**ENSG0000012314**	ADGRE5	protein_coding	chr19	14380501	14408725	+	Human GRCh	OTTHUMG000001	ADGRE5	**adhesion G protein-coupled receptor E5**	Plasma Mem	G-protein cou		-1.080347436	0	0.77608624	0	1	0	9.05760599	9.4433105	9.00765752	9.32273666
5294	**ENSG0000012315**	WDR83	protein_coding	chr19	12666802	12675832	+	Human GRCh	OTTHUMG000001	WDR83	**WD repeat domain 83**	Nucleus	other		1.139517555	0	0.60083747	0	1	0	7.82885143	8.21163319	8.29798519	8.0413784
5295	**ENSG0000012315**	GIPC1	protein_coding	chr19	14477760	14496149	-	Human GRCh	OTTHUMG000001	GIPC1	**GIPC PDZ domain containing family member 1**	Cytoplasm	other		1.110888811	0	0.61708162	0	1	0	10.5642926	10.6333818	10.7511949	10.6821466
5296	**ENSG0000012316**	ACTRT1	protein_coding	chrX	128050962	128052403	-	Human GRCh	OTTHUMG000000	ACTRT1	**actin related protein T1**	Cytoplasm	other		1	0	1	0	NA	0	0.50406728	0.51433723	0.51261751	0.50466073
5298	**ENSG0000012317**	SPRYD7	protein_coding	chr13	49912702	49936490	-	Human GRCh	OTTHUMG000000	SPRYD7	**SPRY domain containing 7**	Other	other		1.027300602	0	0.90785926	0	1	0	8.81834093	8.96967557	9.17011094	8.62085702
5299	**ENSG0000012317**	EBPL	protein_coding	chr13	49660674	49691486	-	Human GRCh	OTTHUMG000000	EBPL	**EBP like**	Cytoplasm	enzyme		1.004730326	0	0.98469909	0	1	0	9.88522789	10.0938501	10.0536905	9.94264309
5300	**ENSG0000012319**	ATP7B	protein_coding	chr13	51930436	52012125	-	Human GRCh	OTTHUMG000000	ATP7B	**ATPase copper transporting beta**	Cytoplasm	transporter		-1.359794561	0	0.19080608	0	1	0	11.0818617	11.4196149	11.1365198	10.6333484
5301	**ENSG0000012320**	ZC3H13	protein_coding	chr13	45954465	46052759	-	Human GRCh	OTTHUMG000000	ZC3H13	**zinc finger CCCH-type containing 13**	Extracellular	other		1.065793734	0	0.72556855	0	1	0	11.0879008	11.3241894	11.3533681	11.2061898
5302	**ENSG0000012321**	NLN	protein_coding	chr5	65722205	65871725	+	Human GRCh	OTTHUMG000000	NLN	**neurolysin**	Cytoplasm	peptidase		-1.073644129	0	0.74068596	0	1	0	11.0506364	11.4570904	11.3185854	11.0489878
5303	**ENSG0000012321**	CENPK	protein_coding	chr5	65517766	65563168	-	Human GRCh	OTTHUMG000001	CENPK	**centromere protein K**	Nucleus	other		1.08941891	0	0.71062076	0	1	0	9.42499167	9.63689615	9.75956071	9.48073036
5304	**ENSG0000012324**	OPTN	protein_coding	chr10	13099449	13138308	+	Human GRCh	OTTHUMG000000	OPTN	**optineurin**	Cytoplasm	other		-1.092501836	0	0.77545127	0	1	0	7.1602248	7.26361781	6.8145814	7.36092452
5305	**ENSG0000012324**	ITIH5	protein_coding	chr10	7559270	7666998	-	Human GRCh	OTTHUMG000000	ITIH5	**inter-alpha-trypsin inhibitor heavy chain 5**	Plasma Mem	other		1.356167425	0	0.74028233	0	1	0	6.06028797	6.08847768	7.0757726	4.92279732
5306	**ENSG0000012326**	ATF1	protein_coding	chr12	50763710	50821162	+	Human GRCh	OTTHUMG000001	ATF1	**activating transcription factor 1**	Nucleus	transcription		1.093534929	0	0.72502831	0	1	0	9.1131118	9.36332233	9.49862665	9.16541423
5307	**ENSG0000012329**	TSFM	protein_coding	chr12	57782761	57808071	+	Human GRCh	OTTHUMG000001	TSFM	**Ts translation elongation factor, mitochondrial**	Cytoplasm	translation re		-1.009432515	0	0.96490597	0	1	0	9.59534174	10.000822	9.91913223	9.67816548
5308	**ENSG0000012330**	NEUROD4	protein_coding	chr12	55019974	55030017	+	Human GRCh	OTTHUMG000001	NEUROD4	**neuronal differentiation 4**	Nucleus	transcription		-5.375050575	-1	0.19896649	0	1	0	2.10619583	1.52474902	1.5108186	1.44316544
5309	**ENSG0000012332**	ARHGAP9	protein_coding	chr12	57472264	57488814	-	Human GRCh	OTTHUMG000001	ARHGAP9	**Rho GTPase activating protein 9**	Cytoplasm	other		-1.863832043	-1	0.15014868	0	1	0	4.505752	4.24521344	3.64509392	4.00440937
5312	**ENSG0000012334**	PFDN5	protein_coding	chr12	53295291	53299452	+	Human GRCh	OTTHUMG000001	PFDN5	**prefoldin subunit 5**	Nucleus	transcription		-1.065977888	0	0.76427969	0	1	0	11.0496588	11.4403829	11.277599	11.0977223
5313	**ENSG0000012335**	SPATS2	protein_coding	chr12	49366584	49527425	+	Human GRCh	OTTHUMG000001	SPATS2	**spermatogenesis associated serine rich 2**	Cytoplasm	other		-1.13446069	0	0.60342451	0	1	0	9.3768972	9.6195056	9.39600839	9.34500216
5314	**ENSG0000012335**	ORMDL2	protein_coding	chr12	55818041	55821879	+	Human GRCh	OTTHUMG000001	ORMDL2	**ORMDL sphingolipid biosynthesis regulator 2**	Cytoplasm	other		-1.103079186	0	0.62840321	0	1	0	9.18915076	9.45014409	9.31436843	9.11645041
5315	**ENSG0000012335**	NR4A1	protein_coding	chr12	52022832	52059507	+	Human GRCh	OTTHUMG000001	NR4A1	**nuclear receptor subfamily 4 group A member 1**	Nucleus	ligand-depen		1.164268994	0	0.48408172	0	1	0	10.9798141	11.0761939	11.3412968	11.0390176
5316	**ENSG0000012336**	PDE1B	protein_coding	chr12	54549601	54579239	+	Human GRCh	OTTHUMG000001	PDE1B	**phosphodiesterase 1B**	Cytoplasm	enzyme		1.872418207	1	0.45085146	0	1	0	2.74272152	2.88620156	3.63618202	2.75139532
5317	**ENSG0000012336**	HOXC13	protein_coding	chr12	53938831	53946544	+	Human GRCh	OTTHUMG000001	HOXC13	**homeobox C13**	Nucleus	transcription		1.201759828	0	0.7356738	0	1	0	5.1437515	5.45675397	5.7716645	5.17560339
5318	**ENSG0000012337**	CDK2	protein_coding	chr12	55966781	55972789	+	Human GRCh	OTTHUMG000001	CDK2	**cyclin dependent kinase 2**	Nucleus	kinase	PF-071040	-1.151488368	0	0.51205455	0	1	0	11.2883649	11.5775591	11.4355382	11.1165097
5319	**ENSG0000012338**	LRP1	protein_coding	chr12	57128483	57213361	+	Human GRCh	OTTHUMG000000	LRP1	**LDL receptor related protein 1**	Plasma Mem	transmembra	paclitaxel-a	1.039467945	0	0.89054382	0	1	0	9.83861727	10.0605076	10.0950976	9.88558253
5320	**ENSG0000012338**	HOXC11	protein_coding	chr12	53973126	53977643	+	Human GRCh	OTTHUMG000001	HOXC11	**homeobox C11**	Nucleus	transcription		1.14881252	0	0.92980562	0	NA	0	1.49311619	0.85305652	1.57483164	0.84034657
5321	**ENSG0000012339**	ATG101	protein_coding	chr12	52069246	52077495	+	Human GRCh	OTTHUMG000001	ATG101	**autophagy related 101**	Cytoplasm	other		1.211108822	0	0.37292461	0	1	0	9.48093734	9.76197027	10.0529056	9.58469347
5322	**ENSG0000012340**	NFE2	protein_coding	chr12	54292111	54301015	-	Human GRCh	OTTHUMG000001	NFE2	**nuclear factor, erythroid 2**	Nucleus	transcription		2.111509765	1	0.62183155	0	NA	0	1.53324611	1.23740264	1.58296775	1.53674173
5323	**ENSG0000012340**	HOXC12	protein_coding	chr12	53954903	53958956	+	Human GRCh	OTTHUMG000001	HOXC12	**homeobox C12**	Nucleus	transcription		-5.013827515	-1	0.36092845	0	1	0	4.30032502	3.75083927	1.97559348	2.88835296
5324	**ENSG0000012341**	IKZF4	protein_coding	chr12	56007659	56038435	+	Human GRCh	OTTHUMG000001	IKZF4	**IKAROS family zinc finger 4**	Nucleus	transcription		-1.054855587	0	0.81964473	0	1	0	8.74001392	9.13693819	9.04673175	8.72574332
5325	**ENSG0000012341**	SMUG1	protein_coding	chr12	54121277	54189008	-	Human GRCh	OTTHUMG000001	SMUG1	**single-strand-selective monofunctional uracil-DNA glycosylase**	Nucleus	enzyme		1.06449361	0	0.7680795	0	1	0	9.53094441	9.69720497	9.8436323	9.49916728
5326	**ENSG0000012341**	TUBA1B	protein_coding	chr12	49127782	49131397	-	Human GRCh	OTTHUMG000001	TUBA1B	**tubulin alpha 1b**	Cytoplasm	enzyme		-1.204696298	0	0.423925	0	1	0	14.1606218	14.5335395	14.3152645	13.9670825
5327	**ENSG0000012342**	EEF1AKMT	protein_coding	chr12	57771492	57782541	+	Human GRCh	OTTHUMG000001	EEF1AKMT3	**EEF1A lysine methyltransferase 3**	Cytoplasm	enzyme		-1.093443193	0	0.77664446	0	1	0	7.98938559	8.39931687	8.18561009	8.0381759
5328	**ENSG0000012344**	KBTBD4	protein_coding	chr11	47572197	47578976	-	Human GRCh	OTTHUMG000001	KBTBD4	**kelch repeat and BTB domain containing 4**	Other	other		1.008590996	0	0.97485326	0	1	0	8.34593224	8.57810898	8.66511248	8.25252171
5329	**ENSG0000012345**	SARDH	protein_coding	chr9	133663560	133739955	-	Human GRCh	OTTHUMG000000	SARDH	**sarcosine dehydrogenase**	Cytoplasm	enzyme		-1.967622463	-1	0.22933629	0	1	0	3.90291801	3.68615327	3.65934424	2.91920799
5330	**ENSG0000012345**	DBH	protein_coding	chr9	133636363	133659329	+	Human GRCh	OTTHUMG000000	DBH	**dopamine beta-hydroxylase**	Cytoplasm	enzyme	disulfiram/	1	0	1	0	1	0	1.29505176	1.36303371	1.35205811	1.29914949
5331	**ENSG0000012347**	ATPAF1	protein_coding	chr1	46632737	46673867	-	Human GRCh	OTTHUMG000000	ATPAF1	**ATP synthase mitochondrial F1 complex assembly factor 1**	Cytoplasm	other		-1.129335124	0	0.57683266	0	1	0	11.0349556	11.3456491	11.1596827	10.9593379
5332	**ENSG0000012347**	STIL	protein_coding	chr1	47250139	47314892	-	Human GRCh	OTTHUMG000000	STIL	**STIL centriolar assembly protein**	Nucleus	other		-1.150334079	0	0.51193586	0	1	0	10.963802	11.3361312	11.1168587	10.8942287
5333	**ENSG0000012348**	HJURP	protein_coding	chr2	233833416	233854566	-	Human GRCh	OTTHUMG000000	HJURP	**Holliday junction recognition protein**	Nucleus	other		-1.233460856	0	0.37796582	0	1	0	10.8070441	11.2077978	10.8846657	10.6904512
5335	**ENSG0000012350**	COL10A1	protein_coding	chr6	116118909	116158747	-	Human GRCh	OTTHUMG000000	COL10A1	**collagen type X alpha 1 chain**	Extracellular	other	collagenas	4.981113179	1	0.71833261	0	NA	0	0.15173887	0.165805	0.16348202	0.62026524
5336	**ENSG0000012350**	AMD1	protein_coding	chr6	110874770	110898879	+	Human GRCh	OTTHUMG000000	AMD1	**adenosylmethionine decarboxylase 1**	Cytoplasm	enzyme		-1.011753848	0	0.9519709	0	1	0	12.2521324	12.4630516	12.5370918	12.1257064
5337	**ENSG0000012354**	NDUFAF4	protein_coding	chr6	96889315	96897891	-	Human GRCh	OTTHUMG000000	NDUFAF4	**NADH:ubiquinone oxidoreductase complex assembly factor 4**	Cytoplasm	other		1.003920398	0	0.98666929	0	1	0	9.03311641	9.38757429	9.45380599	8.9485516
5338	**ENSG0000012355**	USP45	protein_coding	chr6	99432325	99521728	-	Human GRCh	OTTHUMG000000	USP45	**ubiquitin specific peptidase 45**	Nucleus	peptidase		-1.112420119	0	0.66226411	0	1	0	9.00990257	9.17265225	9.22739851	8.66859473
5339	**ENSG0000012356**	PLP1	protein_coding	chrX	103773718	103792619	+	Human GRCh	OTTHUMG000000	PLP1	**proteolipid protein 1**	Plasma Mem	other		6.018789186	1	0.33443762	0	1	0	1.02082119	0.07440702	2.28526116	1.59451608
5341	**ENSG0000012356**	MORF4L2	protein_coding	chrX	103675496	103688158	-	Human GRCh	OTTHUMG000000	MORF4L2	**mortality factor 4 like 2**	Nucleus	other		-1.155398405	0	0.46713817	0	1	0	12.0681928	12.3433902	12.1830361	11.9060196
5343	**ENSG0000012357**	RAB9B	protein_coding	chrX	103822327	103832257	-	Human GRCh	OTTHUMG000000	RAB9B	**RAB9B, member RAS oncogene family**	Plasma Mem	enzyme		-1.134346592	0	0.76700626	0	1	0	5.40652339	5.60612247	5.75835609	4.85086608
5345	**ENSG0000012357**	FAM199X	protein_coding	chrX	104166453	104195902	+	Human GRCh	OTTHUMG000000	FAM199X	**family with sequence similarity 199, X-linked**	Other	other		1.014255975	0	0.94727579	0	1	0	10.4980165	10.8379673	10.8820162	10.4741191
5349	**ENSG0000012359**	RAB9A	protein_coding	chrX	13689128	13710504	+	Human GRCh	OTTHUMG000000	RAB9A	**RAB9A, member RAS oncogene family**	Cytoplasm	enzyme		-1.190181432	0	0.47380671	0	1	0	7.92985083	8.17210559	8.01734047	7.70142989
5350	**ENSG0000012360**	METTL8	protein_coding	chr2	171315746	171434802	-	Human GRCh	OTTHUMG000001	METTL8	**methyltransferase 8, tRNA N3-cytidine**	Cytoplasm	enzyme		-1.134587531	0	0.57599559	0	1	0	9.58065416	9.99836008	9.88758693	9.39759028
5351	**ENSG0000012360**	TTC21B	protein_coding	chr2	165857475	165953851	-	Human GRCh	OTTHUMG000001	TTC21B	**tetratricopeptide repeat domain 21B**	Extracellular	other		-1.147513681	0	0.56545628	0	1	0	10.4572514	10.8544284	10.6380538	10.3923511
5352	**ENSG0000012360**	NMI	protein_coding	chr2	151270470	151289894	-	Human GRCh	OTTHUMG000001	NMI	**N-myc and STAT interactor**	Cytoplasm	transcription		-1.498366816	0	0.59163633	0	1	0	3.07458928	2.85238182	2.83149292	2.73024412
5354	**ENSG0000012361**	ACVR1C	protein_coding	chr2	157526767	157628864	-	Human GRCh	OTTHUMG000001	ACVR1C	**activin A receptor type 1C**	Plasma Mem	kinase	SB-505124	1.353194357	0	0.41500955	0	1	0	7.58528751	8.43336935	8.54531241	8.26609315
5355	**ENSG0000012363**	BAZ2B	protein_coding	chr2	159318979	159616569	-	Human GRCh	OTTHUMG000001	BAZ2B	**bromodomain adjacent to zinc finger domain 2B**	Nucleus	other		-1.014426519	0	0.96096834	0	1	0	8.96331849	9.36028369	9.34664918	8.94695756
5356	**ENSG0000012364**	SLC36A1	protein_coding	chr5	151437046	151492379	+	Human GRCh	OTTHUMG000001	SLC36A1	**solute carrier family 36 member 1**	Plasma Mem	transporter		-1.019184713	0	0.9379037	0	1	0	9.36055842	9.62035703	9.54463423	9.40768297
5357	**ENSG0000012368**	LPGAT1	protein_coding	chr1	211743457	211830763	-	Human GRCh	OTTHUMG000000	LPGAT1	**lysophosphatidylglycerol acyltransferase 1**	Cytoplasm	enzyme		-1.027842425	0	0.89920035	0	1	0	11.6729482	11.7611757	11.8220915	11.5388016
5358	**ENSG0000012368**	BATF3	protein_coding	chr1	212686417	212699840	-	Human GRCh	OTTHUMG000000	BATF3	**basic leucine zipper ATF-like transcription factor 3**	Nucleus	transcription		-1.025655207	0	0.96106799	0	1	0	5.18789552	5.20495903	5.30801538	5.0200015
5359	**ENSG0000012368**	G0S2	protein_coding	chr1	209675412	209676390	+	Human GRCh	OTTHUMG000000	G0S2	**G0/G1 switch 2**	Cytoplasm	other		-8.309224975	-1	0.05015622	0	1	0	3.98250564	3.50858421	2.22868798	2.45034199
5360	**ENSG0000012370**	KCNJ2	protein_coding	chr17	70168673	70180044	+	Human GRCh	OTTHUMG000001	KCNJ2	**potassium inwardly rectifying channel subfamily J member 2**	Plasma Mem	ion channel	nicorandil,	-1.330485589	0	0.59663422	0	1	0	5.34455643	5.08959457	4.62671448	5.20905614
5361	**ENSG0000012372**	RAP2C	protein_coding	chrX	132203024	132219480	-	Human GRCh	OTTHUMG000000	RAP2C	**RAP2C, member of RAS oncogene family**	Cytoplasm	enzyme		1.030742903	0	0.89227186	0	1	0	9.08116231	9.15468784	9.32165204	8.95294614
5362	**ENSG0000012373**	EXOSC9	protein_coding	chr4	121801318	121817021	+	Human GRCh	OTTHUMG000001	EXOSC9	**exosome component 9**	Nucleus	enzyme		-1.05339099	0	0.79654234	0	1	0	9.85906571	10.2271422	10.080799	9.91246473
5363	**ENSG0000012373**	PLA2G12A	protein_coding	chr4	109709989	109730070	-	Human GRCh	OTTHUMG000001	PLA2G12A	**phospholipase A2 group XIIA**	Extracellular	enzyme		1.068118136	0	0.78000761	0	1	0	10.8692683	11.0968808	11.2949078	10.7748861
5364	**ENSG0000012381**	B9D2	protein_coding	chr19	41354417	41364165	-	Human GRCh	OTTHUMG000001	B9D2	**B9 domain containing 2**	Cytoplasm	other		-1.389142905	0	0.34474313	0	1	0	6.59536668	6.53455269	6.52478327	5.85011558
5365	**ENSG0000012381**	COQ8B	protein_coding	chr19	40691514	40725784	-	Human GRCh	OTTHUMG000001	COQ8B	**coenzyme Q8B**	Cytoplasm	kinase		-1.082209269	0	0.72774306	0	1	0	10.2106635	10.5918768	10.4008749	10.2507696
5366	**ENSG0000012383**	PFKFB2	protein_coding	chr1	207034366	207081024	+	Human GRCh	OTTHUMG000000	PFKFB2	**6-phosphofructo-2-kinase/fructose-2,6-biphosphatase 2**	Cytoplasm	enzyme		1.022718722	0	0.92244341	0	1	0	11.2849094	11.7103809	11.7725549	11.2537144
5369	**ENSG0000012389**	RAB38	protein_coding	chr11	88113251	88175443	-	Human GRCh	OTTHUMG000001	RAB38	**RAB38, member RAS oncogene family**	Cytoplasm	enzyme		5.203784204	1	0.26564765	0	1	0	1.73534956	1.79534157	1.7855779	2.28569276
5370	**ENSG0000012390**	GPR83	protein_coding	chr11	94377316	94401419	-	Human GRCh	OTTHUMG000001	GPR83	**G protein-coupled receptor 83**	Plasma Mem	G-protein cou		-1.665013808	-1	0.32010092	0	1	0	4.85386292	4.97903133	4.546223	4.37098574
5371	**ENSG0000012390**	AGO2	protein_coding	chr8	140520156	140635633	-	Human GRCh	OTTHUMG000001	AGO2	**argonaute RISC catalytic component 2**	Cytoplasm	translation re		-1.04809532	0	0.81551012	0	1	0	13.4165928	13.7013377	13.6672603	13.3417773
5372	**ENSG0000012393**	MXD4	protein_coding	chr4	2247432	2262109	-	Human GRCh	OTTHUMG000000	MXD4	**MAX dimerization protein 4**	Nucleus	transcription		-1.085391608	0	0.70895429	0	1	0	11.0272385	11.2155618	11.1258517	10.9333422
5373	**ENSG0000012397**	CKS2	protein_coding	chr9	89311195	89316703	+	Human GRCh	OTTHUMG000000	CKS2	**CDC28 protein kinase regulatory subunit 2**	Other	kinase		1.186962184	0	0.51149373	0	1	0	9.3592666	9.88935016	9.9704914	9.7019899
5375	**ENSG0000012398**	ACSL3	protein_coding	chr2	222860942	222944639	+	Human GRCh	OTTHUMG000001	ACSL3	**acyl-CoA synthetase long chain family member 3**	Cytoplasm	enzyme		-1.298154484	0	0.33886306	0	1	0	12.2060745	12.5265744	12.2599125	11.8600907
5376	**ENSG0000012398**	CHPF	protein_coding	chr2	219538954	219543809	-	Human GRCh	OTTHUMG000000	CHPF	**chondroitin polymerizing factor**	Cytoplasm	enzyme		1.025703517	0	0.93333182	0	1	0	10.0411458	10.3098304	10.2707845	10.1493422
5377	**ENSG0000012399**	DNPEP	protein_coding	chr2	219372043	219400022	-	Human GRCh	OTTHUMG000000	DNPEP	**aspartyl aminopeptidase**	Cytoplasm	peptidase		-1.029873622	0	0.88690583	0	1	0	9.4004168	9.93482466	9.82700729	9.47783136
5379	**ENSG0000012400**	MOGAT1	protein_coding	chr2	222671658	222709930	+	Human GRCh	OTTHUMG000001	MOGAT1	**monoacylglycerol O-acyltransferase 1**	Cytoplasm	enzyme		1.462455669	0	0.64269386	0	1	0	3.26352178	4.28714013	4.59499419	3.66168907
5380	**ENSG0000012400**	OBSL1	protein_coding	chr2	219550728	219571859	-	Human GRCh	OTTHUMG000000	OBSL1	**obscurin like cytoskeletal adaptor 1**	Cytoplasm	other		-3.166238163	-1	0.02847854	1	1	0	5.93542036	6.15346031	5.29815638	4.89280195
5382	**ENSG0000012406**	SLC12A4	protein_coding	chr16	67943474	67969601	-	Human GRCh	OTTHUMG000001	SLC12A4	**solute carrier family 12 member 4**	Plasma Mem	transporter	bumetanic	-1.027323617	0	0.91850234	0	1	0	8.79691163	8.78452283	8.89532787	8.61311719
5383	**ENSG0000012407**	ENKD1	protein_coding	chr16	67662945	67667265	-	Human GRCh	OTTHUMG000001	ENKD1	**enkurin domain containing 1**	Cytoplasm	other		1.109964162	0	0.62289502	0	1	0	8.9462562	9.23860228	9.25153752	9.18357654
5386	**ENSG0000012409**	CTCFL	protein_coding	chr20	57495966	57525652	-	Human GRCh	OTTHUMG000000	CTCFL	**CCCTC-binding factor like**	Nucleus	transcription		1	0	1	0	1	0	0.08942908	0.16406384	0.1521795	0.09399849

5387	**ENSG0000012409**	HMGB1P1	protein_coding	chr20	57487959	57495366	-	Human GRCh	OTTHUMG000000	HMGB1P1	**high mobility group box 1 pseudogene 1**	Other	other		-5.049821436	-1	0.50073947	0	NA	0	0.79022211	0.48649049	0.48550942	0.48100602
5388	**ENSG0000012409**	FAM210B	protein_coding	chr20	56358974	56368663	+	Human GRCh	OTTHUMG000000	FAM210B	**family with sequence similarity 210 member B**	Extracellular	other		1.130859957	0	0.63952912	0	1	0	9.28906931	9.88723997	9.93223371	9.55653486
5389	**ENSG0000012410**	PI3	protein_coding	chr20	45174902	45176544	+	Human GRCh	OTTHUMG000000	PI3	**peptidase inhibitor 3**	Extracellular	other		1	0	1	0	NA	0	0.36338057	0.3739621	0.37219852	0.36399532
5391	**ENSG0000012410**	SNX21	protein_coding	chr20	45833799	45843276	+	Human GRCh	OTTHUMG000000	SNX21	**sorting nexin family member 21**	Cytoplasm	transporter		1.204380468	0	0.45542309	0	1	0	7.81119363	7.6972482	8.03335943	7.86099888
5392	**ENSG0000012410**	SLPI	protein_coding	chr20	45252239	45254564	-	Human GRCh	OTTHUMG000000	SLPI	**secretory leukocyte peptidase inhibitor**	Cytoplasm	other		-5.419190575	-1	0.18077278	0	1	0	2.28504217	1.70272785	1.68307054	1.58654999
5393	**ENSG0000012411**	WFDC3	protein_coding	chr20	45747944	45791932	-	Human GRCh	OTTHUMG000000	WFDC3	**WAP four-disulfide core domain 3**	Extracellular	other		-1.720632251	-1	0.42755229	0	1	0	3.73833894	3.40322856	3.51370115	2.8655387
5394	**ENSG0000012412**	TTPAL	protein_coding	chr20	44475874	44494603	+	Human GRCh	OTTHUMG000000	TTPAL	**alpha tocopherol transfer protein like**	Other	other		-1.024001941	0	0.9149815	0	1	0	8.73078491	8.7829656	8.91560012	8.51556193
5395	**ENSG0000012412**	PREX1	protein_coding	chr20	48624252	48827999	-	Human GRCh	OTTHUMG000000	PREX1	**phosphatidylinositol-3,4,5-trisphosphate dependent Rac excha**	Cytoplasm	other		2.183295158	1	0.23700138	0	1	0	4.29346469	4.65318099	4.97829658	5.47767264
5397	**ENSG0000012414**	SLC12A5	protein_coding	chr20	46021690	46060150	+	Human GRCh	OTTHUMG000000	SLC12A5	**solute carrier family 12 member 5**	Plasma Mem	transporter	bumetanic	-1.7410097	-1	0.21282455	0	1	0	4.43413944	4.85718525	4.04050033	4.33565495
5399	**ENSG0000012414**	SDC4	protein_coding	chr20	45325288	45348424	-	Human GRCh	OTTHUMG000000	SDC4	**syndecan 4**	Plasma Mem	other		1.228196219	0	0.42242064	0	1	0	10.9693874	11.2976169	11.5095298	11.2156466
5400	**ENSG0000012415**	NCOA3	protein_coding	chr20	47501887	47656877	+	Human GRCh	OTTHUMG000000	NCOA3	**nuclear receptor coactivator 3**	Nucleus	transcription		-1.15033557	0	0.51798521	0	1	0	10.8635566	11.1938565	10.989992	10.7738247
5401	**ENSG0000012415**	PIGT	protein_coding	chr20	45416084	45456934	+	Human GRCh	OTTHUMG000000	PIGT	**phosphatidylinositol glycan anchor biosynthesis class T**	Cytoplasm	enzyme		-1.05289529	0	0.80860429	0	1	0	11.5670398	11.8380537	11.7350779	11.5645746
5403	**ENSG0000012415**	MATN4	protein_coding	chr20	45293445	45308529	-	Human GRCh	OTTHUMG000000	MATN4	**matrilin 4**	Extracellular	other		16.0845968	1	0.02432953	1	NA	0	2.03416403	1.69655385	3.41933168	2.79377612
5404	**ENSG0000012416**	NCOA5	protein_coding	chr20	46060991	46089962	-	Human GRCh	OTTHUMG000000	NCOA5	**nuclear receptor coactivator 5**	Nucleus	other		-1.177519166	0	0.48243364	0	1	0	10.1510591	10.4088813	10.2565628	9.93922542
5405	**ENSG0000012416**	VAPB	protein_coding	chr20	58389229	58451101	+	Human GRCh	OTTHUMG000000	VAPB	**VAMP associated protein B and C**	Plasma Mem	other		-1.004637028	0	0.98110423	0	1	0	11.1697579	11.3527617	11.3908684	11.1121357
5406	**ENSG0000012417**	PARD6B	protein_coding	chr20	50731580	50756795	+	Human GRCh	OTTHUMG000000	PARD6B	**par-6 family cell polarity regulator beta**	Plasma Mem	other		-1.004919361	0	0.98261977	0	1	0	8.77391426	9.00250333	9.06125624	8.68586796
5407	**ENSG0000012417**	ATP5F1E	protein_coding	chr20	59025475	59032345	-	Human GRCh	OTTHUMG000000	ATP5F1E	**ATP synthase F1 subunit epsilon**	Cytoplasm	transporter		1.030146606	0	0.88797736	0	1	0	10.7860689	11.1541351	11.0990927	10.929828
5408	**ENSG0000012417**	CHD6	protein_coding	chr20	41402083	41618384	-	Human GRCh	OTTHUMG000000	CHD6	**chromodomain helicase DNA binding protein 6**	Nucleus	enzyme		-1.186131437	0	0.42908366	0	1	0	12.5049225	12.7881936	12.6653616	12.2300105
5409	**ENSG0000012418**	PLCG1	protein_coding	chr20	41136960	41196801	+	Human GRCh	OTTHUMG000000	PLCG1	**phospholipase C gamma 1**	Cytoplasm	enzyme		-1.082540868	0	0.72059225	0	1	0	11.3959761	11.697158	11.6199512	11.2962082
5410	**ENSG0000012419**	TOX2	protein_coding	chr20	43914852	44069616	+	Human GRCh	OTTHUMG000000	TOX2	**TOX high mobility group box family member 2**	Nucleus	transcription		-1.320381544	0	0.43531991	0	1	0	7.62327559	7.92653087	7.64985667	7.32213537
5411	**ENSG0000012419**	SRSF6	protein_coding	chr20	43457893	43466046	+	Human GRCh	OTTHUMG000000	SRSF6	**serine and arginine rich splicing factor 6**	Nucleus	other		-1.185932926	0	0.39030017	0	1	0	12.6922727	12.932119	12.772117	12.4645983
5412	**ENSG0000012419**	GDAP1L1	protein_coding	chr20	44247099	44280947	+	Human GRCh	OTTHUMG000000	GDAP1L1	**ganglioside induced differentiation associated protein 1 like 1**	Other	other		1	0	1	0	1	0	1.49030355	1.55717057	1.54634736	1.49432225
5414	**ENSG0000012419**	ARFGEF2	protein_coding	chr20	48921711	49036693	+	Human GRCh	OTTHUMG000000	ARFGEF2	**ADP ribosylation factor guanine nucleotide exchange factor 2**	Cytoplasm	other		-1.110094455	0	0.61264998	0	1	0	11.4401526	11.7898833	11.6601848	11.3426097
5415	**ENSG0000012420**	ZNFX1	protein_coding	chr20	49237946	49278426	-	Human GRCh	OTTHUMG000000	ZNFX1	**zinc finger NFX1-type containing 1**	Nucleus	transcription		-1.125939024	0	0.55395938	0	1	0	10.613132	11.0006889	10.791744	10.5810907
5418	**ENSG0000012420**	CSE1L	protein_coding	chr20	49046246	49096960	+	Human GRCh	OTTHUMG000000	CSE1L	**chromosome segregation 1 like**	Nucleus	transporter		-1.086463568	0	0.70512524	0	1	0	12.5906994	12.8543509	12.7481041	12.5141094
5419	**ENSG0000012420**	RAB22A	protein_coding	chr20	58309715	58367507	+	Human GRCh	OTTHUMG000000	RAB22A	**RAB22A, member RAS oncogene family**	Cytoplasm	enzyme		-1.105336397	0	0.63358677	0	1	0	10.5325916	10.8735589	10.7166849	10.4822604
5420	**ENSG0000012421**	PTGIS	protein_coding	chr20	49503874	49568137	-	Human GRCh	OTTHUMG000000	PTGIS	**prostaglandin I2 synthase**	Cytoplasm	enzyme	phenylbut:	-4.675407691	-1	0.32760061	0	NA	0	0.98497378	2.01096087	0.99290152	1.31670818
5421	**ENSG0000012421**	STAU1	protein_coding	chr20	49113339	49188367	-	Human GRCh	OTTHUMG000000	STAU1	**staufen double-stranded RNA binding protein 1**	Cytoplasm	transporter		-1.167534384	0	0.45903311	0	1	0	11.8163032	12.2604952	12.0105415	11.7461515
5422	**ENSG0000012421**	CDH26	protein_coding	chr20	59958423	60034011	+	Human GRCh	OTTHUMG000000	CDH26	**cadherin 26**	Plasma Mem	other		-1.265548622	0	0.7752045	0	1	0	2.92831659	2.4711258	2.14863695	2.93668893
5423	**ENSG0000012421**	SNAI1	protein_coding	chr20	49982980	49988886	+	Human GRCh	OTTHUMG000000	SNAI1	**snail family transcriptional repressor 1**	Nucleus	transcription		1.812475589	1	0.1300087	0	1	0	5.84450875	5.52936622	6.0644931	6.46743504
5424	**ENSG0000012421**	MOCS3	protein_coding	chr20	50958818	50963929	+	Human GRCh	OTTHUMG000000	MOCS3	**molybdenum cofactor synthesis 3**	Cytoplasm	enzyme		-1.027330213	0	0.90417503	0	1	0	9.32668965	9.59219578	9.52227567	9.34395133
5425	**ENSG0000012422**	STX16	protein_coding	chr20	58651272	58679526	+	Human GRCh	OTTHUMG000000	STX16	**syntaxin 16**	Cytoplasm	transporter		1.035209918	0	0.86215116	0	1	0	10.8629191	11.0834553	11.1529485	10.8616042
5426	**ENSG0000012422**	PMEPA1	protein_coding	chr20	57648392	57711536	-	Human GRCh	OTTHUMG000000	PMEPA1	**prostate transmembrane protein, androgen induced 1**	Plasma Mem	other		-1.35766535	0	0.70634575	0	1	0	6.23423593	6.70429782	6.47622164	5.78858335
5427	**ENSG0000012422**	RNF114	protein_coding	chr20	49936336	49953885	+	Human GRCh	OTTHUMG000000	RNF114	**ring finger protein 114**	Cytoplasm	transcription		-1.046731402	0	0.81864773	0	1	0	10.6377294	10.8411564	10.822901	10.5478841
5429	**ENSG0000012422**	DDX27	protein_coding	chr20	49219347	49244077	+	Human GRCh	OTTHUMG000000	DDX27	**DEAD-box helicase 27**	Nucleus	enzyme		-1.037196948	0	0.85638607	0	1	0	10.0674754	10.4954884	10.379915	10.1282575
5433	**ENSG0000012424**	BCAS4	protein_coding	chr20	50794894	50877177	+	Human GRCh	OTTHUMG000000	BCAS4	**breast carcinoma amplified sequence 4**	Cytoplasm	other		1.162668586	0	0.76319273	0	1	0	4.67271799	3.92398378	4.34088343	4.61889617
5435	**ENSG0000012425**	TP53TG5	protein_coding	chr20	45372557	45407889	-	Human GRCh	OTTHUMG000000	TP53TG5	**TP53 target 5**	Nucleus	other		2.715571513	1	0.5287112	0	NA	0	1.71690628	1.74139804	1.98144674	1.71833021
5436	**ENSG0000012425**	PCK1	protein_coding	chr20	57561080	57568121	+	Human GRCh	OTTHUMG000000	PCK1	**phosphoenolpyruvate carboxykinase 1**	Cytoplasm	kinase		5.699066934	1	0.36504774	0	NA	0	1.08019669	1.09335868	1.50240556	1.08095714
5438	**ENSG0000012425**	NEURL2	protein_coding	chr20	45888625	45891208	-	Human GRCh	OTTHUMG000000	NEURL2	**neuralized E3 ubiquitin protein ligase 2**	Cytoplasm	other		1.332734816	0	0.3771886	0	1	0	6.12671639	5.63092919	6.36853994	5.98062796
5439	**ENSG0000012426**	MAGEA10	protein_coding	chrX	152133310	152138578	-	Human GRCh	OTTHUMG000000	MAGEA10	**MAGE family member A10**	Nucleus	other		-5.802891488	-1	0.27258441	0	1	0	1.76613684	2.56593884	1.85753542	1.77277894
5440	**ENSG0000012427**	MTRR	protein_coding	chr5	7851186	7906025	+	Human GRCh	OTTHUMG000000	MTRR	**5-methyltetrahydrofolate-homocysteine methyltransferase re**	Cytoplasm	enzyme		-1.11557024	0	0.63664444	0	1	0	9.57117458	9.74157477	9.68758415	9.37110404
5441	**ENSG0000012427**	FASTKD3	protein_coding	chr5	7859159	7869037	-	Human GRCh	OTTHUMG000001	FASTKD3	**FAST kinase domains 3**	Cytoplasm	other		-1.02248029	0	0.93713843	0	1	0	7.86904979	8.29330262	8.18993847	7.94952702
5442	**ENSG0000012429**	PEPD	protein_coding	chr19	33386950	33521823	-	Human GRCh	OTTHUMG000001	PEPD	**peptidase D**	Cytoplasm	peptidase		-1.044752015	0	0.85795361	0	1	0	9.4564417	9.75011371	9.74383941	9.35387034
5443	**ENSG0000012430**	CHST8	protein_coding	chr19	33621953	33773509	+	Human GRCh	OTTHUMG000001	CHST8	**carbohydrate sulfotransferase 8**	Cytoplasm	enzyme		3.127720233	1	0.06752301	0	1	0	2.79471982	2.23076736	2.50759746	3.86445134
5444	**ENSG0000012431**	IQSEC2	protein_coding	chrX	53225828	53321350	-	Human GRCh	OTTHUMG000000	IQSEC2	**IQ motif and Sec7 domain ArfGEF 2**	Cytoplasm	other		-1.02385865	0	0.93064726	0	1	0	7.8058959	8.39725856	8.24011784	7.9700558
5445	**ENSG0000012433**	VAMP7	protein_coding	chrX	155881345	155943769	+	Human GRCh	OTTHUMG000000	VAMP7	**vesicle associated membrane protein 7**	Cytoplasm	transporter		-1.072021123	0	0.76136245	0	1	0	9.70825075	10.0604626	10.024755	9.57413072
5446	**ENSG0000012433**	IL9R	protein_coding	chrX	155997696	156010817	+	Human GRCh	OTTHUMG000000	IL9R	**interleukin 9 receptor**	Plasma Mem	transmembra		-1.606585467	-1	0.52257082	0	1	0	3.5029325	3.85538925	3.35901768	3.39431614
5448	**ENSG0000012435**	STAMBP	protein_coding	chr2	73828916	73873659	+	Human GRCh	OTTHUMG000001	STAMBP	**STAM binding protein**	Nucleus	enzyme		-1.101990133	0	0.62444924	0	1	0	11.0076335	11.3092837	11.1700347	10.9390423
5449	**ENSG0000012435**	NAGK	protein_coding	chr2	71064344	71079808	+	Human GRCh	OTTHUMG000001	NAGK	**N-acetylglucosamine kinase**	Cytoplasm	kinase		1.019969171	0	0.92691672	0	1	0	9.73627882	9.91172224	10.0312683	9.63253672
5450	**ENSG0000012437**	MCEE	protein_coding	chr2	71109684	71130239	-	Human GRCh	OTTHUMG000001	MCEE	**methylmalonyl-CoA epimerase**	Cytoplasm	enzyme		-1.215136044	0	0.35729265	0	1	0	8.41873668	8.4378006	8.24557638	8.18522759
5451	**ENSG0000012437**	PAIP2B	protein_coding	chr2	71182738	71227103	-	Human GRCh	OTTHUMG000001	PAIP2B	**poly(A) binding protein interacting protein 2B**	Other	translation re		-1.092503768	0	0.68088383	0	1	0	10.6926492	10.9988965	10.8537315	10.6556091
5452	**ENSG0000012438**	SNRNP27	protein_coding	chr2	69893956	69905575	+	Human GRCh	OTTHUMG000001	SNRNP27	**small nuclear ribonucleoprotein U4/U6.U5 subunit 27**	Nucleus	other		1.066581473	0	0.7696935	0	1	0	9.16927916	9.42217766	9.45804507	9.28123628
5453	**ENSG0000012438**	MPHOSPH	protein_coding	chr2	71130310	71150101	+	Human GRCh	OTTHUMG000001	MPHOSPH10	**M-phase phosphoprotein 10**	Nucleus	other		1.003401152	0	0.98624612	0	1	0	10.2816099	10.5676946	10.5527609	10.3086637
5454	**ENSG0000012439**	IL17C	protein_coding	chr16	88638572	88640468	+	Human GRCh	OTTHUMG000001	IL17C	**interleukin 17C**	Extracellular	cytokine	MOR106	35.09007226	1	0.01344374	1	NA	0	1.55608526	1.57199145	1.56932645	2.86671635
5455	**ENSG0000012440**	ATP8A1	protein_coding	chr4	42408373	42657121	-	Human GRCh	OTTHUMG000000	ATP8A1	**ATPase phospholipid transporting 8A1**	Cytoplasm	transporter		1.222494708	0	0.41523705	0	1	0	9.21358789	9.48959947	9.58122395	9.54921683
5456	**ENSG0000012442**	USP22	protein_coding	chr17	20999596	21043760	-	Human GRCh	OTTHUMG000001	USP22	**ubiquitin specific peptidase 22**	Nucleus	peptidase		-1.112415052	0	0.60566391	0	1	0	13.2702961	13.4836447	13.3795903	13.1318739
5457	**ENSG0000012442**	POF1B	protein_coding	chrX	85277396	85379717	-	Human GRCh	OTTHUMG000000	POF1B	**POF1B actin binding protein**	Plasma Mem	other		1.779868761	1	0.5158312	0	1	0	2.00931898	3.49182719	2.7519785	3.74234785
5458	**ENSG0000012444**	HIF3A	protein_coding	chr19	46297042	46343433	+	Human GRCh	OTTHUMG000001	HIF3A	**hypoxia inducible factor 3 subunit alpha**	Nucleus	transcription		-1.323434183	0	0.63346332	0	1	0	8.30838744	8.42510733	8.02499963	8.06631173
5459	**ENSG0000012444**	ZNF576	protein_coding	chr19	43596392	43601157	+	Human GRCh	OTTHUMG000001	ZNF576	**zinc finger protein 576**	Nucleus	transcription		1.224989102	0	0.36530746	0	1	0	7.89190722	8.19144404	8.36026031	8.17484009
5461	**ENSG0000012445**	ZNF45	protein_coding	chr19	43912624	43935282	-	Human GRCh	OTTHUMG000001	ZNF45	**zinc finger protein 45**	Nucleus	transcription		-1.003704076	0	0.98858099	0	1	0	8.26209928	8.34486066	8.46235449	8.11264033
5466	**ENSG0000012448**	USP9X	protein_coding	chrX	41085445	41236579	+	Human GRCh	OTTHUMG000000	USP9X	**ubiquitin specific peptidase 9 X-linked**	Plasma Mem	peptidase		-1.09032718	0	0.68243013	0	1	0	12.7600318	13.0525669	12.9508753	12.6742864
5468	**ENSG0000012449**	F13A1	protein_coding	chr6	6144084	6321013	-	Human GRCh	OTTHUMG000000	F13A1	**coagulation factor XIII A chain**	Extracellular	enzyme		1.480904273	0	0.55383463	0	1	0	4.10240799	4.73749886	4.67649555	5.00700105
5469	**ENSG0000012449**	GRM4	protein_coding	chr6	34018643	34155622	-	Human GRCh	OTTHUMG000000	GRM4	**glutamate metabotropic receptor 4**	Plasma Mem	G-protein coı	fasoraceta	1.236960654	0	0.81547776	0	1	0	4.11833027	3.73266729	3.71266961	4.31190982
5470	**ENSG0000012449**	TRERF1	protein_coding	chr6	42224931	42452051	-	Human GRCh	OTTHUMG000000	TRERF1	**transcriptional regulating factor 1**	Nucleus	transcription		-1.090109392	0	0.80065917	0	1	0	8.13428189	8.68584295	8.30834148	8.40211917
5471	**ENSG0000012450**	PACSIN1	protein_coding	chr6	34466061	34535229	+	Human GRCh	OTTHUMG000000	PACSIN1	**protein kinase C and casein kinase substrate in neurons 1**	Cytoplasm	kinase		1.14910023	0	0.63611141	0	1	0	8.59198459	8.7380282	8.90461054	8.73616261
5472	**ENSG0000012450**	BTN2A2	protein_coding	chr6	26383096	26394874	+	Human GRCh	OTTHUMG000000	BTN2A2	**butyrophilin subfamily 2 member A2**	Cytoplasm	other		1.258841086	0	0.36071491	0	1	0	7.45312411	7.74795194	7.89354774	7.80478589
5473	**ENSG0000012452**	SIRT5	protein_coding	chr6	13574227	13615158	+	Human GRCh	OTTHUMG000000	SIRT5	**sirtuin 5**	Cytoplasm	enzyme	suramin	-1.130933272	0	0.61013902	0	1	0	8.32255236	8.62136224	8.45074828	8.24383943
5474	**ENSG0000012453**	MRS2	protein_coding	chr6	24402908	24426194	+	Human GRCh	OTTHUMG000000	MRS2	**magnesium transporter MRS2**	Cytoplasm	transporter		-1.073253135	0	0.73318177	0	1	0	10.411553	10.7214914	10.651715	10.3207724
5475	**ENSG0000012453**	WRNIP1	protein_coding	chr6	2765393	2786952	+	Human GRCh	OTTHUMG000000	WRNIP1	**WRN helicase interacting protein 1**	Nucleus	enzyme		1.026846482	0	0.89482611	0	1	0	10.3904934	10.6861652	10.7225125	10.4096997
5476	**ENSG0000012454**	RRP36	protein_coding	chr6	43021623	43034156	+	Human GRCh	OTTHUMG000000	RRP36	**ribosomal RNA processing 36**	Nucleus	other		-1.046109989	0	0.85042806	0	1	0	10.1744677	10.6653436	10.4653752	10.3185377
5478	**ENSG0000012456**	SNRPC	protein_coding	chr6	34757505	34773857	+	Human GRCh	OTTHUMG000000	SNRPC	**small nuclear ribonucleoprotein polypeptide C**	Nucleus	other		-1.173665191	0	0.46187528	0	1	0	10.5347238	10.8876667	10.5993319	10.488075
5481	**ENSG0000012457**	SERPINB6	protein_coding	chr6	2948159	2972165	-	Human GRCh	OTTHUMG000000	SERPINB6	**serpin family B member 6**	Cytoplasm	other		1.296436994	0	0.3465493	0	1	0	7.79893261	7.6224222	7.88276255	8.100665
5482	**ENSG0000012457**	XPO5	protein_coding	chr6	43522334	43576038	-	Human GRCh	OTTHUMG000000	XPO5	**exportin 5**	Nucleus	other		-1.123191611	0	0.53871342	0	1	0	11.9447311	12.2556245	12.1405141	11.7941628
5483	**ENSG0000012457**	ABCC10	protein_coding	chr6	43427366	43450427	+	Human GRCh	OTTHUMG000000	ABCC10	**ATP binding cassette subfamily C member 10**	Plasma Mem	transporter		1.009948679	0	0.96194009	0	1	0	10.0285201	10.3213574	10.3199634	10.0547924
5484	**ENSG0000012457**	H1-3	protein_coding	chr6	26234212	26234987	-	Human GRCh	OTTHUMG000000	H1-3	**H1.3 linker histone, cluster member**	Nucleus	other		1	0	1	0	1	0	2.70841241	2.80966377	2.79334112	2.71452739
5485	**ENSG0000012458**	PEX6	protein_coding	chr6	42963865	42979181	-	Human GRCh	OTTHUMG000000	PEX6	**peroxisomal biogenesis factor 6**	Cytoplasm	enzyme		-1.131449166	0	0.52809448	0	1	0	10.132318	10.2740098	10.1933115	9.92514437
5486	**ENSG0000012458**	NQO2	protein_coding	chr6	2987987	3019755	+	Human GRCh	OTTHUMG000000	NQO2	**N-ribosyldihydronicotinamide:quinone dehydrogenase 2**	Cytoplasm	enzyme	caricotami	1.158883332	0	0.58849742	0	1	0	8.69307154	8.9613265	9.09135556	8.9026281
5487	**ENSG0000012459**	OARD1	protein_coding	chr6	41033627	41097787	-	Human GRCh	OTTHUMG000000	OARD1	**O-acyl-ADP-ribose deacylase 1**	Nucleus	enzyme		-1.138519422	0	0.55472538	0	1	0	9.26976505	9.66536379	9.50807684	9.16164476
5488	**ENSG0000012460**	UNC5CL	protein_coding	chr6	41026895	41039221	-	Human GRCh	OTTHUMG000000	UNC5CL	**unc-5 family C-terminal like**	Cytoplasm	peptidase		-1.56598856	-1	0.1159756	0	1	0	6.6080943	7.01771739	6.34667288	6.41301281
5489	**ENSG0000012460**	AARS2	protein_coding	chr6	44298731	44313347	-	Human GRCh	OTTHUMG000000	AARS2	**alanyl-tRNA synthetase 2, mitochondrial**	Cytoplasm	enzyme		1.061697514	0	0.7467297	0	1	0	10.2212742	10.3964226	10.537561	10.1935531
5491	**ENSG0000012461**	ZNF391	protein_coding	chr6	27374615	27403908	+	Human GRCh	OTTHUMG000000	ZNF391	**zinc finger protein 391**	Other	other		-1.123067567	0	0.64525463	0	1	0	8.5775492	8.62861193	8.60084537	8.32835041
5492	**ENSG0000012461**	RPS10	protein_coding	chr6	34417454	34426069	-	Human GRCh	OTTHUMG000000	RPS10	**ribosomal protein S10**	Cytoplasm	other		-1.067523492	0	0.74486581	0	1	0	12.6945656	13.0642508	12.883734	12.7519209
5493	**ENSG0000012461**	MOCS1	protein_coding	chr6	39899578	39934551	-	Human GRCh	OTTHUMG000000	MOCS1	**molybdenum cofactor synthesis 1**	Cytoplasm	other		-1.215391922	0	0.42921358	0	1	0	8.23294278	8.69303109	8.31494793	8.23977221

5494	**ENSG0000012463**	H2BC11	protein_coding	chr6	27125897	27132795	-	Human GRCh	OTTHUMG000000	H2BC11	**H2B clustered histone 11**	Nucleus	other		-1.195582164	0	0.47562928	0	1	0	10.7217472	11.0249809	10.8179941	10.5504385
5495	**ENSG0000012464**	MED20	protein_coding	chr6	41905354	41921139	-	Human GRCh	OTTHUMG000000	MED20	**mediator complex subunit 20**	Nucleus	transcription	zeaxanthir	1.01851095	0	0.93360601	0	1	0	9.11377867	9.44225222	9.42874617	9.14193293
5496	**ENSG0000012465**	OR2B6	protein_coding	chr6	27957241	27958182	+	Human GRCh	OTTHUMG000000	OR2B6	**olfactory receptor family 2 subfamily B member 6**	Plasma Mem	G-protein cou		2.617072522	1	0.19854272	0	1	0	3.42010264	3.48948425	3.7213199	4.4805689
5497	**ENSG0000012465**	TBCC	protein_coding	chr6	42744498	42746103	-	Human GRCh	OTTHUMG000000	TBCC	**tubulin folding cofactor C**	Cytoplasm	enzyme		-1.081088193	0	0.72665044	0	1	0	9.03391042	9.24328303	9.19936255	8.89665592
5498	**ENSG0000012466**	SPDEF	protein_coding	chr6	34537802	34556333	-	Human GRCh	OTTHUMG000000	SPDEF	**SAM pointed domain containing ETS transcription factor**	Nucleus	transcription		1.239554287	0	0.79777864	0	1	0	7.41170767	7.16529486	7.39695019	7.67518201
5499	**ENSG0000012467**	TCP11	protein_coding	chr6	35118071	35148610	-	Human GRCh	OTTHUMG000000	TCP11	**t-complex 11**	Cytoplasm	other		2.652517165	1	0.5977962	0	NA	0	0.99124409	1.00936334	1.00634689	1.23309809
5500	**ENSG0000012468**	MAD2L1BI	protein_coding	chr6	43629540	43640941	+	Human GRCh	OTTHUMG000000	MAD2L1BP	**MAD2L1 binding protein**	Nucleus	other		-1.171889134	0	0.50185594	0	1	0	8.86078987	9.26728611	9.0000815	8.80633065
5502	**ENSG0000012470**	KLHDC3	protein_coding	chr6	43014103	43021298	+	Human GRCh	OTTHUMG000000	KLHDC3	**kelch domain containing 3**	Cytoplasm	other		-1.086710499	0	0.70024968	0	1	0	11.8746783	12.2037982	12.0941432	11.8011502
5503	**ENSG0000012471**	GNMT	protein_coding	chr6	42960754	42963880	+	Human GRCh	OTTHUMG000000	GNMT	**glycine N-methyltransferase**	Cytoplasm	enzyme		-1.044310232	0	0.91723934	0	1	0	8.63431058	9.00173953	8.79663618	8.76696134
5504	**ENSG0000012472**	DNAH8	protein_coding	chr6	38715311	39030792	+	Human GRCh	OTTHUMG000000	DNAH8	**dynein axonemal heavy chain 8**	Cytoplasm	enzyme		3.483788567	1	0.36633873	0	1	0	0.74720973	1.81562615	0.84778528	2.81967528
5505	**ENSG0000012473**	TREM1	protein_coding	chr6	41267926	41286682	-	Human GRCh	OTTHUMG000000	TREM1	**triggering receptor expressed on myeloid cells 1**	Plasma Mem	transmembra	PY159, ant	1	0	1	0	1	0	1.40842369	1.50505568	1.48959925	1.41431154
5506	**ENSG0000012473**	MEA1	protein_coding	chr6	43011143	43016868	-	Human GRCh	OTTHUMG000000	MEA1	**male-enhanced antigen 1**	Cytoplasm	other		1.142083934	0	0.53338395	0	1	0	9.91554148	10.1752739	10.2039926	10.1960301
5507	**ENSG0000012474**	KLHL31	protein_coding	chr6	53647916	53665756	-	Human GRCh	OTTHUMG000000	KLHL31	**kelch like family member 31**	Other	other		-1.734709409	-1	0.13950438	0	1	0	6.46685807	6.48727894	5.98966461	5.86614729
5508	**ENSG0000012474**	COL21A1	protein_coding	chr6	56056590	56394094	-	Human GRCh	OTTHUMG000000	COL21A1	**collagen type XXI alpha 1 chain**	Extracellular	other	collagenas	1	0	1	0	1	0	2.84169517	2.94510987	2.92844504	2.84794404
5509	**ENSG0000012476**	CDKN1A	protein_coding	chr6	36676460	36687337	+	Human GRCh	OTTHUMG000000	CDKN1A	**cyclin dependent kinase inhibitor 1A**	Nucleus	kinase		1.075001905	0	0.79437632	0	1	0	6.96482074	7.47519202	7.12464816	7.47060609
5510	**ENSG0000012476**	SOX4	protein_coding	chr6	21593751	21598619	+	Human GRCh	OTTHUMG000000	SOX4	**SRY-box transcription factor 4**	Nucleus	transcription		1.171969958	0	0.48188347	0	1	0	12.8127067	12.8344797	13.1346455	12.8393659
5511	**ENSG0000012476**	GLO1	protein_coding	chr6	38675925	38703145	-	Human GRCh	OTTHUMG000000	GLO1	**glyoxalase I**	Cytoplasm	enzyme		-1.107595796	0	0.63450907	0	1	0	11.6230848	11.9082592	11.7723014	11.5367177
5512	**ENSG0000012477**	CPNE5	protein_coding	chr6	36740775	36839444	-	Human GRCh	OTTHUMG000000	CPNE5	**copine 5**	Plasma Mem	other		1.277513046	0	0.75080349	0	1	0	2.80257808	2.92438098	3.34071801	2.50877259
5513	**ENSG0000012478**	KCNK17	protein_coding	chr6	39299001	39314461	-	Human GRCh	OTTHUMG000000	KCNK17	**potassium two pore domain channel subfamily K member 17**	Plasma Mem	ion channel		-5.492996321	-1	0.17122453	0	1	0	2.39818838	3.09106911	2.4923064	2.40500395
5514	**ENSG0000012478**	RREB1	protein_coding	chr6	7107597	7251980	+	Human GRCh	OTTHUMG000000	RREB1	**ras responsive element binding protein 1**	Nucleus	transcription		-1.025845768	0	0.90448742	0	1	0	12.3860871	12.5536444	12.5955586	12.2707882
5515	**ENSG0000012478**	SSR1	protein_coding	chr6	7268306	7347446	-	Human GRCh	OTTHUMG000000	SSR1	**signal sequence receptor subunit 1**	Cytoplasm	other		-1.097280625	0	0.66701815	0	1	0	12.7816788	13.0926386	12.9865842	12.6787277
5516	**ENSG0000012478**	RIOK1	protein_coding	chr6	7389793	7418037	+	Human GRCh	OTTHUMG000000	RIOK1	**RIO kinase 1**	Cytoplasm	kinase		1.031491191	0	0.87737323	0	1	0	9.71280787	9.95768868	10.0106289	9.72381349
5517	**ENSG0000012478**	NRN1	protein_coding	chr6	5997999	6007605	-	Human GRCh	OTTHUMG000000	NRN1	**neuritin 1**	Cytoplasm	other		-1.005901491	0	0.9845027	0	1	0	9.77601565	10.2511018	10.1550516	9.88886634
5518	**ENSG0000012478**	SLC35B3	protein_coding	chr6	8411435	8435561	-	Human GRCh	OTTHUMG000000	SLC35B3	**solute carrier family 35 member B3**	Cytoplasm	transporter		1.040396265	0	0.85837328	0	1	0	7.8299223	8.06697256	8.06918053	7.91606289
5519	**ENSG0000012478**	RPP40	protein_coding	chr6	4994717	5004063	-	Human GRCh	OTTHUMG000000	RPP40	**ribonuclease P/MRP subunit p40**	Nucleus	enzyme		1.207779284	0	0.51447971	0	1	0	7.25243575	7.84512536	7.91293941	7.64993753
5520	**ENSG0000012478**	ATXN1	protein_coding	chr6	16299112	16761490	-	Human GRCh	OTTHUMG000000	ATXN1	**ataxin 1**	Nucleus	transcription		-1.387762807	0	0.70587907	0	1	0	7.67382706	7.8907592	6.93743872	7.73300762
5521	**ENSG0000012478**	NUP153	protein_coding	chr6	17615035	17706925	-	Human GRCh	OTTHUMG000000	NUP153	**nucleoporin 153**	Nucleus	other		-1.154480447	0	0.4566421	0	1	0	12.3480486	12.6442419	12.4470215	12.2337002
5522	**ENSG0000012479**	DEK	protein_coding	chr6	18223860	18264548	-	Human GRCh	OTTHUMG000000	DEK	**DEK proto-oncogene**	Nucleus	transcription		1.12177992	0	0.57388718	0	1	0	11.8122954	11.958586	12.136616	11.8672707
5523	**ENSG0000012480**	EEF1E1	protein_coding	chr6	8073360	8102559	-	Human GRCh	OTTHUMG000000	EEF1E1	**eukaryotic translation elongation factor 1 epsilon 1**	Cytoplasm	translation re		1.159676953	0	0.53277847	0	1	0	8.93084995	9.33310015	9.46499835	9.13814293
5525	**ENSG0000012481**	RUNX2	protein_coding	chr6	45328157	45664349	+	Human GRCh	OTTHUMG000000	RUNX2	**RUNX family transcription factor 2**	Nucleus	transcription		7.714244578	1	0.03482614	1	1	0	2.49447109	2.61059822	3.61150338	4.1557192
5528	**ENSG0000012483**	LRRFIP1	protein_coding	chr2	237627567	237813682	+	Human GRCh	OTTHUMG000001	LRRFIP1	**LRR binding FLII interacting protein 1**	Cytoplasm	transcription		-1.014469463	0	0.94327111	0	1	0	11.417608	11.7215873	11.6131762	11.5117093
5530	**ENSG0000012483**	RAB17	protein_coding	chr2	237574322	237601614	-	Human GRCh	OTTHUMG000001	RAB17	**RAB17, member RAS oncogene family**	Cytoplasm	enzyme		-1.109249103	0	0.60523638	0	1	0	10.1182055	10.4251257	10.2704693	10.0494045
5535	**ENSG0000012492**	MYRF	protein_coding	chr11	61752636	61788518	+	Human GRCh	OTTHUMG000001	MYRF	**myelin regulatory factor**	Nucleus	transcription		1.676909555	1	0.03716747	1	1	0	7.71106424	8.04283632	8.27255137	8.5845033
5536	**ENSG0000012493**	SCGB1D2	protein_coding	chr11	62242239	62244812	+	Human GRCh	OTTHUMG000001	SCGB1D2	**secretoglobin family 1D member 2**	Extracellular	other		1	0	1	0	1	0	1.60315231	1.67561936	1.66390471	1.60751405
5537	**ENSG0000012493**	SCGB2A1	protein_coding	chr11	62208673	62213943	+	Human GRCh	OTTHUMG000001	SCGB2A1	**secretoglobin family 2A member 1**	Extracellular	other		-5.219735848	-1	0.29123509	0	1	0	2.4563083	1.92928971	1.91245712	1.83051375
5538	**ENSG0000012494**	AHNAK	protein_coding	chr11	62433542	62556235	-	Human GRCh	OTTHUMG000001	AHNAK	**AHNAK nucleoprotein**	Nucleus	other		-1.00279218	0	0.99635753	0	1	0	12.2297535	12.3895129	12.1249559	12.4684485
5539	**ENSG0000012503**	EMC3	protein_coding	chr3	9962682	10011202	-	Human GRCh	OTTHUMG000001	EMC3	**ER membrane protein complex subunit 3**	Nucleus	other		-1.207187029	0	0.41175248	0	1	0	8.97105481	8.98559739	8.83411391	8.70759937
5540	**ENSG0000012504**	SSUH2	protein_coding	chr3	8619386	8745040	-	Human GRCh	OTTHUMG000001	SSUH2	**ssu-2 homolog**	Cytoplasm	other		-1.283262505	0	0.45540563	0	1	0	6.84225455	6.7963403	6.59881807	6.51755884
5542	**ENSG0000012508**	SH3TC1	protein_coding	chr4	8182072	8241803	+	Human GRCh	OTTHUMG000001	SH3TC1	**SH3 domain and tetratricopeptide repeats 1**	Extracellular	other		1	0	1	0	1	0	2.18390884	2.2769221	2.26193438	2.18952879
5543	**ENSG0000012510**	CNOT1	protein_coding	chr16	58519951	58629885	-	Human GRCh	OTTHUMG000001	CNOT1	**CCR4-NOT transcription complex subunit 1**	Cytoplasm	enzyme		-1.185606594	0	0.40887575	0	1	0	13.5354132	13.8639452	13.6228906	13.4075483
5544	**ENSG0000012512**	BBS2	protein_coding	chr16	56465640	56582667	-	Human GRCh	OTTHUMG000001	BBS2	**Bardet-Biedl syndrome 2**	Cytoplasm	other		-1.057050192	0	0.82175066	0	1	0	9.55958697	9.82565366	9.84016938	9.3951185
5545	**ENSG0000012514**	MT1G	protein_coding	chr16	56666730	56668065	-	Human GRCh	OTTHUMG000001	MT1G	**metallothionein 1G**	Nucleus	other		-1.135343755	0	0.76166251	0	1	0	5.57588808	4.8052122	5.12684559	5.12547064
5546	**ENSG0000012514**	MT2A	protein_coding	chr16	56608584	56609497	+	Human GRCh	OTTHUMG000001	MT2A	**metallothionein 2A**	Cytoplasm	other		1.240318548	0	0.43837432	0	1	0	7.72717389	7.05136777	7.53396199	7.7623435
5547	**ENSG0000012514**	PHAF1	protein_coding	chr16	67109041	67148544	+	Human GRCh	OTTHUMG000001	PHAF1	**phagosome assembly factor 1**	Cytoplasm	other		-1.121726394	0	0.57973474	0	1	0	9.46507041	9.81887263	9.62007436	9.42601319
5548	**ENSG0000012516**	GOT2	protein_coding	chr16	58707131	58734342	-	Human GRCh	OTTHUMG000001	GOT2	**glutamic-oxaloacetic transaminase 2**	Cytoplasm	enzyme		-1.061985231	0	0.8042146	0	1	0	12.1603544	12.4797797	12.3890394	12.1181524
5549	**ENSG0000012517**	DOK4	protein_coding	chr16	57471922	57487327	-	Human GRCh	OTTHUMG000001	DOK4	**docking protein 4**	Plasma Mem	other		1.218000508	0	0.49671191	0	1	0	8.40563886	8.87267243	8.93101228	8.82814571
5552	**ENSG0000012524**	CLYBL	protein_coding	chr13	99606669	99909459	+	Human GRCh	OTTHUMG000000	CLYBL	**citramalyl-CoA lyase**	Cytoplasm	enzyme		1.027188886	0	0.92357013	0	1	0	7.94260289	8.52044647	8.43129585	8.14154548
5553	**ENSG0000012524**	TMTC4	protein_coding	chr13	100603625	100675093	-	Human GRCh	OTTHUMG000000	TMTC4	**transmembrane O-mannosyltransferase targeting cadherins 4**	Cytoplasm	enzyme		-1.170916886	0	0.45324556	0	1	0	9.96431991	10.3363784	10.2034153	9.73529147
5554	**ENSG0000012524**	RAP2A	protein_coding	chr13	97434169	97469128	+	Human GRCh	OTTHUMG000000	RAP2A	**RAP2A, member of RAS oncogene family**	Plasma Mem	enzyme		-1.058456862	0	0.79186389	0	1	0	10.5198614	10.7835109	10.710186	10.4713164
5556	**ENSG0000012525**	ABCC4	protein_coding	chr13	95019835	95301475	-	Human GRCh	OTTHUMG000000	ABCC4	**ATP binding cassette subfamily C member 4**	Plasma Mem	transporter		-1.078593667	0	0.78428083	0	1	0	12.2508978	12.6206485	12.6598786	11.9570558
5557	**ENSG0000012526**	EFNB2	protein_coding	chr13	106489745	106535662	-	Human GRCh	OTTHUMG000000	EFNB2	**ephrin B2**	Plasma Mem	kinase	sEphB4-HS	1.303889894	0	0.31029486	0	1	0	8.55753704	8.49214768	8.57543102	9.0268428
5558	**ENSG0000012528**	SOX21	protein_coding	chr13	94709622	94712545	-	Human GRCh	OTTHUMG000000	SOX21	**SRY-box transcription factor 21**	Nucleus	transcription		2.451250794	1	0.12483471	0	1	0	5.68262193	5.23508025	5.63464752	6.81518947
5559	**ENSG0000012530**	TM9SF2	protein_coding	chr13	99446311	99564048	+	Human GRCh	OTTHUMG000001	TM9SF2	**transmembrane 9 superfamily member 2**	Plasma Mem	transporter		-1.154245016	0	0.49264352	0	1	0	11.9612949	12.1590563	12.0522979	11.7379497
5560	**ENSG0000012531**	HROB	protein_coding	chr17	44141906	44162476	+	Human GRCh	OTTHUMG000001	HROB	**homologous recombination factor with OB-fold**	Other	other		-1.548398369	-1	0.06353041	0	1	0	10.3802668	10.4927342	9.94854325	9.92753841
5561	**ENSG0000012533**	KIF25	protein_coding	chr6	167996241	168045091	+	Human GRCh	OTTHUMG000000	KIF25	**kinesin family member 25**	Cytoplasm	enzyme		-2.078493174	-1	0.58484485	0	NA	0	1.95346413	1.75161587	1.28075656	1.82989452
5562	**ENSG0000012534**	IRF1	protein_coding	chr5	132440440	132508719	-	Human GRCh	OTTHUMG000000	IRF1	**interferon regulatory factor 1**	Nucleus	transcription		1.894064695	1	0.03634778	1	1	0	6.22104441	5.81933981	6.93908469	6.23316081
5563	**ENSG0000012535**	UPF3B	protein_coding	chrX	119834022	119852998	-	Human GRCh	OTTHUMG000000	UPF3B	**UPF3B regulator of nonsense mediated mRNA decay**	Nucleus	transporter		-1.043905128	0	0.85004297	0	1	0	9.04158645	9.46685676	9.31413006	9.13231619
5564	**ENSG0000012535**	RNF113A	protein_coding	chrX	119870475	119871733	-	Human GRCh	OTTHUMG000000	RNF113A	**ring finger protein 113A**	Extracellular	enzyme		1.026219774	0	0.93124729	0	1	0	7.79165255	8.11978153	8.18739859	7.76602743
5565	**ENSG0000012535**	SEPTIN6	protein_coding	chrX	119615724	119693370	-	Human GRCh	OTTHUMG000000	SEPTIN6	**septin 6**	Cytoplasm	other		1.098360098	0	0.74850431	0	1	0	8.0999203	8.53408915	8.61970636	8.22658308
5566	**ENSG0000012535**	TMEM255.	protein_coding	chrX	120258650	120311556	-	Human GRCh	OTTHUMG000000	TMEM255A	**transmembrane protein 255A**	Extracellular	other		5.825392727	1	0.73666205	0	NA	0	-0.5583977	-0.556004	-0.2468764	-0.5582602
5567	**ENSG0000012535**	NDUFA1	protein_coding	chrX	119870475	119876662	+	Human GRCh	OTTHUMG000000	NDUFA1	**NADH:ubiquinone oxidoreductase subunit A1**	Cytoplasm	enzyme		1.375070719	0	0.1769918	0	1	0	8.63707484	9.20396396	9.31110463	9.30226001
5569	**ENSG0000012537**	DMAC2L	protein_coding	chr14	50312324	50335558	+	Human GRCh	OTTHUMG000001	DMAC2L	**distal membrane arm assembly component 2 like**	Cytoplasm	transporter		1.019912928	0	0.93593069	0	1	0	8.637823	8.90039228	9.04284293	8.48554369
5570	**ENSG0000012537**	BMP4	protein_coding	chr14	53949736	53958761	-	Human GRCh	OTTHUMG000001	BMP4	**bone morphogenetic protein 4**	Extracellular	growth facto		-2.714468447	-1	0.42959732	0	NA	0	2.30184267	2.56470188	2.0986535	1.56440956
5571	**ENSG0000012538**	PTGER2	protein_coding	chr14	52314305	52328598	+	Human GRCh	OTTHUMG000001	PTGER2	**prostaglandin E receptor 2**	Plasma Mem	G-protein cou	misoprosto	21.56810886	1	0.02848861	1	1	0	1.31848086	1.37392015	2.40905857	2.29893622
5572	**ENSG0000012538**	FAM193A	protein_coding	chr4	2536647	2732573	+	Human GRCh	OTTHUMG000001	FAM193A	**family with sequence similarity 193 member A**	Other	other		-1.287874542	0	0.21320281	0	1	0	11.683822	11.8996499	11.73355	11.2455316
5573	**ENSG0000012538**	GRK4	protein_coding	chr4	2963571	3040760	+	Human GRCh	OTTHUMG000001	GRK4	**G protein-coupled receptor kinase 4**	Plasma Mem	kinase		-1.051132362	0	0.83575213	0	1	0	8.26382175	8.58633924	8.4213371	8.33995765
5574	**ENSG0000012539**	SOX9	protein_coding	chr17	72121020	72126416	+	Human GRCh	OTTHUMG000001	SOX9	**SRY-box transcription factor 9**	Nucleus	transcription		1.51918468	1	0.45658346	0	1	0	8.96634626	9.17113017	9.61243809	9.50681887
5577	**ENSG0000012543**	HS3ST3B1	protein_coding	chr17	14301081	14349404	+	Human GRCh	OTTHUMG000000	HS3ST3B1	**heparan sulfate-glucosamine 3-sulfotransferase 3B1**	Cytoplasm	enzyme		1.05165527	0	0.84832379	0	1	0	8.82141432	8.86777913	9.12140379	8.64328757
5578	**ENSG0000012543**	SLC25A35	protein_coding	chr17	8287763	8295400	-	Human GRCh	OTTHUMG000001	SLC25A35	**solute carrier family 25 member 35**	Cytoplasm	other		-1.180948586	0	0.75067571	0	1	0	8.60991474	8.98826236	8.767555	8.48672392
5579	**ENSG0000012544**	MRPS7	protein_coding	chr17	75261674	75266376	+	Human GRCh	OTTHUMG000001	MRPS7	**mitochondrial ribosomal protein S7**	Cytoplasm	other		-1.113133026	0	0.61885237	0	1	0	10.0746306	10.3460807	10.1793581	10.0094063
5580	**ENSG0000012544**	GGA3	protein_coding	chr17	75236599	75262363	-	Human GRCh	OTTHUMG000001	GGA3	**golgi associated, gamma adaptin ear containing, ARF binding p**	Cytoplasm	transporter		-1.028815402	0	0.88706793	0	1	0	10.513206	10.8203158	10.7484313	10.5333866
5581	**ENSG0000012544**	ARMC7	protein_coding	chr17	75109952	75130272	+	Human GRCh	OTTHUMG000001	ARMC7	**armadillo repeat containing 7**	Cytoplasm	other		1.272865828	0	0.28603093	0	1	0	7.56258606	7.76473246	8.01388614	7.82205389
5582	**ENSG0000012545**	NUP85	protein_coding	chr17	75205659	75235758	+	Human GRCh	OTTHUMG000001	NUP85	**nucleoporin 85**	Cytoplasm	other		-1.171051183	0	0.43923156	0	1	0	10.7743452	11.1129188	10.9377007	10.600303
5583	**ENSG0000012545**	SLC25A19	protein_coding	chr17	75272981	75289510	-	Human GRCh	OTTHUMG000001	SLC25A19	**solute carrier family 25 member 19**	Cytoplasm	transporter		-1.014054809	0	0.9492413	0	1	0	9.35076397	9.77626095	9.71864655	9.39255805
5584	**ENSG0000012545**	MIF4GD	protein_coding	chr17	75266228	75271231	-	Human GRCh	OTTHUMG000001	MIF4GD	**MIF4G domain containing**	Other	other		-1.177595896	0	0.55337333	0	1	0	9.01980011	9.16831749	9.00330475	8.82074662
5585	**ENSG0000012545**	NT5C	protein_coding	chr17	75130225	75131757	-	Human GRCh	OTTHUMG000001	NT5C	**5', 3'-nucleotidase, cytosolic**	Cytoplasm	phosphatase		1.055260929	0	0.7970351	0	1	0	9.59693053	9.92705492	9.78087278	9.88287002
5586	**ENSG0000012545**	MSTO1	protein_coding	chr1	155563235	155614951	+	Human GRCh	OTTHUMG000000	MSTO1	**misato mitochondrial distribution and morphology regulator 1**	Cytoplasm	other		-1.139591771	0	0.52334498	0	1	0	9.90422691	10.2408806	10.0767664	9.78242166
5588	**ENSG0000012548**	TTF1	protein_coding	chr9	132375548	132406851	-	Human GRCh	OTTHUMG000000	TTF1	**transcription termination factor 1**	Nucleus	transcription		1.042059552	0	0.85758045	0	1	0	8.57996685	8.7841163	8.94032353	8.47429969
5589	**ENSG0000012548**	GTF3C4	protein_coding	chr9	132670035	132694953	+	Human GRCh	OTTHUMG000000	GTF3C4	**general transcription factor IIIC subunit 4**	Nucleus	transcription		-1.067450767	0	0.74545946	0	1	0	11.5330341	11.7985829	11.7752368	11.3969738
5590	**ENSG0000012548**	DDX31	protein_coding	chr9	132592997	132670401	-	Human GRCh	OTTHUMG000000	DDX31	**DEAD-box helicase 31**	Nucleus	enzyme		1.086798998	0	0.71150265	0	1	0	9.8152687	10.0998094	10.271796	9.79525673
5591	**ENSG0000012549**	BARHL1	protein_coding	chr9	132582606	132590252	+	Human GRCh	OTTHUMG000000	BARHL1	**BarH like homeobox 1**	Nucleus	transcription		1	0	1	0	1	0	0.78921651	0.89204644	0.87574314	0.79554597
5593	**ENSG0000012550**	PPP1R12C	protein_coding	chr19	55090914	55117637	-	Human GRCh	OTTHUMG000001	PPP1R12C	**protein phosphatase 1 regulatory subunit 12C**	Cytoplasm	phosphatase		1.182609793	0	0.46279741	0	1	0	10.8571911	11.0146411	11.2754966	10.9502873
5594	**ENSG0000012550**	MBOAT7	protein_coding	chr19	54173412	54189882	-	Human GRCh	OTTHUMG000000	MBOAT7	**membrane bound O-acyltransferase domain containing 7**	Plasma Mem	enzyme		-1.15054161	0	0.45387199	0	1	0	11.589877	11.9375813	11.6681762	11.5616918

5595	**ENSG0000012550**	SRMS	protein_coding	chr20	63538489	63547749	-	Human GRCh	OTTHUMG000000	SRMS	**src-related kinase lacking C-terminal regulatory tyrosine and N**	Cytoplasm	kinase	binimetinil	1.241391642	0	0.62141561	0	1	0	8.46050101	8.82430007	9.14397618	8.62016631
5596	**ENSG0000012551**	OPRL1	protein_coding	chr20	64080082	64100643	+	Human GRCh	OTTHUMG000000	OPRL1	**opioid related nociceptin receptor 1**	Plasma Mem	G-protein cou	ZP120, bup	-1.214760777	0	0.7119286	0	1	0	3.84850471	4.56801516	3.9813354	4.21436783
5598	**ENSG0000012552**	SLC2A4RG	protein_coding	chr20	63739776	63744050	+	Human GRCh	OTTHUMG000000	SLC2A4RG	**SLC2A4 regulator**	Cytoplasm	transcription		1.099916923	0	0.71018281	0	1	0	10.8209398	11.1410086	11.2082817	10.9722674
5600	**ENSG0000012553**	FNDC11	protein_coding	chr20	63547891	63556695	+	Human GRCh	OTTHUMG000000	FNDC11	**fibronectin type III domain containing 11**	Cytoplasm	other		-2.249678258	-1	0.26332955	0	1	0	5.13003166	5.62204288	4.78235197	4.40500364
5602	**ENSG0000012553**	PPDPF	protein_coding	chr20	63520765	63522206	+	Human GRCh	OTTHUMG000000	PPDPF	**pancreatic progenitor cell differentiation and proliferation fact**	Other	other		1.202799035	0	0.34791159	0	1	0	11.3413339	11.5725096	11.7596417	11.5625503
5604	**ENSG0000012555**	PLGLB2	protein_coding	chr2	87748087	87759476	+	Human GRCh	OTTHUMG000001	PLGLB1/PLGL	**plasminogen like B2**	Extracellular	peptidase		1.030038665	0	0.96444365	0	1	0	3.79133619	5.01426546	4.69248807	4.4850076
5605	**ENSG0000012557**	IL37	protein_coding	chr2	112911165	112918882	+	Human GRCh	OTTHUMG000001	IL37	**interleukin 37**	Extracellular	cytokine		-1.689680819	-1	0.39027907	0	1	0	4.22503502	4.26511139	3.7915924	3.86890606
5606	**ENSG0000012561**	CHCHD5	protein_coding	chr2	112584240	112589275	+	Human GRCh	OTTHUMG000001	CHCHD5	**coiled-coil-helix-coiled-coil-helix domain containing 5**	Other	other		-1.055637971	0	0.82215654	0	1	0	9.28485141	9.66477479	9.46014905	9.39536784
5607	**ENSG0000012561**	PAX8	protein_coding	chr2	113215997	113278921	-	Human GRCh	OTTHUMG000001	PAX8	**paired box 8**	Nucleus	transcription		-1.114677676	0	0.75158268	0	1	0	6.98439152	7.13175156	7.10078752	6.75313478
5608	**ENSG0000012562**	INSIG2	protein_coding	chr2	118088452	118110997	+	Human GRCh	OTTHUMG000000	INSIG2	**insulin induced gene 2**	Cytoplasm	other		1.013551968	0	0.96202674	0	1	0	8.19168378	8.66921926	8.74339846	8.10920698
5609	**ENSG0000012563**	POLR1B	protein_coding	chr2	112541915	112579818	+	Human GRCh	OTTHUMG000001	POLR1B	**RNA polymerase I subunit B**	Nucleus	enzyme		-1.024196781	0	0.91168684	0	1	0	10.8622722	11.1891391	11.2137039	10.7636463
5610	**ENSG0000012563**	CCDC93	protein_coding	chr2	117915478	118014133	-	Human GRCh	OTTHUMG000000	CCDC93	**coiled-coil domain containing 93**	Extracellular	other		-1.177412252	0	0.43805234	0	1	0	9.96790183	10.25628	10.1070391	9.75659516
5611	**ENSG0000012563**	PSD4	protein_coding	chr2	113157325	113209396	+	Human GRCh	OTTHUMG000001	PSD4	**pleckstrin and Sec7 domain containing 4**	Cytoplasm	other		-1.21620362	0	0.39614439	0	1	0	9.83282928	10.0978418	9.74195825	9.77715347
5612	**ENSG0000012564**	SLC25A23	protein_coding	chr19	6436079	6465203	-	Human GRCh	OTTHUMG000001	SLC25A23	**solute carrier family 25 member 23**	Cytoplasm	transporter		-1.042115311	0	0.84251433	0	1	0	11.0603479	11.4725029	11.2957371	11.173744
5613	**ENSG0000012565**	PSPN	protein_coding	chr19	6375148	6379058	-	Human GRCh	OTTHUMG000001	PSPN	**persephin**	Extracellular	growth facto		-1.144154696	0	0.65886229	0	1	0	6.77938776	6.47201987	6.62776907	6.36038335
5614	**ENSG0000012565**	GTF2F1	protein_coding	chr19	6379572	6393981	-	Human GRCh	OTTHUMG000001	GTF2F1	**general transcription factor IIF subunit 1**	Nucleus	transcription		-1.028271277	0	0.88857957	0	1	0	11.1603768	11.4564467	11.3643165	11.2023094
5615	**ENSG0000012565**	ALKBH7	protein_coding	chr19	6372794	6375250	+	Human GRCh	OTTHUMG000001	ALKBH7	**alkB homolog 7**	Cytoplasm	other		1.058661883	0	0.79091397	0	1	0	9.33879468	9.49879959	9.6247478	9.32650791
5616	**ENSG0000012565**	CLPP	protein_coding	chr19	6361531	6370242	+	Human GRCh	OTTHUMG000001	CLPP	**caseinolytic mitochondrial matrix peptidase proteolytic subun**	Cytoplasm	peptidase	ONC206	1.044060735	0	0.8418887	0	1	0	10.5479411	11.019912	10.8292433	10.8819758
5617	**ENSG0000012565**	TNFSF9	protein_coding	chr19	6531026	6535924	+	Human GRCh	OTTHUMG000001	TNFSF9	**TNF superfamily member 9**	Plasma Mem	cytokine		1.168850735	0	0.78135904	0	1	0	6.32934846	5.63721789	6.13320846	6.27701658
5619	**ENSG0000012567**	THOC2	protein_coding	chrX	123600561	123733056	-	Human GRCh	OTTHUMG000000	THOC2	**THO complex subunit 2**	Nucleus	other		-1.040620333	0	0.84830865	0	1	0	11.8079629	12.0189211	12.0268518	11.7010618
5620	**ENSG0000012568**	MED1	protein_coding	chr17	39404285	39451272	-	Human GRCh	OTTHUMG000001	MED1	**mediator complex subunit 1**	Nucleus	transcription		-1.115147813	0	0.56659534	0	1	0	11.9889728	12.3241238	12.1372731	11.9482757
5621	**ENSG0000012569**	RPL23	protein_coding	chr17	38847860	38853764	-	Human GRCh	OTTHUMG000001	RPL23	**ribosomal protein L23**	Cytoplasm	other		-1.067724655	0	0.73771008	0	1	0	13.7828696	14.0172783	13.9291294	13.7239953
5622	**ENSG0000012570**	ATG4C	protein_coding	chr1	62784132	62865516	+	Human GRCh	OTTHUMG000000	ATG4C	**autophagy related 4C cysteine peptidase**	Cytoplasm	peptidase		-1.032506895	0	0.90000794	0	1	0	7.89624613	8.15673757	8.19659049	7.76293379
5624	**ENSG0000012573**	C3	protein_coding	chr19	6677704	6730562	-	Human GRCh	OTTHUMG000001	C3	**complement C3**	Extracellular	peptidase	efdamrofu	-2.322955952	-1	0.35998463	0	NA	0	3.54302255	3.3612938	2.49178262	3.05917479
5625	**ENSG0000012573**	SH2D3A	protein_coding	chr19	6752160	6767463	-	Human GRCh	OTTHUMG000001	SH2D3A	**SH2 domain containing 3A**	Cytoplasm	other		-1.171865163	0	0.50754727	0	1	0	9.72453591	9.89233722	9.6410029	9.62053128
5626	**ENSG0000012573**	TRIP10	protein_coding	chr19	6737925	6751530	+	Human GRCh	OTTHUMG000001	TRIP10	**thyroid hormone receptor interactor 10**	Cytoplasm	other		-1.012940847	0	0.95345383	0	1	0	8.85545656	9.33384581	9.12191525	9.08652513
5627	**ENSG0000012573**	GPR108	protein_coding	chr19	6729914	6737580	-	Human GRCh	OTTHUMG000001	GPR108	**G protein-coupled receptor 108**	Plasma Mem	G-protein cou		-1.030898879	0	0.88473226	0	1	0	9.78676454	9.99118023	9.9490148	9.76352348
5629	**ENSG0000012574**	FOSB	protein_coding	chr19	45467995	45475179	+	Human GRCh	OTTHUMG000001	FOSB	**FosB proto-oncogene, AP-1 transcription factor subunit**	Nucleus	transcription		1.341376051	0	0.39598547	0	1	0	8.05496683	7.71364649	8.31460586	8.1407391
5630	**ENSG0000012574**	OPA3	protein_coding	chr19	45527767	45602212	-	Human GRCh	OTTHUMG000001	OPA3	**outer mitochondrial membrane lipid metabolism regulator OP**	Cytoplasm	other		-1.090319067	0	0.67747363	0	1	0	10.0570585	10.3562749	10.2289928	9.999017
5631	**ENSG0000012574**	SNRPD2	protein_coding	chr19	45687460	45692569	-	Human GRCh	OTTHUMG000001	SNRPD2	**small nuclear ribonucleoprotein D2 polypeptide**	Nucleus	other		1.030986699	0	0.87898782	0	1	0	10.7775763	11.1151533	11.0771107	10.9017861
5632	**ENSG0000012574**	RTN2	protein_coding	chr19	45485294	45497055	-	Human GRCh	OTTHUMG000001	RTN2	**reticulon 2**	Cytoplasm	other		-1.116390867	0	0.7152198	0	1	0	8.26472126	8.64627436	8.40350377	8.29741305
5633	**ENSG0000012574**	EML2	protein_coding	chr19	45606994	45645602	-	Human GRCh	OTTHUMG000001	EML2	**EMAP like 2**	Cytoplasm	other		1.153657834	0	0.46258955	0	1	0	9.41795248	9.46435921	9.64606844	9.55699224
5634	**ENSG0000012575**	VASP	protein_coding	chr19	45506579	45526989	+	Human GRCh	OTTHUMG000001	VASP	**vasodilator stimulated phosphoprotein**	Plasma Mem	other		1.079806111	0	0.71908697	0	1	0	9.71689298	10.1199678	10.0625049	9.97827256
5635	**ENSG0000012575**	SYMPK	protein_coding	chr19	45815410	45863194	-	Human GRCh	OTTHUMG000001	SYMPK	**symplekin scaffold protein**	Cytoplasm	other		-1.118211202	0	0.55371884	0	1	0	12.4405523	12.718805	12.5427207	12.3744963
5636	**ENSG0000012577**	GPCPD1	protein_coding	chr20	5544439	5611006	-	Human GRCh	OTTHUMG000000	GPCPD1	**glycerophosphocholine phosphodiesterase 1**	Cytoplasm	enzyme		1.102183806	0	0.7819753	0	1	0	9.92222858	10.1487929	10.2612461	10.0013018
5637	**ENSG0000012577**	SDCBP2	protein_coding	chr20	1309909	1329139	-	Human GRCh	OTTHUMG000000	SDCBP2	**syndecan binding protein 2**	Cytoplasm	other		-1.06476344	0	0.87227965	0	1	0	6.75812018	6.82163166	6.2260791	7.06700439
5638	**ENSG0000012577**	PANK2	protein_coding	chr20	3888839	3929887	+	Human GRCh	OTTHUMG000000	PANK2	**pantothenate kinase 2**	Cytoplasm	kinase		-1.197406478	0	0.43126056	0	1	0	10.956074	11.2188031	11.0323945	10.7441128
5640	**ENSG0000012578**	GNRH2	protein_coding	chr20	3043622	3045747	+	Human GRCh	OTTHUMG000000	GNRH2	**gonadotropin releasing hormone 2**	Extracellular	other		-1.494083065	0	0.43752239	0	1	0	5.20459358	5.24053599	5.11951461	4.51724723
5642	**ENSG0000012579**	FOXA2	protein_coding	chr20	22580998	22585455	-	Human GRCh	OTTHUMG000001	FOXA2	**forkhead box A2**	Nucleus	transcription		-1.496844775	0	0.58330028	0	1	0	3.15724555	1.8927314	1.87378284	2.86341029
5645	**ENSG0000012581**	GZF1	protein_coding	chr20	23362182	23373062	+	Human GRCh	OTTHUMG000000	GZF1	**GDNF inducible zinc finger protein 1**	Nucleus	transcription		1.09106647	0	0.69919134	0	1	0	9.20028061	9.35219896	9.51148667	9.22085281
5646	**ENSG0000012581**	PAX1	protein_coding	chr20	21705659	21718481	+	Human GRCh	OTTHUMG000000	PAX1	**paired box 1**	Nucleus	transcription		-1.933413565	-1	0.63390081	0	1	0	8.25847629	8.47349625	7.73586039	7.41083879
5647	**ENSG0000012581**	NAPB	protein_coding	chr20	23374519	23421519	-	Human GRCh	OTTHUMG000000	NAPB	**NSF attachment protein beta**	Cytoplasm	transporter		1.034515977	0	0.88566369	0	1	0	9.06123739	9.15200679	9.36174642	8.87675245
5649	**ENSG0000012581**	NKX2-4	protein_coding	chr20	21395365	21397526	-	Human GRCh	OTTHUMG000000	NKX2-4	**NK2 homeobox 4**	Nucleus	transcription		1.257679696	0	0.74496151	0	1	0	3.66967873	3.99623038	4.17330631	3.87273236
5650	**ENSG0000012581**	CENPB	protein_coding	chr20	3783851	3786740	-	Human GRCh	OTTHUMG000000	CENPB	**centromere protein B**	Nucleus	other		1.088147816	0	0.65506548	0	1	0	11.2885691	11.4549707	11.5326465	11.3990454
5651	**ENSG0000012581**	PSMF1	protein_coding	chr20	1113240	1189415	+	Human GRCh	OTTHUMG000000	PSMF1	**proteasome inhibitor subunit 1**	Cytoplasm	other		-1.089655448	0	0.67229348	0	1	0	11.3948005	11.7505514	11.5635083	11.4123661
5652	**ENSG0000012582**	NKX2-2	protein_coding	chr20	21511017	21514064	-	Human GRCh	OTTHUMG000001	NKX2-2	**NK2 homeobox 2**	Nucleus	transcription		-5.209978544	-1	0.01008512	1	1	0	5.28099663	3.77839905	3.85713501	3.10634013
5653	**ENSG0000012582**	DTD1	protein_coding	chr20	18587942	18766644	+	Human GRCh	OTTHUMG000000	DTD1	**D-aminoacyl-tRNA deacylase 1**	Cytoplasm	enzyme		1.015466312	0	0.94652852	0	1	0	9.60349462	9.91407384	9.93567522	9.61382002
5654	**ENSG0000012582**	CSTL1	protein_coding	chr20	23426104	23444930	+	Human GRCh	OTTHUMG000000	CSTL1	**cystatin like 1**	Extracellular	other		2.66259496	1	0.55395502	0	1	0	1.04694637	1.09229829	1.08490543	1.39548515
5655	**ENSG0000012582**	RBCK1	protein_coding	chr20	407498	432139	+	Human GRCh	OTTHUMG000000	RBCK1	**RANBP2-type and C3HC4-type zinc finger containing 1**	Cytoplasm	transcription		-1.007934912	0	0.96963097	0	1	0	10.7461113	10.919365	10.9356633	10.7074557
5656	**ENSG0000012582**	TMX4	protein_coding	chr20	7977346	8019805	-	Human GRCh	OTTHUMG000000	TMX4	**thioredoxin related transmembrane protein 4**	Cytoplasm	enzyme		-1.153337298	0	0.68268305	0	1	0	10.0557071	10.2966151	10.0883272	9.97123285
5658	**ENSG0000012583**	STK35	protein_coding	chr20	2101827	2177038	+	Human GRCh	OTTHUMG000000	STK35	**serine/threonine kinase 35**	Cytoplasm	kinase		-1.175590309	0	0.39444558	0	1	0	11.6877187	11.9542513	11.6720079	11.6212691
5659	**ENSG0000012583**	SNRPB	protein_coding	chr20	2461634	2470853	-	Human GRCh	OTTHUMG000000	SNRPB	**small nuclear ribonucleoprotein polypeptides B and B1**	Nucleus	other		1.039069449	0	0.85853034	0	1	0	11.6919061	12.0558235	12.0536454	11.7931045
5660	**ENSG0000012584**	NRSN2	protein_coding	chr20	346782	359660	+	Human GRCh	OTTHUMG000000	NRSN2	**neurensin 2**	Plasma Mem	other		1.06744196	0	0.79143894	0	1	0	9.92853625	10.4162089	10.4232279	10.0973238
5661	**ENSG0000012584**	AP5S1	protein_coding	chr20	3820524	3828838	+	Human GRCh	OTTHUMG000000	AP5S1	**adaptor related protein complex 5 subunit sigma 1**	Cytoplasm	other		1.167861448	0	0.46018229	0	1	0	8.46128064	8.51362284	8.65094328	8.65157733
5662	**ENSG0000012584**	RRBP1	protein_coding	chr20	17613678	17682295	-	Human GRCh	OTTHUMG000000	RRBP1	**ribosome binding protein 1**	Cytoplasm	other		1.000147745	0	0.99955446	0	1	0	12.5446084	12.7966694	12.8558031	12.4675825
5663	**ENSG0000012584**	BMP2	protein_coding	chr20	6767686	6780246	+	Human GRCh	OTTHUMG000000	BMP2	**bone morphogenetic protein 2**	Extracellular	growth facto		5.276955563	1	0.21369805	0	1	0	2.21843914	2.28127741	2.27101642	2.70629308
5664	**ENSG0000012584**	ZNF133	protein_coding	chr20	18288283	18316996	+	Human GRCh	OTTHUMG000000	ZNF133	**zinc finger protein 133**	Nucleus	transcription		-1.07357801	0	0.76350713	0	1	0	8.62061463	8.9471679	8.96882485	8.4015827
5665	**ENSG0000012584**	FLRT3	protein_coding	chr20	14322985	14337614	-	Human GRCh	OTTHUMG000000	FLRT3	**fibronectin leucine rich transmembrane protein 3**	Plasma Mem	other		1.523727241	1	0.19896588	0	1	0	5.68565268	5.69500313	6.17825098	6.11821198
5666	**ENSG0000012585**	OVOL2	protein_coding	chr20	17956979	18059188	-	Human GRCh	OTTHUMG000000	OVOL2	**ovo like zinc finger 2**	Nucleus	transcription		-1.286745928	0	0.26601001	0	1	0	9.25868829	9.56218253	9.31442087	8.94246443
5667	**ENSG0000012585**	PCSK2	protein_coding	chr20	17226107	17484578	+	Human GRCh	OTTHUMG000000	PCSK2	**proprotein convertase subtilisin/kexin type 2**	Extracellular	peptidase		-16.08206349	-1	0.06477123	0	1	0	3.34552882	3.13830319	2.56262287	2.48227191
5668	**ENSG0000012586**	GFRA4	protein_coding	chr20	3659248	3663399	-	Human GRCh	OTTHUMG000000	GFRA4	**GDNF family receptor alpha 4**	Plasma Mem	transmembra		1	0	1	0	1	0	1.31879233	1.38373856	1.3732102	1.322688
5669	**ENSG0000012586**	MKKS	protein_coding	chr20	10401009	10434222	-	Human GRCh	OTTHUMG000001	MKKS	**MKKS centrosomal shuttling protein**	Cytoplasm	other		-1.17066083	0	0.45722051	0	1	0	9.31685398	9.46245823	9.34256467	9.07868897
5670	**ENSG0000012586**	BFSP1	protein_coding	chr20	17493905	17569220	-	Human GRCh	OTTHUMG000000	BFSP1	**beaded filament structural protein 1**	Cytoplasm	enzyme		-1.055804417	0	0.88141829	0	1	0	5.5520017	6.36943406	6.26978111	5.58563793
5671	**ENSG0000012586**	DSTN	protein_coding	chr20	17570075	17609919	+	Human GRCh	OTTHUMG000000	DSTN	**destrin, actin depolymerizing factor**	Cytoplasm	other		1.114643723	0	0.62351257	0	1	0	12.1391918	12.4463695	12.61657	12.1997488
5673	**ENSG0000012587**	SNRPB2	protein_coding	chr20	16729961	16742564	+	Human GRCh	OTTHUMG000000	SNRPB2	**small nuclear ribonucleoprotein polypeptide B2**	Nucleus	other		-1.220351483	0	0.36195886	0	1	0	9.61358479	9.9837537	9.71813123	9.45234239
5674	**ENSG0000012587**	MGME1	protein_coding	chr20	17969018	17991122	+	Human GRCh	OTTHUMG000000	MGME1	**mitochondrial genome maintenance exonuclease 1**	Cytoplasm	enzyme		-1.172174766	0	0.48324992	0	1	0	10.8190669	11.0584525	10.9403362	10.5603756
5675	**ENSG0000012587**	LRRN4	protein_coding	chr20	6040546	6054060	-	Human GRCh	OTTHUMG000000	LRRN4	**leucine rich repeat neuronal 4**	Plasma Mem	other		1	0	1	0	NA	0	0.7536691	0.77335228	0.77009424	0.7548217
5676	**ENSG0000012587**	TBC1D20	protein_coding	chr20	423596	456266	-	Human GRCh	OTTHUMG000000	TBC1D20	**TBC1 domain family member 20**	Nucleus	other		-1.130442829	0	0.61124734	0	1	0	10.8322277	11.2515308	11.0678862	10.7511897
5677	**ENSG0000012587**	ITPA	protein_coding	chr20	3208868	3223870	+	Human GRCh	OTTHUMG000000	ITPA	**inosine triphosphatase**	Cytoplasm	enzyme		1.072096632	0	0.74620088	0	1	0	9.74804429	10.1264697	10.1785059	9.86118457
5678	**ENSG0000012587**	TCF15	protein_coding	chr20	604257	610309	-	Human GRCh	OTTHUMG000000	TCF15	**transcription factor 15**	Nucleus	transcription		3.551650182	1	0.25983476	0	NA	0	1.78655385	2.60125915	3.19490328	3.14946366
5680	**ENSG0000012588**	MCM8	protein_coding	chr20	5950652	5998977	+	Human GRCh	OTTHUMG000000	MCM8	**minichromosome maintenance 8 homologous recombination r**	Nucleus	enzyme		-1.091436189	0	0.68464921	0	1	0	10.9385746	11.3040206	11.1475045	10.9210993
5682	**ENSG0000012589**	TMEM74B	protein_coding	chr20	1180561	1185415	-	Human GRCh	OTTHUMG000000	TMEM74B	**transmembrane protein 74B**	Other	other		1.091842403	0	0.81141995	0	1	0	5.97118854	6.63545616	6.37638823	6.49534259
5683	**ENSG0000012589**	FAM110A	protein_coding	chr20	833715	857463	+	Human GRCh	OTTHUMG000000	FAM110A	**family with sequence similarity 110 member A**	Cytoplasm	other		-1.249454248	0	0.34562113	0	1	0	7.8412443	8.14166504	7.56011065	7.9423845
5686	**ENSG0000012590**	MRPS26	protein_coding	chr20	3046052	3048250	+	Human GRCh	OTTHUMG000000	MRPS26	**mitochondrial ribosomal protein S26**	Cytoplasm	other		1.117254607	0	0.57419894	0	1	0	10.1521883	10.3702116	10.480001	10.2922316
5689	**ENSG0000012591**	NCLN	protein_coding	chr19	3185563	3209575	+	Human GRCh	OTTHUMG000001	NCLN	**nicalin**	Cytoplasm	peptidase		1.017978368	0	0.93197268	0	1	0	11.3881718	11.6814488	11.6879817	11.4255203
5690	**ENSG0000012593**	CITED1	protein_coding	chrX	72301638	72307187	-	Human GRCh	OTTHUMG000000	CITED1	**Cbp/p300 interacting transactivator with Glu/Asp rich carboxy**	Nucleus	transcription		1.49974581	0	0.47888022	0	1	0	4.93725278	4.82826813	5.27879834	5.22873808
5691	**ENSG0000012594**	HNRNPR	protein_coding	chr1	23303771	23344336	-	Human GRCh	OTTHUMG000000	HNRNPR	**heterogeneous nuclear ribonucleoprotein R**	Nucleus	other		1.058382532	0	0.78420272	0	1	0	12.327273	12.6166525	12.7065535	12.3537946
5692	**ENSG0000012594**	ZNF436	protein_coding	chr1	23359446	23369836	-	Human GRCh	OTTHUMG000000	ZNF436	**zinc finger protein 436**	Nucleus	transcription		-1.319154006	0	0.23678297	0	1	0	8.77412965	9.01169664	8.77274571	8.39691378
5693	**ENSG0000012595**	MAX	protein_coding	chr14	65006174	65102695	-	Human GRCh	OTTHUMG000001	MAX	**MYC associated factor X**	Nucleus	transcription		1.026516718	0	0.90024163	0	1	0	10.5979367	10.8370421	10.8544868	10.6413193
5694	**ENSG0000012599**	ARMCX5	protein_coding	chrX	102599168	102604159	+	Human GRCh	OTTHUMG000000	ARMCX5	**armadillo repeat containing X-linked 5**	Other	other		-1.047964842	0	0.85478319	0	1	0	8.43253019	8.73004647	8.65226313	8.41427671
5695	**ENSG0000012596**	GDF5	protein_coding	chr20	35433347	35454746	-	Human GRCh	OTTHUMG000000	GDF5	**growth differentiation factor 5**	Extracellular	growth facto		1.66615352	1	0.58315997	0	NA	0	2.5049882	2.59559135	3.07144579	2.40447816
5696	**ENSG0000012596**	MMP24	protein_coding	chr20	35226690	35276998	+	Human GRCh	OTTHUMG000000	MMP24	**matrix metallopeptidase 24**	Plasma Mem	peptidase	marimasta	-4.077296165	-1	0.10345081	0	1	0	3.22558834	3.38840781	2.40481044	2.87288309
5697	**ENSG0000012596**	NECAB3	protein_coding	chr20	33657087	33674463	-	Human GRCh	OTTHUMG000000	NECAB3	**N-terminal EF-hand calcium binding protein 3**	Cytoplasm	other		-1.009935116	0	0.96301026	0	1	0	11.1616674	11.4186145	11.426565	11.1266422

5698	**ENSG0000012596**	ID1	protein_coding	chr20	31605283	31606515	+	Human GRCh	OTTHUMG000000	ID1	**inhibitor of DNA binding 1**	Nucleus	transcription		1.784028153	1	0.35573118	0	1	0	6.93778744	7.01902934	7.26914684	7.89275633
5699	**ENSG0000012597**	RALY	protein_coding	chr20	33993646	34108308	+	Human GRCh	OTTHUMG000000	RALY	**RALY heterogeneous nuclear ribonucleoprotein**	Nucleus	transcription		1.064516248	0	0.750098	0	1	0	11.7563133	12.1195842	12.1193293	11.9129073
5700	**ENSG0000012597**	DYNLRB1	protein_coding	chr20	34516395	34540958	+	Human GRCh	OTTHUMG000000	DYNLRB1	**dynein light chain roadblock-type 1**	Cytoplasm	other		-1.030890515	0	0.87199539	0	1	0	10.4629635	10.7378996	10.5812758	10.5681344
5702	**ENSG0000012597**	EIF2S2	protein_coding	chr20	34088309	34112243	-	Human GRCh	OTTHUMG000000	EIF2S2	**eukaryotic translation initiation factor 2 subunit beta**	Cytoplasm	translation re		-1.191517821	0	0.38369829	0	1	0	10.9872657	11.1481193	10.9610955	10.7825689
5703	**ENSG0000012599**	ERGIC3	protein_coding	chr20	35542038	35557634	+	Human GRCh	OTTHUMG000000	ERGIC3	**ERGIC and golgi 3**	Cytoplasm	other		-1.061115645	0	0.77505464	0	1	0	11.4681544	11.729653	11.6500055	11.4196092
5704	**ENSG0000012599**	ROMO1	protein_coding	chr20	35699272	35700984	+	Human GRCh	OTTHUMG000000	ROMO1	**reactive oxygen species modulator 1**	Cytoplasm	other		1.070786844	0	0.78863487	0	1	0	8.75720836	9.29471662	9.13837981	9.12594187
5707	**ENSG0000012600**	CEP250	protein_coding	chr20	35455164	35519280	+	Human GRCh	OTTHUMG000000	CEP250	**centrosomal protein 250**	Nucleus	other		-1.058444212	0	0.78749393	0	1	0	11.5897459	11.9590936	11.8571107	11.5811402
5708	**ENSG0000012600**	PLAGL2	protein_coding	chr20	32192504	32207743	-	Human GRCh	OTTHUMG000000	PLAGL2	**PLAG1 like zinc finger 2**	Nucleus	transcription		-1.128599751	0	0.62711314	0	1	0	10.5591941	10.7630257	10.7028446	10.3351631
5709	**ENSG0000012600**	MMP24OS	protein_coding	chr20	35201745	35278131	-	Human GRCh	OTTHUMG000000	MMP24OS	**MMP24 opposite strand**	Other	other		1.142885829	0	0.57840041	0	1	0	10.2062584	10.6220136	10.5709566	10.591136
5710	**ENSG0000012601**	GRPR	protein_coding	chrX	16123565	16153518	+	Human GRCh	OTTHUMG000000	GRPR	**gastrin releasing peptide receptor**	Plasma Mem	G-protein cou	lutetium Lu	1.700505382	1	0.47926063	0	1	0	3.0957162	3.00485031	3.36587732	3.33549641
5711	**ENSG0000012601**	KDM5C	protein_coding	chrX	53176283	53225422	-	Human GRCh	OTTHUMG000000	KDM5C	**lysine demethylase 5C**	Nucleus	enzyme		-1.069816299	0	0.72500971	0	1	0	12.024408	12.2228794	12.1603451	11.9357624
5712	**ENSG0000012601**	AMOT	protein_coding	chrX	112774503	112840831	-	Human GRCh	OTTHUMG000000	AMOT	**angiomotin**	Plasma Mem	other		-1.516225538	-1	0.15105697	0	1	0	8.50811491	8.44436971	8.04384945	8.05496459
5713	**ENSG0000012606**	TMEM115	protein_coding	chr3	50354750	50359521	-	Human GRCh	OTTHUMG000000	TMEM115	**transmembrane protein 115**	Cytoplasm	other		-1.008674585	0	0.96593134	0	1	0	9.82826375	9.94698686	9.97395466	9.77690563
5714	**ENSG0000012606**	PSMB2	protein_coding	chr1	35599541	35641526	-	Human GRCh	OTTHUMG000000	PSMB2	**proteasome 20S subunit beta 2**	Cytoplasm	peptidase	bortezomi	-1.208838791	0	0.3220264	0	1	0	11.7441761	12.1174222	11.7682923	11.702531
5715	**ENSG0000012607**	AGO3	protein_coding	chr1	35930718	36072500	+	Human GRCh	OTTHUMG000001	AGO3	**argonaute RISC catalytic component 3**	Cytoplasm	translation re		-1.006558489	0	0.97596212	0	1	0	11.7651428	12.0401302	12.1192698	11.6386595
5716	**ENSG0000012608**	UROD	protein_coding	chr1	45010950	45015575	+	Human GRCh	OTTHUMG000000	UROD	**uroporphyrinogen decarboxylase**	Cytoplasm	enzyme		-1.087935816	0	0.65763587	0	1	0	9.488285	9.64658654	9.4573867	9.49614517
5717	**ENSG0000012609**	ST3GAL3	protein_coding	chr1	43705824	43931165	+	Human GRCh	OTTHUMG000000	ST3GAL3	**ST3 beta-galactoside alpha-2,3-sialyltransferase 3**	Cytoplasm	enzyme		-1.077501182	0	0.75526368	0	1	0	9.53324235	9.52654933	9.6801479	9.16519688
5718	**ENSG0000012610**	TMEM53	protein_coding	chr1	44635238	44674481	-	Human GRCh	OTTHUMG000000	TMEM53	**transmembrane protein 53**	Nucleus	other		-1.175723458	0	0.5753585	0	1	0	6.50601101	6.8518272	6.20915272	6.77218731
5719	**ENSG0000012610**	HECTD3	protein_coding	chr1	45002540	45011324	-	Human GRCh	OTTHUMG000000	HECTD3	**HECT domain E3 ubiquitin protein ligase 3**	Cytoplasm	enzyme		-1.204714107	0	0.38330622	0	1	0	10.6112282	11.0007136	10.6694217	10.5540509
5720	**ENSG0000012621**	KLC1	protein_coding	chr14	103561896	103714249	+	Human GRCh	OTTHUMG000001	KLC1	**kinesin light chain 1**	Cytoplasm	other		-1.194665694	0	0.39291984	0	1	0	12.1069167	12.340335	12.1855774	11.8631319
5721	**ENSG0000012621**	XRCC3	protein_coding	chr14	103697609	103715504	-	Human GRCh	OTTHUMG000001	XRCC3	**X-ray repair cross complementing 3**	Nucleus	enzyme		-1.015165386	0	0.93817431	0	1	0	10.3907937	10.6265757	10.6312376	10.3475112
5722	**ENSG0000012621**	TUBGCP3	protein_coding	chr13	112485011	112588205	-	Human GRCh	OTTHUMG000000	TUBGCP3	**tubulin gamma complex component 3**	Cytoplasm	other		-1.124964392	0	0.56646938	0	1	0	11.0389483	11.2818223	11.1618178	10.9025928
5723	**ENSG0000012621**	MCF2L	protein_coding	chr13	112894378	113099742	+	Human GRCh	OTTHUMG000000	MCF2L	**MCF.2 cell line derived transforming sequence like**	Cytoplasm	other		-1.300002459	0	0.30365167	0	1	0	10.6684394	10.8529947	10.6650208	10.2667736
5725	**ENSG0000012622**	PCID2	protein_coding	chr13	113177536	113208715	-	Human GRCh	OTTHUMG000000	PCID2	**PCI domain containing 2**	Nucleus	transcription		-1.174428431	0	0.42270724	0	1	0	10.6397834	10.9392615	10.7626776	10.4626401
5728	**ENSG0000012624**	LRFN3	protein_coding	chr19	35935358	35946624	+	Human GRCh	OTTHUMG000001	LRFN3	**leucine rich repeat and fibronectin type III domain containing**	Plasma Mem	other		1.042273195	0	0.84875296	0	1	0	8.84722785	9.01316461	9.09136089	8.85295982
5729	**ENSG0000012624**	IGFLR1	protein_coding	chr19	35738801	35742453	-	Human GRCh	OTTHUMG000001	IGFLR1	**IGF like family receptor 1**	Plasma Mem	other		-1.176265315	0	0.60636924	0	1	0	7.23493541	7.63619709	7.16985443	7.37728891
5730	**ENSG0000012624**	CAPNS1	protein_coding	chr19	36139953	36150353	+	Human GRCh	OTTHUMG000001	CAPNS1	**calpain small subunit 1**	Cytoplasm	peptidase		-1.085279043	0	0.75639262	0	1	0	12.3674965	12.7789143	12.5155259	12.4819563
5731	**ENSG0000012624**	PDCD2L	protein_coding	chr19	34404399	34426168	+	Human GRCh	OTTHUMG000001	PDCD2L	**programmed cell death 2 like**	Other	other		1.112865906	0	0.67335648	0	1	0	8.54418354	8.86924801	9.02278514	8.61488932
5733	**ENSG0000012625**	RBM42	protein_coding	chr19	35629030	35637686	+	Human GRCh	OTTHUMG000001	RBM42	**RNA binding motif protein 42**	Nucleus	other		1.001957186	0	0.99269801	0	1	0	10.7405368	10.9739783	10.9030375	10.8259619
5734	**ENSG0000012625**	KIRREL2	protein_coding	chr19	35855861	35867136	+	Human GRCh	OTTHUMG000001	KIRREL2	**kirre like nephrin family adhesion molecule 2**	Plasma Mem	other		-1.275499208	0	0.73580718	0	1	0	4.36766875	4.46458048	4.27087946	4.12574876
5735	**ENSG0000012626**	UBA2	protein_coding	chr19	34428352	34471251	+	Human GRCh	OTTHUMG000001	UBA2	**ubiquitin like modifier activating enzyme 2**	Cytoplasm	enzyme		-1.039801799	0	0.83990934	0	1	0	11.1959958	11.3840451	11.4290767	11.042619
5736	**ENSG0000012626**	FFAR2	protein_coding	chr19	35443907	35451767	+	Human GRCh	OTTHUMG000001	FFAR2	**free fatty acid receptor 2**	Plasma Mem	G-protein cou		1	0	1	0	NA	0	0.466872	0.49078267	0.48686115	0.46828694
5739	**ENSG0000012626**	COX6B1	protein_coding	chr19	35648323	35658782	+	Human GRCh	OTTHUMG000000	COX6B1	**cytochrome c oxidase subunit 6B1**	Cytoplasm	enzyme		1.023757797	0	0.91239214	0	1	0	11.3742429	11.7057341	11.6280466	11.5251378
5741	**ENSG0000012635**	THRA	protein_coding	chr17	40058290	40093867	+	Human GRCh	OTTHUMG000001	THRA	**thyroid hormone receptor alpha**	Nucleus	ligand-depen	3,5-diiodot	-1.065081531	0	0.75318075	0	1	0	12.8657835	13.1610673	13.1029112	12.7731009
5743	**ENSG0000012636**	NR1D1	protein_coding	chr17	40092793	40100589	-	Human GRCh	OTTHUMG000001	NR1D1	**nuclear receptor subfamily 1 group D member 1**	Nucleus	ligand-depen		1.082355366	0	0.74869532	0	1	0	9.5446517	9.90091966	9.76157441	9.88611952
5744	**ENSG0000012639**	FRMD8	protein_coding	chr11	65386621	65413525	+	Human GRCh	OTTHUMG000001	FRMD8	**FERM domain containing 8**	Cytoplasm	other		1.030207404	0	0.87234257	0	1	0	9.93480277	10.2805975	10.2377019	10.0598815
5745	**ENSG0000012643**	PRDX5	protein_coding	chr11	64318121	64321811	+	Human GRCh	OTTHUMG000001	PRDX5	**peroxiredoxin 5**	Cytoplasm	enzyme	auranofin	1.233862372	0	0.38602101	0	1	0	10.4505733	10.64994	10.8062974	10.7674338
5746	**ENSG0000012645**	BCL2L12	protein_coding	chr19	49665142	49673916	+	Human GRCh	OTTHUMG000001	BCL2L12	**BCL2 like 12**	Nucleus	other	NU-0129	1.103293295	0	0.63674025	0	1	0	9.25592426	9.48726066	9.58828945	9.38041628
5747	**ENSG0000012645**	IRF3	protein_coding	chr19	49659569	49665875	-	Human GRCh	OTTHUMG000001	IRF3	**interferon regulatory factor 3**	Nucleus	transcription		1.055232821	0	0.78644247	0	1	0	9.95171996	10.1993863	10.2145857	10.0641672
5748	**ENSG0000012645**	PRMT1	protein_coding	chr19	49675786	49689029	+	Human GRCh	OTTHUMG000001	PRMT1	**protein arginine methyltransferase 1**	Nucleus	enzyme	PRMT1 inh	-1.063316982	0	0.75331341	0	1	0	12.0087206	12.3143511	12.2120642	11.9793952
5749	**ENSG0000012645**	RRAS	protein_coding	chr19	49635292	49640143	-	Human GRCh	OTTHUMG000001	RRAS	**RAS related**	Cytoplasm	enzyme		1.19328305	0	0.65238483	0	1	0	5.11255211	5.31128142	5.2796608	5.47637565
5750	**ENSG0000012646**	PRRG2	protein_coding	chr19	49580646	49591004	+	Human GRCh	OTTHUMG000001	PRRG2	**proline rich and Gla domain 2**	Extracellular	other		1.045070775	0	0.86694074	0	1	0	8.36142693	8.64569714	8.60368054	8.51649485
5751	**ENSG0000012646**	SCAF1	protein_coding	chr19	49642209	49658642	+	Human GRCh	OTTHUMG000001	SCAF1	**SR-related CTD associated factor 1**	Extracellular	other		1.320678742	0	0.16669128	0	1	0	11.5713948	11.7220355	12.0263802	11.8903446
5752	**ENSG0000012646**	PRR12	protein_coding	chr19	49591182	49626439	+	Human GRCh	OTTHUMG000001	PRR12	**proline rich 12**	Extracellular	other		1.048220773	0	0.8133567	0	1	0	12.6681799	12.9425304	12.9090529	12.8230724
5754	**ENSG0000012650**	FLRT1	protein_coding	chr11	64035931	64119173	+	Human GRCh	OTTHUMG000001	FLRT1	**fibronectin leucine rich transmembrane protein 1**	Plasma Mem	other		-1.174311881	0	0.72072451	0	1	0	3.98232083	4.4295402	4.11694321	4.09186693
5755	**ENSG0000012652**	ASL	protein_coding	chr7	66075800	66094697	+	Human GRCh	OTTHUMG000000	ASL	**argininosuccinate lyase**	Cytoplasm	enzyme		1.02442387	0	0.90609198	0	1	0	9.83568882	9.98463277	9.9864569	9.88933846
5756	**ENSG0000012652**	SBDS	protein_coding	chr7	66987680	66995693	-	Human GRCh	OTTHUMG000000	SBDS	**SBDS ribosome maturation factor**	Nucleus	other		1.077640392	0	0.71658399	0	1	0	10.0651164	10.425024	10.4561097	10.2158434
5760	**ENSG0000012656**	STAT5A	protein_coding	chr17	42287547	42311943	+	Human GRCh	OTTHUMG000001	STAT5A	**signal transducer and activator of transcription 5A**	Nucleus	transcription		-1.113607782	0	0.76181794	0	1	0	7.82403824	8.0010768	8.11268028	7.38078829
5761	**ENSG0000012656**	WNK4	protein_coding	chr17	42780610	42797066	+	Human GRCh	OTTHUMG000001	WNK4	**WNK lysine deficient protein kinase 4**	Plasma Mem	kinase	WNK463	-1.608794627	-1	0.26089584	0	1	0	5.84042036	6.59322435	5.87742018	5.69407402
5762	**ENSG0000012658**	BECN1	protein_coding	chr17	42810134	42833350	-	Human GRCh	OTTHUMG000001	BECN1	**beclin 1**	Cytoplasm	other		-1.067801645	0	0.75461296	0	1	0	10.4357341	10.8107324	10.7293305	10.3729777
5763	**ENSG0000012658**	PRKCG	protein_coding	chr19	53879190	53907652	+	Human GRCh	OTTHUMG000000	PRKCG	**protein kinase C gamma**	Cytoplasm	kinase	Go6983, R	-1.439464069	0	0.64566647	0	1	0	3.28528988	3.56827595	3.41955441	2.82447213
5764	**ENSG0000012660**	TRAP1	protein_coding	chr16	3651639	3717553	-	Human GRCh	OTTHUMG000001	TRAP1	**TNF receptor associated protein 1**	Cytoplasm	enzyme	gamitrinib-	-1.111751594	0	0.61405345	0	1	0	12.6909861	13.0882107	12.882832	12.6759986
5765	**ENSG0000012660**	GLIS2	protein_coding	chr16	4314761	4339597	+	Human GRCh	OTTHUMG000001	GLIS2	**GLIS family zinc finger 2**	Nucleus	transcription		1.349466018	0	0.23877811	0	1	0	6.61393495	6.79996116	7.06061776	6.93059003
5766	**ENSG0000012665**	NSRP1	protein_coding	chr17	30115521	30186475	+	Human GRCh	OTTHUMG000001	NSRP1	**nuclear speckle splicing regulatory protein 1**	Nucleus	other		1.029967402	0	0.89006093	0	1	0	9.62667841	9.73831816	9.79972107	9.62621964
5767	**ENSG0000012669**	DNAJC8	protein_coding	chr1	28199456	28233029	-	Human GRCh	OTTHUMG000000	DNAJC8	**DnaJ heat shock protein family (Hsp40) member C8**	Nucleus	other		-1.101469513	0	0.62963719	0	1	0	10.886189	11.1566173	10.9963011	10.8415928
5768	**ENSG0000012670**	AHDC1	protein_coding	chr1	27534035	27604431	-	Human GRCh	OTTHUMG000000	AHDC1	**AT-hook DNA binding motif containing 1**	Nucleus	transcription		1.527890938	1	0.04266832	1	1	0	9.89179402	10.0753392	10.343523	10.5156241
5769	**ENSG0000012670**	IFI6	protein_coding	chr1	27666064	27672212	-	Human GRCh	OTTHUMG000000	IFI6	**interferon alpha inducible protein 6**	Cytoplasm	other		-1.172050673	0	0.84812527	0	1	0	2.83131471	3.12667849	3.10401798	2.65634683
5770	**ENSG0000012673**	DACH2	protein_coding	chrX	86148451	86832604	+	Human GRCh	OTTHUMG000000	DACH2	**dachshund family transcription factor 2**	Nucleus	transcription		-1.350973063	0	0.68662053	0	1	0	4.40206527	5.14391444	4.29328306	4.72864389
5771	**ENSG0000012674**	ZNF384	protein_coding	chr12	6666477	6689572	-	Human GRCh	OTTHUMG000001	ZNF384	**zinc finger protein 384**	Nucleus	transcription		-1.145593947	0	0.47660111	0	1	0	10.987799	11.1533454	11.0290907	10.8049447
5772	**ENSG0000012674**	EMG1	protein_coding	chr12	6970913	6979936	+	Human GRCh	OTTHUMG000001	EMG1	**EMG1 N1-specific pseudouridine methyltransferase**	Nucleus	enzyme		-1.000344595	0	0.99882607	0	1	0	10.1203695	10.3501641	10.4381324	10.0030757
5773	**ENSG0000012675**	SSX1	protein_coding	chrX	48255392	48267444	+	Human GRCh	OTTHUMG000000	SSX1	**SSX family member 1**	Nucleus	transcription		5.695171672	1	0.35983205	0	NA	0	0.49554489	0.51236603	1.0199408	0.49653061
5774	**ENSG0000012675**	UXT	protein_coding	chrX	47651796	47659180	-	Human GRCh	OTTHUMG000000	UXT	**ubiquitously expressed prefoldin like chaperone**	Cytoplasm	transcription		-1.117939331	0	0.60430438	0	1	0	9.40321372	9.65772075	9.49326614	9.32884706
5775	**ENSG0000012675**	CFP	protein_coding	chrX	47623172	47630305	-	Human GRCh	OTTHUMG000000	CFP	**complement factor properdin**	Extracellular	other		-1.322193146	0	0.47467305	0	1	0	6.50823358	6.41069869	6.30436976	6.03664853
5776	**ENSG0000012676**	ELK1	protein_coding	chrX	47635521	47650604	-	Human GRCh	OTTHUMG000000	ELK1	**ETS transcription factor ELK1**	Nucleus	transcription		-1.093334629	0	0.66855622	0	1	0	10.9405435	11.2839551	11.0714058	10.9751982
5777	**ENSG0000012676**	TIMM17B	protein_coding	chrX	48893447	48898143	-	Human GRCh	OTTHUMG000000	TIMM17B	**translocase of inner mitochondrial membrane 17B**	Cytoplasm	transporter		1.105977151	0	0.62009104	0	1	0	8.25582458	8.3364713	8.51373216	8.27987782
5778	**ENSG0000012677**	PCNX4	protein_coding	chr14	60091911	60169133	+	Human GRCh	OTTHUMG000001	PCNX4	**pecanex 4**	Other	other		-1.101397573	0	0.63246123	0	1	0	11.1702254	11.3528684	11.3593463	10.9223982
5779	**ENSG0000012677**	ATG14	protein_coding	chr14	55366391	55411830	-	Human GRCh	OTTHUMG000001	ATG14	**autophagy related 14**	Cytoplasm	other		-1.155137525	0	0.52860118	0	1	0	9.45860065	9.8193597	9.60359305	9.37807614
5780	**ENSG0000012677**	KTN1	protein_coding	chr14	55559072	55701526	+	Human GRCh	OTTHUMG000001	KTN1	**kinectin 1**	Plasma Mem	transmembra		-1.070358141	0	0.69388961	0	1	0	11.6494487	11.9133592	11.8541119	11.5650482
5781	**ENSG0000012677**	SIX1	protein_coding	chr14	60643421	60658259	-	Human GRCh	OTTHUMG000001	SIX1	**SIX homeobox 1**	Nucleus	transcription		-1.174711609	0	0.77876196	0	1	0	8.48652343	8.76084194	8.67051345	8.2140729
5782	**ENSG0000012678**	RHOJ	protein_coding	chr14	63204114	63293508	+	Human GRCh	OTTHUMG000001	RHOJ	**ras homolog family member J**	Cytoplasm	enzyme		1	0	1	0	NA	0	-0.0380843	-0.0187658	-0.021919	-0.036935
5783	**ENSG0000012678**	DLGAP5	protein_coding	chr14	55148112	55191608	-	Human GRCh	OTTHUMG000001	DLGAP5	**DLG associated protein 5**	Nucleus	other		-1.211750583	0	0.39092736	0	1	0	10.717657	11.0356192	10.797279	10.5504669
5784	**ENSG0000012679**	L3HYPDH	protein_coding	chr14	59460363	59484408	-	Human GRCh	OTTHUMG000000	L3HYPDH	**trans-L-3-hydroxyproline dehydratase**	Other	enzyme		-1.15199953	0	0.60365219	0	1	0	7.54522168	7.63054314	7.69104792	7.12799221
5785	**ENSG0000012680**	HSPA2	protein_coding	chr14	64535905	64546173	+	Human GRCh	OTTHUMG000001	HSPA2	**heat shock protein family A (Hsp70) member 2**	Cytoplasm	other		-1.034903748	0	0.91058324	0	1	0	8.83878769	8.94694921	9.11061618	8.52850033
5786	**ENSG0000012680**	ZBTB1	protein_coding	chr14	64503712	64533690	+	Human GRCh	OTTHUMG000001	ZBTB1	**zinc finger and BTB domain containing 1**	Nucleus	transcription		-1.047159903	0	0.84669298	0	1	0	10.0639542	10.2457315	10.2709696	9.91910234
5787	**ENSG0000012681**	TRMT5	protein_coding	chr14	60971441	60981170	-	Human GRCh	OTTHUMG000001	TRMT5	**tRNA methyltransferase 5**	Cytoplasm	other		1.040197219	0	0.86416932	0	1	0	9.22035071	9.38614915	9.54167386	9.11916828
5788	**ENSG0000012682**	SGPP1	protein_coding	chr14	63684216	63728065	-	Human GRCh	OTTHUMG000000	SGPP1	**sphingosine-1-phosphate phosphatase 1**	Cytoplasm	phosphatase		1.305212225	0	0.33115231	0	1	0	7.98930737	7.91999759	8.32600175	8.14888889
5789	**ENSG0000012682**	PLEKHG3	protein_coding	chr14	64704102	64750249	+	Human GRCh	OTTHUMG000000	PLEKHG3	**pleckstrin homology and RhoGEF domain containing G3**	Cytoplasm	other		1.079813725	0	0.75982412	0	1	0	10.2282137	10.470347	10.5190822	10.3460613
5792	**ENSG0000012685**	RHOT1	protein_coding	chr17	32142454	32253374	+	Human GRCh	OTTHUMG000001	RHOT1	**ras homolog family member T1**	Cytoplasm	enzyme		-1.168935224	0	0.43621216	0	1	0	10.6157637	10.8655995	10.7347097	10.3884325
5794	**ENSG0000012686**	OMG	protein_coding	chr17	31272013	31297539	-	Human GRCh	OTTHUMG000001	OMG	**oligodendrocyte myelin glycoprotein**	Plasma Mem	G-protein cou		1	0	1	0	NA	0	0.03644813	0.04622395	0.04460286	0.03701932
5795	**ENSG0000012687**	DYNC2I1	protein_coding	chr7	158856558	158956747	+	Human GRCh	OTTHUMG000001	DYNC2I1	**dynein 2 intermediate chain 1**	Extracellular	other		-1.04199128	0	0.85084169	0	1	0	10.6964878	10.9334993	10.9559496	10.5596492
5796	**ENSG0000012687**	AIF1L	protein_coding	chr9	131096476	131123152	+	Human GRCh	OTTHUMG000000	AIF1L	**allograft inflammatory factor 1 like**	Plasma Mem	other		-1.059591641	0	0.80237286	0	1	0	10.2897008	10.6793808	10.6700319	10.1458681
5797	**ENSG0000012688**	FAM78A	protein_coding	chr9	131258076	131277365	-	Human GRCh	OTTHUMG000000	FAM78A	**family with sequence similarity 78 member A**	Other	other		-1.084202281	0	0.89520047	0	1	0	4.00015752	4.68215286	4.42622045	4.21892082
5798	**ENSG0000012688**	NUP214	protein_coding	chr9	131125573	131234663	+	Human GRCh	OTTHUMG000000	NUP214	**nucleoporin 214**	Nucleus	transporter		-1.071658236	0	0.71567434	0	1	0	11.9928944	12.2402861	12.1467995	11.9394389

5799	**ENSG0000012689**	CTAG2	protein_coding	chrX	154651972	154653579	-	Human GRCh	OTTHUMG000000	CTAG2	**cancer/testis antigen 2**	Cytoplasm	other		1.212455164	0	0.40417337	0	1	0	9.7140564	10.0606243	10.2638215	9.9883686
5801	**ENSG0000012690**	SLC10A3	protein_coding	chrX	154487306	154490690	-	Human GRCh	OTTHUMG000000	SLC10A3	**solute carrier family 10 member 3**	Plasma Mem	transporter		1.058170192	0	0.81893416	0	1	0	8.56299841	8.85730524	8.89550366	8.65547137
5802	**ENSG0000012693**	MAP2K2	protein_coding	chr19	4090321	4124122	-	Human GRCh	OTTHUMG000001	MAP2K2	**mitogen-activated protein kinase kinase 2**	Cytoplasm	kinase	U0126, pir	1.038894447	0	0.84098269	0	1	0	11.6930821	11.9078697	11.9208387	11.7701308
5803	**ENSG0000012694**	HNRNPH2	protein_coding	chrX	101408222	101414133	+	Human GRCh	OTTHUMG000000	HNRNPH2	**heterogeneous nuclear ribonucleoprotein H2**	Nucleus	other		1.109359514	0	0.61597252	0	1	0	10.4776572	10.8083016	10.8421194	10.6922149
5804	**ENSG0000012694**	ARMCX1	protein_coding	chrX	101550547	101554700	+	Human GRCh	OTTHUMG000000	ARMCX1	**armadillo repeat containing X-linked 1**	Other	other		-5.787208166	-1	0.26392805	0	1	0	2.00537408	2.71975886	2.08242455	2.01092801
5805	**ENSG0000012695**	TMEM35A	protein_coding	chrX	101078879	101096367	+	Human GRCh	OTTHUMG000000	TMEM35A	**transmembrane protein 35A**	Cytoplasm	other		5.532786295	1	0.22848636	0	1	0	1.53533534	1.59771283	2.21760006	1.53907072
5806	**ENSG0000012695**	TIMM8A	protein_coding	chrX	101345661	101348742	-	Human GRCh	OTTHUMG000000	TIMM8A	**translocase of inner mitochondrial membrane 8A**	Cytoplasm	transporter		1.360233994	0	0.21764298	0	1	0	8.16941522	8.44222795	8.89408547	8.33919192
5807	**ENSG0000012697**	ZC4H2	protein_coding	chrX	64915802	65034713	-	Human GRCh	OTTHUMG000000	ZC4H2	**zinc finger C4H2-type containing**	Nucleus	other		-1.287687294	0	0.44276849	0	1	0	7.72909094	8.09632489	7.82285396	7.39557866
5808	**ENSG0000012702**	CANX	protein_coding	chr5	179678628	179731641	+	Human GRCh	OTTHUMG000001	CANX	**calnexin**	Cytoplasm	other		-1.076757787	0	0.71950027	0	1	0	14.8573504	15.1198914	15.0848618	14.7096617
5809	**ENSG0000012705**	INTS11	protein_coding	chr1	1311585	1324687	-	Human GRCh	OTTHUMG000000	INTS11	**integrator complex subunit 11**	Nucleus	enzyme		-1.094065162	0	0.63252856	0	1	0	12.844724	13.0541817	12.9642723	12.7285813
5811	**ENSG0000012708**	IPPK	protein_coding	chr9	92613183	92670131	-	Human GRCh	OTTHUMG000000	IPPK	**inositol-pentakisphosphate 2-kinase**	Cytoplasm	kinase		1.037924989	0	0.8675912	0	1	0	9.06856465	9.43514188	9.46766727	9.12087263
5812	**ENSG0000012708**	ZNF484	protein_coding	chr9	92844182	92878038	-	Human GRCh	OTTHUMG000000	ZNF484	**zinc finger protein 484**	Nucleus	transcription		-1.133917992	0	0.66278527	0	1	0	8.00589752	8.37260578	8.21421304	7.91299823
5814	**ENSG0000012708**	FGD3	protein_coding	chr9	92947523	93036236	+	Human GRCh	OTTHUMG000000	FGD3	**FYVE, RhoGEF and PH domain containing 3**	Cytoplasm	other		1.244994904	0	0.52646504	0	1	0	5.8275565	6.31252035	6.46540928	6.08961135
5815	**ENSG0000012712**	HIVEP3	protein_coding	chr1	41506365	42035925	-	Human GRCh	OTTHUMG000001	HIVEP3	**HIVEP zinc finger 3**	Nucleus	transcription		1.64702772	1	0.05888214	0	1	0	7.8470534	8.29138287	8.48407299	8.7133392
5816	**ENSG0000012712**	PPCS	protein_coding	chr1	42456117	42473385	+	Human GRCh	OTTHUMG000000	PPCS	**phosphopantothenoylcysteine synthetase**	Cytoplasm	enzyme		-1.078003281	0	0.70405525	0	1	0	9.47164863	9.68124757	9.57560484	9.41598364
5817	**ENSG0000012712**	EDN2	protein_coding	chr1	41478775	41484683	-	Human GRCh	OTTHUMG000000	EDN2	**endothelin 2**	Extracellular	growth facto		3.777439536	1	0.05906649	0	1	0	3.80271062	2.55732331	4.2502828	4.38749409
5818	**ENSG0000012715**	BCL11B	protein_coding	chr14	99169287	99272197	-	Human GRCh	OTTHUMG000000	BCL11B	**BCL11 transcription factor B**	Nucleus	transcription		1.089501933	0	0.82795721	0	1	0	5.71194777	5.48886961	5.3088536	5.98498079
5819	**ENSG0000012718**	COX7C	protein_coding	chr5	86617928	86620962	+	Human GRCh	OTTHUMG000001	COX7C	**cytochrome c oxidase subunit 7C**	Cytoplasm	enzyme		1.019005088	0	0.93085419	0	1	0	11.362141	11.5799863	11.5724947	11.4169535
5820	**ENSG0000012719**	TRAF2	protein_coding	chr9	136881912	136926607	+	Human GRCh	OTTHUMG000000	TRAF2	**TNF receptor associated factor 2**	Cytoplasm	enzyme		1.078059571	0	0.69727135	0	1	0	9.90548914	10.1210373	10.2522893	9.92578555
5821	**ENSG0000012722**	ABHD8	protein_coding	chr19	17292131	17310236	-	Human GRCh	OTTHUMG000001	ABHD8	**abhydrolase domain containing 8**	Cytoplasm	enzyme		-1.352375523	0	0.30093599	0	1	0	7.59968647	7.93779774	7.54756631	7.37046173
5823	**ENSG0000012724**	ATP13A4	protein_coding	chr3	193398967	193593111	-	Human GRCh	OTTHUMG000000	ATP13A4	**ATPase 13A4**	Cytoplasm	transporter		1.844518946	1	0.24083003	0	1	0	4.4252634	4.12761776	4.94860376	4.84445217
5824	**ENSG0000012725**	PLAAT1	protein_coding	chr3	193241128	193277738	+	Human GRCh	OTTHUMG000001	PLAAT1	**phospholipase A and acyltransferase 1**	Cytoplasm	enzyme		-1.526674002	-1	0.62849838	0	NA	0	3.33111197	3.37531351	3.29862653	2.59501803
5825	**ENSG0000012731**	HELB	protein_coding	chr12	66302493	66343643	+	Human GRCh	OTTHUMG000001	HELB	**DNA helicase B**	Nucleus	enzyme	cyclophosp	-1.123537094	0	0.64339165	0	1	0	7.30605587	7.5753425	7.42185684	7.22516589
5826	**ENSG0000012731**	RAP1B	protein_coding	chr12	68610855	68671901	+	Human GRCh	OTTHUMG000001	RAP1B	**RAP1B, member of RAS oncogene family**	Cytoplasm	enzyme		-1.074650516	0	0.71856113	0	1	0	10.4759152	10.8789533	10.7184511	10.5036244
5828	**ENSG0000012732**	TSPAN8	protein_coding	chr12	71125085	71441898	-	Human GRCh	OTTHUMG000001	TSPAN8	**tetraspanin 8**	Plasma Mem	other		-1.057058855	0	0.86741597	0	1	0	8.59495714	8.64155077	8.5701835	8.53992135
5829	**ENSG0000012732**	BEST3	protein_coding	chr12	69643360	69699476	-	Human GRCh	OTTHUMG000001	BEST3	**bestrophin 3**	Nucleus	ion channel		1.208827797	0	0.81590112	0	1	0	5.46200156	6.03937647	6.46676304	5.12840693
5830	**ENSG0000012732**	RAB3IP	protein_coding	chr12	69738860	69823204	+	Human GRCh	OTTHUMG000001	RAB3IP	**RAB3A interacting protein**	Cytoplasm	other		-1.050203897	0	0.84836596	0	1	0	11.3776365	11.6558973	11.7942848	11.0229819
5831	**ENSG0000012732**	PTPRB	protein_coding	chr12	70515870	70637440	-	Human GRCh	OTTHUMG000001	PTPRB	**protein tyrosine phosphatase receptor type B**	Plasma Mem	phosphatase		-2.374825018	-1	0.18048597	0	1	0	4.29793634	4.21001263	3.84559703	3.5258636
5832	**ENSG0000012733**	DYRK2	protein_coding	chr12	67648338	67665406	+	Human GRCh	OTTHUMG000001	DYRK2	**dual specificity tyrosine phosphorylation regulated kinase 2**	Cytoplasm	kinase	LDN19296	-1.049526818	0	0.83543647	0	1	0	11.1580575	11.3878984	11.4483235	10.9487446
5833	**ENSG0000012733**	YEATS4	protein_coding	chr12	69359710	69390870	+	Human GRCh	OTTHUMG000001	YEATS4	**YEATS domain containing 4**	Nucleus	transcription		-1.039736836	0	0.88651676	0	1	0	9.21269315	9.4461263	9.60493733	8.86468198
5834	**ENSG0000012736**	TAS2R3	protein_coding	chr7	141764097	141765197	+	Human GRCh	OTTHUMG000001	TAS2R3	**taste 2 receptor member 3**	Plasma Mem	G-protein cou		-1.061985994	0	0.87672053	0	1	0	6.51590312	6.25359483	6.59760191	5.98766736
5835	**ENSG0000012736**	TAS2R4	protein_coding	chr7	141776674	141781691	+	Human GRCh	OTTHUMG000001	TAS2R4	**taste 2 receptor member 4**	Plasma Mem	G-protein co	denatoniu	-1.148625869	0	0.67849818	0	1	0	8.00640946	8.13128361	8.15189309	7.63268166
5836	**ENSG0000012736**	TAS2R5	protein_coding	chr7	141790217	141791367	+	Human GRCh	OTTHUMG000001	TAS2R5	**taste 2 receptor member 5**	Plasma Mem	G-protein cou		1.390056301	0	0.47735484	0	1	0	5.56997751	5.79162969	6.05890254	5.96105228
5837	**ENSG0000012737**	CRYGN	protein_coding	chr7	151428832	151440813	-	Human GRCh	OTTHUMG000001	CRYGN	**crystallin gamma N**	Other	other		-1.256823534	0	0.81864303	0	NA	0	2.71939927	2.71683283	2.80924498	2.38069138
5838	**ENSG0000012739**	LRRC61	protein_coding	chr7	150323263	150338156	+	Human GRCh	OTTHUMG000001	LRRC61	**leucine rich repeat containing 61**	Other	other		-1.060711653	0	0.75560923	0	1	0	9.57303761	9.72619018	9.72688386	9.42643866
5840	**ENSG0000012741**	IDUA	protein_coding	chr4	986997	1004564	+	Human GRCh	OTTHUMG000001	IDUA	**alpha-L-iduronidase**	Cytoplasm	enzyme		-1.116483222	0	0.75159255	0	1	0	8.80004836	9.14368916	9.01826053	8.6811504
5841	**ENSG0000012741**	FGFRL1	protein_coding	chr4	1009936	1026898	+	Human GRCh	OTTHUMG000001	FGFRL1	**fibroblast growth factor receptor like 1**	Plasma Mem	transmembra		1.022465558	0	0.92997945	0	1	0	10.8057248	11.0966185	11.0323737	10.9368024
5842	**ENSG0000012741**	TMEM175	protein_coding	chr4	932387	958656	+	Human GRCh	OTTHUMG000001	TMEM175	**transmembrane protein 175**	Cytoplasm	ion channel		1.024208841	0	0.92087946	0	1	0	9.46946255	9.66519862	9.57965615	9.61530976
5843	**ENSG0000012742**	AUNIP	protein_coding	chr1	25831913	25859458	-	Human GRCh	OTTHUMG000000	AUNIP	**aurora kinase A and ninein interacting protein**	Cytoplasm	other		-1.087892354	0	0.71342884	0	1	0	8.68386605	8.93993547	8.8113876	8.63328066
5844	**ENSG0000012744**	PIN1	protein_coding	chr19	9835257	9849689	+	Human GRCh	OTTHUMG000001	PIN1	**peptidylprolyl cis/trans isomerase, NIMA-interacting 1**	Nucleus	enzyme		1.100689756	0	0.63011468	0	1	0	10.1942401	10.5757602	10.5500607	10.4605435
5845	**ENSG0000012745**	FBXL12	protein_coding	chr19	9810267	9827816	-	Human GRCh	OTTHUMG000001	FBXL12	**F-box and leucine rich repeat protein 12**	Cytoplasm	other		-1.131101631	0	0.60862432	0	1	0	8.99700142	9.1922961	9.07721513	8.83846486
5846	**ENSG0000012746**	EMC1	protein_coding	chr1	19215660	19251552	-	Human GRCh	OTTHUMG000000	EMC1	**ER membrane protein complex subunit 1**	Plasma Mem	other		-1.0992023	0	0.62710583	0	1	0	11.2781989	11.5432219	11.4704175	11.1339623
5847	**ENSG0000012747**	PLA2G5	protein_coding	chr1	20028179	20091911	+	Human GRCh	OTTHUMG000000	PLA2G5	**phospholipase A2 group V**	Extracellular	enzyme	varespladi	-5.371906594	-1	0.25531374	0	NA	0	2.7428501	2.11853429	1.69932504	1.24807911
5848	**ENSG0000012748**	UBR4	protein_coding	chr1	19074510	19210266	-	Human GRCh	OTTHUMG000000	UBR4	**ubiquitin protein ligase E3 component n-recognin 4**	Nucleus	enzyme		-1.289207499	0	0.21159726	0	1	0	14.236586	14.5339667	14.2002851	14.0119722
5849	**ENSG0000012748**	HP1BP3	protein_coding	chr1	20740266	20787323	-	Human GRCh	OTTHUMG000000	HP1BP3	**heterochromatin protein 1 binding protein 3**	Nucleus	other		-1.120809065	0	0.56321382	0	1	0	12.7808704	13.0303714	12.9665601	12.5902936
5851	**ENSG0000012751**	SIN3B	protein_coding	chr19	16829398	16880353	+	Human GRCh	OTTHUMG000001	SIN3B	**SIN3 transcription regulator family member B**	Nucleus	transcription		-1.054877403	0	0.80652431	0	1	0	11.2346423	11.3878002	11.3810934	11.1126677
5853	**ENSG0000012752**	SLC35E1	protein_coding	chr19	16549831	16572415	-	Human GRCh	OTTHUMG000001	SLC35E1	**solute carrier family 35 member E1**	Cytoplasm	other		-1.098318988	0	0.66516869	0	1	0	11.0085141	11.4085959	11.2562817	10.9675178
5854	**ENSG0000012752**	EPS15L1	protein_coding	chr19	16355239	16472085	-	Human GRCh	OTTHUMG000001	EPS15L1	**epidermal growth factor receptor pathway substrate 15 like 1**	Plasma Mem	other		1.017205846	0	0.93542799	0	1	0	11.0628	11.3421922	11.3126665	11.1404462
5855	**ENSG0000012752**	KLF2	protein_coding	chr19	16324826	16328685	+	Human GRCh	OTTHUMG000001	KLF2	**KLF transcription factor 2**	Nucleus	transcription		1.57227414	1	0.1450863	0	1	0	6.23286911	6.41177977	6.87401953	6.72359797
5858	**ENSG0000012753**	F2RL3	protein_coding	chr19	16888999	16892606	+	Human GRCh	OTTHUMG000001	F2RL3	**F2R like thrombin or trypsin receptor 3**	Plasma Mem	G-protein cou		1.063927526	0	0.90884814	0	1	0	5.07622282	5.12750329	5.40741897	4.8418132
5859	**ENSG0000012754**	UQCR11	protein_coding	chr19	1597169	1605473	-	Human GRCh	OTTHUMG000001	UQCR11	**ubiquinol-cytochrome c reductase, complex III subunit XI**	Cytoplasm	enzyme		1.019148287	0	0.92983361	0	1	0	10.6046741	11.025058	10.8966386	10.8109848
5860	**ENSG0000012755**	GFER	protein_coding	chr16	1984193	1987749	+	Human GRCh	OTTHUMG000001	GFER	**growth factor, augmenter of liver regeneration**	Nucleus	enzyme	MitoBloCK	1.33941614	0	0.18189831	0	1	0	9.33629958	9.82000848	10.0597159	9.77705909
5861	**ENSG0000012756**	SYNGR3	protein_coding	chr16	1989660	1994275	+	Human GRCh	OTTHUMG000001	SYNGR3	**synaptogyrin 3**	Plasma Mem	other		1.045175132	0	0.9021634	0	1	0	8.46265214	8.98231137	8.92542589	8.66044726
5862	**ENSG0000012756**	PKMYT1	protein_coding	chr16	2968024	2980479	-	Human GRCh	OTTHUMG000001	PKMYT1	**protein kinase, membrane associated tyrosine/threonine 1**	Cytoplasm	kinase	RP-6306, S	1.035514245	0	0.86710755	0	1	0	10.6095375	10.9009598	10.864105	10.7380801
5863	**ENSG0000012757**	WFIKKN1	protein_coding	chr16	629239	634117	+	Human GRCh	OTTHUMG000000	WFIKKN1	**WAP, follistatin/kazal, immunoglobulin, kunitz and netrin dom**	Cytoplasm	other		1.267946701	0	0.42934547	0	1	0	7.84657093	8.08959417	8.39466914	8.04578052
5864	**ENSG0000012758**	WDR24	protein_coding	chr16	684622	690444	-	Human GRCh	OTTHUMG000000	WDR24	**WD repeat domain 24**	Cytoplasm	other		-1.024843789	0	0.9135799	0	1	0	9.19283349	9.44570869	9.36582218	9.22744467
5865	**ENSG0000012758**	FBXL16	protein_coding	chr16	692498	705801	-	Human GRCh	OTTHUMG000000	FBXL16	**F-box and leucine rich repeat protein 16**	Cytoplasm	other		1.369706855	0	0.32948908	0	1	0	8.85236804	9.01381528	9.51953237	8.97752847
5866	**ENSG0000012758**	CHTF18	protein_coding	chr16	788046	800737	+	Human GRCh	OTTHUMG000000	CHTF18	**chromosome transmission fidelity factor 18**	Nucleus	enzyme		1.104921173	0	0.63212323	0	1	0	11.7722727	12.0348506	12.1745796	11.8496763
5867	**ENSG0000012758**	GNG13	protein_coding	chr16	798041	800734	-	Human GRCh	OTTHUMG000000	GNG13	**G protein subunit gamma 13**	Plasma Mem	enzyme		2.545908584	1	0.56414956	0	NA	0	1.57570185	2.04491445	1.65257756	2.86969142
5868	**ENSG0000012760**	MACF1	protein_coding	chr1	39081316	39487177	+	Human GRCh	OTTHUMG000000	MACF1	**microtubule actin crosslinking factor 1**	Cytoplasm	other		-1.106199178	0	0.62310321	0	1	0	13.4019093	13.5788647	13.5745546	13.1620219
5869	**ENSG0000012761**	SMARCA4	protein_coding	chr19	10960932	11079426	+	Human GRCh	OTTHUMG000001	SMARCA4	**SWI/SNF related, matrix associated, actin dependent regulato**	Nucleus	transcription	FHD-286	-1.054766554	0	0.78651248	0	1	0	13.3920371	13.6901707	13.6452191	13.3151258
5870	**ENSG0000012766**	KDM4B	protein_coding	chr19	4969113	5153598	+	Human GRCh	OTTHUMG000001	KDM4B	**lysine demethylase 4B**	Nucleus	enzyme		-1.038444388	0	0.87473088	0	1	0	12.6409264	12.8613479	12.8092339	12.6103398
5871	**ENSG0000012766**	TICAM1	protein_coding	chr19	4815932	4831712	-	Human GRCh	OTTHUMG000001	TICAM1	**TIR domain containing adaptor molecule 1**	Nucleus	other		1.161240909	0	0.49678393	0	1	0	8.82386293	9.03687054	9.09055457	9.10755366
5872	**ENSG0000012772**	METTL25	protein_coding	chr12	82358528	82479239	+	Human GRCh	OTTHUMG000001	METTL25	**methyltransferase like 25**	Extracellular	other		-1.030339087	0	0.92043551	0	1	0	7.1644142	7.43079036	7.41688937	7.11156886
5874	**ENSG0000012777**	EMC6	protein_coding	chr17	3668812	3669668	+	Human GRCh	OTTHUMG000001	EMC6	**ER membrane protein complex subunit 6**	Cytoplasm	other		1.246583332	0	0.37056687	0	1	0	8.45060343	8.69793994	8.79265304	8.8576319
5876	**ENSG0000012780**	METTL16	protein_coding	chr17	2405562	2511891	-	Human GRCh	OTTHUMG000001	METTL16	**methyltransferase 16, RNA N6-adenosine**	Nucleus	enzyme		-1.031604216	0	0.8764642	0	1	0	10.5123647	10.8050554	10.7842959	10.4605463
5877	**ENSG0000012782**	TUBA4A	protein_coding	chr2	219249710	219277902	-	Human GRCh	OTTHUMG000001	TUBA4A	**tubulin alpha 4a**	Cytoplasm	other	docetaxel/	1.897062579	1	0.1045533	0	1	0	6.77878771	6.61369217	7.48462327	7.30297605
5878	**ENSG0000012783**	VIL1	protein_coding	chr2	218419121	218453295	+	Human GRCh	OTTHUMG000001	VIL1	**villin 1**	Cytoplasm	other		1.66154592	1	0.63091712	0	1	0	3.72282736	3.83751927	3.63862997	4.21166217
5879	**ENSG0000012783**	AAMP	protein_coding	chr2	218264129	218270178	-	Human GRCh	OTTHUMG000001	AAMP	**angio associated migratory cell protein**	Plasma Mem	other		1.01854731	0	0.92458087	0	1	0	10.7839331	11.0357725	11.0207294	10.8468054
5880	**ENSG0000012783**	PNKD	protein_coding	chr2	218269651	218346793	+	Human GRCh	OTTHUMG000001	PNKD	**PNKD metallo-beta-lactamase domain containing**	Nucleus	other		1.11082859	0	0.65151131	0	1	0	9.98495855	10.4137885	10.3441029	10.3266403
5881	**ENSG0000012786**	TNFRSF19	protein_coding	chr13	23570370	23676104	+	Human GRCh	OTTHUMG000000	TNFRSF19	**TNF receptor superfamily member 19**	Plasma Mem	transmembra		1.876279522	1	0.26822481	0	1	0	5.28861493	6.00045369	6.25483796	6.31858741
5882	**ENSG0000012787**	RNF6	protein_coding	chr13	26132115	26222314	-	Human GRCh	OTTHUMG000000	RNF6	**ring finger protein 6**	Nucleus	transcription		1.059790989	0	0.76039832	0	1	0	9.67736253	9.78175403	9.90719108	9.67110545
5883	**ENSG0000012788**	ECHS1	protein_coding	chr10	133362485	133373354	-	Human GRCh	OTTHUMG000000	ECHS1	**enoyl-CoA hydratase, short chain 1**	Cytoplasm	enzyme		-1.004310025	0	0.98403297	0	1	0	10.9695191	11.4003059	11.3064204	11.0816486
5884	**ENSG0000012790**	ZNF835	protein_coding	chr19	56661980	56671783	-	Human GRCh	OTTHUMG000001	ZNF835	**zinc finger protein 835**	Other	other		28.34661585	1	0.00977623	1	1	0	2.12792747	2.84442167	5.62480092	2.13408719
5885	**ENSG0000012791**	AKAP9	protein_coding	chr7	91940840	92110673	+	Human GRCh	OTTHUMG000001	AKAP9	**A-kinase anchoring protein 9**	Cytoplasm	other		-1.158103124	0	0.42203347	0	1	0	12.4697202	12.7199617	12.5475676	12.3211751
5886	**ENSG0000012792**	GNG11	protein_coding	chr7	93921735	93928610	+	Human GRCh	OTTHUMG000000	GNG11	**G protein subunit gamma 11**	Plasma Mem	other		1	0	1	0	NA	0	0.82026832	0.84731561	0.8428599	0.82186088
5887	**ENSG0000012792**	SEM1	protein_coding	chr7	96481626	96709880	-	Human GRCh	OTTHUMG000001	SEM1	**SEM1 26S proteasome subunit**	Nucleus	peptidase		1.407355717	0	0.12417645	0	1	0	9.23236119	9.37508694	9.81031971	9.52919691
5888	**ENSG0000012792**	GNGT1	protein_coding	chr7	93591573	93911265	+	Human GRCh	OTTHUMG000000	GNGT1	**G protein subunit gamma transducin 1**	Plasma Mem	enzyme		1	0	1	0	1	0	1.87978208	1.97451466	1.95929828	1.88552694
5889	**ENSG0000012794**	HIP1	protein_coding	chr7	75533298	75738962	-	Human GRCh	OTTHUMG000001	HIP1	**huntingtin interacting protein 1**	Cytoplasm	other		1.030006654	0	0.90246239	0	1	0	11.0448729	11.3873475	11.165622	11.3493099
5890	**ENSG0000012794**	PTPN12	protein_coding	chr7	77537295	77640069	+	Human GRCh	OTTHUMG000000	PTPN12	**protein tyrosine phosphatase non-receptor type 12**	Cytoplasm	phosphatase		1.383913364	0	0.13339022	0	1	0	9.29383074	9.39034877	9.76081079	9.584206
5891	**ENSG0000012794**	POR	protein_coding	chr7	75899200	75986855	+	Human GRCh	OTTHUMG000001	POR	**cytochrome p450 oxidoreductase**	Cytoplasm	enzyme	carboplati	-1.084165722	0	0.70024241	0	1	0	10.2915643	10.6947279	10.5112347	10.3255308
5893	**ENSG0000012795**	STYXL1	protein_coding	chr7	75996338	76048004	-	Human GRCh	OTTHUMG000001	STYXL1	**serine/threonine/tyrosine interacting like 1**	Cytoplasm	phosphatase		-1.117893548	0	0.58729282	0	1	0	8.98986683	9.13207094	9.12772804	8.72138557
5895	**ENSG0000012795**	GNAI1	protein_coding	chr7	79768028	80226181	+	Human GRCh	OTTHUMG000000	GNAI1	**G protein subunit alpha i1**	Plasma Mem	enzyme		1.000461778	0	0.99859551	0	1	0	10.4897549	10.7770727	10.7915922	10.4721873

5896	**ENSG0000012798**	PEX1	protein_coding	chr7	92487020	92528520	-	Human GRCh	OTTHUMG000000	PEX1	**peroxisomal biogenesis factor 1**	Cytoplasm	enzyme		-1.174254508	0	0.47754204	0	1	0	10.0102285	10.4343952	10.2311323	9.87862892
5897	**ENSG0000012798**	MTERF1	protein_coding	chr7	91692008	91880702	-	Human GRCh	OTTHUMG000001	MTERF1	**mitochondrial transcription termination factor 1**	Cytoplasm	transcription		1.103712193	0	0.68818463	0	1	0	8.04037008	8.48744179	8.39012683	8.38567857
5898	**ENSG0000012799**	SGCE	protein_coding	chr7	94524204	94656572	-	Human GRCh	OTTHUMG000000	SGCE	**sarcoglycan epsilon**	Plasma Mem	other		1.682714904	1	0.15645837	0	1	0	8.17179636	8.16635313	8.51896935	8.80331566
5899	**ENSG0000012799**	RBM48	protein_coding	chr7	92528773	92540481	+	Human GRCh	OTTHUMG000001	RBM48	**RNA binding motif protein 48**	Nucleus	other		-1.118486636	0	0.64217612	0	1	0	9.02056543	9.15711697	9.18274911	8.70726294
5900	**ENSG0000012799**	CASD1	protein_coding	chr7	94509219	94557019	+	Human GRCh	OTTHUMG000000	CASD1	**CAS1 domain containing 1**	Cytoplasm	enzyme		1.071718671	0	0.78136627	0	1	0	8.76000307	8.81511543	8.97954663	8.72538863
5901	**ENSG0000012800**	ZNF780B	protein_coding	chr19	40028260	40056231	-	Human GRCh	OTTHUMG000001	ZNF780B	**zinc finger protein 780B**	Other	other		1.117352354	0	0.72935005	0	1	0	9.62820128	9.89421223	10.1522289	9.5589733
5902	**ENSG0000012801**	LRFN1	protein_coding	chr19	39306566	39320863	-	Human GRCh	OTTHUMG000001	LRFN1	**leucine rich repeat and fibronectin type III domain containing :**	Nucleus	other		1.076445231	0	0.77348855	0	1	0	8.80010442	9.1697048	9.18787767	8.97170673
5903	**ENSG0000012801**	ZFP36	protein_coding	chr19	39406847	39409412	+	Human GRCh	OTTHUMG000001	ZFP36	**ZFP36 ring finger protein**	Nucleus	transcription		1.265471607	0	0.39588586	0	1	0	7.79966556	8.08045424	8.10200306	8.30703714
5904	**ENSG0000012803**	SRD5A3	protein_coding	chr4	55346213	55373100	+	Human GRCh	OTTHUMG000001	SRD5A3	**steroid 5 alpha-reductase 3**	Cytoplasm	enzyme		-1.054426167	0	0.79935929	0	1	0	9.63431789	9.69368667	9.6881381	9.51553123
5905	**ENSG0000012804**	SPINK2	protein_coding	chr4	56809860	56821742	-	Human GRCh	OTTHUMG000001	SPINK2	**serine peptidase inhibitor Kazal type 2**	Extracellular	other		-1.552586883	-1	0.67351322	0	NA	0	2.71488294	2.60331602	2.13179415	2.65419143
5906	**ENSG0000012804**	RASL11B	protein_coding	chr4	52862317	52866835	+	Human GRCh	OTTHUMG000001	RASL11B	**RAS like family 11 member B**	Plasma Mem	enzyme		2.531799027	1	0.0925351	0	1	0	2.95562615	3.57966159	4.06624052	3.75584972
5907	**ENSG0000012805**	PAICS	protein_coding	chr4	56435741	56464578	+	Human GRCh	OTTHUMG000001	PAICS	**phosphoribosylaminoimidazole carboxylase and phosphoribos**	Cytoplasm	enzyme		-1.121096169	0	0.56353261	0	1	0	13.2014501	13.594499	13.3550561	13.208649
5908	**ENSG0000012805**	KDR	protein_coding	chr4	55078481	55125595	-	Human GRCh	OTTHUMG000001	KDR	**kinase insert domain receptor**	Plasma Mem	kinase	CHIR-265,	-1.002416025	0	0.99610152	0	1	0	3.90289774	5.23706955	4.70604919	4.78753786
5909	**ENSG0000012805**	PPAT	protein_coding	chr4	56393362	56435615	-	Human GRCh	OTTHUMG000001	PPAT	**phosphoribosyl pyrophosphate amidotransferase**	Cytoplasm	enzyme	L-asparagi	1.004591584	0	0.98369282	0	1	0	10.8488131	11.0857314	11.198933	10.7069633
5910	**ENSG0000012815**	TUBGCP6	protein_coding	chr22	50217689	50245023	-	Human GRCh	OTTHUMG000000	TUBGCP6	**tubulin gamma complex component 6**	Cytoplasm	other		1.03148426	0	0.89333371	0	1	0	11.5280754	11.8183591	11.8078088	11.6188147
5911	**ENSG0000012816**	ADM2	protein_coding	chr22	50481543	50486440	+	Human GRCh	OTTHUMG000001	ADM2	**adrenomedullin 2**	Extracellular	other		-1.528384142	-1	0.17902865	0	1	0	9.44514533	8.60730709	8.76029075	8.47755411
5912	**ENSG0000012818**	DGCR6L	protein_coding	chr22	20314238	20320080	-	Human GRCh	OTTHUMG000001	DGCR6L	**DiGeorge syndrome critical region gene 6 like**	Nucleus	other		1.079953887	0	0.77072203	0	1	0	9.11786046	9.250686	9.40035925	9.12543522
5913	**ENSG0000012819**	DGCR8	protein_coding	chr22	20080221	20111877	+	Human GRCh	OTTHUMG000001	DGCR8	**DGCR8 microprocessor complex subunit**	Nucleus	enzyme		-1.201748712	0	0.31634799	0	1	0	11.4195967	11.6590865	11.4809323	11.1861002
5914	**ENSG0000012820**	ASPHD2	protein_coding	chr22	26429260	26445015	+	Human GRCh	OTTHUMG000001	ASPHD2	**aspartate beta-hydroxylase domain containing 2**	Other	enzyme		-1.235553395	0	0.65732806	0	1	0	4.90850035	4.92069135	4.98016193	4.45263923
5915	**ENSG0000012821**	VPREB3	protein_coding	chr22	23752743	23754425	-	Human GRCh	OTTHUMG000001	VPREB3	**V-set pre-B cell surrogate light chain 3**	Cytoplasm	other		-1.129151998	0	0.87690841	0	NA	0	3.1840494	4.12960002	3.83886429	3.45978311
5916	**ENSG0000012822**	SDF2L1	protein_coding	chr22	21642302	21644299	+	Human GRCh	OTTHUMG000001	SDF2L1	**stromal cell derived factor 2 like 1**	Cytoplasm	other		1.18996489	0	0.51019445	0	1	0	9.51860244	9.81573562	9.93513995	9.81495311
5917	**ENSG0000012824**	GAL3ST1	protein_coding	chr22	30554635	30574665	-	Human GRCh	OTTHUMG000001	GAL3ST1	**galactose-3-O-sulfotransferase 1**	Cytoplasm	enzyme		-1.867043315	-1	0.39863346	0	1	0	3.47621941	3.51566764	3.13596421	3.03840038
5918	**ENSG0000012824**	YWHAH	protein_coding	chr22	31944522	31957603	+	Human GRCh	OTTHUMG000000	YWHAH	**tyrosine 3-monooxygenase/tryptophan 5-monooxygenase acti**	Cytoplasm	transcription		-1.009834158	0	0.96007476	0	1	0	10.7914305	11.0936297	11.0604892	10.8084991
5920	**ENSG0000012825**	RFPL2	protein_coding	chr22	32190435	32205073	-	Human GRCh	OTTHUMG000000	RFPL2	**ret finger protein like 2**	Other	other		1.008880436	0	0.99156494	0	1	0	3.31243613	3.73709825	3.13139931	3.84940999
5922	**ENSG0000012826**	GNAZ	protein_coding	chr22	23070519	23125032	+	Human GRCh	OTTHUMG000001	GNAZ	**G protein subunit alpha z**	Plasma Mem	enzyme		1.108636747	0	0.58367522	0	1	0	8.6337888	8.70321196	8.80081277	8.75287648
5923	**ENSG0000012826**	MGAT3	protein_coding	chr22	39457012	39492194	+	Human GRCh	OTTHUMG000000	MGAT3	**beta-1,4-mannosyl-glycoprotein 4-beta-N-acetylglucosaminylt**	Cytoplasm	enzyme		3.590433217	1	0.09570145	0	1	0	2.67639222	3.10924559	3.83886944	3.57092256
5924	**ENSG0000012827**	ADORA2A	protein_coding	chr22	24417879	24442357	+	Human GRCh	OTTHUMG000001	ADORA2A	**adenosine A2a receptor**	Plasma Mem	G-protein cou	preladenan	1.179110313	0	0.68030133	0	1	0	6.60640564	7.25125019	7.32022784	6.93889295
5925	**ENSG0000012827**	ATF4	protein_coding	chr22	39519695	39522683	+	Human GRCh	OTTHUMG000001	ATF4	**activating transcription factor 4**	Nucleus	transcription		-1.210933613	0	0.35830729	0	1	0	12.8501832	12.8956993	12.7900232	12.5081158
5926	**ENSG0000012827**	A4GALT	protein_coding	chr22	42692121	42721298	-	Human GRCh	OTTHUMG000001	A4GALT	**alpha 1,4-galactosyltransferase (P blood group)**	Cytoplasm	enzyme		1.045415821	0	0.93340256	0	1	0	5.2845566	5.17987039	4.77661997	5.61118961
5928	**ENSG0000012828**	CDC42EP1	protein_coding	chr22	37560480	37569405	+	Human GRCh	OTTHUMG000001	CDC42EP1	**CDC42 effector protein 1**	Extracellular	other		-1.027321763	0	0.96834598	0	1	0	5.00629253	4.7906525	4.39838935	5.20971225
5929	**ENSG0000012828**	APOL3	protein_coding	chr22	36140329	36166177	-	Human GRCh	OTTHUMG000001	APOL3	**apolipoprotein L3**	Cytoplasm	transporter		1	0	1	0	1	0	1.74646619	1.79329374	1.78561601	1.7492389
5930	**ENSG0000012828**	MCHR1	protein_coding	chr22	40679273	40682812	+	Human GRCh	OTTHUMG000001	MCHR1	**melanin concentrating hormone receptor 1**	Plasma Mem	G-protein cou	SB 568849	1.38646334	0	0.71769787	0	1	0	2.13654711	1.84863504	2.57697936	1.72983664
5931	**ENSG0000012829**	TPST2	protein_coding	chr22	26521996	26596717	-	Human GRCh	OTTHUMG000001	TPST2	**tyrosylprotein sulfotransferase 2**	Cytoplasm	enzyme		1.090909363	0	0.80055379	0	1	0	7.4034705	7.60182947	7.75606958	7.417124
5932	**ENSG0000012829**	BAIAP2L2	protein_coding	chr22	38084652	38111015	-	Human GRCh	OTTHUMG000001	BAIAP2L2	**BAR/IMD domain containing adaptor protein 2 like 2**	Cytoplasm	other		-2.934269248	-1	0.04253481	1	1	0	5.85010415	6.06152553	5.14113779	4.71398685
5933	**ENSG0000012830**	MPST	protein_coding	chr22	37019635	37029821	+	Human GRCh	OTTHUMG000001	MPST	**mercaptopyruvate sulfurtransferase**	Cytoplasm	enzyme		-1.097356481	0	0.70283577	0	1	0	10.5464736	10.8246377	10.7097095	10.4527587
5934	**ENSG0000012831**	GALR3	protein_coding	chr22	37823382	37825485	+	Human GRCh	OTTHUMG000001	GALR3	**galanin receptor 3**	Plasma Mem	G-protein cou		1.290422396	0	0.79398294	0	NA	0	2.65149641	3.27431205	3.03673766	3.35083672
5935	**ENSG0000012831**	TST	protein_coding	chr22	37010859	37020183	-	Human GRCh	OTTHUMG000001	TST	**thiosulfate sulfurtransferase**	Cytoplasm	enzyme		-1.101915595	0	0.76106215	0	1	0	8.36945602	8.64811129	8.56450248	8.23207417
5938	**ENSG0000012833**	APOL2	protein_coding	chr22	36226209	36239954	-	Human GRCh	OTTHUMG000001	APOL2	**apolipoprotein L2**	Plasma Mem	ion channel		-1.026457548	0	0.9205007	0	1	0	8.13504563	8.59514149	8.55881361	8.11789181
5939	**ENSG0000012834**	RAC2	protein_coding	chr22	37225270	37259594	-	Human GRCh	OTTHUMG000001	RAC2	**Rac family small GTPase 2**	Cytoplasm	enzyme		-1.3748135	0	0.55200921	0	1	0	5.42659941	5.89064991	5.43276596	5.26694998
5940	**ENSG0000012834**	LIF	protein_coding	chr22	30240453	30246759	-	Human GRCh	OTTHUMG000001	LIF	**LIF interleukin 6 family cytokine**	Extracellular	cytokine		2.134008149	1	0.36385657	0	1	0	2.66355607	2.89669466	2.58942007	3.55836492
5941	**ENSG0000012834**	C22orf23	protein_coding	chr22	37943050	37953669	-	Human GRCh	OTTHUMG000001	C22orf23	**chromosome 22 open reading frame 23**	Other	other		-1.318912845	0	0.6711717	0	1	0	3.49277341	4.52943016	3.82181552	4.0097627
5943	**ENSG0000012839**	APOBEC3F	protein_coding	chr22	39040604	39055972	+	Human GRCh	OTTHUMG000001	APOBEC3F	**apolipoprotein B mRNA editing enzyme catalytic subunit 3F**	Cytoplasm	enzyme		-5.507691179	-1	0.17397096	0	1	0	2.00941765	2.76173179	2.10016401	2.01599487
5944	**ENSG0000012840**	RIBC2	protein_coding	chr22	45413693	45432509	+	Human GRCh	OTTHUMG000001	RIBC2	**RIB43A domain with coiled-coils 2**	Extracellular	other		-1.088910705	0	0.72751195	0	1	0	8.14876451	8.33922276	8.16534062	8.15121043
5946	**ENSG0000012846**	EMC4	protein_coding	chr15	34225013	34230156	+	Human GRCh	OTTHUMG000001	EMC4	**ER membrane protein complex subunit 4**	Cytoplasm	other		-1.09013987	0	0.72185942	0	1	0	9.81055625	10.2399073	10.065436	9.81532347
5947	**ENSG0000012848**	RNF112	protein_coding	chr17	19411125	19417276	+	Human GRCh	OTTHUMG000000	RNF112	**ring finger protein 112**	Nucleus	transcription		-1.330456773	0	0.57777346	0	1	0	4.99633383	5.49977554	5.15772811	4.84585915
5948	**ENSG0000012848**	SPECC1	protein_coding	chr17	20008865	20319026	+	Human GRCh	OTTHUMG000001	SPECC1	**sperm antigen with calponin homology and coiled-coil domain**	Nucleus	other		-1.110341174	0	0.59694021	0	1	0	11.1344385	11.3760315	11.2328856	11.050669
5949	**ENSG0000012851**	CPA4	protein_coding	chr7	130293134	130324180	+	Human GRCh	OTTHUMG000001	CPA4	**carboxypeptidase A4**	Extracellular	peptidase		10.29193885	1	0.08840701	0	1	0	1.45941104	1.4925151	1.48705367	2.25022564
5950	**ENSG0000012851**	DOCK4	protein_coding	chr7	111726110	112206407	-	Human GRCh	OTTHUMG000001	DOCK4	**dedicator of cytokinesis 4**	Plasma Mem	other		-1.275742887	0	0.34804613	0	1	0	11.9755639	12.3635972	12.2142487	11.483751
5951	**ENSG0000012851**	POT1	protein_coding	chr7	124822386	124929983	-	Human GRCh	OTTHUMG000001	POT1	**protection of telomeres 1**	Nucleus	other		1.001566728	0	0.99506468	0	1	0	9.04312065	9.15783917	9.22809701	8.96419192
5953	**ENSG0000012852**	ATP6V1F	protein_coding	chr7	128862856	128865847	+	Human GRCh	OTTHUMG000001	ATP6V1F	**ATPase H+ transporting V1 subunit F**	Cytoplasm	enzyme		1.00023093	0	0.9991779	0	1	0	10.2377457	10.7141287	10.5345981	10.4636397
5954	**ENSG0000012853**	LSM8	protein_coding	chr7	118184144	118204035	+	Human GRCh	OTTHUMG000001	LSM8	**LSM8 homolog, U6 small nuclear RNA associated**	Nucleus	other		-1.013226585	0	0.95271567	0	1	0	10.4163045	10.6663839	10.7367547	10.2892411
5955	**ENSG0000012853**	CDHR3	protein_coding	chr7	105876796	106036432	+	Human GRCh	OTTHUMG000001	CDHR3	**cadherin related family member 3**	Plasma Mem	other		1.329835445	0	0.40719084	0	1	0	6.96616841	7.06345325	7.32701592	7.28099239
5956	**ENSG0000012856**	PRKRIP1	protein_coding	chr7	102363872	102426676	+	Human GRCh	OTTHUMG000001	PRKRIP1	**PRKR interacting protein 1**	Nucleus	other		-1.069459411	0	0.75991567	0	1	0	9.60928809	9.88210924	9.7587947	9.59359994
5957	**ENSG0000012856**	VGF	protein_coding	chr7	101162509	101165569	-	Human GRCh	OTTHUMG000001	VGF	**VGF nerve growth factor inducible**	Extracellular	growth facto		1.332956167	0	0.284894	0	1	0	6.67750008	6.12540581	6.68082556	6.74826762
5958	**ENSG0000012856**	PODXL	protein_coding	chr7	131500262	131558217	-	Human GRCh	OTTHUMG000001	PODXL	**podocalyxin like**	Plasma Mem	kinase		1.053366315	0	0.87731341	0	1	0	9.85620949	10.0091382	10.1659799	9.78993507
5959	**ENSG0000012857**	FOXP2	protein_coding	chr7	114086327	114693772	+	Human GRCh	OTTHUMG000000	FOXP2	**forkhead box P2**	Nucleus	transcription		-1.656902124	-1	0.15580439	0	1	0	8.88794861	9.18078906	8.45025913	8.5259649
5960	**ENSG0000012857**	STRIP2	protein_coding	chr7	129434432	129488399	+	Human GRCh	OTTHUMG000001	STRIP2	**striatin interacting protein 2**	Cytoplasm	other		1.096935314	0	0.72720777	0	1	0	7.3437987	7.74288589	7.72366649	7.58638877
5961	**ENSG0000012858**	IFT22	protein_coding	chr7	101310914	101321823	-	Human GRCh	OTTHUMG000001	IFT22	**intraflagellar transport 22**	Extracellular	other		-1.216796185	0	0.35396196	0	1	0	9.54849303	9.86704821	9.61244308	9.37205843
5962	**ENSG0000012858**	MKLN1	protein_coding	chr7	131110096	131496632	+	Human GRCh	OTTHUMG000001	MKLN1	**muskelin 1**	Cytoplasm	other		-1.15488883	0	0.51039137	0	1	0	12.1229105	12.2728027	12.1661501	11.896015
5963	**ENSG0000012859**	DNAJB9	protein_coding	chr7	108569867	108574850	+	Human GRCh	OTTHUMG000001	DNAJB9	**DnaJ heat shock protein family (Hsp40) member B9**	Nucleus	other		1.089177923	0	0.73967981	0	1	0	8.96225856	9.1237051	9.35715383	8.87907377
5964	**ENSG0000012859**	FLNC	protein_coding	chr7	128830406	128859274	+	Human GRCh	OTTHUMG000000	FLNC	**filamin C**	Cytoplasm	other		-1.228773445	0	0.68213746	0	1	0	5.97555782	5.3035458	5.11722752	5.76117053
5965	**ENSG0000012859**	LRRC4	protein_coding	chr7	128027071	128032107	-	Human GRCh	OTTHUMG000001	LRRC4	**leucine rich repeat containing 4**	Plasma Mem	other		1	0	1	0	1	0	0.80065286	0.87418232	0.86240757	0.80512657
5966	**ENSG0000012859**	CALU	protein_coding	chr7	128739292	128773400	+	Human GRCh	OTTHUMG000001	CALU	**calumenin**	Cytoplasm	other		-1.078638944	0	0.68825763	0	1	0	11.6693776	11.9010416	11.8396212	11.5553184
5967	**ENSG0000012859**	CCDC136	protein_coding	chr7	128790757	128822132	+	Human GRCh	OTTHUMG000001	CCDC136	**coiled-coil domain containing 136**	Cytoplasm	other		1.484399579	0	0.3155698	0	1	0	7.18030128	7.43095037	7.9525851	7.49236979
5968	**ENSG0000012860**	SMO	protein_coding	chr7	129188633	129213545	+	Human GRCh	OTTHUMG000001	SMO	**smoothened, frizzled class receptor**	Plasma Mem	G-protein cou	gemcitabir	1.492942517	0	0.32442868	0	1	0	5.33253616	5.88940965	6.21385869	5.87736165
5969	**ENSG0000012860**	IRF5	protein_coding	chr7	128937457	128950038	+	Human GRCh	OTTHUMG000001	IRF5	**interferon regulatory factor 5**	Nucleus	transcription		1.377320599	0	0.48789475	0	1	0	4.94380579	5.19352132	5.33963837	5.41087754
5970	**ENSG0000012860**	LRRC17	protein_coding	chr7	102913000	102945111	+	Human GRCh	OTTHUMG000001	LRRC17	**leucine rich repeat containing 17**	Extracellular	other		-2.031505309	-1	0.24206426	0	1	0	4.16336658	4.13184255	3.5154451	3.73473721
5971	**ENSG0000012860**	KLHDC10	protein_coding	chr7	130070534	130135705	+	Human GRCh	OTTHUMG000001	KLHDC10	**kelch domain containing 10**	Nucleus	other		-1.135136696	0	0.55347989	0	1	0	10.8943841	11.1648693	11.0395709	10.7323992
5972	**ENSG0000012860**	NDUFA5	protein_coding	chr7	123536997	123557904	-	Human GRCh	OTTHUMG000001	NDUFA5	**NADH:ubiquinone oxidoreductase subunit A5**	Cytoplasm	enzyme		1.084195	0	0.71669566	0	1	0	11.1772954	11.3318808	11.5172351	11.1502406
5973	**ENSG0000012861**	FEZF1	protein_coding	chr7	122301303	122310691	-	Human GRCh	OTTHUMG000001	FEZF1	**FEZ family zinc finger 1**	Other	transcription		1.140838888	0	0.67733742	0	1	0	5.5458761	5.50203383	5.54013169	5.77544152
5975	**ENSG0000012862**	MRPS12	protein_coding	chr19	38930944	38933168	+	Human GRCh	OTTHUMG000001	MRPS12	**mitochondrial ribosomal protein S12**	Cytoplasm	other		1.215068617	0	0.33965669	0	1	0	9.49696001	9.85051543	9.97543301	9.83872133
5976	**ENSG0000012864**	MYO1B	protein_coding	chr2	191245185	191425389	+	Human GRCh	OTTHUMG000001	MYO1B	**myosin IB**	Cytoplasm	other		-1.029273698	0	0.8933055	0	1	0	10.9158832	11.1100459	11.1452792	10.8027736
5977	**ENSG0000012864**	HOXD1	protein_coding	chr2	176188668	176190907	+	Human GRCh	OTTHUMG000001	HOXD1	**homeobox D1**	Nucleus	transcription		1	0	1	0	1	0	1.28287721	1.33513424	1.32663129	1.28599872
5978	**ENSG0000012865**	HOXD3	protein_coding	chr2	176136612	176173102	+	Human GRCh	OTTHUMG000001	HOXD3	**homeobox D3**	Nucleus	transcription		-1.289598051	0	0.67767988	0	1	0	3.81807775	3.72628562	2.89660129	4.02212942
5979	**ENSG0000012865**	MTX2	protein_coding	chr2	176269395	176338025	+	Human GRCh	OTTHUMG000001	MTX2	**metaxin 2**	Cytoplasm	transporter		-1.085094574	0	0.74136599	0	1	0	8.20547619	8.47583482	8.57050485	7.84596493
5980	**ENSG0000012865**	PDE11A	protein_coding	chr2	177623244	178108339	-	Human GRCh	OTTHUMG000001	PDE11A	**phosphodiesterase 11A**	Cytoplasm	enzyme	dyphylline,	-1.481583113	0	0.70605757	0	1	0	3.04917348	1.99546171	1.98534123	2.80281664
5981	**ENSG0000012865**	CHN1	protein_coding	chr2	174798809	175005381	-	Human GRCh	OTTHUMG000001	CHN1	**chimerin 1**	Cytoplasm	other		-1.021746109	0	0.9563517	0	1	0	6.25511989	6.18169552	6.10664917	6.28389141
5982	**ENSG0000012868**	GAD1	protein_coding	chr2	170813213	170861151	+	Human GRCh	OTTHUMG000000	GAD1	**glutamate decarboxylase 1**	Cytoplasm	enzyme		1.438118016	0	0.28467101	0	1	0	5.89363281	5.98228337	6.46358996	6.07741481
5983	**ENSG0000012869**	OSGEPL1	protein_coding	chr2	189746660	189763227	-	Human GRCh	OTTHUMG000001	OSGEPL1	**O-sialoglycoprotein endopeptidase like 1**	Cytoplasm	peptidase		1.11050259	0	0.69348185	0	1	0	8.16700879	8.64592251	8.77022164	8.26516876
5984	**ENSG0000012869**	ORMDL1	protein_coding	chr2	189770267	189784364	-	Human GRCh	OTTHUMG000001	ORMDL1	**ORMDL sphingolipid biosynthesis regulator 1**	Cytoplasm	other		-1.109205834	0	0.63224935	0	1	0	9.97746508	10.126099	10.1314018	9.71032493
5985	**ENSG0000012870**	HAT1	protein_coding	chr2	171922448	171983686	+	Human GRCh	OTTHUMG000001	HAT1	**histone acetyltransferase 1**	Nucleus	enzyme		1.018573231	0	0.93374939	0	1	0	9.47089978	9.75467692	9.6740115	9.60762893
5986	**ENSG0000012870**	HOXD9	protein_coding	chr2	176122719	176124937	+	Human GRCh	OTTHUMG000001	HOXD9	**homeobox D9**	Nucleus	transcription		1.097287697	0	0.7448626	0	1	0	6.7870114	6.91829648	6.88369179	7.01357331
5987	**ENSG0000012871**	HOXD10	protein_coding	chr2	176108790	176119937	+	Human GRCh	OTTHUMG000001	HOXD10	**homeobox D10**	Nucleus	transcription		1	0	1	0	1	0	1.49810859	1.5987149	1.58264714	1.50425024

5988	**ENSG0000012871**	HOXD11	protein_coding	chr2	176104216	176109754	+	Human GRCh	OTTHUMG000001	HOXD11	**homeobox D11**	Nucleus	transcription		1	0	1	0	1	0	1.36933619	1.43832379	1.42715416	1.37348023
5989	**ENSG0000012871**	HOXD13	protein_coding	chr2	176092721	176095944	+	Human GRCh	OTTHUMG000001	HOXD13	**homeobox D13**	Nucleus	transcription		-1.184522338	0	0.75400151	0	1	0	3.84130323	3.8031473	3.5376563	3.85108388
5990	**ENSG0000012873**	HERC2	protein_coding	chr15	28111040	28322179	-	Human GRCh	OTTHUMG000001	HERC2	**HECT and RLD domain containing E3 ubiquitin protein ligase 2**	Cytoplasm	enzyme		-1.201876813	0	0.34291924	0	1	0	12.5076389	12.7349255	12.60226	12.2096676
5991	**ENSG0000012873**	SNRPN	protein_coding	chr15	24823637	24978723	+	Human GRCh	OTTHUMG000001	SNRPN	**small nuclear ribonucleoprotein polypeptide N**	Nucleus	other		-1.020624687	0	0.93476585	0	1	0	8.72826907	8.90074645	8.90188697	8.67612333
5992	**ENSG0000012878**	PSMG2	protein_coding	chr18	12658043	12728945	+	Human GRCh	OTTHUMG000001	PSMG2	**proteasome assembly chaperone 2**	Nucleus	other		-1.105340427	0	0.58253257	0	1	0	10.4238257	10.6222486	10.5208244	10.3030491
5993	**ENSG0000012879**	TWSG1	protein_coding	chr18	9334767	9402420	+	Human GRCh	OTTHUMG000001	TWSG1	**twisted gastrulation BMP signaling modulator 1**	Extracellular	other		1.222041731	0	0.44550809	0	1	0	8.64614198	8.84378358	9.1816519	8.70544151
5994	**ENSG0000012880**	ARHGAP22	protein_coding	chr10	48446036	48656265	-	Human GRCh	OTTHUMG000000	ARHGAP22	**Rho GTPase activating protein 22**	Cytoplasm	other		3.57083923	1	0.00768882	1	1	0	3.87031687	4.64829645	5.50532175	4.71427743
5995	**ENSG0000012881**	WDFY4	protein_coding	chr10	48684873	48982956	+	Human GRCh	OTTHUMG000000	WDFY4	**WDFY family member 4**	Other	other		1	0	1	0	NA	0	0.74486091	0.77366821	0.76893742	0.7465632
5996	**ENSG0000012882**	EIF2AK4	protein_coding	chr15	39934115	40035591	+	Human GRCh	OTTHUMG000001	EIF2AK4	**eukaryotic translation initiation factor 2 alpha kinase 4**	Cytoplasm	kinase	A-92, GCN	-1.094701783	0	0.6663371	0	1	0	11.1719676	11.5230951	11.2631041	11.2501538
5997	**ENSG0000012883**	MYO5C	protein_coding	chr15	52192322	52295804	-	Human GRCh	OTTHUMG000001	MYO5C	**myosin VC**	Cytoplasm	other		-1.173660774	0	0.39267466	0	1	0	11.8968063	12.1145976	11.9325646	11.7200155
5998	**ENSG0000012884**	CGNL1	protein_coding	chr15	57375967	57550717	+	Human GRCh	OTTHUMG000001	CGNL1	**cingulin like 1**	Plasma Mem	other		2.493478874	1	0.0751434	0	1	0	4.56551042	4.99435713	4.59080485	6.13421767
5999	**ENSG0000012887**	TMOD2	protein_coding	chr15	51751597	51816363	+	Human GRCh	OTTHUMG000001	TMOD2	**tropomodulin 2**	Cytoplasm	other		1.202667819	0	0.61894375	0	1	0	6.73108446	7.00133013	7.26353288	6.79846289
6000	**ENSG0000012888**	TTBK2	protein_coding	chr15	42738730	42920809	-	Human GRCh	OTTHUMG000001	TTBK2	**tau tubulin kinase 2**	Cytoplasm	kinase		-1.044403045	0	0.84834591	0	1	0	10.0819246	10.4270437	10.3705096	10.0452868
6001	**ENSG0000012888**	ELL3	protein_coding	chr15	43772605	43777315	-	Human GRCh	OTTHUMG000000	ELL3	**elongation factor for RNA polymerase II 3**	Nucleus	transcription		-1.02860993	0	0.90617175	0	1	0	8.93169651	9.28324516	9.19984712	8.96475268
6002	**ENSG0000012889**	CCDC32	protein_coding	chr15	40528683	40565054	-	Human GRCh	OTTHUMG000001	CCDC32	**coiled-coil domain containing 32**	Other	other		-1.035608411	0	0.87717966	0	1	0	9.06569891	9.27204107	9.17703706	9.09005378
6003	**ENSG0000012890**	INO80	protein_coding	chr15	40978880	41116280	-	Human GRCh	OTTHUMG000001	INO80	**INO80 complex ATPase subunit**	Nucleus	enzyme		-1.086184158	0	0.68142387	0	1	0	11.2421736	11.5137356	11.3818824	11.1965019
6004	**ENSG0000012891**	ICE2	protein_coding	chr15	60419609	60479160	-	Human GRCh	OTTHUMG000001	ICE2	**interactor of little elongation complex ELL subunit 2**	Nucleus	other		-1.137992613	0	0.61510881	0	1	0	11.2402085	11.4496496	11.4772578	10.8528179
6005	**ENSG0000012891**	DLL4	protein_coding	chr15	40929340	40939073	+	Human GRCh	OTTHUMG000001	DLL4	**delta like canonical Notch ligand 4**	Extracellular	other	CTX-009, C	-1.787163606	-1	0.29531202	0	1	0	5.31185532	4.42008037	4.91131248	3.70679848
6006	**ENSG0000012891**	ALDH1A2	protein_coding	chr15	57953424	58497866	-	Human GRCh	OTTHUMG000001	ALDH1A2	**aldehyde dehydrogenase 1 family member A2**	Cytoplasm	enzyme		-1.457013299	0	0.55594435	0	1	0	4.370957	4.95442234	3.78795794	4.73824847
6007	**ENSG0000012892**	MINDY2	protein_coding	chr15	58771192	58861900	+	Human GRCh	OTTHUMG000001	MINDY2	**MINDY lysine 48 deubiquitinase 2**	Nucleus	transporter		1.169778772	0	0.49197691	0	1	0	10.3752744	10.6159784	10.8362735	10.492593
6008	**ENSG0000012892**	IVD	protein_coding	chr15	40405485	40435947	+	Human GRCh	OTTHUMG000001	IVD	**isovaleryl-CoA dehydrogenase**	Cytoplasm	enzyme		-1.128464973	0	0.6106011	0	1	0	11.6937698	12.1058295	11.918568	11.6171447
6009	**ENSG0000012894**	KNSTRN	protein_coding	chr15	40382721	40394288	+	Human GRCh	OTTHUMG000001	KNSTRN	**kinetochore localized astrin (SPAG5) binding protein**	Cytoplasm	other		-1.152396337	0	0.56772428	0	1	0	11.072014	11.3356571	11.155046	10.9316636
6010	**ENSG0000012895**	DUT	protein_coding	chr15	48331011	48343373	+	Human GRCh	OTTHUMG000001	DUT	**deoxyuridine triphosphatase**	Nucleus	enzyme	TAS-114	1.156218494	0	0.55681023	0	1	0	10.3015187	10.5589341	10.7199741	10.4616695
6011	**ENSG0000012896**	CHAC1	protein_coding	chr15	40952962	40956512	+	Human GRCh	OTTHUMG000001	CHAC1	**ChaC glutathione specific gamma-glutamylcyclotransferase 1**	Cytoplasm	enzyme		-1.431836549	0	0.35508221	0	1	0	7.2437844	6.59964342	6.70945276	6.46075893
6012	**ENSG0000012897**	CLN6	protein_coding	chr15	68206992	68257211	-	Human GRCh	OTTHUMG000001	CLN6	**CLN6 transmembrane ER protein**	Cytoplasm	other		-1.032026804	0	0.88339003	0	1	0	11.1005969	11.3713312	11.3908733	10.9923375
6013	**ENSG0000012898**	ARPP19	protein_coding	chr15	52547045	52569883	-	Human GRCh	OTTHUMG000001	ARPP19	**cAMP regulated phosphoprotein 19**	Cytoplasm	transporter		-1.055436198	0	0.79585612	0	1	0	12.5245711	12.7528088	12.76756	12.3665246
6014	**ENSG0000012900**	VPS13C	protein_coding	chr15	61852389	62060473	-	Human GRCh	OTTHUMG000001	VPS13C	**vacuolar protein sorting 13 homolog C**	Cytoplasm	other		-1.083561711	0	0.71227972	0	1	0	11.8885845	12.0728016	12.1351992	11.5979601
6015	**ENSG0000012900**	CALML4	protein_coding	chr15	68190705	68206079	-	Human GRCh	OTTHUMG000001	CALML4	**calmodulin like 4**	Other	other		1.265055968	0	0.41549936	0	1	0	8.5001678	8.76231036	9.19438454	8.49252051
6016	**ENSG0000012900**	ISLR	protein_coding	chr15	74173710	74176872	+	Human GRCh	OTTHUMG000001	ISLR	**immunoglobulin superfamily containing leucine rich repeat**	Extracellular	other		1	0	1	0	1	0	0.62383003	0.75632613	0.73547908	0.6320577
6017	**ENSG0000012902**	THAP10	protein_coding	chr15	70881342	70892433	-	Human GRCh	OTTHUMG000001	THAP10	**THAP domain containing 10**	Other	other		1.237698794	0	0.54507413	0	1	0	6.35119479	6.57114414	6.80471219	6.54254676
6018	**ENSG0000012903**	LOXL1	protein_coding	chr15	73925989	73952137	+	Human GRCh	OTTHUMG000001	LOXL1	**lysyl oxidase like 1**	Extracellular	enzyme		-1.588091927	-1	0.31890924	0	1	0	4.72129018	4.52666523	4.6403334	3.79953008
6020	**ENSG0000012905**	ANAPC13	protein_coding	chr3	134477706	134486716	-	Human GRCh	OTTHUMG000001	ANAPC13	**anaphase promoting complex subunit 13**	Nucleus	other		1.019515247	0	0.9324287	0	1	0	9.37223375	9.80579212	9.67298498	9.58568025
6021	**ENSG0000012907**	MBD4	protein_coding	chr3	129430947	129440179	-	Human GRCh	OTTHUMG000001	MBD4	**methyl-CpG binding domain 4, DNA glycosylase**	Nucleus	enzyme		-1.162008687	0	0.45574879	0	1	0	9.78890985	10.2064081	9.89684609	9.8020635
6022	**ENSG0000012908**	COPB1	protein_coding	chr11	14443440	14500027	-	Human GRCh	OTTHUMG000001	COPB1	**COPI coat complex subunit beta 1**	Cytoplasm	transporter		-1.153652691	0	0.55426344	0	1	0	11.6384832	12.0104571	11.7950759	11.550268
6023	**ENSG0000012908**	PSMA1	protein_coding	chr11	14504874	14643635	-	Human GRCh	OTTHUMG000001	PSMA1	**proteasome 20S subunit alpha 1**	Cytoplasm	peptidase		-1.07303651	0	0.75015079	0	1	0	10.8364106	11.1837977	11.0742604	10.8002897
6024	**ENSG0000012910**	SUMF2	protein_coding	chr7	56064002	56080670	+	Human GRCh	OTTHUMG000001	SUMF2	**sulfatase modifying factor 2**	Cytoplasm	other		-1.140367035	0	0.57331074	0	1	0	11.5417317	11.8013303	11.6822968	11.363513
6025	**ENSG0000012911**	PALLD	protein_coding	chr4	168497052	168928457	+	Human GRCh	OTTHUMG000001	PALLD	**palladin, cytoskeletal associated protein**	Plasma Mem	other		1.166780097	0	0.64353333	0	1	0	8.94087299	9.00038035	8.8893771	9.32749153
6026	**ENSG0000012912**	SPCS3	protein_coding	chr4	176319966	176332245	+	Human GRCh	OTTHUMG000001	SPCS3	**signal peptidase complex subunit 3**	Cytoplasm	peptidase		-1.010343283	0	0.96302608	0	1	0	11.9435829	12.0680893	12.1666666	11.7962953
6028	**ENSG0000012915**	MYOD1	protein_coding	chr11	17719571	17722136	+	Human GRCh	OTTHUMG000001	MYOD1	**myogenic differentiation 1**	Nucleus	transcription		1.434182662	0	0.59582031	0	1	0	4.99582085	5.3865957	4.62436946	6.07566413
6029	**ENSG0000012915**	SERGEF	protein_coding	chr11	17788048	18013047	-	Human GRCh	OTTHUMG000001	SERGEF	**secretion regulating guanine nucleotide exchange factor**	Nucleus	other		1.137208857	0	0.61997026	0	1	0	8.72469833	9.11438237	9.12747803	9.01322143
6030	**ENSG0000012915**	KCNC1	protein_coding	chr11	17734774	17856804	+	Human GRCh	OTTHUMG000001	KCNC1	**potassium voltage-gated channel subfamily C member 1**	Plasma Mem	ion channel	dalfampric	1.639179889	1	0.30184694	0	1	0	4.1859793	4.33945623	4.50226299	4.66299502
6031	**ENSG0000012916**	TPH1	protein_coding	chr11	18017555	18046269	-	Human GRCh	OTTHUMG000001	TPH1	**tryptophan hydroxylase 1**	Nucleus	enzyme	telotristat,	-1.141643813	0	0.64370808	0	1	0	7.37708124	7.36630788	7.29674386	7.17079726
6033	**ENSG0000012917**	E2F8	protein_coding	chr11	19224063	19241620	-	Human GRCh	OTTHUMG000001	E2F8	**E2F transcription factor 8**	Nucleus	transcription		-1.121067035	0	0.6594587	0	1	0	9.22121276	9.85264901	9.5046651	9.4085693
6034	**ENSG0000012918**	DCTD	protein_coding	chr4	182890060	182917936	-	Human GRCh	OTTHUMG000001	DCTD	**dCMP deaminase**	Cytoplasm	enzyme	tetrahydro	-1.210985521	0	0.36037455	0	1	0	10.3451755	10.5932156	10.414081	10.0873659
6035	**ENSG0000012919**	SOX15	protein_coding	chr17	7588178	7590094	-	Human GRCh	OTTHUMG000001	SOX15	**SRY-box transcription factor 15**	Nucleus	transcription		-3.265942771	-1	0.135259	0	1	0	4.183396	4.04277394	2.43871329	3.72324266
6036	**ENSG0000012919**	PIMREG	protein_coding	chr17	6444441	6451469	+	Human GRCh	OTTHUMG000001	PIMREG	**PICALM interacting mitotic regulator**	Nucleus	other		1.043060616	0	0.865819	0	1	0	9.24063249	9.49921458	9.51314034	9.32440743
6037	**ENSG0000012919**	RPAIN	protein_coding	chr17	5419641	5432877	+	Human GRCh	OTTHUMG000001	RPAIN	**RPA interacting protein**	Nucleus	other		-1.213546672	0	0.36288452	0	1	0	10.270259	10.5730358	10.3421684	10.0841943
6040	**ENSG0000012921**	PLD2	protein_coding	chr17	4807152	4823434	+	Human GRCh	OTTHUMG000000	PLD2	**phospholipase D2**	Cytoplasm	enzyme		-1.003015296	0	0.99029362	0	1	0	9.06291977	9.20008261	9.35622167	8.85012912
6042	**ENSG0000012922**	CD68	protein_coding	chr17	7579491	7582111	+	Human GRCh	OTTHUMG000001	CD68	**CD68 molecule**	Plasma Mem	other		2.026158036	1	0.05315382	0	1	0	5.86730292	6.26222101	6.44410392	7.08611178
6043	**ENSG0000012923**	TXNDC17	protein_coding	chr17	6640985	6644541	+	Human GRCh	OTTHUMG000001	TXNDC17	**thioredoxin domain containing 17**	Cytoplasm	enzyme		1.09990419	0	0.73002085	0	1	0	8.43322856	9.04746812	9.01516996	8.73582226
6044	**ENSG0000012924**	ATP1B2	protein_coding	chr17	7646627	7657770	+	Human GRCh	OTTHUMG000001	ATP1B2	**ATPase Na+/K+ transporting subunit beta 2**	Plasma Mem	transporter		-1.478312358	0	0.66538368	0	NA	0	2.90796938	2.81346172	2.95436836	2.14517846
6045	**ENSG0000012924**	FXR2	protein_coding	chr17	7591230	7614897	-	Human GRCh	OTTHUMG000001	FXR2	**FMR1 autosomal homolog 2**	Cytoplasm	other		-1.00474517	0	0.98146663	0	1	0	11.0265473	11.3752022	11.2478315	11.1673513
6046	**ENSG0000012925**	KIF1C	protein_coding	chr17	4997950	5028401	+	Human GRCh	OTTHUMG000000	KIF1C	**kinesin family member 1C**	Cytoplasm	other		1.106408851	0	0.68854101	0	1	0	12.3899708	12.5893038	12.7267274	12.4797273
6047	**ENSG0000012925**	MPDU1	protein_coding	chr17	7583529	7592789	+	Human GRCh	OTTHUMG000001	MPDU1	**mannose-P-dolichol utilization defect 1**	Cytoplasm	other		-1.152179486	0	0.49909967	0	1	0	10.2897971	10.6636017	10.3894683	10.2701559
6048	**ENSG0000012929**	PHF20L1	protein_coding	chr8	132775358	132848807	+	Human GRCh	OTTHUMG000001	PHF20L1	**PHD finger protein 20 like 1**	Nucleus	other		1.030650365	0	0.89169198	0	1	0	10.6199422	10.712531	10.9119903	10.438715
6049	**ENSG0000012929**	DNAAF11	protein_coding	chr8	132570416	132675592	-	Human GRCh	OTTHUMG000001	DNAAF11	**dynein axonemal assembly factor 11**	Cytoplasm	other		-1.073734562	0	0.8529023	0	1	0	6.68854273	7.12211704	7.14348209	6.45232855
6050	**ENSG0000012931**	CCNT1	protein_coding	chr12	48688458	48716998	-	Human GRCh	OTTHUMG000001	CCNT1	**cyclin T1**	Nucleus	transcription		-1.107528945	0	0.64244082	0	1	0	11.2530069	11.4363444	11.3962876	11.0452877
6051	**ENSG0000012931**	PUS7L	protein_coding	chr12	43718992	43758793	-	Human GRCh	OTTHUMG000001	PUS7L	**pseudouridine synthase 7 like**	Nucleus	enzyme		1.102398626	0	0.67379767	0	1	0	11.0311255	11.348171	11.5115776	11.0655253
6052	**ENSG0000012934**	KRI1	protein_coding	chr19	10553085	10566031	-	Human GRCh	OTTHUMG000001	KRI1	**KRI1 homolog**	Nucleus	other		1.173869543	0	0.43481677	0	1	0	10.5376179	10.8028152	11.0049454	10.6816072
6053	**ENSG0000012935**	ILF3	protein_coding	chr19	10654304	10692400	+	Human GRCh	OTTHUMG000001	ILF3	**interleukin enhancer binding factor 3**	Nucleus	transcription		-1.1359937	0	0.50263253	0	1	0	14.0827295	14.3714258	14.2215548	13.9547097
6054	**ENSG0000012935**	SLC44A2	protein_coding	chr19	10602457	10644557	+	Human GRCh	OTTHUMG000001	SLC44A2	**solute carrier family 44 member 2**	Extracellular	transporter		-1.206133734	0	0.38608365	0	1	0	11.5729385	11.9541811	11.6762112	11.4579271
6055	**ENSG0000012935**	AP1M2	protein_coding	chr19	10572671	10587315	-	Human GRCh	OTTHUMG000001	AP1M2	**adaptor related protein complex 1 subunit mu 2**	Cytoplasm	transporter		-1.066547523	0	0.7430664	0	1	0	10.7817738	11.1705906	11.047903	10.7758798
6056	**ENSG0000012935**	CDKN2D	protein_coding	chr19	10566460	10569059	-	Human GRCh	OTTHUMG000001	CDKN2D	**cyclin dependent kinase inhibitor 2D**	Cytoplasm	transcription		1.102100616	0	0.73522636	0	1	0	8.27780181	8.77023221	8.79762659	8.48939082
6057	**ENSG0000012942**	MTUS1	protein_coding	chr8	17643795	17801094	-	Human GRCh	OTTHUMG000001	MTUS1	**microtubule associated scaffold protein 1**	Extracellular	other		1.255383156	0	0.43058975	0	1	0	8.08080528	8.23344159	8.15741479	8.58266372
6058	**ENSG0000012943**	KLK14	protein_coding	chr19	51077495	51084245	-	Human GRCh	OTTHUMG000001	KLK14	**kallikrein related peptidase 14**	Extracellular	peptidase	MDPK67b	-1.593584764	-1	0.41456962	0	1	0	6.59909422	6.54280235	6.07063665	6.17242348
6060	**ENSG0000012945**	KLK10	protein_coding	chr19	51012739	51020175	-	Human GRCh	OTTHUMG000001	KLK10	**kallikrein related peptidase 10**	Extracellular	peptidase		3.15144965	1	0.16110498	0	1	0	2.68607927	3.14978359	3.38597763	3.9032305
6061	**ENSG0000012945**	KLK8	protein_coding	chr19	50996007	51002711	-	Human GRCh	OTTHUMG000001	KLK8	**kallikrein related peptidase 8**	Extracellular	peptidase		11.54459344	1	0.21951731	0	NA	0	0.29622082	0.31354789	1.17903451	0.29723943
6062	**ENSG0000012946**	NGDN	protein_coding	chr14	23469679	23509862	+	Human GRCh	OTTHUMG000001	NGDN	**neuroguidin**	Cytoplasm	other		-1.090414772	0	0.6832604	0	1	0	9.26076686	9.49947233	9.470551	9.07958007
6064	**ENSG0000012946**	ADCY4	protein_coding	chr14	24318349	24335093	-	Human GRCh	OTTHUMG000000	ADCY4	**adenylate cyclase 4**	Plasma Mem	enzyme		-1.253123745	0	0.68354733	0	1	0	4.08438145	4.41646618	4.61107669	2.99277587
6065	**ENSG0000012947**	RAB2B	protein_coding	chr14	21459020	21476960	-	Human GRCh	OTTHUMG000000	RAB2B	**RAB2B, member RAS oncogene family**	Plasma Mem	enzyme		-1.13075111	0	0.54729861	0	1	0	9.43476264	9.55519553	9.46971294	9.25122468
6066	**ENSG0000012947**	BCL2L2	protein_coding	chr14	23298790	23311751	+	Human GRCh	OTTHUMG000000	BCL2L2	**BCL2 like 2**	Cytoplasm	other	ABT-737, r	1.081314781	0	0.72470088	0	1	0	10.4134703	10.5332175	10.6702316	10.44104
6067	**ENSG0000012947**	AJUBA	protein_coding	chr14	22971177	22982551	-	Human GRCh	OTTHUMG000000	AJUBA	**ajuba LIM protein**	Nucleus	transcription		1.374617465	0	0.29403976	0	1	0	7.70865468	7.45480215	8.21708643	7.53721817
6068	**ENSG0000012948**	DTD2	protein_coding	chr14	31446036	31457506	-	Human GRCh	OTTHUMG000001	DTD2	**D-aminoacyl-tRNA deacylase 2**	Other	enzyme		-1.031825541	0	0.90356043	0	1	0	8.56097166	8.85605556	8.85799846	8.47692235
6069	**ENSG0000012948**	PARP2	protein_coding	chr14	20343615	20357904	+	Human GRCh	OTTHUMG000001	PARP2	**poly(ADP-ribose) polymerase 2**	Nucleus	enzyme	talazoparik	-1.136755203	0	0.50747865	0	1	0	10.1008595	10.4230596	10.1476597	10.117702
6070	**ENSG0000012949**	HEATR5A	protein_coding	chr14	31291788	31420550	-	Human GRCh	OTTHUMG000001	HEATR5A	**HEAT repeat containing 5A**	Other	other		1.005105092	0	0.98201121	0	1	0	10.3021519	10.553673	10.6535215	10.1794376
6071	**ENSG0000012951**	FOXA1	protein_coding	chr14	37589552	37596059	-	Human GRCh	OTTHUMG000001	FOXA1	**forkhead box A1**	Nucleus	transcription		-1.08524923	0	0.71733971	0	1	0	13.4537976	13.7109529	13.5472593	13.4437959
6072	**ENSG0000012951**	SNX6	protein_coding	chr14	34561093	34630160	-	Human GRCh	OTTHUMG000001	SNX6	**sorting nexin 6**	Cytoplasm	transporter		-1.010819997	0	0.95425631	0	1	0	9.97349079	10.165568	10.2143477	9.88614874
6073	**ENSG0000012951**	EAPP	protein_coding	chr14	34515938	34539704	-	Human GRCh	OTTHUMG000001	EAPP	**E2F associated phosphoprotein**	Cytoplasm	other		-1.247603577	0	0.34246542	0	1	0	9.70800001	10.216908	9.81950326	9.6639079
6074	**ENSG0000012952**	EGLN3	protein_coding	chr14	33924227	34462774	-	Human GRCh	OTTHUMG000000	EGLN3	**egl-9 family hypoxia inducible factor 3**	Cytoplasm	enzyme	molidustat	1.07866368	0	0.83716109	0	1	0	7.72334145	8.1246934	7.99424526	8.04855198
6075	**ENSG0000012953**	MIS18BP1	protein_coding	chr14	45203190	45253540	-	Human GRCh	OTTHUMG000001	MIS18BP1	**MIS18 binding protein 1**	Nucleus	other		-1.099302575	0	0.63595283	0	1	0	10.7536658	11.053448	10.9673075	10.6334937
6076	**ENSG0000012953**	NRL	protein_coding	chr14	24078662	24115010	-	Human GRCh	OTTHUMG000000	NRL	**neural retina leucine zipper**	Nucleus	transcription		-1.399738585	0	0.28254334	0	1	0	6.3485634	7.11371588	6.64257385	6.2198248
6077	**ENSG0000012953**	RNASE1	protein_coding	chr14	20801228	20802855	-	Human GRCh	OTTHUMG000000	RNASE1	**ribonuclease A family member 1, pancreatic**	Extracellular	enzyme		1.229322251	0	0.84085113	0	1	0	3.76598639	4.45850824	4.93632036	3.31070743
6078	**ENSG0000012955**	NEDD8	protein_coding	chr14	24216857	24232367	-	Human GRCh	OTTHUMG000000	NEDD8	**NEDD8 ubiquitin like modifier**	Nucleus	enzyme		-1.001946242	0	0.99244738	0	1	0	10.8496839	11.2310068	11.1623681	10.9330838

6079	**ENSG0000012956**	DAD1	protein_coding	chr14	22564907	22589224	-	Human GRCh	OTTHUMG000000	DAD1	**defender against cell death 1**	Cytoplasm	other		-1.054669632	0	0.81626335	0	1	0	10.3293611	10.6534779	10.4979482	10.3853805
6080	**ENSG0000012956**	TEP1	protein_coding	chr14	20365667	20413501	-	Human GRCh	OTTHUMG000000	TEP1	**telomerase associated protein 1**	Nucleus	enzyme		-1.173501855	0	0.39615627	0	1	0	9.98808692	10.1712598	9.9536133	9.86496534
6081	**ENSG0000012959**	EPB41L4A	protein_coding	chr5	112142441	112419313	-	Human GRCh	OTTHUMG000001	EPB41L4A	**erythrocyte membrane protein band 4.1 like 4A**	Extracellular	other		-6.158675301	-1	0.00045454	1	0.4	0	5.37688373	4.48993498	4.05235017	3.67185656
6083	**ENSG0000012962**	REEP5	protein_coding	chr5	112876385	112922289	-	Human GRCh	OTTHUMG000001	REEP5	**receptor accessory protein 5**	Extracellular	transporter		-1.07589481	0	0.74621678	0	1	0	10.4519039	10.7116152	10.6107178	10.3905119
6084	**ENSG0000012963**	ITFG1	protein_coding	chr16	47154387	47464149	-	Human GRCh	OTTHUMG000001	ITFG1	**integrin alpha FG-GAP repeat containing 1**	Plasma Mem	other		-1.138490583	0	0.55011882	0	1	0	9.88105446	10.2103557	10.063043	9.7432791
6085	**ENSG0000012964**	QRICH2	protein_coding	chr17	76274049	76308276	-	Human GRCh	OTTHUMG000001	QRICH2	**glutamine rich 2**	Extracellular	other		1.087733672	0	0.76884692	0	1	0	9.07267888	9.31167472	9.49057726	9.05364984
6086	**ENSG0000012965**	FOXJ1	protein_coding	chr17	76136333	76141245	-	Human GRCh	OTTHUMG000001	FOXJ1	**forkhead box J1**	Nucleus	transcription		1.192613483	0	0.43972954	0	1	0	9.46632938	9.68620655	9.90015226	9.6324954
6087	**ENSG0000012965**	SEC14L1	protein_coding	chr17	77088749	77217101	+	Human GRCh	OTTHUMG000001	SEC14L1	**SEC14 like lipid binding 1**	Cytoplasm	transporter		-1.113047836	0	0.61860087	0	1	0	10.367197	10.6717129	10.5408771	10.2659429
6088	**ENSG0000012966**	RHBDF2	protein_coding	chr17	76470891	76501790	-	Human GRCh	OTTHUMG000001	RHBDF2	**rhomboid 5 homolog 2**	Plasma Mem	other		1.114542325	0	0.63466239	0	1	0	9.29070231	9.33501018	9.59547577	9.25221978
6090	**ENSG0000012967**	ARHGEF6	protein_coding	chrX	136665547	136780932	-	Human GRCh	OTTHUMG000000	ARHGEF6	**Rac/Cdc42 guanine nucleotide exchange factor 6**	Cytoplasm	other		-1.014884287	0	0.97626104	0	1	0	5.62668788	5.28740026	5.61817594	5.2724575
6091	**ENSG0000012968**	MAP7D3	protein_coding	chrX	136213220	136256482	-	Human GRCh	OTTHUMG000000	MAP7D3	**MAP7 domain containing 3**	Cytoplasm	other		1	0	1	0	1	0	2.13815707	2.23021507	2.21538259	2.14371988
6092	**ENSG0000012968**	FGF13	protein_coding	chrX	138614727	139222777	-	Human GRCh	OTTHUMG000000	FGF13	**fibroblast growth factor 13**	Extracellular	growth facto		1.086869165	0	0.71193183	0	1	0	8.50784362	8.67367503	8.71721809	8.65150098
6093	**ENSG0000012969**	ASH2L	protein_coding	chr8	38105493	38144076	+	Human GRCh	OTTHUMG000001	ASH2L	**ASH2 like, histone lysine methyltransferase complex subunit**	Nucleus	transcription		-1.145143167	0	0.50138478	0	1	0	10.2237711	10.5881062	10.3504344	10.1856343
6094	**ENSG0000012969**	TTI2	protein_coding	chr8	33473386	33513185	-	Human GRCh	OTTHUMG000001	TTI2	**TELO2 interacting protein 2**	Cytoplasm	other		-1.283889222	0	0.24525213	0	1	0	8.88378027	9.09362992	8.81209174	8.61616626
6095	**ENSG0000012974**	ART1	protein_coding	chr11	3645128	3664416	+	Human GRCh	OTTHUMG000000	ART1	**ADP-ribosyltransferase 1**	Plasma Mem	enzyme		1	0	1	0	1	0	1.43356781	1.48683984	1.47816441	1.43674691
6096	**ENSG0000012974**	CHRNA10	protein_coding	chr11	3665587	3671384	-	Human GRCh	OTTHUMG000000	CHRNA10	**cholinergic receptor nicotinic alpha 10 subunit**	Plasma Mem	transmembra	succinylch	-1.268058411	0	0.51657082	0	1	0	7.12249364	7.05798847	7.09855394	6.51298298
6097	**ENSG0000012975**	CDKN1C	protein_coding	chr11	2883213	2885775	-	Human GRCh	OTTHUMG000000	CDKN1C	**cyclin dependent kinase inhibitor 1C**	Nucleus	other		1.272130987	0	0.43947201	0	1	0	7.49487829	7.86307896	7.85531056	8.04591226
6098	**ENSG0000012981**	SGO1	protein_coding	chr3	20160593	20186206	-	Human GRCh	OTTHUMG000001	SGO1	**shugoshin 1**	Nucleus	other		1.098330217	0	0.67066551	0	1	0	9.21293444	9.56627841	9.71690048	9.24402289
6100	**ENSG0000012982**	RPS4Y1	protein_coding	chrY	2841602	2932000	+	Human GRCh	OTTHUMG000000	RPS4Y1	**ribosomal protein S4 Y-linked 1**	Cytoplasm	other		-1.032445072	0	0.89483516	0	1	0	10.9558931	11.2040887	11.2147251	10.8535463
6105	**ENSG0000012991**	CDH15	protein_coding	chr16	89171748	89195492	+	Human GRCh	OTTHUMG000001	CDH15	**cadherin 15**	Plasma Mem	other		1.016445648	0	0.98466215	0	1	0	6.82271748	6.99050083	6.73002107	7.09774472
6106	**ENSG0000012991**	KLF16	protein_coding	chr19	1852399	1863579	-	Human GRCh	OTTHUMG000001	KLF16	**KLF transcription factor 16**	Nucleus	transcription		1.440122349	0	0.11550649	0	1	0	10.2015608	10.3405719	10.8099035	10.5412833
6107	**ENSG0000012992**	PGAP6	protein_coding	chr16	370788	387113	-	Human GRCh	OTTHUMG000000	PGAP6	**post-GPI attachment to proteins 6**	Plasma Mem	other		-1.046414237	0	0.89825488	0	1	0	13.1341049	13.3604242	13.3116279	13.0763306
6108	**ENSG0000012993**	DOHH	protein_coding	chr19	3490821	3500674	-	Human GRCh	OTTHUMG000001	DOHH	**deoxyhypusine hydroxylase**	Cytoplasm	enzyme		1.325652955	0	0.19968907	0	1	0	8.49019761	8.7027577	9.06429108	8.73938616
6109	**ENSG0000012993**	MAU2	protein_coding	chr19	19320829	19358754	+	Human GRCh	OTTHUMG000001	MAU2	**MAU2 sister chromatid cohesion factor**	Nucleus	other		-1.177296145	0	0.43024433	0	1	0	11.499307	11.8108634	11.6045598	11.3511577
6110	**ENSG0000012994**	SHC2	protein_coding	chr19	416589	461033	-	Human GRCh	OTTHUMG000001	SHC2	**SHC adaptor protein 2**	Cytoplasm	other		-1.208896193	0	0.71906482	0	1	0	7.82677965	7.6454982	7.37600125	7.68810683
6111	**ENSG0000012995**	PLPPR3	protein_coding	chr19	812488	821955	-	Human GRCh	OTTHUMG000001	PLPPR3	**phospholipid phosphatase related 3**	Other	phosphatase		-1.120834134	0	0.89204867	0	1	0	3.89309755	4.14742211	4.20214362	3.54205586
6112	**ENSG0000012996**	ABHD17A	protein_coding	chr19	1876810	1885496	-	Human GRCh	OTTHUMG000001	ABHD17A	**abhydrolase domain containing 17A, depalmitoylase**	Plasma Mem	enzyme		1.175185796	0	0.47608014	0	1	0	10.501887	10.7252324	10.8795491	10.7113599
6114	**ENSG0000012999**	SYT5	protein_coding	chr19	55171196	55180289	-	Human GRCh	OTTHUMG000001	SYT5	**synaptotagmin 5**	Cytoplasm	transporter		1.274355528	0	0.53061798	0	1	0	6.53462887	7.01988574	7.09300795	7.02843825
6115	**ENSG0000012999**	TNNI3	protein_coding	chr19	55151767	55157773	-	Human GRCh	OTTHUMG000001	TNNI3	**troponin I3, cardiac type**	Cytoplasm	transporter		1.356725325	0	0.52034997	0	1	0	5.69352723	5.90714542	6.39387539	5.76306504
6116	**ENSG0000012999**	CBFA2T3	protein_coding	chr16	88874858	88977207	-	Human GRCh	OTTHUMG000001	CBFA2T3	**CBFA2/RUNX1 partner transcriptional co-repressor 3**	Nucleus	transcription		-1.045076842	0	0.90501942	0	1	0	5.66032807	5.60837784	5.69670335	5.48549441
6117	**ENSG0000013000**	GAMT	protein_coding	chr19	1397026	1401570	-	Human GRCh	OTTHUMG000001	GAMT	**guanidinoacetate N-methyltransferase**	Cytoplasm	enzyme		1.080231367	0	0.78229937	0	1	0	10.2290502	10.3820967	10.4785099	10.3065316
6118	**ENSG0000013002**	PUDP	protein_coding	chrX	6667865	7148158	-	Human GRCh	OTTHUMG000000	PUDP	**pseudouridine 5'-phosphatase**	Cytoplasm	enzyme		-1.033216832	0	0.89116352	0	1	0	8.32477909	8.72619874	8.62131859	8.37907613
6119	**ENSG0000013002**	ERMARD	protein_coding	chr6	169751622	169781600	+	Human GRCh	OTTHUMG000000	ERMARD	**ER membrane associated RNA degradation**	Other	other		1.02279609	0	0.91997927	0	1	0	8.93179678	9.39776903	9.32863723	9.08241107
6120	**ENSG0000013002**	PHF10	protein_coding	chr6	169703902	169725566	-	Human GRCh	OTTHUMG000000	PHF10	**PHD finger protein 10**	Nucleus	other		1.057086669	0	0.80339863	0	1	0	10.4892391	10.7984109	10.9379979	10.4357706
6122	**ENSG0000013003**	GALNT8	protein_coding	chr12	4720400	4851927	+	Human GRCh	OTTHUMG000001	GALNT8	**polypeptide N-acetylgalactosaminyltransferase 8**	Cytoplasm	enzyme		1.133110984	0	0.88718528	0	1	0	3.1266216	3.50713432	3.95729117	2.31278729
6124	**ENSG0000013003**	CRACR2A	protein_coding	chr12	3606633	3764819	-	Human GRCh	OTTHUMG000001	CRACR2A	**calcium release activated channel regulator 2A**	Cytoplasm	enzyme		-1.014510263	0	0.9898666	0	1	0	2.58769721	3.16911053	3.14980007	2.59226975
6126	**ENSG0000013005**	STARD8	protein_coding	chrX	68647666	68725842	+	Human GRCh	OTTHUMG000000	STARD8	**StAR related lipid transfer domain containing 8**	Cytoplasm	other		-2.576335267	-1	0.0333393	1	1	0	4.88429453	5.08068882	4.0789136	4.24120959
6127	**ENSG0000013005**	NALF2	protein_coding	chrX	69504326	69532508	+	Human GRCh	OTTHUMG000000	NALF2	**NALCN channel auxiliary factor 2**	Plasma Mem	transmembra		1.813540973	1	0.02977078	1	1	0	6.02325013	5.7912514	6.3637644	6.6230697
6128	**ENSG0000013005**	GDPD2	protein_coding	chrX	70423031	70433390	+	Human GRCh	OTTHUMG000000	GDPD2	**glycerophosphodiester phosphodiesterase domain containing**	Plasma Mem	enzyme		-1.933958021	-1	0.40940102	0	NA	0	3.49185295	2.9303634	2.91264343	2.8279734
6129	**ENSG0000013006**	SAT1	protein_coding	chrX	23783173	23786210	+	Human GRCh	OTTHUMG000000	SAT1	**spermidine/spermine N1-acetyltransferase 1**	Cytoplasm	enzyme		1.203575638	0	0.47870325	0	1	0	8.84268825	9.02870396	9.23329221	9.03333927
6130	**ENSG0000013011**	GNL3L	protein_coding	chrX	54530183	54621521	+	Human GRCh	OTTHUMG000000	GNL3L	**G protein nucleolar 3 like**	Nucleus	other		-1.131605409	0	0.525228	0	1	0	11.9060178	12.2321045	12.110589	11.7481023
6131	**ENSG0000013014**	SH3BP4	protein_coding	chr2	234952017	235055714	+	Human GRCh	OTTHUMG000001	SH3BP4	**SH3 domain binding protein 4**	Cytoplasm	other		-1.014672909	0	0.95574108	0	1	0	10.5471864	10.5785748	10.5243619	10.5691877
6132	**ENSG0000013015**	MOSPD2	protein_coding	chrX	14873421	14922327	+	Human GRCh	OTTHUMG000000	MOSPD2	**motile sperm domain containing 2**	Plasma Mem	other		-1.010443615	0	0.96376866	0	1	0	8.33048578	8.69620148	8.55826649	8.47161468
6133	**ENSG0000013015**	DOCK6	protein_coding	chr19	11199295	11262524	-	Human GRCh	OTTHUMG000001	DOCK6	**dedicator of cytokinesis 6**	Cytoplasm	other		-1.15536075	0	0.629624	0	1	0	10.6781006	10.9805086	10.7336452	10.6266918
6134	**ENSG0000013015**	ECSIT	protein_coding	chr19	11505929	11529172	-	Human GRCh	OTTHUMG000001	ECSIT	**ECSIT signaling integrator**	Nucleus	transcription		-1.162825941	0	0.42289107	0	1	0	10.3980861	10.7124138	10.4661785	10.3280727
6135	**ENSG0000013016**	LDLR	protein_coding	chr19	11089418	11133820	+	Human GRCh	OTTHUMG000001	LDLR	**low density lipoprotein receptor**	Plasma Mem	transporter	iodine I 13	-1.109452226	0	0.72458935	0	1	0	12.0920478	12.6677975	12.3904532	12.1917618
6136	**ENSG0000013016**	ELOF1	protein_coding	chr19	11551147	11559230	-	Human GRCh	OTTHUMG000001	ELOF1	**elongation factor 1**	Nucleus	other		1.120649916	0	0.59298756	0	1	0	9.77543945	9.96189261	10.1193739	9.86554617
6139	**ENSG0000013017**	PRKCSH	protein_coding	chr19	11435284	11450968	+	Human GRCh	OTTHUMG000001	PRKCSH	**protein kinase C substrate 80K-H**	Cytoplasm	enzyme		1.02740275	0	0.89054256	0	1	0	12.9625438	13.2409429	13.2794317	12.9819674
6141	**ENSG0000013017**	CDC16	protein_coding	chr13	114234887	114272723	+	Human GRCh	OTTHUMG000000	CDC16	**cell division cycle 16**	Nucleus	other		-1.118151432	0	0.61048319	0	1	0	10.4492886	10.6662372	10.5721595	10.2968239
6142	**ENSG0000013018**	ZSCAN10	protein_coding	chr16	3088890	3099295	-	Human GRCh	OTTHUMG000001	ZSCAN10	**zinc finger and SCAN domain containing 10**	Nucleus	transcription		-1.847973815	-1	0.68365994	0	NA	0	1.99454064	2.54278468	1.68779063	2.25479619
6143	**ENSG0000013019**	THEM6	protein_coding	chr8	142727223	142736927	+	Human GRCh	OTTHUMG000001	THEM6	**thioesterase superfamily member 6**	Other	other		1.117451423	0	0.6310844	0	1	0	10.6144795	10.8213761	10.9471235	10.7352004
6144	**ENSG0000013020**	NECTIN2	protein_coding	chr19	44846175	44889223	+	Human GRCh	OTTHUMG000001	NECTIN2	**nectin cell adhesion molecule 2**	Plasma Mem	transmembra		-1.06861495	0	0.77765799	0	1	0	9.9571762	10.0870121	10.1070821	9.76558936
6145	**ENSG0000013020**	APOE	protein_coding	chr19	44905791	44909393	+	Human GRCh	OTTHUMG000001	APOE	**apolipoprotein E**	Extracellular	transporter		1.163050594	0	0.79402784	0	1	0	4.89831434	5.75617997	5.27996498	5.77822449
6146	**ENSG0000013020**	TOMM40	protein_coding	chr19	44890569	44903689	+	Human GRCh	OTTHUMG000001	TOMM40	**translocase of outer mitochondrial membrane 40**	Cytoplasm	ion channel		1.076969385	0	0.71304936	0	1	0	11.692434	12.0810566	12.0116051	11.961654
6147	**ENSG0000013020**	APOC1	protein_coding	chr19	44914247	44919349	+	Human GRCh	OTTHUMG000001	APOC1	**apolipoprotein C1**	Extracellular	transporter		-1.121515314	0	0.81509488	0	1	0	4.98688606	5.2032876	5.40209682	4.34668436
6148	**ENSG0000013022**	GADD45G	protein_coding	chr9	89605012	89606555	+	Human GRCh	OTTHUMG000000	GADD45G	**growth arrest and DNA damage inducible gamma**	Nucleus	other		1.085842061	0	0.74386435	0	1	0	10.1020321	10.3727511	10.6115307	9.97626683
6150	**ENSG0000013022**	DPP6	protein_coding	chr7	153748133	154894285	+	Human GRCh	OTTHUMG000001	DPP6	**dipeptidyl peptidase like 6**	Plasma Mem	other		-11.00531221	-1	0.1169662	0	NA	0	2.13923831	2.20678823	1.83752542	1.82567941
6151	**ENSG0000013022**	XPO7	protein_coding	chr8	21919662	22006585	+	Human GRCh	OTTHUMG000001	XPO7	**exportin 7**	Nucleus	transporter		-1.153477454	0	0.48343411	0	1	0	12.0110735	12.3367301	12.1268939	11.9124786
6152	**ENSG0000013023**	ACE2	protein_coding	chrX	15494566	15607236	-	Human GRCh	OTTHUMG000001	ACE2	**angiotensin converting enzyme 2**	Plasma Mem	peptidase	hydrochlo	-1.557471886	-1	0.69100564	0	NA	0	2.15554508	1.98843986	1.61835462	2.16094346
6153	**ENSG0000013024**	FAM98C	protein_coding	chr19	38403093	38409088	+	Human GRCh	OTTHUMG000001	FAM98C	**family with sequence similarity 98 member C**	Other	other		1.076248556	0	0.73926101	0	1	0	9.03055743	9.35420932	9.46143707	9.07532874
6154	**ENSG0000013025**	SAFB2	protein_coding	chr19	5586999	5624046	-	Human GRCh	OTTHUMG000001	SAFB2	**scaffold attachment factor B2**	Cytoplasm	other		1.210320799	0	0.3927988	0	1	0	10.6670419	10.9353967	11.0887638	10.9407589
6155	**ENSG0000013025**	RPL36	protein_coding	chr19	5674947	5691875	+	Human GRCh	OTTHUMG000001	RPL36	**ribosomal protein L36**	Cytoplasm	other		1.112444149	0	0.56263654	0	1	0	12.67432	12.9071099	13.0271811	12.7961115
6156	**ENSG0000013027**	ATP8B3	protein_coding	chr19	1782075	1812276	-	Human GRCh	OTTHUMG000001	ATP8B3	**ATPase phospholipid transporting 8B3**	Cytoplasm	transporter		-1.218235714	0	0.56013194	0	1	0	6.68871999	6.98319356	6.57665971	6.70208132
6158	**ENSG0000013028**	NCAN	protein_coding	chr19	19211958	19252233	+	Human GRCh	OTTHUMG000001	NCAN	**neurocan**	Extracellular	other		-22.75052847	-1	0.01256567	1	1	0	2.0914433	2.76473265	1.66073792	1.63342256
6159	**ENSG0000013029**	KIF1A	protein_coding	chr2	240713761	240824293	-	Human GRCh	OTTHUMG000001	KIF1A	**kinesin family member 1A**	Cytoplasm	other		-1.086434571	0	0.80442677	0	1	0	12.6354123	12.8596302	12.7494735	12.5641119
6160	**ENSG0000013029**	GTPBP3	protein_coding	chr19	17334920	17342731	+	Human GRCh	OTTHUMG000001	GTPBP3	**GTP binding protein 3, mitochondrial**	Cytoplasm	enzyme		1.08222629	0	0.69118473	0	1	0	10.8957339	11.2213924	11.2912763	11.0109709
6162	**ENSG0000013030**	BST2	protein_coding	chr19	17402939	17405630	-	Human GRCh	OTTHUMG000001	BST2	**bone marrow stromal cell antigen 2**	Plasma Mem	other		-2.152826141	-1	0.56711887	0	1	0	2.19347447	2.35933812	1.05779611	2.20361664
6163	**ENSG0000013030**	SLC27A1	protein_coding	chr19	17468769	17506168	+	Human GRCh	OTTHUMG000001	SLC27A1	**solute carrier family 27 member 1**	Plasma Mem	transporter		-1.057420017	0	0.87114882	0	1	0	7.43964902	8.04370488	7.90412371	7.49935289
6164	**ENSG0000013030**	NSUN5	protein_coding	chr7	73302516	73308826	-	Human GRCh	OTTHUMG000001	NSUN5	**NOP2/Sun RNA methyltransferase 5**	Nucleus	enzyme		1.020140551	0	0.92922501	0	1	0	9.65130252	10.0572896	10.009964	9.76424518
6166	**ENSG0000013030**	COLGALT1	protein_coding	chr19	17555649	17583162	+	Human GRCh	OTTHUMG000001	COLGALT1	**collagen beta(1-O)galactosyltransferase 1**	Cytoplasm	enzyme		-1.163315006	0	0.44078617	0	1	0	12.2704849	12.5911103	12.4425326	12.0678976
6167	**ENSG0000013031**	DDA1	protein_coding	chr19	17309518	17323298	+	Human GRCh	OTTHUMG000001	DDA1	**DET1 and DDB1 associated 1**	Nucleus	other		1.09781132	0	0.63200603	0	1	0	10.5501262	10.7534952	10.8609541	10.6503766
6168	**ENSG0000013031**	MRPL34	protein_coding	chr19	17292609	17306843	+	Human GRCh	OTTHUMG000001	MRPL34	**mitochondrial ribosomal protein L34**	Cytoplasm	other		1.041309882	0	0.85859156	0	1	0	9.62385521	10.0246826	9.97525708	9.79084705
6169	**ENSG0000013031**	PGLS	protein_coding	chr19	17511636	17521288	+	Human GRCh	OTTHUMG000001	PGLS	**6-phosphogluconolactonase**	Cytoplasm	enzyme		1.04037485	0	0.8651377	0	1	0	9.89182615	10.2119616	10.2958349	9.88109201
6170	**ENSG0000013033**	LSM7	protein_coding	chr19	2321520	2328611	-	Human GRCh	OTTHUMG000001	LSM7	**LSM7 homolog, U6 small nuclear RNA and mRNA degradation**	Nucleus	other		-1.001834833	0	0.99275103	0	1	0	9.81869914	10.2516495	10.1521856	9.9441543
6171	**ENSG0000013033**	TULP4	protein_coding	chr6	158232236	158511828	+	Human GRCh	OTTHUMG000000	TULP4	**TUB like protein 4**	Cytoplasm	transcription		-1.033527342	0	0.87223175	0	1	0	12.0385021	12.293119	12.1924562	12.0788398
6172	**ENSG0000013034**	SNX9	protein_coding	chr6	157700387	157945077	+	Human GRCh	OTTHUMG000000	SNX9	**sorting nexin 9**	Cytoplasm	transporter		-1.242102624	0	0.28180402	0	1	0	11.3365079	11.6092429	11.3672357	11.0916623
6173	**ENSG0000013034**	RTN4IP1	protein_coding	chr6	106570771	106629498	-	Human GRCh	OTTHUMG000000	RTN4IP1	**reticulon 4 interacting protein 1**	Cytoplasm	enzyme		1.057016585	0	0.81020192	0	1	0	8.43903666	8.65762348	8.74719663	8.46378146
6174	**ENSG0000013034**	QRSL1	protein_coding	chr6	106629578	106668417	+	Human GRCh	OTTHUMG000000	QRSL1	**glutaminyl-tRNA amidotransferase subunit QRSL1**	Cytoplasm	enzyme		-1.027300242	0	0.89991311	0	1	0	10.0320048	10.2965	10.2903941	9.97143309
6175	**ENSG0000013034**	MTRES1	protein_coding	chr6	107028199	107051586	+	Human GRCh	OTTHUMG000000	MTRES1	**mitochondrial transcription rescue factor 1**	Cytoplasm	other		1.240590471	0	0.3297408	0	1	0	7.93178882	8.49883655	8.49889272	8.46423931
6176	**ENSG0000013036**	RSPH3	protein_coding	chr6	158972871	159000202	-	Human GRCh	OTTHUMG000000	RSPH3	**radial spoke head 3**	Extracellular	other		-1.08009492	0	0.68826331	0	1	0	9.42518021	9.54462032	9.53994679	9.248186
6179	**ENSG0000013038**	MLLT1	protein_coding	chr19	6210381	6279975	-	Human GRCh	OTTHUMG000001	MLLT1	**MLLT1 super elongation complex subunit**	Nucleus	transcription		-1.040120104	0	0.83552507	0	1	0	11.6163203	11.8465562	11.7632835	11.6192915
6182	**ENSG0000013039**	AFDN	protein_coding	chr6	167826893	167972023	+	Human GRCh	OTTHUMG000000	AFDN	**afadin, adherens junction formation factor**	Nucleus	other		-1.167226642	0	0.39861522	0	1	0	12.376643	12.5700161	12.4387638	12.1615705
6183	**ENSG0000013040**	ACTN4	protein_coding	chr19	38647649	38731589	+	Human GRCh	OTTHUMG000001	ACTN4	**actinin alpha 4**	Cytoplasm	transcription		-1.044274775	0	0.82576016	0	1	0	13.7564018	14.0409397	14.0090264	13.6870181

6184	**ENSG0000013041**	STK33	protein_coding	chr11	8391868	8594289	-	Human GRCh	OTTHUMG000001	STK33	**serine/threonine kinase 33**	Cytoplasm	kinase		-1.014779689	0	0.95995024	0	1	0	8.27236714	8.16808323	8.15901576	8.2522157
6185	**ENSG0000013041**	NDUFA10	protein_coding	chr2	239892450	240025743	-	Human GRCh	OTTHUMG000001	NDUFA10	**NADH:ubiquinone oxidoreductase subunit A10**	Cytoplasm	transporter		-1.037755513	0	0.8592376	0	1	0	12.4011868	12.7148484	12.6607645	12.3746369
6187	**ENSG0000013042**	ARPC1B	protein_coding	chr7	99374249	99394816	+	Human GRCh	OTTHUMG000001	ARPC1B	**actin related protein 2/3 complex subunit 1B**	Cytoplasm	other		1.029590494	0	0.8847954	0	1	0	9.12162717	9.49523277	9.2442983	9.45466786
6189	**ENSG0000013044**	ZSWIM6	protein_coding	chr5	61332258	61546172	+	Human GRCh	OTTHUMG000001	ZSWIM6	**zinc finger SWIM-type containing 6**	Extracellular	other		-1.159774408	0	0.54128195	0	1	0	10.6379164	10.8954983	10.7474232	10.4510728
6190	**ENSG0000013047**	FCHO1	protein_coding	chr19	17747718	17788568	+	Human GRCh	OTTHUMG000001	FCHO1	**FCH and mu domain containing endocytic adaptor 1**	Plasma Mem	other		1.254035949	0	0.49405056	0	1	0	6.43196526	6.9380722	6.8705765	7.02547165
6191	**ENSG0000013047**	UNC13A	protein_coding	chr19	17601336	17688365	-	Human GRCh	OTTHUMG000001	UNC13A	**unc-13 homolog A**	Plasma Mem	other		-1.293110618	0	0.64749477	0	1	0	10.6897926	10.8977559	10.6864528	10.3093565
6192	**ENSG0000013047**	MAP1S	protein_coding	chr19	17719242	17734513	+	Human GRCh	OTTHUMG000001	MAP1S	**microtubule associated protein 1S**	Cytoplasm	enzyme		1.068457756	0	0.74048011	0	1	0	10.5871049	10.9191013	10.7474138	10.9228918
6194	**ENSG0000013050**	PXDN	protein_coding	chr2	1631887	1744852	-	Human GRCh	OTTHUMG000000	PXDN	**peroxidasin**	Extracellular	enzyme		-1.203054724	0	0.55807052	0	1	0	10.4625266	10.4303151	10.5310735	9.84602988
6195	**ENSG0000013051**	SSBP4	protein_coding	chr19	18418864	18434562	+	Human GRCh	OTTHUMG000001	SSBP4	**single stranded DNA binding protein 4**	Nucleus	transcription		1.25645153	0	0.23710158	0	1	0	10.0779583	10.3410587	10.5913988	10.3337761
6196	**ENSG0000013051**	GDF15	protein_coding	chr19	18374731	18389176	+	Human GRCh	OTTHUMG000001	GDF15	**growth differentiation factor 15**	Extracellular	growth facto	AZD8853	1.019409154	0	0.95390062	0	1	0	6.11601842	6.20560035	5.86771044	6.41814475
6197	**ENSG0000013051**	PGPEP1	protein_coding	chr19	18340598	18369950	+	Human GRCh	OTTHUMG000001	PGPEP1	**pyroglutamyl-peptidase I**	Cytoplasm	peptidase		1.277417214	0	0.6119349	0	1	0	8.82096761	9.21203729	9.42193323	9.15913279
6198	**ENSG0000013051**	IQCN	protein_coding	chr19	18257098	18274500	-	Human GRCh	OTTHUMG000001	IQCN	**IQ motif containing N**	Cytoplasm	other		-1.301901335	0	0.58411119	0	1	0	7.29798334	7.39920801	7.04926827	7.14237995
6199	**ENSG0000013052**	LSM4	protein_coding	chr19	18306236	18323112	-	Human GRCh	OTTHUMG000001	LSM4	**LSM4 homolog, U6 small nuclear RNA and mRNA degradation**	Nucleus	other		1.056221162	0	0.77836477	0	1	0	11.4266467	11.752061	11.792208	11.5148844
6200	**ENSG0000013052**	JUND	protein_coding	chr19	18279694	18281622	-	Human GRCh	OTTHUMG000001	JUND	**JunD proto-oncogene, AP-1 transcription factor subunit**	Nucleus	transcription		1.127098918	0	0.61555193	0	1	0	11.4995319	11.8486122	11.7568199	11.8777353
6202	**ENSG0000013052**	TRPM4	protein_coding	chr19	49157741	49211836	+	Human GRCh	OTTHUMG000001	TRPM4	**transient receptor potential cation channel subfamily M meml**	Plasma Mem	ion channel		-1.023485034	0	0.9187928	0	1	0	10.6473219	11.0334839	11.0113038	10.6145455
6204	**ENSG0000013054**	SULT4A1	protein_coding	chr22	43824509	43862513	-	Human GRCh	OTTHUMG000001	SULT4A1	**sulfotransferase family 4A member 1**	Cytoplasm	enzyme		-1.19530956	0	0.79239478	0	1	0	3.28462874	2.1099604	2.45752091	3.00749779
6205	**ENSG0000013054**	ZNF557	protein_coding	chr19	7069703	7087968	+	Human GRCh	OTTHUMG000001	ZNF557	**zinc finger protein 557**	Nucleus	transcription		-1.10105507	0	0.67355054	0	1	0	9.52639264	9.72640267	9.70942837	9.30925085
6206	**ENSG0000013054**	CRB3	protein_coding	chr19	6463777	6467221	+	Human GRCh	OTTHUMG000001	CRB3	**crumbs cell polarity complex component 3**	Cytoplasm	other		1.190697907	0	0.50722798	0	1	0	9.14712487	9.28365849	9.53089865	9.27912802
6207	**ENSG0000013055**	OLFM1	protein_coding	chr9	135075422	135121180	+	Human GRCh	OTTHUMG000000	OLFM1	**olfactomedin 1**	Cytoplasm	other		3.244722493	1	0.04716152	1	1	0	4.08818897	4.06644729	4.98975071	4.21533444
6208	**ENSG0000013055**	CAMSAP1	protein_coding	chr9	135808487	135907546	-	Human GRCh	OTTHUMG000000	CAMSAP1	**calmodulin regulated spectrin associated protein 1**	Cytoplasm	other		-1.056198074	0	0.77880583	0	1	0	11.2516186	11.5729788	11.5127588	11.193952
6209	**ENSG0000013056**	UBAC1	protein_coding	chr9	135932969	135961373	-	Human GRCh	OTTHUMG000000	UBAC1	**UBA domain containing 1**	Nucleus	other		-1.101563841	0	0.65500143	0	1	0	10.0394455	10.4568368	10.3272532	9.96904531
6211	**ENSG0000013058**	ZBTB46	protein_coding	chr20	63743668	63832038	-	Human GRCh	OTTHUMG000000	ZBTB46	**zinc finger and BTB domain containing 46**	Nucleus	transcription		-1.303822061	0	0.35562368	0	1	0	7.57444637	7.90715732	7.52407427	7.42408194
6212	**ENSG0000013058**	HELZ2	protein_coding	chr20	63558086	63574239	-	Human GRCh	OTTHUMG000000	HELZ2	**helicase with zinc finger 2**	Nucleus	transcription		-1.017739415	0	0.9688859	0	1	0	10.7918205	11.0867874	11.0620622	10.7780963
6213	**ENSG0000013059**	SAMD10	protein_coding	chr20	63974116	63980008	-	Human GRCh	OTTHUMG000000	SAMD10	**sterile alpha motif domain containing 10**	Other	other		1.001258856	0	0.9968014	0	1	0	8.98470653	9.14967477	9.22508695	8.89492557
6214	**ENSG0000013059**	LSP1	protein_coding	chr11	1850904	1892267	+	Human GRCh	OTTHUMG000000	LSP1	**lymphocyte specific protein 1**	Cytoplasm	other		1	0	1	0	NA	0	0.0958344	0.11091404	0.10843329	0.09672353
6215	**ENSG0000013059**	TNNT3	protein_coding	chr11	1919703	1938706	+	Human GRCh	OTTHUMG000000	TNNT3	**troponin T3, fast skeletal type**	Cytoplasm	other		14.97212619	1	0.24246959	0	NA	0	0.38055612	0.38803775	0.38678068	1.18433773
6216	**ENSG0000013059**	TNNI2	protein_coding	chr11	1838981	1841680	+	Human GRCh	OTTHUMG000000	TNNI2	**troponin I2, fast skeletal type**	Cytoplasm	enzyme		1	0	1	0	1	0	0.22410283	0.31433589	0.3000109	0.22964698
6218	**ENSG0000013063**	COL5A1	protein_coding	chr9	134641803	134844843	+	Human GRCh	OTTHUMG000000	COL5A1	**collagen type V alpha 1 chain**	Extracellular	other	collagenas	5.269103004	1	0.08277598	0	1	0	2.31816479	1.66010402	2.22304731	3.37627149
6219	**ENSG0000013063**	ATXN10	protein_coding	chr22	45671798	45845307	+	Human GRCh	OTTHUMG000001	ATXN10	**ataxin 10**	Cytoplasm	other		-1.154969561	0	0.48689963	0	1	0	10.7869485	11.1267261	11.0028318	10.5763213
6220	**ENSG0000013064**	TUBGCP2	protein_coding	chr10	133278635	133318823	-	Human GRCh	OTTHUMG000000	TUBGCP2	**tubulin gamma complex component 2**	Cytoplasm	peptidase		-1.100993781	0	0.65092069	0	1	0	11.336709	11.7325842	11.5416887	11.3349686
6221	**ENSG0000013064**	CALY	protein_coding	chr10	133324072	133336935	-	Human GRCh	OTTHUMG000000	CALY	**calcyon neuron specific vesicular protein**	Plasma Mem	transmembra	trifluopera	6.810231841	1	0.11163805	0	NA	0	1.5592147	2.00445141	2.8187669	2.19724319
6222	**ENSG0000013064**	CYP2E1	protein_coding	chr10	133520406	133561220	+	Human GRCh	OTTHUMG000000	CYP2E1	**cytochrome P450 family 2 subfamily E member 1**	Cytoplasm	enzyme		-1.184926959	0	0.66508453	0	1	0	7.1417625	7.52303916	7.25948392	7.06588448
6223	**ENSG0000013065**	PNPLA7	protein_coding	chr9	137459952	137550402	-	Human GRCh	OTTHUMG000000	PNPLA7	**patatin like phospholipase domain containing 7**	Cytoplasm	enzyme		-1.161670806	0	0.63990878	0	1	0	8.37337483	8.72148889	8.36477373	8.42595261
6225	**ENSG0000013066**	PAK4	protein_coding	chr19	39125770	39182816	+	Human GRCh	OTTHUMG000001	PAK4	**p21 (RAC1) activated kinase 4**	Cytoplasm	kinase	PF-375830	-1.063561353	0	0.74946211	0	1	0	11.8608941	12.1225042	12.0375618	11.8110003
6226	**ENSG0000013067**	MNX1	protein_coding	chr7	156994051	157010663	-	Human GRCh	OTTHUMG000001	MNX1	**motor neuron and pancreas homeobox 1**	Nucleus	transcription		1.137984288	0	0.62186748	0	1	0	8.51386077	8.65296283	8.77481696	8.67575908
6227	**ENSG0000013068**	ZNF337	protein_coding	chr20	25673195	25696853	-	Human GRCh	OTTHUMG000000	ZNF337	**zinc finger protein 337**	Nucleus	transcription		-1.09751198	0	0.71426671	0	1	0	9.35661929	9.46676981	9.54687154	9.01830605
6228	**ENSG0000013069**	CEP85	protein_coding	chr1	26234200	26278808	+	Human GRCh	OTTHUMG000000	CEP85	**centrosomal protein 85**	Nucleus	other		-1.081929341	0	0.69333585	0	1	0	10.8777617	11.0992321	11.0940316	10.6861412
6229	**ENSG0000013069**	TAF4	protein_coding	chr20	61953469	62065881	-	Human GRCh	OTTHUMG000000	TAF4	**TATA-box binding protein associated factor 4**	Nucleus	transcription		-1.026195494	0	0.89129203	0	1	0	10.8540695	11.14309	11.0849792	10.8620807
6230	**ENSG0000013070**	GATA5	protein_coding	chr20	62463497	62475995	-	Human GRCh	OTTHUMG000000	GATA5	**GATA binding protein 5**	Nucleus	transcription		-2.950400003	-1	0.24603542	0	1	0	2.89432811	2.6556706	2.63420843	1.94805148
6231	**ENSG0000013070**	RBBP8NL	protein_coding	chr20	62410237	62427539	-	Human GRCh	OTTHUMG000000	RBBP8NL	**RBBP8 N-terminal like**	Extracellular	other		-1.002864366	0	0.99112385	0	1	0	7.78414923	8.21696036	8.20191204	7.79756099
6232	**ENSG0000013070**	LAMA5	protein_coding	chr20	62307955	62367312	-	Human GRCh	OTTHUMG000000	LAMA5	**laminin subunit alpha 5**	Extracellular	other		-1.117553229	0	0.54643539	0	1	0	11.7918148	12.1004835	11.9258684	11.742678
6233	**ENSG0000013070**	OSBPL2	protein_coding	chr20	62231922	62296213	+	Human GRCh	OTTHUMG000000	OSBPL2	**oxysterol binding protein like 2**	Cytoplasm	transporter		-1.061945193	0	0.78323965	0	1	0	10.7472103	10.9618963	10.8717239	10.709674
6234	**ENSG0000013070**	ADRM1	protein_coding	chr20	62302093	62308862	+	Human GRCh	OTTHUMG000000	ADRM1	**ADRM1 26S proteasome ubiquitin receptor**	Plasma Mem	other		1.017673977	0	0.93663509	0	1	0	11.0777437	11.537728	11.3950815	11.3016211
6235	**ENSG0000013070**	ASS1	protein_coding	chr9	130444961	130501274	+	Human GRCh	OTTHUMG000000	ASS1	**argininosuccinate synthase 1**	Cytoplasm	enzyme		1.461564623	0	0.43152745	0	1	0	8.8551211	9.22259621	8.95385507	9.82315907
6236	**ENSG0000013071**	PRDM12	protein_coding	chr9	130664594	130682986	+	Human GRCh	OTTHUMG000000	PRDM12	**PR/SET domain 12**	Nucleus	transcription		4.991221707	1	0.09016222	0	1	0	1.43191947	2.23697548	3.19203279	2.10783168
6237	**ENSG0000013071**	EXOSC2	protein_coding	chr9	130693721	130707288	+	Human GRCh	OTTHUMG000000	EXOSC2	**exosome component 2**	Nucleus	enzyme		-1.196715486	0	0.33742276	0	1	0	11.0448446	11.3766415	11.1758517	10.8479953
6238	**ENSG0000013071**	POMT1	protein_coding	chr9	131502789	131523806	+	Human GRCh	OTTHUMG000000	POMT1	**protein O-mannosyltransferase 1**	Cytoplasm	enzyme		-1.099684959	0	0.66667065	0	1	0	11.1712719	11.5751089	11.4600103	11.0825823
6239	**ENSG0000013071**	UCK1	protein_coding	chr9	131523801	131531264	-	Human GRCh	OTTHUMG000000	UCK1	**uridine-cytidine kinase 1**	Cytoplasm	kinase		-1.099983666	0	0.67419061	0	1	0	9.81542446	10.2448852	10.1451377	9.70550985
6240	**ENSG0000013072**	FIBCD1	protein_coding	chr9	130902438	130939286	-	Human GRCh	OTTHUMG000000	FIBCD1	**fibrinogen C domain containing 1**	Other	other		1.548115898	1	0.53251802	0	1	0	4.40427227	3.9704594	4.80468602	4.39011722
6241	**ENSG0000013072**	CHMP2A	protein_coding	chr19	58551452	58555105	-	Human GRCh	OTTHUMG000001	CHMP2A	**charged multivesicular body protein 2A**	Cytoplasm	other		1.114691331	0	0.63692141	0	1	0	10.394266	10.8335626	10.7127058	10.7981693
6242	**ENSG0000013072**	UBE2M	protein_coding	chr19	58555712	58558954	-	Human GRCh	OTTHUMG000001	UBE2M	**ubiquitin conjugating enzyme E2 M**	Cytoplasm	enzyme		1.137303937	0	0.52799162	0	1	0	11.068132	11.3522763	11.3665426	11.3568267
6243	**ENSG0000013072**	TRIM28	protein_coding	chr19	58544064	58550722	+	Human GRCh	OTTHUMG000001	TRIM28	**tripartite motif containing 28**	Nucleus	transcription		-1.065283758	0	0.75301625	0	1	0	13.8811977	14.1979801	14.0819095	13.8675263
6244	**ENSG0000013073**	METTL26	protein_coding	chr16	634427	636366	-	Human GRCh	OTTHUMG000000	METTL26	**methyltransferase like 26**	Other	other		1.071025812	0	0.74447214	0	1	0	10.5143732	10.8354491	10.8522698	10.6670931
6245	**ENSG0000013073**	YIPF2	protein_coding	chr19	10922185	10928681	-	Human GRCh	OTTHUMG000001	YIPF2	**Yip1 domain family member 2**	Cytoplasm	other		1.061149442	0	0.77477642	0	1	0	10.402296	10.6414535	10.6931959	10.486138
6246	**ENSG0000013073**	ATG4D	protein_coding	chr19	10543895	10553418	+	Human GRCh	OTTHUMG000001	ATG4D	**autophagy related 4D cysteine peptidase**	Cytoplasm	peptidase		-1.014971232	0	0.94265934	0	1	0	10.1174187	10.4004376	10.3278584	10.1683482
6247	**ENSG0000013074**	EIF2S3	protein_coding	chrX	24054946	24078810	+	Human GRCh	OTTHUMG000000	EIF2S3	**eukaryotic translation initiation factor 2 subunit gamma**	Cytoplasm	translation re		-1.064285471	0	0.75657789	0	1	0	12.3234182	12.6221878	12.5688621	12.233093
6248	**ENSG0000013074**	TMEM160	protein_coding	chr19	47045909	47048624	-	Human GRCh	OTTHUMG000001	TMEM160	**transmembrane protein 160**	Cytoplasm	other		1.555635672	1	0.10193847	0	1	0	7.73521667	7.86931138	8.42801092	8.14529382
6249	**ENSG0000013074**	ZC3H4	protein_coding	chr19	47064187	47113776	-	Human GRCh	OTTHUMG000001	ZC3H4	**zinc finger CCCH-type containing 4**	Nucleus	transcription		-1.075902012	0	0.70393187	0	1	0	11.2762241	11.5217296	11.4471345	11.189168
6250	**ENSG0000013075**	NPAS1	protein_coding	chr19	47019837	47045775	+	Human GRCh	OTTHUMG000001	NPAS1	**neuronal PAS domain protein 1**	Nucleus	transcription		-1.188889183	0	0.62711658	0	1	0	5.64378448	5.52933212	5.32361592	5.50934782
6252	**ENSG0000013075**	MAP3K10	protein_coding	chr19	40191426	40215575	+	Human GRCh	OTTHUMG000001	MAP3K10	**mitogen-activated protein kinase kinase kinase 10**	Cytoplasm	kinase		1.13320952	0	0.55318622	0	1	0	9.04932214	9.12715068	9.38315398	9.04819681
6253	**ENSG0000013076**	ARHGEF16	protein_coding	chr1	3454665	3481113	+	Human GRCh	OTTHUMG000000	ARHGEF16	**Rho guanine nucleotide exchange factor 16**	Cytoplasm	other		-1.094545892	0	0.66971843	0	1	0	9.83398089	10.202707	9.97302418	9.89452464
6254	**ENSG0000013076**	LRRC47	protein_coding	chr1	3778559	3796498	-	Human GRCh	OTTHUMG000000	LRRC47	**leucine rich repeat containing 47**	Other	other		1.014702975	0	0.94571474	0	1	0	10.7489036	11.0355944	11.0515766	10.7654974
6255	**ENSG0000013076**	SESN2	protein_coding	chr1	28259518	28282491	+	Human GRCh	OTTHUMG000000	SESN2	**sestrin 2**	Cytoplasm	enzyme		-1.050663222	0	0.85341202	0	1	0	9.41256497	8.70819427	9.18555303	8.91601108
6256	**ENSG0000013076**	SMPDL3B	protein_coding	chr1	27935000	27959157	+	Human GRCh	OTTHUMG000000	SMPDL3B	**sphingomyelin phosphodiesterase acid like 3B**	Extracellular	enzyme		-1.46055483	0	0.20106703	0	1	0	8.25324402	8.59947532	8.28433194	7.69198674
6257	**ENSG0000013077**	ATP5IF1	protein_coding	chr1	28236109	28246906	+	Human GRCh	OTTHUMG000000	ATP5IF1	**ATP synthase inhibitory factor subunit 1**	Cytoplasm	other		1.075198146	0	0.71817149	0	1	0	10.5215083	10.7257557	10.7546496	10.6623243
6258	**ENSG0000013077**	MED18	protein_coding	chr1	28329002	28335965	+	Human GRCh	OTTHUMG000000	MED18	**mediator complex subunit 18**	Nucleus	transcription		1.002973655	0	0.98870134	0	1	0	8.0184186	8.29166387	8.21250555	8.11658943
6259	**ENSG0000013077**	THEMIS2	protein_coding	chr1	27872543	27886685	+	Human GRCh	OTTHUMG000000	THEMIS2	**thymocyte selection associated family member 2**	Other	other		3.230966739	1	0.02532888	1	1	0	3.80868716	3.97096112	5.19531494	4.40762566
6260	**ENSG0000013077**	CLIP1	protein_coding	chr12	122271432	122422669	-	Human GRCh	OTTHUMG000001	CLIP1	**CAP-Gly domain containing linker protein 1**	Cytoplasm	other		-1.143403121	0	0.47069799	0	1	0	11.7418501	11.9703421	11.837679	11.5716579
6261	**ENSG0000013078**	CCDC62	protein_coding	chr12	122774526	122827528	+	Human GRCh	OTTHUMG000001	CCDC62	**coiled-coil domain containing 62**	Nucleus	transcription		-1.52517059	-1	0.16373861	0	1	0	7.71387458	8.03013187	7.63452126	7.14670476
6262	**ENSG0000013078**	HIP1R	protein_coding	chr12	122834453	122862961	+	Human GRCh	OTTHUMG000001	HIP1R	**huntingtin interacting protein 1 related**	Cytoplasm	other		1.069287509	0	0.77605414	0	1	0	10.2048849	10.4335114	10.5750919	10.1909357
6263	**ENSG0000013080**	ZNF317	protein_coding	chr19	9140397	9163424	+	Human GRCh	OTTHUMG000001	ZNF317	**zinc finger protein 317**	Nucleus	transcription		-1.141961873	0	0.51008442	0	1	0	10.2634604	10.5542056	10.5060858	9.98163065
6264	**ENSG0000013081**	PPAN	protein_coding	chr19	10106362	10112012	+	Human GRCh	OTTHUMG000001	PPAN	**peter pan homolog**	Nucleus	other		1.081982677	0	0.69999683	0	1	0	10.9390998	11.2452778	11.3126741	11.0553606
6265	**ENSG0000013081**	EIF3G	protein_coding	chr19	10115014	10119918	-	Human GRCh	OTTHUMG000001	EIF3G	**eukaryotic translation initiation factor 3 subunit G**	Cytoplasm	translation re		1.069238752	0	0.74809372	0	1	0	11.6048553	11.9800595	11.9853112	11.770776
6267	**ENSG0000013081**	SHFL	protein_coding	chr19	10086122	10093252	+	Human GRCh	OTTHUMG000001	SHFL	**shiftless antiviral inhibitor of ribosomal frameshifting**	Other	other		1.579171119	1	0.24457914	0	1	0	5.10769256	5.58001647	6.22735643	5.18472936
6268	**ENSG0000013081**	DNMT1	protein_coding	chr19	10133342	10231286	-	Human GRCh	OTTHUMG000001	DNMT1	**DNA methyltransferase 1**	Nucleus	enzyme	cladribine/	-1.122341175	0	0.54889903	0	1	0	12.9014718	13.1598275	13.0094296	12.8045323
6269	**ENSG0000013081**	ZNF426	protein_coding	chr19	9523223	9538645	-	Human GRCh	OTTHUMG000001	ZNF426	**zinc finger protein 426**	Nucleus	transcription		-1.092723516	0	0.6665797	0	1	0	9.81299757	10.063692	10.00789	9.66153426
6270	**ENSG0000013082**	SLC6A8	protein_coding	chrX	153687926	153696588	+	Human GRCh	OTTHUMG000000	SLC6A8	**solute carrier family 6 member 8**	Cytoplasm	transporter	guanidinop	1.05885914	0	0.80896915	0	1	0	12.1322626	12.4609728	12.4428284	12.3011829
6271	**ENSG0000013082**	PNCK	protein_coding	chrX	153669733	153689010	-	Human GRCh	OTTHUMG000000	PNCK	**pregnancy up-regulated nonubiquitous CaM kinase**	Other	kinase		1.30720648	0	0.52131719	0	1	0	7.55586086	7.25860885	7.99763096	7.35886729
6272	**ENSG0000013082**	DKC1	protein_coding	chrX	154762742	154777689	+	Human GRCh	OTTHUMG000000	DKC1	**dyskerin pseudouridine synthase 1**	Nucleus	enzyme		-1.063841025	0	0.75366838	0	1	0	11.280653	11.5662608	11.5207516	11.1819507
6273	**ENSG0000013082**	PLXNA3	protein_coding	chrX	154458281	154477779	+	Human GRCh	OTTHUMG000000	PLXNA3	**plexin A3**	Plasma Mem	transmembra		-1.021148167	0	0.93113428	0	1	0	11.5034668	11.751963	11.6834195	11.5337167
6274	**ENSG0000013082**	DUSP9	protein_coding	chrX	153642492	153651326	+	Human GRCh	OTTHUMG000000	DUSP9	**dual specificity phosphatase 9**	Nucleus	phosphatase		1.194745984	0	0.56108135	0	1	0	7.87214143	8.18235325	8.51819835	7.88372198
6275	**ENSG0000013083**	MPP1	protein_coding	chrX	154778684	154821007	-	Human GRCh	OTTHUMG000000	MPP1	**MAGUK p55 scaffold protein 1**	Plasma Mem	kinase		1.087337228	0	0.83503999	0	1	0	5.82611767	5.79613637	5.99373816	5.77602386
6276	**ENSG0000013084**	ZNF331	protein_coding	chr19	53519527	53580269	+	Human GRCh	OTTHUMG000001	ZNF331	**zinc finger protein 331**	Nucleus	transcription		-1.164964943	0	0.45482462	0	1	0	9.91782231	10.1646364	10.0060227	9.73007926

6277	**ENSG0000013085**	ZNF236	protein_coding	chr18	76822557	76972901	+	Human GRCh	OTTHUMG000001	ZNF236	**zinc finger protein 236**	Nucleus	transcription		-1.22420182	0	0.32774742	0	1	0	10.4572226	10.8208324	10.5055071	10.3520617
6279	**ENSG0000013088**	LRP3	protein_coding	chr19	33177603	33208864	+	Human GRCh	OTTHUMG000001	LRP3	**LDL receptor related protein 3**	Plasma Mem	transmembra		1.042721316	0	0.85044044	0	1	0	9.76430913	10.0407629	10.0680267	9.83466282
6280	**ENSG0000013092**	MTRFR	protein_coding	chr12	123233385	123258079	+	Human GRCh	OTTHUMG000001	MTRFR	**mitochondrial translation release factor in rescue**	Cytoplasm	other		-1.001932227	0	0.99202087	0	1	0	9.66489531	9.78580472	9.9010564	9.51767807
6281	**ENSG0000013093**	NOL11	protein_coding	chr17	67717931	67744531	+	Human GRCh	OTTHUMG000001	NOL11	**nucleolar protein 11**	Nucleus	other		1.010886699	0	0.96083724	0	1	0	10.3609608	10.6277337	10.6986877	10.2954429
6282	**ENSG0000013093**	UBE4B	protein_coding	chr1	10032832	10181239	+	Human GRCh	OTTHUMG000000	UBE4B	**ubiquitination factor E4B**	Cytoplasm	enzyme		-1.235745607	0	0.25651276	0	1	0	11.7738504	12.1060738	11.7758888	11.6568268
6283	**ENSG0000013094**	CASZ1	protein_coding	chr1	10636604	10796650	-	Human GRCh	OTTHUMG000000	CASZ1	**castor zinc finger 1**	Nucleus	enzyme		1.346477152	0	0.14793956	0	1	0	8.62655746	8.63839509	8.75011614	9.07152887
6285	**ENSG0000013094**	HSD17B3	protein_coding	chr9	96235306	96302176	-	Human GRCh	OTTHUMG000000	HSD17B3	**hydroxysteroid 17-beta dehydrogenase 3**	Cytoplasm	enzyme		-3.485611147	-1	0.19091415	0	1	0	3.12265741	2.27364921	1.82237557	2.47243439
6287	**ENSG0000013095**	HABP4	protein_coding	chr9	96450169	96491336	+	Human GRCh	OTTHUMG000000	HABP4	**hyaluronan binding protein 4**	Cytoplasm	other		1.310413538	0	0.37148286	0	1	0	6.98505852	7.49984961	7.6446333	7.45451076
6289	**ENSG0000013095**	SLC35D2	protein_coding	chr9	96313444	96383711	-	Human GRCh	OTTHUMG000000	SLC35D2	**solute carrier family 35 member D2**	Cytoplasm	transporter		-1.120502252	0	0.6791985	0	1	0	7.46128306	7.60614741	7.53444848	7.28713368
6290	**ENSG0000013096**	PRRG1	protein_coding	chrX	37349309	37457292	+	Human GRCh	OTTHUMG000000	PRRG1	**proline rich and Gla domain 1**	Plasma Mem	other		1.03342521	0	0.91288189	0	1	0	7.54923403	7.85594561	7.88924751	7.59205307
6291	**ENSG0000013098**	UBA1	protein_coding	chrX	47190861	47215128	+	Human GRCh	OTTHUMG000000	UBA1	**ubiquitin like modifier activating enzyme 1**	Cytoplasm	enzyme	MLN7243	-1.134884071	0	0.5182546	0	1	0	12.9576372	13.2997795	13.0740685	12.9171392
6292	**ENSG0000013098**	RGN	protein_coding	chrX	47078355	47093314	+	Human GRCh	OTTHUMG000000	RGN	**regucalcin**	Nucleus	enzyme		1.00488806	0	0.99563225	0	NA	0	2.58330939	2.8637993	2.98529926	2.40090857
6293	**ENSG0000013099**	POLN	protein_coding	chr4	2071918	2242121	-	Human GRCh	OTTHUMG000000	POLN	**DNA polymerase nu**	Nucleus	enzyme		-1.087494701	0	0.7560709	0	1	0	8.2871368	8.52254777	8.48996082	8.11176748
6295	**ENSG0000013101**	PPIL4	protein_coding	chr6	149504495	149546043	-	Human GRCh	OTTHUMG000000	PPIL4	**peptidylprolyl isomerase like 4**	Nucleus	enzyme		-1.037884908	0	0.86513991	0	1	0	9.83023056	10.0852502	10.0285278	9.8087726
6296	**ENSG0000013101**	ULBP2	protein_coding	chr6	149942014	149949235	+	Human GRCh	OTTHUMG000000	ULBP2	**UL16 binding protein 2**	Plasma Mem	transmembra		1.145837232	0	0.81023849	0	1	0	5.65642663	5.76667956	5.8650168	5.84499518
6297	**ENSG0000013101**	AKAP12	protein_coding	chr6	151239967	151358559	+	Human GRCh	OTTHUMG000000	AKAP12	**A-kinase anchoring protein 12**	Cytoplasm	transporter		-1.123151664	0	0.84443199	0	1	0	11.3297872	10.9810563	10.9675445	11.0853584
6298	**ENSG0000013101**	SYNE1	protein_coding	chr6	152121687	152637801	-	Human GRCh	OTTHUMG000000	SYNE1	**spectrin repeat containing nuclear envelope protein 1**	Nucleus	other		-1.049610866	0	0.88159321	0	1	0	5.38183821	5.56080903	5.21482645	5.59932872
6299	**ENSG0000013101**	ULBP3	protein_coding	chr6	150061053	150069121	-	Human GRCh	OTTHUMG000000	ULBP3	**UL16 binding protein 3**	Plasma Mem	transmembra		-1.169124562	0	0.66670727	0	1	0	6.89191808	7.14494512	6.86176388	6.85159978
6300	**ENSG0000013102**	LATS1	protein_coding	chr6	149658153	149718105	-	Human GRCh	OTTHUMG000000	LATS1	**large tumor suppressor kinase 1**	Nucleus	kinase	TDI-01153	-1.027366619	0	0.89535958	0	1	0	11.6307246	11.8393725	11.8800726	11.5105988
6301	**ENSG0000013103**	EPS8L1	protein_coding	chr19	55072020	55087923	+	Human GRCh	OTTHUMG000001	EPS8L1	**EPS8 like 1**	Cytoplasm	other		1.088627951	0	0.85738836	0	1	0	6.05158519	6.14650194	6.16479042	6.22343944
6303	**ENSG0000013104**	AAR2	protein_coding	chr20	36236131	36270918	+	Human GRCh	OTTHUMG000000	AAR2	**AAR2 splicing factor**	Other	other		-1.156790257	0	0.50882745	0	1	0	9.83837725	10.1182957	9.96507197	9.66556575
6304	**ENSG0000013104**	TTLL9	protein_coding	chr20	31870634	31945000	+	Human GRCh	OTTHUMG000001	TTLL9	**tubulin tyrosine ligase like 9**	Cytoplasm	enzyme		-1.048054769	0	0.9409019	0	1	0	2.52058948	4.01964229	3.73197652	3.13545332
6306	**ENSG0000013105**	RBM39	protein_coding	chr20	35701347	35742312	-	Human GRCh	OTTHUMG000000	RBM39	**RNA binding motif protein 39**	Nucleus	transcription		-1.030676872	0	0.87884507	0	1	0	12.9183386	13.0808808	13.1277116	12.7856089
6309	**ENSG0000013106**	ZNF341	protein_coding	chr20	33731657	33792269	+	Human GRCh	OTTHUMG000000	ZNF341	**zinc finger protein 341**	Nucleus	transcription		1.114593484	0	0.66778017	0	1	0	8.16103973	8.38739532	8.54998733	8.22090908
6310	**ENSG0000013106**	GGT7	protein_coding	chr20	34844720	34872856	-	Human GRCh	OTTHUMG000000	GGT7	**gamma-glutamyltransferase 7**	Plasma Mem	enzyme		-1.122427785	0	0.61789106	0	1	0	10.5138393	10.6571326	10.56316	10.3478773
6312	**ENSG0000013106**	ACSS2	protein_coding	chr20	34872146	34927962	+	Human GRCh	OTTHUMG000000	ACSS2	**acyl-CoA synthetase short chain family member 2**	Cytoplasm	enzyme	MTB-9655	-1.182296991	0	0.52785407	0	1	0	11.5555177	11.9297732	11.6707183	11.4388392
6313	**ENSG0000013108**	EDA2R	protein_coding	chrX	66595637	66639298	-	Human GRCh	OTTHUMG000000	EDA2R	**ectodysplasin A2 receptor**	Plasma Mem	transmembra		-1.284145024	0	0.86658199	0	1	0	3.35251559	2.90363536	2.99305554	2.84789613
6314	**ENSG0000013108**	ARHGEF9	protein_coding	chrX	63634967	63809274	-	Human GRCh	OTTHUMG000000	ARHGEF9	**Cdc42 guanine nucleotide exchange factor 9**	Cytoplasm	other		-1.094054411	0	0.70855292	0	1	0	9.5386	9.96393674	9.77481959	9.54887171
6315	**ENSG0000013109**	C1QL1	protein_coding	chr17	44959693	44968303	-	Human GRCh	OTTHUMG000001	C1QL1	**complement C1q like 1**	Extracellular	other		-1.99908479	0	0.45292054	0	1	0	3.27879279	2.49335083	2.47679213	2.93258163
6316	**ENSG0000013109**	GFAP	protein_coding	chr17	44903159	44916937	-	Human GRCh	OTTHUMG000001	GFAP	**glial fibrillary acidic protein**	Cytoplasm	other		5.150467937	1	0.31762718	0	NA	0	1.3744493	1.39038534	1.38771922	1.74065988
6319	**ENSG0000013110**	ATP6V1E1	protein_coding	chr22	17592136	17628749	-	Human GRCh	OTTHUMG000000	ATP6V1E1	**ATPase H+ transporting V1 subunit E1**	Cytoplasm	transporter		-1.135192882	0	0.56067255	0	1	0	10.2889541	10.6665392	10.4116588	10.2843269
6320	**ENSG0000013111**	ZNF227	protein_coding	chr19	44207547	44237268	+	Human GRCh	OTTHUMG000001	ZNF227	**zinc finger protein 227**	Nucleus	transcription		1.235136763	0	0.40204114	0	1	0	8.7029683	8.70904842	9.20396703	8.58710081
6321	**ENSG0000013111**	ZNF428	protein_coding	chr19	43607224	43619629	-	Human GRCh	OTTHUMG000001	ZNF428	**zinc finger protein 428**	Other	other		1.131010123	0	0.60902836	0	1	0	9.08615511	9.25578634	9.53260486	9.03555957
6323	**ENSG0000013112**	ZNF141	protein_coding	chr4	337814	384868	+	Human GRCh	OTTHUMG000001	ZNF141	**zinc finger protein 141**	Nucleus	transcription		-1.011533482	0	0.96349473	0	1	0	11.599001	11.8326062	11.8712334	11.5202876
6325	**ENSG0000013114**	COX4I1	protein_coding	chr16	85798633	85807068	+	Human GRCh	OTTHUMG000001	COX4I1	**cytochrome c oxidase subunit 4I1**	Cytoplasm	enzyme		1.065497929	0	0.75692107	0	1	0	11.7022411	12.0319511	12.0510128	11.8412443
6326	**ENSG0000013114**	EMC8	protein_coding	chr16	85771758	85799608	-	Human GRCh	OTTHUMG000001	EMC8	**ER membrane protein complex subunit 8**	Cytoplasm	other		1.102521698	0	0.64596018	0	1	0	9.35494203	9.8023166	9.81708148	9.58885263
6327	**ENSG0000013114**	GSE1	protein_coding	chr16	85169525	85676204	+	Human GRCh	OTTHUMG000001	GSE1	**Gse1 coiled-coil protein**	Extracellular	other		-1.017398322	0	0.93539798	0	1	0	14.0175855	14.3097641	14.3604265	13.9020068
6328	**ENSG0000013115**	GINS2	protein_coding	chr16	85676198	85690073	-	Human GRCh	OTTHUMG000001	GINS2	**GINS complex subunit 2**	Nucleus	other		-1.114636517	0	0.59432242	0	1	0	9.76787249	10.1812307	9.99161837	9.74132656
6329	**ENSG0000013116**	CHMP1A	protein_coding	chr16	89640816	89657738	-	Human GRCh	OTTHUMG000001	CHMP1A	**charged multivesicular body protein 1A**	Extracellular	peptidase		-1.073498494	0	0.70754031	0	1	0	11.2737937	11.5158612	11.4271625	11.2054639
6330	**ENSG0000013117**	SH3BGRL	protein_coding	chrX	81202102	81298547	+	Human GRCh	OTTHUMG000000	SH3BGRL	**SH3 domain binding glutamate rich protein like**	Cytoplasm	other		-10.31031968	-1	0.1025083	0	1	0	3.62297944	2.94037916	2.92448626	2.84790706
6331	**ENSG0000013117**	COX7B	protein_coding	chrX	77899440	77907376	+	Human GRCh	OTTHUMG000000	COX7B	**cytochrome c oxidase subunit 7B**	Cytoplasm	enzyme		1.006676896	0	0.97757416	0	1	0	10.8275078	11.2150634	11.1535874	10.9244138
6333	**ENSG0000013118**	F12	protein_coding	chr5	177402133	177416583	-	Human GRCh	OTTHUMG000001	F12	**coagulation factor XII**	Extracellular	peptidase		1.397374732	0	0.25310885	0	1	0	7.10610461	7.19993441	7.51616333	7.53465002
6334	**ENSG0000013118**	PRR7	protein_coding	chr5	177446445	177456286	+	Human GRCh	OTTHUMG000001	PRR7	**proline rich 7, synaptic**	Plasma Mem	other		1.218414423	0	0.46105188	0	1	0	8.72187657	9.13124391	9.19610748	9.13673
6335	**ENSG0000013119**	NFATC1	protein_coding	chr18	79395856	79529325	+	Human GRCh	OTTHUMG000001	NFATC1	**nuclear factor of activated T cells 1**	Nucleus	transcription		-1.072968369	0	0.84097063	0	1	0	6.35834522	6.42275368	6.17362202	6.45301726
6338	**ENSG0000013123**	CAP1	protein_coding	chr1	40040233	40072649	+	Human GRCh	OTTHUMG000000	CAP1	**cyclase associated actin cytoskeleton regulatory protein 1**	Plasma Mem	other		-1.128719533	0	0.54012166	0	1	0	11.6737101	11.9377698	11.7143034	11.6412697
6339	**ENSG0000013123**	PPT1	protein_coding	chr1	40072710	40097260	-	Human GRCh	OTTHUMG000000	PPT1	**palmitoyl-protein thioesterase 1**	Cytoplasm	enzyme	ezurpimtr	-1.084832539	0	0.70571725	0	1	0	10.4164319	10.6238118	10.51179	10.3547868
6340	**ENSG0000013124**	RAB11FIP4	protein_coding	chr17	31391675	31538211	+	Human GRCh	OTTHUMG000001	RAB11FIP4	**RAB11 family interacting protein 4**	Cytoplasm	other		1.041517202	0	0.86181476	0	1	0	9.92384428	10.2973689	10.3854741	9.90195476
6341	**ENSG0000013126**	RLIM	protein_coding	chrX	74582976	74614624	-	Human GRCh	OTTHUMG000000	RLIM	**ring finger protein, LIM domain interacting**	Nucleus	enzyme		-1.060148856	0	0.78172594	0	1	0	12.0257597	12.3247595	12.2798242	11.9345532
6343	**ENSG0000013126**	ABCB7	protein_coding	chrX	75051048	75156732	-	Human GRCh	OTTHUMG000000	ABCB7	**ATP binding cassette subfamily B member 7**	Cytoplasm	transporter		-1.018388909	0	0.93786814	0	1	0	9.88228314	10.2730904	10.2573133	9.8561704
6344	**ENSG0000013132**	TRAF3	protein_coding	chr14	102777449	102911500	+	Human GRCh	OTTHUMG000001	TRAF3	**TNF receptor associated factor 3**	Cytoplasm	enzyme		-1.022830611	0	0.91080003	0	1	0	10.5199612	10.7129018	10.8324125	10.2980333
6345	**ENSG0000013135**	HAUS8	protein_coding	chr19	17049729	17075625	-	Human GRCh	OTTHUMG000001	HAUS8	**HAUS augmin like complex subunit 8**	Cytoplasm	other		-1.062109529	0	0.79191137	0	1	0	8.80495306	8.91695287	8.86899521	8.72085512
6347	**ENSG0000013136**	MRPS25	protein_coding	chr3	15009611	15065339	-	Human GRCh	OTTHUMG000001	MRPS25	**mitochondrial ribosomal protein S25**	Cytoplasm	other		1.079096123	0	0.70672869	0	1	0	12.4055991	12.7279318	12.7389189	12.5794941
6348	**ENSG0000013137**	SH3BP5	protein_coding	chr3	15254353	15341368	-	Human GRCh	OTTHUMG000001	SH3BP5	**SH3 domain binding protein 5**	Cytoplasm	other		2.081335653	1	0.03038519	1	1	0	6.07562192	4.85837236	6.20264118	6.40235444
6349	**ENSG0000013137**	HACL1	protein_coding	chr3	15560699	15601852	-	Human GRCh	OTTHUMG000001	HACL1	**2-hydroxyacyl-CoA lyase 1**	Cytoplasm	enzyme		-1.270709573	0	0.31730383	0	1	0	9.39208856	9.712538	9.44303207	9.13266684
6350	**ENSG0000013137**	TBC1D5	protein_coding	chr3	17157162	18444817	-	Human GRCh	OTTHUMG000001	TBC1D5	**TBC1 domain family member 5**	Extracellular	other		-1.120511335	0	0.59277824	0	1	0	10.9782313	11.1806514	11.145127	10.7343239
6351	**ENSG0000013137**	CAPN7	protein_coding	chr3	15206152	15252916	+	Human GRCh	OTTHUMG000001	CAPN7	**calpain 7**	Cytoplasm	peptidase		-1.07077511	0	0.73689545	0	1	0	10.8108295	11.0417314	11.0016891	10.6905004
6352	**ENSG0000013137**	RFTN1	protein_coding	chr3	16313574	16514026	-	Human GRCh	OTTHUMG000001	RFTN1	**raftlin, lipid raft linker 1**	Plasma Mem	other		1.973224862	1	0.57519641	0	1	0	2.31974247	1.85014272	1.84074388	2.66930157
6353	**ENSG0000013137**	C3orf20	protein_coding	chr3	14675141	14773036	+	Human GRCh	OTTHUMG000001	C3orf20	**chromosome 3 open reading frame 20**	Cytoplasm	other		-1.579584909	-1	0.46364271	0	1	0	4.44801757	4.68908746	4.40073704	3.9302679
6354	**ENSG0000013138**	RBSN	protein_coding	chr3	15070073	15099163	-	Human GRCh	OTTHUMG000001	RBSN	**rabenosyn, RAB effector**	Cytoplasm	other		-1.050797261	0	0.8082901	0	1	0	10.7351701	11.052487	10.948589	10.7415617
6356	**ENSG0000013138**	SLC6A6	protein_coding	chr3	14402576	14489349	+	Human GRCh	OTTHUMG000001	SLC6A6	**solute carrier family 6 member 6**	Plasma Mem	transporter		-1.25660926	0	0.27893653	0	1	0	11.6350642	12.1276528	11.7021315	11.5989644
6357	**ENSG0000013139**	KCNC3	protein_coding	chr19	50311937	50333515	-	Human GRCh	OTTHUMG000001	KCNC3	**potassium voltage-gated channel subfamily C member 3**	Plasma Mem	ion channel	dalfampric	-1.024735564	0	0.93099805	0	1	0	10.0340701	10.5434632	10.3214407	10.2539158
6358	**ENSG0000013140**	NAPSA	protein_coding	chr19	50358472	50365830	-	Human GRCh	OTTHUMG000001	NAPSA	**napsin A aspartic peptidase**	Extracellular	peptidase		-2.047539062	-1	0.27253189	0	1	0	4.30811833	4.34849387	4.03101943	3.44465976
6359	**ENSG0000013140**	NR1H2	protein_coding	chr19	50329653	50383388	+	Human GRCh	OTTHUMG000001	NR1H2	**nuclear receptor subfamily 1 group H member 2**	Nucleus	ligand-depen	RGX-104	1.028414053	0	0.89888304	0	1	0	9.84920593	9.98416239	9.97484872	9.9236751
6360	**ENSG0000013140**	LRRC4B	protein_coding	chr19	50516892	50568435	-	Human GRCh	OTTHUMG000001	LRRC4B	**leucine rich repeat containing 4B**	Plasma Mem	other		-4.472825712	-1	0.1099928	0	1	0	3.48655281	2.91119158	1.09589655	2.49853466
6361	**ENSG0000013143**	PDLIM4	protein_coding	chr5	132257696	132273454	+	Human GRCh	OTTHUMG000000	PDLIM4	**PDZ and LIM domain 4**	Plasma Mem	other		5.748007273	1	0.44756788	0	NA	0	0.64381843	0.65289579	1.05373727	0.64434138
6362	**ENSG0000013143**	KIF3A	protein_coding	chr5	132692628	132737638	-	Human GRCh	OTTHUMG000000	KIF3A	**kinesin family member 3A**	Cytoplasm	enzyme		1.233006251	0	0.35292366	0	1	0	9.38572289	9.31741211	9.74749118	9.38510337
6363	**ENSG0000013144**	MGAT1	protein_coding	chr5	180784780	180815652	-	Human GRCh	OTTHUMG000001	MGAT1	**alpha-1,3-mannosyl-glycoprotein 2-beta-N-acetylglucosaminyl**	Cytoplasm	enzyme		1.075070888	0	0.72414233	0	1	0	10.5314514	10.7354469	10.7871691	10.6451163
6364	**ENSG0000013145**	GFPT2	protein_coding	chr5	180300698	180353336	-	Human GRCh	OTTHUMG000001	GFPT2	**glutamine-fructose-6-phosphate transaminase 2**	Cytoplasm	enzyme		1.435428523	0	0.49384966	0	1	0	4.03416387	4.1320201	4.10714651	4.55792562
6365	**ENSG0000013146**	TUBG1	protein_coding	chr17	42609641	42615238	+	Human GRCh	OTTHUMG000001	TUBG1	**tubulin gamma 1**	Cytoplasm	other	docetaxel/	-1.145928039	0	0.53452916	0	1	0	11.4441693	11.9458829	11.6414063	11.4867276
6366	**ENSG0000013146**	PSME3	protein_coding	chr17	42824385	42843760	+	Human GRCh	OTTHUMG000001	PSME3	**proteasome activator subunit 3**	Cytoplasm	peptidase		-1.058586707	0	0.75852835	0	1	0	11.833853	12.2279416	12.0769138	11.8807593
6367	**ENSG0000013146**	RPL27	protein_coding	chr17	42998273	43002959	+	Human GRCh	OTTHUMG000001	RPL27	**ribosomal protein L27**	Cytoplasm	other		1.072505377	0	0.7385458	0	1	0	11.1559454	11.5033958	11.4752528	11.3656984
6368	**ENSG0000013147**	PSMC3IP	protein_coding	chr17	42572310	42577831	-	Human GRCh	OTTHUMG000001	PSMC3IP	**PSMC3 interacting protein**	Nucleus	transcription		1.100469364	0	0.64876691	0	1	0	8.89005353	8.99383079	9.14877866	8.93516212
6369	**ENSG0000013147**	AOC3	protein_coding	chr17	42851184	42858130	+	Human GRCh	OTTHUMG000001	AOC3	**amine oxidase copper containing 3**	Plasma Mem	enzyme	ASP8232, I	-1.1343584	0	0.71626374	0	1	0	8.30751719	8.58984934	8.54018049	8.03321802
6370	**ENSG0000013147**	ACLY	protein_coding	chr17	41866917	41930542	-	Human GRCh	OTTHUMG000001	ACLY	**ATP citrate lyase**	Cytoplasm	enzyme	bempedoy	-1.203169849	0	0.40656486	0	1	0	13.4803567	13.9945348	13.6388024	13.4656522
6371	**ENSG0000013147**	VPS25	protein_coding	chr17	42773449	42779599	+	Human GRCh	OTTHUMG000001	VPS25	**vacuolar protein sorting 25 homolog**	Cytoplasm	other		-1.124774478	0	0.5668945	0	1	0	9.88788799	10.3175097	10.0257929	9.95225763
6372	**ENSG0000013147**	RAMP2	protein_coding	chr17	42758447	42763041	+	Human GRCh	OTTHUMG000001	RAMP2	**receptor activity modifying protein 2**	Plasma Mem	G-protein co	pramlintid	8.371453959	1	0.02135441	1	1	0	2.37995542	1.87346217	3.73847496	2.76191284
6373	**ENSG0000013148**	AOC2	protein_coding	chr17	42844580	42850707	+	Human GRCh	OTTHUMG000001	AOC2	**amine oxidase copper containing 2**	Plasma Mem	enzyme		-1.238960669	0	0.448155	0	1	0	9.08565347	9.47853522	9.23013651	8.84590908
6376	**ENSG0000013149**	NDUFA2	protein_coding	chr5	140638740	140647771	-	Human GRCh	OTTHUMG000001	NDUFA2	**NADH:ubiquinone oxidoreductase subunit A2**	Cytoplasm	enzyme		1.035005406	0	0.87658336	0	1	0	9.15392219	9.43872877	9.36121465	9.32420164
6377	**ENSG0000013150**	ANKHD1	protein_coding	chr5	140401814	140539856	+	Human GRCh	OTTHUMG000001	ANKHD1/ANI	**ankyrin repeat and KH domain containing 1**	Cytoplasm	other		-1.142262118	0	0.49860177	0	1	0	13.0025353	13.3321343	13.1450649	12.9036925
6378	**ENSG0000013150**	DIAPH1	protein_coding	chr5	141515016	141619055	-	Human GRCh	OTTHUMG000001	DIAPH1	**diaphanous related formin 1**	Plasma Mem	other		-1.162007323	0	0.44686756	0	1	0	12.929457	13.2524639	13.0459478	12.80914
6379	**ENSG0000013150**	NDFIP1	protein_coding	chr5	142108779	142154440	+	Human GRCh	OTTHUMG000001	NDFIP1	**Nedd4 family interacting protein 1**	Cytoplasm	other		1.092317836	0	0.65707576	0	1	0	10.21914	10.2966766	10.4655809	10.2315193
6380	**ENSG0000013150**	UBE2D2	protein_coding	chr5	139526431	139628434	+	Human GRCh	OTTHUMG000001	UBE2D2	**ubiquitin conjugating enzyme E2 D2**	Cytoplasm	enzyme		-1.005651829	0	0.97742711	0	1	0	11.019132	11.2487163	11.2578004	10.9939514
6383	**ENSG0000013155**	EXOC4	protein_coding	chr7	133253073	134066589	+	Human GRCh	OTTHUMG000001	EXOC4	**exocyst complex component 4**	Cytoplasm	transporter		-1.209429181	0	0.35786079	0	1	0	11.2570164	11.4790496	11.2917337	11.0214848

6384	**ENSG0000013158**	ACAP3	protein_coding	chr1	1292390	1309609	-	Human GRCh	OTTHUMG000000	ACAP3	**ArfGAP with coiled-coil, ankyrin repeat and PH domains 3**	Nucleus	transcription		1.089653597	0	0.69787256	0	1	0	13.2521501	13.5219567	13.6564973	13.3035899
6385	**ENSG0000013159**	C1orf159	protein_coding	chr1	1081818	1116361	-	Human GRCh	OTTHUMG000000	C1orf159	**chromosome 1 open reading frame 159**	Other	other		-1.078327256	0	0.73961875	0	1	0	10.6457126	10.9108657	10.7943365	10.6016608
6386	**ENSG0000013162**	ANO1	protein_coding	chr11	69985907	70189530	+	Human GRCh	OTTHUMG000001	ANO1	**anoctamin 1**	Plasma Mem	ion channel	crofelemer	1.859207325	1	0.67348207	0	1	0	1.09712209	2.86917137	3.103004	2.35866727
6387	**ENSG0000013162**	PPFIA1	protein_coding	chr11	70270690	70385312	+	Human GRCh	OTTHUMG000001	PPFIA1	**PTPRF interacting protein alpha 1**	Plasma Mem	phosphatase		-1.06364088	0	0.78274118	0	1	0	11.2548325	11.6044494	11.5281898	11.1998058
6389	**ENSG0000013165**	KREMEN2	protein_coding	chr16	2964216	2968380	+	Human GRCh	OTTHUMG000001	KREMEN2	**kringle containing transmembrane protein 2**	Other	other		1.545037807	1	0.30576717	0	1	0	7.21981639	7.47989281	7.92866697	7.71291895
6390	**ENSG0000013165**	THOC6	protein_coding	chr16	3024027	3027755	+	Human GRCh	OTTHUMG000001	THOC6	**THO complex subunit 6**	Nucleus	other		1.033362486	0	0.86638528	0	1	0	8.85947675	8.99761324	9.02700757	8.90203391
6391	**ENSG0000013165**	TRAF7	protein_coding	chr16	2155698	2178129	+	Human GRCh	OTTHUMG000001	TRAF7	**TNF receptor associated factor 7**	Cytoplasm	enzyme		1.027536833	0	0.8888692	0	1	0	12.070672	12.4399946	12.4361722	12.1469658
6392	**ENSG0000013166**	BARX1	protein_coding	chr9	93951627	93955355	-	Human GRCh	OTTHUMG000000	BARX1	**BARX homeobox 1**	Nucleus	transcription		1.311190161	0	0.56804723	0	1	0	5.53653239	5.81786059	5.68637768	6.19742885
6393	**ENSG0000013166**	NINJ1	protein_coding	chr9	93121496	93134251	-	Human GRCh	OTTHUMG000000	NINJ1	**ninjurin 1**	Plasma Mem	other		1.20240642	0	0.46744936	0	1	0	7.95576266	8.34769632	8.21890148	8.49327812
6395	**ENSG0000013169**	NPHP4	protein_coding	chr1	5862811	5992473	-	Human GRCh	OTTHUMG000000	NPHP4	**nephrocystin 4**	Cytoplasm	other		-1.119468388	0	0.58542215	0	1	0	10.5177074	10.8534476	10.7360116	10.386346
6396	**ENSG0000013171**	MAP1B	protein_coding	chr5	72107234	72209565	+	Human GRCh	OTTHUMG000001	MAP1B	**microtubule associated protein 1B**	Cytoplasm	other		-1.208730025	0	0.44063299	0	1	0	10.2194289	9.83314093	9.77609736	9.93341218
6398	**ENSG0000013172**	IL13RA1	protein_coding	chrX	118727133	118794535	+	Human GRCh	OTTHUMG000000	IL13RA1	**interleukin 13 receptor subunit alpha 1**	Plasma Mem	transmembra	cintredekir	-1.182791355	0	0.50301476	0	1	0	10.2442722	10.4024309	10.2748444	9.98388193
6399	**ENSG0000013172**	WDR44	protein_coding	chrX	118346073	118449961	+	Human GRCh	OTTHUMG000000	WDR44	**WD repeat domain 44**	Cytoplasm	other		-1.22538813	0	0.50563397	0	1	0	9.25427514	9.54177734	9.24855816	9.1208265
6400	**ENSG0000013173**	CKMT2	protein_coding	chr5	81233320	81266398	+	Human GRCh	OTTHUMG000001	CKMT2	**creatine kinase, mitochondrial 2**	Cytoplasm	kinase		1	0	1	0	1	0	1.34864279	1.39311016	1.38583665	1.35128291
6401	**ENSG0000013173**	ZCCHC9	protein_coding	chr5	81301587	81313297	+	Human GRCh	OTTHUMG000001	ZCCHC9	**zinc finger CCHC-type containing 9**	Nucleus	other		1.157087126	0	0.53542325	0	1	0	8.33473836	8.46432598	8.6373587	8.47533572
6404	**ENSG0000013174**	TNS4	protein_coding	chr17	40475828	40501623	-	Human GRCh	OTTHUMG000001	TNS4	**tensin 4**	Cytoplasm	other		-1.097831011	0	0.91180604	0	1	0	3.88499088	3.76529312	3.32777122	4.04883767
6405	**ENSG0000013174**	TOP2A	protein_coding	chr17	40388525	40417896	-	Human GRCh	OTTHUMG000001	TOP2A	**DNA topoisomerase II alpha**	Nucleus	enzyme	bortezomi	-1.085999806	0	0.71046127	0	1	0	12.9684579	13.292123	13.1945252	12.891488
6406	**ENSG0000013174**	STARD3	protein_coding	chr17	39637090	39664201	+	Human GRCh	OTTHUMG000001	STARD3	**StAR related lipid transfer domain containing 3**	Cytoplasm	transporter		-1.00668803	0	0.97434484	0	1	0	9.79963044	9.85566725	10.008426	9.59718622
6407	**ENSG0000013175**	RARA	protein_coding	chr17	40309180	40357643	+	Human GRCh	OTTHUMG000001	RARA	**retinoic acid receptor alpha**	Nucleus	ligand-depen	daunorubi	1.124407158	0	0.58112324	0	1	0	9.01100959	9.31703745	9.42236845	9.16761663
6408	**ENSG0000013177**	PPP1R1B	protein_coding	chr17	39626740	39636626	+	Human GRCh	OTTHUMG000001	PPP1R1B	**protein phosphatase 1 regulatory inhibitor subunit 1B**	Cytoplasm	phosphatase		1.672281176	1	0.54255307	0	1	0	9.2271108	9.73190316	9.59097647	10.4891102
6409	**ENSG0000013177**	KHDRBS3	protein_coding	chr8	135457456	135656722	+	Human GRCh	OTTHUMG000001	KHDRBS3	**KH RNA binding domain containing, signal transduction associ**	Nucleus	other		1.153683403	0	0.65767439	0	1	0	6.3741729	6.50353652	6.82785032	6.24936797
6410	**ENSG0000013177**	CHD1L	protein_coding	chr1	147242654	147295765	+	Human GRCh	OTTHUMG000001	CHD1L	**chromodomain helicase DNA binding protein 1 like**	Nucleus	enzyme		-1.027458998	0	0.92076172	0	1	0	7.8728539	8.34882459	8.18834344	8.01578127
6411	**ENSG0000013177**	PEX11B	protein_coding	chr1	145911350	145918717	-	Human GRCh	OTTHUMG000000	PEX11B	**peroxisomal biogenesis factor 11 beta**	Cytoplasm	other		-1.154291367	0	0.57273639	0	1	0	9.08925537	9.62580595	9.3777579	9.05714129
6412	**ENSG0000013178**	FMO5	protein_coding	chr1	147175351	147243050	-	Human GRCh	OTTHUMG000000	FMO5	**flavin containing dimethylaniline monoxygenase 5**	Cytoplasm	enzyme		1.075052887	0	0.81064597	0	1	0	9.55717992	9.92388135	9.46391539	10.1194701
6413	**ENSG0000013178**	PIAS3	protein_coding	chr1	145848522	145859836	-	Human GRCh	OTTHUMG000000	PIAS3	**protein inhibitor of activated STAT 3**	Nucleus	transcription		-1.17577598	0	0.47542457	0	1	0	10.9605079	11.2146381	10.9932589	10.8318355
6414	**ENSG0000013179**	PRKAB2	protein_coding	chr1	147155106	147172550	-	Human GRCh	OTTHUMG000000	PRKAB2	**protein kinase AMP-activated non-catalytic subunit beta 2**	Cytoplasm	kinase		-1.129911813	0	0.57687951	0	1	0	10.8998627	11.2455995	10.9724709	10.9255076
6416	**ENSG0000013182**	PDHA1	protein_coding	chrX	19343893	19361718	+	Human GRCh	OTTHUMG000000	PDHA1	**pyruvate dehydrogenase E1 subunit alpha 1**	Cytoplasm	enzyme		-1.054267183	0	0.78387198	0	1	0	11.0835048	11.5430739	11.3687511	11.1744368
6417	**ENSG0000013183**	RAI2	protein_coding	chrX	17800049	17861337	-	Human GRCh	OTTHUMG000000	RAI2	**retinoic acid induced 2**	Nucleus	transcription		1.734817746	1	0.44320055	0	1	0	3.55730914	3.28651921	4.08810966	3.39038591
6418	**ENSG0000013184**	MCCC2	protein_coding	chr5	71579531	71658706	+	Human GRCh	OTTHUMG000001	MCCC2	**methylcrotonyl-CoA carboxylase subunit 2**	Cytoplasm	enzyme		-1.181740387	0	0.6277589	0	1	0	13.6863525	13.9880391	13.9818268	13.1903452
6419	**ENSG0000013184**	ZNF304	protein_coding	chr19	57351271	57359898	+	Human GRCh	OTTHUMG000001	ZNF304	**zinc finger protein 304**	Nucleus	transcription		1.036323644	0	0.89411185	0	1	0	8.46979203	9.02554636	9.06762823	8.50642344
6420	**ENSG0000013184**	ZSCAN5A	protein_coding	chr19	56219670	56368383	-	Human GRCh	OTTHUMG000001	ZSCAN5A	**zinc finger and SCAN domain containing 5A**	Nucleus	transcription		1.008121266	0	0.97953066	0	1	0	9.09802633	9.22330617	9.28557761	9.04509592
6423	**ENSG0000013187**	SELENOS	protein_coding	chr15	101270817	101277500	-	Human GRCh	OTTHUMG000001	SELENOS	**selenoprotein S**	Cytoplasm	other		-1.035440764	0	0.88040529	0	1	0	9.90214417	9.95899876	9.99821232	9.77458083
6424	**ENSG0000013187**	CHSY1	protein_coding	chr15	101175727	101252048	-	Human GRCh	OTTHUMG000001	CHSY1	**chondroitin sulfate synthase 1**	Cytoplasm	enzyme		-1.100770105	0	0.64965251	0	1	0	10.6142333	10.9197289	10.8232787	10.4906244
6425	**ENSG0000013187**	SNRPA1	protein_coding	chr15	101281510	101295282	-	Human GRCh	OTTHUMG000001	SNRPA1	**small nuclear ribonucleoprotein polypeptide A'**	Nucleus	other		-1.054101762	0	0.78238485	0	1	0	9.80819935	10.1330416	9.95107118	9.89445064
6426	**ENSG0000013189**	LLGL1	protein_coding	chr17	18225635	18244875	+	Human GRCh	OTTHUMG000000	LLGL1	**LLGL scribble cell polarity complex component 1**	Cytoplasm	other		1.003370561	0	0.9876807	0	1	0	10.3297058	10.7321322	10.6533956	10.4400727
6427	**ENSG0000013191**	NR0B2	protein_coding	chr1	26911489	26913975	-	Human GRCh	OTTHUMG000000	NR0B2	**nuclear receptor subfamily 0 group B member 2**	Nucleus	ligand-depen		-1.010707927	0	0.99190695	0	1	0	1.79939035	2.57834095	2.5549671	1.80563745
6428	**ENSG0000013191**	LIN28A	protein_coding	chr1	26410817	26429728	+	Human GRCh	OTTHUMG000000	LIN28A	**lin-28 homolog A**	Cytoplasm	other		1.546177846	1	0.39756791	0	1	0	3.55349105	4.51831357	4.36799748	4.62702329
6429	**ENSG0000013193**	THAP1	protein_coding	chr8	42836674	42843325	-	Human GRCh	OTTHUMG000001	THAP1	**THAP domain containing 1**	Nucleus	transcription		1.160760349	0	0.53255277	0	1	0	7.50216649	7.79267158	7.96391498	7.6432508
6430	**ENSG0000013194**	RHPN2	protein_coding	chr19	32978592	33064888	-	Human GRCh	OTTHUMG000001	RHPN2	**rhophilin Rho GTPase binding protein 2**	Cytoplasm	other		-1.033973567	0	0.90486592	0	1	0	8.8997493	9.24179808	9.16936994	8.90971778
6431	**ENSG0000013194**	C19orf12	protein_coding	chr19	29698937	29715789	-	Human GRCh	OTTHUMG000001	C19orf12	**chromosome 19 open reading frame 12**	Cytoplasm	other		-1.087504004	0	0.70770133	0	1	0	8.67707693	8.78796266	8.7876224	8.47552931
6432	**ENSG0000013194**	FAAP24	protein_coding	chr19	32972209	32978229	+	Human GRCh	OTTHUMG000001	FAAP24	**FA core complex associated protein 24**	Nucleus	other		-1.449197905	0	0.23135714	0	1	0	6.93073627	7.29218522	6.87368395	6.58956875
6433	**ENSG0000013195**	LRRC9	protein_coding	chr14	59919713	60066817	+	Human GRCh	OTTHUMG000000	LRRC9	**leucine rich repeat containing 9**	Cytoplasm	other		5.323682516	1	0.18845899	0	1	0	2.0654606	2.16246416	2.14686402	2.65094722
6434	**ENSG0000013196**	ACTR10	protein_coding	chr14	58200080	58235636	+	Human GRCh	OTTHUMG000001	ACTR10	**actin related protein 10**	Cytoplasm	other		1.136128701	0	0.55006988	0	1	0	9.31402907	9.46749857	9.66693897	9.37882751
6436	**ENSG0000013197**	GCH1	protein_coding	chr14	54842008	54902826	-	Human GRCh	OTTHUMG000000	GCH1	**GTP cyclohydrolase 1**	Cytoplasm	enzyme		1.041883326	0	0.87523899	0	1	0	9.23363017	9.5386822	9.53115403	9.3428129
6437	**ENSG0000013198**	LGALS3	protein_coding	chr14	55124110	55145423	+	Human GRCh	OTTHUMG000001	LGALS3	**galectin 3**	Extracellular	other	GCS-100, G	1	0	1	0	NA	0	0.80396848	0.81657634	0.81446726	0.80469788
6439	**ENSG0000013200**	DNAJB1	protein_coding	chr19	14514769	14560391	-	Human GRCh	OTTHUMG000001	DNAJB1	**DnaJ heat shock protein family (Hsp40) member B1**	Nucleus	transcription		1.007360703	0	0.96848747	0	1	0	11.6813102	11.704169	11.8016408	11.5910093
6440	**ENSG0000013200**	ZSWIM4	protein_coding	chr19	13795443	13832254	+	Human GRCh	OTTHUMG000001	ZSWIM4	**zinc finger SWIM-type containing 4**	Other	other		-1.300851613	0	0.1701157	0	1	0	9.49322663	9.6767493	9.35305209	9.24825579
6441	**ENSG0000013200**	FBXW9	protein_coding	chr19	12688053	12696631	-	Human GRCh	OTTHUMG000001	FBXW9	**F-box and WD repeat domain containing 9**	Cytoplasm	other		-1.070210648	0	0.7813583	0	1	0	9.20561796	9.86990157	9.6706819	9.31752456
6442	**ENSG0000013200**	RFX1	protein_coding	chr19	13961530	14007039	-	Human GRCh	OTTHUMG000001	RFX1	**regulatory factor X1**	Nucleus	transcription		-1.02173064	0	0.91708316	0	1	0	10.0694014	10.2809778	10.2666593	10.0341952
6443	**ENSG0000013201**	ZNF20	protein_coding	chr19	12092843	12140355	-	Human GRCh	OTTHUMG000001	ZNF20	**zinc finger protein 20**	Nucleus	transcription		-1.184098774	0	0.46899404	0	1	0	7.85754294	8.05557028	7.80325179	7.75459185
6444	**ENSG0000013201**	BRME1	protein_coding	chr19	13882348	13906452	-	Human GRCh	OTTHUMG000001	BRME1	**break repair meiotic recombinase recruitment factor 1**	Nucleus	other		1.410488544	0	0.24604074	0	1	0	7.69870816	8.22276386	8.75647365	7.75665163
6445	**ENSG0000013201**	DCAF15	protein_coding	chr19	13952509	13961449	+	Human GRCh	OTTHUMG000001	DCAF15	**DDB1 and CUL4 associated factor 15**	Other	other		-1.112481901	0	0.60570992	0	1	0	10.3383076	10.6410948	10.3936707	10.3717353
6446	**ENSG0000013202**	CC2D1A	protein_coding	chr19	13906201	13930879	+	Human GRCh	OTTHUMG000001	CC2D1A	**coiled-coil and C2 domain containing 1A**	Cytoplasm	transcription		1.068149372	0	0.73215021	0	1	0	11.1802323	11.39737	11.4971472	11.2195
6447	**ENSG0000013202**	RTBDN	protein_coding	chr19	12825478	12835428	-	Human GRCh	OTTHUMG000001	RTBDN	**retbindin**	Extracellular	other		1.213255272	0	0.77757109	0	1	0	3.59020482	4.3143461	3.1009569	4.68165354
6449	**ENSG0000013210**	TRIM21	protein_coding	chr11	4384897	4393702	-	Human GRCh	OTTHUMG000001	TRIM21	**tripartite motif containing 21**	Nucleus	enzyme		1.503483632	1	0.16103584	0	1	0	5.92747682	5.98169763	6.43808713	6.26131503
6450	**ENSG0000013212**	SPATA6	protein_coding	chr1	48295373	48472208	-	Human GRCh	OTTHUMG000000	SPATA6	**spermatogenesis associated 6**	Cytoplasm	other		1.507882689	1	0.20576654	0	1	0	6.9961688	7.05568666	7.36207492	7.51993937
6451	**ENSG0000013212**	LRRC41	protein_coding	chr1	46261196	46303616	-	Human GRCh	OTTHUMG000000	LRRC41	**leucine rich repeat containing 41**	Cytoplasm	other		-1.078028176	0	0.71778273	0	1	0	11.0707652	11.3324446	11.2827538	10.9442669
6452	**ENSG0000013214**	CCT6B	protein_coding	chr17	34927859	34981078	-	Human GRCh	OTTHUMG000001	CCT6B	**chaperonin containing TCP1 subunit 6B**	Cytoplasm	other		1.246441845	0	0.56131147	0	1	0	6.57009758	6.29357145	6.82349455	6.49210418
6453	**ENSG0000013215**	DHX30	protein_coding	chr3	47802909	47850195	+	Human GRCh	OTTHUMG000001	DHX30	**DExH-box helicase 30**	Nucleus	enzyme		-1.05778287	0	0.7680435	0	1	0	11.9508625	12.2759533	12.2101729	11.891291
6454	**ENSG0000013215**	RAF1	protein_coding	chr3	12582101	12664201	-	Human GRCh	OTTHUMG000001	RAF1	**Raf-1 proto-oncogene, serine/threonine kinase**	Cytoplasm	kinase	regorafeni	-1.113479637	0	0.58333547	0	1	0	12.124916	12.4533353	12.2822584	12.071973
6455	**ENSG0000013216**	SLC6A11	protein_coding	chr3	10816201	10940714	+	Human GRCh	OTTHUMG000001	SLC6A11	**solute carrier family 6 member 11**	Plasma Mem	transporter	tiagabine	1.31495736	0	0.62790385	0	1	0	4.63836942	4.59290315	5.15531059	4.59205086
6456	**ENSG0000013217**	PPARG	protein_coding	chr3	12287368	12434356	+	Human GRCh	OTTHUMG000001	PPARG	**peroxisome proliferator activated receptor gamma**	Nucleus	ligand-depen	T3D-959, p	3.281451486	1	0.18455179	0	1	0	5.38310425	5.4113483	5.57730205	7.32610908
6457	**ENSG0000013218**	NUP210	protein_coding	chr3	13316235	13420322	-	Human GRCh	OTTHUMG000001	NUP210	**nucleoporin 210**	Nucleus	transporter		-1.194716294	0	0.35765953	0	1	0	12.824241	13.1036485	12.8505763	12.7026242
6459	**ENSG0000013219**	HSD17B7	protein_coding	chr1	162790702	162812823	+	Human GRCh	OTTHUMG000000	HSD17B7	**hydroxysteroid 17-beta dehydrogenase 7**	Cytoplasm	enzyme		-1.151548947	0	0.59314455	0	1	0	9.64541342	10.194589	9.88330534	9.69215721
6460	**ENSG0000013219**	ENOSF1	protein_coding	chr18	670318	712662	-	Human GRCh	OTTHUMG000001	ENOSF1	**enolase superfamily member 1**	Other	enzyme		-1.068988255	0	0.80928006	0	1	0	12.2040538	12.3935886	12.3971975	12.0262101
6462	**ENSG0000013220**	EMILIN2	protein_coding	chr18	2847006	2916003	+	Human GRCh	OTTHUMG000001	EMILIN2	**elastin microfibril interfacer 2**	Extracellular	other		-2.218405354	-1	0.28187331	0	1	0	3.65611673	2.28420682	2.2701922	3.09764265
6463	**ENSG0000013220**	SLX1A	protein_coding	chr16	30193875	30197561	+	Human GRCh	OTTHUMG000001	SLX1A/SLX1B	**SLX1 homolog A, structure-specific endonuclease subunit**	Nucleus	enzyme		2.802301951	1	0.04894524	1	1	0	4.99891106	3.96543631	5.84622309	5.0675678
6464	**ENSG0000013225**	ARFIP2	protein_coding	chr11	6474683	6481479	-	Human GRCh	OTTHUMG000001	ARFIP2	**ADP ribosylation factor interacting protein 2**	Cytoplasm	other		-1.061740398	0	0.80050903	0	1	0	11.1327317	11.5397095	11.4421617	11.1068211
6465	**ENSG0000013225**	TRIM5	protein_coding	chr11	5663195	5938619	-	Human GRCh	OTTHUMG000000	TRIM5	**tripartite motif containing 5**	Cytoplasm	enzyme		-1.218730306	0	0.46337065	0	1	0	8.61436028	8.6767976	8.46840639	8.36213237
6468	**ENSG0000013227**	RRP8	protein_coding	chr11	6595072	6603616	-	Human GRCh	OTTHUMG000001	RRP8	**ribosomal RNA processing 8**	Nucleus	enzyme		-1.061979901	0	0.78303744	0	1	0	10.061966	10.1775002	10.2728004	9.78636901
6469	**ENSG0000013228**	TIMM10B	protein_coding	chr11	6481501	6484681	+	Human GRCh	OTTHUMG000001	TIMM10B	**translocase of inner mitochondrial membrane 10B**	Cytoplasm	transporter		-1.073091524	0	0.75498283	0	1	0	10.3307339	10.7039823	10.5787884	10.3129402
6470	**ENSG0000013229**	EFR3A	protein_coding	chr8	131904093	132013642	+	Human GRCh	OTTHUMG000001	EFR3A	**EFR3 homolog A**	Plasma Mem	other		-1.038109162	0	0.85304462	0	1	0	10.1471245	10.316581	10.3081914	10.0695683
6472	**ENSG0000013230**	PTCD3	protein_coding	chr2	86106223	86142157	+	Human GRCh	OTTHUMG000001	PTCD3	**pentatricopeptide repeat domain 3**	Cytoplasm	other		-1.096596217	0	0.68087426	0	1	0	11.8025776	12.0194492	11.9919717	11.6034646
6473	**ENSG0000013230**	IMMT	protein_coding	chr2	86143932	86195472	-	Human GRCh	OTTHUMG000001	IMMT	**inner membrane mitochondrial protein**	Cytoplasm	other		-1.242301173	0	0.30567024	0	1	0	11.784163	12.1357982	11.8397665	11.6063486
6474	**ENSG0000013231**	MRPL35	protein_coding	chr2	86199355	86213790	+	Human GRCh	OTTHUMG000000	MRPL35	**mitochondrial ribosomal protein L35**	Cytoplasm	other		1.073747698	0	0.74125922	0	1	0	10.0670282	10.3794236	10.4501041	10.1605438
6476	**ENSG0000013232**	ILKAP	protein_coding	chr2	238170402	238203708	-	Human GRCh	OTTHUMG000001	ILKAP	**ILK associated serine/threonine phosphatase**	Cytoplasm	phosphatase		-1.045867544	0	0.82384924	0	1	0	10.0507603	10.3186541	10.3425732	9.90139812
6477	**ENSG0000013232**	PER2	protein_coding	chr2	238244044	238290102	-	Human GRCh	OTTHUMG000001	PER2	**period circadian regulator 2**	Nucleus	transcription		-1.080129883	0	0.66571829	0	1	0	11.7449977	11.8553743	11.7445306	11.6818486
6478	**ENSG0000013232**	RAMP1	protein_coding	chr2	237858893	237912106	+	Human GRCh	OTTHUMG000001	RAMP1	**receptor activity modifying protein 1**	Plasma Mem	G-protein co	pramlintid	1.947287825	1	0.10751776	0	1	0	7.73079087	7.73454295	8.55469832	8.29779915
6479	**ENSG0000013233**	SCLY	protein_coding	chr2	238060924	238099413	+	Human GRCh	OTTHUMG000001	SCLY	**selenocysteine lyase**	Cytoplasm	enzyme		-1.052543391	0	0.83792845	0	1	0	10.309333	10.5685856	10.6473618	10.0564456
6480	**ENSG0000013233**	PTPRE	protein_coding	chr10	127907103	128085855	+	Human GRCh	OTTHUMG000000	PTPRE	**protein tyrosine phosphatase receptor type E**	Plasma Mem	phosphatase		-1.250379187	0	0.67087676	0	1	0	4.58478632	3.87087084	4.13913906	4.04599834
6481	**ENSG0000013234**	RAN	protein_coding	chr12	130872037	130877678	+	Human GRCh	OTTHUMG000001	RAN	**RAN, member RAS oncogene family**	Nucleus	enzyme		-1.202299861	0	0.35104873	0	1	0	12.680237	12.9823077	12.7451822	12.5122248
6482	**ENSG0000013235**	PRKAA1	protein_coding	chr5	40759389	40798374	-	Human GRCh	OTTHUMG000001	PRKAA1	**protein kinase AMP-activated catalytic subunit alpha 1**	Cytoplasm	kinase	phenformi	-1.104186667	0	0.65841762	0	1	0	11.4203055	11.4231573	11.4654987	11.1363719
6483	**ENSG0000013235**	CARD6	protein_coding	chr5	40841308	40860175	+	Human GRCh	OTTHUMG000000	CARD6	**caspase recruitment domain family member 6**	Cytoplasm	other		-33.04738361	-1	0.00642496	1	1	0	2.99625309	3.11637032	2.21534599	2.19445449

6484	**ENSG0000013235**	RAP1GAP2	protein_coding	chr17	2755705	3037741	+	Human GRCh	OTTHUMG000001	RAP1GAP2	**RAP1 GTPase activating protein 2**	Cytoplasm	other		-1.009872194	0	0.96336705	0	1	0	11.0701117	11.2789108	11.343702	10.9620652
6485	**ENSG0000013236**	CLUH	protein_coding	chr17	2689386	2712663	-	Human GRCh	OTTHUMG000001	CLUH	**clustered mitochondria homolog**	Cytoplasm	translation re		1.117223291	0	0.58631098	0	1	0	12.5614644	13.0474214	13.1348617	12.7462166
6486	**ENSG0000013237**	INPP5K	protein_coding	chr17	1494577	1516742	-	Human GRCh	OTTHUMG000001	INPP5K	**inositol polyphosphate-5-phosphatase K**	Cytoplasm	phosphatase		-1.204018749	0	0.44164561	0	1	0	10.1144713	10.3637214	10.1534974	9.90862869
6487	**ENSG0000013238**	MYBBP1A	protein_coding	chr17	4538897	4555386	-	Human GRCh	OTTHUMG000000	MYBBP1A	**MYB binding protein 1a**	Nucleus	transcription		1.049089711	0	0.80696555	0	1	0	12.2043925	12.544851	12.5956504	12.2648937
6488	**ENSG0000013238**	RPA1	protein_coding	chr17	1829702	1900082	+	Human GRCh	OTTHUMG000000	RPA1	**replication protein A1**	Nucleus	other		-1.088060364	0	0.71442927	0	1	0	11.4682986	11.9465787	11.7746313	11.4793347
6489	**ENSG0000013238**	SERPINF1	protein_coding	chr17	1762029	1777565	+	Human GRCh	OTTHUMG000000	SERPINF1	**serpin family F member 1**	Extracellular	other		1.6942334	1	0.54538608	0	1	0	5.19300699	6.43265806	6.81896381	6.02123852
6490	**ENSG0000013238**	UBE2G1	protein_coding	chr17	4269259	4366675	-	Human GRCh	OTTHUMG000001	UBE2G1	**ubiquitin conjugating enzyme E2 G1**	Cytoplasm	enzyme		-1.048470828	0	0.84056778	0	1	0	11.3772179	11.5975582	11.5908137	11.263323
6491	**ENSG0000013239**	EEFSEC	protein_coding	chr3	128153481	128408646	+	Human GRCh	OTTHUMG000001	EEFSEC	**eukaryotic elongation factor, selenocysteine-tRNA specific**	Cytoplasm	translation re		-1.092209323	0	0.69314073	0	1	0	9.42388749	9.67964376	9.50212363	9.41997728
6492	**ENSG0000013240**	TBC1D14	protein_coding	chr4	6909242	7033118	+	Human GRCh	OTTHUMG000000	TBC1D14	**TBC1 domain family member 14**	Extracellular	other		-1.109771275	0	0.61124345	0	1	0	11.7912496	12.0083462	11.9358534	11.617594
6493	**ENSG0000013240**	TMEM128	protein_coding	chr4	4235542	4248223	-	Human GRCh	OTTHUMG000001	TMEM128	**transmembrane protein 128**	Other	other		-1.119951638	0	0.63351313	0	1	0	8.47309611	8.60187756	8.67036924	8.0942685
6494	**ENSG0000013242**	COQ3	protein_coding	chr6	99369401	99394195	-	Human GRCh	OTTHUMG000000	COQ3	**coenzyme Q3, methyltransferase**	Cytoplasm	enzyme		-1.152217118	0	0.6138816	0	1	0	7.72699064	8.19662021	7.99255069	7.64392233
6495	**ENSG0000013242**	PNISR	protein_coding	chr6	99397629	99425331	-	Human GRCh	OTTHUMG000000	PNISR	**PNN interacting serine and arginine rich protein**	Nucleus	other		-1.00176092	0	0.99320064	0	1	0	11.9469705	12.2340496	12.2388763	11.9363249
6496	**ENSG0000013242**	POPDC3	protein_coding	chr6	105157900	105180014	-	Human GRCh	OTTHUMG000000	POPDC3	**popeye domain containing 3**	Other	other		1.010944234	0	0.98340035	0	1	0	5.04710123	4.58016148	4.55087654	5.08259462
6497	**ENSG0000013243**	SEC61G	protein_coding	chr7	54752250	54759974	-	Human GRCh	OTTHUMG000000	SEC61G	**SEC61 translocon subunit gamma**	Plasma Mem	transporter		-1.064747202	0	0.77190306	0	1	0	9.70938612	9.94208977	9.87261588	9.63855326
6498	**ENSG0000013243**	LANCL2	protein_coding	chr7	55365337	55433737	+	Human GRCh	OTTHUMG000000	LANCL2	**LanC like glutathione S-transferase 2**	Plasma Mem	other		-1.080765615	0	0.72160839	0	1	0	8.83789755	9.09350732	9.04895182	8.70620742
6499	**ENSG0000013243**	FIGNL1	protein_coding	chr7	50444128	50450390	-	Human GRCh	OTTHUMG000000	FIGNL1	**fidgetin like 1**	Nucleus	enzyme		1.091909918	0	0.69589153	0	1	0	9.88613781	10.148484	10.2623292	9.95353109
6500	**ENSG0000013243**	DDC	protein_coding	chr7	50458436	50565405	-	Human GRCh	OTTHUMG000000	DDC	**dopa decarboxylase**	Cytoplasm	enzyme	DOPA deca	-11.71736682	-1	0.0827093	0	1	0	3.37136329	4.16948936	3.44464757	3.37659409
6502	**ENSG0000013246**	GRSF1	protein_coding	chr4	70815783	70839897	-	Human GRCh	OTTHUMG000001	GRSF1	**G-rich RNA sequence binding factor 1**	Cytoplasm	other		1.026438412	0	0.90066425	0	1	0	11.7709611	12.1790026	12.1943478	11.8207692
6505	**ENSG0000013246**	ANKRD17	protein_coding	chr4	73073376	73258798	-	Human GRCh	OTTHUMG000001	ANKRD17	**ankyrin repeat domain 17**	Nucleus	other		-1.118387733	0	0.56348189	0	1	0	12.8302216	13.1750738	12.9988367	12.7684754
6506	**ENSG0000013246**	UTP3	protein_coding	chr4	70688532	70690551	+	Human GRCh	OTTHUMG000001	UTP3	**UTP3 small subunit processome component**	Nucleus	other		1.118030506	0	0.56582018	0	1	0	9.30784662	9.52986978	9.64367913	9.44743182
6507	**ENSG0000013247**	ITGB4	protein_coding	chr17	75721328	75757818	+	Human GRCh	OTTHUMG000001	ITGB4	**integrin subunit beta 4**	Plasma Mem	transmembra		1.417553432	0	0.43911402	0	1	0	5.17363729	4.88045859	5.42854236	5.27651169
6508	**ENSG0000013247**	WBP2	protein_coding	chr17	75845699	75856507	-	Human GRCh	OTTHUMG000001	WBP2	**WW domain binding protein 2**	Cytoplasm	transcription		-1.056952351	0	0.79557406	0	1	0	11.3987008	11.6466537	11.4844746	11.4483756
6509	**ENSG0000013247**	H3-3B	protein_coding	chr17	75776434	75785893	-	Human GRCh	OTTHUMG000001	H3-3A/H3-3B	**H3.3 histone A**	Nucleus	other		-1.138583648	0	0.50444654	0	1	0	12.6477215	12.8778019	12.742879	12.4907408
6510	**ENSG0000013247**	UNK	protein_coding	chr17	75784806	75825799	+	Human GRCh	OTTHUMG000001	UNK	**unk zinc finger**	Nucleus	transporter		-1.063765863	0	0.78305351	0	1	0	10.1954756	10.432244	10.3654753	10.130147
6511	**ENSG0000013248**	TRIM47	protein_coding	chr17	75874164	75878581	-	Human GRCh	OTTHUMG000001	TRIM47	**tripartite motif containing 47**	Cytoplasm	enzyme		1.353530105	0	0.32270751	0	1	0	6.73623675	6.13302577	6.77032712	6.77053079
6512	**ENSG0000013248**	ZRANB2	protein_coding	chr1	71063291	71081289	-	Human GRCh	OTTHUMG000000	ZRANB2	**zinc finger RANBP2-type containing 2**	Nucleus	other		1.002565313	0	0.98921624	0	1	0	11.383588	11.5814733	11.6373997	11.3202969
6513	**ENSG0000013250**	EIF5A	protein_coding	chr17	7306999	7312463	+	Human GRCh	OTTHUMG000001	EIF5A	**eukaryotic translation initiation factor 5A**	Cytoplasm	translation re	SNS01-T na	1.008830469	0	0.96643701	0	1	0	13.8352257	14.1190624	14.0763246	13.9090579
6514	**ENSG0000013251**	KDM6B	protein_coding	chr17	7834217	7854796	+	Human GRCh	OTTHUMG000001	KDM6B	**lysine demethylase 6B**	Extracellular	enzyme		1.08076469	0	0.71944433	0	1	0	13.0117252	13.3184914	13.399964	13.1108224
6516	**ENSG0000013251**	SLC52A1	protein_coding	chr17	5032600	5052009	-	Human GRCh	OTTHUMG000000	SLC52A1	**solute carrier family 52 member 1**	Plasma Mem	transporter		13.12908175	1	0.04197006	1	1	0	1.66303179	1.72904036	2.07319357	2.61056965
6517	**ENSG0000013251**	GUCY2D	protein_coding	chr17	8002615	8020342	+	Human GRCh	OTTHUMG000001	GUCY2D	**guanylate cyclase 2D, retinal**	Plasma Mem	kinase		-1.047631395	0	0.87177126	0	1	0	8.13234864	8.42602522	8.30724096	8.15245063
6518	**ENSG0000013252**	GPS2	protein_coding	chr17	7311324	7315564	-	Human GRCh	OTTHUMG000001	GPS2	**G protein pathway suppressor 2**	Nucleus	transcription		-1.060079627	0	0.76138214	0	1	0	10.4065335	10.7476593	10.539182	10.5085586
6520	**ENSG0000013253**	DLG4	protein_coding	chr17	7187187	7219841	-	Human GRCh	OTTHUMG000001	DLG4	**discs large MAGUK scaffold protein 4**	Plasma Mem	kinase		-1.01732709	0	0.94859069	0	1	0	8.15202007	8.51488293	8.35589445	8.29918556
6521	**ENSG0000013254**	RIDA	protein_coding	chr8	98102344	98117171	-	Human GRCh	OTTHUMG000001	RIDA	**reactive intermediate imine deaminase A homolog**	Cytoplasm	enzyme		1.059296868	0	0.85240181	0	1	0	7.31468127	7.42420488	7.63459783	7.18480534
6522	**ENSG0000013254**	VPS13B	protein_coding	chr8	99013266	99877580	+	Human GRCh	OTTHUMG000001	VPS13B	**vacuolar protein sorting 13 homolog B**	Nucleus	transporter		-1.158136719	0	0.50815522	0	1	0	11.8706065	12.0904143	11.9467558	11.6923003
6523	**ENSG0000013255**	RGS22	protein_coding	chr8	99960936	100131268	-	Human GRCh	OTTHUMG000001	RGS22	**regulator of G protein signaling 22**	Cytoplasm	other		-2.01039961	-1	0.39298052	0	1	0	3.28656502	3.80569015	3.11082281	3.09113941
6524	**ENSG0000013256**	MATN2	protein_coding	chr8	97868840	98036724	+	Human GRCh	OTTHUMG000001	MATN2	**matrilin 2**	Extracellular	other		-1.840147201	-1	0.35341036	0	1	0	4.87648033	4.87914872	4.26487161	4.58296113
6525	**ENSG0000013256**	REEP2	protein_coding	chr5	138439057	138446969	+	Human GRCh	OTTHUMG000001	REEP2	**receptor accessory protein 2**	Plasma Mem	other		1.536547708	1	0.27517204	0	1	0	7.39560716	7.23846062	7.89577034	7.65408661
6526	**ENSG0000013257**	PCBD2	protein_coding	chr5	134905120	135007959	+	Human GRCh	OTTHUMG000001	PCBD2	**pterin-4 alpha-carbinolamine dehydratase 2**	Nucleus	enzyme		-1.335556532	0	0.194177	0	1	0	10.4139995	10.4936505	10.2955649	9.95109255
6527	**ENSG0000013258**	SDF2	protein_coding	chr17	28648346	28662189	-	Human GRCh	OTTHUMG000001	SDF2	**stromal cell derived factor 2**	Extracellular	enzyme		-1.114589253	0	0.63008829	0	1	0	9.28085895	9.5922	9.48837905	9.14217816
6528	**ENSG0000013258**	FLOT2	protein_coding	chr17	28879335	28897733	-	Human GRCh	OTTHUMG000001	FLOT2	**flotillin 2**	Plasma Mem	other		-1.174084083	0	0.44651226	0	1	0	11.8373045	12.1251256	11.9520644	11.6482134
6529	**ENSG0000013259**	ERAL1	protein_coding	chr17	28855010	28861061	+	Human GRCh	OTTHUMG000001	ERAL1	**Era like 12S mitochondrial rRNA chaperone 1**	Cytoplasm	other		-1.16259569	0	0.45419638	0	1	0	10.6786861	10.9987683	10.8151651	10.5285903
6530	**ENSG0000013260**	PRMT7	protein_coding	chr16	68310951	68360852	+	Human GRCh	OTTHUMG000001	PRMT7	**protein arginine methyltransferase 7**	Cytoplasm	enzyme		-1.034382377	0	0.87027347	0	1	0	10.6973187	10.8145766	10.8819698	10.5319172
6531	**ENSG0000013260**	NIP7	protein_coding	chr16	69337996	69343106	+	Human GRCh	OTTHUMG000001	NIP7	**nucleolar pre-rRNA processing protein NIP7**	Nucleus	other		-1.008959778	0	0.96779819	0	1	0	9.4745639	9.66431367	9.72203793	9.3799362
6532	**ENSG0000013260**	TERF2	protein_coding	chr16	69355567	69408571	-	Human GRCh	OTTHUMG000001	TERF2	**telomeric repeat binding factor 2**	Nucleus	transcription		-1.192136468	0	0.40645085	0	1	0	10.3109678	10.6631196	10.4384631	10.1504853
6533	**ENSG0000013261**	VPS4A	protein_coding	chr16	69311350	69326939	+	Human GRCh	OTTHUMG000001	VPS4A	**vacuolar protein sorting 4 homolog A**	Cytoplasm	enzyme		1.012377809	0	0.94718945	0	1	0	11.0881362	11.3431561	11.2847253	11.1858247
6534	**ENSG0000013261**	MTSS2	protein_coding	chr16	70661204	70686053	-	Human GRCh	OTTHUMG000001	MTSS2	**MTSS I-BAR domain containing 2**	Plasma Mem	other		1.066269467	0	0.72843195	0	1	0	11.6489268	11.8671764	11.9574706	11.6993636
6535	**ENSG0000013262**	HSPA12B	protein_coding	chr20	3732685	3753111	+	Human GRCh	OTTHUMG000000	HSPA12B	**heat shock protein family A (Hsp70) member 12B**	Other	other		-1.969463007	-1	0.51362988	0	1	0	2.73500591	2.63526667	2.48617055	2.27155758
6536	**ENSG0000013262**	ANKEF1	protein_coding	chr20	9986126	10058303	+	Human GRCh	OTTHUMG000000	ANKEF1	**ankyrin repeat and EF-hand domain containing 1**	Nucleus	transcription		-1.1563289	0	0.52172938	0	1	0	9.14287418	9.18247438	9.1285414	8.86492953
6538	**ENSG0000013263**	PCED1A	protein_coding	chr20	2835314	2841190	-	Human GRCh	OTTHUMG000000	PCED1A	**PC-esterase domain containing 1A**	Other	other		-1.064297214	0	0.80461987	0	1	0	8.49753981	8.70110602	8.67867953	8.38064613
6539	**ENSG0000013263**	SNAP25	protein_coding	chr20	10172395	10308258	+	Human GRCh	OTTHUMG000000	SNAP25	**synaptosome associated protein 25**	Plasma Mem	transporter	botulinum	-1.197258546	0	0.71472698	0	1	0	7.22498246	7.46390519	7.31301499	7.02568653
6540	**ENSG0000013264**	BTBD3	protein_coding	chr20	11890723	11926609	+	Human GRCh	OTTHUMG000000	BTBD3	**BTB domain containing 3**	Other	other		1.069108026	0	0.76740579	0	1	0	10.067173	10.2950574	10.4176213	10.0729667
6541	**ENSG0000013264**	PCNA	protein_coding	chr20	5114953	5126626	-	Human GRCh	OTTHUMG000000	PCNA	**proliferating cell nuclear antigen**	Nucleus	enzyme	AOH1996	-1.195002352	0	0.47924938	0	1	0	11.435968	11.8632855	11.5883583	11.3475431
6542	**ENSG0000013266**	NXT1	protein_coding	chr20	23350791	23354771	+	Human GRCh	OTTHUMG000000	NXT1	**nuclear transport factor 2 like export factor 1**	Nucleus	transporter		-1.163424189	0	0.53868153	0	1	0	8.12800622	8.57841169	8.38480427	8.00451262
6543	**ENSG0000013266**	POLR3F	protein_coding	chr20	18466878	18484648	+	Human GRCh	OTTHUMG000000	POLR3F	**RNA polymerase III subunit F**	Nucleus	enzyme		-1.050508889	0	0.83465698	0	1	0	9.03512599	9.29378649	9.33215227	8.85531122
6544	**ENSG0000013266**	RIN2	protein_coding	chr20	19757606	20002457	+	Human GRCh	OTTHUMG000000	RIN2	**Ras and Rab interactor 2**	Cytoplasm	other		1.218182881	0	0.34234776	0	1	0	8.9488124	9.05442362	9.36080296	9.05334767
6545	**ENSG0000013267**	PTPRA	protein_coding	chr20	2873420	3039076	+	Human GRCh	OTTHUMG000000	PTPRA	**protein tyrosine phosphatase receptor type A**	Plasma Mem	phosphatase		-1.214081384	0	0.31495807	0	1	0	10.5926347	10.8432271	10.5628093	10.4616389
6547	**ENSG0000013267**	DAP3	protein_coding	chr1	155687960	155739010	+	Human GRCh	OTTHUMG000000	DAP3	**death associated protein 3**	Cytoplasm	other		-1.196944591	0	0.32838878	0	1	0	10.5508886	10.8720172	10.6726991	10.3543999
6548	**ENSG0000013267**	RHBG	protein_coding	chr1	156369211	156385219	+	Human GRCh	OTTHUMG000000	RHBG	**Rh family B glycoprotein**	Plasma Mem	transporter		-1.71534486	-1	0.55551583	0	NA	0	2.98696168	2.87276743	2.38114503	2.84772841
6549	**ENSG0000013268**	KHDC4	protein_coding	chr1	155913045	155934413	-	Human GRCh	OTTHUMG000000	KHDC4	**KH domain containing 4, pre-mRNA splicing factor**	Nucleus	other		-1.008796103	0	0.96275574	0	1	0	11.6941348	11.9636072	11.94767	11.6920149
6550	**ENSG0000013268**	ATP1A4	protein_coding	chr1	160151586	160186980	+	Human GRCh	OTTHUMG000000	ATP1A4	**ATPase Na+/K+ transporting subunit alpha 4**	Plasma Mem	transporter	digoxin, et	-1.033974863	0	0.9614247	0	1	0	7.55380464	7.45555253	7.65009378	7.24984263
6551	**ENSG0000013268**	NES	protein_coding	chr1	156668763	156677407	-	Human GRCh	OTTHUMG000000	NES	**nestin**	Extracellular	other		-1.111040413	0	0.85441013	0	1	0	8.24444182	8.18805567	8.0780993	8.14858637
6552	**ENSG0000013269**	BCAN	protein_coding	chr1	156641390	156659532	+	Human GRCh	OTTHUMG000000	BCAN	**brevican**	Extracellular	other		1.661572031	1	0.50037562	0	1	0	3.70468026	3.02254694	2.99763952	4.34295013
6554	**ENSG0000013269**	ARHGEF11	protein_coding	chr1	156934840	157045742	-	Human GRCh	OTTHUMG000000	ARHGEF11	**Rho guanine nucleotide exchange factor 11**	Cytoplasm	other		-1.102591246	0	0.58296599	0	1	0	11.608512	11.8246024	11.6646472	11.560266
6555	**ENSG0000013269**	RAB25	protein_coding	chr1	156061160	156070504	+	Human GRCh	OTTHUMG000000	RAB25	**RAB25, member RAS oncogene family**	Cytoplasm	enzyme		1.939468892	1	0.39526872	0	1	0	4.07967281	4.27153185	4.61879566	4.95996212
6556	**ENSG0000013270**	HAPLN2	protein_coding	chr1	156619331	156625725	+	Human GRCh	OTTHUMG000000	HAPLN2	**hyaluronan and proteoglycan link protein 2**	Extracellular	other		1.002506022	0	0.99480385	0	1	0	8.28829818	8.41860581	8.59533445	8.06341959
6559	**ENSG0000013271**	DCAF8	protein_coding	chr1	160215715	160262549	-	Human GRCh	OTTHUMG000000	DCAF8	**DDB1 and CUL4 associated factor 8**	Nucleus	other		-1.193399815	0	0.35003794	0	1	0	12.6639427	12.9507045	12.7626476	12.4524769
6561	**ENSG0000013274**	IGHMBP2	protein_coding	chr11	68903863	68940602	+	Human GRCh	OTTHUMG000001	IGHMBP2	**immunoglobulin mu DNA binding protein 2**	Nucleus	enzyme		-1.033487284	0	0.88425178	0	1	0	10.3679383	10.5351876	10.6002972	10.2025511
6562	**ENSG0000013274**	ACY3	protein_coding	chr11	67642555	67650730	-	Human GRCh	OTTHUMG000001	ACY3	**aminoacylase 3**	Cytoplasm	enzyme		-5.272420844	-1	0.25371098	0	1	0	2.26539201	1.86679858	1.86103963	1.834193
6563	**ENSG0000013274**	ALDH3B2	protein_coding	chr11	67662155	67681224	-	Human GRCh	OTTHUMG000001	ALDH3B2	**aldehyde dehydrogenase 3 family member B2**	Cytoplasm	enzyme		1.62554082	1	0.5729472	0	1	0	3.2070903	3.19846265	2.74647568	4.01582246
6564	**ENSG0000013274**	TESMIN	protein_coding	chr11	68707440	68751520	-	Human GRCh	OTTHUMG000001	TESMIN	**testis expressed metallothionein like protein**	Cytoplasm	other		-1.210429343	0	0.44444266	0	1	0	9.88652681	10.2446871	10.0709402	9.60404086
6565	**ENSG0000013276**	MMACHC	protein_coding	chr1	45500300	45513382	+	Human GRCh	OTTHUMG000000	MMACHC	**metabolism of cobalamin associated C**	Cytoplasm	enzyme		-1.012271818	0	0.9556853	0	1	0	10.5835759	11.0300576	10.9174553	10.6998173
6566	**ENSG0000013276**	DPH2	protein_coding	chr1	43970000	43973369	+	Human GRCh	OTTHUMG000000	DPH2	**diphthamide biosynthesis 2**	Cytoplasm	enzyme		-1.017907119	0	0.93979403	0	1	0	10.3338683	10.8262189	10.6941601	10.4657009
6567	**ENSG0000013277**	TOE1	protein_coding	chr1	45340052	45343973	+	Human GRCh	OTTHUMG000000	TOE1	**target of EGR1, exonuclease**	Nucleus	enzyme		-1.073460591	0	0.74363657	0	1	0	9.03533787	9.22835224	9.16697668	8.9395588
6568	**ENSG0000013278**	NASP	protein_coding	chr1	45583846	45618904	+	Human GRCh	OTTHUMG000000	NASP	**nuclear autoantigenic sperm protein**	Nucleus	other		-1.039114605	0	0.83181444	0	1	0	12.2904277	12.5538237	12.5026546	12.2602678
6569	**ENSG0000013278**	MUTYH	protein_coding	chr1	45329163	45340893	-	Human GRCh	OTTHUMG000000	MUTYH	**mutY DNA glycosylase**	Nucleus	enzyme		-1.129110258	0	0.57503444	0	1	0	10.0945959	10.1126839	10.0837106	9.84616025
6570	**ENSG0000013279**	CTNNBL1	protein_coding	chr20	37693955	37872129	+	Human GRCh	OTTHUMG000000	CTNNBL1	**catenin beta like 1**	Nucleus	other		-1.000480673	0	0.99805242	0	1	0	9.19808597	9.74935199	9.59458564	9.40710288
6571	**ENSG0000013279**	LPIN3	protein_coding	chr20	41340821	41360582	+	Human GRCh	OTTHUMG000000	LPIN3	**lipin 3**	Nucleus	phosphatase		1.015270349	0	0.95099195	0	1	0	9.29722726	9.71987106	9.56905163	9.51801687
6572	**ENSG0000013280**	ZSWIM3	protein_coding	chr20	45857614	45879122	+	Human GRCh	OTTHUMG000000	ZSWIM3	**zinc finger SWIM-type containing 3**	Other	other		1.204481058	0	0.51686381	0	1	0	6.59233119	6.59350589	6.84873946	6.71513706
6573	**ENSG0000013281**	RBM38	protein_coding	chr20	57391396	57409333	+	Human GRCh	OTTHUMG000000	RBM38	**RNA binding motif protein 38**	Nucleus	other		-1.091361156	0	0.69522194	0	1	0	9.52052805	9.78034192	9.63937461	9.47983841
6574	**ENSG0000013282**	VSTM2L	protein_coding	chr20	37903111	37945350	+	Human GRCh	OTTHUMG000000	VSTM2L	**V-set and transmembrane domain containing 2 like**	Extracellular	other		1.590968487	1	0.26694618	0	1	0	6.41489029	5.8377251	6.17686759	6.97085643
6575	**ENSG0000013282**	OSER1	protein_coding	chr20	44195939	44210771	-	Human GRCh	OTTHUMG000000	OSER1	**oxidative stress responsive serine rich 1**	Other	enzyme		-1.129593443	0	0.57103839	0	1	0	8.83647651	9.15695559	9.05424186	8.66284892
6576	**ENSG0000013282**	SERINC3	protein_coding	chr20	44496221	44522085	-	Human GRCh	OTTHUMG000000	SERINC3	**serine incorporator 3**	Cytoplasm	transporter		-1.152928773	0	0.47036245	0	1	0	11.8099224	12.0318572	11.8689196	11.6522965
6577	**ENSG0000013282**	PPP1R3D	protein_coding	chr20	59936663	59940305	-	Human GRCh	OTTHUMG000000	PPP1R3D	**protein phosphatase 1 regulatory subunit 3D**	Cytoplasm	phosphatase		1.051944597	0	0.85623847	0	1	0	7.91231749	8.28476988	8.36137688	7.93430233

6578	**ENSG0000013283**	DMGDH	protein_coding	chr5	78997564	79236038	-	Human GRCh	OTTHUMG000001	DMGDH	**dimethylglycine dehydrogenase**	Cytoplasm	enzyme		-1.363177848	0	0.30320732	0	1	0	7.10085137	7.1608557	6.96498549	6.63169562
6580	**ENSG0000013284**	AP3B1	protein_coding	chr5	78000522	78294762	-	Human GRCh	OTTHUMG000001	AP3B1	**adaptor related protein complex 3 subunit beta 1**	Plasma Mem	transporter		-1.181494636	0	0.43007827	0	1	0	10.7447047	11.2046022	10.9334329	10.6778782
6581	**ENSG0000013284**	ZBED3	protein_coding	chr5	77072072	77087285	-	Human GRCh	OTTHUMG000001	ZBED3	**zinc finger BED-type containing 3**	Cytoplasm	transcription		-1.135315075	0	0.532624	0	1	0	10.6314069	10.7646052	10.4988367	10.6131691
6582	**ENSG0000013284**	PATJ	protein_coding	chr1	61742477	62178675	+	Human GRCh	OTTHUMG000000	PATJ	**PATJ crumbs cell polarity complex component**	Plasma Mem	other		-1.176631869	0	0.45514327	0	1	0	12.158223	12.3588845	12.2829057	11.8454114
6583	**ENSG0000013285**	KANK4	protein_coding	chr1	62236165	62319434	-	Human GRCh	OTTHUMG000000	KANK4	**KN motif and ankyrin repeat domains 4**	Cytoplasm	other		5.244582021	1	0.23448661	0	1	0	1.3236602	1.37053367	1.36287973	1.88908147
6585	**ENSG0000013287**	SYT4	protein_coding	chr18	43267892	43277535	-	Human GRCh	OTTHUMG000001	SYT4	**synaptotagmin 4**	Cytoplasm	transporter		16.2869201	1	0.01617274	1	1	0	2.21113335	2.31360364	3.27658917	2.78152114
6587	**ENSG0000013287**	FBXO44	protein_coding	chr1	11654375	11663327	+	Human GRCh	OTTHUMG000000	FBXO44	**F-box protein 44**	Cytoplasm	enzyme		1.250411088	0	0.55735866	0	1	0	7.09832103	6.59728376	6.94259434	7.25260218
6588	**ENSG0000013288**	CPLANE2	protein_coding	chr1	16231692	16237183	-	Human GRCh	OTTHUMG000000	CPLANE2	**ciliogenesis and planar polarity effector complex subunit 2**	Cytoplasm	enzyme		-1.035245963	0	0.89550364	0	1	0	6.62202799	6.83498883	6.81141213	6.57261688
6589	**ENSG0000013290**	CASP9	protein_coding	chr1	15490832	15526534	-	Human GRCh	OTTHUMG000000	CASP9	**caspase 9**	Cytoplasm	peptidase	caspase-9	-1.096741204	0	0.73718893	0	1	0	8.08011239	8.36206488	8.34968123	7.85988741
6590	**ENSG0000013291**	NMUR2	protein_coding	chr5	152391546	152433368	-	Human GRCh	OTTHUMG000001	NMUR2	**neuromedin U receptor 2**	Plasma Mem	G-protein cou		1	0	1	0	NA	0	1.41828558	1.44718702	1.44240537	1.41997905
6591	**ENSG0000013291**	DCTN4	protein_coding	chr5	150708440	150759095	-	Human GRCh	OTTHUMG000001	DCTN4	**dynactin subunit 4**	Nucleus	other		-1.099314378	0	0.65171444	0	1	0	11.3442785	11.5621573	11.4539633	11.2426185
6592	**ENSG0000013291**	PDE6A	protein_coding	chr5	149857953	149944793	-	Human GRCh	OTTHUMG000001	PDE6A	**phosphodiesterase 6A**	Plasma Mem	enzyme	sildenafil	1	0	1	0	1	0	2.08679771	2.13528319	2.1273157	2.08966112
6593	**ENSG0000013293**	ATP8A2	protein_coding	chr13	25371974	26025851	+	Human GRCh	OTTHUMG000000	ATP8A2	**ATPase phospholipid transporting 8A2**	Plasma Mem	transporter		1.052444298	0	0.92690865	0	1	0	9.56288815	9.48701675	9.90687018	9.13435756
6594	**ENSG0000013293**	MTUS2	protein_coding	chr13	28820339	29505947	+	Human GRCh	OTTHUMG000000	MTUS2	**microtubule associated scaffold protein 2**	Nucleus	other		9.333599422	1	0.00154888	1	0.83	0	3.23367519	4.31529627	5.19506796	6.59358136
6595	**ENSG0000013295**	ZMYM5	protein_coding	chr13	19823482	19863649	-	Human GRCh	OTTHUMG000000	ZMYM5	**zinc finger MYM-type containing 5**	Nucleus	transcription		-1.044249882	0	0.86086548	0	1	0	9.08116493	9.35062721	9.36650593	8.947625
6596	**ENSG0000013295**	USPL1	protein_coding	chr13	30617693	30660770	+	Human GRCh	OTTHUMG000000	USPL1	**ubiquitin specific peptidase like 1**	Cytoplasm	peptidase		1.178193017	0	0.45813667	0	1	0	9.35876658	9.46031179	9.81607522	9.30791667
6597	**ENSG0000013295**	XPO4	protein_coding	chr13	20777329	20903048	-	Human GRCh	OTTHUMG000000	XPO4	**exportin 4**	Nucleus	transporter		-1.056947575	0	0.79730822	0	1	0	10.9717175	11.3368675	11.2414764	10.9535018
6598	**ENSG0000013295**	TPTE2	protein_coding	chr13	19422876	19561625	-	Human GRCh	OTTHUMG000000	TPTE2	**transmembrane phosphoinositide 3-phosphatase and tensin h**	Cytoplasm	phosphatase		-1.660280079	-1	0.58213458	0	NA	0	3.17342325	3.19934392	2.66024893	3.07828199
6599	**ENSG0000013296**	POMP	protein_coding	chr13	28659065	28678959	+	Human GRCh	OTTHUMG000000	POMP	**proteasome maturation protein**	Nucleus	other		-1.086964837	0	0.72366358	0	1	0	10.3196853	10.7241853	10.5603449	10.3158029
6600	**ENSG0000013296**	CDK8	protein_coding	chr13	26254104	26420982	+	Human GRCh	OTTHUMG000000	CDK8	**cyclin dependent kinase 8**	Nucleus	kinase	didehydro-	1.098398318	0	0.6447395	0	1	0	9.50595535	9.76227465	9.89750263	9.56960007
6602	**ENSG0000013297**	WASF3	protein_coding	chr13	26557683	26688948	+	Human GRCh	OTTHUMG000000	WASF3	**WASP family member 3**	Cytoplasm	other		1.020602907	0	0.93953292	0	1	0	9.98854141	10.2422971	10.2764934	9.99862555
6604	**ENSG0000013297**	GPR12	protein_coding	chr13	26755200	26760786	-	Human GRCh	OTTHUMG000000	GPR12	**G protein-coupled receptor 12**	Plasma Mem	G-protein cou		5.606203493	1	0.27252629	0	NA	0	1.27454553	1.29866884	1.78428712	1.27595448
6605	**ENSG0000013301**	CHRM3	protein_coding	chr1	239386565	239915452	+	Human GRCh	OTTHUMG000000	CHRM3	**cholinergic receptor muscarinic 3**	Plasma Mem	G-protein cou	AZD8683, (	-1.236987454	0	0.60423166	0	1	0	8.36739071	8.30816644	7.84896763	8.32654387
6607	**ENSG0000013302**	MYH10	protein_coding	chr17	8474207	8631376	-	Human GRCh	OTTHUMG000001	MYH10	**myosin heavy chain 10**	Cytoplasm	enzyme		-1.07552444	0	0.7154917	0	1	0	11.410433	11.6784998	11.6534466	11.2540532
6608	**ENSG0000013302**	PEMT	protein_coding	chr17	17505563	17591708	-	Human GRCh	OTTHUMG000000	PEMT	**phosphatidylethanolamine N-methyltransferase**	Cytoplasm	enzyme		-1.117977595	0	0.68256305	0	1	0	8.75726477	9.29198632	9.01616062	8.83934828
6609	**ENSG0000013302**	SCO1	protein_coding	chr17	10672474	10698375	-	Human GRCh	OTTHUMG000001	SCO1	**synthesis of cytochrome C oxidase 1**	Cytoplasm	other		-1.033519056	0	0.87317637	0	1	0	10.5997014	10.9224825	10.9013481	10.5413179
6610	**ENSG0000013303**	MPRIP	protein_coding	chr17	17042457	17217679	+	Human GRCh	OTTHUMG000000	MPRIP	**myosin phosphatase Rho interacting protein**	Cytoplasm	other		-1.04177281	0	0.83726209	0	1	0	12.4370652	12.7316269	12.6864849	12.39138
6612	**ENSG0000013305**	MYBPH	protein_coding	chr1	203167811	203175826	-	Human GRCh	OTTHUMG000000	MYBPH	**myosin binding protein H**	Cytoplasm	other		1	0	1	0	NA	0	-0.0740745	-0.0681512	-0.0691437	-0.0737325
6613	**ENSG0000013305**	PIK3C2B	protein_coding	chr1	204422628	204494805	-	Human GRCh	OTTHUMG000000	PIK3C2B	**phosphatidylinositol-4-phosphate 3-kinase catalytic subunit ty**	Cytoplasm	kinase		1.089479431	0	0.69019858	0	1	0	9.14400112	9.2681431	9.50325101	9.05256292
6614	**ENSG0000013305**	DSTYK	protein_coding	chr1	205142505	205211702	-	Human GRCh	OTTHUMG000000	DSTYK	**dual serine/threonine and tyrosine protein kinase**	Cytoplasm	kinase		-1.006647739	0	0.97425105	0	1	0	10.1521569	10.4106224	10.4502686	10.0834607
6615	**ENSG0000013306**	CHIT1	protein_coding	chr1	203212827	203273641	-	Human GRCh	OTTHUMG000000	CHIT1	**chitinase 1**	Extracellular	enzyme		1	0	1	0	1	0	1.14087119	1.18663957	1.17916604	1.14359388
6616	**ENSG0000013306**	SLC41A1	protein_coding	chr1	205789094	205813748	-	Human GRCh	OTTHUMG000000	SLC41A1	**solute carrier family 41 member 1**	Plasma Mem	transporter		-1.089690966	0	0.716078	0	1	0	10.97895	11.2682054	10.9752862	11.0916937
6617	**ENSG0000013306**	LGR6	protein_coding	chr1	202193799	202319781	+	Human GRCh	OTTHUMG000000	LGR6	**leucine rich repeat containing G protein-coupled receptor 6**	Plasma Mem	G-protein cou		-25.56180804	-1	0.00334388	1	1	0	2.42996866	4.05957391	2.51574729	2.43615416
6618	**ENSG0000013306**	TMCC2	protein_coding	chr1	205227946	205285632	+	Human GRCh	OTTHUMG000000	TMCC2	**transmembrane and coiled-coil domain family 2**	Extracellular	other		1.914592199	1	0.07010946	0	1	0	5.86995517	5.77583073	6.52529052	6.38293707
6619	**ENSG0000013308**	DCLK1	protein_coding	chr13	35768652	36131382	-	Human GRCh	OTTHUMG000000	DCLK1	**doublecortin like kinase 1**	Plasma Mem	kinase		-1.179829942	0	0.66526575	0	1	0	5.66861852	6.14509539	5.15848599	6.15600469
6620	**ENSG0000013310**	CCNA1	protein_coding	chr13	36431520	36442870	+	Human GRCh	OTTHUMG000000	CCNA1	**cyclin A1**	Nucleus	other		1.00366207	0	0.99818659	0	1	0	1.72481423	1.01639369	1.00093037	1.73409708
6621	**ENSG0000013310**	COG6	protein_coding	chr13	39655627	39791665	+	Human GRCh	OTTHUMG000000	COG6	**component of oligomeric golgi complex 6**	Cytoplasm	transporter		1.037854926	0	0.87412434	0	1	0	9.53601	9.79387239	9.79852647	9.62032234
6622	**ENSG0000013310**	SPART	protein_coding	chr13	36301638	36370180	-	Human GRCh	OTTHUMG000000	SPART	**spartin**	Cytoplasm	other		-1.365495958	0	0.43442981	0	1	0	6.80855128	7.08012979	6.81295807	6.40401601
6625	**ENSG0000013310**	TRPC4	protein_coding	chr13	37632063	37869802	-	Human GRCh	OTTHUMG000000	TRPC4	**transient receptor potential cation channel subfamily C memb**	Plasma Mem	ion channel		-27.1043419	-1	0.01041341	1	1	0	2.97848569	2.86983002	2.03002406	1.99685533
6626	**ENSG0000013311**	POSTN	protein_coding	chr13	37562583	37598844	-	Human GRCh	OTTHUMG000000	POSTN	**periostin**	Extracellular	other		-2.578619352	-1	0.57585819	0	NA	0	2.20292593	3.04076214	2.33182668	1.4491723
6627	**ENSG0000013311**	RFXAP	protein_coding	chr13	36819222	36829104	+	Human GRCh	OTTHUMG000000	RFXAP	**regulatory factor X associated protein**	Nucleus	transcription		-1.022200171	0	0.93462247	0	1	0	7.89292955	8.18935106	8.16072595	7.87501731
6628	**ENSG0000013311**	TPT1	protein_coding	chr13	45333471	45341284	-	Human GRCh	OTTHUMG000000	TPT1	**tumor protein, translationally-controlled 1**	Cytoplasm	other		-1.142447314	0	0.5036607	0	1	0	14.1954711	14.4165924	14.2613781	14.0454834
6629	**ENSG0000013311**	GPALPP1	protein_coding	chr13	44989529	45037669	+	Human GRCh	OTTHUMG000000	GPALPP1	**GPALPP motifs containing 1**	Other	other		-1.154396938	0	0.51881592	0	1	0	10.1173011	10.418261	10.2970339	9.90777582
6631	**ENSG0000013311**	KL	protein_coding	chr13	33016423	33066143	+	Human GRCh	OTTHUMG000000	KL	**klotho**	Extracellular	enzyme		-1.483228242	0	0.68248411	0	1	0	2.67451376	1.5302905	1.5214325	2.41430382
6632	**ENSG0000013311**	RFC3	protein_coding	chr13	33818069	33966558	+	Human GRCh	OTTHUMG000000	RFC3	**replication factor C subunit 3**	Nucleus	enzyme		-1.031022039	0	0.89516055	0	1	0	11.1195571	11.5125555	11.5503384	10.9761442
6633	**ENSG0000013312**	STARD13	protein_coding	chr13	33103137	33350630	-	Human GRCh	OTTHUMG000000	STARD13	**StAR related lipid transfer domain containing 13**	Cytoplasm	other		-1.008784031	0	0.97310306	0	1	0	7.91546877	8.22302506	8.08152389	8.05682723
6634	**ENSG0000013312**	IRS4	protein_coding	chrX	108719946	108736563	-	Human GRCh	OTTHUMG000000	IRS4	**insulin receptor substrate 4**	Plasma Mem	other		-2.593790851	-1	0.20497679	0	1	0	4.37004078	4.57017702	3.61619306	3.6294332
6635	**ENSG0000013313**	MORC4	protein_coding	chrX	106813871	107000212	-	Human GRCh	OTTHUMG000000	MORC4	**MORC family CW-type zinc finger 4**	Nucleus	other		-1.014403013	0	0.95008404	0	1	0	9.31059496	9.59086607	9.3758828	9.5068927
6636	**ENSG0000013313**	BEX2	protein_coding	chrX	103309346	103311007	-	Human GRCh	OTTHUMG000000	BEX2	**brain expressed X-linked 2**	Nucleus	other		1.061991673	0	0.79686577	0	1	0	8.64046984	8.90096096	8.87369645	8.81269835
6637	**ENSG0000013313**	RNF128	protein_coding	chrX	106693794	106797016	+	Human GRCh	OTTHUMG000000	RNF128	**ring finger protein 128**	Cytoplasm	enzyme		1.204124249	0	0.7176968	0	1	0	4.37386605	4.65361301	4.52886582	4.82693228
6639	**ENSG0000013313**	TBC1D8B	protein_coding	chrX	106802673	106876150	+	Human GRCh	OTTHUMG000000	TBC1D8B	**TBC1 domain family member 8B**	Cytoplasm	other		1.028892782	0	0.92042712	0	1	0	8.05512063	8.11197017	8.17743511	8.05059082
6640	**ENSG0000013314**	TCEAL4	protein_coding	chrX	103576231	103587729	+	Human GRCh	OTTHUMG000000	TCEAL4	**transcription elongation factor A like 4**	Other	other		1.191926624	0	0.46592925	0	1	0	9.15482097	9.54631522	9.62189848	9.49716426
6641	**ENSG0000013316**	BEX1	protein_coding	chrX	103062651	103064171	-	Human GRCh	OTTHUMG000000	BEX1	**brain expressed X-linked 1**	Other	other		-4.662993399	-1	0.00034592	1	0.36	0	4.79063465	5.10455671	3.39573751	3.46382299
6642	**ENSG0000013319**	FAM104A	protein_coding	chr17	73207353	73236753	-	Human GRCh	OTTHUMG000001	VCF1	**VCP nuclear cofactor family member 1**	Other	other		-1.175369022	0	0.46055909	0	1	0	10.0054127	10.4203131	10.2099946	9.87017403
6643	**ENSG0000013319**	SLC39A11	protein_coding	chr17	72645949	73092712	-	Human GRCh	OTTHUMG000001	SLC39A11	**solute carrier family 39 member 11**	Other	transporter		-1.075550852	0	0.73344096	0	1	0	8.72151205	8.87880893	8.84814606	8.58684288
6644	**ENSG0000013321**	EPHB2	protein_coding	chr1	22710839	22921500	+	Human GRCh	OTTHUMG000000	EPHB2	**EPH receptor B2**	Plasma Mem	kinase	ALW-II-49-	-1.00130102	0	0.99616678	0	1	0	7.47593112	7.48949983	7.44073154	7.51996003
6645	**ENSG0000013322**	SRRM1	protein_coding	chr1	24631716	24673281	+	Human GRCh	OTTHUMG000000	SRRM1	**serine and arginine repetitive matrix 1**	Nucleus	other		-1.016006408	0	0.93170991	0	1	0	12.2837907	12.6435202	12.499226	12.4190321
6646	**ENSG0000013324**	BTBD2	protein_coding	chr19	1985438	2034881	-	Human GRCh	OTTHUMG000001	BTBD2	**BTB domain containing 2**	Cytoplasm	other		-1.06181819	0	0.76717256	0	1	0	11.2373286	11.4185853	11.2727768	11.2572181
6647	**ENSG0000013324**	PRAM1	protein_coding	chr19	8490056	8502640	-	Human GRCh	OTTHUMG000001	PRAM1	**PML-RARA regulated adaptor molecule 1**	Other	other		-1.269868106	0	0.68030303	0	1	0	5.24093406	5.84312346	5.43234357	5.25340173
6648	**ENSG0000013324**	KMT5C	protein_coding	chr19	55339853	55348121	+	Human GRCh	OTTHUMG000001	KMT5C	**lysine methyltransferase 5C**	Nucleus	enzyme		1.103932011	0	0.63996993	0	1	0	9.90442051	10.0953346	10.1454605	10.0782012
6649	**ENSG0000013325**	ZNF414	protein_coding	chr19	8509678	8514167	-	Human GRCh	OTTHUMG000001	ZNF414	**zinc finger protein 414**	Nucleus	transcription		1.006710377	0	0.97653056	0	1	0	8.94492358	9.32291049	9.1026249	9.20644376
6650	**ENSG0000013325**	PDE6B	protein_coding	chr4	625573	670782	+	Human GRCh	OTTHUMG000001	PDE6B	**phosphodiesterase 6B**	Cytoplasm	enzyme		1.100766547	0	0.75419973	0	1	0	9.18152421	9.33040636	9.47857448	9.24641168
6651	**ENSG0000013326**	HSPBP1	protein_coding	chr19	55262223	55280381	-	Human GRCh	OTTHUMG000001	HSPBP1	**HSPA (Hsp70) binding protein 1**	Cytoplasm	other		1.045445548	0	0.80727558	0	1	0	10.4123228	10.5754453	10.5744572	10.518225
6652	**ENSG0000013327**	CSNK1G2	protein_coding	chr19	1941172	1981338	+	Human GRCh	OTTHUMG000001	CSNK1G2	**casein kinase 1 gamma 2**	Cytoplasm	kinase		-1.003636972	0	0.98596851	0	1	0	11.5673986	11.8413097	11.7867124	11.624004
6653	**ENSG0000013330**	SLF1	protein_coding	chr5	94618669	94739436	+	Human GRCh	OTTHUMG000001	SLF1	**SMC5-SMC6 complex localization factor 1**	Nucleus	transcription		1.009830382	0	0.96590741	0	1	0	10.1131494	10.3289086	10.5444849	9.82103355
6654	**ENSG0000013331**	CNDP2	protein_coding	chr18	74495816	74523454	+	Human GRCh	OTTHUMG000001	CNDP2	**carnosine dipeptidase 2**	Cytoplasm	peptidase		-1.04317187	0	0.86300052	0	1	0	12.1165504	12.4490102	12.2958249	12.1929792
6655	**ENSG0000013331**	MACROD1	protein_coding	chr11	63998558	64166113	-	Human GRCh	OTTHUMG000001	MACROD1	**mono-ADP ribosylhydrolase 1**	Cytoplasm	enzyme		1.229874279	0	0.40681191	0	1	0	10.1182965	10.2688002	10.5831979	10.2455601
6656	**ENSG0000013331**	WDR74	protein_coding	chr11	62832342	62841809	-	Human GRCh	OTTHUMG000001	WDR74	**WD repeat domain 74**	Nucleus	other		1.145319443	0	0.64586186	0	1	0	11.2008563	11.5820621	11.7644952	11.2994557
6658	**ENSG0000013331**	RTN3	protein_coding	chr11	63681446	63759891	+	Human GRCh	OTTHUMG000001	RTN3	**reticulon 3**	Cytoplasm	other		-1.038193626	0	0.86654727	0	1	0	11.8938289	12.2114832	12.1559916	11.8722115
6659	**ENSG0000013332**	PLAAT4	protein_coding	chr11	63536808	63546462	+	Human GRCh	OTTHUMG000001	PLAAT4	**phospholipase A and acyltransferase 4**	Cytoplasm	enzyme		1	0	1	0	1	0	1.42440667	1.50677748	1.49354047	1.42939865
6661	**ENSG0000013339**	MYH11	protein_coding	chr16	15703135	15857028	-	Human GRCh	OTTHUMG000001	MYH11	**myosin heavy chain 11**	Cytoplasm	other		1.184512896	0	0.8841105	0	1	0	2.88912592	1.01907367	2.65994717	2.22363178
6662	**ENSG0000013339**	CEP20	protein_coding	chr16	15865719	15888625	-	Human GRCh	OTTHUMG000001	CEP20	**centrosomal protein 20**	Cytoplasm	other		1.056860019	0	0.82671119	0	1	0	10.7249303	11.0058941	11.0477184	10.8134531
6663	**ENSG0000013339**	MED10	protein_coding	chr5	6371874	6378571	-	Human GRCh	OTTHUMG000001	MED10	**mediator complex subunit 10**	Nucleus	transcription		1.035084818	0	0.88437265	0	1	0	8.21846901	8.29242155	8.43805849	8.12833701
6664	**ENSG0000013340**	PDZD2	protein_coding	chr5	31639131	32110932	+	Human GRCh	OTTHUMG000001	PDZD2	**PDZ domain containing 2**	Plasma Mem	other		1.439969049	0	0.55323508	0	1	0	5.72405438	6.50548108	6.33368018	6.68325944
6665	**ENSG0000013342**	MORC2	protein_coding	chr22	30925130	30968774	-	Human GRCh	OTTHUMG000001	MORC2	**MORC family CW-type zinc finger 2**	Nucleus	enzyme		-1.000446504	0	0.99828623	0	1	0	11.6305935	11.8512433	11.926585	11.5317208
6666	**ENSG0000013342**	LARGE1	protein_coding	chr22	33162226	33922841	-	Human GRCh	OTTHUMG000001	LARGE1	**LARGE xylosyl- and glucuronyltransferase 1**	Cytoplasm	enzyme		-2.140506556	-1	0.03584518	1	1	0	7.81638634	8.24521031	7.60845694	6.93955618
6667	**ENSG0000013343**	GSTT2B	protein_coding	chr22	23957414	23961195	-	Human GRCh	OTTHUMG000001	GSTT2/GSTT2	**glutathione S-transferase theta 2B**	Cytoplasm	enzyme		1	0	1	0	NA	0	1.47943993	1.49913263	1.49584768	1.48058296
6668	**ENSG0000013345**	MYO18B	protein_coding	chr22	25742144	26031045	+	Human GRCh	OTTHUMG000001	MYO18B	**myosin XVIIIB**	Cytoplasm	other		1	0	1	0	1	0	1.96873929	2.02638232	2.01697527	1.97217118
6669	**ENSG0000013346**	SLC2A11	protein_coding	chr22	23856703	23886312	+	Human GRCh	OTTHUMG000001	SLC2A11	**solute carrier family 2 member 11**	Plasma Mem	transporter		1.161552366	0	0.55850129	0	1	0	9.02571839	9.21660188	9.48688325	9.0525467
6670	**ENSG0000013346**	C1QTNF6	protein_coding	chr22	37180166	37199385	-	Human GRCh	OTTHUMG000001	C1QTNF6	**C1q and TNF related 6**	Extracellular	other		-1.185615814	0	0.52957364	0	1	0	7.58721949	7.46685588	7.57463872	7.07909068
6671	**ENSG0000013347**	FAM83F	protein_coding	chr22	39994954	40043534	+	Human GRCh	OTTHUMG000001	FAM83F	**family with sequence similarity 83 member F**	Other	other		-1.031035334	0	0.88569897	0	1	0	12.0014014	12.4033306	12.3190408	12.0351457
6675	**ENSG0000013359**	ADCK2	protein_coding	chr7	140672945	140696261	+	Human GRCh	OTTHUMG000001	ADCK2	**aarF domain containing kinase 2**	Cytoplasm	kinase		1.01060369	0	0.96490758	0	1	0	8.47875065	8.79221609	8.78942335	8.50841899
6676	**ENSG0000013360**	MKRN1	protein_coding	chr7	140453033	140479536	-	Human GRCh	OTTHUMG000001	MKRN1	**makorin ring finger protein 1**	Cytoplasm	enzyme		-1.138300036	0	0.53816723	0	1	0	10.6579412	10.9998273	10.8527696	10.5187173

6677	**ENSG0000013361**	AGAP3	protein_coding	chr7	151085831	151144436	+	Human GRCh	OTTHUMG000001	AGAP3	**ArfGAP with GTPase domain, ankyrin repeat and PH domain 3**	Nucleus	transcription		1.043095809	0	0.83554443	0	1	0	11.4120585	11.7493705	11.7944395	11.4625601
6678	**ENSG0000013361**	KRBA1	protein_coding	chr7	149714781	149734575	+	Human GRCh	OTTHUMG000001	KRBA1	**KRAB-A domain containing 1**	Other	other		1.022446999	0	0.92443806	0	1	0	9.46583257	9.77039363	9.81276733	9.46700992
6679	**ENSG0000013362**	ACTR3B	protein_coding	chr7	152759749	152855378	+	Human GRCh	OTTHUMG000001	ACTR3B	**actin related protein 3B**	Cytoplasm	other		-1.016736289	0	0.9388149	0	1	0	9.24522011	9.55721494	9.54058174	9.22392717
6680	**ENSG0000013363**	NTS	protein_coding	chr12	85874295	85882992	+	Human GRCh	OTTHUMG000001	NTS	**neurotensin**	Extracellular	other		1	0	1	0	1	0	0.84501811	0.88792833	0.88094035	0.84757852
6681	**ENSG0000013363**	BTG1	protein_coding	chr12	92140278	92145846	-	Human GRCh	OTTHUMG000001	BTG1	**BTG anti-proliferation factor 1**	Nucleus	transcription		1.017806152	0	0.95327149	0	1	0	10.4485895	10.7116628	10.4278524	10.760997
6682	**ENSG0000013364**	LRRIQ1	protein_coding	chr12	85036314	85264457	+	Human GRCh	OTTHUMG000001	LRRIQ1	**leucine rich repeats and IQ motif containing 1**	Other	other		-1.041451826	0	0.87819287	0	1	0	8.14728134	8.32550378	8.33839538	8.03583554
6683	**ENSG0000013364**	C12orf29	protein_coding	chr12	88033846	88050160	+	Human GRCh	OTTHUMG000001	RLIG1	**RNA 5'-phosphate and 3'-OH ligase 1**	Cytoplasm	enzyme		1.152727932	0	0.58073385	0	1	0	9.11541662	9.39311464	9.48835486	9.3435409
6684	**ENSG0000013365**	ATP13A3	protein_coding	chr3	194402672	194498364	-	Human GRCh	OTTHUMG000001	ATP13A3	**ATPase 13A3**	Extracellular	transporter		-1.060746687	0	0.78339957	0	1	0	12.5879569	12.9573168	12.903011	12.5095887
6685	**ENSG0000013366**	SFTPD	protein_coding	chr10	79937467	79982614	-	Human GRCh	OTTHUMG000000	SFTPD	**surfactant protein D**	Extracellular	other		-23.36379079	-1	0.00921361	1	1	0	1.85685513	3.31865523	1.90364735	1.86017694
6686	**ENSG0000013366**	DYDC2	protein_coding	chr10	80344745	80368073	+	Human GRCh	OTTHUMG000000	DYDC2	**DPY30 domain containing 2**	Extracellular	other		-10.33180839	-1	0.09667545	0	NA	0	2.30838331	1.72740502	1.72429345	1.70988292
6687	**ENSG0000013367**	TMEM254	protein_coding	chr10	80078646	80092557	+	Human GRCh	OTTHUMG000000	TMEM254	**transmembrane protein 254**	Other	other		-1.19437573	0	0.42368889	0	1	0	9.26801215	9.55655109	9.31625052	9.14071341
6688	**ENSG0000013368**	TMTC1	protein_coding	chr12	29500840	29784759	-	Human GRCh	OTTHUMG000001	TMTC1	**transmembrane O-mannosyltransferase targeting cadherins 1**	Cytoplasm	enzyme		-1.237837294	0	0.67321321	0	1	0	10.8688436	11.094226	10.7895165	10.6969783
6689	**ENSG0000013370**	KRAS	protein_coding	chr12	25205246	25250936	-	Human GRCh	OTTHUMG000001	KRAS	**KRAS proto-oncogene, GTPase**	Cytoplasm	enzyme	LY3537982	-1.047336792	0	0.82548685	0	1	0	11.0425714	11.2895921	11.2870708	10.9291101
6690	**ENSG0000013370**	IPO8	protein_coding	chr12	30628988	30695869	-	Human GRCh	OTTHUMG000001	IPO8	**importin 8**	Nucleus	transporter		-1.072645861	0	0.74357049	0	1	0	11.0287435	11.3465909	11.2950233	10.9146651
6691	**ENSG0000013370**	LARS1	protein_coding	chr5	146110566	146182696	-	Human GRCh	OTTHUMG000001	LARS1	**leucyl-tRNA synthetase 1**	Cytoplasm	enzyme		-1.000169765	0	0.99935286	0	1	0	12.5990883	12.8016169	12.9290805	12.4267473
6692	**ENSG0000013371**	SPINK5	protein_coding	chr5	148025683	148137382	+	Human GRCh	OTTHUMG000001	SPINK5	**serine peptidase inhibitor Kazal type 5**	Extracellular	other		2.78480292	1	0.21105385	0	1	0	4.13943469	4.38819141	4.14229139	5.4278545
6693	**ENSG0000013373**	IMPA1	protein_coding	chr8	81656914	81686331	-	Human GRCh	OTTHUMG000001	IMPA1	**inositol monophosphatase 1**	Cytoplasm	phosphatase		1.049096926	0	0.85049603	0	1	0	7.94857671	8.20017186	8.19582916	8.05603937
6694	**ENSG0000013373**	LRRCC1	protein_coding	chr8	85107215	85146080	+	Human GRCh	OTTHUMG000001	LRRCC1	**leucine rich repeat and coiled-coil centrosomal protein 1**	Nucleus	transporter		-1.008431829	0	0.96973259	0	1	0	8.59894645	8.88111543	8.88422795	8.57501289
6695	**ENSG0000013374**	E2F5	protein_coding	chr8	85177154	85217158	+	Human GRCh	OTTHUMG000001	E2F5	**E2F transcription factor 5**	Nucleus	transcription		-1.079359889	0	0.76841105	0	1	0	8.31135601	8.51734213	8.62350116	7.9604042
6697	**ENSG0000013377**	CCDC59	protein_coding	chr12	82223681	82358805	-	Human GRCh	OTTHUMG000001	CCDC59	**coiled-coil domain containing 59**	Nucleus	other		1.067792145	0	0.75086102	0	1	0	9.24643553	9.19513369	9.45102027	9.11190968
6698	**ENSG0000013378**	SWAP70	protein_coding	chr11	9664077	9752993	+	Human GRCh	OTTHUMG000001	SWAP70	**switching B cell complex subunit SWAP70**	Cytoplasm	other		-1.069669551	0	0.77553856	0	1	0	8.91561906	9.51553767	9.14938887	9.20918107
6699	**ENSG0000013379**	BMAL1	protein_coding	chr11	13276652	13387266	+	Human GRCh	OTTHUMG000001	BMAL1	**basic helix-loop-helix ARNT like 1**	Nucleus	transcription		1.304740579	0	0.50859047	0	1	0	5.87045642	5.06013034	5.32272622	6.0953773
6701	**ENSG0000013380**	AMPD3	protein_coding	chr11	10308313	10507579	+	Human GRCh	OTTHUMG000001	AMPD3	**adenosine monophosphate deaminase 3**	Cytoplasm	enzyme		-1.093361559	0	0.78458893	0	1	0	6.53045415	7.24103295	6.90320288	6.79354653
6702	**ENSG0000013381**	SBF2	protein_coding	chr11	9776776	10304877	-	Human GRCh	OTTHUMG000001	SBF2	**SET binding factor 2**	Cytoplasm	other		-1.03444686	0	0.86747042	0	1	0	11.1282004	11.349932	11.29343	11.1129995
6703	**ENSG0000013381**	MICAL2	protein_coding	chr11	12094008	12359144	+	Human GRCh	OTTHUMG000001	MICAL2	**microtubule associated monooxygenase, calponin and LIM do**	Cytoplasm	enzyme		1.058639858	0	0.79481969	0	1	0	12.1855886	12.4155103	12.3912697	12.3474896
6704	**ENSG0000013381**	RRAS2	protein_coding	chr11	14277922	14364506	-	Human GRCh	OTTHUMG000001	RRAS2	**RAS related 2**	Plasma Mem	enzyme		1.114010378	0	0.61817678	0	1	0	8.83177192	9.14005408	9.26876496	8.931801
6705	**ENSG0000013383**	HSD17B4	protein_coding	chr5	119452465	119637199	+	Human GRCh	OTTHUMG000001	HSD17B4	**hydroxysteroid 17-beta dehydrogenase 4**	Cytoplasm	enzyme		-1.034854089	0	0.87236033	0	1	0	11.4532001	11.748994	11.7393901	11.375922
6706	**ENSG0000013385**	ZFC3H1	protein_coding	chr12	71609599	71667725	-	Human GRCh	OTTHUMG000001	ZFC3H1	**zinc finger C3H1-type containing**	Extracellular	other		-1.049083727	0	0.82205281	0	1	0	11.4874725	11.8628166	11.8329271	11.4019739
6707	**ENSG0000013386**	TEX15	protein_coding	chr8	30831544	30913008	-	Human GRCh	OTTHUMG000001	TEX15	**testis expressed 15, meiosis and synapsis associated**	Extracellular	other		-1.022470769	0	0.94705289	0	1	0	7.09968781	7.21882407	7.25433611	7.00631502
6708	**ENSG0000013387**	SARAF	protein_coding	chr8	30063003	30083208	-	Human GRCh	OTTHUMG000001	SARAF	**store-operated calcium entry associated regulatory factor**	Cytoplasm	other		-1.114433388	0	0.59334055	0	1	0	11.6689302	11.921437	11.7874546	11.5598047
6709	**ENSG0000013387**	RNF122	protein_coding	chr8	33547754	33567128	-	Human GRCh	OTTHUMG000001	RNF122	**ring finger protein 122**	Cytoplasm	enzyme		-1.031230127	0	0.91825281	0	1	0	8.14163134	8.38121465	8.41066682	8.02458558
6710	**ENSG0000013387**	DUSP26	protein_coding	chr8	33591330	33600023	-	Human GRCh	OTTHUMG000001	DUSP26	**dual specificity phosphatase 26**	Cytoplasm	enzyme		-1.367708206	0	0.67251886	0	1	0	2.5142327	3.32041905	3.01041298	2.52217326
6711	**ENSG0000013388**	DPF2	protein_coding	chr11	65333843	65354262	+	Human GRCh	OTTHUMG000001	DPF2	**double PHD fingers 2**	Nucleus	transcription		-1.160658414	0	0.44702957	0	1	0	11.2520998	11.4770209	11.2877603	11.1131979
6712	**ENSG0000013389**	MEN1	protein_coding	chr11	64803510	64811294	-	Human GRCh	OTTHUMG000000	MEN1	**menin 1**	Nucleus	transcription	DS-1594b,	1.025284925	0	0.90544995	0	1	0	10.9797508	11.2611608	11.2236698	11.086156
6713	**ENSG0000013393**	ERG28	protein_coding	chr14	75649791	75660876	-	Human GRCh	OTTHUMG000001	ERG28	**ergosterol biosynthesis 28 homolog**	Cytoplasm	other		-1.230264093	0	0.36226502	0	1	0	10.4249829	10.8954989	10.4341144	10.4667155
6714	**ENSG0000013393**	GSC	protein_coding	chr14	94768223	94770113	-	Human GRCh	OTTHUMG000001	GSC	**goosecoid homeobox**	Nucleus	transcription		-1.284248449	0	0.75920323	0	1	0	3.3178386	2.07624536	2.62213687	2.87154169
6715	**ENSG0000013394**	DGLUCY	protein_coding	chr14	91060333	91225632	+	Human GRCh	OTTHUMG000001	DGLUCY	**D-glutamate cyclase**	Cytoplasm	enzyme		2.096788702	1	0.00218326	1	0.96	0	8.70933984	8.74411093	9.61786746	9.34373057
6716	**ENSG0000013395**	UNC79	protein_coding	chr14	93333219	93707876	+	Human GRCh	OTTHUMG000000	UNC79	**unc-79 homolog, NALCN channel complex subunit**	Plasma Mem	other		-3.221194423	-1	0.0277138	1	1	0	3.96049795	4.55536683	3.74038115	3.29742301
6717	**ENSG0000013396**	NUMB	protein_coding	chr14	73275107	73458617	-	Human GRCh	OTTHUMG000001	NUMB	**NUMB endocytic adaptor protein**	Plasma Mem	other		-1.132783437	0	0.51248494	0	1	0	11.1891523	11.4489707	11.2791235	11.0900108
6718	**ENSG0000013396**	CATSPERB	protein_coding	chr14	91580696	91780707	-	Human GRCh	OTTHUMG000001	CATSPERB	**cation channel sperm associated auxiliary subunit beta**	Plasma Mem	other		1.050979694	0	0.87939116	0	1	0	6.34558533	6.86635392	7.0270003	6.19702688
6719	**ENSG0000013398**	VRTN	protein_coding	chr14	74303069	74360008	+	Human GRCh	OTTHUMG000001	VRTN	**vertebrae development associated**	Other	other		-1.120360667	0	0.89382972	0	1	0	3.18456753	2.41775163	2.39699085	3.06704614
6720	**ENSG0000013398**	COX16	protein_coding	chr14	70325081	70416984	-	Human GRCh	OTTHUMG000001	COX16	**cytochrome c oxidase assembly factor COX16**	Cytoplasm	other		1.023670376	0	0.91856217	0	1	0	9.25775338	9.71333077	9.65922259	9.39144124
6721	**ENSG0000013398**	TTC9	protein_coding	chr14	70641916	70675366	+	Human GRCh	OTTHUMG000001	TTC9	**tetratricopeptide repeat domain 9**	Extracellular	other		1.393222259	0	0.33084749	0	1	0	5.40083389	4.87749785	5.77865757	5.08119583
6722	**ENSG0000013399**	MED6	protein_coding	chr14	70581257	70600690	-	Human GRCh	OTTHUMG000001	MED6	**mediator complex subunit 6**	Nucleus	transcription		-1.04439923	0	0.85337309	0	1	0	9.18068651	9.4522005	9.53569189	8.94583931
6723	**ENSG0000013400**	EIF2S1	protein_coding	chr14	67360328	67386516	+	Human GRCh	OTTHUMG000000	EIF2S1	**eukaryotic translation initiation factor 2 subunit alpha**	Cytoplasm	translation re		-1.087536369	0	0.69626615	0	1	0	11.7050555	12.146588	12.0233171	11.6519198
6724	**ENSG0000013400**	ADAM20	protein_coding	chr14	70522358	70535015	-	Human GRCh	OTTHUMG000001	ADAM20	**ADAM metallopeptidase domain 20**	Plasma Mem	peptidase		-1.060721027	0	0.88151544	0	1	0	5.93463773	6.04742234	6.33933262	5.30196187
6725	**ENSG0000013401**	LOXL2	protein_coding	chr8	23296897	23425328	-	Human GRCh	OTTHUMG000001	LOXL2	**lysyl oxidase like 2**	Extracellular	enzyme	PAT-1251,	1.08578126	0	0.77123013	0	1	0	8.15261506	8.02166092	8.28749159	8.05250052
6726	**ENSG0000013401**	ELP3	protein_coding	chr8	28089673	28191156	+	Human GRCh	OTTHUMG000001	ELP3	**elongator acetyltransferase complex subunit 3**	Nucleus	enzyme		-1.220351522	0	0.33777354	0	1	0	9.92217319	10.1012204	9.90601663	9.67208081
6727	**ENSG0000013402**	PEBP4	protein_coding	chr8	22713251	23000000	-	Human GRCh	OTTHUMG000001	PEBP4	**phosphatidylethanolamine binding protein 4**	Cytoplasm	other		-1.14539057	0	0.94910564	0	NA	0	1.11910013	0.44982946	0.83134481	0.78358352
6729	**ENSG0000013403**	CTIF	protein_coding	chr18	48539031	48863217	+	Human GRCh	OTTHUMG000001	CTIF	**cap binding complex dependent translation initiation factor**	Cytoplasm	other		1.134603697	0	0.60514667	0	1	0	8.73091886	8.69840898	8.75725369	8.93366352
6730	**ENSG0000013404**	MRO	protein_coding	chr18	50795120	50825402	-	Human GRCh	OTTHUMG000001	MRO	**maestro**	Nucleus	other		1.648747447	1	0.56703177	0	NA	0	2.78597698	2.90326138	3.20168151	3.09379655
6731	**ENSG0000013404**	MBD2	protein_coding	chr18	54151606	54224669	-	Human GRCh	OTTHUMG000001	MBD2	**methyl-CpG binding domain protein 2**	Nucleus	transcription		-1.090296805	0	0.67442381	0	1	0	10.472922	10.8322572	10.6251565	10.5153795
6732	**ENSG0000013404**	IER3IP1	protein_coding	chr18	47152834	47176364	-	Human GRCh	OTTHUMG000001	IER3IP1	**immediate early response 3 interacting protein 1**	Cytoplasm	other		-1.031668081	0	0.88855027	0	1	0	9.83890189	10.0416443	10.0132102	9.79703773
6733	**ENSG0000013405**	MRPS36	protein_coding	chr5	69217760	69230158	+	Human GRCh	OTTHUMG000001	MRPS36	**mitochondrial ribosomal protein S36**	Cytoplasm	other		1.037026842	0	0.897995	0	1	0	8.2125821	8.81972164	8.62613298	8.5622222
6734	**ENSG0000013405**	CCNB1	protein_coding	chr5	69167135	69178245	+	Human GRCh	OTTHUMG000000	CCNB1	**cyclin B1**	Cytoplasm	other		-1.146046835	0	0.56894469	0	1	0	11.5430709	11.8528878	11.6565431	11.4441998
6735	**ENSG0000013405**	CDK7	protein_coding	chr5	69234795	69277430	+	Human GRCh	OTTHUMG000000	CDK7	**cyclin dependent kinase 7**	Nucleus	kinase	BS181, zot	-1.003995785	0	0.98701311	0	1	0	9.13193149	9.42114652	9.44343582	9.09229486
6737	**ENSG0000013407**	IRAK2	protein_coding	chr3	10164919	10243745	+	Human GRCh	OTTHUMG000001	IRAK2	**interleukin 1 receptor associated kinase 2**	Plasma Mem	kinase		1.351011579	0	0.32465251	0	1	0	6.27246249	6.14600781	6.7796308	6.18490284
6738	**ENSG0000013407**	CAMK1	protein_coding	chr3	9757347	9769992	-	Human GRCh	OTTHUMG000001	CAMK1	**calcium/calmodulin dependent protein kinase I**	Cytoplasm	kinase		1.318803135	0	0.24204633	0	1	0	7.67874492	7.18738767	7.56430324	7.90973553
6739	**ENSG0000013407**	THUMPD3	protein_coding	chr3	9362971	9386791	+	Human GRCh	OTTHUMG000000	THUMPD3	**THUMP domain containing 3**	Cytoplasm	enzyme		1.044589203	0	0.84037882	0	1	0	10.3579021	10.7649695	10.789621	10.4396943
6740	**ENSG0000013408**	VHL	protein_coding	chr3	10141778	10153667	+	Human GRCh	OTTHUMG000001	VHL	**von Hippel-Lindau tumor suppressor**	Nucleus	transcription	DT2216	1.007510058	0	0.96571275	0	1	0	10.9163368	11.1495707	11.1095474	10.9806132
6741	**ENSG0000013410**	BHLHE40	protein_coding	chr3	4979437	4985323	+	Human GRCh	OTTHUMG000001	BHLHE40	**basic helix-loop-helix family member e40**	Nucleus	transcription		-1.005431617	0	0.98727274	0	1	0	10.8033126	11.3166278	11.0687547	11.0930898
6742	**ENSG0000013410**	ARL8B	protein_coding	chr3	5122249	5180911	+	Human GRCh		ARL8B	**ADP ribosylation factor like GTPase 8B**	Plasma Mem	enzyme		-1.062410769	0	0.76892785	0	1	0	10.3297173	10.5874294	10.5322647	10.2476787
6743	**ENSG0000013410**	EDEM1	protein_coding	chr3	5187646	5219958	+	Human GRCh	OTTHUMG000001	EDEM1	**ER degradation enhancing alpha-mannosidase like protein 1**	Cytoplasm	enzyme		-1.174125201	0	0.48952241	0	1	0	11.209119	11.372446	11.2607797	10.9406159
6744	**ENSG0000013411**	CNTN6	protein_coding	chr3	1092658	1404217	+	Human GRCh	OTTHUMG000001	CNTN6	**contactin 6**	Plasma Mem	enzyme		-9.908563039	-1	0.66057598	0	NA	0	0.37887055	-0.1558482	-0.1566534	-0.1603771
6745	**ENSG0000013412**	CHL1	protein_coding	chr3	196763	409417	+	Human GRCh	OTTHUMG000000	CHL1	**cell adhesion molecule L1 like**	Plasma Mem	other		5.554063675	1	0.23954379	0	1	0	2.03328557	2.12530412	2.72126373	2.03885507
6746	**ENSG0000013413**	MEIS2	protein_coding	chr15	36889204	37101299	-	Human GRCh	OTTHUMG000001	MEIS2	**Meis homeobox 2**	Nucleus	transcription		-1.228882532	0	0.5588779	0	1	0	8.21435747	8.35152364	8.32740544	7.73177545
6747	**ENSG0000013414**	DPH6	protein_coding	chr15	35217345	35546193	-	Human GRCh	OTTHUMG000001	DPH6	**diphthamine biosynthesis 6**	Cytoplasm	enzyme		1.010663152	0	0.96456861	0	1	0	9.25632403	9.39413018	9.57821129	9.03907331
6748	**ENSG0000013415**	KATNBL1	protein_coding	chr15	34140674	34210096	-	Human GRCh	OTTHUMG000001	KATNBL1	**katanin regulatory subunit B1 like 1**	Nucleus	other		1.113560474	0	0.62479011	0	1	0	9.37530894	9.4709683	9.83254869	9.16013787
6749	**ENSG0000013415**	EMC7	protein_coding	chr15	34084017	34101862	-	Human GRCh	OTTHUMG000001	EMC7	**ER membrane protein complex subunit 7**	Cytoplasm	other		-1.011199619	0	0.95870991	0	1	0	9.32134341	9.67631619	9.54651917	9.44956367
6753	**ENSG0000013418**	PRPF38B	protein_coding	chr1	108692310	108702928	+	Human GRCh	OTTHUMG000000	PRPF38B	**pre-mRNA processing factor 38B**	Other	other		1.088336534	0	0.68885552	0	1	0	10.7581642	10.9072762	11.1442666	10.6644691
6755	**ENSG0000013419**	TSPAN2	protein_coding	chr1	115048011	115089503	-	Human GRCh	OTTHUMG000000	TSPAN2	**tetraspanin 2**	Extracellular	other		1	0	1	0	1	0	1.16699522	1.21586951	1.20787605	1.16989671
6758	**ENSG0000013420**	GSTM3	protein_coding	chr1	109733932	109741038	-	Human GRCh	OTTHUMG000000	GSTM3	**glutathione S-transferase mu 3**	Cytoplasm	enzyme		1.267639444	0	0.49840379	0	1	0	5.84237292	6.0793623	6.12375922	6.25133452
6759	**ENSG0000013420**	SYT6	protein_coding	chr1	114089291	114153880	-	Human GRCh	OTTHUMG000000	SYT6	**synaptotagmin 6**	Cytoplasm	transporter		5.639755241	1	0.29963055	0	NA	0	1.23169905	1.26262226	1.79934949	1.23351741
6760	**ENSG0000013421**	VAV3	protein_coding	chr1	107571161	107965180	-	Human GRCh	OTTHUMG000000	VAV3	**vav guanine nucleotide exchange factor 3**	Extracellular	cytokine		-1.742919994	-1	0.08877963	0	1	0	8.00875687	8.04370246	7.68467768	7.12166177
6761	**ENSG0000013421**	CHIA	protein_coding	chr1	111290851	111320566	+	Human GRCh	OTTHUMG000000	CHIA	**chitinase acidic**	Extracellular	enzyme		1	0	1	0	1	0	1.40057317	1.43335377	1.42794689	1.40250073
6762	**ENSG0000013422**	PSRC1	protein_coding	chr1	109279556	109283186	-	Human GRCh	OTTHUMG000000	PSRC1	**proline and serine rich coiled-coil 1**	Cytoplasm	other		-1.163551383	0	0.4799172	0	1	0	9.26891719	9.2987105	9.2854526	8.93051031
6765	**ENSG0000013424**	SORT1	protein_coding	chr1	109309568	109397918	-	Human GRCh	OTTHUMG000000	SORT1	**sortilin 1**	Plasma Mem	G-protein co	TH1902	1.027642778	0	0.92083831	0	1	0	11.5225467	11.9117644	11.9295772	11.5697185
6766	**ENSG0000013424**	WNT2B	protein_coding	chr1	112466541	112530165	+	Human GRCh	OTTHUMG000000	WNT2B	**Wnt family member 2B**	Extracellular	other		-2.378442551	-1	0.0940938	0	1	0	4.74928175	5.57516424	4.3450787	4.57537055
6767	**ENSG0000013424**	PTGFRN	protein_coding	chr1	116909916	116990353	+	Human GRCh	OTTHUMG000000	PTGFRN	**prostaglandin F2 receptor inhibitor**	Plasma Mem	other		-1.001179141	0	0.99579047	0	1	0	10.2304245	10.5104466	10.443887	10.3070447
6768	**ENSG0000013424**	LAMTOR5	protein_coding	chr1	110401249	110407942	-	Human GRCh	OTTHUMG000000	LAMTOR5	**late endosomal/lysosomal adaptor, MAPK and MTOR activato**	Cytoplasm	other		-1.050753053	0	0.82595882	0	1	0	9.80444763	10.2011438	9.99659698	9.92863455
6770	**ENSG0000013425**	NOTCH2	protein_coding	chr1	119911553	120100779	-	Human GRCh	OTTHUMG000000	NOTCH2	**notch receptor 2**	Plasma Mem	transcription		1.002026134	0	0.99363043	0	1	0	12.5445137	12.8931434	12.8204622	12.640367
6771	**ENSG0000013425**	TRIM45	protein_coding	chr1	117111060	117122587	-	Human GRCh	OTTHUMG000000	TRIM45	**tripartite motif containing 45**	Cytoplasm	enzyme		1.245169121	0	0.40560214	0	1	0	7.64104571	8.16933594	8.24378072	8.0834427
6772	**ENSG0000013425**	CEPT1	protein_coding	chr1	111139479	111185104	+	Human GRCh	OTTHUMG000000	CEPT1	**choline/ethanolamine phosphotransferase 1**	Cytoplasm	enzyme		1.263609338	0	0.30166972	0	1	0	9.40562171	9.47156874	9.87996982	9.47373909

6773	**ENSG0000013425**	CD101	protein_coding	chr1	117001750	117036552	+	Human GRCh	OTTHUMG000000	CD101	**CD101 molecule**	Plasma Mem	other		-1.298195406	0	0.57989813	0	1	0	5.27510709	5.61773993	5.58623132	4.67876924
6774	**ENSG0000013425**	VTCN1	protein_coding	chr1	117143587	117210960	-	Human GRCh	OTTHUMG000000	VTCN1	**V-set domain containing T cell activation inhibitor 1**	Plasma Mem	other	AZD8205,	1	0	1	0	NA	0	0.96697564	0.99794634	0.99285036	0.96880176
6775	**ENSG0000013425**	NGF	protein_coding	chr1	115285904	115338770	-	Human GRCh	OTTHUMG000000	NGF	**nerve growth factor**	Extracellular	growth facto	tanezumab	1	0	1	0	1	0	1.13037531	1.17829418	1.17048884	1.13323407
6776	**ENSG0000013426**	AP4B1	protein_coding	chr1	113894194	113905201	-	Human GRCh	OTTHUMG000000	AP4B1	**adaptor related protein complex 4 subunit beta 1**	Cytoplasm	other		-1.017795521	0	0.9381261	0	1	0	8.60304188	9.04748282	8.97761627	8.6531841
6777	**ENSG0000013426**	NAPG	protein_coding	chr18	10525905	10552764	+	Human GRCh	OTTHUMG000001	NAPG	**NSF attachment protein gamma**	Cytoplasm	transporter		1.00469313	0	0.98210685	0	1	0	10.0265795	10.0654708	10.1425175	9.95255968
6778	**ENSG0000013427**	SPIRE1	protein_coding	chr18	12446512	12658107	-	Human GRCh	OTTHUMG000001	SPIRE1	**spire type actin nucleation factor 1**	Cytoplasm	other		-1.442431489	0	0.2323698	0	1	0	12.4691929	12.6305955	12.2137571	12.0386114
6779	**ENSG0000013428**	PPHLN1	protein_coding	chr12	42238447	42459715	+	Human GRCh	OTTHUMG000001	PPHLN1	**periphilin 1**	Nucleus	other		-1.112527807	0	0.6390283	0	1	0	11.2041271	11.5289719	11.4233695	11.0667398
6780	**ENSG0000013428**	FKBP11	protein_coding	chr12	48921518	48926474	-	Human GRCh	OTTHUMG000001	FKBP11	**FKBP prolyl isomerase 11**	Cytoplasm	enzyme		-1.216683566	0	0.45056941	0	1	0	9.9283337	10.2819426	10.0070679	9.76970859
6781	**ENSG0000013428**	ARF3	protein_coding	chr12	48935723	48957487	-	Human GRCh	OTTHUMG000001	ARF3	**ADP ribosylation factor 3**	Cytoplasm	enzyme		-1.143604613	0	0.49419044	0	1	0	12.4478434	12.7276804	12.5238889	12.3590069
6782	**ENSG0000013429**	TMEM106	protein_coding	chr12	47963569	47968878	+	Human GRCh	OTTHUMG000001	TMEM106C	**transmembrane protein 106C**	Other	other		-1.200611443	0	0.41308941	0	1	0	11.0877643	11.4552263	11.2250792	10.9168121
6783	**ENSG0000013429**	SLC38A2	protein_coding	chr12	46358188	46372773	-	Human GRCh	OTTHUMG000001	SLC38A2	**solute carrier family 38 member 2**	Plasma Mem	transporter		-1.334299134	0	0.2444199	0	1	0	13.5555113	13.654114	13.3703581	13.1843204
6784	**ENSG0000013430**	YWHAQ	protein_coding	chr2	9583967	9630997	-	Human GRCh	OTTHUMG000000	YWHAQ	**tyrosine 3-monooxygenase/tryptophan 5-monooxygenase acti**	Cytoplasm	other		-1.09466821	0	0.66417366	0	1	0	13.016521	13.3577668	13.2317174	12.9481464
6785	**ENSG0000013431**	KIDINS220	protein_coding	chr2	8721081	8837630	-	Human GRCh	OTTHUMG000001	KIDINS220	**kinase D interacting substrate 220**	Nucleus	transcription		-1.117791485	0	0.58908273	0	1	0	12.1545215	12.3475384	12.2687243	11.9736374
6786	**ENSG0000013431**	GRHL1	protein_coding	chr2	9951693	10002277	+	Human GRCh	OTTHUMG000001	GRHL1	**grainyhead like transcription factor 1**	Nucleus	transcription		1.087512857	0	0.7199744	0	1	0	9.08066597	9.32212695	9.54105909	8.99209302
6787	**ENSG0000013431**	ROCK2	protein_coding	chr2	11179759	11348330	-	Human GRCh	OTTHUMG000001	ROCK2	**Rho associated coiled-coil containing protein kinase 2**	Cytoplasm	kinase	ripasudil, (	-1.00675285	0	0.9709296	0	1	0	11.242457	11.4004348	11.4753894	11.1331669
6788	**ENSG0000013432**	RSAD2	protein_coding	chr2	6865557	6898239	+	Human GRCh	OTTHUMG000000	RSAD2	**radical S-adenosyl methionine domain containing 2**	Cytoplasm	enzyme		-2.125726214	-1	0.1890393	0	1	0	4.18428734	3.8177672	3.09239988	3.76055744
6789	**ENSG0000013432**	MYCN	protein_coding	chr2	15940550	15947007	+	Human GRCh	OTTHUMG000000	MYCN	**MYCN proto-oncogene, bHLH transcription factor**	Nucleus	transcription		-22.80845193	-1	0.01514428	1	1	0	2.3317547	2.99096847	1.92720316	1.89856745
6790	**ENSG0000013432**	LPIN1	protein_coding	chr2	11677595	11827409	+	Human GRCh	OTTHUMG000001	LPIN1	**lipin 1**	Nucleus	phosphatase		-1.08457923	0	0.72290718	0	1	0	11.1306794	11.7532311	11.4256041	11.3581397
6791	**ENSG0000013432**	CMPK2	protein_coding	chr2	6840570	6866635	-	Human GRCh	OTTHUMG000001	CMPK2	**cytidine/uridine monophosphate kinase 2**	Cytoplasm	kinase		1.263283567	0	0.61493474	0	1	0	5.02765483	4.73271646	5.15516849	5.03934094
6792	**ENSG0000013433**	IAH1	protein_coding	chr2	9473658	9496543	+	Human GRCh	OTTHUMG000001	IAH1	**isoamyl acetate hydrolyzing esterase 1 (putative)**	Cytoplasm	other		-1.195202077	0	0.43343431	0	1	0	9.11719458	9.22068682	9.0919781	8.8381533
6793	**ENSG0000013433**	LDHA	protein_coding	chr11	18394560	18408425	+	Human GRCh	OTTHUMG000001	LDHA	**lactate dehydrogenase A**	Cytoplasm	enzyme	nedosiran	-1.077630723	0	0.78038453	0	1	0	14.615435	15.0197989	14.851026	14.6363104
6795	**ENSG0000013434**	ANO3	protein_coding	chr11	26188842	26663289	+	Human GRCh	OTTHUMG000001	ANO3	**anoctamin 3**	Plasma Mem	transporter		1	0	1	0	1	0	0.97192469	1.03448499	1.02439355	0.97569919
6796	**ENSG0000013435**	IL6ST	protein_coding	chr5	55935095	55995022	-	Human GRCh	OTTHUMG000000	IL6ST	**interleukin 6 cytokine family signal transducer**	Plasma Mem	transmembra		1.099972389	0	0.66863946	0	1	0	10.4920356	10.8709762	10.8617531	10.7245731
6797	**ENSG0000013436**	FST	protein_coding	chr5	53480626	53487134	+	Human GRCh	OTTHUMG000001	FST	**follistatin**	Extracellular	other		1.238108973	0	0.67369111	0	1	0	4.64020814	5.03765273	4.51854038	5.41850668
6799	**ENSG0000013436**	NAV1	protein_coding	chr1	201539127	201826969	+	Human GRCh	OTTHUMG000000	NAV1	**neuron navigator 1**	Other	enzyme		1.533470847	1	0.18117338	0	1	0	10.964727	11.376574	11.8062883	11.461899
6800	**ENSG0000013437**	CDC73	protein_coding	chr1	193121983	193254815	+	Human GRCh	OTTHUMG000000	CDC73	**cell division cycle 73**	Nucleus	other		1.006542401	0	0.97509967	0	1	0	11.6948832	12.0288699	12.0680847	11.658316
6801	**ENSG0000013437**	TIMM17A	protein_coding	chr1	201955503	201970664	+	Human GRCh	OTTHUMG000000	TIMM17A	**translocase of inner mitochondrial membrane 17A**	Cytoplasm	transporter		-1.024130458	0	0.90754042	0	1	0	10.7328217	11.1352287	11.0623115	10.7670101
6802	**ENSG0000013437**	CRB1	protein_coding	chr1	197268204	197478455	+	Human GRCh	OTTHUMG000000	CRB1	**crumbs cell polarity complex component 1**	Plasma Mem	other		-1.291202184	0	0.66690333	0	1	0	4.17972131	4.80497098	4.71683249	3.66008301
6804	**ENSG0000013439**	ERN2	protein_coding	chr16	23690310	23713226	-	Human GRCh	OTTHUMG000001	ERN2	**endoplasmic reticulum to nucleus signaling 2**	Cytoplasm	kinase		-5.05908368	-1	0.34286222	0	NA	0	1.76283081	1.23527757	0.83932204	1.17544119
6805	**ENSG0000013441**	RPS15A	protein_coding	chr16	18781295	18790383	-	Human GRCh	OTTHUMG000001	RPS15A	**ribosomal protein S15a**	Cytoplasm	other		1.062757186	0	0.76967724	0	1	0	12.6257634	12.8217529	12.9213052	12.656823
6807	**ENSG0000013444**	NARS1	protein_coding	chr18	57600656	57622213	-	Human GRCh	OTTHUMG000001	NARS1	**asparaginyl-tRNA synthetase 1**	Cytoplasm	enzyme		-1.044332116	0	0.82821429	0	1	0	11.9157812	12.05139	12.1025906	11.7447068
6808	**ENSG0000013444**	GRP	protein_coding	chr18	59220158	59230774	+	Human GRCh	OTTHUMG000001	GRP	**gastrin releasing peptide**	Extracellular	growth facto		1	0	1	0	1	0	1.10520711	1.21382208	1.19657803	1.11188283
6809	**ENSG0000013444**	RELCH	protein_coding	chr18	62187255	62310249	+	Human GRCh	OTTHUMG000001	RELCH	**RAB11 binding and LisH domain, coiled-coil and HEAT repeat c**	Cytoplasm	other		1.099348519	0	0.65911984	0	1	0	10.5848643	10.8750036	11.0148748	10.6406314
6810	**ENSG0000013445**	FBH1	protein_coding	chr10	5890203	5937594	+	Human GRCh	OTTHUMG000001	FBH1	**F-box DNA helicase 1**	Nucleus	enzyme		-1.191905727	0	0.38021103	0	1	0	10.7586099	11.1587265	10.9249265	10.6151279
6811	**ENSG0000013445**	RBM17	protein_coding	chr10	6089034	6117457	+	Human GRCh	OTTHUMG000000	RBM17	**RNA binding motif protein 17**	Nucleus	other		1.002072193	0	0.99192869	0	1	0	10.6730585	10.9389061	11.0007298	10.5960338
6813	**ENSG0000013446**	ANKRD16	protein_coding	chr10	5861616	5889906	-	Human GRCh	OTTHUMG000000	ANKRD16	**ankyrin repeat domain 16**	Other	other		1.084839614	0	0.76140719	0	1	0	8.27681931	8.66773053	8.64138281	8.51049683
6814	**ENSG0000013446**	ECHDC3	protein_coding	chr10	11742366	11764070	+	Human GRCh	OTTHUMG000000	ECHDC3	**enoyl-CoA hydratase domain containing 3**	Cytoplasm	enzyme		1.11554393	0	0.72620569	0	1	0	6.92782401	7.04424658	7.00142302	7.19489018
6815	**ENSG0000013447**	IL15RA	protein_coding	chr10	5943639	5978187	-	Human GRCh	OTTHUMG000000	IL15RA	**interleukin 15 receptor subunit alpha**	Plasma Mem	transmembra		1.069257381	0	0.89609858	0	1	0	5.10593832	5.00608075	4.8565995	5.32782967
6816	**ENSG0000013448**	CCNH	protein_coding	chr5	87318416	87412930	-	Human GRCh	OTTHUMG000001	CCNH	**cyclin H**	Nucleus	transcription		-1.062270426	0	0.76973901	0	1	0	10.4659196	10.6685291	10.6521661	10.33512
6818	**ENSG0000013449**	TMEM241	protein_coding	chr18	23197144	23437961	-	Human GRCh	OTTHUMG000001	TMEM241	**transmembrane protein 241**	Other	other		-1.028548456	0	0.90005947	0	1	0	9.12688478	9.46474539	9.3013062	9.25072263
6819	**ENSG0000013450**	KCTD1	protein_coding	chr18	26454910	26657401	-	Human GRCh	OTTHUMG000001	KCTD1	**potassium channel tetramerization domain containing 1**	Nucleus	transcription		-1.019031998	0	0.9380291	0	1	0	10.3513858	10.5066482	10.4966163	10.3174959
6820	**ENSG0000013450**	CABLES1	protein_coding	chr18	23134564	23260470	+	Human GRCh	OTTHUMG000001	CABLES1	**Cdk5 and Abl enzyme substrate 1**	Nucleus	other		-1.186552805	0	0.50336161	0	1	0	9.03403007	9.40986396	9.11506155	8.99326833
6822	**ENSG0000013453**	EMP1	protein_coding	chr12	13196723	13219941	+	Human GRCh	OTTHUMG000001	EMP1	**epithelial membrane protein 1**	Plasma Mem	other		-1.063176014	0	0.88410898	0	1	0	7.65948431	7.69700522	7.56194436	7.6497568
6823	**ENSG0000013453**	SOX5	protein_coding	chr12	23529504	24562544	-	Human GRCh	OTTHUMG000001	SOX5	**SRY-box transcription factor 5**	Nucleus	transcription		-1.334047325	0	0.36574946	0	1	0	9.63203388	9.89756305	9.56587326	9.31034198
6824	**ENSG0000013453**	RERG	protein_coding	chr12	15107783	15348675	-	Human GRCh	OTTHUMG000001	RERG	**RAS like estrogen regulated growth inhibitor**	Nucleus	enzyme		2.812249328	1	0.06925631	0	1	0	4.24236643	4.6433017	5.69960736	5.11282558
6828	**ENSG0000013454**	SPX	protein_coding	chr12	21526296	21541249	+	Human GRCh	OTTHUMG000001	SPX	**spexin hormone**	Nucleus	other		2.366666477	1	0.40112449	0	1	0	1.92610809	2.6124177	3.6325595	1.93689055
6830	**ENSG0000013456**	LRP4	protein_coding	chr11	46856717	46918642	-	Human GRCh	OTTHUMG000001	LRP4	**LDL receptor related protein 4**	Extracellular	other		-1.4150748	0	0.26797045	0	1	0	9.79073182	10.8471728	10.3627918	9.62912473
6832	**ENSG0000013457**	DDB2	protein_coding	chr11	47214465	47239217	+	Human GRCh	OTTHUMG000001	DDB2	**damage specific DNA binding protein 2**	Nucleus	transcription		-1.108832823	0	0.60336867	0	1	0	9.34372131	9.43654634	9.33746372	9.22119134
6833	**ENSG0000013457**	ACP2	protein_coding	chr11	47239302	47248906	-	Human GRCh	OTTHUMG000001	ACP2	**acid phosphatase 2, lysosomal**	Cytoplasm	phosphatase		-1.11575052	0	0.62581637	0	1	0	9.42103924	9.75955679	9.54020333	9.42112533
6835	**ENSG0000013459**	RTL8C	protein_coding	chrX	135032355	135033546	+	Human GRCh	OTTHUMG000000	RTL8C	**retrotransposon Gag like 8C**	Plasma Mem	other		1.188272659	0	0.4335158	0	1	0	9.23050907	9.62875048	9.6612734	9.61167629
6837	**ENSG0000013459**	SOX3	protein_coding	chrX	140502985	140505069	-	Human GRCh	OTTHUMG000000	SOX3	**SRY-box transcription factor 3**	Nucleus	transcription		11.66681132	1	0.6377824	0	NA	0	-0.5358116	-0.531423	0.07399907	-0.5355567
6838	**ENSG0000013459**	RBMX2	protein_coding	chrX	130401987	130413656	+	Human GRCh	OTTHUMG000000	RBMX2	**RNA binding motif protein X-linked 2**	Nucleus	other		-1.139488087	0	0.59057081	0	1	0	8.84361489	8.96996683	8.98446285	8.4989432
6839	**ENSG0000013460**	STK26	protein_coding	chrX	132023302	132075943	+	Human GRCh	OTTHUMG000000	STK26	**serine/threonine kinase 26**	Nucleus	kinase		1.103766989	0	0.6453356	0	1	0	9.60358711	9.85805756	9.9524702	9.73024279
6840	**ENSG0000013462**	PIWIL4	protein_coding	chr11	94543840	94621421	+	Human GRCh	OTTHUMG000001	PIWIL4	**piwi like RNA-mediated gene silencing 4**	Cytoplasm	other		1.294985532	0	0.75750288	0	1	0	3.01175434	3.42552383	3.40518506	3.30714119
6842	**ENSG0000013464**	PUM1	protein_coding	chr1	30931506	31065991	-	Human GRCh	OTTHUMG000000	PUM1	**pumilio RNA binding family member 1**	Cytoplasm	other		-1.103540264	0	0.61599912	0	1	0	12.2853013	12.520973	12.4335402	12.1493802
6844	**ENSG0000013468**	YARS1	protein_coding	chr1	32775237	32818031	-	Human GRCh	OTTHUMG000000	YARS1	**tyrosyl-tRNA synthetase 1**	Cytoplasm	enzyme		-1.125058762	0	0.53642798	0	1	0	11.912178	12.0464365	12.0135621	11.6614932
6845	**ENSG0000013468**	PHC2	protein_coding	chr1	33323623	33431095	-	Human GRCh	OTTHUMG000000	PHC2	**polyhomeotic homolog 2**	Nucleus	other		-1.120590816	0	0.55981191	0	1	0	10.3936404	10.7892275	10.5470112	10.4207503
6846	**ENSG0000013469**	CDCA8	protein_coding	chr1	37692481	37709719	+	Human GRCh	OTTHUMG000000	CDCA8	**cell division cycle associated 8**	Nucleus	other		-1.174953534	0	0.45459052	0	1	0	10.6971782	11.1068663	10.8480133	10.6290981
6847	**ENSG0000013469**	GNL2	protein_coding	chr1	37566816	37595937	-	Human GRCh	OTTHUMG000000	GNL2	**G protein nucleolar 2**	Nucleus	enzyme		-1.046808402	0	0.81156515	0	1	0	10.6899518	11.0628957	10.9613329	10.7054388
6848	**ENSG0000013469**	AGO4	protein_coding	chr1	35808016	35857890	+	Human GRCh	OTTHUMG000000	AGO4	**argonaute RISC component 4**	Cytoplasm	translation re		-1.21756366	0	0.32520327	0	1	0	10.4170537	10.7581611	10.4638675	10.2964716
6849	**ENSG0000013470**	HOOK1	protein_coding	chr1	59814786	59876322	+	Human GRCh	OTTHUMG000000	HOOK1	**hook microtubule tethering protein 1**	Cytoplasm	other		1.051328716	0	0.80671292	0	1	0	10.9258165	11.2175083	11.342306	10.8834032
6850	**ENSG0000013471**	CYP2J2	protein_coding	chr1	59893308	59926773	-	Human GRCh	OTTHUMG000000	CYP2J2	**cytochrome P450 family 2 subfamily J member 2**	Cytoplasm	enzyme		-1.006205849	0	0.9939631	0	1	0	2.74705669	2.5376865	2.52167866	2.75456698
6851	**ENSG0000013471**	BTF3L4	protein_coding	chr1	52056199	52090716	+	Human GRCh	OTTHUMG000000	BTF3L4	**basic transcription factor 3 like 4**	Other	other		-1.023368084	0	0.91703586	0	1	0	11.1525785	11.4603363	11.4986946	11.0384439
6852	**ENSG0000013474**	TUT4	protein_coding	chr1	52408282	52553487	-	Human GRCh	OTTHUMG000000	TUT4	**terminal uridylyl transferase 4**	Nucleus	enzyme		-1.188322317	0	0.41130789	0	1	0	11.6889106	11.9566612	11.7718262	11.4949663
6853	**ENSG0000013474**	PRPF38A	protein_coding	chr1	52404602	52420836	+	Human GRCh	OTTHUMG000000	PRPF38A	**pre-mRNA processing factor 38A**	Nucleus	other		1.019275231	0	0.92379683	0	1	0	10.6073254	10.9396184	10.9631439	10.6246655
6854	**ENSG0000013475**	DSC2	protein_coding	chr18	31058840	31102522	-	Human GRCh	OTTHUMG000001	DSC2	**desmocollin 2**	Plasma Mem	other		1.81163014	1	0.01567462	1	1	0	9.10305589	9.09716132	9.63977528	9.74637119
6856	**ENSG0000013475**	RNF138	protein_coding	chr18	32091874	32131561	+	Human GRCh	OTTHUMG000001	RNF138	**ring finger protein 138**	Nucleus	enzyme		1.225182861	0	0.36954201	0	1	0	9.99656596	10.2586721	10.6047432	10.040197
6857	**ENSG0000013475**	ELP2	protein_coding	chr18	36129444	36180557	+	Human GRCh	OTTHUMG000001	ELP2	**elongator acetyltransferase complex subunit 2**	Cytoplasm	other		1.027166389	0	0.92520072	0	1	0	11.4114483	11.6790142	11.7922541	11.3253402
6858	**ENSG0000013476**	DSG1	protein_coding	chr18	31318160	31359246	+	Human GRCh	OTTHUMG000001	DSG1	**desmoglein 1**	Plasma Mem	other		1	0	1	0	1	0	0.92756096	0.97956614	0.97113357	0.93067971
6859	**ENSG0000013476**	DSC3	protein_coding	chr18	30989365	31042815	-	Human GRCh	OTTHUMG000001	DSC3	**desmocollin 3**	Plasma Mem	other		1.622886671	1	0.53804031	0	NA	0	2.96283039	2.82180001	3.21186232	3.16721702
6860	**ENSG0000013476**	DSC1	protein_coding	chr18	31129236	31162856	-	Human GRCh	OTTHUMG000001	DSC1	**desmocollin 1**	Plasma Mem	other		1.014885459	0	0.98034568	0	1	0	5.15538349	4.77890029	4.96283258	5.0339508
6861	**ENSG0000013476**	DTNA	protein_coding	chr18	34493291	34891844	+	Human GRCh	OTTHUMG000001	DTNA	**dystrobrevin alpha**	Plasma Mem	other		1.171183415	0	0.77502632	0	1	0	7.71930029	8.07841395	7.43913979	8.47535644
6862	**ENSG0000013477**	FHOD3	protein_coding	chr18	36297713	36780220	+	Human GRCh	OTTHUMG000001	FHOD3	**formin homology 2 domain containing 3**	Nucleus	other		1.404826787	0	0.69444983	0	1	0	4.243514	4.81552072	4.57901122	4.71080493
6863	**ENSG0000013477**	TPGS2	protein_coding	chr18	36777647	36829216	-	Human GRCh	OTTHUMG000001	TPGS2	**tubulin polyglutamylase complex subunit 2**	Nucleus	other		-1.194119346	0	0.40391869	0	1	0	11.8861569	12.2030358	11.9657566	11.7362445
6864	**ENSG0000013478**	DAGLA	protein_coding	chr11	61680391	61747001	+	Human GRCh	OTTHUMG000001	DAGLA	**diacylglycerol lipase alpha**	Plasma Mem	enzyme		1.166574512	0	0.42487276	0	1	0	8.77348745	8.99922175	9.08641049	9.02835729
6865	**ENSG0000013480**	SLC43A3	protein_coding	chr11	57406954	57427580	-	Human GRCh	OTTHUMG000001	SLC43A3	**solute carrier family 43 member 3**	Extracellular	transporter		1.640181734	1	0.11098534	0	1	0	7.09987965	7.48645322	7.66598221	7.87931918
6866	**ENSG0000013480**	TIMM10	protein_coding	chr11	57528464	57530803	-	Human GRCh	OTTHUMG000001	TIMM10	**translocase of inner mitochondrial membrane 10**	Cytoplasm	transporter		-1.199920106	0	0.4904667	0	1	0	8.7084805	9.19941372	8.85866612	8.69340529
6868	**ENSG0000013481**	DHX34	protein_coding	chr19	47349315	47382704	+	Human GRCh	OTTHUMG000001	DHX34	**DExH-box helicase 34**	Other	enzyme		1.029690634	0	0.88930197	0	1	0	11.1389088	11.4604106	11.442431	11.2356678
6870	**ENSG0000013482**	FADS2	protein_coding	chr11	61792980	61867354	+	Human GRCh	OTTHUMG000001	FADS2	**fatty acid desaturase 2**	Plasma Mem	enzyme		-1.204777509	0	0.46869374	0	1	0	10.1174283	10.3781423	10.1845643	9.91865545
6871	**ENSG0000013482**	TMEM258	protein_coding	chr11	61768501	61792802	-	Human GRCh	OTTHUMG000001	TMEM258	**transmembrane protein 258**	Cytoplasm	other		1.002075021	0	0.99265986	0	1	0	10.0865931	10.3855027	10.4136277	10.0546872
6874	**ENSG0000013485**	TMEM165	protein_coding	chr4	55395957	55453397	+	Human GRCh	OTTHUMG000001	TMEM165	**transmembrane protein 165**	Plasma Mem	other		-1.012068138	0	0.95010025	0	1	0	10.8969284	11.0767595	11.1250239	10.8076901
6875	**ENSG0000013485**	CLOCK	protein_coding	chr4	55427903	55546909	-	Human GRCh	OTTHUMG000001	CLOCK	**clock circadian regulator**	Nucleus	transcription		-1.040841363	0	0.85001906	0	1	0	10.2416251	10.3945046	10.3966688	10.1437073
6876	**ENSG0000013485**	PDGFRA	protein_coding	chr4	54229280	54298245	+	Human GRCh	OTTHUMG000001	PDGFRA	**platelet derived growth factor receptor alpha**	Plasma Mem	kinase	crizotinib/(	-1.254100145	0	0.75323195	0	1	0	5.58606887	6.13705643	5.48459725	5.78623565

6877	**ENSG0000013486**	GGACT	protein_coding	chr13	100530164	100589528	-	Human GRCh	OTTHUMG000000	GGACT	**gamma-glutamylamine cyclotransferase**	Cytoplasm	enzyme		-1.334921047	0	0.27350789	0	1	0	7.59018785	7.80176147	7.50436686	7.27051381
6878	**ENSG0000013487**	COL4A2	protein_coding	chr13	110305812	110513209	+	Human GRCh	OTTHUMG000000	COL4A2	**collagen type IV alpha 2 chain**	Extracellular	other	collagenas	1.261044649	0	0.48689807	0	1	0	7.30474806	7.9056236	7.67035935	8.06840968
6879	**ENSG0000013487**	CLDN10	protein_coding	chr13	95433604	95579759	+	Human GRCh	OTTHUMG000000	CLDN10	**claudin 10**	Plasma Mem	other		3.076678724	1	0.26637589	0	1	0	1.7935392	3.35794315	2.49839782	4.41483212
6880	**ENSG0000013487**	DZIP1	protein_coding	chr13	95578202	95644706	-	Human GRCh	OTTHUMG000000	DZIP1	**DAZ interacting zinc finger protein 1**	Cytoplasm	other		-1.022477696	0	0.9154027	0	1	0	8.58351913	8.68608326	8.5432538	8.67767893
6881	**ENSG0000013488**	UBAC2	protein_coding	chr13	99200774	99386504	+	Human GRCh	OTTHUMG000000	UBAC2	**UBA domain containing 2**	Cytoplasm	enzyme		-1.080685116	0	0.70989231	0	1	0	10.6002386	10.9932781	10.8946318	10.5297401
6882	**ENSG0000013488**	ARGLU1	protein_coding	chr13	106541673	106568137	-	Human GRCh	OTTHUMG000000	ARGLU1	**arginine and glutamate rich 1**	Other	other		1.040149407	0	0.8647427	0	1	0	11.4731989	11.6399288	11.7501949	11.431307
6883	**ENSG0000013489**	BIVM	protein_coding	chr13	102799119	102841533	+	Human GRCh	OTTHUMG000000	BIVM	**basic, immunoglobulin-like variable motif containing**	Extracellular	other		1.158323095	0	0.49560857	0	1	0	9.5214616	9.76024009	10.0153002	9.56427726
6884	**ENSG0000013489**	ERCC5	protein_coding	chr13	102845831	102875995	+	Human GRCh	OTTHUMG000000	ERCC5	**ERCC excision repair 5, endonuclease**	Nucleus	enzyme		-1.218740755	0	0.31435772	0	1	0	10.8195247	11.1793971	10.8519846	10.7315388
6885	**ENSG0000013490**	TPP2	protein_coding	chr13	102596958	102679958	+	Human GRCh	OTTHUMG000000	TPP2	**tripeptidyl peptidase 2**	Cytoplasm	peptidase		-1.100279938	0	0.65696911	0	1	0	11.8126421	12.0508162	11.9934402	11.6465642
6886	**ENSG0000013490**	POGLUT2	protein_coding	chr13	102784281	102799040	-	Human GRCh	OTTHUMG000000	POGLUT2	**protein O-glucosyltransferase 2**	Cytoplasm	enzyme		1.210196	0	0.53550922	0	1	0	7.52262549	7.57704269	8.02474644	7.41094471
6887	**ENSG0000013490**	CARS2	protein_coding	chr13	110641412	110713603	-	Human GRCh	OTTHUMG000000	CARS2	**cysteinyl-tRNA synthetase 2, mitochondrial**	Cytoplasm	enzyme		-1.208094234	0	0.41118834	0	1	0	11.0176376	11.2593659	11.1463134	10.6750264
6888	**ENSG0000013490**	ARHGAP32	protein_coding	chr11	128965060	129279324	-	Human GRCh	OTTHUMG000001	ARHGAP32	**Rho GTPase activating protein 32**	Cytoplasm	other		1.106248802	0	0.75251421	0	1	0	10.1865838	10.4456014	10.5356342	10.3085639
6889	**ENSG0000013491**	STT3A	protein_coding	chr11	125591712	125625215	+	Human GRCh	OTTHUMG000001	STT3A	**STT3 oligosaccharyltransferase complex catalytic subunit A**	Plasma Mem	enzyme		-1.2113771	0	0.3873174	0	1	0	12.5644404	12.827875	12.5831377	12.3823298
6890	**ENSG0000013491**	ADAMTS8	protein_coding	chr11	130404923	130428609	-	Human GRCh	OTTHUMG000001	ADAMTS8	**ADAM metallopeptidase with thrombospondin type 1 motif 8**	Extracellular	peptidase		-1.170540705	0	0.71816713	0	1	0	6.10952898	6.8247249	6.1839934	6.47855392
6891	**ENSG0000013494**	ACRV1	protein_coding	chr11	125671522	125680874	-	Human GRCh	OTTHUMG000001	ACRV1	**acrosomal vesicle protein 1**	Cytoplasm	other		-1.165584821	0	0.86894052	0	NA	0	3.10305965	2.32871529	2.7511693	2.69151469
6892	**ENSG0000013495**	ETS1	protein_coding	chr11	128458761	128587558	-	Human GRCh	OTTHUMG000001	ETS1	**ETS proto-oncogene 1, transcription factor**	Nucleus	transcription		-5.51914431	-1	0.17619402	0	1	0	1.88442202	2.67753266	1.98215577	1.89152948
6893	**ENSG0000013495**	SLC37A2	protein_coding	chr11	125063302	125090516	+	Human GRCh	OTTHUMG000001	SLC37A2	**solute carrier family 37 member 2**	Cytoplasm	transporter		-1.095605869	0	0.85457907	0	1	0	5.21384336	5.73190046	5.44561048	5.37284092
6894	**ENSG0000013496**	KLB	protein_coding	chr4	39406930	39451533	+	Human GRCh	OTTHUMG000001	KLB	**klotho beta**	Plasma Mem	enzyme		1.192795857	0	0.76246372	0	1	0	6.04065587	6.01578431	6.24461707	6.20309021
6895	**ENSG0000013497**	TMED7	protein_coding	chr5	115613210	115632992	-	Human GRCh	OTTHUMG000001	TMED7	**transmembrane p24 trafficking protein 7**	Cytoplasm	transporter		1.05810211	0	0.80276915	0	1	0	11.5540454	11.7628257	11.9076869	11.5162497
6896	**ENSG0000013498**	APC	protein_coding	chr5	112707518	112846239	+	Human GRCh	OTTHUMG000001	APC	**APC regulator of WNT signaling pathway**	Nucleus	enzyme		-1.07186836	0	0.72794522	0	1	0	10.9500695	11.2061227	11.0682165	10.9412963
6897	**ENSG0000013498**	NREP	protein_coding	chr5	111662621	111997464	-	Human GRCh	OTTHUMG000001	NREP	**neuronal regeneration related protein**	Cytoplasm	other		-1.378451777	0	0.18646114	0	1	0	9.83791619	10.262292	9.84729626	9.56457553
6898	**ENSG0000013498**	WDR36	protein_coding	chr5	111092321	111130502	+	Human GRCh	OTTHUMG000001	WDR36	**WD repeat domain 36**	Extracellular	other		1.011281118	0	0.95758688	0	1	0	10.6475222	11.008847	11.0589389	10.6072767
6899	**ENSG0000013499**	OSTF1	protein_coding	chr9	75088514	75147265	+	Human GRCh	OTTHUMG000000	OSTF1	**osteoclast stimulating factor 1**	Nucleus	transcription		1.063402101	0	0.8060156	0	1	0	7.52968525	7.82850269	7.91760026	7.56382518
6900	**ENSG0000013500**	RFK	protein_coding	chr9	76385526	76394597	-	Human GRCh	OTTHUMG000000	RFK	**riboflavin kinase**	Cytoplasm	kinase		1.292413827	0	0.28102797	0	1	0	9.18858067	9.24419657	9.65271004	9.31727948
6901	**ENSG0000013501**	UBQLN1	protein_coding	chr9	83659968	83707958	-	Human GRCh	OTTHUMG000000	UBQLN1	**ubiquilin 1**	Cytoplasm	other		-1.142581262	0	0.50099799	0	1	0	12.2209126	12.5972751	12.4015206	12.1338958
6902	**ENSG0000013504**	NAA35	protein_coding	chr9	85941146	86025462	+	Human GRCh	OTTHUMG000000	NAA35	**N-alpha-acetyltransferase 35, NatC auxiliary subunit**	Cytoplasm	other		-1.154029834	0	0.51373373	0	1	0	10.8385477	11.260052	11.0512163	10.7470452
6903	**ENSG0000013504**	C9orf40	protein_coding	chr9	74946583	74952912	-	Human GRCh	OTTHUMG000000	C9orf40	**chromosome 9 open reading frame 40**	Other	other		1.307809287	0	0.2098605	0	1	0	8.35320174	8.54616116	8.87336808	8.5956163
6904	**ENSG0000013504**	ANXA1	protein_coding	chr9	73151865	73170393	+	Human GRCh	OTTHUMG000000	ANXA1	**annexin A1**	Plasma Mem	enzyme	hydrocorti	-1.517193517	-1	0.53657403	0	1	0	3.9012323	3.72978401	3.81371693	3.09492675
6905	**ENSG0000013504**	CTSL	protein_coding	chr9	87724051	87731469	+	Human GRCh	OTTHUMG000000	CTSL	**cathepsin L**	Cytoplasm	peptidase	cathepsin	-1.201143565	0	0.4730066	0	1	0	7.30702199	7.6862737	7.43471025	7.13951979
6906	**ENSG0000013504**	CEMIP2	protein_coding	chr9	71683366	71816690	-	Human GRCh	OTTHUMG000000	CEMIP2	**cell migration inducing hyaluronidase 2**	Cytoplasm	enzyme		-1.019299465	0	0.93400158	0	1	0	11.3622791	11.5748206	11.3859071	11.5145811
6907	**ENSG0000013504**	AGTPBP1	protein_coding	chr9	85546539	85742029	-	Human GRCh	OTTHUMG000000	AGTPBP1	**ATP/GTP binding carboxypeptidase 1**	Nucleus	peptidase		-1.092503707	0	0.69130891	0	1	0	10.2567619	10.4999709	10.4323911	10.1239831
6908	**ENSG0000013505**	GOLM1	protein_coding	chr9	86026146	86100173	-	Human GRCh	OTTHUMG000000	GOLM1	**golgi membrane protein 1**	Cytoplasm	other		1.075453769	0	0.80959306	0	1	0	10.3878266	10.3368679	10.4161003	10.4651916
6909	**ENSG0000013506**	ENTREP1	protein_coding	chr9	69324567	69392558	+	Human GRCh	OTTHUMG000000	ENTREP1	**endosomal transmembrane epsin interactor 1**	Cytoplasm	other		-1.185451527	0	0.79637266	0	1	0	9.33382779	10.069395	9.65324486	9.45513984
6910	**ENSG0000013506**	PSAT1	protein_coding	chr9	78297125	78330093	+	Human GRCh	OTTHUMG000000	PSAT1	**phosphoserine aminotransferase 1**	Cytoplasm	enzyme		-1.140777602	0	0.53661759	0	1	0	11.177032	11.0300551	11.1162195	10.7795877
6911	**ENSG0000013507**	ISCA1	protein_coding	chr9	86264546	86283102	-	Human GRCh	OTTHUMG000000	ISCA1	**iron-sulfur cluster assembly 1**	Cytoplasm	other		-1.159034183	0	0.50522864	0	1	0	9.95950708	10.1828911	10.0762095	9.724673
6912	**ENSG0000013507**	ADAM19	protein_coding	chr5	157395534	157575775	-	Human GRCh	OTTHUMG000001	ADAM19	**ADAM metallopeptidase domain 19**	Plasma Mem	peptidase		4.897562498	1	0.26289434	0	1	0	2.03571503	1.38967188	1.37091858	3.30866664
6913	**ENSG0000013507**	HAVCR2	protein_coding	chr5	157085422	157142869	-	Human GRCh	OTTHUMG000001	HAVCR2	**hepatitis A virus cellular receptor 2**	Plasma Mem	other	LB1410, LY	1.246824818	0	0.74359612	0	1	0	3.61155475	4.3789802	3.88089358	4.52255458
6914	**ENSG0000013508**	CCNJL	protein_coding	chr5	160249083	160345396	-	Human GRCh	OTTHUMG000001	CCNJL	**cyclin J like**	Other	other		-17.9048095	-1	0.03027796	1	1	0	2.89001382	1.62170069	1.61157451	1.56316456
6915	**ENSG0000013509**	TAOK3	protein_coding	chr12	118149801	118372907	-	Human GRCh	OTTHUMG000001	TAOK3	**TAO kinase 3**	Cytoplasm	kinase	TAO3 inhit	1.499016203	0	0.10548655	0	1	0	7.51197941	7.71877564	8.08924402	7.98388965
6916	**ENSG0000013509**	USP30	protein_coding	chr12	109023089	109088023	+	Human GRCh	OTTHUMG000001	USP30	**ubiquitin specific peptidase 30**	Cytoplasm	peptidase		-1.238702013	0	0.3702981	0	1	0	9.04818092	9.29674004	9.0837046	8.7881425
6918	**ENSG0000013509**	MSI1	protein_coding	chr12	120341330	120369164	-	Human GRCh	OTTHUMG000001	MSI1	**musashi RNA binding protein 1**	Cytoplasm	other		1.032097164	0	0.87506951	0	1	0	12.5162541	12.7387295	12.8201687	12.4964843
6919	**ENSG0000013510**	HNF1A	protein_coding	chr12	120978543	121002512	+	Human GRCh	OTTHUMG000001	HNF1A	**HNF1 homeobox A**	Nucleus	transcription		5.610160836	1	0.27544566	0	1	0	0.98352781	1.04730054	1.78186009	0.98737191
6920	**ENSG0000013510**	FBXO21	protein_coding	chr12	117141991	117190470	-	Human GRCh	OTTHUMG000001	FBXO21	**F-box protein 21**	Extracellular	enzyme		-1.098101077	0	0.65273965	0	1	0	10.1531486	10.5078688	10.3033961	10.1787466
6921	**ENSG0000013511**	TBX3	protein_coding	chr12	114670255	114684175	-	Human GRCh	OTTHUMG000001	TBX3	**T-box transcription factor 3**	Nucleus	transcription		1.338660069	0	0.35796069	0	1	0	9.65039705	9.51903937	9.85172659	9.95364975
6923	**ENSG0000013511**	HRK	protein_coding	chr12	116856144	116881441	-	Human GRCh	OTTHUMG000001	HRK	**harakiri, BCL2 interacting protein**	Cytoplasm	other		-1.502205507	-1	0.40470018	0	1	0	5.54237347	5.19789106	4.87610341	5.13919468
6924	**ENSG0000013511**	RNFT2	protein_coding	chr12	116738178	116853631	+	Human GRCh	OTTHUMG000001	RNFT2	**ring finger protein, transmembrane 2**	Other	other		-1.055040122	0	0.79799482	0	1	0	9.97796813	10.2701594	10.0701893	10.0782307
6925	**ENSG0000013512**	P2RX4	protein_coding	chr12	121210065	121234106	+	Human GRCh	OTTHUMG000001	P2RX4	**purinergic receptor P2X 4**	Plasma Mem	ion channel		-1.068310898	0	0.79663289	0	1	0	8.78915082	8.85196139	8.99944416	8.43485976
6926	**ENSG0000013512**	BICDL1	protein_coding	chr12	119989236	120094494	+	Human GRCh	OTTHUMG000001	BICDL1	**BICD family like cargo adaptor 1**	Cytoplasm	other		1.143437438	0	0.59214498	0	1	0	8.10522088	8.21532393	8.311514	8.28470667
6927	**ENSG0000013514**	DTX1	protein_coding	chr12	113056730	113098028	+	Human GRCh	OTTHUMG000001	DTX1	**deltex E3 ubiquitin ligase 1**	Nucleus	transcription		1.098365753	0	0.91041034	0	1	0	3.1676771	3.60193267	2.35478163	3.98727064
6928	**ENSG0000013514**	TRAFD1	protein_coding	chr12	112125538	112153604	+	Human GRCh	OTTHUMG000001	TRAFD1	**TRAF-type zinc finger domain containing 1**	Other	other		-1.160549483	0	0.47212572	0	1	0	10.37487	10.6861319	10.5716725	10.1366904
6929	**ENSG0000013516**	DMTF1	protein_coding	chr7	87152409	87196337	+	Human GRCh	OTTHUMG000001	DMTF1	**cyclin D binding myb like transcription factor 1**	Nucleus	transcription		-1.141493376	0	0.51667125	0	1	0	11.8909606	12.0882019	11.9895152	11.6904649
6931	**ENSG0000013518**	TMEM243	protein_coding	chr7	87196160	87220587	-	Human GRCh	OTTHUMG000001	TMEM243	**transmembrane protein 243**	Other	other		1.084649692	0	0.79544775	0	1	0	7.43048001	8.21642462	8.02777871	7.92088704
6932	**ENSG0000013520**	CCDC146	protein_coding	chr7	77122434	77329533	+	Human GRCh	OTTHUMG000001	CCDC146	**coiled-coil domain containing 146**	Cytoplasm	other		1.045323898	0	0.88373691	0	1	0	6.92941914	7.27778388	7.250483	7.06573139
6933	**ENSG0000013521**	TMEM60	protein_coding	chr7	77793728	77798434	-	Human GRCh	OTTHUMG000001	TMEM60	**transmembrane protein 60**	Other	other		-1.163125608	0	0.58057667	0	1	0	7.10545901	7.79576783	7.41687008	7.25689823
6934	**ENSG0000013521**	CD36	protein_coding	chr7	80369575	80679277	+	Human GRCh	OTTHUMG000001	CD36	**CD36 molecule**	Plasma Mem	transmembra	VT1021, ze	1.976098029	1	0.46433214	0	NA	0	2.58555627	2.862005	2.98199143	3.31781714
6937	**ENSG0000013522**	UGT2B28	protein_coding	chr4	69280475	69295050	+	Human GRCh	OTTHUMG000001	UGT2B28	**UDP glucuronosyltransferase family 2 member B28**	Cytoplasm	enzyme		-1.904847014	-1	0.5842705	0	1	0	7.84892536	7.49821128	6.84096914	6.89286042
6938	**ENSG0000013524**	PNPLA8	protein_coding	chr7	108470417	108569666	-	Human GRCh	OTTHUMG000001	PNPLA8	**patatin like phospholipase domain containing 8**	Cytoplasm	enzyme		-1.042321783	0	0.86414201	0	1	0	9.48425662	9.67532194	9.66938427	9.38989102
6939	**ENSG0000013524**	HILPDA	protein_coding	chr7	128455849	128458418	+	Human GRCh	OTTHUMG000001	HILPDA	**hypoxia inducible lipid droplet associated**	Cytoplasm	other		-1.073866806	0	0.75068969	0	1	0	9.04086389	9.40539707	9.28433614	9.01649303
6940	**ENSG0000013524**	GARIN1B	protein_coding	chr7	128709061	128731743	+	Human GRCh	OTTHUMG000001	GARIN1B	**golgi associated RAB2 interactor 1B**	Cytoplasm	other		-1.905189639	-1	0.14613645	0	1	0	6.05245539	6.53766496	5.53402702	5.72366991
6941	**ENSG0000013524**	RINT1	protein_coding	chr7	105532169	105567677	+	Human GRCh	OTTHUMG000001	RINT1	**RAD50 interactor 1**	Nucleus	other		-1.061648247	0	0.79544926	0	1	0	9.74165762	10.0323698	9.95557659	9.68785165
6942	**ENSG0000013525**	SRPK2	protein_coding	chr7	105110704	105399308	-	Human GRCh	OTTHUMG000001	SRPK2	**SRSF protein kinase 2**	Nucleus	kinase		1.001700449	0	0.99341891	0	1	0	11.0348945	11.3338002	11.3862776	10.9682644
6943	**ENSG0000013525**	KCP	protein_coding	chr7	128862042	128910719	-	Human GRCh	OTTHUMG000001	KCP	**kielin cysteine rich BMP regulator**	Extracellular	other		1.579778084	1	0.08916348	0	1	0	7.03947217	6.93285105	7.62115582	7.27376224
6944	**ENSG0000013526**	TES	protein_coding	chr7	116210506	116258783	+	Human GRCh	OTTHUMG000000	TES	**testin LIM domain protein**	Plasma Mem	other		-1.106457281	0	0.56907084	0	1	0	10.4249271	10.5448221	10.4799256	10.2557087
6945	**ENSG0000013527**	MDFIC	protein_coding	chr7	114922094	115019917	+	Human GRCh	OTTHUMG000000	MDFIC	**MyoD family inhibitor domain containing**	Nucleus	other		-1.023731921	0	0.93089104	0	1	0	8.63710974	8.89939436	8.80605349	8.68785445
6946	**ENSG0000013529**	MTO1	protein_coding	chr6	73461578	73509236	+	Human GRCh	OTTHUMG000000	MTO1	**mitochondrial tRNA translation optimization 1**	Cytoplasm	enzyme		-1.05541688	0	0.80441045	0	1	0	9.87723199	10.0731745	10.1289343	9.66504574
6947	**ENSG0000013529**	ADGRB3	protein_coding	chr6	68635282	69390571	+	Human GRCh	OTTHUMG000000	ADGRB3	**adhesion G protein-coupled receptor B3**	Plasma Mem	G-protein cou		1.655540346	1	0.40355066	0	1	0	6.21430367	6.70731186	6.66023228	7.10549512
6948	**ENSG0000013529**	ANKRD6	protein_coding	chr6	89433152	89633834	+	Human GRCh	OTTHUMG000000	ANKRD6	**ankyrin repeat domain 6**	Nucleus	transcription		-1.073986504	0	0.7794354	0	1	0	8.8594624	9.09686979	9.06223376	8.73575357
6950	**ENSG0000013531**	KHDC1	protein_coding	chr6	73241314	73310365	-	Human GRCh	OTTHUMG000000	KHDC1	**KH domain containing 1**	Other	other		1.077970979	0	0.78075518	0	1	0	7.59530791	8.01288355	7.92788785	7.87457311
6951	**ENSG0000013531**	CEP162	protein_coding	chr6	84124241	84227643	-	Human GRCh	OTTHUMG000000	CEP162	**centrosomal protein 162**	Nucleus	other		-1.063133437	0	0.79563298	0	1	0	8.75146431	9.11509602	9.02957133	8.71425105
6952	**ENSG0000013531**	SYNCRIP	protein_coding	chr6	85607779	85644063	-	Human GRCh	OTTHUMG000000	SYNCRIP	**synaptotagmin binding cytoplasmic RNA interacting protein**	Nucleus	other	supinoxin	1.049861332	0	0.80749872	0	1	0	12.5320349	12.8379245	12.9053296	12.5665455
6953	**ENSG0000013531**	SNX14	protein_coding	chr6	85504776	85594156	-	Human GRCh	OTTHUMG000000	SNX14	**sorting nexin 14**	Cytoplasm	transporter		-1.075595723	0	0.73831909	0	1	0	11.5713735	11.9289855	11.8850411	11.4384628
6954	**ENSG0000013531**	NT5E	protein_coding	chr6	85449584	85495791	+	Human GRCh	OTTHUMG000000	NT5E	**5'-nucleotidase ecto**	Plasma Mem	phosphatase	BMS-9861	1.548480273	1	0.54639546	0	1	0	3.88946963	4.21628716	3.60415587	4.92528546
6955	**ENSG0000013532**	MRAP2	protein_coding	chr6	84033772	84090881	+	Human GRCh	OTTHUMG000000	MRAP2	**melanocortin 2 receptor accessory protein 2**	Cytoplasm	other		3.263802214	1	0.13661382	0	1	0	3.00603653	2.05422788	3.75494358	2.78041343
6956	**ENSG0000013533**	EPHA7	protein_coding	chr6	93240020	93419687	-	Human GRCh	OTTHUMG000000	EPHA7	**EPH receptor A7**	Plasma Mem	kinase		1.124569933	0	0.72685379	0	1	0	9.37812973	9.52334895	9.76607754	9.35507965
6957	**ENSG0000013533**	AKIRIN2	protein_coding	chr6	87674860	87702233	-	Human GRCh	OTTHUMG000000	AKIRIN2	**akirin 2**	Nucleus	other		-1.111210353	0	0.58760624	0	1	0	9.78412743	10.0594624	9.94375496	9.67381455
6958	**ENSG0000013533**	ORC3	protein_coding	chr6	87590067	87667453	+	Human GRCh	OTTHUMG000000	ORC3	**origin recognition complex subunit 3**	Nucleus	other		-1.098942348	0	0.65037962	0	1	0	9.98532064	10.2984729	10.1078472	9.98764247
6959	**ENSG0000013533**	LCA5	protein_coding	chr6	79484991	79537458	-	Human GRCh	OTTHUMG000000	LCA5	**lebercilin LCA5**	Extracellular	other		-1.183703118	0	0.5792545	0	1	0	6.97791624	7.41462829	7.01471411	7.07864734
6960	**ENSG0000013534**	MAP3K7	protein_coding	chr6	90513573	90587086	-	Human GRCh	OTTHUMG000000	MAP3K7	**mitogen-activated protein kinase kinase kinase 7**	Cytoplasm	kinase	takinib	-1.014018308	0	0.94956794	0	1	0	10.7417791	10.9713829	11.0859668	10.5466872
6963	**ENSG0000013536**	PRR5L	protein_coding	chr11	36296288	36465204	+	Human GRCh	OTTHUMG000001	PRR5L	**proline rich 5 like**	Cytoplasm	other		1.097645944	0	0.79155052	0	1	0	5.89187443	6.24438928	6.19822059	6.15371765
6964	**ENSG0000013536**	LMO2	protein_coding	chr11	33858576	33892076	-	Human GRCh	OTTHUMG000001	LMO2	**LIM domain only 2**	Nucleus	transcription		-2.104176454	-1	0.2917942	0	1	0	3.33058841	3.7882379	2.60394554	3.1831048
6965	**ENSG0000013536**	PHF21A	protein_coding	chr11	45929319	46121454	-	Human GRCh	OTTHUMG000001	PHF21A	**PHD finger protein 21A**	Nucleus	other		-1.150418411	0	0.49196645	0	1	0	10.4872429	10.6686292	10.5386448	10.3155389
6966	**ENSG0000013537**	NAT10	protein_coding	chr11	34105617	34147670	+	Human GRCh	OTTHUMG000001	NAT10	**N-acetyltransferase 10**	Nucleus	enzyme		-1.037297149	0	0.86348827	0	1	0	11.4124711	11.8338151	11.7580012	11.41863
6967	**ENSG0000013537**	EHF	protein_coding	chr11	34621093	34663288	+	Human GRCh	OTTHUMG000001	EHF	**ETS homologous factor**	Nucleus	transcription		-1.209690646	0	0.73287122	0	1	0	12.0977361	12.5168929	12.3618656	11.7550836

6968	**ENSG0000013537**	ELF5	protein_coding	chr11	34478791	34525193	-	Human GRCh	OTTHUMG000001	ELF5	**E74 like ETS transcription factor 5**	Nucleus	transcription		-1.061846697	0	0.92105469	0	1	0	10.2491071	10.5691117	10.6044981	10.0206569
6969	**ENSG0000013537**	PRRG4	protein_coding	chr11	32829927	32858120	+	Human GRCh	OTTHUMG000001	PRRG4	**proline rich and Gla domain 4**	Plasma Mem	other		1.168777394	0	0.53257781	0	1	0	7.85739156	8.10277738	8.23606105	8.0525053
6970	**ENSG0000013538**	CAPRIN1	protein_coding	chr11	34051731	34102610	+	Human GRCh	OTTHUMG000001	CAPRIN1	**cell cycle associated protein 1**	Cytoplasm	translation re		-1.084759799	0	0.72725096	0	1	0	13.2884369	13.6508286	13.53815	13.2258786
6971	**ENSG0000013539**	ATP5MC2	protein_coding	chr12	53632726	53677408	-	Human GRCh	OTTHUMG000001	ATP5MC2	**ATP synthase membrane subunit c locus 2**	Cytoplasm	transporter		-1.027294565	0	0.90264409	0	1	0	11.6239708	11.8792632	11.8426482	11.6029935
6972	**ENSG0000013539**	DNAJC14	protein_coding	chr12	55820960	55830824	-	Human GRCh	OTTHUMG000001	DNAJC14	**DnaJ heat shock protein family (Hsp40) member C14**	Cytoplasm	other		1.002760848	0	0.98889018	0	1	0	10.3161329	10.6182775	10.6789681	10.2398272
6973	**ENSG0000013540**	CD63	protein_coding	chr12	55725323	55729707	-	Human GRCh	OTTHUMG000001	CD63	**CD63 molecule**	Plasma Mem	other		1.078633378	0	0.7161427	0	1	0	10.9006068	11.161113	11.2145803	11.0181021
6974	**ENSG0000013540**	PRPH	protein_coding	chr12	49295147	49298686	+	Human GRCh	OTTHUMG000001	PRPH	**peripherin**	Plasma Mem	other		-7.719639804	-1	0.02107959	1	1	0	3.73246763	3.13047256	2.77349968	2.21535777
6975	**ENSG0000013540**	AVIL	protein_coding	chr12	57797376	57818734	-	Human GRCh	OTTHUMG000001	AVIL	**advillin**	Plasma Mem	other		-1.417093624	0	0.269762	0	1	0	6.84948751	6.53896941	6.6819646	5.90410983
6976	**ENSG0000013540**	AMHR2	protein_coding	chr12	53423855	53431672	+	Human GRCh	OTTHUMG000001	AMHR2	**anti-Mullerian hormone receptor type 2**	Plasma Mem	kinase	GM102	-1.361620934	0	0.75029437	0	1	0	2.8208364	3.5854615	2.93055376	3.17406294
6978	**ENSG0000013541**	GDF11	protein_coding	chr12	55743122	55757264	+	Human GRCh	OTTHUMG000001	GDF11	**growth differentiation factor 11**	Extracellular	growth facto	luspaterce	1.063195407	0	0.78008251	0	1	0	9.87218884	10.0263223	10.0790613	9.95598156
6979	**ENSG0000013542**	GLS2	protein_coding	chr12	56470944	56488414	-	Human GRCh	OTTHUMG000001	GLS2	**glutaminase 2**	Cytoplasm	enzyme		1.373262147	0	0.25267712	0	1	0	7.20441073	7.37291411	7.68535133	7.54751064
6980	**ENSG0000013542**	ITGA7	protein_coding	chr12	55684568	55716404	-	Human GRCh	OTTHUMG000001	ITGA7	**integrin subunit alpha 7**	Plasma Mem	other		-1.366711748	0	0.27328405	0	1	0	6.6412007	6.49497854	6.36950599	6.14238892
6982	**ENSG0000013543**	FAM186B	protein_coding	chr12	49582885	49605639	-	Human GRCh	OTTHUMG000001	FAM186B	**family with sequence similarity 186 member B**	Other	other		1.560555633	1	0.59994704	0	1	0	4.02759815	3.57894108	4.41592854	3.93608137
6983	**ENSG0000013543**	RDH5	protein_coding	chr12	55720367	55724705	+	Human GRCh	OTTHUMG000001	RDH5	**retinol dehydrogenase 5**	Cytoplasm	enzyme		-1.436519042	0	0.22446173	0	1	0	7.56976226	8.13338672	7.79223212	7.13244807
6984	**ENSG0000013543**	AGAP2	protein_coding	chr12	57723761	57742157	-	Human GRCh	OTTHUMG000001	AGAP2	**ArfGAP with GTPase domain, ankyrin repeat and PH domain 2**	Nucleus	enzyme		2.43959851	1	0.02306196	1	1	0	5.16167163	5.49300161	6.38042663	6.00220652
6985	**ENSG0000013544**	BLOC1S1	protein_coding	chr12	55716038	55720087	+	Human GRCh	OTTHUMG000001	BLOC1S1	**biogenesis of lysosomal organelles complex 1 subunit 1**	Cytoplasm	other		1.038763858	0	0.85101676	0	1	0	9.13502876	9.32361862	9.39283685	9.14167083
6987	**ENSG0000013544**	CDK4	protein_coding	chr12	57747727	57756013	-	Human GRCh	OTTHUMG000001	CDK4	**cyclin dependent kinase 4**	Nucleus	kinase	fulvestrant	-1.169899685	0	0.41442462	0	1	0	11.7542908	12.0909112	11.8484125	11.6696693
6988	**ENSG0000013544**	PPP1R1A	protein_coding	chr12	54575387	54588659	-	Human GRCh	OTTHUMG000001	PPP1R1A	**protein phosphatase 1 regulatory inhibitor subunit 1A**	Cytoplasm	phosphatase		1.004194409	0	0.99623567	0	1	0	3.38167738	3.42189254	2.42399089	3.88406261
6989	**ENSG0000013545**	TROAP	protein_coding	chr12	49323236	49331731	+	Human GRCh	OTTHUMG000001	TROAP	**trophinin associated protein**	Cytoplasm	peptidase		-1.146075588	0	0.48876638	0	1	0	10.9739491	11.2775492	11.1095484	10.845533
6990	**ENSG0000013545**	TSPAN31	protein_coding	chr12	57738013	57750219	+	Human GRCh	OTTHUMG000001	TSPAN31	**tetraspanin 31**	Plasma Mem	other		1.022943533	0	0.92952782	0	1	0	7.91770663	7.97578271	8.01332204	7.92749067
6991	**ENSG0000013545**	B4GALNT1	protein_coding	chr12	57623409	57633239	-	Human GRCh	OTTHUMG000001	B4GALNT1	**beta-1,4-N-acetyl-galactosaminyltransferase 1**	Cytoplasm	enzyme		-1.669090046	-1	0.07412086	0	1	0	7.47675337	7.14972719	6.69676201	6.93507557
6992	**ENSG0000013545**	TFCP2	protein_coding	chr12	51093656	51173135	-	Human GRCh	OTTHUMG000001	TFCP2	**transcription factor CP2**	Nucleus	transcription		1.338215131	0	0.76666514	0	1	0	3.39104128	3.30897145	3.49885419	3.39881567
6993	**ENSG0000013546**	COQ10A	protein_coding	chr12	56266890	56270966	+	Human GRCh	OTTHUMG000001	COQ10A	**coenzyme Q10A**	Cytoplasm	other		1.053257682	0	0.84399793	0	1	0	7.77930056	7.90782936	8.05136876	7.72624802
6994	**ENSG0000013547**	FAIM2	protein_coding	chr12	49866896	49904017	-	Human GRCh	OTTHUMG000001	FAIM2	**Fas apoptotic inhibitory molecule 2**	Plasma Mem	other		3.287274525	1	0.5138325	0	NA	0	0.78749643	0.47367513	1.08566232	0.79093261
6995	**ENSG0000013547**	PAN2	protein_coding	chr12	56316223	56334053	-	Human GRCh	OTTHUMG000001	PAN2	**poly(A) specific ribonuclease subunit PAN2**	Cytoplasm	peptidase		-1.162163124	0	0.52901786	0	1	0	11.4417812	11.6072631	11.4979193	11.2131282
6996	**ENSG0000013547**	ESPL1	protein_coding	chr12	53268299	53293638	+	Human GRCh	OTTHUMG000001	ESPL1	**extra spindle pole bodies like 1, separase**	Nucleus	peptidase		-1.151468488	0	0.50843508	0	1	0	11.7543042	12.0955951	11.9402883	11.6023282
6997	**ENSG0000013548**	KRT7	protein_coding	chr12	52232520	52252186	+	Human GRCh	OTTHUMG000001	KRT7	**keratin 7**	Cytoplasm	other		2.96252895	1	0.2774011	0	1	0	2.92440054	3.75458831	3.24719234	4.59052715
6998	**ENSG0000013548**	ZC3H10	protein_coding	chr12	56118260	56127514	+	Human GRCh	OTTHUMG000001	ZC3H10	**zinc finger CCCH-type containing 10**	Nucleus	other		-1.032546882	0	0.87977431	0	1	0	9.71677891	9.98136335	9.94260121	9.6861444
6999	**ENSG0000013548**	HNRNPA1	protein_coding	chr12	54280193	54287088	+	Human GRCh	OTTHUMG000001	HNRNPA1	**heterogeneous nuclear ribonucleoprotein A1**	Nucleus	other		1.068865404	0	0.75943236	0	1	0	13.9840531	14.2254169	14.3710278	13.9628713
7000	**ENSG0000013550**	ACVR1B	protein_coding	chr12	51951699	51997078	+	Human GRCh	OTTHUMG000001	ACVR1B	**activin A receptor type 1B**	Plasma Mem	kinase	SB-505124	-1.163946243	0	0.46514496	0	1	0	10.3908149	10.5781122	10.3780911	10.2596844
7001	**ENSG0000013550**	OS9	protein_coding	chr12	57693841	57728342	+	Human GRCh	OTTHUMG000001	OS9	**OS9 endoplasmic reticulum lectin**	Nucleus	other		-1.142001982	0	0.50923448	0	1	0	11.8412828	12.0627742	11.9261033	11.6833707
7002	**ENSG0000013551**	MIP	protein_coding	chr12	56449502	56469166	-	Human GRCh	OTTHUMG000001	MIP	**major intrinsic protein of lens fiber**	Plasma Mem	transporter		-1.904489742	-1	0.52407793	0	NA	0	2.89623336	2.38298449	1.84507334	2.69224354
7003	**ENSG0000013551**	KCNH3	protein_coding	chr12	49539030	49558337	+	Human GRCh	OTTHUMG000001	KCNH3	**potassium voltage-gated channel subfamily H member 3**	Plasma Mem	ion channel		-1.783610514	-1	0.1206452	0	1	0	4.67522052	5.23283276	4.52168253	4.45031756
7004	**ENSG0000013552**	LTV1	protein_coding	chr6	143843338	143863812	+	Human GRCh	OTTHUMG000001	LTV1	**LTV1 ribosome biogenesis factor**	Other	other		1.038975513	0	0.86099631	0	1	0	9.16359491	9.56696993	9.53883925	9.29767596
7005	**ENSG0000013552**	MAP7	protein_coding	chr6	136342281	136550819	-	Human GRCh	OTTHUMG000000	MAP7	**microtubule associated protein 7**	Cytoplasm	other		-1.170327702	0	0.45013	0	1	0	11.0954533	11.2351623	11.0344037	10.9450673
7006	**ENSG0000013553**	CD164	protein_coding	chr6	109366514	109382467	-	Human GRCh	OTTHUMG000000	CD164	**CD164 molecule**	Plasma Mem	other		1.058850756	0	0.80085755	0	1	0	12.5249191	12.6738992	12.8604671	12.4306961
7007	**ENSG0000013553**	AFG1L	protein_coding	chr6	108294991	108526796	+	Human GRCh	OTTHUMG000000	AFG1L	**AFG1 like ATPase**	Cytoplasm	other		1.181082377	0	0.50465473	0	1	0	8.08086892	7.91277783	8.35560167	7.9649335
7008	**ENSG0000013554**	NHSL1	protein_coding	chr6	138422043	138692571	-	Human GRCh	OTTHUMG000000	NHSL1	**NHS like 1**	Other	other		-1.126468809	0	0.6493056	0	1	0	10.2533018	10.4904929	10.3193259	10.1779816
7009	**ENSG0000013554**	AHI1	protein_coding	chr6	135283407	135498434	-	Human GRCh	OTTHUMG000000	AHI1	**Abelson helper integration site 1**	Cytoplasm	other		-1.007907338	0	0.97146143	0	1	0	10.9015076	11.1453214	11.190759	10.8228723
7010	**ENSG0000013554**	HEY2	protein_coding	chr6	125747664	125761269	+	Human GRCh	OTTHUMG000000	HEY2	**hes related family bHLH transcription factor with YRPW motif**	Nucleus	transcription		2.065773682	1	0.26332558	0	1	0	3.30540043	4.46900613	4.44032966	4.76454809
7011	**ENSG0000013554**	PKIB	protein_coding	chr6	122471917	122726373	+	Human GRCh	OTTHUMG000000	PKIB	**cAMP-dependent protein kinase inhibitor beta**	Other	other		1.703196457	1	0.14563864	0	1	0	6.27355298	6.50575579	6.58661514	7.17663737
7013	**ENSG0000013557**	NMBR	protein_coding	chr6	142058330	142147122	-	Human GRCh	OTTHUMG000000	NMBR	**neuromedin B receptor**	Plasma Mem	G-protein co		5.672494414	1	0.3323328	0	NA	0	1.29771624	1.31259412	1.71569113	1.29857632
7014	**ENSG0000013558**	SMPD2	protein_coding	chr6	109440724	109443919	+	Human GRCh	OTTHUMG000000	SMPD2	**sphingomyelin phosphodiesterase 2**	Cytoplasm	enzyme		-1.129081309	0	0.59801942	0	1	0	8.07963511	8.19201741	8.05327768	7.9642135
7015	**ENSG0000013559**	MICAL1	protein_coding	chr6	109444062	109465968	-	Human GRCh	OTTHUMG000000	MICAL1	**microtubule associated monooxygenase, calponin and LIM do**	Cytoplasm	enzyme		1.210279969	0	0.46108159	0	1	0	8.55247756	8.52302779	8.67843224	8.78003044
7016	**ENSG0000013559**	REPS1	protein_coding	chr6	138903493	138988261	-	Human GRCh	OTTHUMG000000	REPS1	**RALBP1 associated Eps domain containing 1**	Plasma Mem	other		1.009978156	0	0.96278763	0	1	0	11.1734275	11.4947029	11.4239419	11.2836882
7018	**ENSG0000013560**	TEC	protein_coding	chr4	48135783	48269838	-	Human GRCh	OTTHUMG000001	TEC	**tec protein tyrosine kinase**	Cytoplasm	kinase	ritlecitinib,	-1.069074233	0	0.84381011	0	1	0	6.14507029	6.6688691	6.20390924	6.50750707
7019	**ENSG0000013561**	PRADC1	protein_coding	chr2	73228010	73233239	-	Human GRCh	OTTHUMG000001	PRADC1	**protease associated domain containing 1**	Extracellular	other		-1.001237753	0	0.99641194	0	1	0	8.98754595	9.45093618	9.25436637	9.22502512
7020	**ENSG0000013562**	SEMA4F	protein_coding	chr2	74654228	74683853	+	Human GRCh	OTTHUMG000001	SEMA4F	**ssemaphorin 4F**	Plasma Mem	other		1.016769261	0	0.95787311	0	1	0	7.33958866	7.53176474	7.67845167	7.1831099
7021	**ENSG0000013562**	CCT7	protein_coding	chr2	73233420	73253021	+	Human GRCh	OTTHUMG000001	CCT7	**chaperonin containing TCP1 subunit 7**	Cytoplasm	other		-1.141862785	0	0.55272994	0	1	0	12.421361	12.7263839	12.5082432	12.3522127
7023	**ENSG0000013563**	RAB11FIP5	protein_coding	chr2	73073382	73156721	-	Human GRCh	OTTHUMG000001	RAB11FIP5	**RAB11 family interacting protein 5**	Cytoplasm	other		2.011452221	1	0.0049117	1	1	0	9.41987076	9.58932028	9.67185969	10.728664
7024	**ENSG0000013563**	SMYD5	protein_coding	chr2	73214222	73227221	+	Human GRCh	OTTHUMG000001	SMYD5	**SMYD family member 5**	Nucleus	enzyme		-1.105786483	0	0.64086724	0	1	0	9.87138912	10.0376751	10.0160453	9.64647257
7025	**ENSG0000013563**	DYSF	protein_coding	chr2	71453561	71686763	+	Human GRCh	OTTHUMG000001	DYSF	**dysferlin**	Plasma Mem	other		1.295927223	0	0.53722103	0	1	0	5.39670108	4.97906374	5.33642735	5.54416808
7026	**ENSG0000013563**	CCDC142	protein_coding	chr2	74471986	74483408	-	Human GRCh	OTTHUMG000001	CCDC142	**coiled-coil domain containing 142**	Other	other		-1.121632252	0	0.60484615	0	1	0	9.80941177	10.0974166	10.0346767	9.59723927
7027	**ENSG0000013563**	EMX1	protein_coding	chr2	72916260	72936071	+	Human GRCh	OTTHUMG000001	EMX1	**empty spiracles homeobox 1**	Nucleus	transcription		1.337512062	0	0.55382244	0	1	0	3.88086653	4.39012627	4.36314833	4.36864781
7028	**ENSG0000013564**	KCNMB4	protein_coding	chr12	70366290	70434292	+	Human GRCh	OTTHUMG000001	KCNMB4	**potassium calcium-activated channel subfamily M regulatory b**	Plasma Mem	ion channel	tedisamil	-1.080650567	0	0.858056	0	1	0	5.52235634	5.99438362	5.66584777	5.74527521
7029	**ENSG0000013565**	USP15	protein_coding	chr12	62260338	62417431	+	Human GRCh	OTTHUMG000001	USP15	**ubiquitin specific peptidase 15**	Cytoplasm	peptidase		-1.07908441	0	0.72112545	0	1	0	10.8768322	11.2021239	11.1194914	10.7921838
7030	**ENSG0000013567**	GNS	protein_coding	chr12	64713445	64759431	-	Human GRCh	OTTHUMG000001	GNS	**glucosamine (N-acetyl)-6-sulfatase**	Cytoplasm	enzyme		-1.088358942	0	0.67903951	0	1	0	11.4422644	11.7454534	11.6815739	11.3114844
7031	**ENSG0000013567**	CPM	protein_coding	chr12	68842197	68971570	-	Human GRCh	OTTHUMG000001	CPM	**carboxypeptidase M**	Plasma Mem	peptidase		1.30411233	0	0.47368118	0	1	0	4.74316359	5.15180914	5.12432126	5.18371465
7032	**ENSG0000013567**	MDM2	protein_coding	chr12	68808177	68845544	+	Human GRCh	OTTHUMG000001	MDM2	**MDM2 proto-oncogene**	Nucleus	enzyme	ASTX295, I	-1.156698828	0	0.45243305	0	1	0	12.5427354	12.8840384	12.6996264	12.4134008
7033	**ENSG0000013568**	KLHL36	protein_coding	chr16	84648511	84667686	+	Human GRCh	OTTHUMG000001	KLHL36	**kelch like family member 36**	Cytoplasm	other		1.049657287	0	0.8401911	0	1	0	10.0864216	10.2848037	10.3672272	10.1023069
7035	**ENSG0000013569**	MPHOSPH	protein_coding	chr16	82147798	82170224	-	Human GRCh	OTTHUMG000001	MPHOSPH6	**M-phase phosphoprotein 6**	Nucleus	other		1.012080235	0	0.95948751	0	1	0	8.27172006	8.50881086	8.63753361	8.12590655
7036	**ENSG0000013569**	CHST5	protein_coding	chr16	75528530	75536108	-	Human GRCh	OTTHUMG000001	CHST5	**carbohydrate sulfotransferase 5**	Cytoplasm	enzyme		-1.355532929	0	0.39488482	0	1	0	6.30480553	6.68055788	6.15170803	6.19356129
7037	**ENSG0000013570**	KIAA0513	protein_coding	chr16	85027782	85094230	+	Human GRCh	OTTHUMG000001	KIAA0513	**KIAA0513**	Other	other		1.267334175	0	0.34407465	0	1	0	7.18135449	7.32363693	7.31764581	7.66061052
7038	**ENSG0000013572**	DYNC1LI2	protein_coding	chr16	66720893	66751609	-	Human GRCh	OTTHUMG000001	DYNC1LI2	**dynein cytoplasmic 1 light intermediate chain 2**	Cytoplasm	other		-1.129880299	0	0.56012963	0	1	0	11.9917119	12.2817224	12.1771124	11.8150152
7039	**ENSG0000013572**	FBXL8	protein_coding	chr16	67159932	67164570	+	Human GRCh	OTTHUMG000001	FBXL8	**F-box and leucine rich repeat protein 8**	Cytoplasm	other		-1.050114758	0	0.8593041	0	1	0	9.3897795	9.51954203	9.56756962	9.20733148
7040	**ENSG0000013572**	FHOD1	protein_coding	chr16	67229387	67247481	-	Human GRCh	OTTHUMG000001	FHOD1	**formin homology 2 domain containing 1**	Cytoplasm	other		-1.014929088	0	0.94880482	0	1	0	9.50313865	9.76998908	9.67024868	9.5831729
7041	**ENSG0000013573**	CCDC102A	protein_coding	chr16	57512181	57536571	-	Human GRCh	OTTHUMG000001	CCDC102A	**coiled-coil domain containing 102A**	Other	other		-1.207135641	0	0.69647858	0	1	0	5.89137103	5.94310026	5.51583765	5.90457354
7042	**ENSG0000013574**	SLC9A5	protein_coding	chr16	67237683	67272191	+	Human GRCh	OTTHUMG000001	SLC9A5	**solute carrier family 9 member A5**	Plasma Mem	transporter		1.006071424	0	0.98610673	0	1	0	6.2521131	6.86495831	6.59039404	6.61647427
7043	**ENSG0000013574**	AGT	protein_coding	chr1	230690776	230745576	-	Human GRCh	OTTHUMG000000	AGT	**angiotensinogen**	Extracellular	growth facto	amlodipine	1.526640951	1	0.64979204	0	1	0	11.4583595	11.8367585	12.6431444	11.4514799
7044	**ENSG0000013574**	PCNX2	protein_coding	chr1	232983435	233295725	-	Human GRCh	OTTHUMG000000	PCNX2	**pecanex 2**	Other	other		1.020256914	0	0.9298887	0	1	0	10.3049425	10.6067442	10.5734094	10.3944252
7045	**ENSG0000013575**	KCNK1	protein_coding	chr1	233614106	233672514	+	Human GRCh	OTTHUMG000000	KCNK1	**potassium two pore domain channel subfamily K member 1**	Plasma Mem	ion channel		-1.168442721	0	0.55587446	0	1	0	9.9960936	10.2328613	9.8591708	10.0337595
7046	**ENSG0000013576**	URB2	protein_coding	chr1	229626247	229660200	+	Human GRCh	OTTHUMG000000	URB2	**URB2 ribosome biogenesis homolog**	Nucleus	other		1.029350442	0	0.88981944	0	1	0	10.8393658	11.2164876	11.2847979	10.8205658
7047	**ENSG0000013576**	EGLN1	protein_coding	chr1	231363751	231422287	-	Human GRCh	OTTHUMG000000	EGLN1	**egl-9 family hypoxia inducible factor 1**	Cytoplasm	enzyme		1.241339309	0	0.3376972	0	1	0	11.7645854	12.2538466	12.4582251	12.075369
7048	**ENSG0000013577**	CAPN9	protein_coding	chr1	230747384	230802003	+	Human GRCh	OTTHUMG000000	CAPN9	**calpain 9**	Cytoplasm	peptidase	BLD-2660	1.566388682	1	0.24443906	0	1	0	7.57407292	7.66245904	8.60360914	7.29444855
7049	**ENSG0000013577**	COG2	protein_coding	chr1	230642481	230693982	+	Human GRCh	OTTHUMG000000	COG2	**component of oligomeric golgi complex 2**	Cytoplasm	other		-1.10455068	0	0.6795204	0	1	0	11.2567725	11.5411382	11.5739637	10.929719
7050	**ENSG0000013577**	ABCB10	protein_coding	chr1	229516582	229558707	-	Human GRCh	OTTHUMG000000	ABCB10	**ATP binding cassette subfamily B member 10**	Cytoplasm	transporter		1.007668144	0	0.97172068	0	1	0	10.2215472	10.4183729	10.5061232	10.1292425
7051	**ENSG0000013577**	NTPCR	protein_coding	chr1	232950605	232983882	+	Human GRCh	OTTHUMG000000	NTPCR	**nucleoside-triphosphatase, cancer-related**	Cytoplasm	enzyme		1.087346736	0	0.72017225	0	1	0	10.8381479	11.1232068	11.2392255	10.9030933
7052	**ENSG0000013580**	TAF5L	protein_coding	chr1	229593134	229626122	-	Human GRCh	OTTHUMG000000	TAF5L	**TATA-box binding protein associated factor 5 like**	Nucleus	transcription		-1.008198391	0	0.9678892	0	1	0	10.4900314	10.8150655	10.7360944	10.5668333
7053	**ENSG0000013582**	GLUL	protein_coding	chr1	182378098	182392206	-	Human GRCh	OTTHUMG000000	GLUL	**glutamate-ammonia ligase**	Cytoplasm	enzyme	manganes	-1.062175893	0	0.776362	0	1	0	13.3922152	13.5674494	13.469931	13.3612681
7054	**ENSG0000013582**	STX6	protein_coding	chr1	180972712	181023121	-	Human GRCh	OTTHUMG000000	STX6	**syntaxin 6**	Cytoplasm	transporter		1.018568359	0	0.92703525	0	1	0	10.50921	10.7351228	10.8376474	10.4214908
7055	**ENSG0000013582**	RGS8	protein_coding	chr1	182641816	182684587	-	Human GRCh	OTTHUMG000000	RGS8	**regulator of G protein signaling 8**	Cytoplasm	enzyme		-11.62973244	-1	0.06938594	0	1	0	0.86334479	2.22949435	0.92557608	0.86784148
7056	**ENSG0000013582**	RNASEL	protein_coding	chr1	182573634	182589256	-	Human GRCh	OTTHUMG000000	RNASEL	**ribonuclease L**	Cytoplasm	enzyme		-1.050161349	0	0.88506681	0	1	0	5.1505757	4.91741478	4.7956715	5.16281314
7057	**ENSG0000013582**	DHX9	protein_coding	chr1	182839347	182887982	+	Human GRCh	OTTHUMG000000	DHX9	**DExH-box helicase 9**	Nucleus	enzyme		-1.110203241	0	0.58493	0	1	0	12.9654428	13.2658721	13.1393724	12.8624153

7058	**ENSG0000013583**	KIAA1614	protein_coding	chr1	180912897	180951614	+	Human GRCh	OTTHUMG000000	KIAA1614	**KIAA1614**	Other	other		-1.297848394	0	0.36736877	0	1	0	6.21255834	6.6128341	6.10487903	6.24110183
7059	**ENSG0000013583**	CEP350	protein_coding	chr1	179954674	180114880	+	Human GRCh	OTTHUMG000000	CEP350	**centrosomal protein 350**	Cytoplasm	other		-1.116289696	0	0.60151176	0	1	0	12.5755599	12.8723399	12.7781009	12.4181624
7060	**ENSG0000013583**	NPL	protein_coding	chr1	182789293	182830384	+	Human GRCh	OTTHUMG000000	NPL	**N-acetylneuraminate pyruvate lyase**	Cytoplasm	enzyme		-1.035853633	0	0.94058778	0	1	0	4.74800369	5.30478909	5.01803286	5.03323125
7061	**ENSG0000013583**	NIBAN1	protein_coding	chr1	184790724	184974508	-	Human GRCh	OTTHUMG000000	NIBAN1	**niban apoptosis regulator 1**	Cytoplasm	other		-1.237157072	0	0.62596509	0	1	0	9.97561215	9.58406636	9.23983054	9.87036771
7062	**ENSG0000013584**	PIGC	protein_coding	chr1	172370189	172444086	-	Human GRCh	OTTHUMG000000	PIGC	**phosphatidylinositol glycan anchor biosynthesis class C**	Cytoplasm	enzyme		-1.091643571	0	0.71236066	0	1	0	8.76847786	9.02585532	8.78745328	8.83285318
7063	**ENSG0000013586**	LAMC1	protein_coding	chr1	183023420	183145592	+	Human GRCh	OTTHUMG000000	LAMC1	**laminin subunit gamma 1**	Extracellular	other		-1.080665086	0	0.79824126	0	1	0	11.6266075	11.6955509	11.47019	11.6743295
7064	**ENSG0000013587**	RC3H1	protein_coding	chr1	173931084	174022357	-	Human GRCh	OTTHUMG000000	RC3H1	**ring finger and CCCH-type domains 1**	Cytoplasm	enzyme		-1.136459292	0	0.51090926	0	1	0	11.9997277	12.244854	12.1221618	11.8337605
7065	**ENSG0000013589**	GPR55	protein_coding	chr2	230907318	230961066	-	Human GRCh	OTTHUMG000001	GPR55	**G protein-coupled receptor 55**	Plasma Mem	G-protein co	cannabidic	1	0	1	0	1	0	0.96253229	1.04424598	1.03117825	0.96751205
7066	**ENSG0000013589**	SP110	protein_coding	chr2	230165186	230225729	-	Human GRCh	OTTHUMG000001	SP110	**SP110 nuclear body protein**	Nucleus	transcription		1.02810441	0	0.94495836	0	1	0	6.34388343	6.80650547	6.97552384	6.1227573
7067	**ENSG0000013590**	MRPL44	protein_coding	chr2	223957463	223967714	+	Human GRCh	OTTHUMG000001	MRPL44	**mitochondrial ribosomal protein L44**	Cytoplasm	enzyme		1.013372937	0	0.95746674	0	1	0	9.22026136	9.5651093	9.52912935	9.29965892
7071	**ENSG0000013591**	TTLL4	protein_coding	chr2	218710835	218755416	+	Human GRCh	OTTHUMG000001	TTLL4	**tubulin tyrosine ligase like 4**	Cytoplasm	enzyme		-1.08112412	0	0.68956484	0	1	0	10.8622869	11.0526914	11.0045464	10.7280825
7072	**ENSG0000013591**	USP37	protein_coding	chr2	218450251	218568351	-	Human GRCh	OTTHUMG000001	USP37	**ubiquitin specific peptidase 37**	Nucleus	peptidase		-1.137542337	0	0.53381028	0	1	0	11.1126879	11.4366699	11.3174791	10.9443761
7074	**ENSG0000013591**	ITM2C	protein_coding	chr2	230864639	230879248	+	Human GRCh	OTTHUMG000001	ITM2C	**integral membrane protein 2C**	Cytoplasm	other		1.440684215	0	0.11860922	0	1	0	9.65076338	9.88662055	10.146593	10.1719944
7075	**ENSG0000013591**	SLC19A3	protein_coding	chr2	227683763	227718028	-	Human GRCh	OTTHUMG000001	SLC19A3	**solute carrier family 19 member 3**	Plasma Mem	transporter		-11.86810879	-1	0.12205485	0	NA	0	0.91683466	1.91097822	0.94288598	0.91867585
7076	**ENSG0000013591**	SERPINE2	protein_coding	chr2	223975045	224039318	-	Human GRCh	OTTHUMG000001	SERPINE2	**serpin family E member 2**	Extracellular	other		-1.702759901	-1	0.12666696	0	1	0	8.48098735	9.13309469	8.42459192	7.98433798
7077	**ENSG0000013592**	DNAJB2	protein_coding	chr2	219279342	219286898	+	Human GRCh	OTTHUMG000001	DNAJB2	**DnaJ heat shock protein family (Hsp40) member B2**	Nucleus	other		1.27773643	0	0.31194943	0	1	0	9.99580196	10.1061278	10.404079	10.2386686
7078	**ENSG0000013592**	WNT10A	protein_coding	chr2	218880852	218899581	+	Human GRCh	OTTHUMG000001	WNT10A	**Wnt family member 10A**	Extracellular	other		3.686970644	1	0.15706113	0	NA	0	2.22591862	2.55533268	2.53996532	3.42236611
7079	**ENSG0000013592**	TMBIM1	protein_coding	chr2	218274197	218292586	-	Human GRCh	OTTHUMG000001	TMBIM1	**transmembrane BAX inhibitor motif containing 1**	Cytoplasm	other		1.065953447	0	0.78191665	0	1	0	8.7338141	8.74836108	8.65824429	8.94291957
7080	**ENSG0000013592**	CYP27A1	protein_coding	chr2	218781749	218815293	+	Human GRCh	OTTHUMG000000	CYP27A1	**cytochrome P450 family 27 subfamily A member 1**	Cytoplasm	enzyme		1.119924857	0	0.84124059	0	1	0	4.22081056	4.47155971	4.68179728	4.14807459
7081	**ENSG0000013593**	EIF4E2	protein_coding	chr2	232550659	232583644	+	Human GRCh	OTTHUMG000001	EIF4E2	**eukaryotic translation initiation factor 4E family member 2**	Cytoplasm	translation re		-1.144824045	0	0.52332688	0	1	0	11.0175959	11.3150949	11.1909655	10.8308828
7082	**ENSG0000013593**	ARMC9	protein_coding	chr2	231198546	231376848	+	Human GRCh	OTTHUMG000001	ARMC9	**armadillo repeat containing 9**	Cytoplasm	other		-1.129301869	0	0.5731404	0	1	0	9.91590726	9.95894609	9.98224477	9.59214338
7083	**ENSG0000013593**	CAB39	protein_coding	chr2	230712842	230821075	+	Human GRCh	OTTHUMG000001	CAB39	**calcium binding protein 39**	Cytoplasm	enzyme		1.10191393	0	0.64708494	0	1	0	10.4068909	10.5809302	10.6842793	10.5178259
7084	**ENSG0000013594**	COX5B	protein_coding	chr2	97646062	97648383	+	Human GRCh	OTTHUMG000001	COX5B	**cytochrome c oxidase subunit 5B**	Cytoplasm	enzyme		-1.062901859	0	0.80198324	0	1	0	11.5613909	11.8852245	11.6982767	11.6304319
7085	**ENSG0000013594**	REV1	protein_coding	chr2	99400475	99490035	-	Human GRCh	OTTHUMG000001	REV1	**REV1 DNA directed polymerase**	Nucleus	enzyme		-1.042615019	0	0.85183213	0	1	0	10.517971	10.8039423	10.8378625	10.3634691
7086	**ENSG0000013595**	TSGA10	protein_coding	chr2	98997261	99154964	-	Human GRCh	OTTHUMG000001	TSGA10	**testis specific 10**	Cytoplasm	other		1.024755974	0	0.92367662	0	1	0	8.42813728	8.51198616	8.65770619	8.31051098
7087	**ENSG0000013595**	MFSD9	protein_coding	chr2	102714630	102736888	-	Human GRCh	OTTHUMG000001	MFSD9	**major facilitator superfamily domain containing 9**	Other	transporter		-1.003054871	0	0.99146249	0	1	0	8.45163076	8.6157169	8.61175313	8.44897849
7088	**ENSG0000013595**	TMEM127	protein_coding	chr2	96248514	96265997	-	Human GRCh	OTTHUMG000001	TMEM127	**transmembrane protein 127**	Cytoplasm	other		-1.116073285	0	0.58873332	0	1	0	10.7194681	10.9186298	10.8470674	10.5352786
7090	**ENSG0000013596**	TGFBRAP1	protein_coding	chr2	105264391	105329735	-	Human GRCh	OTTHUMG000001	TGFBRAP1	**transforming growth factor beta receptor associated protein 1**	Cytoplasm	other		-1.129045705	0	0.57333523	0	1	0	10.1680609	10.5145255	10.3419836	10.0873882
7091	**ENSG0000013596**	GCC2	protein_coding	chr2	108449107	108509415	+	Human GRCh	OTTHUMG000001	GCC2	**GRIP and coiled-coil domain containing 2**	Cytoplasm	other		-1.054984368	0	0.79351183	0	1	0	10.6777938	10.7563091	10.7675454	10.5408397
7092	**ENSG0000013597**	MRPS9	protein_coding	chr2	105038069	105099960	+	Human GRCh	OTTHUMG000001	MRPS9	**mitochondrial ribosomal protein S9**	Cytoplasm	other		-1.028770201	0	0.90474935	0	1	0	9.41102267	9.90607307	9.78601269	9.50171989
7094	**ENSG0000013597**	C2orf49	protein_coding	chr2	105337532	105349211	+	Human GRCh	OTTHUMG000001	C2orf49	**chromosome 2 open reading frame 49**	Nucleus	other		-1.030147895	0	0.89022204	0	1	0	9.5844563	9.75790221	9.8111095	9.44401326
7095	**ENSG0000013597**	ANKRD36	protein_coding	chr2	97113153	97264521	+	Human GRCh	OTTHUMG000001	ANKRD36	**ankyrin repeat domain 36**	Other	other		-1.233244472	0	0.32325771	0	1	0	11.2357892	11.6063589	11.3933309	10.9832198
7096	**ENSG0000013599**	EPC2	protein_coding	chr2	148644440	148787569	+	Human GRCh	OTTHUMG000001	EPC2	**enhancer of polycomb homolog 2**	Nucleus	other		1.106030097	0	0.64057219	0	1	0	9.04723337	9.40998348	9.38545759	9.31031826
7097	**ENSG0000013600**	ARHGEF4	protein_coding	chr2	130836914	131047263	+	Human GRCh	OTTHUMG000001	ARHGEF4	**Rho guanine nucleotide exchange factor 4**	Cytoplasm	other		1.147434842	0	0.67865591	0	1	0	6.88063109	6.71191822	7.31670857	6.38409895
7098	**ENSG0000013600**	ISCU	protein_coding	chr12	108562582	108569368	+	Human GRCh	OTTHUMG000001	ISCU	**iron-sulfur cluster assembly enzyme**	Cytoplasm	other		-1.113451249	0	0.59189337	0	1	0	10.0694797	10.2686513	10.1295165	9.97350719
7099	**ENSG0000013601**	ALDH1L2	protein_coding	chr12	105019784	105107643	-	Human GRCh	OTTHUMG000001	ALDH1L2	**aldehyde dehydrogenase 1 family member L2**	Cytoplasm	enzyme		-1.733311972	-1	0.0944783	0	1	0	8.93012309	8.15206415	7.94321576	8.13932453
7100	**ENSG0000013601**	STAB2	protein_coding	chr12	103587273	103766719	+	Human GRCh	OTTHUMG000001	STAB2	**stabilin 2**	Plasma Mem	transmembra		1	0	1	0	1	0	1.14339935	1.19685009	1.18817065	1.14659963
7102	**ENSG0000013602**	SCYL2	protein_coding	chr12	100267140	100341715	+	Human GRCh	OTTHUMG000001	SCYL2	**SCY1 like pseudokinase 2**	Cytoplasm	other		-1.118850605	0	0.57979864	0	1	0	11.0668274	11.2402976	11.1940851	10.8480991
7103	**ENSG0000013602**	CKAP4	protein_coding	chr12	106237881	106304279	-	Human GRCh	OTTHUMG000001	CKAP4	**cytoskeleton associated protein 4**	Cytoplasm	other		-1.064067651	0	0.76391346	0	1	0	9.70691221	9.73468583	9.769813	9.52087265
7104	**ENSG0000013604**	PLXNC1	protein_coding	chr12	94148577	94307675	+	Human GRCh	OTTHUMG000001	PLXNC1	**plexin C1**	Plasma Mem	transmembra		-1.045219767	0	0.95360378	0	1	0	3.59186651	3.42803664	3.81612269	3.01581945
7105	**ENSG0000013604**	APPL2	protein_coding	chr12	105173297	105236203	-	Human GRCh	OTTHUMG000001	APPL2	**adaptor protein, phosphotyrosine interacting with PH domain**	Cytoplasm	other		-1.144166635	0	0.55310354	0	1	0	9.12374221	9.41029523	9.20341627	9.05578739
7106	**ENSG0000013604**	PWP1	protein_coding	chr12	107685799	107713162	+	Human GRCh	OTTHUMG000001	PWP1	**PWP1 homolog, endonuclein**	Nucleus	other		-1.026614665	0	0.89648774	0	1	0	9.97751353	10.2555073	10.1976634	9.98351189
7107	**ENSG0000013604**	DRAM1	protein_coding	chr12	101877580	102012130	+	Human GRCh	OTTHUMG000001	DRAM1	**DNA damage regulated autophagy modulator 1**	Cytoplasm	other		-1.202754498	0	0.69210641	0	1	0	4.67254312	4.70029255	4.36672508	4.68337823
7108	**ENSG0000013605**	WASHC4	protein_coding	chr12	105107324	105169130	+	Human GRCh	OTTHUMG000001	WASHC4	**WASH complex subunit 4**	Cytoplasm	other		1.054056111	0	0.82132074	0	1	0	10.2465704	10.5462462	10.6590582	10.2279549
7109	**ENSG0000013605**	SLC41A2	protein_coding	chr12	104801801	104958744	-	Human GRCh	OTTHUMG000001	SLC41A2	**solute carrier family 41 member 2**	Plasma Mem	transporter		-1.030404031	0	0.91986682	0	1	0	6.74758261	6.96486966	7.10762014	6.47050688
7110	**ENSG0000013605**	VILL	protein_coding	chr3	37988059	38007188	+	Human GRCh	OTTHUMG000001	VILL	**villin like**	Cytoplasm	other		1.126488213	0	0.88953359	0	1	0	2.57913022	3.90540255	3.24311547	3.74323559
7111	**ENSG0000013606**	FLNB	protein_coding	chr3	58008398	58172251	+	Human GRCh	OTTHUMG000001	FLNB	**filamin B**	Cytoplasm	other		1.503873003	1	0.23660507	0	1	0	11.6539816	11.4979484	11.6904164	12.2501987
7112	**ENSG0000013609**	NEK3	protein_coding	chr13	52132639	52159597	-	Human GRCh	OTTHUMG000000	NEK3	**NIMA related kinase 3**	Nucleus	kinase		-1.280973861	0	0.31929175	0	1	0	8.46593941	8.68710347	8.49377187	8.10098878
7113	**ENSG0000013609**	PCDH8	protein_coding	chr13	52842889	52848641	-	Human GRCh	OTTHUMG000000	PCDH8	**protocadherin 8**	Plasma Mem	other		1.278980904	0	0.5588046	0	1	0	5.91222964	5.79823757	5.9128383	6.2374052
7114	**ENSG0000013610**	VPS36	protein_coding	chr13	52412602	52450634	-	Human GRCh	OTTHUMG000000	VPS36	**vacuolar protein sorting 36 homolog**	Cytoplasm	other		-1.220159398	0	0.3884304	0	1	0	10.7267308	11.0241249	10.7953668	10.5179163
7115	**ENSG0000013610**	RNASEH2B	protein_coding	chr13	50909747	51024120	+	Human GRCh	OTTHUMG000000	RNASEH2B	**ribonuclease H2 subunit B**	Nucleus	other		-1.193744698	0	0.42665639	0	1	0	10.602152	10.8438892	10.6343542	10.4272764
7116	**ENSG0000013610**	CKAP2	protein_coding	chr13	52455429	52476628	+	Human GRCh	OTTHUMG000000	CKAP2	**cytoskeleton associated protein 2**	Cytoplasm	other		-1.106096693	0	0.65772768	0	1	0	11.6236264	11.9093466	11.740575	11.5823668
7118	**ENSG0000013611**	TBC1D4	protein_coding	chr13	75283503	75482169	-	Human GRCh	OTTHUMG000000	TBC1D4	**TBC1 domain family member 4**	Cytoplasm	other		-1.079962593	0	0.73937203	0	1	0	11.9116949	12.1258354	12.068164	11.779
7119	**ENSG0000013611**	THSD1	protein_coding	chr13	52377167	52416373	-	Human GRCh	OTTHUMG000000	THSD1	**thrombospondin type 1 domain containing 1**	Extracellular	other		-1.063321295	0	0.91418852	0	1	0	5.54899511	5.6737679	5.74907646	5.30954147
7120	**ENSG0000013612**	BORA	protein_coding	chr13	72727749	72756198	+	Human GRCh	OTTHUMG000000	BORA	**BORA aurora kinase A activator**	Cytoplasm	other		-1.322129246	0	0.22947897	0	1	0	8.76103161	9.0141874	8.61967689	8.56388683
7121	**ENSG0000013614**	LRCH1	protein_coding	chr13	46553168	46753040	+	Human GRCh	OTTHUMG000000	LRCH1	**leucine rich repeats and calponin homology domain containing**	Cytoplasm	other		1.199464487	0	0.60797359	0	1	0	8.48157606	8.74903505	8.94695699	8.65560794
7122	**ENSG0000013614**	SUCLA2	protein_coding	chr13	47745736	48037968	-	Human GRCh	OTTHUMG000000	SUCLA2	**succinate-CoA ligase ADP-forming subunit beta**	Cytoplasm	enzyme		-1.050133861	0	0.82586172	0	1	0	9.96211656	10.4096088	10.3181843	9.95886004
7123	**ENSG0000013614**	RCBTB1	protein_coding	chr13	49531946	49585558	-	Human GRCh	OTTHUMG000000	RCBTB1	**RCC1 and BTB domain containing protein 1**	Nucleus	other		-1.088558022	0	0.72540121	0	1	0	10.1572012	10.4734608	10.3836425	10.0617359
7124	**ENSG0000013614**	MED4	protein_coding	chr13	48053323	48095131	-	Human GRCh	OTTHUMG000000	MED4	**mediator complex subunit 4**	Nucleus	transcription		1.079662146	0	0.71402356	0	1	0	9.54836169	9.81045534	9.88703542	9.64158793
7125	**ENSG0000013614**	PHF11	protein_coding	chr13	49495610	49528981	+	Human GRCh	OTTHUMG000000	PHF11	**PHD finger protein 11**	Nucleus	other		1.075697684	0	0.77720449	0	1	0	7.17416994	7.56347257	7.60436219	7.29377302
7126	**ENSG0000013615**	COG3	protein_coding	chr13	45464898	45536701	+	Human GRCh	OTTHUMG000000	COG3	**component of oligomeric golgi complex 3**	Cytoplasm	other		-1.088697793	0	0.72831364	0	1	0	10.5610209	10.8327329	10.7929569	10.391226
7127	**ENSG0000013615**	LMO7	protein_coding	chr13	75620434	75859870	+	Human GRCh	OTTHUMG000000	LMO7	**LIM domain 7**	Cytoplasm	enzyme		1.011045394	0	0.9667785	0	1	0	9.5464704	9.79612032	9.84629403	9.50838623
7128	**ENSG0000013615**	SCEL	protein_coding	chr13	77535674	77645263	+	Human GRCh	OTTHUMG000000	SCEL	**sciellin**	Cytoplasm	other		-1.835020719	-1	0.72962701	0	NA	0	1.16269367	1.3747069	0.82463152	1.29901116
7129	**ENSG0000013615**	ITM2B	protein_coding	chr13	48232612	48270357	+	Human GRCh	OTTHUMG000000	ITM2B	**integral membrane protein 2B**	Plasma Mem	other		-1.05767945	0	0.79247381	0	1	0	10.999711	11.2392054	11.1890617	10.9243248
7130	**ENSG0000013615**	SPRY2	protein_coding	chr13	80335976	80341126	-	Human GRCh	OTTHUMG000000	SPRY2	**sprouty RTK signaling antagonist 2**	Plasma Mem	other		-1.138576897	0	0.59333239	0	1	0	9.73160774	10.0143529	9.70833278	9.7494529
7131	**ENSG0000013615**	NUDT15	protein_coding	chr13	48037726	48047221	+	Human GRCh	OTTHUMG000000	NUDT15	**nudix hydrolase 15**	Cytoplasm	phosphatase		-1.112315147	0	0.61223733	0	1	0	8.97348073	9.23474782	9.196116	8.74933472
7133	**ENSG0000013616**	RCBTB2	protein_coding	chr13	48488963	48533256	-	Human GRCh	OTTHUMG000000	RCBTB2	**RCC1 and BTB domain containing protein 2**	Cytoplasm	other		-1.492137474	0	0.38198594	0	1	0	4.55140072	4.63397982	4.30166062	4.23614602
7134	**ENSG0000013616**	LCP1	protein_coding	chr13	46125920	46211871	-	Human GRCh	OTTHUMG000000	LCP1	**lymphocyte cytosolic protein 1**	Cytoplasm	other		-1.183456856	0	0.80334131	0	1	0	7.24186634	7.60014228	7.57718232	6.81471747
7135	**ENSG0000013616**	SETDB2	protein_coding	chr13	49444274	49495003	+	Human GRCh	OTTHUMG000000	SETDB2	**SET domain bifurcated histone lysine methyltransferase 2**	Nucleus	enzyme		1.066484704	0	0.81064115	0	1	0	9.0039354	9.26801823	9.38414856	9.01839821
7136	**ENSG0000013619**	SCRN1	protein_coding	chr7	29920104	29990287	-	Human GRCh	OTTHUMG000000	SCRN1	**secernin 1**	Cytoplasm	other		-1.325251631	0	0.35065874	0	1	0	11.6430562	12.1561547	11.8400456	11.3092959
7137	**ENSG0000013619**	C7orf25	protein_coding	chr7	42909273	42912305	-	Human GRCh	OTTHUMG000001	C7orf25	**chromosome 7 open reading frame 25**	Other	other		1.002521148	0	0.98960605	0	1	0	8.55914688	8.73963059	8.69293503	8.61592354
7138	**ENSG0000013620**	TNS3	protein_coding	chr7	47275154	47582558	-	Human GRCh	OTTHUMG000000	TNS3	**tensin 3**	Plasma Mem	phosphatase		1.061506339	0	0.85764109	0	1	0	10.5572603	10.9976298	10.900326	10.8346876
7140	**ENSG0000013621**	CHST12	protein_coding	chr7	2403588	2448484	+	Human GRCh	OTTHUMG000000	CHST12	**carbohydrate sulfotransferase 12**	Cytoplasm	enzyme		-1.038651986	0	0.875748	0	1	0	9.88408069	10.1144327	10.0058801	9.91818307
7141	**ENSG0000013623**	IGF2BP3	protein_coding	chr7	23310209	23470491	-	Human GRCh	OTTHUMG000001	IGF2BP3	**insulin like growth factor 2 mRNA binding protein 3**	Cytoplasm	translation re		-1.185987382	0	0.42250045	0	1	0	11.1744593	11.4953774	11.2799262	11.013833
7142	**ENSG0000013623**	GPNMB	protein_coding	chr7	23235967	23275108	+	Human GRCh	OTTHUMG000000	GPNMB	**glycoprotein nmb**	Plasma Mem	enzyme	glembatun	1.378785987	0	0.49099606	0	1	0	6.06440013	6.18177567	5.8566748	6.8318503
7143	**ENSG0000013623**	RAPGEF5	protein_coding	chr7	22118236	22357154	-	Human GRCh	OTTHUMG000001	RAPGEF5	**Rap guanine nucleotide exchange factor 5**	Nucleus	other		1.059391413	0	0.84993202	0	1	0	10.4196767	10.9020738	10.8429174	10.6444202
7144	**ENSG0000013623**	RAC1	protein_coding	chr7	6374527	6403967	+	Human GRCh	OTTHUMG000000	RAC1	**Rac family small GTPase 1**	Plasma Mem	enzyme		-1.136951327	0	0.52231664	0	1	0	11.996642	12.2856663	12.0653774	11.949377
7145	**ENSG0000013624**	KDELR2	protein_coding	chr7	6445953	6484190	-	Human GRCh	OTTHUMG000000	KDELR2	**KDEL endoplasmic reticulum protein retention receptor 2**	Cytoplasm	other		-1.020176385	0	0.92704692	0	1	0	11.9487144	12.161635	12.2030922	11.8451372
7146	**ENSG0000013624**	NUP42	protein_coding	chr7	23181841	23201011	+	Human GRCh	OTTHUMG000000	NUP42	**nucleoporin 42**	Nucleus	transporter		-1.032471079	0	0.89995864	0	1	0	9.6181012	9.77605491	9.91458095	9.34697435
7148	**ENSG0000013624**	ZDHHC4	protein_coding	chr7	6577434	6589374	+	Human GRCh	OTTHUMG000000	ZDHHC4	**zinc finger DHHC-type palmitoyltransferase 4**	Cytoplasm	other		-1.271485849	0	0.2069453	0	1	0	9.62084403	9.8479089	9.56141009	9.39289681
7150	**ENSG0000013626**	BZW2	protein_coding	chr7	16646131	16706523	+	Human GRCh	OTTHUMG000001	BZW2	**basic leucine zipper and W2 domains 2**	Cytoplasm	translation re		-1.111644022	0	0.62911016	0	1	0	10.8160153	11.1235459	10.9056657	10.8147296
7151	**ENSG0000013626**	DGKB	protein_coding	chr7	14145049	14974777	-	Human GRCh	OTTHUMG000001	DGKB	**diacylglycerol kinase beta**	Cytoplasm	kinase		1.539923966	1	0.75015373	0	1	0	2.93826977	2.50783581	2.81131988	2.94701421
7152	**ENSG0000013627**	TBRG4	protein_coding	chr7	45100100	45112047	-	Human GRCh	OTTHUMG000001	TBRG4	**transforming growth factor beta regulator 4**	Cytoplasm	other		-1.05990924	0	0.77546093	0	1	0	11.2555377	11.508706	11.4924315	11.1267768

7153	**ENSG0000013627**	DDX56	protein_coding	chr7	44565804	44575051	-	Human GRCh	OTTHUMG000001	DDX56	**DEAD-box helicase 56**	Nucleus	enzyme		-1.061432473	0	0.76714606	0	1	0	11.1537178	11.4915416	11.4057634	11.1116378
7154	**ENSG0000013627**	HUS1	protein_coding	chr7	47963288	47979615	-	Human GRCh	OTTHUMG000001	HUS1	**HUS1 checkpoint clamp component**	Nucleus	kinase		-1.046710751	0	0.84654953	0	1	0	9.62772956	9.859558	9.88858681	9.47346455
7155	**ENSG0000013627**	NACAD	protein_coding	chr7	45080437	45088969	-	Human GRCh	OTTHUMG000001	NACAD	**NAC alpha domain containing**	Other	other		1.487617468	0	0.44078682	0	1	0	3.83732759	4.5193879	4.19417228	4.79894678
7157	**ENSG0000013627**	DBNL	protein_coding	chr7	44044640	44069456	+	Human GRCh	OTTHUMG000001	DBNL	**drebrin like**	Cytoplasm	other		-1.161263909	0	0.46239451	0	1	0	11.7180738	12.0491895	11.8709504	11.5722826
7158	**ENSG0000013628**	CCM2	protein_coding	chr7	44999475	45076469	+	Human GRCh	OTTHUMG000001	CCM2	**CCM2 scaffold protein**	Cytoplasm	other		1.00030169	0	0.99900466	0	1	0	8.87423778	8.89928095	8.97859461	8.78832376
7159	**ENSG0000013628**	MYO1G	protein_coding	chr7	44962662	44979088	-	Human GRCh	OTTHUMG000001	MYO1G	**myosin IG**	Cytoplasm	other		1.260986554	0	0.60869933	0	1	0	4.80229822	4.7507562	5.14490664	4.7790912
7160	**ENSG0000013629**	TTYH3	protein_coding	chr7	2631961	2664802	+	Human GRCh	OTTHUMG000001	TTYH3	**tweety family member 3**	Plasma Mem	ion channel		-1.028944882	0	0.89092804	0	1	0	11.3874923	11.5635275	11.588437	11.2881928
7162	**ENSG0000013630**	CIDEB	protein_coding	chr14	24305187	24311430	-	Human GRCh	OTTHUMG000001	CIDEB	**cell death inducing DFFA like effector b**	Cytoplasm	other		2.362480168	1	0.30310371	0	1	0	3.29631688	3.18868911	3.97902487	4.0618109
7164	**ENSG0000013631**	TTC5	protein_coding	chr14	20256558	20305960	-	Human GRCh	OTTHUMG000000	TTC5	**tetratricopeptide repeat domain 5**	Nucleus	other		-1.113346451	0	0.59903511	0	1	0	9.61948201	9.824162	9.66874786	9.54526589
7165	**ENSG0000013632**	NKX2-8	protein_coding	chr14	36580004	36582614	-	Human GRCh	OTTHUMG000000	NKX2-8	**NK2 homeobox 8**	Nucleus	transcription		1.488104316	0	0.45269421	0	1	0	4.20997276	3.68149871	4.35165922	4.22025002
7166	**ENSG0000013635**	NKX2-1	protein_coding	chr14	36516392	36521149	-	Human GRCh	OTTHUMG000001	NKX2-1	**NK2 homeobox 1**	Nucleus	transcription		1.103892763	0	0.71345937	0	1	0	7.62487808	7.78924549	7.5904088	7.95439811
7167	**ENSG0000013636**	ZFHX2	protein_coding	chr14	23520857	23556192	-	Human GRCh	OTTHUMG000001	ZFHX2	**zinc finger homeobox 2**	Nucleus	transcription		1.159578087	0	0.58453389	0	1	0	8.25991728	8.20880031	8.5660234	8.1835111
7168	**ENSG0000013637**	MTHFS	protein_coding	chr15	79833585	79897379	-	Human GRCh	OTTHUMG000001	MTHFS	**methenyltetrahydrofolate synthetase**	Cytoplasm	enzyme		-1.132259803	0	0.61338499	0	1	0	7.79887011	7.9221774	7.72891935	7.73052402
7169	**ENSG0000013637**	ADAMTS7	protein_coding	chr15	78759206	78811464	-	Human GRCh	OTTHUMG000001	ADAMTS7	**ADAM metallopeptidase with thrombospondin type 1 motif 7**	Extracellular	peptidase		1.297971646	0	0.61239175	0	1	0	4.73376842	4.00401004	4.6000711	4.69408355
7170	**ENSG0000013637**	ABHD17C	protein_coding	chr15	80679684	80755621	+	Human GRCh	OTTHUMG000001	ABHD17C	**abhydrolase domain containing 17C, depalmitoylase**	Plasma Mem	enzyme		1.092768357	0	0.76292315	0	1	0	7.25207291	7.40143466	7.43395128	7.39732698
7171	**ENSG0000013638**	IREB2	protein_coding	chr15	78437431	78501453	+	Human GRCh	OTTHUMG000001	IREB2	**iron responsive element binding protein 2**	Cytoplasm	translation re		-1.069035233	0	0.75055754	0	1	0	11.4347862	11.8143139	11.7343184	11.3679
7172	**ENSG0000013638**	ALPK3	protein_coding	chr15	84817356	84873479	+	Human GRCh	OTTHUMG000001	ALPK3	**alpha kinase 3**	Nucleus	kinase		-1.106878147	0	0.76099124	0	1	0	7.83473641	8.0984297	8.12512326	7.52413431
7173	**ENSG0000013640**	TM6SF1	protein_coding	chr15	83107572	83144854	+	Human GRCh	OTTHUMG000001	TM6SF1	**transmembrane 6 superfamily member 1**	Plasma Mem	other		3.811215624	1	0.22791997	0	1	0	2.77769601	2.32994368	3.65167291	2.24460835
7174	**ENSG0000013642**	CIB2	protein_coding	chr15	78104606	78131535	-	Human GRCh	OTTHUMG000001	CIB2	**calcium and integrin binding family member 2**	Cytoplasm	kinase		-1.50709598	-1	0.40187477	0	1	0	5.15898909	5.32516361	4.66921367	5.16909484
7175	**ENSG0000013643**	CALCOCO2	protein_coding	chr17	48831018	48866522	+	Human GRCh	OTTHUMG000001	CALCOCO2	**calcium binding and coiled-coil domain 2**	Nucleus	other		-1.207532717	0	0.40017205	0	1	0	10.9384621	11.2994066	11.022217	10.8095302
7176	**ENSG0000013644**	RSAD1	protein_coding	chr17	50478860	50485974	+	Human GRCh	OTTHUMG000001	RSAD1	**radical S-adenosyl methionine domain containing 1**	Cytoplasm	other		-1.104092128	0	0.63409095	0	1	0	9.79100022	10.080332	10.0223454	9.60893849
7177	**ENSG0000013644**	NMT1	protein_coding	chr17	44957992	45109016	+	Human GRCh	OTTHUMG000001	NMT1	**N-myristoyltransferase 1**	Cytoplasm	enzyme	PCLX-001	-1.166390062	0	0.38627962	0	1	0	11.2022665	11.4987046	11.261902	11.1116503
7178	**ENSG0000013644**	MYCBPAP	protein_coding	chr17	50508425	50531501	+	Human GRCh	OTTHUMG000001	MYCBPAP	**MYCBP associated protein**	Nucleus	other		1.705002342	1	0.37221687	0	1	0	4.40103342	3.78044479	4.68707115	4.44699888
7179	**ENSG0000013645**	SRSF1	protein_coding	chr17	58000919	58007346	-	Human GRCh	OTTHUMG000001	SRSF1	**serine and arginine rich splicing factor 1**	Nucleus	other		-1.01457077	0	0.93878116	0	1	0	12.8310178	13.1179624	13.0885709	12.8318698
7180	**ENSG0000013645**	VEZF1	protein_coding	chr17	57971547	57988259	-	Human GRCh	OTTHUMG000001	VEZF1	**vascular endothelial zinc finger 1**	Nucleus	transcription		-1.11984775	0	0.59750786	0	1	0	11.0871837	11.2206351	11.1104371	10.9569
7182	**ENSG0000013646**	TACO1	protein_coding	chr17	63600882	63608365	+	Human GRCh	OTTHUMG000001	TACO1	**translational activator of cytochrome c oxidase I**	Cytoplasm	other		-1.067235268	0	0.80276896	0	1	0	8.84623177	9.1522824	9.06773666	8.78880574
7183	**ENSG0000013647**	TEX2	protein_coding	chr17	64147227	64263260	-	Human GRCh	OTTHUMG000001	TEX2	**testis expressed 2**	Other	other		-1.070218714	0	0.75251467	0	1	0	10.8343711	11.1548319	11.0736195	10.7652042
7184	**ENSG0000013648**	DCAF7	protein_coding	chr17	63550222	63594286	+	Human GRCh	OTTHUMG000001	DCAF7	**DDB1 and CUL4 associated factor 7**	Cytoplasm	other		-1.105470469	0	0.63181139	0	1	0	12.1624302	12.4302352	12.3155962	12.0563941
7187	**ENSG0000013649**	LIMD2	protein_coding	chr17	63695888	63701172	-	Human GRCh	OTTHUMG000001	LIMD2	**LIM domain containing 2**	Other	other		1.380966769	0	0.24498135	0	1	0	6.55702903	6.6910456	6.9648946	6.93580794
7188	**ENSG0000013649**	BRIP1	protein_coding	chr17	61679139	61863559	-	Human GRCh	OTTHUMG000001	BRIP1	**BRCA1 interacting helicase 1**	Nucleus	enzyme		-1.232001249	0	0.38862939	0	1	0	11.217517	11.5101302	11.3346206	10.9113226
7189	**ENSG0000013650**	KAT7	protein_coding	chr17	49788624	49835026	+	Human GRCh	OTTHUMG000001	KAT7	**lysine acetyltransferase 7**	Nucleus	enzyme		-1.375128227	0	0.12645184	0	1	0	11.7825375	12.0452182	11.6829963	11.4388592
7190	**ENSG0000013651**	RTP4	protein_coding	chr3	187368385	187372076	+	Human GRCh	OTTHUMG000001	RTP4	**receptor transporter protein 4**	Plasma Mem	other		1	0	1	0	NA	0	0.97851479	0.99345903	0.9909629	0.97938087
7191	**ENSG0000013651**	ACTL6A	protein_coding	chr3	179562886	179588407	+	Human GRCh	OTTHUMG000001	ACTL6A	**actin like 6A**	Nucleus	other		-1.153927771	0	0.50388125	0	1	0	10.5919417	11.0244421	10.8097217	10.5115192
7192	**ENSG0000013652**	NDUFB5	protein_coding	chr3	179604690	179627647	+	Human GRCh	OTTHUMG000001	NDUFB5	**NADH:ubiquinone oxidoreductase subunit B5**	Cytoplasm	enzyme		1.066059521	0	0.77859662	0	1	0	10.4295832	10.7152107	10.8282947	10.4446079
7193	**ENSG0000013652**	MRPL47	protein_coding	chr3	179588285	179604649	-	Human GRCh	OTTHUMG000001	MRPL47	**mitochondrial ribosomal protein L47**	Cytoplasm	other		-1.015715481	0	0.946179	0	1	0	9.58077054	9.97924303	9.94984872	9.58004145
7194	**ENSG0000013652**	TRA2B	protein_coding	chr3	185914558	185938103	-	Human GRCh	OTTHUMG000001	TRA2B	**transformer 2 beta homolog**	Nucleus	other		1.002755294	0	0.98942968	0	1	0	12.532264	12.6533158	12.7653021	12.4053766
7195	**ENSG0000013653**	SCN2A	protein_coding	chr2	165194993	165392310	+	Human GRCh	OTTHUMG000000	SCN2A	**sodium voltage-gated channel alpha subunit 2**	Plasma Mem	ion channel	riluzole, zo	1.578150424	1	0.44089881	0	1	0	4.63357124	4.73734568	5.30543853	4.8615016
7196	**ENSG0000013653**	TBR1	protein_coding	chr2	161416297	161425870	+	Human GRCh	OTTHUMG000001	TBR1	**T-box brain transcription factor 1**	Nucleus	transcription		1.495057197	0	0.64485229	0	NA	0	2.9512706	2.42709024	2.62426817	3.25602038
7197	**ENSG0000013653**	MARCHF7	protein_coding	chr2	159712457	159771027	+	Human GRCh	OTTHUMG000001	MARCHF7	**membrane associated ring-CH-type finger 7**	Extracellular	other		1.001227448	0	0.99687777	0	1	0	10.4621122	10.7210547	10.7852493	10.380217
7199	**ENSG0000013654**	GALNT5	protein_coding	chr2	157257705	157318491	+	Human GRCh	OTTHUMG000001	GALNT5	**polypeptide N-acetylgalactosaminyltransferase 5**	Cytoplasm	enzyme		-1.378568747	0	0.81731455	0	1	0	1.23516416	2.98151677	2.71745807	1.23923928
7201	**ENSG0000013656**	TANK	protein_coding	chr2	161136908	161236230	+	Human GRCh	OTTHUMG000001	TANK	**TRAF family member associated NFKB activator**	Cytoplasm	other		-1.021702277	0	0.9336748	0	1	0	7.92364545	8.50574821	8.49573181	7.87878037
7202	**ENSG0000013657**	BLK	protein_coding	chr8	11486894	11564599	+	Human GRCh	OTTHUMG000000	BLK	**BLK proto-oncogene, Src family tyrosine kinase**	Cytoplasm	kinase	osimertinil	5.259833714	1	0.22429399	0	1	0	1.1559535	1.21808806	1.20804362	1.81151019
7204	**ENSG0000013660**	SKIL	protein_coding	chr3	170357342	170396835	+	Human GRCh	OTTHUMG000001	SKIL	**SKI like proto-oncogene**	Nucleus	transcription		-1.112378846	0	0.61509682	0	1	0	10.4116047	10.5929306	10.5450465	10.2150225
7205	**ENSG0000013662**	EPRS1	protein_coding	chr1	219968600	220046530	-	Human GRCh	OTTHUMG000000	EPRS1	**glutamyl-prolyl-tRNA synthetase 1**	Cytoplasm	enzyme		-1.129452733	0	0.55458402	0	1	0	13.1456692	13.4979699	13.3364064	13.0381673
7206	**ENSG0000013663**	HLX	protein_coding	chr1	220879431	220885059	+	Human GRCh	OTTHUMG000000	HLX	**H2.0 like homeobox**	Nucleus	transcription		-1.413334067	0	0.31860859	0	1	0	6.78169469	7.24139636	6.71907247	6.59792544
7207	**ENSG0000013663**	VPS45	protein_coding	chr1	150067279	150145329	+	Human GRCh	OTTHUMG000000	VPS45	**vacuolar protein sorting 45 homolog**	Cytoplasm	transporter		-1.082484991	0	0.69783003	0	1	0	11.0366638	11.3238947	11.2503017	10.9318525
7209	**ENSG0000013663**	KCTD3	protein_coding	chr1	215567304	215621807	+	Human GRCh	OTTHUMG000000	KCTD3	**potassium channel tetramerization domain containing 3**	Plasma Mem	ion channel		-1.295539358	0	0.19876888	0	1	0	12.1228199	12.4505666	12.0780942	11.9217709
7210	**ENSG0000013664**	RPS6KC1	protein_coding	chr1	213051233	213274774	+	Human GRCh	OTTHUMG000000	RPS6KC1	**ribosomal protein S6 kinase C1**	Cytoplasm	kinase		1.064709799	0	0.77173559	0	1	0	9.90421156	10.1596142	10.2866508	9.89492017
7211	**ENSG0000013668**	ZNG1B	protein_coding	chr2	113437691	113496204	+	Human GRCh	OTTHUMG000001	ZNG1B	**Zn regulated GTPase metalloprotein activator 1B**	Nucleus	other		1.073605283	0	0.76240043	0	1	0	8.80050488	9.35593683	9.37341204	8.96880204
7219	**ENSG0000013669**	SMPD4	protein_coding	chr2	130151392	130182750	-	Human GRCh	OTTHUMG000001	SMPD4	**sphingomyelin phosphodiesterase 4**	Cytoplasm	enzyme		1.029168991	0	0.88749358	0	1	0	11.0724704	11.4080374	11.4092116	11.14348
7220	**ENSG0000013670**	WDR33	protein_coding	chr2	127701027	127811187	-	Human GRCh	OTTHUMG000001	WDR33	**WD repeat domain 33**	Nucleus	other		-1.074665975	0	0.70787208	0	1	0	10.9672258	11.3328862	11.1936578	10.9640169
7221	**ENSG0000013671**	CCDC115	protein_coding	chr2	130337933	130342699	-	Human GRCh	OTTHUMG000001	CCDC115	**coiled-coil domain containing 115**	Cytoplasm	other		1.193197096	0	0.43779914	0	1	0	8.30389324	8.53492143	8.76707246	8.44687053
7222	**ENSG0000013671**	SAP130	protein_coding	chr2	127941217	128028120	-	Human GRCh	OTTHUMG000001	SAP130	**Sin3A associated protein 130**	Nucleus	transcription		-1.220452165	0	0.33941138	0	1	0	10.0137059	10.419837	9.96268494	10.0646829
7223	**ENSG0000013671**	BIN1	protein_coding	chr2	127048027	127107288	-	Human GRCh	OTTHUMG000001	BIN1	**bridging integrator 1**	Nucleus	other		1.036131899	0	0.89973987	0	1	0	8.26984875	8.5526048	8.52812072	8.38377341
7224	**ENSG0000013671**	IMP4	protein_coding	chr2	130342877	130347967	+	Human GRCh	OTTHUMG000001	IMP4	**IMP U3 small nucleolar ribonucleoprotein 4**	Nucleus	other		1.022969665	0	0.91374892	0	1	0	9.87985538	10.0763375	10.1684742	9.82126535
7225	**ENSG0000013672**	HS6ST1	protein_coding	chr2	128236716	128318868	-	Human GRCh	OTTHUMG000001	HS6ST1	**heparan sulfate 6-O-sulfotransferase 1**	Plasma Mem	enzyme		1.10101708	0	0.71446273	0	1	0	8.91796985	9.02496012	9.2224649	8.9008244
7226	**ENSG0000013673**	UGGT1	protein_coding	chr2	128091200	128195677	+	Human GRCh	OTTHUMG000001	UGGT1	**UDP-glucose glycoprotein glucosyltransferase 1**	Cytoplasm	enzyme		-1.233875738	0	0.27067964	0	1	0	11.4103231	11.685741	11.4143746	11.2267842
7228	**ENSG0000013673**	STAM	protein_coding	chr10	17644151	17716824	+	Human GRCh	OTTHUMG000000	STAM	**signal transducing adaptor molecule**	Cytoplasm	other		-1.099473005	0	0.63703862	0	1	0	9.78980003	9.974969	9.96323718	9.56808173
7230	**ENSG0000013675**	ABI1	protein_coding	chr10	26746593	26861087	-	Human GRCh	OTTHUMG000000	ABI1	**abl interactor 1**	Cytoplasm	other		-1.118723464	0	0.61026744	0	1	0	10.2667394	10.6262191	10.4179259	10.2508161
7231	**ENSG0000013675**	YME1L1	protein_coding	chr10	27110111	27155266	-	Human GRCh	OTTHUMG000000	YME1L1	**YME1 like 1 ATPase**	Cytoplasm	peptidase		-1.05300149	0	0.79313552	0	1	0	12.0679101	12.341956	12.2838678	12.0110058
7232	**ENSG0000013677**	DNAJC1	protein_coding	chr10	21756548	22003769	-	Human GRCh	OTTHUMG000000	DNAJC1	**DnaJ heat shock protein family (Hsp40) member C1**	Cytoplasm	other		1.031782521	0	0.88890007	0	1	0	8.98294912	9.18685451	9.1203861	9.12545701
7233	**ENSG0000013678**	NIPSNAP3A	protein_coding	chr9	104747683	104760120	+	Human GRCh	OTTHUMG000000	NIPSNAP3A	**nipsnap homolog 3A**	Nucleus	other		1.139337979	0	0.60339206	0	1	0	8.67830562	9.00850714	9.25316193	8.66445688
7234	**ENSG0000013680**	LRRC8A	protein_coding	chr9	128882133	128918039	+	Human GRCh	OTTHUMG000000	LRRC8A	**leucine rich repeat containing 8 VRAC subunit A**	Plasma Mem	ion channel		-1.336456361	0	0.212553	0	1	0	13.8374171	14.0266358	13.6577402	13.5320872
7235	**ENSG0000013680**	CDK9	protein_coding	chr9	127785679	127790792	+	Human GRCh	OTTHUMG000000	CDK9	**cyclin dependent kinase 9**	Nucleus	kinase	AZD5438,	-1.257473461	0	0.28891346	0	1	0	10.1080557	10.4773008	10.0735707	10.0341353
7236	**ENSG0000013681**	TXN	protein_coding	chr9	110243810	110256507	-	Human GRCh	OTTHUMG000000	TXN	**thioredoxin**	Cytoplasm	enzyme	PX-12	-1.016131866	0	0.94446053	0	1	0	10.4562207	10.741016	10.7428093	10.4129558
7237	**ENSG0000013681**	ODF2	protein_coding	chr9	128455186	128501292	+	Human GRCh	OTTHUMG000000	ODF2	**outer dense fiber of sperm tails 2**	Cytoplasm	other		-1.053124461	0	0.7978971	0	1	0	11.3084388	11.6408691	11.5196106	11.329532
7238	**ENSG0000013681**	ECPAS	protein_coding	chr9	111360685	111484745	-	Human GRCh	OTTHUMG000000	ECPAS	**Ecm29 proteasome adaptor and scaffold**	Cytoplasm	other		-1.222376349	0	0.44438425	0	1	0	12.4578211	12.8142829	12.5533485	12.2838361
7239	**ENSG0000013681**	TOR1B	protein_coding	chr9	129803157	129811281	+	Human GRCh	OTTHUMG000000	TOR1B	**torsin family 1 member B**	Cytoplasm	enzyme		1.092098222	0	0.69056059	0	1	0	8.81976961	8.77720978	9.06614345	8.69280923
7240	**ENSG0000013681**	C9orf78	protein_coding	chr9	129827290	129835863	-	Human GRCh	OTTHUMG000000	C9orf78	**chromosome 9 open reading frame 78**	Nucleus	other		1.020499165	0	0.91607667	0	1	0	9.71161076	10.0186065	9.97207202	9.81737789
7241	**ENSG0000013682**	SMC2	protein_coding	chr9	104094260	104141419	+	Human GRCh	OTTHUMG000000	SMC2	**structural maintenance of chromosomes 2**	Nucleus	transporter		-1.165211972	0	0.49786	0	1	0	11.2600256	11.7419419	11.4921616	11.214052
7242	**ENSG0000013682**	KLF4	protein_coding	chr9	107484852	107490482	-	Human GRCh	OTTHUMG000000	KLF4	**KLF transcription factor 4**	Nucleus	transcription		1.170850178	0	0.52925757	0	1	0	10.2036592	10.4146158	10.5697131	10.4173285
7243	**ENSG0000013682**	TOR1A	protein_coding	chr9	129812942	129824244	-	Human GRCh	OTTHUMG000000	TOR1A	**torsin family 1 member A**	Cytoplasm	enzyme		-1.190396017	0	0.40792103	0	1	0	9.66046722	10.0577868	9.87372206	9.46557693
7244	**ENSG0000013682**	RALGPS1	protein_coding	chr9	126914774	127223166	+	Human GRCh	OTTHUMG000000	RALGPS1	**Ral GEF with PH domain and SH3 binding motif 1**	Cytoplasm	other		1.048820246	0	0.83956425	0	1	0	8.82853551	9.16471708	9.05924427	9.05711493
7245	**ENSG0000013683**	NIBAN2	protein_coding	chr9	127505339	127578989	-	Human GRCh	OTTHUMG000000	NIBAN2	**niban apoptosis regulator 2**	Cytoplasm	transcription		1.010136095	0	0.96381883	0	1	0	12.4195276	12.7199948	12.6440308	12.5343857
7248	**ENSG0000013684**	ST6GALNA	protein_coding	chr9	127907886	127917041	-	Human GRCh	OTTHUMG000000	ST6GALNAC4	**ST6 N-acetylgalactosaminide alpha-2,6-sialyltransferase 4**	Cytoplasm	enzyme		1.24330507	0	0.55100298	0	1	0	7.30944247	7.51146071	7.68468911	7.58029376
7249	**ENSG0000013684**	TMOD1	protein_coding	chr9	97501180	97601743	+	Human GRCh	OTTHUMG000000	TMOD1	**tropomodulin 1**	Cytoplasm	enzyme		-1.224154646	0	0.71148251	0	1	0	4.12009269	3.80820608	4.09689985	3.54702351
7250	**ENSG0000013684**	DAB2IP	protein_coding	chr9	121567057	121785530	+	Human GRCh	OTTHUMG000000	DAB2IP	**DAB2 interacting protein**	Plasma Mem	other		1.025883314	0	0.91013283	0	1	0	9.16330478	9.32206677	9.0612432	9.44808064
7251	**ENSG0000013685**	STXBP1	protein_coding	chr9	127579370	127696027	+	Human GRCh	OTTHUMG000000	STXBP1	**syntaxin binding protein 1**	Cytoplasm	transporter		1.198733374	0	0.41526917	0	1	0	9.78954892	10.0420209	10.220283	9.99548599
7252	**ENSG0000013685**	SLC2A8	protein_coding	chr9	127397138	127408424	+	Human GRCh	OTTHUMG000000	SLC2A8	**solute carrier family 2 member 8**	Plasma Mem	transporter		1.055957486	0	0.82890143	0	1	0	9.36224464	9.71308355	9.80183017	9.38361731
7253	**ENSG0000013685**	ANGPTL2	protein_coding	chr9	127087348	127122635	-	Human GRCh	OTTHUMG000000	ANGPTL2	**angiopoietin like 2**	Extracellular	other		5.655094249	1	0.31405972	0	1	0	2.5267566	2.62697421	3.15248868	2.53281299
7254	**ENSG0000013686**	CDK5RAP2	protein_coding	chr9	120388875	120580170	-	Human GRCh	OTTHUMG000000	CDK5RAP2	**CDK5 regulatory subunit associated protein 2**	Cytoplasm	other		-1.110356799	0	0.59173387	0	1	0	12.1538037	12.4677975	12.3200375	12.0863242
7255	**ENSG0000013686**	ZFP37	protein_coding	chr9	113038377	113056759	-	Human GRCh	OTTHUMG000000	ZFP37	**ZFP37 zinc finger protein**	Nucleus	transcription		-1.095737692	0	0.76455422	0	1	0	7.22509616	7.68440204	7.6153525	7.06938143
7256	**ENSG0000013686**	SLC31A2	protein_coding	chr9	113150976	113164140	+	Human GRCh	OTTHUMG000000	SLC31A2	**solute carrier family 31 member 2**	Plasma Mem	transporter		-1.073827268	0	0.82276514	0	1	0	6.78089092	7.31762575	7.07725327	6.92370739

7257	**ENSG0000013686**	SLC31A1	protein_coding	chr9	113221544	113264492	+	Human GRCh	OTTHUMG000000	SLC31A1	**solute carrier family 31 member 1**	Plasma Mem	transporter		-1.083242743	0	0.72532688	0	1	0	10.5467469	11.0633669	10.8575448	10.614659
7258	**ENSG0000013686**	TLR4	protein_coding	chr9	117704175	117724735	+	Human GRCh	OTTHUMG000000	TLR4	**toll like receptor 4**	Plasma Mem	transmembra	eritoran, re	1.270451932	0	0.67998612	0	1	0	5.48233126	6.2146766	6.56888892	5.51323438
7259	**ENSG0000013687**	ZNF189	protein_coding	chr9	101398851	101410660	+	Human GRCh	OTTHUMG000000	ZNF189	**zinc finger protein 189**	Nucleus	transcription		1.035921182	0	0.89305078	0	1	0	9.03435956	9.28534047	9.27798713	9.12701308
7261	**ENSG0000013687**	STX17	protein_coding	chr9	99906654	99974534	+	Human GRCh	OTTHUMG000000	STX17	**syntaxin 17**	Plasma Mem	transporter		-1.119408216	0	0.5867774	0	1	0	10.199006	10.4455265	10.3166565	10.0860538
7262	**ENSG0000013687**	PRPF4	protein_coding	chr9	113275642	113294009	+	Human GRCh	OTTHUMG000000	PRPF4	**pre-mRNA processing factor 4**	Nucleus	other		1.196497573	0	0.34763766	0	1	0	9.95863651	10.3246697	10.4767738	10.2131851
7263	**ENSG0000013687**	FPGS	protein_coding	chr9	127794597	127814327	+	Human GRCh	OTTHUMG000000	FPGS	**folylpolyglutamate synthase**	Cytoplasm	enzyme	pralatrexat	-1.043005977	0	0.84225443	0	1	0	10.5714656	10.8929098	10.7045313	10.6871264
7264	**ENSG0000013687**	USP20	protein_coding	chr9	129834698	129881828	+	Human GRCh	OTTHUMG000000	USP20	**ubiquitin specific peptidase 20**	Cytoplasm	peptidase		-1.078862501	0	0.72452949	0	1	0	10.0009851	10.1998799	10.1859991	9.8306993
7265	**ENSG0000013688**	BAAT	protein_coding	chr9	101354182	101385400	-	Human GRCh	OTTHUMG000000	BAAT	**bile acid-CoA:amino acid N-acyltransferase**	Cytoplasm	enzyme		-1.74171764	-1	0.20780613	0	1	0	5.2221669	5.47744576	5.10586201	4.50672189
7266	**ENSG0000013688**	KIF12	protein_coding	chr9	114086126	114099292	-	Human GRCh	OTTHUMG000000	KIF12	**kinesin family member 12**	Cytoplasm	other		-1.495036237	0	0.32078741	0	1	0	4.5682173	4.76787328	4.62759478	3.90107564
7267	**ENSG0000013688**	ATP6V1G1	protein_coding	chr9	114587706	114598915	+	Human GRCh	OTTHUMG000000	ATP6V1G1	**ATPase H+ transporting V1 subunit G1**	Cytoplasm	transporter		1.103954985	0	0.62288342	0	1	0	10.4615137	10.6036175	10.8072253	10.4535091
7268	**ENSG0000013689**	TEX10	protein_coding	chr9	100302077	100352942	-	Human GRCh	OTTHUMG000000	TEX10	**testis expressed 10**	Nucleus	other		-1.142473219	0	0.53646507	0	1	0	10.0844282	10.3927996	10.2662739	9.91047553
7269	**ENSG0000013689**	GARNL3	protein_coding	chr9	127224265	127393660	+	Human GRCh	OTTHUMG000000	GARNL3	**GTPase activating Rap/RanGAP domain like 3**	Other	other		1.080771656	0	0.73636836	0	1	0	8.82500702	8.95701398	9.19755306	8.70470629
7270	**ENSG0000013689**	MRPL50	protein_coding	chr9	101387633	101398618	-	Human GRCh	OTTHUMG000000	MRPL50	**mitochondrial ribosomal protein L50**	Cytoplasm	other		1.04093962	0	0.85826209	0	1	0	9.28782161	9.69553598	9.67810071	9.41410362
7271	**ENSG0000013690**	DPM2	protein_coding	chr9	127935099	127937854	-	Human GRCh	OTTHUMG000000	DPM2	**dolichyl-phosphate mannosyltransferase subunit 2, regulatory**	Cytoplasm	enzyme		-1.026293642	0	0.88520014	0	1	0	10.0612714	10.2784969	10.1583996	10.1333411
7273	**ENSG0000013692**	TSTD2	protein_coding	chr9	97600080	97633368	-	Human GRCh	OTTHUMG000000	TSTD2	**thiosulfate sulfurtransferase like domain containing 2**	Other	other		-1.15367606	0	0.51863729	0	1	0	9.69069705	9.85901289	9.73934393	9.49290385
7274	**ENSG0000013692**	GABBR2	protein_coding	chr9	98288109	98708935	-	Human GRCh	OTTHUMG000000	GABBR2	**gamma-aminobutyric acid type B receptor subunit 2**	Plasma Mem	G-protein cou	amitriptyli	1.75840342	1	0.37006129	0	1	0	2.67939228	3.60773072	3.11887557	3.94948311
7276	**ENSG0000013693**	PSMB7	protein_coding	chr9	124353465	124415444	-	Human GRCh	OTTHUMG000000	PSMB7	**proteasome 20S subunit beta 7**	Cytoplasm	peptidase		-1.103751187	0	0.63890062	0	1	0	10.8278534	11.2947395	11.0649464	10.8751545
7277	**ENSG0000013693**	NR5A1	protein_coding	chr9	124481236	124507420	-	Human GRCh	OTTHUMG000000	NR5A1	**nuclear receptor subfamily 5 group A member 1**	Nucleus	ligand-depen		-1.198210652	0	0.79074671	0	1	0	3.29664308	3.32011418	2.72546691	3.44749865
7278	**ENSG0000013693**	TRMO	protein_coding	chr9	97904489	97922500	-	Human GRCh	OTTHUMG000000	TRMO	**tRNA methyltransferase O**	Other	enzyme		-1.108535499	0	0.67984418	0	1	0	7.69377709	7.59197995	7.66057511	7.40006822
7279	**ENSG0000013693**	RABEPK	protein_coding	chr9	125200542	125234161	+	Human GRCh	OTTHUMG000000	RABEPK	**Rab9 effector protein with kelch motifs**	Cytoplasm	other		1.117035118	0	0.60673061	0	1	0	8.65841418	8.8030423	9.02712007	8.65500276
7280	**ENSG0000013693**	GOLGA1	protein_coding	chr9	124878275	124948492	-	Human GRCh	OTTHUMG000000	GOLGA1	**golgin A1**	Cytoplasm	other		-1.127193747	0	0.58414156	0	1	0	9.69990936	10.2314181	9.97960973	9.73364736
7281	**ENSG0000013693**	XPA	protein_coding	chr9	97674909	97697340	-	Human GRCh	OTTHUMG000000	XPA	**XPA, DNA damage recognition and repair factor**	Nucleus	other		1.058358608	0	0.84204386	0	1	0	7.49811259	7.68742085	7.9098266	7.33321224
7282	**ENSG0000013693**	NCBP1	protein_coding	chr9	97633668	97673748	+	Human GRCh	OTTHUMG000000	NCBP1	**nuclear cap binding protein subunit 1**	Nucleus	other		-1.155448538	0	0.45101654	0	1	0	10.9675197	11.2124105	11.0862473	10.7681083
7283	**ENSG0000013693**	ANP32B	protein_coding	chr9	97983341	98015943	+	Human GRCh	OTTHUMG000000	ANP32B	**acidic nuclear phosphoprotein 32 family member B**	Nucleus	other		1.080397336	0	0.65312341	0	1	0	11.4118138	11.7197718	11.6980415	11.6215064
7285	**ENSG0000013694**	PDCL	protein_coding	chr9	122798389	122828588	-	Human GRCh	OTTHUMG000000	PDCL	**phosducin like**	Cytoplasm	enzyme		1.035655672	0	0.8666287	0	1	0	8.98833883	9.25011985	9.16265636	9.16148431
7286	**ENSG0000013694**	RPL35	protein_coding	chr9	124857880	124861981	-	Human GRCh	OTTHUMG000000	RPL35	**ribosomal protein L35**	Cytoplasm	other		1.030956142	0	0.86074761	0	1	0	12.5626812	12.8179251	12.782682	12.6776121
7287	**ENSG0000013694**	CTSV	protein_coding	chr9	97029677	97156556	-	Human GRCh	OTTHUMG000000	CTSV	**cathepsin V**	Cytoplasm	peptidase		-1.064430296	0	0.81595031	0	1	0	6.63605381	6.91258746	6.83514926	6.59156819
7288	**ENSG0000013694**	LMX1B	protein_coding	chr9	126613928	126701032	+	Human GRCh	OTTHUMG000000	LMX1B	**LIM homeobox transcription factor 1 beta**	Nucleus	transcription		1.148889467	0	0.85093306	0	1	0	3.49383179	3.66120284	4.22779663	2.56391983
7289	**ENSG0000013695**	ARPC5L	protein_coding	chr9	124862130	124877733	+	Human GRCh	OTTHUMG000000	ARPC5L	**actin related protein 2/3 complex subunit 5 like**	Cytoplasm	other		-1.074175426	0	0.71277433	0	1	0	9.70918829	10.1011548	9.99467104	9.66719689
7290	**ENSG0000013696**	ENPP2	protein_coding	chr8	119557086	119673453	-	Human GRCh	OTTHUMG000001	ENPP2	**ectonucleotide pyrophosphatase/phosphodiesterase 2**	Plasma Mem	enzyme	VPC8a202	-1.291049448	0	0.81191027	0	1	0	2.41480612	1.69085098	1.9697318	2.11650331
7291	**ENSG0000013698**	DSCC1	protein_coding	chr8	119833976	119855894	-	Human GRCh	OTTHUMG000001	DSCC1	**DNA replication and sister chromatid cohesion 1**	Nucleus	other		-1.05584919	0	0.82040452	0	1	0	8.84482058	9.05387508	9.06925613	8.69314154
7292	**ENSG0000013698**	DERL1	protein_coding	chr8	123013170	123042302	-	Human GRCh	OTTHUMG000001	DERL1	**derlin 1**	Cytoplasm	other		-1.069610427	0	0.74737869	0	1	0	10.6512608	10.9371037	10.877665	10.5596692
7293	**ENSG0000013699**	MYC	protein_coding	chr8	127735434	127742951	+	Human GRCh	OTTHUMG000001	MYC	**MYC proto-oncogene, bHLH transcription factor**	Nucleus	transcription	OMO-103,	-1.103890227	0	0.68041453	0	1	0	12.8778993	13.089596	12.882994	12.862051
7294	**ENSG0000013699**	CCN3	protein_coding	chr8	119416446	119424434	+	Human GRCh	OTTHUMG000001	CCN3	**cellular communication network factor 3**	Extracellular	growth facto		1.185131016	0	0.72925717	0	1	0	4.60709773	4.28993777	4.09939859	4.95696367
7296	**ENSG0000013703**	DMAC1	protein_coding	chr9	7796500	7888380	-	Human GRCh	OTTHUMG000000	DMAC1	**distal membrane arm assembly component 1**	Cytoplasm	other		-1.041249767	0	0.85841868	0	1	0	9.32406982	9.50107249	9.48257491	9.24839798
7297	**ENSG0000013704**	RANBP6	protein_coding	chr9	6011025	6015625	-	Human GRCh	OTTHUMG000000	RANBP6	**RAN binding protein 6**	Cytoplasm	other		1.027428535	0	0.8989886	0	1	0	9.91582733	10.1699466	10.2395883	9.89472353
7298	**ENSG0000013705**	POLR1E	protein_coding	chr9	37485948	37503697	+	Human GRCh	OTTHUMG000000	POLR1E	**RNA polymerase I subunit E**	Nucleus	enzyme		-1.093815473	0	0.66285165	0	1	0	9.76228865	10.1836743	9.98347286	9.78901492
7299	**ENSG0000013705**	PLAA	protein_coding	chr9	26903372	26947242	-	Human GRCh	OTTHUMG000000	PLAA	**phospholipase A2 activating protein**	Cytoplasm	other		-1.069848027	0	0.7484459	0	1	0	10.282221	10.6341308	10.5610579	10.2050637
7300	**ENSG0000013707**	IL11RA	protein_coding	chr9	34652162	34661902	+	Human GRCh	OTTHUMG000000	IL11RA	**interleukin 11 receptor subunit alpha**	Plasma Mem	transmembra	oprelvekin	-1.02137463	0	0.94238703	0	1	0	8.31057209	8.89806632	8.69484831	8.53314719
7301	**ENSG0000013707**	UBAP2	protein_coding	chr9	33921693	34049388	-	Human GRCh	OTTHUMG000000	UBAP2	**ubiquitin associated protein 2**	Cytoplasm	other		-1.069347956	0	0.73046282	0	1	0	12.4715579	12.7641387	12.6852728	12.4005897
7302	**ENSG0000013707**	APTX	protein_coding	chr9	32886601	33025130	-	Human GRCh	OTTHUMG000000	APTX	**aprataxin**	Nucleus	phosphatase		-1.097769308	0	0.66212409	0	1	0	11.4726249	11.7284475	11.7101113	11.2500391
7303	**ENSG0000013707**	RNF38	protein_coding	chr9	36336396	36487548	-	Human GRCh	OTTHUMG000000	RNF38	**ring finger protein 38**	Nucleus	enzyme		-1.295076392	0	0.22858227	0	1	0	10.6618847	10.979564	10.6648527	10.4215841
7304	**ENSG0000013707**	TLN1	protein_coding	chr9	35696948	35732195	-	Human GRCh	OTTHUMG000000	TLN1	**talin 1**	Plasma Mem	other		-1.166471851	0	0.41134152	0	1	0	12.5990223	12.9047392	12.6902414	12.4825579
7306	**ENSG0000013707**	SIT1	protein_coding	chr9	35649295	35650931	-	Human GRCh	OTTHUMG000000	SIT1	**signaling threshold regulating transmembrane adaptor 1**	Plasma Mem	other		1	0	1	0	1	0	1.63734986	1.72369255	1.70981289	1.64258097
7308	**ENSG0000013709**	DMRT1	protein_coding	chr9	841690	969090	+	Human GRCh	OTTHUMG000000	DMRT1	**doublesex and mab-3 related transcription factor 1**	Nucleus	transcription		3.939912279	1	0.37832949	0	NA	0	1.43311625	1.00991423	1.00659313	2.143793
7309	**ENSG0000013709**	DNAJB5	protein_coding	chr9	34989641	34998900	+	Human GRCh	OTTHUMG000000	DNAJB5	**DnaJ heat shock protein family (Hsp40) member B5**	Cytoplasm	other		1.173575385	0	0.54292003	0	1	0	8.06921017	8.36811383	8.34517896	8.45567343
7310	**ENSG0000013709**	SPAG8	protein_coding	chr9	35808045	35812272	-	Human GRCh	OTTHUMG000000	SPAG8	**sperm associated antigen 8**	Cytoplasm	other	SPI-62	2.672693023	1	0.02768648	1	1	0	4.27737174	4.37427141	5.18038146	5.08931033
7311	**ENSG0000013710**	DCTN3	protein_coding	chr9	34613545	34620523	-	Human GRCh	OTTHUMG000000	DCTN3	**dynactin subunit 3**	Nucleus	other		-1.141414922	0	0.58758391	0	1	0	10.3364423	10.7629858	10.4928878	10.3401613
7312	**ENSG0000013710**	CD72	protein_coding	chr9	35609982	35646810	-	Human GRCh	OTTHUMG000000	CD72	**CD72 molecule**	Plasma Mem	transmembra		-1.531927092	-1	0.5156564	0	1	0	3.28451344	3.67025139	3.27241733	3.16062597
7313	**ENSG0000013710**	TMEM8B	protein_coding	chr9	35814451	35865518	+	Human GRCh	OTTHUMG000000	TMEM8B	**transmembrane protein 8B**	Plasma Mem	other		1.080499867	0	0.7990529	0	1	0	8.21928722	8.41944286	8.30225098	8.4972874
7314	**ENSG0000013710**	GRHPR	protein_coding	chr9	37422666	37436990	+	Human GRCh	OTTHUMG000000	GRHPR	**glyoxylate and hydroxypyruvate reductase**	Cytoplasm	enzyme	GSK12788	-1.133814242	0	0.53426521	0	1	0	10.8978469	11.249454	11.0678547	10.8077555
7315	**ENSG0000013712**	ALDH1B1	protein_coding	chr9	38392702	38398661	+	Human GRCh	OTTHUMG000000	ALDH1B1	**aldehyde dehydrogenase 1 family member B1**	Cytoplasm	enzyme		-1.069605109	0	0.78048134	0	1	0	10.4201589	10.740448	10.6438062	10.3682466
7316	**ENSG0000013713**	HINT2	protein_coding	chr9	35812960	35815354	-	Human GRCh	OTTHUMG000000	HINT2	**histidine triad nucleotide binding protein 2**	Cytoplasm	enzyme		-1.061359567	0	0.7756692	0	1	0	9.16203381	9.19161846	9.19300816	9.02440301
7317	**ENSG0000013713**	ARHGEF39	protein_coding	chr9	35658875	35675866	-	Human GRCh	OTTHUMG000000	ARHGEF39	**Rho guanine nucleotide exchange factor 39**	Plasma Mem	other		-1.226994736	0	0.29306481	0	1	0	9.91011498	10.0923524	9.78128046	9.78549692
7318	**ENSG0000013714**	IGFBPL1	protein_coding	chr9	38406528	38424454	-	Human GRCh	OTTHUMG000000	IGFBPL1	**insulin like growth factor binding protein like 1**	Extracellular	other		-1.044706787	0	0.88088599	0	1	0	6.15666121	6.82578801	6.700056	6.25635619
7319	**ENSG0000013714**	DENND4C	protein_coding	chr9	19230435	19374281	+	Human GRCh	OTTHUMG000000	DENND4C	**DENN domain containing 4C**	Cytoplasm	other		-1.075806957	0	0.74318402	0	1	0	11.1825741	11.4235409	11.3513199	11.0934805
7320	**ENSG0000013715**	RPS6	protein_coding	chr9	19375715	19380236	-	Human GRCh	OTTHUMG000000	RPS6	**ribosomal protein S6**	Cytoplasm	other		-1.128168742	0	0.54595522	0	1	0	13.9351885	14.2082503	14.0356074	13.8432293
7321	**ENSG0000013716**	CNPY3	protein_coding	chr6	42929480	42939294	+	Human GRCh	OTTHUMG000000	CNPY3	**canopy FGF signaling regulator 3**	Cytoplasm	other		-1.150247689	0	0.48781532	0	1	0	10.4045552	10.578513	10.4656839	10.1967148
7322	**ENSG0000013716**	FOXP4	protein_coding	chr6	41546381	41602384	+	Human GRCh	OTTHUMG000000	FOXP4	**forkhead box P4**	Nucleus	transcription		-1.100222927	0	0.61729953	0	1	0	10.8217753	11.0833674	10.9058531	10.8060084
7323	**ENSG0000013716**	PPIL1	protein_coding	chr6	36854827	36874803	-	Human GRCh	OTTHUMG000000	PPIL1	**peptidylprolyl isomerase like 1**	Plasma Mem	enzyme		-1.045384571	0	0.8333589	0	1	0	9.76537284	10.0461658	9.93682664	9.78706316
7324	**ENSG0000013717**	KLC4	protein_coding	chr6	43040777	43075095	+	Human GRCh	OTTHUMG000000	KLC4	**kinesin light chain 4**	Other	other		-1.035825378	0	0.87357637	0	1	0	9.32758636	9.49607635	9.42925057	9.32077905
7325	**ENSG0000013717**	KIF13A	protein_coding	chr6	17759183	17987635	-	Human GRCh	OTTHUMG000000	KIF13A	**kinesin family member 13A**	Cytoplasm	transporter		-1.067423135	0	0.79468898	0	1	0	9.67747193	9.87976975	9.79744635	9.60462551
7326	**ENSG0000013718**	ZSCAN9	protein_coding	chr6	28224886	28233487	+	Human GRCh	OTTHUMG000000	ZSCAN9	**zinc finger and SCAN domain containing 9**	Nucleus	transcription		-1.277207163	0	0.24755892	0	1	0	9.0016568	9.27266812	9.04785696	8.69499431
7327	**ENSG0000013719**	PIM1	protein_coding	chr6	37170152	37175428	+	Human GRCh	OTTHUMG000000	PIM1	**Pim-1 proto-oncogene, serine/threonine kinase**	Cytoplasm	kinase	TP-3654, S	1.02052388	0	0.94238909	0	1	0	8.97927339	9.34128098	8.77051413	9.50419048
7328	**ENSG0000013719**	GMPR	protein_coding	chr6	16238587	16295549	+	Human GRCh	OTTHUMG000000	GMPR	**guanosine monophosphate reductase**	Cytoplasm	enzyme		1.072810868	0	0.87139509	0	1	0	5.27731551	5.61173663	5.5582385	5.49178994
7329	**ENSG0000013720**	CMTR1	protein_coding	chr6	37433219	37482827	+	Human GRCh	OTTHUMG000000	CMTR1	**cap methyltransferase 1**	Nucleus	enzyme		-1.039946199	0	0.84932972	0	1	0	10.1506226	10.6216324	10.5149954	10.1971754
7330	**ENSG0000013720**	TFAP2A	protein_coding	chr6	10393186	10419659	-	Human GRCh	OTTHUMG000000	TFAP2A	**transcription factor AP-2 alpha**	Nucleus	transcription		1.473474963	0	0.58653287	0	1	0	6.68815353	6.17553952	6.61438493	6.90352232
7332	**ENSG0000013720**	YIPF3	protein_coding	chr6	43511832	43516985	-	Human GRCh	OTTHUMG000000	YIPF3	**Yip1 domain family member 3**	Cytoplasm	other		-1.156070048	0	0.4753149	0	1	0	11.1404829	11.4413992	11.2025722	11.0669957
7333	**ENSG0000013721**	TMEM14B	protein_coding	chr6	10747759	10852753	+	Human GRCh	OTTHUMG000000	TMEM14B	**transmembrane protein 14B**	Other	other		-1.067533003	0	0.76564043	0	1	0	9.68396166	10.0461728	9.96552391	9.62654751
7334	**ENSG0000013721**	TMEM63B	protein_coding	chr6	44126914	44155519	+	Human GRCh	OTTHUMG000000	TMEM63B	**transmembrane protein 63B**	Plasma Mem	ion channel		-1.153051412	0	0.46203409	0	1	0	11.034425	11.230358	11.1018763	10.8346412
7335	**ENSG0000013721**	FRS3	protein_coding	chr6	41770176	41786542	-	Human GRCh	OTTHUMG000000	FRS3	**fibroblast growth factor receptor substrate 3**	Plasma Mem	other		-1.065974724	0	0.79473567	0	1	0	7.86450524	8.10955752	8.13776176	7.66594196
7336	**ENSG0000013722**	TJAP1	protein_coding	chr6	43477523	43506556	+	Human GRCh	OTTHUMG000000	TJAP1	**tight junction associated protein 1**	Plasma Mem	other		1.027193577	0	0.89521661	0	1	0	10.6480037	10.767073	10.8873583	10.5686954
7340	**ENSG0000013726**	KIAA0319	protein_coding	chr6	24544104	24646191	-	Human GRCh	OTTHUMG000000	KIAA0319	**KIAA0319**	Plasma Mem	other		1.130627138	0	0.71809378	0	1	0	5.43974754	5.3790308	5.49983308	5.54814983
7342	**ENSG0000013726**	SLC22A23	protein_coding	chr6	3268962	3457050	-	Human GRCh	OTTHUMG000000	SLC22A23	**solute carrier family 22 member 23**	Other	transporter		-1.068766692	0	0.76083579	0	1	0	10.036091	10.1832173	9.97111296	10.1000379
7343	**ENSG0000013726**	TUBB2A	protein_coding	chr6	3153497	3157544	-	Human GRCh	OTTHUMG000000	TUBB2A	**tubulin beta 2A class IIa**	Cytoplasm	other	docetaxel/	1.415331733	0	0.29442475	0	1	0	6.17796762	6.30289577	6.25513885	6.84784859
7344	**ENSG0000013726**	LRRC1	protein_coding	chr6	53794497	53924125	+	Human GRCh	OTTHUMG000000	LRRC1	**leucine rich repeat containing 1**	Cytoplasm	other		1.177130541	0	0.43768738	0	1	0	8.44457245	8.52744772	8.69654561	8.61678251
7345	**ENSG0000013727**	GCM1	protein_coding	chr6	53126961	53148841	-	Human GRCh	OTTHUMG000000	GCM1	**glial cells missing transcription factor 1**	Nucleus	transcription		1.016634722	0	0.97708709	0	1	0	3.88376673	4.94467845	4.42001789	4.6691739
7346	**ENSG0000013727**	FOXF2	protein_coding	chr6	1389576	1395603	+	Human GRCh	OTTHUMG000000	FOXF2	**forkhead box F2**	Nucleus	transcription		-9.914441335	-1	0.62246739	0	NA	0	1.18590987	0.48040166	0.47806191	0.46714045
7347	**ENSG0000013727**	BPHL	protein_coding	chr6	3118374	3153578	+	Human GRCh	OTTHUMG000000	BPHL	**biphenyl hydrolase like**	Cytoplasm	enzyme		-1.226006831	0	0.36649896	0	1	0	9.08883123	9.28042998	9.1208905	8.79921049
7348	**ENSG0000013727**	RIPK1	protein_coding	chr6	3063824	3115187	+	Human GRCh	OTTHUMG000000	RIPK1	**receptor interacting serine/threonine kinase 1**	Plasma Mem	kinase	eclitasertib	-1.083316381	0	0.68698404	0	1	0	9.39401185	9.64150404	9.57963919	9.27746492
7349	**ENSG0000013728**	TUBB2B	protein_coding	chr6	3223324	3231730	-	Human GRCh	OTTHUMG000000	TUBB2B	**tubulin beta 2B class IIb**	Cytoplasm	other		1.05875307	0	0.87989398	0	1	0	4.8719735	5.37804098	4.69518024	5.51239561
7350	**ENSG0000013728**	UQCC2	protein_coding	chr6	33694293	33711727	-	Human GRCh	OTTHUMG000000	UQCC2	**ubiquinol-cytochrome c reductase complex assembly factor 2**	Cytoplasm	other		-1.078141942	0	0.73018148	0	1	0	9.95099156	10.0017011	9.94306389	9.84603883
7351	**ENSG0000013730**	HMGA1	protein_coding	chr6	34236873	34246231	+	Human GRCh	OTTHUMG000000	HMGA1	**high mobility group AT-hook 1**	Nucleus	transcription		1.413848552	0	0.10459589	0	1	0	12.0502218	12.1676765	12.4270357	12.5709759

7352	**ENSG0000013731**	TCF19	protein_coding	chr6	31158331	31167159	+	Human GRCh	OTTHUMG000000	TCF19	**transcription factor 19**	Nucleus	transcription		-1.055547424	0	0.78895055	0	1	0	10.6708639	10.9490294	10.8560003	10.6540791
7353	**ENSG0000013731**	FLOT1	protein_coding	chr6	30727709	30742732	-	Human GRCh	OTTHUMG000000	FLOT1	**flotillin 1**	Plasma Mem	other		-1.089005756	0	0.62874674	0	1	0	11.1783721	11.3405043	11.1546279	11.1808334
7354	**ENSG0000013733**	IER3	protein_coding	chr6	30743199	30744548	-	Human GRCh	OTTHUMG000000	IER3	**immediate early response 3**	Cytoplasm	other		1.200580413	0	0.68972621	0	1	0	5.96611866	5.99558712	5.81113535	6.4678145
7355	**ENSG0000013733**	MDC1	protein_coding	chr6	30699807	30717447	-	Human GRCh	OTTHUMG000000	MDC1	**mediator of DNA damage checkpoint 1**	Nucleus	other		-1.075495931	0	0.72376376	0	1	0	13.1429533	13.4767559	13.333507	13.1379356
7356	**ENSG0000013733**	PGBD1	protein_coding	chr6	28281572	28302549	+	Human GRCh	OTTHUMG000000	PGBD1	**piggyBac transposable element derived 1**	Extracellular	enzyme		1.017371701	0	0.94201631	0	1	0	7.44907668	7.36792106	7.5000739	7.3511806
7357	**ENSG0000013734**	ATAT1	protein_coding	chr6	30626842	30646823	+	Human GRCh	OTTHUMG000000	ATAT1	**alpha tubulin acetyltransferase 1**	Cytoplasm	enzyme		-1.014207121	0	0.94787305	0	1	0	10.2808885	10.2887015	10.3728859	10.1557479
7358	**ENSG0000013736**	TPMT	protein_coding	chr6	18128311	18155077	-	Human GRCh	OTTHUMG000000	TPMT	**thiopurine S-methyltransferase**	Cytoplasm	enzyme	olsalazine	1.045095741	0	0.85308371	0	1	0	8.60830676	9.10369474	8.99790295	8.85827819
7360	**ENSG0000013739**	RNF144B	protein_coding	chr6	18387350	18468870	+	Human GRCh	OTTHUMG000000	RNF144B	**ring finger protein 144B**	Cytoplasm	enzyme		1.484071391	0	0.27612875	0	1	0	6.76738228	6.75526246	7.21507939	7.10702497
7361	**ENSG0000013740**	NRM	protein_coding	chr6	30688047	30691420	-	Human GRCh	OTTHUMG000000	NRM	**nurim**	Nucleus	other		-1.164400823	0	0.53822377	0	1	0	8.87918793	9.22568034	9.00250338	8.79278901
7362	**ENSG0000013740**	MTCH1	protein_coding	chr6	36965807	36996298	-	Human GRCh	OTTHUMG000000	MTCH1	**mitochondrial carrier 1**	Cytoplasm	other		-1.046494239	0	0.81810139	0	1	0	11.6049184	11.838373	11.8096611	11.5284301
7363	**ENSG0000013741**	VARS2	protein_coding	chr6	30914205	30926459	+	Human GRCh	OTTHUMG000001	VARS2	**valyl-tRNA synthetase 2, mitochondrial**	Cytoplasm	enzyme		-1.070087264	0	0.70835865	0	1	0	10.5287201	10.6894028	10.6602168	10.3976476
7364	**ENSG0000013741**	TAF8	protein_coding	chr6	42050513	42087461	+	Human GRCh	OTTHUMG000000	TAF8	**TATA-box binding protein associated factor 8**	Nucleus	transcription		-1.259824764	0	0.24959234	0	1	0	9.41038529	9.76969507	9.38308508	9.32774814
7365	**ENSG0000013741**	FAM8A1	protein_coding	chr6	17600302	17611719	+	Human GRCh	OTTHUMG000000	FAM8A1	**family with sequence similarity 8 member A1**	Other	other		1.14248456	0	0.52819591	0	1	0	10.1202944	10.414179	10.5053473	10.3418741
7366	**ENSG0000013743**	C6orf52	protein_coding	chr6	10671418	10694797	-	Human GRCh	OTTHUMG000000	C6orf52	**chromosome 6 open reading frame 52**	Other	other		1.40883279	0	0.40971453	0	1	0	5.71031665	5.17992465	6.2021314	5.21498822
7369	**ENSG0000013744**	CPEB2	protein_coding	chr4	15002481	15070153	+	Human GRCh	OTTHUMG000000	CPEB2	**cytoplasmic polyadenylation element binding protein 2**	Cytoplasm	translation re		-1.132992868	0	0.82473471	0	1	0	8.341851	8.33217286	8.23689693	8.15348853
7370	**ENSG0000013746**	FHDC1	protein_coding	chr4	152936323	152979671	+	Human GRCh	OTTHUMG000001	FHDC1	**FH2 domain containing 1**	Cytoplasm	other		1.284252595	0	0.47239671	0	1	0	9.84399173	10.1167742	10.0932096	10.4272445
7371	**ENSG0000013746**	TLR2	protein_coding	chr4	153684070	153706260	+	Human GRCh	OTTHUMG000001	TLR2	**toll like receptor 2**	Plasma Mem	transmembra	XS15, OM	-1.756906726	-1	0.48707765	0	1	0	3.1632391	2.76699399	2.47712901	2.86929778
7374	**ENSG0000013747**	MYO7A	protein_coding	chr11	77128246	77215241	+	Human GRCh	OTTHUMG000001	MYO7A	**myosin VIIA**	Cytoplasm	enzyme		-1.133858637	0	0.66837922	0	1	0	6.51680813	6.75220969	6.44394838	6.58014951
7375	**ENSG0000013747**	FCHSD2	protein_coding	chr11	72836745	73142318	-	Human GRCh	OTTHUMG000001	FCHSD2	**FCH and double SH3 domains 2**	Plasma Mem	other		-1.211822396	0	0.39778445	0	1	0	10.588448	10.8974091	10.7020112	10.3527888
7376	**ENSG0000013748**	ARRB1	protein_coding	chr11	75260122	75351705	-	Human GRCh	OTTHUMG000001	ARRB1	**arrestin beta 1**	Cytoplasm	transcription		1.033471317	0	0.89699962	0	1	0	12.0108303	12.2081493	12.1931302	12.106101
7377	**ENSG0000013749**	SLCO2B1	protein_coding	chr11	75100563	75206549	+	Human GRCh	OTTHUMG000001	SLCO2B1	**solute carrier organic anion transporter family member 2B1**	Plasma Mem	transporter		5.202935414	1	0.26636517	0	1	0	1.92109923	2.01343037	1.99856786	2.52222766
7378	**ENSG0000013749**	THAP12	protein_coding	chr11	76349898	76381132	-	Human GRCh	OTTHUMG000001	THAP12	**THAP domain containing 12**	Nucleus	other		1.023769403	0	0.90987367	0	1	0	10.4875404	10.8043512	10.9037567	10.4084015
7379	**ENSG0000013749**	ANKRD42	protein_coding	chr11	83193712	83260694	+	Human GRCh	OTTHUMG000001	ANKRD42	**ankyrin repeat domain 42**	Nucleus	transcription		1.414437617	0	0.20410543	0	1	0	8.51618541	8.74209363	9.27926325	8.68676551
7380	**ENSG0000013749**	IL18BP	protein_coding	chr11	71998613	72007315	+	Human GRCh	OTTHUMG000001	IL18BP	**interleukin 18 binding protein**	Extracellular	other		-1.009747051	0	0.96637566	0	1	0	10.0916975	10.3311468	10.4005993	9.97469849
7381	**ENSG0000013749**	NUMA1	protein_coding	chr11	72002864	72080693	-	Human GRCh	OTTHUMG000001	NUMA1	**nuclear mitotic apparatus protein 1**	Nucleus	other		-1.127059618	0	0.57584162	0	1	0	13.5550372	13.8170742	13.6763344	13.4350328
7382	**ENSG0000013750**	CCDC90B	protein_coding	chr11	83259081	83286390	-	Human GRCh	OTTHUMG000001	CCDC90B	**coiled-coil domain containing 90B**	Cytoplasm	other		-1.000426546	0	0.99867066	0	1	0	9.87790219	10.0977326	10.2370582	9.68333413
7383	**ENSG0000013750**	SYTL2	protein_coding	chr11	85694224	85811159	-	Human GRCh	OTTHUMG000001	SYTL2	**synaptotagmin like 2**	Cytoplasm	other		1.040562221	0	0.90588476	0	1	0	6.10852115	6.58082809	6.43608579	6.36536902
7384	**ENSG0000013750**	RAB30	protein_coding	chr11	82973133	83071923	-	Human GRCh	OTTHUMG000001	RAB30	**RAB30, member RAS oncogene family**	Cytoplasm	enzyme		1.344944019	0	0.19722005	0	1	0	9.32465359	9.66324171	10.0020605	9.63246346
7385	**ENSG0000013750**	CREBZF	protein_coding	chr11	85657742	85682908	-	Human GRCh	OTTHUMG000001	CREBZF	**CREB/ATF bZIP transcription factor**	Nucleus	transcription		1.161756137	0	0.48526043	0	1	0	11.2231883	11.3060729	11.6850037	11.095187
7387	**ENSG0000013750**	PRCP	protein_coding	chr11	82822936	82970584	-	Human GRCh	OTTHUMG000001	PRCP	**prolylcarboxypeptidase**	Cytoplasm	peptidase		-1.186710691	0	0.43995403	0	1	0	9.93524919	10.2339022	10.11465	9.65274747
7388	**ENSG0000013751**	NARS2	protein_coding	chr11	78435620	78575194	-	Human GRCh	OTTHUMG000001	NARS2	**asparaginyl-tRNA synthetase 2, mitochondrial**	Cytoplasm	enzyme		1.127269264	0	0.58999476	0	1	0	9.8201893	10.023839	10.3012932	9.74849074
7389	**ENSG0000013752**	RNF121	protein_coding	chr11	71929018	71997597	+	Human GRCh	OTTHUMG000001	RNF121	**ring finger protein 121**	Other	other		-1.000423667	0	0.99848395	0	1	0	9.57653865	9.72524032	9.90172649	9.33913246
7390	**ENSG0000013754**	MRPL15	protein_coding	chr8	54135241	54148514	+	Human GRCh	OTTHUMG000001	MRPL15	**mitochondrial ribosomal protein L15**	Cytoplasm	other		-1.188972637	0	0.5155686	0	1	0	9.64574075	10.2666199	9.90468062	9.69663472
7391	**ENSG0000013755**	PI15	protein_coding	chr8	74824534	74855029	+	Human GRCh	OTTHUMG000001	PI15	**peptidase inhibitor 15**	Extracellular	other		3.240081882	1	0.22800624	0	1	0	2.19239555	3.08491344	3.3115512	3.39025938
7392	**ENSG0000013756**	TTPA	protein_coding	chr8	63048553	63086053	-	Human GRCh	OTTHUMG000001	TTPA	**alpha tocopherol transfer protein**	Cytoplasm	transporter		1.966626189	1	0.22831295	0	1	0	4.20877898	4.47897283	5.29740985	4.61331307
7393	**ENSG0000013756**	GGH	protein_coding	chr8	63014881	63039407	-	Human GRCh	OTTHUMG000001	GGH	**gamma-glutamyl hydrolase**	Cytoplasm	peptidase		1.111695185	0	0.67172332	0	1	0	9.31397838	9.49069798	9.72222641	9.27652975
7394	**ENSG0000013757**	SLCO5A1	protein_coding	chr8	69667046	69834978	-	Human GRCh	OTTHUMG000001	SLCO5A1	**solute carrier organic anion transporter family member 5A1**	Plasma Mem	transporter		-1.093527525	0	0.81092098	0	1	0	8.67777078	8.71108788	8.59050856	8.60608693
7395	**ENSG0000013757**	SULF1	protein_coding	chr8	69466624	69660915	+	Human GRCh	OTTHUMG000001	SULF1	**sulfatase 1**	Cytoplasm	enzyme		-1.117445922	0	0.93982069	0	1	0	3.23496765	3.75849921	3.3092579	3.64237852
7396	**ENSG0000013757**	TGS1	protein_coding	chr8	55773446	55826445	+	Human GRCh	OTTHUMG000001	TGS1	**trimethylguanosine synthase 1**	Nucleus	enzyme		1.015000418	0	0.94442487	0	1	0	9.50919736	9.78352925	9.80551106	9.51716245
7397	**ENSG0000013757**	SDCBP	protein_coding	chr8	58552924	58582859	+	Human GRCh	OTTHUMG000001	SDCBP	**syndecan binding protein**	Plasma Mem	enzyme		1.071719431	0	0.79855391	0	1	0	10.9219138	11.2052591	11.2291276	11.0618383
7398	**ENSG0000013760**	NEK1	protein_coding	chr4	169369704	169612632	-	Human GRCh	OTTHUMG000001	NEK1	**NIMA related kinase 1**	Nucleus	kinase		1.007516401	0	0.97494796	0	1	0	8.53384922	9.1088469	9.0600247	8.62740085
7399	**ENSG0000013762**	DDX60	protein_coding	chr4	168216291	168318883	-	Human GRCh	OTTHUMG000001	DDX60	**DExD/H-box helicase 60**	Cytoplasm	enzyme		-1.072083336	0	0.84827089	0	1	0	6.52296138	7.19719959	6.97395568	6.6882782
7401	**ENSG0000013764**	SORL1	protein_coding	chr11	121452314	121633763	+	Human GRCh	OTTHUMG000001	SORL1	**sortilin related receptor 1**	Cytoplasm	transporter		-1.060976961	0	0.8330359	0	1	0	9.9864176	10.1948818	10.00778	10.0585904
7402	**ENSG0000013764**	TMPRSS4	protein_coding	chr11	118077072	118121890	+	Human GRCh	OTTHUMG000001	TMPRSS4	**transmembrane serine protease 4**	Cytoplasm	peptidase		-2.0774724	-1	0.01257984	1	1	0	7.6436227	7.49303963	6.93707764	6.78427886
7403	**ENSG0000013765**	BUD13	protein_coding	chr11	116748170	116772987	-	Human GRCh	OTTHUMG000000	BUD13	**BUD13 homolog**	Nucleus	other		-1.158963962	0	0.50477528	0	1	0	9.37422492	9.64196159	9.48396108	9.20842725
7408	**ENSG0000013769**	CFAP300	protein_coding	chr11	102047437	102084554	+	Human GRCh	OTTHUMG000001	CFAP300	**cilia and flagella associated protein 300**	Cytoplasm	other		10.76434321	1	0.0722018	0	1	0	2.0734846	2.13009628	2.64707325	2.56345872
7409	**ENSG0000013769**	DCUN1D5	protein_coding	chr11	103050686	103092194	-	Human GRCh	OTTHUMG000001	DCUN1D5	**defective in cullin neddylation 1 domain containing 5**	Nucleus	other		1.155586615	0	0.47690317	0	1	0	9.97771515	10.2173047	10.4540183	10.0372984
7410	**ENSG0000013769**	YAP1	protein_coding	chr11	102110447	102233424	+	Human GRCh	OTTHUMG000001	YAP1	**Yes1 associated transcriptional regulator**	Nucleus	transcription	verteporfir	2.17669569	1	0.28040319	0	1	0	7.20975117	6.95365527	6.9691893	8.46676261
7411	**ENSG0000013769**	TRIM29	protein_coding	chr11	120111286	120185529	-	Human GRCh	OTTHUMG000001	TRIM29	**tripartite motif containing 29**	Cytoplasm	transcription		-2.507839893	-1	0.35760076	0	1	0	3.29189096	2.33937617	1.84835282	2.77335394
7412	**ENSG0000013770**	SLC37A4	protein_coding	chr11	119023751	119030906	-	Human GRCh	OTTHUMG000001	SLC37A4	**solute carrier family 37 member 4**	Cytoplasm	transporter		-1.13580817	0	0.55662716	0	1	0	10.7337481	10.9665255	10.8930262	10.5028162
7414	**ENSG0000013770**	POU2F3	protein_coding	chr11	120236640	120319945	+	Human GRCh	OTTHUMG000001	POU2F3	**POU class 2 homeobox 3**	Nucleus	transcription		1.374646915	0	0.6086286	0	1	0	3.55785806	3.79905525	3.13846323	4.30898169
7415	**ENSG0000013771**	RDX	protein_coding	chr11	109864295	110296712	-	Human GRCh	OTTHUMG000001	RDX	**radixin**	Cytoplasm	other		1.025944793	0	0.89866489	0	1	0	11.1689223	11.3914905	11.494526	11.0991917
7416	**ENSG0000013771**	PPP2R1B	protein_coding	chr11	111726908	111766389	-	Human GRCh	OTTHUMG000001	PPP2R1B	**protein phosphatase 2 scaffold subunit Abeta**	Plasma Mem	phosphatase		-1.016527097	0	0.9415047	0	1	0	10.3840112	10.8059883	10.6796307	10.5048805
7417	**ENSG0000013771**	FDX1	protein_coding	chr11	110429948	110464884	+	Human GRCh	OTTHUMG000001	FDX1	**ferredoxin 1**	Cytoplasm	transporter		1.448148436	0	0.18638776	0	1	0	7.53932546	8.0609258	8.51827617	7.82964799
7418	**ENSG0000013772**	CFAP68	protein_coding	chr11	111878935	111885975	+	Human GRCh	OTTHUMG000001	CFAP68	**cilia and flagella associated protein 68**	Nucleus	other		-1.169152946	0	0.58342011	0	1	0	8.08611499	8.38624829	8.27377451	7.83419302
7419	**ENSG0000013772**	FXYD6	protein_coding	chr11	117836976	117877486	-	Human GRCh	OTTHUMG000001	FXYD6	**FXYD domain containing ion transport regulator 6**	Plasma Mem	ion channel		-1.002192725	0	0.99601275	0	1	0	4.87070259	5.01143184	5.12469655	4.73012283
7420	**ENSG0000013772**	ARHGAP2(	protein_coding	chr11	110577042	110713189	-	Human GRCh	OTTHUMG000001	ARHGAP20	**Rho GTPase activating protein 20**	Cytoplasm	other		-1.843558665	-1	0.3339945	0	1	0	3.90550139	2.79977355	2.77869382	3.3033747
7421	**ENSG0000013773**	FXYD2	protein_coding	chr11	117800844	117828698	-	Human GRCh	OTTHUMG000001	FXYD2	**FXYD domain containing ion transport regulator 2**	Plasma Mem	ion channel	cyclothiazi	-10.95974583	-1	0.04863674	1	1	0	2.35873579	2.4893235	1.82988241	1.7665983
7423	**ENSG0000013774**	TMPRSS13	protein_coding	chr11	117900641	117929459	-	Human GRCh	OTTHUMG000001	TMPRSS13	**transmembrane serine protease 13**	Plasma Mem	peptidase		-1.145153745	0	0.77348696	0	1	0	4.44784661	5.06205284	4.52943003	4.80487932
7426	**ENSG0000013776**	ALKBH8	protein_coding	chr11	107502727	107565742	-	Human GRCh	OTTHUMG000001	ALKBH8	**alkB homolog 8, tRNA methyltransferase**	Cytoplasm	enzyme		-1.023925836	0	0.93030622	0	1	0	8.15298749	8.24726745	8.34972904	7.97102572
7427	**ENSG0000013776**	MAP2K5	protein_coding	chr15	67542703	67807117	+	Human GRCh	OTTHUMG000001	MAP2K5	**mitogen-activated protein kinase kinase 5**	Cytoplasm	kinase	BIX 02189	-1.20287671	0	0.41727174	0	1	0	9.19401098	9.33730467	9.16193719	8.96246252
7428	**ENSG0000013776**	UNC13C	protein_coding	chr15	53978201	54628707	+	Human GRCh	OTTHUMG000001	UNC13C	**unc-13 homolog C**	Cytoplasm	other		-1.826513322	-1	0.20442368	0	1	0	5.75843871	6.32748676	5.88006194	4.83404812
7429	**ENSG0000013776**	SQOR	protein_coding	chr15	45631148	45691281	+	Human GRCh	OTTHUMG000001	SQOR	**sulfide quinone oxidoreductase**	Cytoplasm	enzyme		1.556173285	1	0.33010645	0	1	0	4.51854096	4.90892416	4.75770124	5.38664428
7430	**ENSG0000013777**	CTDSPL2	protein_coding	chr15	44427622	44529038	+	Human GRCh	OTTHUMG000001	CTDSPL2	**CTD small phosphatase like 2**	Nucleus	phosphatase		-1.047881718	0	0.83329303	0	1	0	11.2899673	11.6092995	11.5816602	11.2049059
7431	**ENSG0000013777**	SLTM	protein_coding	chr15	58879050	58933679	-	Human GRCh	OTTHUMG000000	SLTM	**SAFB like transcription modulator**	Nucleus	other		1.012123548	0	0.95077212	0	1	0	10.5246703	10.872809	10.8807359	10.5448711
7432	**ENSG0000013780**	THBS1	protein_coding	chr15	39581079	39599466	+	Human GRCh	OTTHUMG000001	THBS1	**thrombospondin 1**	Extracellular	other		1.93625749	1	0.30062775	0	1	0	6.61873342	6.85900273	7.06446882	7.64783093
7433	**ENSG0000013780**	MAPKBP1	protein_coding	chr15	41774434	41827855	+	Human GRCh	OTTHUMG000001	MAPKBP1	**mitogen-activated protein kinase binding protein 1**	Cytoplasm	other		-1.04111276	0	0.85330886	0	1	0	10.5235762	10.780731	10.8115748	10.3746188
7434	**ENSG0000013780**	NUSAP1	protein_coding	chr15	41320794	41381050	+	Human GRCh	OTTHUMG000001	NUSAP1	**nucleolar and spindle associated protein 1**	Nucleus	other		-1.092000356	0	0.71501287	0	1	0	11.4560505	11.9355863	11.7002133	11.5382513
7435	**ENSG0000013780**	NDUFAF1	protein_coding	chr15	41387218	41409403	-	Human GRCh	OTTHUMG000001	NDUFAF1	**NADH:ubiquinone oxidoreductase complex assembly factor 1**	Cytoplasm	other		-1.067723666	0	0.83842502	0	1	0	8.03065578	8.41982524	8.33513839	7.97216054
7436	**ENSG0000013780**	KIF23	protein_coding	chr15	69414246	69448427	+	Human GRCh	OTTHUMG000001	KIF23	**kinesin family member 23**	Cytoplasm	other		-1.09757108	0	0.66705601	0	1	0	10.6113591	11.0434841	10.8973697	10.5704145
7437	**ENSG0000013780**	ITGA11	protein_coding	chr15	68296532	68432163	-	Human GRCh	OTTHUMG000001	ITGA11	**integrin subunit alpha 11**	Plasma Mem	other		2.624128071	1	0.30825585	0	1	0	3.23977185	2.86485445	3.91198701	3.88257244
7438	**ENSG0000013781**	KNL1	protein_coding	chr15	40594020	40664342	+	Human GRCh	OTTHUMG000001	KNL1	**kinetochore scaffold 1**	Nucleus	other		-1.151111207	0	0.47872356	0	1	0	11.6683881	11.9021694	11.759346	11.4933327
7439	**ENSG0000013781**	HAUS2	protein_coding	chr15	42548828	42569994	+	Human GRCh	OTTHUMG000001	HAUS2	**HAUS augmin like complex subunit 2**	Cytoplasm	other		1.008702334	0	0.9684228	0	1	0	9.51546354	9.77339278	9.83039771	9.46323507
7440	**ENSG0000013781**	RTF1	protein_coding	chr15	41408408	41483563	+	Human GRCh	OTTHUMG000001	RTF1	**RTF1 homolog, Paf1/RNA polymerase II complex component**	Nucleus	other		-1.13876613	0	0.52718373	0	1	0	11.0996435	11.4643383	11.2517568	11.0355555
7441	**ENSG0000013781**	PARP6	protein_coding	chr15	72241181	72272999	-	Human GRCh	OTTHUMG000001	PARP6	**poly(ADP-ribose) polymerase family member 6**	Other	enzyme		-1.977078679	-1	0.34251019	0	1	0	4.15035487	5.0496523	3.50070803	4.46271515
7442	**ENSG0000013781**	RPLP1	protein_coding	chr15	69452814	69456205	+	Human GRCh	OTTHUMG000001	RPLP1	**ribosomal protein lateral stalk subunit P1**	Cytoplasm	other		1.202611306	0	0.37655138	0	1	0	12.8885779	13.0957493	13.2538874	13.1514285
7443	**ENSG0000013781**	PAQR5	protein_coding	chr15	69298912	69407780	+	Human GRCh	OTTHUMG000001	PAQR5	**progestin and adipoQ receptor family member 5**	Other	other		1.79985789	1	0.11305571	0	1	0	5.69801837	6.29946818	6.3453827	6.82353439
7444	**ENSG0000013782**	LRRC49	protein_coding	chr15	70853239	71053658	+	Human GRCh	OTTHUMG000001	LRRC49	**leucine rich repeat containing 49**	Cytoplasm	other		-1.159996687	0	0.62704635	0	1	0	7.37411176	7.73334726	7.50056626	7.31795119
7445	**ENSG0000013782**	TUBGCP4	protein_coding	chr15	43369221	43409771	+	Human GRCh	OTTHUMG000001	TUBGCP4	**tubulin gamma complex component 4**	Cytoplasm	other		-1.037896719	0	0.85365193	0	1	0	11.0898676	11.4071369	11.3482431	11.0689694
7446	**ENSG0000013782**	RMDN3	protein_coding	chr15	40735884	40755851	-	Human GRCh	OTTHUMG000001	RMDN3	**regulator of microtubule dynamics 3**	Cytoplasm	other		-1.028777345	0	0.9017808	0	1	0	9.71091987	10.1484287	10.105294	9.69414506
7447	**ENSG0000013782**	ITPKA	protein_coding	chr15	41493393	41503551	+	Human GRCh	OTTHUMG000001	ITPKA	**inositol-trisphosphate 3-kinase A**	Cytoplasm	kinase	GNF362	2.260923785	1	0.37848484	0	NA	0	2.64677791	2.30193046	2.73806888	3.05113183
7448	**ENSG0000013783**	UACA	protein_coding	chr15	70654554	70763558	-	Human GRCh	OTTHUMG000001	UACA	**uveal autoantigen with coiled-coil domains and ankyrin repeat**	Cytoplasm	other		-1.057489479	0	0.83326635	0	1	0	8.83121466	9.26510033	9.15749399	8.83288854
7449	**ENSG0000013783**	SMAD6	protein_coding	chr15	66702236	66782849	+	Human GRCh	OTTHUMG000001	SMAD6	**SMAD family member 6**	Nucleus	transcription		-1.045384481	0	0.87824782	0	1	0	8.93949759	8.91293938	8.87777153	8.87488059

7450	**ENSG0000013784**	PLCB2	protein_coding	chr15	40278176	40307935	-	Human GRCh	OTTHUMG000001	PLCB2	**phospholipase C beta 2**	Cytoplasm	enzyme		-1.322340463	0	0.65762155	0	1	0	3.98897718	3.79498629	3.43223029	3.89631542
7451	**ENSG0000013784**	TMEM62	protein_coding	chr15	43123279	43185144	+	Human GRCh	OTTHUMG000001	TMEM62	**transmembrane protein 62**	Other	enzyme		-1.095656708	0	0.69908674	0	1	0	9.01130074	9.34479648	9.1849268	8.98088843
7452	**ENSG0000013784**	PAK6	protein_coding	chr15	40217428	40277487	+	Human GRCh	OTTHUMG000001	PAK6	**p21 (RAC1) activated kinase 6**	Cytoplasm	kinase		-1.065904601	0	0.79474505	0	1	0	9.22990416	9.78551933	9.45491298	9.48114545
7453	**ENSG0000013784**	ADAM10	protein_coding	chr15	58588809	58749791	-	Human GRCh	OTTHUMG000001	ADAM10	**ADAM metallopeptidase domain 10**	Plasma Mem	peptidase	aderbasib	1.018377047	0	0.93005367	0	1	0	11.1475556	11.3731147	11.4487394	11.096628
7454	**ENSG0000013785**	DUOX1	protein_coding	chr15	45129933	45165576	+	Human GRCh	OTTHUMG000001	DUOX1	**dual oxidase 1**	Plasma Mem	enzyme		1.265258818	0	0.41932407	0	1	0	12.0090242	12.2812601	12.6802934	12.1330736
7456	**ENSG0000013786**	STRA6	protein_coding	chr15	74179466	74212267	-	Human GRCh	OTTHUMG000001	STRA6	**signaling receptor and transporter of retinol STRA6**	Plasma Mem	transporter		10.64709199	1	0.03866837	1	1	0	2.48914445	2.59906494	2.58141341	3.37961379
7458	**ENSG0000013787**	ZNF280D	protein_coding	chr15	56630176	56734086	-	Human GRCh	OTTHUMG000001	ZNF280D	**zinc finger protein 280D**	Other	other		-1.163444112	0	0.5311887	0	1	0	9.4626434	9.67969294	9.54987542	9.25258407
7459	**ENSG0000013787**	SEMA6D	protein_coding	chr15	47184101	47774228	+	Human GRCh	OTTHUMG000001	SEMA6D	**semaphorin 6D**	Plasma Mem	other		-1.34092674	0	0.61942425	0	1	0	9.69110357	10.0592868	9.70054759	9.40257525
7461	**ENSG0000013787**	RSL24D1	protein_coding	chr15	55180806	55197049	-	Human GRCh	OTTHUMG000001	RSL24D1	**ribosomal L24 domain containing 1**	Nucleus	other		1.118317519	0	0.61108019	0	1	0	10.9162562	11.1808581	11.449138	10.836864
7462	**ENSG0000013787**	SPTBN5	protein_coding	chr15	41848146	41894053	-	Human GRCh	OTTHUMG000001	SPTBN5	**spectrin beta, non-erythrocytic 5**	Plasma Mem	other		-1.489034577	0	0.14291992	0	1	0	7.77843845	7.59695449	7.31568491	7.19911607
7463	**ENSG0000013787**	GCOM1	protein_coding	chr15	57591908	57714745	+	Human GRCh	OTTHUMG000001	GCOM1	**GCOM1, MYZAP-POLR2M combined locus**	Other	other		-4.445644709	-1	0.01044596	1	1	0	5.09247099	5.50703522	3.9708513	3.91028716
7464	**ENSG0000013788**	GCHFR	protein_coding	chr15	40764068	40767708	+	Human GRCh	OTTHUMG000001	GCHFR	**GTP cyclohydrolase I feedback regulator**	Cytoplasm	other		1.098990596	0	0.74541055	0	1	0	7.66940072	8.17676424	8.23064749	7.83903755
7465	**ENSG0000013793**	BCAR3	protein_coding	chr1	93561741	93847150	-	Human GRCh	OTTHUMG000000	BCAR3	**BCAR3 adaptor protein, NSP family member**	Cytoplasm	other		-1.221546977	0	0.4685554	0	1	0	7.01201208	7.34121158	7.02996015	6.92311817
7466	**ENSG0000013794**	TTLL7	protein_coding	chr1	83865024	83999150	-	Human GRCh	OTTHUMG000000	TTLL7	**tubulin tyrosine ligase like 7**	Cytoplasm	enzyme		1.024032074	0	0.9308403	0	1	0	8.59506345	8.70181549	8.76972446	8.57017256
7467	**ENSG0000013794**	FNBP1L	protein_coding	chr1	93448118	93554661	+	Human GRCh	OTTHUMG000000	FNBP1L	**formin binding protein 1 like**	Cytoplasm	other		-1.072070975	0	0.73768118	0	1	0	11.4254082	11.6451316	11.6152278	11.28859
7468	**ENSG0000013794**	KYAT3	protein_coding	chr1	88935773	88992953	-	Human GRCh	OTTHUMG000000	KYAT3	**kynurenine aminotransferase 3**	Cytoplasm	enzyme		-1.115552199	0	0.67279044	0	1	0	8.28511417	8.60380649	8.50103758	8.14503986
7469	**ENSG0000013794**	GTF2B	protein_coding	chr1	88852633	88891944	-	Human GRCh	OTTHUMG000000	GTF2B	**general transcription factor IIB**	Nucleus	transcription		-1.141192324	0	0.63182109	0	1	0	8.73146445	9.15489429	8.895359	8.72899713
7471	**ENSG0000013795**	RABGGTB	protein_coding	chr1	75786197	75795086	+	Human GRCh	OTTHUMG000000	RABGGTB	**Rab geranylgeranyltransferase subunit beta**	Cytoplasm	enzyme		-1.077405236	0	0.72972595	0	1	0	10.6069788	10.89839	10.8831066	10.4334523
7472	**ENSG0000013795**	IFI44L	protein_coding	chr1	78619902	78646145	+	Human GRCh	OTTHUMG000000	IFI44L	**interferon induced protein 44 like**	Nucleus	other		-1.796949197	-1	0.75718209	0	1	0	2.15727516	2.5095889	2.0101485	1.91259429
7474	**ENSG0000013796**	ARHGAP29	protein_coding	chr1	94148988	94275068	-	Human GRCh	OTTHUMG000000	ARHGAP29	**Rho GTPase activating protein 29**	Cytoplasm	other		1.026076076	0	0.92765777	0	1	0	8.10572662	8.34237543	8.37088688	8.13084331
7475	**ENSG0000013796**	IFI44	protein_coding	chr1	78649796	78664078	+	Human GRCh	OTTHUMG000000	IFI44	**interferon induced protein 44**	Cytoplasm	other		1	0	1	0	NA	0	1.01527389	1.04308562	1.0384993	1.01690958
7476	**ENSG0000013796**	SLC44A5	protein_coding	chr1	75202129	75611114	-	Human GRCh	OTTHUMG000000	SLC44A5	**solute carrier family 44 member 5**	Plasma Mem	transporter		-4.340587539	-1	0.08458356	0	1	0	5.34839721	4.34112195	4.05233275	4.21822964
7479	**ENSG0000013799**	DBT	protein_coding	chr1	100186919	100249834	-	Human GRCh	OTTHUMG000000	DBT	**dihydrolipoamide branched chain transacylase E2**	Cytoplasm	enzyme		-1.031686052	0	0.87678272	0	1	0	10.3316601	10.5191291	10.5294253	10.241686
7480	**ENSG0000013799**	RTCA	protein_coding	chr1	100266216	100292769	+	Human GRCh	OTTHUMG000000	RTCA	**RNA 3'-terminal phosphate cyclase**	Nucleus	enzyme		-1.159616914	0	0.44321577	0	1	0	9.37580044	9.71640951	9.4614882	9.32463297
7481	**ENSG0000013800**	IFT172	protein_coding	chr2	27444377	27489805	-	Human GRCh	OTTHUMG000001	IFT172	**intraflagellar transport 172**	Extracellular	other		-1.067142895	0	0.77098369	0	1	0	10.5559741	10.8058808	10.8167135	10.3704859
7482	**ENSG0000013801**	SELENOI	protein_coding	chr2	26308547	26395885	+	Human GRCh	OTTHUMG000001	SELENOI	**selenoprotein I**	Cytoplasm	enzyme		-1.002728623	0	0.98974701	0	1	0	12.0573425	12.3446485	12.4160748	11.9518384
7483	**ENSG0000013802**	CGREF1	protein_coding	chr2	27098889	27119128	-	Human GRCh	OTTHUMG000001	CGREF1	**cell growth regulator with EF-hand domain 1**	Extracellular	other		1.02819958	0	0.91741754	0	1	0	9.07202848	9.40090333	9.40238054	9.14451432
7484	**ENSG0000013802**	HADHB	protein_coding	chr2	26243170	26290465	+	Human GRCh	OTTHUMG000000	HADHB	**hydroxyacyl-CoA dehydrogenase trifunctional multienzyme co**	Cytoplasm	enzyme		-1.112197041	0	0.63182697	0	1	0	10.3862296	10.6861867	10.5342632	10.3095035
7485	**ENSG0000013803**	KHK	protein_coding	chr2	27086772	27100762	+	Human GRCh	OTTHUMG000000	KHK	**ketohexokinase**	Cytoplasm	kinase		-1.046213945	0	0.91501126	0	1	0	6.8626622	6.86850675	7.14387768	6.36283188
7486	**ENSG0000013803**	ADCY3	protein_coding	chr2	24819169	24920237	-	Human GRCh	OTTHUMG000000	ADCY3	**adenylate cyclase 3**	Plasma Mem	enzyme		-1.017567643	0	0.93468443	0	1	0	10.2576297	10.5944658	10.4606807	10.375275
7487	**ENSG0000013803**	PPM1B	protein_coding	chr2	44167969	44244384	+	Human GRCh	OTTHUMG000001	PPM1B	**protein phosphatase, Mg2+/Mn2+ dependent 1B**	Cytoplasm	phosphatase		-1.038909709	0	0.8777007	0	1	0	9.25641059	9.74272519	9.68162086	9.24457909
7488	**ENSG0000013803**	PNPT1	protein_coding	chr2	55634061	55693863	-	Human GRCh	OTTHUMG000001	PNPT1	**polyribonucleotide nucleotidyltransferase 1**	Cytoplasm	enzyme		-1.073400796	0	0.76546531	0	1	0	10.9403945	11.2963338	11.1864953	10.9003387
7489	**ENSG0000013803**	DYNC2LI1	protein_coding	chr2	43774039	43810010	+	Human GRCh	OTTHUMG000001	DYNC2LI1	**dynein cytoplasmic 2 light intermediate chain 1**	Cytoplasm	other		-1.136162936	0	0.64218522	0	1	0	7.84723322	8.2973593	7.9396161	7.97212899
7490	**ENSG0000013803**	LHCGR	protein_coding	chr2	48686774	48755730	-	Human GRCh	OTTHUMG000001	LHCGR	**luteinizing hormone/choriogonadotropin receptor**	Plasma Mem	G-protein cou	LH (comple	1	0	1	0	1	0	1.27885778	1.33089234	1.32242485	1.28196567
7491	**ENSG0000013805**	THUMPD2	protein_coding	chr2	39736060	39779267	-	Human GRCh	OTTHUMG000001	THUMPD2	**THUMP domain containing 2**	Other	enzyme		-1.00099261	0	0.99664754	0	1	0	8.41312074	8.65350189	8.65184575	8.41247321
7492	**ENSG0000013806**	CYP1B1	protein_coding	chr2	38066973	38109902	-	Human GRCh	OTTHUMG000001	CYP1B1	**cytochrome P450 family 1 subfamily B member 1**	Cytoplasm	enzyme		1.706523888	1	0.14720187	0	1	0	5.4447336	5.85016097	6.34250027	5.94487226
7494	**ENSG0000013806**	RAB1A	protein_coding	chr2	65070696	65130331	-	Human GRCh	OTTHUMG000001	RAB1A	**RAB1A, member RAS oncogene family**	Cytoplasm	enzyme		-1.055384621	0	0.78725739	0	1	0	11.102698	11.3820179	11.3199069	11.0440611
7495	**ENSG0000013807**	ACTR2	protein_coding	chr2	65227788	65271253	+	Human GRCh	OTTHUMG000001	ACTR2	**actin related protein 2**	Plasma Mem	other		-1.07086819	0	0.75109022	0	1	0	12.3764813	12.659135	12.6150254	12.2579361
7496	**ENSG0000013807**	PREB	protein_coding	chr2	27130756	27134666	-	Human GRCh	OTTHUMG000000	PREB	**prolactin regulatory element binding**	Nucleus	transcription		1.0610811	0	0.78711687	0	1	0	10.7617548	11.0693216	11.1693415	10.7863691
7497	**ENSG0000013807**	SLC5A6	protein_coding	chr2	27199587	27212958	-	Human GRCh	OTTHUMG000000	SLC5A6	**solute carrier family 5 member 6**	Plasma Mem	transporter		-1.097485478	0	0.69186522	0	1	0	10.4984666	10.8457189	10.8142316	10.2828405
7499	**ENSG0000013807**	PREPL	protein_coding	chr2	44316281	44361862	-	Human GRCh	OTTHUMG000001	PREPL	**prolyl endopeptidase like**	Cytoplasm	peptidase		-1.058499843	0	0.79431609	0	1	0	10.5023819	10.8541551	10.7442315	10.5018486
7501	**ENSG0000013808**	EMILIN1	protein_coding	chr2	27078615	27086403	+	Human GRCh	OTTHUMG000000	EMILIN1	**elastin microfibril interfacer 1**	Extracellular	other		1.767879746	1	0.52085465	0	1	0	3.62004076	3.77880366	4.27308628	4.04406788
7502	**ENSG0000013808**	FBXO11	protein_coding	chr2	47789316	47906498	-	Human GRCh	OTTHUMG000001	FBXO11	**F-box protein 11**	Cytoplasm	enzyme		-1.151972395	0	0.47909546	0	1	0	10.528074	10.8128763	10.6630153	10.3841437
7503	**ENSG0000013808**	SIX3	protein_coding	chr2	44941702	44946071	+	Human GRCh	OTTHUMG000001	SIX3	**SIX homeobox 3**	Nucleus	transcription		-1.435639719	0	0.44295132	0	1	0	4.52578392	4.39547044	4.17228831	4.23706977
7504	**ENSG0000013808**	ATRAID	protein_coding	chr2	27212041	27217178	+	Human GRCh	OTTHUMG000001	ATRAID	**all-trans retinoic acid induced differentiation factor**	Plasma Mem	transmembra		-1.248335406	0	0.31394188	0	1	0	10.4863107	10.7569509	10.44983	10.305593
7505	**ENSG0000013809**	CENPO	protein_coding	chr2	24793136	24822376	+	Human GRCh	OTTHUMG000001	CENPO	**centromere protein O**	Nucleus	other		-1.288374071	0	0.20196007	0	1	0	10.8321846	11.2058898	10.8592844	10.631691
7506	**ENSG0000013809**	LRPPRC	protein_coding	chr2	43886224	43996226	-	Human GRCh	OTTHUMG000001	LRPPRC	**leucine rich pentatricopeptide repeat containing**	Cytoplasm	other		-1.024996221	0	0.9049846	0	1	0	12.2624578	12.5749569	12.5701	12.2073325
7507	**ENSG0000013810**	TRIM54	protein_coding	chr2	27282429	27307439	+	Human GRCh	OTTHUMG000000	TRIM54	**tripartite motif containing 54**	Cytoplasm	other		-1.001329644	0	0.99884979	0	1	0	5.9563878	6.35658096	6.1822024	6.16133526
7508	**ENSG0000013810**	DTNB	protein_coding	chr2	25377198	25673647	-	Human GRCh	OTTHUMG000001	DTNB	**dystrobrevin beta**	Plasma Mem	other		-1.221973773	0	0.49767844	0	1	0	9.05075613	9.38207215	9.10230048	8.9238803
7509	**ENSG0000013810**	ACTR1A	protein_coding	chr10	102461881	102502712	-	Human GRCh	OTTHUMG000000	ACTR1A	**actin related protein 1A**	Cytoplasm	other		-1.185889098	0	0.41769127	0	1	0	11.4139222	11.8561501	11.5944906	11.3170405
7510	**ENSG0000013810**	CYP2C9	protein_coding	chr10	94938658	94990091	+	Human GRCh	OTTHUMG000000	CYP2C9	**cytochrome P450 family 2 subfamily C member 9**	Cytoplasm	enzyme		-1.974757903	-1	0.59317008	0	NA	0	2.12929483	1.8228623	1.58905777	1.62018619
7511	**ENSG0000013811**	MFSD13A	protein_coding	chr10	102461395	102477045	+	Human GRCh	OTTHUMG000000	MFSD13A	**major facilitator superfamily domain containing 13A**	Other	other		1.062413271	0	0.80139922	0	1	0	8.17304432	8.71112414	8.5080303	8.56196789
7513	**ENSG0000013811**	MYOF	protein_coding	chr10	93306429	93482334	-	Human GRCh	OTTHUMG000000	MYOF	**myoferlin**	Nucleus	other		-2.033823627	-1	0.0556066	0	1	0	9.890581	9.81581261	9.0600201	9.04103192
7514	**ENSG0000013813**	LOXL4	protein_coding	chr10	98247690	98268194	-	Human GRCh	OTTHUMG000000	LOXL4	**lysyl oxidase like 4**	Extracellular	enzyme		1	0	1	0	NA	0	1.46570484	1.49555381	1.49061721	1.46745458
7515	**ENSG0000013813**	STAMBPL1	protein_coding	chr10	88879734	88975153	+	Human GRCh	OTTHUMG000000	STAMBPL1	**STAM binding protein like 1**	Cytoplasm	enzyme		-1.007204745	0	0.98955473	0	1	0	4.44232509	5.32126971	4.90537007	5.00689898
7517	**ENSG0000013813**	LBX1	protein_coding	chr10	101226994	101229463	-	Human GRCh	OTTHUMG000000	LBX1	**ladybird homeobox 1**	Nucleus	transcription		1	0	1	0	NA	0	-0.402126	-0.3979657	-0.3986613	-0.4018852
7518	**ENSG0000013813**	ATAD1	protein_coding	chr10	87751512	87841361	-	Human GRCh	OTTHUMG000000	ATAD1	**ATPase family AAA domain containing 1**	Plasma Mem	enzyme		-1.019889217	0	0.92844416	0	1	0	10.7527364	11.1052528	11.0856379	10.7259641
7519	**ENSG0000013815**	BTBD16	protein_coding	chr10	122271296	122338159	+	Human GRCh	OTTHUMG000000	BTBD16	**BTB domain containing 16**	Other	other		-1.280400276	0	0.54527743	0	1	0	7.64040924	7.94399843	7.71197313	7.27165637
7520	**ENSG0000013816**	KIF11	protein_coding	chr10	92574105	92655395	+	Human GRCh	OTTHUMG000000	KIF11	**kinesin family member 11**	Nucleus	other	ispinesib, A	-1.168888239	0	0.44900814	0	1	0	11.7689141	12.1081943	11.8771124	11.6672197
7521	**ENSG0000013816**	CUZD1	protein_coding	chr10	122832158	122846175	-	Human GRCh	OTTHUMG000000	CUZD1	**CUB and zona pellucida like domains 1**	Plasma Mem	other		-1.448771381	0	0.39504272	0	1	0	6.0011519	5.86729331	5.8937397	5.16186661
7522	**ENSG0000013816**	TACC2	protein_coding	chr10	121989163	122254545	+	Human GRCh	OTTHUMG000000	TACC2	**transforming acidic coiled-coil containing protein 2**	Nucleus	other		-1.068990321	0	0.7828034	0	1	0	14.513261	14.6978898	14.6603227	14.3849421
7523	**ENSG0000013816**	DUSP5	protein_coding	chr10	110497907	110511533	+	Human GRCh	OTTHUMG000000	DUSP5	**dual specificity phosphatase 5**	Nucleus	phosphatase		-1.329019325	0	0.56851672	0	1	0	4.9052828	4.93014144	4.79939327	4.48344539
7524	**ENSG0000013817**	CALHM2	protein_coding	chr10	103446786	103452402	-	Human GRCh	OTTHUMG000000	CALHM2	**calcium homeostasis modulator family member 2**	Other	other		1.304116647	0	0.59891475	0	1	0	4.2702663	3.84571685	4.15791041	4.38338028
7525	**ENSG0000013817**	ARL3	protein_coding	chr10	102673731	102714397	-	Human GRCh	OTTHUMG000000	ARL3	**ADP ribosylation factor like GTPase 3**	Cytoplasm	enzyme		-1.081601617	0	0.72623121	0	1	0	9.00454904	9.43279564	9.25570241	9.03445692
7526	**ENSG0000013818**	CEP55	protein_coding	chr10	93496612	93529092	+	Human GRCh	OTTHUMG000000	CEP55	**centrosomal protein 55**	Cytoplasm	other		-1.036327884	0	0.863316	0	1	0	9.53953438	9.84498837	9.73507273	9.58676518
7527	**ENSG0000013818**	KIF20B	protein_coding	chr10	89701590	89774939	+	Human GRCh	OTTHUMG000000	KIF20B	**kinesin family member 20B**	Nucleus	enzyme		-1.109136303	0	0.58850843	0	1	0	10.5050637	10.8268593	10.6447452	10.4737643
7528	**ENSG0000013818**	ENTPD1	protein_coding	chr10	95711779	95877266	+	Human GRCh	OTTHUMG000000	ENTPD1	**ectonucleoside triphosphate diphosphohydrolase 1**	Plasma Mem	enzyme	SRF617, ES	5.482388251	1	0.20642214	0	1	0	1.55893916	1.61106929	2.19365695	1.56204396
7529	**ENSG0000013819**	EXOC6	protein_coding	chr10	92826831	93059493	+	Human GRCh	OTTHUMG000000	EXOC6	**exocyst complex component 6**	Plasma Mem	transporter		-1.16973844	0	0.47159337	0	1	0	9.52693017	9.92141256	9.66609242	9.46408813
7530	**ENSG0000013819**	PLCE1	protein_coding	chr10	93993931	94332823	+	Human GRCh	OTTHUMG000000	PLCE1	**phospholipase C epsilon 1**	Cytoplasm	enzyme		7.467261454	1	0.01492785	1	1	0	3.18118209	3.86495351	4.8311162	4.36052561
7531	**ENSG0000013820**	RBP4	protein_coding	chr10	93591687	93601744	-	Human GRCh	OTTHUMG000000	RBP4	**retinol binding protein 4**	Extracellular	other	tinlareban	5.101076643	1	0.38254769	0	NA	0	0.9893781	0.99904406	0.99741912	1.32273241
7532	**ENSG0000013823**	DBR1	protein_coding	chr3	138160988	138174949	-	Human GRCh	OTTHUMG000001	DBR1	**debranching RNA lariats 1**	Nucleus	enzyme		1.061699407	0	0.793696	0	1	0	9.06836413	9.4385191	9.48399822	9.15954427
7533	**ENSG0000013824**	DNAJC13	protein_coding	chr3	132417502	132539032	+	Human GRCh	OTTHUMG000001	DNAJC13	**DnaJ heat shock protein family (Hsp40) member C13**	Cytoplasm	other		-1.178692032	0	0.38996995	0	1	0	11.1535525	11.4477912	11.2278952	11.0210681
7535	**ENSG0000013827**	ANXA7	protein_coding	chr10	73375101	73414076	-	Human GRCh	OTTHUMG000000	ANXA7	**annexin A7**	Plasma Mem	ion channel		-1.132501118	0	0.57767697	0	1	0	11.0680546	11.4673523	11.2592033	11.0227723
7536	**ENSG0000013828**	FAM149B1	protein_coding	chr10	73168119	73244504	+	Human GRCh	OTTHUMG000000	FAM149B1	**family with sequence similarity 149 member B1**	Other	other		-1.176500984	0	0.47885487	0	1	0	9.15600877	9.27608159	9.22269284	8.83124634
7537	**ENSG0000013830**	ASCC1	protein_coding	chr10	72096032	72217134	-	Human GRCh	OTTHUMG000000	ASCC1	**activating signal cointegrator 1 complex subunit 1**	Nucleus	transcription		-1.220508753	0	0.37245823	0	1	0	9.77535559	10.118346	9.84947748	9.61840546
7538	**ENSG0000013830**	PLA2G12B	protein_coding	chr10	72934762	72954806	-	Human GRCh	OTTHUMG000000	PLA2G12B	**phospholipase A2 group XIIB**	Extracellular	enzyme		-1.074815597	0	0.93361507	0	1	0	1.79740011	2.88851434	1.86027899	2.74922059
7539	**ENSG0000013831**	ZNF365	protein_coding	chr10	62374192	62480288	+	Human GRCh	OTTHUMG000001	ZNF365	**zinc finger protein 365**	Cytoplasm	other		-1.010428768	0	0.98532742	0	1	0	4.38387588	4.8371858	4.55633693	4.6826266
7541	**ENSG0000013831**	ADAMTS14	protein_coding	chr10	70672506	70762441	+	Human GRCh	OTTHUMG000000	ADAMTS14	**ADAM metallopeptidase with thrombospondin type 1 motif 14**	Extracellular	peptidase		2.085543217	1	0.50702536	0	1	0	3.37667073	3.5119348	3.00634676	4.22584664
7542	**ENSG0000013832**	RPS24	protein_coding	chr10	78033760	78056813	+	Human GRCh	OTTHUMG000000	RPS24	**ribosomal protein S24**	Cytoplasm	other		-1.079320164	0	0.69196932	0	1	0	13.1097731	13.3516055	13.2479769	13.0466629
7543	**ENSG0000013833**	TET1	protein_coding	chr10	68560337	68694487	+	Human GRCh	OTTHUMG000000	TET1	**tet methylcytosine dioxygenase 1**	Nucleus	enzyme		-1.138793164	0	0.6740198	0	1	0	9.77261534	10.1324636	10.0920802	9.503626
7544	**ENSG0000013834**	DNA2	protein_coding	chr10	68414064	68472121	-	Human GRCh	OTTHUMG000000	DNA2	**DNA replication helicase/nuclease 2**	Cytoplasm	enzyme		1.038894499	0	0.85478223	0	1	0	9.97387563	10.3623796	10.3906084	10.0310415
7547	**ENSG0000013836**	ATIC	protein_coding	chr2	215311956	215349773	+	Human GRCh	OTTHUMG000001	ATIC	**5-aminoimidazole-4-carboxamide ribonucleotide formyltransf**	Cytoplasm	enzyme	gemcitabir	-1.138354191	0	0.5592595	0	1	0	11.6094644	11.9332485	11.7839293	11.4660132
7548	**ENSG0000013837**	SMARCAL1	protein_coding	chr2	216412383	216483053	+	Human GRCh	OTTHUMG000001	SMARCAL1	**SWI/SNF related, matrix associated, actin dependent regulato**	Nucleus	enzyme		-1.181357854	0	0.42219698	0	1	0	9.36292686	9.60945262	9.48241343	9.13040992

7549	**ENSG0000013837**	BARD1	protein_coding	chr2	214725646	214809683	-	Human GRCh	OTTHUMG000001	BARD1	**BRCA1 associated RING domain 1**	Nucleus	transcription		-1.041066459	0	0.85117154	0	1	0	10.9923415	11.3251124	11.346349	10.8569503
7550	**ENSG0000013837**	STAT4	protein_coding	chr2	191029576	191151596	-	Human GRCh	OTTHUMG000001	STAT4	**signal transducer and activator of transcription 4**	Nucleus	transcription		-1.158007966	0	0.81271237	0	1	0	4.4653097	3.2916191	3.57700452	4.19972473
7551	**ENSG0000013837**	MSTN	protein_coding	chr2	190055700	190062729	-	Human GRCh	OTTHUMG000001	MSTN	**myostatin**	Extracellular	growth facto	stamulum:	-1.164928844	0	0.84622488	0	1	0	3.98572889	4.5301992	4.49981921	3.64938964
7552	**ENSG0000013838**	CARF	protein_coding	chr2	202912214	202988263	+	Human GRCh	OTTHUMG000001	CARF	**calcium responsive transcription factor**	Nucleus	transcription		-1.319426907	0	0.25135103	0	1	0	9.11860389	9.13040227	9.01768793	8.60281103
7553	**ENSG0000013838**	ASNSD1	protein_coding	chr2	189661385	189670831	+	Human GRCh	OTTHUMG000001	ASNSD1	**asparagine synthetase domain containing 1**	Other	enzyme		1.023357047	0	0.91352505	0	1	0	9.02010667	9.28554161	9.32954258	9.02190475
7554	**ENSG0000013838**	METTL5	protein_coding	chr2	169810081	169824931	-	Human GRCh	OTTHUMG000001	METTL5	**methyltransferase 5, N6-adenosine**	Nucleus	enzyme		1.07002428	0	0.76526702	0	1	0	7.86591783	8.38726904	8.26370849	8.18836547
7555	**ENSG0000013838**	SSB	protein_coding	chr2	169791933	169812064	+	Human GRCh	OTTHUMG000001	SSB	**small RNA binding exonuclease protection factor La**	Nucleus	enzyme		1.015742743	0	0.94280142	0	1	0	9.82551736	10.20934	10.1374059	9.95587994
7556	**ENSG0000013838**	NAB1	protein_coding	chr2	190646746	190692766	+	Human GRCh	OTTHUMG000001	NAB1	**NGFI-A binding protein 1**	Nucleus	transcription		1.180716551	0	0.38730398	0	1	0	9.66122926	10.0510394	10.1090626	9.98452249
7558	**ENSG0000013839**	PPIG	protein_coding	chr2	169584342	169641406	+	Human GRCh	OTTHUMG000001	PPIG	**peptidylprolyl isomerase G**	Nucleus	enzyme		1.104818344	0	0.62724853	0	1	0	9.97684259	10.3298695	10.4235213	10.1028393
7559	**ENSG0000013839**	FASTKD1	protein_coding	chr2	169528508	169573875	-	Human GRCh	OTTHUMG000001	FASTKD1	**FAST kinase domains 1**	Cytoplasm	other		1.120492784	0	0.62608979	0	1	0	8.86031777	9.05856868	9.25597643	8.88506676
7560	**ENSG0000013840**	MDH1B	protein_coding	chr2	206737763	206765328	-	Human GRCh	OTTHUMG000001	MDH1B	**malate dehydrogenase 1B**	Other	enzyme		1.091951021	0	0.81055716	0	1	0	5.86345645	6.03416776	6.35067732	5.60825088
7561	**ENSG0000013841**	HECW2	protein_coding	chr2	196189099	196593684	-	Human GRCh	OTTHUMG000001	HECW2	**HECT, C2 and WW domain containing E3 ubiquitin protein ligas**	Extracellular	enzyme		1.236438307	0	0.69815838	0	1	0	5.38841759	4.78517835	5.22138442	5.36059219
7562	**ENSG0000013841**	IDH1	protein_coding	chr2	208236229	208266074	-	Human GRCh	OTTHUMG000001	IDH1	**isocitrate dehydrogenase (NADP(+)) 1**	Cytoplasm	enzyme	olutasiden	-1.169463284	0	0.50914413	0	1	0	10.5312234	10.9923199	10.7786366	10.4064157
7563	**ENSG0000013843**	OLA1	protein_coding	chr2	174072447	174248599	-	Human GRCh	OTTHUMG000001	OLA1	**Obg like ATPase 1**	Cytoplasm	enzyme		-1.014485483	0	0.94711206	0	1	0	10.300069	10.5774273	10.5469606	10.3026991
7564	**ENSG0000013843**	CIR1	protein_coding	chr2	174348022	174395712	-	Human GRCh	OTTHUMG000001	CIR1	**corepressor interacting with RBPJ, CIR1**	Nucleus	transcription		-1.188077657	0	0.45043633	0	1	0	8.03484218	8.22244275	7.87202803	8.0190517
7565	**ENSG0000013843**	ITPRID2	protein_coding	chr2	181891730	181930738	+	Human GRCh	OTTHUMG000001	ITPRID2	**ITPR interacting domain containing 2**	Plasma Mem	other		1.021607321	0	0.93039123	0	1	0	10.713646	10.9438303	11.0187637	10.6708902
7566	**ENSG0000013843**	CHRNA1	protein_coding	chr2	174747592	174787935	-	Human GRCh	OTTHUMG000001	CHRNA1	**cholinergic receptor nicotinic alpha 1 subunit**	Plasma Mem	transmembra	succinylch	1	0	1	0	NA	0	1.03044013	1.04937472	1.04622409	1.03154228
7567	**ENSG0000013843**	FAM117B	protein_coding	chr2	202634969	202769757	+	Human GRCh	OTTHUMG000001	FAM117B	**family with sequence similarity 117 member B**	Other	other		-1.191587492	0	0.40976401	0	1	0	10.5746391	10.9482409	10.6598791	10.4960975
7568	**ENSG0000013844**	WDR12	protein_coding	chr2	202874261	203014798	-	Human GRCh	OTTHUMG000001	WDR12	**WD repeat domain 12**	Cytoplasm	other		-1.050723899	0	0.81421651	0	1	0	10.8048891	11.2581207	11.1539833	10.8168181
7569	**ENSG0000013844**	ABI2	protein_coding	chr2	203328280	203447728	+	Human GRCh	OTTHUMG000001	ABI2	**abl interactor 2**	Cytoplasm	other		1.006634294	0	0.97779506	0	1	0	11.4094807	11.5758091	11.6941003	11.2782951
7570	**ENSG0000013844**	ITGAV	protein_coding	chr2	186590010	186680901	+	Human GRCh	OTTHUMG000001	ITGAV	**integrin subunit alpha V**	Plasma Mem	transmembra	abciximab,	-1.358746272	0	0.2771158	0	1	0	12.6411948	13.1689601	12.974497	11.9955344
7571	**ENSG0000013844**	SLC40A1	protein_coding	chr2	189560590	189583758	-	Human GRCh	OTTHUMG000001	SLC40A1	**solute carrier family 40 member 1**	Plasma Mem	transporter		1.463499501	0	0.53585036	0	1	0	5.68843352	5.66673918	6.39668439	5.59259275
7572	**ENSG0000013845**	SLC35A5	protein_coding	chr3	112561709	112585579	+	Human GRCh	OTTHUMG000001	SLC35A5	**solute carrier family 35 member A5**	Cytoplasm	transporter		-1.10088891	0	0.69962787	0	1	0	8.79421929	9.08678771	9.05505243	8.59211863
7573	**ENSG0000013846**	SLC49A4	protein_coding	chr3	122795069	122881139	+	Human GRCh	OTTHUMG000001	SLC49A4	**solute carrier family 49 member 4**	Cytoplasm	other		-1.338966169	0	0.18810182	0	1	0	9.29396087	9.58569555	9.21426129	9.0359684
7574	**ENSG0000013846**	SENP7	protein_coding	chr3	101324205	101513241	-	Human GRCh	OTTHUMG000001	SENP7	**SUMO specific peptidase 7**	Nucleus	peptidase		-1.043160521	0	0.85247348	0	1	0	9.81917217	10.0737292	10.0788659	9.70672352
7577	**ENSG0000013849**	COX17	protein_coding	chr3	119654513	119677454	-	Human GRCh	OTTHUMG000001	COX17	**cytochrome c oxidase copper chaperone COX17**	Cytoplasm	enzyme		1.005953715	0	0.97633819	0	1	0	8.541544	8.66335392	8.59833172	8.62153218
7578	**ENSG0000013849**	PARP9	protein_coding	chr3	122527924	122564577	-	Human GRCh	OTTHUMG000001	PARP9	**poly(ADP-ribose) polymerase family member 9**	Nucleus	enzyme		-1.130332933	0	0.6494608	0	1	0	8.62364573	8.91650071	8.91760457	8.29395453
7579	**ENSG0000013858**	MNS1	protein_coding	chr15	56421544	56465137	-	Human GRCh	OTTHUMG000001	MNS1	**meiosis specific nuclear structural 1**	Cytoplasm	other		1.181425449	0	0.5491488	0	1	0	7.375146	7.90462052	7.90757053	7.7790305
7580	**ENSG0000013859**	USP8	protein_coding	chr15	50424380	50514421	+	Human GRCh	OTTHUMG000001	USP8	**ubiquitin specific peptidase 8**	Cytoplasm	peptidase		-1.081835883	0	0.69023543	0	1	0	11.0423803	11.3418948	11.2388488	10.9734441
7581	**ENSG0000013859**	SECISBP2L	protein_coding	chr15	48988476	49046447	-	Human GRCh	OTTHUMG000001	SECISBP2L	**SECIS binding protein 2 like**	Other	other		-1.086522359	0	0.68176783	0	1	0	10.7958337	11.0240499	10.9714071	10.6540257
7582	**ENSG0000013859**	TMOD3	protein_coding	chr15	51829628	51947295	+	Human GRCh	OTTHUMG000001	TMOD3	**tropomodulin 3**	Cytoplasm	other		-1.077518251	0	0.70686631	0	1	0	11.2294826	11.5376714	11.4860025	11.1038151
7583	**ENSG0000013860**	SPPL2A	protein_coding	chr15	50702266	50765948	-	Human GRCh	OTTHUMG000001	SPPL2A	**signal peptide peptidase like 2A**	Plasma Mem	peptidase		1.000758773	0	0.99701433	0	1	0	10.0284325	10.1510039	10.2869368	9.85983499
7584	**ENSG0000013860**	GLCE	protein_coding	chr15	69160584	69272217	+	Human GRCh	OTTHUMG000001	GLCE	**glucuronic acid epimerase**	Cytoplasm	enzyme		1.021910464	0	0.92857694	0	1	0	8.59365444	8.87104701	8.89044215	8.62239257
7585	**ENSG0000013860**	SHF	protein_coding	chr15	45167214	45201175	-	Human GRCh	OTTHUMG000001	SHF	**Src homology 2 domain containing F**	Nucleus	other		-1.195819998	0	0.58768843	0	1	0	7.14744637	7.28248233	7.22601719	6.79182623
7586	**ENSG0000013861**	APH1B	protein_coding	chr15	63276018	63309126	+	Human GRCh	OTTHUMG000001	APH1B	**aph-1 homolog B, gamma-secretase subunit**	Cytoplasm	peptidase		-1.083110704	0	0.79564552	0	1	0	6.23541293	6.52414634	6.56795678	5.98162881
7587	**ENSG0000013861**	INTS14	protein_coding	chr15	65578753	65611289	-	Human GRCh	OTTHUMG000001	INTS14	**integrator complex subunit 14**	Cytoplasm	other		-1.210796808	0	0.35323449	0	1	0	9.72686559	9.88128677	9.75414736	9.41110472
7588	**ENSG0000013861**	CILP	protein_coding	chr15	65194760	65211473	-	Human GRCh	OTTHUMG000001	CILP	**cartilage intermediate layer protein**	Extracellular	phosphatase		1.145765331	0	0.89015414	0	NA	0	2.96726747	3.10845968	3.61075489	2.16930923
7589	**ENSG0000013861**	PARP16	protein_coding	chr15	65234460	65300618	-	Human GRCh	OTTHUMG000001	PARP16	**poly(ADP-ribose) polymerase family member 16**	Cytoplasm	enzyme		1.022112946	0	0.93328576	0	1	0	8.20520902	8.44102049	8.41688353	8.28487124
7590	**ENSG0000013862**	PPCDC	protein_coding	chr15	75023586	75117462	+	Human GRCh	OTTHUMG000001	PPCDC	**phosphopantothenoylcysteine decarboxylase**	Cytoplasm	enzyme		-1.115520784	0	0.61232704	0	1	0	9.10408821	9.30452383	9.21503018	8.95035357
7591	**ENSG0000013862**	HCN4	protein_coding	chr15	73319859	73368958	-	Human GRCh	OTTHUMG000001	HCN4	**hyperpolarization activated cyclic nucleotide gated potassium**	Plasma Mem	ion channel		-1.006538576	0	0.99501179	0	1	0	2.59126823	2.09126738	2.07404754	2.60033319
7592	**ENSG0000013862**	SEMA7A	protein_coding	chr15	74409289	74433958	-	Human GRCh	OTTHUMG000001	SEMA7A	**semaphorin 7A (John Milton Hagen blood group)**	Plasma Mem	transmembra		2.101580891	1	0.13296334	0	1	0	5.56976202	5.66778763	6.43378687	6.3630649
7593	**ENSG0000013862**	UBL7	protein_coding	chr15	74445977	74461182	-	Human GRCh	OTTHUMG000001	UBL7	**ubiquitin like 7**	Cytoplasm	other		-1.038018842	0	0.87295592	0	1	0	9.56234822	9.77375025	9.62442702	9.63722919
7594	**ENSG0000013863**	ARHGAP24	protein_coding	chr4	85475150	86002668	+	Human GRCh	OTTHUMG000001	ARHGAP24	**Rho GTPase activating protein 24**	Cytoplasm	other		-1.040731285	0	0.94449751	0	1	0	7.12371287	7.06504277	6.74058804	7.29876308
7595	**ENSG0000013864**	FAM13A	protein_coding	chr4	88725955	89111398	-	Human GRCh	OTTHUMG000001	FAM13A	**family with sequence similarity 13 member A**	Cytoplasm	other		-1.057827476	0	0.90362972	0	1	0	8.86476918	9.04451211	8.99986438	8.79307505
7596	**ENSG0000013864**	HERC3	protein_coding	chr4	88592434	88708541	+	Human GRCh	OTTHUMG000001	HERC3	**HECT and RLD domain containing E3 ubiquitin protein ligase 3**	Cytoplasm	enzyme		-1.010728115	0	0.96679549	0	1	0	10.4533185	11.0893387	10.8332363	10.7675059
7597	**ENSG0000013864**	HERC6	protein_coding	chr4	88378739	88443097	+	Human GRCh	OTTHUMG000001	HERC6	**HECT and RLD domain containing E3 ubiquitin protein ligase fa**	Cytoplasm	enzyme		-1.304652387	0	0.60010258	0	1	0	6.26842849	6.71504659	6.31158518	6.15667596
7598	**ENSG0000013864**	HERC5	protein_coding	chr4	88457119	88506163	+	Human GRCh	OTTHUMG000001	HERC5	**HECT and RLD domain containing E3 ubiquitin protein ligase 5**	Cytoplasm	enzyme		-1.599536056	-1	0.2097621	0	1	0	6.99094671	7.50999922	6.83738894	6.71271858
7599	**ENSG0000013865**	PCDH10	protein_coding	chr4	133149294	133208606	+	Human GRCh	OTTHUMG000001	PCDH10	**protocadherin 10**	Plasma Mem	other		1.064903803	0	0.90978668	0	1	0	4.82533404	5.20048777	5.16608742	5.01532277
7600	**ENSG0000013865**	NDST4	protein_coding	chr4	114827763	115113876	-	Human GRCh	OTTHUMG000001	NDST4	**N-deacetylase and N-sulfotransferase 4**	Cytoplasm	enzyme		-1.960076666	-1	0.56340245	0	1	0	2.70998889	2.85266114	2.82983751	2.14303927
7601	**ENSG0000013865**	ZGRF1	protein_coding	chr4	112539333	112636995	-	Human GRCh	OTTHUMG000001	ZGRF1	**zinc finger GRF-type containing 1**	Nucleus	other		-1.196411685	0	0.41160737	0	1	0	10.4667734	10.7113561	10.5206106	10.2546873
7602	**ENSG0000013866**	AP1AR	protein_coding	chr4	112231740	112273110	+	Human GRCh	OTTHUMG000001	AP1AR	**adaptor related protein complex 1 associated regulatory prote**	Cytoplasm	other		1.153690521	0	0.49725584	0	1	0	9.78880475	10.0036028	10.231635	9.8597699
7603	**ENSG0000013866**	COPS4	protein_coding	chr4	83034447	83075818	+	Human GRCh	OTTHUMG000001	COPS4	**COP9 signalosome subunit 4**	Cytoplasm	peptidase		-1.112240778	0	0.65376	0	1	0	9.62405996	9.98191216	9.72822072	9.66396612
7604	**ENSG0000013866**	HNRNPD	protein_coding	chr4	82352498	82374503	-	Human GRCh	OTTHUMG000001	HNRNPD	**heterogeneous nuclear ribonucleoprotein D**	Nucleus	transcription		1.06996928	0	0.74681502	0	1	0	12.2339225	12.5767293	12.6270579	12.3417094
7605	**ENSG0000013866**	PRKG2	protein_coding	chr4	81087370	81215222	-	Human GRCh	OTTHUMG000001	PRKG2	**protein kinase cGMP-dependent 2**	Cytoplasm	kinase		1.333444969	0	0.57564529	0	1	0	4.85981595	4.59538338	4.81473768	5.12228013
7606	**ENSG0000013867**	RASGEF1B	protein_coding	chr4	81426393	82044244	-	Human GRCh	OTTHUMG000001	RASGEF1B	**RasGEF domain family member 1B**	Other	other		-1.355801549	0	0.43346759	0	1	0	8.90461311	9.10927324	8.5228368	8.80795163
7607	**ENSG0000013867**	SEC31A	protein_coding	chr4	82818509	82901166	-	Human GRCh	OTTHUMG000001	SEC31A	**SEC31 homolog A, COPII coat complex component**	Cytoplasm	other		-1.105002782	0	0.6497486	0	1	0	11.8245159	12.109917	12.0083932	11.6983291
7609	**ENSG0000013867**	GPAT3	protein_coding	chr4	83535914	83605875	+	Human GRCh	OTTHUMG000001	GPAT3	**glycerol-3-phosphate acyltransferase 3**	Cytoplasm	enzyme		-1.133345728	0	0.83272865	0	1	0	4.62769459	4.61514509	4.50518407	4.50808166
7611	**ENSG0000013868**	FGF2	protein_coding	chr4	122826682	122898236	+	Human GRCh	OTTHUMG000000	FGF2	**fibroblast growth factor 2**	Extracellular	growth facto	pentosan p	4.101072675	1	0.17467225	0	1	0	2.56186937	2.22940135	2.91433768	2.83035345
7612	**ENSG0000013868**	BBS7	protein_coding	chr4	121824329	121870487	-	Human GRCh	OTTHUMG000001	BBS7	**Bardet-Biedl syndrome 7**	Cytoplasm	other		-1.012573444	0	0.95657788	0	1	0	8.70437778	8.90767601	8.98862388	8.56750016
7613	**ENSG0000013868**	BLTP1	protein_coding	chr4	122152331	122364167	+	Human GRCh	OTTHUMG000001	BLTP1	**bridge-like lipid transfer protein family member 1**	Cytoplasm	transporter		-1.121760989	0	0.59016194	0	1	0	12.4776576	12.6604163	12.5573052	12.3235831
7614	**ENSG0000013869**	BMPR1B	protein_coding	chr4	94757955	95158448	+	Human GRCh	OTTHUMG000001	BMPR1B	**bone morphogenetic protein receptor type 1B**	Plasma Mem	kinase	LDN-19318	1.045039578	0	0.92759013	0	1	0	7.93616061	8.29556988	8.36174265	7.94896321
7615	**ENSG0000013869**	RAP1GDS1	protein_coding	chr4	98261384	98443858	+	Human GRCh	OTTHUMG000001	RAP1GDS1	**Rap1 GTPase-GDP dissociation stimulator 1**	Cytoplasm	other		-1.120640367	0	0.63073404	0	1	0	10.0810942	10.4387662	10.2835162	9.99216415
7616	**ENSG0000013870**	LARP1B	protein_coding	chr4	128061286	128222931	+	Human GRCh	OTTHUMG000001	LARP1B	**La ribonucleoprotein 1B**	Extracellular	other		1.020874197	0	0.92583068	0	1	0	11.1840531	11.4509244	11.4771373	11.2040336
7618	**ENSG0000013873**	PDE5A	protein_coding	chr4	119494397	119628804	-	Human GRCh	OTTHUMG000001	PDE5A	**phosphodiesterase 5A**	Cytoplasm	enzyme	pentoxifyll	1.293859297	0	0.49865656	0	1	0	7.35791871	6.60399006	7.07776901	7.47929747
7619	**ENSG0000013873**	PRDM5	protein_coding	chr4	120684919	120922870	-	Human GRCh	OTTHUMG000001	PRDM5	**PR/SET domain 5**	Nucleus	transcription		1.075401996	0	0.82785828	0	1	0	6.33931096	6.48113656	6.6335578	6.30795843
7620	**ENSG0000013874**	TRPC3	protein_coding	chr4	121874481	121952060	-	Human GRCh	OTTHUMG000001	TRPC3	**transient receptor potential cation channel subfamily C memb**	Plasma Mem	ion channel		6.446077113	1	0.02821478	1	1	0	3.2229757	2.86446756	3.64485955	4.04444119
7621	**ENSG0000013874**	NAAA	protein_coding	chr4	75913660	75941013	-	Human GRCh	OTTHUMG000001	NAAA	**N-acylethanolamine acid amidase**	Cytoplasm	enzyme		-1.03074695	0	0.92861572	0	1	0	8.81199683	9.41741876	9.3443456	8.83736509
7622	**ENSG0000013875**	NUP54	protein_coding	chr4	76107562	76148485	-	Human GRCh	OTTHUMG000001	NUP54	**nucleoporin 54**	Nucleus	other		-1.07035916	0	0.75078461	0	1	0	10.3072189	10.5392298	10.4869894	10.2056835
7624	**ENSG0000013875**	BMP2K	protein_coding	chr4	78776342	78916365	+	Human GRCh	OTTHUMG000001	BMP2K	**BMP2 inducible kinase**	Nucleus	kinase	LP-935509	1.123608205	0	0.61676242	0	1	0	9.63617612	9.68664255	9.97877659	9.56408308
7625	**ENSG0000013875**	G3BP2	protein_coding	chr4	75641849	75724589	-	Human GRCh	OTTHUMG000001	G3BP2	**G3BP stress granule assembly factor 2**	Cytoplasm	enzyme		1.018981602	0	0.93007657	0	1	0	12.1702579	12.4036198	12.5001303	12.0924768
7626	**ENSG0000013875**	SEPTIN11	protein_coding	chr4	76949751	77040384	+	Human GRCh	OTTHUMG000001	SEPTIN11	**septin 11**	Nucleus	other		1.080725639	0	0.70158178	0	1	0	11.416449	11.7047893	11.8352036	11.4314767
7627	**ENSG0000013875**	FRAS1	protein_coding	chr4	78057323	78544269	+	Human GRCh	OTTHUMG000001	FRAS1	**Fraser extracellular matrix complex subunit 1**	Extracellular	other		-1.149303706	0	0.57193216	0	1	0	11.1952606	11.294136	11.0001007	11.2035239
7628	**ENSG0000013876**	SCARB2	protein_coding	chr4	76158737	76234536	-	Human GRCh	OTTHUMG000001	SCARB2	**scavenger receptor class B member 2**	Plasma Mem	transmembra		-1.069890977	0	0.73189697	0	1	0	11.4082632	11.6680278	11.5601137	11.377687
7629	**ENSG0000013876**	CCNG2	protein_coding	chr4	77157207	77433388	+	Human GRCh	OTTHUMG000001	CCNG2	**cyclin G2**	Nucleus	other		-1.363792853	0	0.18108714	0	1	0	9.9213223	10.2115613	9.8586931	9.62221006
7630	**ENSG0000013876**	CNOT6L	protein_coding	chr4	77713387	77819615	-	Human GRCh	OTTHUMG000001	CNOT6L	**CCR4-NOT transcription complex subunit 6 like**	Cytoplasm	enzyme		1.046566191	0	0.85026423	0	1	0	10.8586165	11.1989516	11.3331476	10.7858283
7631	**ENSG0000013876**	USO1	protein_coding	chr4	75724577	75814286	+	Human GRCh	OTTHUMG000001	USO1	**USO1 vesicle transport factor**	Cytoplasm	other		-1.123341636	0	0.59857729	0	1	0	11.5882264	11.8862103	11.763272	11.4451153
7632	**ENSG0000013876**	CDKL2	protein_coding	chr4	75576496	75630716	-	Human GRCh	OTTHUMG000001	CDKL2	**cyclin dependent kinase like 2**	Nucleus	kinase		-1.838525987	-1	0.25892781	0	1	0	4.08330342	3.575625	3.33212599	3.41669259
7633	**ENSG0000013877**	SHROOM3	protein_coding	chr4	76435229	76783253	+	Human GRCh	OTTHUMG000001	SHROOM3	**shroom family member 3**	Cytoplasm	other		-1.004283745	0	0.98512301	0	1	0	11.9866624	12.1559063	12.1556229	11.9764921
7634	**ENSG0000013877**	ANXA3	protein_coding	chr4	78551747	78610451	+	Human GRCh	OTTHUMG000001	ANXA3	**annexin A3**	Cytoplasm	enzyme		3.647799094	1	0.11592547	0	1	0	2.3424913	2.44415489	2.90342172	3.26820002
7635	**ENSG0000013877**	PPA2	protein_coding	chr4	105369077	105474067	-	Human GRCh	OTTHUMG000001	PPA2	**inorganic pyrophosphatase 2**	Cytoplasm	enzyme		-1.000162215	0	0.99940847	0	1	0	10.8583685	11.1255535	11.1902795	10.7713894
7636	**ENSG0000013877**	CENPE	protein_coding	chr4	103105349	103198445	-	Human GRCh	OTTHUMG000001	CENPE	**centromere protein E**	Nucleus	other	GSK92329	-1.062060149	0	0.74539045	0	1	0	11.187208	11.463116	11.3189099	11.2122938
7637	**ENSG0000013878**	GSTCD	protein_coding	chr4	105708778	105847725	+	Human GRCh	OTTHUMG000001	GSTCD	**glutathione S-transferase C-terminal domain containing**	Cytoplasm	enzyme		-1.024310111	0	0.9233122	0	1	0	10.6852726	11.082348	11.0466295	10.6692635
7638	**ENSG0000013878**	INTS12	protein_coding	chr4	105682627	105895986	-	Human GRCh	OTTHUMG000001	INTS12	**integrator complex subunit 12**	Nucleus	other		-1.059039254	0	0.81391511	0	1	0	9.43468225	9.82564208	9.70695149	9.4405448

7639	ENSG0000013879	ENPEP	protein_coding	chr4	110365733	110565285	+	Human GRCh	OTTHUMG000001	ENPEP	glutamyl aminopeptidase	Plasma Mem	peptidase		1.045122185	0	0.9221219	0	1	0	5.97062514	5.96175115	6.14384398	5.86480848
7640	ENSG0000013879	CASP6	protein_coding	chr4	109688622	109703583	-	Human GRCh	OTTHUMG000001	CASP6	caspase 6	Cytoplasm	peptidase		1.038437734	0	0.86967191	0	1	0	8.90571374	9.29268786	9.34839315	8.92761865
7641	ENSG0000013879	LEF1	protein_coding	chr4	108047545	108168956	-	Human GRCh	OTTHUMG000001	LEF1	lymphoid enhancer binding factor 1	Nucleus	transcription		1.349976327	0	0.27610569	0	1	0	8.33860497	8.24524016	8.42023859	8.75705421
7642	ENSG0000013879	HADH	protein_coding	chr4	107989714	108035241	+	Human GRCh	OTTHUMG000001	HADH	hydroxyacyl-CoA dehydrogenase	Cytoplasm	enzyme		-1.09594234	0	0.68272741	0	1	0	10.5838882	10.8371721	10.800737	10.3956327
7643	ENSG0000013879	EGF	protein_coding	chr4	109912883	110013766	+	Human GRCh	OTTHUMG000001	EGF	epidermal growth factor	Extracellular	growth facto		-1.053295208	0	0.94691571	0	1	0	2.99961356	2.89250289	2.06782873	3.35122714
7644	ENSG0000013880	PAPSS1	protein_coding	chr4	107590276	107720234	-	Human GRCh	OTTHUMG000001	PAPSS1	3'-phosphoadenosine 5'-phosphosulfate synthase 1	Cytoplasm	enzyme		-1.084633612	0	0.69632924	0	1	0	10.015026	10.3088581	10.1943456	9.95214333
7645	ENSG0000013880	SEC24B	protein_coding	chr4	109433772	109540896	+	Human GRCh	OTTHUMG000001	SEC24B	SEC24 homolog B, COPII coat complex component	Cytoplasm	transporter		-1.024603537	0	0.90358857	0	1	0	11.2078392	11.5967302	11.4438761	11.3341382
7647	ENSG0000013881	PPP3CA	protein_coding	chr4	101023409	101348278	-	Human GRCh	OTTHUMG000001	PPP3CA	protein phosphatase 3 catalytic subunit alpha	Cytoplasm	phosphatase	voclospori	-1.088080174	0	0.67371322	0	1	0	11.8561432	12.0779684	11.9982929	11.7399574
7648	ENSG0000013882	SLC39A8	protein_coding	chr4	102251080	102431258	-	Human GRCh	OTTHUMG000001	SLC39A8	solute carrier family 39 member 8	Extracellular	transporter		-1.105250588	0	0.68319753	0	1	0	9.5115119	10.0526401	9.73143788	9.66047452
7649	ENSG0000013882	MTTP	protein_coding	chr4	99564081	99623997	+	Human GRCh	OTTHUMG000001	MTTP	microsomal triglyceride transfer protein	Cytoplasm	transporter	lomitapide	1.402588481	0	0.53511573	0	1	0	4.01985744	4.27255228	4.53121298	4.32774568
7650	ENSG0000013882	FBN2	protein_coding	chr5	128257909	128659185	-	Human GRCh	OTTHUMG000001	FBN2	fibrillin 2	Extracellular	other		-1.181556047	0	0.78373068	0	1	0	7.40478621	7.8070752	7.43490069	7.4241002
7651	ENSG0000013883	MAPK8IP3	protein_coding	chr16	1706166	1770351	+	Human GRCh	OTTHUMG000001	MAPK8IP3	mitogen-activated protein kinase 8 interacting protein 3	Cytoplasm	other		-1.018483179	0	0.94093874	0	1	0	12.0415522	12.360402	12.2986648	12.0728625
7652	ENSG0000013883	RGS3	protein_coding	chr9	113444731	113597743	+	Human GRCh	OTTHUMG000000	RGS3	regulator of G protein signaling 3	Nucleus	enzyme		-1.13496648	0	0.58293597	0	1	0	10.505504	10.776312	10.656519	10.3498437
7653	ENSG0000013886	GUCD1	protein_coding	chr22	24540423	24555935	-	Human GRCh	OTTHUMG000001	GUCD1	guanylyl cyclase domain containing 1	Other	other		-1.241832312	0	0.39032065	0	1	0	11.3438104	11.7630936	11.4558785	11.1933817
7654	ENSG0000013889	TTLL8	protein_coding	chr22	50018575	50058298	-	Human GRCh	OTTHUMG000001	TTLL8	tubulin tyrosine ligase like 8	Extracellular	enzyme		1.450011571	0	0.83592076	0	1	0	2.82833627	1.70698939	2.81923244	2.58999429
7655	ENSG0000013894	RNF185	protein_coding	chr22	31160182	31207019	+	Human GRCh	OTTHUMG000001	RNF185	ring finger protein 185	Cytoplasm	enzyme		-1.130950086	0	0.5535481	0	1	0	9.57849535	9.85268063	9.60230872	9.57100071
7656	ENSG0000013894	SHISAL1	protein_coding	chr22	44243665	44312951	-	Human GRCh	OTTHUMG000000	SHISAL1	shisa like 1	Other	other		37.05956634	1	0.00095135	1	0.6	0	3.26592519	3.34894426	4.04847014	4.49173709
7657	ENSG0000013896	PARVG	protein_coding	chr22	44172956	44219533	+	Human GRCh	OTTHUMG000001	PARVG	parvin gamma	Cytoplasm	other		-1.437618598	0	0.44063904	0	1	0	5.26755682	5.56485859	4.94410732	5.19912317
7658	ENSG0000013904	B4GALNT3	protein_coding	chr12	459939	563509	+	Human GRCh	OTTHUMG000001	B4GALNT3	beta-1,4-N-acetyl-galactosaminyltransferase 3	Cytoplasm	enzyme		1.431776796	0	0.45794114	0	1	0	4.96463069	4.94090652	5.5252348	4.88327757
7661	ENSG0000013908	ETV6	protein_coding	chr12	11649674	11895377	+	Human GRCh	OTTHUMG000001	ETV6	ETS variant transcription factor 6	Nucleus	transcription		-1.181695831	0	0.5922685	0	1	0	9.71870424	9.96491184	9.66935079	9.67458489
7662	ENSG0000013911	GABARAPL	protein_coding	chr12	10212458	10223128	+	Human GRCh	OTTHUMG000001	GABARAPL1	GABA type A receptor associated protein like 1	Cytoplasm	other		1.160379195	0	0.57078351	0	1	0	7.98423358	7.99888661	7.87039103	8.38650473
7663	ENSG0000013911	KIF21A	protein_coding	chr12	39293228	39443390	-	Human GRCh	OTTHUMG000001	KIF21A	kinesin family member 21A	Cytoplasm	other		-1.075756956	0	0.68509591	0	1	0	11.0986507	11.4046672	11.3499501	10.981713
7664	ENSG0000013911	CPNE8	protein_coding	chr12	38646822	38907430	-	Human GRCh	OTTHUMG000001	CPNE8	copine 8	Cytoplasm	other		2.696535937	1	0.49710896	0	1	0	1.78659344	1.88774114	2.25530033	1.79274788
7665	ENSG0000013913	YARS2	protein_coding	chr12	32727490	32755897	-	Human GRCh	OTTHUMG000001	YARS2	tyrosyl-tRNA synthetase 2	Cytoplasm	enzyme		-1.043889166	0	0.85895014	0	1	0	9.51194432	9.75120024	9.74341842	9.4113463
7666	ENSG0000013913	FGD4	protein_coding	chr12	32399558	32646050	+	Human GRCh	OTTHUMG000001	FGD4	FYVE, RhoGEF and PH domain containing 4	Cytoplasm	other		-1.024829167	0	0.91238431	0	1	0	10.4371128	10.5755515	10.4593519	10.5027489
7667	ENSG0000013913	ALG10	protein_coding	chr12	34022468	34029694	+	Human GRCh	OTTHUMG000001	ALG10	ALG10 alpha-1,2-glucosyltransferase	Cytoplasm	enzyme		-1.049112076	0	0.83812021	0	1	0	9.77255115	9.89837845	10.0211379	9.4865821
7668	ENSG0000013914	PIK3C2G	protein_coding	chr12	18242961	18648416	+	Human GRCh	OTTHUMG000001	PIK3C2G	phosphatidylinositol-4-phosphate 3-kinase catalytic subunit ty	Cytoplasm	kinase		-16.08203489	-1	0.06479711	0	1	0	2.21460897	1.97760692	1.38548755	1.35400619
7669	ENSG0000013914	SINHCAF	protein_coding	chr12	31280584	31327058	-	Human GRCh	OTTHUMG000001	SINHCAF	SIN3-HDAC complex associated factor	Extracellular	other		-1.15374301	0	0.51300191	0	1	0	11.8670721	12.1196528	12.0396178	11.5975731
7670	ENSG0000013915	PLCZ1	protein_coding	chr12	18683169	18738100	-	Human GRCh	OTTHUMG000001	PLCZ1	phospholipase C zeta 1	Cytoplasm	enzyme		1	0	1	0	NA	0	0.47872432	0.48576625	0.48458137	0.479129
7671	ENSG0000013915	AEBP2	protein_coding	chr12	19404045	19720801	+	Human GRCh	OTTHUMG000001	AEBP2	AE binding protein 2	Nucleus	transcription		1.118749625	0	0.62889276	0	1	0	10.3394397	10.4742449	10.7743817	10.2289944
7672	ENSG0000013915	SLCO1C1	protein_coding	chr12	20695332	20753386	+	Human GRCh	OTTHUMG000001	SLCO1C1	solute carrier organic anion transporter family member 1C1	Plasma Mem	transporter		-1.877206952	-1	0.11192146	0	1	0	7.94710718	8.14574231	7.43862724	7.14420714
7673	ENSG0000013916	ETFBKMT	protein_coding	chr12	31647160	31673114	+	Human GRCh	OTTHUMG000001	ETFBKMT	electron transfer flavoprotein subunit beta lysine methyltrans	Cytoplasm	enzyme		-1.248713808	0	0.51795239	0	1	0	7.23959031	7.53213359	7.23491978	7.0749162
7674	ENSG0000013916	ETNK1	protein_coding	chr12	22625075	22690665	+	Human GRCh	OTTHUMG000001	ETNK1	ethanolamine kinase 1	Cytoplasm	kinase		1.215893839	0	0.43387667	0	1	0	11.269534	11.4651482	11.8927133	11.1714722
7675	ENSG0000013916	ZCRB1	protein_coding	chr12	42312086	42326257	-	Human GRCh	OTTHUMG000001	ZCRB1	zinc finger CCHC-type and RNA binding motif containing 1	Nucleus	other		-1.216561261	0	0.37523738	0	1	0	10.1488491	10.4188151	10.227678	9.89532851
7676	ENSG0000013917	TMEM117	protein_coding	chr12	43835967	44389762	+	Human GRCh	OTTHUMG000001	TMEM117	transmembrane protein 117	Cytoplasm	other		1.179608856	0	0.69687539	0	1	0	5.31786052	5.3199359	5.4049985	5.54941673
7677	ENSG0000013917	PRICKLE1	protein_coding	chr12	42456757	42590355	-	Human GRCh	OTTHUMG000001	PRICKLE1	prickle planar cell polarity protein 1	Nucleus	other		-2.067929345	-1	0.17737123	0	1	0	7.09592788	7.2152069	6.59721978	6.04447937
7678	ENSG0000013917	C1RL	protein_coding	chr12	7089587	7109238	-	Human GRCh	OTTHUMG000001	C1RL	complement C1r subcomponent like	Extracellular	peptidase		1.491305059	0	0.32812639	0	1	0	4.62943137	4.98209391	4.53006602	5.4744472
7679	ENSG0000013918	NDUFA9	protein_coding	chr12	4649095	4694317	+	Human GRCh	OTTHUMG000001	NDUFA9	NADH:ubiquinone oxidoreductase subunit A9	Cytoplasm	enzyme		-1.032464833	0	0.87525177	0	1	0	10.3190977	10.7194819	10.5918163	10.4011489
7680	ENSG0000013918	CLSTN3	protein_coding	chr12	7129698	7158945	+	Human GRCh	OTTHUMG000001	CLSTN3	calsyntenin 3	Plasma Mem	other		1.183776879	0	0.57890319	0	1	0	8.96282149	9.26317265	9.15003653	9.4405985
7681	ENSG0000013918	KLRG1	protein_coding	chr12	8950044	9010760	+	Human GRCh	OTTHUMG000001	KLRG1	killer cell lectin like receptor G1	Plasma Mem	other		4.461277632	1	0.10552226	0	1	0	2.71636564	2.26800908	3.12962686	3.12162043
7682	ENSG0000013919	VAMP1	protein_coding	chr12	6462237	6470677	-	Human GRCh	OTTHUMG000001	VAMP1	vesicle associated membrane protein 1	Cytoplasm	transporter	botulinum	-1.077040843	0	0.77622606	0	1	0	8.33530178	8.5761541	8.46381083	8.29143923
7683	ENSG0000013919	TAPBPL	protein_coding	chr12	6451690	6466517	+	Human GRCh	OTTHUMG000001	TAPBPL	TAP binding protein like	Cytoplasm	other		1.909492327	1	0.06828571	0	1	0	6.07287928	6.70078846	7.47861443	6.32865783
7685	ENSG0000013919	RBP5	protein_coding	chr12	7115736	7128889	-	Human GRCh	OTTHUMG000001	RBP5	retinol binding protein 5	Cytoplasm	transporter		1.201616705	0	0.80035413	0	1	0	2.85068401	2.96281866	2.64937383	3.24402326
7686	ENSG0000013919	PEX5	protein_coding	chr12	7188685	7218574	+	Human GRCh	OTTHUMG000001	PEX5	peroxisomal biogenesis factor 5	Cytoplasm	other		-1.039696646	0	0.84012954	0	1	0	10.3719374	10.6792705	10.646759	10.3141422
7687	ENSG0000013920	PIANP	protein_coding	chr12	6693791	6700815	-	Human GRCh	OTTHUMG000001	PIANP	PILR alpha associated neural protein	Plasma Mem	other		10.04446744	1	0.00339564	1	1	0	2.55603922	3.22525007	5.34647148	3.28137746
7688	ENSG0000013920	SLC38A4	protein_coding	chr12	46764761	46832408	-	Human GRCh	OTTHUMG000001	SLC38A4	solute carrier family 38 member 4	Plasma Mem	transporter		-1.680734937	-1	0.1877753	0	1	0	7.74982036	7.77172196	7.13395738	7.17167123
7689	ENSG0000013921	AMIGO2	protein_coding	chr12	47075707	47079959	-	Human GRCh	OTTHUMG000001	AMIGO2	adhesion molecule with Ig like domain 2	Plasma Mem	other		-1.989527366	-1	0.47788555	0	1	0	3.09040214	2.26467189	2.24932912	2.73436276
7690	ENSG0000013921	SCAF11	protein_coding	chr12	45919131	45992120	-	Human GRCh	OTTHUMG000001	SCAF11	SR-related CTD associated factor 11	Nucleus	other		-1.057091935	0	0.81450359	0	1	0	12.230727	12.5614243	12.5795426	12.0544723
7691	ENSG0000013921	COL2A1	protein_coding	chr12	47972967	48004554	-	Human GRCh	OTTHUMG000001	COL2A1	collagen type II alpha 1 chain	Extracellular	other	collagenas	-5.405540564	-1	0.18612608	0	1	0	2.88699005	2.44624891	2.42909605	2.34581629
7692	ENSG0000013922	PPFIA2	protein_coding	chr12	81257975	81759553	-	Human GRCh	OTTHUMG000001	PPFIA2	PTPRF interacting protein alpha 2	Plasma Mem	phosphatase		1.567821051	1	0.38095604	0	1	0	4.30832491	5.09741036	5.388586	4.98755645
7694	ENSG0000013923	LLPH	protein_coding	chr12	66116555	66130750	-	Human GRCh	OTTHUMG000001	LLPH	LLP homolog, long-term synaptic facilitation factor	Extracellular	other		1.291010364	0	0.2847357	0	1	0	8.52894984	8.59195125	9.06124774	8.55880402
7695	ENSG0000013926	LRIG3	protein_coding	chr12	58872149	58920504	-	Human GRCh	OTTHUMG000001	LRIG3	leucine rich repeats and immunoglobulin like domains 3	Extracellular	other		-1.382859846	0	0.16425111	0	1	0	11.3637525	11.6740606	11.3038173	11.001362
7696	ENSG0000013926	MARCHF9	protein_coding	chr12	57755103	57760411	+	Human GRCh	OTTHUMG000001	MARCHF9	membrane associated ring-CH-type finger 9	Cytoplasm	other		1.074412588	0	0.74828853	0	1	0	9.80049005	10.0033748	10.1583466	9.78372554
7697	ENSG0000013926	INHBE	protein_coding	chr12	57452323	57459280	+	Human GRCh	OTTHUMG000001	INHBE	inhibin subunit beta E	Extracellular	growth facto		-2.184863164	-1	0.18310061	0	1	0	6.80010777	6.93803403	6.04515359	6.14333443
7698	ENSG0000013927	GLIPR1	protein_coding	chr12	75480753	75503863	+	Human GRCh	OTTHUMG000001	GLIPR1	GLI pathogenesis related 1	Extracellular	other		-1.173066624	0	0.94266928	0	NA	0	0.53838515	0.9251426	0.5441039	0.86835187
7700	ENSG0000013928	PHLDA1	protein_coding	chr12	76025447	76031776	-	Human GRCh	OTTHUMG000001	PHLDA1	pleckstrin homology like domain family A member 1	Cytoplasm	other		1.777974242	1	0.22951638	0	1	0	6.14503036	5.85579338	6.3649321	6.79244002
7701	ENSG0000013929	TMEM19	protein_coding	chr12	71686082	71705047	+	Human GRCh	OTTHUMG000001	TMEM19	transmembrane protein 19	Other	other		-1.191608512	0	0.45903589	0	1	0	9.58560613	9.89745834	9.8082491	9.25304789
7702	ENSG0000013929	LGR5	protein_coding	chr12	71439798	71586310	+	Human GRCh	OTTHUMG000001	LGR5	leucine rich repeat containing G protein-coupled receptor 5	Plasma Mem	transmembra	BNC101, p	-1.247181051	0	0.68594759	0	1	0	8.34182911	8.78079988	8.77842544	7.60442274
7703	ENSG0000013930	PTPRQ	protein_coding	chr12	80402178	80680273	+	Human GRCh	OTTHUMG000001	PTPRQ	protein tyrosine phosphatase receptor type Q	Extracellular	other		1.986395632	1	0.67029501	0	NA	0	1.34401908	0.90217805	0.89876019	1.65579936
7704	ENSG0000013931	DUSP6	protein_coding	chr12	89347235	89352501	-	Human GRCh	OTTHUMG000001	DUSP6	dual specificity phosphatase 6	Cytoplasm	phosphatase		-1.411779044	0	0.30758268	0	1	0	7.4390898	7.22026892	6.95728301	7.01153584
7705	ENSG0000013932	POC1B	protein_coding	chr12	89419718	89526047	-	Human GRCh	OTTHUMG000001	POC1B	POC1 centriolar protein B	Cytoplasm	other		1.007481065	0	0.97299527	0	1	0	9.3547493	9.49618163	9.62061932	9.21627147
7706	ENSG0000013932	TMTC3	protein_coding	chr12	88142296	88199887	+	Human GRCh	OTTHUMG000001	TMTC3	transmembrane O-mannosyltransferase targeting cadherins 3	Cytoplasm	enzyme		1.026826434	0	0.90689742	0	1	0	10.2229551	10.5440477	10.5825703	10.2396255
7707	ENSG0000013932	LUM	protein_coding	chr12	91102629	91111494	-	Human GRCh	OTTHUMG000001	LUM	lumican	Extracellular	other		1.719834515	1	0.66549363	0	1	0	2.58522465	2.31641552	3.02880487	2.59512451
7709	ENSG0000013934	SNRPF	protein_coding	chr12	95858952	95903828	+	Human GRCh	OTTHUMG000001	SNRPF	small nuclear ribonucleoprotein polypeptide F	Nucleus	other		1.323519637	0	0.31423784	0	1	0	8.28005258	8.99204102	8.98539589	8.9949
7710	ENSG0000013934	AMDHD1	protein_coding	chr12	95943331	95968720	+	Human GRCh	OTTHUMG000001	AMDHD1	amidohydrolase domain containing 1	Cytoplasm	enzyme		-5.602166556	-1	0.19497179	0	1	0	1.20251812	2.09852536	1.28637345	1.20862308
7711	ENSG0000013935	NEDD1	protein_coding	chr12	96907224	96953780	+	Human GRCh	OTTHUMG000001	NEDD1	NEDD1 gamma-tubulin ring complex targeting factor	Cytoplasm	other		-1.026759882	0	0.89479769	0	1	0	10.3503686	10.685218	10.6567774	10.3203325
7712	ENSG0000013935	SYCP3	protein_coding	chr12	101728648	101739472	-	Human GRCh	OTTHUMG000001	SYCP3	synaptonemal complex protein 3	Nucleus	other		2.130473885	1	0.35849309	0	NA	0	2.94351462	2.43851823	3.41860597	2.79615487
7713	ENSG0000013935	ASCL1	protein_coding	chr12	102957674	102960513	+	Human GRCh	OTTHUMG000001	ASCL1	achaete-scute family bHLH transcription factor 1	Nucleus	transcription		1.220372139	0	0.71252746	0	1	0	4.32911432	4.86452744	4.65231318	4.7388514
7714	ENSG0000013935	GAS2L3	protein_coding	chr12	100573683	100628288	+	Human GRCh	OTTHUMG000001	GAS2L3	growth arrest specific 2 like 3	Other	other		1.018385677	0	0.93687677	0	1	0	10.1114049	10.4931643	10.4654407	10.1903611
7715	ENSG0000013936	TMEM132	protein_coding	chr12	125186386	125662377	+	Human GRCh	OTTHUMG000001	TMEM132B	transmembrane protein 132B	Other	other		1.600584692	1	0.53077539	0	1	0	2.20821274	3.1693879	2.8802215	3.23000182
7716	ENSG0000013937	SLC15A4	protein_coding	chr12	128793194	128823958	-	Human GRCh	OTTHUMG000001	SLC15A4	solute carrier family 15 member 4	Cytoplasm	transporter		-1.058385815	0	0.78647507	0	1	0	9.21853878	9.33509084	9.25513362	9.17658947
7717	ENSG0000013937	TDG	protein_coding	chr12	103965822	103988874	+	Human GRCh	OTTHUMG000001	TDG	thymine DNA glycosylase	Nucleus	enzyme		-1.067751778	0	0.74033047	0	1	0	10.7038565	10.7977076	10.8584019	10.4681706
7718	ENSG0000013940	RITA1	protein_coding	chr12	113185526	113192368	+	Human GRCh	OTTHUMG000001	RITA1	RBPJ interacting and tubulin associated 1	Cytoplasm	other		-1.007285539	0	0.97325974	0	1	0	9.44353319	9.81133849	9.69921231	9.56349401
7719	ENSG0000013941	SDSL	protein_coding	chr12	113422380	113438276	+	Human GRCh	OTTHUMG000001	SDSL	serine dehydratase like	Cytoplasm	enzyme		1.222341284	0	0.60714196	0	1	0	5.93581639	6.33643047	6.43277624	6.2673483
7720	ENSG0000013942	MMAB	protein_coding	chr12	109553715	109573580	-	Human GRCh	OTTHUMG000001	MMAB	metabolism of cobalamin associated B	Cytoplasm	enzyme		-1.188614596	0	0.47860899	0	1	0	9.90965432	10.4715617	10.0728073	9.98675226
7721	ENSG0000013943	GLTP	protein_coding	chr12	109850945	109880541	-	Human GRCh	OTTHUMG000001	GLTP	glycolipid transfer protein	Cytoplasm	transporter		-1.116505108	0	0.62928054	0	1	0	10.0696444	10.4202926	10.1912324	10.0748547
7722	ENSG0000013943	GIT2	protein_coding	chr12	109929804	109996389	-	Human GRCh	OTTHUMG000001	GIT2	GIT ArfGAP 2	Nucleus	other		-1.114108899	0	0.62771425	0	1	0	10.1263761	10.5714054	10.3905441	10.0953761
7723	ENSG0000013943	TCHP	protein_coding	chr12	109900264	109983841	+	Human GRCh	OTTHUMG000001	TCHP	trichoplein keratin filament binding	Cytoplasm	other		-1.011250771	0	0.95817012	0	1	0	9.04397038	9.34298797	9.26572238	9.11015004
7724	ENSG0000013943	FAM222A	protein_coding	chr12	109713825	109770495	+	Human GRCh	OTTHUMG000001	FAM222A	family with sequence similarity 222 member A	Other	other		1.19171675	0	0.50991929	0	1	0	8.48214774	8.6472303	8.72662385	8.79193841
7725	ENSG0000013944	FOXN4	protein_coding	chr12	109277978	109309284	-	Human GRCh	OTTHUMG000001	FOXN4	forkhead box N4	Nucleus	transcription		1.12934885	0	0.87479161	0	1	0	3.24447818	2.7631974	2.94170597	3.25418407
7726	ENSG0000013949	NUP58	protein_coding	chr13	25301524	25365390	+	Human GRCh	OTTHUMG000000	NUP58	nucleoporin 58	Nucleus	other		1.01166824	0	0.95670166	0	1	0	11.7924796	12.0063601	12.1237308	11.6664181
7727	ENSG0000013950	MTMR6	protein_coding	chr13	25246222	25287510	-	Human GRCh	OTTHUMG000000	MTMR6	myotubularin related protein 6	Cytoplasm	phosphatase		1.003833059	0	0.98753089	0	1	0	10.0877138	10.270273	10.433142	9.87607928
7728	ENSG0000013950	SLC46A3	protein_coding	chr13	28700064	28718970	-	Human GRCh	OTTHUMG000000	SLC46A3	solute carrier family 46 member 3	Extracellular	transporter		-1.114920656	0	0.8522767	0	1	0	5.52885556	6.13141281	5.6059832	5.87562883

7729	**ENSG0000013951**	SLC7A1	protein_coding	chr13	29509414	29595688	-	Human GRCh	OTTHUMG000000	SLC7A1	**solute carrier family 7 member 1**	Plasma Mem	transporter		-1.233432246	0	0.27739372	0	1	0	12.8805243	12.828909	12.771098	12.445491
7730	**ENSG0000013951**	PDX1	protein_coding	chr13	27920000	27926313	+	Human GRCh	OTTHUMG000000	PDX1	**pancreatic and duodenal homeobox 1**	Nucleus	transcription		1.123232552	0	0.85812456	0	1	0	4.44179014	4.39146006	4.68312484	4.36040977
7731	**ENSG0000013951**	LNX2	protein_coding	chr13	27545913	27620529	-	Human GRCh	OTTHUMG000000	LNX2	**ligand of numb-protein X 2**	Plasma Mem	other		1.054756992	0	0.80503244	0	1	0	10.449181	10.7385319	10.7637131	10.5517522
7732	**ENSG0000013953**	SUOX	protein_coding	chr12	55997180	56006441	+	Human GRCh	OTTHUMG000001	SUOX	**sulfite oxidase**	Cytoplasm	enzyme		-1.077916174	0	0.74052099	0	1	0	9.80418755	10.2323663	10.0787636	9.81352559
7733	**ENSG0000013953**	CCDC65	protein_coding	chr12	48904110	48931840	+	Human GRCh	OTTHUMG000001	CCDC65	**coiled-coil domain containing 65**	Cytoplasm	other		1.141945309	0	0.84496116	0	1	0	3.99724782	3.95388423	4.12448661	4.06463523
7734	**ENSG0000013954**	SLC39A5	protein_coding	chr12	56230049	56237846	+	Human GRCh	OTTHUMG000001	SLC39A5	**solute carrier family 39 member 5**	Plasma Mem	transporter		2.447115467	1	0.19550711	0	1	0	2.85602673	3.09882305	3.78179185	3.10396957
7735	**ENSG0000013954**	TARBP2	protein_coding	chr12	53500921	53506431	+	Human GRCh	OTTHUMG000001	TARBP2	**TARBP2 subunit of RISC loading complex**	Nucleus	other		-1.013376484	0	0.94890943	0	1	0	9.0549119	9.25455537	9.32598962	8.93007493
7736	**ENSG0000013954**	RDH16	protein_coding	chr12	56951431	56959374	-	Human GRCh	OTTHUMG000001	RDH16	**retinol dehydrogenase 16**	Cytoplasm	enzyme		-1.588378657	-1	0.30924131	0	1	0	4.8645169	4.90488677	4.2331821	4.69016569
7737	**ENSG0000013954**	DHH	protein_coding	chr12	49086656	49094801	-	Human GRCh	OTTHUMG000001	DHH	**desert hedgehog signaling molecule**	Extracellular	peptidase		-1.737603675	-1	0.46926067	0	1	0	3.81039909	3.0778972	3.27499496	3.08373448
7740	**ENSG0000013957**	NPFF	protein_coding	chr12	53506688	53507484	-	Human GRCh	OTTHUMG000001	NPFF	**neuropeptide FF-amide peptide precursor**	Extracellular	other		1.054270501	0	0.90162639	0	1	0	5.68694962	5.87481329	6.15798038	5.38499658
7741	**ENSG0000013957**	NABP2	protein_coding	chr12	56222015	56229854	+	Human GRCh	OTTHUMG000001	NABP2	**nucleic acid binding protein 2**	Nucleus	other		-1.127991104	0	0.5911567	0	1	0	10.395231	11.0098006	10.5885419	10.6303634
7742	**ENSG0000013959**	N4BP2L1	protein_coding	chr13	32400723	32428311	-	Human GRCh	OTTHUMG000000	N4BP2L1	**NEDD4 binding protein 2 like 1**	Other	other		-1.455619236	0	0.24548843	0	1	0	6.72088415	6.80728366	6.47328597	6.29788485
7744	**ENSG0000013961**	SMARCC2	protein_coding	chr12	56162359	56189567	-	Human GRCh	OTTHUMG000001	SMARCC2	**SWI/SNF related, matrix associated, actin dependent regulato**	Nucleus	transcription		-1.134176283	0	0.50548435	0	1	0	12.6968011	12.9793725	12.781398	12.6295381
7745	**ENSG0000013961**	BRCA2	protein_coding	chr13	32315086	32400268	+	Human GRCh	OTTHUMG000000	BRCA2	**BRCA2 DNA repair associated**	Nucleus	transcription		-1.13016315	0	0.53244123	0	1	0	11.3102341	11.4917292	11.3959907	11.1267759
7746	**ENSG0000013962**	KANSL2	protein_coding	chr12	48653211	48682238	-	Human GRCh	OTTHUMG000001	KANSL2	**KAT8 regulatory NSL complex subunit 2**	Nucleus	other		-1.027887172	0	0.89797126	0	1	0	9.84958357	10.1311054	10.1311429	9.77953553
7747	**ENSG0000013962**	CERS5	protein_coding	chr12	50129289	50167533	-	Human GRCh	OTTHUMG000001	CERS5	**ceramide synthase 5**	Cytoplasm	transcription		-1.276965344	0	0.24463474	0	1	0	9.54055284	9.84274175	9.61977472	9.20858516
7748	**ENSG0000013962**	MAP3K12	protein_coding	chr12	53479669	53500063	-	Human GRCh	OTTHUMG000001	MAP3K12	**mitogen-activated protein kinase kinase kinase 12**	Cytoplasm	kinase		-1.063531987	0	0.85341899	0	1	0	8.44088144	8.26753944	8.49937081	8.0364814
7749	**ENSG0000013962**	ITGB7	protein_coding	chr12	53191323	53207282	-	Human GRCh	OTTHUMG000001	ITGB7	**integrin subunit beta 7**	Plasma Mem	transmembra	vedolizum	8.00491111	1	0.02028579	1	1	0	2.52115699	2.10783037	3.18508756	3.4252753
7750	**ENSG0000013962**	GALNT6	protein_coding	chr12	51351247	51392867	-	Human GRCh	OTTHUMG000001	GALNT6	**polypeptide N-acetylgalactosaminyltransferase 6**	Cytoplasm	enzyme		2.13513663	1	0.04900181	1	1	0	4.53068146	4.92092175	4.78656785	5.74465311
7751	**ENSG0000013963**	CSAD	protein_coding	chr12	53157663	53180925	-	Human GRCh	OTTHUMG000000	CSAD	**cysteine sulfinic acid decarboxylase**	Cytoplasm	enzyme		1.021199901	0	0.92439815	0	1	0	9.1047986	9.0959791	9.24307304	9.88882255
7752	**ENSG0000013963**	LMBR1L	protein_coding	chr12	49097136	49110900	-	Human GRCh	OTTHUMG000001	LMBR1L	**limb development membrane protein 1 like**	Plasma Mem	transmembra		-1.217533296	0	0.39915502	0	1	0	9.70289872	9.84047819	9.67631786	9.42224748
7753	**ENSG0000013963**	MYG1	protein_coding	chr12	53299695	53307177	+	Human GRCh	OTTHUMG000001	MYG1	**MYG1 exonuclease**	Nucleus	other		-1.005029189	0	0.98097767	0	1	0	9.45670484	9.71574043	9.70972609	9.45164385
7754	**ENSG0000013963**	ESYT1	protein_coding	chr12	56118250	56144674	+	Human GRCh	OTTHUMG000001	ESYT1	**extended synaptotagmin 1**	Cytoplasm	transporter		-1.199518371	0	0.34754547	0	1	0	12.5769755	12.9435784	12.6644799	12.465491
7755	**ENSG0000013964**	TMBIM6	protein_coding	chr12	49707725	49764934	+	Human GRCh	OTTHUMG000001	TMBIM6	**transmembrane BAX inhibitor motif containing 6**	Nucleus	other		-1.204089371	0	0.39363316	0	1	0	13.5318698	13.8114466	13.6335911	13.2848486
7756	**ENSG0000013964**	ANKRD52	protein_coding	chr12	56237807	56258384	-	Human GRCh	OTTHUMG000001	ANKRD52	**ankyrin repeat domain 52**	Nucleus	transcription		1.032595966	0	0.86073991	0	1	0	12.4508434	12.6876512	12.7281555	12.4807749
7758	**ENSG0000013965**	ZNF740	protein_coding	chr12	53180704	53195142	+	Human GRCh	OTTHUMG000001	ZNF740	**zinc finger protein 740**	Nucleus	transcription		-1.162233048	0	0.45204834	0	1	0	10.7170165	11.0388174	10.8343081	10.6071118
7760	**ENSG0000013966**	WDFY2	protein_coding	chr13	51584455	51767709	+	Human GRCh	OTTHUMG000000	WDFY2	**WD repeat and FYVE domain containing 2**	Cytoplasm	other		-1.056865341	0	0.80188756	0	1	0	10.6340641	10.8196382	10.8642905	10.4359023
7761	**ENSG0000013967**	HNRNPA1L	protein_coding	chr13	52642431	52643773	+	Human GRCh	OTTHUMG000000	HNRNPA1L2	**heterogeneous nuclear ribonucleoprotein A1 like 2**	Other	other		1.194157355	0	0.60300915	0	1	0	6.2696215	6.50520628	6.85100913	6.20643146
7762	**ENSG0000013968**	ESD	protein_coding	chr13	46771256	46797420	-	Human GRCh	OTTHUMG000000	ESD	**esterase D**	Cytoplasm	enzyme		-1.005689339	0	0.97993504	0	1	0	9.66394069	10.0086769	9.93837538	9.73800276
7764	**ENSG0000013968**	RB1	protein_coding	chr13	48303744	48599436	+	Human GRCh	OTTHUMG000000	RB1	**RB transcriptional corepressor 1**	Nucleus	transcription		-1.082631662	0	0.7417323	0	1	0	10.547671	10.9797287	10.8962021	10.4445576
7765	**ENSG0000013969**	SBNO1	protein_coding	chr12	123289109	123364847	-	Human GRCh	OTTHUMG000001	SBNO1	**strawberry notch homolog 1**	Other	enzyme		-1.09511618	0	0.65100356	0	1	0	12.6080566	12.9611382	12.8067582	12.5729332
7766	**ENSG0000013971**	MORN3	protein_coding	chr12	121648742	121672631	-	Human GRCh	OTTHUMG000001	MORN3	**MORN repeat containing 3**	Nucleus	other		1.322070073	0	0.41928032	0	1	0	5.85428719	5.7376297	6.30036904	5.78349523
7767	**ENSG0000013971**	SETD1B	protein_coding	chr12	121804009	121832656	+	Human GRCh	OTTHUMG000001	SETD1B	**SET domain containing 1B, histone lysine methyltransferase**	Nucleus	enzyme		-1.087097879	0	0.70484654	0	1	0	12.1729805	12.4951706	12.2771754	12.2302608
7768	**ENSG0000013971**	VPS33A	protein_coding	chr12	122229564	122266494	-	Human GRCh	OTTHUMG000001	VPS33A	**VPS33A core subunit of CORVET and HOPS complexes**	Cytoplasm	transporter		-1.073802906	0	0.71854317	0	1	0	9.96143783	10.2180297	10.1034344	9.92410339
7769	**ENSG0000013972**	VPS37B	protein_coding	chr12	122865330	122896127	-	Human GRCh	OTTHUMG000001	VPS37B	**VPS37B subunit of ESCRT-I**	Cytoplasm	other		1.070370886	0	0.77728276	0	1	0	9.68852953	10.0237103	9.93101839	9.95389216
7770	**ENSG0000013972**	RHOF	protein_coding	chr12	121777754	121803403	-	Human GRCh	OTTHUMG000001	RHOF	**ras homolog family member F, filopodia associated**	Cytoplasm	enzyme		1.00644728	0	0.9812804	0	1	0	9.56542646	10.0931295	9.88869252	9.84137248
7771	**ENSG0000013972**	DENR	protein_coding	chr12	122752824	122771064	+	Human GRCh	OTTHUMG000001	DENR	**density regulated re-initiation and release factor**	Other	other		1.011795378	0	0.95500686	0	1	0	11.1889934	11.452824	11.5337179	11.1110831
7772	**ENSG0000013973**	DIAPH3	protein_coding	chr13	59665583	60163928	-	Human GRCh	OTTHUMG000000	DIAPH3	**diaphanous related formin 3**	Cytoplasm	enzyme		-1.214015215	0	0.32788322	0	1	0	10.9919017	11.3201007	11.0778978	10.8032518
7773	**ENSG0000013973**	SLAIN1	protein_coding	chr13	77697687	77764242	+	Human GRCh	OTTHUMG000000	SLAIN1	**SLAIN motif family member 1**	Other	other		-1.150974428	0	0.52904526	0	1	0	9.92274743	10.1997926	10.0702643	9.7238532
7774	**ENSG0000013974**	RBM26	protein_coding	chr13	79311824	79406477	-	Human GRCh	OTTHUMG000000	RBM26	**RNA binding motif protein 26**	Other	other		-1.0614084	0	0.77300629	0	1	0	11.7644285	11.9650523	11.9623936	11.6185044
7775	**ENSG0000013976**	SRRM4	protein_coding	chr12	118981541	119163051	+	Human GRCh	OTTHUMG000001	SRRM4	**serine/arginine repetitive matrix 4**	Nucleus	other		-1.005271529	0	0.99641605	0	1	0	2.30824051	1.9722861	1.94430656	2.32032379
7777	**ENSG0000013979**	MBNL2	protein_coding	chr13	97221434	97394120	+	Human GRCh	OTTHUMG000000	MBNL2	**muscleblind like splicing regulator 2**	Nucleus	other		1.085005071	0	0.71937321	0	1	0	8.61415984	8.99795808	8.77293943	9.03246602
7779	**ENSG0000013980**	ZIC5	protein_coding	chr13	99962964	99971767	-	Human GRCh	OTTHUMG000000	ZIC5	**Zic family member 5**	Nucleus	transcription		-1.014332545	0	0.96812664	0	1	0	8.06561542	8.43573874	8.47317591	7.97378262
7780	**ENSG0000013982**	ABHD13	protein_coding	chr13	108218392	108234243	+	Human GRCh	OTTHUMG000000	ABHD13	**abhydrolase domain containing 13**	Other	peptidase		-1.065694576	0	0.77685378	0	1	0	8.86699203	9.02732785	8.97560336	8.77845635
7781	**ENSG0000013983**	RAB20	protein_coding	chr13	110523066	110561722	-	Human GRCh	OTTHUMG000000	RAB20	**RAB20, member RAS oncogene family**	Cytoplasm	enzyme		1.327776674	0	0.46606664	0	1	0	7.40551257	8.14311333	8.04248793	8.22401527
7782	**ENSG0000013983**	GRTP1	protein_coding	chr13	113324163	113364148	-	Human GRCh	OTTHUMG000000	GRTP1	**growth hormone regulated TBC protein 1**	Other	other		1.006476896	0	0.9776291	0	1	0	8.63159752	8.83930941	8.81989291	8.66911335
7783	**ENSG0000013984**	CUL4A	protein_coding	chr13	113208193	113267108	+	Human GRCh	OTTHUMG000000	CUL4A	**cullin 4A**	Nucleus	enzyme		-1.235518288	0	0.30103508	0	1	0	11.3446816	11.722037	11.4435938	11.1739609
7784	**ENSG0000013986**	TTC6	protein_coding	chr14	37595629	38041442	+	Human GRCh	OTTHUMG000001	TTC6	**tetratricopeptide repeat domain 6**	Other	other		-1.146509284	0	0.52517376	0	1	0	12.0272814	12.244261	12.1850374	11.7516711
7785	**ENSG0000013987**	SSTR1	protein_coding	chr14	38207904	38213067	+	Human GRCh	OTTHUMG000001	SSTR1	**somatostatin receptor 1**	Plasma Mem	G-protein co	pasireotide	-1.68325003	-1	0.30905444	0	1	0	5.5024469	5.74383136	5.23887809	4.98636308
7786	**ENSG0000013988**	CDH24	protein_coding	chr14	23047062	23057538	-	Human GRCh	OTTHUMG000000	CDH24	**cadherin 24**	Plasma Mem	other		1.118336084	0	0.64841624	0	1	0	9.58313092	9.79772661	9.75235056	9.87315137
7787	**ENSG0000013989**	REM2	protein_coding	chr14	22883222	22887678	+	Human GRCh	OTTHUMG000001	REM2	**RRAD and GEM like GTPase 2**	Plasma Mem	enzyme		1.04594816	0	0.93342262	0	1	0	4.93565339	4.85750099	5.1816281	4.62801314
7788	**ENSG0000013989**	CBLN3	protein_coding	chr14	24426545	24430954	-	Human GRCh	OTTHUMG000001	CBLN3	**cerebellin 3 precursor**	Extracellular	other		-1.110013731	0	0.79954934	0	1	0	5.93733118	6.43218493	6.14098847	6.06863648
7789	**ENSG0000013990**	TSSK4	protein_coding	chr14	24205696	24208362	+	Human GRCh	OTTHUMG000001	TSSK4	**testis specific serine kinase 4**	Cytoplasm	kinase		-1.800795086	-1	0.36839953	0	1	0	4.51149222	4.53987319	4.33265536	3.66320693
7790	**ENSG0000013991**	NOVA1	protein_coding	chr14	26443090	26598033	-	Human GRCh	OTTHUMG000000	NOVA1	**NOVA alternative splicing regulator 1**	Nucleus	other		1.171366859	0	0.56146072	0	1	0	8.60772291	8.9075619	8.97109845	8.8874499
7791	**ENSG0000013991**	FITM1	protein_coding	chr14	24130659	24132849	+	Human GRCh	OTTHUMG000001	FITM1	**fat storage inducing transmembrane protein 1**	Extracellular	other		1.163947168	0	0.72375081	0	1	0	5.27002989	5.73850607	5.4333713	5.86430131
7792	**ENSG0000013991**	MDGA2	protein_coding	chr14	46840092	47675605	-	Human GRCh	OTTHUMG000000	MDGA2	**MAM domain containing glycosylphosphatidylinositol anchor**	Plasma Mem	other		1.060443186	0	0.93875931	0	1	0	3.48411084	3.88522671	4.00057963	3.430829
7793	**ENSG0000013992**	TMX1	protein_coding	chr14	51240162	51257655	+	Human GRCh	OTTHUMG000001	TMX1	**thioredoxin related transmembrane protein 1**	Cytoplasm	enzyme		-1.061985734	0	0.79043418	0	1	0	9.95663597	10.2489289	10.2871814	9.74612405
7794	**ENSG0000013992**	FRMD6	protein_coding	chr14	51489100	51730727	+	Human GRCh	OTTHUMG000001	FRMD6	**FERM domain containing 6**	Cytoplasm	other		-2.327688266	-1	0.01348619	1	1	0	6.07920871	6.09404726	5.39547466	5.25426544
7795	**ENSG0000013994**	PELI2	protein_coding	chr14	56117814	56301524	+	Human GRCh	OTTHUMG000001	PELI2	**pellino E3 ubiquitin protein ligase family member 2**	Cytoplasm	enzyme		-3.047228908	-1	0.00236275	1	0.96	0	6.13787852	6.64695886	5.79956244	5.32589962
7796	**ENSG0000013997**	RTN1	protein_coding	chr14	59595976	59870776	-	Human GRCh	OTTHUMG000000	RTN1	**reticulon 1**	Cytoplasm	other		1.444799465	0	0.44406098	0	1	0	5.53209632	5.44514668	6.05997575	5.58944654
7797	**ENSG0000013997**	ARMH4	protein_coding	chr14	57999735	58298139	-	Human GRCh	OTTHUMG000001	ARMH4	**armadillo like helical domain containing 4**	Extracellular	other		1.14251016	0	0.67797531	0	1	0	6.6089817	6.04690718	6.55973585	6.44850522
7798	**ENSG0000013997**	SYT16	protein_coding	chr14	61811974	62112550	+	Human GRCh	OTTHUMG000001	SYT16	**synaptotagmin 16**	Other	transporter		-1.474715538	0	0.66493767	0	1	0	2.91674179	3.03543172	3.01619658	2.47570881
7799	**ENSG0000013997**	SLC38A6	protein_coding	chr14	60981114	61114316	+	Human GRCh	OTTHUMG000001	SLC38A6	**solute carrier family 38 member 6**	Plasma Mem	transporter		-1.017964533	0	0.9470264	0	1	0	8.25227047	8.3913686	8.46199397	8.12468687
7800	**ENSG0000013997**	NAA30	protein_coding	chr14	57390544	57415906	+	Human GRCh	OTTHUMG000001	NAA30	**N-alpha-acetyltransferase 30, NatC catalytic subunit**	Cytoplasm	enzyme		1.000242351	0	0.99909828	0	1	0	10.1901782	10.3468974	10.4253433	10.0944426
7803	**ENSG0000013999**	DCAF5	protein_coding	chr14	69050881	69153150	-	Human GRCh	OTTHUMG000001	DCAF5	**DDB1 and CUL4 associated factor 5**	Cytoplasm	other		-1.153562891	0	0.49149003	0	1	0	11.1056009	11.3741671	11.1961342	10.9683469
7804	**ENSG0000013999**	RAB15	protein_coding	chr14	64945814	64973226	-	Human GRCh	OTTHUMG000001	RAB15	**RAB15, member RAS oncogene family**	Cytoplasm	other		-1.218201715	0	0.53604108	0	1	0	7.31359853	7.2908935	7.28892976	6.882406
7805	**ENSG0000014000**	WDR89	protein_coding	chr14	63597039	63641871	-	Human GRCh	OTTHUMG000001	WDR89	**WD repeat domain 89**	Other	other		1.289453569	0	0.25352146	0	1	0	8.41185258	8.64606546	9.04751922	8.51239492
7806	**ENSG0000014000**	ESR2	protein_coding	chr14	64084232	64338112	-	Human GRCh	OTTHUMG000001	ESR2	**estrogen receptor 2**	Nucleus	ligand-depen	sulindac/ta	1.18414794	0	0.81519443	0	1	0	3.71677264	3.53283351	3.63652122	3.85587255
7807	**ENSG0000014001**	KCNH5	protein_coding	chr14	62699464	63102037	-	Human GRCh	OTTHUMG000000	KCNH5	**potassium voltage-gated channel subfamily H member 5**	Plasma Mem	ion channel		24.36389193	1	0.00748097	1	1	0	1.08511672	1.19034522	2.65853566	2.33915233
7808	**ENSG0000014001**	STON2	protein_coding	chr14	81260652	81436465	-	Human GRCh	OTTHUMG000001	STON2	**stonin 2**	Cytoplasm	other		1.67998253	1	0.30092607	0	1	0	9.94084357	10.2902159	10.6785519	10.6200003
7809	**ENSG0000014002**	EFCAB11	protein_coding	chr14	89794669	89954777	-	Human GRCh	OTTHUMG000001	EFCAB11	**EF-hand calcium binding domain 11**	Other	other		-1.002115141	0	0.99312395	0	1	0	8.73137164	8.77748101	8.9241942	8.55159283
7811	**ENSG0000014004**	PTGR2	protein_coding	chr14	73851831	73885726	+	Human GRCh	OTTHUMG000001	PTGR2	**prostaglandin reductase 2**	Cytoplasm	enzyme	glycyrrheti	1.088590239	0	0.71622577	0	1	0	9.26854704	9.46349773	9.62838706	9.26771151
7812	**ENSG0000014004**	JDP2	protein_coding	chr14	75427716	75474111	+	Human GRCh	OTTHUMG000001	JDP2	**Jun dimerization protein 2**	Nucleus	transcription		-1.71067102	-1	0.41291696	0	1	0	5.3943425	3.40398687	3.79405754	4.81821425
7813	**ENSG0000014005**	AK7	protein_coding	chr14	96392128	96489427	+	Human GRCh	OTTHUMG000001	AK7	**adenylate kinase 7**	Cytoplasm	kinase		1.056617438	0	0.85671487	0	1	0	6.52480872	6.91967719	7.05768615	6.45573081
7814	**ENSG0000014006**	FAM181A	protein_coding	chr14	93918894	93929608	+	Human GRCh	OTTHUMG000001	FAM181A	**family with sequence similarity 181 member A**	Cytoplasm	other		1	0	1	0	1	0	1.49797001	1.5891752	1.57455779	1.50351485
7815	**ENSG0000014009**	SLC24A4	protein_coding	chr14	92322581	92501481	+	Human GRCh	OTTHUMG000001	SLC24A4	**solute carrier family 24 member 4**	Plasma Mem	transporter		1.210514532	0	0.67594953	0	1	0	5.3597637	4.82167227	5.64528771	4.86186408
7816	**ENSG0000014009**	FBLN5	protein_coding	chr14	91869411	91947987	-	Human GRCh	OTTHUMG000001	FBLN5	**fibulin 5**	Extracellular	other		-1.684778427	-1	0.50634973	0	1	0	2.72535478	3.05859283	2.81620144	2.46388259
7818	**ENSG0000014010**	CLBA1	protein_coding	chr14	104985775	105010482	+	Human GRCh	OTTHUMG000001	CLBA1	**clathrin binding box of aftiphilin containing 1**	Other	other		1.0225176	0	0.92859572	0	1	0	8.7733164	9.02154135	9.0799129	8.75383564
7819	**ENSG0000014010**	WARS1	protein_coding	chr14	100333790	100376805	-	Human GRCh	OTTHUMG000001	WARS1	**tryptophanyl-tRNA synthetase 1**	Cytoplasm	enzyme		-1.141982553	0	0.51128454	0	1	0	11.3088133	11.5339379	11.3563762	11.2007213
7821	**ENSG0000014015**	WDR20	protein_coding	chr14	102139503	102224847	+	Human GRCh	OTTHUMG000001	WDR20	**WD repeat domain 20**	Nucleus	other		-1.054706223	0	0.79083779	0	1	0	9.75640856	10.0346217	9.97040684	9.706478
7822	**ENSG0000014015**	NIPA2	protein_coding	chr15	22838644	22869362	+	Human GRCh	OTTHUMG000001	NIPA2	**NIPA magnesium transporter 2**	Cytoplasm	other		-1.042219752	0	0.83641743	0	1	0	10.3186972	10.6060214	10.5177914	10.3233133
7823	**ENSG0000014019**	SLC12A6	protein_coding	chr15	34229784	34338060	-	Human GRCh	OTTHUMG000001	SLC12A6	**solute carrier family 12 member 6**	Plasma Mem	transporter		-1.160951984	0	0.50427908	0	1	0	9.18581297	9.71063037	9.48109919	9.12046393
7824	**ENSG0000014025**	DUOXA1	protein_coding	chr15	45117366	45129938	-	Human GRCh	OTTHUMG000001	DUOXA1	**dual oxidase maturation factor 1**	Plasma Mem	other		1.384077081	0	0.25838349	0	1	0	8.74783304	9.25210763	9.68059277	9.06370146

7825	**ENSG0000014025**	MFAP1	protein_coding	chr15	43804492	43824690	-	Human GRCh	OTTHUMG000000	MFAP1	**microfibril associated protein 1**	Extracellular	other		1.180204436	0	0.50260056	0	1	0	8.61921657	8.81925672	9.07218008	8.71647711
7826	**ENSG0000014026**	TCF12	protein_coding	chr15	56918623	57299281	+	Human GRCh	OTTHUMG000001	TCF12	**transcription factor 12**	Nucleus	transcription		-1.07362241	0	0.73775806	0	1	0	11.6082861	11.9043512	11.8324117	11.5196447
7827	**ENSG0000014026**	SORD	protein_coding	chr15	45023147	45077185	+	Human GRCh	OTTHUMG000001	SORD	**sorbitol dehydrogenase**	Cytoplasm	enzyme		-1.174720385	0	0.62002473	0	1	0	14.4469892	14.6984565	14.5712559	14.1691522
7828	**ENSG0000014026**	SERF2	protein_coding	chr15	43777087	43784427	+	Human GRCh	OTTHUMG000000	SERF2	**small EDRK-rich factor 2**	Other	other		-1.107452906	0	0.61351948	0	1	0	12.3231628	12.6379897	12.4011937	12.3466914
7829	**ENSG0000014026**	ZSCAN29	protein_coding	chr15	43358172	43371043	-	Human GRCh	OTTHUMG000001	ZSCAN29	**zinc finger and SCAN domain containing 29**	Nucleus	transcription		-1.001025067	0	0.996118	0	1	0	10.2027999	10.416159	10.4367439	10.1750561
7830	**ENSG0000014027**	DUOXA2	protein_coding	chr15	45114326	45118421	+	Human GRCh	OTTHUMG000001	DUOXA2	**dual oxidase maturation factor 2**	Cytoplasm	other		1.162662586	0	0.87166812	0	1	0	3.3561511	3.7918943	3.92369416	3.51761498
7831	**ENSG0000014027**	DUOX2	protein_coding	chr15	45092650	45114172	-	Human GRCh	OTTHUMG000001	DUOX2	**dual oxidase 2**	Plasma Mem	enzyme		1.203901193	0	0.63897963	0	1	0	8.7475782	9.50591693	9.44442133	9.36057541
7832	**ENSG0000014028**	LYSMD2	protein_coding	chr15	51723011	51751585	-	Human GRCh	OTTHUMG000001	LYSMD2	**LysM domain containing 2**	Other	other		1.440877996	0	0.27161254	0	1	0	5.89138704	5.85072562	6.34354348	6.09987891
7833	**ENSG0000014028**	SLC27A2	protein_coding	chr15	50182196	50236385	+	Human GRCh	OTTHUMG000001	SLC27A2	**solute carrier family 27 member 2**	Cytoplasm	transporter		-1.128056499	0	0.68467153	0	1	0	9.44191727	9.88163562	9.68298498	9.38862275
7834	**ENSG0000014028**	FGF7	protein_coding	chr15	49423237	49488775	+	Human GRCh	OTTHUMG000001	FGF7	**fibroblast growth factor 7**	Extracellular	growth facto		-1.391355868	0	0.72220715	0	1	0	3.73992737	3.78618939	3.68096861	3.22995056
7835	**ENSG0000014028**	HDC	protein_coding	chr15	50241947	50265965	-	Human GRCh	OTTHUMG000001	HDC	**histidine decarboxylase**	Cytoplasm	enzyme		-1.968332334	-1	0.44051484	0	1	0	3.21632163	3.70912505	2.85800392	3.09225461
7836	**ENSG0000014029**	GCNT3	protein_coding	chr15	59594875	59640239	+	Human GRCh	OTTHUMG000001	GCNT3	**glucosaminyl (N-acetyl) transferase 3, mucin type**	Plasma Mem	enzyme		5.712019389	1	0.38360103	0	NA	0	0.98059503	0.99092903	1.37407158	0.98119015
7837	**ENSG0000014029**	BNIP2	protein_coding	chr15	59659146	59689339	-	Human GRCh	OTTHUMG000001	BNIP2	**BCL2 interacting protein 2**	Cytoplasm	other		-1.090821743	0	0.67906481	0	1	0	11.018954	11.3917882	11.3221764	10.8804827
7838	**ENSG0000014030**	GTF2A2	protein_coding	chr15	59638062	59657541	-	Human GRCh	OTTHUMG000001	GTF2A2	**general transcription factor IIA subunit 2**	Nucleus	transcription		-1.130747424	0	0.61746118	0	1	0	9.93398985	10.1771239	10.085943	9.73820716
7839	**ENSG0000014031**	SRP14	protein_coding	chr15	40035690	40039181	-	Human GRCh	OTTHUMG000001	SRP14	**signal recognition particle 14**	Cytoplasm	other		-1.135601662	0	0.56811037	0	1	0	10.6676527	11.0596502	10.8216934	10.6427693
7840	**ENSG0000014032**	BAHD1	protein_coding	chr15	40439721	40468236	+	Human GRCh	OTTHUMG000001	BAHD1	**bromo adjacent homology domain containing 1**	Nucleus	other		1.164330612	0	0.47021945	0	1	0	9.62792542	10.0303009	10.0873969	9.94126299
7841	**ENSG0000014032**	DISP2	protein_coding	chr15	40358219	40378621	+	Human GRCh	OTTHUMG000001	DISP2	**dispatched RND transporter family member 2**	Plasma Mem	other		1.260864195	0	0.59197096	0	1	0	6.42761558	6.80818363	6.78607767	6.93949256
7842	**ENSG0000014032**	CDAN1	protein_coding	chr15	42723544	42737128	-	Human GRCh	OTTHUMG000001	CDAN1	**codanin 1**	Nucleus	other		-1.058155597	0	0.78650193	0	1	0	10.1403608	10.3110384	10.3796485	9.90376263
7843	**ENSG0000014033**	TLE3	protein_coding	chr15	70047790	70098176	-	Human GRCh	OTTHUMG000001	TLE3	**TLE family member 3, transcriptional corepressor**	Nucleus	other		-1.053073876	0	0.81752817	0	1	0	12.1914611	12.5539846	12.2467538	12.4073001
7844	**ENSG0000014035**	ANP32A	protein_coding	chr15	68778535	68820897	-	Human GRCh	OTTHUMG000001	ANP32A	**acidic nuclear phosphoprotein 32 family member A**	Cytoplasm	other		-1.040840559	0	0.83299955	0	1	0	11.7896531	12.0735687	11.9699826	11.8153218
7845	**ENSG0000014036**	COMMD4	protein_coding	chr15	75336020	75343224	+	Human GRCh	OTTHUMG000001	COMMD4	**COMM domain containing 4**	Cytoplasm	other		-1.024833913	0	0.9066681	0	1	0	9.90960367	10.2687451	10.2395908	9.8859379
7846	**ENSG0000014036**	UBE2Q2	protein_coding	chr15	75843307	75901078	+	Human GRCh	OTTHUMG000001	UBE2Q2	**ubiquitin conjugating enzyme E2 Q2**	Cytoplasm	enzyme		-1.053775916	0	0.80207077	0	1	0	9.28414321	9.41612854	9.52027721	9.02657959
7847	**ENSG0000014036**	PSTPIP1	protein_coding	chr15	76993359	77037475	+	Human GRCh	OTTHUMG000001	PSTPIP1	**proline-serine-threonine phosphatase interacting protein 1**	Cytoplasm	other		-11.0204463	-1	0.1924005	0	NA	0	0.83760286	0.9021851	0.47897597	0.47183946
7848	**ENSG0000014037**	ETFA	protein_coding	chr15	76188555	76311730	-	Human GRCh	OTTHUMG000001	ETFA	**electron transfer flavoprotein subunit alpha**	Cytoplasm	transporter		-1.039453148	0	0.85161409	0	1	0	10.9169682	11.1810581	11.1199773	10.8950226
7850	**ENSG0000014038**	HMG20A	protein_coding	chr15	77420412	77485607	+	Human GRCh	OTTHUMG000001	HMG20A	**high mobility group 20A**	Nucleus	transcription		-1.081664203	0	0.67996081	0	1	0	9.61875538	9.94671704	9.7437883	9.67038524
7851	**ENSG0000014038**	SCAPER	protein_coding	chr15	76347904	76905444	-	Human GRCh	OTTHUMG000001	SCAPER	**S-phase cyclin A associated protein in the ER**	Extracellular	other		1.033019782	0	0.89702352	0	1	0	9.40331486	9.79842818	9.8330487	9.43834857
7852	**ENSG0000014039**	TSPAN3	protein_coding	chr15	77041404	77083984	-	Human GRCh	OTTHUMG000001	TSPAN3	**tetraspanin 3**	Plasma Mem	other		-1.093840835	0	0.67788068	0	1	0	11.5663629	11.7886897	11.6938621	11.4581162
7853	**ENSG0000014039**	SKIC8	protein_coding	chr15	78277835	78299703	-	Human GRCh	OTTHUMG000001	SKIC8	**SKI8 subunit of superkiller complex**	Nucleus	other		-1.076773948	0	0.74892537	0	1	0	9.31201811	9.97279689	9.74386566	9.45042978
7854	**ENSG0000014039**	NCOA2	protein_coding	chr8	70109782	70403808	-	Human GRCh	OTTHUMG000001	NCOA2	**nuclear receptor coactivator 2**	Nucleus	transcription		-1.077686139	0	0.72166638	0	1	0	11.0324328	11.3042734	11.2235891	10.9523076
7855	**ENSG0000014039**	NEIL1	protein_coding	chr15	75346955	75357115	+	Human GRCh	OTTHUMG000001	NEIL1	**nei like DNA glycosylase 1**	Nucleus	enzyme		-1.016650116	0	0.95526106	0	1	0	9.0878836	9.22409886	9.37923708	8.83868751
7856	**ENSG0000014040**	MAN2C1	protein_coding	chr15	75355207	75368612	-	Human GRCh	OTTHUMG000001	MAN2C1	**mannosidase alpha class 2C member 1**	Cytoplasm	enzyme		-1.007876001	0	0.97072935	0	1	0	10.8208953	10.9717709	11.0376745	10.7207767
7857	**ENSG0000014040**	DNAJA4	protein_coding	chr15	78264086	78282196	+	Human GRCh	OTTHUMG000001	DNAJA4	**DnaJ heat shock protein family (Hsp40) member A4**	Nucleus	other		1.219978177	0	0.66124703	0	1	0	6.16040204	5.72102007	6.27305609	6.00266719
7858	**ENSG0000014040**	TLNRD1	protein_coding	chr15	81000923	81005788	+	Human GRCh	OTTHUMG000001	TLNRD1	**talin rod domain containing 1**	Cytoplasm	other		-1.003589385	0	0.98728803	0	1	0	10.0694086	10.3317603	10.3252424	10.068445
7859	**ENSG0000014041**	TPM1	protein_coding	chr15	63042620	63071915	+	Human GRCh	OTTHUMG000001	TPM1	**tropomyosin 1**	Cytoplasm	other		1.001623959	0	0.99557121	0	1	0	10.304329	10.3802202	10.2056014	10.4684437
7860	**ENSG0000014044**	IGF1R	protein_coding	chr15	98648539	98964530	+	Human GRCh	OTTHUMG000001	IGF1R	**insulin like growth factor 1 receptor**	Plasma Mem	transmembra	IGF-1R inh	1.491169457	0	0.17715296	0	1	0	11.5435655	11.6797549	12.5090536	11.331244
7861	**ENSG0000014045**	ARRDC4	protein_coding	chr15	97960703	97973833	+	Human GRCh	OTTHUMG000001	ARRDC4	**arrestin domain containing 4**	Plasma Mem	other		-1.075743219	0	0.81143242	0	1	0	8.78752626	9.1296494	8.93856475	8.8363074
7862	**ENSG0000014045**	PIF1	protein_coding	chr15	64815632	64825668	-	Human GRCh	OTTHUMG000001	PIF1	**PIF1 5'-to-3' DNA helicase**	Nucleus	enzyme		-1.079169859	0	0.71304368	0	1	0	9.56171403	9.7548592	9.65842567	9.49392132
7863	**ENSG0000014045**	USP3	protein_coding	chr15	63504511	63594640	+	Human GRCh	OTTHUMG000001	USP3	**ubiquitin specific peptidase 3**	Cytoplasm	peptidase		-1.071859397	0	0.73340902	0	1	0	11.4059537	11.6794889	11.6325217	11.288855
7864	**ENSG0000014045**	CYP11A1	protein_coding	chr15	74337759	74367646	-	Human GRCh	OTTHUMG000001	CYP11A1	**cytochrome P450 family 11 subfamily A member 1**	Cytoplasm	enzyme	levoketocc	1.329626246	0	0.68839729	0	1	0	3.31376709	3.9312035	3.70223416	4.09544886
7865	**ENSG0000014046**	BBS4	protein_coding	chr15	72686179	72738475	+	Human GRCh	OTTHUMG000001	BBS4	**Bardet-Biedl syndrome 4**	Cytoplasm	other		-1.094169097	0	0.67332704	0	1	0	9.54115936	9.67773104	9.6500154	9.35461908
7866	**ENSG0000014046**	PML	protein_coding	chr15	73994673	74047827	+	Human GRCh	OTTHUMG000001	PML	**PML nuclear body scaffold**	Nucleus	transcription	arsenic tric	1.02739362	0	0.90499943	0	1	0	9.51021836	9.74352921	9.67851066	9.64522271
7867	**ENSG0000014046**	CYP1A1	protein_coding	chr15	74719542	74725536	-	Human GRCh	OTTHUMG000001	CYP1A1	**cytochrome P450 family 1 subfamily A member 1**	Cytoplasm	enzyme		-2.729636931	-1	0.4212732	0	NA	0	3.61770697	3.38658922	2.89980989	2.36773373
7868	**ENSG0000014047**	ADAMTS17	protein_coding	chr15	99971437	100342005	-	Human GRCh	OTTHUMG000001	ADAMTS17	**ADAM metallopeptidase with thrombospondin type 1 motif 17**	Extracellular	other		1.115351285	0	0.77298919	0	1	0	7.78571262	7.54611865	7.83689724	7.76611238
7869	**ENSG0000014047**	LINS1	protein_coding	chr15	100559369	100603230	-	Human GRCh	OTTHUMG000001	LINS1	**lines homolog 1**	Other	other		-1.003043865	0	0.99161472	0	1	0	9.36559133	9.41402734	9.55413007	9.19173067
7870	**ENSG0000014047**	ULK3	protein_coding	chr15	74836118	74843346	-	Human GRCh	OTTHUMG000001	ULK3	**unc-51 like kinase 3**	Cytoplasm	kinase		-1.120840431	0	0.60185913	0	1	0	10.4196803	10.7530973	10.6217321	10.2997721
7872	**ENSG0000014047**	PCSK6	protein_coding	chr15	101289784	101525202	-	Human GRCh	OTTHUMG000001	PCSK6	**proprotein convertase subtilisin/kexin type 6**	Extracellular	peptidase		1.241709671	0	0.48514785	0	1	0	7.48997694	7.58075932	8.03565926	7.47263563
7873	**ENSG0000014048**	CCDC33	protein_coding	chr15	74202705	74336472	+	Human GRCh	OTTHUMG000001	CCDC33	**coiled-coil domain containing 33**	Cytoplasm	other		10.64546771	1	0.03877818	1	1	0	2.46138447	2.57263859	2.55479575	3.36101642
7874	**ENSG0000014048**	CELF6	protein_coding	chr15	72284727	72320157	-	Human GRCh	OTTHUMG000001	CELF6	**CUGBP Elav-like family member 6**	Cytoplasm	other		1.134183308	0	0.75414828	0	1	0	5.60754266	6.05428547	6.24104019	5.61953588
7875	**ENSG0000014049**	SCAMP2	protein_coding	chr15	74843730	74873365	-	Human GRCh	OTTHUMG000001	SCAMP2	**secretory carrier membrane protein 2**	Cytoplasm	other		1.005868937	0	0.97696753	0	1	0	10.2355833	10.4955622	10.3557834	10.4016711
7877	**ENSG0000014050**	LMAN1L	protein_coding	chr15	74812716	74825757	+	Human GRCh	OTTHUMG000001	LMAN1L	**lectin, mannose binding 1 like**	Plasma Mem	other		3.415749945	1	0.15770617	0	NA	0	2.56720678	3.03038651	3.07928769	4.09408112
7878	**ENSG0000014051**	HAPLN3	protein_coding	chr15	88877294	88895597	-	Human GRCh	OTTHUMG000001	HAPLN3	**hyaluronan and proteoglycan link protein 3**	Extracellular	other		1.994211857	1	0.15438167	0	1	0	5.17917944	4.60196995	5.4002613	5.6847895
7880	**ENSG0000014052**	POLG	protein_coding	chr15	89305198	89334861	-	Human GRCh	OTTHUMG000001	POLG	**DNA polymerase gamma, catalytic subunit**	Cytoplasm	enzyme	lamivudine	1.011668885	0	0.95262756	0	1	0	11.2209887	11.4818176	11.5067612	11.2189254
7882	**ENSG0000014052**	FANCI	protein_coding	chr15	89243945	89317261	+	Human GRCh	OTTHUMG000001	FANCI	**FA complementation group I**	Nucleus	other		-1.190220641	0	0.42961202	0	1	0	11.994183	12.3442364	12.1456353	11.809556
7883	**ENSG0000014052**	ABHD2	protein_coding	chr15	89087459	89202355	+	Human GRCh	OTTHUMG000001	ABHD2	**abhydrolase domain containing 2, acylglycerol lipase**	Cytoplasm	enzyme		1.026170031	0	0.92153372	0	1	0	11.7046659	12.1072725	12.0152021	11.88461
7884	**ENSG0000014052**	WDR93	protein_coding	chr15	89690811	89743638	+	Human GRCh	OTTHUMG000001	WDR93	**WD repeat domain 93**	Other	other		1.427181861	0	0.53354319	0	1	0	4.29843962	3.3040253	3.91435483	4.4371435
7885	**ENSG0000014053**	TICRR	protein_coding	chr15	89575469	89631056	+	Human GRCh	OTTHUMG000001	TICRR	**TOPBP1 interacting checkpoint and replication regulator**	Nucleus	other		-1.145283918	0	0.49474383	0	1	0	11.3821226	11.8458329	11.5977216	11.3570328
7886	**ENSG0000014053**	NTRK3	protein_coding	chr15	87859751	88256791	-	Human GRCh	OTTHUMG000001	NTRK3	**neurotrophic receptor tyrosine kinase 3**	Plasma Mem	kinase	ONO-7579	5.65097614	1	0.31003922	0	NA	0	1.04223368	1.05848258	1.52278781	1.0431699
7887	**ENSG0000014054**	DET1	protein_coding	chr15	88494440	88514681	-	Human GRCh	OTTHUMG000001	DET1	**DET1 partner of COP1 E3 ubiquitin ligase**	Nucleus	other		-1.10503576	0	0.72274557	0	1	0	7.8426414	8.25186685	8.19349059	7.66360602
7888	**ENSG0000014054**	MFGE8	protein_coding	chr15	88898683	88913468	-	Human GRCh	OTTHUMG000001	MFGE8	**milk fat globule EGF and factor V/VIII domain containing**	Extracellular	other		1.025328744	0	0.94008762	0	1	0	8.20461189	8.30605594	8.12700398	8.41900696
7889	**ENSG0000014054**	ZNF710	protein_coding	chr15	90001324	90082206	+	Human GRCh	OTTHUMG000001	ZNF710	**zinc finger protein 710**	Nucleus	transcription		1.118355259	0	0.5961767	0	1	0	9.13928092	9.36328217	9.45718909	9.28623774
7890	**ENSG0000014055**	UNC45A	protein_coding	chr15	90930180	90954093	+	Human GRCh	OTTHUMG000001	UNC45A	**unc-45 myosin chaperone A**	Plasma Mem	other		-1.052942635	0	0.8087914	0	1	0	10.7502777	11.0508492	10.9225321	10.7760714
7891	**ENSG0000014055**	ST8SIA2	protein_coding	chr15	92393881	92468728	+	Human GRCh	OTTHUMG000001	ST8SIA2	**ST8 alpha-N-acetyl-neuraminide alpha-2,8-sialyltransferase 2**	Cytoplasm	enzyme		-1.052517445	0	0.95943916	0	1	0	3.27606012	3.40667018	2.89676196	3.60088867
7892	**ENSG0000014056**	MCTP2	protein_coding	chr15	94231538	94483952	+	Human GRCh	OTTHUMG000001	MCTP2	**multiple C2 and transmembrane domain containing 2**	Other	other		-1.330239257	0	0.32185213	0	1	0	8.62155353	8.63711519	8.3135661	8.29876708
7893	**ENSG0000014056**	FURIN	protein_coding	chr15	90868588	90883564	+	Human GRCh	OTTHUMG000001	FURIN	**furin, paired basic amino acid cleaving enzyme**	Cytoplasm	peptidase	hexa-D-arg	1.196216771	0	0.41421672	0	1	0	11.5826373	11.4391548	11.7572874	11.6716225
7894	**ENSG0000014057**	IQGAP1	protein_coding	chr15	90388242	90502239	+	Human GRCh	OTTHUMG000001	IQGAP1	**IQ motif containing GTPase activating protein 1**	Cytoplasm	other		-1.131769947	0	0.57102631	0	1	0	12.5392504	12.7957113	12.6820784	12.3686619
7895	**ENSG0000014057**	CRTC3	protein_coding	chr15	90529923	90645345	+	Human GRCh	OTTHUMG000001	CRTC3	**CREB regulated transcription coactivator 3**	Nucleus	other		1.052189382	0	0.81442904	0	1	0	10.154601	10.3157375	10.4549139	10.1078371
7896	**ENSG0000014059**	EFL1	protein_coding	chr15	82130206	82262773	-	Human GRCh	OTTHUMG000001	EFL1	**elongation factor like GTPase 1**	Cytoplasm	translation re		-1.166062474	0	0.47181581	0	1	0	9.97673155	10.3062951	10.0745518	9.882521
7897	**ENSG0000014060**	SH3GL3	protein_coding	chr15	83447228	83618743	+	Human GRCh	OTTHUMG000001	SH3GL3	**SH3 domain containing GRB2 like 3, endophilin A3**	Cytoplasm	other		1.061974823	0	0.90080839	0	1	0	6.23221638	6.56086159	6.35058271	6.58874957
7898	**ENSG0000014061**	SEC11A	protein_coding	chr15	84669538	84716460	-	Human GRCh	OTTHUMG000001	SEC11A	**SEC11 homolog A, signal peptidase complex subunit**	Cytoplasm	peptidase		1.004844625	0	0.98064055	0	1	0	10.9232939	11.1141003	11.175259	10.8596918
7899	**ENSG0000014062**	SEPTIN12	protein_coding	chr16	4777606	4788398	-	Human GRCh	OTTHUMG000001	SEPTIN12	**septin 12**	Cytoplasm	other		1.709401993	1	0.65561298	0	NA	0	2.80834027	2.73601941	3.06988107	3.26056204
7900	**ENSG0000014063**	GLYR1	protein_coding	chr16	4803203	4847288	-	Human GRCh	OTTHUMG000001	GLYR1	**glyoxylate reductase 1 homolog**	Nucleus	other		1.000617409	0	0.99736068	0	1	0	11.7685702	12.0044488	12.0018609	11.7731971
7901	**ENSG0000014065**	PMM2	protein_coding	chr16	8788823	8862534	+	Human GRCh	OTTHUMG000001	PMM2	**phosphomannomutase 2**	Cytoplasm	enzyme		-1.058935769	0	0.78211956	0	1	0	11.3487734	11.6282544	11.5864568	11.2554268
7902	**ENSG0000014067**	SLC5A2	protein_coding	chr16	31483002	31490860	+	Human GRCh	OTTHUMG000001	SLC5A2	**solute carrier family 5 member 2**	Plasma Mem	transporter	dapagliflo:	1.193860851	0	0.83710837	0	1	0	8.11708482	8.13090494	8.62359486	7.94796647
7903	**ENSG0000014067**	ITGAX	protein_coding	chr16	31355134	31382999	+	Human GRCh	OTTHUMG000001	ITGAX	**integrin subunit alpha X**	Plasma Mem	transmembra		1	0	1	0	1	0	1.09664818	1.15000429	1.14132951	1.09983827
7904	**ENSG0000014068**	TGFB1I1	protein_coding	chr16	31471585	31477960	+	Human GRCh	OTTHUMG000001	TGFB1I1	**transforming growth factor beta 1 induced transcript 1**	Nucleus	transcription		1.061602771	0	0.84697617	0	1	0	5.75419727	6.14962555	5.88190636	6.14594346
7905	**ENSG0000014068**	RUSF1	protein_coding	chr16	31489471	31509309	-	Human GRCh	OTTHUMG000001	RUSF1	**RUS family member 1**	Other	other		-1.0654588	0	0.77351427	0	1	0	11.8858262	12.0913839	12.0975181	11.7135564
7906	**ENSG0000014069**	ARMC5	protein_coding	chr16	31458080	31467166	+	Human GRCh	OTTHUMG000001	ARMC5	**armadillo repeat containing 5**	Cytoplasm	other		1.062779213	0	0.79894437	0	1	0	8.94099719	9.13398721	9.35225172	8.80018174
7907	**ENSG0000014069**	PARN	protein_coding	chr16	14435700	14632728	-	Human GRCh	OTTHUMG000001	PARN	**poly(A)-specific ribonuclease**	Nucleus	enzyme		-1.166262963	0	0.41918657	0	1	0	10.9252491	11.2258143	10.9690121	10.8561673
7908	**ENSG0000014071**	FTO	protein_coding	chr16	53701692	54158512	+	Human GRCh	OTTHUMG000001	FTO	**FTO alpha-ketoglutarate dependent dioxygenase**	Nucleus	enzyme		-1.183069568	0	0.42539501	0	1	0	11.1724777	11.5503058	11.2779839	11.0853546
7909	**ENSG0000014074**	UQCRC2	protein_coding	chr16	21953288	21983660	+	Human GRCh	OTTHUMG000001	UQCRC2	**ubiquinol-cytochrome c reductase core protein 2**	Cytoplasm	enzyme		-1.073628415	0	0.74057807	0	1	0	11.7216281	12.1445573	12.0094077	11.7150421
7910	**ENSG0000014074**	CDR2	protein_coding	chr16	22345936	22437165	-	Human GRCh	OTTHUMG000001	CDR2	**cerebellar degeneration related protein 2**	Cytoplasm	other		-1.036225592	0	0.86986962	0	1	0	9.52586616	9.95787799	9.86667819	9.55625147
7912	**ENSG0000014075**	ARHGAP17	protein_coding	chr16	24919389	25015666	-	Human GRCh	OTTHUMG000001	ARHGAP17	**Rho GTPase activating protein 17**	Cytoplasm	other		-1.141929257	0	0.51730693	0	1	0	10.3806173	10.6665585	10.5280642	10.2210994
7913	**ENSG0000014079**	MYLK3	protein_coding	chr16	46702282	46790407	-	Human GRCh	OTTHUMG000001	MYLK3	**myosin light chain kinase 3**	Cytoplasm	kinase		-1.409524137	0	0.24755678	0	1	0	7.00614848	6.93693793	6.60015069	6.64056652

7915	ENSG0000014080	NKD1	protein_coding	chr16	50548396	50649249	+	Human GRCh	OTTHUMG000001	NKD1	NKD inhibitor of WNT signaling pathway 1	Other	other		-1.36029162	0	0.79760233	0	1	0	3.39082411	3.85130957	3.53557527	3.2287045
7916	ENSG0000014082	DHX38	protein_coding	chr16	72093613	72112912	+	Human GRCh	OTTHUMG000001	DHX38	DEAH-box helicase 38	Nucleus	enzyme		-1.03745466	0	0.84722295	0	1	0	10.7287062	11.0392081	10.9859862	10.702758
7917	ENSG0000014083	TXNL4B	protein_coding	chr16	72044289	72094431	-	Human GRCh	OTTHUMG000001	TXNL4B	thioredoxin like 4B	Nucleus	enzyme		-1.104038514	0	0.67035156	0	1	0	9.11158183	9.46414722	9.26646177	9.10838429
7918	ENSG0000014083	MARVELD:	protein_coding	chr16	71626161	71642114	-	Human GRCh	OTTHUMG000001	MARVELD3	MARVEL domain containing 3	Plasma Mem	other		-1.043252502	0	0.87715325	0	1	0	8.99587821	9.24174951	9.16385495	8.98551966
7920	ENSG0000014083	ZFHX3	protein_coding	chr16	72782885	73891871	-	Human GRCh	OTTHUMG000001	ZFHX3	zinc finger homeobox 3	Nucleus	transcription		-1.047681852	0	0.82582986	0	1	0	12.4220566	12.5232852	12.4767502	12.3665305
7921	ENSG0000014083	CLEC18B	protein_coding	chr16	74408631	74421478	-	Human GRCh	OTTHUMG000001	CLEC18B	C-type lectin domain family 18 member B	Cytoplasm	other		-1.889830095	-1	0.26906468	0	1	0	4.8384019	4.93726405	4.30977349	4.3665306
7922	ENSG0000014084	CPNE2	protein_coding	chr16	57092583	57148369	+	Human GRCh	OTTHUMG000001	CPNE2	copine 2	Cytoplasm	other		1.019428837	0	0.94493216	0	1	0	8.0085529	7.58573704	7.73242131	7.93268739
7923	ENSG0000014085	NLRC5	protein_coding	chr16	56989485	57083531	+	Human GRCh	OTTHUMG000001	NLRC5	NLR family CARD domain containing 5	Cytoplasm	transcription		1.096018551	0	0.78794851	0	1	0	6.17281503	6.52002931	5.77159853	6.87115592
7924	ENSG0000014085	KATNB1	protein_coding	chr16	57735739	57757244	+	Human GRCh	OTTHUMG000001	KATNB1	katanin regulatory subunit B1	Cytoplasm	enzyme		-1.1258154	0	0.51692206	0	1	0	10.5055862	10.8403578	10.5861712	10.5136062
7925	ENSG0000014085	KIFC3	protein_coding	chr16	57758217	57863053	-	Human GRCh	OTTHUMG000001	KIFC3	kinesin family member C3	Cytoplasm	enzyme		1.583664229	1	0.20435424	0	1	0	5.67273164	5.40789151	6.06352697	5.71643707
7926	ENSG0000014087	ADAMTS1(	protein_coding	chr16	77247813	77435034	-	Human GRCh	OTTHUMG000001	ADAMTS18	ADAM metallopeptidase with thrombospondin type 1 motif 1!	Extracellular	peptidase		1.596756597	1	0.55109915	0	1	0	2.25616815	4.01088725	3.2958105	4.12578277
7927	ENSG0000014087	NUDT7	protein_coding	chr16	77722492	77742260	+	Human GRCh	OTTHUMG000001	NUDT7	nudix hydrolase 7	Cytoplasm	enzyme		1.157617779	0	0.75226165	0	1	0	4.50387408	5.10286002	4.86789096	5.06711007
7928	ENSG0000014090	GCSH	protein_coding	chr16	81081945	81096395	-	Human GRCh	OTTHUMG000001	GCSH	glycine cleavage system protein H	Cytoplasm	enzyme		1.13799506	0	0.5649957	0	1	0	8.86956951	9.23604111	9.33419957	9.0788449
7929	ENSG0000014093	CMTM3	protein_coding	chr16	66603874	66613892	+	Human GRCh	OTTHUMG000001	CMTM3	CKLF like MARVEL transmembrane domain containing 3	Extracellular	cytokine		-2.010015734	-1	0.14952231	0	1	0	4.30614437	4.53762969	3.36493456	4.25555358
7930	ENSG0000014093	CMTM2	protein_coding	chr16	66579448	66588275	+	Human GRCh	OTTHUMG000001	CMTM2	CKLF like MARVEL transmembrane domain containing 2	Extracellular	cytokine		-1.914011977	-1	0.41774523	0	NA	0	3.18009602	3.96642413	3.52409716	2.7133214
7931	ENSG0000014093	CDH11	protein_coding	chr16	64943753	65126112	-	Human GRCh	OTTHUMG000001	CDH11	cadherin 11	Plasma Mem	other		1.483020399	0	0.37167039	0	1	0	5.37357177	5.62420896	5.52937093	6.25407911
7932	ENSG0000014093	NOL3	protein_coding	chr16	67170154	67175737	+	Human GRCh	OTTHUMG000001	NOL3	nucleolar protein 3	Nucleus	other		1.27265622	0	0.25986982	0	1	0	8.62661168	8.94524566	9.13955264	8.98638456
7933	ENSG0000014094	MAP1LC3E	protein_coding	chr16	87383953	87404779	+	Human GRCh	OTTHUMG000001	MAP1LC3B	microtubule associated protein 1 light chain 3 beta	Cytoplasm	other		-1.129755627	0	0.56694798	0	1	0	10.6567255	10.9666144	10.7217789	10.6424917
7934	ENSG0000014094	MBTPS1	protein_coding	chr16	84053761	84116942	-	Human GRCh	OTTHUMG000001	MBTPS1	membrane bound transcription factor peptidase, site 1	Cytoplasm	peptidase	sonepcizur	-1.209634938	0	0.35710164	0	1	0	12.2007044	12.4445573	12.3261936	11.8571809
7935	ENSG0000014094	CDH13	protein_coding	chr16	82626965	83800640	+	Human GRCh	OTTHUMG000001	CDH13	cadherin 13	Plasma Mem	other		1.010377659	0	0.99737783	0	NA	0	0.73621527	0.07059836	0.06556559	0.74292291
7936	ENSG0000014094	ZCCHC14	protein_coding	chr16	87406246	87493024	-	Human GRCh	OTTHUMG000001	ZCCHC14	zinc finger CCHC-type containing 14	Other	other		-1.014514704	0	0.94433114	0	1	0	11.285495	11.5663332	11.6059775	11.1945485
7937	ENSG0000014095	MEAK7	protein_coding	chr16	84476355	84554033	-	Human GRCh	OTTHUMG000001	MEAK7	MTOR associated protein, eak-7 homolog	Cytoplasm	other		1.11222439	0	0.70689998	0	1	0	8.8962005	9.20022199	9.33298517	9.98001035
7939	ENSG0000014096	OSGIN1	protein_coding	chr16	83931311	83966332	+	Human GRCh	OTTHUMG000001	OSGIN1	oxidative stress induced growth inhibitor 1	Other	growth facto		-1.032599119	0	0.92977064	0	1	0	7.50990722	7.42245455	7.39790244	7.46453344
7940	ENSG0000014096	IRF8	protein_coding	chr16	85899116	85922606	+	Human GRCh	OTTHUMG000001	IRF8	interferon regulatory factor 8	Nucleus	transcription		1	0	1	0	1	0	0.23152918	0.3442157	0.3264854	0.23852517
7941	ENSG0000014098	RHOT2	protein_coding	chr16	668100	674174	+	Human GRCh	OTTHUMG000001	RHOT2	ras homolog family member T2	Cytoplasm	enzyme		1.071932947	0	0.73890999	0	1	0	12.0025525	12.3250086	12.3616433	12.1329263
7942	ENSG0000014098	RPL3L	protein_coding	chr16	1943974	1957606	-	Human GRCh	OTTHUMG000001	RPL3L	ribosomal protein L3 like	Cytoplasm	other		1.983825609	1	0.31558704	0	1	0	5.45480556	5.30148665	6.08851139	6.19579335
[truncated: 1,675,014 more chars]
